# Experimental Resurrection of Ancestral Mammalian CPEB3 Ribozymes Reveals Deep Functional Conservation

**DOI:** 10.1093/molbev/msab074

**Published:** 2021-03-15

**Authors:** Devin P. Bendixsen, Tanner B. Pollock, Gianluca Peri, Eric J. Hayden

**Affiliations:** 1 Biomolecular Sciences Graduate Programs, Boise State University, Boise, ID, USA; 2 Department of Biological Science, Boise State University, Boise, ID, USA

**Keywords:** RNA, fitness landscape, CPEB3, phylogenetics, ancestral sequence resurrection

## Abstract

Self-cleaving ribozymes are genetic elements found in all domains of life, but their evolution remains poorly understood. A ribozyme located in the second intron of the cytoplasmic polyadenylation binding protein 3 gene (*CPEB3*) shows high sequence conservation in mammals, but little is known about the functional conservation of self-cleaving ribozyme activity across the mammalian tree of life or during the course of mammalian evolution. Here, we use a phylogenetic approach to design a mutational library and a deep sequencing assay to evaluate the in vitro self-cleavage activity of numerous extant and resurrected CPEB3 ribozymes that span over 100 My of mammalian evolution. We found that the predicted sequence at the divergence of placentals and marsupials is highly active, and this activity has been conserved in most lineages. A reduction in ribozyme activity appears to have occurred multiple different times throughout the mammalian tree of life. The in vitro activity data allow an evaluation of the predicted mutational pathways leading to extant ribozyme as well as the mutational landscape surrounding these ribozymes. The results demonstrate that in addition to sequence conservation, the self-cleavage activity of the CPEB3 ribozyme has persisted over millions of years of mammalian evolution.

## Introduction

**Fig. 1.**
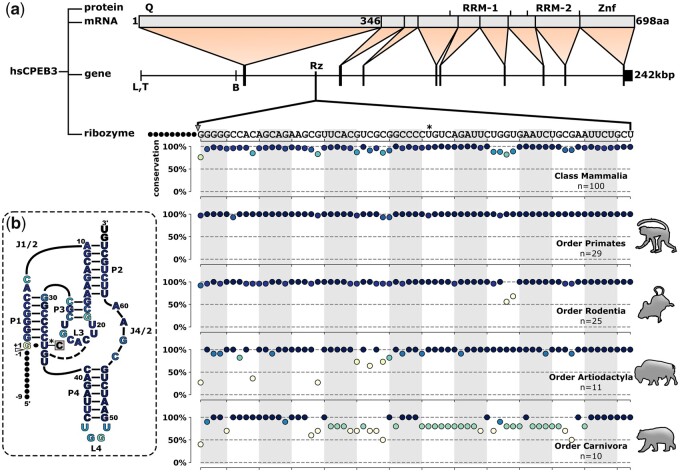
Sequence conservation and secondary structure of CPEB3 ribozyme. (*a*) Mapping and conservation of the CPEB3 ribozyme. Protein, mRNA and gene are adapted from Salehi-Ashtiani et al. (2006). Four notable domains are identified in the protein primary structure (Q, glutamine-rich domain; RRM, RNA-binding domains; Znf, zinc finger). Vertical dividers in the mRNA indicate splice sites. Tissue-specific untranslated exons are marked below the gene with letters (L, liver; T, testis; B, brain). Translated exons are indicated as large vertical lines in the “gene” diagram. Self-cleaving CPEB3 ribozyme location is indicated as Rz in the second intron and the human CPEB3 sequence is shown expanded below with the cleavage site indicated by a gray arrowhead. An asterisk marks the human SNP (U36C). Plots indicate the conservation of each nucleotide in the consensus sequence of the 100 identified mammalian CPEB3 sequences within each clade. (*b*) Secondary structure of the consensus ribozyme sequence. Triangle indicates self-cleavage site and black circles indicate cleaved sequence. Nucleotides are colored according to conservation across the Class Mammalia. Asterisk indicates the location of a SNP (U36C) in the human ribozyme sequence.

Self-cleaving ribozymes are noncoding RNA elements found in genomes across all domains of life (Webb et al. 2009; Perreault et al. 2011; Roth et al. 2014). These RNA elements are known to catalyze the site-specific cleavage of the phosphodiester backbone of transcripts in which they are found, but the biological functions of self-cleavage activity remains an active area of investigation (Jimenez et al. 2015; Weinberg et al. 2019). The CPEB3 ribozyme is an interesting example that was found in the second intron of the cytoplasmic polyadenylation element-binding 3 (*CPEB3*) gene in humans (fig. 1*a*) (Salehi-Ashtiani et al. 2006). The protein coding region of this gene encodes a functional prion protein that is involved in synaptic plasticity and long-term memory (Stephan et al. 2015). The ribozyme sequence and location in the CPEB3 intron is highly conserved in mammals (fig. 1*a*) suggesting that the self-cleavage activity of the ribozyme may also have a role in memory. In fact, a single nucleotide polymorphism in humans located in the ribozyme sequence showed statistical association with poor performance on a memory test in homozygous individuals (Vogler et al. 2009). It has been hypothesized that the self-cleavage activity of the CPEB3 ribozyme alters cotranscriptional processing of the CPEB3 pre-mRNA, but the mechanism is not known (Webb and Lupták 2011). Despite the potential functional role of this self-cleaving ribozyme in mammals, very few of the mammalian ribozymes have been functionally characterized, preventing an evaluation of the conservation of self-cleavage activity across the mammalian tree of life and over the course of mammalian evolution.

An experimental approach to understanding ancient gene function and evolution is termed ancestral sequence *resurrection* (Thornton 2004; Carletti et al. 2020). This approach involves the bioinformatic prediction of ancient gene sequences followed by experimental synthesis and functional characterization of these sequences in the lab. By resurrecting multiple ancestral nodes in a phylogeny, it is possible to evaluate the predicted ancestral states and reconstruct the step-by-step mutational pathways that evolution likely followed. Ancestral protein resurrections have been used broadly to better understand evolutionary causes of protein structure and function for numerous applications in several fields (Hittinger and Carroll 2007; Merkl and Sterner 2016; Hochberg and Thornton 2017). For example, ancestral protein resurrections have been used to evaluate ancient bio-geochemistry (Gaucher et al. 2003; Kacar et al. 2017), to identify starting points for designing novel biomolecules (Zakas et al. 2017; Alva and Lupas 2018), and to evaluate the predictability of evolution (Natarajan et al. 2016; Stern et al. 2017). Given the number of mammal genomes that are now sequenced, it is possible to predict and resurrect the nucleotide sequence changes that have occurred in the CPEB3 ribozyme during mammalian evolution to study the conservation and evolution of self-cleavage ribozyme activity. However, because numerous mutational changes have occurred over evolutionary time, the number of genes that need to be resurrected necessitates high-throughput experimental approaches.

In recent years, it has become possible to use high-throughput sequencing to characterize the function or molecular phenotype of numerous neighboring genotypes (McCandlish 2011). The resulting mapping of genotype (sequence) to phenotype (function), often referred to as a fitness landscape, can be used to determine the accessibility of mutational pathways from low to high activity. This data can be used to evaluate past evolution, in nature or in the laboratory, and may enable the forecasting of evolutionary outcomes (Kogenaru et al. 2009; Franke et al. 2011; Lobkovsky et al. 2011; de Visser and Krug 2014). Experimental fitness landscapes have been studied for several phenotypes, such as microbial growth rates in specific environments and molecular phenotypes, such as enzymatic activities of proteins and RNA molecules (Romero and Arnold 2009; Li et al. 2016; Sarkisyan et al. 2016). The genetic sequences (genotypes) studied are often chosen based on molecular structures or sequence variation resulting from natural or experimental evolution. Combinatorial DNA synthesis can yield numerous combinations of the nucleotide changes. Functional characterization of these combinatorial libraries elucidates the possible evolutionary trajectories to higher fitness (Weinreich et al. 2006; Hayden 2016). With appropriate library design, the fitness landscape can be applied to ancestral sequence resurrection in order to evaluate numerous ancestral nodes and the functional effects along mutational pathways to extant sequences.

Here, we use a phylogenetically guided library design to characterize the self-cleavage activity of extant and resurrected CPEB3 ribozymes by deep sequencing of the ribozyme reaction products following in vitro transcription. We used 100 extant ribozyme sequences from 99 mammalian genomes (two variants in humans) to predict ancestral ribozyme sequences and to guide a combinatorial library of ribozymes for high-throughput sequencing-based characterization. We identified 13 mutational positions that accounted for a majority of the extant sequence diversity and designed a combinatorial DNA library that contained 27,648 sequences comprised of all the possible combinations of the mutations observed at these positions. This combinatorial library contained most of the extant ribozyme sequences, the predicted ancestral nodes and the numerous combinations of nucleotide changes on the parsimonious mutational trajectories in between. In addition, the majority of sequences in our library represent random combinations of the naturally occurring mutations, enabling a comparison between ribozymes that survived natural selection and those that did not. We simultaneously determined the in vitro activity of all library sequences during a cotranscriptional self-cleavage reaction. Following in vitro transcription, the RNA was reverse-transcribed with a 5′-RACE protocol that added the same primer binding site to both cleaved and uncleaved molecules, which were then PCR amplified to add sequencing adaptors and replicate-specific indexes. We quantified the number of reads mapping to the cleaved and uncleaved form of each sequence, and used the ratio to determine the fraction cleaved of each sequence variant as a measure of relative ribozyme activity (Dupont et al. 2015; Hayden 2016; Kobori and Yokobayashi 2016; Zhang et al. 2020). We also used polyacrylamide gel electrophoresis (PAGE) to analyze several individual ribozymes in order to analyze additional species and to validate some of the sequencing-based measurements. We used this data to evaluate the conservation of this ribozyme activity over millions of years of mammalian evolution.

## Results and Discussion

### Ribozyme Sequence and Activity Has Been Conserved since the Divergence of Placentals and Marsupials

**Fig. 2.**
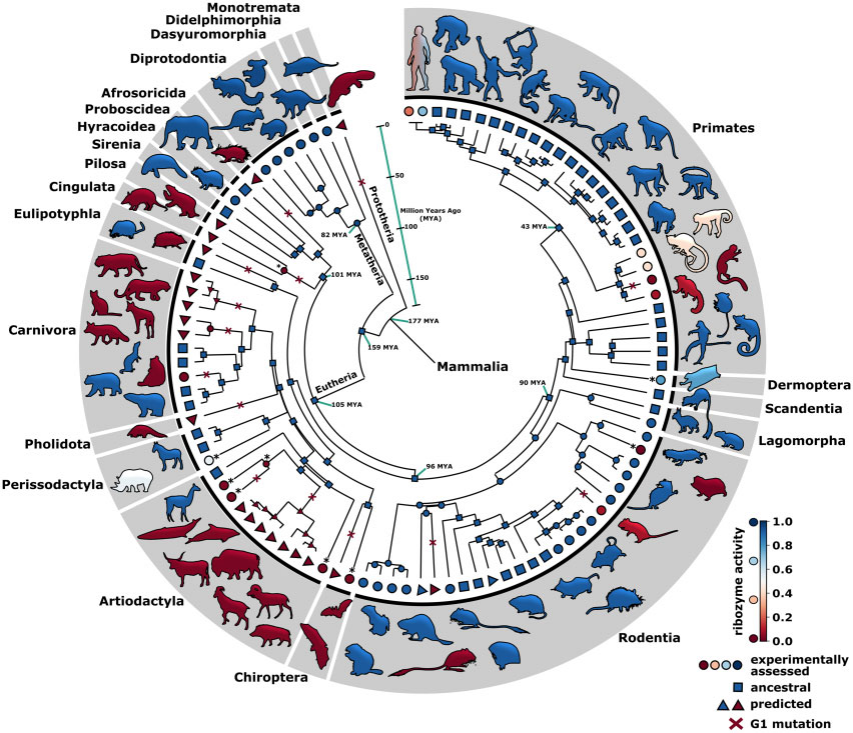
In vitro activity of extant and ancestral mammalian CPEB3 ribozymes mapped onto the mammalian tree of life. Phylogenetic tree derived from the 99 mammalian species with identified CPEB3 ribozyme sequences. Each node indicates a ribozyme sequence that is either found in an extant species (outer) or represents a predicted *ancestral sequence* (inner). The color of the node indicates the in vitro self-cleaving ribozyme activity (see inset). Animal silhouettes are colored according to their respective ribozyme activities. Square nodes indicate a single highly functional, highly conserved *ancestral sequence*. Circle nodes indicate ribozyme sequences that were biochemically assessed using high-throughput sequencing. Circle nodes with asterisks indicate sequences assessed using gel electrophoresis. Triangle nodes indicate a sequence with predicted ribozyme activity based on mutational effects observed in the data. Red “x” indicates a mutation at position G1. Species names are indicated in supplementary figure S1, Supplementary Material online.

To predict ancient ribozyme sequences, we mapped the known CPEB3 ribozyme sequences onto a mammalian tree of life and used maximum likelihood to predict the ribozyme sequences of common ancestors at the nodes of the tree (fig. 2, supplementary fig. A1, Supplementary Material online). We found that the ribozyme sequence predicted at the divergence between marsupials and placentals was identical to the ribozyme sequence in 41 of the extant mammals in our data set. This suggests that the sequence has been 100% conserved for ∼159 My in several lineages (fig. 2, blue squares). We will refer to this conserved ribozyme sequence as the *ancestral sequence* for this study, although there may have been different sequences in the earliest mammals. It was not possible to predict the sequence of deeper branches in the phylogeny (fig. 2 “177 Ma”) because the CPEB3 ribozyme has not been found outside of mammals, presenting a lack of outgroups. This conserved *ancestral sequence* was also predicted at 64 out of 97 intermediate nodes of the tree. All nucleotides of common ancestor nodes were predicted with a high probability of *P *>* *0.95, except for a single node where two nucleotides were predicted with a slightly lower probability of *P *>* *0.82 (supplementary data file S1 and fig. S2, Supplementary Material online, node 190). A maximum parsimony approach resulted in identical predictions of the ribozyme sequences at the ancestral nodes in the tree.

Despite the ribozyme sequence conservation, there still exists sequence variation in the extant and predicted CPEB3 ribozymes (fig. 1). In order to understand the evolution of ribozyme activity, we next set out to determine the activity of all the ribozymes, extant and predicted ancestors, and map the activity onto the phylogenetic tree. The combinatorial library of sequences was transcribed in vitro, and deep sequencing of the cotranscriptional reaction product was used to determine the fraction of reads in the cleaved state (fraction cleaved) for each sequence. We found that this sequencing-based activity was highly correlated between three experimental replicates (supplementary fig. S3, Supplementary Material online). Two additional approaches were used to assign activity to ribozymes that were not in our combinatorial library due to the challenge of synthesizing more divergent sequences, such as those with length differences. Ribozymes from nine organisms or ancestral nodes (Malayan flying lemur, rhino, minke whale, minke whale/dolphin, dolphin, pig, microbat, armadillo/sloth, Damara mole rat) were assayed separately for cotranscriptional self-cleavage activity under identical conditions, but analyzed using denaturing PAGE (fig. 2, asterisks, supplementary fig. S4, Supplementary Material online). The activity of the remaining ribozymes was predicted to be the same as ribozymes in the high-throughput assay with similar nucleotide changes (fig. 2 triangles). Specifically, 21 sequences were predicted to have very low activity due to the presence of a mutation at the G1 position, which dramatically reduced self-cleavage activity in our sequencing-based data (fig. 2, “G1 mutations”). Two sequences (American beaver and prairie vole) were predicted to have high ribozyme activity because they only contained mutations in L4, and mutations in this structural element have little effect on ribozyme activity in our data and in previous publications (Salehi-Ashtiani et al. 2006; Webb and Lupták 2011). Using all three approaches, a ribozyme activity was assigned to every node and leaf in the mammalian phylogenetic tree. Notably, the results confirmed that the *ancestral sequence* was highly active in our self-cleavage data with most of the reads in the cleaved form (fig. 2, blue squares, fraction cleaved = 0.92), consistent with previous reports of the elephant and rabbit ribozyme which is the same sequence (Salehi-Ashtiani et al. 2006). This extensive conservation of high self-cleavage activity over geological time scales, and in multiple lineages, supports the hypothesis that the self-cleavage activity has an important functional role in mammals.

### Multiple Occurrences of Reduced Ribozyme Activity

The mapping of ribozyme activity onto the mammalian tree suggests multiple separate occurrences of reduced ribozyme activity despite the overall high sequence conservation (fig. 2). The individual nucleotide change from a G to an A at the first nucleotide position (G1A) appears to be responsible for the majority of instances where ribozyme activity is greatly reduced. This nucleotide forms the first base pair in the ribozyme structure and is immediately adjacent to the self-cleavage site. The 5′ hydroxyl of G1 is the leaving group in the self-cleavage reaction. This G1 forms a wobble base pair with U36 in most highly active sequences, which was previously shown to be a binding site for Mg^2+^ (Skilandat et al. 2016). In our data, all sequences with a G1A mutation showed very low activity (supplementary fig. S5, Supplementary Material online). In the rat for example, G1A is the only nucleotide change from the *ancestral sequence*, and this mutation reduces the fraction cleaved from 92% to 3%. A slow self-cleavage rate for the rat ribozyme was previously reported (Salehi-Ashtiani et al. 2006). Further, numerous studies on the structurally related HDV self-cleaving ribozyme have shown that G1 mutations typically reduced activity except when “fast-folding” variants that prevent misfolding were studied (Cerrone-Szakal et al. 2008). In addition to G1A, there are a few other instances of reduced activity, most notably in primates, including humans. However, these primate mutations often lead to only slightly reduced ribozyme activity. The strong effect and common occurrence of G1 mutations allows for both high sequence conservation and high variability in ribozyme activity. It is worth noting that the effect of fast or slow self-cleavage on the function of cells and tissues remains unknown, and it has been suggested that slower self-cleavage activity is beneficial for memory (Vogler et al. 2009).

Naturally occurring mutations that maintain high ribozyme activity occur almost exclusively in the L4 loop. Both rodents and marsupials have ribozymes with mutations in L4. These mutations are not related by descent based on the phylogenetic distance between these groups and the more extensive variation in marsupials. The tolerance of mutations in this structural element is not surprising based on the distance of this element from the active site, and numerous prior mutational studies in the structurally related HDV ribozyme. The existence of these “nearly neutral” mutations in L4 in multiple species further support the hypothesis that mutations that maintain self-cleavage activity have been preserved by evolution for millions of years in many lineages.

### The RNA Fitness Landscape around CPEB3 Ribozymes

**Fig. 3.**
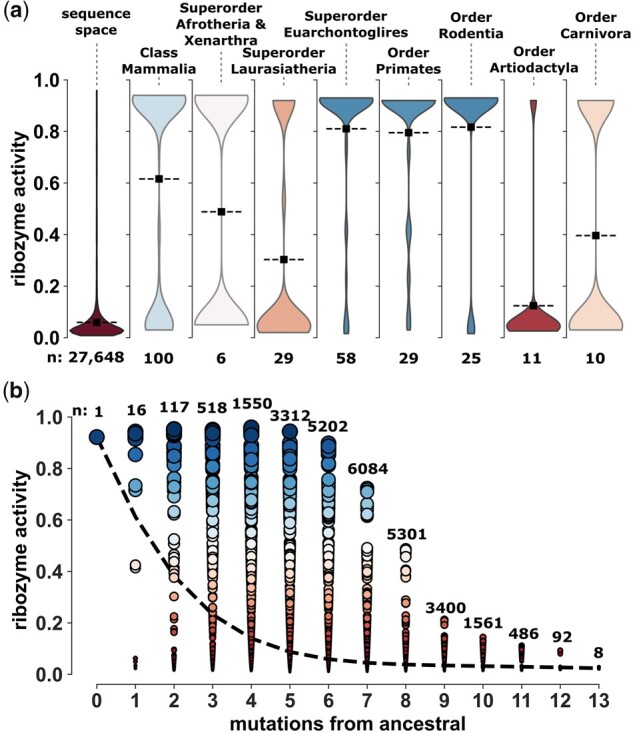
Distributions of ribozyme activities. (*a*) Distributions of ribozyme activities for taxonomic groups. Dashed lines indicate mean of ribozyme activities. Plots are colored according to the median ribozyme activity. Extant sequences with predicted low ribozyme activities were assumed to have ∼0.05 activity for the distribution. (*b*) Ribozyme activity of all 27,648 sequences in the phylogenetic mutational library plotted as a function of mutations from the highly conserved *ancestral sequence*. Each node indicates a unique ribozyme sequence and the color and size of the node indicate ribozyme activity. The number of sequences (*n*) that correspond to each mutational distance from the *ancestral sequence* is shown. The dashed line passes through the mean activity at each mutational distance.

In addition to the ribozymes predicted to have persisted in mammals over evolutionary time, our combinatorial library also contained numerous (>27,000) random mixtures of the naturally occurring mutations. These sequences can be considered representative of the RNA fitness landscape surrounding the extant and predicted ribozymes. Most of these combinations of mutations may have never occurred together or were eliminated from populations by natural selection. The vast majority of sequences generated by random mixtures of mutations were essentially inactive, and 26,490 sequences showed *ribozyme activity* < 0.2 (fig. 3*a*, supplementary figs. S6 and S7, Supplementary Material online). In contrast, the distribution of activity of extant mammalian ribozymes is shifted toward high ribozyme activity (mean > 0.6). This shifted distribution is evident within the class Mammalia, as well as within most orders, such as Primates and Rodentia. The orders Carnivora and Artiodactyla, and super orders Laurasiatheria and Afrotheris/Xenarthra showed more sequences with low activity, and corresponding lower mean of the bimodal distribution of ribozyme activities (fig. 3*a*). A plot of *ribozyme activity* as a function of mutational distance (*mutations from ancestral*) showed that the ribozyme activity dropped rapidly with increasing numbers of randomly mixed extant mutations, on average (dashed line) (fig. 3*b*). However, there were some combinations up to six or seven mutations that maintained high ribozyme activity. These high-activity variants with six or more mutations often contained four mutations in the L4 loop combined with the mutations C9U and/or G30A (supplementary fig. S8, Supplementary Material online). This analysis of the mutational landscape showed that the accumulation of naturally occurring mutations, when mixed at random, quickly reduces ribozyme activity, suggesting that the conservation of high-activity ribozymes for millions of years may have required eliminating such combinations from evolving populations.

**Fig. 4.**
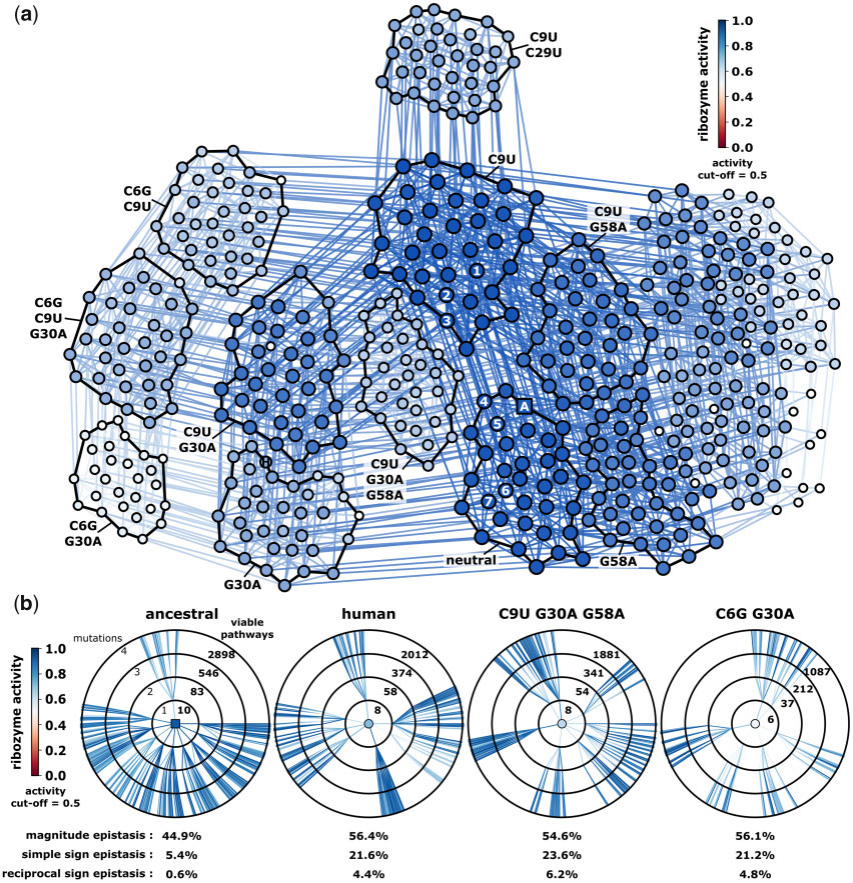
Genotype network and mutational neighborhoods. (*a*) A “top down” view of the CPEB3 ribozyme genotype network as a force-directed graph. Nodes represent individual sequences. Ribozyme activity is represented with node color and size (see inset for color scale). Only nodes with *ribozyme activity* > 0.5 are shown. Edges connect nodes that differ by a single nucleotide change and are colored as the average of the two connected nodes. Clusters of nodes that are outlined in black all share a common group of mutations, which are labeled. Extant ribozyme sequences are labeled: 1 = opossum/koala, 2 = Tasmanian devil/common wombat/wallaby, 3 = bushtail possum, 4 = rock hyrax, 5 = mouse and several other rodents, 6 = pika/marmot/and several other rodents, 7 = chinchilla, A = *ancestral sequence*, H = human ribozyme. (*b*) The mutational neighborhood surrounding specific ribozyme sequences. The center node represents the ribozyme variant labeled above. Each concentric circle represents all the sequences at that mutational distance (1–4) that are accessible to the central ribozyme sequence through mutational pathways while maintaining *ribozyme activity* > 0.05. Edges are colored based on the ribozyme activity of the genotype with higher mutations (see color bar).

We next constructed a genotype network as a visual representation of the mapping of genotype (sequence) to phenotype (self-cleavage activity) (fig. 4*a*). For this network, nodes represent individual sequences and edges connect sequences that can be interconverted by a simple mutation (single nucleotide change). We plotted all sequences from our deep sequencing data that showed *ribozyme activity* > 0.5, which emphasizes mutations that maintain higher self-cleaving potential and also reduces the total number of nodes and edges for visual clarity. The force-directed graph showed clusters of genotypes that tend to be more connected within the cluster than between clusters. The graph indicates that the *ancestral sequence* is well connected to other sequences with high ribozyme activity (fig. 4*a*). Several extant ribozymes are also found within this highly connected network (fig. 4*a*, numbers). As a quantitative measure of mutational connectedness, we also counted the number of “viable pathways” that connect the *ancestral sequence* to genotypes up to four mutations away where all mutational steps pass through genotypes with *ribozyme activity* > 0.5. This analysis confirmed that the *ancestral sequence* had more “viable pathways” between genotypes than representatives from other clusters on the graph, including the human CPEB3 sequence (fig. 4*b*). In addition, the viable pathways connected to the *ancestral sequence* have very little reciprocal sign epistasis, which is a common measurement of ruggedness in molecular fitness landscapes (Szendro et al. 2013). Only 0.6% of mutational pairs around the *ancestral sequence* showed reciprocal sign epistasis, whereas those around the human and other groups showed ∼4–6% reciprocal sign epistasis. The entire data set on average showed 2.9% reciprocal sign epistasis (supplementary fig. S9, Supplementary Material online). Combined, the analysis of the number and ruggedness of pathways indicate that the ancestral CPEB3 ribozyme is mutationally robust because there are numerous ways to maintain ribozyme activity despite several mutations. This analysis suggests that these properties of the genotype network may have contributed to the conservation of ribozyme activity during mammalian evolution.

### Mutational Pathways to Extant Sequences

**Fig. 5.**
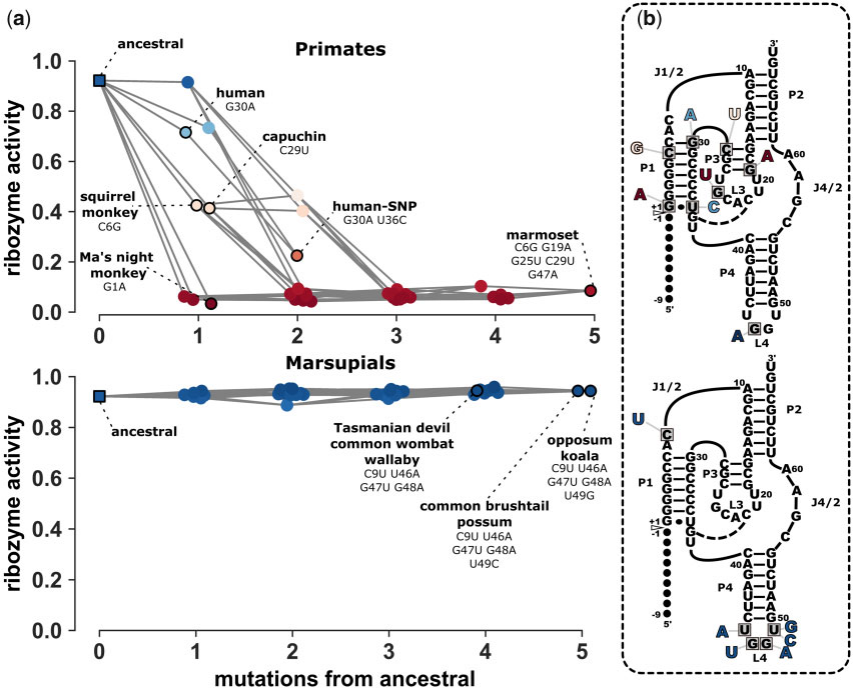
Potential evolutionary pathways from the *ancestral sequence* to extant primates and marsupials. (*a*) The top panel depicts the mutational pathways from the *ancestral sequence* to 29 extant primate sequences. The bottom panel depicts the mutational pathways between the *ancestral sequence* and six extant marsupial sequences. Each data point (node) indicates the relative ribozyme activity (*y*-axis) and the number of mutations from the *ancestral sequence* (*x*-axis). Nodes have been horizontally jittered for help with visualization. Color of nodes indicates ribozyme activity (scaled as in other figures). Nodes of ancestral and extant sequences have black edges and are labeled with species names and specific mutations. Nodes without black edges are predicted intermediate sequences. Gray lines (edges) connect nodes that differ by a single mutation. (*b*) The top secondary structure indicates the mutations found in primates relative to the *ancestral sequence*. The bottom structure shows mutations found in marsupials. Mutational nucleotides are colored according to the single mutation effect on the ribozyme activity of the *ancestral sequence*, with color scaled as identical to other figures. The triangle indicates the self-cleavage site and the upstream sequence is indicated as black dots.

We next explored the mutational pathways from the *ancestral sequence* to extant sequences in primates and marsupials (fig. 5*a*). Both groups have species with up to five mutational differences from the *ancestral sequence*; however, the mutational pathways are vastly different. Within primates, the majority of genomes retained the *ancestral sequence* and high ribozyme activity. However, for the six extant primates that acquired ribozyme mutations, the activity ranges from intermediate to low, with a general trend that activity was lower with more mutations. These mutations are found throughout the ribozyme structure with varying levels of mutational effects (fig. 5*b*). In contrast, sequences in the marsupial lineage acquired up to five mutations without any major reduction in ribozyme activity with the majority of mutations isolated in the L4 loop with minimal mutational effect. The mutational pathways from the *ancestral sequence* to extant marsupial sequences all maintain high activity demonstrating that the marsupial ribozymes are part of a neutral network. The marsupial ribozyme evolution did not involve a loss and regain of activity. The primate mutations, on the other hand, appear to be very recent and lineage specific. The root of primates, and the majority of primate genomes, maintain the ancestral ribozyme, suggesting that the ribozymes are still highly active in most primates. Although it is possible that the reduced activity in humans and other primates indicates that the ribozyme has lost a functional role in these species, the consequence of slower ribozyme self-cleavage remains unknown. It is possible that the human ribozyme has evolved to an intermediate activity because it allows proper timing or coordination with other cellular components.

### In Vitro Data Show Self-Cleavage Potential of Intrinsic Ribozyme Activities

The sequence upstream of the human CPEB3 ribozyme has been shown to cause a misfolded structure (Alt P1) that slows the observed self-cleavage rate by ∼250-fold, hiding the intrinsically fast reaction of this ribozyme (Chadalavada et al. 2010). We analyzed the sequences upstream of the mammalian ribozymes in our data and found that despite considerable variation, the consensus sequence of the first eight nucleotides is identical to the sequence found in humans (supplementary fig. S10, Supplementary Material online). Because the ribozyme part of Alt P1 is highly conserved, the upstream consensus suggests that there is considerable misfolding potential in other species. However, it was also previously shown that a single nucleotide change upstream of the human ribozyme was sufficient to destabilize Alt P1 and reveal the intrinsically fast self-cleavage of the human CPEB3 ribozyme (supplementary fig. S11, Supplementary Material online) (Chadalavada et al. 2010). Analyzing the misfolding potential of all 100 ribozymes with their native upstream sequences was beyond the scope of this investigation. Instead, in our experiments we used an upstream sequence that was unrelated to any naturally occurring sequence in order to prevent bias toward any specific taxonomic group, and to reveal the “intrinsic” activities of the studied ribozymes. Our deep sequencing data and subsequent PAGE analysis of several constructs confirmed that our upstream sequence prevented misfolding in the human ribozyme (supplementary fig. S12, Supplementary Material online), and did not cause decreased activity in ribozymes with low observed activity in our deep sequencing data (supplementary fig. S13, Supplementary Material online). Specifically, we found that the human CPEB3 ribozyme had higher activity than the human SNP variant with our chosen upstream sequence but not with the native upstream sequence as expected from the previous studies of misfolding (supplementary figs. S12 and S13, Supplementary Material online). We also performed additional computational predictions which indicated that our upstream sequence prevented misfolding in the human and elephant sequence (Supplementary Material online). Interestingly, a PAGE based analysis of the elephant ribozyme, which is the *ancestral sequence*, with the upstream sequence from the elephant genome suggested that there is misfolding potential in elephants (supplementary fig. S13, Supplementary Material online). We conclude that the upstream sequence used in our sequencing-based assay revealed the “intrinsic activity” of most ribozymes. We interpret this intrinsic activity only as self-cleavage potential, which could be modulated by cellular environments.

The deep conservation of sequence and activity suggests an important functional role for the CPEB3 ribozyme. However, several challenges remain in being able to understand if and how ribozyme self-cleavage could contribute to organismal fitness. First, the ribozyme self-cleavage activity could be different in vivo than in our in vitro assay. As mentioned above, the intronic RNA sequence immediately 5′ of the ribozyme could slow self-cleavage in other organisms. It has been observed that the activity of the CPEB3 ribozyme is tissue dependent, with highest activity observed in the brain (Salehi-Ashtiani et al. 2006). This tissue dependence suggests that there are cell-type specific variables that can alter ribozyme activity. It is possible, for example, that specific proteins can bind to the mRNA and increase self-cleavage activity, which has been observed in other ribozymes, including the structurally similar HDV ribozyme and group I self-splicing introns (Chadalavada et al. 2007; Vicens et al. 2008). Although ribozyme self-cleavage is expected to decrease the expression of the CPEB3 protein, the effect of decreased expression on cellular function is complex and depends upon the downstream interaction between several mRNA and the CPEB3 protein. The function of the CPEB3 protein itself depends on posttranslational modifications, and this protein can activate or repress translation of bound mRNA depending on these modifications (Drisaldi et al. 2015; Ford et al. 2019). The downstream effect of changing CPEB3 protein levels would depend upon the state of the cell and the posttranslational modification signaling pathways. Finally, even if ribozyme activity were shown to effect memory as has been proposed, there remain challenges in understanding the evolution of cognitive traits (Allen and Fortin 2013; Miller et al. 2020). Additional research into these areas will be needed in order to fully understand the causes and consequences of the high conservation of ribozyme sequence and self-cleavage activity.

## Conclusion

We have reported the relative self-cleavage activity of the CPEB3 ribozymes found in the genomes of 99 extant mammals and numerous predicted ancestral ribozymes. The results support the hypothesis that CPEB3 ribozymes have had a functional role in mammals for over 100 My of evolution. Our in vitro data indicate that intrinsically high self-cleavage activity has been conserved in many taxonomic group since early mammals. However, we also found that not all ribozymes in extant mammals showed high self-cleavage activity in our data. Challenges remain in understanding the functional role of this ribozyme, and further investigation into cell specific regulation of ribozyme self-cleavage activity is warranted. A better understanding of the CPEB3 ribozyme will contribute to our ever-expanding understanding of the biological and evolutionary importance of noncoding RNA elements.

## Materials and Methods

### Mammalian CPEB3 Ribozyme Phylogenetic Mutational Library Design

Mammalian CPEB3 ribozyme sequences were identified using Ensemble Genome Browser with the search tool BlastN and default settings (Yates et al. 2020), UCSC Genome Browser with the search tool BLAT and default settings (Kent et al. 2002) and from previous literature (Webb and Lupták 2011). Ribozyme sequences identified in annotated genomes were verified to occur within the expected CPEB3 gene (supplementary data file S1, Supplementary Material online). Previous analyses showed that HDV-like ribozymes were not found outside the CPEB3 gene in humans and other mammals (Webb and Lupták 2011). The 100 ribozyme sequences included in this study are not exhaustive. Several new ribozyme sequences have been identified following publication of new genomes during the analysis and writing of this study. These new sequences were not included in the analyses presented here; however, they have been included in the supplement for reference (supplementary data file S1, Supplementary Material online).

The ribozyme sequences were aligned and 13 mutational positions were identified that maximized phylogenetic coverage. For this study, only the mutations that occurred in the length of the ribozyme (67 nt) were considered. Of the 100 CPEB3 ribozyme sequences, 71 species had only differences within these 13 mutational positions. Of the 71 species in this group, 41 of these species had the same identical ribozyme sequence. A degenerate “doped” DNA oligonucleotide was synthesized with an appended T7 promoter for in vitro transcription. At each “doped” position, the DNA library was synthesized with equal mixtures of two or three nucleotide phosphoramidites representing the nucleobases found in the extant ribozymes at that position. Two phosphoramidites were included at ten nucleotide positions, and three phosphoramidtes were included at three positions, creating 27,648 (2^10^ × 3^3^) unique sequences. The library contained the same sequence upstream of the cleavage site (5′-GGACCAUUC-3′) which is not found in any of the mammalian genomes. This sequence was also chosen because it does not form a known alternative structure in the human ribozyme, and did not appear to form any significant alternative pairing with P1 in any of the ribozyme sequences included in the library. A common sequence was added to the 3′-end of the transcript to act as a universal primer binding site during reverse transcription (Wilkinson et al. 2006; Bendixsen et al. 2019).

### Cotranscriptional Self-Cleavage Assay

Cotranscriptional self-cleavage reactions were carried out as previously described (Bendixsen et al. 2019). Three replicate transcriptions were performed and sequenced. Briefly, the T7 promoter was made double stranded by mixing 20 pmol of template and 20 pmol of a short oligo complementary to the promoter (T7-TOP+). The DNA was annealed by heating and slow cooling in T7 transcription buffer (10× = 500 µl 1 M Tris pH 7.5, 50 µl 1 M DTT, 20 µl 1 M spermidine, 100 µl 1 M MgCl_2_, 320 µl RNase-free water). The template and primer were diluted 10-fold in 1X T7 buffer, and 2 µl of diluted template/primer were added to a 50 µl reaction with T7 buffer (1×), 1 µl rNTP (25 mM, NEB), 1 µl T7 RNA polymerase (200 units, Thermo Scientific) and 41 µl RNase free water (Ambion) at 37 °C for 20 min. The transcription and cotranscriptional self-cleavage reaction were terminated by adding 15 µl of 50 mM EDTA. RNA was purified and concentrated with Direct-zol RNA MicroPrep and TRI-Reagent (Zymo Research) eluted with 7 µl RNAse free water. Concentration was determined by absorbance at 260 nm (ThermoFisher NanoDrop) and samples were normalized to 5 µM. Purified RNA (five picomoles) was mixed with 20 picomoles of reverse transcription primer in a volume of 10 µl, heated at 72 °C for 3 min and cooled on ice. 4 µl SMARTScribe 5× First-Strand Buffer (Clontech), 2 µl dNTP (10 mM), 2 µl DTT (20 mM), 2 µl phased template switching oligo mix (10 µM), 1 µl water and 1 µl SMARTScribe Reverse Transcriptase (10 units, Clontech) were added. The phased template switching oligo mix consisted of four oligonucleotides that were phased by the addition of 9, 12, 15, or 18 nucleotides (Bendixsen et al. 2020). The mixture was incubated at 42 °C for 90 min and stopped by heating to 72 °C for 15 min. cDNA was purified on a silica-based column (DCC-5, Zymo Research) and eluted into 7 µl water. Illumina adapter sequences and indexes were added using PCR. A unique index combination was assigned to each replicate. The PCR reaction contained 1 µl purified cDNA, 12.5 µl KAPA HiFi HotStart ReadyMix (2×, KAPA Biosystems), 2.5 µl forward, 2.5 µl reverse primer (Illumina Nextera Index Kit) and 5 µl water. Several cycles of PCR were examined using gel electrophoresis and a PCR cycle was chosen that was still in logarithmic amplification, prior to saturation. Each PCR cycle consisted of 98 °C for 10 s, 63 °C for 30 s and 72 °C for 30 s. PCR DNA was purified on silica-based columns (DCC-5, Zymo Research) and eluted in 30 µl water. The final product was then verified using gel electrophoresis.

### High-Throughput Sequencing

The indexed PCR products from the three replicates were pooled together in equimolar concentrations based on fluorescent quantification. The two libraries were sequenced using an Illumina HiSeq 4000 on separate lanes (Genomics and Cell Characterization Core Facility, University of Oregon). For each lane 25% PhiX was added to increase the nucleotide diversity during sequencing.

### Sequencing Data Analysis

Sequencing data were analyzed using custom Python scripts on the Boise State R2 computer cluster (BSURC). The scripts identified the reverse transcription primer binding site at the 3′-end to determine nucleotide positions and then determined if the sequence was cleaved or uncleaved by the absence of presence of the 5′-upstream sequence. Nucleotide identities at the 13 variable positions were determined. For each unique genotype in the library the number of cleaved and uncleaved sequences were counted and ribozyme activity (fraction cleaved) was calculated as: $f_{\mathrm{cleaved}}=n_{\mathrm{cleaved}}/(n_{\mathrm{cleaved}}+n_{\mathrm{uncleaved}})$.

### CPEB3 Ribozyme Phylogenetic Tree Construction and Ancestral Sequence Prediction

For the 99 mammalian species with known CPEB3 ribozyme sequences, a phylogenetic tree was constructed based on the tree-of-life and its evolutionary timescale (Hedges et al. 2015). The 99 species were loaded into TimeTree and a phylogenetic tree that showed the 99 extant species and ancestral progenitors in the class Mammalia was generated (Kumar et al. 2017). Using this phylogenetic tree, Molecular Evolutionary Genetics Analysis 7 (MEGA7) software was used to infer ancestral sequences (Kumar et al. 2016). Ancestral sequences were predicted using two methods: 1) maximum likelihood using the Tamura–Nei model and uniform rates among sites and 2) maximum parsimony. Both methods resulted in identical inferred ancestral sequences.

### Network Graph and Mutational Pathway Analysis

Visualizations of genotype network graphs were constructed using Gephi (Bastian et al. 2009). Each node represents a unique genotype and edges connect genotypes that differ by a single mutation. Nodes are colored according to their ribozyme activity. ForceAtlas 2 was used to approximate genotype repulsion using a Barnes–Hut calculation. For visualization purposes, hubs were dissuaded and overlap was prevented. In order to assess the mutational neighborhood around a given genotype, mutational pathway analysis was used. For this analysis, a mutational pathway was followed for up to four mutations, encompassing a total of 37,200 pathways. Pathways that encountered a genotype with ribozyme activity <0.5 were ended. Relative prevalence of three classes of pairwise epistasis were assessed using the Genonets Server.(Khalid et al. 2016; Aguilar-Rodríguez et al. 2017). Pairwise epistasis was assessed using mutational pairs or squares. Two precise mutations can occur in either order, and are represented by subgraphs of four connected genotypes. Each square consists of a starting reference genotype, two single and one double mutant.

## Supplementary Material

Supplementary data are available at *Molecular Biology and Evolution* online.

## Supplementary Material

msab074_Supplementary_Data
