# Supplementary material for: Experimental Resurrection of Ancestral Mammalian CPEB3 Ribozymes Reveals Deep Functional Conservation
Source: Mol Biol Evol. 2021 Mar 15;38(7):2843–53. doi: 10.1093/molbev/msab074 (PMC8233481; doi:10.1093/molbev/msab074)
Supplement: msab074_Supplementary_Data [file msab074_supplementary_data.zip › CPEB3Phylo_Bendixsen_etal_MBE_SuppMat-v3.docx]

**Supplementary Material**

**Experimental resurrection of ancestral mammalian CPEB3 ribozymes reveals deep functional conservation**

Devin P. Bendixsen^1†^, Tanner B. Pollock^2^, Gianluca Peri^1^ and Eric J. Hayden^1,2^

^1^Biomolecular Sciences Graduate Programs, Boise State University, Boise, ID, USA.

^2^Department of Biological Science, Boise State University, Boise, ID, USA.

^†^Present address: Department of Zoology: Population Genetics, Stockholm University, Stockholm, Sweden

Corresponding Authors

Devin Bendixsen: [devinbendixsen@u.boisestate.edu](mailto:devinbendixsen@u.boisestate.edu)

Eric Hayden: [erichayden@boisestate.edu](mailto:erichayden@boisestate.edu)

 **Figure S1: CPEB3 phylogenetic tree with species names.**

Phylogenetic tree derived from the 99 mammalian species with identified CPEB3 ribozyme sequences. Extant species are toward the outside. Each node indicates a ribozyme sequence that is either found in an extant species (outer) or represents a predicted ancestral sequence (inner). The tree is identical to **Figure 2** and includes species names.

**Figure S2: Phylogenetic tree and ancestral sequence reconstruction.**

Phylogenetic tree derived from the 99 mammalian species with CPEB3 ribozyme sequences. Numbers at extant and ancestral nodes correspond to ribozyme sequences in Data File S1.

*
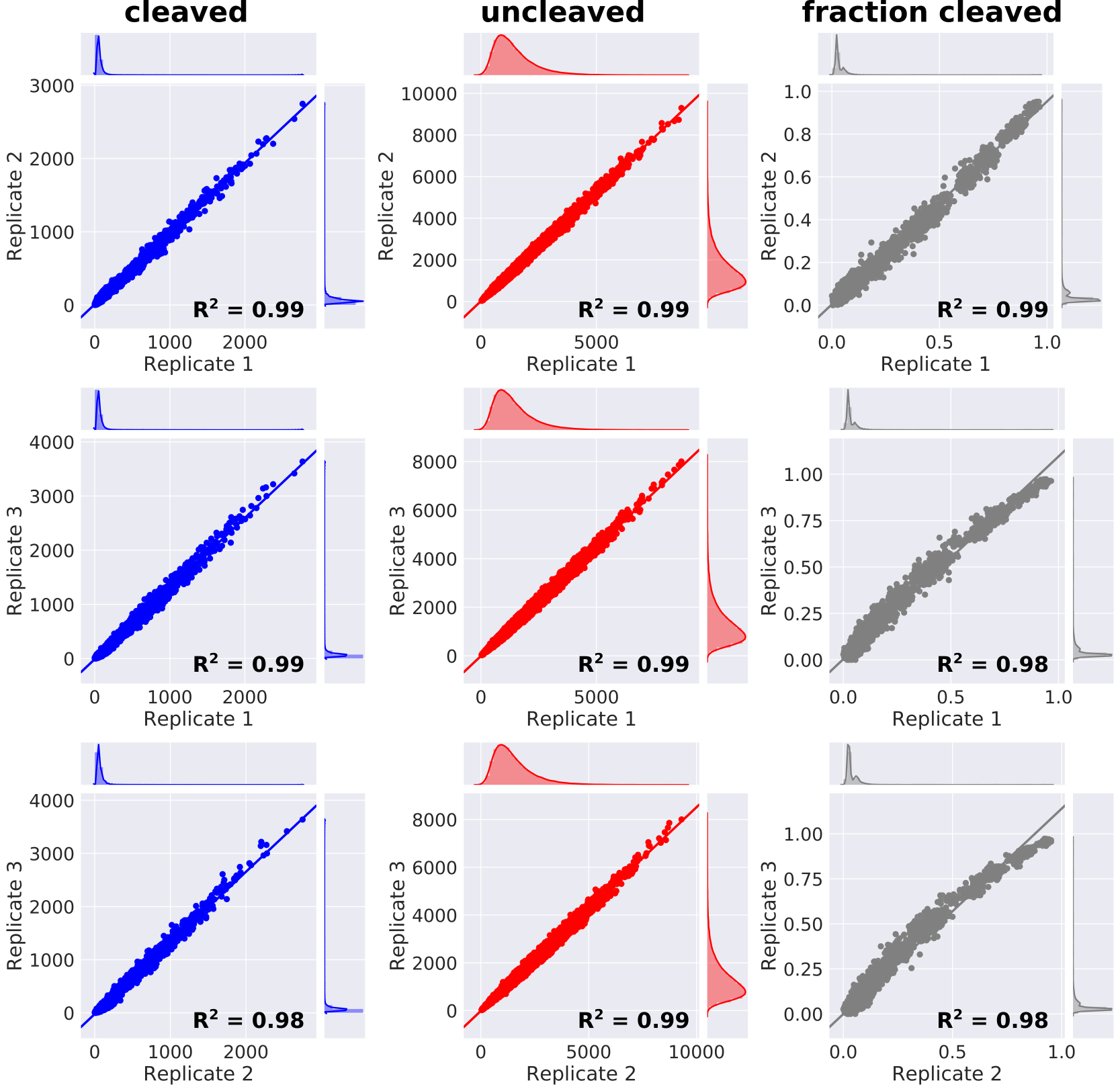
*

**Figure S3: Correlation of high-throughput sequencing replicates.**

Correlation of number of cleaved reads, uncleaved reads and fraction cleaved (ribozyme activity) for each of the three replicates. Each figure consists of 27,648 genotypes present in the phylogenetic fitness landscape library. Each data point for cleaved and uncleaved represents the frequency that a specific genotype was observed in a particular replicate (x-axis) vs. another replicate (y-axis). The fraction cleaved is a product of observed cleaved and uncleaved reads for each genotype. Sequence kernel density is also reported from each replicate in the jointplot.


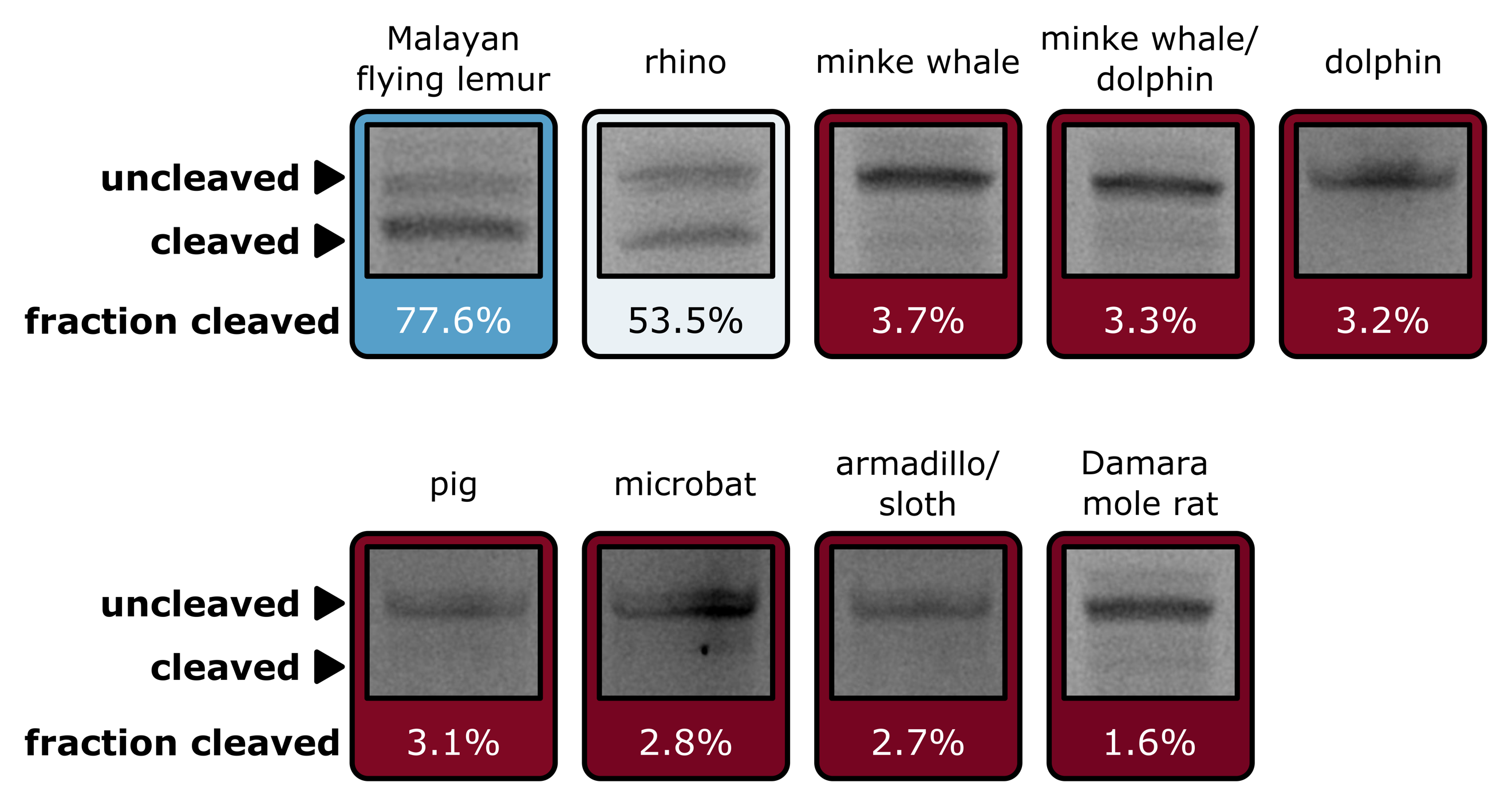


**Figure S4: Gel-based assay of intrinsic co-transcriptional self-cleavage activity of species not found in phylogenetic library.**

Gel-based assay depicting cleaved and uncleaved bands for nine species/nodes. Labels with two species separated by a slash, indicate ancestral nodes between the two species. Background is colored according to ribozyme activity (fraction cleaved) on the same color scale used in **Figure 2.** Reactions were performed under co-transcriptional conditions identical to the sequencing-based analysis (37 °C, 20 minutes)


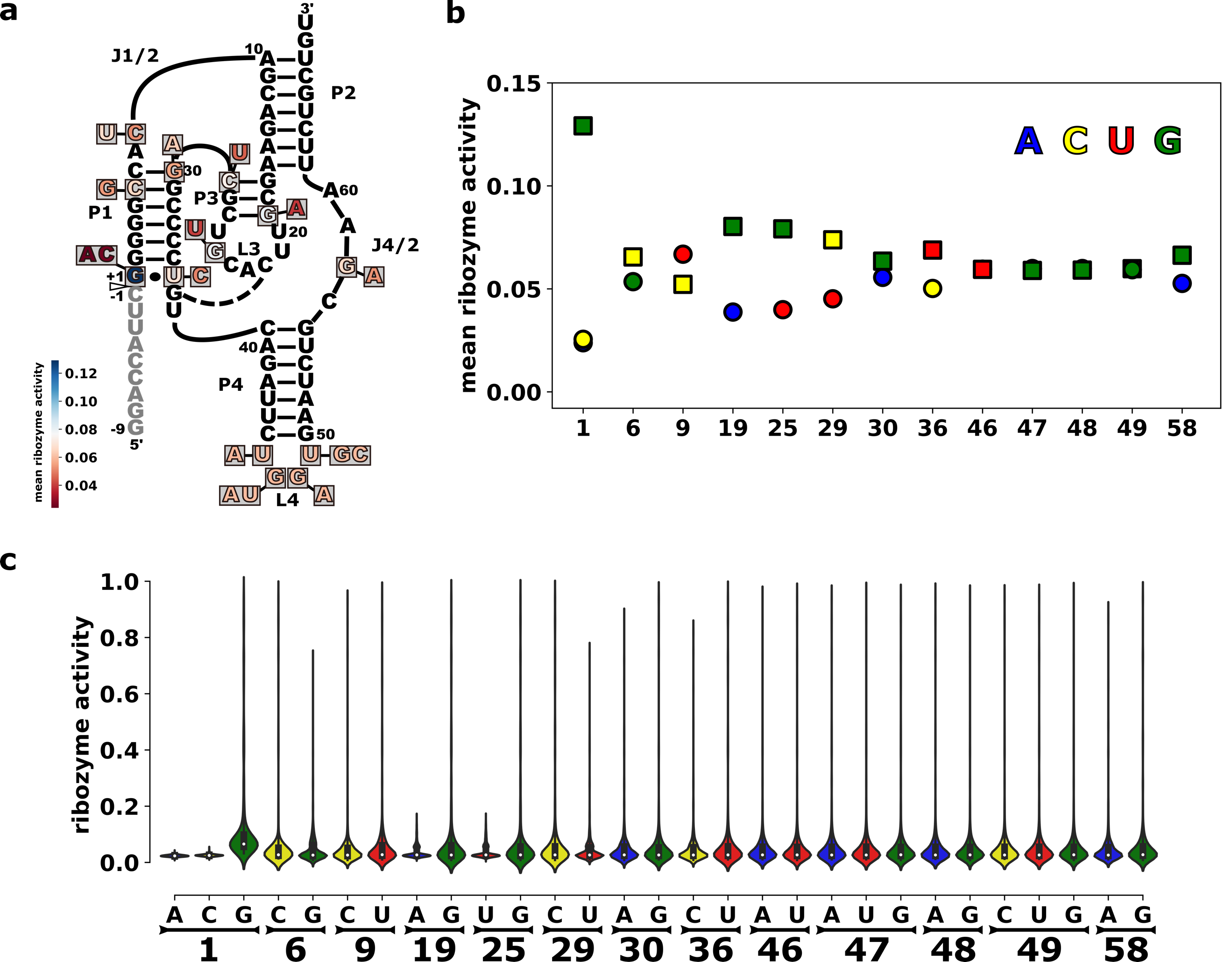


**Figure S5: The average effects of mutations at specific ribozyme positions regardless of mutational background.**

**(a)** Secondary structure of the *ancestral* CPEB3 ribozyme with mutational nucleotides indicated. Each nucleotide at each mutational position is colored by the mean activity of all ribozyme sequences with that nucleotide. Dashed line indicates a tertiary interaction. **(b)** The average of all genotypes with the corresponding mutation at the corresponding position. Position in the CPEB3 ribozyme are listed on the x-axis and mean ribozyme activity is on the y-axis. Color of node indicates the nucleotide at the position (A=blue, C=yellow, U=red, G=green). Square nodes represent nucleotides that are in the highly-conserved, highly-active *ancestral* sequence. **(c)** The distributions of ribozyme activities containing a given mutation at each position. Nucleotide and position in the CPEB3 ribozymes are indicated on the x-axis. Mean is indicated by white dot. Color corresponds to panel b.


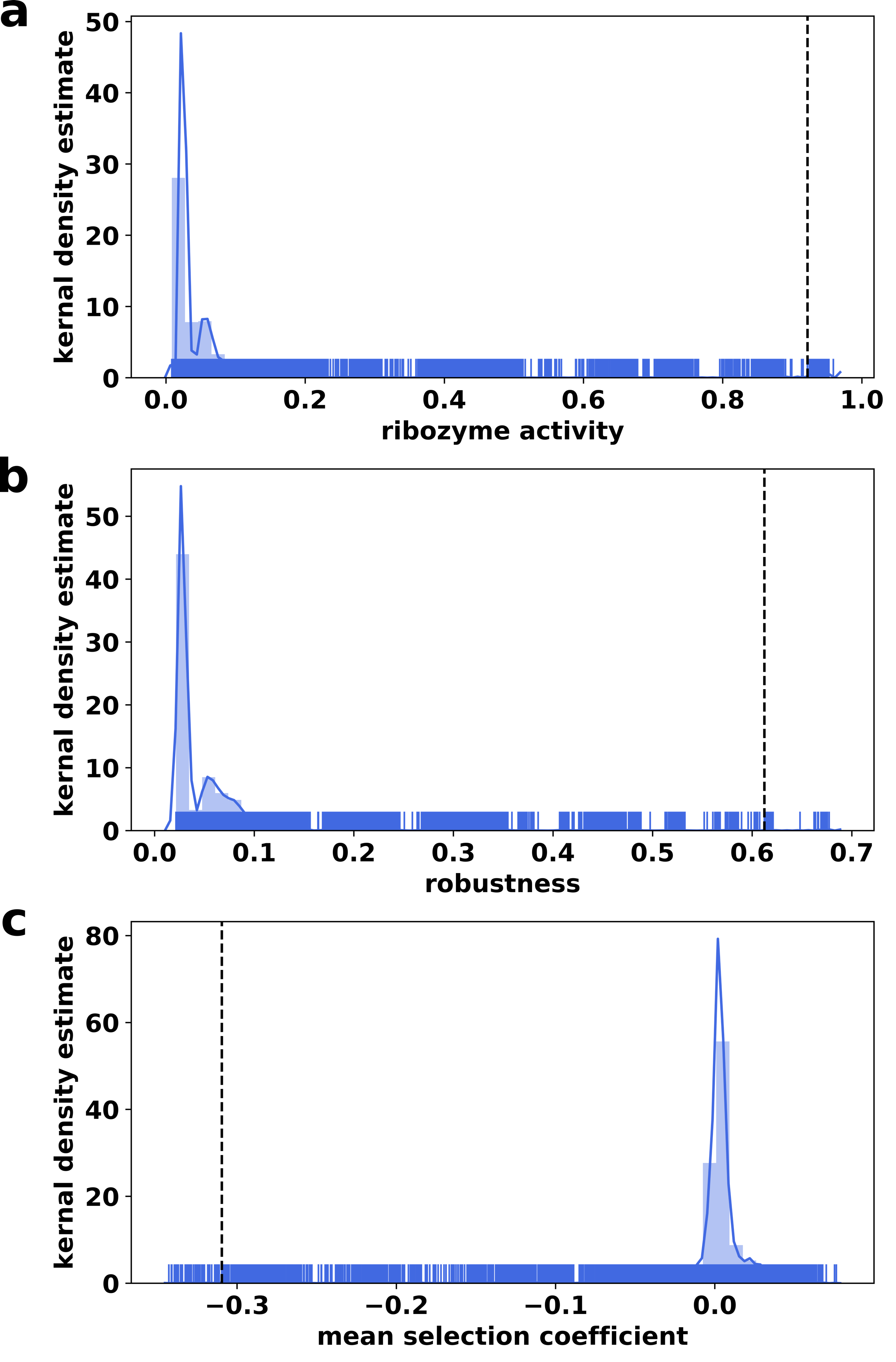


**Figure S6: Distributions of ribozyme activity, robustness and selection coefficients.**

**(a)** Distribution of ribozyme activity measured as fraction sequences cleaved during 20 minutes of transcription. The ribozyme activity of the *ancestral* sequence is indicated by the dotted line. **(b)** Distribution of mutational robustness. Robustness for each genotype was calculated as the average ribozyme activity of all mutational neighbors that differed by a single mutation. The robustness of the *ancestral* sequence is indicated by the dotted line. **(c)** Distribution of mean selection coefficients. Selection coefficients were calculated for each genotype as the difference in ribozyme activity between a genotype and its mutational neighbors that differed by a single mutation.


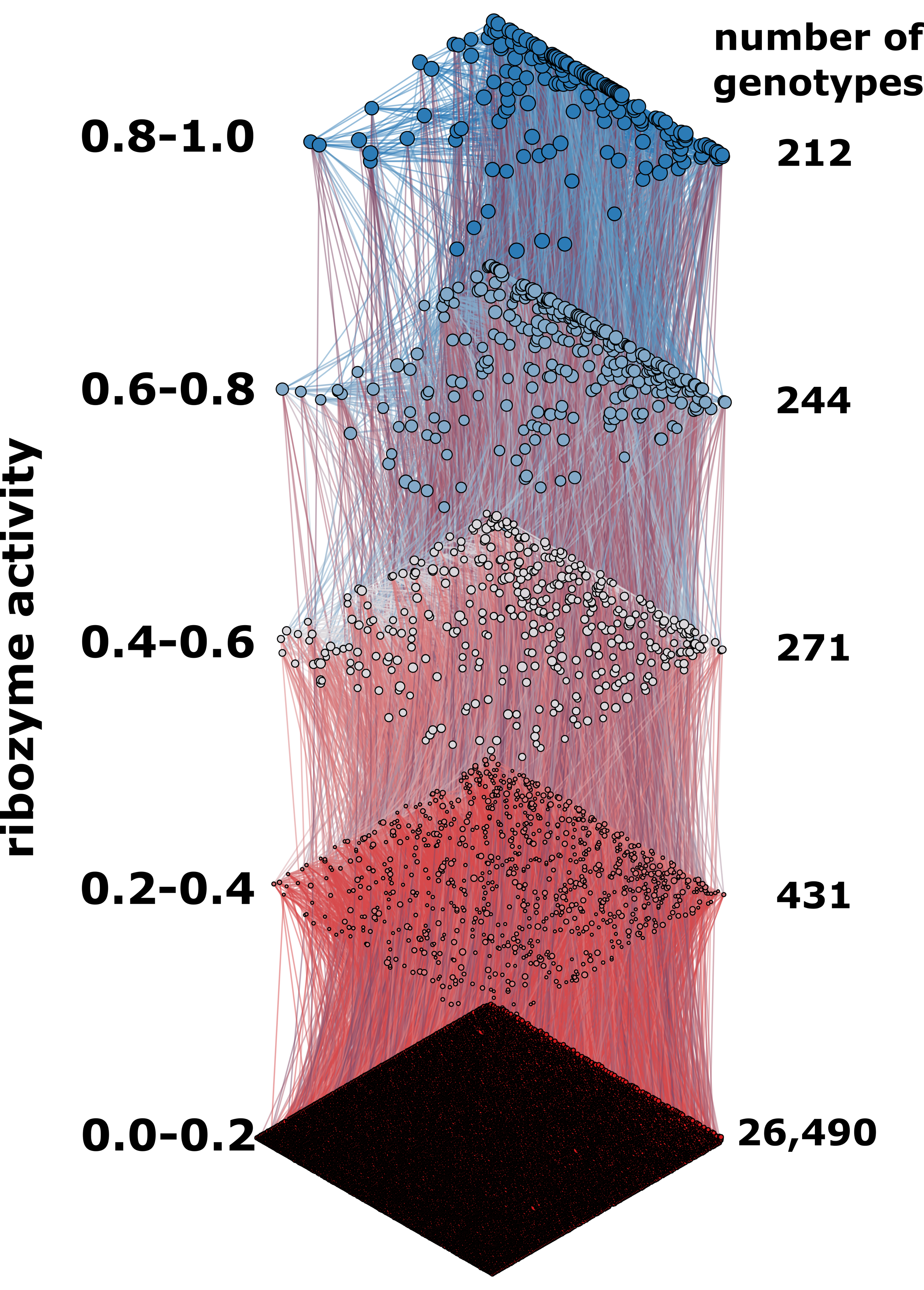


**Figure S7: Fitness landscape near the mammalian CPEB3 ribozyme sequences.**

Fitness landscape of the 27,648 sequences assessed by high-throughput sequencing assay. Each node indicates a unique sequence and nodes that differ by a single mutation are connected by an edge. The node size and color indicate the self-cleaving ribozyme activity of each sequence. For visualization purposes the fitness landscape is broken into five levels of ribozyme activity. The respective number of genotypes found in each of the five levels are shown.

**
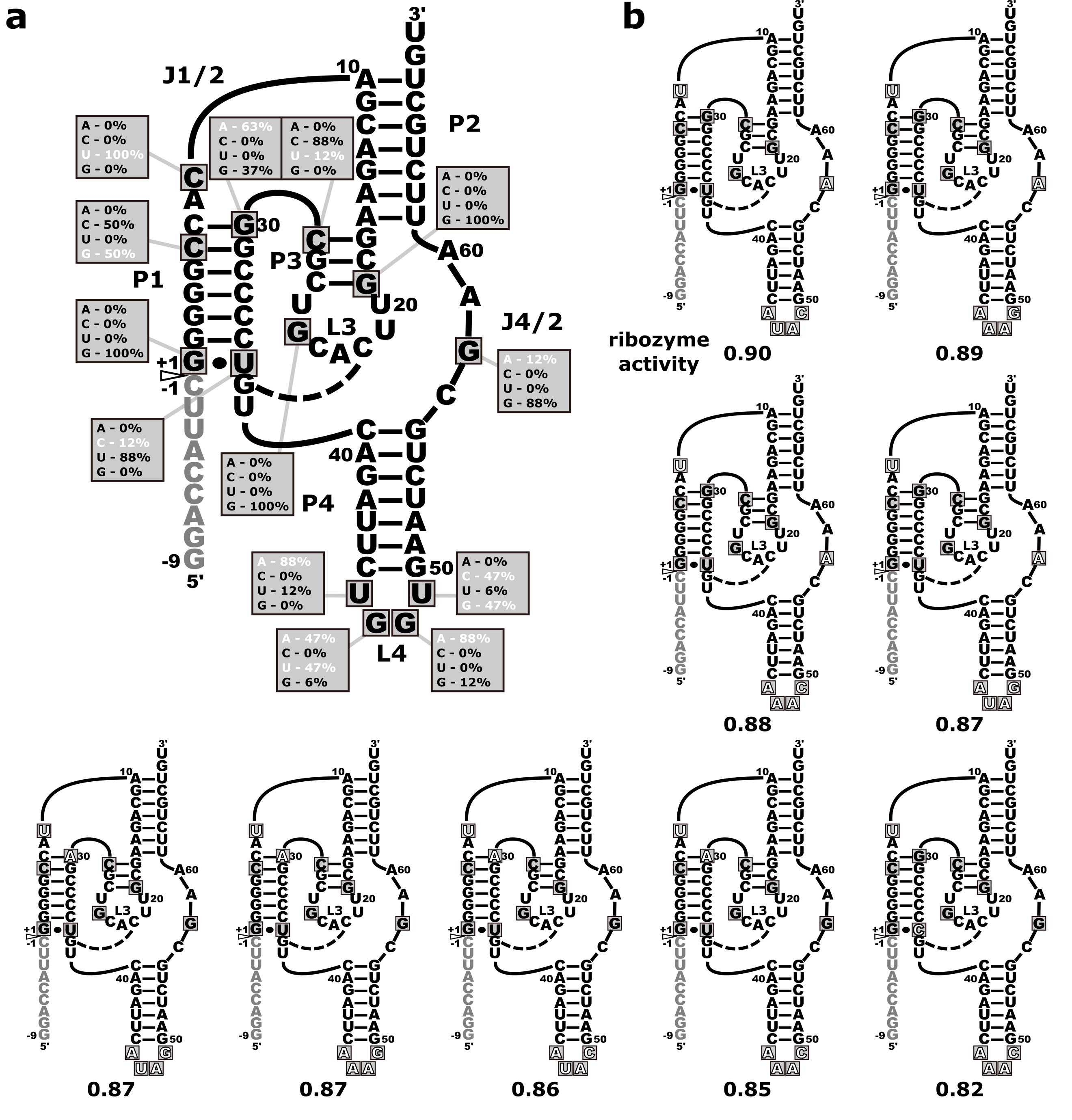
**

**Figure S8 Ribozyme sequence conservation of high-mutation, high-fitness ribozymes.**

**(a)** Secondary structure of the *ancestral* CPEB3 ribozyme with mutational nucleotides indicated. The conservation of each node in the 32 ribozyme sequences that contained at least 6 mutations, yet maintained >0.7 ribozyme fitness is shown. White lettering indicates mutations not found in the ancestral sequence. Dashed line indicates a tertiary interaction. **(b)** Secondary structure of top nine CPEB3 ribozyme sequences with at least 6 mutations. Ribozyme activity is indicated below each structure.

*
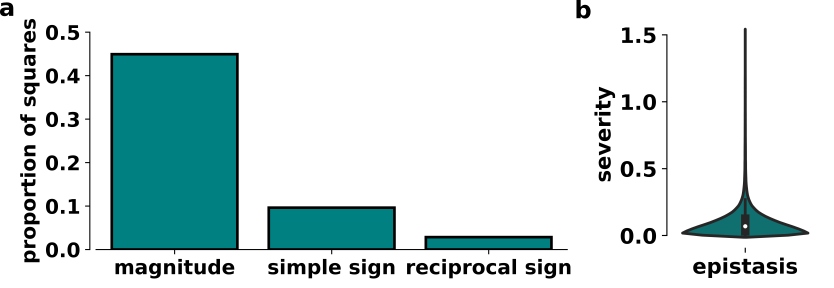
***Figure S9: Pairwise epistasis in the fitness landscape.**

**(a)** Relative prevalence of three classes of pairwise epistasis (Khalid et al. 2016; Aguilar-Rodríguez et al. 2017). Pairwise epistasis was assessed using mutational pairs or squares. Two precise mutations can occur in either order, and are represented in our landscapes by subgraphs of four connected genotypes. Each square consists of a starting reference genotype (wt), two single mutants and a double mutant. Magnitude epistasis occurs when the magnitude of a mutations effect on ribozyme activity depends on the genetic background of the mutation. Simple sign epistasis occurs when a single mutant has lower activity than the reference and the double mutant. Reciprocal sign epistasis occurs when both single mutants have lower activity than the reference and double mutant. **(b)** The distribution of severity of epistatic interactions in the fitness landscape. Epistatic values for each square of mutation pairs was calculated as *ε =* log_10_ (*W*_AB_**W*_wt_ / *W*_A_**W*_B_), where *W*_A_ and *W*_B_ are the fitness of RNA variants with a single mutation, *W*_AB_ is the fitness of the variant with both mutations, and *W*_wt_ is the fitness of the reference genotype.

**
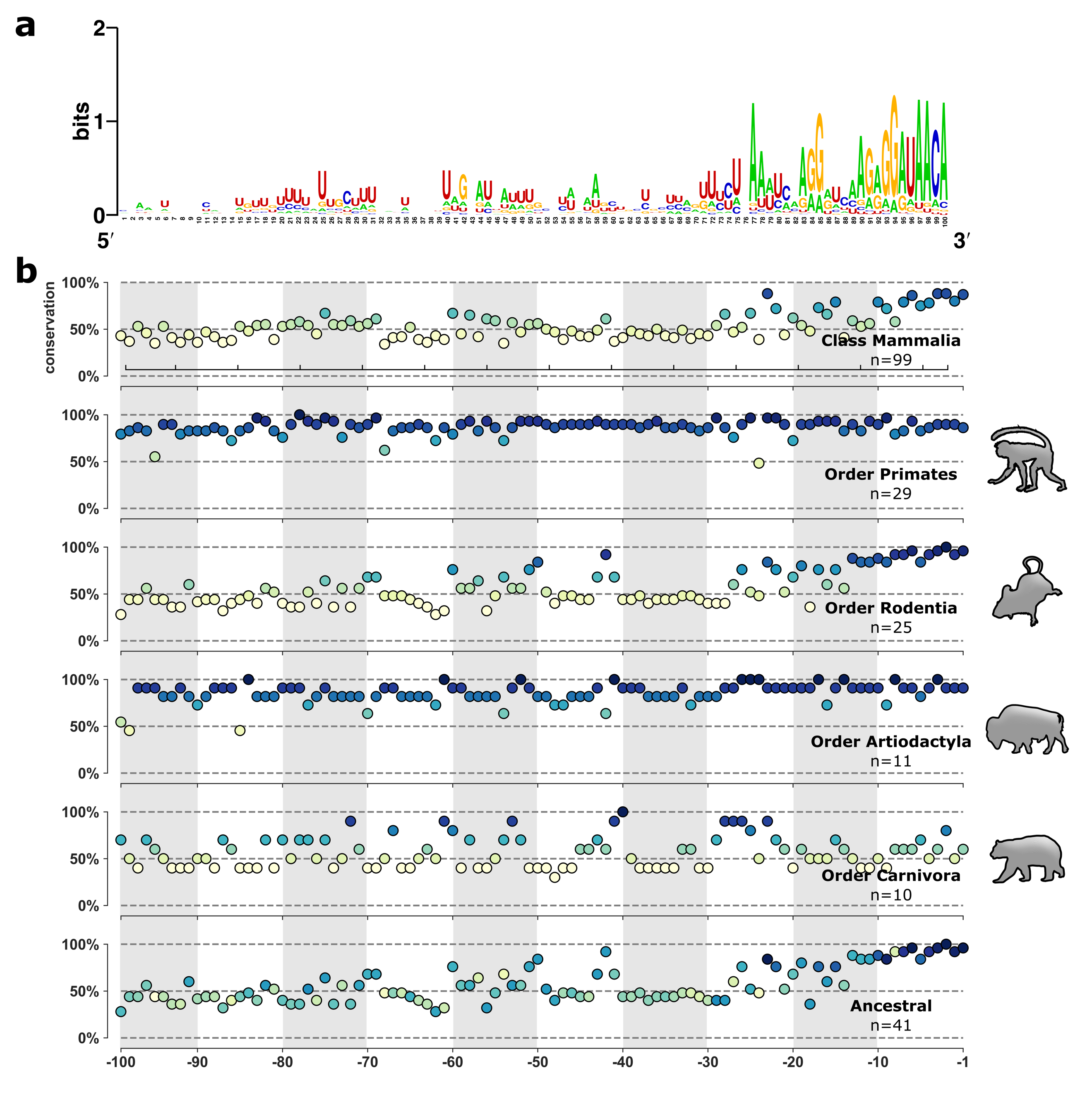
**

**Fig. S10 Conservation of the sequence upstream of the ribozyme.**

**(a)** Sequence logo of upstream sequence conservation for the 99 mammalian species generated using WebLogo (Crooks et al. 2004). **(b)** The conservation of nucleotide identities at the 100 nucleotides upstream of the ribozyme. Nucleotide positions (x-axis) are numbered relative to the cleavage site of the ribozyme. Taxonomic groups and the number of genomes analyzed *n* is indicated.

**
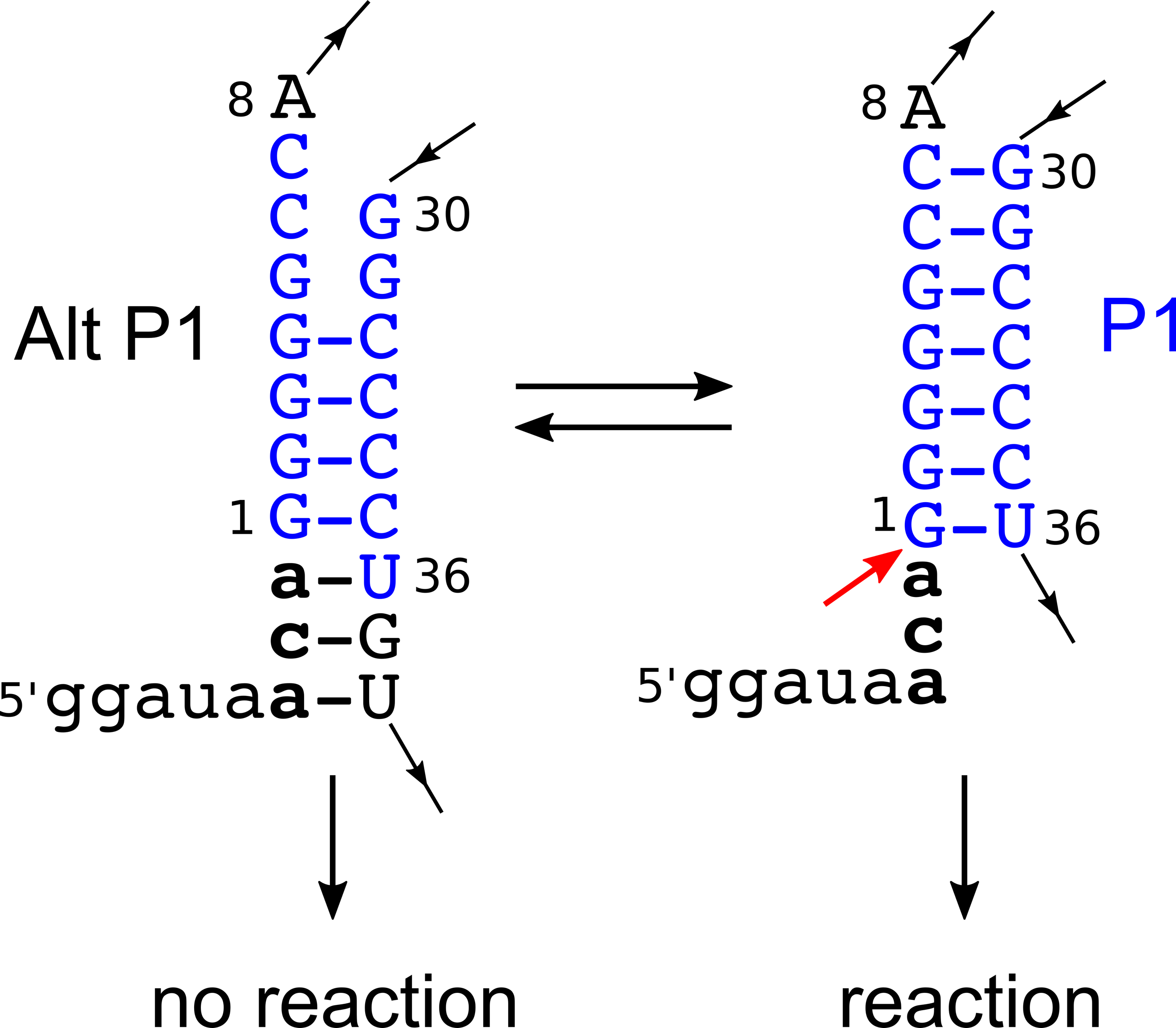
**

**Figure S11. Proposed equilibrium between Alt P1 and P1.**

The sequence of the ancestral ribozyme is shown with competing structures as predicted in the human ribozyme. The consensus upstream sequence is used. The conserved first three upstream nucleotides involved in the misfolded structure (Alt P1) are shown in bold. The nucleotides involved in the native P1 helix are colored blue. Nucleotides upstream of the cleavage site are written as lower case. The red arrow indicates the site of ribozyme self-cleavage. Nucleotides are numbered as in the main text.


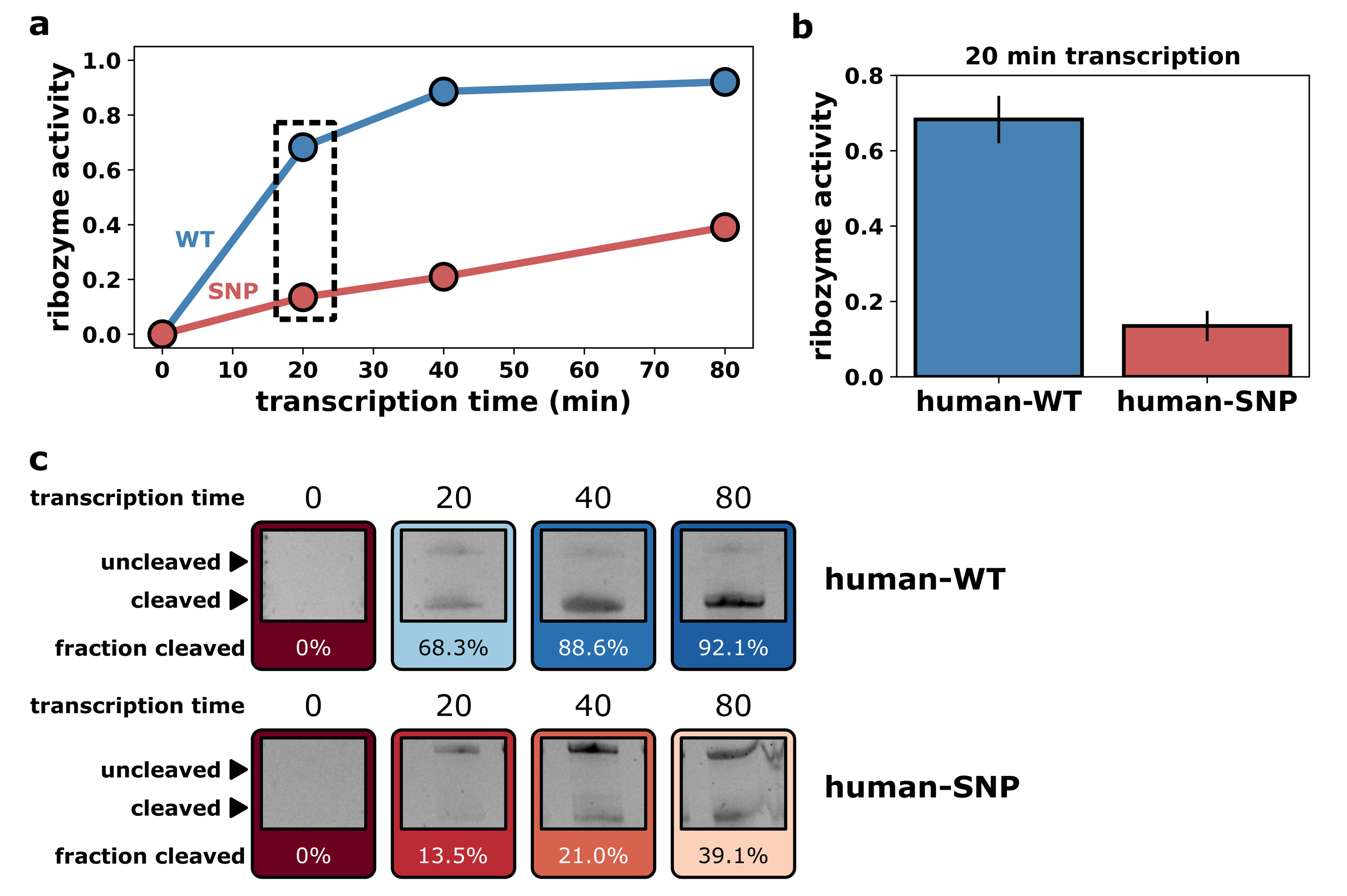


**Figure S12: Validation of intrinsic co-transcriptional self-cleavage activity of human-WT and human-SNP CPEB3 ribozymes.**

**(a)** Time-course transcription for ribozyme activity. Ribozyme activity is measured as the fraction cleaved at each timepoint (0, 20, 40 80 mins). Samples were allowed to co-transcriptionally cleave and were run on 10% denaturing polyacrylamide gel, visualized with GelRed (Biotium) and quantified by densitometry. Human-SNP is shown in red and human-WT is shown in blue. The two sequences only differ at the U36C SNP mutation. Reactions conditions were identical to the sequencing-based experiments (37°C, 10 mM MgCl_2_). The sequence upstream of the cleavage site is the same as in the sequencing based library. **(b)** Mean of four replicates for the 20-minute co-transcriptional assay. This timepoint is what was used for high-throughput sequencing. Error bars indicate standard deviation. **(c)** Gel-based assay for data presented in panel (a). Background is colored according to ribozyme activity (fraction cleaved) on the same color scale used in **Fig. 2**.

**
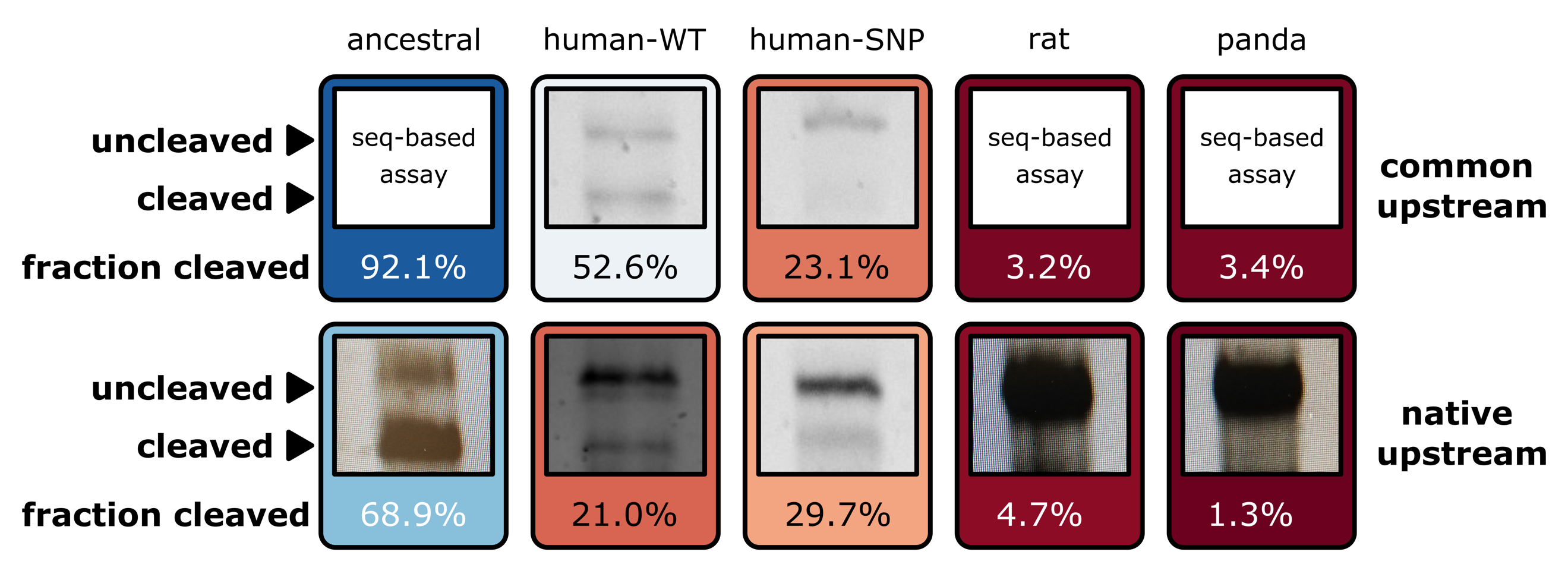
Figure S13: Comparison of co-transcriptional self-cleavage activity of native or common upstream sequence.**

The common upstream sequence refers to the sequence that was used in the sequencing-based experiments, which was the same for all ribozyme sequences.

**SUPPLEMENTAL DISCUSSION**

**Structural prediction of misfolding potential**

The sequence immediately upstream of the ribozyme could lead to misfolding if there is significant non-native base pairing potential. To evaluate the potential of the upstream sequence to cause misfolding we computationally predicted RNA structures using algorithms that allow for pseudoknotted structures (HotKnots, DOI: 10.1261/rna.1689910). We compared the predicted structures of a highly active sequence (elephant) and a low activity sequence (rat), with either their native upstream sequence or the sequence used in our library. Interestingly, the native upstream sequence of the elephant shows a stable misfolded structure with an alternative P1. This alternative P1 structure was previously reported for the human CPEB3 ribozyme, where it was demonstrated that this structure slowed the observed self-cleavage rate. This alternative structure was not predicted for the elephant sequence with the library upstream sequence. We observed that the elephant sequence showed lower self-cleavage with the native upstream sequence, analyzed by PAGE, than was detected in our sequencing experiments with the library upstream, supporting the misfolding potential of the elephant ribozyme. The rat sequence only differed from the elephant sequence at the G1 nucleotide. The most stable predicted structures include a native-like fold for both the native and library upstream sequences. This indicates that the low activity of the rat ribozyme sequence is not caused by alternative structures, but from more subtle structural differences around the active site caused by the G1A mutational difference. The library upstream sequence is predicted to form base pairs with L4 of the native structure (“native structure plus”), but our results suggest that these base pairs do not inhibit ribozyme activity. In addition, the predicted structures suggest that there is a competing structure between the first nucleotide (C-1) upstream of the cleavage site in the library upstream sequence and the L3 pseudoknot of the native structures. However, the self-cleavage activity of the elephant and rat sequences was similar with both upstream sequences, suggesting that this interaction does not have a large effect on activity. The native upstream sequence in the rat has more predicted interactions with the first two upstream nucleotides (C-1 and A-2). Together, this analysis further confirms that the upstream sequence used in our sequencing experiments prevents a known misfolding potential for many of the ribozymes, revealing the intrinsic activity of the ribozyme sequences.

**Elephant native upstream**

GGAAAACAGGGGGCCACAGCAGAAGCGUUCACGUCGCGGCCCCUGUCAGAUUCUGGUGAAUCUGCGAAUUCUGCU

The top three structures from HotKnots are shown in dot bracket notation and using Pseudoviewer (below). Most stable structures are shown from left to right.

**5'- .....((((((([[[[.{{{{{{{(((.......)))..)))))))(((((((]]]])))))))....}}}}}}} -3' -20.93 kcal/mol**

**5'- ........(((((((..[[[[[[[(((.......))))))))))..(((((((....)))))))....]]]]]]] -3'    -20.05 kcal/mol**

**5'- .....(((((((.....[[[[[[[(((.......)))..)))))))(((((((....)))))))....]]]]]]] -3'    -18.83 kcal/mol**

Alt P1

native structure


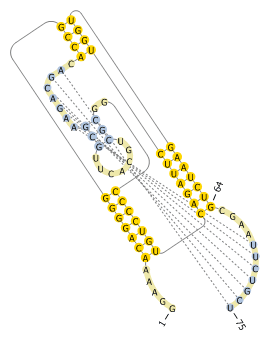

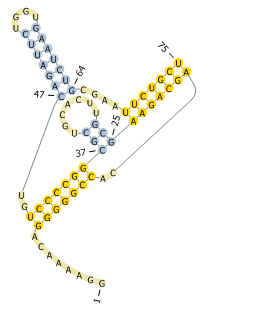

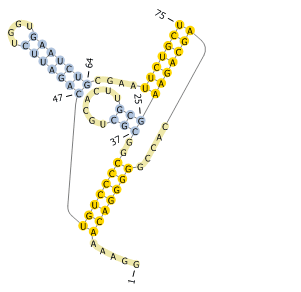


**Elephant library upstream (Fraction Cleaved = 0.92)**

GGACCAUUCGGGGGCCACAGCAGAAGCGUUCACGUCGCGGCCCCUGUCAGAUUCUGGUGAAUCUGCGAAUUCUGCU

5'- ..((((..((((((((..[[[[[[[(((.......))))))))))).[[[[[[[))))]]]]]]]....]]]]]]] -3' -22.45 kcal/mol

5'- ........((((((((..[[[[[[[(((.......))))))))))).(((((((....)))))))....]]]]]]] -3'    -20.79 kcal/mol

5'- ..((((..((((((((...[[[[[.(((.......))))))))))).{{{{...))))...]]]]]....}}}}.. -3'    -19.85 kcal/mol

native structure plus


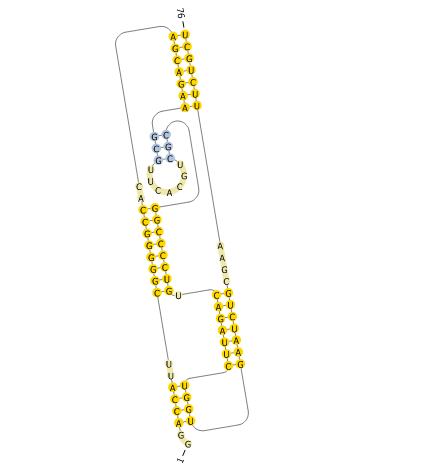

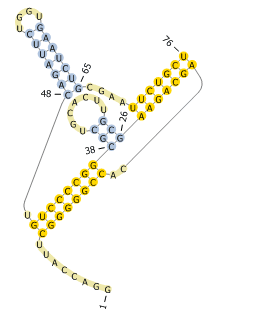

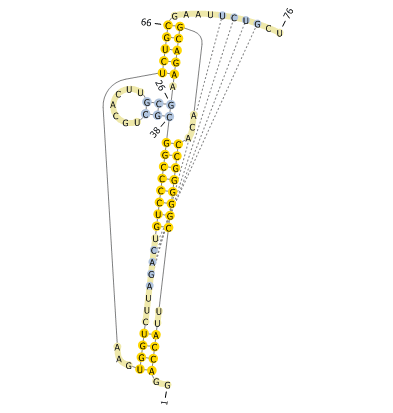


native-like structure

**Rat native upstream**

GAGGAUAACAGGGGCCACAGCAGAAGCGUUCACGUCGCGGCCCCUGUCAGAUUCUGGUGAAUCUGCGAAUUCUGCU

**5'- .......(((((((((..[[[[[[[(((.......))))))))))))(((((((....)))))))....]]]]]]] -3'    -21.99 kcal/mol**

**5'- .(((((.(((((((((.........(((.......))))))))))))(((((((....)))))))...)))))... -3'    -19.28 kcal/mol**

**5'- .......(((((((((.........(((.......))))))))))))(((((((....)))))))........... -3'    -18.84 kcal/mol**

native-like structure


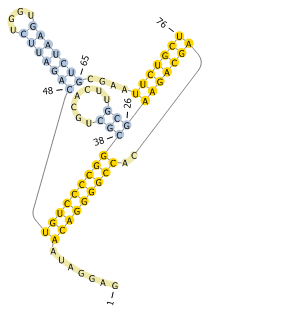

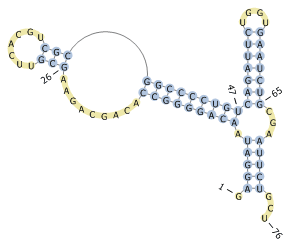

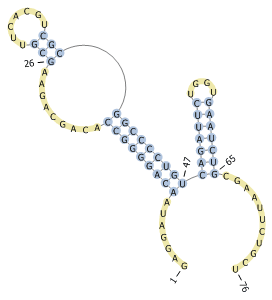


**Rat library upstream (fraction Cleaved = 0.03)**

GGACCAUUCAGGGGCCACAGCAGAAGCGUUCACGUCGCGGCCCCUGUCAGAUUCUGGUGAAUCUGCGAAUUCUGCU

5'- ..((((..((((((((..[[[[[[[(((.......))))))))))).[[[[[[[))))]]]]]]]....]]]]]]] -3' -23.14 kcal/mol

5'- ........((((((((..[[[[[[[(((.......))))))))))).(((((((....)))))))....]]]]]]] -3' -21.49 kcal/mol

5'- ..((((..((((((((...[[[[[.(((.......))))))))))).{{{{...))))...]]]]]....}}}}.. -3' -20.54 kcal/mol

native structure plus


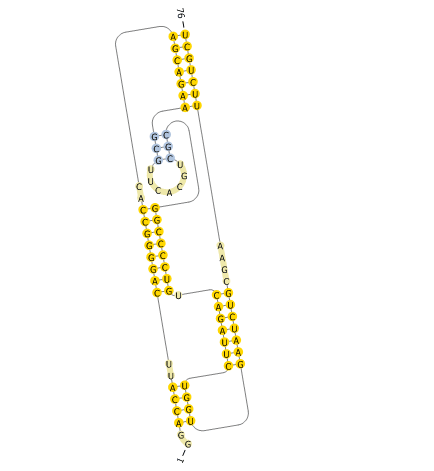

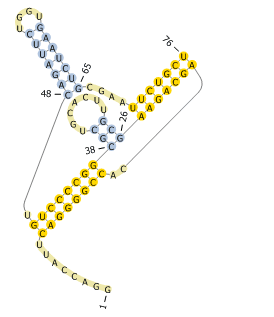

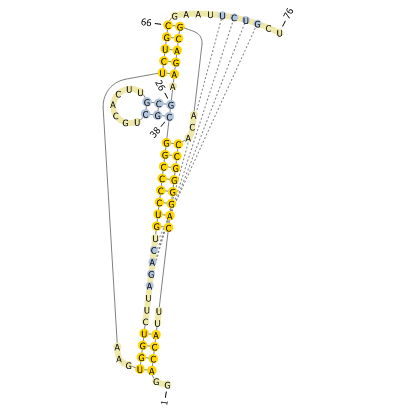


native-like structure

**Human native upstream**

GGAUAACAGGGGGCCACAGCAGAAGCGUUCACGUCGCAGCCCCUGUCAGAUUCUGGUGAAUCUGCGAAUUCUGCU

**5'- .....((((((([[[[.{{{{{{{(((.......)))..)))))))(((((((]]]])))))))....}}}}}}} -3'    -20.82 kcal/mol**

**5'- .....(((((((.....[[[[[[[..[[[[[[.{{{{{{))))))).........]]]]]]}}}}}}.]]]]]]] -3'    -19.16 kcal/mol**

**5'- .....(((((((.....[[[[[[[(((.......)))..)))))))(((((((....)))))))....]]]]]]] -3'    -18.72 kcal/mol**


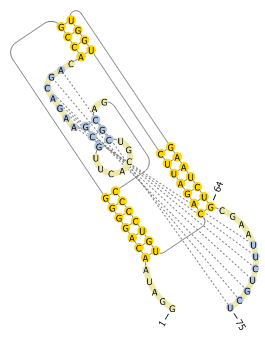

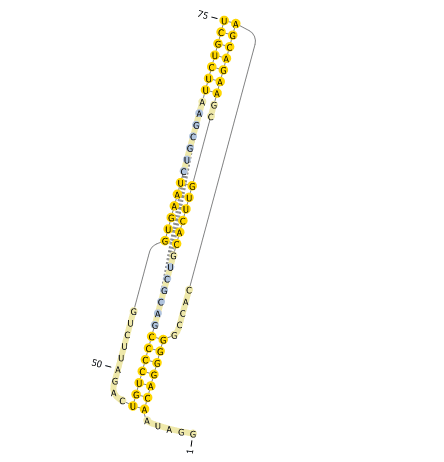

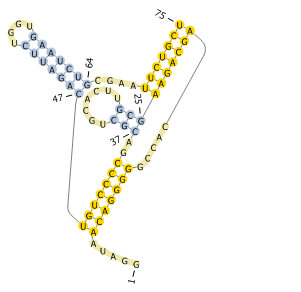


native-like structure

Alt P1

**Human library upstream**

GGACCAUUCGGGGGCCACAGCAGAAGCGUUCACGUCGCAGCCCCUGUCAGAUUCUGGUGAAUCUGCGAAUUCUGCU

**5'- ..((((..(((((((...[[[[[[[(((.......))).))))))).[[[[[[[))))]]]]]]]....]]]]]]] -3'    -20.41 kcal/mol**

**5'- ........(((((((...[[[[[[[(((.......))).))))))).(((((((....)))))))....]]]]]]] -3'    -18.75 kcal/mol**

**5'- .........(((([[[[.{{{{{{{(((.......)))..))))...(((((((]]]])))))))....}}}}}}} -3'    -18.01 kcal/mol**

native structure plus


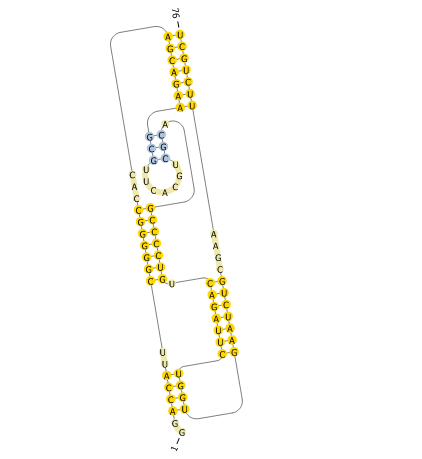

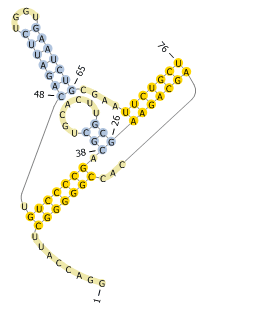

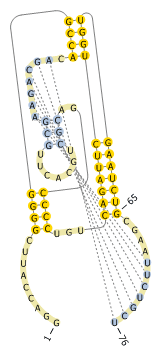


native-like structure
