# Supplementary material for: Experimental Resurrection of Ancestral Mammalian CPEB3 Ribozymes Reveals Deep Functional Conservation
Source: Mol Biol Evol. 2021 Mar 15;38(7):2843–53. doi: 10.1093/molbev/msab074 (PMC8233481; doi:10.1093/molbev/msab074)
Supplement: msab074_Supplementary_Data [file msab074_supplementary_data.zip › Data File S1 - Sequencing_Data.pdf]

Genotype	fitness	delta	Replicate 1 fitness	Replicate 1 cleaved	Replicate 1 uncleaved
CGTATCACATACG	0.025	0.004	0.021	34	1557
AGCGGTATTGAGG	0.021	0.003	0.022	21	937
CGTGGCGCTAGGA	0.032	0.014	0.046	20	417
CCTGGTATTGGCA	0.026	0.003	0.023	27	1147
CGCAGCGCTTATA	0.024	0.005	0.02	30	1505
CCCATTGCTGACG	0.026	0.005	0.033	72	2138
AGTGTTACATATA	0.021	0.003	0.017	38	2143
CCTGTCGTAAACA	0.022	0.006	0.014	41	2805
CCCATTATTAGTA	0.026	0.004	0.021	70	3334
CCCGTCGCAAACG	0.027	0.004	0.021	53	2440
AGTATCATTAGTG	0.025	0.004	0.024	27	1080
CCTATTATAAATA	0.03	0.003	0.027	159	5666
CCCGTTGTAGGCA	0.023	0.005	0.019	33	1693
AGTATTATTTGCG	0.026	0.002	0.026	35	1286
GCCAGCGTTGACA	0.046	0.01	0.049	81	1566
GGCATTATTGGGA	0.049	0.009	0.036	43	1149
CCTATCGCTAATG	0.028	0.001	0.03	54	1772
ACCGTCATTTACG	0.026	0.002	0.027	60	2190
ACCGTCACAGGCG	0.029	0.001	0.027	47	1707
CCTAGCATAGACG	0.025	0.005	0.019	37	1883
CCTGTTGTTGACA	0.023	0.005	0.017	28	1665
CGCGGCACTGGGG	0.023	0.003	0.026	13	490
CCCGGTGTTGATA	0.024	0.003	0.027	44	1600
ACTGTCGCTGGGA	0.025	0.005	0.027	24	860
ACTATTACTAATA	0.024	0.001	0.024	125	5123
ACCGTTGTTTGGA	0.021	0.005	0.028	31	1089
GCCAGCATTGATA	0.051	0.008	0.041	102	2389
GGTATTATTGGTA	0.093	0.02	0.066	71	1007
CGCGGTGCTGACG	0.019	0.007	0.025	23	899
CCCATTATATGTG	0.025	0.001	0.026	64	2440
GGCAGCGTAAACA	0.068	0.011	0.064	137	2014
GCCGTCACATATA	0.052	0.005	0.046	103	2155
CCCATTATAAATG	0.025	0.002	0.026	117	4374
ACCAGCACAAGTA	0.02	0.002	0.018	85	4521
ACCGTTGCTAGCA	0.023	0.002	0.024	56	2261
AGCAGCGTTAGTA	0.025	0.003	0.023	41	1708
AGCAGCATAAGGA	0.02	0.003	0.019	44	2331
CGTAGTGCTGACA	0.024	0.004	0.029	27	917

CCCGGCGCTTGTA	0.018	0.004	0.017	24	1369
GCCAGCGCAAACA	0.054	0.004	0.054	122	2153
GCCATTGTAGGTG	0.047	0.01	0.034	34	977
ACTATTACATGGA	0.023	0.001	0.022	52	2341
GCTGGTGCAAGGA	0.115	0.016	0.131	86	571
AGCATCGCTAGGG	0.02	0.002	0.023	25	1083
CGTAGTGTTTATG	0.027	0.004	0.027	18	641
GGTGGCATTTGTA	0.468	0.059	0.441	371	471
ACTGGCACAAATA	0.023	0.001	0.025	72	2833
AGTAGTGTTTGGG	0.016	0.004	0.01	5	497
GGCAGCGCAAACA	0.052	0.004	0.048	101	2024
CCTGTCGTAAGTG	0.024	0.001	0.024	26	1069
ACCATCGTATACG	0.025	0.001	0.023	61	2591
GCTATTATAGATG	0.067	0.01	0.053	96	1710
CCCGGTGCATATG	0.022	0.006	0.016	21	1282
GCTATTATTGACG	0.061	0.002	0.06	104	1641
GCCGGTACTGATG	0.064	0.002	0.062	60	902
CCCGGCATATACA	0.023	0.003	0.022	77	3472
CCTGGTACTGGCA	0.022	0.004	0.017	22	1306
CGTAGCACAAGGA	0.029	0.003	0.027	30	1082
AGTGTTATATGTA	0.022	0.002	0.019	32	1622
ACCATCGTATGTA	0.021	0	0.02	52	2498
AGCGTTACTGGCA	0.019	0.002	0.02	31	1507
CCTGGCGTTTAGA	0.023	0.006	0.031	33	1033
ACTGTCGTTGAGG	0.016	0.002	0.018	12	662
CGCGGTACAAACG	0.021	0.005	0.023	41	1758
CGTAGCATATGCA	0.026	0.005	0.034	47	1340
AGCAGCATAAAGA	0.021	0.003	0.017	59	3488
GGCGTTACTAGTA	0.064	0.006	0.057	70	1152
ACTGGCGCAGATA	0.034	0.005	0.035	33	910
GCCGTTGTTTGTG	0.057	0.006	0.052	36	662
AGTATCGTTAGTG	0.028	0.004	0.027	25	901
GGTATTACTAACG	0.106	0.007	0.103	139	1214
GCCGTTGTATATG	0.052	0.012	0.037	40	1054
ACCATCATTGACG	0.025	0.003	0.022	59	2669
AGTAGCGCTTACG	0.019	0.008	0.018	16	851
GCCAGCACATGTG	0.049	0.006	0.042	52	1194
GGCATTATTTACG	0.064	0.007	0.059	100	1589
AGCGTTACTGACA	0.02	0.003	0.017	39	2320
GCCATTACTAAGG	0.046	0.005	0.053	91	1620

AGCATCATTTACA	0.021	0.001	0.022	88	3875
ACCATCATATAGA	0.023	0.003	0.021	86	4020
ACCATTGCTTGTA	0.023	0.002	0.021	53	2418
CCTATTGCAGGCA	0.023	0.001	0.022	43	1910
ACCATTACAAACG	0.023	0.005	0.027	160	5812
AGTATTATTGAGA	0.024	0.003	0.027	56	1995
CCTATTATATGGA	0.027	0.005	0.02	45	2156
ACTATTGTATACA	0.024	0.003	0.021	78	3711
GGCATTGCAAATA	0.055	0.005	0.049	110	2132
GGTATTACATGTA	0.08	0.009	0.081	106	1198
GGCATTACTTATG	0.062	0.002	0.06	83	1310
AGCAGCATAAGTA	0.02	0.002	0.023	71	3051
ACTGGTATTTATA	0.028	0.002	0.025	52	2054
GCCATTATTTATG	0.057	0.002	0.059	133	2104
ACCAGCACTAGCA	0.021	0.001	0.019	75	3835
CCCATCATATACA	0.026	0.001	0.026	144	5343
AGTGGTGCTAACA	0.02	0.001	0.02	26	1305
CCCAGTATATACA	0.025	0.002	0.026	121	4528
AGTATTATAAACA	0.021	0.003	0.019	89	4500
GGCGGTACATGTA	0.071	0.013	0.059	66	1047
CCCATCATTTAGA	0.026	0.004	0.025	75	2870
CCCATCATTTGCG	0.027	0.005	0.02	41	2041
CCCATTATTGGCA	0.023	0.004	0.021	54	2539
AGCATCGTATAGG	0.02	0.002	0.018	29	1599
CCTAGCACAAATA	0.025	0.002	0.025	94	3711
AGTGTTATTTGCG	0.024	0.007	0.026	22	833
ACCGGCGCATGTG	0.024	0.006	0.033	35	1030
AGCGGCATTGGCA	0.025	0.002	0.022	26	1151
GCCGTCGCTTATA	0.057	0.007	0.056	65	1086
GCCGTTACTAGCA	0.054	0.003	0.053	83	1491
CGCGGTACTGACA	0.026	0.003	0.025	47	1860
GGCATTGCAGACA	0.055	0.005	0.061	107	1639
CGCATCATAGAGA	0.027	0.005	0.022	50	2243
ACTGTTACTGATG	0.029	0.004	0.024	33	1371
CCCGGCATAAATA	0.028	0.002	0.025	101	3866
CGCAGCACATGTA	0.023	0.002	0.021	37	1710
AGCGTCGTTGATG	0.027	0.006	0.033	40	1184
CCTAGTACATGTA	0.027	0.004	0.022	49	2151
CCTATCGCTAAGG	0.028	0.008	0.028	38	1313
GGCATTGCTAATG	0.069	0.009	0.063	82	1222

CCTGTCATAAATG	0.023	0.001	0.023	54	2306
GCTATTGTATATG	0.059	0.008	0.057	82	1359
ACTGTTATTGACA	0.023	0.005	0.016	40	2421
ACCATCATAAAGA	0.024	0.003	0.021	107	5099
GGCGGTGCTAACG	0.052	0.01	0.043	38	843
CGTGTCATAAAGA	0.027	0.002	0.025	41	1618
ACCGGTGCAAACA	0.023	0.005	0.022	83	3745
CCTATTGCTTACG	0.027	0.004	0.024	45	1840
CGCAGTACAAGCG	0.026	0.003	0.028	45	1557
CCCATTATAAAGG	0.025	0.003	0.026	84	3135
CCCATTACTTAGA	0.024	0.002	0.026	71	2670
ACCAGCGTATAGA	0.025	0.003	0.02	43	2081
CGTATCGTTTAGG	0.022	0.006	0.015	12	772
GCCGGTGTTAGTA	0.175	0.017	0.173	174	830
ACCGGTACAAGCA	0.025	0.001	0.027	105	3821
CCCAGTACATGTA	0.022	0.003	0.02	57	2751
GGCATCGCAGACG	0.107	0.001	0.107	138	1152
ACTAGCGCTGATG	0.027	0.006	0.019	25	1294
ACCATCACTGATA	0.022	0.002	0.025	125	4973
GGTGGCACTAAGA	0.21	0.014	0.202	173	685
AGTAGCGTTTAGA	0.028	0.003	0.027	25	896
CCCATTATTGACA	0.024	0.001	0.022	93	4089
CGCATTACATATA	0.026	0.006	0.017	49	2830
GCTATCGTTTGCG	0.057	0.005	0.056	49	820
ACCAGCGCAAACG	0.021	0.003	0.018	55	2958
CCCGGCGTTAGCA	0.025	0.002	0.024	43	1755
GGTATTGCATACA	0.084	0.006	0.077	102	1220
GCCAGTGTTGACG	0.051	0.007	0.042	52	1181
GCCGTTACTGGCA	0.059	0.006	0.051	68	1267
AGCATCACAAATA	0.023	0.005	0.018	88	4926
CCCGTTATTAAGA	0.025	0.003	0.023	63	2712
CCTGTTACTAAGG	0.024	0.001	0.022	27	1205
AGTGGCGTTAATG	0.027	0.01	0.02	9	431
ACCGGTGCTTGTG	0.022	0.002	0.023	23	957
CCTGTCGCTAAGG	0.029	0.004	0.025	24	929
CGCATTATAAATG	0.023	0.001	0.024	75	3054
ACTGTCGTAAACA	0.025	0.003	0.028	69	2408
CGTGGCACAAGCG	0.024	0.007	0.018	14	782
CGCATTACTAACG	0.025	0.005	0.018	41	2176
AGCATTACAGACA	0.024	0.002	0.022	84	3752

GGTATTACATGGG	0.1	0.02	0.102	67	593
ACCGGCATTAGCG	0.027	0.007	0.028	44	1513
GCCAGCACAAATG	0.048	0.001	0.047	105	2150
AGTAGCATATGCG	0.024	0.003	0.029	31	1051
AGTAGCATTAGTA	0.024	0.006	0.029	41	1353
AGCGTCACAGGGA	0.024	0.003	0.019	24	1209
ACCGTTACTAACA	0.024	0.003	0.026	137	5143
CCCGTCGTAGGTA	0.027	0.001	0.028	47	1633
GCCAGTACAGGCA	0.049	0.005	0.044	83	1813
CCTAGCATATATA	0.025	0.002	0.022	63	2763
CCTATCGCAAATA	0.026	0.001	0.024	88	3547
AGCAGTACTGATA	0.021	0.002	0.021	56	2671
ACCGTCGCAAGCA	0.021	0.003	0.018	47	2556
AGTGGTGTAAATA	0.022	0.008	0.023	38	1631
CCTAGCACAAACA	0.021	0.002	0.019	76	3965
GCTATCATTGATG	0.064	0.007	0.058	87	1402
CCCAGCACTAATG	0.026	0.003	0.026	69	2635
CCTAGCATAGGGA	0.044	0.012	0.044	58	1256
CCTGGCACAGGTA	0.026	0.005	0.023	31	1295
GCCAGTACTGGCA	0.048	0.005	0.051	82	1536
CCTATCACAGAGA	0.026	0.004	0.02	51	2481
CGCATCATTGATG	0.029	0.001	0.03	55	1795
CCTGGTATAAGTA	0.028	0.005	0.023	43	1823
AGTAGTACAGGCG	0.027	0.005	0.023	22	948
ACTAGCATTGAGA	0.027	0.005	0.026	50	1871
AGCATTATATACA	0.021	0	0.021	109	5055
GGCGGTGTAGATG	0.089	0.007	0.084	61	664
AGTATCACTAACG	0.027	0.004	0.021	35	1650
ACCGGCGTATGTA	0.022	0.004	0.024	33	1327
ACCGGTACTTATG	0.021	0.001	0.023	53	2244
AGCAGCGCTAAGG	0.028	0.004	0.023	27	1150
CCTAGTGTTAACA	0.026	0.003	0.022	64	2781
GGTAGTACTTACA	0.065	0.018	0.051	59	1094
ACCAGCGTTTATA	0.024	0.004	0.018	43	2336
CCTAGCATAAAGA	0.028	0.001	0.028	76	2686
CCTAGTGTAAATA	0.023	0.004	0.018	60	3312
GGCAGCGTAGACA	0.068	0.008	0.061	98	1514
ACCGTCGCTGAGA	0.021	0.002	0.021	34	1603
AGCGGCATTTATA	0.024	0.002	0.027	42	1521
CGCAGCGCATACG	0.028	0.007	0.037	57	1475

CCTATTATTTAGG	0.023	0.001	0.022	36	1635
CGCGTCATTAACA	0.02	0.003	0.017	46	2636
GGTATTGCATGCA	0.081	0.019	0.061	60	921
ACCATTGTAAGGA	0.025	0.003	0.022	54	2372
CGCATTATAAAGA	0.024	0.003	0.025	89	3403
CCCGGTATTAATA	0.024	0.002	0.023	72	3083
AGCATTGCTTAGA	0.024	0.002	0.027	46	1681
AGTGGTATAAATA	0.023	0.003	0.027	62	2274
GGTGGCGCAGGGA	0.166	0.051	0.106	36	304
CCCATTGTAAACA	0.025	0.001	0.025	120	4665
ACCATCGCATATA	0.021	0	0.021	90	4259
GGCATCATAAATA	0.082	0.012	0.071	261	3429
GCCATTGCTTATG	0.049	0.004	0.045	54	1148
AGTGGTACATGTG	0.021	0.004	0.019	14	737
AGCGTTACTAATG	0.023	0.001	0.024	44	1820
ACCAGCATAAAGG	0.023	0.002	0.021	55	2564
ACCGGTGCTAATA	0.022	0.004	0.025	66	2586
CGTGTCATTAATG	0.022	0.004	0.017	21	1209
CCCATTGCAGGTA	0.023	0.002	0.025	52	2046
GGTATTATTGGTG	0.089	0.003	0.085	58	622
CCTATTGTAGACG	0.027	0.002	0.028	61	2098
ACTGGTGTTGATA	0.022	0.003	0.018	24	1331
GCTAGTGTAAGTA	0.052	0.003	0.053	82	1470
AGCGTTATATGCA	0.022	0.004	0.018	41	2301
ACTGTCATTAATG	0.026	0.001	0.026	52	1928
ACTATTACATAGG	0.024	0.004	0.019	46	2333
CGCGTTATTAGCA	0.029	0.001	0.03	52	1665
ACTGTCGTTTGCG	0.021	0.004	0.021	16	730
GCCGGTATAAGTA	0.077	0.003	0.074	141	1772
AGTGTCATATATG	0.028	0.005	0.029	34	1147
CCTAGTGCAAATA	0.021	0.004	0.024	77	3153
AGCGGCACAAATA	0.019	0.002	0.021	58	2662
GGCAGTATAAGGA	0.049	0.004	0.049	74	1425
CCTAGTGTTAGTG	0.023	0.002	0.024	29	1183
ACCGGTATAGAGA	0.02	0.001	0.018	41	2294
GCCATCACAGGTA	0.052	0.004	0.047	84	1685
ACCATCACTAAGG	0.019	0.001	0.02	58	2856
CCTAGCATATGTA	0.025	0.002	0.022	45	2018
ACTAGCGTATGCG	0.029	0.004	0.025	26	1007
CGTGTTGTTTAGG	0.034	0.016	0.016	8	483

AGCGTCACTTGTA	0.017	0.002	0.015	26	1697
ACTATTATAAACA	0.027	0.002	0.03	184	6053
GCCATTGTTTATG	0.044	0.006	0.037	49	1274
CCCATCGCAAATA	0.022	0.001	0.021	97	4510
AGCATTGTTAAGG	0.021	0.001	0.022	37	1652
CGTAGCGTAAATG	0.027	0.002	0.024	29	1166
GGTATCGCAAGGA	0.119	0.023	0.092	72	708
CCCAGTACATATA	0.026	0.005	0.028	114	4008
GCCATTATAAATG	0.047	0.005	0.041	142	3334
AGCAGTGTAAATA	0.025	0.006	0.018	67	3613
AGCATCGTAAACA	0.021	0.001	0.023	95	4119
AGCAGTATTGGGA	0.025	0.003	0.023	38	1642
ACTATTACATACG	0.025	0.001	0.027	93	3414
CCTAGTATAAACA	0.025	0.004	0.022	96	4291
CCCGTCACTAGGG	0.03	0.004	0.026	28	1070
ACTGTTATTGGCG	0.027	0.006	0.019	26	1369
CCTGTCGCTGACA	0.03	0.004	0.025	44	1695
CCCATTATTTGTA	0.028	0.004	0.022	63	2761
GGTGGTACTTATA	0.082	0.003	0.079	67	781
GGTAGCATATGTA	0.072	0.006	0.064	66	970
AGTATTATTAATA	0.021	0.001	0.019	71	3640
ACCGTTGCTAGGA	0.021	0.004	0.017	27	1525
GGTGGCGCTTACA	0.17	0.02	0.145	101	596
ACTAGTACAGACG	0.023	0.007	0.016	39	2438
CGTAGCATAAAGA	0.023	0.003	0.019	35	1792
CGCAGTATTAGCG	0.022	0.006	0.019	25	1322
GCTATCGTAAATA	0.056	0.004	0.05	116	2218
AGTGGTACTTGTA	0.025	0.009	0.018	16	858
GGCATTGCAAGGA	0.053	0.003	0.051	57	1055
CCTGTCACAGACA	0.021	0.002	0.02	50	2507
AGCGTTATTTGTG	0.022	0.002	0.019	23	1177
AGTAGCATAAACG	0.026	0.001	0.025	49	1910
ACCATCGCTTAGA	0.028	0.002	0.03	74	2359
ACTGGTGTAGACA	0.02	0.005	0.017	27	1597
CCCAGTGTATAGA	0.022	0.005	0.018	38	2056
AGCAGTATAAGGG	0.02	0.002	0.021	36	1662
CGCAGCGTATATA	0.025	0.005	0.031	61	1885
GCTAGTATATACA	0.063	0.004	0.061	153	2359
ACTGTTGTTAACA	0.027	0.003	0.023	56	2332
ACTAGCATAGAGG	0.028	0.005	0.032	43	1302

CCCAGCACTAACG	0.026	0.003	0.021	64	2930
AGTGGCGTAGGGA	0.022	0.006	0.015	7	455
CGCATTGCTAACA	0.027	0.004	0.025	60	2375
AGCAGCGTATGTA	0.023	0.001	0.021	41	1884
ACCAGTATATGTA	0.023	0.003	0.02	75	3624
CCCGTCACATACA	0.025	0.003	0.022	83	3681
GGCAGCATTTAGG	0.052	0.001	0.053	44	790
ACTATTACTGATG	0.019	0.002	0.022	60	2676
AGCGTTACATGTG	0.018	0.002	0.02	27	1303
ACTAGCGTAAGCA	0.027	0	0.027	55	1946
CCTAGTATTAACG	0.025	0.002	0.026	59	2237
CCCGTTATAAAGG	0.03	0.007	0.023	54	2305
CCCGTCACAGGCG	0.031	0.004	0.036	60	1629
CGCGTTACAGACG	0.024	0.003	0.023	34	1463
AGCGTCGCAGACA	0.018	0.002	0.017	35	2079
CCTAGTGTATAGA	0.023	0.002	0.024	43	1770
ACCATTGCTGGTA	0.023	0	0.023	50	2168
AGCGGTATTTAGA	0.024	0.004	0.027	44	1561
GCCGGTACATAGA	0.059	0.003	0.057	78	1293
CGCGTTGTATACA	0.024	0.004	0.02	38	1895
GCTGGCATAAAGA	0.654	0.034	0.65	1292	697
GGTGTCGTAAACG	0.108	0.03	0.09	77	781
CCCGTCACTAACG	0.026	0.002	0.026	67	2485
ACCGTCATATAGA	0.022	0.001	0.024	67	2740
GCCATCATAGACG	0.061	0.006	0.053	125	2235
AGTATTGTAAACG	0.018	0.005	0.013	27	2073
CCCAGCGCTGACA	0.021	0.003	0.017	52	3070
GCCGTCACATGGG	0.044	0.002	0.042	35	797
CGCAGTATAGATA	0.022	0.004	0.027	75	2676
AGCAGCATTGATG	0.021	0.004	0.017	31	1810
GCTAGCGTAAGGG	0.094	0.018	0.071	41	535
CCTATCACATGGA	0.027	0.004	0.022	46	2052
AGTATCGCTAACA	0.025	0.003	0.021	44	2055
GCCATTGCAAATA	0.044	0.002	0.043	121	2713
GCTATCGCATAGA	0.046	0.007	0.051	58	1070
AGTAGCACATATA	0.022	0.003	0.017	35	1988
CCCATCGTTAATG	0.026	0.003	0.027	70	2493
CCTGGCATAAGTG	0.023	0.005	0.021	25	1167
GGCATTACATGCG	0.065	0.008	0.054	75	1319
AGCGTTATTGAGG	0.022	0.002	0.019	22	1124

CGCAGCGTAGGCG	0.026	0.004	0.027	28	1005
CCCGTCGCAAGCA	0.023	0.003	0.019	52	2645
GCCGGTACTTACA	0.061	0.009	0.057	98	1629
GCCGTCATATGCA	0.056	0.003	0.057	102	1679
ACTGGTGCTTATA	0.024	0.002	0.021	35	1593
GCCGTCGTAGATA	0.056	0.009	0.043	57	1262
CGCGTTACAGATA	0.026	0.003	0.023	53	2238
CCTGTCACATACA	0.025	0.001	0.026	69	2587
GCCGTTGTAGACG	0.046	0.01	0.033	41	1218
CGTAGTGTTAGCG	0.025	0.005	0.02	12	593
GCTAGCACTAACG	0.065	0.005	0.062	84	1272
CGCAGCGCTGGTA	0.029	0.003	0.034	33	950
AGCAGTGCAAGGA	0.023	0.002	0.02	33	1594
GCTGTCATAAGCG	0.063	0.012	0.046	49	1015
CGTGTCACTGAGA	0.028	0.002	0.029	27	899
ACCATCATTTGCG	0.025	0.002	0.024	58	2407
ACTGTCATATATA	0.027	0.006	0.02	58	2916
GGCGGTATTGACA	0.071	0.009	0.069	94	1273
CCCAGTGTTTATG	0.03	0.006	0.025	41	1582
CCTATTATTAGCG	0.027	0.005	0.02	40	1943
AGCGTTGTATGCG	0.027	0.004	0.021	25	1177
GGTGTCACAGATA	0.086	0.021	0.061	64	982
ACCGTCATAAATA	0.029	0.003	0.024	122	4936
CGCAGCACAGGGA	0.03	0.005	0.024	24	973
AGCGTTGTAAACA	0.022	0.002	0.02	59	2898
AGTATCATAAACA	0.024	0.004	0.022	81	3591
ACCAGTGTATACG	0.024	0.001	0.026	60	2245
AGCGTCATTGACG	0.019	0.003	0.019	33	1695
ACCGGCACATGCA	0.022	0.002	0.021	60	2825
AGTGGTATTTGCA	0.024	0.002	0.024	23	934
CGCATTATATGTA	0.022	0	0.022	57	2533
GCCATCGCATATA	0.044	0.006	0.04	80	1921
AGTATCACATACG	0.028	0.003	0.028	47	1650
ACTATTATAGACA	0.027	0.001	0.027	121	4332
CCTGTCACAAACA	0.026	0.004	0.021	70	3254
ACTATCGTTGGGG	0.03	0.005	0.035	28	777
ACTGGTATTGGCA	0.027	0.008	0.017	23	1308
ACCGGCACTGGGG	0.026	0.003	0.024	20	829
GCCAGTATTGACG	0.054	0.007	0.044	83	1816
CCTAGCGCTGGCA	0.02	0.004	0.018	27	1467

CCCGTTATTGGCA	0.027	0.001	0.027	59	2094
CGTAGCACTTGTA	0.018	0.006	0.026	25	945
GGCAGCGTATACA	0.058	0.013	0.046	81	1672
CGCAGCGTTAGTA	0.023	0.004	0.021	29	1349
CCCATTGTATGTA	0.029	0.002	0.027	64	2279
GGCAGTGCTGATA	0.05	0.012	0.035	39	1086
CCTATTACAGGGG	0.04	0.003	0.035	40	1088
AGTATCATTTACG	0.022	0.006	0.014	22	1604
ACCAGTACATGTA	0.026	0.001	0.028	114	3975
CCCGGTGTTAAGG	0.025	0.006	0.024	28	1123
ACCATTGCATGTA	0.024	0.001	0.024	73	3033
GCCATTATTGGTG	0.054	0.005	0.056	79	1322
AGCGGTACTAGTA	0.02	0.001	0.022	35	1583
AGTGGTACTGATA	0.028	0.002	0.031	37	1149
AGCGTCATTTGGA	0.018	0.002	0.02	25	1228
AGTAGCGCTTGTG	0.021	0.004	0.015	8	531
CCCAGCATAGAGA	0.024	0.002	0.023	59	2537
GGCATCGCTAACA	0.076	0.008	0.066	127	1797
CCCAGTATTTAGA	0.024	0.002	0.023	57	2389
GGTGGCATTAATA	0.482	0.052	0.443	701	880
GGTGGCATAGGGA	0.384	0.059	0.368	268	461
CGCATCGCTGGGG	0.023	0.002	0.024	16	647
CCCAGTATAGGGA	0.032	0.001	0.032	53	1587
GCCGTTATTGAGA	0.05	0.005	0.048	68	1362
GCCGTTGCAAGTG	0.046	0.011	0.031	26	807
CCTATTATTGAGG	0.029	0.001	0.029	49	1666
AGCAGTATAAGGA	0.018	0.004	0.017	42	2498
ACCAGCACAAAGA	0.023	0.002	0.02	103	5067
GGTGTCGTTAATA	0.107	0.012	0.114	96	746
GCTGTCATAGGTG	0.063	0.003	0.064	44	647
GGCGGCACAAGTA	0.128	0.025	0.098	137	1255
GGTGTCATAGATG	0.088	0.004	0.086	61	652
ACTATTGCTAGCG	0.026	0.003	0.024	42	1698
ACTGTCATAGAGA	0.028	0.006	0.034	62	1769
CGTGTCGTAAGGA	0.022	0.005	0.017	14	831
CCCAGTATAGGTA	0.026	0	0.027	67	2416
ACCGTTGCTAGTA	0.021	0.002	0.018	37	1995
GCCGTTACAAACG	0.053	0.006	0.054	105	1827
CGCGTTATTAGTG	0.029	0.006	0.024	28	1126
ACCGGTACTGATA	0.019	0.003	0.022	73	3243

CGTATCGCTGAGA	0.02	0.002	0.018	18	969
CGTAGTACATACA	0.03	0.009	0.017	30	1712
CCCGTCGTAGACA	0.022	0.001	0.021	59	2738
AGCATTGCTAGCA	0.022	0.005	0.025	53	2090
ACCGGCATTTATA	0.027	0.002	0.024	64	2572
CGCAGTACTAACG	0.025	0.004	0.022	41	1807
CGCATTGCATACA	0.025	0.002	0.023	55	2366
GGCATCATATGTA	0.08	0.017	0.059	115	1832
GGCGTTGCATGCA	0.056	0.005	0.063	64	956
ACCGTCATTGGTA	0.028	0.001	0.026	50	1885
CGCGTCGTAAATG	0.022	0.003	0.018	29	1608
AGTGGCACAAATA	0.021	0.003	0.017	33	1857
CCCATCATATGTA	0.028	0.007	0.022	75	3357
CGTAGCATTTACA	0.025	0.003	0.025	37	1432
AGTATTGCATATA	0.027	0.005	0.024	46	1902
CGCATCATATACG	0.022	0.003	0.021	56	2638
GCCAGCATATGCA	0.046	0.002	0.043	104	2292
GGCGGTGTTGATA	0.067	0.005	0.066	63	893
ACCGTCGCAGGGA	0.023	0.005	0.016	22	1322
GGCGGTACAAACA	0.06	0.004	0.063	137	2049
CCTGGCGCTTGGG	0.034	0.009	0.03	16	524
AGTGTCGCTGGCG	0.019	0.004	0.016	10	631
GGCATCACAAGCA	0.066	0.008	0.056	121	2040
GCCGTCATAGACG	0.056	0.004	0.058	93	1519
ACTGTCATTAGTA	0.032	0.002	0.034	64	1808
CCTGGTGCAAGCA	0.02	0.002	0.016	26	1571
CCTGGCGTAGGGA	0.017	0.004	0.023	20	860
CCCATTGTTAGTG	0.03	0.002	0.027	45	1624
GGTATCACATAGG	0.133	0.033	0.094	68	653
GCTAGTGTTAGGA	0.056	0.006	0.047	40	807
GGTATTACTTAGG	0.109	0.02	0.124	98	690
CCCATTACTAGCA	0.026	0.002	0.03	99	3253
CGCATCACTGATA	0.02	0.002	0.019	47	2368
GGCGGTACTAGGA	0.064	0.011	0.058	49	801
GCCATCACTGATA	0.044	0.005	0.038	92	2341
GGCAGTATTAATA	0.054	0.003	0.054	148	2604
CGTATTATAGGGG	0.028	0.007	0.019	15	791
CGCGTTGCAGACA	0.025	0.007	0.018	32	1747
GCTATTATAGGTA	0.061	0.001	0.062	105	1595
AGCATTATAAACG	0.023	0.002	0.024	101	4052

GCCATCGTAAACA	0.052	0.007	0.049	156	3033
ACCAGTACTGACG	0.028	0.004	0.024	65	2649
ACTAGTATTTACG	0.024	0.004	0.021	44	2030
GGCGTTGCTAGTA	0.058	0.006	0.058	51	827
GCCAGCGCAAATA	0.049	0.008	0.044	93	2003
GGCATTACTAAGA	0.053	0.009	0.043	84	1868
GGTGTCGCTAATA	0.078	0.007	0.078	71	837
ACTGTCACTAAGA	0.023	0.005	0.017	37	2174
ACCATCATTAATA	0.021	0.001	0.02	121	5813
CCCAGTGCTGGCG	0.026	0.004	0.027	40	1460
ACCAGTGTAGATA	0.021	0.003	0.024	68	2771
GGTATTACTGAGA	0.096	0.013	0.078	79	933
CGCGGTACAGGCG	0.022	0.005	0.02	19	930
GCCGGTGTAAATG	0.405	0.057	0.373	614	1033
CGCATCGCAGATA	0.024	0.001	0.024	53	2120
ACCAGCACAAGTG	0.024	0.002	0.026	79	2932
CGCGTCATATACA	0.022	0.001	0.022	57	2554
GCCAGCACTAAGG	0.057	0.006	0.049	67	1292
ACTGTCATATGGA	0.032	0.001	0.031	46	1418
AGCGTTGCTAGTA	0.022	0.003	0.018	23	1256
GCTAGTACAAGTA	0.053	0.014	0.034	54	1517
AGCATCATAAGGA	0.022	0.002	0.022	64	2863
CCCAGTACAAAGG	0.029	0.004	0.024	62	2487
GGCGTCATTAAGG	0.066	0.006	0.061	52	804
GCTATTGCTTAGA	0.056	0.006	0.054	57	1007
ACCAGTACAGGCG	0.018	0.004	0.015	39	2564
GCCAGCGCTAGTG	0.048	0.005	0.055	38	659
CCTATTATAAGCA	0.026	0.004	0.022	79	3540
AGCATTATTAGGA	0.021	0.006	0.014	36	2485
ACTGTTGTAAGTA	0.024	0.004	0.019	33	1745
ACTATTGTAAGCG	0.026	0.002	0.027	58	2096
ACTAGTACAGAGG	0.028	0.002	0.03	54	1725
CGTAGCGCATGTG	0.028	0.007	0.023	15	642
AGCGGTGTAAGGG	0.024	0.008	0.03	27	872
GCTATTACTGGTA	0.05	0.012	0.035	42	1155
AGTAGCGCAAGCG	0.02	0.002	0.022	19	836
GCTGTCGTATACA	0.059	0.005	0.057	79	1312
AGTGGCACAAAGA	0.021	0.005	0.025	32	1245
CGCGGTACAGGCA	0.02	0.008	0.009	13	1363
CGTAGTATTAGTA	0.028	0.001	0.026	33	1218

ACCGGTACTAGCA	0.018	0.002	0.02	63	3058
CCCATCGCTAACA	0.021	0.003	0.018	72	4012
AGCGTCGTTAATG	0.021	0.002	0.02	28	1403
GCCATTGTTTGCA	0.052	0.002	0.054	85	1502
ACCAGCGTAAATA	0.03	0.005	0.022	81	3604
CGCATTGCAGAGG	0.026	0.002	0.022	24	1049
CCTAGCACAGAGA	0.027	0.002	0.026	50	1896
CCCGTCACAAGCA	0.029	0.005	0.023	72	3117
AGTATCGTTTGTA	0.025	0.003	0.029	35	1176
CGCAGCGTAGAGG	0.025	0.007	0.028	26	900
AGTATTGTAGATG	0.026	0.004	0.03	42	1375
CGTGTCGTATACG	0.029	0.007	0.02	20	993
CGCGGCATAGAGG	0.023	0.005	0.017	14	796
ACCGGCATTAATG	0.023	0.003	0.02	42	2110
GCTAGCGTAAATA	0.061	0.003	0.056	105	1755
GCTGGTACTAAGA	0.087	0.013	0.073	76	970
CCCAGTGTAGGCA	0.027	0.001	0.027	50	1814
ACCGTTACAAACA	0.021	0.001	0.019	120	6102
AGCATCGTTGGCG	0.022	0.002	0.02	33	1593
AGCGGTACAAAGG	0.021	0.004	0.017	25	1480
ACCATCATAGAGA	0.024	0.002	0.027	99	3530
CCTGTCATTAGGA	0.033	0.002	0.032	42	1289
CCTGTCACTTACG	0.028	0.005	0.021	31	1454
GCTGTTGTTTGGG	0.062	0.013	0.076	30	366
ACTATCACAAATA	0.024	0.004	0.02	111	5534
AGCATTGTAGGTA	0.023	0.004	0.021	40	1863
ACCGGCGCTTATA	0.025	0.005	0.032	42	1263
ACCGTTGTTGGTG	0.029	0.011	0.017	17	972
ACTAGTGTTGAGA	0.018	0.003	0.014	23	1600
AGTAGTGCTAATA	0.023	0	0.023	36	1563
ACTATTATTTAGG	0.029	0.006	0.021	41	1896
CGTATCGTAGGTG	0.033	0.009	0.021	16	732
AGTGTTATAAACG	0.026	0.003	0.023	45	1894
CCCGTCACAGACA	0.026	0.001	0.027	98	3500
AGCATCGTAGGGG	0.018	0.003	0.014	19	1338
ACTATCATAAACA	0.032	0.002	0.029	148	4981
AGCAGTATTTATG	0.021	0.002	0.02	42	2008
CCCGTCACAAATG	0.025	0.001	0.023	67	2830
CGCAGCATTTACG	0.025	0.002	0.022	36	1578
CCCAGCGCAAATA	0.022	0.002	0.023	99	4296

CGCATTATTAATA	0.023	0.002	0.021	80	3722
CCTGTCATAAAGA	0.024	0.001	0.022	54	2401
CCTGGTATAAGGA	0.025	0.003	0.027	39	1382
ACTAGTGTAAAGG	0.03	0.004	0.027	51	1873
AGCAGCGTTTAGA	0.023	0.007	0.02	31	1502
ACCATCACATGCA	0.023	0.002	0.02	92	4408
CGTGTCACTTGCA	0.029	0.003	0.026	23	846
GCCGGTGTAAGTG	0.415	0.061	0.408	434	631
ACTAGTGCATGGA	0.021	0.005	0.014	22	1598
AGTAGCGTAAAGA	0.026	0.006	0.029	43	1459
CGCAGCATTGACG	0.025	0.002	0.022	36	1615
CGCAGCGTAAATA	0.025	0.002	0.024	60	2471
GCCATCATTAACA	0.053	0.003	0.05	208	3984
AGCAGTATATGCA	0.02	0.003	0.02	63	3084
GGTATCGTATGTA	0.145	0.02	0.137	135	854
GCCGTTGCAAATA	0.047	0.005	0.042	80	1844
GCCAGTATATAGG	0.054	0.008	0.056	90	1513
CGTGGCACTTACA	0.028	0.009	0.026	31	1148
ACTAGCGCTGGGA	0.026	0.003	0.027	30	1087
CCCGGTGTTAGTG	0.025	0.002	0.028	30	1036
GGCATTATAGAGA	0.063	0.003	0.061	117	1807
CCTGTCATTTGGA	0.022	0.002	0.019	20	1027
ACCAGCACTAATA	0.024	0.002	0.026	146	5564
ACCGTTATATGTA	0.028	0	0.028	83	2874
CGCGGTGCATATA	0.021	0.003	0.018	27	1470
CCCGTCACTAGCA	0.029	0.005	0.024	59	2398
ACTATTGCATATG	0.022	0.005	0.019	44	2330
ACCGGCGCAAACG	0.021	0.001	0.022	44	1942
GGCATTGCATGCA	0.064	0.006	0.073	99	1264
CGCAGTGCAGGGA	0.021	0.005	0.018	15	841
CCCGTTACATACA	0.024	0.003	0.024	89	3553
GGTGGTATAGATA	0.134	0.01	0.147	149	868
ACCGTCGCTTGGA	0.02	0.005	0.016	21	1258
GCTATTGCTGAGA	0.052	0.005	0.049	51	991
CCCATTGCTAAGA	0.026	0.003	0.022	58	2568
CGTGGTGCTTGGA	0.026	0.006	0.023	11	458
CCCGTTATAGAGA	0.024	0.002	0.023	55	2366
CCTGTTACTAATA	0.022	0.002	0.023	60	2514
ACCAGTACAGACG	0.022	0.001	0.022	81	3663
AGCAGTGTTGGCG	0.022	0.001	0.022	30	1353

CCCATCATTAAGG	0.025	0.004	0.02	50	2453
ACTAGTGTAAATG	0.03	0.005	0.023	62	2616
ACTATCGTAGACA	0.028	0.005	0.021	53	2520
ACTGGTGTATGGA	0.031	0.007	0.022	21	938
ACTAGCATAAGTG	0.032	0.001	0.03	54	1746
CGCATTGCTGACA	0.023	0.003	0.019	37	1931
ACCAGTACAAGTG	0.023	0.003	0.023	71	2990
ACTAGTATTAGTA	0.023	0.001	0.022	57	2547
AGCATTGCTGGGA	0.022	0.004	0.017	21	1209
AGCATTGCTAATG	0.023	0.002	0.023	48	2076
GCCAGTGTAGACA	0.043	0.002	0.042	83	1872
CCCATCGTATAGA	0.025	0.004	0.02	48	2339
ACCGTTGTTAACA	0.022	0.004	0.019	54	2755
GCCGTTGCAAATG	0.044	0.002	0.046	58	1190
AGCAGCGTTGGCA	0.021	0.004	0.015	23	1485
GGCAGCGTAGAGA	0.066	0.005	0.06	64	1004
CGCATCATAGATG	0.028	0.002	0.026	59	2252
CCCAGCATAGATG	0.027	0.002	0.027	65	2372
CCCGGCACAGGGA	0.032	0.003	0.033	48	1399
AGTGTTATAAACA	0.022	0.004	0.017	54	3111
GCCATCACTAGCG	0.058	0.002	0.055	92	1567
ACTAGCACTAACA	0.024	0.004	0.02	84	4124
CCTAGCACATGCA	0.026	0.009	0.019	42	2178
GGTGGTACTTACA	0.085	0.009	0.073	71	899
AGTATTGCTTACA	0.023	0.004	0.02	35	1695
CCCGGCATAAGGG	0.038	0.002	0.036	45	1214
ACCGTCATATGGG	0.022	0.004	0.018	19	1022
ACTAGCGCAAGTG	0.021	0.002	0.02	27	1307
CGCGTCGCTAAGA	0.021	0.002	0.019	25	1310
AGCGGTGTATATA	0.024	0.004	0.028	52	1824
ACTAGTGTTTGTA	0.024	0.003	0.023	38	1585
CGCGGTATTTACG	0.025	0.002	0.022	27	1203
GGTGGCGCAAAGG	0.333	0.045	0.329	171	349
GGCGGCGTATGTG	0.474	0.075	0.467	351	401
CGCATCGCTTATA	0.021	0.005	0.014	26	1790
CGCATCGCTTAGA	0.024	0.007	0.026	34	1292
AGTGTTATTAATA	0.023	0.003	0.023	54	2338
CGTAGTACTAGGG	0.026	0.001	0.028	13	459
CGTGGTATAAACA	0.025	0.004	0.019	37	1937
AGCAGCACAAACG	0.021	0.001	0.023	67	2882

CGCGTTATATAGA	0.025	0.005	0.031	58	1822
CGCAGCGTTTACG	0.028	0.002	0.031	38	1191
GGCGTTGTAAACG	0.079	0.023	0.053	71	1274
ACCATTACAAAGA	0.023	0.003	0.028	169	5883
CGCGGCACAAACG	0.028	0.004	0.033	57	1684
GCTGGCGCAAGTG	0.82	0.03	0.801	823	205
CCCGTTGTTTGGA	0.023	0.004	0.022	23	1023
AGTATTACTAGTA	0.02	0.003	0.019	36	1889
GCCAGCACAGGTA	0.053	0.007	0.046	71	1488
CGCATTATAGATG	0.025	0.003	0.02	42	2037
AGTGTTACAAATG	0.022	0.005	0.016	26	1650
GCCGGCGCAGGTG	0.747	0.051	0.734	619	224
AGCAGCATAGGGG	0.016	0.001	0.015	23	1498
GCCAGCATTTGGG	0.057	0.012	0.044	31	681
ACCATCATTGGCG	0.024	0.002	0.027	73	2643
CCTAGTACATATG	0.027	0.002	0.03	56	1839
ACCATCACAGAGA	0.02	0.002	0.018	76	4035
ACCGGTGTATGTA	0.02	0.005	0.014	23	1662
ACTATCATTTGGA	0.031	0.004	0.036	63	1681
GCCGTTACATACA	0.053	0.003	0.056	133	2233
CCTAGCATAAATA	0.023	0.003	0.019	75	3848
ACCGTTATAAAGG	0.018	0.005	0.017	41	2366
GCCGTTACATACG	0.051	0.008	0.04	62	1502
ACTATCATAAGGG	0.033	0.005	0.027	41	1478
ACTGGTATAAACG	0.027	0.007	0.021	43	1966
CCCAGCATAAAGA	0.027	0.001	0.026	98	3669
CGCGTCATTAGGA	0.018	0.004	0.015	18	1200
AGCAGCATAGAGA	0.016	0.001	0.015	36	2306
ACTGTTGTATGTA	0.034	0.01	0.021	35	1604
CCTATCGTAAGCA	0.026	0.003	0.029	75	2522
CCCATTACATGTG	0.029	0.005	0.031	70	2175
CCCGGTATATATG	0.022	0.002	0.02	41	2006
ACCGTCGTAAAGA	0.022	0.002	0.022	48	2149
AGTATCGTATATA	0.019	0.004	0.013	28	2138
ACCGGCATAGGGA	0.028	0.004	0.023	30	1280
CCCATCATAAGCA	0.024	0.001	0.023	105	4511
ACCATTATAGACA	0.023	0.002	0.026	149	5582
GCCAGTACAAGGG	0.053	0.009	0.046	58	1213
ACCGTTGCTGACG	0.02	0.003	0.018	31	1735
ACCAGTGCTTGTG	0.027	0.002	0.03	43	1397

AGCGTTATAAAGG	0.023	0.004	0.018	36	1946
ACCAGCATTAAGG	0.023	0.004	0.019	43	2211
GGTGGTGTATGCG	0.185	0.034	0.173	80	382
CCCATTATAAACA	0.024	0.002	0.021	148	6870
CCTGTCGCTAACG	0.028	0.002	0.025	36	1389
CGCGTCATTGGCG	0.023	0.006	0.018	19	1047
CCTGTTACTAATG	0.021	0.002	0.023	38	1628
CCTAGCACAAGCG	0.024	0.003	0.021	35	1637
ACTAGCGTTGACG	0.025	0.003	0.027	33	1184
GCCGGTGCTAATA	0.056	0.006	0.054	70	1236
ACCAGCATAAGCA	0.025	0.001	0.025	110	4224
GCCATTATTTACG	0.054	0.003	0.058	134	2188
GCCGTCATTAATA	0.051	0.006	0.045	120	2525
CCTGGTGTTGATG	0.022	0.004	0.026	26	967
ACTGGTATATGCG	0.025	0.004	0.028	34	1178
GGCAGCGCAAGGA	0.068	0.004	0.062	56	842
CCCATCATATGCG	0.026	0.004	0.031	83	2552
CGTAGCGCTGACA	0.028	0.001	0.027	30	1089
AGCGGCGTTTGCG	0.02	0.004	0.022	12	528
ACCGGCGTTTAGA	0.025	0.001	0.025	27	1064
CGCAGCATTAACA	0.024	0.003	0.02	66	3224
ACTAGCGTAGGTG	0.032	0.008	0.025	24	921
GCCAGTACAGACG	0.056	0.003	0.052	90	1644
ACCATCGCTTGGG	0.023	0.005	0.024	28	1135
AGTAGTGCTAACA	0.023	0.005	0.017	28	1659
CCCATCACAAATA	0.025	0	0.025	147	5830
GGTATTACAAACA	0.089	0.014	0.075	184	2271
CCCGGCACAAGTG	0.027	0.007	0.026	45	1683
AGCATCGTTTAGA	0.018	0.002	0.021	38	1780
AGCATTGTTAACA	0.025	0.004	0.021	75	3414
GCCATCATTAGTG	0.057	0.006	0.057	94	1566
AGCGTTACAGGTA	0.022	0.007	0.012	21	1700
CCCGGCACTTGGA	0.029	0.01	0.027	35	1265
GGTAGCGCTAACG	0.067	0.005	0.06	45	706
ACCATCGCATGGG	0.028	0.006	0.023	31	1318
CGCATCGTATATA	0.024	0.001	0.024	60	2404
ACCATCACTAACG	0.022	0.001	0.022	100	4400
CCCGGTACTAATA	0.026	0.003	0.021	63	2945
CCTAGCGTATATA	0.022	0.003	0.018	42	2231
AGTAGTACTGAGA	0.023	0.006	0.017	20	1154

CCCAGTACAAATG	0.028	0.003	0.024	82	3335
CCCAGCGTATAGA	0.027	0.003	0.022	49	2165
ACCATCGCTTGCG	0.027	0.006	0.034	59	1675
ACCGTTGCATACA	0.021	0.001	0.022	77	3390
GCTATTATTTACA	0.058	0.009	0.047	116	2374
CGCGTTATTTGCA	0.022	0.003	0.023	34	1443
AGTAGCGTTGGGG	0.02	0.003	0.023	10	420
GCCAGTACATGTG	0.059	0.006	0.058	76	1242
CCCGTTACATAGG	0.032	0.006	0.024	37	1511
GCCAGTGCTGACG	0.051	0.008	0.044	49	1064
ACCGTCGCAGACA	0.022	0.003	0.021	60	2755
GGTAGCGCAAGTG	0.08	0.009	0.086	43	455
CCTGTCACATATG	0.028	0.003	0.026	41	1527
AGCATTATTTGTA	0.022	0.001	0.022	66	2908
GGCATTACATACA	0.055	0.007	0.048	132	2591
GGCGTCGCATACA	0.062	0.009	0.056	88	1488
GCCATCATTTGGA	0.051	0.013	0.052	79	1439
ACTAGCGTTTATA	0.033	0.006	0.025	46	1765
ACCGGTGCATACG	0.023	0.001	0.023	41	1733
ACTATCATAGAGG	0.029	0.004	0.024	43	1785
ACCAGCGCATGGG	0.025	0.006	0.029	34	1133
GCTGGTATATGGA	0.148	0.019	0.162	144	744
ACCAGCATTAACG	0.025	0.002	0.024	87	3497
CCTAGTGTATACA	0.025	0.002	0.023	61	2573
AGCATTGTAAGGG	0.024	0.007	0.017	26	1503
ACCGGCATAAGTA	0.023	0.003	0.023	65	2723
CGCGTCGCTGATA	0.02	0.004	0.015	21	1355
CGTAGCATTTAGA	0.019	0.009	0.013	14	1087
GCTAGTGCAGGCA	0.061	0.004	0.063	62	927
CCCGTTACATACG	0.027	0.004	0.025	62	2371
AGCGTCGTTGACA	0.022	0.003	0.024	43	1752
AGTATTACAAGTG	0.021	0.005	0.022	32	1416
AGTATTGTTGACG	0.024	0.003	0.021	27	1258
CGCATTATATGCA	0.027	0.003	0.025	64	2531
CCCATCGCTGATA	0.025	0.004	0.02	56	2750
GCTATCACTAAGG	0.058	0.004	0.054	63	1111
CGCATTACAAGGG	0.03	0.006	0.026	33	1244
ACCGGCACAAGTA	0.022	0.001	0.023	73	3121
GGTATTATATACG	0.12	0.014	0.099	141	1279
CGCGTCACATGCA	0.026	0.004	0.031	59	1852

AGTAGTATAAGTG	0.029	0.002	0.026	37	1365
AGCGGCATTAACA	0.024	0.003	0.027	58	2100
CCTATCATATATA	0.024	0	0.024	99	4101
ACTAGCATATATA	0.023	0.002	0.022	78	3500
GCCATCGCTTGTA	0.045	0.007	0.038	44	1117
CCCGGTGTAAACA	0.021	0.002	0.019	56	2907
ACTGGTATAAATA	0.025	0.005	0.023	78	3388
CCTGGCGTATATA	0.029	0.004	0.023	42	1756
CGCATCATTAATG	0.026	0.003	0.027	67	2379
GCCATTGTAGATA	0.05	0.007	0.051	111	2074
CCTATTACAGGTA	0.028	0.004	0.023	53	2262
CGCAGCACTAATA	0.021	0.004	0.017	43	2544
ACTGTTGCTGAGA	0.02	0.002	0.023	32	1388
GCCGGCACTTGTA	0.116	0.004	0.114	124	962
CCTGTCACTGACG	0.033	0.008	0.03	43	1392
AGTGGTGTAAATG	0.019	0.006	0.011	12	1051
GCCATCGTTAAGG	0.05	0.003	0.054	68	1185
ACCAGCGCATATA	0.02	0.004	0.017	66	3885
ACCATTATTGATG	0.024	0.003	0.019	63	3178
CCTAGTGTTAGTA	0.017	0.003	0.014	24	1672
AGTATTACTGGCG	0.019	0.003	0.023	30	1293
AGCATCATAGAGA	0.02	0.003	0.017	51	2912
CGCATTATAAACG	0.025	0.005	0.02	66	3224
AGTGTTATTAACG	0.022	0.001	0.021	32	1498
GGTGTTATTTGTA	0.092	0.013	0.078	65	764
AGCGGTGTATGTA	0.022	0.003	0.022	31	1348
ACCATTATTGACG	0.022	0.001	0.021	73	3380
AGCGTTGTTAAGA	0.017	0.001	0.018	28	1562
CCCATTGCAGGCA	0.031	0.002	0.034	89	2500
AGTAGCATATACA	0.025	0.003	0.03	73	2381
ACCATTACAGGCA	0.021	0.002	0.023	100	4164
AGCAGCATTTGCG	0.02	0.005	0.025	38	1465
AGCATTACTTGTA	0.023	0.005	0.021	52	2467
ACTGTTACTTATA	0.023	0.001	0.023	63	2694
GCCGTCACTAGGG	0.046	0.003	0.05	40	753
CGCATCATTTGGA	0.025	0.004	0.023	36	1499
CGCATTATTGAGA	0.023	0.003	0.027	56	2019
AGTATCACAAGCA	0.021	0.002	0.021	41	1866
CGTATTGCAGATA	0.026	0.001	0.026	38	1429
AGCAGTGTTTACA	0.021	0.006	0.019	46	2324

ACCATTACAGAGG	0.022	0.007	0.03	84	2749
CGTATTGTTAATA	0.03	0.003	0.027	52	1845
ACCGTCACTTACG	0.028	0.003	0.023	55	2302
ACCATTGTTTATG	0.023	0.002	0.023	51	2127
GCCATTACAAGCA	0.054	0.005	0.054	148	2590
CGCATCACTTAGA	0.024	0.001	0.022	40	1797
GCCAGTACAGGCG	0.05	0.002	0.047	60	1209
ACTATTGCAAGCA	0.026	0.004	0.025	80	3118
CCTAGTGCTGGTA	0.032	0.003	0.03	39	1259
AGCAGTACTTACA	0.025	0.001	0.026	78	2891
ACCAGCACTGGTA	0.023	0.003	0.021	59	2785
AGTAGCGTTTGCG	0.022	0.006	0.019	11	580
GCTATTATAAAGG	0.07	0.006	0.062	120	1818
GCTATTATATATG	0.068	0.006	0.067	137	1902
CGTGGTATATGGG	0.016	0.002	0.019	9	461
ACCGGCACTTGCA	0.024	0.006	0.017	39	2284
CCCGTCACATATG	0.027	0.002	0.025	52	2017
AGTGTTATTTACA	0.022	0.004	0.016	31	1875
CCTAGCATTTGTA	0.031	0.004	0.036	59	1590
CGCGTTGCTGATA	0.029	0.002	0.03	42	1349
CCCAGCGCTAACA	0.022	0.002	0.023	88	3793
GCTATCATTGACA	0.062	0.007	0.062	135	2053
CGTAGTACAAATA	0.029	0.002	0.031	68	2141
CGCGTTACAAGTA	0.023	0.005	0.031	63	2000
AGTATTATAAGCA	0.02	0.002	0.023	64	2733
CGCAGTATATGCG	0.028	0.002	0.03	43	1404
CGTATTACTAATA	0.022	0.002	0.021	53	2523
CCTAGCACTAGTA	0.027	0.004	0.022	43	1915
GCCATTACAGGTA	0.047	0	0.046	89	1826
GCCAGTACTAAGA	0.052	0.004	0.05	103	1974
AGTATTGTAAGTA	0.023	0.006	0.015	30	1980
CGTAGTGTAAAGA	0.026	0.004	0.031	41	1301
GCCATTGCAAGCA	0.045	0.006	0.042	85	1953
CGTATTGTATACA	0.024	0.002	0.025	49	1889
CCTAGCATTTGTG	0.029	0.004	0.029	32	1074
ACTGTCGTAAACG	0.028	0.007	0.018	28	1542
CCCGGCACATGTA	0.028	0.002	0.025	53	2081
AGCGGCACAAAGA	0.02	0.002	0.021	41	1884
GGTAGCATAAAGA	0.077	0.011	0.063	87	1299
ACCATTATTTATG	0.023	0.003	0.021	69	3292

CCCATTGCAGATG	0.021	0.002	0.022	48	2096
CGCGGTGCTAACA	0.027	0.003	0.025	42	1670
CGTGTTGCTAGTG	0.028	0.01	0.017	9	528
ACCAGCGCATACA	0.02	0.002	0.017	75	4351
GGCATCGCTGGTA	0.081	0.008	0.09	88	887
CCTATCACAGACG	0.03	0.003	0.034	81	2289
CCTGTCGCTAACA	0.023	0.003	0.019	45	2263
CCCAGCGTAAAGA	0.021	0.003	0.018	51	2799
ACTAGCATTGGTA	0.021	0.005	0.015	25	1651
GGTAGCACATACG	0.081	0.009	0.068	63	857
ACCAGTGTATAGA	0.027	0.003	0.028	69	2355
ACCGTTATTTGGA	0.023	0.003	0.019	30	1578
ACTAGTGCTGATA	0.026	0.002	0.024	56	2320
CCTATTGCAAACA	0.026	0.001	0.027	109	3975
ACTGTCACATAGA	0.025	0.004	0.025	50	1990
GCCATCGCTAGGA	0.054	0.004	0.06	61	953
ACCAGCATAAATG	0.021	0.004	0.018	73	4039
AGCGTCACATACA	0.021	0.004	0.015	46	2946
ACTGTTACAGAGA	0.024	0	0.024	51	2046
CCTATCATTAGCA	0.025	0.005	0.021	63	2900
GCCGTCGCATATA	0.052	0.007	0.049	63	1213
ACCAGTGCTGACG	0.021	0.005	0.014	30	2121
AGCGTTGCATATG	0.024	0.005	0.018	24	1319
CGCATTACTAAGA	0.023	0.002	0.025	57	2193
CCTGGTACAAGCA	0.027	0.006	0.026	49	1814
CGCATCGCTGGTA	0.018	0.001	0.017	20	1156
ACTGGTATAAATG	0.03	0.007	0.028	58	1980
CCCAGTGCTAATG	0.021	0.003	0.018	37	1978
CGTAGTACTGATA	0.022	0.003	0.026	35	1337
AGCATCGCTGATG	0.022	0.001	0.023	38	1603
AGCATCGCTAGTA	0.017	0.004	0.018	35	1934
CGCGTTGTTGAGA	0.024	0.003	0.028	29	1022
ACTATTGTAAATA	0.024	0.002	0.022	100	4463
CCCGGTACAAACA	0.023	0.004	0.02	86	4153
CCTGTCGCTTACA	0.023	0.002	0.022	39	1740
AGTATCGCAGGTG	0.023	0.005	0.023	18	765
GCCATCATTTATG	0.052	0.004	0.046	94	1929
CCCGGCACATATG	0.031	0.001	0.03	58	1884
CGCGGTATTGATA	0.025	0.004	0.024	38	1575
CCTGTCGTTAACA	0.019	0.003	0.018	39	2167

AGTATCATAGGCA	0.023	0.006	0.021	37	1703
GCCAGCACTTATA	0.053	0.007	0.051	108	2027
GGCGGTATATGTA	0.075	0.005	0.068	80	1099
GGTAGTACAAATG	0.066	0.001	0.064	84	1228
CCCATTATAAGCA	0.023	0.002	0.022	97	4305
ACCAGCGCATAGG	0.026	0.002	0.024	41	1664
AGCAGCGCTAGGG	0.019	0.002	0.016	12	760
ACTAGTGCTGGTG	0.024	0.003	0.022	25	1127
AGTAGTGTAGATG	0.028	0.006	0.019	21	1077
CGCATCACATAGA	0.023	0.003	0.019	42	2145
GCCGGCATAAATA	0.386	0.035	0.361	1155	2046
GGTGTCATAGAGA	0.086	0.016	0.07	54	718
AGCATCATTAACA	0.022	0	0.022	107	4770
GCTGTTGTAAACG	0.066	0.003	0.066	89	1255
ACTGGTACTTACA	0.023	0.002	0.02	49	2351
ACTGGTACATATA	0.024	0.002	0.022	62	2721
CCCAGTGCAGATG	0.029	0.006	0.02	37	1774
CCCGGTGTAGGTG	0.032	0.001	0.033	30	873
GGCAGTATTAGTG	0.05	0.007	0.05	55	1045
CGTGTCGTTAACA	0.024	0.001	0.023	31	1317
GCCGGTACTAACG	0.063	0.012	0.047	69	1400
CCCGGCGCATGTG	0.019	0.005	0.017	20	1147
ACCATCATAAGCA	0.026	0.003	0.022	106	4736
GCTATTACTGAGA	0.051	0.008	0.05	74	1419
AGCGTTGCTAATA	0.022	0.006	0.016	36	2151
CCTGGTACTAACG	0.024	0.002	0.027	37	1349
ACCATTGTTGGCG	0.022	0.003	0.02	47	2326
GCCATCACTGGCA	0.046	0.001	0.046	85	1780
CCTAGTGCTAGCA	0.026	0.004	0.02	38	1880
CGTAGCACTGGTA	0.029	0.003	0.033	29	852
ACTATTACTTATA	0.021	0.003	0.024	94	3880
AGCAGTATTAGCA	0.023	0.001	0.024	70	2842
ACTATTATAGACG	0.027	0.002	0.025	75	2902
AGTAGTGTATGCA	0.027	0.005	0.02	27	1306
CGTGTTGCATAGA	0.024	0.003	0.019	17	872
CCTGTCATAAGTA	0.024	0.002	0.022	51	2233
CCCATCGCAAGTG	0.023	0.005	0.02	40	1968
CGCATCGTTAGCG	0.028	0.008	0.017	25	1443
CCTAGCGTAAATA	0.025	0.004	0.019	53	2756
CGTGTTGTTAGGA	0.032	0.007	0.023	14	599

CCCAGCGTTTATG	0.022	0.008	0.018	30	1624
GCTGTCACAGGGG	0.068	0.012	0.075	35	434
ACTGTTATTAAGG	0.026	0.002	0.023	35	1487
CGCAGCATTGATG	0.029	0.005	0.032	44	1352
GGCGGTATTTGGA	0.079	0.015	0.067	52	721
CCCATCGCAAACA	0.024	0.002	0.027	133	4876
ACCGTCGTTAACA	0.026	0.003	0.03	77	2513
CCCATCACATATA	0.029	0.004	0.027	123	4376
AGCATTGTAAGTA	0.021	0.003	0.02	56	2799
GGTGTCACATACA	0.08	0.016	0.067	75	1050
GGCGGTACATACA	0.064	0.008	0.062	98	1480
ACTGTCACTGGTG	0.031	0.006	0.027	26	950
CCTGGTATATGCG	0.028	0.006	0.019	22	1149
AGTATTATTGGCA	0.023	0.002	0.019	34	1724
ACCGTTGTTGAGA	0.023	0.003	0.02	30	1489
ACTATCACAAGTA	0.029	0.003	0.03	104	3332
GGTAGTACATACG	0.065	0.005	0.062	60	902
CGCGTTACTAAGA	0.027	0.004	0.022	41	1784
ACCGGCGTTAGCG	0.028	0.003	0.024	19	772
GCTGTCGCAGACA	0.041	0.005	0.033	35	1011
AGTAGTGCTAACG	0.025	0.01	0.022	25	1122
ACCAGTATTAAGG	0.025	0.003	0.026	69	2615
AGCGTCGTATAGA	0.026	0.003	0.023	35	1516
GCTATTATAAACG	0.068	0.003	0.073	199	2542
AGCAGCGCAAAGG	0.025	0.005	0.018	27	1452
GGTATTACATACA	0.093	0.013	0.074	141	1762
ACTAGCACAAGCG	0.024	0	0.024	48	1920
CGTGTTATTAAGG	0.02	0.003	0.024	23	951
CCTAGCACTAGTG	0.028	0.005	0.035	45	1235
AGCGTCGCTAGCA	0.02	0.006	0.013	21	1536
AGCAGCACAGATA	0.023	0.005	0.016	44	2785
GCCAGTATTTATA	0.057	0.004	0.052	145	2654
CGCAGCGCTAATA	0.025	0.003	0.024	46	1903
CCTAGTACTTATA	0.027	0.002	0.027	67	2370
CCTATTACTTACG	0.027	0.004	0.022	48	2144
ACTATTACTGACG	0.027	0.003	0.024	60	2393
GCCAGTATTGATA	0.053	0.011	0.054	143	2501
CCCGGTGTATAGG	0.027	0.002	0.027	28	1022
AGTGGTATTAACA	0.021	0.003	0.022	41	1848
CGTGGCGTTTGCA	0.025	0.01	0.012	7	559

GGTGTTATTGGTA	0.065	0.005	0.059	39	623
GGCAGTGTATATA	0.059	0.003	0.057	98	1633
ACCGGTATAAACA	0.022	0.004	0.022	111	4870
CCCATCATATGGA	0.029	0.002	0.03	73	2373
GGCAGCGCTGACG	0.062	0.005	0.062	65	985
GGCAGTACAAGTG	0.051	0.003	0.052	66	1197
ACCGGCATATGTG	0.031	0.004	0.031	46	1458
CCTGTCGCTTATA	0.028	0.003	0.024	41	1658
CGCAGCACTTGGG	0.029	0.004	0.032	22	665
GGCAGCATATATA	0.056	0.014	0.053	117	2107
ACTATTATAAACG	0.034	0.003	0.031	133	4113
CCCATTGTAGATG	0.027	0.001	0.029	62	2086
GGTGTCGCAAGTG	0.098	0.015	0.081	40	454
GGTGTCGTAGGCG	0.095	0.019	0.071	27	353
AGTATTATTGGGA	0.018	0.004	0.012	15	1236
AGCGTCGTTTATA	0.021	0.005	0.018	30	1630
GCCAGCATTTACG	0.053	0.007	0.047	86	1739
ACCATTATTAATA	0.025	0.001	0.024	152	6308
AGTAGTATTAGTA	0.022	0.006	0.014	25	1756
ACTATTATAAATA	0.029	0.003	0.025	162	6395
GGCATCATAGACA	0.074	0.013	0.065	176	2512
ACCATTGCAAACA	0.022	0.002	0.019	112	5689
ACTGTTACAAACA	0.023	0.001	0.023	101	4348
GCTATTACTTAGG	0.058	0.004	0.053	50	890
CCTATCATTTACG	0.027	0.004	0.025	63	2417
GCTAGTACAAACG	0.063	0.007	0.059	101	1600
ACTAGTGCATGCA	0.022	0.003	0.019	47	2419
ACCATTGCAAGCA	0.023	0.003	0.025	94	3697
ACCGGCGTAAGCG	0.03	0.009	0.018	15	840
ACCGTCGCAAACA	0.023	0.003	0.023	89	3862
GCTATTGTTGGCA	0.054	0.007	0.046	52	1069
ACTAGTGTTAACG	0.028	0.001	0.029	63	2142
GGTGGCATAAACG	0.724	0.048	0.702	1279	542
CCCATTACAGAGG	0.024	0.002	0.025	53	2071
ACTGTTACATATA	0.025	0.003	0.022	71	3170
ACCATCGCATGGA	0.026	0.004	0.022	48	2168
ACTATTACTAATG	0.025	0.001	0.025	89	3435
CGCGTTATTGATA	0.021	0.004	0.015	29	1877
CCCATTGTATGGG	0.024	0.001	0.024	29	1182
GGCATCGCAGGTA	0.092	0.017	0.075	82	1008

ACTGTCGCATGTA	0.031	0.005	0.025	40	1534
AGCATCACTAATA	0.022	0.003	0.019	81	4078
GCTAGCGTTGGCA	0.058	0.01	0.045	36	769
ACTAGTACTAACG	0.024	0.002	0.022	65	2865
CGCAGCACAGAGG	0.023	0.003	0.027	29	1047
AGCATCATAAATA	0.022	0.002	0.021	125	5936
GCTATCACTAATG	0.062	0.003	0.058	95	1533
CCCATTGCAAATG	0.024	0.002	0.026	82	3055
GCTAGCACTAAGA	0.064	0.009	0.077	109	1307
GGCGGTGTATAGA	0.065	0.003	0.067	58	809
ACCGTTACAGATA	0.024	0.006	0.018	79	4204
ACCGTTGTAGATG	0.023	0.003	0.022	36	1610
ACTATTACAAATA	0.024	0.003	0.025	164	6444
CCCGGCATAGACG	0.027	0.006	0.022	47	2045
CCCATCACAAGTA	0.027	0.002	0.026	104	3940
GCCATTATAAGCA	0.056	0.003	0.057	202	3312
CCCGGTATTTATA	0.026	0.001	0.026	64	2387
ACCGTTGTATGCA	0.028	0.003	0.026	52	1930
AGTATCACAAAGA	0.029	0.005	0.025	51	1982
GCCATCACTAGCA	0.05	0.004	0.046	105	2199
GCCAGCATAGGTA	0.059	0.004	0.053	94	1667
CCTATCGCAGACG	0.025	0.001	0.024	45	1801
ACCGTTATATGTG	0.023	0.001	0.022	41	1799
ACCATCGCTAGTG	0.024	0.001	0.024	42	1701
CCTATTACAAAGG	0.026	0.004	0.022	54	2440
ACCAGTACTGACA	0.022	0.001	0.02	95	4692
GGCGGTGTTAACA	0.071	0.014	0.069	89	1197
ACCGGCACAAACA	0.021	0.004	0.021	112	5115
ACCATCGCTGACG	0.026	0.006	0.019	42	2228
AGTATTGTTAGTA	0.024	0.005	0.022	35	1573
AGTGGCGTATATA	0.022	0.004	0.023	21	901
ACCGGTACAAATA	0.022	0.002	0.02	105	5244
CCCATTGCTAACG	0.023	0.003	0.02	54	2662
CCCGTTACAGATA	0.025	0.002	0.023	69	2949
CCTAGCGTATAGA	0.021	0.001	0.021	34	1594
AGTGGCATAAGGA	0.018	0.005	0.011	7	647
CCTATCGCAGATA	0.024	0.004	0.026	65	2404
AGCATTGTAGGCG	0.02	0.003	0.024	41	1663
CGTGGCGTTGATA	0.024	0.009	0.017	12	697
CGCGTTGCTAGCA	0.022	0.001	0.021	28	1289

**CCCATCATAGGCA**	0.024	0.001	0.024	78	3223
**GCTGTTGTTTGCA**	0.058	0.005	0.052	44	805
**CCTGTCGTATATA**	0.021	0.002	0.021	44	2085
**ACCGGCGTTAATG**	0.024	0.001	0.022	21	948
**CCTATCGCTGGTA**	0.025	0.005	0.03	45	1464
**CGCGGTATTAGTA**	0.022	0.002	0.025	38	1472
**ACCGGTGCAAATA**	0.024	0.002	0.023	77	3265
**CCCATTATTAACG**	0.026	0.003	0.022	85	3698
**GCTAGCGCAAATA**	0.062	0.007	0.067	104	1453
**CCCAGTATATATA**	0.028	0.005	0.023	101	4217
**CGCGGCGTAAATG**	0.028	0.01	0.015	17	1130
**CCCATCATTAAGA**	0.022	0.003	0.02	73	3661
**CCTGTCATTAACG**	0.026	0.003	0.022	43	1889
**ACCATCACAGATG**	0.023	0.002	0.023	75	3186
**ACTGGTACAGACG**	0.026	0.002	0.029	47	1591
**AGCAGCATTAATG**	0.022	0.005	0.02	51	2466
**AGCGTTGCTGAGA**	0.023	0.003	0.027	34	1224
**ACTGGCGTAAAGA**	0.033	0.003	0.028	36	1231
**ACCGTTGTTTGCA**	0.026	0.002	0.025	41	1577
**CCTAGTGCTGGCG**	0.024	0.002	0.022	26	1163
**CGCGGCACAAGTG**	0.025	0.002	0.027	31	1104
**GCTATTATAGGGG**	0.077	0.007	0.087	66	696
**ACCGGTACATATG**	0.027	0.002	0.025	70	2735
**GCTATCGTTAGTA**	0.052	0.01	0.04	49	1174
**GGTAGTGCAGGGA**	0.051	0.013	0.049	24	468
**CCTATCACTAACG**	0.026	0.002	0.025	72	2793
**GCCGTTATATATA**	0.06	0.002	0.062	169	2575
**CGCAGCGCTTATG**	0.027	0.002	0.026	29	1096
**CGTATCATTGACA**	0.023	0.003	0.019	41	2093
**AGCATCACTTGTA**	0.019	0.004	0.022	48	2123
**ACTGTCGTATACA**	0.026	0.004	0.021	43	1973
**CCTGTTATTGACA**	0.026	0.004	0.031	72	2250
**CCCATTACATGCG**	0.027	0.002	0.027	67	2377
**CCTATTATTTGCA**	0.032	0.003	0.028	63	2173
**ACCGTTGCAAGCA**	0.02	0.002	0.022	62	2730
**CCCAGTGCTGGGA**	0.027	0.004	0.031	37	1160
**CCCAGTGTAGACG**	0.025	0.003	0.028	55	1927
**CCCAGCGCTTGTA**	0.021	0.001	0.021	42	2006
**CGCAGTGCAAGTA**	0.024	0.005	0.018	30	1607
**ACTGTCGCATAGA**	0.022	0.004	0.018	26	1432

CCCATTGTAGAGA	0.029	0.003	0.025	59	2262
AGCGTTACAAGTA	0.023	0.001	0.022	52	2275
AGCAGTGTTAATG	0.022	0.002	0.025	47	1857
CGCATTATTAACA	0.022	0.002	0.019	73	3749
GGCATCACAAGCG	0.085	0.012	0.069	112	1502
ACCGTTACTAACG	0.023	0.002	0.025	87	3409
CGCAGTGCATGTA	0.022	0.001	0.023	32	1371
ACTAGCACTTGTA	0.028	0.003	0.023	53	2241
AGCGTTGTTGGCA	0.019	0.003	0.015	19	1245
ACTAGCGCAGACA	0.021	0.001	0.021	59	2746
GCCGGTACAGGTG	0.08	0.009	0.089	63	643
CGCAGTACAAACA	0.025	0.003	0.022	80	3514
CCCGGTATAGAGG	0.026	0.004	0.024	32	1305
CCTATTACTTATA	0.024	0.001	0.024	77	3093
ACTATTGCATACG	0.024	0.004	0.022	59	2593
CGCGTCACAAAGA	0.024	0.002	0.021	47	2159
GCCGTTGCATATA	0.051	0.008	0.053	70	1261
ACCGGCACAGGCA	0.019	0.001	0.02	54	2652
GGCGGCACTGGCA	0.133	0.019	0.111	103	823
ACCAGTACTGAGA	0.019	0	0.019	63	3291
ACCGGTACTAATG	0.02	0.004	0.022	64	2793
GGTAGCATTAATA	0.076	0.009	0.065	96	1374
GCTATTATTAACA	0.064	0.006	0.057	182	2998
AGCATTACAAATA	0.022	0.003	0.021	115	5487
GCCGGTATAAGTG	0.144	0.013	0.129	166	1116
CGTAGCGCAAAGA	0.027	0.001	0.025	31	1191
GCCATCGCAGACA	0.044	0.01	0.031	65	2009
CGCGTCGCTGACG	0.022	0.001	0.021	25	1168
CCCAGCATATAGA	0.026	0.003	0.03	88	2873
ACCGTTATTTGCA	0.02	0.003	0.018	42	2336
ACTATTACTAACG	0.021	0.002	0.021	75	3524
CCTATTACAAGTA	0.028	0.004	0.023	77	3244
GCCGGTGCAAGCA	0.055	0.004	0.058	71	1146
CCTGTCATTAAGA	0.019	0	0.019	37	1877
CGCGGTGTATGCG	0.019	0.007	0.009	8	843
CCCGTTATAAGGG	0.035	0.006	0.026	38	1396
ACCGTTACAAGGA	0.02	0.002	0.017	51	2929
CGTATCATAGATG	0.028	0.005	0.035	55	1497
GGTATCACATGCG	0.118	0.018	0.1	76	681
CCCGTTGCAAGCA	0.019	0.006	0.016	39	2377

AGCGTTACTGGGA	0.023	0.005	0.028	28	985
GCCGTCACAAGGA	0.051	0.009	0.041	58	1354
CCTATCGTAGGGG	0.051	0.012	0.033	28	816
GCCAGTATATATG	0.061	0.002	0.063	130	1935
CCCGTCGCTGGCA	0.022	0.004	0.022	38	1655
ACTGTCACTAACA	0.021	0.002	0.021	68	3224
CGTAGTGTTTAGG	0.024	0.005	0.026	12	449
CCTGGCGCTAACG	0.029	0.005	0.022	28	1268
CCCGTCACATGCA	0.025	0.002	0.028	74	2602
CCCATTACAGGCG	0.033	0.003	0.028	61	2093
GCCGTTGCAAAGA	0.046	0.009	0.035	49	1340
GGCGGCGCATGTG	0.183	0.042	0.176	109	511
CCCGTCGCTAACG	0.022	0.001	0.022	45	1965
GCCGTCACTTAGG	0.047	0.01	0.056	50	844
ACCGGTATTAAGA	0.026	0.001	0.026	69	2632
ACCATCGTAGAGG	0.025	0.001	0.024	38	1557
GCCATTATATAGA	0.051	0.005	0.045	130	2753
CCCATCATAAGCG	0.024	0.005	0.02	63	3106
AGTGGTGTTAAGA	0.022	0.004	0.018	18	1010
CCCGGTACAGGGA	0.038	0.007	0.04	54	1302
CGTATTATAGATA	0.029	0.001	0.028	74	2556
ACCGGCGCAAGCA	0.024	0.003	0.021	36	1651
ACTAGCGCTGATA	0.022	0.007	0.013	28	2091
AGTATCGCATGTG	0.026	0.013	0.007	6	824
GCCGTCACTGATA	0.056	0.008	0.052	91	1668
GCCATTATTGAGA	0.05	0.004	0.05	114	2186
ACTAGCACTGACA	0.025	0.004	0.025	75	2982
GGTATTATAAGTA	0.102	0.023	0.084	160	1742
GGCGGCGTAAACA	0.209	0.031	0.185	294	1291
GCCAGTGCTAGCA	0.044	0.008	0.054	74	1296
GGCATTACAGACA	0.06	0.004	0.055	132	2288
AGTAGCGCAAATA	0.024	0.004	0.019	34	1781
ACTATCACAGACA	0.023	0.001	0.024	99	4059
AGCGGTGTTGAGA	0.025	0.004	0.028	30	1058
ACTAGCGCATGCA	0.023	0.001	0.024	55	2191
ACTAGCATAAACG	0.025	0.003	0.029	86	2906
CCCGTTGCATACA	0.024	0.002	0.022	66	2951
ACCATTGTAAATA	0.023	0.001	0.023	117	5016
CCCATTATTGATG	0.023	0	0.023	64	2695
CCTATTGCATGGG	0.027	0.003	0.023	25	1055

CCCATTGCTTGTA	0.023	0.002	0.023	46	1935
CCCAGTATTAGTA	0.026	0.001	0.027	76	2791
AGCATCATTGGCA	0.025	0.002	0.023	56	2424
CGCAGTGCTTATA	0.028	0.003	0.028	43	1502
AGTGGTATTGACG	0.017	0.004	0.013	10	766
AGCAGTACTGACG	0.022	0.005	0.023	51	2125
CCCATTACAGACA	0.024	0	0.024	111	4540
CGTGTCGTAAGTG	0.024	0	0.024	19	773
ACTAGCACTTAGG	0.031	0.004	0.027	36	1311
GCCATCATTAGCA	0.05	0.005	0.045	117	2499
CGCAGTACAAACG	0.024	0.002	0.025	59	2270
ACCATTATTTACG	0.023	0.004	0.021	74	3535
AGTATCACTAAGA	0.029	0.004	0.023	39	1688
ACCATTACAAGCA	0.021	0.002	0.023	131	5577
AGCGTCGTTAACA	0.021	0.003	0.023	54	2330
CCCGTTGTTTGCG	0.022	0.002	0.021	23	1079
CCTGTTGTAAACG	0.024	0.003	0.02	40	1952
CCTATCACTTACA	0.024	0.004	0.026	91	3353
CCTGTTGCAAACA	0.027	0.004	0.025	72	2764
GCTGTCATTGGTA	0.053	0.006	0.056	46	782
ACTATTGTAAACA	0.026	0.001	0.025	115	4478
ACTGGTGTAGAGA	0.021	0.005	0.029	33	1110
CCCGGCACTTGCG	0.024	0.004	0.023	33	1382
ACCATTGCAGATA	0.026	0.004	0.025	96	3748
AGTGTCATAGATA	0.023	0.008	0.019	35	1808
CCCGTTATAAATG	0.023	0.003	0.026	81	3019
AGCATTGCAAATA	0.021	0.001	0.022	86	3848
GCCAGTACTTGTG	0.053	0.006	0.06	61	956
ACTGTTGCAAGCG	0.025	0.004	0.024	32	1305
CGTATCATAGAGA	0.028	0.007	0.036	61	1638
GGTGTTATAAGCA	0.085	0.001	0.084	100	1092
AGCAGCACTTGTG	0.024	0.008	0.017	22	1260
ACTATTGCATACA	0.027	0.004	0.023	92	3857
ACTATCATAAAGA	0.024	0.002	0.021	82	3770
CCCAGTGTTGGTG	0.019	0.004	0.016	17	1049
GCCATTGCTGACG	0.048	0.005	0.043	56	1246
ACCGTCACATGGA	0.021	0.005	0.026	57	2150
ACTGGTGTTTGCA	0.029	0.008	0.019	19	981
GGCATTGTTTGTG	0.073	0.018	0.058	48	778
ACCGGCACTGACA	0.026	0.002	0.023	75	3156

GCCGTCATAGGTG	0.056	0.004	0.058	54	883
AGCAGTATTAGCG	0.018	0.003	0.02	36	1806
ACCGGTGTTGAGA	0.025	0.003	0.027	36	1287
GCTGGCATTGGGA	0.638	0.044	0.607	509	330
CCCAGTACAGATA	0.024	0.004	0.022	78	3536
ACCGTCACTAATG	0.023	0.002	0.025	66	2589
GCTATCATAAATA	0.067	0.006	0.061	203	3107
CCCGGCGCTGACA	0.022	0.001	0.023	53	2229
GCCGTTGCTTAGG	0.052	0.004	0.051	34	628
CCTGTTGCTAATG	0.027	0.006	0.019	26	1334
ACCGGCGCTGGCA	0.025	0.006	0.016	19	1159
ACCGTTACAAATA	0.023	0.003	0.019	116	6005
AGCAGCGCAGATG	0.03	0.005	0.027	35	1285
ACCAGTATATAGG	0.022	0.002	0.025	63	2436
CGTGTTATTTACA	0.024	0.004	0.023	35	1486
AGTATTATATATA	0.023	0.004	0.022	82	3567
ACCATCGTAAATG	0.025	0.003	0.02	61	2985
AGCATCGTTTACA	0.023	0.005	0.023	64	2663
AGTATCATATGCG	0.025	0.002	0.025	32	1259
GGCGGTACTAACG	0.068	0.004	0.063	67	993
CGCGTTGTAGGCG	0.027	0.007	0.021	21	982
ACCATTGTTTATA	0.023	0.002	0.021	64	3023
AGCGTCATTAATG	0.022	0.003	0.025	52	2070
GCCGTTGCTAGGG	0.054	0.015	0.045	27	569
ACCAGTGCAGAGA	0.024	0.003	0.022	55	2450
GCCAGTACAAGGA	0.054	0.004	0.058	101	1628
GCCAGTATAAGCA	0.052	0.008	0.044	135	2918
ACTATTACTGGTG	0.025	0.005	0.019	34	1712
ACTAGCATTGACA	0.026	0.002	0.024	66	2683
CCCATCGTATACA	0.022	0	0.022	82	3644
ACCGTCATATAGG	0.023	0.002	0.025	43	1685
AGTGTCGCATACA	0.024	0.002	0.025	35	1360
CGCATCATAGGTG	0.028	0.003	0.032	42	1287
AGCATTGCTGGCG	0.023	0.004	0.019	30	1547
ACTGTTGTAAACA	0.029	0.001	0.028	81	2815
GCTGTCGTAAGGA	0.047	0.016	0.042	32	736
CGCGTTGTAGATG	0.028	0.005	0.021	21	996
GCCATCGTATACA	0.048	0.002	0.044	110	2373
ACCATTGCAGGTA	0.026	0.005	0.023	60	2505
GGTGGTACAGACG	0.102	0.027	0.071	47	617

CGCAGCGTATAGA	0.021	0.002	0.018	26	1457
CCTAGCGCTTAGG	0.027	0.004	0.031	25	787
AGTAGCACAGATG	0.025	0.001	0.024	28	1149
GGTAGCGCATAGA	0.075	0.018	0.05	36	686
ACTATTATAGAGG	0.029	0.004	0.024	50	2038
CCCGGCATAAAGA	0.028	0.004	0.027	77	2782
AGCGTCATTAGCA	0.022	0.004	0.017	33	1952
AGTATCATAGATA	0.025	0.003	0.02	49	2356
AGCGTTACAGACG	0.021	0.003	0.016	29	1737
GGTATTATAAAGG	0.102	0.017	0.09	123	1247
AGCATTACTGACG	0.025	0.002	0.026	67	2485
ACCAGCGCAGATA	0.021	0.005	0.016	59	3680
GGCGTTGTTGAGA	0.069	0.015	0.048	39	780
CCCATTGTATGTG	0.028	0.006	0.02	35	1719
GCTAGTGTAAATA	0.051	0.008	0.039	93	2265
AGCATTACTGGCA	0.024	0.002	0.025	54	2103
ACCGTTGTAAAGG	0.023	0.006	0.018	32	1706
GGCATTGTAGGGA	0.057	0.008	0.049	53	1021
ACCGGCACATATG	0.021	0.001	0.019	42	2138
GGCAGTACTTGCG	0.049	0.008	0.038	38	962
CGCATTGTATAGA	0.025	0.002	0.022	39	1744
ACCGTTACATGTG	0.019	0.003	0.021	48	2207
ACCGTCACAAGTG	0.026	0.003	0.022	50	2176
CGCAGTGCTTACA	0.02	0.002	0.017	27	1566
ACTGTTGTTAGTG	0.025	0.002	0.024	23	924
AGTAGCACTAGGG	0.022	0.003	0.021	13	600
GGCAGCACAAACA	0.051	0.008	0.042	114	2578
GCCATCGTTTAGA	0.06	0.013	0.043	55	1231
GCCAGCATAAGTA	0.061	0.002	0.059	152	2444
CCTGTCGTAGATA	0.023	0.007	0.02	38	1839
CGTAGCGCTTAGA	0.023	0.007	0.014	10	710
ACCAGCATTAAGA	0.02	0.002	0.023	82	3540
CGTGGTACTTACG	0.017	0.004	0.012	9	754
GCCGTTGTTAACA	0.042	0.004	0.039	69	1709
CCCGTCACTGACA	0.025	0.002	0.024	69	2854
AGTAGTATAAATA	0.024	0.006	0.016	58	3598
CCCGGTGCTTGCA	0.021	0.001	0.02	31	1485
AGCATCATAGACA	0.022	0.002	0.025	110	4274
ACCGTTGCTAGGG	0.028	0.004	0.026	28	1034
CGTATCGCATGGA	0.024	0.004	0.028	25	858

AGTAGTATAAGGG	0.018	0.008	0.029	31	1047
GGTATCGTTAACA	0.139	0.029	0.127	188	1291
GCTGGCACTAGTA	0.217	0.016	0.219	208	742
CCCATCATAAGTA	0.027	0.004	0.023	94	4076
GGTAGCACAGGCG	0.058	0.012	0.041	24	561
GGCAGTGCAGGTA	0.054	0.007	0.045	42	886
GGCATTGCAAATG	0.068	0.01	0.055	87	1481
CCTAGCACATGCG	0.032	0.008	0.041	60	1402
AGTAGCACATAGG	0.025	0.004	0.03	26	837
CGCGGCACTAACG	0.021	0.004	0.019	27	1398
CGTGTTGCATGCG	0.027	0.002	0.027	19	679
GCTAGCGTTTATA	0.06	0.001	0.059	66	1056
CCTATCGCTAGCG	0.025	0.002	0.024	32	1284
GCCGGTACTTAGA	0.061	0.006	0.053	62	1100
CCCGGTGCAAATA	0.024	0.002	0.022	64	2853
AGTAGTACTTAGG	0.024	0.005	0.023	19	801
AGTGGTATATGTA	0.024	0.005	0.025	31	1193
GCCAGTGCAAGCA	0.036	0.006	0.044	70	1530
CCCGTCATATGGA	0.03	0.002	0.03	53	1719
AGTAGTATTAAGA	0.024	0.002	0.024	48	1968
GCCAGCGTAAATG	0.065	0.007	0.059	96	1519
CCCGGCGTAAGGA	0.022	0.003	0.023	34	1459
CGTGTTATAAATG	0.027	0.001	0.027	47	1689
ACCATCGCTGGCA	0.018	0.002	0.02	53	2547
AGCATCACAAACG	0.021	0	0.021	79	3767
CGCATTATAAGGA	0.023	0.002	0.023	54	2244
GGCGTTATATGCA	0.064	0.013	0.066	100	1408
AGCAGTATAGGTA	0.022	0.004	0.022	55	2438
GCTGGTATAAATA	0.152	0.015	0.133	301	1958
ACCATCGCAAACA	0.021	0.001	0.02	117	5811
AGCGGCGTATAGG	0.025	0.002	0.024	16	664
CGCAGTGCATAGG	0.03	0.01	0.015	13	837
AGTATCGCAAATA	0.021	0.001	0.021	48	2186
AGTGTTGTAGATG	0.027	0.011	0.015	14	921
CCCAGCGTATGTA	0.024	0.004	0.019	43	2192
CCCGTTGCAAATA	0.026	0.004	0.022	72	3160
CCCGGCACTAGTA	0.033	0.006	0.026	52	1942
CCCATCGTAGATA	0.025	0.003	0.023	71	3079
CCCATTACTAAGG	0.028	0.004	0.029	71	2407
AGCATCATAGGCG	0.025	0.001	0.024	53	2144

AGTGGTGTATATA	0.021	0.002	0.021	27	1258
CCCAGTATTGGGA	0.032	0.005	0.037	54	1403
CCCGTCGCTGGTG	0.019	0.003	0.015	15	964
CGTATCGTTGGGG	0.018	0.008	0.008	4	496
ACCGGTACTTACA	0.02	0.002	0.018	66	3667
AGTATTGTTGGTG	0.02	0.007	0.012	9	736
GCTGGCACATGCG	0.482	0.047	0.465	404	464
GCTATCATAGGTA	0.064	0.006	0.064	91	1335
AGTATTACAAAGA	0.023	0.002	0.02	57	2773
AGCGTTATAAGCA	0.025	0.004	0.029	83	2738
CGCGTCGTTTGCA	0.02	0.005	0.013	15	1127
AGCATCGTAAACG	0.022	0.004	0.018	54	2945
ACCAGTACAGACA	0.022	0.001	0.022	123	5540
AGTAGTGTTAATA	0.025	0.004	0.024	44	1820
CCTAGCATATACA	0.027	0.003	0.026	82	3043
ACTGGCACATGTG	0.029	0.006	0.026	28	1052
AGTAGTACAAGTG	0.027	0.004	0.028	35	1205
ACCATCATATAGG	0.022	0.001	0.024	67	2743
GGCGGTATTAGCA	0.085	0.001	0.085	100	1076
GGTATTACAGATA	0.081	0.009	0.074	128	1592
ACCAGCGTTAGGA	0.021	0.001	0.022	31	1393
ACCGGTATTTAGA	0.022	0.001	0.024	49	2024
GCTGTTATAAATG	0.066	0.012	0.064	106	1557
ACCAGTACTAAGA	0.025	0.001	0.025	113	4467
AGCGTTGCTGGCA	0.021	0.003	0.016	19	1140
CCCAGTGTAGGGG	0.034	0.006	0.028	26	912
GGCGTCACAAATA	0.063	0.007	0.054	125	2188
GCCATCATAGAGA	0.052	0.008	0.043	107	2392
CCCAGCACATACG	0.027	0.002	0.026	75	2835
CCTAGTGCTAATG	0.025	0.003	0.023	37	1566
CCTATTGTAGAGA	0.026	0.004	0.021	43	2018
AGCGGTACAAATA	0.023	0.005	0.03	92	2925
GCTATCACTAAGA	0.062	0.008	0.052	89	1638
ACCGTCACTAACA	0.023	0.001	0.025	125	4920
CGTATTATTGACG	0.032	0.003	0.032	47	1406
AGCAGTATAAATA	0.023	0.003	0.021	123	5755
AGCGTCATAGGGG	0.02	0.003	0.024	32	1278
GCCATTGTAAGGG	0.055	0.009	0.052	57	1032
CCCAGTGTAGACA	0.023	0.001	0.023	65	2791
ACCGTCGTATGTG	0.021	0.002	0.02	21	1032

CCCGGCGTTAACG	0.026	0.002	0.025	38	1482
AGTATTGCAGACA	0.023	0.005	0.025	48	1841
CGCAGTGCATATG	0.022	0.008	0.019	22	1157
CGTGGTGCAAAGA	0.027	0.006	0.021	20	951
CCTGTCGCAAATA	0.025	0.003	0.022	56	2546
AGCGTCACTGATA	0.017	0.004	0.015	31	2034
CCTAGCGTATGTA	0.027	0.007	0.018	28	1511
GGTATCGCTTGTA	0.098	0.003	0.1	67	602
ACCAGTATTTGGG	0.025	0.001	0.024	34	1405
GGCATCATTGAGA	0.081	0.015	0.063	95	1402
AGCGGCGCAGGTG	0.022	0.008	0.016	11	664
GCTATCGCTAGCA	0.043	0.007	0.04	45	1094
GGCGTTATTGACA	0.065	0.013	0.048	77	1517
CCCGGCGCATATA	0.023	0.004	0.024	51	2061
ACTGTTACTAGCG	0.029	0.002	0.029	40	1349
AGTATCGTAGGCA	0.023	0.008	0.023	28	1212
CCCATCACTTGGG	0.032	0.006	0.04	55	1333
AGCAGCGTTAATA	0.02	0.004	0.015	37	2464
GGTGTTGTAGGTA	0.083	0.003	0.079	42	488
CCCAGTGTATGGG	0.024	0.004	0.019	20	1016
CCCGGCGCTAACG	0.023	0.002	0.023	41	1723
CGTAGTACAAGTA	0.017	0.003	0.013	18	1413
ACTAGCGTATGGG	0.03	0.009	0.025	19	728
ACCATTGCTTGCG	0.024	0.005	0.022	43	1888
CGCGGTATAAACA	0.025	0.004	0.025	70	2712
CCCATCACTTATA	0.026	0.003	0.024	90	3625
ACCATTATAGGCA	0.022	0.003	0.018	70	3763
AGCATTATATATG	0.023	0.002	0.023	74	3149
CCTAGTATTGGCG	0.029	0.003	0.034	43	1236
GGTGGTATATGTA	0.13	0.031	0.099	83	754
AGCGTTACAAACG	0.018	0.003	0.015	38	2531
GGCGTCGCAGAGA	0.071	0.007	0.062	54	815
GCCATTATTAGTA	0.054	0.003	0.052	136	2485
GGCATCGTTGACG	0.096	0.019	0.1	124	1111
ACCATTGCAGAGA	0.023	0.003	0.022	64	2849
AGCATTGTTTACA	0.022	0.001	0.021	56	2651
ACCATCGCATAGA	0.023	0.003	0.02	61	2988
AGCATCGCAAGGA	0.022	0.001	0.023	43	1859
CGCATTACATACA	0.023	0.003	0.027	91	3291
CGTATTATAGACG	0.023	0.005	0.019	33	1748

AGCGTCATATACA	0.02	0.003	0.017	56	3221
CCCGGTATTAGGA	0.022	0.003	0.02	28	1383
ACCGTCATTAACA	0.027	0.003	0.026	111	4223
ACTATTATATACA	0.024	0.001	0.023	118	4970
AGCGTCACAGGCA	0.023	0.002	0.022	40	1806
ACCATCATAGGCA	0.024	0.003	0.021	77	3645
CCCATCGTAAGGA	0.022	0.001	0.02	45	2184
CCTATCACATACA	0.023	0.001	0.022	94	4169
CCCATCACATGTA	0.025	0.001	0.024	77	3197
GGCAGCACATGGG	0.043	0.003	0.042	30	692
CCTGTCACAAATG	0.027	0.005	0.032	62	1890
CGCGGCGTTAATA	0.025	0.007	0.021	29	1347
CCTAGTGTTTACA	0.022	0.005	0.023	51	2153
AGTATCATAAGGG	0.028	0.001	0.03	34	1111
GCCAGTATTGAGA	0.048	0.001	0.047	87	1767
CGCGTCATATAGG	0.027	0.004	0.032	35	1065
AGCAGCGTTTATG	0.018	0.003	0.018	24	1326
CCTAGTATAGGCA	0.024	0.003	0.029	57	1919
CGTATCGTTTGGA	0.025	0.007	0.02	16	798
AGTGTCGTTAATA	0.015	0.002	0.012	19	1545
CGCAGCACTTGGA	0.026	0.001	0.027	29	1032
CCCAGCGCAAGTA	0.022	0.002	0.019	55	2771
ACTAGTGCTAGCA	0.022	0.003	0.026	61	2249
CCCGGTATATGCG	0.026	0.004	0.022	36	1586
ACTGGCGCTGACG	0.022	0.005	0.017	14	807
CGCAGCATATATA	0.025	0.001	0.025	69	2681
ACTAGCGTTTACA	0.033	0.004	0.029	56	1859
ACCAGCGCAAGGA	0.019	0.002	0.019	45	2378
GCCATTATAAAGA	0.052	0.002	0.053	203	3639
AGTGTCACAGACA	0.021	0.004	0.017	28	1597
ACCAGCGTAAATG	0.025	0.002	0.027	64	2332
AGTAGTATTGGCA	0.02	0.001	0.019	26	1365
GCTAGTGTATGCA	0.063	0.001	0.064	87	1283
CCCGGTACAAATA	0.025	0.004	0.024	86	3566
ACTGGCGCTTACA	0.026	0.003	0.029	29	958
CCCGGTATAGGGG	0.04	0.002	0.037	32	838
CCCGTCGCAGACA	0.019	0.003	0.016	46	2797
ACTGTCGTTTATA	0.033	0.005	0.04	61	1449
ACTATTACATGCA	0.022	0.002	0.022	79	3481
ACCAGCATAAACG	0.023	0.003	0.021	88	4078

CCTGGCACTTGTA	0.025	0.002	0.026	32	1220
GGCGTCATAAGTA	0.059	0.006	0.067	110	1530
CGCATCATATGCG	0.026	0.001	0.024	43	1718
GCCGGTATTTACA	0.082	0.007	0.077	150	1801
AGCATCATAGGCA	0.02	0.005	0.02	57	2812
CCTGTTACAAACA	0.024	0.003	0.027	96	3420
CCCGTTGCAAAGA	0.028	0.006	0.02	46	2209
AGCAGTGCAAACA	0.022	0.002	0.023	82	3465
CCTATTATAGGTA	0.027	0.002	0.026	68	2546
CCTGTCACAGAGA	0.023	0.002	0.02	34	1629
CCTGGTGTAAATA	0.024	0.001	0.023	50	2141
GGCATTATAAGCA	0.056	0.002	0.055	141	2416
CCCAGTATAGGCA	0.027	0.003	0.026	71	2676
CCCAGCATTAAGA	0.028	0.003	0.026	78	2953
GGTGTTGTAGATA	0.088	0.019	0.082	69	772
CCCAGTATAAATG	0.022	0.001	0.021	78	3606
CGCATCACATGCA	0.022	0.001	0.023	56	2370
GCCGTTATTAGGA	0.058	0.01	0.044	56	1211
AGCATTATTGATG	0.019	0.002	0.021	51	2378
ACTGTCGTTTGTA	0.027	0.007	0.025	26	1016
CCCAGCGCTTACG	0.025	0.004	0.021	39	1814
ACTGTCATTAATA	0.023	0.005	0.025	73	2904
CCCGTTACATATA	0.024	0.001	0.025	85	3291
GCCGGCGCAGAGG	0.74	0.04	0.743	663	229
CCTAGTGTTTGTA	0.026	0.003	0.022	31	1383
GCTATTGTTTACA	0.056	0.002	0.055	98	1689
AGTGTCACAGGGA	0.029	0.005	0.022	17	742
AGTGTTATTGATA	0.017	0.003	0.013	23	1696
GGCGGCGTTTATA	0.206	0.038	0.181	163	736
GCTAGTATTGGTG	0.063	0.01	0.066	51	720
CCTATCACTGGCA	0.023	0.002	0.025	54	2126
CCTGTTGCTGACG	0.024	0.005	0.02	27	1299
CCCGGTACAAGCA	0.028	0.005	0.024	63	2614
AGCGGTACTTGCA	0.026	0.002	0.024	34	1370
GCCATTACAAACG	0.054	0.008	0.044	131	2862
CCTAGTACATACA	0.026	0.003	0.021	69	3259
AGTATCATAAATA	0.019	0.003	0.02	73	3621
AGTGTCACTAGTA	0.018	0.004	0.012	14	1199
GCCGTCATTTACG	0.052	0.007	0.05	71	1341
CCCATCGCATGTG	0.027	0.006	0.019	33	1680

GGCAGTACTAATA	0.054	0.009	0.044	103	2259
AGTAGCATTGGGG	0.023	0.002	0.026	16	602
ACCATTGCTAACA	0.024	0.003	0.022	103	4591
CCCGGTGTTTAGA	0.021	0.003	0.019	23	1160
ACCGTCACTAAGG	0.021	0.002	0.019	40	2017
GCCAGCACATGCA	0.052	0.005	0.045	90	1916
GCCATCGCTAGTG	0.053	0.015	0.069	67	901
AGTAGTACATGGG	0.025	0.004	0.028	21	722
CCCAGTATTTATA	0.024	0.001	0.023	83	3450
CCTGGCACAGGTG	0.028	0.006	0.037	30	776
CCTAGTATAAATA	0.024	0.004	0.02	87	4262
CCTATCACAAATA	0.027	0.003	0.031	160	4951
GCCGGTGCTAATG	0.113	0.024	0.088	83	857
AGCGGTACTGGTA	0.02	0.004	0.022	26	1148
GGCGGCGTTTACA	0.208	0.041	0.181	192	868
GCCGTCACAAGCG	0.054	0.003	0.055	82	1404
AGTAGTATAGGCA	0.027	0.006	0.021	32	1480
ACTGTTGCAGATA	0.027	0.004	0.023	48	2019
AGCATCATTAAGA	0.023	0.001	0.022	81	3593
AGCATCATATAGG	0.024	0.002	0.021	45	2113
CCCAGTGCTAACA	0.026	0.002	0.028	92	3173
GCTGGCACATACA	0.228	0.027	0.207	330	1261
CCCGGCGCAAACA	0.021	0.003	0.025	79	3118
CCTGTCACTGGGG	0.05	0.005	0.044	28	612
AGCAGCACATGCG	0.019	0.001	0.02	34	1705
CGCGGTGTATAGG	0.025	0.003	0.03	23	755
CGTATTACTAGGA	0.026	0.005	0.029	34	1145
ACTAGTGTTAATA	0.024	0.004	0.019	58	2970
CCCGGTACAGGCA	0.025	0.005	0.017	36	2048
ACTGTTACAAAGA	0.021	0.002	0.02	59	2958
ACTGTTGTTGGTA	0.029	0.003	0.032	36	1076
CCTAGTACAAGGA	0.025	0.002	0.023	41	1776
GGCGTTGTTGGCA	0.055	0.012	0.055	42	721
AGTATTATTTACA	0.022	0.002	0.025	72	2800
ACCGTTATTAGTA	0.024	0.003	0.028	75	2592
AGTATTATTGGTG	0.022	0.001	0.023	25	1073
GGTGTCATTGATA	0.094	0.006	0.088	90	930
CGCAGTGTAAACA	0.028	0.003	0.025	70	2752
CCCGTTGCTGATA	0.024	0.003	0.024	46	1895
GCCGGTACAGGCG	0.058	0.016	0.062	57	857

CCCAGCATATACG	0.027	0.001	0.028	85	2910
GGCATTATATACG	0.063	0.004	0.058	114	1866
ACCATTGCATATG	0.027	0.005	0.022	62	2813
ACCATCGCAAATA	0.021	0.003	0.018	98	5290
CCCGTCGTATGCG	0.022	0.005	0.02	31	1489
AGCATCGTTTATG	0.023	0.002	0.024	43	1768
CGTATTGTAAACA	0.023	0.006	0.017	43	2499
CGCGTTATAGGCA	0.02	0.001	0.021	34	1621
CCCATCATTGGGG	0.037	0.009	0.027	30	1073
CGCGGCACAGACA	0.025	0.003	0.028	60	2092
ACTAGTGCATATG	0.026	0.006	0.018	36	1997
CCTGTTATTAAGG	0.027	0.002	0.026	36	1335
ACTATTATAGGCA	0.023	0.003	0.019	57	2886
AGCGGCGTATATA	0.02	0.002	0.017	28	1575
AGTATTATAGGTG	0.027	0.005	0.02	28	1380
GCCATTATTTAGA	0.055	0.006	0.047	106	2158
ACCATCACTTAGA	0.023	0.002	0.021	76	3562
GCTGTTATTGGTA	0.069	0.006	0.067	60	830
CCCATTGCTGGCG	0.023	0.005	0.016	27	1632
GGCATCATTGATG	0.09	0.015	0.072	108	1398
CGCAGCATAGATG	0.024	0.004	0.029	46	1566
CCTGTCATAAGGG	0.045	0.002	0.044	51	1101
CCTGGTACTAACA	0.03	0.001	0.03	67	2204
GGCGGTATAGATG	0.117	0.009	0.11	104	843
ACTATTATAGGTA	0.027	0.002	0.025	70	2714
ACTATCGCTGGTG	0.023	0.003	0.027	29	1061
GCTAGCATAGAGG	0.077	0.009	0.066	51	721
CGTATTACAGACA	0.03	0.001	0.028	66	2285
CCTAGCGCAGAGG	0.024	0.007	0.022	21	934
GCTATTATAAAGA	0.069	0.004	0.065	191	2759
CGCAGTATTTAGA	0.024	0.007	0.017	30	1707
GCCATCACTAATA	0.048	0.01	0.04	125	2984
CGCGTTATTAGTA	0.029	0.002	0.029	49	1630
ACCAGTACTGATG	0.024	0.001	0.023	67	2897
CGCAGTGCAAACA	0.029	0.001	0.028	74	2591
GCTAGCGTAAGCA	0.067	0.002	0.064	80	1169
CGCGGCGCTGAGA	0.02	0.002	0.023	21	889
CCTGTCACATGCA	0.021	0.002	0.019	36	1848
CCCGTTGCATGCA	0.025	0.004	0.019	41	2085
CGCAGTGCAGATA	0.021	0.002	0.022	35	1590

ACCATCGTAGATG	0.026	0.005	0.019	41	2095
CCCATTATAGGTG	0.03	0.006	0.023	42	1817
AGCGGTGTTGAGG	0.019	0.004	0.018	13	705
GCCATTGTAAAGA	0.045	0.003	0.047	107	2169
ACTAGCATATGTA	0.024	0.001	0.024	58	2400
GCCATTGTAGGCA	0.053	0.004	0.056	93	1573
ACCGGTGTAAACG	0.022	0.004	0.025	53	2040
ACCATTGCAAGCG	0.025	0.003	0.021	57	2658
CCCATTACTTGTA	0.029	0.004	0.035	90	2514
ACCGGCACTTATA	0.022	0.004	0.02	60	2919
CCCGTTACTAAGA	0.029	0.004	0.023	62	2602
GCTAGCACTGGTG	0.054	0.006	0.046	29	598
ACTGTTGCAAACG	0.027	0.006	0.019	38	2001
GCTAGTATTAACA	0.062	0.007	0.052	141	2554
CGTGTCATAGGCA	0.03	0.002	0.032	36	1076
CGCGGTACTTGGG	0.023	0.005	0.029	16	527
GGTGGCATATGGA	0.488	0.051	0.474	380	422
CCCGGTGCTTGTA	0.024	0.005	0.031	42	1322
AGCATCATTAGCG	0.019	0.001	0.019	40	2031
CCCAGCACTAGTG	0.024	0.004	0.029	50	1649
ACCAGTACTAATA	0.022	0.001	0.022	131	5836
GCCAGCATTAGTA	0.055	0.002	0.052	113	2043
AGTGTCACTTGTG	0.027	0.002	0.028	18	614
CCTATTACAAATA	0.027	0.001	0.026	131	4852
GGTATCGCAAACG	0.134	0.021	0.107	116	970
ACCGGTACAAGGA	0.024	0.004	0.03	78	2548
GCCGTCATATACA	0.05	0.005	0.049	137	2675
GGTGTTACAGATA	0.079	0.018	0.053	59	1056
CCCGGCGCTTACA	0.021	0.004	0.026	56	2105
ACCGTCATATATA	0.024	0.002	0.026	102	3777
CGCATCGTTGAGA	0.025	0.005	0.031	49	1524
GCTGTTATATGGA	0.07	0.017	0.048	45	896
GCTAGCATAAATG	0.061	0.006	0.065	114	1628
GCCGTTATTTATA	0.052	0.003	0.05	107	2017
AGTGTTGCTGACA	0.021	0.004	0.025	30	1178
CCCGTCACAGACG	0.028	0.001	0.027	64	2279
CGCGTCGCATATG	0.026	0.005	0.025	28	1107
ACCGGCGTATATG	0.022	0.005	0.018	15	829
GCCATTGCAAGCG	0.049	0.009	0.05	73	1385
GGCAGTATATGGG	0.06	0.004	0.059	54	861

ACTATCGCTTGCG	0.019	0.001	0.019	24	1218
ACCAGTATATACA	0.024	0.002	0.022	124	5436
GCCATCACTGAGG	0.058	0.003	0.057	71	1175
AGCGTCGTATACG	0.016	0.002	0.015	22	1492
ACTGGCACTAGCG	0.029	0.007	0.019	19	984
GCTGTTATTTGGA	0.054	0.011	0.04	31	737
ACCGGTGTAGGTA	0.026	0.005	0.019	27	1405
ACTGTCGCATGCA	0.026	0.012	0.017	28	1655
AGCATCGTTAACG	0.025	0.003	0.025	62	2383
GGTATCGCTGACA	0.097	0.023	0.088	87	906
AGCAGTGTATACA	0.021	0.002	0.021	62	2857
GCCGGTATTGACA	0.075	0.005	0.078	150	1781
ACTAGCATTTATA	0.031	0.004	0.026	73	2754
CGCATTGTTAACG	0.029	0.005	0.034	63	1774
GCCAGTATATGTA	0.05	0.004	0.053	127	2274
CCTAGTGTTAGCG	0.03	0.006	0.024	30	1198
ACCAGTGCAAGTA	0.024	0.001	0.022	68	2955
CGCATCATTTATG	0.025	0.004	0.025	49	1922
CCCAGCACAGATG	0.027	0.003	0.024	55	2279
CGTAGTATTAAGG	0.029	0.002	0.03	29	952
GCCAGCGCAAGGA	0.049	0.01	0.037	40	1041
CCCGGCGTAGGTA	0.018	0.002	0.019	28	1480
CGCAGCACTAACA	0.026	0.003	0.022	65	2838
ACCATCGTTTGTG	0.027	0.002	0.026	37	1366
CGTATCATATGTG	0.023	0.006	0.016	19	1155
GCCAGTACTAGGG	0.051	0.009	0.039	38	945
GGCATTATAGGTG	0.058	0.001	0.058	72	1179
GGCGGTGCTAAGG	0.069	0.018	0.077	45	541
GGCGTTGCAAAGG	0.056	0.008	0.065	55	786
ACTATTGTATGGA	0.026	0.003	0.022	39	1748
CGCAGCACAAGCA	0.025	0.003	0.022	51	2264
GCCATCATTAGGA	0.059	0.003	0.055	93	1602
ACCGTCACATATA	0.026	0.002	0.024	103	4210
ACTGTCATAGGTA	0.026	0.001	0.024	40	1597
GCTATTATATATA	0.064	0.001	0.063	191	2855
GCTATCATATAGA	0.063	0.002	0.062	127	1922
CGTGGTGTAAAGA	0.028	0.001	0.03	31	1003
GCCAGTATTGAGG	0.055	0.003	0.051	67	1256
GGCATCGTAAGGA	0.118	0.02	0.095	128	1220
AGTGGTGTATGGA	0.017	0.004	0.012	8	679

CCCGTTGCTTGCG	0.021	0.006	0.012	14	1165
ACCGTCACTAATA	0.023	0.003	0.027	114	4146
ACTAGCGCTAGCA	0.022	0.001	0.023	46	1967
CCTAGTGTATGCG	0.023	0.002	0.024	35	1437
GGTATTACTTATA	0.085	0.008	0.083	129	1417
CCTATCATTAACG	0.028	0.002	0.029	89	2996
CGTATTGCTGACA	0.022	0.001	0.021	29	1346
CCCATCGCTTATG	0.025	0.007	0.026	49	1828
GCTGTTATTGACG	0.061	0.003	0.059	60	954
CCCGGCATATGTG	0.022	0.002	0.023	34	1455
ACTAGTGTTAGTA	0.024	0.003	0.02	40	1941
GGCGGCATAAGTA	0.287	0.049	0.273	424	1127
ACCAGCATAAACA	0.024	0.003	0.023	148	6361
GCCAGCACTGGTG	0.069	0.005	0.064	61	885
CCTATCGTAAGGA	0.024	0.005	0.019	32	1692
CCTGGTATATATA	0.025	0.004	0.022	47	2124
GGCGTCGCTTGGG	0.064	0.019	0.052	23	423
GCCGGTATTAATA	0.082	0.002	0.082	191	2149
GCCAGCATTTACA	0.05	0.002	0.048	127	2538
CGTGGTACAAGGA	0.024	0.005	0.02	17	853
ACTATTGCTGATA	0.022	0.001	0.021	58	2747
ACTATTGTTGGTG	0.036	0.005	0.029	35	1185
ACCGGTACTTGGA	0.023	0.004	0.017	31	1795
AGCATCGCTAACG	0.022	0.001	0.023	54	2323
ACTATTGCAAAGG	0.027	0.003	0.022	53	2314
CGCGGTATATATA	0.025	0.005	0.028	56	1966
CGTGTTGTAAGGG	0.021	0.007	0.03	18	574
CGTAGCGCTAAGA	0.029	0.005	0.031	29	920
ACCAGTATTTATG	0.022	0.001	0.022	60	2698
ACCGTTATAAGTG	0.028	0.005	0.024	50	2059
ACTGTCATTTACA	0.026	0.004	0.031	74	2317
AGCATTATTTAGA	0.022	0.003	0.02	58	2863
ACTATTGTTGATG	0.029	0.004	0.023	42	1749
ACCAGCACAAACG	0.022	0.002	0.02	89	4443
GCTGGTGTAGATA	0.429	0.02	0.415	536	757
GCTATTATATGCA	0.058	0.003	0.054	114	2015
GCCGTCACTTACG	0.064	0.007	0.074	99	1244
CCTATTATTGGCG	0.024	0.006	0.016	29	1754
CGTATCATTAGGA	0.03	0.002	0.028	36	1233
GCTAGCGCAGGCA	0.065	0.01	0.051	44	823

CCCAGTATTGAGA	0.022	0.002	0.023	57	2375
CCCGGCGCAAATA	0.025	0.002	0.025	72	2835
CGTAGTGCAGATG	0.023	0.008	0.021	14	661
CGTGGCACTTGCA	0.019	0.004	0.015	12	783
ACCGGCACTAGTA	0.022	0.003	0.017	43	2427
ACTAGTGCTAGTA	0.02	0.002	0.017	36	2065
CCCAGTATTGATA	0.025	0.002	0.027	92	3267
ACTAGTATATATA	0.025	0.003	0.02	79	3781
ACCGTTGCTGGCA	0.024	0.006	0.016	30	1840
CCCGGTACAGACA	0.025	0.003	0.023	70	2920
ACTAGTGCAAACA	0.022	0.003	0.018	78	4255
CCCAGTATAAATA	0.024	0.004	0.019	108	5602
CCTATTGCATAGG	0.028	0.006	0.019	30	1528
CCTATTACATAGA	0.025	0.002	0.026	74	2727
GCTAGCATTAATA	0.061	0.007	0.053	115	2049
GGCGGCGTATGGA	0.215	0.025	0.196	126	518
CCCGTTATAGGGA	0.04	0.006	0.031	43	1334
ACTAGCACTGACG	0.03	0.007	0.029	52	1743
CGTAGTGTATAGG	0.028	0.002	0.026	16	599
AGCGGTATTAGTG	0.022	0.003	0.024	26	1039
GGCAGTATTTGTA	0.062	0.006	0.056	82	1370
CGTATTACTAGGG	0.031	0.01	0.024	20	817
ACCAGTGTAAGCG	0.024	0.004	0.018	40	2145
CCTAGTGCAAGCA	0.027	0.005	0.02	46	2304
ACCGGCGCTAACA	0.022	0.007	0.014	28	1931
CCCATCGTTGAGA	0.029	0.002	0.032	61	1858
CCTGGCGCATAGG	0.02	0.005	0.027	20	715
GCCGTTGCAAAGG	0.045	0.003	0.047	47	956
CGCGGCATTAAGA	0.025	0.009	0.019	28	1427
ACCAGTATAGATA	0.021	0.001	0.02	94	4577
ACCATTGCAAGGG	0.025	0.004	0.027	52	1884
ACCATCGCTAGCA	0.023	0.002	0.02	64	3130
ACTATCGCTTACA	0.022	0.002	0.023	64	2747
ACCAGCACAAGGG	0.031	0.002	0.03	51	1654
ACTAGTGTAGATG	0.025	0.004	0.031	54	1687
GGCATTACTAACA	0.058	0.005	0.059	164	2639
GCCATCACAAGTG	0.057	0.005	0.05	100	1898
GCTGTTGTTGATG	0.066	0.007	0.064	47	686
ACTATCGCAGGCA	0.026	0.002	0.024	50	2037
ACCATCACTTACA	0.022	0.002	0.021	114	5435

CGTGTTACTGGCG	0.028	0.002	0.026	21	777
ACTGGTGCTGACA	0.025	0.002	0.027	48	1704
CCTAGCACATATA	0.026	0.002	0.025	71	2800
CGCGTTGTAAGCG	0.026	0.003	0.022	24	1061
CGTAGTATAAGTA	0.022	0.005	0.028	47	1617
GGTAGTATAAGCA	0.06	0.008	0.05	69	1318
CGCGGTGCATGTG	0.02	0.007	0.012	9	714
AGTGTTGCTAGTA	0.022	0.002	0.02	19	933
CCTATTGTAAGTA	0.025	0.003	0.021	53	2529
AGTGTTACATGCG	0.028	0.005	0.024	24	977
AGTATTATAAAGA	0.022	0.005	0.016	54	3382
CGCAGCGCTGATA	0.024	0.002	0.023	36	1515
CGCAGTGCAGACA	0.023	0.006	0.019	36	1889
ACCGGTGCAAATG	0.027	0.004	0.022	45	1979
CCCATTATTTATA	0.025	0.001	0.024	95	3941
AGTATCATAGACA	0.024	0.004	0.024	66	2635
CCCATTGTATGCG	0.025	0.007	0.024	45	1853
ACTATCACAAACG	0.022	0.002	0.021	83	3914
AGTAGTACAAACG	0.025	0.005	0.018	35	1917
AGCATTACATGCA	0.022	0.004	0.027	80	2889
GCCGTTATTAACA	0.058	0.007	0.048	136	2682
CCCAGTGTTTGTA	0.025	0.002	0.022	36	1600
GCCGTTGCAGGGG	0.052	0.009	0.048	27	532
CCCAGCACAAACG	0.024	0.003	0.02	72	3488
AGTGTTATATGGG	0.02	0.004	0.016	12	737
CGTGTTGTAAGTG	0.018	0.003	0.022	18	785
CCCATCACAGATA	0.023	0.004	0.018	75	4137
CCCAGCGCTGGGA	0.02	0.002	0.022	29	1305
GCCAGCATAGATA	0.055	0.002	0.053	155	2770
GCCGTTATATGCG	0.056	0.008	0.048	66	1317
CCTGTTATAGATA	0.024	0.002	0.026	64	2368
ACCGGCACTGAGA	0.021	0.005	0.028	62	2186
CCCGTCATTAAGA	0.027	0.005	0.021	57	2651
ACTGTTATAGATA	0.025	0.003	0.021	61	2849
GCTGTCATTTGGA	0.06	0.006	0.067	51	713
GCTATTATTAGGA	0.06	0.001	0.058	81	1308
ACCATCACATATG	0.023	0.004	0.026	101	3749
ACTGGTGCTAAGG	0.03	0.004	0.031	27	842
CCCAGCACTGAGA	0.027	0	0.027	62	2219
AGTATTGTAAATA	0.024	0.001	0.024	75	3108

ACCATTGTAAACG	0.027	0.001	0.026	96	3570
CCTAGTATAAGGG	0.035	0.006	0.036	48	1271
AGCATCGCTTGGA	0.024	0.004	0.02	25	1245
AGTGTCATATGGG	0.019	0.003	0.021	14	639
GCTATTGTATGGG	0.063	0.006	0.058	46	745
GCCATCATTGGCG	0.065	0.001	0.067	106	1481
CCCGGCGTATAGA	0.022	0.004	0.017	28	1603
CCCAGTGTATATA	0.027	0.005	0.027	74	2627
ACCGTTACAGATG	0.024	0.002	0.026	71	2638
CCCGTCATATACG	0.026	0.003	0.025	63	2505
ACCATTGTATGTA	0.024	0.003	0.026	71	2645
ACTGTTGTAAATG	0.022	0.002	0.019	35	1812
AGTATTGTTTATA	0.026	0.001	0.025	49	1946
GCCGGTGTATACG	0.397	0.053	0.393	501	773
CGTATCATATGGG	0.03	0.004	0.031	27	853
CGTAGTATAGATA	0.029	0.006	0.02	35	1709
GGCGTCGCAGGTA	0.065	0.02	0.075	60	745
AGCAGCGCTAGTG	0.018	0.006	0.017	19	1081
GCTATCATTGGGA	0.059	0.016	0.076	70	847
CCCATCGTTTGTG	0.023	0.004	0.026	39	1488
GCTAGCGTAGACG	0.091	0.011	0.077	71	847
ACTATTACATGTA	0.023	0.001	0.024	84	3420
CGTATTGCAAACA	0.026	0.007	0.019	45	2380
GGCAGCACAGGTA	0.051	0.01	0.036	48	1272
CCCGTTACAGATG	0.03	0.002	0.028	60	2068
ACCAGTATAGATG	0.021	0.003	0.022	70	3103
CCCATCACATGTG	0.026	0.004	0.021	47	2224
ACCGTCACAGGTA	0.025	0.002	0.026	71	2649
AGTAGTACTTGCA	0.026	0.006	0.021	25	1148
CCTATTGTAAACA	0.028	0.002	0.026	112	4151
CGTGGTGTAAGCG	0.032	0.009	0.024	14	565
ACTGGCGTATACG	0.035	0.005	0.036	23	620
ACCATCACAGATA	0.02	0	0.02	112	5424
CGCGTCACAAGGA	0.025	0.004	0.019	29	1469
ACCAGCGCTTACG	0.02	0.004	0.016	31	1918
GCCGTTGTAAGGA	0.044	0.011	0.032	35	1070
ACCATTACTGATA	0.021	0.001	0.02	108	5180
CCCGTTACAGGCG	0.024	0.005	0.029	48	1601
GCCAGCATTAACA	0.052	0.006	0.056	188	3193
ACTATCATTGAGG	0.03	0.001	0.03	52	1677

CGCATTGTATACA	0.024	0.002	0.022	57	2484
ACCGGCGCTAGTA	0.026	0.009	0.015	17	1146
AGTATCACAAGTA	0.025	0.003	0.029	50	1680
AGTATTATTAGTA	0.025	0.003	0.022	51	2298
ACTAGCGCTGGCA	0.023	0.001	0.022	38	1673
AGCGTCACAAATA	0.022	0	0.021	78	3575
AGTATTGTAAGTG	0.025	0.004	0.023	31	1294
ACCGGCGCTTGGA	0.024	0.005	0.022	17	752
GGCGTTGTTGATG	0.071	0.009	0.059	47	756
AGTGGCGCAAGCA	0.019	0.007	0.019	18	941
AGTGTCATTGACA	0.02	0.001	0.021	32	1518
AGCGGCGCTAGGG	0.026	0.005	0.033	18	526
GGCGTCACTAACA	0.064	0.01	0.058	113	1850
CCCATTATTGATA	0.028	0.001	0.029	113	3745
CCCGGTATAAATA	0.028	0.002	0.026	101	3837
GCTAGTATTGGCG	0.059	0.008	0.047	42	843
ACCAGCGTTGATA	0.022	0.002	0.019	43	2182
CCCATTGTAAACG	0.026	0.003	0.022	74	3362
AGCGTTATATATA	0.021	0	0.021	69	3184
CGCGGTACAAATA	0.021	0.007	0.015	41	2678
GGCGGCATAAATA	0.303	0.053	0.276	651	1707
ACTATCACAGGGA	0.025	0.003	0.022	34	1495
GCCGTCGCAGGTG	0.051	0.012	0.068	39	532
GGCATTGCTTAGA	0.051	0.01	0.062	60	908
GCCAGTATAGGTA	0.052	0.006	0.046	91	1906
ACTATTGCTTACG	0.026	0.005	0.021	45	2091
GCTATCATTAACG	0.066	0.01	0.053	111	1995
CGCAGCGCAAGTA	0.024	0.003	0.02	35	1688
AGCATCATAGATG	0.025	0.002	0.022	62	2740
AGCAGTACTAACG	0.02	0.004	0.025	64	2489
CGCATCACAAGCA	0.027	0.004	0.021	61	2854
CCTATTATTAGGA	0.028	0.005	0.027	56	1981
CCTATCATTGGTG	0.029	0.005	0.022	32	1404
CGTAGCACTTATA	0.025	0.002	0.022	29	1296
GCCGTCGCTTGTA	0.046	0.003	0.049	40	779
CGTATTATTAGTA	0.025	0.004	0.02	40	1967
CCCGGTATAAAGG	0.027	0.003	0.024	47	1891
ACTAGCACATACG	0.026	0.002	0.026	59	2220
GCTGGTATTAATA	0.16	0.024	0.147	253	1472
CGTGTCATTTACA	0.022	0.002	0.024	34	1356

**CCTATCGTTGACG**	0.028	0.002	0.028	51	1764
**GCCATTGCATGGA**	0.046	0.008	0.035	40	1102
**ACTATCGCTTGCA**	0.028	0.005	0.021	41	1936
**ACCGGTATTTACA**	0.017	0.003	0.012	36	2935
**CCTGTTACAAACG**	0.029	0.003	0.025	55	2123
**CGCGTCATAAGCA**	0.023	0.002	0.024	54	2195
**CGCAGCACAGGCG**	0.029	0.002	0.03	38	1228
**AGTATTGCTGGCA**	0.028	0.008	0.021	23	1076
**CCTAGCATTTATA**	0.026	0.001	0.026	64	2367
**CCTGGTACTAATA**	0.026	0.002	0.028	62	2160
**ACCAGCATATATA**	0.025	0.001	0.027	125	4528
**AGCGGCGCATGCG**	0.022	0.007	0.02	18	888
**CGCAGCATTAACG**	0.021	0.002	0.023	49	2069
**CGTGGTGTAGACA**	0.022	0.006	0.015	14	925
**CCTGGCGTTAAGG**	0.019	0.003	0.017	14	813
**CGCGTCGCAAAGG**	0.031	0.007	0.023	26	1120
**GCTGTTATTAACG**	0.058	0.006	0.059	79	1253
**ACCATCATAAGTG**	0.025	0.006	0.02	67	3207
**CCCAGTGCAAATA**	0.025	0.005	0.019	70	3600
**CGTAGTGCAGGCG**	0.026	0.005	0.029	17	560
**CGCGTCACTAATA**	0.025	0.002	0.022	54	2370
**CCTGGCATTTACA**	0.024	0.003	0.021	39	1844
**CGCAGTATTAACA**	0.026	0.004	0.022	69	3088
**CGTGGTACTGGGA**	0.025	0.008	0.032	16	485
**AGCATTATAGGGG**	0.019	0.003	0.015	27	1808
**GCCATCGTAGAGG**	0.057	0.011	0.06	65	1021
**ACTGTCACATGCA**	0.027	0	0.026	57	2107
**CGTAGTGCTAATA**	0.024	0	0.023	28	1174
**AGCATCACTGACA**	0.021	0.003	0.018	61	3306
**CCTAGCATAGGTA**	0.029	0.005	0.031	55	1740
**CCTGGCACATAGA**	0.023	0.006	0.016	23	1374
**GCCATCACTTAGA**	0.051	0.01	0.037	65	1715
**GGTGTCATATATA**	0.099	0.018	0.074	89	1112
**AGCATCATTTAGA**	0.019	0	0.02	52	2576
**CCTATTATAAGTG**	0.029	0.006	0.024	59	2419
**CGCAGCACAAATA**	0.024	0.004	0.021	68	3224
**GCTGTTGTAAGCG**	0.056	0.013	0.049	42	819
**GCTATTACTAGCA**	0.056	0.002	0.058	101	1627
**GCCAGCATTTGCG**	0.044	0.006	0.035	46	1283
**ACCGTTACTTACG**	0.024	0.002	0.022	57	2593

GGTGGCACTGATA	0.198	0.039	0.183	160	715
CGTATTATTTGTA	0.024	0.002	0.025	40	1588
CGCGGTGTTGGGA	0.02	0.002	0.018	11	606
CGCATTACAAGTA	0.021	0.004	0.023	58	2490
CGCGGTGCTTGCA	0.019	0.006	0.013	14	1042
CCTATTGCTTGTA	0.023	0.003	0.022	39	1768
CGCGGCGTAAACG	0.022	0.004	0.023	30	1255
CGTGTTGTAAATA	0.025	0.004	0.025	43	1677
GGCATTGTATGGG	0.069	0.013	0.052	37	669
CCCATCATAAACA	0.024	0.001	0.023	159	6849
ACCATCATATATG	0.026	0.003	0.022	87	3822
CGTAGCGTAAGCG	0.026	0.005	0.022	19	833
ACTAGTACTAGGA	0.019	0.003	0.017	38	2188
ACTATTATTTATA	0.025	0.001	0.024	99	3990
ACTAGCATAAGCA	0.027	0.001	0.025	75	2943
AGCATTACTTGTG	0.021	0.005	0.026	44	1660
GCCGTCACAAGCA	0.064	0.012	0.053	116	2070
GCTAGTGTTAGCA	0.057	0.002	0.06	79	1234
CCTATTGTTAGGG	0.03	0.003	0.025	26	1006
AGTGGTACATACA	0.026	0.006	0.022	37	1678
ACCGTCGCTAATA	0.021	0.003	0.024	71	2877
CCCAGCACTGGCA	0.03	0.003	0.034	78	2250
GCTATCATAAGGA	0.068	0.004	0.064	97	1427
GCTATCGTATAGG	0.057	0.003	0.053	46	825
CGCAGTACTAACA	0.021	0.003	0.018	52	2913
CCTGGCGTAGACG	0.027	0	0.027	28	1008
GGCATTACTAGGG	0.061	0.002	0.064	55	800
ACTAGTGTAGAGG	0.024	0.001	0.022	29	1272
ACTATTGCTTGCA	0.025	0.001	0.024	54	2201
GCCATCGTTGAGG	0.053	0.007	0.044	42	915
GGTAGTACATGGG	0.06	0.011	0.061	31	476
AGCATTACATGGA	0.021	0.003	0.018	36	2002
CGCAGCACTAGGG	0.024	0.005	0.019	17	889
ACTGTTATTGAGG	0.022	0.005	0.02	22	1088
CGCAGCACAAATG	0.024	0.003	0.023	50	2163
GCTATCACAAACG	0.066	0.006	0.058	119	1927
GCCGTTGCTAATA	0.039	0.002	0.038	58	1480
ACCATTACAGACA	0.022	0.001	0.022	139	6238
CCTAGTGCATATA	0.02	0.003	0.018	43	2348
CCCATTGCAAAGA	0.024	0.002	0.025	83	3185

GGTGTCACTAGTA	0.077	0.011	0.065	44	637
AGCGTTACTTACA	0.021	0.003	0.025	59	2312
CCCATCACTTATG	0.026	0.007	0.02	52	2531
CGCATTGTTGGTA	0.031	0.002	0.028	35	1221
ACTGTTACAAGTA	0.023	0.008	0.019	49	2560
CCTATCGCTAGCA	0.025	0.005	0.022	48	2097
CCCGTTACTGGGG	0.048	0.004	0.043	37	823
CCTATTGTATAGG	0.026	0.002	0.025	40	1575
GCTAGCACTTATA	0.067	0.009	0.059	91	1440
ACCATTACATATA	0.025	0.001	0.023	152	6413
CGCATCGTTTGTA	0.019	0.002	0.021	33	1520
ACTGGTACTTGTA	0.024	0.005	0.019	31	1588
GCTAGTATAAGCG	0.058	0.004	0.058	76	1241
GGTGGCACATGTA	0.198	0.035	0.175	145	682
AGTAGTATTGGCG	0.021	0.006	0.012	12	967
ACCATCATAAGTA	0.025	0.002	0.026	129	4794
AGCGTTATTAGGG	0.021	0.007	0.013	13	1011
CGCAGCGCTAGCG	0.023	0.003	0.019	19	998
CGTATTGCAAAGA	0.025	0.002	0.022	35	1550
ACCATCATTTGTG	0.023	0.002	0.019	46	2314
AGCGTTACTAACA	0.023	0.003	0.021	65	2980
ACCATCGCTAACG	0.022	0.001	0.021	60	2759
CCCGTTGCAAGTG	0.02	0.006	0.016	25	1508
ACTGTCGTTGACA	0.028	0.006	0.022	35	1592
ACCGTTACATGGA	0.023	0.001	0.024	58	2325
CCCATCGTAGACG	0.022	0	0.022	55	2451
CCCGGCGTAAACA	0.023	0.003	0.021	70	3219
ACTATCGCATGTG	0.032	0.008	0.027	41	1474
CGCATTACTTACG	0.026	0.001	0.027	47	1679
CGCAGCATTGAGA	0.027	0.002	0.024	37	1503
CCTGTTGTATATA	0.024	0.002	0.027	53	1935
ACTGTTGTTAGCG	0.03	0.002	0.028	31	1068
CGTGGCACTTGGG	0.032	0.01	0.034	13	365
GCTATCGCAAACA	0.053	0.006	0.045	88	1880
CCTAGCATAAGCG	0.024	0.004	0.027	48	1762
CCTGGCGTAGATG	0.025	0.01	0.025	23	879
GCCAGCATAAAGG	0.057	0.008	0.047	87	1759
AGCGTCGCTTACA	0.025	0.004	0.024	43	1785
CGCATCATTAAGG	0.028	0.003	0.023	41	1740
ACTAGTATAGACG	0.026	0.003	0.027	64	2292

CCCATTACAAAGA	0.024	0.002	0.021	93	4286
ACTATTATTAGCA	0.03	0.004	0.026	83	3054
CCCGGTGTTTACA	0.025	0.003	0.03	56	1814
CCTAGTGCATAGA	0.019	0.003	0.017	30	1778
ACCATCGTTAACA	0.024	0.001	0.026	100	3780
CCCAGTGTTTACA	0.026	0.003	0.028	71	2427
AGCGGTATTGACG	0.021	0.002	0.023	31	1293
CCCATCGCTAGCG	0.025	0.004	0.03	56	1830
CCTGGTGTTAGCA	0.022	0.002	0.021	27	1262
ACCATCGCAAAGA	0.022	0.001	0.021	81	3770
CCCGGTACTAGTA	0.027	0.002	0.024	47	1894
GCTGGTGTATACA	0.426	0.039	0.435	629	817
AGCAGTATATATG	0.019	0.001	0.02	55	2643
GCCAGCACTAACA	0.054	0.001	0.054	166	2889
CCTATCGCATGGA	0.031	0.003	0.027	40	1443
CCCATTGTATACA	0.026	0.003	0.026	95	3502
ACCGGTACTAACA	0.023	0.001	0.022	102	4575
AGCGTCGCAGGTG	0.021	0.008	0.032	27	827
ACCAGTACATAGA	0.023	0.001	0.024	102	4110
CCCATTGCATAGG	0.028	0.004	0.028	46	1625
CCTGGCGTAAACA	0.022	0.001	0.021	51	2333
AGTATCATAAGGA	0.022	0.007	0.023	40	1701
CCCATTATTTGGG	0.024	0.004	0.026	34	1284
GCCGGTGCTTGCA	0.065	0.003	0.064	61	896
GGCAGTGCATAGA	0.051	0.004	0.046	51	1052
CCCATCGTTGACG	0.025	0.001	0.026	57	2122
CGTATTGCAAGCG	0.031	0.005	0.038	41	1040
AGCAGTGTTTACG	0.029	0.007	0.03	47	1504
AGTATTGTAAAGA	0.025	0.007	0.023	50	2110
ACTGGTACAGGCA	0.021	0.003	0.017	32	1813
CCCGTCGCATACG	0.026	0.004	0.024	48	1920
CCCGTCACTTGTA	0.026	0.003	0.03	60	1916
GGTGGCATAGATA	0.486	0.062	0.442	614	774
CGCAGTACATAGA	0.026	0.004	0.026	50	1852
CCCATCACTGGGA	0.042	0.006	0.033	57	1646
ACCAGTGTAGATG	0.029	0.003	0.026	53	1954
GGTATCATAAACA	0.123	0.024	0.095	223	2123
CCCATTGCTAGTG	0.024	0.004	0.021	35	1617
AGCGGTATAAACG	0.025	0.003	0.023	51	2142
ACTGTCATATACG	0.031	0.004	0.025	49	1926

AGTAGCACTAAGA	0.026	0.009	0.015	21	1427
CGTGTCGCTTATG	0.027	0.007	0.028	21	738
ACCGGCACAGATA	0.023	0.001	0.022	73	3251
CCCGGCGTTGAGA	0.02	0.003	0.017	24	1381
ACCGGCACTAACG	0.022	0.002	0.025	62	2468
CGCGTCATATGCA	0.025	0.007	0.02	37	1844
GCTGTCATAGGGG	0.077	0.002	0.074	36	451
CCCGGTACTAACG	0.025	0.005	0.019	40	2104
ACCATTGCAGGGA	0.02	0.005	0.014	29	2023
CGTAGTGTTTGTG	0.024	0.006	0.017	9	507
ACTGGTGTAGATA	0.028	0.004	0.026	42	1596
ACTATCACTAGCG	0.029	0.001	0.03	67	2152
AGCATTATTGACG	0.018	0.001	0.017	42	2436
CCCATTACAGATG	0.023	0.003	0.02	56	2814
CCTGGTATTGATA	0.026	0.007	0.017	28	1614
CCCATCATATAGA	0.026	0.002	0.023	81	3496
AGCGGTGCTGATG	0.027	0.004	0.022	22	956
CGTAGTGTTTGGG	0.037	0.005	0.044	15	323
CCCGTCGTAAGCG	0.022	0.005	0.019	31	1606
GGCATTGCTAGTG	0.066	0.005	0.067	53	742
AGCAGTACAAGGA	0.02	0.002	0.017	38	2206
ACCAGTGCTAGCG	0.023	0.002	0.019	37	1891
ACCGTCACAAACG	0.024	0.001	0.025	88	3429
ACTGTCACAAACA	0.023	0.002	0.021	82	3913
AGCAGTACTGGGA	0.021	0.002	0.019	26	1325
GGTAGCGTAAACA	0.091	0.008	0.086	120	1273
GGCATTGTTGGTG	0.069	0.002	0.069	48	651
CGCGGCACTTGGG	0.026	0.002	0.026	14	525
AGTGGTATATGTG	0.03	0.006	0.034	22	626
CCCAGTACTGGCG	0.029	0.007	0.034	56	1593
CCCGTCGTTTGGA	0.019	0.007	0.013	15	1159
CCTGGTATTGATG	0.024	0.002	0.022	26	1135
ACCAGTATAAATG	0.026	0.002	0.026	115	4310
GCCATTGTTTGTG	0.047	0.009	0.041	41	956
GGCGGTACTGGCA	0.062	0.017	0.063	57	855
AGTATTGCATACG	0.024	0.006	0.031	44	1363
CCCAGCATATATA	0.029	0.002	0.027	108	3838
ACCGGTGCAAACG	0.021	0	0.02	46	2212
AGCGTTACAAACA	0.021	0.003	0.017	65	3764
CCCAGCATTTGCG	0.022	0.003	0.02	34	1680

ACCATTACTTACG	0.025	0.002	0.022	86	3868
GCCAGTATAAGTA	0.054	0.003	0.049	144	2767
CCCGTTGCTTAGA	0.027	0.006	0.024	36	1442
ACCGGCGCAAATG	0.025	0.002	0.021	36	1672
GGCATTACATATA	0.057	0.004	0.052	137	2508
GCCAGTGCATATA	0.048	0.002	0.049	85	1646
CGTGTCGCTTAGA	0.02	0.01	0.027	20	722
CGTGGCGCAGGTA	0.028	0.005	0.021	12	563
ACTATCACATGTG	0.026	0.003	0.027	58	2095
GCCAGTGCATGCG	0.049	0.009	0.041	40	942
AGTATCGCATGCA	0.026	0.002	0.027	35	1264
AGTATCGTTGACA	0.021	0.002	0.018	33	1763
CCTATCGTAAAGG	0.023	0.005	0.016	29	1804
ACCATCGTTAACG	0.029	0.004	0.03	80	2558
AGCGTCACAAGGA	0.019	0.002	0.017	28	1638
AGTGTTGCTAATA	0.027	0.005	0.021	31	1476
CGTGGTATTAGGA	0.026	0.007	0.018	12	641
ACTATCGTAGAGA	0.023	0.005	0.017	29	1714
CCCAGTATAAAGA	0.027	0.002	0.027	107	3838
CCCATCACTAGGG	0.04	0.002	0.039	60	1468
AGCGTCGTAAGCG	0.028	0.001	0.028	40	1404
ACCATTGCTAAGG	0.02	0.001	0.021	47	2217
GGCGTTGCAAGGG	0.055	0.005	0.049	31	602
ACTATTGCTGAGA	0.027	0.008	0.019	41	2111
AGCATCGTAAGTG	0.022	0.004	0.018	35	1948
AGTGTCATATACA	0.02	0.005	0.026	53	1971
AGCGTTGCATGCA	0.02	0.001	0.021	32	1510
AGCGTCGCAGGTA	0.025	0	0.025	30	1186
CCTATTACAAACA	0.028	0.002	0.03	162	5251
ACCGGCGCTTACA	0.028	0.001	0.028	43	1467
CCTATTACTAAGG	0.025	0.005	0.018	34	1893
CCTGGTATAAAGG	0.026	0.001	0.027	39	1399
CCCGGCGCAAGTG	0.026	0.006	0.018	23	1237
CGTAGTGCTTGTA	0.029	0.004	0.025	18	707
ACCAGCGCAAGCA	0.023	0.004	0.021	76	3622
AGCGGTACAAGCA	0.02	0.002	0.019	40	2100
CCTGGTGCATATA	0.023	0.004	0.017	29	1639
GCCGGCATTTGCA	0.371	0.04	0.349	523	975
ACCAGTGTTTACG	0.025	0.001	0.025	47	1832
GGCGGCATATACG	0.56	0.062	0.528	914	818

CCCATTACAGAGA	0.024	0.002	0.021	65	2964
GCCGGCACAAGCA	0.095	0.006	0.102	191	1680
ACCAGTACAGATA	0.021	0.002	0.021	111	5150
CCTGTCATAGACG	0.026	0.005	0.032	56	1689
ACTGTTACTGGGA	0.024	0.002	0.023	27	1153
ACCAGCGCTAAGA	0.018	0.001	0.019	61	3124
CGTAGCGTAAGCA	0.02	0.002	0.019	22	1162
ACTGTCACATATG	0.03	0.005	0.024	42	1735
CGCAGCACTGATG	0.028	0.007	0.022	29	1272
GGCAGCGCAAATA	0.058	0.01	0.044	83	1789
CCCAGCGCTTGCA	0.023	0.001	0.023	55	2308
AGCGTCGTTGATA	0.023	0.002	0.02	33	1601
GGCATCGCAAGCA	0.088	0.005	0.093	154	1505
GGCGTTACATATG	0.066	0.012	0.049	57	1101
GGCGTCACATAGG	0.066	0.007	0.057	52	861
AGTAGTATTTGCA	0.024	0.003	0.02	30	1449
ACTGTCACAGAGG	0.025	0.004	0.027	32	1154
ACTGTTGCAAACA	0.025	0.001	0.024	77	3080
GGCGTTGCAGACG	0.067	0.012	0.054	46	806
GCCATTACAAATA	0.051	0.005	0.046	183	3806
GGTATTACAAATA	0.089	0.018	0.075	188	2325
GCCGTCGTTTATG	0.052	0.006	0.044	32	700
GCTGGTATTAAGA	0.165	0.014	0.164	200	1020
GGCATTGTTAATA	0.074	0.012	0.063	127	1902
CGCATTATTGACG	0.025	0.004	0.03	58	1880
GGCAGCGTATGGG	0.055	0.003	0.055	36	622
GCCAGTATATATA	0.053	0.002	0.052	183	3370
AGTATTACTAGGA	0.025	0.001	0.025	33	1303
ACTAGTATAAATA	0.023	0.001	0.022	114	4989
ACCGGCACATGTA	0.027	0.002	0.027	75	2677
CCCGTTATAGATG	0.031	0.001	0.033	74	2147
AGCGGTACTGATA	0.026	0.008	0.016	31	1852
AGCAGCGCTTATA	0.022	0.005	0.025	45	1786
CGCATCATTAAGA	0.024	0.004	0.03	85	2791
ACCATTGTATGTG	0.025	0.001	0.026	48	1800
CCTAGTACAGACG	0.021	0.003	0.017	31	1823
GCCGGTATAAGGA	0.077	0.01	0.075	112	1385
CCTAGCACTTGCG	0.03	0.008	0.026	29	1096
ACTGTTATAGACG	0.026	0.003	0.023	44	1835
CCTGTCGCTAGTA	0.022	0.003	0.022	28	1237

ACTGTTATAAATG	0.025	0.002	0.025	70	2676
CGCATCACTAACA	0.029	0.004	0.025	88	3464
CCCGTTGTTAATA	0.021	0.002	0.019	50	2548
CCCAGTACAAGTG	0.03	0.003	0.027	58	2106
ACTATTATATACG	0.03	0.006	0.023	78	3259
AGCGTCGTAGACA	0.02	0.002	0.017	35	2046
AGCATTACAAGTG	0.02	0.004	0.015	39	2509
AGCGTTGTAAGGA	0.02	0.005	0.019	27	1432
GGCGGCACATACA	0.134	0.017	0.138	232	1455
GGCATCACTAGCA	0.077	0.006	0.068	119	1630
GGCATTACAAATA	0.057	0.004	0.052	182	3334
CGTAGCACATATA	0.028	0.01	0.017	28	1615
CCCGTCACATGGA	0.022	0.006	0.015	28	1795
ACCATTGCTAACG	0.021	0.002	0.019	63	3312
CCCGGTGTTGACG	0.023	0.004	0.023	33	1414
CCTAGTATTGACA	0.021	0.001	0.02	51	2525
GCCGTTATTAGTG	0.05	0.012	0.034	39	1100
GGCGGTACAAGGA	0.061	0.009	0.059	57	904
GGCAGTGCTGACA	0.049	0.003	0.046	58	1214
ACCGGCATAGACG	0.026	0.003	0.022	45	1988
AGCATCGCATAGG	0.024	0.004	0.023	36	1528
AGTGTTATTGATG	0.022	0.006	0.03	33	1079
GGCGTTATATAGG	0.059	0.011	0.051	47	880
GGCATTACTGATG	0.061	0.007	0.056	78	1305
CGCGGTATAAGCA	0.023	0	0.022	40	1738
ACCATCACATACA	0.024	0	0.024	163	6746
CCTATTACTAGTA	0.025	0.003	0.022	57	2511
CCCGGCACTGGGA	0.033	0.007	0.023	28	1187
ACCGTTATTAACA	0.024	0.004	0.019	82	4143
GGCGGTGCTGGTA	0.06	0.017	0.049	29	562
ACTGTCACAGATA	0.023	0	0.022	59	2564
ACCATTGTAGGTA	0.023	0.004	0.017	39	2249
ACCGGTATAGATA	0.025	0.002	0.023	78	3256
CGTGTCGCAAGTA	0.027	0.004	0.032	32	975
CCTATCACAAGCG	0.029	0.005	0.022	52	2289
AGTGGTACAGGGG	0.027	0.005	0.023	11	467
CCCATCATTTATG	0.026	0.004	0.032	85	2573
ACTAGTATATATG	0.026	0.001	0.025	62	2455
ACTGTCATAAAGA	0.025	0.002	0.025	66	2539
ACTGGCATAAACA	0.028	0.003	0.024	75	3057

GCTAGCGCTTATA	0.052	0.003	0.048	49	974
ACTATTGTTGGGA	0.026	0	0.026	35	1289
ACCGGCGTATGCA	0.028	0.004	0.026	39	1486
ACCGGTGCAGAGA	0.02	0.007	0.018	32	1790
GGTATTGTTAACA	0.107	0.017	0.085	133	1428
ACTAGCACAAGGG	0.027	0.002	0.025	33	1268
AGTGGTGCAAGTA	0.025	0.009	0.016	15	939
ACCAGTACTGATA	0.021	0	0.021	99	4573
ACTATCGTTAGTG	0.024	0.003	0.023	29	1237
GCTAGTGTATAGA	0.057	0.001	0.058	75	1219
CGTGTCACAGGCA	0.024	0.008	0.018	19	1028
AGTAGTACATACA	0.024	0.005	0.017	39	2271
GGCAGTGCTAATA	0.06	0.008	0.053	81	1445
CCCATTACATACA	0.027	0.003	0.023	120	5027
ACCATCACAAGCG	0.026	0.002	0.025	87	3404
CCTGGTATATAGA	0.029	0.007	0.033	56	1653
ACTGGCACATACA	0.028	0.002	0.025	61	2406
CCTAGCGTAAACA	0.026	0.003	0.023	73	3098
CGCGTTGCAGGGG	0.026	0.006	0.023	14	592
CCTAGCATTTATG	0.025	0.005	0.019	31	1567
ACCAGTGCATATG	0.023	0.002	0.019	45	2292
ACCATTATAAATA	0.024	0.001	0.025	197	7830
GCTATCATATATA	0.067	0.01	0.054	146	2572
CCCAGTGTAGGCG	0.019	0.002	0.019	30	1529
CGTGTCGTAGAGA	0.025	0.009	0.033	28	810
AGTATCGTATACG	0.023	0.003	0.022	34	1506
GGTAGTACAAGGA	0.053	0.01	0.046	46	964
ACTATCGTAAAGA	0.022	0.001	0.021	57	2599
ACTGTCATTTGGG	0.037	0.002	0.038	33	825
GGCAGTACATGTG	0.05	0.001	0.049	48	923
CGTATCATAGGGG	0.025	0.01	0.021	14	646
CCCGTTGCAGGCA	0.021	0.004	0.016	30	1797
ACTATCGCTTGGA	0.022	0.002	0.018	26	1381
AGTGTCGTAGATG	0.024	0.003	0.02	17	847
GGCGTCATATGCA	0.056	0.011	0.041	55	1290
CGTGTTGTTAGCG	0.023	0.011	0.015	9	597
GCTGGTATATATA	0.141	0.024	0.138	248	1544
CGCATTGCTAATA	0.025	0.003	0.03	68	2217
GCCATCATAAATA	0.053	0.004	0.049	231	4528
ACCAGTGTAAGCA	0.025	0.001	0.024	66	2732

CCTATCGCAAATG	0.028	0.006	0.021	47	2197
GCCAGCATATATG	0.055	0.004	0.053	111	1999
GGTATCATTTGTA	0.125	0.017	0.111	116	933
ACTGTCATTAACA	0.027	0.003	0.024	73	2963
AGCAGTGCTAGGA	0.024	0.002	0.022	30	1350
CGCATTGCAGGTG	0.028	0.006	0.026	25	931
CCTGTTATATGCG	0.022	0.005	0.015	23	1503
ACCAGTACAGATG	0.02	0.001	0.019	63	3257
GCCAGCACATACG	0.058	0.003	0.061	130	1986
GCCGTCATAAACA	0.053	0.008	0.05	172	3240
GCCGGCGCTTGTG	0.762	0.049	0.77	641	191
ACCATTATTAAGG	0.024	0.004	0.018	56	3016
ACCAGTACTTAGG	0.025	0.001	0.025	55	2186
ACCAGCGCTTAGA	0.019	0.004	0.015	36	2329
ACCATTACAGATA	0.022	0.002	0.021	123	5876
CCCAGTATAAGGG	0.028	0.002	0.03	50	1629
CGTAGTACTAGCA	0.025	0.005	0.027	31	1099
GCTGGTGTATATA	0.407	0.026	0.396	528	805
CCTATCGTATATA	0.027	0.003	0.028	73	2567
CCTAGCATTAACA	0.029	0.003	0.029	91	3020
AGCGGCACTTGTA	0.022	0.002	0.022	27	1212
ACCGGCGTTAACG	0.028	0.002	0.03	29	930
CCTATCATTAGTA	0.023	0.002	0.02	57	2743
GGTAGTGTAAAGA	0.056	0.004	0.062	65	989
CCTGTTGTATGCA	0.025	0.004	0.023	35	1499
AGCGGCGCTTGTG	0.026	0.011	0.012	7	568
ACCGTTATAAACG	0.024	0.002	0.022	78	3457
ACTATTGCATGCA	0.026	0.002	0.023	63	2687
AGCATTATTAGTG	0.025	0.002	0.027	54	1965
GCTGTCACATGGA	0.06	0.012	0.044	36	787
CGTATTACAAGTA	0.018	0.003	0.015	31	1982
CGTGGTACAAAGG	0.028	0.01	0.018	15	807
ACTATCATAGAGA	0.03	0.004	0.028	73	2524
ACCGTCGCTTGGG	0.027	0.005	0.026	21	787
ACCATCACTGAGA	0.025	0.001	0.024	83	3417
AGCAGTGTAAATG	0.023	0.002	0.02	47	2330
ACCAGTGTTAATG	0.026	0.004	0.023	48	2079
CGTGGTACATGCA	0.033	0.008	0.044	43	945
GCCGTCGCAAACA	0.046	0.002	0.044	81	1769
GGTATCATTAGGA	0.132	0.021	0.112	106	842

GCCATCGCTTACA	0.046	0.004	0.042	73	1682
CCCATTGTTTGGA	0.024	0.005	0.02	29	1424
ACCATTATAAGGA	0.024	0.003	0.024	89	3599
ACTAGCACATACA	0.023	0.001	0.022	89	4011
ACCGTCATAAACA	0.024	0.002	0.022	120	5293
AGCAGTATTGGTA	0.022	0.002	0.022	46	2035
AGCATTGTATGTA	0.019	0.004	0.013	30	2250
CGCATCGTATGTG	0.033	0.004	0.028	38	1316
ACCAGTGCAAATA	0.023	0.002	0.022	110	4830
CGCAGCACTGGGA	0.024	0.003	0.026	23	864
AGTAGTATTTATG	0.024	0.003	0.025	38	1482
CGTGTCACTAGGA	0.018	0.005	0.025	20	773
ACTGTCACTGGCG	0.02	0.004	0.015	16	1052
GCCATTACTAACG	0.056	0.004	0.05	116	2212
AGTGGCGCTTATA	0.025	0.002	0.029	24	804
GCCATCGTAGATG	0.051	0.003	0.048	75	1492
ACTGGTGTAAGGA	0.026	0.005	0.02	21	1026
GCTGTCACTGGCG	0.058	0.013	0.041	32	751
GGTAGCATATGGA	0.057	0.01	0.044	32	698
GCCGTCACTGGTA	0.053	0.004	0.055	66	1129
GGTATCACAAGTG	0.118	0.024	0.096	79	743
CGCATTACTGATA	0.027	0.004	0.022	49	2211
GGCGGCACTTATG	0.255	0.054	0.219	216	772
GCCATCGCTGATG	0.051	0.004	0.045	45	954
GGTAGCATAAATA	0.077	0.011	0.061	117	1795
ACCATCGTTGAGG	0.02	0.002	0.022	31	1379
GCTGGTGTAAATG	0.757	0.044	0.729	1136	422
GGCATCATTAAGA	0.082	0.01	0.071	156	2042
ACTGTTGCAGGCA	0.025	0.003	0.026	42	1585
CCCGGCATAAACG	0.025	0.004	0.029	82	2720
CGCATCACAGATA	0.025	0.001	0.026	74	2759
AGTATTACAAGGA	0.021	0.003	0.023	41	1706
CCCATCGCTTATA	0.024	0.002	0.026	77	2831
CCTATTGCTAACA	0.024	0.003	0.025	83	3226
ACCGTTACAAAGA	0.026	0.003	0.023	103	4297
CCCATTATATACA	0.027	0.004	0.022	120	5444
CCTGTCACAAGCA	0.022	0.002	0.022	51	2260
CGCATCGCAAACA	0.026	0.004	0.023	73	3151
CCCATCGCTAATG	0.026	0.002	0.023	57	2373
ACCAGCACAGACG	0.028	0.003	0.031	87	2730

GGCAGCATATGGG	0.058	0.011	0.047	43	872
ACCATCATAAATG	0.025	0.003	0.028	140	4925
GGCGGTGTAAATA	0.062	0.012	0.06	92	1443
GCTGTTGCTTATA	0.052	0.003	0.054	50	869
CCTATCGCTTGGA	0.023	0.004	0.021	26	1187
ACTGTCGTAGACA	0.024	0.003	0.021	36	1667
CCTGGCGTTTGGG	0.034	0.006	0.042	20	451
CGTGTCATAAATG	0.024	0.004	0.022	32	1451
AGTAGTATATATA	0.023	0.003	0.027	74	2682
CCCGTTGTTAACG	0.028	0.004	0.028	53	1849
AGCGTCGTTTATG	0.021	0.008	0.029	35	1179
CCTGTCACTAGCA	0.025	0.002	0.024	43	1781
ACTGTCGCTAGTG	0.023	0.006	0.03	29	941
GCTGTCATAGGGA	0.072	0.004	0.068	43	594
ACTAGTATATGGA	0.025	0.004	0.019	36	1814
ACTGGCATTAAGG	0.031	0.002	0.029	32	1061
CCCGTCACTTGTG	0.025	0.009	0.019	27	1395
AGTGGCATTAGCA	0.025	0.007	0.034	22	632
CGCGTTATAGACG	0.028	0.003	0.024	38	1536
CGTGGCGCAGATA	0.025	0.01	0.023	21	887
ACCGGTACTGAGA	0.021	0.003	0.016	41	2489
ACTGTCATAAATG	0.025	0.002	0.027	67	2413
ACCAGCACAAATA	0.021	0	0.022	155	7026
ACTATCATATACA	0.029	0.008	0.021	93	4305
GGCGGTGCATACG	0.067	0.014	0.067	59	823
GGTATTGCTGAGG	0.1	0.016	0.096	53	497
AGCAGCACAGAGA	0.022	0.002	0.02	41	1999
CCCATTGCTAAGG	0.023	0.001	0.025	44	1747
CCTGTTATAAGCA	0.024	0.004	0.02	46	2284
CCTATCGCAGAGG	0.025	0.005	0.026	33	1215
CCCAGCGCAAGCG	0.023	0.001	0.021	41	1945
CGTGTTACTAACG	0.025	0.003	0.025	31	1200
ACCGGCATAGAGG	0.022	0.004	0.024	30	1206
AGCGTTGTTAGTG	0.018	0.002	0.022	22	999
ACCGGCGCAAGCG	0.024	0.005	0.021	28	1318
ACCAGTACTAATG	0.022	0.002	0.02	76	3817
CCTGTCATTGGTG	0.023	0.006	0.025	24	944
CGTAGCGCAGAGA	0.025	0.003	0.021	17	808
AGTGGTGCATGCA	0.023	0.003	0.027	24	866
GCTAGCGCTGACA	0.059	0.012	0.043	49	1100

GCCGTTACTGGGA	0.052	0	0.052	45	816
CGTAGTGCATATA	0.027	0.007	0.035	41	1120
GCCATTGCTGGGG	0.041	0.008	0.038	23	581
AGTATCGTAAGGA	0.018	0.004	0.014	18	1305
CCCAGTGCATATA	0.022	0.003	0.022	62	2776
AGCATTGTAAACG	0.023	0.005	0.026	79	2907
CCCGTTATTTGTA	0.024	0.002	0.022	45	2017
GCCGGTACAAACA	0.061	0.002	0.059	159	2546
ACTGTTATATACA	0.024	0.003	0.02	65	3134
GGTATCGCAGAGG	0.126	0.03	0.105	60	513
ACCATTGTAAGCA	0.024	0.001	0.024	80	3292
GCTAGTGTAGACG	0.057	0.008	0.046	55	1152
AGCGGCACAGGCA	0.019	0.004	0.025	37	1459
AGCGGTATAAGGA	0.024	0.002	0.022	39	1722
CCTATCGTTAGGA	0.027	0.011	0.023	32	1380
AGCAGCGCATGCA	0.022	0	0.022	40	1762
CCCAGCACAGATA	0.02	0.002	0.022	76	3444
ACTAGTGCAGGGG	0.02	0.005	0.013	13	973
GGTATCACAGGCA	0.086	0.002	0.086	81	856
ACTGGCGCATACG	0.027	0.003	0.025	27	1043
ACTATTATTGAGA	0.026	0.003	0.029	80	2719
GCTGGCGTAAGGA	0.873	0.023	0.851	1079	189
GCTATTACTAATA	0.057	0.004	0.062	160	2431
GCTAGTGCTTGCA	0.05	0.005	0.056	57	965
AGCGGCATAAACA	0.022	0.004	0.017	43	2508
ACCATTGCAAGTG	0.023	0.003	0.024	59	2399
CCTATCATTAAGG	0.026	0.004	0.027	59	2165
AGCAGTACTGGTG	0.022	0	0.022	29	1263
CCTGGCATATACA	0.028	0.005	0.03	74	2355
CCCATCACAGGTA	0.025	0.002	0.022	62	2703
ACTAGCACTAACG	0.024	0.003	0.022	56	2440
ACCAGCGTAGATA	0.028	0.002	0.026	65	2434
ACTATCATAAGCA	0.026	0.002	0.024	80	3237
CGTAGCGTATGTA	0.023	0.005	0.021	20	935
AGCAGTATTAGTG	0.023	0.003	0.026	43	1627
ACTATCACTAGTG	0.023	0.002	0.025	49	1949
ACTATTGCTTATA	0.025	0.001	0.026	78	2868
ACTAGTATAAACA	0.024	0.002	0.025	130	5029
GCCATTATTAGTG	0.055	0.005	0.047	86	1725
ACCGTTGTATATA	0.025	0.002	0.024	63	2537

CGCGGCATTTGGA	0.023	0.01	0.012	10	820
AGCAGTGTTTATA	0.024	0.002	0.022	47	2079
CGCAGCACATATG	0.028	0.005	0.025	41	1604
ACCAGTGTTTGCA	0.028	0.002	0.03	58	1893
CCTGGTGTAGAGA	0.025	0.003	0.028	32	1096
CGTAGCATATACA	0.024	0.001	0.023	43	1851
GCCAGCATTGACA	0.052	0.006	0.054	137	2390
CGCGTCACAAACG	0.023	0.004	0.02	46	2209
AGTAGCACTGGCG	0.022	0.007	0.027	23	832
ACCAGTATTAGCG	0.024	0.001	0.025	64	2465
AGCATTGTAAGCA	0.022	0.001	0.023	68	2931
CGCATTGCATATA	0.027	0.006	0.018	40	2160
ACTGTCGCAGACA	0.024	0.001	0.023	50	2104
CGCAGCACAAACA	0.026	0.004	0.022	76	3378
GCTATTATATACG	0.058	0.007	0.05	105	1994
CGCGTCATTTAGA	0.021	0.002	0.019	26	1356
CGCGGCACTGACA	0.026	0.005	0.023	42	1815
GGCGTTACAAACA	0.058	0.002	0.06	148	2327
CGTATCACTTACG	0.035	0.009	0.026	32	1196
GGTATTGCAGACA	0.076	0.017	0.054	64	1121
AGCGGCATAGACA	0.023	0.003	0.022	41	1796
CGCAGTGTATAGA	0.021	0.002	0.019	28	1445
GCCGGCACAAGGG	0.224	0.009	0.212	185	689
CCCATCGCATATG	0.02	0.004	0.017	37	2170
CGTATCATTTACA	0.024	0.001	0.023	51	2198
AGTATCGTTTACG	0.023	0.002	0.02	26	1251
ACTATCGCTAACG	0.023	0.003	0.021	48	2209
GGCGGCACTTACA	0.133	0.038	0.096	138	1307
AGTAGCGTAGGGG	0.016	0.002	0.015	10	640
CCCATCATTGGGA	0.039	0.006	0.033	56	1652
ACCGGTGTATACA	0.029	0.008	0.036	89	2401
CGTATTATAGGCA	0.025	0.005	0.02	36	1770
GCCATCATTAGCG	0.053	0.002	0.052	94	1718
GCTGTCGCATATG	0.055	0.005	0.057	35	580
ACCGGCGTTTGCA	0.024	0.003	0.02	23	1131
ACCGGTATTAATA	0.024	0.004	0.02	68	3394
ACCAGTATAAAGA	0.023	0.001	0.025	126	4983
GGTATCGCTAACA	0.102	0.01	0.088	116	1195
CCTAGTACAAGTG	0.031	0.009	0.028	44	1555
AGCAGCACAAACA	0.019	0.002	0.018	74	4069

GGCAGCATTAACA	0.054	0.004	0.049	125	2405
ACCGTTACAAACG	0.022	0.003	0.019	75	3956
CGCATCATTGACG	0.027	0.004	0.022	43	1934
ACTATCATTTGCA	0.023	0.002	0.02	46	2275
CCCATTATTAGTG	0.024	0.002	0.024	55	2255
ACTGGCATATACA	0.024	0.003	0.023	59	2464
ACTGTCACAAGTG	0.03	0.003	0.029	41	1372
GGTAGCGCTAACA	0.07	0.009	0.058	55	893
ACTGGTGCAAACA	0.024	0.001	0.024	67	2674
ACCGTCGTAAGCA	0.024	0.002	0.021	44	2043
CGTATCGCAGATA	0.023	0.005	0.024	34	1411
ACCAGTATTGAGA	0.023	0.002	0.022	67	2958
AGCAGCACTTAGA	0.02	0.003	0.024	43	1778
CGTGTCGTTTACA	0.023	0.005	0.028	31	1079
ACTAGTATATACA	0.024	0.001	0.025	97	3814
AGCGGTGCTTATA	0.021	0.005	0.014	20	1379
AGCATTGTTGACA	0.021	0.005	0.019	50	2614
AGTGGTGCAGATA	0.024	0.006	0.016	17	1065
AGCATTGCTAGGG	0.024	0.005	0.017	18	1025
ACCAGCGCAAAGG	0.022	0.003	0.019	36	1885
AGCGGTGTTGATA	0.024	0.002	0.026	40	1475
GGCATCGCTAAGA	0.085	0.024	0.063	86	1286
GGTAGCATAAGTG	0.068	0.01	0.054	40	707
CCCAGCATAAATG	0.025	0.002	0.028	98	3444
ACCGGCGCTAGTG	0.022	0.004	0.028	28	972
GGCATTATAAATG	0.063	0.007	0.054	142	2512
GCCATTATAGACG	0.053	0.003	0.051	132	2471
ACTATTATAAAGA	0.026	0.003	0.025	117	4585
CCCATTATATATG	0.028	0.002	0.03	101	3311
AGCATCGCAGGCG	0.021	0.001	0.022	40	1779
CGTGTTGTTTACG	0.021	0.004	0.017	12	685
CCTATCGTTTATG	0.025	0.004	0.021	37	1707
AGCGGTGTTTGCA	0.029	0.005	0.023	28	1189
GGCGTCGTTGGCA	0.079	0.004	0.085	68	735
CCTATTGTTTATG	0.029	0.002	0.032	57	1748
ACCAGTACTAGGA	0.023	0.002	0.02	57	2822
GCTATCGCTTGCA	0.052	0.002	0.053	56	998
CCTGTCGTTGAGA	0.025	0.011	0.019	23	1213
CCCATTACTAGTA	0.029	0.004	0.026	79	2974
GGCATTACATATG	0.061	0.011	0.046	79	1635

ACTATCGCATATA	0.024	0.002	0.02	64	3064
GCTGGTGCATGTA	0.122	0.006	0.113	86	675
GGCGTCATAGACA	0.072	0.015	0.053	100	1780
ACCAGCGTTAATG	0.025	0.002	0.025	49	1886
CCCGTCATAGAGA	0.028	0.002	0.031	73	2296
ACCAGTATTAATG	0.026	0.001	0.027	96	3480
GCCAGTGCTAATA	0.041	0.002	0.039	70	1742
ACCGTCATTGATA	0.022	0.002	0.024	73	2995
ACTGGTACAGATA	0.025	0.001	0.025	66	2588
AGCATTACATGTA	0.02	0.003	0.021	62	2867
GCCAGTGTAAGGA	0.05	0.007	0.049	59	1148
ACTAGTGCATGCG	0.023	0.003	0.025	38	1505
GGCGGTGCAAATA	0.056	0.012	0.05	73	1383
CCCAGCATAAATA	0.028	0.003	0.024	127	5152
ACCGTTGTTTAGA	0.024	0.006	0.03	45	1463
ACCATCACTAAGA	0.023	0.004	0.018	83	4497
GCTATCGCATATA	0.05	0.009	0.041	62	1435
CGCAGTACAGGCG	0.023	0.007	0.021	27	1286
CCCGGCATAGGTG	0.03	0.006	0.03	35	1147
CGTGTTGCAAATG	0.026	0.006	0.026	30	1111
AGTAGCGCATACG	0.024	0.003	0.027	29	1027
CCCATTACAAGCG	0.025	0.005	0.022	64	2842
GGCGTCACATGTA	0.064	0.007	0.062	83	1254
ACTGGTACATGTG	0.028	0.001	0.027	35	1248
CGTGTCACAGATA	0.022	0.001	0.021	29	1343
AGCATTATAAATG	0.022	0.003	0.018	75	4177
ACTATTATAAGGG	0.027	0	0.026	56	2061
CCCGGCATATGCG	0.025	0.003	0.023	37	1581
AGCATTACTGGCG	0.022	0.002	0.02	36	1737
GCTGTTACTGATA	0.065	0.004	0.071	86	1125
ACCAGCACTGGTG	0.02	0.003	0.019	32	1671
ACTATTGTTAAGA	0.025	0.003	0.022	60	2646
ACCATTGCTGACA	0.024	0.003	0.022	82	3611
CCCGGCGCAAGCG	0.024	0.002	0.021	32	1482
CCCAGCGCATAGA	0.021	0.003	0.019	44	2304
ACTGGTATAGATG	0.026	0.004	0.023	31	1343
CGCATTACTTGTA	0.028	0.003	0.023	40	1704
CCCAGTACAGAGA	0.025	0.004	0.023	63	2715
GCTATTACTTGTA	0.058	0.018	0.048	68	1352
CGTGTCACTAATG	0.02	0.002	0.019	21	1091

AGTAGCGCAGAGA	0.028	0.007	0.021	20	916
CGCGTCACTAATG	0.022	0.002	0.024	38	1544
CGCGGCATAAACG	0.021	0.004	0.026	38	1423
AGCAGTGTTTAGG	0.015	0.004	0.012	12	1003
ACCGGTGCTGATA	0.024	0.003	0.02	41	2037
AGTGGTGCAGGGG	0.024	0.006	0.017	6	352
AGTAGCGTAAGCG	0.02	0.004	0.015	14	892
ACTATTATAGGTG	0.033	0.003	0.029	52	1759
GCTAGTGCAAATA	0.056	0.01	0.042	83	1891
CGCAGTGTTAACG	0.029	0.004	0.029	43	1425
GCCATCGTTGGGG	0.062	0.021	0.075	48	593
ACTGGCACAGACA	0.026	0.004	0.022	53	2333
AGCGGTGTTAATG	0.026	0.008	0.017	22	1239
GGCAGCACTTATA	0.047	0.005	0.05	85	1629
CCTGTTGCAAGCA	0.023	0.001	0.023	45	1874
ACCAGCACTTGGA	0.024	0.005	0.03	67	2170
ACCAGCACTAAGA	0.021	0.001	0.02	83	4050
GCCAGTATTAACA	0.048	0.004	0.053	196	3514
GCTGGTGCAAATA	0.117	0.017	0.111	139	1110
CGCGGCGCTGATG	0.022	0.004	0.026	22	821
ACCAGTGCAGGCG	0.02	0.003	0.021	38	1815
ACCGGCACAAGCG	0.022	0.001	0.021	45	2073
GGCGTCACTAGCG	0.061	0.011	0.05	46	873
AGCATCGCAGATA	0.024	0.002	0.026	66	2495
GGCATTACTGAGG	0.065	0.004	0.061	60	927
CCCAGCATTAACA	0.024	0.003	0.022	100	4446
GGCGGCGTAGGCA	0.199	0.031	0.196	154	632
CCCATTACTGGTG	0.023	0.003	0.019	31	1639
CGTGTCATATACA	0.023	0.006	0.016	31	1860
GCTGTTGCTGGGG	0.051	0.021	0.021	7	328
ACCAGTGTATACA	0.023	0.004	0.023	80	3379
ACCATCGTTGACG	0.025	0.004	0.02	44	2196
AGTGTCGTAAGGG	0.032	0.008	0.021	13	594
AGCGTTATTGATA	0.021	0.003	0.019	45	2378
AGTAGTACAAATA	0.024	0.003	0.02	57	2809
ACCGTTATATACA	0.025	0.003	0.022	92	4004
CCCGTTACAAATA	0.025	0.005	0.02	87	4262
AGTATTGCTGGTA	0.021	0.004	0.016	17	1072
GCTAGTGCTGGCG	0.056	0.013	0.064	60	879
GGTGTCACAAAGA	0.085	0.007	0.079	84	981

ACTGGCGCATAGA	0.03	0.007	0.029	24	790
AGCATCATAAGCA	0.022	0.005	0.016	64	3892
ACCAGTGTTTACA	0.022	0.002	0.019	54	2720
ACTATTACTAACA	0.023	0.002	0.022	118	5323
GCTAGTATTAATG	0.051	0.002	0.048	78	1539
CGTGTCGTATATA	0.025	0.002	0.026	31	1146
ACCGGCGCTGAGA	0.023	0.004	0.027	28	998
GCCATCATTAATG	0.059	0.008	0.05	133	2518
ACCATTACTGGGA	0.028	0.004	0.034	69	1978
CCTATTGTTGGCA	0.029	0.007	0.02	34	1699
CCTATCGTTGGCA	0.021	0.003	0.02	31	1495
GCCATCGCATACG	0.047	0.006	0.046	68	1405
AGCGGCGCTGGCA	0.016	0.004	0.022	23	1042
AGCAGTATAGACA	0.019	0.002	0.016	61	3746
GCTATCACAAGTA	0.055	0.004	0.05	88	1688
CCTGGCACTAAGA	0.029	0.009	0.025	40	1547
AGCATTGTAAGTG	0.023	0.004	0.023	44	1839
GGTAGCACATATG	0.075	0.009	0.074	62	780
CCCATTGTAGGCA	0.025	0	0.025	57	2258
AGTGTTATTTGTG	0.019	0.002	0.018	15	825
ACTATTACTTGTG	0.026	0.002	0.029	56	1887
CCCAGTATAAGGA	0.025	0.003	0.023	62	2652
ACTATCGTTGAGA	0.025	0.005	0.031	50	1584
AGCAGCGTAGGGA	0.023	0.001	0.023	33	1406
AGCAGCATTTACA	0.022	0.002	0.024	78	3177
AGTATCGTTGATG	0.022	0.01	0.019	22	1125
GCCGTCATATACG	0.055	0.007	0.056	102	1730
ACCGGCGCAGATA	0.026	0.004	0.022	35	1521
CCCGGCACTAGGA	0.026	0.008	0.019	28	1447
AGCAGTATTAGTA	0.021	0.002	0.021	59	2712
CCTAGTACTAATA	0.025	0.003	0.021	66	3074
AGTGGTACTGACA	0.021	0.005	0.021	29	1355
CCTAGTGTATACG	0.025	0.003	0.027	50	1795
ACCATCGTAAGGA	0.026	0.003	0.024	52	2090
CGTATCGTAAATA	0.026	0.002	0.025	64	2535
AGTATTATTTAGG	0.026	0.007	0.025	34	1318
CCTATTACATAGG	0.028	0.002	0.028	48	1649
CGTAGCATTAAGA	0.02	0.002	0.019	28	1441
AGTATCATTGACA	0.023	0.002	0.025	54	2144
CCCGGCGCTAGCA	0.022	0.005	0.015	26	1693

CCCGGTACAAGGA	0.023	0.004	0.028	54	1909
CGCATTGTTTACG	0.034	0.001	0.033	52	1523
GCCATTGTAAGCA	0.049	0.006	0.041	91	2127
GGTAGCACTTACG	0.082	0.003	0.078	53	624
ACCGGTGTTTGTA	0.023	0.004	0.02	27	1312
ACTGGCACAAACA	0.024	0.003	0.026	83	3066
ACCATTACTAACA	0.023	0.001	0.022	166	7262
GCTATTGTAAAGA	0.053	0.009	0.042	84	1939
AGTATCATATATA	0.023	0.004	0.018	52	2780
AGCAGTGCTTGCG	0.02	0.001	0.019	23	1168
CCCGTTGTTAAGG	0.021	0.004	0.021	27	1244
CGTGTTGTATATA	0.027	0.004	0.022	28	1224
CCTGGTACTAGTG	0.025	0.004	0.021	20	915
ACTATCGTAGGCG	0.026	0.002	0.025	38	1510
CCTGGTACAAACG	0.027	0.007	0.026	47	1759
GCCATCACATATG	0.054	0.001	0.053	118	2095
CCCATCGTAGGCA	0.02	0.002	0.023	56	2389
CGTATCATAAGTG	0.025	0.001	0.023	36	1504
CCCAGCGCTGGCA	0.024	0.001	0.023	51	2187
CGCATCACTAGTG	0.023	0.003	0.026	41	1560
GCCGTCATTTGCG	0.061	0.006	0.068	73	1001
CGCGGTATTGACA	0.021	0.003	0.023	42	1805
GCCATTGTTAAGA	0.05	0.005	0.044	81	1746
CCCATTGCTAGGA	0.027	0.004	0.024	41	1697
ACCGTTGTAGATA	0.025	0.003	0.023	53	2270
ACTATTATTAAGA	0.025	0.004	0.027	103	3661
CCTGTTATATATA	0.031	0.005	0.025	70	2720
CCCGGCACTGGGG	0.039	0.004	0.044	35	753
GCTATCGCTTGTA	0.062	0.006	0.059	53	847
AGCGTTATTTGGG	0.021	0.008	0.026	25	923
GGCGGTATAAAGA	0.07	0.016	0.062	99	1510
CCTAGCATTGAGG	0.024	0.001	0.026	31	1151
AGCATCACATGTG	0.019	0.006	0.018	36	1995
GCCATTGTAAGGA	0.046	0.001	0.045	71	1513
GGTGTTGCATGCG	0.069	0.016	0.052	27	491
AGTGTTACATATG	0.026	0.003	0.023	29	1251
ACCAGTGTTAAGA	0.024	0.004	0.024	60	2443
ACTAGTGCTGACG	0.024	0.002	0.026	43	1611
AGCATTACATACA	0.021	0.002	0.018	81	4310
CCTAGCGTTTAGA	0.024	0.007	0.017	24	1384

ACCAGCACTGAGG	0.025	0.004	0.026	51	1929
AGCGTCGCTGGTA	0.02	0.002	0.019	21	1065
CGTATCGTTAAGG	0.03	0.008	0.021	21	971
CGCATCGTTAAGA	0.021	0.003	0.019	38	1964
ACTAGTGCTTAGA	0.025	0.007	0.015	29	1930
CGCGTCACTGGCG	0.026	0.007	0.035	42	1154
CCCAGCGCAAGGA	0.028	0.006	0.022	51	2261
ACTATCACATATA	0.026	0.002	0.025	114	4432
GGTAGTATAGACA	0.066	0.01	0.053	78	1407
ACCAGCGCATGCG	0.022	0.004	0.018	32	1758
GGTAGTGTATATA	0.064	0.006	0.057	59	982
ACCAGTACTTGGA	0.023	0.003	0.019	48	2441
ACCAGTGTTAATA	0.021	0.002	0.02	70	3371
GCCATCGCAAACG	0.048	0.004	0.052	100	1807
GGTGTTGCTTATG	0.084	0.011	0.088	44	456
GCCATTACAAGCG	0.056	0.006	0.053	108	1932
CCCATCGTTAGCA	0.026	0.001	0.028	70	2446
GGTGTTATTTAGA	0.079	0.005	0.074	61	768
ACCATCGTTGGCA	0.022	0.002	0.02	44	2110
CCTATCACAGGCA	0.025	0.005	0.026	67	2531
CGCGGCACTTATG	0.024	0.007	0.015	14	910
ACTGTCACTAGGA	0.022	0.004	0.017	24	1407
CGTATCGCAAGCA	0.026	0.001	0.027	41	1473
GCTAGTATATGTA	0.06	0.004	0.053	87	1545
ACTATCGCTAACA	0.027	0.005	0.02	71	3397
ACTGTCGTAAAGA	0.029	0.005	0.022	37	1659
ACTAGTACATGCA	0.025	0.005	0.019	61	3198
GCTGGCGCTAATA	0.625	0.025	0.599	942	631
CCTGGTACAAGGG	0.031	0.005	0.037	32	833
CGTAGCATAAATG	0.023	0.001	0.023	37	1545
AGTGGCACTTACA	0.021	0.001	0.021	25	1159
ACTATTACTTGGA	0.023	0.002	0.02	42	2106
ACTGTTGCTTGCA	0.023	0.004	0.019	29	1467
GCCATCGCTAATG	0.044	0.003	0.043	59	1298
CGTGTCATTAACG	0.026	0.005	0.023	31	1326
GCTAGTACAAGCA	0.057	0.008	0.048	82	1629
GCCATCATTAATA	0.057	0.004	0.052	198	3588
ACTATTGCTTAGA	0.028	0.001	0.027	60	2177
CGCGTTATTGAGA	0.021	0.005	0.021	29	1341
CCTGTCATTGGCA	0.024	0.003	0.02	29	1405

ACCATTGTAAATG	0.025	0.002	0.024	81	3243
GCTAGCACTGGGA	0.068	0.018	0.053	38	679
GCTAGTGTAGACA	0.057	0.007	0.055	96	1656
GCCATTATAAGTG	0.056	0.002	0.059	134	2156
CGCAGTACATATA	0.024	0	0.024	58	2402
ACTGTTATATACG	0.028	0.004	0.031	68	2096
GGCATCGTATAGA	0.102	0.006	0.094	137	1326
ACCATTGCAAATA	0.024	0.001	0.024	134	5359
GCCATCATTTGCA	0.058	0.005	0.052	120	2192
AGTAGTGTTGACG	0.021	0.002	0.02	19	932
CGCAGTACTTACA	0.024	0.002	0.025	58	2258
ACCATCACAAACG	0.027	0.001	0.029	159	5272
ACTAGCGCAAAGG	0.025	0.005	0.026	38	1420
CGCGTCGTTAACA	0.026	0.004	0.02	41	2051
AGTAGTATAGACA	0.023	0.002	0.021	52	2406
ACTAGTACATATA	0.022	0.001	0.022	103	4489
AGTGTCACTGGTG	0.023	0.008	0.012	7	590
ACCATTATTGGCA	0.022	0.001	0.023	74	3195
CCTAGTATAAGTA	0.026	0.005	0.023	63	2673
AGCGTTATTTACG	0.023	0.003	0.019	31	1619
GGTGTTACAAGCG	0.084	0.014	0.065	45	644
ACTGGTACATATG	0.026	0.002	0.029	44	1492
GGTGGCATTAGCA	0.486	0.063	0.427	422	566
ACTATTACAGGCG	0.023	0.002	0.023	51	2181
ACTATTGTTAGCG	0.027	0.001	0.028	46	1577
ACCGTCACTAGTA	0.024	0.002	0.025	73	2889
CGTAGTATTGAGA	0.023	0.003	0.022	22	959
GCTAGTATAAGTA	0.066	0.002	0.063	129	1930
CGCGTTGTAGAGA	0.026	0.003	0.022	27	1210
CGCATTATAAACA	0.027	0	0.027	131	4734
GCTGTTGTTAGGA	0.055	0.003	0.052	35	639
AGCAGCATATGCA	0.021	0.004	0.027	75	2710
CCCAGTGTAAACA	0.026	0.003	0.023	87	3669
GCCATTGCATGCA	0.051	0.005	0.057	89	1485
ACTGGTACAAGTA	0.022	0.001	0.023	55	2294
CGTGGCACTTACG	0.027	0.012	0.023	17	727
CCTGGTGCAAGTA	0.019	0.005	0.016	24	1499
ACCATCGTAAAGA	0.025	0.001	0.024	80	3226
ACCGTCGTAAATG	0.027	0.004	0.028	52	1798
CGTATCACAGGTG	0.018	0.001	0.017	14	821

CCTGTCACTTAGA	0.024	0.008	0.02	29	1389
GGCAGCGTTAAGG	0.056	0.006	0.047	39	788
CCCAGCACAGACA	0.026	0.001	0.026	105	3874
GCTATTGCAGAGA	0.054	0.01	0.057	72	1181
ACTGTCACTTGCA	0.026	0.002	0.023	41	1723
GCTATCGCAGACA	0.049	0.004	0.052	77	1391
CCCATTACAAATA	0.027	0.002	0.025	158	6106
CCTAGCGCATATA	0.023	0.002	0.022	55	2410
CGTATCGTTAACA	0.025	0.002	0.026	54	1996
ACCAGCATATACA	0.022	0.001	0.024	124	5092
CCCATCGCTGAGA	0.021	0.002	0.019	41	2095
ACCAGTATAAACG	0.025	0.002	0.024	113	4513
CCCAGCGTTAGCG	0.02	0.002	0.017	29	1642
CCTAGCGCAAAGG	0.024	0.008	0.022	27	1219
CCCGGCGTAAGCA	0.025	0.002	0.027	61	2225
CGCAGTATTAGGG	0.022	0.002	0.02	19	934
ACTGTTGTAGGTG	0.031	0.008	0.02	18	894
CCTAGCACATACG	0.025	0.003	0.022	42	1891
CCCGGTGCAAGGA	0.02	0.002	0.022	34	1516
GCTGTCACAAATG	0.061	0.004	0.055	70	1194
CCTATTACAGGCG	0.027	0.005	0.021	37	1742
GCCATTATATACA	0.05	0.003	0.051	211	3917
GGCGGTATAGGCA	0.07	0.006	0.063	68	1008
AGTAGCGTATGCA	0.02	0.005	0.014	16	1154
CCCATTATAAATA	0.025	0.002	0.022	144	6471
GGTGTTACATGTG	0.083	0.007	0.074	51	637
CGTGTCGTATGTG	0.03	0.009	0.018	11	616
CGCAGCACTTGCG	0.019	0.002	0.018	20	1089
ACCGTCATTGGCA	0.023	0.003	0.028	66	2298
ACTAGCATTAGTG	0.028	0.003	0.023	35	1483
GCCATTGTTAATA	0.046	0.005	0.044	112	2456
CGCATCATTAATA	0.024	0.001	0.025	92	3633
CCTGGTGTTGGGG	0.02	0.007	0.022	10	452
AGCGGTGCAAACA	0.019	0.002	0.017	40	2360
GGTATTATATATG	0.107	0.005	0.101	150	1341
ACCGTCATATGGA	0.027	0.006	0.027	55	1988
GCCGGCGCTAGTG	0.761	0.042	0.774	661	193
CCTGGTATAGGGA	0.045	0.008	0.035	33	915
CCCATCGCTGGCG	0.027	0.002	0.027	41	1504
AGCGGCGCTAATA	0.025	0.005	0.022	36	1592

GGTGGTGCTGGGA	0.071	0.035	0.038	13	330
ACTAGTATAAGCG	0.024	0.003	0.019	41	2081
ACTATTACTAGTA	0.022	0.002	0.019	62	3152
CCTAGCACTGACA	0.033	0.007	0.024	53	2187
CGCATCGTAGATA	0.025	0.003	0.026	59	2173
GGCATCACAGGCA	0.066	0.015	0.05	82	1543
GCCATCACATACG	0.054	0.005	0.053	126	2244
CCCGTCACTAACA	0.029	0.004	0.034	124	3570
GGCGGTGCTAATA	0.055	0.004	0.049	57	1117
ACCAGCGTATGGA	0.026	0.002	0.028	42	1485
AGCATCACATGGG	0.023	0.002	0.02	31	1486
CCCGGTGTTTATG	0.024	0.007	0.03	36	1155
CCCATCGCAAGTA	0.025	0.004	0.023	68	2930
CGCGTTGTTAGCA	0.026	0.005	0.019	24	1219
GCCATTGTTTGCG	0.053	0.005	0.049	51	992
ACCAGCGCTTGCG	0.019	0.006	0.014	21	1514
CCCGGTATTAAGA	0.027	0.003	0.031	74	2301
GCTAGTGTTGATA	0.06	0.007	0.066	85	1210
ACTGTCATTGACA	0.025	0.001	0.027	65	2341
GCCAGCATAAGTG	0.049	0.006	0.042	76	1726
CGTAGCGCAAACG	0.027	0.003	0.024	29	1170
CCTAGTGTATGTA	0.023	0.003	0.027	50	1788
GCCAGTACTAATA	0.05	0.004	0.046	130	2696
CGTGTTGCATGTG	0.036	0.003	0.036	22	586
CCTAGCATTTGCG	0.028	0.007	0.02	25	1212
GGCGTCATTTGGG	0.07	0.022	0.039	21	517
GCCATCATAGGGG	0.099	0.008	0.088	78	807
CCTAGTATTAATG	0.026	0.003	0.026	56	2096
CGTGTTGCTAACA	0.022	0.003	0.02	28	1377
ACTAGCGCATAGG	0.03	0.005	0.024	26	1073
GGCATTATTTAGG	0.068	0.005	0.073	87	1101
AGCATCATTGGTA	0.025	0.002	0.022	49	2178
AGTAGCATTTATA	0.024	0.004	0.023	43	1866
GCTATTATATGCG	0.069	0.01	0.063	91	1357
ACTGTCGCAGAGA	0.03	0.003	0.025	37	1435
CGCATCACAGACA	0.026	0.004	0.02	65	3110
ACTATCGCAAACA	0.027	0.006	0.024	100	4097
GCCATCACTAATG	0.051	0.005	0.044	111	2391
ACTGTCGTTAATA	0.025	0.009	0.015	31	2000
GCCGGTACTTATA	0.058	0.003	0.056	94	1572

ACCGTTGCATACG	0.021	0.004	0.02	45	2240
ACTATTGTATGTA	0.028	0.007	0.02	51	2454
GCTATTACTTGCG	0.081	0.013	0.084	83	900
CCCGTTATATATA	0.026	0.004	0.02	71	3411
CCCATTATAGACG	0.029	0.001	0.029	96	3272
ACCATTGTATACG	0.029	0.001	0.031	86	2714
GGTAGCATTGAGG	0.06	0.011	0.052	30	545
CGTGTTACAAAGA	0.023	0.004	0.017	26	1491
GCTGTTGTATAGG	0.065	0.006	0.057	38	625
CCTATTACTAAGA	0.024	0.003	0.02	59	2915
GGTGTCGCTGGCA	0.083	0.017	0.062	33	500
ACCATCACTTGTA	0.02	0.002	0.018	62	3454
GCCGTTATAAATG	0.055	0.004	0.049	115	2240
GGCGGCACAAACA	0.138	0.022	0.134	293	1900
CGTGGCGTAGGTA	0.022	0.007	0.019	12	605
ACCATCATATACG	0.026	0.003	0.021	89	4094
GCCATCACTGGGA	0.05	0.011	0.038	45	1135
GCTAGTATAAAGA	0.059	0.005	0.054	121	2138
GGCGGCGCATGGG	0.191	0.037	0.202	92	363
ACTGTCGTTGGCG	0.023	0.004	0.022	17	749
CGCAGCACATGCG	0.028	0.003	0.025	35	1363
CCCATCACTAGTA	0.028	0.004	0.025	74	2907
GCTGTCGCTGATA	0.051	0.008	0.059	46	734
CCCAGTGTAAGGA	0.029	0.007	0.028	52	1774
GCTGGTATTAATG	0.446	0.049	0.49	599	623
CGCATCACAGACG	0.027	0.005	0.019	42	2113
CCCAGCGCTTGCG	0.028	0.007	0.029	40	1321
ACTGGTATTGATA	0.026	0.006	0.025	50	1963
GCCAGCGTAAATA	0.052	0.001	0.051	126	2321
CGCATTGTTTGCA	0.025	0.002	0.026	38	1432
ACTAGCATATAGA	0.027	0.008	0.02	51	2465
GGTGGCGCAAACA	0.156	0.027	0.14	150	925
CGCATCATATACA	0.027	0.003	0.025	95	3774
AGCGTCATAAAGG	0.025	0.007	0.021	37	1725
ACTAGTGCAGACA	0.026	0.002	0.024	77	3142
CCCGTCGCTTGGG	0.022	0.005	0.017	12	714
CCTAGCGCTTACA	0.021	0.001	0.021	46	2138
GCCGGTACAAAGA	0.061	0.002	0.063	114	1693
CCTGGCATAAATA	0.025	0.001	0.024	67	2717
ACTGGTACTGACG	0.028	0.006	0.019	27	1362

CCCGTCACTAATA	0.028	0.003	0.023	77	3206
AGCGTTGCTAGTG	0.023	0.003	0.024	22	907
ACTAGCGCTGACG	0.023	0.002	0.022	31	1381
ACCAGTGTTGACA	0.023	0.002	0.02	56	2696
ACCGGCATTGACA	0.025	0.005	0.031	89	2751
ACCGGTGTTGGGG	0.029	0.004	0.031	19	587
CCTATCGCTTGGG	0.02	0.004	0.021	18	827
CCCGGCGCAAGCA	0.02	0.004	0.016	36	2227
GCCGTCATTTGTA	0.049	0.001	0.05	71	1347
ACTGTTACAAATG	0.023	0.005	0.02	51	2471
GGTGTCGTAGACA	0.088	0.023	0.067	57	794
CCTATCGTAAATG	0.026	0.002	0.026	68	2521
CCCAGTACAAAGA	0.027	0.003	0.024	96	3881
CGCGGTATATGCA	0.024	0.001	0.026	40	1512
ACCATTACATATG	0.019	0.001	0.021	93	4420
AGTAGCGTATAGG	0.03	0.004	0.032	27	827
CCCAGCACAAGTA	0.027	0.001	0.026	89	3365
CCTATTATTTAGA	0.027	0.003	0.023	59	2453
CGCGGTATAAGCG	0.019	0.002	0.022	27	1217
ACTATCACTGACG	0.028	0	0.028	60	2052
CGCATTATAAGTA	0.023	0.001	0.024	77	3096
CCTGGTATAGATA	0.027	0.002	0.025	54	2093
ACTAGTACTAATG	0.023	0.002	0.027	76	2783
ACCAGCACTAGCG	0.025	0.002	0.026	67	2558
CCCATTACTTGTG	0.019	0.002	0.018	32	1769
CCCATTATTTACA	0.027	0.001	0.026	111	4078
ACCGTTGCATATA	0.024	0.001	0.023	65	2809
CCCGTCGCAGACG	0.027	0.004	0.024	47	1878
ACTATCGTATACG	0.032	0.004	0.028	56	1935
ACTGGTGTTAATA	0.024	0.005	0.024	44	1797
CCTGTCGCAAATG	0.027	0.006	0.019	29	1508
ACTAGTATTAACA	0.025	0.003	0.022	89	4044
CGCATCGCAGGCA	0.024	0.005	0.018	31	1708
GCCAGTGTATGTA	0.053	0.005	0.047	71	1429
AGCGTCATAGACA	0.021	0.002	0.023	68	2876
GGTAGTGTTGGGA	0.067	0.017	0.045	20	426
CGTAGTATAAGGG	0.032	0.012	0.017	13	756
ACTAGTGTTTGGA	0.024	0.004	0.025	30	1166
GGCGGCGTATGCA	0.203	0.04	0.167	153	763
CCTATTATTAACA	0.026	0.003	0.021	95	4363

ACTAGCGTTTAGA	0.025	0.001	0.025	33	1312
ACTATCATAAAGG	0.03	0.003	0.027	73	2609
CCCATTACTGGGG	0.03	0.006	0.03	38	1212
CCCATCACATAGG	0.024	0.002	0.021	43	2012
CGCAGTACTTATA	0.02	0.002	0.017	35	1967
AGTATTACATGCA	0.023	0.003	0.019	37	1867
CCTGGCACTAATA	0.022	0.003	0.021	45	2095
CGTGGTATTTACA	0.028	0.001	0.03	35	1135
CGCATTACAGGGG	0.034	0.003	0.03	26	850
CCCATTACATATA	0.027	0.001	0.028	128	4477
AGCATCATTGGCG	0.023	0.006	0.021	42	1954
GCCAGCGTTAGTG	0.066	0.001	0.065	62	897
ACCATCATTAGTA	0.023	0.003	0.027	103	3692
ACTATTATTAACA	0.027	0.001	0.025	129	5013
CCTGTCGTTGGGG	0.014	0.004	0.02	11	550
CGTATCGCAAACA	0.022	0.001	0.021	49	2288
ACTGTCGTAGATA	0.027	0.003	0.029	48	1612
ACTATCGTATAGA	0.03	0.003	0.027	54	1944
CCTAGTATTAGCA	0.031	0.007	0.022	49	2137
AGCGGTGTAAGGA	0.021	0.007	0.025	30	1172
ACCATCATTTAGA	0.025	0.002	0.023	77	3273
CCCATTGTAAAGG	0.02	0.003	0.018	42	2318
AGTGTTACTAATG	0.037	0.007	0.028	38	1316
CCTATCGCTTGCA	0.024	0.002	0.021	40	1837
CCCATTATTAACA	0.024	0.002	0.021	118	5430
AGCAGTGCTGAGA	0.022	0.009	0.016	23	1395
GCTGTCGTTAACA	0.053	0.011	0.042	55	1267
ACTGTTGCTAACA	0.024	0.005	0.018	48	2589
GCTAGTGCTTAGG	0.049	0.002	0.047	26	533
AGTATCATTAGGG	0.024	0.006	0.027	26	944
ACCAGTATTAGCA	0.02	0.002	0.018	63	3536
GCCGGTGTAGGCA	0.174	0.032	0.163	182	937
CCTAGTGTTGACA	0.021	0.004	0.016	34	2135
GCCAGTGCTAATG	0.044	0.001	0.043	51	1148
CGCGTTATAAATA	0.02	0.002	0.019	66	3319
AGCGGCACTTGCG	0.022	0.004	0.026	20	764
ACTAGTATTTGCA	0.024	0.002	0.023	49	2108
AGCATCACAAAGA	0.021	0	0.02	76	3634
ACCAGCACATACG	0.023	0.003	0.019	72	3761
CGCGTCATATATA	0.023	0.002	0.025	57	2269

ACTGTTATTAGGG	0.029	0.006	0.034	34	973
ACTGTTATATGGG	0.03	0.007	0.02	20	966
GCCAGTGCAAATA	0.041	0.002	0.04	87	2109
AGCGGTATTTGTA	0.022	0.003	0.018	27	1514
ACTGTCGCAAAGA	0.022	0.003	0.02	39	1940
AGCGGCACTTATG	0.018	0.002	0.016	15	927
CCCGGTGCAGATG	0.03	0.003	0.032	39	1162
ACCGTCGTTTACG	0.023	0.003	0.025	34	1337
CGCGTTACTTATG	0.029	0.006	0.035	43	1172
ACTGGCACTTGTA	0.024	0.003	0.028	40	1364
AGCGGCACATGCG	0.02	0.004	0.018	18	1006
ACCATTACAAATA	0.021	0.002	0.02	174	8567
ACCAGCATTTACG	0.022	0.001	0.023	64	2665
AGTGGCGTAGGGG	0.012	0.009	0.018	5	266
CCCAGCATAAGCA	0.025	0.004	0.021	79	3720
ACTATTATTAGTA	0.027	0.002	0.03	98	3198
GCCGTCGCTAACA	0.053	0.005	0.047	67	1372
CCTGTTGTAAGTA	0.024	0.003	0.02	36	1763
AGTATCGCAAGCG	0.027	0.001	0.026	29	1078
AGCGTTGTTTGCG	0.022	0.005	0.017	16	902
AGTATCACAGACA	0.023	0.005	0.019	39	2038
GGCGTTATAGACG	0.054	0.005	0.05	64	1207
CGTATTACTTGTA	0.027	0.007	0.025	32	1251
GCCATCGCTAGTA	0.045	0.006	0.041	53	1227
GGTAGCATTAGCG	0.071	0.016	0.054	33	579
CCTGGCGTAGGTG	0.02	0.006	0.018	12	671
ACCGTTACATATA	0.021	0.002	0.018	85	4668
CGTATCGCAGACA	0.027	0.002	0.027	45	1622
CGTATTGTAAACG	0.029	0.004	0.024	42	1704
GGCGGTATAGACA	0.076	0.016	0.07	117	1550
ACCATTATTGACA	0.024	0.001	0.023	116	4885
CCCGTTATTGGTG	0.027	0.002	0.024	33	1325
CCCGTCGCAGATG	0.024	0.003	0.025	37	1465
ACCGTTACATATG	0.026	0.005	0.027	83	2945
AGCATCACAGACG	0.024	0.003	0.023	63	2630
CCCATCGCAAAGG	0.029	0.003	0.033	72	2107
GCCATCACTAACG	0.05	0.002	0.047	123	2492
ACCATTATATGCA	0.025	0.003	0.026	113	4225
GCTAGTGTAAACG	0.048	0.003	0.047	78	1599
CGCGTTGTTAATA	0.021	0.003	0.019	35	1851

ACTATCGTATATG	0.029	0	0.028	52	1790
GCCATTGCATGTG	0.05	0.009	0.043	45	996
CCCGTTATTAACA	0.026	0.003	0.024	94	3864
GGCGTTGCATATG	0.067	0.004	0.068	60	828
GCCGTTATTAGCG	0.058	0.01	0.067	81	1136
CCTGTTATTGATG	0.029	0.008	0.026	37	1412
CCTGGTACTAGTA	0.028	0.005	0.034	48	1375
ACTGTCGCAGGCG	0.022	0.006	0.021	21	958
AGTAGCGCATGCA	0.026	0.005	0.02	21	1004
ACTATCGCATGCA	0.023	0.003	0.021	51	2358
CGTATTGCATGCA	0.025	0.004	0.025	33	1281
ACCATTACTGACA	0.021	0.002	0.02	108	5417
AGTATCACATAGG	0.021	0.005	0.027	28	1022
CCTGTTGTTTGGG	0.015	0.001	0.017	12	698
ACTGTCATAAGCA	0.028	0.006	0.024	58	2377
CCTAGCGTATGCA	0.022	0.001	0.023	40	1717
GGCAGCGTATACG	0.066	0.008	0.056	70	1186
CCTGGTGTTTGCG	0.021	0.005	0.027	20	708
ACTAGCATATATG	0.028	0.005	0.025	57	2211
CCCAGTATTTGCA	0.026	0.002	0.023	59	2495
CGTGTCGTTTGGG	0.037	0.009	0.034	14	401
CCTGGCATTGATG	0.023	0.002	0.021	23	1097
CGTGTCATTGGCA	0.028	0.005	0.034	32	899
AGTAGCACTAGTG	0.022	0.004	0.018	16	870
CGTATCACTAATA	0.025	0.006	0.018	39	2141
ACTAGTACTGGGA	0.029	0.001	0.028	42	1432
CCTGGCGTATACA	0.023	0.003	0.027	52	1880
CCCGGTACAGACG	0.029	0.003	0.031	64	1978
CGTAGCACATACA	0.024	0.006	0.016	29	1794
AGCGTTGTTAACG	0.023	0.005	0.02	32	1604
CGTAGTGCTAGCG	0.027	0.008	0.016	9	565
AGTGGTACTAATA	0.025	0.003	0.026	41	1566
GCCGTCGCTAGTG	0.052	0.015	0.031	20	632
GCCATCGTAGATA	0.047	0.002	0.048	97	1921
ACTATCATTAGGA	0.027	0.003	0.031	65	2000
GCCATTACAAAGA	0.054	0.006	0.054	165	2879
CCCGGCGTTTAGG	0.024	0.005	0.023	21	874
AGTATCGCATAGA	0.022	0.006	0.013	17	1254
GGTGTCACAGGGA	0.074	0.023	0.05	25	474
AGTATTGCATGTG	0.021	0.006	0.022	23	1034

ACTGGCGTTTAGA	0.022	0.004	0.023	20	834
CGCGGTACAGACG	0.026	0.002	0.025	34	1315
ACTATTACTAAGG	0.02	0.001	0.02	50	2437
ACTGGTACATACA	0.026	0.001	0.028	86	2997
ACCAGCACAGAGG	0.026	0.002	0.024	47	1926
AGTAGCGTAGATG	0.022	0.003	0.023	20	865
CCCGTTATTAGCA	0.019	0.005	0.018	43	2408
CGCAGTATTTGCA	0.023	0.005	0.028	49	1720
ACCATTATTTGGG	0.022	0.005	0.022	39	1746
GCCGTTGCAGACA	0.039	0.008	0.028	41	1409
CCCGGCATTAAGA	0.025	0.003	0.028	64	2205
ACCGGTACTAGCG	0.024	0.003	0.024	49	1965
CGCATCATAGACA	0.025	0.007	0.017	59	3413
ACCGGTACAAGCG	0.018	0.002	0.016	39	2432
GGTGTCACTAACA	0.08	0.011	0.065	82	1171
CGCATTACTGACA	0.028	0.001	0.028	68	2395
AGTGTCGCAAGTA	0.021	0.002	0.022	25	1137
ACTGGTGCTTGTG	0.025	0.001	0.023	17	722
GCTATCATAAAGA	0.061	0.003	0.057	135	2222
GCCGGTATTTAGA	0.077	0.007	0.083	113	1241
AGTATTATAGGGG	0.025	0.005	0.031	38	1191
CGTGTTATAGATG	0.031	0.007	0.021	25	1180
AGTGTTGCAGATG	0.029	0.002	0.028	23	799
ACCAGCGCATACG	0.024	0.005	0.018	43	2375
CGTATCGCAAAGG	0.025	0.006	0.017	19	1110
AGTATTATATGGG	0.023	0.001	0.023	27	1158
CGTGTTACAAGCA	0.028	0.003	0.032	49	1491
ACTGTTATATGTG	0.026	0.001	0.025	37	1419
AGCGGTACAAGGG	0.015	0.003	0.011	11	1013
GCCATCGTAGACG	0.051	0.005	0.046	79	1635
CGTATTATTGGCG	0.023	0.008	0.02	25	1242
CGCGGCGCTAGTA	0.026	0.011	0.011	11	992
AGCAGTGTAGAGG	0.022	0.002	0.024	29	1186
GGCATCACATATA	0.07	0.01	0.062	142	2147
ACCGTTGCTGAGA	0.025	0.002	0.027	48	1735
GGTGTTACATAGG	0.08	0.01	0.075	48	590
ACTGTCATTTATA	0.023	0.003	0.021	47	2206
GCCGGCACTAGCA	0.11	0.007	0.106	162	1362
AGCGTTACAGATG	0.024	0.003	0.022	37	1612
CGCATCGTTAATA	0.024	0.002	0.028	76	2675

**CGCATCATAAACG**	0.026	0.004	0.023	78	3245
**AGTAGTACTTACA**	0.024	0.008	0.015	28	1903
**GGCGGCACAAATG**	0.275	0.054	0.228	352	1194
**AGTATCACAAGCG**	0.024	0.002	0.026	33	1238
**AGCATTATTGACA**	0.022	0.005	0.022	90	4040
**CCTATCGCAAGCG**	0.028	0.002	0.025	43	1685
**GCTGTTGCTAGCA**	0.058	0.002	0.06	52	820
**ACCGTCATATGCA**	0.027	0.003	0.025	73	2864
**GCTAGTGTTGGCA**	0.052	0.011	0.052	52	939
**GCCGGTACTAGTG**	0.072	0.012	0.086	73	778
**CCTATTGCAAGTG**	0.024	0.003	0.021	38	1733
**AGTAGCACAGGTA**	0.021	0.008	0.012	14	1191
**ACCAGTACTGGCG**	0.02	0.004	0.016	30	1877
**CGTGTCGTAAACA**	0.019	0.001	0.02	35	1701
**CGTGTCGCTAGGA**	0.032	0.003	0.035	22	613
**CGCGTCGCTTGCG**	0.032	0.007	0.026	23	863
**AGTGTTATAAAGA**	0.022	0.002	0.02	44	2212
**ACCAGCGTTAGTA**	0.025	0.006	0.017	35	2020
**GCCAGCGCAAGTA**	0.053	0.01	0.059	80	1287
**AGCGGTACTTATA**	0.019	0.002	0.018	35	1894
**CGTGGTGTAGACG**	0.028	0.001	0.027	18	639
**GGCATTACTGGTA**	0.051	0.003	0.047	65	1305
**GCTGTCATTTATG**	0.055	0.003	0.058	56	908
**GCCATCATATATA**	0.053	0.007	0.043	151	3364
**CGTATCGTAGACA**	0.024	0.004	0.022	42	1852
**CCTGTTACAAAGG**	0.024	0.005	0.017	25	1415
**GCCAGTATAGGGG**	0.092	0.007	0.093	70	684
**CCCATTGCAAACA**	0.026	0.002	0.024	115	4755
**CGCATTGCTTGTG**	0.026	0	0.026	24	910
**ACTATTACAGATA**	0.022	0.004	0.02	87	4319
**ACCGTCACATACG**	0.023	0.003	0.022	64	2858
**ACCAGCATATACG**	0.023	0.003	0.019	65	3280
**ACCATTACTAGCA**	0.021	0.002	0.023	104	4452
**AGCGGTACATGCA**	0.018	0.004	0.014	25	1741
**CGCGTCATAAGTG**	0.022	0.005	0.022	31	1367
**GGCGTCGTAGATA**	0.072	0.015	0.052	64	1161
**AGTGGCGTATACA**	0.028	0.005	0.023	24	1005
**CGCGTCGCATGCG**	0.028	0.005	0.029	32	1057
**GCCGTCACTAACG**	0.058	0.003	0.061	105	1622
**CGCATCGCATATA**	0.024	0.007	0.018	40	2206

ACCGTTGCTTACG	0.022	0.003	0.023	40	1687
CGTGTTACTTGCG	0.019	0.001	0.017	12	674
AGCATCACTTACG	0.021	0.004	0.025	61	2407
ACCGGTGTTGGCG	0.025	0.006	0.021	29	1325
CGCATTATAGATA	0.025	0.004	0.021	69	3178
GCCGGTACTAGTA	0.055	0.005	0.058	78	1256
AGTAGCGTAAACG	0.025	0.003	0.021	30	1380
GCCGGCACTGGGG	0.208	0.025	0.215	109	397
GCTAGCGCTTGCA	0.067	0.017	0.053	44	793
GGCATTACTGACA	0.051	0.011	0.038	79	2021
CGCGTCATTTACG	0.023	0.002	0.025	36	1393
GGTGGTGTTAATA	0.111	0.008	0.1	82	735
AGTGTCGCAAATA	0.027	0.003	0.022	38	1666
CCTGGTGTTAATG	0.028	0.003	0.03	38	1215
ACTAGTACAAGCG	0.024	0.002	0.023	53	2209
CCCAGTATTGGTA	0.026	0.003	0.023	51	2145
GGTATTACTAATA	0.098	0.014	0.096	184	1734
GGCAGTGTAAAGA	0.051	0.006	0.051	81	1509
ACTATCGCTGGGA	0.026	0.007	0.02	25	1252
AGCATTATATGTG	0.024	0.004	0.025	55	2186
ACTGTCATTGATA	0.028	0.006	0.026	60	2207
CGCGGTATTTGTA	0.025	0.003	0.029	34	1143
CGCATCGTTTAGA	0.028	0.001	0.027	41	1462
AGCATCGTAAAGA	0.021	0.002	0.018	56	2976
CCTATTATTAGTG	0.025	0.003	0.026	49	1871
GGTAGTACTAACG	0.06	0.005	0.062	61	916
CCTGTTATAGGCA	0.024	0.003	0.026	46	1714
AGTGGTGTAAACA	0.021	0.007	0.015	26	1690
GCCATCATTTAGG	0.056	0.007	0.064	101	1470
CGTATTACTAACA	0.026	0.002	0.029	73	2487
ACTGTCGCATATG	0.024	0.005	0.017	24	1394
AGTAGCACAAGTG	0.027	0.003	0.026	29	1072
AGCAGCATTGGGA	0.021	0.004	0.016	24	1487
CCTGTTACTTATA	0.028	0.002	0.026	53	1951
GCTGTTACTAATG	0.063	0.011	0.066	73	1029
CGCGGCGCTAAGA	0.028	0.014	0.018	21	1137
CCCGTCATATGTA	0.028	0.005	0.029	69	2295
GCCATCGTTAAGA	0.047	0.009	0.059	99	1583
GGTAGTGCTGATG	0.068	0.008	0.058	29	474
GCCGGTGTAGAGG	0.362	0.065	0.375	298	497

GGCGTCGCTGACA	0.06	0.007	0.051	65	1204
ACTAGTACAAGCA	0.025	0.001	0.026	99	3725
ACCATCGTTAAGA	0.025	0.005	0.023	62	2655
ACCGTCGTTGGCA	0.033	0.007	0.024	34	1402
ACCGTTACTAATA	0.02	0.002	0.02	99	4848
CCTATCGCTAGTA	0.021	0.004	0.018	36	1958
CCCGGTGTAGAGG	0.017	0.003	0.015	16	1027
CGCGTTATTTGGG	0.025	0.009	0.037	26	676
AGTATCGTTAAGA	0.024	0.001	0.023	38	1601
ACCGTTACAGACA	0.024	0.003	0.028	131	4515
GCTATTGCTGGCG	0.047	0.005	0.054	59	1042
ACTAGCGCAAATA	0.021	0.001	0.02	75	3648
GCTAGCATTGGCA	0.056	0.007	0.056	63	1054
GGTAGTGTAGATA	0.059	0.008	0.047	50	1003
ACTGTTGTTGGGG	0.028	0.005	0.022	13	579
CCTATTACAAATG	0.028	0.003	0.026	86	3248
GCTGGTATAAGGG	0.422	0.038	0.448	339	418
GGTAGTGCAAATG	0.053	0.01	0.048	44	868
ACCGTTACTTATA	0.021	0.002	0.02	74	3608
ACTGTTGTAAGCA	0.02	0.003	0.02	36	1784
GCTGTCGTTTAGG	0.064	0.015	0.084	41	448
CCCGGTGTAAAGA	0.022	0.003	0.018	36	1945
CCCGGCACAAGCA	0.023	0.004	0.02	56	2796
GGTGTCGTATGTA	0.099	0.009	0.097	60	560
CCCATCATTAATA	0.026	0.002	0.026	136	5041
AGCAGTGCTTGTA	0.019	0.002	0.018	25	1358
CGCGGTGCTAACG	0.024	0.001	0.023	27	1128
AGCGTCGCAGACG	0.022	0.003	0.026	39	1444
AGCATCATAGGGG	0.025	0.003	0.022	39	1727
ACTGGTACTAAGA	0.027	0.001	0.026	58	2151
AGCATCATTAGCA	0.02	0.001	0.021	62	2943
CGCATTGCTTGCA	0.026	0.005	0.02	28	1364
GCCATCGTAGGCA	0.053	0.005	0.051	78	1454
CCCGGCACTTGCA	0.029	0.01	0.019	36	1861
GCCATCATAAAGG	0.063	0.003	0.063	135	2019
GCTGTCGTAGACG	0.063	0.014	0.081	58	658
AGCGTTATAGGCG	0.025	0.002	0.025	34	1315
GGCATTGCAGATA	0.056	0.001	0.056	93	1581
CGTGGCACTAAGG	0.022	0.01	0.036	21	560
ACCGTTGCTAATG	0.024	0.002	0.026	54	1997

ACCATTACAAGGG	0.026	0.002	0.024	60	2440
CCCAGCGTATATA	0.024	0.007	0.018	53	2851
AGTAGCATTAAGA	0.021	0.004	0.022	40	1771
CCTATTACTAGGA	0.026	0.002	0.022	41	1802
CCCAGCATTGACG	0.034	0.002	0.033	73	2160
GCCGTTGCAGACG	0.051	0.016	0.033	35	1011
GCTGTCACTAGCG	0.061	0.007	0.065	51	731
AGCGTCGCTAAGG	0.023	0.008	0.016	16	1015
CCCATTGCTTGGG	0.027	0.01	0.017	18	1017
CCCATCGCTGGTG	0.025	0.001	0.025	32	1270
AGCAGTGCTTGTG	0.015	0.001	0.015	15	964
ACTATCACATGTA	0.025	0.007	0.024	77	3090
CCTGTCATATAGA	0.031	0.004	0.036	63	1677
CGCATCACATATG	0.029	0.005	0.023	48	2020
ACCGGCATAAGCG	0.027	0.001	0.027	52	1867
ACCATTGCAGAGG	0.022	0.002	0.021	43	1968
ACTGTCACATGTA	0.027	0.001	0.027	58	2126
GGCAGTATTTGGG	0.052	0.015	0.055	40	683
AGCGGTATAAATG	0.019	0.002	0.021	46	2141
GCTGGTGCTGGGA	0.113	0.019	0.131	53	351
GCTAGTACTAGCA	0.071	0.008	0.061	83	1271
AGCGTCGTTAGTA	0.022	0.005	0.016	23	1451
CCTAGTATTGAGA	0.027	0.006	0.02	37	1852
CCTGGCGTAAGCA	0.024	0.003	0.02	34	1688
ACTGTCATAAGTG	0.022	0.001	0.023	37	1542
CGTGTCATTAACA	0.026	0.005	0.02	38	1895
CCCAGCGCAGGCA	0.026	0.003	0.028	69	2425
CCTGTCATTTACA	0.025	0.001	0.023	49	2085
AGTAGCGTAGAGA	0.019	0.009	0.013	12	935
GCCAGTACTTGGG	0.051	0.002	0.049	42	822
CGTGGCACATAGG	0.03	0.006	0.034	19	540
CCCGGCGCATAGG	0.018	0.005	0.011	12	1075
ACCAGTACTTGTA	0.019	0.001	0.018	60	3187
ACTGTCGTATGCA	0.024	0.002	0.021	28	1306
GCCAGTACATATG	0.061	0.007	0.061	107	1641
ACCAGCACTGATG	0.03	0.004	0.026	62	2342
ACTGGCGTTAGTA	0.026	0.002	0.023	21	888
CGCATTGCAAGCA	0.025	0.002	0.022	48	2093
AGCGTCGCTGACA	0.025	0.003	0.029	52	1752
ACCGTTATTAACG	0.023	0.003	0.02	58	2789

CGCATCATTTGCA	0.024	0.004	0.021	43	2038
ACCGGCGTTTGTA	0.03	0.007	0.021	22	1036
ACCGGTGCTAAGG	0.022	0.003	0.019	24	1208
CCCGGTGCTGGCA	0.022	0.003	0.019	26	1325
GGCATCACATGCA	0.067	0.008	0.071	130	1709
AGCATTACATATA	0.024	0.001	0.022	92	4037
CCCAGTACTGATG	0.024	0.003	0.02	43	2074
ACTATCGTTGACG	0.026	0.005	0.027	49	1777
ACCGTTACTGGGG	0.027	0.001	0.025	24	924
CCTATTGTAAAGA	0.026	0.005	0.02	59	2948
GGTAGCACTAATA	0.065	0.008	0.053	72	1286
CCCGGCGCTGGTA	0.026	0.007	0.022	28	1260
GCCAGCATATGTA	0.052	0.001	0.051	116	2177
CCTATTGTTAGTA	0.027	0.005	0.021	44	2019
GGTGGTATAGGGG	0.187	0.039	0.157	64	344
CGCATCATTGAGA	0.028	0.004	0.023	49	2047
GGCAGTGTTAGCG	0.056	0.005	0.054	42	734
AGTATCGTAGACG	0.022	0.004	0.023	31	1321
AGCATTGTAGACA	0.019	0.001	0.017	53	3057
CCCATCACTTGCG	0.026	0.001	0.025	52	1989
ACCAGTATTAACA	0.024	0.001	0.024	144	5736
ACCGTCACAAGCA	0.024	0.001	0.023	93	4005
GCTAGTATAAATA	0.065	0.008	0.057	175	2921
ACCAGTGCTAAGG	0.025	0.004	0.019	35	1812
GGTAGTGTTGGCA	0.061	0.01	0.051	29	539
CGCAGCACATATA	0.02	0.002	0.02	46	2312
ACTATCGCAGACA	0.024	0.006	0.017	50	2980
GCCATTGCTAGGG	0.044	0.007	0.036	27	724
CGCAGCACATGTG	0.026	0.005	0.03	36	1160
GCCGGCACATATA	0.115	0.003	0.116	230	1746
GCCATTGTTAATG	0.054	0.006	0.046	77	1602
CCTAGTATTAGTA	0.03	0.001	0.031	62	1951
ACTGGTGCTAGGA	0.019	0.001	0.019	18	950
GGCATTATAAGTA	0.063	0.008	0.06	156	2440
GGTAGCACTTACA	0.07	0.016	0.066	67	947
CCCATTATAGGTA	0.024	0.003	0.02	59	2917
AGTGGTACTTACA	0.025	0.003	0.022	27	1225
CGTATCGCTAGCG	0.029	0.011	0.029	25	836
GCCATCACTGGCG	0.054	0.004	0.055	97	1668
ACTGTCGCAGGCA	0.021	0.002	0.018	29	1545

**AGTATTATTAGCA**	0.023	0.002	0.022	50	2271
**CCCAGTACTAACG**	0.024	0.004	0.024	70	2883
**ACTAGCGCTAACA**	0.022	0.004	0.017	52	2960
**CCCGTTATATACA**	0.026	0.004	0.02	80	3831
**CCCGTCACATACG**	0.03	0.005	0.023	58	2488
**AGCATTGCAAACA**	0.019	0.004	0.02	82	4085
**ACCAGCACAGACA**	0.02	0.001	0.02	106	5184
**GGCAGCACATGCG**	0.052	0.007	0.056	54	910
**AGTATTGCATGTA**	0.022	0.004	0.017	24	1367
**AGCAGTGCTAACA**	0.021	0.001	0.02	56	2730
**ACCGGTGCATGGG**	0.03	0.004	0.035	31	848
**ACTATCATATAGA**	0.027	0.001	0.028	86	2936
**ACTGTTATTAGGA**	0.027	0.006	0.019	30	1567
**CCTAGCACAAGTA**	0.027	0.005	0.034	88	2471
**GCTAGCACAGGCG**	0.066	0.008	0.064	62	900
**GGCAGTGCTAACA**	0.053	0.007	0.043	67	1474
**CCTAGTACAGGCA**	0.028	0.004	0.028	55	1904
**GCTATTGTAAGGG**	0.062	0.008	0.054	55	955
**AGCATTATAGACG**	0.02	0.001	0.021	63	2957
**GGCATCACTTACG**	0.094	0.006	0.093	138	1352
**CCTAGTGCTTACA**	0.022	0.006	0.014	32	2204
**CCCATTGTAAGCG**	0.032	0.004	0.034	78	2233
**GCTGTCATATAGA**	0.064	0.003	0.06	76	1182
**GCTAGCATTTACA**	0.062	0.004	0.057	100	1646
**ACTAGTGTTTAGA**	0.023	0.002	0.02	36	1721
**CCCAGCATATGCG**	0.026	0.005	0.025	54	2066
**CCTATTGCTAATA**	0.023	0.002	0.021	66	3039
**ACTGTTACAGAGG**	0.027	0.005	0.033	39	1150
**GGCGTCATTAGGA**	0.061	0.007	0.069	62	840
**GCCATTATTTGGG**	0.05	0.009	0.05	56	1058
**ACCATTGTTGAGA**	0.02	0.002	0.019	43	2178
**CCCGTTGCAGATA**	0.025	0.002	0.024	57	2273
**CCTGGTACATATA**	0.019	0.002	0.018	36	2016
**CGTGGCGTTAGGA**	0.024	0.01	0.013	6	445
**CGTATTGTTAGCG**	0.03	0.008	0.027	23	834
**ACCAGCGCAGACG**	0.018	0.003	0.018	40	2152
**ACCGGCATTAACA**	0.023	0	0.023	85	3542
**CCCGGCATATGGA**	0.028	0.003	0.027	46	1637
**ACCAGCACATGCG**	0.022	0.004	0.02	55	2649
**ACTGTTATTTAGG**	0.027	0.006	0.022	27	1183

CCTATTGCATGTA	0.023	0.004	0.023	50	2096
CCCGTCACAAGTG	0.026	0.008	0.018	35	1875
GCCGTTATAAATA	0.058	0.003	0.058	205	3341
GGTAGTATAAACA	0.06	0.007	0.051	107	2009
CGCGTCGTTAGTG	0.03	0.005	0.037	31	798
GCCAGTATAGACA	0.053	0.002	0.051	166	3118
CGCATCATAAATG	0.025	0.005	0.022	71	3153
ACTATCACTGGCA	0.019	0	0.019	48	2469
GGTGTCATATGTA	0.093	0.01	0.088	80	832
AGCATTACATAGA	0.021	0.006	0.013	37	2740
ACCATTGTAGATA	0.027	0.003	0.024	81	3318
GCTAGCGCAAACA	0.066	0.007	0.075	128	1586
ACCGGCATAGGTG	0.027	0.009	0.03	36	1150
ACCATCATAGAGG	0.023	0.003	0.023	51	2131
ACTGGTGTAAAGA	0.028	0.006	0.019	33	1670
CCCGTCGTTTGCA	0.022	0.003	0.022	39	1757
CGCAGTGCAAGTG	0.03	0.006	0.021	24	1104
ACTAGCATTTAGA	0.024	0.004	0.02	39	1951
CCTGGTACTGATA	0.024	0.006	0.032	54	1656
AGCAGCACAAGTG	0.027	0.002	0.028	51	1759
CGTGGTACTTGGG	0.03	0.009	0.02	8	384
GGCATTGCTGAGG	0.085	0.017	0.062	46	698
CGTATTATATACA	0.028	0.007	0.018	53	2897
ACTAGTGCAGATA	0.021	0.002	0.018	50	2681
AGTATCATAAACG	0.024	0.002	0.022	50	2259
AGTATCATAGGGG	0.022	0.002	0.019	20	1017
CCCGGCACAAAGA	0.027	0.003	0.024	66	2663
ACTATTATTTGGG	0.028	0.003	0.024	34	1379
ACCGGTATTAGCA	0.024	0.002	0.022	55	2469
GGCATTACTAATA	0.054	0.003	0.051	133	2469
ACCGGCGCAGATG	0.026	0.004	0.021	26	1224
ACTGGTACTGGTA	0.026	0	0.025	36	1382
AGCGTTACAAATA	0.02	0.001	0.02	77	3689
AGTAGCATTGAGG	0.024	0.004	0.027	23	843
AGCGTTATAAACG	0.019	0.003	0.015	42	2677
GGCAGTATATGTG	0.062	0.014	0.042	51	1165
ACCATTATAAGTG	0.026	0.002	0.028	97	3330
CCTGTCGCTTAGA	0.024	0.009	0.034	39	1110
AGTGGCACTTATA	0.022	0.003	0.018	19	1040
GCTGTCACAAACA	0.052	0.007	0.043	88	1936

ACTATTGTTAAGG	0.03	0.005	0.034	59	1687
CCCGTTACAGGGA	0.028	0.002	0.025	37	1426
GCTATTACTAGTA	0.059	0.008	0.049	78	1500
GCCATTGTTTACA	0.049	0.003	0.045	98	2070
CCTATCATATACG	0.027	0.002	0.023	74	3110
CCCAGTATTGACG	0.029	0.002	0.027	64	2303
CGCAGCATTTAGG	0.028	0.004	0.023	25	1046
ACCAGTGCATACG	0.025	0.001	0.025	66	2545
CCCGGTGTTAGCG	0.024	0.001	0.026	29	1093
CCTGTTATAGGTG	0.029	0.008	0.018	21	1143
ACCAGTGTTAGGG	0.025	0.003	0.026	29	1085
CCCATCGCAGGCA	0.021	0.002	0.019	52	2643
CGCGTCACAAACA	0.024	0.002	0.023	77	3284
GGCAGTGTAGAGG	0.052	0.006	0.044	36	774
AGCATCGCTTAGA	0.017	0.002	0.02	32	1580
ACTAGCGCTAGTA	0.021	0.003	0.017	31	1800
ACTGGTGCTAACA	0.024	0.003	0.021	44	2079
CCTAGCATAAACG	0.028	0.005	0.021	58	2686
GGCGGCGCAAGCA	0.108	0.017	0.114	124	962
CCCATTGTTGATA	0.025	0.006	0.017	49	2835
CCTAGCGCTTGTA	0.023	0.001	0.024	35	1408
AGCGTTATAAGGA	0.022	0.004	0.017	34	1978
CCTAGCACTGGCA	0.027	0.01	0.017	26	1541
GGCATTATAGATG	0.067	0.004	0.065	111	1594
ACCATCGTTTACA	0.024	0.004	0.019	59	3050
GCCGTCATAAGCA	0.057	0.006	0.051	117	2190
ACTATCACTAAGG	0.029	0.003	0.029	62	2111
ACCATCGCTGGTA	0.023	0.003	0.02	45	2197
ACTGGCGCTAGCA	0.026	0.005	0.024	18	746
AGTATTACTGACA	0.022	0.002	0.021	51	2330
CCCATTATTGAGG	0.026	0.003	0.024	46	1859
CCTGGTACTTGTA	0.025	0.004	0.02	24	1200
ACTAGTACTGACA	0.022	0.004	0.023	83	3539
CCCGGTATTTGGG	0.026	0.004	0.021	19	876
AGCATCACATACA	0.023	0.002	0.021	88	4100
ACCATTGTTTAGA	0.022	0.003	0.019	44	2307
AGCGTCATTAATA	0.022	0.003	0.02	64	3132
GCTAGCATATACG	0.068	0.007	0.062	87	1320
AGCGGCACATGTG	0.021	0.005	0.016	13	821
ACCGGCGTATATA	0.027	0.002	0.029	48	1611

GGCATCGCAAACG	0.113	0.02	0.104	179	1539
CCTAGCATTGAGA	0.02	0.003	0.017	29	1671
CGCGGCGCAGAGA	0.024	0.003	0.027	27	976
AGCATTACTAGTG	0.023	0.003	0.02	36	1732
CCTATCATTTAGA	0.026	0.006	0.018	46	2465
AGCGTCATTTAGA	0.029	0.003	0.033	60	1741
CGTAGTACAGGCA	0.025	0.003	0.022	23	1032
AGTGGTGTTTAGA	0.023	0.004	0.024	17	697
AGCGTCACTAACA	0.024	0.002	0.025	72	2793
CCTGGTATATACG	0.027	0.001	0.026	40	1487
AGCGTTACATATG	0.025	0.003	0.022	38	1712
ACTAGCGCTTGTG	0.02	0.001	0.022	21	954
AGTGGCACAAACA	0.028	0.004	0.024	45	1793
CGTGTCACAAAGA	0.025	0.003	0.021	31	1428
ACCGGCGCTAATA	0.026	0.003	0.022	37	1651
ACCAGCACATGGA	0.021	0.002	0.02	50	2500
GCCAGCGCTAACA	0.055	0.005	0.062	119	1805
GCTAGCATTGACA	0.063	0.004	0.068	116	1580
CGTATTACTAGCA	0.026	0.005	0.019	35	1767
GGTGTTGTAGACA	0.084	0.003	0.086	77	816
GCTGGCATAGAGA	0.635	0.026	0.621	816	497
GCTATCGCTTACA	0.055	0.01	0.046	62	1296
GCCATCATAAGGG	0.081	0.004	0.076	91	1110
GCCATCACAAAGG	0.051	0.006	0.058	116	1876
CCTAGTATAAGCG	0.027	0.005	0.022	44	1954
ACCATTACAAGTG	0.025	0.003	0.026	84	3150
AGTATTGCATACA	0.025	0.005	0.032	73	2181
ACCGTTATTAGCA	0.023	0.004	0.018	47	2622
CGTGGTACTTGGA	0.024	0.005	0.017	11	636
ACCAGTATAAGTG	0.024	0	0.024	71	2871
GCCAGTGCAGATA	0.048	0.001	0.047	74	1505
GCCAGCATAGACA	0.054	0.008	0.043	132	2923
CCCGTCATTAATA	0.024	0.004	0.02	72	3561
AGCGTCATATGTA	0.022	0.003	0.019	41	2159
GCTAGTACAGGCA	0.069	0.003	0.074	91	1145
AGCATTACATGCG	0.026	0.003	0.029	62	2057
ACCATCGCTAGGA	0.02	0.004	0.016	34	2139
ACCAGCGCTTGCA	0.024	0.002	0.026	72	2736
CCCGTCATATATG	0.029	0.004	0.023	53	2241
ACCGGCACTAAGG	0.026	0.006	0.019	32	1612

AGTGTTGTTAGTA	0.02	0.004	0.014	14	1006
GGTGGCGTAAGTA	0.408	0.068	0.414	330	468
AGCAGTACTAGTA	0.021	0.002	0.02	49	2408
GGCGGCACTTATA	0.141	0.029	0.128	166	1131
GCTAGCGTAGAGA	0.061	0.011	0.074	66	826
GCCGTCGCAAATG	0.04	0.001	0.041	44	1017
CCCAGCGCTTGTG	0.025	0.004	0.021	25	1159
CCCATTGTTGGCA	0.023	0.001	0.022	42	1865
CCTGTCACTAAGA	0.023	0.005	0.028	52	1805
CGCAGTGCAGGGG	0.019	0.006	0.014	8	573
CGTGGCACAAGTA	0.031	0.004	0.025	29	1123
GCCAGCACAGGGG	0.074	0.001	0.074	57	714
ACTATTGTTTACG	0.027	0.005	0.022	45	1977
CCTATTACAGACA	0.03	0.003	0.03	116	3692
CCTATTGCATACG	0.023	0.006	0.015	34	2185
CCTATTGCAGACG	0.027	0.002	0.027	58	2073
CCTATTATTAATG	0.026	0.003	0.027	83	3004
CCCGGTGCAGGGA	0.023	0.002	0.02	22	1061
CCTAGCGTATGGA	0.024	0.009	0.016	20	1227
ACCGGTGCAGGTG	0.023	0.005	0.016	18	1079
GGTGTCATTGGCA	0.097	0.016	0.075	48	588
CGCATCGCATATG	0.027	0.006	0.035	51	1399
GGCGTCGCATGCA	0.068	0.02	0.058	64	1032
GGTGGCATTTGCA	0.487	0.055	0.458	378	448
GGTAGCACTAGCA	0.058	0.007	0.063	54	802
GGTATCACAGACA	0.092	0.014	0.083	117	1289
GCCAGTGCATACG	0.055	0.004	0.049	67	1288
GCCGGTACATGGA	0.056	0.001	0.056	60	1007
CGCAGTACTGGGG	0.034	0.007	0.035	23	636
CGTATCGTTGACA	0.023	0.006	0.018	29	1578
ACCGTTGCTTAGA	0.017	0.004	0.012	20	1696
GGTATCGTTTATA	0.145	0.019	0.15	178	1005
ACCGGCATAAAGA	0.026	0.004	0.025	75	2906
ACTATTGTATGGG	0.026	0.002	0.025	31	1190
CCCAGCGTTTATA	0.021	0.005	0.015	38	2424
GGCAGTACTAGGA	0.049	0.005	0.053	57	1015
CCTGGCGTAAGTA	0.024	0.002	0.026	41	1513
CGCGGTACTGATG	0.025	0.006	0.029	29	968
GGTATTATAAACA	0.1	0.017	0.087	255	2675
ACCGTTACTGATG	0.022	0.003	0.027	67	2430

**ACTAGTATATGTG**	0.029	0.004	0.024	42	1731
**GCTGTCGTTAGCA**	0.053	0.002	0.054	48	845
**ACCATTACAGGTG**	0.025	0.001	0.027	69	2482
**AGCATCGTTGGGG**	0.02	0.003	0.017	17	986
**ACTAGCACTAAGG**	0.024	0.004	0.027	46	1631
**CGCAGCACTGACG**	0.028	0.002	0.03	44	1411
**AGCATTATATATA**	0.019	0.002	0.021	113	5167
**ACCGTCATATGTA**	0.023	0	0.023	64	2703
**ACTATTGTAGGGG**	0.023	0.004	0.019	22	1113
**ACCGTCGCTTGCA**	0.024	0.001	0.023	45	1923
**GGCGGCGTTTAGG**	0.425	0.071	0.415	274	386
**GCCAGCACAAAGA**	0.053	0.007	0.053	129	2292
**GGCATCATATGGA**	0.077	0.012	0.072	100	1284
**CCCATCGTTGGGG**	0.038	0.006	0.032	28	851
**CCTATCGTAGACG**	0.023	0.007	0.019	38	1982
**ACTAGTGTTAGCG**	0.03	0.007	0.021	29	1331
**ACCAGTGCAAGGG**	0.027	0.001	0.028	45	1557
**AGCGGCATAGGGA**	0.018	0.003	0.022	28	1272
**CCCGTCGTTGAGA**	0.02	0.001	0.019	30	1527
**AGTATCGCAGGTA**	0.021	0.003	0.024	24	980
**GGCAGTGCTAACG**	0.048	0.003	0.049	58	1122
**CCCGTCGTATACA**	0.02	0.005	0.014	39	2815
**ACTAGCATTAGGA**	0.027	0.005	0.02	36	1726
**CCTATTGCAGGTG**	0.027	0.003	0.03	40	1306
**ACCGGCACAGAGG**	0.027	0.001	0.029	41	1371
**CCTATCGTTAAGG**	0.025	0.008	0.016	25	1511
**CCCAGTGCTGATA**	0.024	0.005	0.017	40	2288
**GCCAGTACAAATG**	0.052	0.003	0.052	120	2198
**GCTATTACAAAGG**	0.059	0.007	0.068	121	1671
**AGTAGCGTTAACA**	0.024	0.006	0.018	32	1779
**ACTAGTGCAGGCG**	0.02	0.003	0.022	31	1396
**ACTAGCACTAGTA**	0.028	0.002	0.029	70	2370
**CGTATTACTAAGG**	0.025	0.004	0.02	24	1166
**AGTAGTATTAATA**	0.024	0.003	0.023	62	2687
**CCCGGTATAAGCG**	0.024	0.003	0.023	41	1767
**ACCATCATTGATA**	0.023	0.003	0.022	94	4226
**AGCAGCGTATAGG**	0.024	0.003	0.022	28	1224
**CGCGGCGTATATA**	0.026	0.008	0.02	25	1249
**CCCGTCGTTAGTA**	0.024	0.003	0.021	37	1744
**CGTAGCATATGCG**	0.023	0.003	0.021	19	905

AGTGTCGTATAGG	0.029	0.014	0.026	17	629
AGTGGTGTATGCA	0.026	0.001	0.026	24	891
GCCATCACAGGCA	0.051	0.002	0.053	110	1981
CCCGTTGTTGACG	0.021	0.001	0.021	34	1592
GCTATTATATGTA	0.064	0.008	0.067	143	2004
CCCATCATAGACG	0.031	0.002	0.03	94	3045
GCCGTTACTTAGA	0.061	0.007	0.059	75	1191
CGTGGTACAAACA	0.023	0.003	0.022	40	1804
GCTAGTACTAATG	0.068	0.011	0.053	71	1264
CGTGGCGCATACA	0.029	0.005	0.023	25	1074
GCTAGTATATGCA	0.06	0.008	0.06	103	1608
AGCGGTATATAGA	0.02	0.003	0.015	31	1992
CCCAGTACATGTG	0.022	0.002	0.025	49	1908
CCCGGCGTATGTG	0.026	0.002	0.025	26	1009
CCCATTATTTAGG	0.023	0.004	0.024	51	2119
CGCAGTATAGAGG	0.026	0.002	0.027	35	1238
GCTAGCATTGATA	0.063	0.005	0.064	110	1619
ACCATCATAAACA	0.025	0.003	0.021	162	7481
AGCAGTATTTACA	0.02	0.003	0.018	65	3451
CCCGTCGTTAACG	0.026	0.002	0.023	47	1963
CCTGTTATATGTA	0.025	0.001	0.025	47	1850
CCCATTGCTTAGG	0.025	0.003	0.028	39	1331
GCTATTGTAAACG	0.068	0.008	0.057	114	1874
CCTATCATAGGTG	0.048	0.011	0.039	61	1489
CCCGGTACTTGCG	0.026	0.002	0.028	38	1319
AGCGTCATAAATG	0.019	0.002	0.021	56	2607
CCTAGTGCTAACG	0.02	0.005	0.012	21	1750
AGCATCGCAAACG	0.024	0.001	0.024	68	2743
AGTGGCATAAGTA	0.022	0.003	0.024	19	780
ACTAGCACATATA	0.023	0.002	0.02	81	3923
AGCGGCGTTTATA	0.024	0.005	0.022	29	1264
AGTATTATTTGGA	0.023	0.004	0.021	30	1414
CCTATTGTTAATG	0.028	0.002	0.028	62	2188
GGTGGTATAGGCG	0.268	0.082	0.216	114	413
CCCGTCATTGATA	0.025	0.005	0.02	55	2661
GGCATCACAAAGG	0.087	0.005	0.084	145	1578
GGCATCACAGATG	0.085	0.008	0.078	119	1410
CCTGTCGTTAGCA	0.029	0.001	0.029	44	1452
CGTATTATTGAGA	0.029	0.005	0.026	41	1515
CCCAGCATTGGCG	0.031	0.004	0.025	38	1465

ACCATCGTAGATA	0.026	0.004	0.023	70	3001
GCCAGCATAAAGA	0.055	0.004	0.055	165	2820
AGTGGTATAGATA	0.027	0.002	0.026	42	1569
ACCATTGTTGACA	0.024	0.002	0.026	85	3194
ACCGTCGTAAGTA	0.025	0.002	0.025	53	2031
GCTAGCATAGAGA	0.07	0.006	0.066	89	1268
ACCAGCACAAATG	0.023	0.004	0.022	101	4432
GGTAGTACAAGGG	0.065	0.005	0.059	37	593
CGTGTTATAAACA	0.022	0.004	0.018	43	2365
GCTAGCATAAATA	0.062	0.006	0.055	150	2597
ACCAGTGCATACA	0.023	0.003	0.019	78	3968
ACCATCACTGGTA	0.024	0.001	0.025	80	3092
CCCGTCGCAAAGG	0.022	0.005	0.021	33	1518
ACTGGTGTATACA	0.027	0.005	0.023	44	1846
AGCATTATATAGG	0.021	0.006	0.015	34	2205
CCCAGCACTAAGA	0.029	0.004	0.024	72	2945
GCCATTGTTAACA	0.051	0.006	0.045	118	2531
AGCGGCGCTTATG	0.022	0.008	0.012	10	833
GGCAGTATTAGCG	0.055	0.005	0.051	57	1050
ACTGTCATTGGCA	0.022	0.002	0.024	38	1534
CGTATCATATAGA	0.023	0.001	0.024	49	2015
ACCATTACTAAGG	0.023	0	0.023	75	3241
GGTAGCACAGATA	0.076	0.011	0.067	76	1061
CCCGTCACAGAGG	0.03	0.003	0.033	50	1487
ACTGGTATTTAGA	0.023	0.003	0.024	36	1477
ACTGGTATTGACA	0.026	0.001	0.028	59	2038
CGTAGTACAAGGG	0.031	0.007	0.04	28	671
GGTGTCATAGGCA	0.094	0.022	0.063	48	716
AGTAGTACTTATG	0.024	0.004	0.029	34	1153
GGCGTCATAAGGA	0.058	0.002	0.054	67	1163
ACTGTTATAAACA	0.025	0.001	0.026	105	3994
CCCGTTATAGATA	0.023	0.004	0.019	60	3165
CCCGGTACATATG	0.027	0.004	0.022	42	1841
CCTGGCGCAGATG	0.024	0.004	0.021	19	904
GCCGGTGTAAATA	0.172	0.023	0.157	318	1708
GCCAGTACATAGG	0.052	0.004	0.058	73	1189
GCCAGTATAAGGA	0.051	0.003	0.053	114	2027
ACTAGCACAGATA	0.023	0.002	0.025	87	3407
ACCAGCGTAAGCG	0.021	0.003	0.017	30	1699
CCCATTGTTTGTG	0.027	0.003	0.026	38	1415

CCTGGTGTAAACA	0.022	0.001	0.021	51	2335
AGTGTTACTAACA	0.02	0.002	0.017	35	2043
GGCAGTATTTATA	0.048	0.006	0.041	90	2105
CGCATCGCAAGTA	0.024	0.002	0.023	45	1871
GGCGTTATAAACA	0.061	0.01	0.053	144	2572
CCTAGCGCAAGTG	0.024	0.004	0.025	32	1233
AGCGTTGCTTATA	0.017	0.003	0.021	37	1749
GGTGTTACTGGCA	0.068	0.005	0.064	48	698
CCCGTTATAGACA	0.027	0.001	0.026	90	3434
ACTAGCACTTAGA	0.021	0.004	0.023	54	2311
CCCATTGTAGACG	0.026	0.002	0.023	58	2434
ACTGTTGCAGGCG	0.027	0.001	0.026	30	1125
CCTATTACAAACG	0.03	0.001	0.028	100	3463
CGTATTGTTGATG	0.027	0.005	0.033	33	956
CGTATTACAAAGG	0.029	0.001	0.027	43	1522
GCCATTGTTGGGG	0.052	0.001	0.05	33	629
CGCAGCGCTTACG	0.02	0.005	0.014	17	1231
ACTAGCATAGACA	0.024	0.002	0.022	69	3123
GCCAGCATTGGGA	0.057	0.008	0.048	46	920
AGCGTCACTAGCA	0.022	0.002	0.025	49	1920
ACCATTATTTGGA	0.02	0.002	0.017	42	2463
AGCGTTATTTGCG	0.021	0.003	0.023	29	1206
CCTGTCACAAGTA	0.026	0.008	0.016	33	2095
CGCATTGTAAGCG	0.022	0	0.022	36	1599
CGCAGTGTTGATG	0.029	0.009	0.017	19	1076
CGCGTCGTTGAGG	0.018	0.003	0.02	16	770
ACTAGTACTAGCA	0.022	0.003	0.026	79	2939
CCCAGTATATGCG	0.025	0.004	0.019	40	2074
ACTGGCGTAAATA	0.027	0.003	0.027	49	1799
GGTATCGCATATA	0.114	0.016	0.095	112	1071
CGCGTTATAGATG	0.029	0.005	0.025	38	1508
AGTATTGTTAACA	0.025	0.001	0.025	63	2461
CCTAGTGTTAACG	0.028	0.006	0.026	52	1944
AGTATTATTAACG	0.021	0.004	0.024	55	2273
GGCGGTGCTGAGG	0.064	0.003	0.065	31	445
AGCATCACTGAGG	0.019	0.002	0.021	32	1475
GGTATCATTTGCA	0.118	0.032	0.086	95	1005
ACCGTTACATACA	0.023	0.004	0.019	96	5066
ACCGTTACATAGA	0.021	0.002	0.023	78	3312
GCTGGCATTAATA	0.656	0.044	0.641	1460	817

AGTATTATAAGTG	0.023	0.001	0.023	44	1880
AGCGTTGCTTGTG	0.027	0.009	0.038	31	777
ACCATTGTTAGGA	0.024	0.001	0.026	49	1857
ACTATCGCAGGGA	0.026	0.003	0.022	29	1304
AGTAGTATTGACG	0.019	0.008	0.008	11	1294
ACCGTCACAGATA	0.024	0.003	0.02	76	3781
ACCGGTACAAAGA	0.021	0.001	0.021	87	3967
CGTAGCACTTGCA	0.028	0.002	0.026	26	982
CCTATTGTATATA	0.023	0.002	0.023	71	3076
AGTATTATAAATG	0.023	0	0.024	73	3016
CCCAGTACAGACA	0.028	0.002	0.03	128	4148
ACTGTCGCTTGCA	0.027	0.007	0.026	38	1412
CCTGGTATTGGTA	0.03	0.005	0.025	26	1034
CGCAGCGTTAGCA	0.024	0.003	0.023	34	1456
CCCAGCATAAACG	0.024	0.002	0.022	86	3839
CGCATTATTGGTA	0.02	0.003	0.023	42	1750
GGCATTGCATGCG	0.066	0.011	0.062	64	964
CCTAGCGTAAGGA	0.027	0.003	0.026	37	1409
ACTGTCGTAGGGA	0.028	0.007	0.025	22	862
CGCATCATTGGGA	0.025	0.002	0.027	33	1189
CCCATCACAAACG	0.026	0.003	0.026	108	4014
GCCGTTATAAACG	0.054	0.006	0.047	108	2197
GCTATCGTTAGGA	0.054	0.007	0.063	57	841
AGTATCACATGTG	0.025	0.004	0.029	35	1178
CCCAGCACAAGGA	0.033	0.006	0.026	63	2325
CCTAGTACATGGA	0.025	0.004	0.02	32	1549
GCTAGTATTGGTA	0.068	0.004	0.064	76	1119
AGTAGTACAGGGG	0.03	0.001	0.03	20	638
AGCGTTACAAAGG	0.023	0.005	0.021	34	1592
ACTAGCGCTTAGA	0.022	0.001	0.023	38	1605
GGTGTCACAGAGA	0.085	0.025	0.059	48	761
GCCAGCACTGAGA	0.055	0.005	0.054	82	1423
CGTGGCATATATA	0.026	0.003	0.03	37	1188
AGCAGTGTAGGGG	0.019	0.002	0.021	24	1125
GGTAGTACATAGA	0.061	0.005	0.055	55	942
GCTGTCGTTAGGA	0.047	0.009	0.043	29	643
GGTAGCGTTGGCA	0.085	0.012	0.07	39	516
GCTATCGCTGAGA	0.049	0.007	0.049	41	799
GCCGTCATTAGTG	0.062	0.004	0.057	67	1101
GCCAGTATAAACG	0.058	0.004	0.053	165	2925

CCCGGCGCATACG	0.026	0.007	0.031	54	1684
ACCGGTACTTGCG	0.022	0.003	0.025	44	1735
CGCATTACATAGA	0.026	0.001	0.027	57	2090
ACTGGTGCTGGTA	0.019	0.002	0.021	21	972
ACTAGTGTAAGTG	0.029	0.006	0.021	35	1628
GGTATTGTTAAGG	0.114	0.012	0.103	81	706
GGCAGCATTGACA	0.039	0.005	0.045	90	1890
ACTAGCGCAGAGG	0.023	0.005	0.022	23	1032
AGCAGCATTGGTA	0.02	0.008	0.017	31	1744
CCTGGCATTAACG	0.034	0.005	0.037	56	1478
ACTGTTATTTATA	0.022	0.001	0.022	58	2611
GCCGTCACATGCA	0.055	0.006	0.049	88	1701
AGCATTATTAACG	0.022	0.002	0.026	85	3241
AGCATCGTTGAGA	0.022	0.002	0.02	36	1744
AGCATTGCAAGGA	0.024	0.003	0.026	50	1854
CCTAGCGCTTGCG	0.026	0.004	0.022	21	945
ACCATCACTTGCG	0.023	0.001	0.023	58	2431
GCCGTTGCTAAGA	0.061	0.006	0.052	58	1047
GGTATCACATACA	0.103	0.017	0.09	141	1429
ACTATTATTTACG	0.03	0.004	0.035	93	2567
CGTGTCGTTGGTG	0.019	0.004	0.021	10	457
GCCGTCACAGACA	0.053	0.004	0.048	116	2292
AGCATCGCAAGGG	0.021	0.003	0.017	23	1327
CCTATCACTGGTG	0.026	0.004	0.024	32	1329
GCTATCATTAACA	0.06	0.002	0.063	180	2694
AGCAGTACATGTA	0.019	0.001	0.019	49	2574
CCCAGTGCTAGTA	0.026	0.001	0.025	51	1974
GGTGTCACAAGCG	0.112	0.02	0.09	62	626
CCTGTTATAGGCG	0.027	0.002	0.03	37	1209
ACCAGTGCAGGGA	0.021	0.002	0.022	36	1638
AGTATTGTTAGGA	0.03	0.005	0.036	43	1136
ACTAGCATAAGCG	0.029	0.005	0.03	58	1870
CCCGGCACAAATG	0.021	0.003	0.017	42	2423
AGCGTCGCTGACG	0.021	0.002	0.022	28	1225
CCTGGTACAAGTA	0.023	0.001	0.022	41	1807
GCCAGTGTTGATG	0.053	0.005	0.046	52	1072
GGCATCGCAAACA	0.094	0.011	0.081	197	2245
AGCAGTACATACA	0.017	0.002	0.017	64	3720
ACTGGTATTGATG	0.031	0.009	0.019	23	1217
AGTAGTACTTAGA	0.027	0.004	0.033	45	1315

CCTGTCATAGACA	0.028	0.001	0.027	68	2492
CCCGTCATTTAGG	0.028	0.01	0.02	26	1292
GGCATCGCAAATA	0.086	0.009	0.078	184	2168
GCCGGTGCTAACG	0.105	0.016	0.087	87	917
GGCATTATATATA	0.06	0.005	0.061	179	2778
AGCATCATTAATG	0.02	0.001	0.02	63	3026
CGTATTATAAACA	0.026	0.002	0.025	96	3802
CCCGGTGCTTATG	0.027	0.007	0.017	18	1061
AGCGTCACTGGCA	0.022	0	0.023	36	1560
ACTGGTACAAGCG	0.024	0.006	0.016	23	1412
CCTGTTGCAAATG	0.026	0.006	0.02	34	1651
GGTAGTGTATGCA	0.062	0.016	0.046	37	773
AGCAGCGCATGGG	0.027	0.003	0.029	26	861
ACCATTGCAGATG	0.024	0.002	0.023	61	2612
CCCATCGTATGTG	0.024	0.002	0.022	42	1846
ACTAGTACAAATG	0.025	0.001	0.025	84	3341
GGCGGCGCATATG	0.184	0.036	0.168	128	634
AGCAGTGCTTATG	0.016	0.002	0.014	20	1454
AGCATCGTTAGTG	0.021	0.002	0.018	26	1410
AGCAGCATAGACG	0.023	0.003	0.024	55	2224
ACTATTGTAGACG	0.026	0.001	0.025	57	2182
ACCGGTATTGGGA	0.025	0.009	0.013	17	1316
ACCATCACTAGTA	0.022	0.001	0.022	87	3885
CGCGTTACAGGGG	0.028	0.009	0.035	27	748
ACTAGCGCAAGGG	0.024	0.003	0.024	25	1013
ACTATTACATATG	0.023	0.002	0.021	71	3379
ACCGGTACATGCG	0.022	0.005	0.015	33	2165
CCTATTATATGGG	0.024	0.008	0.014	20	1455
ACTATTGCTAATA	0.024	0.003	0.023	85	3568
CCTAGTGTAAGGA	0.028	0.002	0.027	46	1667
ACCGGCGCTGATG	0.022	0.002	0.02	22	1099
ACCGTCATTGACG	0.024	0.004	0.028	62	2160
AGCAGTGCTAAGA	0.021	0.003	0.017	31	1792
GCTGGTACTAATG	0.097	0.016	0.108	100	822
ACCATCATTTGTA	0.027	0.003	0.024	81	3246
CGTGGTATAGATA	0.022	0.002	0.023	31	1324
GCCAGTACAAGTA	0.052	0.006	0.047	116	2370
AGCATTATTTGGA	0.02	0.001	0.022	46	2082
CGTGGCACAGATA	0.026	0.006	0.034	39	1100
CCTATTGCATGTG	0.03	0.002	0.03	40	1314

GGCGGTATAGGGG	0.08	0.008	0.07	45	595
AGCGTTACAGAGA	0.022	0.007	0.014	25	1744
CCTGTCACTTGTG	0.024	0.005	0.031	31	981
CCCATTATAGGCG	0.026	0.003	0.027	60	2143
GCTGTCATAGATA	0.071	0.006	0.071	114	1489
GGTGGCATTGGTA	0.464	0.047	0.454	359	431
GCCGTTACAGATA	0.055	0.008	0.053	109	1938
GCTGGCACTTATA	0.225	0.018	0.243	287	892
CGTGTCATTAATA	0.024	0.004	0.03	55	1789
GCTAGTGTAGGTA	0.059	0.01	0.046	49	1016
GGCATTACAAACG	0.062	0.002	0.062	148	2226
GCTGTTGTAGACA	0.057	0.007	0.051	62	1142
CGCAGCATTTAGA	0.024	0.001	0.026	42	1571
CGCGTCGTATAGG	0.02	0.003	0.018	17	926
GCCGGTACTTAGG	0.071	0.014	0.052	37	681
AGCATCATTTAGG	0.026	0.003	0.026	46	1725
CCTGTCATATGGA	0.025	0.005	0.022	30	1358
ACCGGTATAGGTA	0.024	0.004	0.018	36	1912
CCTATTATTAGTA	0.029	0.003	0.025	71	2734
CCCAGCGCAGGTA	0.021	0.003	0.019	40	2042
CCTAGCGTATGGG	0.027	0.002	0.03	24	778
CGCGTCATAAGCG	0.026	0.003	0.022	32	1394
GCCATTACTAATG	0.056	0.004	0.061	140	2167
CGCGTCACTAACA	0.024	0.004	0.018	48	2577
CCTGGCACTAGGA	0.027	0.002	0.026	29	1080
CCCGTTGTAAAGG	0.021	0.003	0.025	43	1697
ACCATTACTAATG	0.024	0.004	0.027	123	4422
AGCATCGCATATA	0.024	0.005	0.02	53	2626
CCCGTCGTAGGTG	0.021	0.003	0.025	29	1119
GCCAGCGCTAATA	0.045	0.002	0.043	70	1544
CCTGGCGCTAATA	0.029	0.006	0.037	60	1561
ACTAGCACATAGG	0.026	0.007	0.017	28	1615
CCTATTGCAGACA	0.028	0.003	0.024	72	2932
CGCGTTGCAAAGA	0.021	0.004	0.017	28	1665
CCTGGCGCAAGCA	0.027	0.006	0.034	52	1461
GCCAGTACAAGCA	0.05	0.007	0.053	131	2345
GGTATCGTTAGTG	0.147	0.018	0.138	79	494
GCCAGTATATACA	0.052	0.002	0.049	175	3380
CGTAGTATAGACA	0.03	0.005	0.032	57	1719
GGCAGTATAAGGG	0.057	0.006	0.063	70	1047

GCTATTGTAGGCG	0.05	0.001	0.051	62	1160
CCCGGCACTAGGG	0.029	0.004	0.034	35	999
GGCATCATTGATA	0.081	0.009	0.081	167	1904
GCTGGTGTTGATA	0.416	0.037	0.412	436	621
ACCATTATAAACG	0.023	0.003	0.019	104	5350
GGCATTGCTAGCA	0.053	0.005	0.058	76	1224
GCTATTGTAAATA	0.054	0.004	0.051	144	2702
GCTAGTACATGTA	0.061	0.013	0.058	79	1294
AGTGTTACATAGG	0.022	0.004	0.019	18	910
ACTATCGCAGATA	0.024	0.004	0.02	57	2754
CGCGGCGCTGACG	0.016	0.003	0.016	14	875
GCTGGTGTAAATA	0.399	0.039	0.386	709	1127
AGCGGCGTATGTG	0.018	0.004	0.023	16	677
ACTGTCATATGTA	0.024	0.006	0.015	32	2047
CCTGGCATTGAGG	0.027	0.004	0.032	26	783
GGCATTGCAAGTA	0.054	0.009	0.042	62	1404
GCCAGTACATGGG	0.048	0.005	0.047	48	972
AGCGGCATTGGCG	0.022	0.006	0.013	7	513
GCTGTTGTATATA	0.056	0.01	0.043	59	1301
AGTGTCGTAAATG	0.023	0.003	0.022	27	1189
GGCGTTATAAGTA	0.057	0.008	0.05	80	1519
ACTAGCACAGACA	0.026	0.003	0.022	80	3553
CGTGTTGCAGAGG	0.02	0.003	0.024	14	571
ACTGTCACAGAGA	0.02	0.003	0.022	43	1954
ACTAGCATTGGCA	0.025	0.004	0.02	36	1807
ACTGTCGTAAGCA	0.029	0.004	0.028	46	1588
ACCAGTGCAAACA	0.02	0.001	0.019	95	4984
GGTAGCACTAGGA	0.071	0.011	0.056	33	557
ACTAGCGCATATG	0.027	0.002	0.026	42	1547
GCCAGCGTAGGTG	0.057	0.003	0.06	48	757
GGTAGTGTAAATA	0.063	0.003	0.058	85	1383
CCCATCATTTACA	0.024	0.002	0.021	93	4236
CGTGGTATTAAGG	0.03	0.009	0.031	21	650
CGCGGCATATGGG	0.019	0.009	0.025	15	589
ACCATTATTGGTA	0.023	0.007	0.017	50	2962
CGTATTACAAGCG	0.028	0.004	0.024	36	1436
CCCAGCGCATGCG	0.023	0.005	0.015	25	1628
CGTGTTACAAACA	0.027	0.005	0.02	46	2237
CGCATCATAAGTA	0.024	0.004	0.019	58	2977
CGTATTGTATACG	0.026	0.006	0.02	27	1294

GGCGGCACTAAGG	0.287	0.036	0.277	253	660
CCCGGTACATGTG	0.024	0.004	0.029	43	1465
GGCGTCACAAACG	0.07	0.012	0.053	87	1546
ACTATTGTTAGCA	0.025	0.008	0.019	45	2347
CCCAGCACTTACA	0.025	0.002	0.024	90	3650
GCCGGTACTTATG	0.078	0.002	0.08	81	933
GGCGTTGCAGATG	0.069	0.009	0.059	51	819
CCCGTTGTTGACA	0.025	0.001	0.024	51	2033
GGTATCATTTGGA	0.129	0.025	0.112	83	659
GGCAGTGTTAAGA	0.053	0.001	0.052	67	1217
CGCATCATATGTG	0.023	0.003	0.024	39	1604
GCCGGCGCAGGGA	0.481	0.043	0.473	376	419
AGCATTACAGGCA	0.021	0.003	0.018	46	2557
ACCAGTGCATGTA	0.022	0.003	0.024	65	2661
CCTATTATTTATG	0.029	0.003	0.032	72	2149
AGTGTCATATATA	0.026	0.004	0.022	45	2015
ACTATCGTTAATG	0.03	0.004	0.026	52	1957
CCTATTACAGGCA	0.025	0.005	0.018	47	2507
CGCATCATATGCA	0.029	0.002	0.031	83	2606
GGCGTCACAGGCG	0.064	0.01	0.05	46	872
CGCATTGCAGACA	0.027	0.004	0.022	51	2271
CGTGGTGTAGGGA	0.025	0.005	0.024	12	495
AGCAGCATTAGCA	0.023	0.003	0.02	50	2465
ACCGTCATATACA	0.024	0.001	0.024	100	3996
GGTGTCGTTTGCG	0.111	0.021	0.081	25	283
ACTGGCACAGGTG	0.026	0.002	0.026	22	836
ACCGGTACATATA	0.02	0.001	0.022	90	4064
CGTAGTGTAGATA	0.022	0.002	0.02	25	1211
ACTAGCGTTAAGA	0.032	0.007	0.029	49	1626
CCTATCACTTGTA	0.029	0.005	0.027	62	2254
CGTATCATATAGG	0.023	0	0.022	30	1304
ACTAGTACAAATA	0.024	0.002	0.022	123	5571
CCTATCATAGGGA	0.043	0.007	0.035	58	1588
ACCATTACTTGGG	0.02	0.003	0.016	30	1851
ACCGGCGTTAGTA	0.022	0.003	0.021	26	1214
ACCAGTACTTAGA	0.023	0.004	0.018	60	3313
CCCGGTACTGACG	0.031	0.006	0.025	42	1642
CGTGTCACAAATA	0.027	0.001	0.027	52	1861
GCTATCACTTATA	0.054	0.007	0.044	81	1745
CGCATTGCTAATG	0.026	0.006	0.022	32	1456

ACTATCGCAAAGG	0.032	0.007	0.039	69	1685
ACCATTGTTTGTA	0.026	0.003	0.023	54	2258
GCCATCATATGGG	0.064	0.009	0.053	57	1016
ACTGTTACTGACA	0.023	0.001	0.022	58	2600
CCCGTCGCTAGCA	0.024	0.005	0.019	39	2053
ACCAGCACTAGGG	0.032	0.003	0.035	46	1272
CCTAGTATTTACA	0.025	0.005	0.023	59	2483
GCTGTCGCATAGA	0.058	0.007	0.062	47	717
CCTGGTGCATGTA	0.026	0.004	0.025	33	1309
CCCATCACAGACG	0.033	0.004	0.031	94	2894
AGTATTGTATATG	0.027	0.005	0.022	34	1517
CGTGGTGTAAGTA	0.027	0.004	0.023	19	822
GGCGGCACTAACG	0.28	0.029	0.265	350	971
AGTATTACTAGCA	0.024	0.002	0.022	42	1849
AGCATTACTGAGA	0.023	0.004	0.023	55	2334
ACTATTATTGACG	0.027	0.002	0.024	63	2550
GCTAGTGTAAAGA	0.056	0.002	0.054	100	1746
AGTATTGTTTGGG	0.023	0.002	0.022	15	661
GCCGTCATATGTA	0.059	0.008	0.051	95	1761
CGTAGTGTATATG	0.027	0.002	0.028	23	811
CGCAGTGTAGAGA	0.024	0.003	0.023	32	1344
GCCATCGCATGTA	0.049	0.003	0.046	68	1396
GCTAGCGCTGGCA	0.061	0.017	0.039	29	719
AGTGTCGTAAAGA	0.022	0.002	0.024	31	1262
CCCAGTACAGATG	0.023	0.002	0.02	47	2315
AGCAGTGTTTAGA	0.019	0.005	0.016	26	1579
AGCAGCATAAATA	0.022	0.002	0.02	98	4924
AGTGGTGCAAGGG	0.024	0.014	0.04	21	501
GCCAGCGTAAGTG	0.06	0.006	0.057	60	991
AGTATCATTGATA	0.026	0.004	0.02	46	2207
AGCGTTATTAATG	0.017	0.003	0.017	36	2073
ACTGGTGTTTGGA	0.025	0.004	0.025	19	734
CCCGTCGTAAGTA	0.022	0.005	0.022	49	2188
CCCAGTACTTACA	0.028	0.003	0.029	108	3626
AGTGTTACAAGCA	0.022	0.002	0.02	33	1644
ACCAGCGTAGATG	0.025	0.008	0.013	23	1689
AGCATCACAGGCA	0.02	0.004	0.015	38	2565
ACTATCACTAGCA	0.022	0.001	0.023	72	3002
GCTAGCATAGATG	0.064	0.004	0.069	89	1199
GCCGTCGTTAAGA	0.049	0.002	0.049	59	1135

ACCATCGCTGACA	0.023	0.004	0.018	68	3640
ACTAGCATAGAGA	0.03	0	0.03	68	2217
GGCATCGTATGCG	0.107	0.017	0.128	141	960
CGTGTCATATGTA	0.021	0.004	0.015	20	1299
ACCGTTGTAAATG	0.022	0.003	0.021	51	2402
ACTAGTGCTAGGA	0.026	0.005	0.031	50	1544
CGTATTGTTTATG	0.021	0.001	0.023	23	987
CCCATCACTGGCG	0.038	0.005	0.038	79	1995
CCTATCGTAGGCA	0.02	0.003	0.017	33	1958
CCTATCGTTAACG	0.03	0.009	0.021	45	2133
ACTAGTGCATATA	0.024	0.003	0.027	89	3186
ACCATTGTAAGCG	0.026	0.004	0.021	53	2470
GCCAGTACTGATG	0.06	0.002	0.057	81	1331
ACCATCACAAATA	0.022	0.001	0.023	186	7877
CCCATTGCATACA	0.028	0.003	0.024	92	3790
GGCGTTGTAGGGA	0.059	0.006	0.055	39	672
GCCGTTGCTAGTA	0.05	0.001	0.048	48	952
CGTGGTATATGTG	0.023	0.005	0.018	11	617
CCTGTTATTTATG	0.026	0.001	0.026	40	1500
GCCGTTATAAGCA	0.052	0.005	0.059	134	2138
GGCAGTGTAAATG	0.051	0.001	0.053	87	1563
CCTATCACAAGGA	0.027	0.003	0.027	65	2371
GCTAGTATAGAGA	0.062	0.004	0.057	85	1405
ACCGGTGTAAAGA	0.023	0.004	0.024	52	2120
ACCATTGCTGGCA	0.02	0.001	0.021	55	2530
CCTGTTGCAAGGG	0.021	0.005	0.024	22	910
CCCGTCGCTTAGA	0.024	0.001	0.023	35	1502
CCTATTACATGCA	0.03	0.002	0.03	86	2829
CCTAGCGTATACA	0.022	0.003	0.021	51	2400
CCTAGCGTATACG	0.028	0.008	0.017	28	1593
CGTGGTGCAAATA	0.026	0.004	0.027	35	1279
CCTGGTGCAAAGA	0.03	0.003	0.029	49	1618
CGTGGTATAAGCG	0.021	0.003	0.018	13	713
GGCGTTATAAATA	0.063	0.012	0.061	166	2563
AGCATTACAAATG	0.022	0.002	0.021	81	3757
GGTATCACTAACG	0.134	0.022	0.124	146	1036
GCTATTGTAAGTA	0.055	0.003	0.059	109	1725
AGTATCATTAGTA	0.024	0.006	0.031	57	1773
GCTAGTACTAGCG	0.058	0.013	0.041	38	897
CCCAGTATATGTG	0.022	0.002	0.023	48	1996

GCCATTATTAGGG	0.07	0.011	0.059	74	1172
CCCGTTGTTAGGG	0.023	0.001	0.024	20	827
ACTGGTATAAACA	0.027	0.001	0.025	84	3286
CGTGGTGCATACG	0.026	0.007	0.02	14	690
ACTAGTGTAGGTA	0.022	0.006	0.015	24	1577
CCCATCATTGGCG	0.037	0.002	0.035	69	1910
ACCGTCGCAGATA	0.02	0.001	0.021	53	2469
AGTGGTGTATGTG	0.023	0.005	0.024	14	578
AGCGTTACATACG	0.02	0.003	0.023	45	1897
AGCGTCGTATGTG	0.023	0.004	0.021	24	1146
ACTGGCGCAGGCG	0.024	0.004	0.029	21	713
ACTATTATTGGCG	0.024	0.002	0.022	53	2383
GCCAGCGCATGTG	0.041	0.012	0.026	21	787
CCCAGTATAGACG	0.022	0.004	0.017	47	2760
ACTATCATTAGGG	0.029	0.003	0.029	40	1331
AGTGGCACATATG	0.024	0.004	0.022	16	706
CCTGTTACAAGCA	0.027	0.003	0.024	53	2177
CCCAGTACTGAGG	0.024	0.005	0.019	32	1667
GGCGGCACAGGCG	0.253	0.039	0.225	197	677
AGCGGTATTGAGA	0.02	0.001	0.02	29	1448
ACCAGCACAAGGA	0.026	0	0.025	75	2871
AGCAGCGCATGCG	0.027	0.005	0.029	36	1191
AGTAGCATAGGTG	0.029	0.003	0.03	28	899
CCCGGCATATATA	0.028	0.001	0.029	86	2918
ACTATTATATGGG	0.026	0.002	0.027	45	1620
GCTGGCGCTTGGA	0.63	0.044	0.572	408	305
CGTGTCGTTTACG	0.024	0.007	0.025	20	794
AGCGGTATAAGTA	0.02	0.002	0.017	39	2315
GGTAGCACTTGGG	0.063	0.009	0.054	20	353
GCTAGTATTGACA	0.062	0.004	0.062	120	1826
CCTATCATATGCG	0.022	0.003	0.019	41	2089
CGCATCGCATAGG	0.026	0.005	0.032	38	1138
CGCGGCGTAGATA	0.026	0.005	0.023	28	1174
CGTATCATATGGA	0.021	0.001	0.02	27	1296
AGCGTTACTTGGA	0.027	0.004	0.022	25	1103
CCTATTGTATGTG	0.026	0.005	0.021	32	1481
GGCAGTATTAGTA	0.061	0.003	0.057	95	1573
AGTGTTGTTAACA	0.024	0.004	0.021	36	1669
ACTATTACTAGCA	0.024	0.003	0.024	82	3310
CCTAGCGTTAGTG	0.028	0.007	0.019	19	971

CGTGTCGTAGACA	0.017	0.004	0.013	16	1228
ACCAGCACAGGGA	0.026	0.002	0.027	48	1739
AGTGGCGTAAGTG	0.028	0.012	0.018	7	388
CCTAGCATTGACA	0.023	0.007	0.022	53	2373
AGTGGCGTTAGCA	0.026	0.006	0.031	22	677
GCCATCATAGGTG	0.069	0.009	0.062	77	1172
GCCGGTGTAGACG	0.396	0.053	0.385	455	728
CGCATTATAGGCG	0.027	0.003	0.024	37	1501
CGCGTCACAGACA	0.021	0.002	0.018	44	2374
AGTATCATTGACG	0.029	0.005	0.022	32	1445
CCTGTTGCTGACA	0.028	0.004	0.023	42	1771
CGCAGTGTAAGCG	0.026	0.002	0.023	29	1228
CGCAGTACAGAGG	0.025	0.002	0.022	23	1043
GGCGGTGTTGGCA	0.059	0.013	0.047	34	697
AGTGGCATAGACA	0.021	0.002	0.023	23	985
CCCGGCGCAGAGA	0.024	0.004	0.022	34	1516
GCTGGTATTAACA	0.167	0.022	0.179	329	1508
GGTGTTACTTGTA	0.071	0.006	0.075	55	678
CGCGTCACATACG	0.024	0.006	0.017	32	1824
GGTGTCGTTTAGG	0.102	0.013	0.089	31	317
CGCGGCATAAATG	0.018	0.002	0.015	21	1334
AGCGTTGTTGGGA	0.017	0.005	0.016	14	839
CCTGGTGCAAACA	0.022	0.001	0.021	49	2314
GGTGTTATAAGCG	0.085	0.015	0.066	52	737
CCTGTCATTTATG	0.024	0.003	0.019	28	1415
ACTATTGTATATA	0.029	0.003	0.026	90	3341
GGCAGCGTAGAGG	0.058	0.006	0.059	46	736
AGCAGCATAGGGA	0.017	0.001	0.015	34	2206
ACCGTTATAAATA	0.023	0.002	0.021	106	5017
GCCGTTATTAACG	0.059	0.01	0.048	85	1688
ACTGGCGTAGAGG	0.031	0.01	0.042	18	413
ACTGTCATATGCA	0.023	0.003	0.026	53	2022
ACCGGTGCAGGCA	0.023	0.003	0.019	36	1822
ACCGGCGCATGGA	0.029	0.008	0.019	18	931
ACTAGCGCATACA	0.021	0.006	0.012	39	3092
CCTGTCGTTAGGG	0.021	0.007	0.013	9	698
GGTATCATTAATA	0.124	0.012	0.114	229	1784
CGCATCGCAGACA	0.026	0.002	0.024	53	2162
GCTATCATAGAGG	0.066	0.004	0.061	71	1088
GGCGTTATATACA	0.06	0.006	0.052	104	1888

GCCGGTGTTAACG	0.394	0.059	0.384	516	828
GGTGTTATAGAGG	0.095	0.011	0.096	54	511
CGCAGTACTAGCA	0.023	0.006	0.031	56	1747
GCCAGTGCAGACA	0.05	0.01	0.042	68	1553
AGTATCGCATATA	0.024	0.003	0.02	37	1815
GCCGTTACAAGTG	0.047	0.005	0.053	65	1157
ACTATTGCAAGTG	0.026	0.006	0.019	39	2051
GCTATCATAGGCG	0.066	0.004	0.07	92	1225
CGCATCACATACG	0.027	0.003	0.026	60	2270
GCTGTTGTTAGTG	0.055	0.009	0.044	30	653
GGCATCACTGATA	0.065	0.006	0.06	120	1877
ACCATTACATGCG	0.028	0.002	0.025	83	3233
AGCATCACTTATA	0.025	0.003	0.028	91	3104
CGCATTACAAAGA	0.025	0.003	0.023	65	2780
ACCAGTGCATGGG	0.029	0.01	0.023	30	1263
CGCGGCGCAAAGA	0.022	0.004	0.027	39	1410
GGTGGTATAAATA	0.128	0.024	0.121	194	1403
CCTGGTACTTAGA	0.03	0.004	0.032	39	1190
CCCGGCGTAAGTG	0.022	0.005	0.015	20	1290
ACTGTTGCAGACG	0.028	0.006	0.023	37	1576
AGTAGTATAAGCG	0.022	0.003	0.026	36	1351
CCTAGCATATACG	0.028	0.003	0.023	47	1983
GGCATCATAAGCA	0.079	0.012	0.07	169	2231
CCCGGTGCAAACG	0.022	0.003	0.019	36	1884
AGCGTCGCAAATA	0.023	0.003	0.023	61	2558
CCTGTTATTTGGG	0.024	0.007	0.033	27	795
GGTATCACTTAGG	0.121	0.022	0.11	70	567
GGTGTTGTTTGCG	0.071	0.007	0.078	29	342
AGTGGTGTTGACA	0.019	0.004	0.014	15	1026
GGCATCACAAGGG	0.088	0.012	0.077	91	1088
CCCAGCGTAAATG	0.02	0.003	0.017	45	2635
CCCGGCGTAGAGG	0.03	0.007	0.03	30	964
CCCGGCATTGACG	0.029	0.009	0.024	41	1672
CGCGGCATAAGGA	0.022	0.005	0.018	22	1206
GGCGTTACAAGGA	0.057	0.02	0.037	41	1072
CCCGGTATAAGTG	0.019	0.002	0.02	35	1735
CCTATTACTAACA	0.024	0.001	0.024	100	4136
GCTATTACATAGA	0.051	0.004	0.046	85	1751
GGCAGCATTTATG	0.057	0.005	0.063	79	1171
CCTGTTACAGGTA	0.024	0.004	0.018	27	1505

AGTAGTACAGGCA	0.031	0.006	0.023	30	1288
CGTATTATAAAGA	0.027	0.001	0.027	77	2785
AGTAGTGCTTACA	0.025	0.005	0.019	25	1311
CGTATCATTGACG	0.022	0.002	0.02	29	1440
CCTGGCACTGACA	0.026	0.005	0.026	49	1822
ACTGGCATATGGG	0.028	0.006	0.021	14	656
GGCGGCGCTTGTG	0.189	0.054	0.184	90	398
GCCATTGCTAAGG	0.051	0.009	0.039	45	1100
ACTGGCGTTTGTG	0.029	0.008	0.022	8	362
ACTGGTACTTGCA	0.024	0.005	0.026	44	1649
ACTGTCGTTGACG	0.03	0.006	0.024	25	1015
ACCAGCGTTGGGA	0.023	0.004	0.028	29	994
ACCGTCACAAATA	0.024	0.001	0.024	134	5509
AGCAGTATTGACA	0.022	0.003	0.018	63	3415
CCCGTTACTAATA	0.027	0.003	0.023	79	3293
CGTAGCATAGAGA	0.028	0.005	0.027	33	1182
AGTGTTGCAAATA	0.025	0.004	0.02	37	1798
ACTAGCACAAATA	0.021	0.003	0.018	91	5065
AGTAGCGTTTAGG	0.027	0.007	0.027	18	644
ACCATCACAGGTA	0.025	0.002	0.025	89	3421
AGTAGCGTTAAGA	0.024	0.001	0.025	30	1173
GCTATTGCATACG	0.057	0.008	0.062	79	1205
AGTATTACTAACG	0.027	0.007	0.021	45	2069
CCCATCACTGACA	0.03	0.003	0.027	105	3855
AGCATCGTTTGCA	0.029	0.002	0.03	60	1910
GCTGGTATAGGGA	0.13	0.007	0.132	82	539
ACTATCACAAAGG	0.023	0.004	0.027	75	2668
GGTATCACTAGGG	0.123	0.02	0.135	72	460
CGCATTACAGGCA	0.024	0.003	0.027	57	2081
CCCATTGCAGACG	0.027	0.005	0.022	55	2395
ACTGGCATTAGCG	0.028	0.009	0.017	16	952
ACTGTTATATATG	0.025	0.008	0.014	29	2007
AGCATTGCAGGCG	0.023	0.004	0.026	46	1710
GGTATTACTGATA	0.084	0.011	0.081	116	1311
ACCGGTATATAGA	0.025	0.006	0.017	43	2559
CCCATCGTAAACA	0.026	0.002	0.024	114	4652
GCCGGTGCAGATA	0.056	0.005	0.062	79	1188
ACCATTACATAGA	0.02	0.003	0.02	90	4444
CGCGGCACTGGCA	0.028	0.009	0.031	40	1237
ACTGGTGTAAAGG	0.03	0.006	0.022	24	1067

**ACCAGTGTAAGGA**	0.025	0.006	0.019	40	2061
**AGTAGCACAGATA**	0.025	0	0.025	46	1808
**CCCATTACTGACG**	0.037	0.002	0.039	101	2471
**CCTGTCGTAGGCG**	0.019	0.005	0.014	14	953
**CGTATCATTTGCA**	0.023	0.004	0.027	40	1449
**AGTGTCGCTAGCG**	0.014	0.003	0.011	8	699
**GGCAGTATTAAGA**	0.048	0.003	0.045	85	1797
**GCCGTCACATACA**	0.051	0.002	0.049	127	2477
**AGCATTATTTATA**	0.021	0.002	0.018	77	4111
**AGTATTGTTGGCA**	0.02	0.009	0.012	15	1228
**CCCAGTGTTGGGA**	0.023	0.004	0.024	26	1071
**CCTGTTGCTTAGA**	0.022	0.002	0.019	25	1275
**GCCATTGCAAAGA**	0.045	0.003	0.045	84	1789
**GCCAGCGTTAACA**	0.053	0.005	0.053	112	2021
**GGCAGCGCAAGTG**	0.057	0.013	0.047	45	922
**GCTGTCACAAAGG**	0.06	0.012	0.045	44	936
**CCCGGCACATAGA**	0.024	0.002	0.027	56	2015
**CGTATTGTTAGGA**	0.026	0.008	0.016	15	918
**CCTAGCGCAGATA**	0.023	0.004	0.018	40	2243
**GGCATTGCAAAGA**	0.053	0.007	0.05	83	1592
**ACTATCGTAGGCA**	0.027	0.003	0.028	50	1715
**ACCGTCATAGGTA**	0.021	0.003	0.017	42	2371
**ACTGTCATTAACG**	0.023	0.003	0.025	54	2074
**CCCAGCATTTACA**	0.024	0.001	0.026	90	3390
**GCCATTATATATG**	0.05	0.004	0.05	135	2578
**CGTGTCGTAAAGA**	0.032	0.003	0.028	32	1105
**CCCAGTATTAAGA**	0.028	0.002	0.03	97	3161
**ACTATTGTTTGTG**	0.033	0.004	0.029	40	1359
**ACCATCGCAGGTA**	0.024	0.003	0.022	57	2489
**CCCAGCGCATACA**	0.022	0.003	0.021	80	3763
**CGCGTTATTAATA**	0.024	0.004	0.021	53	2494
**AGTGGTACATAGA**	0.023	0.004	0.022	25	1105
**CCTATCATTTGTA**	0.027	0.004	0.027	65	2326
**CGTGGCGCTTGCG**	0.026	0.008	0.037	14	363
**GCCGGTACAAGGG**	0.08	0.007	0.072	61	791
**AGCGGTGCATACG**	0.022	0.003	0.019	24	1225
**GGCATCATTTAGA**	0.079	0.005	0.072	121	1562
**GCTAGCATAAGTA**	0.067	0.009	0.057	106	1757
**GGTGGTGTAGACG**	0.192	0.035	0.16	91	478
**AGCATCGCATGCG**	0.019	0.006	0.012	20	1669

CCCGGCATTTACG	0.029	0.005	0.024	41	1676
CCTATCATTAATA	0.023	0.002	0.019	86	4334
CGTAGTGTATGCA	0.02	0.002	0.022	20	871
GCCAGTACAAGTG	0.052	0.002	0.054	85	1478
AGCGGTGCAGGGA	0.014	0.003	0.013	10	775
ACCGGCACTGAGG	0.02	0.001	0.019	27	1378
ACCGGCACAGAGA	0.022	0.002	0.02	50	2458
GGTATCGTAGAGG	0.12	0.016	0.102	54	477
AGCGGCATAGACG	0.021	0.004	0.024	18	738
GGCAGCGTTTGTG	0.07	0.013	0.087	52	549
GGCATTACAGATA	0.063	0.009	0.055	124	2132
GGTGTTATAGACG	0.089	0.032	0.097	82	765
GCTATTGCAAATG	0.052	0.003	0.05	77	1465
CGTGGCGTAGGCG	0.025	0.003	0.025	10	397
CGCATCACTGACA	0.025	0.005	0.025	67	2654
GCCATCACATGTA	0.057	0.004	0.052	114	2092
CGCATCGTAGGCA	0.023	0.004	0.019	35	1852
CGCGTTGCTTAGG	0.029	0.002	0.03	21	682
GGTGGCATATACA	0.487	0.06	0.443	690	866
AGCAGTGCAGGTA	0.021	0.003	0.024	36	1441
GGCATCGTTTGCG	0.118	0.005	0.123	110	783
ACTGGCACAAATG	0.022	0.001	0.023	44	1864
GCCGTCACAGAGG	0.056	0.005	0.058	61	985
ACCATTACTTGCA	0.022	0.004	0.021	86	3938
GCTATCATTTATG	0.061	0.008	0.056	86	1447
CCCAGCACATGCG	0.026	0.002	0.029	63	2110
ACCGGCGCAAATA	0.026	0.003	0.026	56	2076
GGCAGCATTGACG	0.057	0.005	0.063	67	993
GCTAGTATAAGGA	0.057	0.005	0.049	72	1388
CCTGGTGTTTGTA	0.034	0.006	0.039	38	930
ACCGTCACTGGGA	0.028	0.002	0.025	32	1251
CGTAGCGCATGGG	0.017	0.001	0.019	9	464
GGTATCACAAGGG	0.116	0.01	0.119	71	528
GCCATTATATGGA	0.055	0.002	0.057	118	1966
CCCGGTGTAAACG	0.022	0.001	0.021	44	2073
CCCAGTGCATACA	0.022	0	0.023	75	3223
CCCAGTACAGGGA	0.035	0.002	0.033	56	1646
GGCATCGTTGGCA	0.091	0.014	0.075	87	1072
ACTAGCATTAACA	0.026	0.001	0.027	106	3759
CCTGGTACTTGGA	0.026	0.003	0.026	24	916

CCTGGCATTTATA	0.026	0.002	0.024	42	1726
CGCAGTATAGGCG	0.032	0.003	0.033	42	1243
ACCATTACAAATG	0.022	0.001	0.023	132	5615
ACCGGCGTTGACG	0.028	0.002	0.026	20	757
CGCATCGCATACG	0.024	0.005	0.028	49	1719
CCCGTTGTTTAGG	0.031	0.001	0.032	32	980
ACTGTTGTTGATA	0.028	0.003	0.026	46	1719
AGCAGCGCATGTA	0.025	0.007	0.024	37	1478
AGCATTGCTAACA	0.024	0.004	0.027	87	3131
CCTGGCATATGCA	0.03	0.007	0.019	31	1584
CCCAGCATTAAGG	0.025	0.005	0.025	51	1995
ACTGTTACATACA	0.024	0.002	0.023	80	3427
AGCATTGCAGGTG	0.028	0.007	0.019	25	1326
GCTGGTATTGGTA	0.167	0.008	0.158	128	681
GCCGGCATTAGTA	0.386	0.035	0.378	594	977
ACTGTTGTATGGA	0.023	0.005	0.024	29	1166
CGCGTCATAAACG	0.026	0.005	0.022	50	2224
CGTGTTATATACA	0.022	0.004	0.028	53	1874
CGCGTCGTTGACG	0.021	0.003	0.021	23	1082
CCTAGTACATGCG	0.03	0.002	0.029	42	1413
GGCAGCACAAAGG	0.049	0.002	0.048	61	1206
GCTAGTGTAAGGA	0.051	0.007	0.05	54	1026
CGCATCATAGAGG	0.031	0.002	0.034	48	1358
GCCATTATTAAGG	0.05	0.004	0.047	94	1894
CGCGTCGCTGGCG	0.024	0.005	0.031	33	1023
CCTATCGTATGTA	0.027	0.007	0.02	40	1994
CCCATTACAAGTA	0.025	0.004	0.031	120	3731
AGCGTTACAGGCA	0.025	0.001	0.023	44	1853
GGCAGCGCAGATG	0.062	0.018	0.038	36	901
AGTGTTATAAAGG	0.022	0.002	0.025	36	1431
ACCATCGTTGATA	0.022	0.001	0.023	63	2645
GGCGTTGCTTGCA	0.05	0.003	0.047	36	724
CCTGTTGTTAACG	0.022	0.005	0.029	45	1531
AGCAGCGTTAGCG	0.023	0.001	0.023	28	1199
AGTGTTACAGATA	0.022	0.003	0.026	46	1734
CCTGGCGTTGGTA	0.027	0.011	0.024	23	952
ACCAGTGCAAGTG	0.021	0.007	0.012	25	2072
ACCATCATTTACG	0.022	0.001	0.022	76	3449
CCTGTCGTTAATG	0.024	0.006	0.023	32	1387
CGCGTTATTAAGA	0.026	0.006	0.02	37	1857

**CCTGGCATAGATA**	0.024	0.001	0.023	45	1936
**ACCGGTATATGCA**	0.022	0.004	0.02	50	2429
**ACTATCACTAACG**	0.029	0.002	0.027	87	3144
**AGCAGTGTAAACG**	0.023	0.001	0.023	59	2552
**ACTGTTATTGGCA**	0.025	0.002	0.026	43	1604
**GCCAGTATTTGCG**	0.053	0.008	0.043	63	1396
**CCTGTCATTAATA**	0.026	0.005	0.026	70	2651
**AGTGGTACTAGTA**	0.023	0.004	0.028	29	1016
**ACTAGCGCTGGTA**	0.03	0.003	0.027	40	1421
**CCCAGCATAGGCA**	0.028	0.001	0.027	75	2695
**ACCAGCACATATA**	0.023	0.005	0.017	96	5481
**AGTAGTACTAGTA**	0.026	0.001	0.025	36	1388
**CCCAGCACTGGTA**	0.026	0.001	0.027	56	1986
**AGCATTATTGGCA**	0.022	0.002	0.025	64	2546
**CGTATTGTAGACA**	0.023	0.007	0.014	25	1745
**CGCAGCACTTAGA**	0.026	0.006	0.018	25	1400
**CGTGGCGCTAAGA**	0.025	0.003	0.023	18	751
**ACTGTCATAGATA**	0.029	0.004	0.029	73	2479
**AGTATCACATGGA**	0.026	0.006	0.017	19	1078
**CCTAGCGCATATG**	0.025	0.004	0.026	33	1258
**AGCGGTATTGGGA**	0.022	0.008	0.01	10	965
**GGCAGTGTTTGTA**	0.049	0.003	0.052	50	905
**AGCATTACTAATA**	0.02	0.002	0.021	91	4274
**CCCATTGCAAGTA**	0.026	0.002	0.024	75	3006
**CCCGGCGTAAAGG**	0.024	0.007	0.014	20	1366
**AGCGGTATAAGCA**	0.025	0.002	0.027	64	2328
**AGTGTTATTAGCA**	0.022	0.002	0.022	34	1494
**GCCATTATAGGTA**	0.051	0	0.051	125	2342
**CCCATTATTAGCA**	0.025	0.005	0.021	77	3606
**CCTATCACTTGCA**	0.027	0.002	0.028	67	2325
**AGCAGTATATACA**	0.018	0.002	0.016	73	4427
**CCTGTCACAAAGA**	0.025	0.003	0.022	50	2240
**GGCATTATTAATA**	0.061	0.01	0.054	168	2937
**CCTGGTGCTAGCA**	0.02	0.003	0.022	30	1335
**GCCAGTGTAGATA**	0.048	0.005	0.047	87	1775
**GCCGTCGTAGGCA**	0.041	0.009	0.042	45	1015
**CGCAGTACATAGG**	0.023	0	0.023	29	1256
**CCCGTTACTGAGG**	0.028	0.006	0.021	29	1351
**GCCGTTGTTGACA**	0.045	0.005	0.038	57	1428
**AGCATTACTTACA**	0.023	0.002	0.026	91	3422

AGTGTTGCTTACA	0.021	0.001	0.021	22	1038
ACCATTATTAACA	0.025	0.001	0.024	153	6349
ACTGTCATAAACA	0.029	0	0.03	111	3617
CGTGTTGCAGACA	0.024	0.005	0.023	28	1169
CCTAGCGTTGAGG	0.028	0.002	0.029	28	947
CCCGTCGTAAGCA	0.02	0.001	0.019	47	2461
GCCATCGTTAGTG	0.053	0.001	0.051	57	1064
CGCGTCATTGACA	0.025	0.004	0.028	59	2054
ACCAGCATATGTA	0.026	0.006	0.027	91	3250
CCCAGCACAGGCA	0.025	0.003	0.022	62	2805
ACCGTCGCTGAGG	0.023	0.005	0.029	33	1104
AGCGGCGCAAATA	0.022	0.003	0.023	49	2088
GCTATTGTAGAGA	0.064	0.004	0.061	91	1409
CCTGTCATTTATA	0.025	0	0.024	50	2007
ACTAGCGCTAGCG	0.026	0.004	0.022	28	1234
AGTGTCGCTTACA	0.023	0.008	0.014	16	1139
AGTAGTACATAGG	0.027	0.005	0.03	33	1055
ACTAGCACAAGTG	0.029	0.003	0.031	59	1828
ACCAGCATAGGGA	0.027	0.005	0.025	44	1702
CGCGTTATTAACG	0.025	0.004	0.019	35	1786
CGCGGCGCTAGGA	0.026	0.002	0.023	20	835
GCTGTTATTAAGG	0.066	0.001	0.068	66	904
ACTGGCATAAGGG	0.028	0.009	0.025	21	812
AGCGTTATATGTG	0.028	0.001	0.028	43	1486
CGTATTACTGGCA	0.028	0.004	0.025	34	1331
CGCGTCACTTACA	0.024	0.003	0.02	41	2040
AGCAGCACTGACG	0.026	0.008	0.014	31	2137
GCTATCATAAGCA	0.061	0.004	0.056	125	2116
GCTAGTATAGGTA	0.064	0.007	0.055	77	1326
GCTAGCGCTAATG	0.049	0.01	0.037	31	814
ACCAGTGCTTAGG	0.026	0.003	0.028	39	1375
GCTATCATAAACG	0.065	0.004	0.061	144	2230
AGCGGTGCAAGGA	0.028	0.004	0.023	26	1092
GGCATTATAAACA	0.054	0.003	0.05	199	3746
ACCAGCATTGACG	0.025	0.004	0.028	66	2256
CCCGTTGCATAGG	0.032	0.005	0.032	40	1220
AGCATTGTTTGCA	0.024	0.002	0.025	48	1901
AGCAGCACATACG	0.02	0.001	0.021	47	2242
CGTATCGTTGAGA	0.022	0.005	0.017	19	1077
GCTAGTGCTGAGG	0.055	0.006	0.048	31	618

GCCATTATAGACA	0.056	0.003	0.057	215	3546
CCTGGTATTAGTA	0.027	0.002	0.027	39	1388
CCCATCATATATA	0.026	0	0.025	121	4639
CGTATCATAAAGG	0.026	0.003	0.027	47	1678
ACTGTCATTGAGG	0.024	0.006	0.019	22	1115
CCCGTTGTAAGTA	0.027	0.003	0.023	47	2013
CCTGGTATATGTA	0.028	0.004	0.026	42	1589
CCCAGCATTGATG	0.027	0.005	0.028	61	2094
GCCGGTGTTTAGG	0.414	0.063	0.381	283	460
ACCAGCATTAGCG	0.022	0.002	0.023	53	2212
AGCATCGCAGGTG	0.025	0.01	0.012	15	1236
AGTATCACTAACA	0.023	0.003	0.022	51	2289
ACTGGCATTAAGA	0.023	0.003	0.022	40	1807
CGTAGTGTAAGTA	0.025	0.002	0.022	25	1102
CCTAGTACATAGG	0.03	0.004	0.034	50	1413
CGCGTCATATATG	0.025	0.004	0.022	34	1513
ACCGTTACTGACA	0.023	0.002	0.022	89	3941
CCCAGTATTGGCA	0.022	0.001	0.023	56	2349
ACTATCGTTAGCA	0.026	0	0.026	52	1951
AGCATTACTAACG	0.023	0.004	0.019	58	3033
CGCAGTACTAGTA	0.034	0.001	0.033	55	1613
CCCGGCGTAGGTG	0.023	0.002	0.022	21	920
CGCAGCGCATATA	0.025	0.008	0.034	59	1665
GCCATCGTAAGTA	0.054	0.008	0.049	91	1765
ACTAGTGCTTAGG	0.026	0.004	0.02	25	1215
CCTGGCATAGGGA	0.038	0.01	0.026	26	969
ACCATTACAGAGA	0.018	0.002	0.02	84	4169
GCCGTCGTAAAGG	0.059	0.01	0.045	40	841
ACCGTTGCAGGCG	0.023	0.005	0.019	29	1490
ACCATTATTTATA	0.024	0.001	0.023	114	4806
GCCAGCGCTTATA	0.059	0.004	0.061	78	1198
AGCGGCATATGCG	0.023	0.005	0.029	18	593
ACCGTTGCAGAGG	0.028	0.005	0.021	30	1391
ACCATTGCAAAGG	0.025	0.003	0.029	82	2783
CCCGGCGTTTAGA	0.023	0.003	0.022	30	1341
GCCGGTACAGAGG	0.073	0.006	0.064	57	833
GGTATCGCAAGTG	0.144	0.02	0.123	85	607
AGTAGCGTTTATG	0.027	0.006	0.03	26	831
CGTATTGTATGCG	0.023	0.008	0.034	34	964
ACTAGCACAAACG	0.023	0.003	0.022	66	2959

GGTATTATTTGCG	0.117	0.018	0.091	69	686
ACCGTCGCAAAGG	0.023	0.005	0.017	29	1686
ACCAGTACATGCA	0.022	0.002	0.025	110	4325
CCCGTCGTTAAGA	0.02	0.001	0.02	41	2001
ACCGTCATTGAGA	0.021	0.002	0.022	49	2216
ACCGTTGCTTGTG	0.03	0.003	0.031	38	1169
ACTGTTATATGCG	0.023	0.002	0.019	28	1410
CGCAGTGTAGACA	0.024	0.006	0.018	36	1958
CCCATTATTAATG	0.02	0.003	0.015	55	3548
GCCATTACTGATA	0.047	0.007	0.05	119	2265
ACCAGCACTGACA	0.025	0.001	0.023	89	3726
GCTAGTGTAAGCG	0.054	0.004	0.056	61	1025
ACTATTATAGATG	0.025	0.002	0.027	78	2864
CCTGTTGTTGACG	0.025	0.002	0.024	30	1242
ACCAGCATAGATA	0.023	0.002	0.021	86	4086
AGCAGCGCTGATA	0.023	0.006	0.015	26	1704
AGTGTCGCAGGGA	0.03	0.005	0.033	21	610
GGCATTGTAGAGG	0.081	0.009	0.071	63	828
CCCGTCATAAACG	0.024	0.004	0.021	66	3052
CCTGGCGTTAACA	0.024	0.006	0.017	32	1884
ACCAGTGCATGCG	0.021	0.004	0.018	36	1919
GGTATTGTAAACG	0.112	0.018	0.092	119	1180
AGCGTCATATATG	0.019	0.003	0.019	39	1987
ACTAGCATTGACG	0.03	0.007	0.023	39	1668
ACTATCGCTAGCG	0.028	0.005	0.03	46	1476
CGCGGCACAAGCA	0.029	0.004	0.034	64	1811
AGCAGCATATGTA	0.021	0.004	0.016	43	2650
ACTGGTGTATGCA	0.021	0.005	0.014	19	1346
CCCATCACTGAGG	0.029	0.003	0.029	55	1822
ACCGGCGCAGACG	0.021	0.003	0.016	25	1512
ACCATTGCAAAGA	0.022	0.003	0.017	67	3854
CGCGGTATTAACG	0.022	0.003	0.026	41	1562
CCTATCGTAAATA	0.023	0.004	0.018	64	3503
CCCATTACTAATA	0.026	0.001	0.025	122	4714
GCCGGTACTAAGA	0.058	0.006	0.052	77	1416
CCTATTGCTTATA	0.025	0.002	0.023	56	2363
AGCGGTGCATGGG	0.031	0.01	0.032	21	637
GCCGGCACATGGG	0.23	0.016	0.22	164	582
CCCGGCATTAGGG	0.032	0.004	0.026	24	892
ACCAGTGTATATA	0.024	0.003	0.02	61	2988

GGCAGCGCTTGGG	0.068	0.012	0.072	33	425
CGTATTGCTAACG	0.025	0.007	0.016	20	1253
ACCATCACAAAGG	0.026	0.004	0.021	71	3305
CCCAGCATTTAGA	0.028	0.002	0.027	62	2244
GCTAGCACAGGTA	0.069	0.008	0.079	84	976
GCTATTACTGACG	0.06	0.008	0.049	71	1368
AGTGTTACAGGTA	0.022	0.006	0.029	33	1102
AGTAGTGCTAGCA	0.027	0	0.026	30	1113
CGCGTCGTAGAGA	0.021	0.001	0.022	26	1176
CCTAGTATAAACG	0.028	0.003	0.024	73	2956
ACTGGCGTTGGCA	0.025	0.002	0.027	24	854
CCCGTCGCTGATG	0.02	0	0.021	29	1375
ACCGGCACTAATA	0.024	0.002	0.022	80	3555
GGCGTTATTGGGA	0.067	0.003	0.065	50	724
GCTATTATTAGGG	0.072	0.014	0.06	58	909
ACCGGCGTTTGGG	0.03	0.005	0.023	10	428
AGCATCGTTAAGG	0.021	0.003	0.017	28	1623
ACTGTTACTAACA	0.022	0.001	0.022	76	3309
AGCATCGTAAGGG	0.02	0.002	0.02	30	1479
ACCGGCGTTGGGG	0.021	0.011	0.036	14	372
ACTGGTATTGAGG	0.025	0.007	0.022	20	899
CGTATCACTAAGG	0.025	0.005	0.032	33	1013
CCTGTTACTGATA	0.024	0.005	0.028	54	1874
CGCATTGTAAGCA	0.023	0.004	0.018	42	2251
CGCATCACAAATA	0.029	0.004	0.024	95	3803
CGCAGCGCAAACA	0.024	0.001	0.025	66	2536
ACTATTGCAGATG	0.026	0.001	0.026	58	2177
CCTAGTACAAGCG	0.027	0.003	0.03	52	1696
ACCGTCGCTTATA	0.022	0.001	0.022	49	2163
CCCATCGTATGCA	0.023	0.003	0.026	72	2691
GGTATTATAGATA	0.105	0.007	0.103	206	1788
CCCGTCGTTTGTA	0.023	0.006	0.025	38	1488
CCCAGCATATGTA	0.03	0.005	0.024	67	2771
GGTATTGTTAGCA	0.072	0.021	0.047	46	932
AGTAGCATAGGTA	0.032	0.005	0.026	35	1330
ACCGGCATAAGCA	0.024	0.002	0.023	69	2950
CGCATCACAGGGA	0.028	0.005	0.032	42	1278
CGCGGCGCAAACA	0.024	0.003	0.024	49	1997
CGTGGCGTAAATG	0.022	0.008	0.011	8	731
ACTGGTATAGGGA	0.025	0.003	0.026	26	992

ACCGGTACAGAGG	0.02	0.006	0.017	33	1887
GCTATTATATGGA	0.055	0.006	0.051	77	1420
AGCATCACTTACA	0.025	0.003	0.029	96	3245
ACTATCACAGATA	0.028	0.005	0.021	80	3657
CGTGGTGTTGACA	0.02	0.002	0.023	21	907
ACCATCATTAACA	0.025	0.001	0.024	147	6000
ACCAGCACTTATA	0.021	0.001	0.022	103	4502
AGTGTTATTGACA	0.025	0.001	0.027	45	1623
ACTATCACATACA	0.026	0.003	0.022	103	4592
CCCATCGCATATA	0.025	0.002	0.025	86	3396
GGTGGTACTGGCA	0.089	0.013	0.072	43	556
AGCATTACAGACG	0.021	0	0.02	57	2724
CCTAGTATATGCA	0.028	0.005	0.022	48	2130
CCCAGTGCTGACA	0.024	0.003	0.02	51	2482
GCTATTATAAACA	0.063	0.004	0.059	226	3635
ACCAGCATTGACA	0.023	0.003	0.02	79	3799
ACTATTATAGGGG	0.032	0.006	0.036	43	1139
CGTGTCATAGACA	0.018	0.002	0.015	25	1618
GGTAGCATTTACA	0.072	0.018	0.055	62	1059
ACCAGTACAAGGA	0.022	0.002	0.025	90	3542
AGCGTTGTAGGCA	0.027	0.002	0.024	36	1462
CCCGGCACTTACG	0.026	0.004	0.021	40	1872
CCTAGCGCTAGCA	0.023	0.002	0.023	38	1643
CCTAGCACTGGTA	0.023	0.003	0.021	31	1413
AGCGTTGTATAGG	0.023	0.003	0.022	25	1121
CCTGTCACAGGGG	0.049	0.012	0.06	47	741
GCCGTTGCTTGCG	0.049	0.01	0.045	30	633
CGCAGCATATACA	0.022	0.003	0.021	65	2984
CCTGTTATAGGGG	0.033	0.009	0.04	35	834
CCTGTTGTAGGCA	0.021	0.004	0.015	22	1402
GCTATTATTTAGA	0.05	0.005	0.048	87	1735
GGCATCATTTATA	0.084	0.009	0.083	188	2077
ACCATCACAAACA	0.023	0.001	0.021	179	8373
AGTGTTATAAGGG	0.023	0.005	0.027	26	945
ACTGTCGCATGGA	0.023	0.003	0.021	24	1146
GCTGTTACATATA	0.055	0.004	0.061	106	1642
GGTATTACATGCG	0.104	0.014	0.111	98	784
GCTAGTACAGATA	0.06	0.002	0.059	105	1681
GCTATTGTTGAGA	0.062	0.005	0.059	71	1131
CGCATTACAAATG	0.028	0.002	0.027	75	2698

GGCATTGTTGGTA	0.076	0.017	0.058	59	952
CCTAGCGCAAGTA	0.023	0.005	0.018	40	2187
CCCATCATAGACA	0.025	0.002	0.027	134	4757
AGCGTTGTTGACA	0.021	0.003	0.018	35	1878
AGTATCGCATGTA	0.029	0.007	0.026	35	1291
CGTGGCGTTAGCG	0.024	0.002	0.023	10	421
ACCGGTGTAGACA	0.022	0.003	0.02	46	2277
GCTATTGCTAATA	0.05	0.003	0.046	91	1880
CGTAGCGTAAAGA	0.022	0.008	0.014	18	1293
ACTAGTGCTAGCG	0.026	0.005	0.022	32	1437
GCCGGCGCTAGCA	0.498	0.03	0.483	640	685
CCCATTATTAAGA	0.029	0.003	0.025	100	3911
CGCAGTGTATGGG	0.026	0.004	0.02	15	735
CCTGTTATTAGTG	0.02	0.002	0.018	21	1153
AGTAGTGCTAATG	0.028	0.003	0.028	29	1002
CCCATTACAGGTG	0.029	0.006	0.021	39	1835
CGCAGCGTAGATA	0.024	0.001	0.022	40	1763
AGCATCGCAGGTA	0.023	0.004	0.017	29	1671
CGCGTCGTATGCA	0.026	0.008	0.019	26	1358
ACCAGCGCAGACA	0.022	0.002	0.024	99	4067
AGCGTCACAAGCA	0.021	0.003	0.016	38	2351
CGCATTACTTGGG	0.027	0.007	0.034	31	877
CCCAGTATAGAGA	0.024	0.002	0.021	61	2798
CCTGTCACTAGGG	0.029	0.005	0.032	27	813
GGCAGCGTTGGCA	0.05	0.009	0.042	39	888
ACCATCACTGACG	0.025	0.002	0.024	64	2598
CCCAGCGTAAGCA	0.02	0.002	0.017	48	2776
CCCATTGCAAGGA	0.027	0.006	0.02	43	2131
CCTATCATTTGTG	0.027	0.005	0.021	35	1670
CCCAGCATTAGTG	0.021	0.003	0.02	36	1806
GCCGTCGCTAGTA	0.057	0.004	0.063	57	855
GGCAGTATTAGGA	0.049	0.007	0.041	52	1230
GGTAGTACATATA	0.067	0.008	0.06	87	1362
AGTATTGTATGGA	0.02	0.002	0.02	24	1176
ACTATTACAAGTA	0.026	0.003	0.021	83	3821
AGTAGTACAGATG	0.023	0.006	0.015	19	1255
GCCATCACATATA	0.052	0.008	0.042	128	2909
GCCGGTACAGGGA	0.052	0.005	0.047	41	839
ACCAGCACTGAGA	0.022	0.004	0.017	54	3059
AGCATCGCATATG	0.023	0	0.022	43	1876

AGCGTTACTTGCA	0.026	0.001	0.025	43	1686
GGTGGTATTGGGG	0.265	0.084	0.248	78	237
GCCGGTGCTGGGA	0.055	0.011	0.069	37	496
AGCGTCATATATA	0.023	0.004	0.022	68	2996
GCCATTGCTGATA	0.041	0.007	0.034	59	1670
GCCATTGCATATG	0.05	0.005	0.044	60	1295
ACTAGTGCAAGTG	0.026	0.002	0.024	41	1697
ACCATCGTATGCA	0.026	0.004	0.02	56	2723
AGCGGTGCAGATA	0.02	0.001	0.021	31	1481
ACTGGCGTTTGTA	0.025	0.005	0.019	16	837
CGTAGCACAAAGG	0.03	0.006	0.021	23	1055
CCTAGCATTAATG	0.027	0.007	0.018	36	2006
CCCATTACAAGCA	0.027	0.002	0.026	110	4157
AGCAGCATTTAGG	0.02	0.005	0.026	40	1506
CCCGTCACTGGTG	0.02	0.006	0.026	32	1216
CGTGTTATATAGA	0.026	0.001	0.028	38	1314
GCTATTATAAGCG	0.064	0.003	0.064	108	1587
ACTATCATATGGA	0.026	0.002	0.024	50	1999
CCCATCATTTAGG	0.025	0.004	0.028	55	1879
GGCGGCATTAAGG	0.559	0.062	0.538	596	511
GGCGGTACATATA	0.06	0.012	0.049	77	1479
AGTAGCGCTGATG	0.028	0.007	0.018	15	796
CCTATCGCATGTG	0.032	0.011	0.021	27	1252
AGCAGTATAAGTG	0.021	0.002	0.019	43	2281
AGTAGCATAAGTA	0.026	0.001	0.025	50	1966
ACCAGTACAAAGG	0.022	0.002	0.024	82	3366
GGCATCGTAGAGG	0.115	0.018	0.109	98	797
ACCGTTATTTGTA	0.024	0	0.023	52	2167
AGTATTACAGGGA	0.025	0.002	0.023	28	1182
AGTATCGTAAACG	0.022	0.001	0.021	39	1778
CGTGTTGTAGGTG	0.027	0.007	0.024	12	497
AGCAGCGTAGGGG	0.02	0.006	0.023	27	1169
ACCAGTACATGGG	0.015	0.002	0.014	26	1801
ACCGTTGTTAATA	0.026	0.002	0.023	65	2715
ACTATCATTGATA	0.027	0.002	0.026	85	3225
AGCAGTATTTGTA	0.02	0.004	0.018	42	2345
AGTATCGCATACA	0.022	0.005	0.022	41	1865
AGCGGCATTGATA	0.025	0.002	0.024	37	1503
ACCAGTGTAAAGA	0.025	0.001	0.023	69	2930
AGTATCGCTGGCG	0.023	0.001	0.023	23	977

GCCGTTGCAGAGA	0.044	0.006	0.04	38	908
AGTAGTGTAAATA	0.026	0.006	0.018	44	2422
CCCAGTGTAAGCA	0.021	0.001	0.022	57	2560
CCTGTTACAGGTG	0.03	0.006	0.034	36	1022
ACCGTTACAAGCA	0.023	0.001	0.021	89	4159
CCTGTCGTTTACA	0.022	0.002	0.023	42	1751
GGTATCACTTGGA	0.084	0.016	0.067	45	630
CCTGGCACTAATG	0.024	0.003	0.029	39	1312
CGCAGTATTTATG	0.024	0.004	0.024	39	1559
GCCATCGTTTGTA	0.049	0.005	0.045	59	1261
CCTAGTGTAGGGG	0.025	0.004	0.025	19	756
CGCATTGCTGGGG	0.026	0.003	0.031	20	627
ACTAGCATATAGG	0.026	0.004	0.025	42	1642
AGCATTGCTAATA	0.022	0.002	0.024	70	2866
CCCGTCACATAGA	0.026	0.003	0.024	58	2395
ACTAGTATTAATA	0.024	0.001	0.025	105	4086
GCTGTCGCTGAGG	0.06	0.007	0.052	23	417
CGCAGCACAAGTA	0.023	0.003	0.025	54	2122
AGCGTCACTTATA	0.021	0.004	0.016	38	2296
GCCGTTACAAGTA	0.057	0.009	0.059	114	1833
CCTGGTATTAGGA	0.025	0.006	0.024	27	1080
CCCATTGCAAACG	0.022	0.005	0.016	55	3324
GGCGGCATAGGCA	0.3	0.059	0.259	319	913
GGTGTTACTTATG	0.087	0.016	0.066	45	639
CGTGTCGTATGCG	0.027	0.001	0.027	18	639
GGTAGCACAAACA	0.063	0.009	0.052	92	1678
CCCGTTATAAATA	0.025	0.002	0.023	105	4550
CCCGTTGCTGACG	0.021	0.002	0.019	30	1562
CGTGGCGTATGTA	0.023	0.008	0.02	13	629
CCCGGTATATAGA	0.025	0.004	0.021	47	2157
AGCGTCGCTAATA	0.02	0.005	0.015	29	1908
GGTAGCACAAGTG	0.078	0.02	0.051	35	646
AGTATTATTTGCA	0.019	0.003	0.017	34	1986
AGTAGTGCATGGG	0.022	0.002	0.02	11	550
CGCAGTATTGACA	0.026	0.005	0.022	54	2395
ACTGTTACAAGGG	0.031	0.009	0.026	31	1169
CGCATTGCTGGTG	0.024	0.003	0.019	17	861
CCTAGCGCTAGTA	0.024	0.005	0.018	30	1599
GCCATTATTAACA	0.05	0.001	0.049	217	4172
GGTGTCGTAAATG	0.109	0.025	0.099	79	719

ACTGGTGCATGCA	0.024	0.003	0.027	40	1423
CCTATCACTTATA	0.025	0.003	0.023	76	3218
CCTATCGCAAACA	0.025	0.002	0.022	87	3829
AGCAGTATAAATG	0.022	0.003	0.025	93	3584
GGTATTGCAAGGG	0.094	0.013	0.079	46	533
AGCATTGTTGGGA	0.02	0.005	0.013	17	1289
CGCGGTACTGGCG	0.024	0.001	0.025	20	772
ACTGGCGCTTGGA	0.031	0.007	0.035	16	447
GGCAGCACAAGGG	0.05	0.008	0.045	43	918
CGTGTTATATGCG	0.015	0.001	0.014	13	897
CGCGTTATTAAGG	0.02	0.003	0.017	20	1157
AGCAGTATTAGGA	0.021	0.002	0.023	45	1952
AGTGTCATTAACG	0.021	0.009	0.014	20	1439
ACTAGTACTAGTA	0.018	0.001	0.019	53	2790
GCCATCACTTGTA	0.053	0.007	0.055	100	1709
ACCATCATTTGCA	0.023	0.003	0.018	62	3413
CGTAGCACTAACA	0.022	0.004	0.022	40	1763
AGCAGCGTATGCG	0.023	0.005	0.018	25	1332
AGCGTTATAGAGA	0.02	0.003	0.019	39	2008
ACTATCATTAACA	0.025	0.005	0.019	85	4396
CCCAGCATAGACA	0.031	0.005	0.024	97	3912
GCCATTATTAACG	0.049	0	0.049	140	2700
CGTATTGTATGGG	0.021	0.008	0.013	9	692
ACCAGCATAGAGG	0.026	0.002	0.025	40	1579
ACCATCGCATGTA	0.019	0.002	0.021	68	3200
GCCGGTGTTTACG	0.41	0.091	0.35	369	684
CCTATCACAAATG	0.026	0.005	0.02	68	3288
ACCGGCATAAGGA	0.026	0.001	0.025	50	1936
AGTATTACATATG	0.022	0.003	0.022	44	1915
AGCATTATTAAGG	0.021	0.005	0.015	35	2342
CGCAGCACTTGTG	0.022	0.006	0.017	16	919
AGTATTATAGGCG	0.021	0.003	0.017	27	1540
CCCGTCGCAGAGA	0.022	0.001	0.021	38	1799
ACCATCACTTATA	0.022	0.002	0.019	97	4953
GCCGTTATAGAGG	0.059	0.009	0.049	59	1155
GGTATTACAAGTG	0.108	0.02	0.083	87	963
GGTAGTATTAACA	0.065	0.007	0.057	92	1520
GGCGTCACAAACA	0.062	0.004	0.061	149	2303
GGCAGCATTTGCA	0.053	0.001	0.053	70	1256
CCTGTCATATACA	0.023	0.007	0.013	35	2634

CGCGGTGTAAGCG	0.028	0.008	0.022	20	907
CGCATCGCTGATG	0.03	0.004	0.03	41	1321
GCTGGCATTGACG	0.857	0.016	0.846	1334	243
GCTGGCACAGATG	0.455	0.042	0.447	450	556
GCCATCATTGAGG	0.055	0.005	0.056	78	1318
CCCAGCACTAGGA	0.029	0.003	0.025	46	1807
ACCGGCATAAACG	0.024	0.001	0.024	65	2628
GGTGTTATTAGTA	0.071	0.002	0.068	66	898
GCTAGTGCAAGTA	0.056	0.007	0.046	62	1285
AGCAGCACTAGCG	0.025	0.004	0.029	43	1430
CGTATCGTAAGTA	0.022	0.004	0.018	30	1611
CGCAGTGTAAGTG	0.023	0.006	0.018	21	1176
CCTGGCGCAGACA	0.017	0.004	0.012	19	1605
GCCATCGTAAATG	0.052	0.005	0.045	95	2010
CGTATTACTTACA	0.022	0.002	0.022	45	1986
AGTGGTGTTTGTG	0.027	0.004	0.026	12	449
CGTAGCGTAGATG	0.035	0.002	0.038	30	764
GCTATCGTTGACG	0.057	0.01	0.052	60	1104
CCCGGTGCTTACG	0.029	0.006	0.021	26	1199
CGTGGCATTGACG	0.021	0.003	0.022	15	657
AGCGTCACATGTA	0.021	0.002	0.019	36	1903
AGTAGTATAAGGA	0.024	0.007	0.015	26	1667
ACCGGTGCAGATG	0.026	0.002	0.023	32	1370
GCCGTCACAAAGA	0.058	0.005	0.059	131	2071
GGCGTTGCAGACA	0.053	0.009	0.041	51	1203
GGCGGTACATGTG	0.076	0.005	0.076	53	645
GCCATCGTAAGCA	0.056	0.008	0.064	134	1952
GCTGTCATTTGCA	0.06	0.012	0.043	43	961
ACTATCGTAGATG	0.025	0.006	0.016	28	1694
CCCAGCATTAGCA	0.022	0.003	0.023	70	2944
GGTAGCGTATGCA	0.068	0.005	0.061	47	726
CCTATTGCATACA	0.026	0.002	0.023	78	3283
GCCAGCACAAGCG	0.047	0.001	0.045	73	1545
AGCAGCACTGGCG	0.027	0.002	0.025	35	1355
GGCGTTACTGACA	0.065	0.005	0.06	96	1492
CGTGGTGTTTATA	0.026	0.008	0.036	27	717
GCCAGTATAGGTG	0.054	0.009	0.041	54	1277
ACTGGCGCATACA	0.025	0.004	0.03	35	1135
CGTAGTGTTTAGA	0.028	0.003	0.027	18	661
ACCAGCACATGTA	0.021	0.002	0.019	75	3915

CGCAGCATTTGGG	0.032	0.009	0.024	18	731
AGCAGCATATACG	0.02	0.003	0.023	59	2460
GGCATCACAAATA	0.07	0.009	0.066	210	2989
ACCAGTGTATGTG	0.031	0.002	0.032	48	1451
CGCAGTGTTTAGG	0.021	0.01	0.012	9	766
ACTGTTATTTGCA	0.024	0.004	0.029	49	1617
ACTATTACTTAGA	0.028	0.004	0.024	72	2933
ACTGGCACTGGTA	0.026	0.005	0.024	29	1199
GCCAGTATAAACA	0.05	0.003	0.053	246	4412
ACTATTGTATACG	0.028	0.002	0.026	62	2335
ACCGTCACATGCA	0.02	0.001	0.019	66	3398
CGTAGTGCTAACG	0.024	0.004	0.019	16	846
ACTGGCACTGATA	0.024	0.001	0.023	41	1739
ACCAGTACTAACA	0.022	0.001	0.022	139	6257
GGCATCGTAAAGG	0.121	0.018	0.097	131	1213
CCTGGTATAAACA	0.022	0.001	0.023	70	2967
GGCAGCGCTAAGA	0.044	0.007	0.038	41	1048
AGTGTCATAAGCG	0.02	0.006	0.02	22	1065
ACTAGTGTTTATA	0.026	0.006	0.022	55	2397
ACTATTGCAGACG	0.023	0.002	0.023	55	2341
CGCGTTGTTGGCA	0.024	0.001	0.023	25	1040
GCCGTTGTTTAGA	0.041	0.001	0.043	45	1010
CGTATTGCAAATG	0.032	0.006	0.026	36	1371
CGTATTACATATG	0.023	0.004	0.016	26	1555
GGTATCGTAAGCG	0.136	0.021	0.107	76	632
GCTAGCACAAGGG	0.062	0.003	0.058	43	701
CCTGGTGTTGGCA	0.024	0.008	0.016	16	984
CCCAGCACAGAGA	0.021	0.001	0.021	55	2536
GCCATCGCATACA	0.041	0.001	0.04	85	2065
ACTGTCGCATGCG	0.03	0.011	0.046	54	1129
GGCAGCGCATGGA	0.063	0.007	0.065	51	735
GGCGTTATTGAGG	0.053	0.013	0.049	37	713
CGTAGTACAAGTG	0.024	0.002	0.021	20	947
GGCATCGCTGGCA	0.085	0.01	0.073	77	982
ACTATTATATGGA	0.026	0.003	0.022	54	2422
ACCAGCATTTATG	0.023	0.004	0.019	48	2452
CGCATCGCAAATG	0.025	0.002	0.023	46	1980
GGCAGTACTTGTA	0.051	0.006	0.043	54	1188
AGCGGCGCATAGG	0.024	0.008	0.035	28	773
GCTATCACTAGGA	0.059	0.005	0.064	67	982

GCCAGTATAAGCG	0.048	0.002	0.046	90	1858
GCCATTATTGGGG	0.062	0.007	0.066	51	726
CCTGTCGCTTGCG	0.019	0.007	0.026	20	742
GGTAGTGTAGGCA	0.052	0.003	0.049	36	695
CCCAGTACAGGCA	0.025	0.001	0.025	75	2960
GCTGGTGCTTACA	0.106	0.009	0.097	84	778
GCCAGTGCTTATA	0.036	0.011	0.043	61	1361
GGTAGTATAAGGA	0.064	0.007	0.059	62	994
CCCGGTACTTGTG	0.03	0.001	0.03	38	1237
CCCATTGTTAACA	0.025	0.002	0.021	80	3662
ACCATTACTGATG	0.022	0.003	0.024	83	3346
AGCAGTGTTGATG	0.024	0.005	0.016	24	1431
CGCATCGCTTGCG	0.023	0.006	0.016	18	1086
GCTGGCACTGACA	0.215	0.014	0.204	261	1018
GGCGTCGCAAGTG	0.074	0.005	0.069	55	740
GGCGGTGTAGACG	0.093	0.013	0.083	71	783
CCCGGCGTTTGTA	0.018	0.005	0.016	21	1324
GCCATCACAAACA	0.05	0.006	0.043	186	4171
GGTATTGCATGCG	0.086	0.011	0.075	44	539
CCCATTGTAAATG	0.03	0.001	0.032	95	2856
ACTGGTATAGGGG	0.032	0.002	0.032	19	569
AGCGGCGTAGAGA	0.022	0.001	0.02	20	973
CCTGTCGCATACA	0.025	0.002	0.025	56	2190
CCCAGCATTTATG	0.027	0.003	0.032	71	2178
AGCGTTGCAAGGG	0.021	0.004	0.016	15	915
ACTAGTGCTTATA	0.026	0.004	0.02	51	2533
AGCATTATAGGTA	0.018	0.002	0.02	62	3018
ACCAGCACTTGGG	0.022	0.005	0.018	22	1201
ACCGGTACTGGCG	0.022	0.002	0.019	27	1419
ACTAGCGCTGAGA	0.02	0.003	0.02	32	1572
CCCGGCATTTATA	0.025	0.003	0.029	72	2442
CGTGTCATAGGGG	0.034	0.002	0.034	16	455
GCTAGCACTAATA	0.068	0.014	0.052	105	1918
AGCATCACTAAGG	0.029	0.001	0.029	63	2073
ACCATCGCAGGCA	0.026	0.003	0.022	67	2974
GGCAGTGTATAGG	0.051	0.003	0.047	41	827
CCTATTACTTACA	0.024	0.003	0.021	71	3267
ACTGTTATTAACA	0.026	0.003	0.022	75	3304
CGCGGCGTATGTA	0.026	0.002	0.024	24	984
CCTATCGCAAAGA	0.027	0.002	0.026	67	2490

ACCATTGTTTGCA	0.026	0.001	0.024	56	2275
CCCAGCACAAAGA	0.027	0.003	0.03	112	3669
ACCAGCACTAGTA	0.022	0.001	0.022	77	3479
ACCAGCACAGATA	0.021	0.001	0.021	103	4787
GGTATTATAGATG	0.115	0.028	0.091	121	1211
ACCGTTACTAATG	0.02	0.002	0.019	61	3097
CCTATCGCAAAGG	0.023	0.004	0.021	35	1652
ACCAGTATAGGTA	0.022	0.002	0.024	73	2911
AGCATTGCAAAGA	0.021	0.004	0.02	54	2707
GCTGGTACTAATA	0.084	0.007	0.075	107	1321
CGCGTCATATAGA	0.028	0.002	0.03	53	1685
GGTAGTGTTAGCA	0.056	0.011	0.059	43	680
AGCGGTGTTAGCA	0.025	0.009	0.016	20	1248
GCTGGCACAGGTG	0.481	0.063	0.487	350	369
GCCATTGTATACA	0.046	0.005	0.041	106	2469
CCTATCATTTGGA	0.023	0.006	0.017	28	1636
ACCATCACAGACA	0.021	0.001	0.023	133	5669
ACTAGCACAAATG	0.024	0.001	0.022	66	2877
AGCATCATTTATA	0.022	0.003	0.018	66	3645
GCCAGTACTAGCA	0.045	0.005	0.05	100	1891
CGCAGTACATGGG	0.019	0.005	0.013	12	921
CCTGTCGCAAACG	0.03	0.001	0.031	53	1643
ACCGTCGCAAGCG	0.021	0.004	0.017	31	1779
AGCATTATAAGCA	0.021	0.003	0.024	105	4198
CCCGGCGCATATG	0.022	0.005	0.029	45	1512
CGTAGCGTTTACG	0.034	0.006	0.027	21	761
CGCAGTGCTGACG	0.029	0.005	0.029	33	1123
AGCGGTATTTGTG	0.017	0.005	0.012	11	906
CCTGTCACTGACA	0.019	0.003	0.016	37	2291
CGTGGCATATGCA	0.023	0.007	0.021	20	931
ACTGTCACTTGTA	0.02	0.007	0.013	22	1666
AGCGTTGTAGACA	0.021	0.002	0.017	38	2134
AGCATCGTTAGGG	0.02	0.004	0.017	20	1137
GGTATCGCAAATA	0.107	0.017	0.097	143	1335
CGCAGTATATACG	0.022	0.001	0.022	46	2041
ACTAGTGCTGGCG	0.024	0.004	0.019	27	1371
ACTGGCGTTTGCA	0.03	0.004	0.029	25	848
GCCGTCATAAGTG	0.05	0.008	0.048	69	1379
CGTGGTGCATATG	0.021	0.004	0.016	11	679
GCCATCACTAGTA	0.047	0.01	0.043	89	1967

CCCAGCGTAAACG	0.024	0.001		0.022	71	3096
CCTGGCATAAACA	0.024	0.003		0.02	58	2866
AGCAGTGTAAAGA	0.022	0.004		0.027	73	2592
ACTGGCGCAAACA	0.026	0.006		0.024	36	1464
AGTGTCGTTTGGA	0.019	0.005		0.019	11	577
ACCAGTATAAGTA	0.025	0.001		0.024	103	4239
GCTGGTACTTGCG	0.096	0.01		0.109	66	542
GGTAGTATAAAGG	0.075	0.012		0.08	80	920
AGTATCGCAGAGA	0.02	0.007		0.025	28	1072
GCCATTATAAACA	0.054	0.002		0.052	292	5296
CGCAGCATAAGTA	0.02	0.005		0.018	43	2410
ACCGGTGCAAGTA	0.024	0.005		0.03	70	2247
CCTAGCGTTTGCG	0.021	0.007		0.014	14	972
CCTATCGCAAGTA	0.024	0.001		0.023	53	2270
GCTGGCATTGGTG	0.862	0.019		0.84	755	144
ACCATCGTTGACA	0.024	0.003		0.022	68	3011
AGCATTATAAATA	0.021	0		0.022	153	6899
ACCAGTATTGGTA	0.017	0.001		0.016	41	2492
GCTGTTATAAATA	0.06	0.004		0.061	157	2403
GGCATTATTAGGG	0.052	0.006		0.045	44	944
GCCAGTACTTGCA	0.053	0.002		0.055	91	1557
CGCATCGTTGACA	0.024	0.006		0.017	39	2220
ACTGTTATTAACG	0.025	0.001		0.025	53	2035
GGCGGTGTATGTG	0.099	0.01		0.085	50	538
CCTGGCACAAACG	0.024	0.001		0.026	49	1837
GGTATCGCTGGCA	0.096	0.014		0.08	57	652
CCTGTCGTAAGGA	0.028	0.008		0.026	34	1292
AGTAGTATATGTA	0.019	0.002		0.016	30	1822
CGCGTTACAAATA	0.026	0.003		0.023	70	2964
CGCAGCATAGATA	0.027	0.003		0.024	58	2387
ACCATCATTAGGA	0.024	0.003		0.02	54	2638
AGTAGCATAGATA	0.02	0.001		0.021	45	2113
ACTATCATTGGGG	0.029	0.004		0.024	24	982
AGCATCGTTGGTG	0.027	0.005		0.025	29	1118
CCCATCGCAGACA	0.024	0.002		0.025	92	3558
GGTAGTGTTTGGA	0.056	0.018		0.056	25	425
CGTGGTACTGGTG	0.03	0.001		0.028	12	415
GCCAGCATAGATG	0.051	0.004		0.053	105	1887
AGTGGCGCAAGCG	0.025	0.003		0.021	13	600
ACCATTATTAACG	0.026	0.004		0.021	95	4386

CCTAGCGTTGACA	0.025	0.005	0.018	35	1964
ACCAGTGCTTATG	0.017	0.002	0.018	35	1915
ACTATCACTGAGA	0.022	0.004	0.024	61	2446
CCTGGTATTGAGA	0.027	0.002	0.025	32	1245
CCTGGTGTTTACA	0.022	0.002	0.024	37	1512
GGTATCGCTGAGA	0.109	0.024	0.091	65	651
CCCGGTATTGGTA	0.024	0.007	0.019	28	1458
ACTAGCGCATGTA	0.02	0.003	0.02	40	1999
CGTAGCATAAATA	0.027	0.001	0.028	72	2474
GGCGGTATATACA	0.075	0.006	0.068	120	1634
AGCAGTACTTATG	0.02	0.003	0.025	50	1942
AGTAGTGTTGGCA	0.018	0.005	0.017	16	953
AGCATTACTAGCA	0.023	0.004	0.021	60	2827
GCTATTATTTGTA	0.061	0.004	0.056	97	1642
AGTATTATTAGCG	0.024	0.008	0.018	27	1515
ACCATCACTGACA	0.026	0.002	0.025	109	4337
AGCAGTACAAGTA	0.022	0.002	0.024	70	2868
CCCATCGTTGAGG	0.026	0.005	0.019	29	1512
GCCATCATAAGCA	0.057	0.005	0.05	159	3017
AGTAGCACTAGCG	0.027	0.005	0.034	32	921
AGCATTATATACG	0.023	0.003	0.019	61	3219
ACCGGCACTAACA	0.024	0.003	0.027	118	4192
GCCATCACAGACA	0.051	0.002	0.048	143	2855
ACCGTCACTAGGA	0.02	0.003	0.016	36	2213
AGCATTGTAGGGG	0.019	0.002	0.017	22	1276
GCCATTGCATACA	0.043	0.002	0.043	97	2136
CCCATTGTAGGGA	0.025	0.003	0.023	34	1471
AGCGTCACAGATA	0.022	0.004	0.026	65	2394
AGCATCGTTAACA	0.022	0.002	0.022	75	3395
CCTGGCATAGAGA	0.022	0.007	0.021	31	1429
ACCATTGCTAGGG	0.022	0.001	0.021	33	1528
GCCGTCATATATA	0.045	0.002	0.042	101	2303
CGCAGTGCAAAGA	0.027	0.004	0.033	57	1654
AGTGGCGCAGGTA	0.015	0.004	0.016	10	627
CCCAGCACAGGTG	0.025	0.003	0.029	48	1595
AGCATTACTTAGG	0.023	0.002	0.026	44	1661
ACCATCACAGACG	0.03	0.003	0.033	102	2992
GCCAGCGCTGATG	0.05	0.003	0.049	42	811
GCCATTACAAGTG	0.049	0.007	0.056	103	1728
ACCAGTGCAAAGA	0.025	0.003	0.024	82	3289

CGCGGTACAGGGG	0.027	0.008	0.036	22	596
CGTATTATAAATA	0.024	0.002	0.022	84	3800
CCCGTTACTAACG	0.026	0.005	0.021	51	2367
ACCATCACAAGTG	0.025	0.002	0.028	91	3166
CGCGTCGCTTACG	0.025	0.003	0.024	26	1072
ACCATTATAAGCG	0.021	0.003	0.023	86	3578
CCCGTTATAAACG	0.022	0.003	0.019	62	3271
CGCGGCACTTGGA	0.026	0.007	0.018	16	851
AGCGTCGTAAAGG	0.021	0.002	0.021	29	1358
ACCGTCACAGAGA	0.021	0.002	0.022	65	2828
GCCGGTGCAAAGA	0.07	0.015	0.055	70	1207
ACCGTCGCATACA	0.021	0.002	0.019	61	3133
AGTGGTACAGACA	0.025	0.005	0.021	32	1481
CGTGTTACAGGTG	0.03	0.001	0.031	22	684
ACCATCGCTGATA	0.021	0.002	0.023	74	3180
ACCAGCGTTAATA	0.025	0.003	0.028	79	2775
AGCATTGTATAGG	0.025	0.004	0.03	46	1511
ACTGGCACTGAGA	0.023	0.001	0.023	31	1336
ACTGTTATAAAGG	0.029	0.004	0.033	65	1876
ACTGGCGTATGCA	0.026	0.005	0.021	22	1050
ACCGGTGCTAGGA	0.025	0.001	0.027	38	1394
GGCATCACTGACG	0.083	0.001	0.083	131	1446
ACCATCACATATA	0.022	0.002	0.023	140	5946
AGTGTCACTAAGA	0.024	0.009	0.015	20	1278
AGTATCATTTGCG	0.026	0.001	0.027	29	1040
AGTATCATAAAGA	0.025	0.003	0.021	54	2537
CCCATTATATACG	0.027	0.004	0.022	86	3766
CCCGGTGTATGCG	0.026	0.006	0.019	21	1082
GGTATCATAAGTG	0.127	0.02	0.112	102	807
GGTGGCGCTAAGA	0.177	0.051	0.146	90	527
ACCAGCATTTGCA	0.022	0.004	0.027	82	2904
CGTGGTATAGATG	0.025	0.008	0.015	12	774
ACTAGTGCATAGA	0.021	0.005	0.017	39	2283
CCTGGTACAAATG	0.029	0.002	0.029	49	1639
AGTGGTATTAATA	0.024	0.002	0.022	38	1669
GGTAGCATTGATG	0.058	0.005	0.057	41	677
CGTGGCGCTGACG	0.023	0.005	0.029	18	594
AGCATCGCTAAGA	0.024	0.003	0.021	47	2163
CGTAGTGTATACA	0.027	0.005	0.025	35	1360
GCCGTTATAGAGA	0.056	0.005	0.051	91	1693

CCCAGTGTTGATA	0.026	0.002	0.024	54	2184
CCTAGTACATGCA	0.025	0.006	0.019	44	2233
CGCGTTACTAGCA	0.022	0.003	0.019	33	1694
AGCAGTATTAATA	0.021	0.003	0.016	71	4246
AGTAGTATTGGGA	0.028	0.004	0.023	22	942
ACCAGTGCTGATA	0.026	0.001	0.025	73	2805
CCTAGCATTGGTA	0.028	0.004	0.022	31	1376
GCCATTACATGGA	0.059	0	0.059	97	1542
CCTGGTGTTGAGA	0.027	0.008	0.015	16	1055
ACTATTGTAAACG	0.031	0.002	0.033	102	3000
CGCATCGCTAGTG	0.033	0.009	0.023	26	1110
ACTAGTACAGATA	0.02	0.001	0.02	77	3782
ACCGGCATATAGA	0.025	0.003	0.028	68	2396
GCCATCGTTAGGA	0.039	0.011	0.035	41	1124
CGTGTTATAGGCG	0.021	0.003	0.025	22	871
CCCAGCATAAAGG	0.027	0.004	0.025	61	2391
ACCGTTGTAAGCA	0.023	0.006	0.03	71	2285
AGTGTCATAAATA	0.027	0.003	0.023	59	2526
ACCGTTGCTTGCG	0.021	0.001	0.022	30	1361
CCCAGCATAGAGG	0.036	0.003	0.034	57	1611
GCTGTTGTATACG	0.054	0.016	0.039	41	1002
GCCAGCACAAATA	0.054	0.005	0.052	171	3147
AGTGTCATAGACG	0.021	0.003	0.023	29	1207
GGTGGTACTGATA	0.081	0.021	0.057	50	826
CCTATTGTTGACA	0.024	0.002	0.026	69	2580
GCTATTGCTAAGA	0.052	0.008	0.052	73	1334
CCTATTGTAGATA	0.024	0.003	0.02	58	2809
GCTGGCACTGAGA	0.209	0.029	0.194	167	695
GGCAGCATAGGCG	0.046	0.006	0.046	54	1112
ACCATTACATGTA	0.02	0.002	0.017	79	4522
AGCGTTATATAGA	0.02	0.001	0.02	46	2226
AGCGGCATAGGGG	0.031	0.005	0.024	20	821
ACCATTATAGGCG	0.024	0.002	0.021	59	2699
AGCGGTGTAAACA	0.021	0.001	0.02	51	2490
GCCATTACAGGCA	0.06	0.011	0.052	104	1913
CCTATTGTAGAGG	0.026	0.005	0.027	40	1441
CCTATTATTGACA	0.026	0.001	0.025	83	3229
CGCGGTATATATG	0.025	0.006	0.021	29	1323
GGTAGTATAAATG	0.071	0.014	0.057	80	1334
CCTAGTGCAGGGA	0.026	0.007	0.017	19	1102

AGTGTTGCAAGTA	0.023	0.001	0.024	28	1160
AGTGGTATTGAGG	0.015	0.005	0.009	5	548
ACTGGCATAGGTA	0.028	0.007	0.018	25	1360
GGTATCGTTGGCA	0.12	0.005	0.115	82	629
ACTGTTATATATA	0.022	0	0.022	74	3288
ACCATTGCTTACG	0.024	0.002	0.021	54	2576
CCCGGTGCATAGA	0.025	0.007	0.015	24	1608
AGCGGTACAGATA	0.025	0.003	0.021	45	2084
ACTGTCGCTTAGA	0.026	0.006	0.027	32	1152
AGCATTGCTGACA	0.02	0.004	0.016	42	2547
GGCGTCACATACA	0.062	0.002	0.061	127	1955
ACTATTGCAGATA	0.024	0.003	0.022	73	3174
CCTATTATAGACA	0.027	0	0.027	111	3933
AGTATCACTGAGG	0.03	0.006	0.028	25	869
CCCGTTGTATGTA	0.025	0.001	0.025	45	1726
ACCAGCACTAGTG	0.021	0.004	0.023	54	2249
ACTAGTGTATATA	0.028	0.001	0.027	77	2771
CGCATCATATATA	0.025	0.002	0.022	74	3342
ACCGTTGTTTACA	0.022	0.002	0.02	43	2160
CCTGTCATAGGCG	0.027	0.003	0.031	38	1204
CGCAGTATAAAGA	0.025	0.001	0.024	72	2944
ACCATCATAAACG	0.026	0.003	0.025	134	5250
GCTATCACAAAGA	0.064	0.003	0.063	120	1788
CGTAGCACTGATG	0.02	0.001	0.021	19	880
CCCATCATTAACA	0.025	0.005	0.019	102	5198
CCTGTCGCTGGCA	0.023	0.008	0.028	33	1151
GCCATTATTAAGA	0.052	0.003	0.048	152	3014
CGTATTATTTATG	0.032	0.005	0.035	53	1459
GGTAGCACAGACA	0.086	0.009	0.083	101	1113
GGCAGTGCTTAGG	0.05	0.015	0.047	29	590
GGCATCGCAGGCG	0.086	0.005	0.084	95	1030
CCTGTCGCATAGA	0.024	0.002	0.021	30	1416
AGCGTCGCAAGGA	0.025	0.001	0.027	32	1161
CGCGGTATAAACG	0.025	0.002	0.027	50	1773
GCCATCATATGTA	0.05	0.004	0.047	124	2531
ACTAGCACTTATA	0.023	0.003	0.019	62	3223
ACCGTCGCAAATA	0.023	0.002	0.021	74	3469
GCCGGCATTAGCA	0.394	0.032	0.393	653	1010
GCCAGCGTATGCG	0.06	0.012	0.056	59	1000
CGCGTCATAGACA	0.029	0.001	0.032	82	2521

AGTGGCGTTTACA	0.023	0.006	0.015	12	768
AGTATCATATACA	0.023	0.003	0.019	57	2963
ACCATCATAAATA	0.024	0.001	0.023	163	6974
CCCGGTGTAGACA	0.025	0.003	0.022	44	1970
AGCGTCGCTTGGG	0.016	0.003	0.02	13	648
GGTGTCACAAGGA	0.087	0.014	0.079	54	627
GGCGTTATATGCG	0.054	0.008	0.05	49	923
CGCGTTACTAGCG	0.026	0.003	0.022	25	1090
CCTATTGTTGGGA	0.025	0.003	0.027	31	1131
GCCGTTACAAGGG	0.056	0.009	0.058	56	906
AGCATTACTGATA	0.019	0.001	0.018	57	3073
CCTGTTGTTTAGA	0.03	0.003	0.027	33	1168
CGTATCGCTAATA	0.023	0.001	0.024	41	1669
AGTATCGTATAGA	0.021	0.003	0.02	31	1482
CGTATCGTATGCA	0.026	0.005	0.02	30	1453
ACCATTGTATATG	0.028	0.002	0.026	65	2414
ACCATTATAGACG	0.023	0.003	0.02	77	3765
GCCAGCATAGAGA	0.058	0.006	0.05	102	1938
CGTGTTACAGGTA	0.029	0.005	0.033	35	1041
AGCGGTATTTACA	0.021	0.002	0.023	50	2140
CGTAGCGCTGAGA	0.028	0.003	0.028	20	698
GCCATCGTTTATG	0.048	0.009	0.036	42	1138
AGTAGCATTTATG	0.02	0.005	0.013	17	1293
ACTGGTGTTTATG	0.032	0.003	0.032	29	874
AGCGGTATAAAGA	0.024	0.004	0.024	59	2358
GCCAGTATTGACA	0.051	0.002	0.054	152	2643
CGCAGTACTTAGA	0.032	0.004	0.03	47	1538
CGCGTCGTAAATA	0.027	0.003	0.024	55	2264
CCTGTCGCTAGGA	0.021	0.001	0.019	20	1015
CCCGTTACATGCG	0.025	0.01	0.011	21	1823
ACTGGCGCTTGCA	0.026	0.009	0.017	11	626
GCTATTGCTTGTA	0.052	0.008	0.042	40	911
CCTGTTGTAGACG	0.022	0.004	0.019	29	1514
ACTATTATAGGCG	0.029	0.005	0.023	57	2416
ACCAGTATAAATA	0.024	0.001	0.023	166	6904
CCCATTATTGACG	0.028	0.006	0.022	61	2762
CCCATTGCTTGTG	0.027	0.004	0.022	31	1364
GGCAGCGCAAAGG	0.062	0.006	0.053	51	905
ACCATTGCATACG	0.021	0.003	0.024	77	3159
ACCGTTATTAGCG	0.023	0.003	0.021	37	1764

ACTATCACAAGCA	0.023	0.002	0.02	74	3606
AGCGGTATAGACG	0.019	0.006	0.018	27	1489
GGCATCATTTAGG	0.097	0.022	0.082	93	1042
ACTAGTACAAAGA	0.022	0.003	0.018	73	3933
ACCAGTGCAGACG	0.022	0.002	0.025	64	2455
ACCAGCACATGTG	0.021	0.001	0.022	54	2402
CGCGTCGTAAACA	0.022	0.004	0.026	64	2441
ACCGTTATAGGTA	0.023	0.003	0.024	57	2272
CCTGTCACATATA	0.026	0.003	0.024	59	2387
AGCATTGCTGATA	0.019	0.004	0.015	36	2324
GCCGGTGTAGAGA	0.162	0.044	0.151	158	888
GCTGGTGCTTGCG	0.291	0.004	0.289	141	347
GCCGGTATAGATG	0.161	0.022	0.16	225	1184
CCTAGTGCAAGTA	0.025	0.001	0.025	56	2214
AGCGTCGTAAACA	0.02	0.002	0.018	53	2896
GGCATTATATAGG	0.072	0.012	0.062	86	1309
AGCGGTACAGGGA	0.019	0.004	0.016	17	1028
ACTAGCACAAGTA	0.018	0.001	0.018	58	3199
CCTATTACAAAGA	0.023	0.002	0.021	78	3565
AGCAGCACAAAGG	0.024	0.004	0.021	42	1928
ACCGTCACAAATG	0.023	0.004	0.022	72	3244
GGTAGTGCAGACA	0.055	0.011	0.041	39	905
ACCGTTGCTGGCG	0.028	0.006	0.036	55	1467
GCCAGCATTAATA	0.05	0.004	0.045	148	3119
CCTGGCGCTAATG	0.022	0.003	0.018	19	1059
AGCAGTGTAGGGA	0.019	0.004	0.014	20	1454
CGTATCGCAAGTA	0.021	0.003	0.024	34	1375
CCTGTTGCAAGCG	0.021	0.003	0.018	24	1312
CGTATCATAGACG	0.026	0.003	0.024	42	1705
GCTAGCACAGATA	0.067	0.007	0.068	111	1525
AGCAGTGTATATA	0.019	0.003	0.016	44	2642
GGTGTTGTATGGA	0.088	0.023	0.061	31	478
CGTATCACTGGTA	0.024	0.008	0.014	14	995
CCTGGCACATGTG	0.032	0.005	0.038	35	888
ACTGTTACAAGTG	0.024	0.004	0.019	31	1583
ACCGTTACTTACA	0.021	0.002	0.021	83	3935
GCTGGTACAAACG	0.115	0.016	0.106	139	1176
ACTGGCGTTGACA	0.029	0.001	0.028	37	1282
CGCATTATAGACG	0.023	0.002	0.024	53	2137
AGCGGCATTAATA	0.021	0.005	0.015	29	1971

ACCGGCGTTAGTG	0.026	0.003	0.023	14	597
ACCGTTACTGGCG	0.028	0.004	0.024	39	1606
AGCAGTATAGAGG	0.02	0.003	0.018	32	1707
CGCATCACTGGCA	0.022	0.001	0.023	42	1777
ACCGGCACATAGA	0.02	0.001	0.019	50	2526
CGTATCATAAAGA	0.023	0.002	0.025	67	2578
GGCGTCACTTATG	0.058	0.017	0.044	41	891
CGCATTATTTGGG	0.028	0.006	0.023	23	989
ACCGGCATTAAGG	0.024	0.004	0.019	28	1425
CGCAGTACTTGTG	0.03	0.009	0.019	18	921
CGCATTACTGGTG	0.022	0.002	0.024	26	1046
CCCATCATAAGGA	0.033	0.003	0.036	105	2824
ACCGTTATTTGTG	0.025	0.004	0.021	32	1473
AGCGGTGCTGACG	0.027	0.003	0.023	26	1112
AGTAGTGTAGGCG	0.019	0.005	0.013	11	823
GCTATTGCTAGCG	0.056	0.007	0.047	44	899
CCCAGCGCAAAGA	0.02	0.001	0.02	65	3129
ACTATTGTTTACA	0.026	0.003	0.024	72	2904
GCCAGCACTGACG	0.056	0.003	0.055	80	1371
CGTATCACAGATA	0.025	0.006	0.027	47	1718
GCCGGTATTTGTA	0.082	0.007	0.077	100	1194
CCCAGCATTAACG	0.027	0.002	0.025	76	2948
GGCGGTGTAGGCA	0.065	0.005	0.059	48	759
AGCATCACAGGCG	0.019	0.003	0.016	37	2279
GCCGGTACTGGTA	0.056	0.004	0.051	54	1009
GCCGTCATTTACA	0.057	0.007	0.048	102	2043
ACTAGCGCTTACA	0.02	0.003	0.018	43	2297
CGCGTTACATGGA	0.027	0.004	0.03	39	1255
GCCAGTACAGACA	0.052	0.004	0.049	125	2449
CCCGGCGTTAACA	0.021	0.004	0.016	42	2658
CGCATCGTTTGCG	0.025	0.006	0.024	29	1167
AGCGTTGTAAATA	0.021	0.002	0.019	57	2955
AGCGGCGTTGGTA	0.022	0.006	0.017	14	799
GCCGGTATAGGTG	0.144	0.009	0.133	125	813
ACTATTGCTAGGA	0.021	0.001	0.023	42	1824
CCTGGCACTTGCA	0.025	0.001	0.023	31	1294
AGCGTTGTAGATG	0.023	0.003	0.02	27	1332
ACTGGCACATATG	0.027	0.003	0.025	35	1385
CGTGTTACTGACA	0.019	0.001	0.02	28	1393
ACTGGCATTAGCA	0.026	0.011	0.022	36	1627

GCCAGTACTAACG	0.053	0.005	0.05	98	1864
ACCATTACTTACA	0.021	0.002	0.018	107	5759
ACCAGTGTAAGTG	0.029	0.005	0.03	56	1795
ACTGTCATTTGTG	0.03	0.012	0.019	19	991
ACCATTGTAAACA	0.023	0.001	0.025	133	5279
CGCGTCGTATGTG	0.023	0.005	0.015	14	904
CGCATCATTAGGG	0.033	0.01	0.02	21	1045
AGCGTTATTGACG	0.024	0.004	0.024	41	1655
AGCGTTGTAGGCG	0.022	0.002	0.023	26	1105
CCTGTCGTTAATA	0.027	0.004	0.021	41	1953
GCTAGCGCAGAGA	0.065	0.011	0.05	41	785
AGTGGCGCTTATG	0.024	0.001	0.025	12	472
GCCAGTGTTGGGG	0.04	0.011	0.028	14	492
CCCGGCGTAAATG	0.022	0.004	0.016	29	1835
GCCGGTATTTATG	0.158	0.019	0.154	186	1021
GCTGTTATATACA	0.064	0.003	0.061	124	1894
GCTGGTACATACA	0.082	0.008	0.078	114	1356
GCTATCACAAATA	0.054	0.006	0.049	140	2702
CGCAGCATATAGA	0.027	0.002	0.025	49	1926
AGCATTGTATATA	0.023	0.001	0.022	74	3366
GGCGGTACTGAGA	0.062	0.009	0.054	49	860
CCTGTCGCTGAGA	0.024	0.005	0.03	34	1097
CGTGTTACAAGGG	0.026	0.015	0.011	9	786
CGTATTATAGACA	0.025	0.002	0.025	68	2683
ACCAGCATAGACA	0.023	0.003	0.022	103	4526
GGTGTTGCTGGCG	0.075	0.006	0.076	33	402
CCTATTACTTAGA	0.026	0.005	0.032	72	2189
ACTATTACTTATG	0.029	0.002	0.031	87	2712
CGTATCATAAGCA	0.025	0.003	0.022	50	2195
ACCAGTATATAGA	0.02	0.002	0.023	90	3853
ACCGTTACTTGGG	0.032	0.002	0.032	40	1225
AGCATTACTGAGG	0.021	0.003	0.017	26	1529
CGTATTATTTAGA	0.025	0.006	0.016	28	1683
CGCAGTGCAAAGG	0.027	0.006	0.028	34	1179
AGTGTCATAGACA	0.026	0.001	0.027	50	1777
GCCAGCACATATA	0.057	0.012	0.043	113	2499
CCTGTTGCTAACA	0.027	0.006	0.03	70	2280
GCCAGTATAGATG	0.056	0.005	0.052	104	1895
GCCATTGCAGACA	0.043	0.003	0.043	88	1968
CGCGTTGCTGGCA	0.027	0.008	0.017	18	1057

GGCATTGTAAATA	0.067	0.006	0.062	167	2523
AGCATTGCAAGGG	0.02	0	0.02	29	1407
GCCGTTACAAATG	0.055	0.006	0.051	95	1759
ACTGGCATTGGCG	0.03	0.007	0.027	23	818
CGCATTGCAAACG	0.026	0.001	0.027	58	2124
AGCGGCACTGGCA	0.028	0.004	0.025	32	1268
CGCATTATTGACA	0.024	0.003	0.023	66	2830
CCTGGCACATATA	0.027	0.004	0.029	58	1976
GCCATCGTTAGGG	0.051	0.01	0.064	48	701
GGCATTACTTGTG	0.075	0.008	0.078	80	941
GGCAGCGCTGACA	0.063	0.006	0.06	84	1306
CGTGTCGTTAAGA	0.022	0.006	0.017	16	946
GGCGGTACATGGA	0.074	0.015	0.073	62	788
CGCAGCGCTTACA	0.025	0.004	0.02	32	1595
ACCAGCGCTGAGA	0.019	0.005	0.026	64	2418
CCTGTCGTTAGTG	0.018	0.006	0.014	13	896
ACTAGTACTGAGG	0.024	0.003	0.02	30	1480
ACTATTGCTTAGG	0.024	0.002	0.021	29	1372
ACCGTCGTTTGTA	0.025	0.003	0.023	32	1351
ACCGTCACTTATG	0.024	0.003	0.023	50	2093
AGTGGTGTAGGTA	0.025	0.005	0.024	18	747
CGCGTCATTAGTA	0.027	0.008	0.015	25	1632
GCTGTTGTTTAGG	0.061	0.008	0.062	38	578
ACTAGCGCAAAGA	0.02	0.003	0.024	61	2506
GCTGTTACAAGGA	0.054	0.013	0.038	40	1019
GGCGGCATAAACA	0.294	0.048	0.271	660	1777
AGCGTTGTAAGCA	0.023	0.002	0.025	48	1906
CCCAGTATTAACA	0.021	0.002	0.02	97	4640
GCTGGTGCTTACG	0.302	0.028	0.302	212	489
CGTATTGTAAAGG	0.022	0.003	0.023	28	1172
ACCGGTATAAGTG	0.02	0	0.019	36	1813
CCCAGTGTTGACA	0.024	0.004	0.022	54	2410
AGCAGTATTTAGG	0.022	0.003	0.026	44	1623
CCCATCACAAACA	0.027	0.002	0.024	156	6311
CGTGTCACAAACA	0.029	0.001	0.027	56	2010
CCCAGTACAAACA	0.026	0.003	0.023	129	5431
GCTAGTACAAAGA	0.073	0.007	0.067	122	1700
AGCATCACAGATG	0.027	0.002	0.025	64	2462
CCCGGCGTTAAGA	0.02	0.006	0.012	21	1725
GGCAGCGTTGATA	0.063	0.001	0.065	87	1247

GCTATCACTAGGG	0.055	0.008	0.06	48	747
GCTAGCATAGGTA	0.06	0.011	0.044	56	1217
AGTAGCGTATGGA	0.022	0.002	0.024	21	850
GGTGGTACATACA	0.081	0.011	0.066	69	978
CGCATCATTTACG	0.024	0.004	0.021	41	1959
GCCGGTATATGGA	0.082	0.026	0.071	85	1116
AGTAGCATTTGTA	0.024	0.003	0.02	28	1339
CGCATCATTAACG	0.022	0	0.022	59	2626
CCTGGCGCATGGA	0.023	0.003	0.019	16	833
ACCATTGTTAACA	0.026	0.002	0.027	116	4122
AGCATCGCATACA	0.023	0.003	0.02	59	2944
ACCAGTACAAACG	0.025	0.001	0.025	125	4965
AGCGGTGCTTGTA	0.025	0.002	0.023	22	930
GCTATTATTAGCA	0.063	0.007	0.055	113	1935
CCTATTATATACA	0.027	0.001	0.025	111	4269
AGCATTACAAGTA	0.021	0.001	0.019	69	3511
CCTGTCATAGGCA	0.024	0.006	0.017	30	1752
CCTATTACTAGTG	0.026	0.007	0.022	37	1675
ACTAGCACTTACA	0.021	0.002	0.021	68	3100
CCTGTTACAAATA	0.024	0.004	0.022	72	3227
ACTAGTACAGGTA	0.021	0.002	0.023	55	2339
ACCGTTGCAGGCA	0.023	0.002	0.021	42	1967
AGCGGCATTAGCA	0.023	0.002	0.022	29	1310
CGCGGTGCAAACG	0.025	0.004	0.023	32	1343
GGCATTACTTACA	0.062	0.007	0.054	119	2098
CCCAGTGTAAATG	0.026	0.002	0.028	73	2502
GGTGGTGTAAGCG	0.205	0.024	0.185	92	406
CGCAGTACAAGTA	0.025	0.001	0.023	52	2182
AGTAGTATAAGCA	0.022	0.004	0.018	41	2205
ACTATCATATAGG	0.033	0.007	0.026	56	2133
CCCGTTGTAGGTA	0.024	0.001	0.026	37	1407
CCTATTACTGGCA	0.029	0.006	0.02	43	2118
CCCGGTATTGGGA	0.029	0.003	0.026	27	1031
CCTAGTACAAGTA	0.025	0.002	0.027	67	2456
CGCATTGTTAGTA	0.023	0.004	0.019	31	1579
CCTATTACAAGCA	0.025	0.004	0.023	78	3289
CCTATCATTAACA	0.027	0.003	0.025	111	4381
ACTATCACAAATG	0.024	0.001	0.025	94	3662
ACTATCACTGGTA	0.022	0.002	0.019	44	2219
GCTATCGTATGGG	0.065	0.008	0.055	37	636

CCTATCGTAAGGG	0.027	0.004	0.023	31	1306
AGTAGCGTTTATA	0.028	0.005	0.022	27	1218
GCCATCGCATAGA	0.045	0.007	0.046	65	1352
CCCGGCATAAACA	0.025	0.001	0.024	105	4219
CGCATCGTAAGGA	0.022	0.003	0.018	29	1602
ACCATTATTGGGA	0.022	0	0.022	46	2089
GCCAGCGTTTACA	0.054	0.003	0.051	87	1616
CCCGGCACTTGTG	0.024	0.001	0.022	26	1140
ACCATCGTTAAGG	0.024	0.003	0.02	37	1854
GCTATTGCTGAGG	0.067	0.012	0.05	38	724
GGCAGCGTTAGTG	0.059	0.015	0.056	43	725
CCTAGTATTTAGA	0.03	0.003	0.03	59	1902
GCCAGTGTTAATG	0.049	0.006	0.04	55	1330
CCCGGTACAGAGG	0.024	0.006	0.032	45	1341
AGTGTTATAAATA	0.021	0.002	0.024	75	3115
GGCGTTACAAATA	0.059	0.009	0.046	112	2299
ACCATTGTAGATG	0.025	0.003	0.024	57	2304
CCTATTATAAACG	0.028	0.005	0.021	80	3762
GCCATTGTTAACG	0.045	0.006	0.052	94	1714
GCTATTGTATGCA	0.054	0.003	0.053	84	1506
AGCGGCGCTGGGG	0.014	0.004	0.021	9	425
CGCATCATAGATA	0.023	0.002	0.021	68	3178
GCTATTACAAGTA	0.063	0.002	0.06	117	1822
CGTAGTACAAACA	0.024	0.007	0.02	44	2142
ACCAGCATAAGTA	0.021	0.002	0.02	78	3879
CCCGGCACAGGCA	0.027	0.006	0.019	41	2164
GGTGGTATATAGA	0.124	0.026	0.102	82	722
CCTAGTATTTATA	0.026	0.003	0.027	71	2567
ACTATCATAAATG	0.032	0.003	0.027	96	3427
ACTAGTGCTTACG	0.026	0.006	0.021	35	1652
ACCGGTACTGGCA	0.021	0.002	0.022	55	2418
AGCAGCACAAGGA	0.019	0.002	0.022	42	1903
GGCGTTGTAAACA	0.064	0.002	0.061	116	1783
ACTAGTACAAGTA	0.021	0.002	0.022	77	3441
ACTGTTATAAGTA	0.028	0.007	0.02	52	2557
ACTATCATTGACA	0.023	0	0.023	79	3336
ACTAGCATTGGGG	0.031	0.007	0.035	26	722
GGCATCACATGGA	0.066	0.009	0.058	79	1292
CCTAGTGTAAACG	0.025	0.002	0.026	63	2330
CGTATCACTGATA	0.027	0.006	0.019	29	1523

CCTATCATAAGCG	0.029	0.004	0.027	71	2579
CGCGTCGCTTACA	0.025	0.004	0.022	37	1618
GCCAGCACTAATG	0.055	0.004	0.059	109	1724
GCCATTGCATAGG	0.043	0.003	0.048	50	990
CCTATCGCTAATA	0.026	0.007	0.019	54	2785
AGTAGCGCTAATA	0.026	0.004	0.021	31	1415
CGCAGTATTGATA	0.022	0.001	0.022	50	2230
CGCGGCACAGAGG	0.022	0.005	0.029	24	817
CGCATTATTTATA	0.026	0.004	0.022	65	2828
AGCATCGTTTATA	0.026	0.006	0.02	51	2456
CCCGTTACAAGGG	0.025	0.002	0.024	35	1409
CGTGGTACTTAGA	0.024	0.004	0.021	16	758
CGCATCGCTAGGA	0.026	0.005	0.033	40	1158
ACCAGTATTGATA	0.024	0.004	0.028	114	3923
ACCAGCGTTTACG	0.025	0.004	0.02	32	1608
CCCATTACATGCA	0.024	0.001	0.023	83	3498
ACCGTCACAAGTA	0.019	0.001	0.02	72	3500
GGTATCGCATGCG	0.132	0.022	0.106	68	572
ACCGTTATATACG	0.023	0.003	0.023	62	2639
GGCATTACTTAGA	0.059	0.009	0.048	73	1451
GGCATCATAGATG	0.095	0.007	0.087	144	1517
GGCGTTGTATAGA	0.058	0.015	0.048	49	974
CCTAGTGTAAAGA	0.023	0.004	0.027	66	2369
GGCGTCGTTGAGA	0.074	0.008	0.068	59	804
CCTATCACAAAGG	0.026	0.007	0.017	42	2404
CCTATTGCAAATA	0.026	0.002	0.024	98	4037
CCTGGCATTGGTG	0.025	0.002	0.024	19	765
CGTAGTGTAAGCA	0.027	0.007	0.017	18	1058
CGCAGTATTAATA	0.027	0.004	0.025	77	3029
ACCGTCACTGATA	0.019	0.003	0.022	78	3398
ACCATTGTAAGTA	0.024	0.004	0.02	68	3332
CCTATTGTATAGA	0.024	0.007	0.019	41	2166
CCTGTCGTAAGTA	0.022	0.003	0.02	35	1744
GCCGTTACATAGG	0.056	0.004	0.053	56	1001
AGCATTACAGAGG	0.023	0.002	0.02	39	1889
CGCGGCGTTTATG	0.021	0.007	0.017	13	763
ACCATTATTGAGG	0.022	0.003	0.021	51	2421
AGCATCATTAGGG	0.022	0.005	0.015	27	1720
GCTGTTGTAAACA	0.061	0.011	0.047	84	1717
CGCGTTGTTGATA	0.028	0.004	0.022	30	1336

CGCGTTGCATACA	0.021	0.004	0.027	51	1855
CCTAGTGCTTGGA	0.026	0.006	0.032	38	1161
ACCAGCACTAAGG	0.026	0.002	0.025	63	2423
AGTATTACAAACA	0.025	0.003	0.025	92	3638
CGCGTTACTTGGA	0.019	0.003	0.022	22	974
AGTATTATAGGTA	0.026	0.005	0.024	50	2021
AGCATCACTGATA	0.02	0.003	0.02	63	3088
CGCATCGCAGGTA	0.025	0.008	0.037	50	1315
CGCGTTGCTTACG	0.022	0.003	0.021	23	1068
AGTGTTGTAGGTA	0.025	0.002	0.023	21	892
CCTGGTGCTTGGG	0.021	0.004	0.017	9	508
CGCAGCGCATGCA	0.024	0.006	0.031	51	1612
CGCAGTGTAAACG	0.026	0.002	0.023	44	1842
CGTGTTATTAGCA	0.023	0.002	0.022	26	1177
CGCAGTGTATATA	0.024	0.003	0.021	41	1867
ACTAGTATAGGTA	0.027	0.002	0.025	55	2155
GCTGTTATTAGGA	0.071	0.01	0.079	71	832
CGTATCGCAAATG	0.029	0.005	0.025	36	1399
ACCGTCGCTGGCA	0.023	0.007	0.015	27	1832
GGCGGTGCAGGCA	0.065	0.017	0.048	37	736
ACCGTTATAAGGA	0.025	0.001	0.025	63	2411
GGTGTTATAAGGA	0.081	0.02	0.056	48	815
CGTGTTGTAGGCG	0.03	0.005	0.028	18	629
CCTGGTACAAGTG	0.031	0.005	0.024	28	1162
GGTAGCACTGAGA	0.065	0.011	0.06	44	684
GCTAGTACAGACA	0.063	0.003	0.06	107	1665
AGCGGCGCTAGCG	0.023	0.008	0.012	9	763
ACTAGTATAAATG	0.026	0.002	0.028	90	3140
ACTATTGCTAATG	0.026	0.004	0.025	67	2601
GCCATCACATGCA	0.046	0.007	0.045	112	2377
CGCGTTACTAATA	0.023	0.003	0.025	58	2267
AGTATTATATGTA	0.025	0.003	0.024	62	2523
ACCAGTGCTAACG	0.025	0.002	0.026	71	2632
GCCATTACTTGTG	0.051	0.006	0.054	66	1157
GCTGGTGCATGCA	0.115	0.023	0.098	74	684
CGCGGTATAGATG	0.021	0.003	0.018	23	1231
ACCATCGTTGGCG	0.022	0.005	0.017	34	2023
ACTGGTACATAGA	0.02	0.005	0.014	29	2054
GCCAGCGTTGGTA	0.047	0.004	0.047	47	954
CGCATCGTTAGTA	0.023	0.002	0.024	42	1737

ACCATCACTAGCA	0.023	0.001	0.022	99	4332
ACCGGTATAAGCG	0.023	0.005	0.019	39	1986
CGCAGCGTATGCA	0.026	0.004	0.024	38	1555
AGTAGCATTTACA	0.021	0.004	0.022	42	1911
AGCGTTACTTACG	0.027	0.002	0.026	40	1508
ACTGTCACAGGCG	0.033	0.004	0.033	40	1169
AGCGGTATTAGGA	0.023	0.004	0.021	29	1365
AGCATCATATGCG	0.023	0.004	0.018	41	2247
AGCGTTGTTAGGG	0.021	0.009	0.009	8	836
GGCAGCACAGAGA	0.054	0.005	0.047	63	1286
CCTGGTGCTGATA	0.03	0.004	0.025	34	1329
AGTGTCGTAGACG	0.021	0.006	0.03	29	926
ACTATTACAGACG	0.025	0.003	0.02	63	3029
ACTAGTGCAAGCA	0.02	0.003	0.018	49	2725
AGTGTTACTTGGA	0.023	0.006	0.017	14	812
ACCAGTGTAGACA	0.023	0.001	0.024	77	3134
GCTATTATTGGGG	0.067	0.013	0.053	35	631
ACCAGTACTTGCA	0.02	0.002	0.022	80	3604
CGTAGCACTAATA	0.022	0.005	0.027	46	1632
CCCAGTGTTAAGG	0.026	0.007	0.021	31	1471
CGTGGTATAGACA	0.027	0.003	0.023	31	1320
CCTATCATTAGTG	0.026	0.003	0.022	43	1911
ACCAGCGCTGGCA	0.022	0.004	0.023	56	2404
AGTAGTGTTAATG	0.027	0.002	0.03	36	1171
AGTGTCGCTAACA	0.028	0.009	0.022	30	1319
AGCAGCACTAAGA	0.02	0.006	0.027	64	2269
GCCGTCATTAACG	0.059	0.007	0.05	91	1738
CGCGGTATTAATA	0.026	0.002	0.026	57	2094
CCTATCGCTGGCA	0.026	0.008	0.014	24	1669
CGCATTGCAAGGG	0.023	0.009	0.025	26	1028
GCTGTTATTAGGG	0.059	0.007	0.059	36	573
GGTATTATTGATA	0.117	0.015	0.097	166	1547
GCCGGTGCATGTA	0.061	0.005	0.054	48	841
GGCGGTATAAACG	0.124	0.017	0.124	189	1341
ACTAGTGTTGACG	0.023	0.003	0.023	37	1602
AGTATTGCTAGGA	0.022	0.002	0.025	24	945
ACTAGTACATACA	0.024	0.002	0.024	106	4396
CGCATCACATATA	0.029	0.004	0.026	79	2939
ACTGTTATTAGCG	0.029	0.003	0.028	36	1263
AGCGGTACAGGGG	0.019	0.004	0.023	17	722

GGCGTTACATACG	0.057	0.003	0.055	68	1158
CGCGTTACAAGCA	0.023	0.004	0.02	42	2059
CCTGGTGTAGGTA	0.025	0.002	0.023	26	1088
CGTGTTATATACG	0.021	0.006	0.017	22	1303
ACTGGTACAAGCA	0.025	0.002	0.027	68	2429
GCCGTCGCATAGG	0.046	0.005	0.041	24	562
CCCATCATAAAGA	0.026	0.004	0.021	99	4658
AGCAGTGCATAGA	0.028	0.003	0.032	58	1774
GGCATTACAAAGG	0.07	0.01	0.066	111	1568
ACCAGTATTTATA	0.023	0.005	0.017	74	4249
CCCGTCGCTTATG	0.025	0.007	0.023	33	1401
ACCAGTACAGGTA	0.021	0.002	0.023	77	3228
ACCAGTGTTAGCG	0.027	0.003	0.024	38	1574
GCCAGTGTAGGCG	0.045	0.01	0.059	73	1155
ACTAGCATAGGCA	0.029	0.002	0.028	63	2156
GCTGTCGTAGATG	0.066	0.009	0.061	46	711
CGTATCGTAAAGA	0.029	0.008	0.025	46	1805
CGCAGTGCAAACG	0.024	0.004	0.026	45	1701
CGCGGTATTGGGA	0.028	0.005	0.035	27	754
ACCGGCACTTGTA	0.023	0.002	0.021	45	2095
AGTATTGTTTACA	0.024	0.001	0.024	45	1816
ACCGTTATTAAGA	0.025	0.005	0.022	69	3076
GCTATTATTGGCA	0.054	0.006	0.047	75	1536
ACCATTACTGACG	0.025	0.002	0.024	71	2837
ACCAGCACATATG	0.021	0.002	0.02	67	3366
CGCGTCGCAAGTG	0.033	0.014	0.016	17	1054
GCTAGTATTGATA	0.061	0.003	0.064	120	1745
CCCGTCGTTAATA	0.024	0.007	0.019	50	2584
ACTATTATAAGTG	0.029	0.005	0.022	62	2737
ACCGGTGCTAATG	0.022	0.002	0.022	36	1581
CCCAGCACATACA	0.024	0.002	0.022	92	4145
ACCAGTGTAAACG	0.025	0.002	0.024	71	2884
ACTAGCACTTGGG	0.024	0.009	0.011	10	872
ACCGGTATATACA	0.026	0.002	0.023	84	3607
CCCATCATATGCA	0.024	0	0.023	90	3781
CGTATCACAGACA	0.025	0.002	0.022	44	1960
AGTATTATTAACA	0.025	0.003	0.021	80	3660
GGTATCATAGGTA	0.114	0.021	0.104	122	1053
AGTATTATAAGGA	0.024	0.001	0.023	51	2126
CCCAGCATAAGCG	0.026	0.003	0.03	81	2609

ACCATCACAGGCG	0.023	0.002	0.022	51	2245
CGTATCACTTATA	0.029	0.005	0.027	45	1636
AGCAGTACAGAGG	0.025	0.004	0.024	35	1429
ACTGTCACATAGG	0.032	0.005	0.037	47	1235
AGTAGCGCATATG	0.023	0.002	0.024	23	936
AGTATTATTTACG	0.02	0.003	0.024	45	1826
ACCGGTATTAAGG	0.029	0.002	0.029	48	1619
CGTATCGTTGGTA	0.025	0	0.025	25	990
ACTATCGTAAGCA	0.025	0.003	0.023	55	2358
CCTAGCACAAATG	0.026	0.004	0.024	58	2337
ACTATCATTGAGA	0.025	0.003	0.024	58	2408
CGTGTTGCAAAGA	0.022	0.003	0.026	31	1161
ACTAGTACTGATG	0.033	0.009	0.023	51	2214
GGCGGCGCTAGCA	0.098	0.013	0.097	79	735
ACTATTGCAAACA	0.027	0.001	0.025	124	4898
ACCGGCACTGACG	0.024	0.006	0.033	60	1765
GGCATTATTAACG	0.06	0.006	0.052	108	1953
GGTGTCATAAGGA	0.099	0.018	0.075	56	689
GCCGTCGTATATA	0.052	0.006	0.059	90	1429
ACCGTTATAGACA	0.023	0.003	0.019	73	3710
ACTAGTATTGAGG	0.031	0.002	0.029	40	1337
GGTAGTGCAAACA	0.056	0.004	0.051	67	1252
ACCGGCGCATGTA	0.033	0.003	0.033	42	1214
ACTAGTATAAACG	0.027	0.002	0.024	78	3134
ACCATCGTAGACA	0.028	0.001	0.028	99	3387
CCCGGCGTAAACG	0.022	0.003	0.023	47	1988
ACCGGCACAAACG	0.019	0.001	0.02	63	3045
GGTATTATATGGG	0.109	0.018	0.098	71	657
GCTGTTGCAAACA	0.054	0.002	0.053	88	1571
CGCATTATTAATG	0.024	0.003	0.026	64	2353
GGCGTTGCTGGGG	0.055	0.004	0.055	21	360
AGCATCGTAGACA	0.025	0.005	0.024	74	2950
ACTAGTATAGATG	0.027	0.003	0.024	55	2202
GGTGTTATTTGCG	0.093	0.009	0.087	48	501
ACTATCATAAGCG	0.028	0.005	0.022	53	2329
AGCGTTACTGATA	0.024	0.002	0.023	52	2213
ACCGTTGTTAATG	0.025	0.001	0.027	50	1828
GGCAGTGCTTGCG	0.053	0.003	0.049	32	621
ACCAGTATATACG	0.023	0.003	0.02	71	3549
AGCATTATTAATG	0.022	0	0.023	75	3226

ACCGTCGCAAGGA	0.026	0.003	0.026	46	1715
CCTAGCATATAGG	0.023	0.002	0.025	36	1405
GGCAGCGTTGGTA	0.058	0.009	0.046	38	792
CCCATCGTTAAGA	0.025	0.003	0.022	58	2564
CGCAGCGTTAACA	0.025	0.005	0.02	46	2213
AGCAGTGCAAGTG	0.019	0.005	0.015	20	1352
AGTAGTGTTTGTA	0.017	0.001	0.018	19	1027
CGCATTGCTTACA	0.025	0.007	0.021	42	1974
CCTGTCACAAGGG	0.03	0.003	0.033	34	997
GCCGGTATTAAGA	0.077	0.001	0.075	128	1579
AGCGTCATATGGG	0.02	0.005	0.026	29	1070
CGTATCATATATA	0.025	0.003	0.021	51	2387
GGCATTACTGGCA	0.048	0.006	0.042	60	1379
ACCATCATTGAGA	0.022	0.001	0.021	67	3099
AGTATTGTTTGTG	0.031	0.012	0.025	23	880
GGCATTATTAGTG	0.068	0.009	0.058	75	1217
CGTGTTATTGAGA	0.022	0.004	0.017	17	1008
GGTAGCACAAATG	0.078	0.008	0.072	76	983
ACTAGCGCAGGGA	0.022	0.008	0.013	17	1313
GCCGGCATTTAGA	0.394	0.023	0.377	532	881
ACCGTTACATGTA	0.022	0.005	0.028	95	3300
ACTATTACTGACA	0.022	0.001	0.023	91	3842
CGTGGTACAGACA	0.019	0.002	0.017	23	1363
GCCAGCATAAGCA	0.055	0.004	0.059	162	2588
CCTATCGCTTACA	0.028	0.007	0.025	64	2464
GCTGGCACTTGGG	0.45	0.066	0.411	202	290
GGCGTCATAGGTG	0.063	0.005	0.07	60	799
CGCAGCATTAGTA	0.025	0.002	0.024	44	1814
AGCAGCGTAGATA	0.023	0.003	0.025	58	2228
CCCATTACTAGTG	0.031	0.004	0.026	55	2071
GCTATTACTAGGA	0.064	0.007	0.074	85	1066
GCTGTCGCTAGCG	0.068	0.006	0.063	31	463
GCCAGCACAGGGA	0.062	0.005	0.054	60	1051
ACTATCGTTAAGA	0.027	0.005	0.03	66	2144
CCCGGCGTAAGTA	0.023	0.003	0.02	41	2043
ACCAGCATTAGGG	0.027	0.002	0.028	34	1161
CCCAGTGCAGGCG	0.024	0.002	0.026	40	1508
CCCGTCGTTTATA	0.022	0.003	0.018	38	2110
ACCATTACATGCA	0.021	0.002	0.021	102	4820
CCCGGCATTTATG	0.025	0.004	0.02	34	1650

CGCAGTATAAAGG	0.026	0.004	0.021	42	1916
AGTATCGCAAGTA	0.022	0.004	0.02	29	1445
GCTGTCATAAAGA	0.062	0.002	0.06	97	1518
GCCATCACAGATA	0.047	0.005	0.044	119	2606
GCCAGCGCTAGCA	0.053	0.006	0.048	61	1204
CCCGGCACAAGGA	0.025	0.003	0.023	44	1906
GCCGTCGTAAAGA	0.053	0.009	0.063	91	1362
ACCAGCGCAAATG	0.025	0.001	0.027	76	2716
ACCAGTATTTAGA	0.021	0	0.021	65	3102
CCCATCGTAAATA	0.023	0.002	0.02	92	4452
GGCAGCACATGTA	0.049	0.005	0.043	61	1366
CGTGTCGTTGGCA	0.03	0.009	0.023	16	682
CCCAGCACAGGGG	0.042	0.009	0.03	37	1200
ACTATCATTGGCG	0.026	0.005	0.02	45	2252
GGCATCGCTTATG	0.105	0.008	0.099	101	920
CGTAGCATTAATA	0.026	0.002	0.029	54	1838
ACCAGCACAGGCA	0.023	0.001	0.024	89	3641
AGTGTTATTAATG	0.021	0.006	0.022	38	1658
ACTATCACAGATG	0.027	0.002	0.03	83	2725
ACCAGTGTTAAGG	0.022	0.005	0.016	27	1640
GGCGGCATATACA	0.318	0.05	0.308	588	1324
CCTATTACTTGGA	0.029	0.003	0.032	57	1698
GCCGGTGTAAGCA	0.175	0.026	0.177	249	1154
CCTATCGTTAGTG	0.023	0.007	0.018	24	1323
GGTAGTATAAGTG	0.053	0.015	0.045	37	789
AGCGTCATTTAGG	0.023	0.006	0.023	27	1160
ACTGTCACAAGCA	0.025	0.001	0.025	66	2572
ACTGTCATATGTG	0.028	0.003	0.023	31	1291
ACTAGTGCTTACA	0.026	0.001	0.026	69	2556
AGCATCGTATGCG	0.022	0.005	0.018	30	1661
GGCAGTACTAGCG	0.061	0.008	0.059	62	981
AGTATTGCTAACG	0.021	0.003	0.018	28	1554
ACTGGCACAAAGG	0.027	0.006	0.018	23	1261
AGCATTATATAGA	0.023	0.001	0.023	86	3610
GGCGGCGCAGACG	0.191	0.031	0.156	135	731
CCCGGCGCTGGCA	0.021	0.007	0.011	17	1531
AGCGTTACAAGTG	0.023	0.005	0.023	33	1419
CCCGTTATTTATA	0.023	0.003	0.024	66	2710
CCCGGCACAGACA	0.023	0.002	0.024	74	2990
AGCATCGCAAAGA	0.025	0.007	0.018	48	2551

GGTGGTGCAAGGA	0.072	0.015	0.058	31	499
ACTATCGTTGGGA	0.026	0.005	0.019	22	1117
ACTAGTATTAGGA	0.026	0.004	0.02	39	1867
AGCAGTATAGGCA	0.024	0.002	0.024	63	2547
CGTGGCGTTTGGA	0.022	0.01	0.024	9	370
CGTGTCACTAATA	0.021	0.001	0.02	32	1599
CCTGTCATATATG	0.027	0.002	0.029	51	1687
CCTATCACAGGCG	0.034	0.008	0.027	47	1707
AGTAGCACAGAGA	0.025	0	0.026	34	1293
CGTGGTGTAGATA	0.02	0.002	0.018	17	924
GCCGGTACATACA	0.057	0.006	0.061	132	2042
CGTGGTATTAGTG	0.024	0.007	0.028	15	525
CCTATTATAGAGG	0.031	0.003	0.027	50	1808
AGTAGCGTATGCG	0.02	0.01	0.033	24	712
CGCAGTGTTGAGA	0.025	0.005	0.03	34	1103
CCCGTCGTAAAGG	0.018	0.002	0.019	30	1528
GCTAGCATTAGGA	0.058	0.006	0.049	48	926
CCTAGCACTAACA	0.023	0.004	0.018	58	3183
CCTAGCGTTTGTA	0.026	0.006	0.02	27	1312
ACCGGTGCAAAGA	0.023	0.002	0.026	64	2366
GCCAGTGCAGGGA	0.042	0.003	0.038	32	802
CGTATCGTTTACA	0.027	0.003	0.024	38	1578
CGTGGTACATGTG	0.02	0.004	0.022	14	613
ACTATCATTTGCG	0.032	0.006	0.024	41	1660
GGCAGTATATATG	0.054	0.008	0.051	89	1671
AGCATTGTAAATA	0.022	0.001	0.023	100	4264
AGTAGCATAAGGG	0.026	0.003	0.023	22	949
GCCGGCGTTTACA	0.85	0.031	0.856	1718	289
ACCGGTACAAACA	0.024	0.003	0.027	157	5673
AGCATTATTAGCG	0.022	0.005	0.019	40	2059
CCTGGCATAAGCA	0.029	0.004	0.028	55	1933
CCCGGCGTATATG	0.021	0.003	0.02	28	1389
CCTGTCACATAGG	0.023	0.005	0.019	22	1167
CGTGTTGCTAGCG	0.027	0.005	0.021	14	640
ACCGTTATAAACA	0.023	0.004	0.019	98	5113
CCCGGCACAAATA	0.03	0.002	0.03	109	3504
CCTGTCGTATACA	0.023	0.004	0.018	40	2159
CGTATTGCAAACG	0.028	0.004	0.03	49	1568
CGCGGTATTAGCA	0.03	0.002	0.032	47	1408
GGCATTGCAGAGG	0.061	0.01	0.052	41	741

CCTATTGCATGCA	0.024	0.004	0.019	45	2306
GCTAGTACAAATA	0.06	0.005	0.054	141	2475
GCCAGCACAGACA	0.055	0.002	0.052	140	2563
GCCGTTGTATACG	0.05	0.005	0.043	52	1156
ACCGGTGTAGGCG	0.025	0.004	0.028	40	1387
CCTGTTATAGACG	0.032	0.008	0.025	44	1718
GGCGGTATTGGCG	0.109	0.008	0.101	65	580
CCTGTCGTTAAGA	0.026	0.001	0.027	43	1554
CGCATTATTAGCA	0.027	0.004	0.032	80	2410
AGTGTCGCATATG	0.019	0.005	0.012	10	815
CGCAGTATTGACG	0.03	0.004	0.025	40	1582
CGCAGTGTATAGG	0.032	0.002	0.03	32	1022
GCTATCACAGATA	0.073	0.009	0.061	112	1711
GGCGTTGTTTATG	0.073	0.005	0.078	55	651
CGCGTTGCTTGGA	0.022	0.003	0.019	16	848
GCTAGCACTTGCG	0.065	0.014	0.046	32	660
CGCGGCACTGGTG	0.024	0.003	0.028	21	721
AGCGTCACATGGA	0.018	0.004	0.015	21	1412
GCCATTACTAGCA	0.054	0.003	0.056	127	2144
CGCATCACATACA	0.024	0.004	0.021	69	3295
AGCAGCACAGACA	0.022	0.004	0.017	52	3055
CCTATCGTTTACA	0.026	0.002	0.024	56	2269
AGTGGCATTGGGG	0.035	0.011	0.019	3	151
GGCATCACAAGTG	0.083	0.01	0.078	127	1491
AGCAGTATAAGTA	0.019	0.002	0.018	66	3626
GGCGTCATATAGG	0.072	0.012	0.059	55	873
GCTGGTACTAACA	0.081	0.005	0.08	125	1429
CCTGTCATATGCA	0.024	0.004	0.019	36	1874
CGCGTCATAAGGA	0.021	0.004	0.024	37	1512
CCTAGCGTTAAGG	0.028	0.006	0.024	28	1117
AGTAGCATATAGG	0.032	0.006	0.025	25	983
ACCATTATTGGTG	0.023	0.004	0.018	39	2074
AGTAGCGTTGGTA	0.023	0.009	0.036	29	777
AGCAGTACATACG	0.022	0.002	0.021	53	2481
CCCATTATAAACG	0.025	0.005	0.021	102	4692
CCTAGTGCATACA	0.024	0.005	0.018	52	2766
GCCGGCATATATA	0.394	0.048	0.363	867	1521
GGTGTCACTTATA	0.088	0.023	0.069	65	880
GCTGTCATAGAGA	0.065	0.014	0.053	60	1071
CGTGGCATAAAGA	0.028	0.006	0.035	45	1234

ACTGTCATTGATG	0.03	0.006	0.028	42	1437
GCCATCGCTTACG	0.048	0.001	0.047	58	1188
GCCGTTATAGATA	0.057	0.004	0.054	132	2327
CGCGTTATTGGCG	0.022	0.006	0.019	20	1021
AGCGGTACTAACG	0.024	0.007	0.018	29	1573
GGCATCGCAAAGG	0.099	0.016	0.084	105	1147
ACCGTTGCTGATG	0.022	0.002	0.022	37	1618
AGCGGTACTTATG	0.023	0.005	0.017	21	1229
AGTGGTACAAGGG	0.022	0.01	0.015	9	607
GCCGGTGTAGGGA	0.138	0.052	0.113	80	625
GCTAGCATTAGCA	0.068	0.009	0.058	87	1402
ACCATCACTAGCG	0.023	0.004	0.024	69	2788
AGCAGCATTTATG	0.023	0.002	0.022	45	2006
CCTGGTGCAGATG	0.024	0.004	0.026	25	928
ACTATCACTAATG	0.025	0.001	0.023	71	2962
AGTATCGCTGGCA	0.023	0.006	0.021	22	1030
ACTGTTGCTAGCA	0.023	0.002	0.024	42	1735
GGCGTTATAAATG	0.058	0.006	0.049	86	1667
AGTAGCGTAAATA	0.023	0.005	0.018	37	2065
AGCATCATAGGGA	0.024	0.001	0.026	68	2542
ACCGGTATTTGCA	0.021	0.002	0.022	44	1942
GGTAGCGTATGTA	0.079	0.021	0.053	36	647
ACTATCACTAAGA	0.025	0.004	0.02	69	3436
CGTGTTGCAAGTG	0.023	0.002	0.025	18	705
CGTAGCGTTAAGG	0.022	0.01	0.009	6	696
GCTGGTATTGACA	0.153	0.005	0.149	202	1156
ACCATTGCTAAGA	0.023	0.003	0.02	62	3093
CGCGTCGCTGAGG	0.021	0.002	0.024	18	721
CCCGTTATATGTG	0.03	0.006	0.023	40	1695
ACTGGTATAGGTA	0.03	0.005	0.026	38	1416
GCTATCACTAGTA	0.06	0.002	0.062	85	1287
GGCATCACATAGA	0.066	0.008	0.056	97	1626
CGTGTCACAAGTG	0.018	0.004	0.015	14	924
GGTGGCGCATATA	0.166	0.015	0.167	137	682
CCCGTTGTTTGTA	0.023	0.008	0.03	43	1398
AGTGGCATATGGA	0.013	0.003	0.017	9	534
CCTATCGTTTAGA	0.024	0.002	0.021	35	1623
GGCAGTATTAACA	0.055	0.006	0.057	156	2559
CCCATTACATAGA	0.027	0.003	0.023	77	3288
GGCATCGCAGGCA	0.078	0.015	0.056	75	1260

CCTAGTACATAGA	0.026	0.001	0.026	55	2092
ACTAGTACTTGCG	0.022	0.001	0.02	31	1529
ACCAGTGCTAATA	0.022	0.002	0.025	94	3639
ACTATCACATAGA	0.025	0.002	0.022	72	3172
ACCAGCATATGTG	0.023	0.004	0.018	39	2177
GGTATCACTTACG	0.127	0.027	0.096	92	867
CCCATTGTTAAGG	0.024	0.002	0.026	48	1824
CGCGGCATTAGCA	0.026	0.007	0.023	32	1354
GCCATTACAAATG	0.056	0.001	0.055	156	2682
CCCAGCGTTGATA	0.023	0.001	0.022	55	2461
CCCATTGCATATG	0.021	0.003	0.018	38	2052
CCTAGTGCTTATA	0.029	0.005	0.026	54	2045
GGTAGCATTGAGA	0.07	0.008	0.062	53	806
ACTGGTACTAGTA	0.027	0.002	0.024	44	1812
CCCGTCGTTGATG	0.025	0.004	0.021	29	1350
GCCGGCATAGATA	0.395	0.038	0.387	842	1331
ACTGGCACAGATG	0.023	0.002	0.024	30	1219
AGTGTCATAAACA	0.022	0.001	0.022	59	2571
GCTAGCACTGGCA	0.068	0.012	0.063	69	1022
CCTGTTACAGACG	0.027	0.001	0.025	41	1577
CGTAGCATAGACG	0.024	0.004	0.028	34	1188
ACTAGCGCAAGGA	0.021	0.001	0.023	42	1812
AGCGTCGTAGGCA	0.018	0.003	0.018	25	1369
ACCGTTGTTGGGA	0.023	0.006	0.014	15	1060
ACTGTTACTGAGA	0.028	0.004	0.034	67	1916
AGTGGTACTAATG	0.019	0.002	0.017	17	1002
GGCAGCGCTTAGG	0.069	0.008	0.075	47	581
CCCGGCGCTGGCG	0.022	0.004	0.02	22	1052
CGTAGTATATACA	0.028	0.001	0.028	58	2011
GCCATTGTAAGTG	0.051	0.009	0.046	65	1351
CGTAGTGCAAATG	0.028	0.004	0.033	34	984
ACCAGTGCTAACA	0.022	0.002	0.02	86	4145
ACTAGTACTTAGG	0.021	0.003	0.018	30	1607
GGCAGTATTTGGA	0.054	0.008	0.051	57	1055
AGCATCGCTTGTA	0.023	0.003	0.023	37	1554
GCCATTGCTTGGA	0.043	0.001	0.043	37	823
GCTATTGTTGGCG	0.051	0.013	0.043	48	1078
AGCGTCATAAGTA	0.022	0.003	0.026	69	2623
AGTGGTATTGACA	0.027	0.008	0.016	23	1418
AGCATTATATGTA	0.019	0.001	0.018	65	3573

GCTATCGTAAGCG	0.067	0.01	0.054	62	1092
AGCGTTGTTAGTA	0.022	0.006	0.025	38	1479
GCCATTATAAGGA	0.049	0.006	0.057	143	2352
GCTGTTACTTACG	0.065	0.004	0.059	51	812
CCCATTGCTAATG	0.029	0.004	0.029	69	2276
CCTGTCATTGACA	0.024	0.002	0.023	53	2234
ACTGTTATTAATG	0.031	0.004	0.036	72	1931
ACCAGCATATGGA	0.021	0.002	0.024	53	2159
CCCAGCGTAAGCG	0.024	0.003	0.027	53	1894
GGCGTCGCAGGCA	0.058	0.01	0.046	45	931
ACCATTACTAGGG	0.029	0.006	0.025	52	2037
CCTAGTGCTGGGG	0.034	0.005	0.04	25	594
ACTATTACTGAGA	0.024	0.002	0.023	65	2748
ACTGTTACAAATA	0.023	0.001	0.024	106	4315
CCTGTTGCAGATG	0.025	0.006	0.019	23	1211
CGCGTTACATACG	0.026	0.004	0.021	34	1620
CCTGTTGCAGGGG	0.023	0.009	0.01	7	666
GGTAGTACTTGTG	0.07	0.008	0.079	43	499
CGCATTGTAAAGA	0.029	0.003	0.027	69	2450
GCCAGTGCTAAGA	0.038	0.005	0.031	41	1301
GCTATCGTAGATG	0.058	0.002	0.06	64	1002
AGCAGTACTGGCA	0.02	0.003	0.022	41	1842
ACTGGTACAAGTG	0.019	0.001	0.019	25	1319
ACCAGTGTAAACA	0.022	0.001	0.021	95	4386
CCTGTTGTAAACA	0.024	0	0.024	69	2847
AGCGGTGTAGACA	0.019	0.005	0.014	25	1792
CGTATCATTTAGA	0.027	0.004	0.022	31	1401
CCTATTATATGCA	0.027	0.005	0.033	98	2852
CCCGGTACTGAGA	0.023	0.004	0.017	31	1741
AGCATTGTAGATA	0.023	0.001	0.025	72	2798
GGTGTCATAGACG	0.101	0.024	0.083	62	686
GCTGTTGTAAGTA	0.057	0.001	0.055	64	1097
CCTGTTATTTGCG	0.027	0.007	0.02	22	1094
ACCAGCATAAGCG	0.023	0.003	0.021	60	2785
CCTAGTACAGACA	0.029	0.003	0.028	85	2928
CGCAGTGCATACG	0.026	0.004	0.029	36	1223
AGCGTCGCTTGGA	0.023	0.005	0.022	19	841
ACCATTGTTGATG	0.026	0.006	0.031	61	1924
CGTATCGTTGATG	0.029	0.003	0.03	30	954
ACCATTACATACA	0.024	0.002	0.025	174	6860

CCCAGCACAGGTA	0.022	0.002	0.019	46	2323
GCCAGCGCAGATA	0.056	0.002	0.057	85	1413
AGCATCGTTTGGA	0.019	0.003	0.022	31	1351
ACCGGTACTAATA	0.022	0	0.022	88	3985
CGCATTGCTAGTA	0.02	0.002	0.018	27	1493
ACCGTTATAAGTA	0.023	0.002	0.022	75	3370
CCCATTATAAGTG	0.024	0.001	0.024	69	2781
ACCAGCGTATACA	0.026	0.003	0.025	79	3054
GGCAGCACAAATA	0.054	0.002	0.055	146	2498
AGCGGCGTAAATA	0.021	0.006	0.02	41	2027
CCCGTTATTGGTA	0.024	0.006	0.016	31	1864
CGCGTTACAAGGG	0.019	0.002	0.022	22	997
CCCAGCGTAAATA	0.021	0.003	0.025	99	3791
ACCATCGTTTGCA	0.025	0.002	0.023	51	2213
ACCATTGCTTGTG	0.02	0.005	0.025	42	1617
GCTAGCGTATACG	0.08	0.013	0.07	71	943
CCCATTGCATATA	0.025	0.001	0.024	80	3223
AGCGGCGTTTAGA	0.024	0.007	0.025	23	909
GGCGGCGCTTATA	0.109	0.02	0.117	98	739
CCTAGTATAGATA	0.024	0.005	0.017	49	2786
CGTAGCGCATATG	0.031	0.01	0.017	16	903
CCTAGTGTTAGGA	0.024	0.005	0.017	22	1243
ACCATTATAAACA	0.022	0.002	0.025	206	8096
GCTGGCGTATGCG	0.948	0.009	0.938	1075	71
ACCAGCACTGGGG	0.024	0.006	0.016	16	982
CCTATTGCTGGCA	0.021	0.002	0.018	30	1677
GCCATCGCTAACG	0.045	0.005	0.042	65	1465
ACTGGTGTTGACA	0.024	0.005	0.019	29	1495
CCCATTACAGGTA	0.025	0.001	0.024	65	2628
GCTGTTGTATGGG	0.071	0.013	0.055	30	518
GCCATCATTGACA	0.052	0.004	0.053	169	3022
CCCGTCACAAGTA	0.026	0.003	0.025	69	2646
CGCAGCATTTACA	0.021	0.005	0.018	44	2459
CCTATTACAGATA	0.024	0.002	0.027	94	3362
GGTAGTACAAAGA	0.058	0.009	0.048	62	1242
CGCATCATTAGTA	0.023	0.002	0.021	48	2271
CGCATTGTAGGGA	0.027	0.004	0.029	30	1018
ACCAGTGTTGGTA	0.023	0.004	0.018	31	1712
ACCGGTGCTGGTA	0.021	0.003	0.018	26	1400
CCCGTTGTAGGGG	0.027	0.009	0.016	15	914

CCCATTGCTAATA	0.025	0.001	0.024	86	3499
GCTGGTATTTGCA	0.158	0.022	0.141	137	836
ACCGGCACAAGGG	0.025	0.005	0.023	29	1239
GCCAGCATTAGCA	0.059	0.008	0.059	129	2045
CGTGTTACAAATA	0.021	0.002	0.02	47	2262
CCCATTGCATGGA	0.025	0.005	0.02	36	1789
ACCGTTACTAGGA	0.026	0.002	0.023	55	2297
CCCGTTGTAGATA	0.02	0.005	0.016	35	2201
ACTGGCACTAATA	0.024	0.005	0.018	42	2350
CCTATTGTAAGGA	0.028	0.006	0.02	39	1914
GCTGTTATAAGCA	0.054	0.008	0.047	79	1590
GCTAGTATAAGTG	0.057	0.006	0.064	83	1215
ACCGTTGCAAAGG	0.023	0.002	0.02	37	1769
GGTATTATTTGGA	0.102	0.009	0.09	86	874
CCCATTGCAGGTG	0.026	0.003	0.022	32	1438
GCCATTATTGGCA	0.046	0.008	0.041	89	2065
CGTGGTGCAAGCA	0.026	0.008	0.015	14	945
AGCAGCGCTTAGA	0.023	0.004	0.025	32	1274
ACTAGTGTATAGA	0.024	0.001	0.024	51	2096
CGTAGCGCAGAGG	0.028	0.006	0.024	14	578
AGTGGTGTTTGGA	0.018	0.008	0.028	16	559
GCTATCACAGACG	0.054	0.006	0.052	79	1447
AGCGTCACAAAGG	0.024	0.003	0.027	46	1665
GCTGGCGCATACA	0.614	0.064	0.619	991	609
ACCATCGTAAGCA	0.027	0.002	0.025	81	3107
ACTAGTGTAGAGA	0.028	0.002	0.028	54	1888
GGTGGCGTTAGGG	0.667	0.033	0.648	311	169
GCCATCATTAAGA	0.052	0.004	0.05	141	2703
AGCGGTGCAAAGA	0.02	0.001	0.021	35	1629
CCCAGCACAAATG	0.026	0.001	0.025	83	3214
GGCGTCGCATGGG	0.088	0.011	0.073	38	485
GGCAGCATTGGTA	0.061	0.01	0.048	59	1163
GCCAGTACTAGGA	0.057	0.006	0.059	82	1315
CCCGTCATTAGCA	0.025	0.004	0.022	54	2454
GGCGTCGTAGACA	0.079	0.014	0.061	78	1206
CCTATTGTTAATA	0.024	0.003	0.02	64	3189
GGTATTACTTGCA	0.084	0.011	0.069	69	932
CCTGTCGCAGAGA	0.024	0.006	0.02	27	1324
CCCATTGCTGACA	0.023	0.006	0.017	55	3095
GCTATCGCTTAGA	0.058	0.01	0.049	44	851

AGCGGCGCAAAGA	0.02	0.001	0.019	28	1413
GCCATTATAAGTA	0.061	0.006	0.056	186	3130
CCCAGCGTTAACG	0.026	0.002	0.024	58	2357
ACCGTTGTATGTG	0.035	0.004	0.041	54	1261
CGCAGCACATGGA	0.028	0	0.028	38	1297
CGTGTCATAAGTA	0.021	0.005	0.016	23	1427
ACTAGCGTAAAGA	0.035	0.003	0.035	76	2086
GGTGTCACAGGTA	0.08	0.024	0.051	36	676
CGTATTGTAGAGA	0.025	0.003	0.022	26	1157
CCTGGCACATACA	0.026	0.006	0.023	51	2138
GCTAGCATTTATG	0.054	0.004	0.052	60	1091
CCCAGCGCAAGGG	0.024	0.007	0.021	25	1188
CCCGTCGTAAACG	0.02	0.004	0.022	54	2439
AGCATTATAGGGA	0.019	0.003	0.015	38	2511
CCCGGTACAGATG	0.029	0.005	0.036	65	1744
CCCGTCATTAGTG	0.027	0.002	0.024	37	1476
ACCAGCGCAAACA	0.018	0.003	0.014	80	5610
GGTGTCGCAGAGA	0.068	0.009	0.069	42	565
CGTAGCATATGGA	0.021	0.003	0.02	20	990
CCTGTTACATGGA	0.02	0.002	0.017	24	1349
CGTATCATTGGTA	0.027	0.002	0.029	35	1193
GGCAGCATAGACA	0.055	0.005	0.052	116	2125
AGCATCACTTATG	0.024	0.002	0.023	52	2178
GGCGGTACTGACA	0.058	0.012	0.043	57	1280
ACTGTTATTTACG	0.032	0.006	0.026	43	1611
GCTGTTATAGAGG	0.063	0.01	0.059	46	731
GCTGGCATTTGCA	0.652	0.037	0.635	813	467
CCCGGTATAGAGA	0.024	0.003	0.028	57	1968
CCTAGTATAGGTG	0.024	0.004	0.019	23	1166
GGCAGTGCAAAGA	0.047	0.018	0.073	100	1279
GCCAGCGTAGATG	0.065	0.005	0.063	71	1049
AGTGTCATTAACA	0.021	0.003	0.018	41	2183
GCCATTATATGCG	0.056	0.001	0.058	115	1880
GCCATTATAGGCA	0.056	0.007	0.048	120	2386
ACCAGCGCATATG	0.021	0.003	0.019	40	2058
CCCAGCGTTAATA	0.024	0.004	0.02	59	2921
GCCATCATAAGTA	0.056	0.002	0.055	172	2983
CCCATCACTGACG	0.043	0.007	0.033	87	2577
ACTATTGCAAATA	0.023	0.004	0.019	89	4686
AGCAGCGTAAGGG	0.023	0.006	0.025	31	1225

GCTATCGTTGATA	0.055	0.003	0.059	81	1302
GCTATTACTGGGG	0.064	0.008	0.07	46	611
CCCGTTATATGTA	0.025	0.001	0.025	64	2479
CCTATTGTTTAGA	0.027	0.005	0.03	55	1781
GCCATCGCAAGCA	0.046	0.008	0.036	70	1878
GGCGGCATATGTA	0.291	0.042	0.276	342	899
CCCATTGCTGAGG	0.027	0.003	0.026	36	1329
GCTATCATAAACA	0.06	0.008	0.048	160	3172
ACCGTCGTATATA	0.025	0.001	0.024	57	2292
CGCGTCGTTAACG	0.026	0.002	0.024	33	1367
AGCGGTGCAGAGA	0.021	0.007	0.028	31	1061
ACTGTCGCTAGGA	0.019	0.003	0.023	25	1061
CCTGTTACTTAGA	0.025	0.004	0.023	34	1431
AGTAGCGCTTATA	0.023	0.002	0.02	23	1141
ACCATCACTTGCA	0.023	0.003	0.022	84	3662
AGCAGCATTGGGG	0.028	0.005	0.033	43	1241
ACTGTTGTATACA	0.023	0.002	0.022	51	2248
AGTGTCATTTAGA	0.025	0.004	0.028	33	1127
ACTGGTGTTAACG	0.026	0.003	0.027	35	1263
ACCGGTGTAAGTA	0.026	0.002	0.024	48	1980
GCTGGCATATAGA	0.638	0.046	0.639	986	558
ACCGGTGCTAAGA	0.022	0	0.022	45	2013
GCCAGTGTTGATA	0.049	0.007	0.052	79	1426
ACCAGCACATGGG	0.02	0.001	0.021	30	1404
ACCGTTGCTGGGA	0.026	0.006	0.031	38	1203
ACCGGCATATACA	0.022	0.002	0.019	66	3399
ACTGGCGCAAGTA	0.028	0.009	0.014	14	958
AGCAGTGCAAAGA	0.021	0.002	0.022	51	2267
GGTAGCACAGACG	0.072	0.017	0.05	40	757
AGTATTGTAGGGG	0.027	0.001	0.027	22	784
GCTAGTACTGGCG	0.063	0.005	0.07	60	802
AGTAGTACTAGCA	0.025	0.001	0.024	34	1409
ACTATTGCATGGA	0.025	0.001	0.025	45	1778
GCTGGTATAGACG	0.442	0.048	0.445	501	624
CCCGGCATTGAGA	0.021	0.004	0.015	28	1792
CCTATCATAAACA	0.029	0.004	0.026	146	5550
ACTGGCGCAAGGG	0.034	0.006	0.041	25	578
GCCAGTATTAAGG	0.049	0.002	0.047	81	1633
CGCATTATAAAGG	0.023	0.005	0.018	42	2266
GGCAGCGCATACA	0.06	0.004	0.065	111	1596

CGTAGTGTTAGTA	0.027	0.006	0.023	18	776
ACCGTCACAAGCG	0.023	0.004	0.019	43	2263
CCTATTGTTTACA	0.025	0.001	0.026	68	2597
AGTATCATAGGGA	0.02	0.004	0.018	24	1342
CCCATCATATACG	0.025	0.002	0.023	82	3476
CGTGTCGCTGGTA	0.027	0.007	0.018	12	653
GGCATCGCAGACA	0.092	0.017	0.072	136	1756
CCCAGTGCAGACG	0.022	0.006	0.015	30	1987
GCTATTGCAAGTG	0.054	0.003	0.057	61	1014
GGCGGCATAGGGG	0.306	0.059	0.256	215	625
ACTAGTGTAGACA	0.026	0.005	0.023	63	2736
GCCGTCATTTAGG	0.044	0.011	0.033	31	919
AGCAGTGCAGAGG	0.024	0.006	0.016	18	1143
CCCAGTGTTAGCA	0.02	0.003	0.018	39	2080
GCTGGCACATATA	0.222	0.024	0.215	298	1086
AGCGGTATATACG	0.021	0.002	0.023	41	1719
AGCGTTGCAGATG	0.026	0.004	0.021	26	1201
CGCATTGTATGTG	0.03	0.008	0.019	23	1180
AGTATTGTTAGTG	0.02	0.003	0.019	21	1058
GCTGTCGTAAAGG	0.055	0.004	0.05	39	739
GGCATCACAAGTA	0.065	0.003	0.07	148	1978
CCCATCGTATAGG	0.028	0.001	0.026	48	1784
CGTGGCACTAACA	0.023	0.003	0.026	38	1422
GCCGGCACAGAGG	0.201	0.024	0.184	165	734
GCTAGTGCATATG	0.05	0.012	0.051	51	949
ACCATTGCATAGA	0.019	0.001	0.018	57	3051
GGCGGCATTTACA	0.295	0.052	0.267	394	1079
GCCGTCACTTGCA	0.054	0.007	0.059	90	1428
GCCATTGTAGACA	0.045	0.002	0.045	107	2281
ACTGTTACTTACA	0.023	0.002	0.023	65	2763
GGCATTGCTGACA	0.048	0.003	0.044	70	1523
AGCATCACTAGCG	0.025	0.005	0.021	43	2040
GCTGTCACATATG	0.053	0.004	0.054	54	939
GGCAGTGTTGGTA	0.053	0.003	0.054	46	806
AGCGTCGTTTACA	0.016	0.001	0.017	32	1831
AGCGGTGTTAAGA	0.026	0.003	0.03	42	1364
CGTAGTATTTACA	0.023	0.002	0.025	39	1534
CCTATCATAAATG	0.027	0.004	0.023	86	3617
ACTAGTGCTAATA	0.021	0.003	0.021	69	3254
GCCATCACATACA	0.053	0.005	0.051	181	3346

CGCGGTACAAGCG	0.024	0.003	0.021	25	1162
GCTAGCGTAAGTG	0.079	0.019	0.053	41	733
CGTAGTATTGATA	0.024	0.002	0.022	30	1353
ACCATCATTTATA	0.025	0.001	0.024	110	4509
ACCGTTGCTAAGA	0.026	0.005	0.029	64	2174
GCTAGCGCATACA	0.059	0.013	0.048	62	1242
CGCATTGTAAGGG	0.023	0.004	0.026	28	1065
CCCGTCACTTACG	0.023	0.002	0.02	42	2024
AGTGGCACAGGCG	0.028	0.002	0.031	16	500
CGCATCGTTGACG	0.029	0.002	0.026	41	1534
ACTATCGTAGATA	0.023	0.003	0.021	56	2601
AGCATTACAGGCG	0.022	0.003	0.024	49	2030
CGTGTCATTGACA	0.025	0.003	0.03	46	1505
AGCGGTACTGACG	0.018	0.007	0.009	13	1376
GGCGTTACAGAGA	0.062	0.01	0.048	56	1107
CCCAGCGCATGGA	0.024	0.006	0.016	31	1950
GGCATCGTATAGG	0.111	0.014	0.105	109	931
AGCATTATTTATG	0.021	0.003	0.021	55	2610
GCTAGTACAAATG	0.06	0.006	0.065	108	1546
GGCATTACAAATG	0.068	0.004	0.068	157	2157
AGCAGCACATGGA	0.023	0.004	0.02	34	1699
CCTAGCACTTGTA	0.026	0.004	0.023	38	1597
CCTATCATAAAGA	0.027	0.003	0.022	89	3900
AGTGTCGCTGAGA	0.021	0.004	0.024	19	758
GCTATTGTTGATG	0.066	0.014	0.047	52	1065
AGCAGTATAGGGA	0.018	0.001	0.016	34	2065
CCTATTGCTGGTG	0.029	0.009	0.031	35	1082
CGCAGTATTTGCG	0.022	0.002	0.019	22	1150
GCCATTGTAAATA	0.048	0.002	0.044	135	2902
GGTGGCGTTAAGG	0.657	0.044	0.651	423	227
AGCATCACAAGGG	0.025	0.005	0.028	51	1775
CGTAGTATTTGCA	0.018	0.001	0.016	18	1079
CGCGGTACATGCA	0.017	0.001	0.016	25	1542
GCCAGTGTAAACA	0.047	0.004	0.047	130	2638
CCCATCGTATGGG	0.021	0.004	0.021	26	1206
GCCGTCACTAATA	0.049	0.01	0.035	83	2301
CCTGTCGCTAGGG	0.024	0.006	0.026	17	649
GGTATTATTGGGA	0.097	0.004	0.091	74	739
ACCGGCATATGTA	0.026	0.001	0.027	60	2184
GCTATCACAGACA	0.055	0.01	0.042	85	1924

GCCGGTGTATGCA	0.184	0.021	0.187	225	976
CCCGGTGCTTGGG	0.035	0.01	0.021	11	516
GCCGGTACTAGGA	0.066	0.005	0.06	59	927
AGCGTTACTAATA	0.02	0.004	0.016	46	2886
ACTATTGTAAGCA	0.021	0.002	0.02	60	2959
CCCATTGTAGACA	0.026	0.002	0.025	82	3241
ACTAGTGTTGATA	0.029	0.001	0.028	63	2199
CGCATCACAAAGG	0.028	0.001	0.027	56	2009
ACTATTGTAGGCA	0.027	0.004	0.031	67	2076
ACTATTACAAAGA	0.02	0.001	0.02	95	4600
CGCATTACTTGTG	0.023	0.005	0.021	26	1210
CGCATTGTAGATG	0.026	0.003	0.023	35	1480
AGCATCATATGTA	0.022	0.005	0.025	83	3260
GGTAGTATAAATA	0.061	0.004	0.056	128	2162
CCCATTACTAAGA	0.026	0.002	0.024	88	3541
GGCGTTACAGATG	0.053	0.015	0.046	49	1010
CCTATCACTGACA	0.023	0.002	0.019	60	3018
AGTGGTACAAATA	0.023	0.003	0.019	40	2041
CGCAGTACTGGCA	0.024	0.001	0.022	34	1490
GCTGTTGCTAATA	0.059	0.009	0.045	53	1112
ACCGGTATTTACG	0.025	0.002	0.027	53	1893
ACTGGTGCTTGTA	0.02	0.001	0.021	26	1194
ACCGTCGTTGACA	0.025	0.005	0.022	46	2053
CGTAGCGCTTAGG	0.03	0.004	0.024	14	558
CCCATTGCTTACA	0.021	0.005	0.018	54	2991
GCCATTGCTAACA	0.042	0	0.042	99	2235
CCTAGCACTAATA	0.027	0.002	0.027	79	2879
CGCATTGTATAGG	0.027	0.004	0.022	27	1228
GGTGTCACTAGCA	0.076	0.006	0.068	54	744
ACCGTTACAGGGA	0.02	0.003	0.016	27	1710
ACCATTGTAAAGA	0.024	0.001	0.024	88	3561
GCTATCGCTAGTA	0.052	0.005	0.058	64	1035
AGCATCGTTGGGA	0.024	0.003	0.029	36	1219
CGCATCGCAGGTG	0.019	0.004	0.017	16	931
GCCAGCATAAGGA	0.056	0.007	0.048	83	1652
ACTGTTACAGGCG	0.024	0.004	0.022	29	1296
GCTATTATTTGCA	0.058	0.002	0.06	101	1579
GCCAGTACATGTA	0.053	0.001	0.051	99	1829
AGCATTGTAAACA	0.022	0.002	0.02	87	4332
CGTATCATTTGCG	0.023	0.003	0.021	23	1056

ACCGGTGTTAAGA	0.023	0.005	0.017	30	1695
CGTATTACATGCG	0.032	0.003	0.034	42	1177
ACCAGTACAAATA	0.02	0.001	0.019	140	7392
AGTAGTGCTTAGA	0.019	0.003	0.014	13	941
ACTGGCGCTTAGA	0.019	0.003	0.016	10	608
ACCAGCGCTAGTA	0.023	0.002	0.023	63	2723
CGCATCGTATACA	0.024	0.002	0.022	58	2634
GCTAGTGTAAGTG	0.071	0.008	0.064	68	1002
ACTAGTGTTGATG	0.026	0.002	0.022	32	1403
CGTGTCATTGGTA	0.029	0.006	0.021	18	850
GCCGGTATATATG	0.163	0.02	0.163	237	1216
ACCAGCGCTAGCA	0.02	0.002	0.021	63	2987
ACCAGTGCTTGCG	0.02	0.003	0.02	32	1558
GCTATCATATACA	0.065	0.005	0.059	163	2611
ACTGGCATATAGA	0.025	0.005	0.026	43	1619
GCTGTTGCAAGGA	0.063	0.01	0.05	39	739
CGCATCATTGGCA	0.022	0.004	0.018	34	1896
GCTGGTACAGGCA	0.079	0.009	0.073	67	856
GCCAGCGTTTGTG	0.048	0.009	0.06	50	783
GCCAGTGTAGAGG	0.043	0.01	0.057	55	917
AGCATTGCATGGG	0.024	0.001	0.025	29	1144
CCCATTGTAAATA	0.022	0.001	0.022	98	4355
CGTGGTACATACA	0.022	0.006	0.014	22	1532
CCCAGCGTAGAGG	0.025	0.001	0.025	36	1376
ACTAGTACTAGCG	0.022	0.002	0.021	38	1806
AGCGTTACAGATA	0.026	0.003	0.023	58	2424
ACCGGCGCTAATG	0.028	0.002	0.026	38	1396
ACTATCACTGATA	0.025	0.003	0.027	97	3470
ACCGGCATTTACG	0.03	0.009	0.018	31	1730
ACCGTTGCTTACA	0.021	0.002	0.018	47	2573
CGTAGTACTAATG	0.026	0.003	0.024	26	1062
ACTATTGCTAACG	0.025	0.002	0.022	59	2596
GCCGGTGCTTAGA	0.059	0.009	0.049	38	742
AGTGGTGTTTGGG	0.02	0.007	0.013	4	306
CCCGGTGTTAAGA	0.025	0.003	0.027	46	1633
AGCAGCGTTAGCA	0.025	0.002	0.026	48	1776
ACCGGTATAGGGG	0.025	0.004	0.022	19	841
AGTATTGTAGATA	0.02	0.004	0.021	46	2189
CGTGGCACAAGGA	0.027	0.003	0.028	24	823
CCCGTCACTTATA	0.026	0.002	0.023	61	2625

GGCGGTACAGAGG	0.08	0.022	0.069	46	623
GGCATTATAAACG	0.061	0.006	0.062	168	2525
GGTGTCGCTAACG	0.102	0.027	0.097	60	561
AGCGGCGTAGGGG	0.022	0.005	0.029	20	678
GGCAGTATAAATG	0.064	0.002	0.061	131	2011
AGTGGTGCTAGCG	0.028	0.01	0.026	13	482
GCTGTCACAAGCG	0.058	0.008	0.059	57	908
CCCGTTGCTTGGA	0.021	0.003	0.025	28	1090
CCTGTCATAAACG	0.025	0.003	0.022	56	2507
GCTGTCACAAAGA	0.058	0.002	0.06	87	1360
CGCAGTGCTTAGA	0.026	0.003	0.03	32	1035
GGCAGTGCTTATG	0.058	0.01	0.046	40	821
GGTGTTATTAATA	0.087	0.01	0.073	108	1365
AGTAGCATAAACA	0.021	0	0.021	65	3054
GCTATTGCAAATA	0.049	0.003	0.053	118	2123
GCCGTCGTTGGCA	0.058	0.007	0.059	59	945
ACTGTTGTATGTG	0.028	0.002	0.028	30	1033
ACTATTGCTTGTG	0.02	0.004	0.015	22	1432
CCCGTTGCTAACA	0.025	0.003	0.029	89	2973
AGCAGCACATGCA	0.026	0.003	0.024	55	2223
CGTGGCGTATAGA	0.022	0.009	0.014	9	623
CCTATCGTAGATG	0.023	0.002	0.025	45	1754
CCTAGCGCTTATA	0.019	0.001	0.018	37	2011
GGCATCGTTAACA	0.1	0.013	0.085	195	2094
AGCGTCATTGAGG	0.022	0.003	0.026	30	1120
ACTAGTACTTGTA	0.024	0.002	0.026	63	2358
AGCATCGCTAAGG	0.024	0.001	0.025	39	1515
GCTGTTGTTGATA	0.051	0.013	0.051	55	1025
CCCGTCACAGAGA	0.03	0.005	0.03	66	2139
AGTGGTATATGCA	0.022	0.006	0.018	22	1195
CCCAGTGCTGAGG	0.024	0.005	0.02	22	1071
CGTATCACATATA	0.022	0.004	0.017	34	1917
ACTAGTACAAAGG	0.027	0.002	0.025	61	2363
ACTATCATAGGCA	0.027	0.004	0.023	54	2333
GCCAGCGTAAACA	0.049	0.004	0.046	129	2692
ACTAGTGCTAGGG	0.026	0.006	0.022	22	973
ACTGTTACTAATA	0.023	0.006	0.016	55	3302
ACTGGTGTTGGCA	0.019	0.001	0.018	18	992
CGTGTTGTAAATG	0.026	0.004	0.023	25	1081
GGTAGTACTGACA	0.054	0.005	0.05	59	1112

GGTGTCGCAGACG	0.114	0.016	0.092	52	515
ACTGGCACTAATG	0.027	0.002	0.027	40	1427
GGTATTGCAAGTG	0.099	0.005	0.093	65	633
CCTAGTACTTAGG	0.027	0.001	0.025	28	1093
CCTAGTATAGACA	0.028	0.002	0.03	91	2951
CGCGTCATTGGGG	0.031	0.004	0.032	20	596
AGTGGCGCTGAGA	0.012	0.003	0.015	10	668
CCCGGTGCTGAGA	0.029	0.008	0.021	28	1334
CGCGGCGCTGGCA	0.02	0.001	0.02	19	943
ACCATCATATGCA	0.024	0.003	0.027	113	4070
CGCATCATTAGTG	0.026	0.002	0.029	48	1587
GGCGGTATAAGTA	0.077	0.014	0.076	105	1283
CGCAGCATTAATA	0.023	0.002	0.025	76	2943
CGTATTATTAGTG	0.025	0.005	0.025	31	1222
CGTAGCGCTTACA	0.022	0.004	0.019	21	1089
CCCGTCATTTAGA	0.027	0.007	0.019	39	2042
ACCAGTATAGGCG	0.024	0.002	0.027	61	2231
AGCATCGTTAGCG	0.017	0.001	0.018	27	1470
CCTGTTATATGGG	0.026	0.007	0.026	25	952
AGCAGCACTTGCA	0.024	0.003	0.021	38	1789
ACCAGTGCATAGA	0.021	0.001	0.02	55	2672
ACCGTTACAAATG	0.025	0.004	0.024	91	3714
CCCGTTGTATGTG	0.025	0.005	0.018	23	1234
CCCATTGCTAGCA	0.022	0.003	0.023	62	2643
CGCGGTACTTGTG	0.031	0.009	0.04	30	712
CGCAGCATTTGCA	0.021	0.005	0.015	26	1734
CGCAGTGTTGATA	0.021	0.002	0.022	34	1490
GCCGTCATAGACA	0.052	0.005	0.045	110	2339
CCCGTCGCATACA	0.022	0.001	0.021	64	3003
GGCAGTGTATGCG	0.045	0.01	0.039	33	817
ACTATTGCTAACA	0.027	0.005	0.028	110	3878
CGCATCGCTAACA	0.025	0.003	0.024	63	2576
GCCGGTGCAGGTG	0.095	0.014	0.076	46	563
ACTGTTACATATG	0.023	0.005	0.02	39	1882
CGTAGCATTAACG	0.02	0.003	0.016	22	1326
ACCGTCGTAGATA	0.024	0.001	0.025	53	2092
ACCATCGTAAGTG	0.026	0.004	0.022	44	1981
AGTATTATTGATG	0.02	0.003	0.017	30	1766
CCCGTCATTGGTA	0.024	0.004	0.029	51	1683
AGCGGCGCATGGA	0.023	0.007	0.016	14	844

CCCGGCGCTTGTG	0.023	0.001	0.022	21	935
GCCAGTGCAAAGA	0.039	0.005	0.035	56	1564
CCCATCGTTGGCG	0.024	0.003	0.021	38	1771
AGCGGCGCATGCA	0.023	0.001	0.023	29	1257
CGCAGTATATGCA	0.025	0.003	0.025	55	2129
CGTATTACAGGCG	0.026	0.001	0.024	28	1127
CGCGTCGTTAGCG	0.026	0.008	0.019	18	912
ACTAGCACTGGTA	0.024	0.004	0.026	53	1958
ACCAGCGTTGACA	0.02	0.004	0.017	42	2400
CCCGTTGTATACA	0.024	0.004	0.02	52	2576
GGCGGTACTGGCG	0.069	0.009	0.056	37	627
GGCATTACTAATG	0.059	0.012	0.044	77	1657
CGTATCATTGATA	0.023	0.003	0.026	52	1933
AGCATCATTGGGG	0.015	0.002	0.015	23	1469
CCTAGTACTGAGA	0.018	0.004	0.019	33	1683
CGCATCATATAGA	0.021	0.001	0.02	55	2642
ACCGGTATATATA	0.024	0.002	0.021	77	3594
ACCGTTACAGACG	0.024	0.002	0.025	71	2773
GCCGGCGTATGTA	0.85	0.025	0.855	1440	244
CGCATCGTAGACG	0.031	0.005	0.038	68	1726
GGTGTCATTTACG	0.083	0.016	0.06	37	581
GGCGTTATAGGCG	0.063	0.004	0.056	50	837
GGCGGCGTTAATG	0.442	0.06	0.413	441	627
CCTAGTACAAGCA	0.022	0.001	0.021	56	2625
AGTATCGCTAAGA	0.019	0.002	0.016	22	1339
ACTATCATTAACG	0.03	0.002	0.029	91	3059
GGCAGCACTTACA	0.054	0.011	0.041	67	1576
AGCATCACTTAGG	0.022	0.002	0.023	36	1561
GCCAGTATAGAGA	0.055	0.006	0.047	101	2064
AGCGTTGCTGGTA	0.018	0.004	0.015	16	1082
ACTGGCGCAAAGA	0.031	0.006	0.036	39	1054
GCTATTACAAACG	0.06	0.005	0.053	120	2153
AGCATTGTAAGCG	0.02	0.004	0.026	53	1957
CGCGGTATAAAGA	0.027	0.003	0.025	50	1939
CCCAGCACATGGG	0.042	0.006	0.051	69	1291
GGCATCACTTGTA	0.068	0.015	0.067	97	1353
AGCGTCATAGGTG	0.024	0.004	0.021	26	1202
AGTAGCATAGGCA	0.025	0.001	0.026	37	1401
CCTATCATAAATA	0.023	0.001	0.021	113	5232
CCTGTCGTTTACG	0.024	0.002	0.021	26	1217

AGCAGTACTAGGG	0.029	0.005	0.022	26	1151
ACTGGTGCTTACA	0.023	0.002	0.02	35	1721
CGCGGTGTTTGCA	0.023	0.003	0.018	17	904
AGCAGTATATGTG	0.019	0.001	0.02	39	1925
GCCATTGCTTATA	0.041	0.005	0.042	70	1604
CCCATCATAGATG	0.027	0.003	0.028	83	2923
ACTATTATAGAGA	0.026	0.003	0.023	73	3163
AGCATTACTAAGA	0.023	0.002	0.025	81	3112
ACTGTCACATGTG	0.028	0.008	0.02	24	1165
CCTGTTGTTAGGA	0.026	0.004	0.03	33	1064
GCTATTACAAATG	0.067	0.005	0.06	131	2057
AGCGTTGCTGATA	0.028	0.003	0.025	40	1553
AGCGTCATTGGTA	0.019	0.004	0.015	22	1489
GCCGTTGCATGGG	0.055	0.012	0.052	26	475
CGCGTCGTATGGG	0.022	0.003	0.025	16	637
CCTGTTACTGACA	0.025	0.002	0.023	49	2116
GCTGTCACTAAGG	0.069	0.003	0.071	57	744
GGTATTGTTGAGA	0.102	0.012	0.097	86	803
CCCAGTACAGGCG	0.023	0.004	0.028	53	1859
GCTGTCATTAGTG	0.065	0.007	0.056	42	707
AGCGGTGTAAATG	0.02	0.003	0.017	27	1532
CGTATCGCTAATG	0.027	0.001	0.026	30	1132
CGTATTACTGAGG	0.032	0.005	0.026	26	978
GGCGTCACTGACA	0.062	0.01	0.057	89	1464
ACTGGCATATGTA	0.029	0.003	0.026	45	1715
ACCAGTGTTGAGG	0.03	0	0.03	36	1181
GGCAGTATATACG	0.067	0.002	0.066	115	1622
CGTGGTGCATAGA	0.026	0.009	0.02	15	726
CCTAGTGTAAACA	0.023	0.007	0.018	65	3494
ACTAGTGCAAGTA	0.02	0.004	0.015	40	2670
CCCGTTATAAGCA	0.024	0.001	0.026	82	3059
AGCGTTGCAAGCG	0.027	0.009	0.02	26	1269
AGTGGTGTTTACA	0.023	0.004	0.019	18	944
GCTATCACAGATG	0.062	0.006	0.053	74	1311
ACCGTTGCATAGA	0.023	0.002	0.024	52	2089
CGCATTATTGGCG	0.028	0.003	0.024	33	1361
AGTAGCACAAGGA	0.02	0.003	0.023	30	1261
ACTATTACTGAGG	0.028	0.006	0.021	40	1883
CCCGGCACTTGTA	0.022	0.003	0.02	34	1649
CCTAGTATTGGGA	0.024	0.006	0.02	24	1162

GCTGTCGTTGAGA	0.059	0.003	0.058	40	653
CCCAGTACAAGCA	0.025	0.002	0.026	103	3820
CCTGGCGTAAATG	0.023	0.003	0.019	27	1373
GCCGGTGTATGGG	0.352	0.067	0.337	198	390
GCCATCGTAAAGA	0.053	0.005	0.047	100	2030
CCTAGTGCTAAGA	0.032	0.005	0.027	51	1834
ACCGGCGTAAAGA	0.024	0.002	0.023	40	1683
CGTATCGTAGACG	0.029	0.004	0.025	33	1292
GGCGGTGCTAGCA	0.058	0.015	0.054	44	775
ACCATCATTAATG	0.025	0.001	0.024	99	4029
AGTATTGCTAATA	0.025	0.001	0.027	56	2047
GGTATCACTGGGA	0.09	0.018	0.065	41	589
ACCGGCATTTGTA	0.023	0.002	0.02	37	1768
ACTGGTACTGGCA	0.03	0.006	0.033	57	1665
GGTGTTGTAGGGA	0.087	0.013	0.085	37	399
ACCAGCGTTGATG	0.023	0.003	0.022	31	1410
GCTATCGTAGACG	0.071	0.005	0.066	82	1161
AGCATCATAAACA	0.021	0.002	0.02	122	6037
AGCGTTGCATACG	0.027	0.009	0.016	26	1576
AGTAGCACTTAGG	0.028	0.01	0.017	12	683
CGCGGTGCTTAGA	0.023	0.003	0.026	23	867
CGTGGTGCAGGTG	0.029	0.012	0.016	7	420
GCCAGCGTATGTA	0.055	0.012	0.057	81	1337
ACTATTGCAAGTA	0.027	0.003	0.026	79	2989
CCCGGTACTAGGG	0.027	0.01	0.035	37	1008
AGTATCGTAGACA	0.024	0.001	0.024	50	2029
CCTATCATAAGGA	0.03	0.003	0.033	81	2397
CCCGGTACTAGCA	0.027	0.001	0.028	62	2155
AGTGTTACTTGCA	0.021	0.005	0.027	30	1089
AGCAGCGTAAATA	0.019	0.004	0.019	64	3314
AGCAGTACTTATA	0.02	0.002	0.02	60	2871
GCCGGTATTGATG	0.162	0.014	0.154	188	1033
ACTATTACAAGTG	0.023	0.001	0.022	59	2565
AGCATTACAGATA	0.024	0.001	0.025	90	3560
CGTATCATATACA	0.025	0.002	0.022	62	2777
ACCAGCGCAGAGG	0.021	0.001	0.021	30	1422
GGCATCGCTTACA	0.08	0.017	0.062	95	1446
AGCATCATAAGGG	0.022	0.003	0.025	61	2372
CGCGTCACTTAGG	0.028	0.004	0.025	24	934
AGTGTTACAGGGA	0.028	0.007	0.02	17	851

GGCGGTATTGGTA	0.072	0.01	0.06	52	812
GGCATCATTAGGG	0.061	0.013	0.043	46	1015
CGTATCATATGCA	0.026	0.003	0.022	41	1790
CGCATCATTTGTG	0.022	0.001	0.021	30	1377
AGCGTCACAGGCG	0.023	0.002	0.022	28	1231
AGCATTGCATGCG	0.019	0.002	0.021	33	1504
CCCGTCGCATATG	0.026	0.003	0.028	45	1561
GGCGGCACTAAGA	0.119	0.023	0.104	124	1074
AGCGGCACTTAGA	0.022	0.005	0.027	33	1205
GCTATCATATGTA	0.066	0.008	0.055	101	1737
CGTATCGTTGGCA	0.024	0.002	0.024	26	1050
CCTATTGCAAACG	0.023	0.003	0.019	54	2741
ACTAGTATAGACA	0.032	0.001	0.033	112	3332
ACCATCACAGGTG	0.03	0.004	0.025	56	2199
GGCGTTGCAGGGG	0.066	0.007	0.055	24	409
ACCGTCACTTAGA	0.023	0.002	0.025	64	2534
GGCAGCACTGAGG	0.053	0.008	0.055	47	808
ACTAGTGCAAGGA	0.023	0.003	0.022	42	1882
CCTGTCATAGATG	0.028	0.001	0.027	42	1493
AGTATCGTATGTA	0.023	0.003	0.018	28	1495
CGTGTTGTTGATA	0.023	0.003	0.028	28	973
CGCATCACAGGCA	0.027	0	0.026	59	2182
ACTGTCGCAAGTG	0.026	0.001	0.026	31	1141
CGCATTGCTGGGA	0.025	0.008	0.035	32	880
CCCAGTACAGGTA	0.03	0.002	0.032	78	2329
CGCGTTATTAGCG	0.025	0.005	0.022	26	1177
CGCGGCGTTGACA	0.031	0.004	0.028	38	1342
AGCGTCGTATGGG	0.019	0.009	0.022	18	804
CCCGTTATTTACG	0.026	0.005	0.019	41	2074
CGCAGTATAGACG	0.027	0.005	0.031	59	1840
AGCAGTGTAAGGG	0.019	0.006	0.027	31	1101
ACCATTATATATA	0.024	0.001	0.026	159	6034
ACCATTGCTAGCG	0.022	0.001	0.023	52	2208
GCCAGCGTTGGTG	0.05	0.003	0.051	35	654
ACTGTTACAGGTA	0.026	0.003	0.03	55	1766
ACTGGCACATAGG	0.032	0.003	0.03	31	1007
CCCGGCACAAACG	0.027	0.004	0.023	67	2864
ACCAGTGTATGTA	0.025	0.002	0.024	55	2223
CCTGGCGCAGAGG	0.025	0.003	0.023	17	728
GGCAGCGTAAGGA	0.06	0.003	0.06	60	942

CCTAGTGCTGACA	0.024	0.002	0.021	43	2045
CGTGTCATTAAGA	0.018	0.002	0.017	23	1354
AGCATTACAAGGG	0.024	0.002	0.024	45	1812
CCCAGTGCTAAGG	0.029	0.006	0.024	34	1372
ACCGTTATAAAGA	0.021	0.003	0.019	74	3801
AGCAGCGCATACA	0.025	0.001	0.023	60	2545
CGCGGCGTTGGTG	0.035	0.01	0.027	13	467
CGTATTACTGGGA	0.032	0.006	0.025	22	870
CGTATCGCAAACG	0.028	0.009	0.022	35	1561
CCTATCGTTAATG	0.027	0.005	0.022	44	1921
CCTAGCGTAAACG	0.025	0.004	0.021	42	1981
AGCAGCGCAAATA	0.019	0.002	0.02	60	2888
ACCAGCATATGCA	0.024	0.002	0.026	95	3513
CGTAGTGTATACG	0.026	0.004	0.021	20	951
CGTGGCGTATACA	0.024	0.007	0.018	19	1019
CCTGGTGTTTAGA	0.026	0.006	0.018	18	990
CCCAGCACTAATA	0.025	0.002	0.022	88	3867
CCCGGCATATGCA	0.025	0.004	0.021	49	2248
CCTGTCGCTTGGG	0.022	0.006	0.028	16	564
CCTATTATAAGCG	0.027	0.006	0.019	48	2443
CCCAGTGTTAATA	0.024	0	0.024	71	2908
CCTAGTGTTAATG	0.026	0.005	0.023	44	1893
GGCAGTACAAGTA	0.05	0.003	0.047	91	1856
GCCAGCACTAGGG	0.052	0.006	0.043	38	839
CCTGGTGCTGATG	0.026	0.005	0.027	23	842
AGCATTATTGATA	0.019	0.001	0.02	78	3827
ACTATTACAGACA	0.027	0.003	0.03	141	4635
ACTATTACTGGGA	0.026	0.002	0.026	44	1619
CCCATCACATAGA	0.022	0.004	0.02	70	3370
AGCATTGTTTGTA	0.02	0.002	0.018	33	1814
CCCGTTGTTAGTG	0.022	0.007	0.028	32	1104
CCCGGTATAGGCG	0.031	0.005	0.025	34	1347
AGTGGCACTGAGA	0.026	0.005	0.021	17	776
CGCGGTATTTATG	0.026	0.005	0.032	35	1047
ACTGTCGTTAGTG	0.028	0.001	0.029	22	747
ACTGGTGTTTATA	0.027	0.003	0.031	44	1398
GGCGGCATTGACG	0.497	0.06	0.485	642	681
GCTGGTGCAGGCA	0.129	0.02	0.143	97	579
AGCGGTACAGGTA	0.014	0.002	0.014	21	1465
CCTGTCGTAGGGG	0.035	0.006	0.034	21	604

CCTATCGCATATA	0.028	0.002	0.026	73	2721
ACCAGCGCTGGGA	0.02	0.001	0.019	27	1382
GGCATCGTAAATA	0.113	0.016	0.094	247	2375
AGTGTTGTATGGA	0.027	0.004	0.022	18	786
CGCAGCATATGCG	0.027	0.006	0.027	38	1381
ACCGTCATAAATG	0.026	0.004	0.022	70	3168
AGTAGCGTAGGCA	0.018	0.003	0.015	15	996
ACCAGCACTAACA	0.023	0	0.023	146	6133
CCTAGCACTTACA	0.025	0.005	0.019	47	2460
GGCAGTATTAAGG	0.06	0.007	0.057	74	1231
AGTAGCGCTAGTG	0.026	0.004	0.025	14	552
ACCAGTGCAGGCA	0.023	0.004	0.022	53	2362
AGCGGCACTGATA	0.023	0.002	0.021	33	1506
CGTGTTATTGGCA	0.022	0.004	0.017	17	963
AGTGTCGCAAGCA	0.021	0.002	0.023	27	1123
GCTATTGTTAGCG	0.061	0.002	0.058	64	1034
ACCAGTGCATGCA	0.021	0.001	0.02	60	2870
CGTATCGTAGATA	0.026	0.004	0.027	47	1692
ACTAGTACAGACA	0.021	0.003	0.02	80	3917
GCCATTGTAAGCG	0.051	0.01	0.039	60	1469
AGCAGTGCATGCG	0.024	0.005	0.018	24	1299
CGTGGTGTAGATG	0.029	0.016	0.008	4	525
CGCATTACTAGTG	0.025	0.006	0.034	45	1293
CCCGGTATTAGCA	0.026	0.004	0.029	65	2148
CGCGGCATAAACA	0.026	0.002	0.024	65	2661
GGTATCATTAACG	0.131	0.011	0.117	143	1078
CCCGGTACTGGTA	0.025	0.002	0.028	44	1536
ACTGTTATTAAGA	0.026	0.003	0.023	56	2426
GCCGTCGTATACA	0.051	0.003	0.056	99	1676
CGCGGTGCTTACA	0.02	0.004	0.017	22	1285
AGCGGTGCATGCG	0.021	0.008	0.016	16	956
ACCATCGCAGACG	0.022	0.004	0.025	63	2473
CCCATCGTTTGCA	0.022	0.004	0.02	46	2286
CGCGGCGCAAAGG	0.024	0.001	0.025	24	951
ACTATCGTTGGCA	0.028	0.004	0.023	37	1564
GCCGGTGTTAGCA	0.174	0.031	0.175	207	974
CGTAGTATTTATA	0.027	0.006	0.021	32	1513
CGCGGCGCATATG	0.024	0.004	0.029	27	912
CGCAGTATAGATG	0.027	0.004	0.033	60	1749
CGTATTGCTAAGG	0.027	0.004	0.022	19	857

ACCATCGTAGGGA	0.028	0.007	0.027	44	1568
CGCGTTATAAGTG	0.027	0.001	0.028	39	1333
AGTATTACTGGTA	0.021	0.005	0.028	40	1397
CCTATCACATGTG	0.029	0.006	0.029	58	1929
AGCGTTACATGGA	0.026	0.002	0.025	35	1357
GGTATTATAGACA	0.106	0.007	0.098	200	1838
CGTGTTGCATGTA	0.022	0.001	0.023	21	891
GGCGTTGCTAAGG	0.074	0.009	0.066	47	667
CGCATCACTTACG	0.023	0.002	0.02	37	1844
CCCGGTACATACA	0.023	0.002	0.024	81	3276
ACTAGTGCATACA	0.024	0.004	0.022	76	3306
AGCGGCGTTTGTG	0.022	0.01	0.009	5	526
GCCGGTGTAAAGG	0.366	0.051	0.334	365	727
AGTAGTGCAAAGG	0.021	0.003	0.02	19	948
GCTAGTACAGGTA	0.055	0.01	0.048	57	1141
ACTAGCGCTAAGG	0.024	0.009	0.017	21	1208
GCCAGCATTAATG	0.051	0.002	0.053	119	2137
ACTGTCATATACA	0.026	0.002	0.026	77	2915
GCCGGTACATGTA	0.065	0.006	0.058	84	1352
ACCATTGCTAATA	0.022	0.002	0.021	94	4363
GGCATCACATGCG	0.081	0.011	0.066	91	1282
ACTGTTGCAGGTA	0.029	0.002	0.032	47	1418
GGCAGTACTAAGG	0.055	0.005	0.062	69	1041
CGCATCGCAAGCG	0.024	0.008	0.026	40	1516
GGCGTCACTAGTA	0.059	0.012	0.045	55	1170
ACTAGCACTGATA	0.021	0.004	0.014	40	2765
ACTGGCATATATG	0.029	0.003	0.033	45	1335
ACCGTCGTATGTA	0.027	0.002	0.024	43	1759
CGTGGTGCTGAGA	0.017	0.006	0.009	6	670
CCTGGTATTTGTA	0.023	0.003	0.028	35	1228
ACCAGCACAAGCA	0.023	0.003	0.019	93	4809
CCCGTTACAGGTA	0.022	0.002	0.022	45	2045
ACCGGTGTAAATG	0.025	0.002	0.022	43	1887
CGTATTGCTTACG	0.028	0.009	0.016	16	985
CCCGGCACTAATA	0.027	0.002	0.025	72	2823
CCCAGTACTAACA	0.025	0.004	0.019	89	4597
CGTGGTGTAAACA	0.026	0.003	0.025	34	1347
CGCAGTGCTAACG	0.028	0.002	0.026	38	1409
CCCGTTGTTGATA	0.023	0.002	0.02	39	1870
CGTGGCACTTGGA	0.021	0.006	0.019	10	508

ACCGTTACTAGCA	0.019	0.001	0.019	63	3289
ACCAGCGCAAATA	0.023	0.001	0.022	113	5063
ACCATCACAGGCA	0.021	0.002	0.019	77	3926
AGCAGCGCTTGTG	0.024	0.003	0.027	27	982
AGCGTTGTTAGCA	0.027	0.005	0.033	51	1476
CCCAGTGCATGCG	0.026	0.002	0.024	37	1495
ACCGTTGCATGCA	0.026	0.002	0.029	67	2230
ACTATCACTTATG	0.029	0.006	0.025	61	2346
GGCGTTATAAGCG	0.064	0.012	0.068	76	1038
AGCGTTGCTTACG	0.017	0.004	0.012	15	1250
ACCGTCATAGACA	0.024	0.002	0.025	99	3809
GGTAGCATTTATA	0.082	0.005	0.076	93	1136
CGCAGTACAAGCA	0.027	0.007	0.023	55	2294
GCTGTCATTTACA	0.059	0.007	0.062	88	1335
CCCATTGCTTAGA	0.022	0.003	0.019	38	1944
AGCGGCACATGGG	0.016	0.005	0.011	7	614
GGTAGCGTAGATA	0.081	0.011	0.065	58	839
CCCGTTGCTTGTA	0.023	0.002	0.022	33	1476
ACCAGCACTGGCA	0.022	0.002	0.021	52	2484
GGTATCATAAGGG	0.116	0.022	0.093	66	643
CCTGGTGCTAGGA	0.027	0.003	0.026	24	914
ACTATTACAAACA	0.023	0.002	0.02	135	6568
GCCGTCATAGATA	0.053	0.007	0.043	96	2119
CCTGTCGCTGGTG	0.027	0.007	0.026	17	644
GCTAGCATAAACG	0.071	0.003	0.07	137	1813
AGTGTCGTTGGCA	0.025	0.004	0.024	19	785
ACCATCGTTGGTG	0.026	0.006	0.028	34	1178
ACCGTCATAAAGA	0.024	0.002	0.025	90	3535
CGCATCACAAGGG	0.027	0.007	0.02	26	1272
CCCGGCACTTGGG	0.03	0.004	0.025	24	924
ACTAGCATAAAGA	0.029	0.002	0.027	88	3182
CGCGTTGCTTGTA	0.024	0.005	0.022	22	958
AGCATCGCAGGCA	0.025	0.005	0.029	58	1972
CGTATCGCATAGA	0.027	0.002	0.024	30	1199
CGCGTTACAAGGA	0.02	0.005	0.025	37	1453
CGCGTTACAGGTG	0.027	0.004	0.027	25	905
AGTAGTGCTAGGG	0.02	0.005	0.026	14	528
GCTGTTATATATG	0.073	0.007	0.081	100	1131
AGCATTGCTAAGG	0.022	0.002	0.022	35	1564
GGCATTATATACA	0.057	0.01	0.046	143	2943

GCCAGCATTAAGG	0.054	0.004	0.048	79	1554
AGCAGTGCAGATG	0.022	0.002	0.02	29	1453
GCCATCGCATGCA	0.058	0.003	0.057	84	1387
CCCAGTACAGGGG	0.041	0.002	0.039	46	1119
CCCAGTACTAGCA	0.026	0.003	0.024	74	3065
CGCAGTATTAGTA	0.02	0.004	0.02	39	1893
CCCGGTATATACA	0.026	0.002	0.028	90	3088
AGCGGTGCTAACA	0.02	0.005	0.014	29	2056
ACTGGCATTGATG	0.026	0.005	0.029	32	1075
CGCGGCGCTGGGA	0.02	0.003	0.015	10	641
ACCGTCACTGACA	0.022	0.002	0.019	73	3683
CCTATTGTATGCG	0.025	0.004	0.024	39	1618
GGCATTATTTGCA	0.057	0.004	0.052	87	1575
CGTGGTGTTTGGG	0.02	0.009	0.011	3	268
AGCATTACTAACA	0.02	0.001	0.02	90	4378
ACTGTTGCATGCA	0.02	0.005	0.013	22	1662
AGTATCACATATG	0.031	0.006	0.029	43	1433
AGCGGTGCATGGA	0.016	0.002	0.013	12	920
GGCGGTGTTGACG	0.077	0.01	0.066	47	667
CGTATTACTGGTG	0.025	0.009	0.017	14	791
AGTATTGTTTACG	0.02	0.003	0.016	22	1347
ACCGGTGTTAATG	0.027	0.009	0.016	24	1518
GCCATCGTATGCG	0.046	0.009	0.038	52	1300
GCTAGTACAGGCG	0.05	0.011	0.053	49	872
ACCGGCATTAAGA	0.021	0.004	0.016	40	2488
AGCATCACTAACG	0.022	0.002	0.024	78	3151
AGCAGTACAAGCG	0.022	0.002	0.023	51	2122
AGTAGTGCTTGTA	0.023	0.004	0.029	26	880
ACTAGTGCTAAGG	0.026	0.002	0.028	43	1498
CCCGTCGTTGAGG	0.022	0.003	0.019	20	1037
CCCGGTGTTAATA	0.022	0.001	0.024	48	1956
CCCATCACTGGTG	0.026	0.002	0.027	42	1495
CGCGTTATAAACA	0.022	0.002	0.02	69	3447
GCTGTTACAAGTA	0.06	0.006	0.054	72	1265
CCCGGCACTGAGG	0.032	0.004	0.027	34	1209
AGCATTGTATGCA	0.019	0.006	0.01	24	2273
CGCGTCACTAAGA	0.021	0.004	0.016	28	1735
ACCGGTGTAAGCA	0.023	0.003	0.02	43	2130
GCCGGTGCATGCA	0.06	0.005	0.054	58	1013
CGTAGCGTATGCG	0.025	0.003	0.021	15	692

CCCGGCATTAGCA	0.026	0.004		0.029	66	2215
CGCGGCGTATGTG	0.028	0.008		0.029	20	673
CCCGTTATTAATA	0.026	0.001		0.028	101	3560
ACCAGCATAAATA	0.022	0.001		0.022	136	6100
CCCAGCACTGATG	0.034	0.006		0.041	83	1953
CCCGTTATATGCG	0.023	0.007		0.022	41	1785
ACCAGCGTAGGCG	0.025	0.005		0.028	40	1380
CGCATTATTGGCA	0.026	0.004		0.022	42	1827
CGTAGCGTTTATG	0.032	0.007		0.023	17	709
ACCAGTGCTTGTA	0.026	0.004		0.025	55	2112
ACTAGTGCAAAGG	0.023	0.001		0.025	46	1808
ACCGTTGTAAAGA	0.024	0.001		0.026	65	2461
AGTAGTGCAAATA	0.028	0.001		0.028	56	1977
ACTGTTGCAAATG	0.026	0.005		0.022	45	1972
CCCAGTATTGATG	0.025	0.002		0.026	62	2295
ACCAGCGCTTATA	0.025	0.003		0.026	84	3118
CCCATCGCAGGCG	0.024	0.002		0.023	42	1751
AGCGGTGTTTACA	0.022	0.005		0.024	40	1647
ACCAGTATAAAGG	0.019	0.003		0.015	50	3214
CCTGTTATTTGTG	0.025	0.008		0.015	17	1112
CCCATCGCAGGTG	0.026	0.004		0.027	39	1407
CCCGTCATTTGTA	0.026	0.003		0.027	52	1871
ACCATTACTAGTG	0.02	0		0.02	56	2803
GCCATTGTAAAGG	0.043	0.004		0.038	60	1522
GCTGTCGCAGGCA	0.053	0.007		0.043	31	685
AGCGTCACTAGGG	0.021	0.005		0.023	22	950
GGCAGTGTAAACG	0.052	0.007		0.045	69	1468
ACTAGTACTAACA	0.023	0.001		0.022	105	4695
ACTATTGCTGGCG	0.023	0.004		0.02	34	1650
CCCAGCACAAATA	0.026	0.001		0.025	126	5012
CGCGTCATAGGCG	0.029	0.003		0.033	35	1034
GCCGTTATTAATA	0.057	0.005		0.051	139	2581
CGCGGCACTAGCG	0.025	0.008		0.016	14	859
CCTGGTACTGATG	0.024	0.003		0.02	23	1125
GGCGTTATTGGCG	0.047	0.005		0.054	46	808
CCCGTTGCTAACG	0.025	0.002		0.022	46	2076
GGCATTACTTAGG	0.062	0.006		0.057	58	963
AGTATCACAAACG	0.025	0.004		0.03	58	1869
AGCGGCACTGACA	0.029	0.005		0.028	57	1952
GCTATCGTATATG	0.061	0.007		0.07	88	1170

AGCGGTGTTGGTA	0.024	0.002	0.025	25	959
AGCAGTATAAAGA	0.023	0.001	0.024	96	3981
CCTGTCGTAAAGG	0.025	0.001	0.023	28	1178
CGCAGCATATGTA	0.03	0.005	0.023	46	1987
CCTGTCGCTTACG	0.024	0.003	0.024	28	1140
GGTAGCGCAAACG	0.088	0.008	0.079	67	781
GCTAGCATTTACG	0.066	0.009	0.059	70	1122
CGCATCGCTTAGG	0.02	0.004	0.024	23	941
CCTGGCGCTAACA	0.022	0.003	0.018	31	1661
CCTATCATAGAGA	0.024	0.004	0.021	59	2724
CCCAGCATTGATA	0.023	0.001	0.023	72	3085
ACCATTGTATACA	0.027	0.003	0.03	130	4216
CCCGTCGCTAGGG	0.022	0.002	0.021	19	890
CCCGGCGCTAATA	0.022	0.003	0.019	46	2319
CCCAGTACAAATA	0.026	0.003	0.024	128	5253
GCTAGCGCTAGTG	0.068	0.001	0.068	37	508
CCCGTTGCATGGA	0.026	0.007	0.025	34	1300
AGCGTCACAAATG	0.022	0.002	0.019	45	2269
CCTATCGCAGACA	0.023	0.003	0.019	55	2828
AGTGTTGCTTAGA	0.023	0.002	0.021	17	783
GCTATTGCATAGA	0.052	0.005	0.048	61	1211
CCTATTATAGGCG	0.027	0.002	0.026	53	1969
GGCATTACAAAGA	0.06	0.002	0.063	158	2361
ACTAGTGCTTGCG	0.023	0.005	0.028	35	1212
GGCGGTATTAAGA	0.074	0.011	0.059	74	1187
GGCGTCACAAGTG	0.066	0.01	0.055	57	976
AGCGTTGTATATA	0.018	0.005	0.011	25	2179
CCCAGCGCAGATA	0.022	0.003	0.026	80	2969
ACCGTTACTAAGA	0.022	0.002	0.024	88	3623
CCCAGCGCTTGGG	0.023	0	0.023	21	892
CCCATCATATAGG	0.031	0.005	0.026	61	2243
ACTATTACATATA	0.024	0.003	0.024	123	5036
ACTGGCACTAAGA	0.028	0.003	0.027	47	1700
ACCGGTATATATG	0.031	0.005	0.026	55	2060
ACCGTTACTTATG	0.023	0.002	0.023	59	2475
CCTGTCACAGGTA	0.023	0.003	0.026	41	1516
AGCAGCGCTAGCA	0.024	0.006	0.02	34	1686
ACCATTGTTAGCA	0.025	0.001	0.027	70	2565
AGTGGCATTGATA	0.022	0.004	0.016	14	879
CGTATTATTTATA	0.022	0.007	0.013	30	2369

ACTATCGCATACG	0.025	0.003	0.021	45	2052
CCCATTGCTAGTA	0.02	0.001	0.021	50	2287
CGTATCACTTACA	0.028	0.006	0.036	65	1753
AGCGTTGCATATA	0.02	0.003	0.016	32	1930
CCTAGCGCAAACG	0.029	0.008	0.018	35	1950
CGTATCATTAAGG	0.032	0.01	0.025	35	1375
GGCAGCGCAGACA	0.044	0.004	0.04	58	1398
CCTAGTGCTAAGG	0.025	0.001	0.025	33	1277
ACCGTCGCAAATG	0.026	0.001	0.025	58	2227
GCTGTCGCAAACG	0.054	0.002	0.051	50	934
ACCGTCACTTGTA	0.023	0.001	0.023	60	2495
GGCGTTATTAGGA	0.061	0.009	0.049	45	882
AGCATTGTTTGCG	0.024	0.004	0.018	26	1410
AGCATCATAAGTA	0.023	0.005	0.026	103	3908
ACTGTCACTAATG	0.026	0.004	0.021	38	1788
ACTAGCGCATATA	0.024	0.004	0.021	59	2741
GCCAGCACTAACG	0.055	0.005	0.054	102	1787
CGCGGCGTTAAGA	0.023	0.003	0.024	25	1036
CCTGGCGCAAGTA	0.027	0.002	0.026	35	1332
GCCGGCACATAGG	0.23	0.003	0.235	235	767
AGTATCACTTACA	0.02	0.005	0.022	43	1905
CGCAGTGCAAGCA	0.022	0.005	0.017	30	1704
AGTATTGCAAATA	0.025	0.005	0.019	49	2578
CCCGGTACATGTA	0.025	0.003	0.021	47	2181
AGCAGCATTTATA	0.025	0.001	0.024	70	2844
GCTAGCGTATGCA	0.057	0.011	0.043	48	1056
GGCGTTGTAAAGA	0.072	0.006	0.077	106	1268
AGCGTCGTTTGCA	0.021	0.006	0.016	22	1395
CCCAGTACTGACA	0.03	0.002	0.028	96	3358
GGCAGCGCTTACG	0.068	0.005	0.063	62	922
GCTGGTGTAAGGA	0.401	0.045	0.394	327	503
AGCGGTGTTGGGA	0.02	0.005	0.014	10	716
GCTAGCATTAAGG	0.069	0.016	0.049	51	984
AGTAGTATTGGGG	0.024	0.004	0.028	16	550
CCCATTATATATA	0.023	0.001	0.025	125	4813
ACTATTATTGGCA	0.025	0.002	0.027	66	2418
ACCATTACATACG	0.023	0.001	0.023	103	4431
GCCAGCATAGACG	0.051	0.004	0.047	102	2055
ACCGGCATTGATA	0.024	0.006	0.015	39	2508
CCCGGTGTAAGCG	0.028	0.005	0.027	39	1380

AGCAGTGCTAGTA	0.018	0.002	0.016	27	1623
GGCATCACAGGCG	0.082	0.012	0.07	99	1312
CCCAGTGTATACA	0.024	0.001	0.023	69	2976
CCCGTCATTTGGA	0.027	0.004	0.029	48	1590
ACCATCGTTTGCG	0.027	0.005	0.023	35	1485
CCCGTCATAGGGG	0.046	0.004	0.047	51	1036
ACCATCGCAGGCG	0.023	0.001	0.022	42	1900
CGCGTCACATACA	0.026	0.004	0.022	55	2481
AGCGTTACATGCA	0.023	0.002	0.021	44	2043
CGCGTTATTGACG	0.025	0.008	0.02	29	1392
CCTAGTACAAACA	0.025	0.002	0.023	97	4120
CCCGTCGTTAGGG	0.019	0.004	0.019	17	865
CGCAGCATTGACA	0.023	0.003	0.026	63	2335
GCTATTGCAGGTA	0.051	0.006	0.05	54	1034
CCTATCATAAACG	0.026	0.004	0.027	104	3805
AGCAGCGCAGACA	0.02	0.005	0.026	59	2214
ACCGGTACAGGGG	0.023	0.003	0.026	25	920
ACTAGTGCTAACG	0.023	0.001	0.023	51	2182
ACCATCATATATA	0.026	0.002	0.024	142	5685
GGCGTCATTTAGG	0.062	0.011	0.06	46	725
GGTGTTATATGTG	0.096	0.015	0.078	57	677
ACTATCGTTTGTA	0.028	0.002	0.029	50	1676
CGTAGTACTTAGA	0.029	0.003	0.03	28	893
CGTAGTATATATA	0.025	0.004	0.03	60	1912
ACTATCGTAAGGG	0.032	0.005	0.026	35	1310
ACTATTACATAGA	0.02	0.002	0.019	70	3631
CGCATCACATGCG	0.023	0.006	0.014	26	1789
CGCAGCGCTAACA	0.024	0.003	0.028	58	2038
AGCATCATATACG	0.021	0.002	0.022	73	3212
AGCGGTGTTGGTG	0.022	0.001	0.022	14	632
ACCGTTACAAGGG	0.026	0.004	0.025	46	1824
ACCATCGCTAAGA	0.025	0.004	0.022	71	3228
CCTAGTACTTGGG	0.02	0.004	0.023	20	833
ACCGTCGCTGGGA	0.023	0.004	0.024	29	1205
GCCGTCGCTGACG	0.052	0.008	0.041	33	772
AGCATCATTGGGA	0.024	0.007	0.034	58	1673
GCCGGTACTGAGG	0.067	0.007	0.075	59	727
AGCAGTGCTAATG	0.022	0.001	0.02	34	1628
CCCAGCACATAGA	0.023	0.004	0.022	63	2798
GGCAGTGCAGATA	0.046	0.006	0.041	55	1273

CCCATCACAAGGA	0.026	0.002	0.027	81	2902
GCCAGTACAAAGA	0.055	0.001	0.056	145	2448
AGTGGCACATGCG	0.028	0.014	0.009	5	577
CGTATCATAGGTA	0.024	0.004	0.021	32	1509
CCCGTCGTAAAGA	0.024	0.003	0.021	52	2457
GCTATTACTAAGA	0.053	0.011	0.041	80	1875
CCTGGTGTAGAGG	0.03	0.005	0.028	23	809
ACTAGCACAGGTA	0.027	0.002	0.03	67	2164
GCCAGCGCTTACG	0.053	0.004	0.059	58	927
ACTATCACAAAGA	0.023	0.004	0.018	68	3724
GGCAGCGTTGGTG	0.073	0.008	0.067	40	558
CCCGGTACTTAGG	0.026	0.005	0.02	22	1105
CGCGGTACATGCG	0.028	0.004	0.028	29	1006
CGCAGTATATAGG	0.025	0.002	0.028	41	1435
AGCGGTATTAATA	0.021	0.002	0.019	51	2651
AGTGTCACTGGCA	0.023	0.007	0.021	20	947
CCTAGTGTTTGCA	0.023	0.004	0.029	45	1518
AGCGTTGTAGAGA	0.02	0.005	0.021	31	1414
CCCGGCGCATGGA	0.022	0.008	0.023	28	1214
CCTAGCACAAACG	0.024	0.002	0.023	55	2387
ACCAGTGCAAGCG	0.02	0.001	0.02	47	2291
CGTGTCGCTAACA	0.02	0.003	0.021	31	1411
CGTATTACAGGTG	0.029	0.009	0.024	24	963
GGTAGCACATACA	0.069	0.012	0.059	83	1326
GGCAGCACTTGTA	0.055	0.006	0.052	58	1049
GCTGGTGTTTACG	0.744	0.048	0.729	762	283
CGTAGCATTGACA	0.021	0.001	0.021	33	1519
ACCGTTGTATATG	0.026	0.002	0.026	45	1654
AGTATTGCAGGTG	0.024	0.004	0.026	21	788
AGTATTGCAAGGA	0.027	0.005	0.028	37	1270
ACCGGTGTTGGCA	0.028	0.006	0.021	29	1359
ACCAGCGCTGATA	0.02	0.003	0.022	77	3374
CCCGGCACATGGA	0.026	0.005	0.019	32	1651
ACCGGTGCATGTA	0.022	0	0.022	41	1831
AGCGTCGCTTGCA	0.025	0.004	0.02	26	1277
GGTATCGCATACG	0.132	0.029	0.105	102	866
GCTGGCATTAGTA	0.647	0.044	0.659	927	480
ACTATCATTAAGG	0.033	0.004	0.028	59	2080
CGCGTCGCAAGCA	0.025	0.003	0.024	37	1507
CCCATTGCATGCA	0.026	0.003	0.025	68	2699

CCCGTTGTAAGCA	0.021	0.003	0.017	38	2203
GCCATCGTTTGGG	0.046	0.003	0.048	36	709
ACCGTCACAAGGA	0.021	0.002	0.024	63	2588
CCCGGTGTAGAGA	0.022	0.004	0.023	32	1384
CCCGTTGCTAGTG	0.021	0.003	0.016	19	1145
AGTGTCACTAGGA	0.022	0.002	0.018	15	796
GCCAGCACTAGGA	0.051	0.005	0.043	56	1238
AGCATTGTTAACG	0.02	0.003	0.016	39	2395
CCTATTGCTGGTA	0.022	0.004	0.018	31	1655
ACCGTTACAGAGG	0.02	0	0.02	39	1900
AGTGTTACAAATA	0.026	0.001	0.025	66	2554
CGTGTTGCTAAGA	0.029	0.007	0.029	26	875
GGCGTTACATAGG	0.07	0.007	0.072	69	883
CGCAGTACTGACG	0.02	0.001	0.02	29	1387
AGCGTTATTGACA	0.023	0.001	0.025	63	2467
GCTGGTGCAAATG	0.283	0.022	0.282	262	668
ACCAGCGCATGTA	0.022	0.001	0.021	57	2716
GGTATTACATATA	0.089	0.016	0.084	159	1743
ACCATCGCTTGCA	0.02	0.001	0.022	59	2680
ACTATCACTAACA	0.025	0.002	0.022	109	4769
CCCATCGTAGGTA	0.025	0.005	0.031	64	2021
CCCGGTATATGCA	0.025	0.002	0.028	67	2347
GGCGTTACTGATA	0.062	0.013	0.048	68	1349
AGCATTACTAATG	0.023	0.005	0.017	50	2957
GCTATTACAAGGA	0.061	0.005	0.053	79	1416
GCTAGTGCTGGTA	0.055	0.003	0.051	37	686
CCTGTCATATATA	0.024	0.006	0.017	45	2593
ACTGGCACTAGTA	0.025	0.004	0.025	39	1533
ACCGTCGCATGCG	0.022	0.005	0.021	33	1540
AGTGTCATTAGTA	0.021	0.002	0.023	27	1151
AGTGTCACATGGA	0.026	0.01	0.014	12	824
ACCGTTATAAATG	0.027	0.003	0.023	78	3272
CGTGGCATTAATA	0.023	0.004	0.017	21	1189
CCTAGTATATATG	0.031	0.003	0.034	78	2221
ACTATTGCTTATG	0.026	0.002	0.028	59	2047
CCTGGCACATGTA	0.025	0.004	0.019	29	1460
GGTAGTACTTGGA	0.068	0.008	0.061	34	527
CCCGGCGTTTGTG	0.025	0.005	0.027	24	854
GCTGTCATATATG	0.059	0.005	0.052	61	1101
GCCAGTGTATATA	0.046	0.001	0.046	90	1882

GGCGTTACTGGCG	0.062	0.004	0.057	47	777
ACTATTACTTAGG	0.021	0.006	0.021	43	2010
CCCGTTATATAGA	0.025	0.003	0.027	70	2514
CGCAGCGTTGGTA	0.02	0.004	0.022	23	1045
GCTATCACAGGGG	0.058	0.005	0.053	39	695
GGCGTTACATGTA	0.061	0.002	0.06	77	1214
AGTAGCGTTGACA	0.027	0.001	0.027	36	1285
ACCGGCGTATGTG	0.023	0.007	0.026	16	604
ACCATTACTAACG	0.023	0.001	0.023	113	4708
ACCAGCACTTGCG	0.024	0.002	0.026	56	2079
AGTATCGCTGAGA	0.018	0.005	0.011	12	1048
CCTGGTGCAAGTG	0.028	0.006	0.022	20	882
CGCGGCACAGGCG	0.018	0.006	0.014	13	942
GGCGTCACATGTG	0.062	0.012	0.05	47	884
AGTGGTACAAGCA	0.022	0.004	0.018	25	1341
AGTATCATTAATA	0.022	0.002	0.019	57	2981
ACCGGTATAAATA	0.024	0.002	0.025	115	4508
AGCAGCGTTAACA	0.024	0.003	0.028	78	2724
AGTGGCGCTAACA	0.027	0.002	0.028	32	1128
ACCGTCGCAAGTA	0.026	0.003	0.031	74	2351
CCTATTGCTTAGA	0.025	0.001	0.026	45	1715
CCCATCATTGACG	0.038	0.007	0.031	81	2554
GCTAGCGCTTACG	0.057	0.001	0.059	43	688
GCCAGTACTTACG	0.049	0.004	0.052	79	1432
GCCATTGTTTAGG	0.043	0.007	0.052	51	936
CCCGGCGCTGACG	0.02	0.005	0.014	21	1493
GCCGGTACTGGTG	0.061	0.009	0.065	46	659
CGCGGCGTTTACG	0.021	0.001	0.021	17	790
CCCATCGCTAGCA	0.024	0.003	0.026	70	2639
AGCGTCACTGATG	0.02	0.003	0.023	34	1439
CCCGGTGCTTGGA	0.022	0.006	0.014	14	1023
GGTGTCACAGGCA	0.08	0.013	0.072	58	746
CGCAGCGTTTACA	0.023	0.006	0.023	40	1727
GGTGGCACAGGTG	0.371	0.062	0.318	169	362
CGTGGCGTTAACG	0.017	0.008	0.008	5	624
CCCAGCACATGTA	0.025	0.004	0.023	65	2703
AGTATTGTATAGA	0.024	0.007	0.02	36	1742
AGCGGTGTTAGGG	0.02	0.006	0.017	11	620
CGCGGTGCAGACA	0.027	0.005	0.023	36	1509
GGCAGTATAAAGG	0.061	0.002	0.06	98	1536

CGTGTCGTTTGCA	0.026	0.005	0.02	15	744
AGCATCGCTTACA	0.023	0.003	0.024	60	2451
CGCGGCGCAGGCA	0.029	0.001	0.028	33	1137
CGCGGCATTTGGG	0.022	0.005	0.029	13	432
ACCAGCGTAGGTG	0.021	0	0.021	24	1146
CCCGTTACTGATG	0.029	0.003	0.032	56	1716
GCCGTTATATACG	0.055	0.008	0.043	77	1708
CCCAGCGTTAAGA	0.026	0.002	0.026	56	2105
GCTAGTGCTAATG	0.058	0.008	0.066	65	921
ACTGGCACATACG	0.031	0.001	0.03	45	1451
AGCAGCGTTTGTA	0.021	0.005	0.024	33	1337
GCTGTCGCTGGGA	0.053	0.005	0.056	23	391
AGCAGCGCAAATG	0.02	0.004	0.015	28	1875
CGCGTTGCTGATG	0.029	0.004	0.029	28	934
AGTGGCATAAACA	0.023	0.006	0.031	46	1432
GCCGGTGCTAAGG	0.125	0.023	0.097	69	644
CGTGTTACTAAGA	0.03	0.007	0.027	36	1292
ACTATCGCAAATA	0.025	0.002	0.025	101	4002
AGTATTATTAAGA	0.022	0.002	0.024	66	2707
CGCAGTACAAGGA	0.023	0.003	0.022	37	1639
GCCGTCACTAGCA	0.048	0.002	0.046	77	1589
AGTGTTGCAGATA	0.03	0.01	0.02	24	1189
CCTATTGTTTAGG	0.022	0.004	0.021	28	1286
CCCGTCGCAAGCG	0.025	0.003	0.023	40	1688
AGCGTTGCAGATA	0.023	0.003	0.019	34	1801
AGTAGCGCTGGGG	0.034	0.006	0.026	10	382
AGTATCATTAACA	0.021	0.005	0.016	49	2956
GCCGTCATTAATG	0.056	0.012	0.045	79	1696
CGCATCGCAGACG	0.027	0.004	0.022	39	1722
ACTGGCGTAAACG	0.031	0.004	0.026	22	837
ACCATCATTGGGG	0.03	0.006	0.039	47	1168
CCCAGCGCTTAGG	0.028	0.002	0.029	33	1097
ACCAGCGTAAACA	0.024	0.002	0.02	80	3829
GCCGTCATATAGA	0.06	0.006	0.056	103	1751
CCCAGTATATACG	0.025	0.003	0.024	78	3140
GCCGGCACTTAGG	0.201	0.027	0.222	184	644
CGCGTTGCATACG	0.034	0.004	0.034	46	1297
CCTGGCATTAGCA	0.028	0.006	0.02	32	1535
CCCATTGTTTGCG	0.027	0.006	0.019	31	1563
CCCATTATAGATA	0.024	0.002	0.024	110	4492

CCTGGTACAGGCA	0.028	0.008	0.018	26	1430
AGTGTTACAGGCG	0.023	0.004	0.018	17	930
ACTGGCGCTGACA	0.026	0.004	0.03	28	915
CCCATCGTTTACA	0.021	0.005	0.016	48	3026
CCTGGTGTTGGTA	0.02	0.006	0.013	11	851
AGTATTATATGCA	0.021	0.003	0.024	55	2216
ACTGTCGCTTGTA	0.026	0.003	0.024	31	1273
GGCATTGTAGACA	0.067	0.006	0.064	123	1810
CCTATTATTGGTA	0.019	0.001	0.018	39	2127
CGCGGCACAAATA	0.023	0.002	0.024	57	2321
AGCGGTACAAGTA	0.023	0.002	0.025	54	2093
GGTGGCGTATGCG	0.667	0.059	0.668	446	222
ACTGTTGCAGATG	0.02	0.002	0.022	30	1319
GGTAGTGTTTGGG	0.056	0.012	0.046	15	309
AGCAGTACAAGTG	0.02	0.004	0.021	43	1970
GCTGGCACAAACG	0.45	0.031	0.444	678	850
CGCGTTATATACG	0.024	0.004	0.02	34	1704
GGCATTATTGGTA	0.052	0.003	0.047	69	1385
GGCATCATAAGCG	0.088	0.015	0.08	137	1573
GGCAGTACTTATG	0.061	0.002	0.059	69	1103
AGCGTTGCAGGCA	0.018	0.002	0.018	26	1433
ACCGTTATATATA	0.026	0.001	0.025	101	3917
ACCGTTGCATGGG	0.027	0.011	0.025	28	1114
ACTGTCACAGACA	0.026	0.003	0.022	62	2804
ACTATCGCTAATA	0.024	0.005	0.02	64	3151
ACTGGCGTATAGG	0.033	0.016	0.025	13	511
CCCATCGTTGGTA	0.03	0.002	0.027	50	1787
CCCAGTATATAGG	0.027	0.003	0.023	48	2048
ACCATCATTAGCG	0.025	0.001	0.023	65	2723
GCTAGTGCTAGCG	0.06	0.014	0.041	35	816
CGTGTTACTAGTG	0.02	0.009	0.029	23	765
CCCATCGCTGGGG	0.032	0.005	0.036	33	882
CCCAGTACATAGA	0.028	0.001	0.027	81	2880
CGCATCGTAAGTA	0.025	0.002	0.024	53	2151
AGCGTTGTTTACA	0.021	0.004	0.022	43	1877
GGCAGCACTTGCA	0.053	0.005	0.046	57	1190
CCTGTTACATACA	0.023	0.004	0.018	47	2615
GCTATTGCTTGGG	0.05	0.005	0.048	27	538
AGTGTTGCATGGA	0.019	0.007	0.012	9	736
GCCGTCGTATAGA	0.054	0.005	0.051	54	1015

AGCAGTATATACG	0.021	0.003	0.025	72	2852
ACCGTTGTAAATA	0.026	0.002	0.025	89	3420
CGCGGCATATATA	0.021	0.002	0.017	32	1812
GGCGGTATAGGGA	0.068	0.013	0.065	57	822
AGCAGTATTAACG	0.025	0	0.025	75	2894
CGTGTCATAGACG	0.02	0.001	0.019	21	1074
CCCAGCGCTTACA	0.023	0.003	0.019	59	3000
GCCGTTATTGATG	0.051	0.008	0.044	62	1342
GCTGGCGCAAAGA	0.621	0.04	0.618	893	553
CCTGGCGTTAACG	0.022	0.002	0.021	23	1096
ACCAGTATAGACA	0.023	0.003	0.022	111	5001
GGTAGTACAGACA	0.06	0.013	0.046	60	1252
ACCGTTGTAAACG	0.028	0.001	0.027	67	2413
CCCGGCATATGTA	0.024	0.005	0.017	37	2152
GGTATCATAAAGG	0.121	0.014	0.101	114	1014
CGCAGCATAAAGA	0.024	0.003	0.021	55	2561
CCTATTGCAGAGG	0.03	0.005	0.025	36	1425
GCCGGTACATGCA	0.059	0.003	0.058	86	1398
CCTAGTATAGGGA	0.038	0.003	0.04	52	1239
CGCGTTGCTGGGA	0.024	0.007	0.026	17	634
AGCATTATTGGTA	0.022	0.001	0.023	60	2544
GCTATTACATATG	0.06	0.005	0.055	93	1600
ACCGTCATTTATA	0.022	0.003	0.021	68	3136
ACTAGTATATAGG	0.025	0.005	0.018	34	1867
GGTATTATTGACA	0.098	0.009	0.086	144	1533
GGTAGCATAAAGG	0.075	0.015	0.068	58	793
ACCATTATATGGA	0.026	0	0.026	77	2904
GGTGTCATTAGCG	0.099	0.023	0.076	43	526
AGCGGTGCAAACG	0.023	0.001	0.023	36	1537
CCTATTGTAGGTG	0.03	0.006	0.021	28	1286
GGTAGTGTTAATG	0.06	0.012	0.043	35	771
GCCATCACATGGA	0.055	0.006	0.063	106	1576
ACTATCGCAAGCG	0.027	0.005	0.025	45	1758
CGCAGCACAGACA	0.023	0.001	0.022	60	2609
ACTAGTATAGATA	0.024	0.004	0.021	73	3483
CCCATCACTGGCA	0.032	0.004	0.028	77	2660
GCTGGTATAAGGA	0.167	0.015	0.152	144	804
CCCGTCACATGTA	0.029	0.001	0.028	70	2435
CGTATCGCAAATA	0.021	0.002	0.021	44	2040
GCCGTTACTAAGA	0.06	0.009	0.07	111	1471

CCCGGTATTGATA	0.024	0.005	0.026	59	2211
CCCGTTACATAGA	0.026	0.002	0.027	67	2426
CGCATTATATGCG	0.031	0.001	0.029	50	1668
AGTATTATTGGGG	0.022	0.003	0.025	22	844
ACTAGTGTTTGTG	0.027	0.001	0.026	31	1163
CCCGTTGCTAGCG	0.026	0.003	0.026	36	1371
GGTAGCGTTTACG	0.087	0.007	0.078	43	508
AGTAGTGTAGGTG	0.024	0.004	0.027	19	672
CGTAGCACATAGG	0.027	0.002	0.026	21	791
CCTATCGTTAGGG	0.031	0.009	0.031	30	927
ACCGGCGTTAATA	0.028	0.004	0.024	42	1741
ACTAGCGTTTAGG	0.034	0.01	0.021	18	824
ACTAGTATAGGTG	0.029	0.006	0.022	31	1388
ACTGGTGCAAGGA	0.027	0.005	0.022	27	1225
AGCGGCGCAAACA	0.02	0.001	0.019	43	2240
AGTGTTATTAACA	0.019	0.002	0.021	53	2415
CCTGGCGCTTACA	0.027	0.005	0.024	35	1434
CCTAGCGTAGACG	0.031	0.003	0.029	47	1548
AGTAGCACTGGGA	0.022	0.005	0.015	12	772
AGCGGCGTAAACG	0.019	0.001	0.017	21	1200
AGTGTTACAAACA	0.021	0.001	0.021	54	2570
CGCATCGCTTGTA	0.026	0.002	0.028	37	1269
GCTATCGTTTATA	0.054	0.012	0.044	68	1461
ACTATCGCTAAGA	0.027	0.003	0.03	75	2441
CGCGTTATAAACG	0.026	0.004	0.025	58	2237
ACCATTGCTTATG	0.026	0.005	0.021	46	2179
CGTGTTGTAGAGG	0.026	0.011	0.021	12	547
ACCGTCATATACG	0.024	0.001	0.024	66	2681
ACCGTTGTAAACA	0.024	0.003	0.021	75	3513
CGTAGTATAAACA	0.029	0.005	0.023	61	2555
CCCAGTATTTACG	0.029	0.001	0.029	75	2472
CCCGTTGTTGGGG	0.026	0.003	0.029	20	669
GGCATCGTAGGCA	0.101	0.012	0.086	119	1266
ACCGTCATATGTG	0.023	0.006	0.017	31	1761
CCCGGTGTTGACA	0.03	0.007	0.023	43	1830
CCCGTTACTAAGG	0.028	0.002	0.032	55	1691
GCTATTGCTGGTA	0.049	0.008	0.06	59	918
AGCAGCACTAGCA	0.022	0.005	0.016	36	2261
CCCATTATTAGGA	0.026	0.001	0.025	62	2442
ACTATTATTGACA	0.023	0.003	0.02	77	3741

CGTGGCACTGGTG	0.024	0.015		0.005	2	402
GCTAGCACTTACA	0.052	0.003		0.048	76	1501
ACCAGCACATAGA	0.023	0.003		0.019	74	3912
GCCGGTGCTGATA	0.06	0.008		0.057	60	1000
ACCGTTGCTTGCA	0.024	0.004		0.026	50	1891
CCCGTTACTTGCA	0.024	0.003		0.025	55	2125
CCTAGTATTTATG	0.025	0.004		0.023	40	1719
GCTATCGCTAACG	0.061	0.013		0.048	59	1177
AGTAGCGCAAGCA	0.027	0.004		0.021	26	1205
GCTATTACTGATG	0.059	0.005		0.066	87	1235
ACTGTTACAGGTG	0.026	0.004		0.027	30	1075
GGCAGTATAAAGA	0.054	0.007		0.048	116	2325
CGTGGTATAAATG	0.022	0.005		0.018	21	1161
GGTGTCACTAATA	0.084	0.017		0.07	87	1156
CCTATCATTGATG	0.033	0.005		0.03	69	2243
AGCATCATAAAGA	0.023	0.001		0.022	97	4233
CGCATTATTTGCA	0.024	0.006		0.016	33	2026
AGCATCATTTACG	0.021	0.002		0.02	53	2557
GGTATTGTAGGCA	0.087	0.015		0.083	76	835
CGCAGCGCAAAGG	0.023	0.005		0.02	23	1128
GGTGGTATAAACA	0.15	0.024		0.139	197	1224
GCTAGCGTTTACA	0.061	0.003		0.063	78	1167
GGTGTTGCATATA	0.059	0.01		0.052	46	844
GGTAGCGTTGACA	0.096	0.026		0.08	65	744
ACCAGTATATGCA	0.023	0.003		0.019	76	3860
GGTGTCATATACA	0.091	0.015		0.073	91	1152
CGCGGCGTTTGTA	0.028	0.009		0.024	20	810
CGCAGCACATACA	0.024	0.003		0.02	57	2773
AGTGTCACTTGCA	0.024	0.003		0.025	25	993
AGTAGTGCTGGTA	0.023	0.007		0.028	21	720
CCTAGCATATGGA	0.033	0.001		0.033	47	1357
ACTGGTACAAAGA	0.024	0.002		0.027	70	2499
GGCGTCATAAAGA	0.069	0.017		0.053	91	1612
GGTAGCACTGGCA	0.054	0.011		0.04	28	676
CCCGGCATTTGCA	0.029	0.004		0.023	46	1914
ACTGTTGTATAGG	0.029	0.007		0.03	32	1026
CGTATTACTAAGA	0.028	0.005		0.034	63	1771
ACCGTCGCTGACG	0.025	0.008		0.027	48	1761
GGCATTGTAGATG	0.074	0.01		0.076	91	1111
CCTGGCGCATACG	0.025	0.01		0.016	19	1191

ACCGGTATTAGTG	0.025	0.004	0.024	38	1529
AGCATTACTAGGG	0.02	0.002	0.019	27	1400
AGTAGTGTAAACG	0.025	0.005	0.024	42	1688
GGTATCATAGACG	0.118	0.02	0.097	106	982
CGTATCGTTGAGG	0.027	0.007	0.018	14	770
GCTAGCGCTAATA	0.059	0.008	0.07	86	1136
GCCATTGTTGACA	0.046	0.005	0.04	84	2009
ACTGGTATAAGTA	0.025	0.003	0.023	53	2235
GCTGGCGTAAAGG	0.943	0.014	0.947	1300	73
AGCGTTACTTAGA	0.018	0.001	0.016	27	1613
CCCATTGTATACG	0.026	0	0.026	67	2517
AGTGTTACATACG	0.023	0.002	0.023	31	1341
AGTGGCGCAGATA	0.023	0.004	0.028	27	925
GCCAGTGTTAACA	0.037	0.007	0.031	68	2127
CCCATTGTAGAGG	0.026	0.002	0.028	46	1576
ACCATCACAAGCA	0.022	0.001	0.021	114	5347
CCCGGCGTTAGGG	0.027	0.006	0.033	24	698
CGTGTCACTTACA	0.022	0.002	0.022	30	1328
CCTATTATTAATA	0.029	0.005	0.023	102	4326
GCCAGCATAAACA	0.054	0.004	0.056	248	4157
GGCAGCGTTTATG	0.057	0.011	0.044	39	852
CGCATCGCATAGA	0.028	0.002	0.028	47	1643
GCTAGTGTTTGGG	0.048	0.005	0.05	28	529
AGTAGCGTTAGCA	0.022	0.002	0.021	22	1035
CGCGTCGCAGATG	0.02	0.003	0.024	30	1205
GGCATCGTTTGCA	0.116	0.023	0.095	116	1111
AGCGGCGCTGACA	0.022	0.002	0.02	30	1443
GCCGGCGCAAACA	0.486	0.043	0.483	1036	1109
ACTGTTACATGGA	0.026	0.002	0.023	40	1693
ACCATCGCAAGGA	0.022	0.003	0.02	55	2649
ACCATTACAGGCG	0.022	0.003	0.02	61	2943
AGCATTACTTGCA	0.021	0.004	0.026	63	2355
GCTATCGCAGGCG	0.045	0.004	0.049	45	873
CCTGGTACTTACA	0.023	0.002	0.024	43	1769
GCTGTCGTAGGTG	0.067	0.017	0.091	44	441
GGTAGTGCTGATA	0.055	0.007	0.049	38	742
ACCGTCATAGAGA	0.025	0.002	0.029	75	2530
AGTGTCACTTATG	0.025	0.01	0.017	14	834
CGCGTCGTTTGCG	0.026	0.004	0.028	23	800
CCCGTTGCTAATG	0.029	0.006	0.024	43	1746

CGTGTTATTAATA	0.022	0.002	0.025	50	1916
CCTATCATAGAGG	0.044	0.003	0.042	71	1605
ACTATTACAGGTG	0.024	0.002	0.023	46	1970
ACCGGCACTTACA	0.022	0.003	0.018	62	3337
ACTAGCGTTAACA	0.03	0.003	0.026	60	2269
ACTGTCGCAAATA	0.027	0.003	0.026	74	2774
CGTGTTACTAATG	0.025	0.009	0.021	25	1166
CCCGTTATTTGTG	0.023	0.006	0.016	21	1307
ACTGTCATAGGCG	0.025	0.004	0.02	25	1229
AGCGTCGTATATA	0.023	0.003	0.02	44	2170
ACCGGTACAGACG	0.025	0.006	0.018	49	2666
CCTATTGTAAAGG	0.025	0.004	0.024	52	2135
CGCATCACTTGGG	0.024	0.003	0.027	25	910
CGTATTGCAAGTA	0.018	0.002	0.015	22	1409
CGTATCATTAATG	0.025	0.004	0.031	55	1708
CGCGTCACATATG	0.027	0.006	0.032	51	1539
CCTAGCGTAAAGA	0.028	0.006	0.022	47	2135
ACCATTACTTAGG	0.024	0.002	0.024	62	2483
CGCGGCATAAAGA	0.027	0.004	0.021	39	1804
AGCGTTATATATG	0.021	0.002	0.02	39	1951
GCCATCACTTGTG	0.056	0.004	0.05	63	1201
AGTAGTACTTACG	0.023	0.004	0.018	22	1215
AGCGGTATTAATG	0.025	0.004	0.019	32	1630
GGCGTCATTAGCA	0.062	0.003	0.065	84	1200
CCCATCGCTAATA	0.025	0.007	0.019	71	3650
ACCAGTATTGGCG	0.02	0.005	0.021	48	2201
AGTGTCATTTGCG	0.018	0.005	0.012	9	724
GGCATCGTTAACG	0.111	0.019	0.092	133	1309
CGTATCGTTAATA	0.022	0.001	0.024	46	1856
GCTAGCGCAAGTG	0.065	0.01	0.052	34	616
CCCGGCGCAAGGG	0.027	0.006	0.034	37	1042
CCCATTATAGACA	0.025	0.001	0.025	122	4782
AGCAGTATTTGTG	0.024	0.002	0.027	39	1409
CCTGTCGCATGGG	0.029	0.004	0.026	18	664
AGCAGTATTTGCA	0.021	0.003	0.018	44	2362
AGCATTATAGAGA	0.022	0.002	0.021	66	3143
AGTGGCATTTACA	0.021	0.003	0.025	21	805
CCCAGCATATAGG	0.027	0.003	0.025	47	1870
CCTGTCACAAATA	0.022	0.004	0.021	68	3116
GCTATTATATACA	0.064	0.005	0.06	188	2964

ACCATTATTGATA	0.021	0.002	0.018	85	4550
CCCGGTATAGATA	0.025	0.001	0.024	65	2624
ACCATTGTATATA	0.027	0.005	0.025	92	3645
GCCAGCACTGAGG	0.051	0.016	0.041	42	971
CCCAGCACTTGCG	0.023	0.001	0.024	39	1610
AGCATCACATAGA	0.022	0.001	0.021	58	2685
ACCGGCGCTGGGA	0.024	0.003	0.025	19	751
CCCGTTACAGACA	0.026	0.006	0.018	63	3374
AGTGGTGCAAGCG	0.027	0.007	0.022	15	661
GGTATTGTTGGTG	0.107	0.017	0.09	46	464
ACTGGTGCTGGCA	0.025	0.003	0.028	35	1225
AGTGGTGCTGGTG	0.02	0.007	0.025	10	386
CGCAGTGCTGAGA	0.029	0.002	0.027	29	1054
AGCGGCATAGATA	0.02	0.002	0.019	32	1697
CCCATCATAGAGA	0.027	0.003	0.023	74	3214
GGTGTTATTGATG	0.09	0.008	0.089	66	674
ACCGGCATAAACA	0.024	0.002	0.023	102	4314
ACCAGCATTGATA	0.022	0.003	0.02	72	3611
CCCAGCGTATGCA	0.025	0.008	0.016	37	2315
CCCAGTGCAGGTA	0.026	0.007	0.025	47	1823
CGCGGTACAGACA	0.024	0.004	0.018	37	2015
ACCGTTGTATGTA	0.024	0.004	0.021	38	1786
GGCGTCGTAAAGA	0.07	0.008	0.062	76	1152
GCTATTGTTTGCA	0.045	0.002	0.046	61	1254
GGTAGCATAAACA	0.067	0.009	0.064	123	1801
CCTAGCATAGGCG	0.031	0.006	0.023	32	1338
GCTATTGTTGATA	0.057	0.003	0.058	94	1540
CCCGTCGTAGAGA	0.017	0.003	0.013	23	1787
CGCATCGCATGCA	0.024	0.001	0.022	43	1878
AGCAGCGCAAGTA	0.024	0.002	0.025	49	1950
GCTATCACTTGCA	0.053	0.015	0.037	49	1264
ACTATTGTTGAGG	0.031	0.007	0.028	40	1413
ACCATCATATGGA	0.022	0.002	0.023	65	2730
GCTAGTATTTAGG	0.069	0.008	0.06	54	840
CCCATCACAGGCG	0.036	0.006	0.028	63	2185
CGCATTACATACG	0.029	0.005	0.028	63	2169
CGTATTACAGAGG	0.022	0.002	0.023	23	993
AGTAGCATAGATG	0.027	0.005	0.019	26	1332
CCTGTCATTTGTA	0.029	0.003	0.029	42	1408
AGCGTCATAGAGA	0.022	0.004	0.028	60	2100

CCTGTCGCAGATG	0.024	0.007	0.033	39	1146
GCCGTTATTAATG	0.045	0.012	0.032	60	1820
CGCATCATAAGGA	0.024	0.003	0.021	46	2176
AGTGTCGTTAAGA	0.026	0.007	0.018	20	1070
CGCAGTACAAAGA	0.022	0.001	0.023	55	2380
CGCAGTACATGGA	0.022	0.007	0.012	18	1424
CGCATTACTAACA	0.022	0.003	0.02	66	3220
GCCATCATAAATG	0.055	0.005	0.052	171	3093
GGCGTTACTGACG	0.063	0.011	0.05	51	974
ACTGTTATAAGGA	0.026	0.002	0.026	51	1882
CGCGGTATAGACG	0.026	0.001	0.026	36	1371
GGTAGCACTAACG	0.073	0.006	0.067	56	786
CCCGGCACTAAGG	0.025	0.003	0.027	38	1378
ACCATTATATGGG	0.028	0.002	0.027	51	1813
CGTAGCATTGACG	0.029	0.005	0.022	22	965
ACTGTTGCAGAGA	0.029	0.006	0.021	32	1525
ACCGTTATTAATA	0.024	0.002	0.025	99	3891
ACTAGTGTTGGCG	0.024	0.004	0.025	42	1622
CCTATTATTAAGA	0.028	0.001	0.026	84	3164
GGCATTGTTAACG	0.077	0.01	0.074	110	1378
CCCGTCATTAACG	0.026	0.004	0.025	61	2420
AGCAGCATTGAGA	0.023	0.004	0.018	37	2013
CCCATCGCAGATA	0.025	0.002	0.022	72	3248
CCTGTCGTATATG	0.027	0.001	0.027	35	1264
CGCGTTGTAAACA	0.024	0.002	0.022	52	2291
ACCAGCATAGATG	0.021	0.003	0.018	53	2915
CCCGTCGTAAGGA	0.028	0.006	0.025	43	1648
AGTAGCGTTTACG	0.033	0.004	0.031	28	885
CGTAGTATAAATA	0.027	0.004	0.028	73	2518
GGTGTCGCAGACA	0.079	0.017	0.065	52	742
ACCATCGTAGGCG	0.027	0.004	0.022	44	1953
ACCGGCATTGACG	0.026	0.003	0.029	53	1762
ACTAGTACATGTA	0.024	0.002	0.022	68	2984
CGTGTTGTATGTG	0.021	0.006	0.014	9	637
AGCATTATAGGTG	0.019	0.004	0.019	38	1924
GCCAGCGTTGACG	0.064	0.007	0.058	71	1151
ACCAGCACAAACA	0.023	0.001	0.022	171	7493
GGTAGTATTAGCG	0.062	0.008	0.052	35	642
ACCATTATAAGTA	0.022	0.004	0.017	93	5244
GGCATCATTAGCA	0.079	0.009	0.07	135	1788

**AGCGGCGTAAGCG**	0.027	0.006	0.02	16	787
**CCCATTGCTGATG**	0.027	0.007	0.018	33	1832
**AGCAGTACTTACG**	0.02	0.002	0.018	35	1960
**AGTGGTATAAGCG**	0.019	0.004	0.016	12	747
**CGTATCACATGTG**	0.029	0.003	0.027	25	898
**GCCAGTATAAAGA**	0.058	0.004	0.053	172	3081
**GCTATTGTTAGTG**	0.058	0.011	0.06	59	930
**CGCGGTGCATACA**	0.024	0.003	0.021	35	1665
**GGCGGTACTAACA**	0.059	0.009	0.061	102	1571
**GGCAGCATAAATA**	0.052	0.007	0.053	158	2830
**GCTAGTATAAATG**	0.069	0.006	0.061	127	1960
**CGTGGCACAAAGA**	0.02	0.008	0.015	17	1121
**CGTGTTATTAAGA**	0.021	0.004	0.016	22	1383
**GGCGGCGTTGGTA**	0.214	0.03	0.2	131	525
**GCCATTGCAGGGA**	0.044	0.011	0.031	32	996
**AGCGGTATAAATA**	0.022	0.003	0.025	89	3512
**GCTGGTACTGGCG**	0.105	0.015	0.085	50	536
**CGCGGCATTGATG**	0.023	0.005	0.022	20	885
**CCTAGTGCTAATA**	0.024	0.004	0.019	48	2447
**CCCAGCGTTAGCA**	0.026	0.003	0.025	56	2186
**GGTGTTACTGATA**	0.087	0.008	0.078	81	962
**CCTGGCACAGGCA**	0.024	0.003	0.02	31	1532
**ACCGGTACATACG**	0.024	0.002	0.021	65	3007
**AGTAGTGTTAAGA**	0.025	0.002	0.023	32	1381
**GCCGTCGTTTGTA**	0.05	0.009	0.037	32	834
**AGCGGTGCATATG**	0.018	0.006	0.026	29	1108
**AGTAGTATAGAGA**	0.027	0.004	0.031	55	1730
**GGCGGTATAAGTG**	0.125	0.015	0.12	129	945
**GGCATTATAAAGG**	0.063	0.003	0.058	111	1787
**CGTAGTACTGGGA**	0.031	0.009	0.044	28	614
**ACTGGCGCATGCA**	0.027	0.01	0.015	14	920
**CCCAGTGTTAACG**	0.027	0.001	0.027	64	2265
**AGTATTGTTAGGG**	0.02	0.004	0.016	13	807
**ACTGTTACATGCA**	0.023	0.002	0.023	55	2314
**ACTATCGTAAACA**	0.027	0.004	0.022	81	3543
**ACTGGCGTTGGTA**	0.024	0.003	0.02	15	754
**CCTGTCGTTGATA**	0.023	0.004	0.022	36	1600
**CGCGTCGCTTGCA**	0.025	0.008	0.017	20	1175
**CGCGTCGCTTGGG**	0.031	0.007	0.041	22	513
**ACCGGTATTGGGG**	0.02	0.007	0.018	15	797

ACTAGCGTTAATG	0.03	0.007	0.022	31	1350
AGCATCATTGATA	0.021	0.002	0.02	69	3425
AGTGTCACTAAGG	0.022	0.004	0.026	23	873
CCCGTTGTAAATG	0.026	0.004	0.02	45	2233
GGCGGCATATGTG	0.554	0.063	0.547	652	541
CGTAGTGCTAACA	0.029	0.005	0.028	36	1268
GGTGGCGCAAGGA	0.169	0.017	0.159	80	423
CGTGTCATATACG	0.03	0.008	0.019	24	1245
ACTATTACAAGGG	0.029	0.003	0.031	62	1915
ACTATCGTTGATG	0.04	0.005	0.044	63	1383
CCCAGCACAAAGG	0.028	0.003	0.023	52	2201
CGCGGTGCTGATG	0.021	0.003	0.025	21	821
CCTAGCGTAGACA	0.029	0.004	0.029	67	2234
GGCAGCGCAGAGG	0.05	0.011	0.052	38	692
GGTATTATATGTA	0.096	0.012	0.079	123	1427
ACTATTATAAGGA	0.026	0.006	0.018	54	2909
AGCAGTACTTGTA	0.018	0.005	0.014	28	1948
GGCATTGTTGACA	0.062	0.004	0.064	103	1517
AGCAGTACAGGCA	0.021	0.003	0.017	39	2241
CCCGTTATTAGTG	0.025	0.003	0.029	46	1555
CCTGTTGTATGGA	0.027	0.006	0.019	21	1066
CCTGGTGTTAACA	0.022	0.004	0.025	50	1913
CCCATCGTAAAGG	0.022	0.004	0.025	61	2334
CGTAGTACAAGCG	0.033	0.002	0.035	35	951
GCTATCGCAAAGA	0.051	0.008	0.051	67	1250
ACCGGTGTTTAGG	0.022	0.009	0.011	9	832
CCCAGTATATGTA	0.031	0.003	0.034	104	2940
ACTATCGCAAGCA	0.028	0.003	0.024	71	2924
AGTGGTACAGATA	0.024	0.009	0.012	17	1419
AGTGGCACATGGA	0.029	0.004	0.031	25	782
AGCATCATTAACG	0.024	0.001	0.023	76	3220
AGCGGTATAAAGG	0.023	0.001	0.021	31	1437
CGTATCACAGATG	0.026	0.004	0.027	33	1186
CCCGGTATTTACA	0.023	0.004	0.021	57	2609
CCCGGTGTAGGGG	0.036	0.003	0.034	23	659
CCTGGTACTTATG	0.034	0.008	0.033	36	1047
ACTATCGCTAGGA	0.022	0.004	0.019	31	1616
CCTGGCATAAACG	0.026	0.005	0.03	60	1959
CCTAGCGCTGATA	0.019	0.003	0.023	46	1936
ACCATTGCTTATA	0.023	0.001	0.024	81	3276

AGCGTCGTAGGGG	0.021	0.004	0.016	15	895
CGCGTTATTTACA	0.024	0.002	0.023	50	2103
GGCAGCGCATGTA	0.053	0.012	0.043	47	1039
CGTATCGTATAGA	0.022	0.001	0.023	33	1406
ACCAGCGTATGTA	0.021	0.001	0.02	42	2026
CCTAGTACTGACA	0.02	0.002	0.017	43	2523
CCTATCACTAGTA	0.028	0.001	0.027	70	2519
CCTGTTACTAGGG	0.023	0.004	0.019	16	810
GGTGGCATAGACA	0.477	0.049	0.443	649	815
AGTGGCGTAAGGA	0.019	0.004	0.017	11	643
CGCGGCGCTTATG	0.032	0.008	0.024	21	861
CCCGGCACAGAGA	0.024	0.003	0.02	38	1853
ACCGTCGTAGGGG	0.017	0.009	0.007	5	698
AGCGGTACTAATG	0.019	0.001	0.017	29	1647
CCCGTTGTTAGCG	0.032	0.004	0.037	46	1194
GCCGGTATAGAGA	0.075	0.018	0.081	123	1396
ACTGGTATAAAGA	0.024	0.002	0.026	64	2392
CCTATCATTAGCG	0.028	0.003	0.025	51	1982
GCTAGTGCAGGGA	0.057	0.003	0.053	41	739
GGTGTCACATATA	0.071	0.017	0.05	55	1041
AGTATTACAGGGG	0.028	0.005	0.026	22	823
CCTGGTACTAAGG	0.03	0.008	0.04	41	979
CCCATCATTGGCA	0.029	0.006	0.028	79	2763
GGCGTTGTTAGCA	0.065	0.019	0.068	65	895
GCCAGTATTGATG	0.054	0.003	0.051	87	1633
GGCAGTACTGATA	0.052	0.007	0.047	80	1606
ACTGTTGTTGGCG	0.024	0.002	0.022	29	1273
CGCATTATAAGCG	0.023	0.003	0.026	52	1926
ACTAGCATATGGA	0.027	0.006	0.018	32	1730
CGTGTTGCATACG	0.023	0.003	0.021	18	851
ACTAGCGCAAGCA	0.025	0.004	0.024	61	2511
AGTGGTGTATACG	0.026	0.003	0.024	18	717
ACTATTATATAGA	0.026	0.004	0.021	73	3482
ACTGTCGTATAGA	0.026	0.006	0.023	30	1283
ACTAGTATTAAGG	0.026	0.01	0.012	24	1899
ACCATCATTAGCA	0.027	0.003	0.027	108	3949
GGTAGTACTGAGA	0.063	0.008	0.061	51	783
CGCATTATAGGTA	0.022	0.003	0.018	38	2087
CGTATCACATAGG	0.016	0.003	0.02	20	1000
GCCAGCACAAGGA	0.051	0.007	0.046	73	1520

ACTATTGCTTGTA	0.023	0.006	0.019	39	2061
ACCATCATTAGGG	0.031	0.003	0.029	47	1561
AGCATCACAAGTA	0.024	0.002	0.025	83	3220
CCTATCGTTGATA	0.029	0.002	0.027	60	2145
GGCAGTACAAAGG	0.049	0.008	0.046	63	1319
GGCGTTGTAGACA	0.07	0.014	0.057	76	1254
CGCATTGCATGTA	0.034	0.005	0.027	45	1599
AGCGTTACTAAGA	0.02	0.001	0.02	46	2253
GGCGTCATAGATG	0.065	0.013	0.058	64	1044
ACTGTTGTTGGGA	0.021	0.004	0.025	21	803
AGCAGCACTGGCA	0.025	0.002	0.027	49	1750
CCTAGCATAAATG	0.027	0.004	0.022	58	2549
ACCATCACTAATA	0.023	0.002	0.021	133	6141
ACTAGCATTTATG	0.028	0.002	0.029	54	1832
ACCGTTACTAAGG	0.024	0.001	0.023	53	2237
ACTGGTGCAAAGA	0.025	0.001	0.023	44	1842
GCCATTACATATA	0.05	0.009	0.038	117	2980
GCCGTCACTGGGG	0.069	0.009	0.058	31	508
CGTAGTACTAAGA	0.028	0.003	0.03	37	1184
CGTATTGCTGGCG	0.021	0.006	0.014	12	861
AGTATCGCAGACG	0.022	0.004	0.017	20	1163
CCCGGCACTAACA	0.027	0.001	0.027	96	3398
CCTAGTATTGATA	0.028	0.004	0.022	56	2530
ACTATTGCATGTA	0.027	0.005	0.03	74	2434
AGCAGTATTAATG	0.021	0.003	0.02	57	2757
GGTATCATAGACA	0.127	0.025	0.106	177	1487
ACTGGTACTAGGA	0.017	0.003	0.017	24	1353
CGTAGCGTAGACG	0.021	0.004	0.016	14	889
GCCGGCACAAAGG	0.212	0.016	0.189	241	1035
GGCGTCATTAACG	0.067	0.01	0.054	78	1359
GGTGTCACTGACA	0.07	0.005	0.067	63	874
ACTAGTATTAGTG	0.029	0.004	0.026	44	1620
CCCGTCGCAAAGA	0.021	0.003	0.022	56	2544
ACCATCATTGGTA	0.026	0.004	0.028	79	2779
CCCGTTACTGGTG	0.029	0.003	0.025	31	1185
GCCGTCGTTTAGA	0.052	0.011	0.042	38	876
AGCAGTACTGGCG	0.022	0.006	0.017	24	1403
CCCATTGCTAACA	0.023	0.001	0.021	86	3957
AGTAGCGTTAACG	0.018	0.004	0.013	15	1113
ACCAGTGTTTGTA	0.021	0.003	0.017	31	1783

AGTGTCGTTTACG	0.021	0.006	0.012	10	795
ACTATCGCTAGCA	0.028	0.006	0.021	50	2284
CCCATCGTAAGCA	0.028	0.002	0.025	83	3221
CGCGGTACTAATG	0.021	0.001	0.019	26	1317
CGCAGTGCATGCG	0.022	0.006	0.022	22	962
AGCATCGTTGACG	0.027	0.005	0.023	46	1912
ACTAGTACATACG	0.026	0.005	0.02	57	2832
GCTGTCGCAAGCA	0.056	0.008	0.055	53	919
CGCGGTACAGGTG	0.028	0.002	0.025	20	780
GGCAGTGTAGGCG	0.053	0.01	0.044	39	842
CCCATTACTTGCA	0.029	0.002	0.026	77	2862
GGCGGCATATGCA	0.283	0.043	0.25	323	971
CGTAGCACAAGTG	0.02	0.005	0.025	24	944
ACCATCGTATACA	0.028	0.002	0.024	94	3787
CCCATTATTGGCG	0.026	0.001	0.026	55	2094
GGTAGTACATGCG	0.071	0.024	0.069	42	569
AGTGGTACAAACA	0.025	0.003	0.025	55	2121
CCTAGTATTTGCA	0.021	0.001	0.02	40	1912
CGCGTCATTGGCA	0.021	0.005	0.015	20	1348
GGTATTACTAAGG	0.108	0.026	0.093	89	870
CCCAGTACATGGG	0.026	0.007	0.035	51	1393
GCCATCGCTGGGG	0.049	0.014	0.059	34	546
ACTAGTATTTAGG	0.024	0.003	0.029	44	1483
GGCGTTATAAGTG	0.061	0.006	0.057	61	1010
GCCGGTATTGATA	0.079	0.009	0.067	125	1752
GGCGGTATATATA	0.073	0.008	0.073	123	1560
CGCATTATTAGGA	0.023	0.001	0.022	39	1701
GGCGTTGCATACA	0.061	0.012	0.047	70	1407
AGTGGTGTAAAGA	0.023	0.001	0.022	27	1186
GGCGTCACTGATG	0.064	0.008	0.054	54	952
ACTGGCGCAGGCA	0.03	0.01	0.028	22	771
AGTGTTACTAGTA	0.019	0.003	0.015	20	1309
GGTAGTACTTATG	0.067	0.007	0.06	50	790
CCCATCATATGGG	0.039	0.005	0.036	57	1537
AGTGTCACTTACG	0.028	0.003	0.024	24	990
GCTATTGTTGGTA	0.05	0.011	0.056	61	1036
CCCGGTACTAGGA	0.022	0.006	0.023	35	1510
CGCGGCGTTAGTA	0.029	0.003	0.031	29	899
AGCGGTGCAAGGG	0.026	0.004	0.025	19	755
ACTAGCGCTAATA	0.026	0.003	0.022	67	2918

CGTATCGTTAGTA	0.022	0.001	0.021	28	1288
GGCGGCATTAGTA	0.279	0.033	0.244	315	974
AGTGGCACAAATG	0.028	0.005	0.022	22	975
GCCAGCACTTATG	0.054	0.009	0.066	88	1255
GCTATTGTAAGTG	0.06	0.01	0.049	60	1177
AGCATCATTTGTA	0.019	0.002	0.021	54	2483
GCCAGTATAGACG	0.057	0.01	0.051	105	1935
CGTATTATATACG	0.027	0.002	0.028	56	1939
ACTGTCGCTAGCG	0.029	0.006	0.037	41	1073
AGTATTACAGATA	0.023	0.002	0.021	53	2427
ACTAGCGCTTGCA	0.018	0.001	0.019	34	1785
CCCATTACATGTA	0.022	0.001	0.021	70	3270
CCCAGCGTATGGG	0.03	0.009	0.02	21	1042
ACCGGCACATACA	0.021	0.002	0.02	82	4019
ACTATCGTTTACG	0.026	0.003	0.031	53	1678
GGCGTCATAGGCA	0.061	0.002	0.059	77	1236
CCCAGCGTTAATG	0.023	0.002	0.021	44	2101
GCCATTATAAACG	0.051	0.005	0.05	182	3467
AGTAGTATAAATG	0.022	0.002	0.023	54	2250
CGCGTTACAAAGA	0.026	0.003	0.022	48	2092
AGCAGTACATAGA	0.022	0.004	0.024	63	2535
CCTATCATAGGCG	0.033	0.002	0.035	59	1646
CCTAGCGCTAAGA	0.02	0.007	0.03	55	1749
ACCATCACATAGA	0.025	0	0.024	108	4311
ACCATTGTAGGCG	0.022	0.004	0.019	47	2434
GCTGTTGCTAGCG	0.052	0.006	0.054	33	578
CCCATCACAGATG	0.028	0.002	0.03	81	2587
ACTGGCGTAAAGG	0.029	0.002	0.029	15	506
CCTGGCATATACG	0.034	0.005	0.04	62	1493
CCCAGCGCTTATA	0.02	0.003	0.02	57	2822
CCCATTACAGGGG	0.042	0.005	0.046	60	1255
ACCGTCACTTAGG	0.022	0.006	0.014	22	1549
AGCATCATAGATA	0.021	0.002	0.019	75	3957
CCCGTCGTAGATG	0.028	0.004	0.022	34	1498
ACCATCGCTTGGA	0.023	0.003	0.025	49	1911
AGCGGTATATGTA	0.019	0.004	0.013	25	1900
CCCATCACAGGCA	0.026	0.001	0.026	84	3183
CCTAGCGCTAATG	0.024	0.004	0.018	26	1392
GCTATTGTTTATG	0.065	0.013	0.047	53	1083
GCCATCACAAGGA	0.054	0.008	0.047	90	1812

GCTATTACTAGGG	0.07	0.008	0.082	67	751
AGCATCATAAACG	0.022	0.001	0.021	86	4008
GGCGTTGCATAGA	0.059	0.012	0.042	40	913
ACCATTATAAAGA	0.022	0.002	0.019	112	5717
GCCATTGCATAGA	0.046	0.006	0.044	68	1495
AGCATCGCAGACA	0.024	0.003	0.02	57	2836
CGCGTCACAAATA	0.025	0.004	0.02	61	2916
GCCATTGCAAACG	0.044	0.008	0.033	67	1940
CGTAGCGTAGGTA	0.029	0.008	0.027	21	756
GGCATCGTAAATG	0.115	0.015	0.115	205	1577
GCCAGTGCAAGGG	0.042	0.013	0.024	21	869
CCCAGCACATGCA	0.024	0.001	0.024	76	3156
AGTATTGCTAGTG	0.03	0.001	0.029	26	864
AGCATCACAAAGG	0.023	0.002	0.021	57	2690
CGCATCGTTGATG	0.027	0.001	0.027	39	1413
GGCATTGCATATA	0.055	0.004	0.051	91	1684
ACTAGCATTAAGA	0.027	0.003	0.028	73	2560
CCTGTCACTTGCA	0.031	0.002	0.027	42	1493
ACTGGCACTTGCG	0.021	0.004	0.026	25	933
GCTGTCGTATAGA	0.053	0.004	0.053	43	770
AGTGTCACAAGCA	0.021	0.003	0.019	27	1430
CGCGGCGTAAACA	0.024	0.003	0.021	41	1916
CGTGGTACTTGCA	0.025	0.002	0.027	23	832
ACTATTACTTGCA	0.023	0.001	0.024	67	2734
GGTGGTATTAAGA	0.134	0.031	0.13	120	804
CCTATCACTAAGA	0.024	0.004	0.02	62	3041
ACCGTCGCATGTA	0.023	0.001	0.021	43	2035
CCCGGTATAAGGG	0.032	0.004	0.038	47	1191
CCTAGTATATACA	0.028	0.003	0.024	83	3330
CGCAGTGCTGACA	0.026	0.007	0.018	30	1595
GCCAGCGCTGACG	0.059	0.005	0.052	59	1072
CCTAGTGCAGAGA	0.025	0.003	0.024	42	1733
GGTATCGCAAGCA	0.102	0.027	0.083	90	990
CGCGTTGTAAGCA	0.023	0.006	0.024	39	1580
CGCGGCATAGGTA	0.024	0.006	0.032	36	1081
CGTGTTACATGTG	0.02	0.005	0.023	20	838
GGTAGTACAAGTA	0.065	0.006	0.061	74	1134
CGTATTACTTATA	0.025	0.002	0.021	40	1822
AGTGTCATATGGA	0.023	0.003	0.024	26	1042
CGCGGCGTATGCA	0.024	0.004	0.019	23	1192

AGCAGCGTATATG	0.021	0.005	0.02	33	1656
AGCATTATAAACA	0.021	0	0.022	151	6751
CCTGGTATATATG	0.023	0.003	0.021	31	1449
GGTGGCGTTAAGA	0.394	0.065	0.332	224	451
GCCGTCATATATG	0.06	0.005	0.055	91	1555
GGCATTGCAAACA	0.055	0.011	0.045	105	2213
ACCGGTATAGACG	0.023	0.002	0.023	53	2206
ACTGTTACTGACG	0.027	0.006	0.022	36	1569
AGTAGTGTAAGGG	0.035	0.008	0.031	25	771
GGCGGCATAAGCA	0.302	0.042	0.282	441	1121
CCCAGTACTTGCA	0.026	0.002	0.026	71	2615
GGCGTCACTGGGG	0.057	0.008	0.066	40	570
GGCAGTACTGGTA	0.051	0.011	0.038	44	1118
GGTGTTACAAAGA	0.083	0.004	0.08	108	1237
GGTGTCGTTAGGA	0.093	0.004	0.098	40	367
ACCGTTACTTGCA	0.019	0.005	0.014	40	2810
GCCGGCGCATGGA	0.496	0.034	0.498	447	451
GCTAGTGTTTACG	0.068	0.011	0.057	62	1030
GGCAGTACTTACG	0.056	0.02	0.031	38	1203
CCTAGCACTAGGG	0.042	0.013	0.028	28	975
AGTGTTATAAGCA	0.024	0.004	0.021	41	1881
GCCAGTACATATA	0.057	0.007	0.047	134	2727
CGTGTCATTAGTA	0.027	0.007	0.018	23	1268
ACCGTTATTGACA	0.023	0.006	0.015	48	3161
CGCGTTATATGCG	0.032	0.004	0.027	36	1318
CCCGGCACTGATA	0.025	0.003	0.028	68	2330
GCCATCACTGGTG	0.063	0.009	0.071	79	1035
CCTATTGCAAAGG	0.023	0.003	0.027	53	1926
GCCGTCACTGGTG	0.054	0.011	0.041	32	751
CCCAGTGCTGGTG	0.026	0.006	0.034	37	1062
CGCGTTATTTACG	0.024	0.004	0.022	31	1409
ACTATCGCAAATG	0.028	0.003	0.025	62	2453
AGCATCACTGGTA	0.021	0.004	0.025	51	1968
ACCGGTGCAGACA	0.021	0.004	0.017	45	2652
GCCGGTATAAATG	0.169	0.012	0.162	313	1614
AGCGTCGCATATG	0.019	0.007	0.013	17	1320
ACCGTCATAGATA	0.024	0.006	0.017	58	3344
CCCATCACAGGGA	0.034	0.005	0.027	54	1926
CGCGGCATATGCA	0.026	0.006	0.026	36	1362
ACTAGCGCTGGCG	0.029	0.003	0.025	27	1067

GCCAGTACATACA	0.061	0.002	0.06	182	2833
CCTAGTGCTGACG	0.025	0.006	0.019	28	1440
ACTATCATTTACG	0.033	0.001	0.035	86	2349
CCTGGCACAAATA	0.027	0.005	0.025	67	2573
AGTGGTATAGGCG	0.036	0.01	0.023	14	582
GGCGGCGCTGATG	0.176	0.023	0.156	92	497
CGTAGCATTGGTA	0.022	0.003	0.019	18	935
CCCAGCATAAACA	0.028	0.003	0.028	160	5525
CCCAGTATTGACA	0.026	0.006	0.023	83	3478
CCTGGTGCAAGCG	0.027	0.007	0.025	24	922
AGCATCGCAAATA	0.021	0.002	0.018	65	3632
AGCATCGTAGATG	0.018	0.003	0.018	37	2020
GGTAGCGCTGGTG	0.073	0.016	0.051	17	319
CCTGGCGTTGGCG	0.024	0.003	0.021	14	649
CGCAGCATAAATG	0.024	0.002	0.027	66	2371
AGCATTACTAGTA	0.025	0.001	0.026	69	2586
CCTATTATTAAGG	0.026	0.002	0.023	51	2215
CCCGGCGCAAACG	0.027	0.008	0.022	49	2158
ACTATCGTAAGGA	0.027	0.005	0.024	40	1653
GGTATTATAAATA	0.108	0.016	0.091	271	2712
AGTATCACTTATA	0.023	0.003	0.023	43	1787
CCCGGTGCTAATG	0.023	0.004	0.025	33	1281
AGCGTCATAAACA	0.018	0.002	0.02	82	4024
GGCAGTGTAGACG	0.061	0.012	0.046	51	1067
GGCGTCATAAACA	0.062	0.004	0.056	140	2368
GCCGTTGTTGGCA	0.051	0.007	0.059	57	906
ACCGTCGTATGGG	0.022	0.01	0.033	28	808
CCTATCACAAAGA	0.024	0.002	0.023	82	3492
AGCAGCGCAGATA	0.024	0.001	0.022	46	2014
AGTAGCACTTGCA	0.022	0.001	0.023	26	1114
AGCGTCACATGTG	0.023	0.004	0.018	24	1338
GGTATTGCAAAGA	0.08	0.012	0.08	106	1212
ACCAGTGTTAGGA	0.028	0.006	0.02	32	1593
ACCAGTGCTGAGG	0.024	0.004	0.02	28	1406
CCTATCACATATA	0.024	0.002	0.027	103	3774
AGTATTACAGGTG	0.029	0.007	0.039	44	1088
ACCGTCATTAGCA	0.021	0.002	0.021	58	2770
GGCATTGTTAACA	0.069	0.006	0.067	144	2014
AGTATCGCTAACG	0.024	0.004	0.02	28	1375
AGCAGCACTAATG	0.022	0.003	0.019	41	2173

ACCGTTGCAGGGA	0.021	0.004	0.027	37	1356
AGCATTGTTGAGA	0.022	0.003	0.02	40	1952
GGCAGCATAAAGG	0.049	0.012	0.041	60	1390
ACTAGTACTTGTG	0.023	0.002	0.02	31	1555
ACTATTATTGGGG	0.034	0.007	0.037	44	1158
GGTGTTATTTACG	0.092	0.007	0.082	58	647
GGCATTGCTGACG	0.068	0.006	0.06	65	1024
ACCGGTACAGGGA	0.023	0.001	0.024	34	1395
ACCAGTACATAGG	0.022	0.004	0.023	64	2684
CGCAGCACTGAGG	0.02	0.002	0.022	21	954
CCCAGTATTTACA	0.026	0.004	0.021	77	3631
AGCAGTACAAATA	0.021	0.002	0.022	106	4689
AGCAGTGCATATA	0.022	0.002	0.022	54	2359
CCTATCATTTGCA	0.031	0.008	0.025	62	2380
ACCAGTGCAAATG	0.023	0.002	0.026	81	3092
CCTATTGTAGGTA	0.023	0.007	0.019	35	1837
AGCGTCGTTGAGA	0.022	0.005	0.026	31	1179
CGCAGTGCTGGCA	0.026	0.002	0.029	29	979
CGCGGCACTAGTA	0.029	0.005	0.035	48	1310
CCCGTCGCTAGCG	0.023	0.003	0.018	25	1371
CGCGGCACATACA	0.024	0.003	0.021	46	2175
CGCGGTATATGTA	0.022	0.003	0.02	33	1628
GGCATCACTGGTA	0.062	0.008	0.053	68	1211
CGTATTGTAAGTG	0.024	0.007	0.021	24	1122
ACCATCATTAAGA	0.023	0.002	0.021	88	4099
CCTAGCATTAAGA	0.024	0.001	0.022	49	2187
AGTAGTGTATACG	0.028	0.005	0.024	33	1323
GGTAGTACAAGCA	0.056	0.013	0.039	48	1191
CCCGGTGTAGGGA	0.025	0.005	0.019	17	876
CCCGGTACATGCG	0.025	0.007	0.023	37	1573
AGCAGCATATGTG	0.019	0.003	0.015	27	1785
ACCATCATTGGCA	0.023	0.001	0.021	67	3085
CCTAGTGCAGATG	0.024	0.003	0.027	39	1402
CGTGGTGCAAACG	0.028	0.005	0.022	19	859
ACTGGTATTAAGA	0.021	0.003	0.022	43	1926
AGTATCGCAAACG	0.027	0.002	0.028	45	1586
GGCATCATTAATA	0.085	0.009	0.078	218	2566
CCCAGCACTAGCA	0.025	0.003	0.024	68	2806
ACTGTTGTTGAGG	0.028	0.004	0.023	20	833
ACTATCGTTTAGA	0.03	0.007	0.02	34	1637

AGCGTTACAGACA	0.02	0.003	0.018	46	2575
CCTAGCGTTAACA	0.026	0.006	0.024	62	2527
CGCATTGTTAAGA	0.024	0.001	0.024	45	1849
AGCGTTGTTGACG	0.025	0.003	0.028	39	1353
GCTATTACAAGCA	0.054	0.002	0.055	112	1941
AGCGTCATTTACA	0.022	0.002	0.022	57	2500
ACTGGCGTTTATA	0.024	0.003	0.022	25	1122
CCCGTCATAAATA	0.027	0.003	0.023	102	4392
GCCATCGCTTGGA	0.06	0.009	0.047	39	784
GGCATCATATACA	0.078	0.015	0.067	207	2860
GGTAGTGCTGGGA	0.053	0.023	0.064	26	378
GCTGTTGCAAGTG	0.066	0.005	0.059	40	634
AGCGTTATAAACA	0.02	0.002	0.021	90	4235
CCTATTGCTTGCG	0.021	0.001	0.021	25	1178
CGCATTGCTGATA	0.026	0.004	0.021	38	1754
CGTATTATATGCG	0.033	0.007	0.026	35	1333
GGCGTTGTTGACA	0.062	0.012	0.046	55	1138
CGTAGTGCATAGA	0.026	0.005	0.02	16	789
GCTAGTATAAACA	0.065	0.005	0.057	184	3031
CCCGGTATAGACG	0.022	0.002	0.023	49	2068
CCCGGTACTTATG	0.031	0.001	0.032	54	1618
CGTAGCATTTGCA	0.03	0.004	0.025	29	1117
CCTATCACAGACA	0.024	0.003	0.028	108	3791
CCTGGTGCTAGTA	0.025	0.004	0.027	33	1189
CCTATTACTAATG	0.032	0.002	0.03	84	2681
AGCGGCACAGGTG	0.02	0.004	0.025	20	779
AGCAGTGCTGATA	0.022	0.004	0.018	36	1933
CCTGGCATAGGTA	0.031	0.006	0.026	34	1283
CCCATTATTAGCG	0.024	0.002	0.026	63	2358
CGCGTTACAAGTG	0.022	0.004	0.02	27	1338
CCCGTCATTGACA	0.029	0.002	0.029	90	2991
ACCGTTACTAGCG	0.028	0.006	0.035	82	2239
CGCATTATAGAGG	0.03	0.004	0.027	43	1557
GCCGTCACTAGCG	0.055	0.005	0.05	55	1037
AGTGGTACAGGTA	0.022	0.002	0.024	23	955
GCCATCACAAATG	0.052	0.004	0.047	125	2525
CGCGTTATAGAGA	0.021	0.005	0.013	22	1668
CCCAGTGTTAGTA	0.02	0.005	0.023	43	1831
CCCATTATATGCA	0.021	0.002	0.019	68	3574
AGCGTTATTGGTG	0.022	0.006	0.018	19	1045

CGTATCGCATGTG	0.031	0.003	0.032	25	766
GCTATTACTGAGG	0.06	0.005	0.054	51	896
CCCGTCGCAAACA	0.022	0.005	0.016	62	3804
GGTGGCACATATA	0.2	0.048	0.158	175	934
AGTATTACAAATA	0.024	0	0.024	93	3759
ACCAGCATTTGTA	0.027	0.002	0.029	84	2794
CCCAGTGCAAGCA	0.022	0.001	0.023	64	2752
CCCAGTATAGATG	0.026	0.002	0.024	66	2631
GCCAGTACTGGCG	0.053	0.01	0.056	71	1200
ACCATCACAGGGG	0.027	0.01	0.017	23	1350
AGTATCGCTAAGG	0.027	0.003	0.031	29	921
ACCATTATTTACA	0.027	0.003	0.023	115	4972
GCTATTGCAGGCG	0.054	0.009	0.043	48	1077
ACTAGTGTTAATG	0.028	0.006	0.023	46	1939
ACCGGCGCATAGA	0.023	0.002	0.021	26	1201
CGCATTATATAGA	0.029	0.001	0.027	72	2571
GGTGTTGTTGGCG	0.074	0.008	0.064	27	397
GCCGTTGTAAATA	0.049	0.005	0.047	107	2182
ACTATTGCTGACG	0.023	0.002	0.021	44	2079
CGCGGCGCATGGG	0.021	0.003	0.021	12	564
GGCGGTGTAGGGA	0.048	0.007	0.054	31	546
CCTGGCGCTGAGA	0.023	0.008	0.018	16	870
GCCGGTATTAGGA	0.074	0.01	0.071	84	1091
AGCGTCGCAAAGG	0.022	0.002	0.023	31	1338
ACCGGCATTTACA	0.026	0.004	0.021	59	2759
CGCAGCGCAAACG	0.019	0.001	0.021	39	1796
AGTAGCATATGTG	0.028	0.004	0.024	25	1003
ACTATTATTAGGA	0.029	0.003	0.026	62	2342
AGCGGTATTGACA	0.02	0.003	0.02	46	2202
ACCATCGTAAGCG	0.026	0.004	0.022	48	2175
CCTGTCGTAGATG	0.023	0.005	0.017	20	1184
GCTAGTATAAAGG	0.064	0.006	0.064	103	1502
GGCGTTGTTAGGA	0.071	0.018	0.084	58	635
AGTAGTGTTGGGA	0.021	0.006	0.013	8	632
AGTAGCACTAGCA	0.028	0.004	0.024	32	1302
CCCGGTGCAGGTG	0.027	0	0.027	25	887
CCTATCATTTGGG	0.029	0.007	0.019	21	1063
GCTAGTGCTTATA	0.055	0.001	0.056	72	1219
CGCGTTGCTGACA	0.025	0.006	0.016	26	1561
GGCATCACTAATG	0.095	0.013	0.082	152	1707

ACTGGCATTTACA	0.025	0.004	0.021	44	2053
CGTATTGCTTGGA	0.022	0.006	0.026	21	777
AGTAGTATTGGTA	0.026	0.003	0.024	30	1230
CCCATTGTAGGCG	0.024	0.002	0.024	42	1720
CCTGGCGCTAGCA	0.024	0.002	0.021	24	1144
CCTGGTGTATACG	0.03	0.002	0.027	33	1181
CGCGTTGTAGACG	0.027	0.003	0.031	36	1143
GGCAGCATTAGTA	0.05	0.004	0.052	78	1410
ACCGTCATTAGGA	0.028	0.004	0.03	60	1954
CCCGTTGCTGAGA	0.023	0.001	0.022	35	1526
AGCATCGTAGGGA	0.024	0.001	0.025	41	1604
AGCGTTGTTTAGG	0.023	0.01	0.033	30	881
CCCATTGCTTATA	0.026	0.003	0.022	61	2697
GGCGTCACTAGTG	0.061	0.001	0.062	47	709
AGTAGTGCTGGCA	0.022	0.005	0.02	18	900
AGTATTGCTGAGA	0.024	0.001	0.023	28	1198
ACCAGTATTGACA	0.021	0.004	0.018	81	4334
GGCAGCACTTACG	0.051	0.009	0.052	60	1093
ACCGTTACTTGTA	0.018	0.001	0.017	47	2738
CCCGGCGCATGCA	0.019	0.003	0.015	32	2035
CGTGGTATATACG	0.027	0.006	0.022	21	936
AGTATCACATGTA	0.023	0.003	0.022	33	1483
GCCAGTGTAAAGA	0.05	0.007	0.056	114	1918
ACCGGCGTATACA	0.026	0.004	0.021	41	1941
GCTGGCGTTTATG	0.943	0.016	0.927	1055	83
ACTGGTGCAGGCA	0.023	0.003	0.019	27	1406
GGCGTTATTGATA	0.064	0.006	0.055	88	1498
AGCGTTGTATACA	0.019	0.001	0.021	50	2361
CCCATTGTATAGG	0.029	0.005	0.032	59	1808
CGCGTCATAAATA	0.021	0.002	0.019	58	3058
CCTATTATAAAGG	0.029	0.005	0.022	63	2773
AGCGTTATTGAGA	0.025	0.002	0.023	41	1776
GCCGGTGCAAATA	0.056	0.001	0.058	97	1578
AGCGGCGTTAGTG	0.021	0.005	0.027	16	577
CCCATTGCTGGCA	0.022	0.001	0.021	43	2047
ACTATTGTAAATG	0.029	0.005	0.024	71	2863
GCTGTTATTTATA	0.065	0.008	0.062	98	1484
CCTGTCATAGGGG	0.036	0.004	0.038	30	769
CCCATCACTGGTA	0.024	0.004	0.02	50	2492
ACTATTACAAATG	0.024	0.001	0.022	98	4312

ACTGTCATAGACA	0.023	0.001	0.023	62	2577
CCCGTCATAGACG	0.026	0.002	0.025	57	2254
CCTAGTATAAGTG	0.026	0.002	0.029	54	1823
GGTATCGCTAATG	0.133	0.024	0.1	86	773
CGCAGCATAAGCG	0.026	0.003	0.023	40	1724
AGTGTCGTTAGCG	0.02	0.003	0.021	15	712
CGTATCATAGGCA	0.025	0.002	0.026	43	1627
CCTGGTGCTTATG	0.025	0.001	0.026	21	793
ACCAGCACTAACG	0.021	0.002	0.024	91	3708
GCCATCATTTAGA	0.051	0.008	0.046	97	2011
CGCATTGCAAGCG	0.026	0.002	0.027	41	1465
CCCGTTGCAGGCG	0.024	0.004	0.027	40	1452
GCCGTTGTTGATG	0.052	0.01	0.039	35	857
CGTAGTACATACG	0.022	0.003	0.025	27	1042
CGCGGTACAAGGA	0.021	0.001	0.023	31	1326
CCCATTACTAACG	0.025	0.002	0.021	74	3396
ACTGGCACAAACG	0.03	0.004	0.026	53	1953
CCTATTGTATACG	0.026	0.004	0.028	64	2216
ACCATCGTAGGTA	0.028	0.003	0.028	60	2095
GCTGTTACTAGCA	0.064	0.015	0.043	48	1064
GCCGTCACAGGCG	0.053	0.003	0.048	53	1043
CCTGTTATATATG	0.026	0.002	0.024	48	1952
AGCGTCGCTAATG	0.026	0.007	0.034	49	1408
CCTGGCATTGAGA	0.029	0.004	0.024	32	1311
GCCATCGTAGGGA	0.05	0.02	0.022	21	942
CGCGTTACAGGGA	0.027	0.008	0.016	18	1096
ACCAGCATTTGTG	0.025	0.003	0.023	43	1862
CCCGGTACATGGA	0.024	0.002	0.028	44	1549
CGTAGCATTAACA	0.023	0.003	0.02	40	1922
CGCAGCACTAGGA	0.026	0.003	0.026	34	1291
CCCAGTACATACA	0.026	0.003	0.022	98	4322
AGCATCGCAGATG	0.025	0.001	0.024	43	1749
CCTGTCACTTGTA	0.035	0.002	0.039	53	1322
CCCAGTGCATAGA	0.027	0.003	0.024	49	2014
CCCATCGCATGTA	0.021	0.002	0.019	50	2559
CGTAGTACTAGTG	0.025	0.005	0.017	13	732
CCTATTGTTAACA	0.028	0.004	0.026	87	3263
ACTGGTATTAGCG	0.029	0.006	0.029	31	1032
CGCAGCGCTTGTA	0.022	0.006	0.029	35	1164
GCCGGCGCTGACA	0.479	0.029	0.462	700	814

GGCGGCACATACG	0.265	0.052	0.228	288	977
ACTAGTATAAGTA	0.026	0.002	0.024	79	3200
GCCGTCGCATAGA	0.052	0.005	0.05	46	883
AGCATTATAGACA	0.021	0.001	0.02	95	4644
AGCATTGCAGACG	0.021	0.006	0.015	30	2031
GCTAGCACATGTA	0.066	0.006	0.058	77	1257
ACCAGTATTAGTG	0.023	0.003	0.021	50	2300
CGTAGTGCTGGCA	0.02	0.007	0.013	9	696
AGCGGCGCTTGGG	0.029	0.011	0.018	9	480
CGCGGCGTATAGA	0.028	0.001	0.028	28	979
ACCGTCACAAACA	0.023	0.002	0.02	122	5990
CCTGTCGCTTGTA	0.022	0.003	0.022	25	1115
ACCATTACTTATG	0.025	0.002	0.026	96	3552
CCCATTATTGGTG	0.022	0.001	0.02	36	1721
CGCAGCGTTAGTG	0.025	0.006	0.019	18	932
GGCAGCACTAACG	0.054	0.012	0.046	75	1543
GGCGTTGCTTGTA	0.066	0.011	0.081	59	668
ACCGTTGTATACG	0.031	0.005	0.023	45	1905
ACTGTTGTAAATA	0.027	0.004	0.026	79	2904
CGCAGTACTTGCG	0.021	0.001	0.022	22	994
AGTATTATATACG	0.021	0.003	0.021	52	2399
ACCATTACTGAGA	0.019	0.002	0.017	63	3706
CCCGTTACTAACA	0.029	0.004	0.023	87	3653
GCTAGTGTTAGTG	0.059	0.009	0.048	38	756
GCCGTCATTAGCA	0.055	0.009	0.062	110	1675
GGCAGTGCATACG	0.05	0.005	0.046	52	1077
CGCATTATTAGTA	0.026	0.003	0.022	54	2440
AGTAGCGTAAGTA	0.018	0.005	0.011	16	1393
CCCGTTACTGACA	0.025	0.001	0.025	74	2860
CGTAGCATAGAGG	0.037	0.011	0.03	21	687
CCCGTCGTAAGTG	0.024	0.001	0.023	34	1440
CCTAGCACAGACG	0.029	0.004	0.024	40	1657
CCCGTTATTAGGA	0.024	0.002	0.022	40	1756
CCTATCGTTTATA	0.029	0.005	0.029	63	2147
AGCGGTGCTGGGA	0.021	0.003	0.019	14	708
CCCATCATTTGGA	0.022	0.002	0.024	50	2039
CCCGGCATTAAGG	0.027	0.004	0.028	41	1439
AGCGTTGTTTGGG	0.027	0.004	0.028	18	629
CCCATTACTAGCG	0.027	0	0.027	61	2205
CGCGTTGTTTGTA	0.019	0.003	0.019	20	1048

GCTAGCGTAGGTG	0.08	0.013	0.083	45	497
AGTGTCGCAAGGA	0.027	0.007	0.036	29	769
AGCATTGCTTACG	0.026	0.007	0.025	48	1842
CGTGGTGTTGACG	0.033	0.005	0.03	17	548
GGCGTTACAGACG	0.058	0.008	0.047	55	1112
GCCGGTACATACG	0.075	0.006	0.083	124	1370
CGTGGTATATATA	0.032	0.005	0.025	33	1313
GCCAGCACATGCG	0.058	0.006	0.059	84	1348
CCTGTTGCAGATA	0.028	0.003	0.025	47	1847
CGCATTGTAAAGG	0.03	0.007	0.028	47	1630
AGCAGTGCAGATA	0.021	0.001	0.022	50	2200
CGTGGTGCTGAGG	0.018	0.006	0.01	4	391
CCCAGTACTTGTA	0.024	0.004	0.029	65	2210
GGCAGTGTATACG	0.054	0.005	0.05	64	1214
AGTGTCATAAGTA	0.021	0.003	0.02	34	1693
GGCATTACAAGTG	0.065	0.005	0.059	91	1445
CCTGGTGTAAAGA	0.022	0.003	0.019	32	1659
GGCGTCACTGGCA	0.058	0.019	0.042	48	1093
GCCGTTGCATAGA	0.045	0.007	0.036	39	1048
ACCATCACATACG	0.026	0.001	0.024	103	4103
AGTAGTATAGGTG	0.023	0.003	0.025	24	921
AGCGTCACTGGTA	0.02	0.005	0.019	28	1454
CGCAGCATTGATA	0.031	0.005	0.024	54	2169
GCCGGTGCATATA	0.065	0.009	0.061	81	1237
GCCAGTGTAAATG	0.049	0.006	0.041	71	1679
ACTGGTACTGGCG	0.029	0.002	0.027	27	982
CCTGTTGCTTATG	0.023	0.002	0.022	23	1028
GGTGTTATTTGCA	0.083	0.011	0.086	66	699
AGCATCACAGACA	0.019	0.002	0.02	77	3737
AGCATCACTTGCG	0.022	0.001	0.02	35	1677
AGCGGCATAGGTA	0.021	0.006	0.014	16	1151
CCCATTGTTGACA	0.025	0.001	0.025	75	2877
GCTGTTACAAACA	0.055	0.004	0.053	119	2124
GCCAGTATTTATG	0.05	0.003	0.048	87	1744
CCCGTTGCTTACG	0.026	0.005	0.03	52	1684
ACTATCACAAGGG	0.033	0.009	0.044	63	1362
CCTGGCGCAAACG	0.026	0.004	0.02	32	1535
ACCAGCGCAGAGA	0.018	0.002	0.016	43	2691
CGCGGTATTTACA	0.026	0.001	0.024	44	1766
CGTATCACATATG	0.029	0.006	0.021	29	1365

CGCGTCGCTTATG	0.022	0.002	0.025	24	939
ACCGTCATAGGCA	0.025	0.001	0.025	62	2443
AGTGGTGCTAAGG	0.025	0.009	0.013	8	625
AGCGTTACTGACG	0.021	0.005	0.015	23	1556
AGCGTTGTTGAGG	0.02	0.002	0.02	17	814
GGTATTACTAGCG	0.117	0.013	0.121	99	717
ACCATTATTAGTG	0.024	0.003	0.024	65	2641
CCCGTCATAGGCA	0.025	0.003	0.023	55	2388
ACTATTATTAGTG	0.028	0.005	0.021	46	2168
ACCGGTGCTTACG	0.023	0.005	0.016	23	1391
AGTGGCGCTGGTG	0.016	0.003	0.012	4	340
GGTAGCGTAGGTG	0.079	0.018	0.057	23	380
AGCGGCACATGCA	0.028	0.007	0.031	52	1604
ACCAGCGCAGGCG	0.023	0.004	0.018	30	1650
CCTGGTATAAACG	0.027	0.002	0.029	57	1929
CCTGGCATTAATA	0.028	0.004	0.023	54	2248
AGCAGTACATGCA	0.021	0.003	0.022	59	2603
AGCGGTGCATAGG	0.027	0.004	0.032	26	778
ACTAGCGCAGATA	0.022	0.003	0.026	67	2501
CGCATCGTTAACA	0.023	0.002	0.021	60	2738
GCCAGTGCAAGGA	0.045	0.005	0.045	50	1051
CCTATTGCAAGCA	0.03	0.006	0.021	59	2720
CGTATCGTATGTA	0.025	0.009	0.019	27	1370
CGCATCGCTGGCA	0.024	0	0.023	34	1430
AGCAGCACTAACA	0.019	0.002	0.022	77	3415
GCCATTGTATGCA	0.048	0.004	0.053	96	1715
CCCAGTGCTTACG	0.023	0.005	0.016	28	1702
CCTATCATTGGCA	0.022	0.002	0.021	44	2080
ACCGTTACTGACG	0.026	0.005	0.021	50	2336
GGTAGCATATACG	0.079	0.014	0.073	72	920
ACTAGCATTTGTG	0.031	0.002	0.033	45	1337
ACCGTCGCTGACA	0.026	0.002	0.025	65	2564
GCCGGCACAGAGA	0.125	0.018	0.115	150	1155
GCCATCACAAGCG	0.052	0.001	0.05	102	1922
GCCATCATTAACG	0.058	0.004	0.062	174	2635
ACCAGTGTATGCA	0.024	0.003	0.026	64	2431
CGCGGTGCAAGTA	0.018	0.001	0.017	23	1331
CCTGGTATAGACG	0.029	0.003	0.025	36	1424
AGCGTTGCAGGCG	0.024	0.006	0.019	21	1080
AGTGTTGTTGACA	0.031	0.011	0.025	31	1231

AGTAGTATAAAGG	0.028	0.009	0.021	33	1544
ACCGGCGTAGACA	0.03	0.003	0.029	58	1928
ACCATTATTTAGA	0.019	0.004	0.017	62	3617
GGCAGTACAGAGG	0.05	0.005	0.047	46	939
CCTGGTGTTTGCA	0.021	0.002	0.023	24	1041
AGCGGCACTTACA	0.016	0.002	0.014	25	1758
CCTGGCATTGGGA	0.021	0.008	0.022	17	765
ACCATCATTGATG	0.028	0.004	0.022	70	3093
GCTATTATTAGTG	0.072	0.006	0.066	90	1265
AGTATCGTAAAGA	0.022	0.003	0.018	38	2043
GCCAGCACTAATA	0.05	0.002	0.047	131	2628
GCTGTCACAAATA	0.056	0.001	0.057	113	1867
GCCGGTGCAGAGG	0.13	0.02	0.107	69	577
CCTGTTATATACG	0.022	0.003	0.026	50	1874
ACCAGTGTAAATG	0.028	0.001	0.027	72	2641
CCCAGTATAAGTG	0.027	0.001	0.028	66	2252
ACCATTATTGGCG	0.021	0.002	0.02	55	2692
GGCGTTGTATGCA	0.065	0.016	0.05	49	924
CCTATCGCTAAGA	0.024	0.004	0.03	60	1967
ACTGGCATTTATA	0.023	0.004	0.028	56	1977
CCTATCACAAACG	0.026	0.004	0.025	87	3406
CCCGGCGCAGATA	0.024	0.003	0.021	43	1958
GCTGTCGTATGCA	0.064	0.007	0.069	68	922
GCCAGCACAAAGG	0.055	0.003	0.051	82	1514
GGCATTATTAGGA	0.059	0.006	0.051	72	1334
CCCATCGCTGGCA	0.018	0.003	0.014	33	2346
GCTGTCGTTTGTA	0.051	0.015	0.036	24	646
GCCGGTGCAGGTA	0.064	0.008	0.06	49	768
AGCGGTGTTGACA	0.024	0.003	0.021	34	1596
AGCAGCGCTTACG	0.023	0.003	0.022	32	1431
CCTATTGCTTGCA	0.025	0.003	0.027	53	1899
CGCATCACTTGGA	0.024	0.002	0.022	29	1313
AGCGTTATTTATA	0.018	0.002	0.016	41	2522
GGTATTATTAACG	0.109	0.013	0.101	145	1290
CGTAGCACAAGCA	0.022	0.004	0.027	38	1377
ACCATTACTTATA	0.022	0.002	0.023	122	5200
AGCGGTGCTGAGG	0.023	0.002	0.02	14	690
GCCAGTATAAATA	0.056	0.003	0.052	239	4315
CGTGTTATTTGTG	0.028	0.005	0.029	21	695
CCCGGTGCTGACA	0.03	0.005	0.027	54	1927

ACCGTCGTTTGCA	0.028	0.009	0.022	32	1437
CCCAGCATTTGTA	0.029	0.001	0.028	64	2237
GGCATTGTTGATG	0.079	0.013	0.069	74	1002
CCCATTGCATGTA	0.026	0.001	0.026	65	2469
CCTATCGTTTGCG	0.026	0.001	0.028	34	1192
CCCAGCACTTACG	0.023	0.004	0.028	64	2229
ACTAGCACTAGCA	0.023	0.004	0.018	49	2719
AGTAGCGCTAGGA	0.029	0.009	0.018	13	717
CCTGGCATATATG	0.025	0.004	0.02	28	1362
ACCATTATAGATG	0.026	0.003	0.021	76	3486
ACCGGTGCAGGGA	0.018	0.003	0.022	28	1225
CCTAGTACTAGTA	0.031	0.003	0.027	53	1916
AGTATTACATACA	0.021	0.001	0.02	59	2864
CCCGTTACTAGCA	0.02	0.003	0.025	66	2550
ACCAGTACAAATG	0.021	0.001	0.021	102	4730
CGTATCGTAAGGA	0.03	0.007	0.02	25	1236
ACCGGCACAAGTG	0.026	0.003	0.03	61	1990
ACTAGCACAGGCA	0.023	0.002	0.023	58	2413
CGCGGTATAGGCG	0.031	0.011	0.033	31	918
CCTATTGCATATA	0.024	0.002	0.027	76	2787
CGTGTCACATACG	0.022	0.003	0.018	22	1172
ACCGTCACTGAGG	0.023	0.002	0.021	32	1486
GGCGGTACATACG	0.072	0.018	0.059	62	981
ACCGTCGCAGAGA	0.025	0.003	0.021	39	1828
GCTATTGTATACG	0.056	0.003	0.053	83	1491
CCCGGTGTAGATG	0.026	0.007	0.033	41	1204
GCTGTCGCATGGG	0.045	0.021	0.017	6	357
GGCGTTACTTGGA	0.064	0.018	0.039	31	771
ACTGTTGCTAGCG	0.027	0.001	0.027	30	1075
ACTATCACTTATA	0.024	0.003	0.028	106	3647
CGCAGCGTTTATG	0.024	0.005	0.025	30	1154
CCCATTATTTGTG	0.024	0.005	0.029	61	2016
CCCGTTATTAGTA	0.028	0.007	0.026	59	2191
ACTGTCGTATACG	0.026	0.007	0.026	32	1206
ACTAGCACATGCA	0.026	0.004	0.029	80	2635
CCTGTCACTAATA	0.022	0.004	0.026	64	2395
ACTGGTACTGACA	0.023	0.004	0.026	65	2407
CCCGGTGCTAATA	0.026	0.003	0.03	71	2335
ACCAGTGTTAGTA	0.023	0.003	0.019	39	2012
CCCGTTGCAGACG	0.028	0.006	0.021	38	1761

GCCATTGTAGGGG	0.046	0.008	0.038	32	819
ACTAGTATTGACG	0.026	0.001	0.028	55	1924
CCCATCGCAAACG	0.022	0.006	0.022	76	3304
CGCGTTACTAGTA	0.018	0.004	0.023	35	1456
GCCGTTGTTTAGG	0.052	0.008	0.047	33	671
AGCGGCATTGAGG	0.027	0.01	0.019	9	455
ACCAGCGCAAGTA	0.021	0.001	0.023	80	3396
CCCGGCATTGGGA	0.03	0.004	0.03	34	1106
ACCATTATATATG	0.021	0.002	0.021	85	4049
ACTAGCACTTGCA	0.026	0.004	0.022	49	2192
GGCGTCATTAATA	0.065	0.007	0.06	118	1836
AGCAGTGCTTGCA	0.02	0.002	0.022	34	1482
AGTGGTATAGACA	0.021	0.002	0.02	31	1536
ACTGGCACTAGGG	0.028	0.002	0.031	23	726
GGTGTCGCATACA	0.081	0.025	0.064	55	804
CGCGTTGCTTACA	0.028	0.005	0.026	40	1489
CGCGTTATTTAGG	0.032	0.002	0.034	31	881
GGCAGCACAGGTG	0.052	0.008	0.047	41	838
GCTGTCATATACG	0.065	0.008	0.057	71	1172
CGCGTTACTTACA	0.02	0.007	0.011	21	1894
GGTATCATTAGTG	0.126	0.013	0.113	77	602
AGCGGCATTAATG	0.025	0.007	0.035	28	774
GCCGTTGTTAAGA	0.054	0.004	0.05	67	1262
AGCGGTACTGGCA	0.021	0.003	0.018	26	1406
ACCAGCGTAAGGG	0.023	0.002	0.024	28	1158
AGCATTACATAGG	0.021	0.001	0.02	41	2007
ACCGTTGTTGACG	0.029	0.002	0.031	51	1601
GGCGTTGTTAGTG	0.06	0.016	0.039	25	615
ACTGTTATAGAGA	0.026	0.004	0.02	41	2014
ACTATTATTAATG	0.035	0.001	0.033	115	3373
CGCAGTACAGAGA	0.024	0.004	0.02	35	1684
GGTGTCATTGATG	0.113	0.019	0.09	58	587
CGCAGCGTAGAGA	0.026	0.007	0.018	25	1330
CCCGTTATTAACG	0.026	0.002	0.023	60	2552
GGCAGCGTAAACG	0.057	0.005	0.054	85	1475
GGTATTGTAAGCG	0.103	0.006	0.097	79	735
AGCATTGTAAAGA	0.022	0.001	0.021	64	2939
ACCATTGCTGGGG	0.023	0.006	0.018	21	1154
AGCATTATAGATA	0.022	0.003	0.021	98	4498
GGCGGTGCATACA	0.05	0.01	0.043	53	1172

GGCGTTGTATGCG	0.062	0.004	0.058	44	710
CCTGGTATTGACG	0.029	0.003	0.026	33	1256
GCCAGCGCATGCA	0.047	0.006	0.044	56	1230
GGCGTTGCAGGCG	0.064	0.013	0.047	40	809
CCTAGCGCTGACA	0.022	0.004	0.027	57	2025
GGCATCATTAATG	0.1	0.008	0.092	179	1757
CGCGTCATTTATG	0.026	0.007	0.031	42	1331
AGTATCGTAAATA	0.022	0.002	0.022	62	2699
GGCAGTACAGATG	0.06	0.01	0.047	65	1305
GCCATCACAGATG	0.045	0.005	0.044	91	1965
CGCATCGCTAATA	0.024	0.001	0.023	53	2298
CGCGTTGCATATA	0.021	0.003	0.02	33	1635
GCCATTACTAGCG	0.049	0.006	0.053	82	1452
CGTATTATATGGA	0.022	0.003	0.023	35	1514
ACTGTCGCTGAGG	0.028	0.009	0.018	15	815
CCCGTTATTGAGG	0.025	0.003	0.021	30	1371
AGCGTCGTAAGTG	0.021	0.008	0.017	23	1300
ACCGTTACAGGCA	0.022	0.002	0.02	63	3097
CGCGTTACTGGCG	0.021	0.007	0.012	13	1041
GGCAGTATTTGCA	0.053	0.006	0.054	80	1410
ACTATTATAGATA	0.032	0.002	0.03	129	4194
GGCGTCATTGACG	0.06	0.003	0.057	70	1148
GCTGTCACAGGCA	0.059	0.005	0.065	65	936
GCTGTCGTTAGTA	0.052	0.003	0.055	45	768
GGCATCACTGGCG	0.063	0.002	0.064	87	1279
AGTAGCACAAAGA	0.024	0.005	0.019	35	1815
GCCAGCGTAGAGA	0.053	0.007	0.061	76	1171
CGTGGTATTGACG	0.026	0.01	0.028	21	736
CCTAGTACTTGGA	0.031	0.007	0.022	29	1287
GCCGTCACTGACG	0.05	0.002	0.047	66	1348
GGCAGTATAAGCG	0.057	0.013	0.043	62	1364
GGCGTTACTTGGG	0.074	0.006	0.072	38	492
CGTAGTATATGGG	0.026	0.006	0.018	13	704
CCCGTTGCTGGCG	0.025	0.002	0.025	29	1128
GGCATTACATGGA	0.058	0.012	0.044	60	1317
AGCGTCATAAGGA	0.019	0.003	0.017	34	1915
CCCATCGTATGCG	0.024	0.003	0.019	40	2030
GGCATCGCATGCA	0.081	0.011	0.084	117	1276
GGCAGTATTGACG	0.057	0.005	0.051	65	1211
CCCGTCGCTGACG	0.023	0.003	0.026	46	1714

CCCAGTGCAGATA	0.024	0.004	0.02	53	2622
AGCGGTGTAGAGG	0.022	0.007	0.012	10	808
CCCGGCGCTAGTG	0.021	0.003	0.024	26	1043
CGCGGTGCTGACA	0.023	0.003	0.019	25	1275
ACCAGTGTTTGTG	0.029	0.006	0.021	27	1247
CCCGGCGCTGAGG	0.021	0.004	0.016	15	926
CCCATTGCAGACA	0.024	0.001	0.022	80	3549
ACCATCGTTTGGG	0.033	0.002	0.031	35	1105
GGCGTTGTAGGCG	0.069	0.012	0.055	41	709
CGCAGTATAGGCA	0.023	0.006	0.018	35	1936
CGCAGTATAAGCG	0.026	0.004	0.022	38	1721
ACTAGTACAAACA	0.023	0.002	0.022	128	5645
CCTGTCACAGATA	0.025	0.006	0.018	40	2153
GCCATTGTTGGCA	0.055	0.008	0.045	61	1300
CGTGGTGCAGATG	0.025	0.006	0.016	10	609
CCTATCACATACG	0.031	0.004	0.025	72	2769
AGCGGCATTTGTG	0.027	0.003	0.025	12	465
GCCAGTACTTACA	0.052	0.001	0.052	124	2251
ACTGTCACATACG	0.023	0.004	0.019	37	1907
AGCGTTACTAGTA	0.019	0.002	0.017	30	1739
CGTGGTGTTTGCG	0.015	0.003	0.014	6	430
AGCGTTACTGAGA	0.021	0.005	0.016	26	1561
GCCATCACAGAGA	0.052	0.004	0.05	107	2019
AGTGGCACAGACA	0.022	0.001	0.021	27	1285
CGTGTTATAAGGA	0.02	0.001	0.02	23	1155
CCTATTGTTTGCG	0.029	0.003	0.028	36	1240
GCCATCGTAGGTG	0.049	0.003	0.044	42	912
ACCAGTACATGGA	0.022	0.004	0.017	53	3034
AGTGGTACTAGCA	0.021	0.007	0.02	22	1091
CCTAGTGCATGCA	0.026	0.002	0.029	56	1897
AGCGTCGTAGACG	0.025	0.005	0.021	31	1455
GGCGGTGCTGAGA	0.064	0.006	0.061	41	634
GCTGGCATAAGTA	0.66	0.042	0.681	1201	563
GGCATTATTAGTA	0.061	0.009	0.049	97	1873
CGCGGTATATAGA	0.022	0.001	0.021	33	1544
AGCAGCGCAGGTG	0.02	0.001	0.022	21	938
CCTATTGTTAGCA	0.024	0.002	0.021	45	2065
CCCGTCGCTTATA	0.022	0.001	0.021	44	2066
AGCAGCATTGACA	0.022	0.002	0.02	60	2976
ACTAGCATAAACA	0.027	0.002	0.025	114	4445

GCCGGCACATGTA	0.11	0.008	0.101	150	1337
ACCGTCGTAAACG	0.024	0.002	0.026	54	1984
AGCAGTATTGATG	0.019	0.002	0.019	39	2000
CCTAGTACTAATG	0.025	0.002	0.024	46	1888
CCCGTTATTTATG	0.021	0.005	0.015	30	1910
CCCATCATAGATA	0.025	0.001	0.024	107	4288
CGCGTTGTAAATA	0.026	0.001	0.027	64	2313
CCCGGCGTTTGCA	0.019	0.004	0.015	25	1594
CGCAGTGCATGGA	0.023	0.003	0.019	18	919
CGTAGTACTTGGG	0.031	0.013	0.017	8	463
GGTATTGCAAATA	0.076	0.006	0.075	129	1598
CCTAGTGCTAACA	0.028	0.007	0.018	49	2747
AGCATTATATGGA	0.018	0.001	0.018	46	2448
AGTATCACTAATG	0.026	0.001	0.027	45	1610
GCCATTATTGACG	0.057	0.005	0.05	109	2070
ACTGGTATTAATA	0.029	0.005	0.028	78	2734
ACTAGTATTGGCA	0.027	0.003	0.031	60	1878
GGCGTCACTTGTG	0.064	0.011	0.057	41	681
CCTGTCGTTTGCA	0.017	0.006	0.01	13	1274
ACTGTCACAGGTA	0.02	0.003	0.017	33	1859
CCCGTTATATACG	0.027	0.004	0.023	59	2531
AGCATTACAAACG	0.021	0.002	0.018	72	3836
ACCGTTACTGGTA	0.023	0.005	0.016	38	2303
GGTATCACTAAGA	0.095	0.013	0.082	97	1092
GCCAGCATTAGTG	0.061	0.008	0.05	71	1339
GGTGGCACAAAGA	0.212	0.022	0.214	232	854
CCCGTCATTTACA	0.026	0.002	0.029	90	3060
CCTATCATAAGTG	0.026	0.003	0.021	53	2453
AGTATCATTGGGG	0.026	0.004	0.024	17	706
ACCGTTGCATAGG	0.022	0.006	0.022	32	1425
ACCATTACTGGTG	0.021	0.002	0.02	44	2210
AGCAGTGCAAATG	0.021	0.002	0.022	49	2222
CGCGTCACTTGTG	0.028	0.004	0.022	23	1019
CGTATCATAAACA	0.027	0.001	0.027	97	3542
CCTGTTACAGGCA	0.023	0.005	0.022	40	1781
CCTAGCACATACA	0.026	0.002	0.028	85	2928
ACTGTCACTGACA	0.027	0.003	0.026	67	2480
ACCAGTGCAGACA	0.021	0.002	0.018	64	3498
CCTAGTATAGACG	0.022	0.004	0.019	39	2013
CCCATCGCTTGTA	0.026	0.003	0.023	54	2246

ACCATTATTTGTA	0.023	0.003	0.022	75	3347
CGCGGCGCAGGTG	0.018	0.002	0.019	12	623
AGCGGCATTAACG	0.028	0.003	0.025	22	846
CGTATTGTATAGG	0.029	0.005	0.023	21	893
CCCATCGCATGCA	0.022	0.002	0.02	59	2850
GCCAGTGTAGGTA	0.048	0.013	0.051	64	1179
GCTAGCGCATAGA	0.066	0.002	0.065	55	797
GCCGGCGTATATA	0.861	0.021	0.855	2068	352
CCCGTCGTTAGCG	0.022	0.003	0.02	28	1359
GGCATTACTTGGA	0.063	0.007	0.055	60	1024
CGTAGTGTTTATA	0.023	0.002	0.025	26	1004
CCTAGCGTAGGCA	0.026	0.003	0.029	46	1535
AGTAGTATTAGGA	0.022	0.001	0.024	28	1147
CCTAGCGCAAACA	0.025	0.003	0.021	70	3318
AGTGTCATTAGCG	0.026	0.005	0.022	20	890
GCCATTACTTGCG	0.06	0.008	0.055	75	1298
CCCGGCACAGGCG	0.03	0.004	0.028	45	1537
CCTGTCGTAGACG	0.023	0.007	0.016	23	1396
ACTGTTACATGTA	0.02	0.004	0.016	39	2357
ACCGTTGCAAATG	0.02	0.004	0.021	54	2502
GGTGGTACAAAGG	0.096	0.012	0.081	48	546
GGCGGCATTGAGG	0.536	0.066	0.506	475	463
ACTGTTGCTAACG	0.023	0.004	0.018	30	1663
CCCGGCGCTAGTA	0.02	0.002	0.019	30	1589
ACCAGTACTAGCA	0.022	0.001	0.02	87	4185
GGTATTACTTAGA	0.095	0.012	0.083	89	987
GGTAGCACAAACG	0.08	0.009	0.083	95	1055
AGTGGTACTAAGG	0.024	0.005	0.031	24	751
ACCGGTGTAAATA	0.025	0.003	0.021	66	3069
ACCAGTACAGAGA	0.02	0.001	0.018	70	3857
GCCGGCACATGCA	0.108	0.013	0.093	149	1447
ACTATCATTTAGG	0.026	0.003	0.024	39	1611
ACCATTATTTGTG	0.022	0.004	0.026	59	2177
GCTGGTGTAAGTG	0.728	0.049	0.754	798	261
CGTGGTGCTGGCA	0.015	0.007	0.011	7	640
GCCGTCATATAGG	0.06	0.005	0.056	68	1156
ACTATTATTGGGA	0.024	0.002	0.021	33	1563
AGCAGCGCTAGCG	0.018	0.005	0.011	13	1178
AGCATTGCTAAGA	0.023	0.003	0.022	49	2136
GCCAGCATTTATA	0.053	0.003	0.055	142	2459

ACTATCGCAAGGA	0.024	0.004	0.019	38	1921
GCTGGTGCTAATA	0.115	0.018	0.122	135	970
CGCATTGTTGACA	0.027	0.005	0.025	52	2056
AGCAGCATTAACA	0.022	0.001	0.022	86	3814
GCTGGCATTTACA	0.66	0.033	0.637	1165	663
CGTGGCGTTAGTG	0.028	0.003	0.033	12	356
GGCGTTATAAACG	0.054	0.003	0.055	96	1637
CGTGTCGTAAGCA	0.026	0.005	0.033	39	1150
GCCGGTACAAGGA	0.052	0.006	0.054	72	1262
ACCGTCGCTGATA	0.025	0.002	0.025	59	2294
AGCAGTGTTGGCA	0.018	0.003	0.015	23	1471
CCTGTTACATGTA	0.026	0.003	0.022	39	1751
CCCATCGCTTAGA	0.026	0.004	0.021	45	2109
ACTATCACTGACA	0.024	0.005	0.017	60	3525
CCCGTTGTTTATA	0.022	0.004	0.017	34	1913
CGCATTGTTAGTG	0.024	0.003	0.028	33	1156
CGCGGTGTTAGTA	0.028	0.006	0.02	18	878
AGTATCGCTGACA	0.024	0.004	0.018	28	1543
CCCATCACTTGTA	0.026	0.002	0.024	62	2554
CCCATTACTGGTA	0.032	0.003	0.027	65	2335
AGTATCACTGGTA	0.024	0.007	0.014	16	1102
CCTGTCGTAAGGG	0.022	0.004	0.019	16	828
AGTATCGTATACA	0.02	0.002	0.02	45	2238
GGTGTCACAAATA	0.088	0.011	0.077	120	1442
ACCAGTGTTAACA	0.024	0.002	0.027	97	3476
CGCATCGCATGCG	0.024	0.002	0.024	33	1326
ACCGTCGCATAGA	0.019	0.002	0.017	33	1935
GCTATCATAAGTA	0.059	0.005	0.059	119	1912
CCCAGTGTAAGTG	0.023	0.002	0.025	43	1711
CGCAGTGTAAAGA	0.023	0.005	0.02	41	2003
ACCATTACTAAGA	0.02	0.003	0.016	81	4896
GCCGTTGTAAGCG	0.057	0.009	0.064	75	1101
CGCATCATTAACA	0.023	0.001	0.024	96	3902
ACCATCGTTAATA	0.024	0.003	0.02	73	3628
GGCAGCACAGATG	0.053	0.004	0.047	58	1180
ACCATTACTAGCG	0.021	0.001	0.022	72	3274
GGCAGCGTATGCA	0.065	0.002	0.062	78	1177
AGCATCATATGTG	0.023	0.005	0.018	39	2141
CGTGGCATAAGTG	0.026	0.004	0.031	18	555
ACCGGTGCAAAGG	0.031	0.008	0.024	36	1463

AGCAGCGCAGGGA	0.02	0	0.019	21	1086
GGTGTTGCTAATA	0.08	0.01	0.077	70	834
AGTGGCGCTAAGG	0.025	0.009	0.03	15	492
ACTAGCGTTAGGG	0.03	0.01	0.044	30	649
CCTGGCGTTTATA	0.023	0.004	0.021	31	1449
GGCATTATTGGGG	0.045	0.016	0.038	29	741
CCTGTTGCAAGTG	0.027	0.001	0.026	31	1150
CCTAGTACTAACA	0.021	0.004	0.02	65	3161
ACCAGCATTGGTG	0.023	0.004	0.022	34	1547
GGCGTTACTAATA	0.066	0.008	0.072	132	1700
CGCGGCGTATACA	0.026	0.004	0.021	31	1469
AGCGGTGCATAGA	0.026	0.006	0.022	27	1191
GCCGGCGCAAACG	0.744	0.045	0.75	1467	489
CCTAGCATAGGCA	0.028	0.005	0.032	61	1830
GGTGGTATAAGCA	0.136	0.023	0.121	112	816
CGCAGTGTAAATG	0.028	0.003	0.031	54	1704
CGCGTCGCTGGTG	0.028	0.005	0.024	17	687
CCTGTTATTGACG	0.026	0.004	0.021	35	1596
CGCATTGCATGTG	0.028	0.002	0.029	35	1158
GCCGTCACAGGCA	0.052	0.006	0.044	70	1538
CCTAGTATAAAGG	0.022	0.001	0.022	45	2019
CGTATTACATGCA	0.026	0.004	0.03	51	1658
CGCGGCACAAAGG	0.022	0.011	0.037	46	1190
GGTGTCGTAGATA	0.09	0.005	0.083	66	725
CGTATCGTTAACG	0.035	0.003	0.033	52	1503
CGCGGCGCTGATA	0.024	0.004	0.027	36	1276
GCTGGCACTTGTA	0.213	0.022	0.239	207	658
ACCAGCGCAAGCG	0.026	0.001	0.027	57	2068
ACTGGCGTATGTG	0.037	0.007	0.047	19	386
CCCAGTACTGACG	0.032	0.004	0.029	62	2072
AGCGGCGCTAACA	0.027	0.004	0.02	36	1725
CGCAGCGCATGTA	0.027	0.003	0.03	42	1337
ACTGGCATATGCG	0.027	0.007	0.018	17	951
CCCAGTGCAGACA	0.025	0.004	0.019	60	3031
CCCATTGTTAAGA	0.022	0.002	0.024	63	2542
CGCGGTGTTTATG	0.022	0.003	0.026	22	822
AGTGTTACAGACG	0.023	0.002	0.025	29	1148
GCCATTATATGCA	0.057	0.001	0.056	165	2785
GGCATCATTAACG	0.088	0.008	0.086	181	1934
CGTGTCGCAAAGG	0.024	0.003	0.027	21	754

ACCAGCGTATGTG	0.028	0.008	0.017	23	1313
CGCAGTGTATGCA	0.025	0.003	0.025	37	1472
CCCAGTGCAGGCA	0.024	0.006	0.018	38	2079
GGCAGCATAGGGG	0.051	0.007	0.045	40	855
AGCGGTGTAAGCG	0.021	0.004	0.017	21	1196
GGTAGTATAGGTA	0.064	0.009	0.053	51	905
ACCATCGCTTACG	0.024	0.001	0.025	54	2103
CCTGTTGTTAAGA	0.03	0.003	0.034	55	1581
CGCAGCGTTTGCA	0.027	0.004	0.028	37	1297
GGCATCGCATAGA	0.091	0.01	0.079	102	1197
GGCGTTACATGCA	0.056	0.012	0.043	58	1294
ACCAGCACTGGGA	0.025	0.002	0.024	38	1514
CGCATTATTTGGA	0.027	0.006	0.023	35	1481
AGTATTGTAGAGA	0.024	0.002	0.023	36	1506
GCCGTTACTAACA	0.056	0.007	0.051	118	2195
ACCAGTACAAACA	0.021	0.001	0.019	150	7836
AGTATTACTTAGA	0.023	0.005	0.016	27	1665
CCCATTGTATGCA	0.025	0.001	0.026	66	2510
CCCGGTACATACG	0.027	0.002	0.026	56	2108
GCTGTCATTAGGG	0.063	0.021	0.038	22	558
CCCGTCGCAAGTA	0.026	0.005	0.021	50	2289
ACCAGTGCTTATA	0.023	0.003	0.02	61	3017
GCCAGTACAGATA	0.053	0.001	0.052	132	2384
CGCATCGTAAAGA	0.021	0.001	0.022	55	2430
ACCGTCACTGGGG	0.028	0.01	0.042	36	828
AGTGTTACTTACA	0.02	0.006	0.011	18	1663
CGTAGCGTATGGA	0.023	0.003	0.025	18	716
CGTAGCATAGATA	0.027	0.002	0.029	49	1655
AGTAGTATTTATA	0.025	0.003	0.021	48	2217
GGTGGTACAAGGA	0.088	0.008	0.095	66	628
GGCGTCGCTAGTA	0.06	0.003	0.057	48	797
GCCAGTATATGGG	0.059	0.006	0.057	62	1018
GCTAGTACTGAGA	0.053	0.005	0.06	70	1095
ACCAGTGTAGAGA	0.022	0.001	0.023	53	2266
AGTAGTATAAACA	0.024	0.002	0.024	86	3568
AGTGGTGCATATG	0.021	0.008	0.016	11	671
CGCGGTGCAAACA	0.027	0.002	0.026	55	2030
CCTGTTATTAGCA	0.03	0.001	0.031	57	1792
GGCAGTATAGAGA	0.054	0.003	0.057	94	1555
ACTAGTACTGGGG	0.029	0.007	0.039	31	768

GCCGTTATTGAGG	0.049	0.004		0.044	45	985
CGCGTTGCTTATG	0.028	0.002		0.027	24	875
AGTAGCGTAGGCG	0.024	0.004		0.02	15	737
ACCATTATTAGTA	0.021	0.003		0.023	90	3863
AGCGTTGCAAGTA	0.021	0.003		0.016	29	1798
ACTGGCGTTAACA	0.024	0.003		0.029	43	1457
CCTAGTGTAGATG	0.025	0.003		0.024	38	1578
ACCATTGTATGCG	0.029	0.004		0.031	67	2066
AGCAGTACAGATA	0.021	0.004		0.024	82	3347
ACCGGCATATACG	0.023	0.007		0.014	31	2184
CGCAGCACATAGA	0.026	0.004		0.021	38	1778
GGCGTTGTTTGCG	0.065	0.01		0.065	41	594
AGCATCGTAGGTA	0.023	0.005		0.016	29	1825
AGCAGTGCTTATA	0.021	0.002		0.024	48	1991
CCTAGTGTATGCA	0.029	0.008		0.02	38	1878
CCTGTTGCAAATA	0.027	0.004		0.031	78	2414
GCCGGCACAGGTA	0.103	0.012		0.088	110	1136
GGCATTGCTTGCA	0.053	0.006		0.045	49	1043
AGTATCGTTTGGG	0.032	0.014		0.022	14	617
GCCATTATTAATG	0.054	0.005		0.048	135	2667
ACTGGCGTTAATA	0.033	0.003		0.032	46	1375
CGCATCACATGGA	0.029	0.004		0.03	48	1553
AGCATTGCTGACG	0.02	0.004		0.025	48	1864
CCTAGCATAAACA	0.025	0.004		0.025	99	3823
GGTATTATTAATA	0.103	0.019		0.081	190	2142
ACCGGCGCAAGTG	0.03	0.004		0.027	30	1086
ACCGGTACAGGTA	0.022	0.003		0.025	62	2437
AGTAGTACTGGTG	0.025	0.001		0.026	18	666
GGTATTATTTGCA	0.102	0.014		0.097	112	1045
CGCATCATAAAGA	0.024	0.004		0.02	71	3450
ACCAGTACTGAGG	0.019	0.002		0.016	35	2142
ACTATCACTTGGA	0.022	0.001		0.023	43	1834
CCTAGCACTTGCA	0.028	0.001		0.029	54	1799
AGTGTTACAAGTG	0.026	0.002		0.023	26	1086
AGCATCGCTAACA	0.023	0.004		0.02	61	3012
AGTATTGTAAGCA	0.025	0.002		0.024	47	1950
ACCATTACAGGGA	0.026	0.002		0.025	57	2246
CCTGGTACTAGCA	0.027	0.002		0.026	39	1438
CGCGGTATAAATG	0.03	0.003		0.03	55	1763
ACCGTCGCTAAGG	0.019	0.002		0.017	24	1348

**CCCGGCATAAGTA**	0.024	0.004	0.019	53	2686
**CGTGGCACTAGGG**	0.024	0.004	0.028	12	417
**AGCATCGCTAATA**	0.021	0.004	0.026	78	2880
**AGCATTATTGGGA**	0.022	0.001	0.02	38	1827
**CGCATTGTATGCG**	0.028	0.007	0.02	27	1325
**CCCATCATAAATG**	0.026	0.001	0.026	111	4118
**CCCGGTGTTGGCA**	0.022	0.002	0.019	23	1167
**GCTGGTGTATGCA**	0.408	0.045	0.393	391	605
**CCCAGTATAAGTA**	0.026	0.002	0.026	96	3559
**ACTGTCACAAGCG**	0.028	0.001	0.027	44	1601
**CCTATCACTGACG**	0.042	0.004	0.042	81	1856
**GCCGGCGTATGCG**	0.927	0.017	0.92	1303	114
**AGTGTTACAAGCG**	0.026	0.005	0.033	37	1075
**CCTAGTGTAGGCA**	0.024	0.002	0.023	38	1638
**CCCGGCGCTAACA**	0.021	0.001	0.019	53	2688
**CCCGTCACTTAGA**	0.026	0	0.027	54	1961
**ACTGTTATAGGGG**	0.031	0.005	0.038	30	764
**CCCATCACAGAGG**	0.03	0.003	0.032	63	1888
**GCTAGTATTTGTA**	0.062	0.006	0.061	87	1342
**CGTAGTGCAGAGA**	0.025	0.004	0.02	15	730
**CCCGTTATTGACG**	0.027	0.002	0.023	49	2056
**ACCAGCGCATGTG**	0.024	0.002	0.023	38	1616
**CCCATCGCAAGGG**	0.019	0.001	0.019	27	1361
**CGCGTCACAGGTA**	0.026	0.003	0.025	37	1436
**CCCGTTACAAGTA**	0.025	0.001	0.024	65	2674
**AGTGGTGTTAAGG**	0.021	0.004	0.017	10	565
**CGCATTGTTAATA**	0.026	0.002	0.026	69	2578
**ACCGTTACTGAGA**	0.022	0.003	0.025	68	2605
**CCCGTCACTGGCG**	0.037	0.004	0.035	55	1506
**CGTGTTATTGATA**	0.022	0.002	0.023	32	1343
**ACTAGCATTGATA**	0.024	0.003	0.021	55	2541
**GGCGGTGTAAGCA**	0.079	0.011	0.074	80	997
**CGTATCGTAGAGG**	0.024	0.006	0.017	15	869
**ACTGGCACATGTA**	0.022	0.003	0.019	31	1637
**CCCATCGTTAGTA**	0.025	0.004	0.02	45	2246
**GCCGTTATTTAGG**	0.056	0.001	0.055	59	1018
**AGCATCACATATA**	0.025	0.004	0.022	82	3643
**AGCGGTATATGTG**	0.017	0.007	0.008	9	1151
**GCCAGTACTGGTG**	0.05	0.005	0.056	54	912
**ACTAGTGTAAACG**	0.029	0.003	0.025	65	2584

GCTATTACTTGGA	0.071	0.007		0.063	69	1021
ACCGTTGCAAACA	0.019	0.001		0.02	84	4110
CGTGGCATATACG	0.028	0.005		0.02	13	631
CCTAGTACTTGTA	0.027	0.001		0.025	42	1635
AGCAGTGTATATG	0.023	0.002		0.026	48	1790
GCCAGCGTATGCA	0.057	0.001		0.057	88	1460
ACTGTCACAAATA	0.025	0.005		0.019	73	3718
AGCATTATAGGCA	0.023	0.002		0.026	78	2961
GGTGGCATATATA	0.489	0.059		0.485	725	771
CCCATCACTAACG	0.026	0.001		0.027	97	3502
GCTGTTATAAACG	0.062	0.003		0.062	100	1510
AGCGGTGTTAGTG	0.021	0.003		0.025	22	848
CCTAGCGTTGGTA	0.026	0.003		0.031	39	1219
CGTAGTACTTACA	0.025	0.002		0.023	31	1335
GGTGGCACTTGTG	0.378	0.055		0.369	181	310
GGTAGTATTTATA	0.071	0.004		0.069	91	1235
GGCGTTATAAGCA	0.062	0.003		0.059	107	1692
AGTATCACTGACA	0.02	0.004		0.016	29	1821
AGCATCGTTAATG	0.021	0.003		0.019	44	2225
ACTAGCACTTGTG	0.022	0.005		0.014	20	1387
AGTGTTACAAAGA	0.027	0.003		0.023	45	1871
ACCGGTACTGAGG	0.023	0.005		0.018	31	1653
GGCATCACAAACG	0.087	0.023		0.073	173	2211
CCTGGCGTAAAGG	0.028	0.004		0.034	33	938
ACCAGCGCTGACA	0.02	0		0.02	73	3562
GGCAGTACAGGCA	0.061	0.01		0.054	73	1278
CCTAGTGCTTATG	0.028	0.002		0.027	35	1272
GGCAGCATAGAGA	0.051	0.006		0.044	66	1426
CCCGTTGTAAACG	0.025	0.005		0.022	55	2393
CGCAGCGCAGGGA	0.023	0.007		0.032	30	920
AGCATCATTAAGG	0.022	0.005		0.017	40	2381
GCCATCACATAGA	0.061	0.011		0.046	101	2115
ACTGGTGCTAGTA	0.023	0.004		0.028	39	1359
CGCATCGTAGGGA	0.027	0.006		0.03	35	1118
ACCGGTGTATGCA	0.024	0.004		0.029	50	1683
CCCAGCGTAGATG	0.028	0.001		0.028	56	1935
ACTATTGCATATA	0.023	0.003		0.022	79	3584
GCCGGTACTAAGG	0.067	0.004		0.063	64	945
CCTATCGTATGCG	0.028	0.002		0.026	42	1548
CGCGGCGCTTACG	0.024	0.004		0.03	29	952

GCCAGCATATACG	0.054	0.008	0.059	135	2167
CGCATTATAAGCA	0.023	0.003	0.018	56	3032
ACCGTCGTTGACG	0.02	0.003	0.017	23	1365
CGTAGTGTATGGA	0.023	0.007	0.014	10	681
GGCGGCGCAGAGG	0.17	0.032	0.168	94	464
GGCAGCGCTAGCA	0.055	0.008	0.048	50	983
GGCGTCGCATATA	0.069	0.009	0.067	83	1154
AGCAGTATAGGGG	0.018	0.001	0.016	26	1588
ACTGTTGTTAATA	0.029	0.003	0.029	65	2161
AGCGGTGTAAACG	0.021	0.003	0.02	35	1692
GCTATTGCAGACA	0.046	0.008	0.057	94	1560
CCCAGTGCATACG	0.025	0.004	0.025	54	2105
CCCATTACTGATA	0.023	0.001	0.021	77	3587
CCTATCGCTGGGG	0.029	0.005	0.022	16	724
GGCAGTGTAGGGG	0.048	0.002	0.046	33	683
AGCGTCACATAGG	0.023	0.002	0.021	28	1306
AGCAGCGCTAGTA	0.021	0.001	0.02	31	1521
GGCGTTACATGGA	0.064	0.009	0.07	67	888
GCTGTCACATATA	0.055	0.012	0.042	62	1428
ACCGGCATTTATG	0.024	0.002	0.023	37	1597
ACTAGCATTAGCG	0.025	0.005	0.019	32	1612
CCCAGCATTGAGG	0.031	0.005	0.026	41	1518
CGCGTCACTTATA	0.023	0.004	0.028	52	1781
GGCATCGCTGATA	0.082	0.006	0.074	106	1317
ACCGGTACATGGA	0.019	0.003	0.014	32	2296
CCTGGTGTATATA	0.019	0.001	0.02	34	1685
GGCATTGTAGAGA	0.07	0.004	0.074	104	1309
ACTATCGCATACA	0.024	0.002	0.023	80	3373
CGTAGTATTTGGA	0.022	0.002	0.025	17	675
CCTAGCATTTGGG	0.031	0.003	0.026	21	776
CCTGGTATATGCA	0.028	0.005	0.02	34	1638
CCTAGCACTAGGA	0.026	0.003	0.021	31	1446
ACTATTACTTGTA	0.024	0.003	0.022	62	2742
CGCAGCGTATACA	0.025	0.001	0.025	56	2152
CCCGTTACATGTA	0.024	0.002	0.026	64	2445
CGTGTCACAGAGG	0.026	0.006	0.019	13	658
ACCGGTACATAGA	0.023	0.005	0.02	62	2995
CGCGGTGCTAGCA	0.02	0.006	0.012	14	1108
AGTATCGCTGATG	0.023	0.006	0.03	29	946
CGTGTTACAAACG	0.028	0.008	0.02	32	1569

GCCGGTACTGACG	0.063	0.012	0.072	89	1145
CCTAGCATTGGCA	0.026	0.003	0.026	41	1543
CGCAGTATATAGA	0.021	0.003	0.022	49	2208
CCTATCATTTACA	0.024	0.004	0.022	74	3358
CCTATTATTGGCA	0.022	0.003	0.019	42	2228
CCTAGTATAGGTA	0.028	0.002	0.03	53	1702
ACTATTGCATGGG	0.028	0.003	0.03	39	1262
ACCATTACAGACG	0.023	0	0.023	97	4076
GGTGGCGCAGACA	0.168	0.033	0.143	107	641
ACCGGTGCAGACG	0.026	0.003	0.028	44	1504
AGTAGCGCTGAGA	0.019	0.001	0.02	18	868
GGTATCATATGGA	0.113	0.025	0.093	89	866
ACCAGTATAAACA	0.021	0.001	0.023	161	6975
ACCAGTATTAGGA	0.022	0.001	0.024	64	2618
CGCAGCATTGGTA	0.027	0.004	0.022	31	1389
ACTAGCGTAAGGA	0.033	0.002	0.031	44	1387
GCTAGCATTTATA	0.06	0.007	0.053	90	1624
GCCATCACTAACA	0.052	0.005	0.045	156	3312
GGCGTCGTTTGCA	0.063	0.006	0.056	49	827
GGTGGCATTGGCA	0.454	0.042	0.451	346	421
GCCGGTATTGGGA	0.081	0.002	0.083	69	767
CGTATTGCTGACG	0.025	0.005	0.022	23	1019
CCCGGTGTTAGGA	0.027	0.004	0.029	32	1076
CCTAGTGTTTACG	0.029	0.003	0.025	38	1511
GGTATTGCTTAGG	0.107	0.01	0.094	45	434
AGCGTTACAAAGA	0.021	0.002	0.018	49	2600
CGCATCACAAACA	0.027	0.003	0.025	111	4252
AGCGGTACTAGCA	0.022	0.006	0.013	23	1685
ACTATTACAGGCA	0.02	0.001	0.019	58	3043
CCTATTACTAATA	0.024	0.005	0.023	94	3979
CCCGGCGTTTGCG	0.027	0.007	0.024	24	966
CGCGGCGCTTACA	0.025	0.005	0.031	41	1272
GGCGTCATAAACG	0.07	0.011	0.065	117	1685
CGTGGTGTAAGCA	0.023	0.008	0.013	11	860
AGTATCACAAATA	0.021	0.004	0.023	64	2710
CCCGTTACTTAGA	0.021	0.005	0.02	41	2002
GCTATCACTAGCG	0.057	0.004	0.052	64	1173
CGTGGCGCTGACA	0.024	0.008	0.014	13	910
CGCATCATATGGG	0.036	0.003	0.04	46	1095
ACCGGTGCTAACA	0.02	0.003	0.019	57	2975

GGCGTCACATGGG	0.071	0.017	0.076	50	606
ACCATTACAGATG	0.025	0.002	0.022	89	3989
ACTGGCATATATA	0.032	0.002	0.032	79	2360
AGTATTGTAGGCG	0.026	0.003	0.022	25	1123
GCTATTATATAGG	0.06	0.01	0.047	69	1408
GCCGTTACAAATA	0.057	0.008	0.046	134	2784
GCTGGTGTAAGGG	0.725	0.056	0.756	572	185
AGTAGCATTTGCA	0.02	0.004	0.019	24	1242
GGCGTTGTTTGGA	0.064	0.016	0.058	38	615
ACCATTGCAAACG	0.024	0.003	0.023	89	3727
GGCAGTGTAAGTG	0.053	0.012	0.04	38	911
CGCGTCATAAGGG	0.03	0.002	0.032	28	846
CGCGTCGTTTATA	0.022	0.005	0.021	28	1314
ACTGGCACTTGTG	0.018	0.006	0.016	12	755
ACCAGCACAGGTG	0.024	0.002	0.027	53	1910
GCCGTCGTTGGCG	0.06	0.015	0.04	28	665
GCTATTGCTTATG	0.049	0.003	0.047	50	1021
ACCAGCACTTATG	0.022	0.001	0.021	62	2833
CCCGGTACTTACA	0.024	0.001	0.023	61	2547
GCCAGTACAGGTG	0.055	0.002	0.057	68	1119
CGCATTGTTAGGA	0.021	0.004	0.018	22	1183
AGCAGCATAAGGG	0.021	0.004	0.016	32	2013
CCTAGTACATATA	0.025	0.002	0.021	63	2915
CGCAGTATATATA	0.026	0.003	0.022	69	3000
ACCATTATATAGG	0.021	0.001	0.019	58	2962
CCCGTTGTTAATG	0.024	0	0.024	42	1732
ACTGGCGTAGACA	0.027	0.002	0.027	36	1310
CGCGTCACAAGTA	0.027	0.003	0.024	48	1979
CGTAGTATTTAGA	0.026	0.005	0.021	24	1101
GCCGGTGTAAACA	0.183	0.029	0.171	370	1797
CCTATCGTAGGGA	0.034	0.002	0.031	36	1138
GGCGGTGCTGGCA	0.062	0.008	0.051	37	694
GGCAGCATTGATG	0.05	0.013	0.044	51	1112
ACCATCGTTGATG	0.023	0.003	0.02	36	1778
CGTAGCACTGGCG	0.019	0.005	0.013	9	711
CCCGTCACAGATA	0.026	0.004	0.022	67	3038
GCCAGTACAGGGG	0.053	0.01	0.06	47	730
ACCGGCGTTTATG	0.024	0.012	0.013	9	681
CCCGGCGTTTATG	0.02	0.006	0.015	18	1167
CCCATTATAGGGG	0.04	0.006	0.033	48	1392

ACTGGTGTTAACA	0.025	0.003	0.022	43	1922
CGTGTTATAAGGG	0.023	0.003	0.026	20	746
GGCATTGTAGACG	0.073	0.008	0.065	87	1255
GCCAGTGTAGAGA	0.046	0.007	0.052	70	1264
GCCGGTGCTGGTG	0.096	0.011	0.081	38	430
GCCAGTGTATGGA	0.048	0.007	0.049	53	1018
ACCGTTATTGACG	0.028	0.005	0.027	58	2121
GCTGTCGTTTACA	0.06	0.009	0.06	61	950
AGCATTGTTAGCA	0.019	0.002	0.021	51	2323
GCCGGCGCTGGCA	0.48	0.054	0.5	532	532
ACTATTGCAGACA	0.027	0.002	0.028	97	3340
ACCGTCGCTGGGG	0.019	0.005	0.023	19	812
CCCGTCGCTTACA	0.024	0.001	0.023	61	2565
ACCATCATAGACA	0.025	0.003	0.029	159	5355
CCCATCACAGGGG	0.04	0.005	0.038	55	1401
AGTAGTGCAAGGA	0.022	0.001	0.022	22	977
ACCGTTATAGAGG	0.026	0.003	0.022	37	1675
ACTAGCATTTACA	0.027	0.003	0.031	89	2806
ACCAGTACAAAGA	0.024	0.002	0.024	133	5471
AGTATCGTTAGCA	0.022	0.002	0.023	34	1416
CCCGTTACATGGG	0.026	0.004	0.02	24	1180
GCTATTATTGGGA	0.061	0.008	0.05	56	1068
AGCGTTGCTAACG	0.022	0.003	0.018	29	1595
AGTGGCGTTGAGA	0.019	0.008	0.009	5	540
ACCAGCATAAAGA	0.024	0.003	0.02	93	4522
ACCGGCACATACG	0.019	0.001	0.019	48	2490
ACTGGTATTAACA	0.027	0.001	0.028	79	2702
CCCGGCATATAGG	0.035	0.002	0.033	45	1337
GCCAGCATTGGTA	0.056	0.006	0.048	81	1605
GGTGGCGTATGTA	0.394	0.049	0.382	258	418
ACCAGCATATGGG	0.023	0.006	0.014	19	1327
CGTATTATAGAGA	0.027	0.004	0.022	41	1811
GCTATTGCTGACG	0.053	0.004	0.047	53	1070
CGTGGTGTAGAGG	0.022	0.004	0.027	11	399
ACTATCGCAAAGA	0.03	0.004	0.028	78	2748
AGTAGCATATGCA	0.026	0.002	0.025	42	1618
AGTATTGCTGATG	0.027	0.006	0.028	30	1051
CCCATCGCAGACG	0.023	0.001	0.022	53	2370
AGCAGCGTATGCA	0.024	0.003	0.025	47	1846
ACCGGCATATATA	0.026	0.002	0.023	76	3234

GCTGTCATTGGCG	0.058	0.01	0.044	36	787
GGTATTGTATACA	0.107	0.014	0.106	159	1345
AGTAGTATTTGGA	0.024	0.007	0.015	17	1106
CCCATTATTAATA	0.026	0.001	0.024	130	5244
ACTAGCATAAATA	0.027	0.003	0.023	109	4554
ACTATTATAAGCG	0.035	0.007	0.027	78	2776
GGTGTTGCATACA	0.076	0.013	0.089	87	888
GGCATTGCAAGGG	0.065	0.016	0.046	36	754
CGTGGCATTGGGG	0.026	0.006	0.018	5	278
CCTGTTACTTGGA	0.022	0.004	0.02	22	1067
GCCGTTACTTGCG	0.057	0.009	0.048	44	876
GGCATCATAGAGA	0.077	0.016	0.058	112	1816
CGTATTATTAAGG	0.036	0.006	0.028	44	1538
ACTAGCACTAGGG	0.024	0.001	0.025	25	969
ACCGTTGCAGAGA	0.022	0.004	0.022	45	1956
CCCGGTGCAAAGA	0.024	0.002	0.022	45	2014
ACCGTCGTAGACG	0.025	0.007	0.018	27	1471
AGCAGTATTGAGG	0.017	0.002	0.018	28	1536
CGCATTGCAAAGA	0.026	0.001	0.025	55	2155
GGCAGTGTTAACG	0.048	0.01	0.037	47	1223
CGCGGTGTAGACG	0.019	0.005	0.025	26	995
CGTGGTGTATGCA	0.022	0.007	0.012	8	679
ACCGTCATTTGTG	0.027	0.003	0.022	31	1356
GGTAGTATTGACA	0.061	0.005	0.054	71	1250
CGCGGCGTAGGCG	0.026	0.005	0.019	13	654
AGCAGCACTGATG	0.02	0.003	0.017	28	1641
CCCATCGCTGATG	0.026	0.001	0.025	49	1878
GCTGTCATATGTA	0.064	0.018	0.038	47	1187
AGCGTCACAGACG	0.024	0.006	0.029	54	1801
CCCATTACATACG	0.027	0.004	0.022	73	3282
ACTGTCACTTATG	0.026	0.005	0.023	35	1463
GGTATTATAAACG	0.106	0.012	0.096	183	1726
AGTAGTGCATGCG	0.019	0.003	0.015	12	771
AGCATCATTGAGA	0.024	0.006	0.019	50	2532
GCCGGCGCATGTG	0.754	0.047	0.737	750	267
GGCATCGCATAGG	0.111	0.02	0.098	97	895
ACCAGTATAGGGA	0.025	0.005	0.018	32	1707
ACTGGTGTTTAGA	0.024	0.003	0.023	25	1072
ACCAGCACTTGTG	0.024	0.004	0.027	55	2002
ACTAGTACAGGCA	0.024	0.004	0.023	63	2643

ACTGGTATAGGCG	0.025	0.005	0.019	19	985
CCTATTATAAACA	0.022	0.002	0.024	142	5687
GGCAGTACAGGCG	0.046	0.008	0.051	46	852
ACCATTGTTGATA	0.023	0.001	0.022	66	2998
ACTGGCATTGGTA	0.029	0.006	0.03	32	1049
GCCGGTGCTAAGA	0.062	0.008	0.052	54	985
CCTGGTGCTGACA	0.025	0.002	0.023	40	1678
CCCAGCGCTGAGG	0.026	0.001	0.027	34	1204
GGTAGCACAAGCA	0.075	0.015	0.066	72	1022
CCTATTGCATATG	0.027	0.004	0.026	51	1907
GGCATTACAAACA	0.056	0.005	0.051	177	3311
CCCAGCATATACA	0.028	0.003	0.031	140	4332
GCTAGCATTGGCG	0.075	0.007	0.068	56	762
AGCGTCATTAGTG	0.023	0.006	0.018	23	1276
AGTAGCGCATGTA	0.029	0.002	0.032	34	1014
CCCAGCATTAATG	0.023	0.005	0.019	53	2800
ACCAGTATTGAGG	0.025	0.003	0.023	45	1908
CGCGTTATTGGGA	0.022	0.007	0.022	21	953
ACCAGTATAGAGG	0.026	0.003	0.022	50	2191
GCCATTACTGGGA	0.054	0.003	0.058	67	1095
CCCATCATTAGCA	0.027	0.002	0.024	93	3753
GGCGGTATTGAGA	0.077	0.01	0.076	73	892
AGTATCATATACG	0.021	0.005	0.014	27	1942
GGCGGCATAGAGA	0.292	0.061	0.284	317	800
GCCAGTGCATAGG	0.043	0.011	0.028	23	786
ACCATCATTTAGG	0.022	0.003	0.02	45	2199
AGTATTGTTTGGA	0.029	0.005	0.023	23	983
GGCATTATAGGGA	0.05	0.012	0.038	56	1415
ACTAGCATATGTG	0.023	0.004	0.018	29	1553
CGTGTTATATATG	0.027	0.005	0.02	24	1150
GGTAGCGTTGAGA	0.081	0.002	0.083	47	519
GCCGGTGCAAGCG	0.104	0.028	0.09	82	825
GGTAGCGTTGACG	0.075	0.021	0.057	32	534
ACCAGTGCAGATG	0.029	0.003	0.025	52	2037
GCTAGCGCTAGCA	0.061	0.014	0.064	62	900
ACCGTTGTAGGCG	0.023	0.002	0.02	34	1647
GGCAGTATAGGTG	0.046	0.005	0.046	49	1016
GGTGTTGTATGTA	0.076	0.01	0.068	45	617
GCCGGTACAGATG	0.069	0.006	0.074	84	1049
ACTATTATAAATG	0.025	0.001	0.025	113	4458

CGCGGCACATAGA	0.022	0.002	0.023	31	1292
AGTGTTGCAAACA	0.022	0.002	0.022	42	1885
GCTATTGCAAGCA	0.051	0.007	0.055	90	1550
ACCGGCGTAAGCA	0.02	0.002	0.021	39	1799
ACCGGTACTTGCA	0.018	0.003	0.014	37	2656
GCTGGTATAAAGG	0.43	0.026	0.409	472	682
CCCGGTGCAGATA	0.026	0.007	0.022	45	1968
ACTATCATTTAGA	0.022	0.002	0.024	61	2466
GGCAGTACAGGTA	0.059	0.003	0.057	75	1234
ACCGGTACAAATG	0.02	0.002	0.021	77	3507
GGCATCACAAGGA	0.068	0.009	0.056	91	1529
ACTGTTATTTATG	0.024	0.004	0.026	43	1608
CGCATTACTTGGA	0.023	0.004	0.018	23	1242
CCCAGTATTGGGG	0.043	0.006	0.042	45	1016
AGTAGCATAAATG	0.02	0.005	0.02	38	1898
GGTATTGTTTATG	0.107	0.011	0.093	74	719
GCCAGCGCTTGCA	0.05	0.008	0.057	64	1054
GGCGTCATTGACA	0.062	0.013	0.059	91	1443
AGCGGTACTGGCG	0.022	0.002	0.019	17	865
CCTATTATATGTA	0.026	0.001	0.027	81	2916
CCCATCACTAGCG	0.026	0.004	0.024	56	2302
ACCGGTATTAACA	0.025	0.003	0.024	95	3871
GCCGTCACTTATG	0.058	0.01	0.045	52	1096
GGCGTCGCAGACG	0.073	0.016	0.051	48	895
AGTAGTATAGATA	0.025	0.004	0.023	57	2396
CCTGTTATTAATG	0.026	0.003	0.022	41	1856
GGCGGCACATAGA	0.139	0.02	0.13	155	1034
AGCGTTGTAAATG	0.021	0.007	0.029	54	1829
CGCAGTATAAATA	0.025	0.002	0.022	87	3925
AGTAGCATAAGTG	0.02	0.002	0.02	26	1304
GCCAGCACAAGTG	0.054	0.004	0.055	83	1437
ACCGTCACATAGG	0.024	0.003	0.02	39	1896
CGCATTACTTATA	0.024	0.003	0.026	65	2422
GGTATTACAAGGA	0.085	0.012	0.082	95	1060
CCTATCATTTAGG	0.025	0.002	0.023	39	1624
CCTAGTATATATA	0.028	0.001	0.026	87	3204
AGTAGTGTTTACA	0.024	0.003	0.027	40	1428
GCTAGCGCTAGTA	0.066	0.013	0.069	57	769
CCTGTTACTGATG	0.024	0.008	0.022	27	1221
GCCAGCGCATATA	0.051	0.007	0.044	74	1617

CCCATCGTTTAGA	0.022	0.004	0.023	47	1963
GCCATCGTTAATG	0.057	0.012	0.045	72	1545
AGTGGCACATAGG	0.026	0.006	0.024	12	488
CGTGTCACATATA	0.023	0.002	0.026	36	1359
CGCATCACTAATG	0.029	0.004	0.025	55	2121
CGTAGCATAAGTG	0.02	0.004	0.021	22	1029
GGCATCGCTGAGA	0.085	0.024	0.082	87	971
CCTGGTGTTAACG	0.02	0.005	0.015	20	1308
GCTAGCATAGGTG	0.065	0.004	0.059	43	680
AGTAGCGCTTGCG	0.017	0.001	0.019	11	556
CGTGTCGCTAAGA	0.026	0.004	0.02	19	915
AGCAGCATAAACG	0.02	0.002	0.021	74	3401
GGCGTTGCATGCG	0.057	0.01	0.053	36	639
CGTATTGTTAGCA	0.024	0.003	0.023	29	1234
CCCATCGTTTACG	0.024	0.003	0.027	59	2140
ACCATCGTTTATA	0.029	0.001	0.03	84	2675
GCTGTCACTGGTA	0.068	0.012	0.084	64	699
AGCGGTATATATG	0.016	0.004	0.012	20	1678
AGCAGTACTAATA	0.023	0.002	0.021	76	3561
GGTAGCATAGGTA	0.071	0.013	0.062	55	834
ACTGGCATAGATG	0.028	0.004	0.023	30	1263
CGTGGCATAGACG	0.022	0.008	0.022	14	617
AGCAGCATTGGCA	0.025	0.004	0.024	48	1914
GGCGGTACTGAGG	0.073	0.007	0.064	38	554
AGCAGCATTAGGA	0.022	0.002	0.019	34	1760
ACCGTTGCAGATA	0.022	0.001	0.022	62	2781
CCCATCGCAAGCA	0.021	0.003	0.018	64	3411
CCTGGCGCAAAGG	0.019	0.002	0.02	18	864
CGCGGCATTTAGA	0.026	0.003	0.021	23	1048
AGCATCGCAGGGA	0.025	0.008	0.019	26	1342
ACCGTTGTATAGA	0.026	0.004	0.022	42	1882
ACCAGTACTAAGG	0.02	0.002	0.022	62	2758
CGCAGTGTATGGA	0.029	0.007	0.023	26	1103
AGTAGTGTTTGGA	0.023	0.002	0.02	17	822
ACTGTCACAAGGG	0.021	0.006	0.025	25	974
GGCAGCATTAATA	0.049	0.009	0.038	90	2294
AGCATTGTTTGTG	0.023	0.002	0.025	31	1205
CCCATCGTAAGTA	0.026	0.003	0.028	83	2914
ACCAGTGCATATA	0.017	0.002	0.02	73	3598
GCCATTGCTTGCA	0.044	0.005	0.044	62	1336

GCTAGTGCAAACG	0.06	0.005	0.053	73	1296
ACTAGTACTTACG	0.022	0	0.022	49	2182
GCCAGTACAAATA	0.054	0.006	0.046	161	3342
GCTATTGCATGTA	0.055	0.002	0.054	69	1207
GGCATCGTATGTG	0.115	0.01	0.102	101	885
CCTAGTATTTGGG	0.026	0.008	0.035	33	911
ACTAGCGTAAATA	0.028	0.004	0.022	65	2914
ACTAGCACAAGCA	0.024	0.003	0.023	79	3319
AGTGTCATAGGGG	0.027	0.003	0.028	19	657
GCTATCGTAAGTA	0.055	0.004	0.054	84	1472
CGCATTGCTTGGA	0.021	0.007	0.011	12	1036
ACTGGCATTGACA	0.029	0.003	0.032	63	1908
GCCAGCACAAGTA	0.048	0.007	0.044	95	2088
GGTGTCGCTAACA	0.083	0.015	0.067	61	850
GGTATCGCTGGTA	0.092	0.01	0.079	48	558
GCCATCGTTAGCA	0.049	0.004	0.053	92	1636
GGCGTCGTTTGGA	0.067	0.009	0.055	33	564
CCCGGCGTTGACG	0.024	0.005	0.02	29	1434
CCCGGTACTGAGG	0.025	0.007	0.028	33	1167
GGCAGTATAAACA	0.055	0.004	0.052	180	3280
AGCGTTATAGGGA	0.023	0.001	0.023	37	1573
GCTATTGTTTGTA	0.058	0.001	0.06	80	1251
ACTGGCGTTAAGG	0.026	0.004	0.021	10	469
CCTAGCATTAGGA	0.023	0.004	0.027	37	1316
ACTGTCGCTTGGA	0.024	0.005	0.031	31	956
AGTAGTATTAACA	0.02	0.002	0.02	53	2586
GGTAGCGTAGAGA	0.073	0.013	0.07	42	555
ACTATCGTTGGCG	0.025	0.002	0.027	45	1595
CCCGTTGCTAGCA	0.023	0.002	0.021	41	1914
AGTATTATAGAGG	0.025	0.006	0.021	32	1482
CGCGTTACTGAGA	0.025	0.004	0.027	37	1348
GCTATTACAAACA	0.06	0.003	0.065	210	3026
CCCGGTACTGGCA	0.026	0.003	0.03	53	1715
ACCGGTACTAAGA	0.019	0.004	0.015	47	3173
AGCATCATAGAGG	0.024	0.003	0.027	56	1998
CCCGGCGTTAATA	0.02	0.003	0.017	41	2347
ACCGTTATTGAGA	0.021	0.004	0.015	34	2192
ACTAGTACTGATA	0.029	0.005	0.022	80	3529
ACTGTTGCAAGTA	0.025	0.002	0.021	42	1918
GCTATCGTAAAGG	0.059	0.007	0.049	58	1120

AGTATTGTTAAGA	0.02	0.003	0.019	34	1790
CGCGGTGTATATG	0.024	0.01	0.02	19	918
GCTGTCATAAACA	0.066	0.006	0.058	137	2231
AGTATTGCAGATA	0.021	0.003	0.022	41	1790
GGTGGTACAGAGG	0.098	0.019	0.084	34	371
ACCAGCACTTACG	0.024	0.002	0.024	73	2960
CCTGTCACTAACA	0.025	0.002	0.027	77	2741
AGTAGCACTTATG	0.023	0.005	0.025	24	952
CGTGTTATAAGCG	0.024	0.008	0.023	26	1125
CGTGGCACATGCG	0.024	0.008	0.034	21	589
AGCAGTGTTGAGG	0.029	0.008	0.022	24	1068
AGCGGTGCTAGCG	0.023	0.001	0.021	19	871
GGCGGTATTAACA	0.078	0.015	0.063	111	1654
GGCAGCATATACA	0.048	0.006	0.055	134	2312
CGCATTATATATA	0.026	0	0.025	93	3641
GCTAGTACAAGGG	0.062	0.002	0.064	56	815
AGCAGCACAGAGG	0.02	0.002	0.022	31	1360
CCCGTCACAAAGA	0.027	0.002	0.029	88	2915
AGTATTATAGGGA	0.022	0.004	0.017	25	1462
CCTATTATAGAGA	0.023	0.003	0.026	76	2814
GGTATCACAAATA	0.102	0.009	0.09	181	1833
AGTATCGCAGATA	0.023	0.002	0.025	39	1509
GGCATCACAGAGA	0.073	0.007	0.067	115	1591
CCTAGTGTTAATA	0.024	0.004	0.02	51	2483
GCCATTGTTAGGA	0.049	0.005	0.048	59	1164
GCCGTCGCATGTG	0.052	0.018	0.029	19	645
GCTGTCACTGGTG	0.05	0.015	0.032	18	544
CCCGTCATAAGGG	0.046	0.004	0.04	57	1377
CCCAGTGCTTACA	0.029	0.004	0.029	76	2502
GCCATCATTTACG	0.054	0.005	0.047	103	2075
CGCAGTATATGGA	0.028	0.006	0.025	40	1548
AGTATCATTAGCA	0.024	0.002	0.021	40	1879
GCTATTGCTGGTG	0.058	0.014	0.05	34	651
GCCATCACTTGGG	0.058	0.01	0.068	65	890
CCCATCGTTGATG	0.025	0.004	0.03	62	1979
ACTGGCGTAGGTA	0.027	0.006	0.036	29	771
AGTGGTATATAGG	0.023	0.005	0.03	21	676
CGTAGCATAGGCG	0.024	0.004	0.022	18	797
GGCGGCGCATAGG	0.169	0.03	0.134	83	537
GCTGGTATTGATA	0.159	0.017	0.164	207	1059

ACCAGCGCTGATG	0.026	0.004	0.022	38	1698
CCCGGCATAGGCA	0.024	0.002	0.025	53	2052
CCTATCACTAACA	0.024	0.001	0.024	107	4280
CGCGGCGTTAACG	0.028	0.008	0.026	27	1023
AGTATCACTAGGA	0.028	0.004	0.032	36	1078
ACCGGTGTTAACG	0.022	0.003	0.021	38	1775
GGTATTACAGAGA	0.083	0.015	0.063	78	1170
ACCATCGCAAGCG	0.024	0.003	0.028	67	2320
ACTGTTATAAGCG	0.026	0.005	0.019	32	1688
GGCGTCATTAATG	0.065	0.01	0.052	70	1289
CGCAGCATAGGCA	0.026	0.002	0.027	52	1851
CGTATCGCTGGCG	0.02	0.003	0.018	17	903
GCTATCATTTGTG	0.062	0.004	0.064	64	931
CGTAGTATAGGCG	0.031	0.004	0.035	27	755
GCCGTCACATATG	0.055	0.007	0.064	99	1448
ACCATTGCATGCA	0.023	0.001	0.022	70	3120
GGCATCATAAACA	0.083	0.012	0.073	270	3435
ACTAGTACTGACG	0.029	0.002	0.028	59	2018
CGTATCACATACA	0.024	0.001	0.023	51	2136
GGCGGTATAAATA	0.081	0.013	0.067	154	2133
CCCATCACTTACA	0.023	0.002	0.02	86	4156
CCTGTCGTAAACG	0.024	0.003	0.025	47	1839
CGCATTGCTGAGA	0.029	0.006	0.021	27	1272
CGCGTTATAAGGA	0.025	0.005	0.018	30	1676
CGTGGCACTGATA	0.02	0.003	0.023	24	998
GGTGTTGTATATA	0.083	0.004	0.078	73	861
GCCATTGTTTGGG	0.043	0.008	0.033	24	699
AGCATTGTTGATG	0.029	0.005	0.033	57	1672
CGCGTCGCAGACA	0.025	0.005	0.018	35	1931
ACTGGCACTAGGA	0.028	0.004	0.033	37	1099
CGTAGCGCAGGGA	0.027	0.004	0.028	15	521
ACTGTTGCATACA	0.024	0.005	0.018	44	2466
CGTGGTACATACG	0.027	0.006	0.03	27	879
ACCGGTGCTGAGA	0.023	0.004	0.028	45	1576
ACTGTCATAAATA	0.026	0.003	0.027	95	3433
ACCATTGTAAAGG	0.026	0.004	0.021	51	2394
ACCGTCGCAGAGG	0.021	0.006	0.027	32	1163
ACTAGTATTTAGA	0.022	0.007	0.013	30	2209
ACCGGCATTGAGA	0.025	0.003	0.029	54	1835
GGTGGTGTAGATG	0.196	0.037	0.183	102	455

AGTGGCGCTAACG	0.024	0.006	0.032	22	666
CCCAGCACAGGGA	0.043	0.009	0.031	58	1806
GGTAGTATAAAGA	0.058	0.006	0.05	83	1585
GCTATTATTGGTG	0.065	0.012	0.049	48	939
CCTAGTACAAATA	0.025	0.002	0.022	88	3953
AGCATCGCAAGCG	0.027	0.011	0.016	30	1795
CCCAGTACAAGTA	0.026	0.004	0.025	87	3432
AGCATTGCTTAGG	0.024	0.001	0.026	32	1214
GGTAGTACTAATG	0.078	0.007	0.073	74	938
CGCATCACTAAGG	0.028	0.003	0.029	49	1639
CCTGTTGCAGGTA	0.021	0.003	0.017	20	1186
CCTATTGCAGGGG	0.024	0.006	0.021	21	999
AGCATTGCAGGGA	0.02	0.001	0.018	27	1441
ACTGTCACAAACG	0.027	0.003	0.023	53	2274
CGCATTATATGGG	0.025	0.005	0.02	24	1150
ACCATCATATGTA	0.02	0.001	0.018	75	4006
GCTATTGCAGATA	0.053	0.006	0.05	78	1481
CGCATCACAGATG	0.026	0.002	0.025	48	1896
GGCAGTACAAATA	0.048	0.003	0.044	122	2620
CGTATCGCAGGTA	0.022	0.002	0.025	24	953
GGTGGTGTTGATG	0.222	0.057	0.177	82	381
GCCGTCGCAGACG	0.052	0.01	0.038	37	942
ACCATTGCTGAGG	0.023	0.004	0.019	33	1726
ACCGGTATTAGGG	0.022	0.004	0.022	24	1060
GGCGGTGCATATA	0.057	0.005	0.057	59	968
AGCGGCACATAGA	0.024	0.002	0.023	33	1406
GCCATCGTTTGTG	0.053	0.002	0.05	51	970
CCTGGCGTTTGTG	0.026	0.003	0.025	15	587
GGCATCATTGGTA	0.071	0.012	0.062	88	1333
CGCGGTACAAGGG	0.024	0.004	0.024	20	829
AGTGGCATATACA	0.03	0.007	0.02	22	1080
GCCGGTACAAGCA	0.053	0.002	0.05	91	1713
GGTGGTGCAGATG	0.105	0.022	0.1	47	425
GCTATCATAGGGG	0.084	0.004	0.09	55	556
AGCAGTACTAATG	0.022	0.004	0.023	55	2343
GCCAGTGTTTATG	0.047	0.011	0.046	53	1091
CCCATTACATATG	0.028	0.002	0.025	75	2870
GGTGGTACTGAGA	0.082	0.01	0.073	46	587
CCCGTCATATGCA	0.029	0.007	0.026	71	2616
GCTAGTGCTGATA	0.05	0.004	0.047	54	1103

CCCATCACAAAGG	0.027	0.003	0.029	82	2757
GCCAGTATAAGGG	0.062	0.008	0.053	69	1226
GCCATTATATATA	0.057	0.003	0.054	216	3807
GCTAGCACATGCG	0.063	0.002	0.064	59	859
CCCATTATAAGCG	0.025	0.004	0.019	62	3168
GCTAGCACAAGTG	0.068	0.008	0.057	60	998
CCTGGTGCTTACA	0.023	0.003	0.019	29	1494
ACTGTCGTAAGTG	0.024	0.005	0.021	20	921
AGCAGCGCTGAGG	0.018	0.006	0.027	25	909
ACCAGTGTAAATA	0.021	0.001	0.022	93	4180
CCTAGCATAAGCA	0.026	0.003	0.022	58	2534
CCCATCACTGGGG	0.04	0.01	0.028	32	1123
ACCAGCGCTAGGA	0.017	0.003	0.021	44	2031
CGTGTTATAAATA	0.025	0.005	0.026	64	2402
ACCGTTGTAGGGG	0.03	0.003	0.029	27	892
CCCGGTGCATGTG	0.028	0.008	0.038	38	955
CGCGTCATTAGCG	0.023	0.001	0.024	28	1116
GGTATCACAGAGA	0.096	0.016	0.088	92	951
ACTGGCATTGGTG	0.028	0.005	0.025	19	730
AGCAGCGCAAGTG	0.019	0.001	0.018	25	1379
CGCGTTATATACA	0.024	0.001	0.023	67	2817
AGCGGCACAGAGG	0.029	0.004	0.03	23	742
AGCATCGCTTGCA	0.022	0.002	0.024	46	1856
CGTAGCGTATAGA	0.024	0.006	0.016	16	961
ACTAGTGCTGAGA	0.023	0.008	0.025	48	1900
CGTATTACTGAGA	0.026	0.005	0.027	38	1355
GGTAGCACTAAGA	0.06	0.006	0.053	50	896
ACTATTGTTTGGA	0.024	0.004	0.02	28	1403
GGCAGTGTTGACA	0.058	0.012	0.042	58	1320
AGTATCGTTTAGG	0.023	0.005	0.029	26	862
CCCATCGTATATA	0.023	0.002	0.022	72	3268
ACTATTGCAAAGA	0.024	0.002	0.021	73	3449
CGCAGTATAAACA	0.026	0.003	0.021	90	4147
GCTGGCACATATG	0.467	0.036	0.455	527	630
GGCGGCACAGGCA	0.129	0.028	0.106	122	1034
GCTAGCGTAGGCA	0.057	0.011	0.053	49	882
CGCGTTGTAAATG	0.03	0.003	0.031	48	1481
AGCATTATTAATA	0.021	0.003	0.024	126	5201
GGTATCGTATACG	0.156	0.017	0.135	127	817
GCTGGCATTGACA	0.623	0.054	0.642	1155	643

GGCAGCGTTTACA	0.06	0.011	0.049	72	1401
GCTAGTACTGATA	0.063	0.008	0.06	90	1409
GCCGTTGTATGGG	0.057	0.01	0.042	26	589
GGCGTTGCTGGCG	0.053	0.005	0.052	38	697
ACCGTCGTAGGGA	0.024	0.004	0.019	20	1050
GCCGTCACTTACA	0.056	0.006	0.049	98	1916
GGCAGTATTAGCA	0.05	0.007	0.053	88	1585
AGCAGCGTTGATG	0.025	0.006	0.023	31	1342
ACCATTATAAGCA	0.025	0.001	0.025	130	5044
ACCGGTGTTTGGG	0.021	0.005	0.015	11	708
AGCGTCACTTACG	0.02	0.005	0.018	28	1529
AGTATTATAGATA	0.025	0.004	0.03	95	3057
ACCATCACAAATG	0.025	0.003	0.021	109	4962
ACCATCGTAGAGA	0.022	0.001	0.02	47	2266
AGTGTTACTAATA	0.027	0.006	0.03	61	1966
ACCATCATAGATA	0.022	0.002	0.025	124	4855
GGCGTTGTAAATA	0.069	0.016	0.053	94	1696
CGCATCACAAAGA	0.027	0.007	0.021	60	2752
CCTGGCACTAACG	0.026	0.004	0.02	29	1400
AGTGTTGTTGACG	0.023	0.005	0.016	14	885
GCTATCGCTGACA	0.048	0.003	0.05	64	1224
AGTGGTGTAGAGG	0.027	0.006	0.02	11	530
AGTAGTGCAGAGA	0.028	0.004	0.026	26	991
AGCGGTGCAGGGG	0.027	0.013	0.015	8	518
AGTAGCACTTGTA	0.026	0.002	0.029	32	1086
AGTGTTGTTTAGG	0.032	0.01	0.027	16	568
ACTAGTACAGGGA	0.023	0.001	0.024	37	1491
AGCATTGTAGAGA	0.021	0.003	0.017	38	2137
CCTAGCGCAGAGA	0.021	0.007	0.012	20	1630
CGCGTCGTAAGGG	0.025	0.004	0.027	24	880
AGTAGTATTAATG	0.021	0.002	0.021	40	1854
ACTATCATAGACG	0.032	0.007	0.025	66	2593
AGCAGCACATACA	0.022	0.003	0.022	76	3329
CGCAGCATATGGG	0.037	0.016	0.024	21	845
CCTAGTACTTACA	0.024	0.004	0.026	69	2607
CCCATTATATGTA	0.022	0.002	0.022	77	3465
AGTATCACTTGGG	0.018	0.003	0.014	8	578
AGCAGCACAGGGG	0.02	0	0.021	27	1285
AGCATCACTAATG	0.021	0.003	0.02	58	2809
CGCAGCGTTTGGG	0.026	0.011	0.017	10	581

CCCGGCGTTTATA	0.02	0.004	0.023	43	1854
CGCGGTACTTAGA	0.027	0.002	0.024	28	1136
GGCATCACAGGTA	0.063	0.007	0.053	85	1508
ACCGGCATAGGTA	0.028	0.003	0.028	55	1918
GCTGTTACTTACA	0.061	0.003	0.065	93	1339
CCCATCGCAGGGA	0.028	0.004	0.025	40	1578
CCCGGCATTGGTG	0.032	0.009	0.028	29	995
AGTGTCACTGACG	0.021	0.001	0.023	22	946
ACTAGCGCAGAGA	0.024	0.001	0.023	41	1733
AGCAGCACTAGGG	0.019	0.007	0.01	11	1061
GCTATCGCATGCA	0.056	0.008	0.047	57	1159
ACCGTCATTGACA	0.021	0.002	0.023	78	3338
GCCAGTATTAGGG	0.051	0.01	0.045	48	1008
CGCGGCACAAGGA	0.024	0.003	0.025	31	1231
CGTGGTGCTGGGG	0.023	0.017	0.046	13	267
ACCAGTGCTAGTA	0.022	0.001	0.023	57	2448
AGCATCACTAGCA	0.019	0.003	0.016	45	2824
GCCGTTGTTAGCG	0.044	0.01	0.044	40	875
CCCAGTACTTATG	0.025	0.003	0.02	43	2063
GCCATCACAAAGA	0.05	0.003	0.05	140	2688
AGTAGTGCAAACG	0.024	0.003	0.02	28	1393
CCTGTTACATAGG	0.026	0.003	0.029	34	1142
ACTGTTGCATACG	0.026	0.001	0.025	39	1537
GGCATCGTAAGCG	0.11	0.022	0.085	109	1173
CGCATTGCTTATG	0.028	0.001	0.027	33	1173
AGCGGTACAGACA	0.022	0.003	0.021	47	2228
GCCATCATTTGCG	0.058	0.002	0.056	86	1446
ACTGTCATTTGCG	0.028	0.006	0.02	22	1079
GGTGTCGTATACG	0.113	0.02	0.106	65	550
GGCGGTACAGATA	0.063	0.004	0.06	81	1277
CCCAGTATTTGTA	0.024	0	0.025	58	2290
GCCATCATTGGTA	0.054	0.006	0.059	109	1751
ACCGGTACTTATA	0.025	0.002	0.023	79	3296
ACCATCGCTAACA	0.022	0.001	0.022	111	4964
ACTGGTGCTTAGA	0.02	0.003	0.016	19	1135
AGTGGCGCAAATG	0.022	0.004	0.017	14	817
GCTATTACATGTA	0.062	0.005	0.055	100	1708
ACCATCATTTACA	0.024	0.002	0.025	120	4759
GCTGTTGCTAAGG	0.054	0.009	0.046	28	577
CGCGGCACTTAGA	0.025	0.003	0.021	24	1123

ACTATCGTTAAGG	0.029	0.002	0.027	43	1546
ACTAGCGTAAACA	0.024	0.003	0.022	67	2911
GCTGTTGCAAATA	0.06	0.004	0.056	85	1440
GCTGTTATTTGCA	0.074	0.013	0.056	59	987
GCTATCGTATATA	0.055	0.006	0.049	87	1684
CCTGGTACATGTA	0.025	0.002	0.024	37	1503
AGTATCACATGCG	0.026	0.001	0.024	26	1068
CCCAGTACTTACG	0.026	0.002	0.025	62	2417
AGTAGCATAAGCA	0.026	0.002	0.029	57	1940
ACCATCGTAAACG	0.024	0.001	0.022	73	3257
GGTATTACAAGCA	0.082	0.007	0.073	111	1410
GCCGGCATTGGTA	0.394	0.029	0.394	482	741
CCCGTTATAAGGA	0.028	0.004	0.031	70	2180
GCTGTCATATGCA	0.058	0.011	0.042	54	1221
ACCAGTACAAGCG	0.022	0.003	0.018	60	3204
CCCAGTACAAGGA	0.026	0.004	0.024	61	2512
AGCAGTGTTGACA	0.023	0.003	0.02	44	2143
GCTATTGTATGCG	0.068	0.013	0.084	89	970
ACCATCGCAAAGG	0.023	0.004	0.024	55	2237
CGCGGCACAGACG	0.021	0.002	0.018	24	1304
CCTGTCATTGATA	0.032	0.001	0.031	63	1977
ACCGTTGTTGGTA	0.027	0.004	0.023	32	1346
GCTATTGCTAGGA	0.045	0.003	0.049	41	796
ACCAGTACTAACG	0.024	0.003	0.027	113	4031
CGTATTACAAGCA	0.026	0.002	0.024	51	2065
AGCATTATTAGCA	0.02	0.003	0.017	57	3300
GGTAGCATAAGCG	0.065	0.004	0.065	55	787
GGCAGCACTAGCA	0.051	0.012	0.04	60	1428
GGTATTGTTTACA	0.098	0.003	0.1	121	1087
GCTAGTACATAGG	0.057	0.005	0.052	50	906
AGCGTCGCATAGA	0.025	0.001	0.024	35	1434
GGTGGCGTTGATG	0.666	0.041	0.663	506	257
AGTGGCGCAAGTA	0.02	0.007	0.011	10	888
GCTAGCACAGGCA	0.071	0.014	0.061	72	1104
AGCGGCGTTGGGG	0.023	0.004	0.021	9	418
GGCAGCGTAGGCA	0.052	0.005	0.047	50	1006
ACTGGTATATATA	0.028	0.002	0.031	80	2520
CGCAGCACTAACG	0.028	0.004	0.023	43	1799
GCCATCATATATG	0.055	0.001	0.054	128	2232
ACTGGTACAAGGA	0.024	0.003	0.023	42	1752

GGCAGTACTTAGA	0.044	0.004	0.039	52	1281
ACCAGCGCATGCA	0.021	0.004	0.023	77	3224
GGCGGTATAAGGA	0.079	0.021	0.072	85	1091
AGCGTCGTAGATA	0.025	0.006	0.032	62	1866
AGTAGCATTAATA	0.02	0.001	0.02	51	2481
GCCGTTGCATGTA	0.047	0.003	0.046	52	1070
CCCGGTACAAAGG	0.03	0.001	0.029	54	1791
CCCGTTACTGATA	0.026	0.004	0.026	69	2622
ACTAGCATTAACG	0.03	0.003	0.027	63	2246
GCCGGTATATACG	0.153	0.024	0.135	224	1430
ACTGTTATAGGCA	0.027	0.001	0.027	52	1847
GGTAGCATAAGTA	0.077	0.011	0.061	72	1107
ACTATTATTAACG	0.03	0.003	0.028	97	3423
ACCGGTGTTAACA	0.025	0.001	0.027	67	2453
GGTATCACATATA	0.108	0.021	0.095	143	1365
GCTGGTATTTACA	0.164	0.02	0.145	191	1122
CCCGTCGCTTACG	0.023	0.008	0.017	27	1531
GGTGTCGCAGATG	0.096	0.027	0.115	55	425
ACCAGTGTATGCG	0.029	0.005	0.027	47	1681
ACTATCACTTAGA	0.025	0.001	0.025	63	2463
AGTATTGCATAGA	0.019	0.004	0.017	24	1405
GCTGTTATAGATA	0.068	0.004	0.063	105	1562
CGTGGCATAAGCA	0.028	0.003	0.03	34	1101
ACTATCACTTGTA	0.024	0.002	0.021	56	2577
GGCAGTGCTGAGG	0.044	0.01	0.03	19	604
GGTGGTGCTAATA	0.058	0.012	0.046	36	753
GCCAGTATAAAGG	0.056	0.001	0.057	117	1953
AGCAGCGTAAACA	0.02	0.003	0.024	87	3531
GGTATTGCAAGCG	0.079	0.019	0.052	43	780
GCTATTACTTATG	0.063	0.004	0.059	80	1286
ACTGGTACATACG	0.028	0.004	0.023	41	1725
AGCATTATAAGTG	0.024	0.002	0.022	58	2631
CGTAGCACTTGGA	0.019	0.003	0.015	11	707
GGCGTTGTAAAGG	0.076	0	0.075	69	845
CGCGGTACTAAGA	0.027	0.004	0.023	34	1470
GCTATTGCATGTG	0.043	0.003	0.04	36	862
CCCATCGTTTGTA	0.022	0.003	0.018	36	1985
GGCATTATAAGGA	0.054	0.005	0.047	90	1816
GCTATCACATATA	0.061	0.006	0.052	114	2059
GGCAGTGCTAGTA	0.053	0.006	0.053	51	917

GCTGTTATTTGTA	0.069	0.004	0.065	68	977
CCTGGTGCTTGCG	0.028	0.003	0.024	17	678
ACTGGCATAAATA	0.025	0.002	0.022	69	3042
CCTATCGCTAACG	0.029	0.003	0.024	49	1978
ACCGTTATAGATA	0.027	0.003	0.024	83	3409
GCCGTCGTTGATA	0.056	0.009	0.062	70	1062
AGCGTCATAGGCA	0.017	0.001	0.015	30	1943
GCTATCATTAGCG	0.079	0.019	0.057	70	1166
AGTAGTGTTAACG	0.02	0.005	0.019	25	1275
CGCGTCACTGACA	0.024	0.001	0.022	46	2070
ACTAGTGTTGACA	0.028	0.003	0.024	60	2404
ACTAGTATTGACA	0.027	0.004	0.021	64	3028
AGCGGTGTAGGTA	0.021	0.005	0.023	26	1089
CGTATTACAGGGG	0.03	0.008	0.023	17	730
GCCATCGTAAACG	0.055	0.002	0.059	123	1978
ACCGTTGCAAACG	0.021	0.001	0.02	56	2726
AGTATTGTTAATG	0.027	0.001	0.027	45	1646
AGCGTTATATACG	0.021	0.005	0.017	36	2118
CCTATCGTATGGA	0.021	0.001	0.021	31	1443
GCTATTACAGACG	0.058	0.007	0.048	79	1559
GCTGTCATTGATA	0.062	0.01	0.049	69	1332
GCCATCACAAGTA	0.054	0.005	0.06	154	2409
CCTGTTGTTGATA	0.026	0.004	0.021	35	1669
ACCAGTATAAGCG	0.024	0.003	0.02	61	3044
AGCAGTATAAACG	0.022	0.002	0.024	85	3508
AGCGTTATAGATG	0.02	0.004	0.019	35	1838
CCTATCATAGACA	0.025	0.005	0.021	85	3976
GCCATCGTATGTG	0.051	0.004	0.051	61	1142
AGCAGCATTAGTG	0.022	0.004	0.022	36	1577
GGCATCGCTAGGA	0.079	0.009	0.073	67	856
GCCATCGCAGACG	0.054	0.015	0.035	49	1360
ACTAGTACTTACA	0.025	0.002	0.023	81	3378
GGTATCATAGGCA	0.126	0.01	0.112	121	961
AGCATTGCAGATA	0.024	0.001	0.025	66	2557
CCTAGTGTAGACA	0.025	0.001	0.024	59	2352
ACTATTGTTGACG	0.026	0.006	0.031	61	1938
GGTATCGCTAAGA	0.104	0.018	0.091	88	879
ACCAGCATTGGCA	0.023	0.006	0.019	50	2586
CCTAGTACAGATG	0.031	0.002	0.029	51	1706
GCCGTTATAGGTA	0.051	0.006	0.042	67	1510

GCTATCATAGATG	0.064	0.013	0.05	80	1535
CCTATCGTAAGTG	0.029	0.001	0.03	49	1569
AGTGTTGTATACG	0.019	0.006	0.025	27	1045
ACTAGTGCTTGGG	0.026	0.003	0.023	20	850
AGTGTTATTGAGA	0.025	0.004	0.021	28	1312
GCTAGCACTAAGG	0.059	0.015	0.038	34	868
GGTATCATTGAGG	0.132	0.016	0.121	79	574
CGCATTATTGATG	0.021	0.004	0.015	28	1827
ACTGTTGCTAGTG	0.027	0.008	0.016	17	1022
AGCAGTACTAACA	0.02	0.002	0.019	75	3913
CGTATTACAAGGA	0.026	0.006	0.026	37	1386
CGCGGTGTTAGCA	0.024	0.009	0.014	15	1082
ACCATTGTTGGCA	0.022	0.001	0.024	50	2075
GCTATTATAGAGG	0.06	0.008	0.051	66	1220
CGCGTTGTTTGCG	0.02	0.005	0.013	10	773
CGCAGTGTTTACA	0.024	0.005	0.027	47	1663
GCTATTACTAGTG	0.061	0.006	0.067	76	1054
CCCGTCACAAGCG	0.027	0.005	0.024	54	2158
AGCGTCGCAGATG	0.024	0.008	0.033	43	1252
GGCATCGTTTACA	0.107	0.024	0.084	145	1571
CCCGGTATAGATG	0.022	0.003	0.022	40	1809
AGTATTGTTTATG	0.016	0.001	0.016	20	1227
ACTGTCGCAGACG	0.026	0.011	0.024	35	1420
CGCATCATAAGTG	0.026	0.001	0.026	54	2060
AGCGGTACATGGG	0.021	0.001	0.021	16	743
ACTATCGCAGGGG	0.026	0.004	0.022	19	857
CCCATCGTTGGCA	0.024	0.002	0.021	43	2000
ACCATTACATGGA	0.021	0.003	0.019	66	3335
ACTAGTATTTGGG	0.032	0.01	0.018	20	1119
AGTAGTGCTGAGA	0.022	0.004	0.017	16	919
CGCGGTACTGGCA	0.02	0.005	0.015	19	1244
AGCGTTGCATAGA	0.015	0.006	0.021	32	1474
ACTATCATAGATA	0.029	0.006	0.031	101	3193
GGTGGCACTTGCA	0.181	0.026	0.161	106	553
ACTAGTATTAAGA	0.026	0.004	0.021	60	2859
AGCATTGTTAATA	0.022	0.002	0.022	76	3398
GCTAGCACTTACG	0.059	0.005	0.066	70	993
CGTAGTACATATA	0.028	0.005	0.021	36	1671
AGCGTCACTTACA	0.025	0.004	0.026	65	2398
GCTAGCGTTAACG	0.076	0.008	0.082	77	865

AGTGGTACTGGCG	0.02	0.005	0.02	12	590
CGCGGCATTTGCG	0.03	0.005	0.024	16	640
AGCGTCGCTGGGA	0.022	0.005	0.015	13	870
GGCATCACTAGCG	0.078	0.004	0.076	101	1225
CGCAGCGCTAGGA	0.028	0.006	0.036	35	937
CGTAGTGCTTACA	0.019	0.003	0.016	16	997
CCCATCACTAAGA	0.026	0.001	0.027	92	3356
CCTATCACTGATG	0.032	0.005	0.031	60	1876
AGTATCGTTTGCA	0.023	0.002	0.025	32	1245
GGCGGTGTTGGGA	0.055	0.005	0.057	27	448
ACCGTCGTTGGTA	0.026	0.002	0.028	34	1169
CCCAGCACTAACA	0.024	0.003	0.023	105	4396
CCTATCACATAGG	0.033	0.006	0.036	72	1907
CGTAGCATTAGCA	0.029	0	0.029	36	1227
AGCATCACTAAGA	0.021	0.004	0.023	67	2801
AGCGGCACTGGCG	0.023	0.011	0.013	10	750
AGTAGCGTAAACA	0.02	0.002	0.018	38	2076
CCTATCACAAACA	0.026	0.001	0.025	131	5190
ACTGTTGCTGGCG	0.025	0.003	0.023	23	992
GCTGTTATTTAGG	0.06	0.013	0.042	31	703
GCTAGCATATAGG	0.055	0.009	0.049	53	1025
GCCAGCATATAGA	0.05	0.002	0.051	114	2135
ACTATTGCTGACA	0.023	0.003	0.02	60	2901
CCCAGCATATATG	0.027	0.003	0.023	61	2554
AGCGTCACAAGTA	0.021	0.002	0.022	55	2464
GGCAGTACATATA	0.047	0.008	0.053	116	2069
GGCGTTGTAAGGA	0.057	0.008	0.063	60	898
CCCGTCGTTTATG	0.019	0.008	0.012	17	1392
ACCATTGCATGGA	0.021	0.004	0.02	43	2151
CGTAGCGTATATA	0.025	0.003	0.02	26	1271
CCCATCATTGACA	0.03	0.003	0.029	121	3990
GGCAGTATAGAGG	0.048	0.004	0.043	44	976
GGCGTCATTTGCG	0.071	0.013	0.059	47	747
CCTATTGTAGACA	0.021	0.003	0.017	49	2845
CGCAGCATTTGTG	0.019	0.006	0.016	17	1063
CGTGGCGTAAATA	0.026	0.009	0.015	19	1217
CGTAGTATTTGTG	0.024	0.004	0.029	21	695
ACTAGTATAAGGG	0.028	0.007	0.019	27	1416
ACCGTCACTTGGG	0.021	0.002	0.018	19	1038
CCTAGCGTAAGTG	0.021	0.002	0.018	24	1306

AGCGTCACAAGTG	0.022	0.007	0.031	50	1575
CCTGTTGCTTATA	0.031	0.004	0.036	60	1609
ACTGGCACTTAGA	0.026	0.006	0.025	33	1282
CCTGTTGCATGCA	0.023	0.003	0.02	32	1579
CCTAGTGCAGACG	0.023	0.003	0.024	41	1674
GCCGGCACTAAGG	0.217	0.034	0.223	233	813
AGTAGTGTTTAGG	0.028	0.003	0.03	23	744
ACTAGCGCAAACA	0.02	0.001	0.019	73	3767
GCCGGCACTTGCG	0.202	0.02	0.212	187	695
GGTGTTACATATG	0.084	0.009	0.078	61	721
ACCGTTGTAGACG	0.023	0.005	0.02	37	1803
CGCGGCATTTACG	0.028	0.006	0.023	23	966
CGTGTTACATGCA	0.024	0.004	0.027	33	1171
CCTAGCATAAAGG	0.025	0.005	0.017	31	1765
GGCATTATATAGA	0.061	0.012	0.056	122	2039
AGCGGTATAAGTG	0.027	0.001	0.026	36	1356
AGCGTCGCATGCA	0.021	0.001	0.021	35	1661
CCTGGTATTTGGA	0.019	0.002	0.018	18	969
ACTGTCGTATAGG	0.025	0.003	0.028	25	874
ACCGGCATTAACG	0.023	0.001	0.025	55	2134
GGCATCATATATG	0.082	0.013	0.068	134	1837
AGTATTGTATATA	0.02	0.004	0.015	36	2407
ACTATCGCTGATA	0.025	0.002	0.023	59	2507
GCCAGCATTGAGG	0.052	0.006	0.044	55	1208
CCCATCACTGATA	0.024	0.003	0.024	85	3508
GCTGTTATAAAGG	0.063	0.017	0.045	51	1095
ACTAGTGCAGGTA	0.029	0.003	0.032	60	1821
CGCATCGTAAATA	0.026	0.002	0.026	87	3283
AGTGTTGCTAGGA	0.016	0.004	0.011	8	695
AGCAGTGTTTGGA	0.018	0.003	0.017	19	1097
ACTGTTGCTTGTG	0.03	0.005	0.023	21	908
CCTGTCACTGGTG	0.036	0.009	0.028	24	832
ACCAGTACAGAGG	0.018	0.003	0.019	46	2370
ACCAGCACATGCA	0.02	0	0.02	83	4065
GGTAGCGCATGCG	0.071	0.002	0.069	37	500
GCTGGTATATGCG	0.445	0.048	0.438	396	509
ACCATCATAGGCG	0.025	0.001	0.026	61	2322
AGTGTCACAAGGG	0.034	0.007	0.028	19	666
GCTGGTATATACA	0.152	0.015	0.151	249	1397
CCTGTCATAAACA	0.025	0.004	0.022	79	3484

CCCAGTGCATATG	0.025	0.007	0.016	29	1799
AGTGGTGTAGGCA	0.02	0.006	0.013	10	746
GGCAGTGTTTACA	0.051	0.003	0.049	71	1392
CCTAGCATTGGGA	0.04	0.009	0.027	31	1115
ACCATTGTTTACG	0.03	0.002	0.028	62	2181
CGCGTTATTTGTG	0.027	0.008	0.017	16	910
GCTATCATATAGG	0.063	0.011	0.05	65	1246
GGTAGTATATGCA	0.062	0.004	0.065	72	1040
CGCATCATAGGTA	0.026	0.005	0.021	44	2077
AGTAGTACAGAGA	0.02	0.003	0.025	38	1481
ACCAGCATATAGA	0.025	0.002	0.023	75	3224
ACTAGCGCTAGTG	0.028	0.005	0.022	25	1129
ACCATTACTAGGA	0.024	0.001	0.023	76	3296
GCTATCGCTAACA	0.049	0.007	0.051	90	1692
GCTAGCGTAGGGA	0.054	0.012	0.039	23	560
GGCATTACAAGCA	0.055	0.011	0.04	93	2219
CCTAGCGCATAGA	0.026	0.002	0.028	51	1773
GCTGTTATTTGGG	0.048	0.013	0.03	16	514
CGCATTATAGGCA	0.028	0.003	0.029	66	2239
GCCATCACTTGCG	0.058	0.004	0.063	89	1324
CGCAGCGCTGGCA	0.021	0.001	0.022	27	1198
CCTAGTGCATAGG	0.029	0.009	0.016	19	1157
ACTGGTATAGACG	0.028	0.004	0.023	33	1433
ACTGTCACTAATA	0.024	0.002	0.022	67	3030
ACCGGTGCTTATA	0.019	0.004	0.013	28	2059
GGCATCATTAAGG	0.09	0.006	0.083	119	1311
ACCAGTACATATA	0.02	0.001	0.02	122	5924
GGTATTGTTGGCA	0.1	0.019	0.078	62	729
ACTGGTGCTTAGG	0.028	0.009	0.02	13	630
CCTATTACTAGCG	0.028	0.004	0.03	57	1822
GGCATTATTGAGA	0.059	0.006	0.052	94	1708
CCCAGCGCTGGTA	0.02	0.002	0.022	40	1789
ACCAGCGCTGGTG	0.022	0.001	0.023	28	1213
ACCGTCGCTTACA	0.021	0.001	0.019	52	2618
CGCATCATTTGGG	0.025	0.005	0.024	22	897
CGCGGTACATGTA	0.022	0.005	0.022	32	1412
CCTATCGTTGAGG	0.028	0.007	0.029	36	1191
ACTAGCATTTGCG	0.027	0.006	0.023	30	1252
AGCATCATTGGTG	0.019	0.005	0.013	20	1537
CGTATTATAGAGG	0.024	0.003	0.029	37	1242

CCTGGTACATACA	0.029	0.003	0.033	77	2264
AGCAGTATTTGGA	0.021	0.002	0.024	41	1677
ACCATCGTATGCG	0.024	0.002	0.023	45	1944
ACCAGCGTAAGCA	0.026	0.002	0.024	64	2649
GCTGGTACAAGGA	0.071	0.014	0.067	56	786
ACTGGCGCTAAGA	0.02	0.005	0.016	14	862
GGTATCATATGTA	0.133	0.025	0.106	132	1118
ACTGTCACTTGTG	0.017	0.002	0.017	17	993
AGTGGCACTTGTA	0.021	0.005	0.016	14	847
CCCGGCGCAGACA	0.024	0.003	0.021	53	2513
GGTGGTGTTTATG	0.219	0.023	0.216	109	396
CCTGGTATAGGCA	0.03	0.004	0.025	35	1375
GGCATCGCAGATA	0.084	0.016	0.075	124	1534
ACCAGCGTAAGGA	0.025	0	0.025	46	1810
CCCAGCATAGGTA	0.027	0.005	0.022	49	2181
GCCATTGCAAGGG	0.048	0.009	0.043	42	944
CCCGTCACTAAGA	0.027	0.004	0.024	61	2462
CGCAGCATTAGTG	0.025	0.007	0.035	44	1207
ACTAGTGCATGTA	0.02	0.002	0.021	47	2236
CGCGTCACAAAGG	0.028	0.003	0.031	44	1393
GGTGGTATTGATA	0.136	0.006	0.13	107	716
ACCAGCACAGAGA	0.022	0.002	0.021	77	3585
CGTGGTACTTATA	0.021	0.006	0.015	17	1113
ACCATCGCTGGTG	0.021	0.004	0.026	33	1229
ACCAGCATAAGTG	0.023	0.001	0.021	58	2743
AGTGGCACTAGCA	0.02	0.003	0.018	16	882
AGTATCACAAAGG	0.023	0.003	0.024	35	1404
AGCATCACATGTA	0.019	0.002	0.02	55	2753
GCCGGTATATGTG	0.17	0.014	0.161	176	916
GGCGGTGTTTGGA	0.073	0.017	0.061	34	527
GCTATCGCATGGA	0.055	0.006	0.058	51	822
ACTAGCATAAATG	0.033	0.004	0.027	80	2877
CCTGGCGCAAGGG	0.017	0.006	0.024	16	655
CCTGTTGCTTGTA	0.022	0.005	0.015	18	1188
GCCGGTATTGAGG	0.145	0.012	0.153	133	738
GGTGGTACTGGTA	0.078	0.009	0.077	37	445
GGTAGTGCATACA	0.058	0.005	0.054	58	1013
CCCAGCATAGGCG	0.039	0.004	0.044	85	1847
ACCGGTGTTTACA	0.029	0.002	0.026	52	1969
ACCGGTATTAACG	0.026	0.007	0.02	47	2267

CCTATCGCAGGCA	0.025	0.003	0.024	51	2044
AGTGTTACTGGTG	0.023	0.004	0.018	12	664
AGCGGCATAAATG	0.024	0.006	0.015	15	953
ACTGTTGCTTACA	0.028	0.002	0.027	57	2019
CCCATTACTTGGG	0.028	0.007	0.024	34	1382
CGTAGCATTTGGG	0.027	0.01	0.016	8	507
AGTAGTATATATG	0.02	0.002	0.022	37	1679
ACCGGCACAGGTA	0.025	0	0.026	58	2206
CGCGGTATAGGCA	0.021	0.002	0.019	25	1320
GCTGTCACATACA	0.058	0.002	0.059	98	1561
GCCGTCACTGATG	0.052	0.006	0.061	71	1101
GCCAGTATTGGTG	0.058	0.009	0.053	57	1027
GGTGGTATTAGGA	0.142	0.018	0.137	81	512
CCTATTGTAGATG	0.023	0.002	0.021	42	1927
CCTGGTATTTAGA	0.026	0.002	0.027	34	1242
CCTAGCATATATG	0.024	0.003	0.025	48	1910
ACCAGTGCTGGGA	0.021	0.001	0.021	31	1454
GGCATTATAGGCG	0.058	0.004	0.053	66	1179
ACCAGTATTTGGA	0.024	0.002	0.022	48	2162
ACTGTTGTAGGCA	0.028	0.004	0.029	41	1370
GGCAGTACTGACG	0.049	0.013	0.039	48	1170
GCCATCGCTTATA	0.045	0.004	0.045	76	1615
ACTATTGTAAAGA	0.03	0.004	0.028	91	3143
GCTGGCATTAAGG	0.863	0.019	0.841	1175	222
GGTATTATTGATG	0.12	0.029	0.093	102	994
CCCGGCGTTGACA	0.022	0.002	0.019	42	2165
GGCATCATAAACG	0.085	0.006	0.077	205	2460
GGTATCGTATATG	0.14	0.01	0.125	109	761
CCCGTTATTAATG	0.023	0.001	0.022	51	2307
GCCGTTACTAGGG	0.051	0.014	0.06	46	723
GGTAGTATTGAGG	0.066	0.008	0.064	45	657
AGCGTCATTAGGA	0.024	0.003	0.024	38	1534
ACTATTGCTGAGG	0.026	0.006	0.024	34	1369
ACCATCACAAGGA	0.027	0.002	0.028	93	3188
GCCATCGCTTATG	0.045	0.012	0.058	61	992
CCCATCATATGTG	0.027	0.007	0.02	48	2391
AGCATCGTAGGCA	0.021	0.005	0.015	33	2122
CGTGTTGTTAAGG	0.028	0.006	0.024	15	609
ACCGGTGCTGGGG	0.024	0.007	0.016	11	667
ACCATCATTGAGG	0.025	0.004	0.02	44	2111

AGCAGTGCTGGGG	0.022	0.003	0.021	14	647
GCTATCGCATGTG	0.064	0.008	0.06	44	693
CCTATCACAGATG	0.027	0.003	0.028	66	2266
GGTAGCGCTAAGA	0.057	0.007	0.051	36	665
GCCGGTGCAGACG	0.115	0.015	0.114	109	844
CCCAGTATAGACA	0.023	0.003	0.026	107	4072
ACTAGCATAGGTG	0.026	0.006	0.02	24	1148
GGCAGCGCAGATA	0.049	0.004	0.046	62	1295
ACCGGTATATAGG	0.031	0.004	0.029	45	1498
CGCGGCATAGGTG	0.025	0.005	0.025	18	690
GCCGGTGCTTATG	0.118	0.012	0.103	84	729
CGTGTTATTTGTA	0.032	0.003	0.035	38	1043
GCTGGTGTTTATA	0.423	0.047	0.413	455	648
CCTAGCATAGATG	0.025	0.003	0.026	47	1757
AGTGTTGCAAAGA	0.025	0.005	0.018	23	1279
CCCGTCATAGGGA	0.038	0.005	0.038	58	1454
AGCGTTGTAAAGA	0.022	0.002	0.021	45	2128
ACCATTGCATATA	0.024	0.006	0.017	67	3970
AGCGTCGTAAGTA	0.022	0.004	0.021	41	1911
GGCGTCATTTGGA	0.061	0.008	0.05	40	764
AGTAGCACTGACA	0.023	0.003	0.021	36	1644
CGCGGTGCAAATA	0.025	0.005	0.02	41	1976
ACTGTTATTGGTA	0.031	0.008	0.02	29	1412
ACTAGCACATGGA	0.022	0.001	0.021	39	1776
GCCAGCATAAGGG	0.071	0.003	0.067	71	983
ACTATCGCAAGTG	0.03	0.009	0.031	53	1631
GGCGGTATTTAGG	0.123	0.022	0.108	75	621
CCTAGCGCTTGTG	0.023	0.01	0.016	13	793
GGTGTTATTAGGA	0.084	0.015	0.072	50	649
GGCATTGTAGGTG	0.072	0.025	0.054	46	805
ACTATTGCAGGCG	0.027	0.004	0.026	50	1841
AGTGTCGTATACA	0.019	0.008	0.03	45	1454
CGTATCGTTTGGG	0.029	0.012	0.018	10	534
CGTATTATTGACA	0.023	0.003	0.02	45	2184
AGCAGCATATGGG	0.019	0.002	0.019	28	1443
GCTAGTGTTAACG	0.057	0.005	0.052	66	1196
CCCGGCGTTAGGA	0.018	0.002	0.015	18	1158
CCCATTATTGGGA	0.029	0.004	0.024	43	1719
CCTGTTGTTTGCG	0.03	0.001	0.031	31	969
AGCGGTATATGGA	0.022	0.005	0.018	27	1455

CGTATCATTAACG	0.025	0.004	0.021	40	1884
ACCGGCGTAGATA	0.021	0.003	0.024	41	1671
GCTGGCACAAGTA	0.223	0.016	0.217	281	1012
AGTATTGCAAAGA	0.02	0.005	0.014	26	1891
CCCGTCACTTGGA	0.027	0.004	0.024	36	1457
AGCGTTGTTAGGA	0.021	0.003	0.017	19	1083
AGCGTCGTAAACG	0.02	0.002	0.022	44	2002
CGCGTTATATATA	0.026	0.003	0.027	71	2540
CGCGTCGCAGAGA	0.023	0.005	0.028	33	1141
CCTAGTGTATGTG	0.025	0.005	0.031	39	1210
GGCAGTACTTGGG	0.057	0.004	0.062	38	572
AGTGTCGTATATG	0.027	0.007	0.019	17	886
GCTAGCATAAAGG	0.063	0.004	0.067	91	1276
ACTAGCGTTTATG	0.032	0.011	0.019	22	1118
CCCAGTGTATACG	0.022	0.004	0.017	38	2170
AGCATCGCATAGA	0.029	0.002	0.027	54	1938
CGTATTGCAGGGA	0.015	0.004	0.01	8	831
GCCGTCACTTATA	0.06	0.003	0.057	100	1662
CGTATTATTAGCA	0.031	0.006	0.022	42	1868
ACCATTACTAATA	0.021	0	0.021	143	6702
ACTGGTATAGATA	0.027	0.005	0.02	48	2347
CCCGTTACTGAGA	0.025	0.002	0.028	51	1747
CGTGGCGCTTATG	0.019	0.006	0.013	7	530
GGTATTATATATA	0.127	0.012	0.115	250	1932
AGTAGCACATACA	0.027	0.002	0.025	55	2186
AGCGTCATAAACG	0.023	0.002	0.024	64	2577
CGTGGCGCAAACA	0.023	0.003	0.027	36	1293
ACCATTACAAGGA	0.02	0.002	0.023	89	3821
GGTGTTGCATATG	0.083	0.011	0.076	44	533
AGCAGTACAAGCA	0.019	0.007	0.016	51	3078
CCTAGTACTTATG	0.029	0.003	0.025	41	1580
CGCGGCACTTGTA	0.028	0.003	0.033	36	1069
CGTGGCGTATGCA	0.022	0.003	0.017	12	674
GCCGGTATAGATA	0.08	0.002	0.077	157	1887
ACCATCGCTAGCG	0.026	0.002	0.028	55	1886
ACTATCACAAGGA	0.024	0.001	0.023	63	2673
ACTGTCATTGGTG	0.029	0.005	0.027	26	937
GCCGGCACAGACG	0.205	0.023	0.19	277	1180
ACCGTCGCAAAGA	0.024	0.004	0.019	48	2529
GCTGTTGTTAACA	0.055	0.006	0.047	75	1506

CCTATCGCATACA	0.021	0.004	0.018	54	2967
ACCGGCATTGGTG	0.026	0.006	0.018	19	1027
AGCGGTGTTAGGA	0.023	0.006	0.015	14	942
GCTGTCATATGGG	0.064	0.017	0.048	29	579
CCTAGTGCATGGA	0.02	0.003	0.021	28	1329
GGCGTCGCATGGA	0.059	0.013	0.044	31	669
AGCAGTGTAGGCA	0.024	0.005	0.017	30	1690
ACTAGTACATGTG	0.027	0.007	0.017	33	1859
AGCAGCATAGATA	0.019	0.001	0.018	60	3216
ACCGTTACTTGGA	0.02	0.003	0.017	34	1975
ACCATCGCTTGTA	0.021	0.005	0.015	40	2544
CGCGTTACAAAGG	0.026	0.005	0.022	31	1373
GGCGTTATAGACA	0.069	0.012	0.053	103	1832
ACTAGCGTATACG	0.026	0.001	0.025	36	1396
CGCGTCATTAAGA	0.025	0.004	0.023	41	1721
CGCGTCACATGGA	0.027	0.006	0.021	27	1256
GCTAGCGTTTAGA	0.067	0.011	0.082	66	736
GCTGGTGCATGGG	0.301	0.076	0.248	91	276
CCTATCGCAGGCG	0.029	0.001	0.028	37	1281
ACCGTCACTGAGA	0.023	0.001	0.024	62	2553
CGCATTATAGAGA	0.026	0.002	0.023	54	2297
CCTAGCGCAGACA	0.022	0.001	0.021	50	2369
CCTATCGTTAGCG	0.029	0.003	0.026	38	1449
GCTGGCGCTGACA	0.627	0.04	0.59	803	557
GCTGTTACAGAGA	0.063	0.006	0.066	71	999
GCCGTTATTAGTA	0.056	0.004	0.053	91	1634
AGCGGTGTAAGTG	0.016	0.004	0.016	18	1080
AGCGGCACATACG	0.019	0.001	0.021	26	1223
GGCGTCGCAGGGA	0.068	0.019	0.052	33	601
CCTATTACTAGCA	0.027	0.002	0.024	63	2562
CCTGTCACAGGTG	0.027	0.005	0.02	20	960
CCTAGTGTATATA	0.024	0.001	0.022	52	2308
AGTATTATTTATA	0.022	0.004	0.021	63	2972
CGTAGTGCATGGA	0.022	0.005	0.016	10	606
AGTGTCACATATG	0.019	0.001	0.021	22	1025
GCCGGCATAGGCG	0.662	0.042	0.647	818	446
CCCGGTGCTAGGA	0.026	0.001	0.025	30	1150
AGTGTTATAAGTG	0.022	0.003	0.018	23	1250
CCCGTCGCTAGTA	0.024	0.006	0.02	36	1792
CGCATCGTAAATG	0.023	0.005	0.021	49	2267

GGCAGTATTTACA	0.047	0.005	0.046	95	1987
CGCAGCATTTGTA	0.023	0.001	0.024	39	1576
ACCGGTACTAGGG	0.018	0.002	0.019	27	1384
ACCGTCACTTATA	0.022	0.002	0.021	72	3417
CGCGTTGCTAACG	0.021	0.008	0.01	14	1382
AGTGTCGTAAACA	0.023	0.005	0.017	32	1825
AGCAGCACAAATG	0.025	0.001	0.026	70	2667
AGTATCGCAGACA	0.021	0.003	0.019	31	1640
GCCGGCACTAGTA	0.1	0.013	0.097	132	1222
GGCGGCATTAGGA	0.291	0.059	0.25	224	673
CCCAGCGTAAGTG	0.026	0.006	0.031	55	1716
AGTAGTACTAATA	0.026	0.003	0.022	48	2144
GCCATCATAGGCA	0.053	0.008	0.044	105	2274
CCTGGCATAGACG	0.027	0.006	0.019	27	1406
CCTAGCACATAGA	0.025	0.003	0.029	61	2052
ACCAGTACTAGTA	0.022	0.004	0.02	78	3804
GGCAGCACTGGGG	0.057	0.009	0.05	36	679
ACTAGCACTTGCG	0.025	0.004	0.03	43	1406
GGCGGTGCTTAGA	0.054	0.016	0.042	28	633
CCCAGCATTAGGG	0.036	0.001	0.038	49	1247
GCTGTCATAGGCA	0.065	0.01	0.077	86	1033
AGTGGTATAAATG	0.026	0.002	0.026	31	1169
GGCATTGTAAAGG	0.083	0.009	0.074	105	1321
AGCAGCACATGTA	0.025	0.001	0.024	52	2122
GGTGTTACTAATA	0.068	0.012	0.062	79	1188
ACTATTGCAAGGG	0.027	0.006	0.023	37	1575
GGCAGTGCAAACA	0.051	0.005	0.044	86	1867
ACTATTATATGTA	0.027	0.003	0.028	96	3359
GCTAGCACAAGGA	0.058	0.005	0.063	76	1125
CGTATCGTTGGTG	0.032	0.001	0.03	20	640
ACCATCGCAGACA	0.022	0.001	0.023	101	4280
CGTAGCGCTTACG	0.032	0.006	0.025	19	755
ACCATCACATGTG	0.022	0.002	0.021	58	2758
GGCATTGCAGGTG	0.065	0.011	0.069	54	727
ACCGGCGTTAACA	0.022	0.004	0.022	47	2072
CCCATCGTATGGA	0.026	0.002	0.027	49	1777
CCCATCATTGGTA	0.026	0.003	0.023	58	2430
AGCGGTGTTGGCA	0.028	0.004	0.028	30	1052
GCCATTACATGCA	0.06	0.001	0.061	143	2211
CGCGGTACTTAGG	0.026	0.004	0.03	23	742

GGTAGTACAGGGA	0.066	0.015	0.047	28	562
ACTAGCGCAGGCG	0.028	0.003	0.031	37	1148
GCCAGCACAAACA	0.047	0.005	0.051	179	3363
CGCGTCACAAGGG	0.025	0.004	0.028	27	944
CGTGGCGTATATA	0.022	0.005	0.029	23	768
CGCGTTACTTGCG	0.024	0.004	0.02	20	1000
CCCATTGCTAGCG	0.027	0.002	0.025	44	1746
CGCATTACAAAGG	0.025	0.004	0.023	45	1877
GGCATCATAGAGG	0.084	0.005	0.078	111	1316
ACTGGTGTATAGG	0.032	0.013	0.021	18	822
GGCGGCATTTGCA	0.29	0.056	0.254	288	844
ACCGGTATTTGGA	0.021	0.003	0.024	38	1527
CCCGGCGCAAGGA	0.023	0.003	0.019	28	1478
ACTAGTGTAGATA	0.03	0.006	0.026	69	2555
CCCATCATTAGCG	0.026	0.002	0.024	60	2463
ACCGTCACATGTA	0.02	0.001	0.02	64	3119
ACCATTACTTAGA	0.019	0.002	0.021	81	3837
GGCATCATAGGCA	0.072	0.012	0.06	111	1751
GCCATTGCTAAGA	0.043	0.006	0.044	71	1530
ACTATCATAAGTG	0.026	0.001	0.027	64	2320
GCCATCGTATGTA	0.051	0.001	0.049	79	1525
CCTATCGTTAGCA	0.025	0.001	0.023	44	1848
CCCAGTGTTAGCG	0.022	0.005	0.015	23	1502
GCTGTTATAAGCG	0.071	0.006	0.064	71	1036
AGCGGTGCTTACG	0.02	0.007	0.016	16	960
ACCGTTGTTAAGG	0.027	0.002	0.029	41	1372
ACTGGTACTAACG	0.025	0.006	0.017	30	1744
GCTATCACATGCA	0.058	0.005	0.061	101	1552
AGCATTGTTAATG	0.027	0.003	0.032	72	2201
GGTGTCACATACG	0.104	0.01	0.094	74	713
ACTGTCATTAGCA	0.025	0.002	0.025	47	1869
ACTGTTACTGGTG	0.026	0.005	0.021	20	933
CCCGGCACAAGTA	0.026	0.006	0.017	44	2541
CCCGTCACTGATA	0.025	0.003	0.023	58	2517
CGTATTATTTGGA	0.021	0.004	0.023	27	1129
GGTATTACTAAGA	0.076	0.009	0.062	90	1353
AGTATTACTGATA	0.024	0.005	0.023	53	2251
GGTATCGCTAGGA	0.104	0.021	0.074	43	537
GCTGTCGTATGGA	0.062	0.004	0.057	40	664
GGCAGCACATGCA	0.052	0.005	0.059	93	1474

GGCATTGTAAGTG	0.071	0.01	0.067	79	1106
GGTATCATTGATG	0.129	0.016	0.125	115	808
AGCAGCGTAAAGA	0.027	0.003	0.031	77	2384
ACTGTCGTTAACG	0.023	0.006	0.016	21	1325
CCCATCGTTAGTG	0.028	0.004	0.022	37	1609
AGCAGCACTGATA	0.021	0.003	0.019	45	2288
CCCGTCGCTAGGA	0.027	0.005	0.032	48	1463
CGCAGCACTTATG	0.028	0.003	0.025	34	1300
AGTGTCACATGTA	0.022	0.002	0.02	24	1164
GGCAGTACAAATG	0.058	0.012	0.052	98	1804
GGTATTACATACG	0.112	0.035	0.08	101	1168
GGCGGCGTAGGGA	0.134	0.034	0.12	79	577
GCCATTATAGATA	0.053	0.001	0.054	193	3390
CCCGGCACATACA	0.026	0.005	0.025	81	3203
CGCGGCGCTAGCA	0.027	0.008	0.025	28	1102
GCTGTCATATAGG	0.065	0.002	0.065	54	782
AGCATCACAGGGG	0.02	0.003	0.017	25	1474
ACCATCACTTATG	0.022	0.001	0.022	69	3089
AGTGTTATAGAGG	0.025	0.002	0.025	24	936
ACCGGCACAAATA	0.023	0.004	0.018	85	4742
AGCGGTGTATATG	0.017	0.002	0.015	18	1184
ACCAGTGCTAATG	0.023	0.003	0.02	47	2281
GGTGTTGCAGACG	0.08	0.007	0.079	47	550
CCCAGCGTTGACA	0.022	0.003	0.018	52	2770
AGCAGTGTATAGG	0.023	0.001	0.022	29	1288
ACTAGCGTTAATA	0.025	0.002	0.025	59	2332
GCCGTTATTTGCG	0.058	0.01	0.045	47	990
AGTGGCACTAATG	0.021	0.003	0.02	15	735
CGTGGCATAGGCA	0.023	0.006	0.016	12	758
GGCGTCATATGGG	0.057	0.009	0.044	33	713
AGCAGTATAAAGG	0.021	0	0.021	53	2512
AGCAGTATATGTA	0.023	0	0.023	68	2924
CGCGTTATATGCA	0.027	0.003	0.024	47	1872
CGTATCGCTGATG	0.023	0.007	0.021	19	904
AGTGGCACTTATG	0.019	0.003	0.019	12	624
CGCATTGTAGATA	0.023	0.002	0.02	47	2264
GGCAGTACAAGGA	0.05	0.005	0.044	63	1356
CCCAGCGCAAACA	0.024	0.003	0.02	96	4697
CGCGTCGTTGAGA	0.02	0.002	0.023	24	1015
AGTGTCATTAAGA	0.022	0.002	0.025	38	1506

GGTATTGTTAGTA	0.087	0.013	0.078	75	887
ACTGGCACTTACA	0.022	0.002	0.024	49	2013
ACCGGTGTAAGTG	0.037	0.004	0.031	42	1298
ACTAGCACTTACG	0.024	0.002	0.02	40	1924
CCTAGCACTAATG	0.021	0.005	0.015	26	1765
GGCAGTGTAGATG	0.049	0.012	0.047	45	914
AGCAGTACTGGTA	0.022	0.003	0.021	37	1748
GGCGGCGCAGGTA	0.093	0.017	0.073	54	686
CCCATTATATGGG	0.028	0.001	0.028	44	1512
CGTGGTGTAGGTA	0.021	0.009	0.01	7	662
CCCGTTGTAGAGG	0.024	0.001	0.025	30	1150
CGCGGTATAGATA	0.025	0.001	0.024	48	1912
CGCGGTGTAGGTA	0.02	0.004	0.019	18	945
ACCAGTACTAGCG	0.02	0.003	0.016	47	2814
AGTGTTGTATGTA	0.018	0.004	0.013	16	1194
CGCGTTGCATGCG	0.026	0.006	0.018	17	921
CGTATCATAAGCG	0.029	0.001	0.028	42	1481
CCTGTCGCTTATG	0.024	0.006	0.018	18	961
AGTAGTGCATATG	0.021	0.006	0.014	14	989
CGTGGCGTTTACG	0.03	0.004	0.027	14	503
AGCATTGTATAGA	0.019	0.004	0.025	59	2291
CCCGGTGCTGATG	0.027	0.004	0.023	23	980
CGCGTTGTATGTA	0.025	0.005	0.023	31	1304
GCCAGTGCAGGCG	0.04	0.003	0.039	45	1118
ACTGTCACTTACA	0.022	0.002	0.021	52	2426
GGCATTGCATGTG	0.064	0.008	0.068	67	925
CGCATCGCTAACG	0.026	0.001	0.024	46	1851
ACCGGTACTGACA	0.022	0.002	0.02	69	3447
CGCGGCGCAAATG	0.027	0.003	0.029	39	1288
CCTATCACTGGTA	0.025	0.006	0.017	36	2050
AGCATCATTGACA	0.019	0	0.019	71	3718
CCCGTTATTTGCA	0.024	0.001	0.022	47	2088
ACTGTTATAGGGA	0.03	0.006	0.022	26	1172
ACTATCACATATG	0.026	0.004	0.023	67	2898
GGCATCACTTAGG	0.098	0.016	0.091	91	907
GGCGGCACTGGCG	0.244	0.041	0.232	175	579
AGCATTACAAAGG	0.02	0.003	0.016	42	2654
CGTAGCACTTACG	0.028	0.003	0.025	24	945
GCTATCATTTATA	0.065	0.003	0.069	158	2118
GCTGTTGCTGATA	0.05	0.007	0.057	52	858

CGTGGTGCAGGCG	0.027	0.008	0.031	16	498
ACCATTATTTGCG	0.027	0.003	0.023	57	2417
ACTGTCACAGACG	0.023	0	0.023	39	1639
ACCGGTACATAGG	0.025	0.004	0.018	36	1925
AGTGTCGCTTGCG	0.023	0.012	0.017	9	529
ACTGTTGTTAATG	0.035	0.004	0.041	63	1489
AGTATTATATACA	0.019	0.002	0.018	66	3608
GGCATTGCTAAGG	0.069	0.003	0.071	62	817
GGCGGCACTGATA	0.121	0.02	0.105	138	1180
CCTGTCGCAAAGA	0.024	0.006	0.019	35	1821
CCCGTTGCTTGGG	0.026	0.007	0.018	15	823
GGCATCATATGTG	0.086	0.009	0.079	109	1273
GGCGTCGCAAATG	0.077	0.004	0.078	85	1008
ACCATCACTAGTG	0.02	0.002	0.018	47	2572
CCCGGCGTTGGTA	0.026	0.004	0.02	25	1233
CCCATCGCTTACA	0.024	0.003	0.021	72	3315
GGCGTCGCATGTA	0.062	0.006	0.063	67	1001
GCCATTGTTTGTA	0.039	0.007	0.044	61	1314
GGTGGTGCTGGCG	0.079	0.007	0.073	23	294
CCCGTTACTTACG	0.026	0.002	0.024	49	2027
GGTGGTATTTAGG	0.257	0.032	0.242	131	410
CGCGGTGTTGATG	0.018	0.005	0.011	9	805
AGTGGCATTTATA	0.025	0.009	0.012	10	858
CGTATCACAGGCG	0.029	0	0.029	35	1157
CCCGGTGTATGGA	0.024	0.009	0.016	18	1123
ACTATTGCAGGTA	0.026	0.002	0.024	54	2211
GCCAGTACTTGGA	0.058	0.005	0.055	66	1138
CCCGGTATTGGGG	0.037	0.009	0.029	21	709
CGTGTTGTTGACG	0.018	0.004	0.017	13	742
ACCGGCATTGGGA	0.026	0.012	0.016	20	1193
AGTATTACATACG	0.024	0.004	0.019	37	1940
CGCATTACAGAGG	0.031	0.006	0.033	45	1318
GGCATCGTAGATG	0.114	0.022	0.094	125	1199
GCTATTATAGGTG	0.065	0.011	0.053	64	1150
CCTATTATAAATG	0.023	0.004	0.019	75	3836
CGCGTTGCTAACA	0.023	0.003	0.02	37	1848
GCCAGCGCAGGGG	0.047	0.008	0.05	25	479
CGCGTTGTATGCA	0.026	0.005	0.026	35	1307
CGCGTCACTGAGG	0.025	0.005	0.026	22	839
GCTATCGCAAGCA	0.052	0.006	0.061	82	1260

CCTATTATTGATG	0.025	0.003	0.021	48	2221
GGTAGTATAGGGA	0.055	0.004	0.053	44	789
ACTAGTGTATACA	0.026	0.002	0.026	78	2975
AGCAGCACTAGGA	0.024	0.004	0.022	33	1456
ACCAGTGCTGACA	0.021	0.002	0.02	64	3104
CCTGGCATTGGCA	0.031	0.006	0.023	28	1194
CGCATCACTGGGA	0.023	0.004	0.019	24	1238
CGTAGTATAAATG	0.031	0.005	0.025	41	1615
GGCGGTGTATATA	0.067	0.009	0.059	70	1113
GCTATTGTTAGGG	0.08	0.004	0.081	57	649
ACCAGCATAAGGA	0.019	0.004	0.016	39	2452
GCTGGTGCTAGGA	0.108	0.017	0.095	53	506
GCTATCGTTTGTG	0.07	0.01	0.058	44	713
AGTGTTATAAGCG	0.023	0.006	0.025	31	1204
AGTATTGTTAGCA	0.025	0.001	0.026	43	1601
ACTAGCGCAGGGG	0.018	0.011	0.032	23	698
CCTGGCGCATGCA	0.025	0.005	0.027	37	1313
CGCAGTGTTAGCA	0.027	0.001	0.026	35	1313
GCTATTATTGAGA	0.058	0.004	0.062	105	1576
CCCGTCGCAGGCG	0.023	0.003	0.018	24	1299
AGCAGTATATATA	0.018	0.001	0.016	69	4200
GGCGTTGTTTGGG	0.077	0.026	0.089	43	442
GCCAGCGCTGGTA	0.052	0.007	0.054	47	827
AGCGGTGTAGATG	0.022	0.009	0.014	16	1165
AGCGGTGCAAGTA	0.024	0.003	0.027	40	1464
CCCATTATTGAGA	0.026	0.005	0.03	88	2877
GCCGGTGTTAAGG	0.399	0.058	0.382	326	528
CCCGTTGTAGAGA	0.025	0.001	0.024	38	1566
AGCGTCACTGAGA	0.022	0.002	0.025	41	1601
GGTGTCGTTAACG	0.106	0.036	0.07	46	612
ACCAGTACTTACA	0.023	0.001	0.022	111	5029
ACCGGTGCTGGGA	0.024	0.003	0.021	21	995
GCCAGTATTAGCA	0.05	0.001	0.049	107	2099
CCTAGTACAGGGA	0.025	0.004	0.02	25	1207
CGCATCGTATATG	0.022	0.005	0.015	25	1655
CCCGTCATTGAGA	0.027	0.003	0.023	47	1971
ACCAGCGTAAACG	0.025	0.003	0.021	51	2404
GGTGGCGTTGGTA	0.402	0.067	0.374	161	269
CCTGTCATTTGCA	0.026	0.004	0.026	41	1536
GGCATTACTTGCA	0.056	0.005	0.05	79	1515

GCCGGTGTTGAGG	0.408	0.053	0.371	264	447
AGCGTTGCATGGA	0.021	0.002	0.024	25	1019
ACTATTATTAATA	0.028	0.001	0.028	144	5053
CCCAGCGCAGACG	0.021	0.003	0.018	38	2027
CGCGGTATAAGGA	0.025	0.002	0.026	35	1306
ACCGGTATTGGTA	0.02	0.001	0.021	36	1705
GCTAGTGCTAACA	0.052	0.01	0.045	75	1590
CGCAGCGTTGGGA	0.023	0.001	0.023	17	735
CCCGTTGCATAGA	0.023	0.001	0.025	47	1858
CCCGGTGCATGCA	0.018	0.004	0.014	26	1879
CGCATTACAGACG	0.025	0.002	0.023	46	1954
AGTAGTGCAGACG	0.02	0.003	0.015	15	972
CGTATTGCAAGTG	0.027	0.007	0.02	19	945
GGCAGTGTTTAGA	0.051	0.007	0.044	43	941
ACTGTTACAAACG	0.027	0.002	0.026	70	2663
CGCGTTGCAAGCG	0.031	0.005	0.029	34	1129
CGTGGTACTGATG	0.029	0.003	0.026	18	668
ACCATTATTGAGA	0.021	0.002	0.023	80	3394
CCCATCATTAGGA	0.027	0.003	0.03	67	2194
AGTAGCGTAGGGA	0.015	0.004	0.01	8	767
AGTGGCACATACG	0.023	0.01	0.013	10	786
CCCGGTACAAGCG	0.028	0.003	0.029	58	1935
GCTATCACTAACG	0.059	0.006	0.057	101	1665
CGTATTGCATGGG	0.029	0.006	0.036	22	586
GGCAGCGTAAAGA	0.057	0.008	0.059	95	1504
GCTGTTACATACA	0.058	0.014	0.048	81	1624
ACCGGCATTTAGG	0.023	0.005	0.027	33	1172
CCCGGCGTATGGG	0.026	0.004	0.021	18	831
ACCATTGTTAATG	0.027	0.006	0.023	62	2589
CCTGTTACTAACG	0.021	0.003	0.024	43	1721
ACCGGTATATGGG	0.026	0.006	0.018	21	1134
CGCATTACAGGGA	0.027	0.009	0.014	20	1388
GGCAGCATTGGCA	0.05	0.005	0.055	70	1213
AGCATCGCAAGCA	0.023	0.007	0.021	55	2584
CCCATCGCATGCG	0.026	0.004	0.021	42	1945
CGTATCATATATG	0.027	0.007	0.02	33	1607
AGCGTTGCTTACA	0.02	0.005	0.014	25	1750
CGTGTTATAGGGG	0.038	0.005	0.04	21	500
ACCGTCACATGTG	0.027	0.001	0.028	55	1892
GGCGTCACAGATA	0.058	0.012	0.058	95	1539

CGCATCGTTTATG	0.029	0.001	0.027	40	1435
CGTGGCATAAGCG	0.02	0.006	0.029	16	545
CCCATCATAAACG	0.027	0.002	0.03	139	4563
CCCAGCATAGACG	0.026	0.001	0.026	70	2675
GGCGTTGTAAGCA	0.063	0.009	0.051	64	1182
CCTATTACTGATG	0.027	0.002	0.027	55	2006
CCCAGTATTAATG	0.028	0.004	0.024	74	2972
ACCGTTACTTAGA	0.02	0.004	0.025	71	2774
AGCAGTACTAAGG	0.023	0.002	0.022	40	1806
GCCATTATAGGGA	0.056	0.006	0.048	65	1280
CCCATTGTAAAGA	0.028	0.003	0.026	86	3285
GGCGTCGTTAATG	0.077	0.006	0.085	79	847
ACCAGCGTAAGTA	0.027	0.008	0.021	51	2386
CCCGTCGTTAGCA	0.021	0.006	0.02	40	1986
ACCAGTGCAAACG	0.025	0.002	0.025	84	3228
CGTATCGCTAAGA	0.023	0.003	0.02	27	1294
CCCGGTGCTAACA	0.023	0.001	0.024	60	2484
GGTATCACTGGTG	0.116	0.008	0.112	61	484
AGCATTGCTTGCA	0.017	0	0.017	32	1815
GCCAGTATATGCA	0.057	0.001	0.057	145	2408
AGCAGTATAGAGA	0.023	0.003	0.019	52	2721
GCCGGCACTGACA	0.107	0.019	0.083	152	1690
GCCAGCGCAAGCA	0.051	0.007	0.053	88	1573
CCTAGTATTAGTG	0.029	0.004	0.024	38	1516
CCTGTTGTATGCG	0.027	0.004	0.022	25	1131
ACCATCACTGATG	0.028	0.001	0.029	80	2703
ACTAGTGTAGGGA	0.022	0.006	0.019	25	1287
GGTGTTACTTAGG	0.092	0.024	0.071	37	482
AGTGTTACTAGCA	0.025	0.006	0.018	24	1284
CGTGGCATAAACA	0.025	0.006	0.02	35	1693
CGCATTGCTAGGA	0.025	0.003	0.023	25	1081
GGCATTATTTGTA	0.052	0.002	0.054	92	1619
GCTGTTACTAACA	0.049	0.01	0.034	60	1680
GGCGGTGCAAGCA	0.054	0.003	0.051	54	1003
CCCATCATTGATA	0.026	0.006	0.019	73	3860
CCTAGTGTAAGCG	0.024	0.001	0.024	37	1530
CCCATCACAAGCA	0.025	0.003	0.024	108	4328
CCTATTGCTGATA	0.026	0.006	0.019	47	2368
GGCATTATAAGTG	0.059	0.003	0.056	96	1610
GCTAGCATATATA	0.062	0.004	0.057	122	2019

AGCGGCACAAAGG	0.019	0.006	0.023	26	1098
CGTATTATAAGCA	0.024	0.003	0.02	49	2367
ACCGTCGTTTGGG	0.02	0.006	0.011	6	564
CCCATTATAGAGG	0.028	0.002	0.024	55	2202
CGTATCACAAGTA	0.024	0.004	0.023	39	1650
GCTAGCGCATATG	0.053	0.01	0.043	33	734
GGCGGCATTTAGA	0.316	0.039	0.278	280	728
AGCGGTACATGCG	0.032	0.004	0.027	31	1134
ACCGGCGTAAACA	0.022	0.005	0.017	42	2393
ACCGGCATTGAGG	0.021	0.002	0.024	29	1199
GGTAGCACTGACA	0.079	0.01	0.065	71	1017
CCCGGTGCATGCG	0.026	0.006	0.034	36	1033
CCCAGCGCTGATA	0.025	0.001	0.025	67	2616
CGCAGCATTGGGG	0.037	0.009	0.034	24	676
ACCGGTGCTAGCA	0.022	0.003	0.017	36	2022
AGCAGCGTATAGA	0.022	0.008	0.03	56	1788
ACCGGCATTGGTA	0.029	0.001	0.029	49	1633
CGTAGTGCTAGTA	0.019	0.002	0.021	17	795
AGTATTGCTTGGG	0.033	0.016	0.024	15	615
CCCGGTATTGACA	0.028	0.003	0.027	70	2512
ACCGTTATTTACA	0.024	0.001	0.024	82	3344
ACTATTACTAGCG	0.024	0.001	0.024	58	2352
GCCATTACTTATA	0.05	0.009	0.04	100	2376
GCCGTCACTGACA	0.048	0.005	0.048	101	1990
AGCAGTGCTGGTA	0.022	0.002	0.023	30	1250
ACCATTATAAGGG	0.024	0.006	0.032	73	2197
ACTATCGTTAATA	0.027	0.002	0.025	71	2790
ACCATCACAGGGA	0.03	0.004	0.025	50	1925
CCCGTTATTTGGA	0.026	0.006	0.017	26	1478
GCCGGTATTAACA	0.081	0.005	0.086	204	2179
AGTATTGTAGGTA	0.024	0.004	0.019	26	1345
ACTATTGTAGATA	0.026	0.006	0.018	57	3057
GGCGTTGTTTAGA	0.066	0.007	0.058	46	749
CCTGTCATAAGCA	0.023	0.001	0.023	53	2253
GGCAGCATTTATA	0.057	0.003	0.054	96	1686
GCCATTGCTGACA	0.044	0.005	0.039	72	1772
CCTAGTACTGATA	0.03	0.001	0.032	77	2363
CGCAGTATTGGCG	0.02	0.004	0.019	23	1165
GCTGTTATTTATG	0.063	0.004	0.059	59	935
GGTGGTATTGGGA	0.117	0.013	0.107	45	375

**ACCAGTGTAGGGA**	0.022	0.006	0.015	22	1469
**CGCGGCACTAACA**	0.022	0.003	0.02	45	2185
**CGCATTACTAGGA**	0.025	0.004	0.029	41	1397
**AGCATTGCAAATG**	0.021	0.001	0.022	59	2668
**AGTATTGTAAGCG**	0.025	0.004	0.03	41	1316
**GGCATTACAGGCA**	0.061	0.008	0.064	102	1490
**GGTGGTGTTGACA**	0.129	0.016	0.128	76	516
**CCCATCGCTGGGA**	0.019	0.001	0.018	27	1455
**ACTATCGTATACA**	0.025	0.005	0.018	58	3158
**GCTATCATTAGTA**	0.062	0.003	0.063	106	1587
**CCCGGTATATGGG**	0.021	0.006	0.018	18	961
**AGCAGCATATATG**	0.022	0.002	0.024	58	2362
**GCCAGCGCTAAGA**	0.044	0.004	0.042	54	1228
**GCTATTGCTTATA**	0.05	0.004	0.05	77	1458
**GCCGGCATAGAGA**	0.379	0.033	0.389	597	936
**CCTGTTACTGGTG**	0.031	0.01	0.019	16	835
**CGCGTTATTAACA**	0.023	0.002	0.025	67	2589
**ACCGTTGTTTGCG**	0.028	0.006	0.023	27	1142
**AGTGTTGCAGGCG**	0.026	0.009	0.021	14	649
**CGTAGCATAGGTG**	0.028	0.006	0.022	16	697
**AGTGTTACTAGGG**	0.025	0.007	0.016	11	671
**CCTAGCGCATAGG**	0.024	0.005	0.018	18	991
**CCTAGTATATAGA**	0.026	0.001	0.024	53	2191
**CGCGGCACAAAGA**	0.029	0.004	0.03	54	1760
**GGCGTCACAGATG**	0.073	0.006	0.065	72	1035
**AGTGTTGTTAAGA**	0.023	0.002	0.022	28	1238
**CGTATCATAAATA**	0.022	0.001	0.02	74	3572
**CGCAGTGCTAACA**	0.02	0.004	0.022	45	1999
**CCCATCGCATACA**	0.024	0.001	0.023	94	3942
**GGTGTCGTAGGTA**	0.09	0.014	0.082	47	528
**CCCGGTGTATGTA**	0.028	0.004	0.023	34	1431
**AGCAGTACTGACA**	0.02	0.001	0.019	55	2869
**ACCAGTACAGGGA**	0.027	0.003	0.025	48	1908
**ACTATCGCATGCG**	0.025	0.001	0.024	36	1468
**CGTGTCACAAGCA**	0.021	0.005	0.021	30	1374
**CGCGTCGCTGATG**	0.026	0.004	0.031	31	962
**ACCGTTACAGGCG**	0.027	0.002	0.026	53	2008
**CCTAGCACAGGGG**	0.04	0.016	0.033	29	848
**GGCGTTGTATGTA**	0.069	0.006	0.064	62	903
**ACTAGCATAGATA**	0.026	0.003	0.022	67	2929

AGTAGCGCTAATG	0.026	0.005	0.021	21	980
GCCATCGCAAATA	0.039	0.001	0.04	98	2370
CCCAGTACAGAGG	0.026	0.004	0.022	40	1799
GGTAGTATAGGGG	0.047	0.002	0.05	32	603
GGCGGCACATAGG	0.277	0.04	0.255	220	643
AGCAGCGCAGGGG	0.023	0.007	0.022	16	727
ACTATTGCTGATG	0.024	0.003	0.024	50	2051
ACCGGCGTTAGGG	0.03	0.003	0.027	14	510
AGCGGTATTTGGA	0.015	0.003	0.012	14	1149
ACTGGTACTAACA	0.021	0.005	0.015	48	3128
GCTATTATAGATA	0.066	0.005	0.059	155	2465
ACTATCGTTTGCG	0.027	0.005	0.019	22	1125
AGCATCATATGCA	0.021	0.002	0.023	78	3244
CCCAGCACTTGTA	0.026	0.003	0.025	56	2218
GGCATCGCTAGCA	0.072	0.011	0.064	83	1219
CGTAGCGTTGGGA	0.037	0.002	0.037	19	494
GCCATTGTAAACA	0.045	0.006	0.047	153	3124
CGCATCGCAAGCA	0.025	0.008	0.015	34	2209
CCTGGCATAAATG	0.026	0.002	0.024	44	1816
AGCATTATATGCA	0.02	0.001	0.021	77	3513
CGCGGCGTAGACG	0.027	0.004	0.032	30	894
CCCAGTACTGGGG	0.039	0.006	0.031	32	1010
AGCGGTACAAAGA	0.021	0.002	0.023	51	2195
ACTGGTACTAATA	0.023	0	0.023	70	2995
ACTGTTATTTGTG	0.026	0.002	0.025	27	1052
ACCATTATATACA	0.024	0.003	0.024	155	6252
CGCGGCGCTAACA	0.023	0.005	0.016	29	1733
CCCGGTGTAAATA	0.023	0.002	0.02	53	2631
CCTGGTACTTACG	0.022	0.003	0.025	30	1194
CGCATTACAAGTG	0.028	0.005	0.022	41	1817
CCTGTTATAGAGG	0.022	0.004	0.022	29	1277
AGCAGTGTAAAGG	0.02	0.001	0.021	38	1766
CGCAGTATAAGGA	0.023	0.004	0.02	40	1923
GCCGTCGTATGTA	0.064	0.009	0.053	56	1003
ACTATCATTAAGA	0.031	0.005	0.026	84	3118
ACTGGTGTAAGCG	0.023	0.005	0.028	28	976
AGTGGTGCAAGCA	0.019	0.002	0.021	22	1029
CCTGGCGCTTGCA	0.022	0.004	0.023	24	1030
CGCATCGTTTGTG	0.025	0.005	0.022	25	1096
ACCAGCGTAAAGG	0.023	0.004	0.019	35	1844

**CGTAGCGTTTGGA**	0.031	0.004	0.026	15	569
**GCTGTTGTAGATA**	0.055	0.005	0.057	74	1226
**GGCAGTGCAAATG**	0.054	0.015	0.052	64	1178
**CCCGTTATTTAGA**	0.025	0.004	0.021	44	2048
**AGCATTGTATACG**	0.022	0.001	0.022	54	2378
**AGTAGCACAAACA**	0.023	0	0.024	62	2572
**CGCATCGTAAACG**	0.031	0.002	0.028	70	2409
**CGTGTCGCTGACA**	0.024	0	0.023	27	1135
**CCCAGCGCAAACG**	0.023	0.002	0.027	74	2687
**GGCGTTATATATG**	0.054	0.008	0.043	58	1288
**GCCGTCGCAAACG**	0.056	0.007	0.049	63	1214
**GCTATTACTTGCA**	0.07	0.012	0.054	75	1319
**GGTAGTATAGAGA**	0.066	0.013	0.059	63	1013
**ACTATCACAAACA**	0.026	0.002	0.024	140	5688
**CGTATCATATACG**	0.025	0.001	0.025	49	1934
**CCCGTCACATAGG**	0.028	0.003	0.024	40	1597
**CGTAGCACATACG**	0.03	0.004	0.024	30	1228
**CCCATTATTAAGG**	0.025	0.003	0.021	53	2528
**AGTGTTGTTAGGG**	0.019	0.005	0.021	11	507
**ACCAGTATTTACA**	0.022	0.002	0.023	102	4423
**CCTGTTATATAGA**	0.031	0.006	0.023	48	2046
**CGCATTATTTGTA**	0.025	0.004	0.02	41	2012
**AGCAGCGTTTACG**	0.021	0.004	0.019	29	1515
**GGTGGCGCAGGCA**	0.158	0.014	0.178	87	403
**GCTAGTACAGACG**	0.058	0.006	0.053	68	1226
**GGTAGTGCTTGCG**	0.05	0.017	0.033	13	376
**ACCAGTGCAAGCA**	0.021	0.001	0.02	67	3367
**ACTGTCATAAGGA**	0.021	0.003	0.017	30	1691
**ACTGGTGCATAGA**	0.02	0.001	0.021	31	1438
**CCCATCACTTGGA**	0.028	0.002	0.03	57	1838
**GGCGTCGTTAACG**	0.081	0.012	0.068	68	928
**ACTAGTATTTGGA**	0.027	0.002	0.027	43	1558
**ACTATTACTTACG**	0.026	0.003	0.03	82	2693
**ACCAGTGCTGATG**	0.027	0.002	0.027	52	1856
**CCTGTCACTGGCG**	0.034	0.002	0.035	34	948
**ACTGGTATATGGA**	0.028	0.003	0.024	32	1292
**AGTGGTGTAGACG**	0.023	0.007	0.025	18	704
**AGCAGCATTTGGG**	0.017	0.005	0.016	17	1072
**CCTGGCGTTTGCA**	0.031	0.004	0.028	33	1136
**CCCGGTGTATATG**	0.025	0.008	0.017	24	1408

CCCATTGTAAGGG	0.022	0.002	0.022	36	1624
CGCATTACTTAGA	0.027	0.008	0.017	29	1696
AGCGGCACAGATG	0.021	0.004	0.016	18	1092
CCCGTCGCAGGTA	0.02	0.001	0.021	38	1753
ACTGGTATATGTA	0.024	0.003	0.025	44	1713
ACCGTTGCAGGTA	0.021	0.008	0.016	30	1831
GGCATCACTAACG	0.098	0.019	0.074	136	1691
GGTGGTATAGGCA	0.123	0.025	0.103	66	572
GGCGTCGTTGATG	0.075	0.009	0.062	46	691
CGCGGCACTGATA	0.022	0.003	0.02	30	1500
ACTGTCACATGGA	0.021	0.002	0.019	30	1539
AGCATCACAAGGA	0.024	0.005	0.018	44	2453
GCCAGCATTGGCG	0.062	0.011	0.052	54	988
ACTATCGTATGCG	0.026	0.002	0.028	42	1482
AGTATTATTTATG	0.022	0.002	0.021	40	1870
ACTGTCACTTGGG	0.031	0.003	0.028	21	728
GGCATTGCAGATG	0.067	0.014	0.069	79	1069
GGCAGCGTATGCG	0.071	0.026	0.062	55	835
CCCATTGCTTATG	0.027	0.007	0.017	32	1812
CGCATTGTTGAGG	0.024	0.005	0.025	26	1018
GCTATTACTAACA	0.059	0.003	0.055	144	2459
ACTGTCGTTAGCG	0.025	0.006	0.03	27	884
GGTATCGTATATA	0.139	0.021	0.118	163	1219
CCTGGTACTAGGG	0.028	0.003	0.026	18	684
ACTGGCATTGATA	0.03	0.002	0.027	49	1749
CGCGTTACATATA	0.022	0.001	0.021	48	2264
CCTGTCGCATGTG	0.031	0.01	0.024	24	969
GGTGGTACAAACG	0.106	0.012	0.095	95	900
GCTGTTATAGAGA	0.063	0.006	0.071	83	1079
AGTAGTGCAAGTG	0.021	0.004	0.024	20	819
CCCGGCACAAAGG	0.025	0.004	0.019	36	1853
CCCGGTGCTTACA	0.023	0.002	0.025	51	1980
AGCATTATATGCG	0.02	0.001	0.02	49	2348
GGCGGTATTAGGA	0.076	0.015	0.071	62	809
GCTGGTACAGATA	0.074	0.002	0.075	102	1263
ACCATCACTGGGG	0.027	0.003	0.023	28	1183
CGCATTACAAACG	0.023	0.005	0.029	81	2695
CGTGGCACTAAGA	0.024	0.006	0.02	19	940
CCTATCACTTACG	0.03	0.002	0.028	65	2220
CGTAGTATTAAGA	0.028	0.001	0.027	37	1333

AGTATCACTTACG	0.026	0.005	0.021	29	1326
CCCGTTATAAACA	0.027	0.002	0.025	125	4964
CGCGTTGTATATA	0.026	0.004	0.028	46	1595
CGCATCGCTGACA	0.022	0.002	0.024	51	2031
GCTGGCGCTGGGA	0.635	0.041	0.612	356	226
GCCAGTGCTAGCG	0.051	0.009	0.053	54	960
GGCATTATATGCA	0.059	0.005	0.053	111	1999
AGCATTACAGGTG	0.018	0.001	0.018	31	1723
ACCGTCATTAATG	0.026	0.005	0.022	58	2583
CGCAGTACTGGCG	0.022	0.004	0.018	18	975
CCCAGCGCATACG	0.022	0.006	0.015	33	2206
ACCATCACTTGGG	0.03	0.009	0.043	55	1213
AGCGTTATTAGTG	0.022	0.003	0.023	33	1400
ACTATTACAGGTA	0.029	0.002	0.03	85	2783
ACCAGCGTAGACA	0.022	0.003	0.02	59	2903
GGCATCGCTTGCA	0.073	0.001	0.072	79	1024
ACTGTTGCTGGCA	0.019	0.002	0.021	29	1344
ACTAGTGTAAGCG	0.028	0.005	0.021	36	1712
AGTGGTGTTTGTA	0.022	0.004	0.022	15	669
CGCGTTATTGGCA	0.026	0.008	0.016	23	1373
GGCATCGCTTATA	0.08	0.016	0.057	85	1395
GGTAGTGCAAAGA	0.057	0.008	0.045	41	868
ACCAGTGCTGAGA	0.021	0.001	0.019	43	2168
ACCATTGTATAGA	0.025	0.003	0.021	59	2713
CGTATTGTTGGCA	0.021	0.006	0.02	20	975
CCTATTACATACA	0.026	0.004	0.022	92	4047
CCCGTCGTTTACA	0.023	0.003	0.021	49	2275
GCTGTTATAAGTA	0.066	0.006	0.058	91	1485
AGCAGCATATACA	0.024	0.003	0.024	97	3924
ACCATTGTATGCA	0.023	0.005	0.029	83	2790
ACCATTGTAGGCA	0.023	0.002	0.02	51	2445
CCCATCGCATACG	0.024	0.004	0.021	54	2517
CCTATTGTAAATG	0.025	0.003	0.022	60	2688
CCTATTGTAGGCA	0.025	0.004	0.02	38	1867
ACTAGTGCAGGGA	0.022	0.003	0.024	35	1398
CCCGTCGCATATA	0.024	0.002	0.026	68	2509
GGTATTGCTGACG	0.084	0.018	0.078	62	732
ACTGTTATAAGGG	0.027	0.004	0.023	29	1247
CCTGTTATTTATA	0.027	0.001	0.028	61	2126
CCTAGTGTAGGTA	0.021	0.007	0.016	24	1491

**ACCATCATTTATG**	0.027	0.003	0.029	91	3051
**GCTAGTGTAGATG**	0.058	0.007	0.049	54	1042
**ACCATCGCAGATG**	0.025	0.002	0.022	48	2130
**GGCATCATAAGTA**	0.077	0.011	0.068	169	2313
**CGCGGTGTAGGGA**	0.035	0.006	0.027	20	708
**CGCAGTGTAAGCA**	0.028	0.005	0.028	50	1757
**GGCGTCACTAGGG**	0.058	0.003	0.055	32	551
**AGTGTTGTTAGGA**	0.02	0.006	0.016	12	728
**GCCGGCACAAGCG**	0.214	0.016	0.226	290	994
**CCCATTACTGAGA**	0.023	0.007	0.013	38	2808
**CGTGTTACAGACA**	0.022	0.003	0.018	30	1598
**GGTGTTGTTTGTA**	0.09	0.01	0.076	43	521
**CCCGGTGTTTAGG**	0.028	0.003	0.027	25	917
**GCCATTGTATGTA**	0.054	0.006	0.054	93	1635
**GGCGTCATAAGGG**	0.062	0.009	0.051	49	918
**ACCAGCGTAAAGA**	0.025	0.002	0.027	73	2677
**AGCGGCACATATA**	0.022	0.002	0.021	43	2032
**CGCAGCGTTTGCG**	0.021	0.005	0.022	21	916
**GCTGGCACAAATG**	0.451	0.044	0.442	625	789
**AGCGTTGTAAGGG**	0.022	0.003	0.025	25	985
**CGCAGTACATACG**	0.026	0.003	0.025	49	1914
**AGTGTTATAGGGG**	0.02	0.007	0.012	9	766
**CGCATCACATGTG**	0.02	0.004	0.017	26	1521
**AGTGTTGTAAATG**	0.023	0.002	0.02	28	1386
**AGCAGTGCTAACG**	0.02	0.005	0.026	50	1897
**GCCGGCATATACA**	0.39	0.033	0.393	1012	1565
**ACTGTTACTAGGA**	0.021	0.004	0.019	32	1622
**CGTGGTATATATG**	0.022	0.002	0.024	19	765
**ACCGGCATTTGCA**	0.025	0.006	0.016	32	1983
**CGTAGTATATGTA**	0.021	0.003	0.024	32	1296
**GGCGTCATAGACG**	0.067	0.006	0.06	77	1206
**GGTAGTGTAAGTA**	0.063	0.017	0.048	40	795
**ACTGTCGTTGATA**	0.033	0.004	0.033	46	1345
**GCCAGTGCTGACA**	0.04	0.003	0.044	72	1564
**CCCGTCATAGACA**	0.028	0.001	0.029	99	3302
**CCCGGCACAGGTA**	0.026	0.005	0.031	60	1876
**AGCATTACAAACA**	0.022	0.002	0.024	133	5381
**GCTGTTATTAGCG**	0.064	0.012	0.048	40	802
**CCTGTTGTTAATA**	0.025	0.001	0.025	54	2139
**CCCGTCATAAACA**	0.026	0.001	0.025	122	4693

GCTGGTATAGGCA	0.152	0.013	0.16	167	877
ACTGTTATAGATG	0.031	0.004	0.026	47	1753
ACTGTTATATGCA	0.023	0.003	0.027	60	2187
CGCGGTACTTACA	0.027	0.004	0.032	55	1689
GGTATTATTGGCA	0.079	0.014	0.064	71	1041
AGCAGCACTAAGG	0.02	0.004	0.018	28	1511
GCTGGTGCATACA	0.12	0.013	0.103	115	1006
GCTATCACTGGCG	0.046	0.009	0.042	59	1335
CGCGGCGTAGAGG	0.017	0.005	0.019	12	611
AGCATTGCAAACG	0.019	0.004	0.014	41	2835
GCTAGTGTTAGTA	0.05	0.004	0.054	64	1132
CGCATTGCAGATA	0.024	0.008	0.019	40	2030
ACCATTATATAGA	0.021	0.003	0.025	112	4406
ACCATTGTTGACG	0.025	0.002	0.024	55	2232
AGCATCGCAGAGA	0.019	0.001	0.018	36	1925
CGCGTTACAGGCA	0.026	0.004	0.024	37	1521
GGCAGTGCATACA	0.058	0.014	0.04	61	1461
GCTGGCACATGCA	0.215	0.023	0.217	247	893
CGTAGTGCTAATG	0.023	0.003	0.022	17	744
GGTATTACAGGTA	0.082	0.007	0.076	81	991
GCCGTTATTGGTA	0.053	0.013	0.047	63	1276
GCCGGCATTAATA	0.395	0.031	0.379	976	1600
CCCGTCGCTTGCG	0.021	0.001	0.02	24	1184
GGCGTTATATGTA	0.061	0.013	0.054	75	1312
CCTATTATATGCG	0.027	0.001	0.025	55	2153
AGCGGCATTAGTA	0.026	0.003	0.031	42	1331
GCTGTCACATGTG	0.059	0.009	0.066	50	704
ACCGTCGTTGGGA	0.016	0.007	0.007	6	888
CCTATTATTAACG	0.021	0.004	0.018	56	3029
ACTATCGCATAGA	0.023	0.001	0.023	51	2190
CGCGTCGTATATG	0.023	0.001	0.024	28	1126
ACTAGTGTTAGTG	0.029	0.004	0.033	43	1276
GGTGTCATAAACG	0.105	0.012	0.099	103	937
GGTGTCACAGGCG	0.073	0.03	0.043	23	506
CCTGTTGTTAGTA	0.025	0.005	0.02	28	1380
AGTGGTACTTGCA	0.024	0.001	0.025	22	860
ACCGTTGTTAAGA	0.026	0.005	0.019	38	1960
CCTATCATATGTG	0.028	0.005	0.026	53	2009
CCTATTGCAAATG	0.03	0.004	0.024	62	2494
CGCATTACATGCA	0.022	0.003	0.02	43	2139

AGTGGCACTGATA	0.02	0.004	0.023	25	1042
AGCATCGCTGACG	0.019	0.004	0.024	46	1884
GCTGTTGCATAGG	0.041	0.013	0.048	30	593
ACTGGTGCAAACG	0.028	0.002	0.028	46	1606
ACTATTACATGTG	0.025	0.002	0.023	57	2416
CCCAGTACTTATA	0.026	0.003	0.024	78	3164
GGCGGTGTAAACA	0.07	0.001	0.068	112	1540
GCCGTTATTAAGG	0.06	0.003	0.062	82	1239
ACTGTCATATGCG	0.034	0.002	0.035	49	1337
GGTATCGCATACA	0.104	0.007	0.101	133	1187
AGTATTGCAAGTA	0.02	0.005	0.017	29	1696
GGTATTGCATAGA	0.075	0.007	0.065	62	895
ACCGTCACTAGCG	0.026	0.003	0.029	57	1930
CCCGTCGTATAGG	0.027	0.003	0.027	33	1174
GCCAGCATTTGCA	0.057	0.006	0.05	99	1895
GGCAGCACTGATG	0.053	0.009	0.049	51	1000
ACCGTCACAGGTG	0.024	0.003	0.022	36	1620
ACCATCGTTTATG	0.029	0.002	0.027	52	1861
GCTGTTGTTAACG	0.05	0.012	0.039	42	1030
CGTAGTGTAAATA	0.025	0.003	0.02	36	1734
AGTGGTGCAAATG	0.024	0.003	0.026	26	970
CGCATTGCTTAGA	0.025	0.002	0.027	37	1339
AGCATCGTTTGCG	0.021	0.005	0.027	38	1346
CCCATCACATGCA	0.026	0.003	0.022	83	3606
ACCAGTACATATG	0.023	0.002	0.024	92	3667
CCCGTTACAAAGA	0.023	0.001	0.025	76	3013
CCCGGTGCAGAGG	0.024	0.003	0.025	22	873
CGTGTCGTTTAGG	0.031	0.011	0.017	8	465
GGTAGCGCAGACA	0.083	0.018	0.072	69	887
ACTAGCATTAATG	0.025	0.004	0.029	69	2348
AGCGGCATATGTA	0.024	0.002	0.022	32	1441
CGCATTATTTAGG	0.023	0.004	0.025	34	1343
CCCAGTGCTAGTG	0.026	0.003	0.028	38	1304
GGCATCGTTAATA	0.114	0.018	0.102	207	1826
CGCATCGTATGCG	0.034	0.002	0.035	48	1340
CCTATCATAGACG	0.025	0.003	0.024	67	2688
GGTGGCGTAGGCA	0.341	0.05	0.293	175	422
GGCATCGTAGACA	0.104	0.025	0.081	163	1838
CCTATTGTATATG	0.027	0.006	0.021	44	2085
AGCAGCACAAATA	0.022	0.001	0.02	82	4003

ACTGTCACAAAGA	0.025	0.003	0.023	63	2724
AGTGGCATTGGTA	0.016	0.004	0.01	5	520
CGTGGTACTAACG	0.022	0.002	0.023	21	894
AGCAGTGCAAACG	0.023	0.003	0.022	54	2415
CCCAGCGCTTAGA	0.023	0.001	0.023	46	1945
CCCGTTGTAAACA	0.025	0.003	0.021	74	3425
GCTAGCACAGAGA	0.064	0.01	0.078	86	1016
CCTATTGCTGGGA	0.025	0.005	0.03	35	1145
GGCGTCGTATGCG	0.081	0.016	0.062	45	680
CGTGGCGCTAGCA	0.031	0.005	0.032	25	755
AGCATTGCAGGCA	0.019	0.002	0.017	34	1978
ACTGTTGCTAGGG	0.021	0.005	0.022	17	760
GCTATTATTTACG	0.059	0.008	0.049	85	1633
AGTGTTGTAGATA	0.023	0.008	0.026	35	1337
GCCATCGCTGGCA	0.055	0.004	0.056	72	1210
CCCGGTACTTGCA	0.025	0	0.026	48	1801
CCTGTCGTTGACA	0.025	0.004	0.03	56	1823
ACTATTGCAAACG	0.025	0.005	0.021	73	3335
CGCATTACATAGG	0.029	0.007	0.038	57	1425
AGCGTTGTAAAGG	0.022	0.004	0.018	27	1447
CGTAGCATATATG	0.022	0.005	0.019	24	1236
GGCGGCACAGATG	0.258	0.046	0.231	255	851
ACTAGCGTAGGGA	0.027	0.009	0.017	16	932
GCCATCATAAACG	0.056	0.001	0.056	199	3331
GGTAGTATTAAGA	0.063	0.013	0.054	68	1182
CCCAGCGCAGACA	0.025	0.005	0.019	65	3381
GGTAGCGCAAGTA	0.053	0.001	0.054	46	803
GCCAGCGCAAGCG	0.046	0.006	0.038	40	1002
ACCGTTGCTTATG	0.022	0.002	0.024	38	1553
CCTATCACTAGGG	0.035	0.003	0.033	41	1210
CCCATCGTTGGTG	0.024	0.004	0.026	36	1358
ACTAGTATTTATA	0.029	0.002	0.031	101	3118
GGTGGTATATACA	0.132	0.022	0.112	137	1081
CGCGGCGCATAGA	0.027	0.011	0.015	16	1017
AGTGGTGCATACA	0.024	0.001	0.026	32	1199
GGCATTGTATATA	0.058	0.006	0.051	103	1930
CCTATCATTGAGA	0.025	0.004	0.02	46	2301
GCCAGTGCTGATG	0.04	0.004	0.038	38	961
CGCAGCGCAGATA	0.023	0.003	0.025	44	1730
ACTGGTATTAAGG	0.026	0.007	0.017	21	1187

GCTAGCACAAATA	0.063	0.006	0.058	143	2325
ACTATTGTAGAGG	0.03	0.002	0.027	42	1527
GCTAGTATATATG	0.06	0.001	0.06	95	1497
GCTAGCACATATA	0.064	0.005	0.071	132	1725
CCCATCGCTAAGA	0.023	0.005	0.018	53	2847
GCTAGCGTAAACG	0.083	0.009	0.075	90	1105
AGCAGTGTAGGTA	0.026	0.005	0.027	45	1631
CCCGGCGCATGGG	0.021	0.002	0.018	15	797
CCTAGCATAGATA	0.021	0.003	0.026	68	2572
AGCATTACAGAGA	0.025	0.002	0.025	67	2572
AGCGGCGCAGATA	0.027	0.002	0.025	38	1491
CGCAGCGTTAATG	0.029	0.006	0.025	33	1309
CGTGGTGTAAGTG	0.03	0.009	0.023	13	550
GGCGTTACTGGCA	0.07	0.013	0.06	66	1026
CGTATTACATGGA	0.024	0.003	0.02	25	1241
ACCAGCGTTAAGA	0.025	0.001	0.024	53	2126
CGCGTTGCTAATA	0.021	0.002	0.019	33	1674
GCCGTTACAAAGA	0.056	0.004	0.053	108	1937
CCTAGCGCAGGGG	0.029	0.007	0.022	14	630
CGTAGCGTTTGTA	0.023	0.005	0.019	14	729
GGCGGTGTTTACG	0.079	0.009	0.068	53	728
CGCATTACTAGCA	0.024	0.005	0.03	62	2025
GCCATCATAAAGA	0.051	0.008	0.053	177	3169
CCTAGCGTTTAGG	0.025	0.007	0.015	14	913
AGCAGCACAGGTA	0.024	0.006	0.015	29	1863
GGTATTGCTAGCA	0.08	0.009	0.08	70	805
AGTATCATATGGG	0.029	0.006	0.021	19	904
ACCGTTACAGGTA	0.022	0.002	0.022	61	2664
ACTGTTACTGAGG	0.022	0.004	0.018	21	1137
CCCAGTATTGAGG	0.02	0.003	0.017	28	1650
CCTGTTACTTGCG	0.02	0.005	0.017	18	1049
CGCATTGCTTGCG	0.022	0.002	0.022	23	1000
CGTGTCATAAACA	0.022	0.001	0.021	53	2443
CCTGTCACTAGTG	0.029	0.007	0.02	21	1052
GGCAGTGTTAGCA	0.052	0.006	0.045	54	1153
CGTGTTGTATACA	0.02	0.003	0.024	30	1199
GCTATCGTTGGTA	0.053	0.008	0.059	57	903
ACTATTATATATG	0.031	0.001	0.03	100	3238
CCCGTCACAGGCA	0.028	0.002	0.029	73	2486
GGCATTACTAGCA	0.058	0.004	0.054	94	1662

AGCAGTATTGACG	0.02	0.002	0.023	51	2196
CGCGGTATTAACA	0.025	0.002	0.027	62	2210
CCTATTATTTACA	0.023	0.001	0.023	85	3545
GGTATCATAAGCG	0.135	0.014	0.115	105	810
GCCATTGCAAGTA	0.043	0.006	0.035	62	1692
CCCGTCACTTACA	0.023	0.004	0.02	60	2901
AGCAGCATTTACG	0.021	0.003	0.018	39	2074
AGCGGCATTAGGA	0.025	0.006	0.018	18	1010
GCTATCGTAGGCA	0.057	0.01	0.043	53	1172
GCTAGTATTAACG	0.068	0.006	0.06	97	1528
ACCAGTGCAAGGA	0.025	0.001	0.026	61	2329
GGCATCACTAACA	0.069	0.011	0.055	146	2491
CCTAGTATAAATG	0.023	0.002	0.026	76	2887
CCCGTTGTTTATG	0.022	0.002	0.025	34	1335
AGCGGTGCTAATA	0.02	0.002	0.023	42	1824
CGCAGTGTTAATA	0.031	0	0.031	65	2047
ACTGGCGCTTATA	0.03	0.008	0.034	29	814
ACCGGCACATGTG	0.023	0.01	0.017	29	1637
ACCGTCGTATGGA	0.022	0.002	0.021	27	1263
AGCAGTATTGATA	0.022	0.004	0.018	60	3216
CCCGTCGTTAAGG	0.017	0.003	0.021	28	1291
CCCAGTACATGCG	0.025	0.004	0.021	44	2045
GGCGGTACTGATA	0.059	0.005	0.052	66	1201
GGTGGCACAGGCA	0.2	0.028	0.191	139	587
AGCGGTACAGACG	0.022	0.001	0.024	38	1536
GCCGTTACAGACA	0.061	0.003	0.06	124	1946
CGTATCACAGGTA	0.023	0.003	0.024	29	1200
AGTATTGCAGACG	0.025	0.004	0.022	31	1376
CGTAGTACAAGGA	0.027	0.004	0.028	31	1071
GCTGGTACTGAGA	0.073	0.005	0.08	62	713
AGCATTGTTGAGG	0.02	0.003	0.019	26	1310
CGCATCATATATG	0.026	0.003	0.022	50	2225
GGCATCGTAAACA	0.103	0.017	0.091	253	2541
CGCGGCGTTGGTA	0.028	0.004	0.027	20	729
CGTGGCGTAAAGG	0.028	0.007	0.024	13	525
ACTGTCGCTGACG	0.03	0.004	0.032	44	1334
GCCGTTACTTATA	0.066	0.004	0.065	115	1656
GGCATTACAGGTA	0.056	0.006	0.056	92	1537
CGCGTTGTATAGA	0.025	0.004	0.031	42	1304
CCCATCGTTAGCG	0.026	0.002	0.024	45	1847

GGCAGTGTTTATA	0.051	0.007	0.042	57	1298
GGTGTTGTTAGTA	0.076	0.027	0.056	34	573
GCCATTACTAATA	0.044	0.005	0.039	122	3033
CCCGGTATAAACG	0.022	0.002	0.02	55	2697
GGCGTCGCAAATA	0.061	0.008	0.051	83	1540
CGCGGCACTAGGA	0.021	0.001	0.019	20	1030
GGCAGTGCTGACG	0.049	0.004	0.047	40	820
ACCGTTATAGGCA	0.022	0.001	0.023	60	2516
AGTGTTGCTAACA	0.024	0.003	0.019	29	1489
GCCAGTATAGGCA	0.055	0.005	0.053	117	2072
CCCATTGTTTACA	0.024	0.004	0.02	59	2852
CGTGGCGCTTGTG	0.03	0.01	0.045	17	361
ACTGTTACTTAGG	0.025	0.002	0.025	27	1064
AGTATTACTTATA	0.02	0.001	0.018	46	2452
CGTATCATAGACA	0.025	0.002	0.025	65	2541
GGTATCGTTAGGA	0.142	0.01	0.13	96	642
AGTATTGCAGGGG	0.018	0.002	0.016	10	600
CGCGTCACTTGTA	0.025	0.003	0.024	31	1282
ACCATCACATGCG	0.025	0.004	0.027	82	2962
CGCGTTGCAGACG	0.025	0.004	0.03	34	1096
GCCGTTGTTTACA	0.053	0.005	0.046	65	1353
CGCGGCATTTATA	0.024	0.004	0.021	32	1503
GGCGTTGCAAACA	0.056	0.012	0.04	74	1768
CGTGGCATAGACA	0.029	0.004	0.031	39	1234
AGCGTCGTAAATG	0.025	0.008	0.035	66	1794
CGCGTTACTGACG	0.019	0.003	0.021	30	1425
CCTGGTATTAATA	0.025	0.001	0.025	57	2234
CGCGGCATAAATA	0.024	0.002	0.025	63	2430
GGTATCGTTTGCG	0.129	0.049	0.077	40	482
CGTAGTGTAGAGG	0.029	0.012	0.012	7	585
GCCGGTGTTAACA	0.166	0.027	0.154	271	1488
GCCATTACTGAGA	0.041	0.008	0.039	67	1630
GCTATTGCTAGTG	0.05	0.002	0.048	43	857
CGTAGCACAGGTG	0.023	0.004	0.026	17	629
ACTATCGTTTACA	0.026	0.002	0.028	69	2437
GGTATCGTTTAGA	0.142	0.007	0.132	111	729
CCCATTACATGGA	0.025	0.002	0.023	56	2420
GCTAGTATTTACA	0.055	0.008	0.043	79	1742
GGCGTTACTTACA	0.061	0.005	0.056	82	1391
ACCAGCGCTAACG	0.025	0.003	0.021	52	2463

CGTGTTACTTGGG	0.025	0.006	0.026	13	480
GGCGGCGTTAACA	0.209	0.029	0.186	232	1015
GGTATCGCATAGG	0.136	0.02	0.108	61	502
ACCGTCATATATG	0.025	0.006	0.017	43	2494
AGTGTTGCTAATG	0.026	0.006	0.02	20	974
ACTGGTATATACA	0.031	0.004	0.036	93	2486
ACCATCACTTGGA	0.024	0	0.024	60	2472
ACTGTTGCATGTG	0.033	0.003	0.029	35	1178
AGCATTGTAAAGG	0.025	0.004	0.023	50	2114
CGTAGTACTGAGA	0.028	0.001	0.026	27	1005
AGTGTTGCTGGTA	0.023	0.004	0.017	13	744
CGTGTCATATAGA	0.027	0.003	0.023	30	1267
CGTATCGTTGATA	0.025	0.004	0.021	29	1366
CCTGTTGTTGAGG	0.024	0.005	0.029	26	865
GGCATTGCTAAGA	0.054	0.007	0.055	73	1257
GGTGGTATATACG	0.288	0.032	0.258	235	675
CGTATTACTGACA	0.021	0.005	0.014	29	1997
GCTATTGCATAGG	0.059	0.009	0.072	68	870
ACTGTCATTTGTA	0.026	0.003	0.025	41	1585
GCTGTCATATACA	0.059	0.002	0.056	105	1771
CCTGTCATAAGCG	0.028	0.003	0.026	42	1601
CCCATTGTAAGGA	0.025	0.001	0.026	57	2172
CCCAGTGTATGTA	0.026	0.005	0.024	49	1983
GCCGTTATATAGA	0.057	0.007	0.05	97	1827
GCCATCGCAGATA	0.046	0.005	0.048	80	1602
GCCGGTGTAGACA	0.165	0.027	0.151	222	1250
GCTGGCACAGGGA	0.199	0.033	0.2	130	520
ACCGTCATTAAGA	0.022	0.004	0.018	52	2899
CGTATCACTAACA	0.027	0.01	0.019	44	2224
CCTGTTATATACA	0.03	0.003	0.026	73	2755
ACTATTGCAGGTG	0.023	0.001	0.025	36	1417
GCCATTACAGATG	0.052	0.008	0.06	117	1822
AGTGTCATATACG	0.023	0.003	0.019	26	1318
GCTAGCATTAGCG	0.07	0.01	0.057	54	900
GCCAGTGTAAACG	0.055	0.003	0.052	96	1758
CGCAGTGTATACA	0.03	0.002	0.03	66	2161
CCTAGTGTTGAGG	0.024	0.005	0.021	22	1012
CGTAGTACAGATA	0.03	0.006	0.037	56	1460
GGCGGTATTAATG	0.122	0.027	0.102	117	1034
AGCAGCGTAAGTG	0.021	0.005	0.014	20	1399

AGCATTATTAACA	0.022	0.001	0.021	117	5337
CCCAGTATTAACG	0.025	0.001	0.025	79	3025
GCCGTTGTAAATG	0.052	0.005	0.052	79	1433
GGTGTTGTAAGTA	0.085	0.016	0.091	73	727
ACTAGCGTATACA	0.026	0.002	0.029	67	2270
ACTATCGTAAATA	0.029	0.004	0.024	88	3574
ACTATTGTATGCA	0.026	0.006	0.022	54	2357
CGTAGCATATAGA	0.027	0.006	0.023	31	1320
CCTAGCGCTGATG	0.025	0.002	0.022	23	1011
CCCGTCGTTGGCA	0.027	0.005	0.023	40	1666
CCCATTGTAGGTA	0.027	0.001	0.026	57	2131
CGCATTATTTACG	0.03	0.002	0.033	64	1901
ACCAGTGTAAGTA	0.023	0.002	0.021	59	2775
CCTAGTGTTGGGG	0.027	0.004	0.029	20	679
CCTGTCATTAGGG	0.033	0.008	0.024	21	859
AGCGGTGCAAAGG	0.018	0.003	0.022	24	1051
GGTAGCATAGGGA	0.057	0.013	0.047	40	806
GGCAGTATAGGTA	0.054	0.01	0.062	92	1389
GCTGTTGTAAGGA	0.048	0.01	0.034	29	820
CGTGTTATTGACG	0.028	0.006	0.032	32	958
CCCATCACTAATG	0.027	0.002	0.026	83	3117
CGCAGCACAAGCG	0.021	0.005	0.015	23	1527
GGCGGCACAGACA	0.145	0.024	0.14	220	1347
ACCAGTGTTGACG	0.028	0.006	0.022	42	1834
ACTGGTGCAGATA	0.019	0.001	0.019	35	1795
CCCATCGTTTATG	0.024	0.003	0.025	48	1907
CGCGGTACATACG	0.026	0.005	0.021	30	1400
CCCGTTACTGGCA	0.027	0.005	0.026	52	1981
GCCGTCGCTTGGA	0.05	0.005	0.043	27	601
AGCGGTATATACA	0.022	0.002	0.023	63	2703
ACCGGCACTAATG	0.026	0.003	0.022	52	2291
GCTATCATTTAGG	0.057	0.005	0.055	60	1027
ACCAGTACAAGGG	0.021	0.005	0.028	59	2082
AGCGTTGCAAGTG	0.024	0.004	0.024	28	1158
CCTATCGCATGCA	0.024	0.004	0.019	44	2248
CCCGTCATAAGCA	0.021	0.002	0.019	62	3157
AGTATTACATAGA	0.025	0.003	0.027	57	2078
GGTATCACATGTA	0.095	0.02	0.076	88	1068
CCTATCACATGCG	0.028	0.001	0.027	53	1915
CGCATCGCAGATG	0.026	0.002	0.028	42	1447

GCCATCATATGCA	0.053	0.002		0.052	142	2567
ACCGTTGTAGGGA	0.025	0.002		0.027	34	1244
GCCAGCATTGGCA	0.055	0.009		0.043	75	1679
GGCGTTATTTGGA	0.065	0.013		0.053	43	773
CCTGGTACAGAGG	0.025	0.002		0.023	24	1008
AGCATTATTTAGG	0.02	0.003		0.017	31	1826
CGCATTGCATGCA	0.03	0.006		0.024	42	1744
CGCAGTATATACA	0.024	0.002		0.025	79	3111
CCTAGCGCTAGCG	0.034	0.008		0.026	27	1012
CCCGTTATTGATA	0.024	0.002		0.022	59	2623
CGCGGTGTTTGGA	0.032	0.008		0.028	16	565
AGCATTGCAAGCA	0.023	0.002		0.022	57	2561
GCTGTCGTTGACG	0.067	0.015		0.071	46	605
CGCAGCACAGGTA	0.024	0.001		0.026	39	1489
ACTAGCGTAGGCG	0.028	0.006		0.035	34	932
GCCATTACAGACA	0.051	0.002		0.049	148	2888
CCCGGCATAGATG	0.026	0.002		0.024	43	1780
ACCGTTATAAGCA	0.027	0.002		0.025	89	3422
ACTATTGCAGAGG	0.03	0.006		0.037	62	1629
CGTATTACAGGTA	0.023	0.001		0.025	35	1375
GCCAGCGCTTGTG	0.057	0.011		0.058	37	599
ACTAGCATAAAGG	0.025	0.004		0.02	42	2029
ACCAGCACTTGTA	0.021	0.002		0.024	74	2969
ACCATCGTATATG	0.023	0.004		0.025	59	2267
GCTGGTGCTAAGA	0.114	0.011		0.105	88	748
CCCAGTATATGCA	0.023	0.002		0.026	82	3072
ACTGGCATAGGCA	0.026	0.003		0.025	37	1469
GGTATCGCTAGTG	0.124	0.001		0.124	70	495
CCCGTTGTTAGTA	0.024	0.003		0.026	43	1609
GGTAGCGTAAAGA	0.088	0.021		0.074	73	911
ACTGGTATAAGCA	0.03	0.003		0.028	59	2049
CGCAGCGCAAGCA	0.026	0.003		0.024	44	1806
ACCAGCGCTTACA	0.019	0.002		0.017	60	3490
CGTATCATTTGTA	0.02	0.005		0.026	38	1405
GCTGGCGCATGGA	0.617	0.031		0.596	485	329
GGCGTTACTAGTG	0.064	0.013		0.057	48	796
AGCGTCGCAAGGG	0.022	0.001		0.02	18	881
CCTGTCATTAATG	0.026	0.003		0.022	40	1772
ACCAGTACAAGCA	0.023	0.002		0.02	105	5077
ACCGTCACATGCG	0.025	0.006		0.019	39	2027

ACTAGTGTAGACG	0.028	0.002	0.03	57	1838
CCTATCGTAGGTG	0.027	0.007	0.023	29	1236
GGCGGCGTAAAGA	0.203	0.035	0.165	190	960
AGCAGTGTTAACG	0.027	0.006	0.022	46	2078
AGCGTTATTAACA	0.022	0.003	0.02	69	3371
GCCATTATAGAGA	0.051	0.011	0.041	109	2540
GGCGGCACAGACG	0.253	0.045	0.23	258	864
GCTAGTGCATAGA	0.063	0.004	0.062	66	1006
ACTGTCATTTACG	0.027	0.003	0.031	52	1600
ACTGTCATAAACG	0.027	0.002	0.025	61	2373
GCTGTTGTAAAGA	0.06	0.012	0.043	55	1218
ACCATTGTTTGTG	0.028	0.003	0.025	36	1386
GCTATTGTTTACG	0.059	0.01	0.048	60	1201
CCTATTGTTGGGG	0.034	0.007	0.025	21	824
AGTAGCGCTGGTA	0.028	0.004	0.024	18	718
GGCAGCGTAGGTA	0.065	0.012	0.057	55	918
CCCATCACTAATA	0.025	0.002	0.021	102	4721
GGCATCGCTAGCG	0.093	0.015	0.074	72	898
CCCGTCATATGGG	0.039	0.003	0.038	46	1165
AGTAGTATTGACA	0.018	0.001	0.019	39	2022
CCCAGTGCATGGA	0.023	0.003	0.024	37	1522
CCTGGCGTTTACG	0.025	0.001	0.023	23	961
AGCAGTATTGGCG	0.021	0.003	0.021	33	1547
GGCAGTGCAAGGA	0.044	0.005	0.05	46	869
GCCAGCGTTTGTA	0.049	0.005	0.042	49	1104
AGTATTACTTAGG	0.019	0.002	0.017	20	1163
CGCAGCGTTGGTG	0.02	0.004	0.014	10	695
CCTGGCGCTTATA	0.02	0.002	0.02	25	1196
GGCGTCGCTAAGA	0.059	0.009	0.057	59	982
GCTATTGTAGATG	0.059	0.002	0.057	72	1184
CCCAGCACTTATG	0.028	0.005	0.022	44	1993
CGTGTTGTATGGG	0.027	0.001	0.028	13	450
AGCGGCACTGAGA	0.021	0.004	0.017	22	1267
CGCGTTGTTTACG	0.026	0.005	0.033	34	998
CGTGGCGCAAATA	0.024	0.008	0.017	20	1181
CCCGTCATTAGCG	0.026	0.005	0.025	40	1577
CGTGGTACATAGA	0.025	0.005	0.028	26	901
CCCGGTGCAAGGG	0.029	0.005	0.025	21	835
GCCATTACTAGGG	0.056	0.003	0.055	61	1058
GGCAGTGTAGACA	0.047	0.003	0.051	80	1491

CGTGGCGTAAGCA	0.032	0.005		0.037	34	881
AGTATCATTTGCA	0.027	0.003		0.022	35	1534
GGCGTCATTGATG	0.073	0.007		0.065	63	907
CCCGGCATTTGGA	0.025	0.002		0.026	35	1324
CGCAGCACTTACA	0.022	0.001		0.02	48	2295
CCCGGTATAAACA	0.024	0.001		0.023	95	4088
GCCGTTACATATA	0.06	0.011		0.071	151	1981
GCTGGTATTTGCG	0.434	0.042		0.442	326	411
AGTATTACTGATG	0.027	0.003		0.032	50	1528
GCTGTCACAGACG	0.067	0.005		0.065	68	980
AGTGGTATAAACA	0.023	0.002		0.026	60	2287
AGCATTATTAAGA	0.022	0.003		0.019	72	3757
GGTGTTGTTTATG	0.093	0.021		0.08	40	462
GCTGTTGTAAGTG	0.06	0.005		0.054	44	776
AGTATCACTTGCG	0.024	0.004		0.021	19	880
ACCGGTACAAGTA	0.02	0.002		0.023	81	3497
ACCGTCATAGAGG	0.021	0.001		0.019	29	1506
GCCGGTATTAGGG	0.158	0.013		0.163	120	617
GGCGTCGTTTGTA	0.071	0.006		0.065	54	773
GCTAGCGTTAAGA	0.072	0.017		0.058	65	1052
ACTGGTGTAGATG	0.03	0.003		0.032	32	981
GGCATCGCATGTG	0.117	0.022		0.099	86	783
CCCGTCATTAGGG	0.04	0.004		0.037	38	982
CGCGTCGCAGACG	0.024	0.003		0.029	37	1261
ACTGTTGCTGGGG	0.027	0.004		0.028	16	555
ACCAGCGTTGAGA	0.03	0.005		0.025	44	1733
GCCAGTGCTTGGG	0.05	0.013		0.047	24	488
ACCGTCATTGGCG	0.023	0.001		0.024	40	1596
CCCATTACTGACA	0.025	0		0.025	99	3846
ACTAGTGCTGGCA	0.022	0.002		0.021	38	1766
ACCGGCGTAAGGA	0.023	0.002		0.02	25	1196
CCCGGTACTAACA	0.027	0.002		0.026	86	3210
CCCATTGCAAATA	0.024	0.003		0.024	110	4486
GGCGTCGCTAACA	0.066	0.009		0.061	85	1319
ACCGTTATTGGCG	0.017	0.001		0.016	26	1602
GCTAGTACAGATG	0.075	0.005		0.068	79	1075
GGTGGCGCTAACA	0.154	0.019		0.134	114	735
GCCGTTACATGCA	0.059	0.006		0.062	100	1503
GGTGGCGTATAGA	0.394	0.054		0.372	245	413
GCCATTACTAAGA	0.051	0.005		0.048	118	2365

CGTAGTGTTGATA	0.026	0.006	0.017	16	919
CGCATTATTTATG	0.025	0.004	0.03	56	1808
AGTGTTACATAGA	0.027	0.002	0.025	36	1379
AGCATTGTAGACG	0.023	0.001	0.021	44	2041
CGTATTGCATGTA	0.02	0.006	0.013	16	1192
AGCGTCGCATACA	0.021	0.001	0.019	41	2132
ACCGGCGCAGACA	0.021	0.002	0.019	36	1816
GCCAGTGCTGGCG	0.043	0.004	0.048	59	1159
ACCAGCACTAATG	0.023	0.001	0.023	74	3190
ACCGTCACAGACA	0.021	0.002	0.018	78	4271
GCCGTCGTTTACA	0.051	0.006	0.05	70	1344
CCTGTTATAGAGA	0.025	0.003	0.027	51	1811
GCCATTACTTGTA	0.056	0.005	0.06	114	1780
CCCATCGCTAGGG	0.025	0.004	0.024	27	1090
CGCATCACTGGCG	0.027	0.004	0.022	36	1578
ACCAGCATTGAGA	0.019	0.005	0.013	36	2750
CGTGTCGCTTAGG	0.028	0.004	0.023	12	514
CGCGGCGTAGAGA	0.02	0.006	0.011	11	954
CCCAGCGTAGGCG	0.023	0.001	0.023	34	1462
GCTGTCGTAAATA	0.048	0.003	0.045	71	1506
CGTAGTGCAAACA	0.022	0.002	0.024	40	1610
ACCATCGCAGATA	0.023	0.005	0.024	94	3791
ACTATCGTTGGTA	0.023	0.002	0.022	32	1398
CCTGTCACAGGGA	0.036	0.003	0.034	37	1063
GCCGGTATTAATG	0.15	0.01	0.147	233	1357
GGTAGCGCAGGGA	0.067	0.004	0.069	30	406
CGTATTATTGAGG	0.03	0.003	0.032	32	980
AGCAGTACATGCG	0.021	0.003	0.02	36	1725
GGTAGTATATGTG	0.072	0.01	0.073	58	741
CCTGTTACTAAGA	0.025	0.003	0.027	51	1846
CCCGTTATTGGGG	0.03	0.001	0.031	25	787
ACTATCATATATA	0.028	0.002	0.026	113	4244
ACTAGTGTATGTA	0.023	0.003	0.023	47	1958
CCCATCGCAGAGA	0.025	0.002	0.027	64	2344
AGTGGTACTGACG	0.026	0.009	0.015	13	835
GGTGTCGCAAATA	0.085	0.011	0.086	86	914
CGTATTGCATACA	0.02	0.004	0.025	43	1685
AGCGTTGTAAACG	0.019	0.001	0.021	40	1901
CCCATTGCTGGGA	0.025	0.006	0.018	24	1321
CCTATCGTAGGTA	0.026	0.002	0.026	44	1678

CCTATCGTATAGG	0.027	0.003	0.031	44	1392
CGTAGCATTGGCA	0.027	0.001	0.028	29	992
ACCAGTGCAGATA	0.023	0.003	0.019	65	3363
CCCAGCACAAGTG	0.027	0.003	0.022	49	2160
CCTGTCATTAGTG	0.025	0.004	0.021	25	1164
ACTATCATTGGGA	0.025	0	0.024	36	1436
GGCATCATTAGGA	0.072	0.003	0.069	97	1303
GCCAGCGTAGACG	0.057	0.005	0.062	82	1248
CCTAGTGTATAGG	0.026	0.005	0.032	42	1281
ACTATTACAAAGG	0.025	0.003	0.022	68	3078
GCTGTCACTTGGG	0.053	0.025	0.038	19	478
CCCGTTACTAGGA	0.022	0.002	0.025	43	1678
ACTAGTACAAGTG	0.022	0.002	0.023	53	2226
GGCGTCATAAGCA	0.075	0.015	0.071	118	1545
CCTGTCGTAAAGA	0.023	0.002	0.022	43	1890
ACCGTCACTAGCA	0.022	0.004	0.019	62	3251
GGTGTCATTAGTA	0.085	0.002	0.082	63	708
AGCGTTATTTGGA	0.026	0.001	0.027	36	1305
CCTAGCATTAGCG	0.027	0.003	0.028	40	1384
AGTGTCGTATATA	0.02	0.005	0.015	22	1441
CCCGGTATTTGCG	0.026	0.007	0.021	28	1325
GGTGGTGTAGGCA	0.096	0.015	0.078	42	495
GGTGTCATATGCA	0.093	0.009	0.088	76	785
AGCATCATATAGA	0.022	0.002	0.02	67	3309
GGTGTTACTGATG	0.084	0.02	0.057	35	581
AGTGGTACAAGTA	0.025	0.005	0.017	26	1467
GCCGTCACTTGGA	0.051	0.004	0.056	60	1011
CCTGGTATTAGTG	0.03	0.004	0.036	39	1047
GCCATCGCAGGTG	0.046	0.007	0.053	48	850
GCTAGTACTTGTG	0.06	0.012	0.074	53	664
CCCAGCGTAAGGA	0.02	0.002	0.021	42	1961
GGTATTGTTTACG	0.12	0.012	0.111	89	714
GCCGTTATAAAGA	0.064	0.005	0.058	147	2403
CCTGTTGCTTGTG	0.033	0.007	0.03	26	834
AGCGTCACTTGCA	0.021	0.002	0.023	41	1762
GGCAGTGTATGGG	0.056	0.004	0.062	37	564
CGCGGTGCTGGTA	0.022	0.008	0.015	12	803
ACTATCGTATGTA	0.027	0.002	0.028	57	1995
ACTAGTATAAGCA	0.025	0.002	0.024	76	3121
AGTGGCACAAGCA	0.021	0.005	0.015	18	1185

GGCGGCGCATACG	0.182	0.021	0.183	168	748
CCTATCGTAAGCG	0.026	0.001	0.026	50	1850
GGCGGCGCTAACA	0.1	0.009	0.092	112	1112
CCTAGCACAGACA	0.024	0.005	0.024	69	2843
ACTATTACTGATA	0.023	0.003	0.023	94	3971
CCTATCATAGATG	0.025	0.007	0.019	52	2641
ACTATCACATGGA	0.022	0.004	0.027	61	2170
CGCAGTGCAGGCA	0.029	0.005	0.021	27	1238
CCTAGCGCAAGCA	0.019	0.002	0.022	51	2302
ACCGTTGCATGTG	0.02	0.002	0.023	33	1423
GCCGGTACAGGTA	0.065	0.004	0.062	73	1103
GGCGTTATATATA	0.06	0.006	0.052	106	1952
ACCATTATAGGGA	0.025	0.005	0.021	42	1959
ACTATCACATGCA	0.024	0.001	0.025	82	3201
GGCATTATATGCG	0.057	0.006	0.052	70	1281
AGCAGTGTTTGCA	0.019	0.006	0.017	28	1600
CGCAGTACAGATA	0.025	0.005	0.025	57	2192
AGCGGTGTAAAGA	0.025	0.002	0.026	46	1734
CGCGGCATTGGCA	0.023	0.004	0.023	28	1179
AGTAGCATAGGGG	0.021	0.003	0.022	20	902
GGTATTATTAGCA	0.097	0.018	0.084	118	1281
ACTATTGTATAGA	0.023	0.004	0.028	71	2474
AGCGTCACAGGTA	0.021	0.006	0.029	46	1555
AGCGGCGTAAGGA	0.025	0.005	0.019	19	972
CGCGTCATAGATA	0.028	0.002	0.025	54	2077
GGCATTATAGACG	0.058	0.005	0.065	115	1665
AGTAGTGCATGCA	0.019	0.001	0.019	23	1169
GCTATTGCTAGTA	0.053	0.002	0.05	62	1171
AGCATTGCTGATG	0.024	0.004	0.02	33	1652
ACTAGCATTGATG	0.03	0.003	0.026	46	1738
GGCAGCATAAAGA	0.054	0.011	0.043	92	2062
ACTGTCATATATG	0.032	0.006	0.033	65	1920
AGCGTTACTTGTG	0.023	0.002	0.022	23	1043
AGTATTACTTGCA	0.016	0.001	0.018	27	1508
CGTAGCGCAGGCG	0.029	0.004	0.025	18	688
GCCGGCACTTGTG	0.233	0.049	0.211	160	598
ACTATCACTGATG	0.031	0.003	0.034	79	2262
GCCATCGTTGACG	0.054	0.005	0.058	90	1461
GGTGGTGTAAGTG	0.201	0.027	0.187	91	396
CCCGGCGTTAGCG	0.024	0.003	0.027	31	1108

ACTAGCATTTGTA	0.026	0.004	0.026	52	1933
ACTATTGTAGAGA	0.029	0.004	0.026	62	2309
CCCGTTATATGGG	0.022	0.001	0.024	29	1205
ACTAGCGCAGATG	0.024	0.006	0.016	21	1333
AGCGTTATATACA	0.021	0.001	0.019	64	3233
GGTAGTGTTGGTA	0.061	0.012	0.071	38	495
CCTAGCGCAAGCG	0.026	0.008	0.019	25	1270
CCTATTGTTAACG	0.028	0.003	0.032	78	2374
GCCGTTGTAAAGA	0.043	0.006	0.051	77	1444
CGCGGCGCTTGCG	0.022	0.002	0.021	15	687
ACCGTTATAGGGA	0.023	0.008	0.031	44	1374
GCCGTCGCTGGGA	0.043	0.009	0.035	20	556
GGCGGCGCTAATA	0.105	0.008	0.104	120	1039
GCCATTGCTTACA	0.04	0.007	0.035	65	1778
GCCAGCGTAGACA	0.053	0.003	0.048	97	1904
CGTATTACATGTA	0.026	0.007	0.016	27	1676
CGTGGCACAAGTG	0.019	0.003	0.018	13	705
CGCATTGTAAACA	0.026	0.001	0.026	88	3298
ACCGGTACAGACA	0.019	0.003	0.015	63	4130
CGTAGTATAGGTA	0.031	0.007	0.021	25	1155
AGTAGTGCTTGCA	0.017	0.004	0.012	10	838
ACTATTGCTAGGG	0.028	0.004	0.029	39	1302
CCTAGCGCTTGCA	0.027	0.007	0.03	46	1506
ACCATCGCATGCA	0.021	0.004	0.015	54	3482
CCCATTATTGGGG	0.04	0.003	0.042	48	1096
CCCGTCGTAAATG	0.021	0.002	0.019	40	2072
CCCATCACATGCG	0.026	0.002	0.027	67	2372
GGCAGTACTGGCA	0.055	0.006	0.046	57	1182
ACTAGTGCATACG	0.025	0.006	0.026	57	2119
ACTAGTACTTATG	0.025	0.002	0.027	62	2221
AGTATCGCTGACG	0.021	0.006	0.015	17	1120
CGTGGCGCAGGGA	0.016	0.007	0.008	4	478
CCTATTACTTAGG	0.024	0.003	0.019	29	1484
ACTATTATAAGCA	0.026	0.001	0.026	101	3837
GCCATCATAAGGA	0.056	0.006	0.053	114	2048
AGCAGTACAGAGA	0.022	0.005	0.016	36	2196
ACTATTGCATGTG	0.029	0.001	0.03	57	1822
CGTATCGTTTACG	0.026	0.004	0.021	24	1132
GGTAGCGCATATA	0.072	0.002	0.072	66	847
GCTGTTACATGTG	0.064	0.014	0.052	41	753

CCTAGTACTAGCG	0.027	0.007	0.023	32	1373
CCCGGCATAGGGA	0.033	0.001	0.035	47	1297
GGCATTGTAAAGA	0.073	0.013	0.068	125	1714
GCCGTCGTATATG	0.054	0.001	0.053	53	943
GCTGGCACTAACA	0.209	0.016	0.203	315	1240
CCCGTCGCTGGTA	0.022	0.005	0.015	21	1378
CCCGGCGTTAGTA	0.021	0.004	0.027	43	1558
ACCATTGCTAGTG	0.022	0.004	0.021	44	2050
CCCGTCACAAATA	0.026	0.002	0.027	117	4178
CGCGGTGCTAATG	0.024	0.002	0.022	22	999
CCCGTTATTTAGG	0.025	0.005	0.029	43	1419
AGTAGTACTTGTA	0.023	0.004	0.018	24	1285
AGTAGTATTAACG	0.024	0	0.023	43	1834
GCCGTCATAGGCA	0.052	0.008	0.041	70	1627
GCCATCGCAAACA	0.045	0.005	0.044	118	2578
CCTAGCATTTACG	0.029	0.006	0.024	42	1678
GGTAGCGCTTACG	0.07	0.008	0.059	33	527
GGCATTACAGATG	0.068	0.007	0.058	92	1490
CGTAGTACAAACG	0.026	0.003	0.029	42	1419
CGCAGTGCAAATG	0.023	0.004	0.026	40	1493
ACTATCGCTGACA	0.024	0.002	0.021	60	2761
ACTAGCGCAAATG	0.028	0.001	0.028	57	1962
CCTATTGTTTGGG	0.035	0.009	0.023	22	927
CCTGTCATTGAGG	0.026	0.004	0.03	29	947
ACCAGTGTAGGTA	0.028	0.007	0.019	38	1914
CCCGTCACATATA	0.026	0.003	0.022	72	3201
CGCGTTACTAATG	0.025	0.008	0.024	38	1579
CGTATCATAGGGA	0.03	0.004	0.029	33	1118
GCTGTTATTTACA	0.063	0.011	0.057	89	1472
ACCGTTATTTAGA	0.023	0.002	0.022	49	2229
CCTGGTGTAAACG	0.023	0.002	0.021	36	1682
CCTGTCACTTAGG	0.022	0.008	0.016	15	937
ACTGTCATTTGCA	0.022	0.002	0.025	42	1621
CGCGGTGTTAGGA	0.023	0.004	0.021	16	742
ACTATCGTAAATG	0.029	0.006	0.021	53	2432
AGCGGCACAAACA	0.02	0.003	0.017	50	2903
CGCGTCATTTAGG	0.028	0.002	0.025	24	923
ACTAGCGTTGGTA	0.028	0.007	0.037	41	1072
CCTGTCACAAGTG	0.023	0.004	0.019	25	1309
CCTATCGCTTGCG	0.021	0.006	0.013	16	1193

ACTGTCATTTAGG	0.025	0.01	0.021	23	1073
GGCGTCGCATGTG	0.072	0.01	0.071	43	560
GCTATTACAGAGG	0.059	0.005	0.061	66	1016
AGCAGCGTTGAGG	0.02	0.004	0.024	23	952
AGCGTCGCTAAGA	0.025	0.009	0.022	35	1564
AGCATCGCATACG	0.025	0.006	0.019	43	2163
GCTGGCGTAAATA	0.886	0.015	0.886	2288	295
AGTATCGTTTAGA	0.027	0.009	0.015	19	1279
CCCATCGTATACG	0.026	0.004	0.02	54	2664
CGTGTTGCATGGG	0.037	0.017	0.042	19	433
GGCAGCGCTGGCA	0.06	0.008	0.056	52	869
CCTATTGCTAGTA	0.029	0.005	0.025	52	2001
CCTATCACTTATG	0.027	0.001	0.027	60	2146
AGCAGCACTTGTA	0.021	0.001	0.023	41	1780
CCTAGCATATGCA	0.023	0.003	0.021	42	1993
CCCATTGCATACG	0.025	0.003	0.029	78	2611
CGTGGTACAAGTG	0.032	0.007	0.03	22	713
ACTATTACAAGCA	0.023	0.003	0.022	94	4221
CGTGTTGCTGGGA	0.025	0.003	0.022	11	492
ACCGTCGCTAGGG	0.03	0.003	0.025	26	1004
CCCATCGTTAATA	0.026	0.003	0.023	78	3313
CCCATTACTTACA	0.027	0.004	0.023	94	4010
CGCATCATTGACA	0.029	0.004	0.023	68	2840
GCCATCGTATAGG	0.045	0.01	0.037	41	1070
AGTATCGTTGAGA	0.022	0.007	0.017	21	1246
GCCATCATTGGTG	0.056	0.002	0.059	75	1206
CCTATCATAAAGG	0.029	0.002	0.028	76	2667
AGTAGTACTAACG	0.023	0.005	0.026	40	1477
ACTAGCACATGTA	0.019	0.003	0.015	44	2837
ACTGGTATATACG	0.025	0.001	0.023	37	1567
CCCGGTGTTAACA	0.023	0.005	0.023	52	2240
AGTAGCATTGGTA	0.026	0.002	0.023	24	1013
GCCGGTATAGACA	0.083	0.016	0.086	196	2086
ACCGTCGTATAGA	0.024	0.001	0.023	36	1540
CCCGTCGCATAGA	0.02	0.001	0.019	36	1888
ACCGGTATTAATG	0.022	0.002	0.021	49	2270
GCTGTCATAAGTG	0.067	0.005	0.06	65	1017
GCCAGTATATGTG	0.055	0.006	0.064	104	1518
CCTGGCACATACG	0.027	0.008	0.019	28	1458
ACCAGCGCTAGCG	0.021	0.003	0.019	31	1632

CGCATCGTTGGGG	0.025	0.004	0.023	14	593
GGCGTTATTAACG	0.062	0.011	0.047	64	1310
CGCGTTACATGTG	0.022	0.003	0.02	23	1111
GCCAGCGCATGGG	0.052	0.005	0.058	32	522
CGCATTACATGTG	0.023	0.001	0.024	36	1467
GGTATCATTTATG	0.14	0.018	0.128	117	800
GGTATTGTTTGTG	0.088	0.01	0.074	43	536
ACCATTACTGAGG	0.024	0.002	0.025	64	2483
AGCAGCATATATA	0.02	0.002	0.017	63	3634
CCCATTGTTGAGA	0.022	0.002	0.021	41	1926
AGTAGCGCTAACG	0.024	0.007	0.016	16	1007
GGTATTATTAGGG	0.109	0.017	0.1	69	622
CCTAGCACTTACG	0.028	0.005	0.03	48	1569
AGCGTTGCAAGGA	0.024	0.004	0.025	31	1225
GCCAGCGTAAGGA	0.042	0.003	0.046	58	1199
ACTGTTGCAAAGA	0.023	0.007	0.025	57	2196
CGTGTTATAGACA	0.02	0.001	0.018	33	1805
AGCATCATAAGTG	0.022	0.001	0.023	61	2599
CGTATCACTAACG	0.029	0.002	0.028	44	1544
GCTAGTATAAACG	0.06	0.008	0.049	103	1999
ACTGTTACTAACG	0.023	0.006	0.021	45	2146
ACCATCATAGATG	0.025	0.001	0.025	88	3365
CGCGGTGCAGAGG	0.023	0.004	0.025	17	659
CGCGTTGCTTATA	0.025	0.007	0.03	41	1335
GCCGGTGCATAGG	0.115	0.018	0.129	83	560
CCCATTGTTAGCG	0.028	0.006	0.021	35	1624
CGTGGTATAAGGA	0.028	0.001	0.026	24	905
GCTATTACATGCA	0.057	0.013	0.043	75	1670
GGTGTCGCTAGTA	0.067	0.012	0.051	30	561
CGTATCGCAAGGG	0.028	0.005	0.023	18	762
ACTATTGTAAAGG	0.031	0.009	0.019	45	2271
GCCGGTACAAATA	0.064	0.003	0.064	161	2355
GGTGTTATAAATG	0.089	0.004	0.085	109	1176
CCTATCATATACA	0.027	0.002	0.026	115	4323
ACCGTTATTGGTA	0.022	0.002	0.022	44	1986
CGCGTCATTGGTG	0.024	0.004	0.027	22	785
CCCGGTGCATGTA	0.024	0.003	0.022	35	1547
GGCAGCATAGGTG	0.052	0.002	0.051	58	1070
AGTGGCGTAAATG	0.021	0.005	0.021	12	553
ACCGGCGTTTATA	0.026	0.009	0.027	40	1449

CGCATTGTTAGCA	0.025	0.002	0.026	44	1668
GCTAGCATAAGCA	0.064	0.005	0.06	111	1739
AGCATTGTTAGCG	0.029	0.004	0.035	57	1581
ACTATCATATATG	0.034	0.002	0.031	88	2726
CCCGTTGTAAATA	0.024	0.002	0.022	66	3003
AGTGTCGTAGACA	0.018	0.005	0.013	19	1450
ACCGTTGTAAGTG	0.022	0.004	0.026	40	1471
CCCAGCGCTAAGA	0.024	0.002	0.021	54	2563
CCCATTGTTGGCG	0.023	0.007	0.016	26	1551
CGCGGCATATGTG	0.017	0.007	0.022	17	742
GCCGTTACTGATG	0.062	0.004	0.067	75	1051
ACTGTTGTTAAGG	0.028	0.008	0.022	24	1082
CGCGTTGTAGAGG	0.023	0.002	0.02	16	780
GCTGTCATTGACA	0.061	0	0.062	90	1366
AGCGGTGCTAGCA	0.017	0.002	0.021	27	1260
GGCATTACTAGCG	0.059	0.009	0.046	57	1180
CCTGTTGTTAACA	0.023	0.002	0.022	50	2255
CCTATTGTTGGTA	0.028	0.003	0.023	36	1503
CCTATTGCAGGGA	0.022	0.006	0.014	20	1397
GGCGGTGTTAGCG	0.096	0.022	0.084	52	566
ACTATCATTTATG	0.027	0.004	0.023	55	2352
GCTAGCACAGACA	0.06	0.009	0.048	79	1584
AGTATTACTAACA	0.024	0.005	0.019	60	3076
CGTGTTGCAAACG	0.026	0.004	0.026	28	1043
GGCAGTATAAGTG	0.059	0.009	0.046	65	1345
AGCAGTGCAAGCA	0.024	0.002	0.025	55	2151
GGTGGTATAGGTA	0.129	0.015	0.116	76	578
CCTATTACATGGA	0.025	0.003	0.021	41	1936
CCTGGTATAGATG	0.025	0.006	0.021	27	1272
AGTATCGTAAATG	0.022	0.002	0.018	33	1771
ACCGTTGTATGGA	0.025	0.003	0.027	37	1327
CCCATTGTATGGA	0.023	0.001	0.022	40	1797
GCCGGTACTTACG	0.074	0.009	0.084	95	1030
CCCGGTATAGGGA	0.038	0.006	0.042	49	1111
CCCAGTACTTGGG	0.024	0.002	0.024	27	1105
CGCGTCGTTTACA	0.022	0.002	0.02	31	1505
GGCGTCGCAGGCG	0.067	0.006	0.066	48	677
ACTAGCGTAGAGA	0.027	0.005	0.019	28	1443
AGTAGCGTTAGCG	0.025	0.007	0.016	11	693
AGCATCGCTAATG	0.03	0.004	0.025	55	2129

GGCGTTGTTAACG	0.079	0.011	0.069	76	1031
GCTAGCGTTTGCG	0.081	0.01	0.07	40	529
CCTGTCGTTAGGA	0.024	0.004	0.028	28	980
ACCATTGTAGGGG	0.025	0.002	0.027	34	1245
CCCATCATAAATA	0.026	0.001	0.028	177	6216
CCCAGTGTAAAGG	0.021	0.001	0.02	39	1890
GGTATCGTTTACA	0.132	0.019	0.107	139	1158
GCTGTCGTAAACA	0.058	0.009	0.049	80	1543
CGTAGTACAGGTG	0.016	0.004	0.019	12	633
AGCGTCGCTTGCG	0.022	0.007	0.014	13	946
GGCGTCGCTAGCA	0.062	0.019	0.06	60	941
ACCAGTATTGGGA	0.021	0.004	0.019	35	1774
CCCGGCATTGATG	0.027	0.003	0.029	46	1528
ACCGTTATATGGA	0.025	0.005	0.029	60	2029
CCTGGTGTAGATA	0.021	0.004	0.024	38	1537
ACTAGCATTTGGA	0.028	0.004	0.022	33	1452
CCTATTATAGATG	0.025	0.003	0.021	56	2651
AGCGTCGTTAGTG	0.021	0.002	0.021	21	977
GGCATTATTAACA	0.058	0.004	0.054	174	3072
AGTAGCGTTGAGA	0.016	0.007	0.016	14	871
ACCATTGTTAATA	0.026	0.002	0.023	95	3987
ACCGGTGTTTGCA	0.024	0.003	0.022	32	1450
ACCGTTATTAATG	0.024	0.002	0.021	56	2618
AGCGTCACATATG	0.023	0.007	0.019	34	1773
GGTGGCACATACG	0.405	0.065	0.375	334	557
GGCGGTGCTTGGA	0.052	0.013	0.058	29	474
AGCGGCGTTAACA	0.024	0.004	0.018	31	1661
CCTGGCGCATGCG	0.023	0.005	0.023	23	961
CCTGTTACAAGGA	0.027	0.006	0.021	35	1654
CCCAGCGCTAGTA	0.024	0.006	0.016	37	2312
GCCAGCACTGATA	0.055	0.011	0.063	131	1945
ACTATTATTTGTA	0.03	0.001	0.028	76	2594
GGTAGCATTTGCA	0.078	0.007	0.083	70	772
CGCATTACATGCG	0.026	0.006	0.027	40	1462
GGCGTCATAAGTG	0.06	0.005	0.064	74	1084
CCTAGCGTAAGCG	0.018	0.005	0.018	26	1405
AGTATCATTTATG	0.025	0.005	0.021	31	1458
CCTGTTATAGGTA	0.027	0.009	0.023	37	1604
GCCATTACATGTG	0.059	0.016	0.046	75	1545
AGTATTGTAGGCA	0.019	0.003	0.022	30	1326

GCCAGCGCATAGA	0.054	0.005	0.059	69	1103
CCCGTCGCATGTA	0.026	0.006	0.025	51	1985
GCTGGTGTTTGCA	0.396	0.04	0.408	331	481
AGTATCGCATATG	0.026	0.006	0.019	23	1202
GGCGTCACATATA	0.061	0.006	0.06	103	1628
ACTGGTACAGATG	0.027	0.006	0.019	28	1479
CCTATCGTATACA	0.031	0.007	0.024	70	2852
ACCAGCGCAGGTA	0.019	0.003	0.019	46	2413
CGCGGCACAAGGG	0.016	0.003	0.019	16	822
GCCAGCATTAACG	0.048	0.005	0.055	127	2187
ACCATTATAGAGA	0.023	0.002	0.024	93	3806
GGTGGTGTTAGTA	0.103	0.03	0.082	44	490
AGCGTCATAGATG	0.024	0.002	0.021	36	1662
GGTATTACAGGCG	0.081	0.013	0.068	59	812
CGCATCGCAGGGG	0.03	0.008	0.023	18	752
CCCGTCGTAAACA	0.019	0.003	0.015	57	3627
GCCGGTGTATATG	0.409	0.064	0.409	440	635
ACTGGCACATGCG	0.026	0.003	0.022	25	1092
CGTGTCACTGATA	0.019	0.006	0.018	22	1201
GCCATCGCATGGA	0.053	0.006	0.046	46	947
GCCGTTGCTGACG	0.045	0.011	0.061	60	924
CCCAGCACTAGGG	0.04	0.005	0.035	47	1315
AGTGTTACTAAGA	0.021	0.004	0.017	26	1549
GGCATCACAAATG	0.084	0.004	0.086	189	2012
CCCGGCGCAGAGG	0.024	0.001	0.023	23	972
CCCAGTGCAAATG	0.023	0.002	0.025	61	2381
GCCGGCATTGATG	0.729	0.018	0.742	1143	398
ACTGGTGCTTATG	0.027	0.006	0.019	18	925
ACCAGTATTAGTA	0.024	0.002	0.023	85	3598
AGTGTTATAGACG	0.024	0.001	0.024	30	1237
AGTGGCGTTGGGG	0.037	0.026	0.056	9	151
ACTGTTATAAACG	0.03	0.002	0.029	78	2644
GCTATTACATATA	0.06	0.004	0.055	142	2432
ACCGGCATTAGCA	0.026	0.005	0.019	47	2378
CCCGTTATAAAGA	0.026	0.002	0.025	82	3226
GCTAGTGTTGACA	0.054	0.007	0.055	76	1313
CGTGTCACATATG	0.023	0.001	0.024	25	1033
AGCATTATAAGTA	0.021	0.004	0.024	107	4275
GGCATCACAGATA	0.068	0.007	0.062	138	2098
AGTGTTGTTAACG	0.018	0.002	0.02	21	1018

ACCGTTGCTAATA	0.022	0.003	0.018	55	2941
CGCAGTACTAATG	0.028	0.002	0.031	55	1713
CGCGTCGTTAAGG	0.03	0.004	0.026	24	913
AGCGTCATAAGTG	0.017	0.005	0.011	20	1738
CCTGGTACATAGG	0.025	0.002	0.022	20	888
GCCAGTGCTGATA	0.044	0.006	0.038	55	1381
ACCAGTGTAGGCA	0.021	0.002	0.02	44	2163
ACCGGTGTTGGTA	0.027	0.005	0.02	23	1134
CGCAGCGCATACA	0.023	0.006	0.016	36	2168
ACTGGTGCTAATA	0.024	0.003	0.024	48	1975
ACTATTATTTGCG	0.032	0.005	0.026	49	1852
GGTATTACAAAGG	0.104	0.016	0.09	113	1146
AGTGGTACATATA	0.027	0.002	0.024	40	1617
CCCATCATTTACG	0.024	0.003	0.021	63	2869
CCTAGTGCAGGTA	0.031	0.002	0.027	42	1486
GCCATCATAGATA	0.057	0.003	0.054	175	3090
ACTGGTACTAGTG	0.023	0.003	0.021	23	1095
CCTAGTACAAAGA	0.029	0.002	0.032	95	2919
ACCGGCGTAGGTA	0.022	0.002	0.024	28	1139
ACTGTTACAGATA	0.022	0.005	0.021	63	2911
AGCAGTGCAAATA	0.019	0.002	0.02	65	3259
CGTGTTATTTATA	0.026	0.004	0.023	34	1456
AGTATTGCTAGCG	0.019	0.008	0.031	29	915
CCCGGCGTAAGGG	0.022	0.006	0.015	14	920
CCTAGTACTTGCG	0.023	0.003	0.027	34	1241
ACCATCATATACA	0.02	0.002	0.018	111	6009
CGCATCACAAGCG	0.027	0.007	0.029	56	1894
CCCGGCGCTAAGA	0.022	0.003	0.022	41	1783
CGTGTTATAGAGA	0.019	0.003	0.02	24	1179
ACCGTTATTGATG	0.023	0.004	0.018	38	2104
CCTAGTATAAAGA	0.027	0.004	0.033	106	3108
AGTGGCGTAAATA	0.021	0.006	0.017	23	1292
AGTAGCACATGTG	0.033	0.004	0.028	26	916
CGTATCATATGTA	0.027	0.002	0.024	44	1773
CCTAGCACAAAGA	0.028	0.003	0.026	71	2702
AGTGTCGTAGAGA	0.021	0.003	0.02	19	941
GCCGTCATTGGGA	0.057	0.017	0.051	49	915
GCCGTTATTAGCA	0.053	0.005	0.057	103	1718
ACCGTTATATGGG	0.021	0	0.021	26	1239
AGTGTTGCTTATA	0.027	0.006	0.02	23	1125

GGCGGTGTATGCG	0.082	0.008	0.08	51	587
CCTGGCATATGTA	0.024	0.001	0.025	37	1448
ACTATTACTGGTA	0.025	0.003	0.025	63	2505
CCTATTGTAAATA	0.023	0.003	0.022	91	4063
CCCGGCGCATGTA	0.022	0.002	0.02	32	1581
CGTAGCATTGATG	0.021	0.005	0.018	16	873
CGCGTTATTTAGA	0.027	0.003	0.028	41	1422
AGTAGTATATACG	0.019	0.002	0.018	32	1774
GCCGTCGCTAGGA	0.043	0.004	0.037	25	645
GGCATCATATACG	0.096	0.004	0.091	179	1787
ACCATCGTTAGTA	0.027	0.006	0.021	49	2341
ACTGTCATTTAGA	0.024	0.002	0.022	37	1667
AGTGTCACTAGTG	0.029	0.002	0.027	21	770
AGCGTTGCATGGG	0.022	0.001	0.024	18	727
ACTAGTGTTTATG	0.026	0.002	0.024	36	1470
GGCATCATAAAGG	0.096	0.023	0.069	128	1716
CCTAGCGTTAATA	0.021	0.001	0.022	51	2305
GGTGGTACAGGCG	0.098	0.017	0.109	49	399
ACCATTATAAATG	0.022	0.002	0.02	107	5212
GCCGTTATAGGCG	0.07	0.006	0.067	79	1092
GGTATCGTAAGTA	0.134	0.017	0.144	162	961
ACCATCGCAGGGG	0.034	0.007	0.026	27	996
CCTGTCACTTATG	0.029	0.007	0.02	25	1238
CGTAGTACTTGCG	0.022	0.001	0.022	15	669
ACCAGTATTTGTA	0.023	0.002	0.024	70	2888
GCCAGTGTTTACA	0.046	0.005	0.052	92	1667
CGCGGCACATATA	0.023	0	0.023	44	1864
CGCGGCACTTGCA	0.022	0.007	0.017	22	1277
CGCATTGTTAGCG	0.029	0.006	0.037	47	1235
CCTGTCGCAGAGG	0.03	0.006	0.036	31	819
GGTATCGTAAAGA	0.144	0.022	0.133	173	1126
ACCGTTGCTAACA	0.021	0.004	0.016	53	3311
AGTATCATTAGCG	0.022	0.004	0.02	25	1232
ACTAGTGTTTACG	0.029	0.005	0.026	43	1607
ACCGTCATTTGCG	0.022	0.001	0.023	36	1523
GGTGGCGTAGACA	0.384	0.057	0.364	309	541
CCCGGCACTAATG	0.025	0.004	0.024	46	1876
ACCAGCACAGGCG	0.023	0.003	0.024	50	2002
CCCGGTACTGGCG	0.034	0.005	0.03	39	1262
GGCAGTACTGGCG	0.055	0.015	0.044	38	822

AGCAGCGCTGACG	0.023	0.003	0.024	35	1416
ACCAGTGTAAAGG	0.024	0.003	0.026	56	2081
CCTGTTACTGGTA	0.023	0.004	0.027	35	1253
ACTGGCATTAACA	0.03	0.002	0.028	72	2482
AGTGTCATTGGTA	0.026	0.004	0.031	30	936
CGCAGCACTAGTA	0.023	0.004	0.019	31	1626
CGTATTATTTACG	0.026	0.007	0.018	30	1650
CCTGGTATATGGG	0.023	0.001	0.021	16	730
CGCGTCATAAATG	0.025	0.006	0.02	42	2081
AGCAGTGCAGGCG	0.021	0.002	0.018	24	1281
CCCATTACAAAGG	0.024	0.001	0.024	72	2935
GCTGGTATTAGCA	0.16	0.011	0.158	188	999
AGTATTGTTGACA	0.029	0.006	0.026	47	1763
ACCGTTATATAGA	0.026	0.002	0.026	77	2900
AGTATTACTTGTA	0.02	0.003	0.024	37	1529
ACTGTCACATACA	0.025	0.006	0.017	53	3041
GGCATTGCATGGG	0.072	0.019	0.049	34	659
ACCGTTGTTTATA	0.025	0.004	0.027	59	2123
GCTAGCATATGTA	0.06	0.01	0.047	68	1389
CGCAGCGCATGCG	0.023	0.003	0.026	28	1030
ACTATTATATGCA	0.024	0.002	0.021	70	3289
CCCGGCACAGATA	0.026	0.006	0.034	94	2664
ACCAGCATTAGTA	0.024	0.001	0.024	75	2989
AGTATTATATAGG	0.026	0.007	0.031	51	1606
GCTATTACATGGA	0.059	0.006	0.051	65	1203
CGTAGCACAGAGA	0.027	0.005	0.033	37	1090
AGCGGTATATAGG	0.024	0.003	0.026	29	1071
CGTAGCGTTGGTA	0.024	0.004	0.027	18	659
CCCAGTACTGGTG	0.029	0.007	0.019	28	1434
CGTATTATATATA	0.023	0.006	0.015	44	2851
CCCGTTACAAGCG	0.025	0.003	0.021	45	2144
CGTGTCGCAAATG	0.028	0.004	0.025	28	1086
CGCAGCATATGTG	0.023	0.002	0.02	27	1320
GCTAGTATTAGGA	0.055	0.003	0.052	61	1117
GGTATCACTGGCA	0.083	0.008	0.078	63	745
CGCATTGCAGGTA	0.025	0.008	0.036	53	1410
CCCATCGTAAGCG	0.025	0.002	0.023	55	2332
GCTGTCATAGACG	0.065	0.009	0.078	89	1051
CCCAGCGTTGGCA	0.02	0.001	0.019	35	1819
CCTATCGTAAACA	0.026	0.002	0.024	89	3689

GCCGTTGCATGCA	0.037	0.005	0.032	36	1083
ACTATCGCAGAGA	0.025	0.002	0.026	53	1994
CCCAGTGTTTAGA	0.026	0.003	0.03	50	1631
CCTGTTATAAGGA	0.024	0.003	0.021	36	1700
CCCGGTACATGGG	0.032	0.003	0.029	31	1028
ACCGTTGCAGATG	0.026	0.004	0.02	37	1809
GCCGTCATTGGGG	0.063	0.01	0.077	46	552
CGCATCATAAACA	0.026	0.006	0.025	130	5045
GGCGTTATTGGTA	0.055	0.001	0.054	55	968
GGTGTTACATGGG	0.08	0.022	0.052	25	452
CCTAGTACTAAGA	0.027	0.003	0.025	58	2227
GCTGGTGCAAAGA	0.111	0.011	0.101	94	839
CCCATTGTAGATA	0.025	0.003	0.02	63	3075
CCTAGCATAAGTA	0.024	0.007	0.023	59	2520
ACCAGCATAGGTA	0.023	0.002	0.022	59	2644
CGCATCGCATGTA	0.022	0.003	0.017	29	1640
CGCGGCATTGGGA	0.026	0.005	0.023	16	675
GCCAGCGTAAGCA	0.058	0.005	0.052	90	1639
ACCAGCATAGGTG	0.026	0.002	0.024	36	1493
CCCAGTGCAGGGA	0.024	0.006	0.019	25	1304
GCTATCATATGCA	0.056	0.003	0.055	105	1793
GGCGGCGCTAAGG	0.173	0.031	0.158	92	491
CCTAGCACTTGTG	0.026	0.004	0.023	26	1103
AGCGTTGTTGATG	0.02	0.006	0.015	18	1219
CCCATCGTAGAGA	0.021	0.002	0.02	42	2104
CGCAGTGCATACA	0.026	0.005	0.022	47	2090
GCTATCATAGGTG	0.072	0.007	0.078	79	938
CCCAGTGCAAGTA	0.025	0.002	0.022	59	2572
ACTATTGTATGCG	0.034	0.004	0.031	55	1740
AGTAGTGCAAACA	0.021	0.002	0.018	37	2040
AGTGGCGTATGGA	0.023	0.003	0.019	9	455
GCTGTTACATAGA	0.066	0.007	0.058	70	1133
GCCATTGTATGTG	0.051	0.006	0.054	63	1094
CGTATCACTAGGA	0.023	0.005	0.018	20	1075
GGCAGCACAAGCA	0.053	0.007	0.049	86	1667
CCTAGTACTTGTG	0.023	0.005	0.02	26	1262
AGTAGTATTTACG	0.023	0.002	0.022	32	1449
CCCGTCATAGATA	0.026	0.002	0.023	71	3078
GCCAGCGTATGTG	0.056	0.011	0.058	51	828
CCTATTACTGGTA	0.023	0.003	0.022	42	1834

CGTAGCGTTAGCA	0.023	0.002	0.026	24	913
ACTATCGCTTATG	0.028	0.003	0.025	43	1652
CCCGGCACAAGCG	0.03	0.007	0.039	79	1926
GCTAGCACTAGTA	0.055	0.003	0.059	73	1155
CGCATCGCTAATG	0.021	0.001	0.019	30	1569
GCTGTTATATAGG	0.072	0.004	0.073	67	848
AGTAGTGCTTGTG	0.02	0.004	0.021	13	607
AGTAGCACATGTA	0.028	0.006	0.024	34	1374
CCCGTTGCATGCG	0.024	0.001	0.023	33	1410
CGTAGCACAGACA	0.025	0.002	0.027	45	1629
ACTAGTATATGCA	0.025	0.001	0.026	72	2671
AGCGGCGCTTATA	0.021	0.003	0.025	34	1338
ACTATTACATACA	0.024	0.002	0.023	121	5162
CGCAGTACTGAGG	0.028	0.003	0.03	31	1017
AGCGTTGCATGTA	0.023	0.002	0.026	41	1534
AGTATTGTAAGGG	0.027	0.006	0.022	23	1040
AGCGGTATATGCA	0.016	0.002	0.015	28	1844
AGTAGCACAAATA	0.024	0.002	0.023	60	2594
CCTGGCGTTGATA	0.023	0.003	0.019	27	1392
GCCGGTGCTTGGA	0.061	0.013	0.08	41	471
GCTATTATTGGCG	0.055	0.011	0.067	95	1320
AGCGTTGCTTAGA	0.025	0.001	0.026	31	1140
CCTAGCGCTTATG	0.019	0.004	0.015	18	1185
CCTATCGCATAGA	0.023	0.004	0.02	40	1958
ACCGTCATATGCG	0.027	0.006	0.023	47	1999
CGCAGCACAGGCA	0.02	0.006	0.024	41	1690
CCTGGTGCATGCA	0.021	0.002	0.018	24	1275
GGTGGTGCATAGG	0.115	0.013	0.115	44	339
GCCGGCATATGCA	0.376	0.039	0.365	660	1148
GCTGTTACTGATG	0.056	0.007	0.055	44	763
ACCGGCATTGGCG	0.021	0.001	0.019	24	1214
AGCGTCACTAACG	0.022	0.004	0.017	35	2067
ACTGGTGTATGCG	0.029	0.006	0.03	25	803
GGTATTACTGACA	0.074	0.016	0.074	106	1327
GCTATTATAGGGA	0.067	0.008	0.057	60	988
CCCGGTGTATACG	0.019	0	0.019	30	1567
AGTAGCGTATATA	0.024	0.002	0.025	38	1511
ACCATTACTGGGG	0.022	0.003	0.024	28	1126
CGCAGCACTGGGG	0.028	0.008	0.023	14	605
CCCATTGTTAGTA	0.023	0.003	0.019	46	2337

GGTATTATAAATG	0.114	0.012	0.106	214	1796
GGCAGTGTTGATG	0.066	0.006	0.058	55	900
GCTGGTATTTATA	0.164	0.02	0.177	243	1129
AGCGGCGTTAATG	0.017	0.002	0.015	13	872
CCCATCGTTGACA	0.024	0.002	0.022	68	3009
GGTGGCGTAAACA	0.376	0.036	0.373	465	783
CCCGTTATAAGCG	0.024	0.001	0.022	49	2159
GGTAGCGTATACA	0.082	0.016	0.064	67	982
GCTATCGTAGACA	0.067	0.009	0.054	92	1618
ACCGTCGTAAACA	0.027	0.004	0.027	86	3055
CGTGTCGTTGAGG	0.026	0.006	0.017	10	577
AGCGTCATAAATA	0.026	0.004	0.021	83	3832
CGTATCGTTTATA	0.02	0.003	0.018	26	1455
AGTGTCGTTGGCG	0.018	0.004	0.016	10	610
AGTGTTATAGATA	0.021	0.006	0.015	31	2093
ACCATCACTAATG	0.024	0.004	0.02	85	4170
ACCAGCATTTGGG	0.026	0.001	0.026	30	1126
AGCGTCACATGGG	0.022	0.007	0.022	24	1052
AGCATCACAAGCG	0.023	0.002	0.025	63	2466
GGCGGTGTAAGTG	0.087	0.026	0.064	47	684
CGCATTGCTTACG	0.03	0.003	0.027	38	1354
GCTGGCATAAATA	0.643	0.028	0.636	1812	1035
AGCAGCGTAGATG	0.021	0.002	0.024	39	1607
CCCGTTATAGGTA	0.032	0.008	0.021	46	2121
GGTGGTATAAGTA	0.124	0.017	0.112	112	888
GGCAGCGCATACG	0.062	0.002	0.059	68	1077
CCCAGCATAAGGG	0.039	0.003	0.035	57	1551
CCCGGCACATATA	0.023	0.002	0.025	75	2875
GGTATTGTAGATG	0.113	0.014	0.107	92	766
GCTATTATTAGTA	0.058	0.002	0.055	109	1880
GGTGGTGCTAACA	0.063	0.005	0.056	46	780
CGCATTGTAAGGA	0.027	0.005	0.02	32	1562
CGCGGTATTTGCG	0.022	0.001	0.024	20	829
GCTGTTGCTTACA	0.055	0.006	0.048	53	1050
AGCATCACATACG	0.023	0.002	0.022	70	3116
ACCATCACAAAGA	0.024	0.003	0.02	116	5708
GGTATTGTTGACA	0.099	0.018	0.083	96	1056
ACCGGTGTTTATA	0.021	0.002	0.019	34	1771
CCTATTATTTACG	0.022	0.004	0.027	65	2313
CCCAGCGCATGCA	0.023	0.001	0.025	70	2749

GGCGGCATAAGGA	0.286	0.055	0.266	304	840
AGCAGCGCTTACA	0.027	0.003	0.025	51	2022
AGCATCGCTTACG	0.018	0.002	0.016	29	1795
CGTGTTACTGAGA	0.026	0.002	0.029	28	935
ACTAGTACAGGGG	0.033	0.008	0.04	36	858
GGTGTTGCTGAGA	0.058	0.005	0.064	34	496
CCTGGTATTTATA	0.026	0.003	0.023	42	1793
ACTGTTGCATGGG	0.021	0.005	0.02	18	877
GGCATCATTTGTG	0.091	0.02	0.063	73	1093
GCCAGTACTTATG	0.054	0.008	0.055	80	1386
CCTGGCGCAGGCG	0.027	0.007	0.021	19	876
AGCGTTATTTACA	0.024	0.002	0.024	61	2514
ACCGTCGTTAGGG	0.023	0.004	0.021	17	800
CGCATCGTTTAGG	0.028	0.005	0.024	26	1037
GCCGTTGCAGATG	0.052	0.008	0.04	32	764
GCTGTCGTTTGCA	0.065	0.003	0.06	46	715
GCCGGCGCATAGG	0.757	0.028	0.763	752	234
CCCGTTGCAGAGA	0.027	0.005	0.025	43	1666
GGTGGCGTAAATA	0.381	0.059	0.35	424	786
GCCGTCGTTTACG	0.067	0.004	0.066	61	857
CCTAGTACATACG	0.022	0.004	0.019	39	1969
GGTATTACTGATG	0.105	0.006	0.097	96	895
AGTGTTGTAGAGA	0.023	0.003	0.023	24	1031
GGTGGCGCATGGG	0.326	0.022	0.297	90	213
AGTATCGCTAGCG	0.02	0.006	0.014	13	895
ACTGGCACAAGGG	0.025	0.005	0.027	23	831
AGTGGTACTTGTG	0.026	0.003	0.029	16	539
CCTATCGTATGTG	0.028	0.007	0.021	30	1395
GCTATTACTGATA	0.061	0.005	0.054	105	1837
CCCATTATTTAGA	0.024	0	0.024	75	2987
GGTATCATAAGTA	0.126	0.025	0.107	164	1365
AGCATCATAAGCG	0.023	0.007	0.017	44	2622
GCTAGCACAGAGG	0.06	0.006	0.065	53	764
ACCGTTGTTTAGG	0.031	0.01	0.017	18	1040
ACTAGTGTTAGGG	0.032	0.007	0.042	43	990
AGCATTACAGGGG	0.019	0.001	0.018	24	1325
AGCAGCGTTTACA	0.021	0.003	0.018	39	2122
GGTAGTGTTTACG	0.058	0.007	0.06	38	591
CGTATCACAAAGA	0.025	0.002	0.022	46	2031
ACCGGCATTAGTA	0.025	0.002	0.024	51	2108

GGCAGTGCTTACA	0.049	0.005	0.051	63	1162
CGCAGCGTTGACG	0.026	0.003	0.025	32	1258
GCTATCACAGGCA	0.065	0.01	0.051	73	1366
CGTGGTGTAAATG	0.026	0.009	0.026	22	824
CGCGTCGCTAACA	0.024	0.001	0.022	44	1918
ACTATTACTGGCG	0.028	0.005	0.033	63	1854
CCCAGTACTGGCA	0.025	0.005	0.033	75	2225
ACTATCGCTAGTA	0.022	0.005	0.018	39	2157
CGCAGCGCAAAGA	0.029	0.002	0.03	58	1896
CGCAGTATTTGTG	0.025	0.007	0.032	39	1170
AGTGTTGCATGCA	0.025	0.008	0.015	15	1005
ACCGGTGTATACG	0.025	0.002	0.023	38	1586
CGTAGTATAAACG	0.03	0.009	0.027	46	1646
GCCGGTACATATA	0.061	0.008	0.056	109	1854
GCCATTGCTGGCA	0.044	0.007	0.033	46	1330
GCCAGCGCAAGTG	0.053	0.004	0.053	52	920
ACCGTCATTTGGG	0.027	0.004	0.023	24	1038
CGCGTCACAAATG	0.026	0.004	0.026	52	1917
AGTGTTGCAGGCA	0.021	0.004	0.016	15	918
CCCATCATTTATA	0.026	0.002	0.023	97	4085
CCCGGCGCAGGCA	0.019	0.002	0.017	30	1779
ACTAGCGCAAGTA	0.024	0.002	0.021	51	2323
ACTATCATAAATA	0.028	0.002	0.026	135	5002
GCCATTACAAAGG	0.059	0.006	0.055	112	1930
GGCGTCATAGAGG	0.074	0.025	0.042	37	854
GCTATTATTTATG	0.071	0.005	0.067	105	1456
GCCAGTATTGGCA	0.057	0.01	0.044	80	1755
ACTGTTGTTTAGG	0.024	0.005	0.024	20	827
CCCGGCATTAGCG	0.029	0.007	0.019	28	1450
AGTATTGCAAGGG	0.027	0.004	0.028	26	900
AGCATCACATATG	0.023	0.003	0.026	71	2620
ACCATTGTATGGG	0.029	0.003	0.029	40	1335
GGCGGTGTATGTA	0.061	0.007	0.068	59	811
CGCAGCACTAGCG	0.032	0.001	0.032	40	1215
CCCATCGTTTATA	0.023	0.002	0.024	66	2682
ACTGTTGTAGGGG	0.028	0.001	0.026	19	700
CGTATCACAGACG	0.031	0.007	0.023	33	1393
AGCAGTACTAAGA	0.022	0.005	0.026	68	2597
GCTAGCACTGAGA	0.053	0.003	0.05	51	971
GCTGGCGCTAGGA	0.62	0.043	0.618	483	298

GGTATTGCTGGCG	0.071	0.018	0.046	34	709
CCTGTTGCTTAGG	0.024	0.005	0.029	22	735
GCCGTCATAAACG	0.054	0.01	0.041	95	2236
CCCAGTACATATG	0.024	0.001	0.023	62	2627
CGTATTACAGAGA	0.021	0.005	0.015	24	1548
GGCGTCGTAAGCG	0.077	0.017	0.057	48	793
GCCAGCGTATGGA	0.061	0.012	0.055	55	950
CCCGGTACTGATA	0.024	0.002	0.027	60	2185
AGTATCGCTGATA	0.024	0.006	0.02	29	1394
CCCGGCGCTGGGA	0.017	0.004	0.021	20	925
CCTGGCGCAAGCG	0.023	0.003	0.02	20	963
GCCAGTACATGGA	0.05	0.01	0.043	64	1423
ACTGGCGTTAACG	0.033	0.001	0.035	24	670
CGTATCACATAGA	0.018	0.003	0.017	25	1435
CCCGTCATTAAGG	0.029	0.008	0.017	29	1648
AGCAGTATTGGCA	0.023	0.003	0.028	62	2168
AGTATCGTAAGTG	0.03	0.004	0.033	41	1199
CCCGTTACAAGTG	0.027	0.001	0.028	55	1919
GCCGTTATAAACA	0.06	0.009	0.05	174	3310
GGTATCACAAACA	0.105	0.024	0.079	154	1797
AGCGTCGCTAACG	0.027	0.004	0.028	44	1543
AGCAGCGTTAATG	0.022	0.002	0.021	38	1743
ACCAGTATATGGA	0.023	0.003	0.027	76	2740
GCCGGCATAAACA	0.39	0.037	0.371	1237	2099
CGTGGTATATGCA	0.026	0.002	0.029	30	1016
ACCGTTACAGGGG	0.023	0.003	0.025	27	1059
GGTATTGTTAATA	0.099	0.014	0.088	128	1334
CGCGTTACTAACG	0.021	0.006	0.016	28	1706
ACCAGCATTGGCG	0.026	0.002	0.026	44	1664
CGCAGTATTTAGG	0.026	0.005	0.032	39	1163
ACCGGTATAAGGG	0.023	0.002	0.022	29	1294
GCCAGTACTGACG	0.058	0.006	0.051	81	1506
CGCGTCATTGGTA	0.025	0.008	0.02	24	1184
CCCATCATTAATG	0.028	0.002	0.025	84	3299
GCTGTCACAGAGG	0.047	0.017	0.023	16	678
GGCGGCACATATA	0.129	0.021	0.119	204	1510
CCTGTTATATGGA	0.029	0.005	0.022	33	1450
AGCGGTATTGGCA	0.019	0.004	0.015	22	1450
AGCGGCATAAGGA	0.023	0.004	0.018	22	1222
CGCAGTGCAAATA	0.023	0.001	0.021	50	2288

CCCGTTGCTTATA	0.024	0.006	0.018	38	2069
GGCATCGTTGGTA	0.096	0.014	0.081	83	946
GCTATTACATACA	0.065	0.006	0.057	150	2494
GCTATCATTAAGA	0.072	0.012	0.056	113	1896
CGTGGCACTAATA	0.026	0.003	0.03	39	1278
ACCAGTGTTTAGG	0.028	0.008	0.027	34	1241
GCTAGTGTTGGGA	0.067	0.007	0.056	38	635
GCCAGTACTAACA	0.051	0.008	0.042	130	2955
GGTAGCGCAGGTA	0.064	0.006	0.057	35	583
GGTGTCATTGACG	0.1	0.029	0.074	46	575
GCTAGCATAAGTG	0.054	0.009	0.042	49	1130
AGCAGTGCTAATA	0.023	0.001	0.021	54	2496
CCTATTGCTAATG	0.024	0.004	0.019	41	2156
ACCGTTATTGGCA	0.02	0.002	0.019	41	2085
GGTATCGCAGAGA	0.097	0.024	0.076	63	771
ACCGTCGTATACG	0.021	0.002	0.018	30	1634
GGTGTTACAAACG	0.081	0.01	0.067	75	1045
CGTGGTATAAGTG	0.02	0.004	0.026	19	725
CCCGTCGCTTGCA	0.02	0.004	0.016	28	1761
CCCAGTATATGGA	0.03	0.004	0.03	64	2063
ACCATTATAGGTG	0.024	0.004	0.022	51	2227
GGTGGTGTTAAGG	0.199	0.029	0.221	99	349
CCTGTCGCTAAGA	0.029	0.003	0.034	50	1422
ACTGTCGCAAGTA	0.022	0.003	0.018	33	1841
CCTGTCGCAAGTG	0.028	0.006	0.026	27	1014
AGCGGCGTATATG	0.019	0.004	0.013	11	831
AGCAGTATAAGCA	0.02	0.001	0.022	81	3606
AGCGTCACTGACG	0.023	0	0.023	38	1612
CCCGGCACTTAGA	0.024	0.008	0.021	37	1697
CCTAGCGCTAATA	0.022	0.002	0.02	51	2437
CGTGTCACTAACG	0.027	0.005	0.023	28	1174
ACTGGTGTTTGCG	0.026	0.01	0.027	18	650
AGTGGCGTTGATA	0.032	0.006	0.028	21	735
CGCATTGCTGACG	0.021	0.002	0.018	25	1336
ACCAGCGCTAACA	0.021	0.001	0.022	105	4607
GCCGTCGTAGGCG	0.051	0.003	0.053	38	673
CCCATTATAAGGG	0.036	0.006	0.03	60	1917
CGTATCACTGGGA	0.025	0.006	0.022	17	745
GGCAGCGCAAGCG	0.062	0.013	0.055	54	927
CCCGTTATTGACA	0.027	0.004	0.022	64	2867

ACCATCGCTTATG	0.025	0.006	0.019	40	2041
AGTGTCACATATA	0.023	0.004	0.018	30	1624
AGCGTTGCTTGTA	0.019	0.007	0.012	13	1108
GCCGGTATAGGCG	0.152	0.021	0.16	168	884
AGCAGCATAGACA	0.023	0.003	0.02	70	3398
CGTATTACTTGTG	0.028	0.01	0.015	14	927
ACTATCATTGATG	0.03	0.005	0.024	56	2241
CGTAGTACTGACA	0.026	0.002	0.025	35	1353
AGCAGCACTTAGG	0.018	0.009	0.013	16	1250
GGTAGTGCTAACG	0.054	0.007	0.051	36	676
AGCATCATATATG	0.024	0.001	0.024	70	2890
ACCGTCGTAGAGG	0.029	0.005	0.021	21	963
CCCATCGCAAATG	0.027	0.004	0.032	91	2721
GCTGTTACTAATA	0.059	0.001	0.06	106	1657
CGTGTCACAAAGG	0.027	0.008	0.016	15	930
ACCAGCGTTTAGA	0.026	0.004	0.02	35	1689
CCTGGTGTAAGCG	0.023	0.005	0.028	31	1082
CGCAGTGTAGGCA	0.027	0.004	0.032	42	1291
GCTATCGCTAAGA	0.062	0.008	0.069	76	1019
GCTGTTGTTAGCG	0.062	0.006	0.053	40	710
ACTATCATAGGTA	0.028	0.01	0.019	44	2287
GCTATTACATACG	0.06	0.004	0.055	99	1707
CGCAGTGTTGGCA	0.025	0.004	0.026	29	1097
CCCATCACAAAGA	0.025	0.002	0.027	118	4301
CCTGTCGTATGTA	0.027	0.005	0.026	38	1436
AGTATCGTTAATA	0.022	0.004	0.019	43	2181
CCTATTACTGACA	0.027	0.002	0.027	81	2920
CCCAGTATTAAGG	0.02	0	0.02	45	2182
AGTGGCGCAGATG	0.027	0.003	0.027	15	544
CCCATCGCTTAGG	0.029	0.007	0.023	31	1294
GGTATTATAGGCG	0.09	0.02	0.075	69	852
GCTGTTATTAATA	0.061	0.006	0.056	114	1911
GCTGTTGCAGGTA	0.057	0.008	0.06	37	580
AGTGTTATTGGCG	0.023	0.004	0.028	25	854
AGCAGTACAAACA	0.02	0.001	0.019	96	4847
ACTAGTGCTGAGG	0.021	0.002	0.019	24	1224
CGTAGCACAAATA	0.024	0.001	0.025	51	2011
ACTAGTGCAGACG	0.025	0.001	0.027	53	1933
GCCAGTGCAAGCG	0.04	0.006	0.032	38	1147
AGCATCGCAAGTG	0.023	0.001	0.022	40	1799

ACCGTCATTAATA	0.024	0.004	0.026	100	3817
AGCGGTGTTGGCG	0.021	0.002	0.019	17	871
CGTATCACAAAGG	0.031	0.006	0.03	40	1291
GGCATTGCAGGCG	0.062	0.004	0.058	62	1005
GCCGTCGTTAGTA	0.051	0.005	0.045	48	1020
CGTAGTGTTAGTG	0.028	0.002	0.03	16	524
ACCATCATTGACA	0.025	0.002	0.027	128	4562
ACCATCGTAAATA	0.025	0.001	0.024	112	4527
AGTGTTACTTGTA	0.02	0.006	0.014	15	1057
CCCGTCACATGTG	0.028	0.005	0.025	42	1666
GCCATCGTAAGGA	0.042	0.007	0.032	45	1364
GCTGTCATAAATG	0.061	0.009	0.061	93	1437
CCTGTTACAGACA	0.026	0.004	0.03	74	2422
CGTGTCGCAGAGA	0.021	0.005	0.015	12	797
CCTATTACTAACG	0.025	0.002	0.024	67	2758
GGCGGTGTAAAGA	0.067	0.015	0.06	64	1003
GGCATTGTTTATG	0.088	0.015	0.068	75	1024
CGCATCGTTGGCG	0.023	0.004	0.018	24	1311
GGCGGCACAGAGG	0.253	0.054	0.215	159	579
AGCGTTGCAGACG	0.022	0.003	0.019	27	1371
AGTGTCACAAATA	0.018	0.002	0.017	36	2144
AGTGTTGCATATA	0.02	0.004	0.026	35	1327
GGTGTCATTAATG	0.12	0.017	0.121	103	745
GCTATTGTAGATA	0.057	0.004	0.053	102	1817
ACCAGCATATAGG	0.023	0.004	0.019	42	2158
AGTATTGCAGAGA	0.028	0.006	0.021	27	1258
GGCGGTACTGGGG	0.064	0.013	0.063	25	371
GGTGTCGTAAACA	0.1	0.008	0.09	98	995
CCTGGCACAAGTA	0.026	0.004	0.02	36	1762
CGCGTCGTAGGTG	0.021	0.006	0.029	22	743
CCCGTCGCATGCG	0.021	0.005	0.016	23	1443
CCCATCGCTGAGG	0.027	0.012	0.022	31	1375
AGTATTATAAACG	0.026	0.003	0.03	92	3013
ACTGTTATTTGCG	0.028	0.007	0.028	32	1130
GCTGTCACTAGGG	0.048	0.007	0.045	24	510
GGCGGTACATGCA	0.061	0.007	0.052	61	1106
ACCAGCATTTATA	0.025	0.002	0.022	86	3739
CGCGGTGCAGGGA	0.019	0.004	0.014	10	700
ACCATTACTTGTG	0.025	0.004	0.022	53	2411
CGCGGCGCAGAGG	0.02	0.004	0.023	16	689

GCCAGCATTTGTG	0.057	0.006	0.061	73	1130
CGCATCACTTGTG	0.024	0.002	0.026	33	1233
AGCATTGTTAGGG	0.014	0.001	0.014	16	1105
CCTGTCGCTGACG	0.022	0.002	0.02	22	1100
CGCATTACAGGTG	0.023	0.003	0.023	29	1259
CGTAGCGCATGCA	0.022	0.005	0.02	21	1040
ACCGGTACAGATA	0.021	0.003	0.018	69	3785
CGTGGCACAGGTG	0.028	0.006	0.02	10	488
CCCGTCATTTGCA	0.029	0.002	0.027	58	2127
GCCGTCATTAAGG	0.051	0.003	0.048	60	1181
CCTAGCATTTAGA	0.028	0.004	0.031	53	1665
GGTGTCGTAAGTA	0.095	0.004	0.091	76	760
ACCAGCACAAGCG	0.025	0.004	0.024	78	3198
AGCATCACAAGTG	0.024	0.001	0.023	53	2292
GCCGTCGTTTGCA	0.052	0.007	0.053	55	981
ACTAGTACAAGGA	0.022	0.003	0.023	63	2678
CGTATTACTGATA	0.021	0.003	0.024	43	1749
CCTGGTGCAGGGG	0.026	0.007	0.018	9	496
ACCAGCGCAAGTG	0.025	0.002	0.024	46	1887
AGCATTGTATATG	0.026	0.002	0.024	53	2181
CCTGTCGCTAATG	0.024	0.007	0.016	20	1216
GGCAGCGTTGACA	0.059	0.007	0.053	74	1317
GGCATCACTGATG	0.084	0.008	0.076	108	1322
ACTGTTGCAGGGA	0.019	0.005	0.026	29	1095
GCTATTATTAGCG	0.065	0.007	0.06	80	1245
CCTATCATAGATA	0.026	0.002	0.027	100	3664
GCCATCATATAGG	0.063	0.005	0.059	98	1570
ACTGGTACAGACA	0.024	0.003	0.022	62	2716
GGCATCATAAGGA	0.074	0.008	0.071	132	1718
CCCAGTGTTGGCA	0.02	0.005	0.013	22	1655
CCTAGCGCAGGCA	0.027	0.001	0.025	42	1629
GCTAGTACATATA	0.062	0.005	0.057	111	1832
CGTGTCATAAGCA	0.026	0.003	0.029	47	1551
GGTGTCGCTAGGG	0.109	0.011	0.099	31	282
CGTAGCGCAAGCA	0.026	0.005	0.019	23	1193
ACCATTACTGGCA	0.021	0.003	0.018	65	3520
GCCGGTACTAGCA	0.066	0.001	0.065	95	1370
ACTGGTATTTGTG	0.029	0.004	0.024	22	885
GGCGGTGCAGAGG	0.07	0.021	0.068	33	453
GGTATCGTTGACA	0.127	0.008	0.12	145	1060

AGTATTATATGGA	0.026	0.001	0.028	51	1793
ACCATCGTAGGCA	0.024	0.003	0.023	53	2300
GCTATTATAAGCA	0.064	0.003	0.061	148	2289
GGCGTCGTATATA	0.078	0.014	0.06	81	1265
AGTGTCGTATGTG	0.022	0.004	0.016	12	717
ACTGGCATATAGG	0.035	0.007	0.045	43	919
GCTATTGTTAAGG	0.066	0.011	0.054	63	1104
ACCAGTATAAGGA	0.025	0.001	0.024	81	3227
CCCGTCGCAAATG	0.025	0.001	0.023	47	1982
CGCAGCATTAGCA	0.02	0.003	0.024	47	1926
CCCATCGCTGACA	0.026	0.003	0.022	74	3273
AGCGTTACATGTA	0.02	0.004	0.019	36	1899
CCTATTGTTAGGA	0.027	0.006	0.023	35	1512
ACCGGTGCAGATA	0.021	0.003	0.022	52	2275
GCCATTACTTACA	0.053	0.006	0.045	119	2551
ACTGTTATAGACA	0.029	0.001	0.028	79	2708
CCCATCACTAGCA	0.026	0.002	0.023	80	3348
GGCGTTACTAGGA	0.065	0.005	0.071	66	866
AGCGGTGTTTAGG	0.017	0.001	0.017	13	738
CGCGGCGCAAACG	0.023	0.002	0.026	39	1436
CCCGGCGTTTACG	0.021	0.001	0.023	30	1287
ACCAGCACAGGTA	0.022	0.003	0.021	68	3124
AGCAGTGTTTATG	0.025	0.005	0.019	28	1425
CGTATCGCATGTA	0.026	0.003	0.026	32	1187
CGTGTTACTGGGG	0.036	0.002	0.037	17	440
CGTATTGTTAACG	0.023	0.007	0.017	21	1227
ACCGGTATAGATG	0.026	0.003	0.023	46	1950
CGCAGTACTTAGG	0.019	0.003	0.022	21	936
CCCGGTACTTACG	0.022	0.006	0.013	24	1805
CCCGTTACATGCA	0.025	0.002	0.026	70	2646
AGTGTCGCTGGTG	0.019	0.004	0.024	11	453
AGCGGTATAAACA	0.023	0.002	0.024	87	3515
ACTATTGTTTAGA	0.023	0.003	0.019	39	1991
ACCAGCACAGATG	0.024	0.002	0.021	64	3024
AGCAGCATAAGCG	0.018	0.002	0.015	32	2089
CGTGTCACTAGTA	0.033	0.007	0.031	30	953
CCTAGCATAGGGG	0.039	0.009	0.038	35	892
GGTATCGTAGATA	0.15	0.022	0.127	157	1080
CCCAGCGCTGGCG	0.026	0.001	0.026	34	1267
CCCAGCGCTAATA	0.02	0.003	0.023	81	3372

ACCAGCGTTTATG	0.027	0.002	0.026	39	1480
GGCGGTGTTAAGA	0.067	0.017	0.065	63	908
AGTAGCGTTAATG	0.037	0.006	0.03	34	1098
GGTATTATTAGTA	0.104	0.016	0.091	139	1389
ACCGTTACTGGGA	0.029	0.003	0.028	44	1541
CGTGGCGCAAAGA	0.028	0.008	0.017	16	941
ACCGTTGCAAGGA	0.023	0.004	0.027	50	1807
ACCATTGCAAGTA	0.022	0.003	0.018	64	3497
CCCGGCGCAAATG	0.028	0.002	0.026	50	1910
CCCAGCACTGAGG	0.026	0.004	0.03	47	1536
AGTAGTACATGGA	0.025	0.002	0.022	24	1056
AGCATCGTATGGA	0.02	0.001	0.018	29	1604
ACCGGTGTAGATG	0.029	0.007	0.02	28	1403
AGCGGTGCTAAGG	0.019	0.001	0.019	15	782
AGCATTGTATGCG	0.021	0.001	0.02	33	1617
CCTATTACTGAGA	0.02	0.002	0.022	46	2030
GCTGTCACTTACA	0.053	0.007	0.043	56	1251
ACCATCATAGGGG	0.025	0.001	0.026	33	1234
CGCGGTGCAAGCG	0.019	0.005	0.018	18	970
CCCAGCGTTGGGG	0.035	0.009	0.023	17	722
CCTGTTATTGAGA	0.024	0.004	0.021	34	1550
AGCATCGTTGATA	0.019	0.002	0.016	39	2439
GGCATTACAAGTA	0.052	0.008	0.044	102	2203
CCTGTTATTTAGG	0.021	0.003	0.018	19	1014
CCCGTTGCAAATG	0.025	0.001	0.026	57	2151
AGCATTGCATACG	0.022	0.004	0.026	62	2280
GGTGGTGCAGACA	0.061	0.003	0.059	44	705
GCCGGCATTGATA	0.396	0.022	0.39	724	1134
CCCGTCGCTAAGA	0.027	0.003	0.028	58	2011
GCTATCATAAATG	0.062	0.009	0.052	116	2106
GGCGTTGCTAAGA	0.052	0.008	0.05	50	947
GCCAGCGCTTACA	0.053	0.004	0.054	82	1424
CGTAGCGTTGAGG	0.028	0.01	0.037	19	497
ACCAGCGCAGATG	0.026	0.003	0.023	44	1882
CGTATTGTTGGGG	0.026	0.006	0.018	9	485
GCCGGTGCAGATG	0.1	0.019	0.102	79	697
ACTATCACTGGGA	0.028	0.006	0.022	31	1350
CGTAGTACAGACA	0.028	0.005	0.027	43	1552
ACCGGCATTAATA	0.027	0.003	0.028	92	3160
AGCATTGTTAAGA	0.023	0.003	0.02	50	2480

CGTGGTGTATATA	0.028	0.004	0.024	24	972
CGCAGTGTTTAGA	0.021	0.004	0.026	30	1106
GCCGTTACTTGTG	0.064	0.005	0.07	61	811
GGCGTCACTGACG	0.061	0.007	0.051	58	1080
CGCGTCGTTGATG	0.023	0.003	0.023	22	953
CGCATTGCATAGG	0.03	0.002	0.028	31	1064
CGCAGTATAAATG	0.026	0.004	0.023	59	2482
ACCATTACAGGTA	0.021	0.001	0.022	81	3529
GGCGGTACTTGTG	0.076	0.027	0.048	29	573
AGTAGCATAAATA	0.023	0.003	0.019	60	3138
AGTGGCACTGGCA	0.027	0.003	0.024	18	747
GCCGGTATAGACG	0.17	0.026	0.157	231	1237
CCTGTTGCTGGTG	0.023	0.008	0.013	10	756
CCTAGCGCAAATG	0.025	0.003	0.025	45	1760
GCTGTTATTTACG	0.061	0.006	0.054	54	945
GCCAGTATTAGTG	0.051	0.009	0.057	85	1404
GGCGGTACAAATA	0.069	0.004	0.064	137	2006
CGTAGTATATACG	0.026	0.001	0.027	37	1334
CGCGTTATAAAGG	0.022	0.004	0.021	33	1539
GCCAGTGCAAGTG	0.043	0.002	0.041	42	973
GGCGTTACAAGGG	0.07	0.024	0.045	35	737
GCTATCGTTTACA	0.056	0.001	0.055	86	1478
CGCGTTGTTTGTG	0.025	0.01	0.017	12	687
AGCGTTATTTGTA	0.02	0.002	0.018	32	1728
AGTAGTACTAAGA	0.027	0.001	0.029	50	1690
GGCAGTACAAACA	0.054	0.009	0.054	159	2772
CCTATCACAGATA	0.027	0.006	0.022	79	3530
GGCAGTATTAATG	0.062	0.008	0.051	84	1560
CCCATTACTGAGG	0.027	0.003	0.025	48	1847
CCCAGCACTTAGG	0.024	0.002	0.027	42	1517
AGCGGCACAGAGA	0.019	0.002	0.018	25	1357
ACCGGTGTAGGTG	0.025	0.002	0.023	24	1013
GCCAGCATATACA	0.051	0.007	0.055	185	3184
CCTATCACAAGGG	0.038	0.003	0.039	56	1387
CGCGGTACTAACA	0.021	0.002	0.02	45	2262
GGTATCGTATGCA	0.132	0.025	0.109	113	923
CGCAGCATAGGTG	0.03	0.004	0.028	29	1009
GGTAGCACAGAGG	0.061	0.015	0.048	24	472
GGTGGTACTTATG	0.102	0.027	0.084	47	512
GCTGTTACAGGCG	0.064	0.004	0.059	48	759

GGTAGCACATGGA	0.069	0.021	0.067	51	713
CCCGTTATAGGCA	0.028	0.002	0.025	62	2371
ACTAGCACTAATG	0.028	0.003	0.028	66	2278
CCTGTCGCATACG	0.024	0.005	0.031	45	1411
ACCGGTACTTGGG	0.019	0.003	0.015	18	1174
GGTAGTGTTGAGA	0.086	0.029	0.056	35	589
ACTAGTACTGAGA	0.025	0.005	0.019	50	2591
CGCATTACTAATA	0.026	0.002	0.024	70	2903
GCCGGTGCTAGTG	0.104	0.02	0.092	57	563
CGCATCATTTACA	0.03	0.002	0.033	102	3024
CCCATCGTAGGTG	0.024	0.003	0.028	40	1364
CCCAGCGTAGGCA	0.024	0.004	0.024	53	2118
CGCGGCACATGGG	0.03	0.004	0.03	22	705
CCCATTATATGCG	0.026	0.002	0.024	66	2706
ACTATCACTAGTA	0.023	0.007	0.025	74	2851
CCCGGCACTTACA	0.022	0.002	0.023	59	2546
ACCAGTGCTGGGG	0.017	0.002	0.019	19	999
CGTATCGCTGATA	0.023	0.003	0.021	27	1279
GCCATCATATAGA	0.056	0.008	0.066	178	2515
CGTGTCGTAAATG	0.025	0.001	0.024	27	1120
ACTAGCGCAAACG	0.026	0.001	0.027	56	2036
CGCGGTACTAATA	0.021	0.003	0.021	43	1975
GCCGTTACAGGGA	0.044	0.005	0.039	39	953
ACCGGTATATACG	0.021	0.003	0.017	38	2245
AGCATTGCAAAGG	0.019	0.005	0.014	28	1991
ACCGTCGTTTACA	0.022	0.004	0.018	37	2021
AGTGGCATAGGTA	0.025	0.011	0.012	7	582
AGCATTGTTTATG	0.023	0.006	0.02	36	1736
CGTGTCGCTAACG	0.028	0.003	0.03	30	957
GCTGTTACAGATA	0.055	0.003	0.051	84	1550
GCCATCGCAGATG	0.044	0.007	0.038	45	1152
GCTATCACAAACA	0.058	0.005	0.057	162	2668
ACCAGTGTTGATA	0.024	0.003	0.027	68	2442
ACTGGCGTTTATG	0.022	0.013	0.015	8	542
GCCATTATTGATG	0.052	0.003	0.052	107	1935
GGTGTCGCATAGA	0.093	0.02	0.071	45	593
GGCAGCATAAATG	0.053	0.005	0.049	98	1916
ACTGGTACTTATA	0.022	0.002	0.024	55	2236
AGTGTCGTTAATG	0.024	0.007	0.016	14	888
ACTGTCACTAGGG	0.027	0.005	0.02	19	932

CCTGGCACATGGA	0.027	0.006	0.019	22	1115
GCTAGTATTAGCA	0.062	0.008	0.073	112	1418
AGCGTCACTTAGG	0.024	0.004	0.025	25	993
GGTATCACTAATA	0.098	0.022	0.078	124	1469
ACCGTTGTTGAGG	0.027	0.004	0.022	23	1021
AGCAGCGCTGACA	0.021	0.001	0.02	40	1947
GCTGGTACTTACG	0.104	0.009	0.093	73	710
CCTATTACATATG	0.021	0.001	0.022	55	2410
CCCAGTGCATGTA	0.026	0.002	0.028	58	1987
AGTAGTACTAATG	0.026	0.005	0.019	28	1460
GCTGTTATTTGCG	0.067	0.004	0.071	51	664
ACCGGCGCTTGCA	0.02	0.002	0.021	24	1127
GCCGGTGTAGGCG	0.34	0.051	0.321	299	632
GGCAGTGCTAGTG	0.051	0.012	0.053	38	685
GGTAGTATTGGGA	0.067	0.008	0.075	45	557
AGTGGTACAGGTG	0.031	0.003	0.028	17	591
CCTATCATTAATG	0.03	0.001	0.031	92	2894
ACTGGTGTAAGTA	0.029	0.003	0.028	44	1517
CCTATTATAAAGA	0.028	0.001	0.028	114	3964
GGCATCATATATA	0.079	0.012	0.07	186	2490
ACTGGTACAAAGG	0.023	0.003	0.019	29	1490
GCTATCACTTGGG	0.053	0.012	0.042	27	614
ACTGTTGTTAACG	0.027	0.003	0.026	42	1554
CGCAGCGCTAAGG	0.033	0.007	0.043	42	941
ACTGTTGTTTATA	0.03	0.003	0.033	59	1717
GGTATCGTTAACG	0.136	0.022	0.105	91	773
GCTATTACTTGTG	0.063	0.004	0.059	57	917
GCTATCGTATAGA	0.055	0.002	0.053	70	1251
CGCGTCATTGACG	0.023	0.002	0.02	28	1377
CGCATCGTAAACA	0.025	0.002	0.023	85	3652
CCCGTCATTAACA	0.025	0.002	0.023	94	3967
CGCGGTATATGGG	0.02	0.006	0.014	10	708
CGTGGTGCATGCG	0.019	0.003	0.015	8	524
GCTAGCGTTGATA	0.067	0.007	0.06	71	1119
CGCATTACTAGTA	0.022	0.002	0.025	50	1989
GCCATCACTTATA	0.052	0.002	0.05	125	2374
CCCGGTGTATGCA	0.025	0.004	0.029	49	1629
CCTGGTACAAACA	0.026	0.005	0.022	62	2788
CGCGTTGTAAGGA	0.021	0.002	0.02	24	1164
GCCATTATTGACA	0.05	0.001	0.05	165	3154

AGCGGCGTAAAGG	0.019	0.007	0.029	25	835
AGCATCGCTGGTG	0.022	0.003	0.017	19	1073
GGTGGTACTAATA	0.089	0.015	0.077	89	1064
CGCGGTACAAAGG	0.023	0.005	0.017	19	1123
ACTGGTATATAGG	0.026	0.003	0.026	28	1038
CCTAGCACAGATA	0.023	0.005	0.019	51	2578
GGTGTTGTAGGCG	0.081	0.011	0.069	32	434
AGTAGTACTAACA	0.024	0.003	0.021	47	2224
CGCATTGTTGGGA	0.024	0.006	0.019	18	914
ACTAGCATATGCA	0.027	0.003	0.031	79	2474
CGCGTTATAGATA	0.026	0.001	0.025	57	2258
CGTGTCACAAACG	0.031	0.009	0.021	28	1308
CGCAGTATTAAGA	0.023	0.006	0.016	37	2281
GGCATTGTAGGTA	0.064	0.014	0.049	57	1105
ACCATCATTAAGG	0.03	0.004	0.026	78	2874
CGCAGCGTTAGGA	0.027	0.005	0.028	27	934
CGCATCATTTATA	0.027	0.005	0.023	65	2776
CCTGTCGTATGCG	0.017	0.001	0.018	18	975
ACCGTTACTAGGG	0.023	0.004	0.017	28	1573
GCCAGCATATGTG	0.051	0.002	0.054	83	1446
AGCGTCGCAAATG	0.017	0.003	0.018	32	1727
AGTGTCGCTTAGA	0.026	0.001	0.027	20	713
CCTATCGCTGATA	0.023	0.001	0.023	49	2088
AGCATTATAAAGA	0.022	0	0.022	112	4876
AGTAGCGTAGATA	0.027	0.003	0.023	32	1351
CGCGTTGTATACG	0.025	0.008	0.016	21	1264
CGCATTGCAGGGA	0.024	0.004	0.029	30	996
AGTGTCACATACA	0.031	0.005	0.028	51	1789
CGTAGCGCAAGCG	0.03	0.008	0.021	17	775
ACTAGCATTTACG	0.026	0.001	0.025	46	1798
CCTAGTACTAGGA	0.025	0.004	0.023	35	1520
GGCATTATAGGCA	0.061	0.009	0.054	106	1856
GGCGGTGTAAGGA	0.069	0.02	0.06	45	710
AGTGGCGTAGGTA	0.031	0.008	0.02	12	585
AGCGTCGCATGTG	0.017	0.003	0.015	15	1013
GGTAGCACAAGGG	0.078	0.014	0.06	31	485
GCCAGTATTTGTA	0.051	0.008	0.04	81	1921
CGTATTGTTTAGA	0.028	0.003	0.031	33	1037
CGCAGCGCATATG	0.027	0.006	0.019	23	1198
ACTGGTGTAGGTG	0.025	0.002	0.024	15	615

GGCGTCGTTAATA	0.069	0.007	0.066	89	1254
GCCAGTATTAGTA	0.056	0.004	0.053	118	2108
GCTATTATTTGGA	0.059	0.005	0.065	83	1197
AGTATTACTGACG	0.025	0.002	0.027	42	1527
ACTAGCATTAAGG	0.025	0.005	0.03	52	1673
GCCATCACTTGCA	0.048	0.002	0.047	94	1895
GCTAGTACTAGGA	0.059	0.005	0.061	59	916
AGCGTCATTTATA	0.02	0.001	0.022	53	2412
GGCAGCACATATG	0.057	0.005	0.061	82	1265
GGCGGCGCAGATA	0.103	0.008	0.105	106	902
GCTGTTATTAATG	0.073	0.007	0.063	79	1169
CCTGGCGTAAGGA	0.019	0.003	0.02	24	1171
CCCATTGCTGAGA	0.027	0.003	0.024	51	2044
GCTGTCGTATGGG	0.064	0	0.063	27	401
ACTGGTACAGGGA	0.025	0.007	0.024	26	1072
AGTATTACAAGGG	0.024	0.003	0.023	28	1166
ACCGTTGCAAGTA	0.02	0.002	0.019	48	2511
GCCGGCGTTAGTA	0.842	0.039	0.833	1358	272
GGTAGTGCTAATA	0.061	0.003	0.058	63	1029
CCTGGTGTTGAGG	0.016	0.005	0.01	8	757
GCCAGCATATAGG	0.058	0.004	0.053	80	1423
GGCGTTACATAGA	0.059	0.011	0.058	79	1293
ACCATTGTTGAGG	0.025	0.005	0.019	30	1557
CCCGGTACTAGCG	0.028	0.003	0.031	48	1488
CCTAGTATTGGGG	0.04	0.007	0.043	31	695
AGCGTCGTAAGGG	0.024	0.007	0.016	16	992
CCCATCACTTAGA	0.031	0.004	0.027	74	2712
CCTGTTGCATACA	0.024	0.005	0.017	39	2274
GGCGTCGCTTAGA	0.065	0.011	0.051	40	745
CCTATTGTAAGCG	0.022	0.002	0.024	49	1994
CCCATTGTTAATA	0.026	0.003	0.024	84	3476
AGCGTTGCTAGGG	0.022	0.007	0.017	13	768
GCTAGCGCAGAGG	0.06	0.006	0.053	25	451
GGCATTACTAGTG	0.056	0.009	0.054	64	1118
CGTAGTATTGGCG	0.032	0.006	0.025	16	634
AGCGGCACAGACG	0.024	0.007	0.015	18	1180
ACTATTATTAAGG	0.031	0.005	0.032	83	2478
CCTATCACTTGGG	0.036	0.003	0.034	35	1008
CGCAGCGTAAAGG	0.028	0	0.028	37	1264
GGTGTTATAAGGG	0.091	0.02	0.065	36	517

ACTAGCATAAGGG	0.03	0.005	0.033	39	1133
CCCAGTGTTGGCG	0.024	0.007	0.016	23	1428
GCTGGTACTAGCG	0.111	0.005	0.111	64	511
CGCATCACATGTA	0.025	0	0.026	53	2024
CCCGGTGCAAGTG	0.027	0.006	0.032	37	1117
GCCATCACTTACG	0.054	0.002	0.054	109	1906
GCCATTATAGGTG	0.058	0.005	0.063	100	1482
AGCATTACATATG	0.023	0.004	0.018	51	2851
AGTATCGTAGGGA	0.023	0.008	0.014	14	991
GGTAGTGCAGAGG	0.051	0.022	0.05	24	455
AGCGGCGTAGGTA	0.019	0.006	0.021	21	964
CCTGGTGTTAAGA	0.027	0.001	0.026	32	1208
CGCGTCACAGAGA	0.022	0.004	0.023	35	1498
AGTATTATATGCG	0.027	0.003	0.024	42	1688
CCTGGTGTTTATA	0.026	0.003	0.027	36	1322
AGTGTTATAGGCA	0.02	0.003	0.019	24	1254
CGCATCGCTGGGA	0.022	0.005	0.015	14	894
GGTATCATTTGTG	0.121	0.009	0.115	75	575
CGTATTATTTAGG	0.029	0.006	0.023	27	1150
ACTGTCATAGGCA	0.03	0.006	0.022	38	1694
AGTAGTGTATACA	0.022	0.005	0.016	29	1815
GCCGGTATATGTA	0.07	0.005	0.063	107	1579
CGCGGTACATGGA	0.024	0.001	0.024	25	1029
CCCGTTATTGATG	0.027	0.005	0.025	46	1797
AGCGTCATTGACA	0.025	0.005	0.018	47	2558
CGCAGCATATGCA	0.024	0.001	0.025	53	2092
ACTGGCACAGAGG	0.03	0.003	0.026	24	917
AGTAGTATTGATA	0.024	0.001	0.023	47	2013
GGCATTATTAAGA	0.057	0.005	0.054	126	2204
CGTAGTATTAACG	0.032	0.002	0.028	36	1230
CGCAGTATAGGTA	0.029	0.005	0.033	60	1762
CCCGGTGTTGAGG	0.016	0.004	0.01	9	891
CCCGTTACTAGTA	0.031	0	0.031	68	2101
CGTGTCGCAAACA	0.029	0.008	0.02	34	1674
CGTGTTATAAACG	0.029	0.002	0.026	45	1668
ACTGTTGCAAATA	0.024	0.002	0.025	77	2995
GGCAGCATATGGA	0.048	0.009	0.047	58	1182
CCTGTCGCAAGCG	0.023	0.001	0.023	28	1212
CCTGGCATAGACA	0.027	0.004	0.033	71	2104
GGCGTTGTTAATA	0.065	0.008	0.075	113	1397

ACCAGCATTAATG	0.027	0.004	0.028	91	3159
GGCATCACATACA	0.072	0.013	0.057	151	2509
CCTAGTACAGGTG	0.031	0.002	0.029	36	1186
GGTGTTGTATAGA	0.084	0.004	0.089	62	633
CGTGTTACATACA	0.026	0.003	0.026	44	1669
AGTATTATTGACG	0.023	0.006	0.029	51	1728
GCTATTGCTTGCG	0.054	0.015	0.035	27	737
ACTATCGTTAGCG	0.029	0.004	0.023	31	1289
ACCAGCGTTAACA	0.026	0.003	0.022	73	3219
CGCGGCATTGGGG	0.037	0.009	0.025	12	464
ACTATTATTGATA	0.028	0.003	0.026	101	3771
ACTATTGCTGGTG	0.022	0.003	0.026	33	1243
CGTAGTACTAGTA	0.022	0.001	0.02	23	1130
GCTGGTGTAAACA	0.423	0.026	0.402	703	1047
GCTATCATATATG	0.065	0.006	0.064	117	1704
GCCATCATAGGTA	0.057	0.005	0.05	106	1996
GGTATCATATATA	0.13	0.012	0.121	225	1636
CGCATTGCTTGTA	0.025	0.003	0.021	28	1283
GCCATCACTTACA	0.045	0.003	0.044	127	2733
GCCATTATTTACA	0.057	0.006	0.052	172	3157
GGTATTGTAGACA	0.093	0.022	0.065	86	1232
GGTGGTATTTGCA	0.127	0.003	0.131	86	571
ACCATCGTAAACA	0.023	0.001	0.022	105	4690
CGCGGTGTAAGGG	0.019	0.001	0.02	13	633
CCTATTATAAGGA	0.032	0.007	0.026	70	2628
ACTGTCGTATATA	0.024	0.001	0.024	48	1936
AGCAGCGTTAAGA	0.023	0.002	0.024	45	1851
CCTGTCATAGAGG	0.026	0.003	0.021	24	1121
GCTGTCGTTTATA	0.044	0.007	0.042	40	921
GGCATTGTTTGTA	0.068	0.01	0.06	69	1089
CCCGTCACTGACG	0.035	0.005	0.027	57	2049
CGTAGCACTTAGG	0.022	0.011	0.017	11	642
GCCATTATATGGG	0.056	0.005	0.059	77	1229
AGTATCATTTGTG	0.022	0.003	0.02	23	1126
GCTATCGCTGGGA	0.053	0.009	0.06	34	532
CCTATCGCATGCG	0.024	0.007	0.02	30	1458
ACTGTTATAAATA	0.027	0.005	0.021	87	3989
CCCATTACTAATG	0.028	0.002	0.027	83	2997
ACTAGCGTAAAGG	0.026	0.003	0.026	34	1264
GCCGTCGCTAGCG	0.049	0.007	0.058	43	701

AGTATTGCAAACA	0.026	0.001	0.028	70	2468
ACTAGTACAGAGA	0.021	0.002	0.022	59	2683
ACCAGTGTTAGCA	0.025	0.002	0.025	58	2295
ACTGTTGCTTATG	0.022	0.004	0.019	25	1275
CGTATTACATGTG	0.031	0.011	0.017	20	1137
GCTATTGTTTGTG	0.058	0.01	0.047	37	745
ACTATTGTAAGGA	0.027	0.004	0.024	51	2105
GCCATCGCTTGCA	0.051	0.008	0.056	72	1217
AGCATTGTTGGCA	0.027	0.002	0.029	51	1701
CCCAGCACATATG	0.03	0.005	0.034	86	2410
ACCGGCATTTGGA	0.025	0.005	0.018	24	1275
AGTGGCGCAGGGA	0.02	0.003	0.018	9	482
AGTATTGCTAACA	0.025	0.003	0.028	60	2070
CCCAGCGTATGCG	0.025	0.006	0.018	31	1690
GCCGGTATAAGGG	0.147	0.022	0.158	147	783
AGTGTCACATGTG	0.02	0.006	0.022	18	793
CCCGTTACAAATG	0.024	0.004	0.022	66	2908
CGCATTGCAAATA	0.021	0.002	0.019	53	2781
GCTATCACAAGCG	0.068	0.009	0.056	70	1177
GCTAGTGTAGAGG	0.058	0.004	0.059	47	743
CCCAGTACATGCA	0.022	0.003	0.018	59	3188
CCTGGTATTTGCA	0.026	0.005	0.028	36	1231
GGTGGTACAAAGA	0.079	0.02	0.054	54	938
GGTAGTACTTGCA	0.075	0.013	0.057	42	694
CCTGGTATATGGA	0.027	0.004	0.03	35	1130
GCTATTGCAGGTG	0.055	0.008	0.049	39	751
CGTATCGTTAGGA	0.022	0.002	0.019	17	889
GCCAGCGCAAACG	0.061	0.01	0.047	69	1410
AGCGGTGTTAAGG	0.022	0.005	0.02	19	936
GGCGGCGTAGACG	0.448	0.051	0.429	436	580
CCTATCGCTTAGA	0.029	0.008	0.018	31	1652
GGTATCACAGGGG	0.114	0.025	0.12	61	446
GCCGTTGTTGATA	0.051	0.007	0.048	64	1272
GGCATTATAGACA	0.059	0.008	0.056	152	2548
GCTATTACATGTG	0.059	0.001	0.057	66	1083
CCCGGTACTGACA	0.028	0.001	0.028	72	2496
CCTGGTACTGGGA	0.025	0.008	0.02	16	772
CCTATTGTTTATA	0.028	0.002	0.026	65	2468
GCTGTTGCATGGA	0.068	0.006	0.073	46	582
CCTGGCATAGAGG	0.03	0.007	0.025	21	812

GGCATTATTAAGG	0.063	0.007	0.065	95	1375
GGTAGCGTAGGTA	0.088	0.019	0.097	59	547
GCTATTACTGGTG	0.054	0.009	0.044	39	840
AGCGGTACAAATG	0.016	0.002	0.015	31	2051
AGCATCGCATGTG	0.025	0.003	0.022	32	1449
AGTATTGTAAATG	0.026	0.003	0.022	48	2163
GGCAGTACAGACG	0.062	0.006	0.055	76	1318
CGCAGCGTATGCG	0.023	0.004	0.019	20	1051
CGCATTGTTAAGG	0.025	0.001	0.025	33	1285
GCCAGCGCTGGGG	0.058	0.009	0.046	20	418
AGTATCACTAGTG	0.029	0.001	0.028	30	1044
AGTGTCGCTTATA	0.02	0.002	0.022	22	988
CCCAGCGTAAACA	0.023	0.002	0.021	90	4228
GCTAGTACATATG	0.065	0.007	0.057	77	1266
ACTGTCGCAGGTA	0.021	0.003	0.025	33	1298
AGTATTACAAACG	0.023	0.004	0.021	52	2433
CCCAGTGTTGACG	0.024	0.003	0.024	46	1841
GGTGGCGCAGACG	0.3	0.029	0.277	172	448
AGTGTCATAAAGG	0.023	0.003	0.022	28	1236
GCCGTCGTATAGG	0.05	0.002	0.048	32	638
GGTATCACATGCA	0.105	0.018	0.083	94	1043
AGTATTATTGACA	0.021	0.001	0.02	53	2542
AGCATCGCTGATA	0.022	0.003	0.023	51	2192
GGCGTCGCTTGGA	0.065	0.008	0.054	34	596
GGCGGCACTAATG	0.272	0.038	0.23	287	960
GGTAGTACTGGGA	0.066	0.019	0.043	24	530
CGCAGCACTAAGG	0.025	0.004	0.022	28	1243
GCTATTGTTTAGA	0.053	0.007	0.047	60	1207
GCTAGCGTAAGCG	0.08	0.007	0.071	61	801
AGCGGCACAAGGG	0.016	0.003	0.017	13	746
AGCGTTATTAACG	0.022	0.001	0.02	44	2110
CGTGGCGCTTACA	0.03	0.01	0.022	19	834
ACCGGTATTTGTA	0.024	0.004	0.02	42	2027
AGCATCGTTAAGA	0.024	0.003	0.02	50	2459
AGTGGTACAAAGA	0.022	0.004	0.023	37	1564
AGCGTTGTTTATA	0.018	0.005	0.02	34	1633
CCTGTTGTTGGCG	0.024	0.001	0.025	25	960
GCTATTGTTTATA	0.055	0.005	0.047	83	1682
CGCAGTGTTGGCG	0.023	0.004	0.018	15	821
AGCGTTATTAAGG	0.02	0.001	0.02	31	1525

GCCGGTACATGGG	0.063	0.013	0.075	53	652
AGTATCATTAGGA	0.031	0.003	0.026	37	1372
ACCATTGTTGGGG	0.028	0.001	0.027	29	1027
ACTGGTGCAAATG	0.032	0.007	0.034	49	1409
GCTATCATTAATG	0.075	0.009	0.066	125	1763
GGCGTCATTAAGA	0.073	0.013	0.055	78	1342
GCTGTTACATGGG	0.051	0.008	0.054	32	562
GCTATCATTTGGG	0.067	0.008	0.062	53	805
CGTATTATTAATG	0.032	0.003	0.033	61	1809
ACCGGTACTGACG	0.022	0.002	0.024	55	2221
AGCGTCGTTGGCG	0.02	0.008	0.02	21	1013
GCCGTCACTAGGA	0.056	0.005	0.062	75	1130
CCTGTTATTAAGA	0.024	0.005	0.022	45	2012
AGCATCATTAATA	0.023	0.002	0.024	109	4471
ACTAGCATAAGTA	0.025	0.003	0.029	85	2881
AGCATTGCAAGCG	0.027	0.005	0.029	54	1840
CCTATTACAGAGA	0.025	0.002	0.027	70	2507
CCTAGTATTAACA	0.026	0.006	0.021	71	3337
ACCGTCATTTATG	0.024	0.002	0.025	52	2038
ACTATCACTTGCA	0.02	0.002	0.021	56	2638
CCTGTTATATGTG	0.025	0.006	0.021	29	1327
ACCAGCGCTAAGG	0.025	0.003	0.025	40	1578
CCCGGTGTTAATG	0.022	0.002	0.024	36	1452
ACCGTCATAAGGG	0.023	0.003	0.02	27	1295
CCTAGTGCAGGTG	0.016	0.005	0.017	18	1019
AGCGTTATAAGGG	0.023	0.007	0.018	24	1281
CCCATCACAAATG	0.027	0.003	0.025	98	3851
GCTAGCACTAATG	0.058	0.009	0.052	63	1155
CGTATCGCTTGGG	0.034	0.007	0.032	15	453
AGTGGCGTAAAGA	0.022	0.004	0.018	17	929
ACTATCGTTGACA	0.023	0.003	0.023	55	2330
GCTGTCATTGACG	0.063	0.005	0.056	63	1065
ACTGGCACTGGGG	0.033	0.008	0.043	23	515
AGTATTGTATACG	0.02	0.002	0.023	36	1539
ACTAGCGTATAGA	0.022	0.005	0.015	25	1650
CGTGGTATATAGG	0.026	0.009	0.02	13	646
CCTATTATTGATA	0.021	0.001	0.02	70	3361
ACTAGTATTTGTG	0.026	0.004	0.03	42	1361
ACTGGCACAAGCA	0.023	0.003	0.026	56	2105
CGCAGTATAAGTG	0.026	0.003	0.03	50	1643

GCTAGCACATGGG	0.07	0.014	0.051	34	637
CGCGTCATATGTG	0.028	0.008	0.026	31	1154
CCTGGTACAGACA	0.023	0.002	0.02	46	2282
ACTGGCACTAACG	0.031	0.004	0.034	53	1529
AGCGGCACTAAGA	0.022	0.005	0.018	28	1567
ACCATCACTGGCG	0.028	0.003	0.025	50	1956
CGTGTTGCTAGGA	0.032	0.005	0.034	20	572
GGCATTGTATGGA	0.07	0.01	0.058	64	1040
CGTAGTGCTTGTG	0.024	0.004	0.028	14	488
GCCATTGCATGGG	0.043	0.011	0.045	32	680
GCTGTTACTTAGA	0.059	0.001	0.06	59	932
GGCATCATTAGTA	0.076	0.008	0.073	136	1725
ACTATTACTAGGA	0.024	0.004	0.018	46	2451
AGTAGCATAGGCG	0.024	0.011	0.04	40	959
AGTGTTGCATATG	0.022	0.005	0.016	14	840
CCTGTTATAAGTA	0.028	0.001	0.029	67	2252
CGCGGCGCTGACA	0.02	0.001	0.018	26	1442
GGTGTCATTTACA	0.094	0.013	0.099	94	860
ACCAGCGTATACG	0.03	0.005	0.023	45	1888
ACCGGTGCTTACA	0.023	0.001	0.021	49	2261
AGTGTCATAGAGA	0.024	0.005	0.018	24	1284
ACCATTATTGGGG	0.029	0.005	0.028	33	1144
GCTGGTATTTGGA	0.156	0.018	0.131	98	651
AGCGTTATAGGTG	0.02	0.007	0.021	26	1186
ACCGGTACAGGCG	0.025	0.003	0.023	45	1894
GGCATCATTTGCA	0.087	0.011	0.085	130	1408
CGTGTCATTGATA	0.021	0.006	0.019	26	1328
ACCAGCGTTAGGG	0.023	0.004	0.017	16	933
ACTGGTGCATATA	0.024	0.002	0.025	51	1985
CCTAGCATTAATA	0.025	0.001	0.024	72	2903
AGCAGTATAAACA	0.023	0.003	0.021	121	5667
GGCGTTGCAGAGG	0.048	0.012	0.035	22	601
CCTATCATATAGG	0.031	0.006	0.033	68	2006
CCCGTCGCTGAGG	0.023	0.003	0.027	28	1004
GCCATTGCTTAGG	0.052	0.006	0.054	48	839
GCTGTTGCTAACA	0.056	0.008	0.046	56	1152
CCTATCATTTGCG	0.028	0.003	0.026	46	1705
CCTATTACAGGTG	0.032	0.011	0.018	27	1513
CGTATTGCTTGCA	0.023	0.002	0.021	22	1047
CGTATTGCTTAGG	0.025	0.003	0.025	15	584

AGTAGCACTAACG	0.028	0.001	0.027	41	1451
CGCATCACTAGGG	0.031	0.001	0.031	33	1041
CGCGGTGCTTGTG	0.022	0.005	0.018	11	614
GGCAGTATTAGGG	0.049	0.006	0.044	36	788
AGTATCGTTTACA	0.026	0.001	0.025	43	1667
ACCAGTGTATAGG	0.029	0.005	0.022	35	1532
CGCGGTACTAGGA	0.023	0.005	0.015	16	1017
AGCGGCACTAGGA	0.025	0.006	0.019	20	1033
ACTAGCACATAGA	0.023	0.007	0.014	39	2686
ACTGTTGCATAGA	0.023	0.003	0.024	43	1780
AGTATCGTAGGCG	0.022	0.002	0.023	24	1002
CCCGGCGTAGGCA	0.022	0.003	0.026	47	1735
GGCGGTGCAGAGA	0.056	0.016	0.036	26	688
AGCAGTATAGACG	0.022	0.002	0.022	54	2413
ACCATCACTTACG	0.024	0.003	0.02	72	3610
CCTAGTATATGGG	0.025	0.006	0.019	20	1035
GCTAGTGCTAATA	0.052	0.01	0.05	78	1487
GCTATCACTTATG	0.066	0.011	0.051	67	1239
CCTGGCGCAAATA	0.022	0.004	0.019	38	1998
GCTAGCATTTGGG	0.076	0.01	0.062	35	527
ACCGTTGTATACA	0.024	0.004	0.019	54	2755
ACCATCGTTGGTA	0.025	0.002	0.024	43	1736
GGCAGCATTAACG	0.046	0.001	0.048	82	1624
CCTAGCGTAAATG	0.025	0.005	0.031	60	1868
GCCAGCATTTATG	0.056	0.004	0.05	91	1725
CGTGTCATATGCA	0.021	0.002	0.023	29	1241
CGTGGCACAGGCG	0.023	0.002	0.021	12	558
GCCATTATAGATG	0.058	0.005	0.051	125	2350
GCTATCGTTGAGG	0.07	0.017	0.078	54	639
CGCAGTGTAGATG	0.019	0.003	0.019	24	1252
GCCATCGCTAGCA	0.045	0.006	0.052	79	1448
GGCAGCATAGGTA	0.051	0.008	0.062	83	1266
GCTGGCACTAACG	0.435	0.049	0.394	486	749
ACTGGTATTTACA	0.025	0.002	0.024	50	2070
CGCAGTGTAGACG	0.02	0.005	0.013	18	1405
CGTGGCGCTTGCA	0.023	0.005	0.019	11	578
GGTAGTACTGGTG	0.072	0.003	0.072	36	462
CGCAGCGTTGACA	0.023	0.003	0.021	39	1777
CCCAGCATATGCA	0.026	0.005	0.033	103	3026
CGCAGCGCTAAGA	0.023	0.003	0.024	36	1445

CCCATTGTTAACG	0.022	0.003	0.023	61	2541
GGTGTTACTAGCA	0.069	0.004	0.074	66	829
ACCAGTGCTGGTA	0.021	0.003	0.019	39	1981
GCCGTCACAGGGA	0.052	0.001	0.053	56	1008
GGTGGTGTAGATA	0.12	0.021	0.107	82	687
ACTGTTATTGATG	0.021	0.006	0.015	23	1551
AGCGGCATTTACA	0.022	0.006	0.018	28	1572
ACTATTGTTTATA	0.026	0.004	0.026	74	2814
AGCATTGTAAATG	0.021	0.004	0.016	47	2922
GGCAGTATAGACA	0.054	0.007	0.049	116	2240
CCTGTTGCAGACG	0.03	0.003	0.031	45	1424
GCCATTATAAATA	0.056	0.002	0.053	288	5186
CCTGGCACTGGCA	0.027	0.007	0.017	22	1274
CGTGTTGCATGCA	0.032	0.009	0.027	26	940
GGCAGTATAAGCA	0.054	0.006	0.053	117	2109
AGTGTCATAAGGA	0.025	0.004	0.03	37	1195
GGTAGTGCTTGCA	0.061	0.007	0.069	43	584
GCTAGTGTATATA	0.055	0.006	0.063	107	1582
CGCGTCGTTGATA	0.023	0.002	0.022	32	1394
AGTAGCACAAAGG	0.018	0.002	0.019	22	1134
GCCGGCGCTTGGA	0.486	0.043	0.495	359	366
CCTGTCGCTAGCG	0.031	0.004	0.029	28	925
GGCGGCATTAGCG	0.547	0.071	0.512	580	553
ACCATCGCAGGTG	0.023	0.005	0.019	31	1620
GGCGGCACTGACG	0.256	0.034	0.219	253	902
AGCGGTACATATG	0.018	0.005	0.024	36	1445
GGCGGTATTAATA	0.072	0.006	0.063	108	1601
AGTATCGCTAATG	0.026	0.005	0.019	25	1307
AGCGGCACATACA	0.021	0.001	0.02	45	2165
CCTATCACTAAGG	0.029	0.003	0.025	48	1892
GCCAGTGCAGGTG	0.045	0.005	0.043	33	735
AGTGTCGCAGATA	0.021	0.005	0.028	33	1164
CCCAGCGCTTATG	0.03	0.004	0.034	55	1575
CGCAGCGCAAGCG	0.027	0.006	0.034	43	1237
CCCATTGTTTGGG	0.025	0.007	0.015	15	1000
AGCGTTATAGATA	0.022	0.003	0.018	52	2827
ACTATTGTTAGGG	0.028	0.008	0.019	23	1191
GCTGTCATAGGTA	0.069	0.005	0.064	65	945
CGTATTGTTTGGA	0.031	0.008	0.023	18	758
CGTATCGTTAAGA	0.023	0.002	0.021	31	1458

CCCAGTGCTGAGA	0.023	0.005	0.021	36	1692
GCTGGTACAAATA	0.074	0.007	0.065	121	1733
AGTAGCGTAGGTA	0.028	0.007	0.02	19	923
GCTGGTACATGTA	0.075	0.002	0.072	75	969
AGTATCACATGGG	0.029	0.004	0.023	18	779
ACCATTGCTAGTA	0.021	0.001	0.02	59	2862
GGTATCGCTTATA	0.111	0.016	0.091	92	923
GCTGGCATATGGG	0.829	0.034	0.811	750	175
ACCAGTGTTAGTG	0.027	0.005	0.021	31	1450
AGTAGCGCTGGCA	0.027	0.004	0.028	25	853
AGTATCGCTAGCA	0.026	0.002	0.024	32	1326
AGTAGCGCATACA	0.023	0.003	0.025	37	1441
CGTGTCGTTTGTA	0.025	0.011	0.01	7	711
AGCAGTGTATGCG	0.02	0.004	0.016	23	1448
AGCATCACTGATG	0.02	0.003	0.017	37	2132
ACCGGTGTAGAGG	0.028	0.006	0.02	24	1150
AGTGTTATATGCG	0.029	0.003	0.027	30	1061
GGTAGTATATACG	0.07	0.017	0.047	50	1020
GCTGGTGTTGACA	0.396	0.047	0.392	446	693
GGCGGTGCTGGCG	0.075	0.013	0.082	47	526
ACCGGTACTGGGA	0.024	0.003	0.028	40	1373
GCCGTCGCATACA	0.049	0.003	0.051	82	1526
ACCAGCGTTTAGG	0.026	0.004	0.021	24	1134
AGTAGTGTTAACA	0.021	0.003	0.017	33	1864
AGCGTTGCATACA	0.021	0.004	0.027	59	2162
GCTATTGTATAGA	0.051	0.007	0.059	90	1447
CCCGGCATTTAGG	0.022	0.003	0.023	27	1139
CGTATCGCTAGGA	0.025	0.005	0.019	18	931
CGCAGTGCTTATG	0.026	0.009	0.038	39	985
ACTATCGCTTACG	0.026	0.003	0.022	38	1683
GGCATCACATATG	0.087	0.007	0.082	140	1569
ACCGGCACAGACA	0.021	0.002	0.021	76	3562
CGTAGTATTGATG	0.029	0.006	0.026	26	961
ACCAGTATAAGCA	0.021	0.001	0.022	100	4455
ACTGGTATAGACA	0.027	0.006	0.023	55	2381
CGTATCATTTATG	0.024	0.007	0.033	48	1420
GGCGGCGTTGGCA	0.216	0.033	0.19	151	642
CCCGTTGTTTGGG	0.027	0.003	0.023	17	736
GGTAGTATTAACG	0.057	0.002	0.059	67	1072
CCCAGTGCAAGGA	0.025	0.002	0.024	46	1832

GGCGTCGTATAGA	0.074	0.017	0.051	47	866
AGCGTTATAGAGG	0.018	0.005	0.012	17	1372
CCTGTCACATGTG	0.021	0.005	0.014	16	1096
GGTGGTACTTGGA	0.078	0.012	0.077	35	422
GCCGTCACAGAGA	0.049	0.003	0.052	81	1485
AGCATTATAGAGG	0.018	0.004	0.024	47	1941
CGCATTGTATGCA	0.024	0.008	0.014	27	1971
GCTAGCACTTGCA	0.068	0.008	0.057	65	1072
GCCAGTGCATAGA	0.046	0.004	0.051	68	1255
AGCATCACATGGA	0.02	0	0.02	41	2012
CGCAGTACATGTA	0.021	0.001	0.022	42	1846
CGCGGTGTTAATG	0.028	0.009	0.028	28	980
CGTGGCGTAGACA	0.026	0.005	0.03	29	938
ACCAGCGCTGACG	0.029	0.002	0.032	66	2018
GCCAGTGTTAAGA	0.042	0.007	0.031	51	1580
CGCAGCATAAACA	0.022	0.001	0.021	81	3695
GCCGTCGTTTGGG	0.054	0.009	0.053	23	407
GGCGTTACAAATG	0.063	0.011	0.048	69	1372
ACCATTGCTGAGA	0.023	0.004	0.02	48	2368
ACCATTATAGATA	0.022	0.002	0.024	132	5307
CGTAGCACTAGTA	0.025	0.005	0.025	28	1102
GCTATCACAGAGG	0.053	0.013	0.037	40	1033
CGCGTTGCTAGTG	0.026	0.005	0.031	25	779
GCTAGTGTATGGG	0.064	0.01	0.07	43	567
CGTGTCGTAAATA	0.023	0.003	0.025	42	1647
CCCGTCATATACA	0.024	0.003	0.02	77	3703
CCTGTCGCTAGCA	0.026	0.002	0.026	38	1397
CGTATTGTTGACA	0.028	0.005	0.022	32	1454
CCTGGCGTAAATA	0.024	0.003	0.025	57	2221
CGCAGCGTTGATG	0.029	0.002	0.029	32	1086
CGCAGCACTAATG	0.022	0.006	0.019	32	1635
ACTATTGTTAGTG	0.027	0.005	0.026	41	1561
GGCAGCATTTACA	0.051	0.006	0.047	90	1820
ACCGGTACTAGTA	0.018	0.004	0.021	58	2675
GCCGGTGCTGACA	0.057	0.006	0.059	69	1104
CCTAGTATATGTA	0.026	0.002	0.029	62	2101
AGCGGTACAGGTG	0.018	0.001	0.019	19	970
CCCGTTGTAGGGA	0.024	0.004	0.026	29	1106
GGTAGCGCTGGGA	0.077	0.027	0.115	38	293
AGCGGCGCAAGCA	0.027	0.007	0.028	41	1448

AGCGGCACAAGTG	0.023	0.006	0.014	15	1025
GGCGTCACATAGA	0.062	0.005	0.06	81	1265
CGTGTTATTTAGA	0.026	0.005	0.019	21	1107
CCCAGTATTAGGA	0.025	0.006	0.017	36	2090
AGCAGCGTAAGCG	0.021	0.003	0.018	28	1509
CCTGGCACTAGTA	0.026	0.002	0.027	39	1402
GGTATTGTAAATG	0.107	0.017	0.087	106	1115
GCTATCATTGGTA	0.056	0.01	0.05	72	1371
ACCGGTATAGAGG	0.021	0.004	0.016	22	1331
ACCGTCACTGATG	0.03	0.002	0.033	70	2024
ACCATTGTTGGTA	0.024	0.005	0.022	43	1875
CGCATTATATATG	0.025	0.002	0.026	61	2278
CGTATCGCAGGCA	0.023	0.008	0.026	30	1116
GGTGGCGCATACA	0.162	0.024	0.15	130	734
GCCATTACATAGA	0.049	0.001	0.05	107	2047
GCTGGTATTTGTA	0.16	0.023	0.149	143	816
GCCATTGTTGGCG	0.047	0.007	0.051	67	1243
GGTAGTGCAGGTG	0.058	0.004	0.057	25	417
GCCGTTATATGGA	0.052	0.007	0.042	55	1248
ACCGGCATAGATA	0.025	0.003	0.029	84	2815
AGCGTTGTATGCA	0.023	0.003	0.028	44	1551
AGCGTTGCTGACG	0.021	0.004	0.026	33	1214
AGCATCGTAAGTA	0.021	0.001	0.023	61	2649
AGCATCATTAGTA	0.021	0.001	0.019	57	2899
CGTGTCGCATGTG	0.018	0.002	0.021	13	612
GCCAGCGTTAATA	0.048	0.007	0.042	85	1953
CCTAGCGTTAAGA	0.025	0.007	0.022	37	1619
GGCATTACTAACG	0.058	0.009	0.064	124	1812
AGCGTCGCTTACG	0.022	0.006	0.022	29	1291
GCTATTACAGAGA	0.058	0.008	0.047	74	1494
CCTATCATATATG	0.029	0.005	0.022	62	2743
CGCGGCACTAGCA	0.022	0.002	0.019	28	1423
AGTAGTGCATAGA	0.017	0.004	0.011	12	1067
AGCAGTGCATATG	0.024	0.003	0.025	42	1655
CGTATTACTGACG	0.033	0.008	0.023	32	1377
GCCAGTATTTACA	0.052	0.007	0.042	120	2765
GCCAGTACAGGTA	0.053	0.006	0.049	83	1610
GCTATTGTAGACG	0.065	0.005	0.067	96	1333
GCTGTCACATACG	0.063	0.006	0.055	64	1092
CCCAGTGTAGATG	0.026	0.002	0.023	43	1796

GCTGTTACAGGGG	0.078	0.012	0.075	40	490
AGTAGTACATGTA	0.02	0.002	0.017	27	1521
CCCATTACTAGGG	0.029	0.002	0.026	39	1433
GGTATTATAGGTG	0.098	0.017	0.085	68	728
CCTGTCACAGAGG	0.024	0.006	0.016	16	954
AGCAGTGTAAGCA	0.02	0.002	0.017	44	2472
AGCGTCGTTAAGG	0.021	0.004	0.021	22	1027
AGCGGCACTAGTA	0.022	0.002	0.019	26	1325
CGCATCATTGAGG	0.024	0.004	0.018	24	1274
GGCATCATAGGCG	0.067	0.003	0.069	99	1335
GCTAGTGCAGATA	0.056	0.005	0.048	69	1354
AGTAGTATTGAGA	0.024	0.001	0.023	35	1469
CCTGGTATTTACA	0.025	0.007	0.023	43	1853
CGTGTTACTGGTG	0.014	0.003	0.011	7	650
GGCGTCGTAGATG	0.08	0.02	0.058	49	789
AGCGGCGCTGAGG	0.023	0.012	0.006	4	688
GGTAGTATAGGTG	0.067	0.005	0.061	38	587
ACTGGTGTTTACG	0.023	0.004	0.024	23	945
GCCGGTATAAACG	0.163	0.024	0.145	289	1711
ACCGGCGTTGAGG	0.018	0.005	0.024	13	540
CCCGTTGCAGACA	0.021	0.002	0.018	51	2760
GCTGGCACTTAGG	0.476	0.031	0.477	315	345
AGCATCGTATACA	0.02	0.005	0.017	57	3335
GGCGGTACTTATA	0.061	0.006	0.054	64	1132
AGCAGCATTGGCG	0.025	0.008	0.035	46	1258
CCCAGCACAAACA	0.025	0.002	0.027	152	5452
ACTAGTGCAAATA	0.023	0.002	0.021	88	4049
GCCATTATTGGGA	0.047	0.005	0.055	75	1297
AGCGTCGTTGACG	0.019	0.004	0.019	26	1375
CCCATTGCAAGCG	0.027	0.005	0.023	56	2354
CCTAGCACTGGCG	0.027	0.006	0.026	29	1098
AGCAGCGCATGGA	0.023	0.002	0.025	30	1167
CCCAGTATATAGA	0.025	0.006	0.033	100	2975
CGCAGCGTATGTG	0.025	0.003	0.027	26	942
CCCAGTGCATAGG	0.022	0.008	0.012	17	1397
GGTAGTGCATGTA	0.058	0.012	0.042	30	678
ACTAGTATTAGCA	0.03	0.004	0.025	64	2488
CCTAGTATTGGTG	0.024	0.006	0.028	29	1014
CCTGTTGTATATG	0.033	0.001	0.034	47	1339
AGCGGCGTAAACA	0.023	0.005	0.018	37	2022

AGCGTCGCAAACA	0.022	0.002	0.019	55	2825
AGCAGCGTATACG	0.021	0.002	0.023	44	1910
ACCAGCACATACA	0.021	0.002	0.019	113	5727
GCCAGTATATAGA	0.051	0.007	0.043	107	2360
CGTGGCGTAAGCG	0.024	0.002	0.022	11	498
AGTAGCATTAATG	0.031	0.003	0.03	47	1530
CGCGGCACAAATG	0.025	0.006	0.033	52	1521
GGCATTACATACG	0.062	0.004	0.06	117	1817
AGCGTCATATGGA	0.022	0.003	0.024	39	1562
ACCGTCGTTTGGA	0.026	0.002	0.023	24	1018
GCTAGTGCATAGG	0.058	0.007	0.049	36	704
ACCGTCACAAAGA	0.023	0.004	0.019	78	4062
GGCGGTACTGATG	0.078	0.013	0.063	53	788
CGCGGCATAAGTA	0.022	0.003	0.022	38	1655
CCCGGCACTAGCG	0.025	0.004	0.019	31	1613
ACTAGTATAGGCA	0.022	0.002	0.02	45	2257
CGTAGTGTAAGCG	0.026	0.006	0.022	17	763
CCCGGCACTGACA	0.027	0.001	0.026	72	2684
ACTAGTGCTGGTA	0.022	0.005	0.015	23	1508
GCCGTTATAAGTA	0.056	0.003	0.054	120	2105
ACCAGTGCTTACA	0.024	0.006	0.019	59	3066
AGCGGCGCTTAGG	0.028	0.007	0.019	12	626
GGCAGTACTAGTG	0.057	0.005	0.058	56	914
CGTATTACAAGGG	0.026	0.004	0.025	29	1112
CGTGTCATAGAGG	0.026	0.004	0.02	16	765
GGTAGCATAGATG	0.059	0.013	0.046	39	801
CCTAGCGCATACA	0.023	0.001	0.024	67	2698
CGTGTCGTAGGGA	0.024	0.009	0.026	16	611
GCCATCATAAACA	0.057	0.001	0.055	282	4808
CGCGGCACATACG	0.023	0.006	0.015	22	1401
CCTATTGTAAGGG	0.024	0.005	0.02	30	1447
CCTGGTATTAAGA	0.025	0.002	0.024	41	1702
GGTGTCACTTGCG	0.094	0.011	0.1	45	406
ACTATTGCTGGTA	0.019	0.003	0.014	27	1847
CCCAGCGTAGGTG	0.029	0.006	0.02	24	1149
AGCATCGTAAAGG	0.024	0.003	0.028	60	2112
AGTATCGTTAGGG	0.026	0.001	0.026	19	716
AGCGTCATTGGGA	0.022	0.003	0.026	29	1094
GCCAGTGCATGTG	0.047	0.01	0.037	30	788
CGCAGTGCTTGTA	0.024	0.003	0.022	25	1097

CGCATTGCATGGA	0.027	0.005	0.033	40	1175
GGTGTTATTTACA	0.08	0.016	0.067	75	1043
AGTGTTGTAGACA	0.021	0.005	0.023	35	1458
CCTAGTATTTGTA	0.026	0.003	0.03	50	1607
AGCATCATTTGCA	0.019	0.002	0.022	60	2688
CGCATCATAAGCA	0.029	0	0.029	89	2986
AGTGGTATATATA	0.025	0.001	0.023	43	1809
GCCATCACAGAGG	0.053	0.001	0.052	76	1398
ACCGTTATAGAGA	0.029	0.004	0.026	66	2440
GGTAGTGCAAAGG	0.05	0.009	0.05	30	565
GGCGGTGTAAATG	0.093	0.016	0.076	85	1032
CCCGGTATTTACG	0.024	0.004	0.021	38	1764
AGTATTACTAGGG	0.028	0.003	0.024	26	1036
GCCGGCATTGACG	0.688	0.072	0.722	1261	485
CCTGTCGTTGGCG	0.021	0.004	0.023	19	810
ACCATTGCAGACG	0.026	0.004	0.028	86	2967
GGTATCACTAGTA	0.102	0.015	0.081	86	975
GCTAGTACTTACA	0.066	0.005	0.068	109	1492
CGTGTTATTTACG	0.02	0.004	0.016	18	1088
AGTATCGTATGCA	0.027	0.007	0.019	33	1685
AGCGTCGTTAGGG	0.031	0.004	0.036	26	694
GCTGGTATAAACA	0.16	0.021	0.157	339	1827
GGTGTTACATATA	0.082	0.005	0.075	100	1240
AGCATTACTGACA	0.023	0.002	0.025	86	3400
AGTATCGTTTATA	0.02	0.003	0.016	30	1836
ACTATTGTTTAGG	0.031	0.007	0.022	29	1304
CGTGTCATTGATG	0.031	0.005	0.028	25	882
CCCAGTGTAAGTA	0.027	0.002	0.026	68	2499
ACCGGTACAGATG	0.02	0.001	0.019	44	2264
GGCATTATTAATG	0.069	0.008	0.062	126	1897
ACTGTCGTTTAGG	0.033	0.003	0.035	24	661
AGCAGCGTTGATA	0.02	0.005	0.013	26	1906
AGTGGTATAGAGG	0.018	0.005	0.019	12	617
CGTGTCACTGACA	0.025	0.001	0.025	34	1341
GCTAGTATTGAGA	0.058	0.004	0.053	70	1254
AGTATCGTTTATG	0.024	0.005	0.017	19	1115
CGTGGTACTAACA	0.024	0.009	0.013	20	1478
ACTATTGTTGATA	0.025	0.004	0.03	79	2527
GCCATCATTTGGG	0.055	0.01	0.064	52	755
AGTATCGTTAGGA	0.027	0.001	0.026	25	950

GCTATCATTTGCA	0.058	0.01	0.044	65	1411
ACTATCACTTGGG	0.021	0.001	0.019	22	1113
CCTAGTGCAAATG	0.024	0.005	0.017	35	2006
AGCAGTGTAAACA	0.023	0.002	0.022	81	3597
CCTAGCACAAGGG	0.035	0.008	0.027	33	1190
CGTAGTACATGCA	0.026	0.002	0.023	29	1227
ACTGGCATTTAGA	0.027	0.001	0.028	42	1436
GCCATTGCTGGTG	0.057	0.002	0.06	47	739
GCCGTTGCAAACA	0.044	0.006	0.04	81	1959
ACTATCATTAGTG	0.027	0.003	0.025	47	1823
CCCGGTATATACG	0.023	0.004	0.024	49	2034
CCTATTATTTGTG	0.029	0.003	0.026	43	1593
CCTGGCACTTATG	0.025	0.007	0.016	17	1073
GGCAGTGTAAACA	0.058	0.002	0.056	127	2161
CCCAGCGCAGGGG	0.04	0.011	0.025	22	874
CGTGGTGCTTGCG	0.03	0.006	0.023	10	430
AGCGGCGCTTAGA	0.022	0.001	0.023	22	939
GCCAGCATTTAGG	0.049	0.005	0.042	54	1218
CCCGGCGTATACA	0.022	0.003	0.018	48	2623
GGCAGTGCATGGA	0.044	0.012	0.053	40	715
GCCGTCACTTGGG	0.055	0.003	0.058	41	662
AGCATTGCTTGTA	0.03	0.001	0.028	49	1678
AGCGGTGTATGTG	0.022	0.004	0.021	19	895
GCCAGCACAAGGG	0.059	0.008	0.059	60	963
GGTGGTATTAACA	0.118	0.008	0.115	145	1121
CCTGGCACTTGGG	0.024	0.003	0.028	18	614
CCTAGCGTTAGCA	0.026	0.004	0.026	42	1598
ACCAGTGCAGGTA	0.022	0.004	0.026	61	2322
GCCAGCGTAAAGG	0.063	0.002	0.063	71	1064
AGTGGCGTTAGTG	0.024	0.007	0.018	5	272
CGCAGCATATGGA	0.018	0.003	0.019	28	1427
GGCGTCGCAAACA	0.066	0.012	0.052	91	1656
CGTGTTATAGGTA	0.033	0.007	0.037	41	1070
CGCAGCACTGGCA	0.027	0.006	0.034	55	1545
GGTGTCATAGACA	0.098	0.006	0.09	102	1034
GGCGGTATTTAGA	0.067	0.009	0.061	60	921
CCCGGTATAAGTA	0.03	0.002	0.032	81	2438
GCCGTTGCTTGGA	0.039	0.015	0.044	29	625
CCTGTCGTAAATG	0.028	0.009	0.029	49	1652
AGCAGTATTGAGA	0.018	0.001	0.018	41	2281

GGCAGCGTAAATA	0.063	0.006	0.057	113	1872
AGCGGCATAAGTA	0.022	0.006	0.014	24	1647
ACTAGTGCTAGTG	0.027	0.005	0.034	45	1285
GGCATTGTATAGA	0.067	0.007	0.06	87	1354
GCTAGTATAGACA	0.066	0.007	0.059	121	1935
CGTATCGCTAACA	0.021	0.005	0.014	27	1838
ACTGTCGCAAACG	0.02	0.004	0.024	46	1848
ACTATCGCTGAGG	0.028	0.006	0.02	25	1229
ACTAGCATTAGTA	0.028	0.002	0.024	54	2185
CCCGGTATTGGCA	0.022	0.008	0.011	19	1730
ACCGGCACTGGCA	0.026	0.002	0.023	48	2012
CGTATTATAGATG	0.021	0.002	0.023	41	1723
ACCGGCACTTGCG	0.023	0.005	0.019	28	1479
AGCGTCGTAAGCA	0.022	0.004	0.017	35	2005
CCTGTTACATATA	0.031	0.001	0.032	78	2360
CCCAGTATAAACG	0.025	0.002	0.026	107	3951
CCCATCATAGAGG	0.039	0.002	0.037	76	1995
GGTATCACATAGA	0.105	0.016	0.098	114	1046
ACTATCATTTGTA	0.027	0.003	0.029	70	2334
GCCGTTGTAAACA	0.046	0.004	0.051	121	2244
GGCATCACTGGCA	0.06	0.006	0.06	86	1349
GCTGTTACAAGCA	0.06	0.005	0.057	82	1365
CCCAGTGCTAAGA	0.024	0.003	0.023	54	2287
GGCATCACTAGTG	0.087	0.004	0.09	109	1100
AGTAGTGTTGATA	0.019	0.001	0.018	25	1362
CGCAGTATTGGCA	0.023	0.003	0.02	31	1533
CCCAGCATATGTG	0.021	0.002	0.018	35	1880
CGTGTCACATGCG	0.027	0.006	0.025	21	820
GCCAGTACAGAGA	0.048	0.007	0.038	69	1743
CCCGTTATAGACG	0.025	0.001	0.026	63	2316
CCTGTCGCTAATA	0.023	0.004	0.027	55	1983
ACCGTCGCATATA	0.022	0.001	0.023	61	2624
GGTGGCACAGATA	0.203	0.034	0.176	175	822
ACTATCACTGGGG	0.033	0.008	0.042	38	871
GGTGTTACATGCA	0.075	0.011	0.069	65	877
ACCGTCGTAAATA	0.024	0.001	0.025	74	2883
AGCAGCGCTAATA	0.025	0.005	0.02	44	2183
CCTGTTACAGAGA	0.019	0.004	0.014	23	1623
ACTGGTACATGTA	0.021	0.005	0.016	34	2031
AGTAGCATTGAGA	0.023	0.002	0.022	30	1349

GGCGTCGTATGTG	0.073	0.019	0.058	38	616
GCTGTCGCAAACA	0.062	0.002	0.06	82	1296
GCTGTTGCTTGCA	0.053	0.004	0.048	35	701
ACCGTCACTTACA	0.02	0.002	0.021	80	3813
CCTATTACATGTA	0.029	0.003	0.024	66	2629
GGCGTTGTAAGTA	0.059	0.011	0.065	81	1169
CGTAGTATTGGGA	0.027	0.005	0.022	14	625
ACCGGTACTAGGA	0.022	0.003	0.024	49	1978
AGTATCGCAGAGG	0.022	0.006	0.023	19	811
AGTGTCGTTTGTA	0.027	0.002	0.024	20	821
ACTGGCACAGGTA	0.026	0.002	0.023	31	1335
CCCATTACAAACG	0.023	0.002	0.021	89	4216
CCTGGCATTGACA	0.028	0.003	0.025	52	1999
AGCGTCGCTTATA	0.019	0.003	0.018	30	1597
ACCGTCACATGGG	0.025	0.001	0.026	30	1117
ACCATTGCAGGCG	0.024	0.001	0.024	53	2190
GCCGGTATTTGGA	0.078	0.013	0.064	62	914
ACTAGTACTTGGA	0.021	0.002	0.018	32	1705
GGCAGTGCAGACA	0.047	0.003	0.044	69	1493
ACTAGTATTAGGG	0.029	0.008	0.018	21	1141
GCTAGCATTGATG	0.062	0.013	0.048	49	981
ACTGTCATTTATG	0.028	0.009	0.016	25	1529
AGCAGCACTAATA	0.022	0.002	0.023	70	2988
CCTGTTACTTGTA	0.028	0.002	0.03	43	1410
GGTGGCACAGACG	0.389	0.061	0.355	327	595
ACCATTACAAGTA	0.022	0.001	0.024	113	4538
CGCGTTACTGGTA	0.028	0.008	0.021	27	1244
ACCGGTGTATAGG	0.025	0.003	0.027	32	1136
GCTGGTGTAAAGG	0.731	0.047	0.724	854	326
AGCATCACAGGTA	0.02	0.002	0.019	45	2323
ACCGTCGCTTGCG	0.022	0.005	0.015	20	1303
CGCATTATTTGCG	0.026	0.004	0.027	36	1302
CCCAGTGTAAATA	0.025	0.002	0.025	97	3761
ACTAGTGTATGTG	0.027	0.004	0.03	39	1257
GGTATTACTGGCA	0.077	0.018	0.052	48	870
GCCAGTGCTGGGG	0.046	0.011	0.041	20	466
CCTGGCGCAGAGA	0.025	0.002	0.027	29	1053
ACCAGTATAGGCA	0.023	0.003	0.023	76	3242
CGTAGCGCAAGGA	0.024	0.006	0.031	25	777
GGCGGCGTATGTA	0.202	0.046	0.193	164	685

AGCAGTGTAGAGA	0.023	0.005	0.016	27	1614
GGCATCATTAACA	0.082	0.017	0.068	200	2724
ACCGGTACTAACG	0.022	0.004	0.019	57	2942
AGCGGTGCTTGCA	0.021	0.002	0.021	22	1035
AGCGGCGTTGACA	0.019	0.003	0.018	26	1426
CCCGTCACAAGGG	0.039	0.001	0.04	55	1304
CCCGGTATTAACG	0.028	0.001	0.029	63	2100
CCCGTCGCTTGTG	0.02	0.004	0.023	22	935
ACTGTCGTAAGTA	0.029	0.002	0.026	42	1579
ACTAGTACTAAGG	0.026	0.005	0.021	43	2049
AGCGGTGTAAATA	0.021	0.002	0.02	50	2486
GGTATTATAGAGA	0.085	0.015	0.085	124	1327
CGCGTCGCATAGA	0.023	0.004	0.024	28	1160
CGTGGCGTAGATA	0.023	0.006	0.03	23	737
GCTAGCGCTGGTA	0.06	0.011	0.067	43	598
GCTGGCACATAGA	0.205	0.027	0.23	241	808
AGTATTATATAGA	0.024	0.002	0.021	55	2507
AGCGGTGCTAGGA	0.02	0.001	0.019	17	895
GCTATTGTTAGCA	0.059	0.002	0.061	90	1387
GGTATCATATGCA	0.133	0.011	0.125	168	1173
GGCAGTGTTGGCG	0.055	0.014	0.045	34	723
CCCAGCACATATA	0.025	0.004	0.024	95	3874
CGCGGCACATGTA	0.025	0.004	0.021	32	1471
GCTATTATTTATA	0.063	0.007	0.059	145	2324
CGCATCACTAGTA	0.025	0.003	0.028	57	1957
ACTGGCACTGAGG	0.023	0.006	0.016	13	810
CCTGTCATTAGCG	0.024	0.004	0.018	23	1254
GCCAGTGCTTACG	0.045	0.001	0.043	47	1035
CCTGGCGCTAGTA	0.03	0.008	0.019	20	1022
CCTGTTATTGGGA	0.022	0.008	0.017	16	913
ACCAGCGTATATA	0.025	0.006	0.024	74	2958
CCTATCACTGATA	0.024	0.003	0.022	67	3014
ACCAGTACTTGTG	0.021	0.003	0.02	41	2020
AGTATCATTAAGG	0.023	0.003	0.026	38	1417
GCCGGCATAGACA	0.372	0.035	0.373	885	1485
GCCGGTATAAGCA	0.077	0.002	0.077	167	1990
CCTATTGTTGATA	0.026	0.005	0.019	47	2393
CCTAGTGCTTAGA	0.021	0.004	0.017	25	1479
CCTATTGTTGACG	0.026	0.002	0.027	49	1794
AGCAGTGTTAAGA	0.023	0.005	0.02	41	1986

ACTAGCGCTGACA	0.022	0.003	0.021	50	2380
ACTGGCGCTAACA	0.028	0.003	0.024	28	1133
AGTGGTGTAGATA	0.022	0.004	0.016	19	1166
CGTGGCATTAAGG	0.022	0.006	0.03	13	425
CGCGGCACTTATA	0.028	0.002	0.027	42	1529
ACTGGTACTTGGG	0.034	0.007	0.025	17	666
CGTGTCGTTTATG	0.026	0.006	0.023	18	762
GCTAGCGTAGACA	0.073	0.008	0.062	80	1215
CCTATTGCAAAGA	0.027	0.003	0.029	81	2707
GCTAGTGTATAGG	0.048	0.005	0.048	42	842
GCCAGTACTTATA	0.052	0.005	0.047	102	2077
CGCGTTGTTGGGG	0.026	0.004	0.028	13	455
CGTAGCGCATAGG	0.021	0.007	0.013	8	594
GGCGTCGTTTACG	0.087	0.012	0.07	56	740
GGCGGCGTTAAGG	0.441	0.052	0.399	305	460
ACTAGCGCTTAGG	0.029	0.004	0.033	32	951
AGTGTTGTTAAGG	0.016	0.004	0.015	12	781
CGTAGCACATAGA	0.025	0.009	0.016	19	1159
CGCGTTGCAGGGA	0.022	0.002	0.024	20	828
GCCGTTATAGACA	0.049	0.01	0.037	90	2358
GGCAGCGCATGGG	0.063	0.007	0.058	36	589
GCTAGCGCAAAGA	0.063	0.003	0.066	77	1087
CCCATCGTAAATG	0.023	0.002	0.022	67	3016
AGTGGTGTTAGGA	0.011	0.004	0.005	3	568
AGTGTCGCAAACA	0.021	0.003	0.017	30	1730
AGTGGTGTTAGTA	0.017	0.006	0.009	8	929
GCTGTCGCATGTG	0.063	0.015	0.083	42	462
GCCAGTGCTAGGG	0.045	0.004	0.04	28	669
GCCGTCATAGGTA	0.053	0.005	0.05	80	1530
GGCGTCGTAGGCG	0.067	0.012	0.049	37	714
GGCGGCGTAAATG	0.472	0.048	0.473	649	723
GGTGTCGTAAATA	0.094	0.008	0.087	102	1069
GGCAGCACAGATA	0.047	0.001	0.047	87	1766
GGCGTTATAGAGA	0.061	0.005	0.057	76	1257
CGTATTACTTGGG	0.026	0.004	0.02	15	726
CCCGTTGTTTAGA	0.025	0.004	0.025	36	1406
ACTAGCACTAAGA	0.022	0.004	0.028	77	2722
CGCGTCATTTACA	0.02	0.003	0.018	36	1991
GGCATTACAGGCG	0.062	0.01	0.05	60	1149
CCTAGCGCTAACA	0.024	0.001	0.023	65	2705

CGTAGTATAGGGA	0.025	0.011	0.04	29	700
GCCGGCACTAAGA	0.112	0.011	0.096	148	1387
CGTGGCGCTAGGG	0.02	0.006	0.016	5	312
AGCAGCACTGAGA	0.023	0.004	0.02	36	1766
GGTATTGTTTGTA	0.104	0.028	0.075	64	784
CGCATCACTTATA	0.025	0.002	0.023	60	2562
GGTGTTATTGAGA	0.079	0.02	0.052	40	731
GGTGGTGCTAAGA	0.075	0.02	0.091	53	529
AGTAGTGTTGGCG	0.016	0.002	0.016	12	724
CCTATCGCTTATA	0.023	0.006	0.022	51	2252
GCCGTTGTAAACG	0.043	0.005	0.041	64	1514
CGTATTACATACA	0.022	0.002	0.021	54	2557
ACCAGTATTGATG	0.021	0.005	0.025	65	2558
ACCAGTGTTGGCG	0.026	0.008	0.016	33	2011
CCCGGCATAGGTA	0.027	0.007	0.024	45	1808
ACTGGTGTATACG	0.026	0.006	0.017	20	1157
GCCATTACAGACG	0.061	0.005	0.055	114	1959
ACTGTTGTATGCG	0.034	0.01	0.021	24	1118
GCTGTCGTAGAGG	0.076	0.01	0.073	32	405
CCCGGCGTTGATA	0.025	0.003	0.026	48	1773
GCTGTCGCAAATA	0.057	0.012	0.041	53	1243
CCTAGTGTTGGCA	0.023	0.003	0.024	35	1439
GGCAGCACATAGA	0.051	0.003	0.049	71	1365
ACTGGTATTGGCG	0.024	0.009	0.022	19	856
GCCGGTATTTATA	0.08	0.007	0.071	134	1758
GGCGGCGTTTACG	0.444	0.052	0.402	354	527
GGTAGTGTAAAGG	0.064	0	0.065	48	692
ACTGTTACTTGTG	0.023	0.005	0.027	31	1137
CGCATTGCAGGCA	0.022	0.003	0.02	32	1562
GGCAGTGCTTACG	0.05	0.012	0.034	34	975
CCCAGCGCAGGTG	0.026	0.007	0.027	36	1302
GGCATCATTGGCG	0.081	0.004	0.076	96	1161
CGCAGTGTAAATA	0.027	0.004	0.022	60	2645
CCCGGTGTAAGTA	0.026	0.004	0.022	40	1751
ACTAGTACATGCG	0.023	0.003	0.021	42	1966
GCCGTCGTTAACA	0.049	0.001	0.047	80	1612
GGCAGTACATGTA	0.056	0.005	0.05	80	1508
CGTATTATTAACG	0.028	0.006	0.028	60	2072
AGCAGCATTAACG	0.023	0.004	0.022	56	2438
AGCAGTACAGGGG	0.02	0.004	0.019	20	1049

GCCGTCACAGACG	0.058	0.005	0.065	103	1491
GCTAGTGTAAACA	0.059	0.004	0.055	135	2307
AGCAGCGCAGGCA	0.021	0.001	0.02	31	1522
AGTAGTGTATATA	0.024	0.002	0.023	39	1691
ACCAGTACTGGTG	0.019	0.003	0.021	44	2015
GGTGGCGCTTGGA	0.182	0.052	0.136	48	304
CGTAGTGTTTGTA	0.024	0.006	0.03	21	683
AGCGGCATTAGGG	0.023	0.011	0.033	15	438
AGTATTACTAATG	0.026	0.005	0.02	43	2089
AGCATTACAAGCA	0.022	0.001	0.021	74	3447
CCTGTTATAGATG	0.032	0.008	0.03	50	1628
GCCAGTGCTAGTG	0.049	0.005	0.055	46	796
CGCGGTGCTAGGA	0.025	0.004	0.027	21	748
ACCAGTACAGGTG	0.021	0.001	0.019	41	2111
ACCATCGTTTAGG	0.023	0.003	0.019	26	1350
AGTGGTGCAGGCG	0.022	0.007	0.013	8	606
GGTATTGTATGGA	0.103	0.024	0.078	59	702
GCTAGTGCAGGTA	0.057	0.008	0.06	56	871
CCTGGCGCATACA	0.025	0.002	0.023	41	1774
GGTAGCGTTAACG	0.077	0.021	0.067	55	762
AGCATCGTTAGCA	0.023	0.003	0.027	59	2098
ACCATTACAAACA	0.021	0	0.021	185	8680
AGCAGCGCATAGG	0.023	0.002	0.022	23	1034
ACCGGTGTATGCG	0.029	0.006	0.023	29	1259
GGCGGTACTAATA	0.061	0.009	0.05	84	1585
ACTATCACAAGTG	0.026	0.003	0.022	53	2406
CGCGTCGTATGTA	0.022	0.006	0.016	22	1337
GCTATCACAGGTA	0.056	0.004	0.061	76	1180
GGTGTTGCAAGCG	0.073	0.021	0.051	29	536
AGTATCGCAAATG	0.022	0.002	0.019	32	1611
GGTGTTATTGATA	0.074	0.014	0.068	72	981
CGTATCATTAGCA	0.026	0.002	0.023	41	1714
CCTGTTACTAGCG	0.028	0.008	0.021	25	1140
CGCAGTGCATGCA	0.025	0.003	0.022	34	1497
ACCGGCACAAATG	0.019	0.005	0.013	37	2763
AGCGTCATTAACA	0.018	0.001	0.02	65	3208
ACTATTGTATATG	0.03	0.003	0.026	59	2229
ACTGTTGTTTATG	0.027	0.004	0.032	40	1203
CGCGGTATTAAGG	0.027	0.006	0.022	25	1087
ACCAGCGTTGGTA	0.024	0.008	0.013	20	1513

GGCAGTGTAAGCG	0.051	0.008		0.048	54	1060
CCTGGCATTAAGA	0.03	0.002		0.027	43	1561
CCCGGCATAAGCG	0.025	0.003		0.021	41	1913
GCCAGTGTTAATA	0.045	0.008		0.037	77	1979
GCTGTTGTATATG	0.062	0.008		0.051	49	912
ACTGGCATTGAGG	0.03	0.005		0.036	30	800
CCTATTGCAGGTA	0.027	0.005		0.024	46	1837
AGCATTGCTTGTG	0.021	0.003		0.018	21	1175
GCCGTCACTGAGG	0.048	0.003		0.05	44	836
CCCAGCACTGACA	0.035	0.003		0.034	110	3137
AGTGTTGTTTAGA	0.027	0.003		0.032	27	830
AGCATTACTTGGA	0.02	0.001		0.02	35	1737
AGCAGTGTAAGGA	0.02	0.003		0.02	34	1632
ACTAGTATTGGGA	0.027	0.003		0.024	30	1207
GCTAGCGTATGGA	0.073	0.004		0.069	50	675
AGTGTCACTGATG	0.021	0.004		0.016	16	957
AGTGGCGCAAATA	0.022	0.004		0.017	23	1347
CCCGTCGTAGACG	0.021	0.002		0.02	38	1872
CCTAGTGCAAGGA	0.024	0.004		0.024	40	1620
CCCGGCACATGCG	0.022	0.004		0.016	27	1666
GGCGTTGTAGGGG	0.054	0.008		0.043	23	513
CCCGGTATAGACA	0.023	0.003		0.028	85	2939
GCTGGTACAGACA	0.068	0.009		0.061	79	1217
AGTGTTACATGTG	0.028	0.01		0.015	14	944
ACTAGCACATGCG	0.022	0.001		0.02	33	1592
CCCGTCGTATAGA	0.019	0.003		0.015	28	1848
GCTATTGCTGGGG	0.057	0.008		0.052	27	496
CGCATCATAGGCA	0.027	0.002		0.026	61	2259
CGTATCATAGAGG	0.02	0.002		0.022	24	1062
CGTGTCGTATGGA	0.018	0.009		0.023	15	641
GGTATCACTAAGG	0.126	0.021		0.113	91	711
GGTAGTGCAGATA	0.053	0.008		0.043	38	852
GGTGGCACTTAGA	0.21	0.019		0.196	128	524
GCTGGTGTAGGGA	0.389	0.037		0.366	229	396
CGTAGTGCAAACG	0.022	0.003		0.017	18	1021
CGTAGCGTATACA	0.022	0.001		0.021	30	1403
GCCAGCGCTAGTA	0.055	0.009		0.048	53	1054
CCTATCGCAAGGA	0.024	0		0.024	44	1778
CGTATCGCATGCA	0.031	0.005		0.023	28	1168
CCTGTTACATATG	0.029	0.005		0.023	37	1542

GCCAGCACAGATA	0.054	0.006	0.046	108	2226
AGTATTGTATGCA	0.021	0.003	0.021	36	1651
GGCGTTATAGGCA	0.068	0.009	0.068	90	1241
CCTGGCGTATGCA	0.029	0.002	0.027	37	1329
CCCAGCGCAAATG	0.025	0.003	0.022	52	2362
AGCAGCGTTGGGG	0.022	0.003	0.017	15	847
CGCGTCGTTAGTA	0.025	0.002	0.022	27	1198
ACTAGCACTGAGA	0.023	0.002	0.024	53	2162
CGCAGTGTTGACA	0.026	0.003	0.025	45	1749
GGTGGCACTTGTA	0.186	0.011	0.194	125	520
ACTAGCATTAATA	0.027	0.003	0.024	88	3636
CCCGGCATAGATA	0.026	0.005	0.022	61	2689
GCTGGCGTAGGCA	0.883	0.025	0.897	1196	138
GGCATCATAAAGA	0.083	0.015	0.073	186	2379
ACTGTCGCAAACA	0.026	0.003	0.022	67	3044
ACTAGCACTGATG	0.026	0.003	0.023	44	1829
ACTGGCATTAGGA	0.026	0.009	0.037	41	1075
CGTATCGCTAAGG	0.029	0.008	0.019	17	857
GCCGTCACATGGA	0.051	0.008	0.04	45	1088
CCCGGTGCTGAGG	0.025	0.003	0.022	17	766
GCCATCGCATATG	0.047	0.012	0.036	49	1318
CCTGTCACTTACA	0.027	0.005	0.022	48	2119
CGCGGCATATGGA	0.026	0.004	0.02	22	1085
GGCATCACAGACA	0.066	0.013	0.06	141	2201
AGCGTTATTAGCG	0.019	0.004	0.014	20	1421
CCCAGCACAAGCA	0.027	0.002	0.025	95	3713
CCCGTTGTAAAGA	0.021	0.004	0.016	38	2275
GGCAGCGCATATA	0.067	0.003	0.067	95	1315
GGCAGCGCAGACG	0.053	0.016	0.033	35	1015
GCTATCGTTGATG	0.059	0.008	0.05	50	957
CGTATCATTGGCG	0.021	0.006	0.014	16	1160
CGCAGCGTAAGCA	0.027	0.004	0.032	57	1734
CCCATTGTATATG	0.025	0.004	0.02	46	2203
ACCGGTATTTATA	0.023	0.005	0.026	75	2784
CCTATTACTGGGG	0.038	0.008	0.028	25	882
GCCGTCGTTGACA	0.047	0.004	0.042	57	1295
GGCAGCATTAGCA	0.051	0.007	0.048	72	1415
ACCAGTACAAGTA	0.022	0.003	0.024	112	4590
CGCGTCACATAGG	0.021	0.002	0.019	21	1108
ACCGTTACAGAGA	0.022	0.003	0.018	54	3005

ACCGTTGCTAGCG	0.025	0.006	0.024	38	1566
CCCATTGCAGAGA	0.022	0.002	0.02	48	2409
GCTGGCATTAACA	0.65	0.042	0.66	1535	789
ACCAGCATAGAGA	0.024	0.002	0.023	69	2953
CGCAGCGCTGACA	0.026	0.007	0.018	32	1745
ACTATTGTTAATG	0.026	0.006	0.018	44	2387
CCCATCGCAAAGA	0.025	0.001	0.024	79	3278
CCTGGTGCTAACG	0.026	0.005	0.02	23	1146
CCCGTTATAGGCG	0.022	0.003	0.026	45	1668
CCCATCACTTACG	0.028	0.002	0.026	72	2712
GCCGTCACTGGCA	0.056	0.004	0.056	82	1374
GGCGGTGCAAAGA	0.066	0.005	0.07	74	977
CGCGTCGTAAGTG	0.029	0.001	0.028	29	1019
CGTGTTACTTGTA	0.027	0.001	0.027	25	905
ACTATTGTATGTG	0.028	0.001	0.03	51	1665
GCTATTGCTGACA	0.049	0.004	0.044	69	1496
ACCATTGCTTGCA	0.023	0.004	0.02	52	2575
AGTGGCGTTAGCG	0.022	0.008	0.022	6	269
AGCATTGCAGATG	0.023	0.004	0.023	43	1831
CCCAGCACAGGCG	0.029	0.002	0.026	51	1881
CGTATCGCTTGCA	0.018	0.001	0.019	19	962
GGCAGTACTTACA	0.05	0.007	0.051	95	1756
CGTGGTATAGGGA	0.021	0.003	0.018	11	610
ACCGGCATATATG	0.026	0.005	0.029	58	1914
CCCATTACTTACG	0.028	0.002	0.026	71	2703
CCTATCGTAAAGA	0.027	0.003	0.027	73	2582
AGTGGCATATATA	0.025	0.003	0.029	29	971
AGTAGCGTAGAGG	0.025	0.013	0.043	35	776
CGTGGCATATGTA	0.022	0.004	0.027	24	876
CCTAGTGCTTGTG	0.023	0.004	0.017	16	945
CCCGTTGCAAGGA	0.032	0.003	0.036	59	1599
GGTGTCACTAATG	0.092	0.018	0.073	57	719
GGCATCACTTATG	0.096	0.017	0.076	103	1257
CGCATCGTTAATG	0.025	0.002	0.025	45	1727
CGCATCATTTAGG	0.023	0.004	0.025	34	1302
CCTGTCACTGGGA	0.024	0.007	0.018	17	921
CCTAGTGTAGGTG	0.024	0.005	0.018	19	1041
ACCGTCGTTTATA	0.025	0.003	0.027	50	1826
ACCGTCATTAGCG	0.02	0.001	0.019	38	1964
CCCAGCACTTATA	0.025	0	0.024	78	3128

GGCGGCGCTAGGA	0.096	0.021	0.078	48	566
ACCGTCACATACA	0.021	0.003	0.017	82	4801
GGTGTTATATGGG	0.072	0.013	0.064	34	494
GCCGTTATAAGCG	0.059	0.014	0.062	98	1495
ACTATTGTTAACA	0.025	0.005	0.023	86	3618
GGTGTTGCAGGCG	0.088	0.011	0.074	35	440
GGTAGTGCAAGGG	0.063	0.01	0.057	25	414
AGCAGCGCAAGGG	0.021	0.005	0.017	18	1017
ACTGGCATTAGTA	0.026	0.006	0.019	30	1519
GGCGTCATATAGA	0.071	0.016	0.058	77	1248
GCCGTCGTAGACG	0.052	0.01	0.064	62	912
AGTGGCACTGACG	0.023	0.003	0.025	19	730
ACCGGTGCAGAGG	0.019	0.005	0.013	15	1109
ACCATCACTGGGA	0.028	0.002	0.026	44	1639
AGTGGTACAAAGG	0.025	0.004	0.018	17	914
AGTAGCGCAGGGG	0.025	0.008	0.029	13	435
AGTGTCGCAGACA	0.033	0.002	0.035	46	1271
ACTATTATTTACA	0.029	0.003	0.028	108	3723
ACCAGTATATATA	0.024	0.002	0.026	142	5295
GCCATCATTAAGG	0.06	0.005	0.054	107	1863
GCCAGCATATGGG	0.065	0.014	0.051	42	783
AGCATCGTATGCA	0.022	0.004	0.02	48	2319
CCCGGCATTGGGG	0.031	0.005	0.034	26	732
GCTAGTACATACA	0.058	0.007	0.049	103	2002
AGCGGTGTTTGGA	0.019	0.004	0.017	13	752
CGCATTGTATATA	0.024	0.004	0.021	53	2441
ACCAGCATTTAGG	0.025	0.001	0.024	44	1827
CCCAGCGTTTACG	0.023	0.001	0.022	43	1887
GCCGTTGCTAGGA	0.054	0.003	0.052	41	755
GGCGGTATTGGCA	0.074	0.013	0.061	55	851
AGCGGTACATACA	0.031	0.003	0.028	73	2536
ACCGTCGTATGCA	0.028	0.003	0.023	42	1769
GCCATTACTGATG	0.048	0.005	0.054	95	1673
GGTGTTACTTACA	0.079	0.016	0.064	63	922
CCTGGCGTTTGTA	0.031	0.012	0.032	34	1029
GCCGTTGTTGGGG	0.054	0.003	0.058	25	405
CCCGGCGTATGCG	0.025	0.007	0.016	20	1224
AGCGGCATATGGG	0.028	0.008	0.033	15	438
GCCGTCATAAGTA	0.05	0.008	0.039	81	2008
GGCATCGTAAACG	0.116	0.014	0.104	188	1628

ACTGTCGTAGGTG	0.03	0.008	0.02	15	747
ACCATTGTTGGTG	0.029	0.003	0.029	37	1235
CCCATTGTAAGTG	0.026	0.003	0.023	49	2044
GGCGTTATAGAGG	0.055	0.006	0.049	43	839
CCTAGTGCTTGGG	0.029	0.001	0.03	21	678
GCTGGCACAGATA	0.216	0.023	0.216	288	1044
ACTATCGCTTAGA	0.026	0.001	0.025	43	1702
AGTAGCGCTAACA	0.025	0.002	0.022	36	1619
CCCATTATTTGCG	0.025	0.009	0.036	76	2013
AGTAGCGTTAGTG	0.028	0.005	0.025	17	660
AGCGGCACTTGTG	0.026	0.003	0.022	15	668
ACCATCGCAAGGG	0.023	0.001	0.021	35	1594
ACTGGTGCATGTA	0.027	0.007	0.029	42	1407
GGTGTTGCATACG	0.081	0.027	0.047	30	615
GGTAGTGTAGACG	0.06	0.02	0.036	26	687
GGTGTCACTGATA	0.073	0.008	0.07	61	810
ACCGGTGCTTGTA	0.021	0.003	0.019	31	1561
GCCGGTGCTGGCG	0.07	0.009	0.082	65	725
CGTGGTGCTAACG	0.031	0.008	0.023	15	631
CGTGGTGTTGAGA	0.025	0.008	0.029	17	568
GGCGGCACATATG	0.273	0.057	0.252	287	853
GGCATTGCTAATA	0.057	0.005	0.052	99	1823
ACTGTTACTAGTA	0.025	0.001	0.024	54	2182
ACTATCGCATATG	0.025	0.003	0.021	41	1867
GCCAGTGTAGATG	0.044	0.008	0.054	67	1170
ACCGTTGCATGGA	0.024	0.002	0.022	34	1511
ACCGGTACAGGTG	0.021	0.004	0.026	44	1641
AGTGGTATTGGTA	0.018	0	0.018	17	919
CCCGGTACATATA	0.027	0.004	0.032	92	2789
GCCATTATTGATA	0.056	0.002	0.058	173	2800
GGTGGCACATAGA	0.198	0.044	0.162	134	692
GGTAGCGCAAAGG	0.073	0.022	0.051	31	580
ACTGGTACTGGGG	0.031	0.003	0.031	17	540
CGCAGTACATGCG	0.022	0.003	0.023	31	1329
ACTGGCACTTGCA	0.021	0.003	0.019	27	1425
AGCGTTGCTAGCG	0.018	0.001	0.02	22	1087
ACCGTCATAAGCA	0.024	0.003	0.02	70	3481
AGTGGTGCAGGTG	0.025	0.004	0.028	14	478
CCCGTCATAAGTA	0.021	0.004	0.02	60	2993
ACTGTTGCATGGA	0.025	0.004	0.03	37	1208

ACTATCACAGGTA	0.027	0.007	0.017	44	2526
GCTAGCGTATGCG	0.07	0.017	0.063	44	653
AGCGGTGCTGGCA	0.02	0.004	0.026	28	1055
CCCAGTACATACG	0.023	0.002	0.021	59	2812
CGTGTTATTAATG	0.027	0.003	0.028	36	1261
GGCGTTGTATATG	0.073	0.003	0.074	69	863
ACTGGTGTTAGCA	0.025	0.005	0.022	27	1201
AGCGTCATAGAGG	0.015	0.003	0.014	18	1284
ACCATTGTTAGTG	0.027	0.007	0.026	46	1748
CCTGTCATAAAGG	0.028	0.005	0.023	39	1693
ACCAGTGTTTGGA	0.023	0.008	0.014	20	1372
AGTGTCACAAAGA	0.026	0.004	0.024	38	1538
CGCGTTATAGGGG	0.042	0.008	0.035	25	688
GCCGGTACTGATA	0.052	0.011	0.068	107	1470
CCCGGCATTAGTG	0.021	0.002	0.019	25	1325
GCTATCATAAGCG	0.07	0.009	0.06	87	1367
CGTGTCATAAATA	0.028	0.005	0.021	48	2210
CCTGTTATAGACA	0.022	0.003	0.02	54	2682
AGTGTTATAAGGA	0.025	0.005	0.02	30	1452
CGTAGCGTATATG	0.018	0.004	0.017	15	855
AGCATCGCTGGCA	0.023	0.001	0.023	42	1770
GCCAGCGTATACA	0.062	0.003	0.058	118	1926
AGTATTGTATGTG	0.025	0.003	0.028	32	1102
CGTATCGCAGGGA	0.02	0.003	0.02	15	748
CGTAGCGTAAGTA	0.023	0.001	0.023	26	1100
ACTGTTGTTAAGA	0.025	0.002	0.022	37	1637
CGCGGCACAGATG	0.031	0.005	0.032	38	1165
CGCGGTATTTATA	0.028	0.003	0.026	43	1628
GCTATCATAGACA	0.065	0.01	0.055	127	2191
AGCGTTATTAATA	0.02	0.003	0.024	83	3331
AGCGGCGCTAGCA	0.027	0.009	0.016	20	1210
GGCGTCGTTGGGG	0.074	0.012	0.079	36	420
ACTAGTGCTTATG	0.029	0.002	0.029	48	1632
CCCGTCACTGAGG	0.025	0.005	0.022	30	1358
CCCAGCGCATGTA	0.023	0.003	0.025	61	2427
AGCGGCGTTAATA	0.028	0.005	0.034	57	1626
GGCGTTACAAACG	0.069	0.009	0.068	110	1509
CCCGGTATTGAGG	0.026	0.008	0.015	18	1208
GGCATTATTGATA	0.057	0.01	0.045	103	2204
GCCGGTATATAGA	0.074	0.003	0.071	115	1506

GCTAGCATTAGTA	0.064	0.005	0.068	98	1334
CCCAGCACTGACG	0.046	0.003	0.043	102	2278
CGTGGCGCTAATG	0.026	0.009	0.017	11	643
AGCGTCACAAGGG	0.017	0.001	0.018	22	1180
GCCGGTGTTAATA	0.18	0.025	0.177	287	1339
CCCGTTACTGGCG	0.035	0.002	0.033	47	1359
GCTATCGTAGGTG	0.055	0.01	0.041	29	681
GCCATCATTAGTA	0.053	0.006	0.046	113	2339
ACCAGCGTTGAGG	0.019	0.003	0.02	21	1022
ACCATCGTTAGCA	0.024	0.004	0.02	52	2543
GGCGGCGCTTGTA	0.106	0.022	0.084	59	640
GCTATCGTTGGGA	0.065	0.003	0.068	53	722
AGTGTTACTTGCG	0.023	0.009	0.017	13	745
GCTGTCACATAGG	0.061	0.009	0.062	41	616
GGCGTCGTAGGCA	0.076	0.011	0.067	67	938
GCCATCGCAAAGG	0.044	0.003	0.04	51	1236
GGTGTCGTAAAGA	0.083	0.007	0.084	72	786
GCCATCATAGGCG	0.066	0.006	0.061	93	1428
CGCATCGTTGGTG	0.027	0.006	0.027	26	945
ACTATTGTTTGCA	0.028	0.003	0.026	53	2010
CCTGGCGCTAAGG	0.029	0.001	0.031	24	758
GCCGTCATAGATG	0.048	0.012	0.039	57	1416
CCTAGTGTAGACG	0.025	0.003	0.023	40	1710
AGCATCATTTATG	0.026	0.003	0.026	64	2393
ACCGGCACTGATG	0.023	0.004	0.028	48	1678
ACCAGTGCATGTG	0.024	0.001	0.023	42	1751
GCTATCGCTGATA	0.049	0.006	0.049	64	1254
GCCGGTACTAATG	0.072	0.008	0.078	102	1205
CGTGTCGCTGGCG	0.022	0.005	0.028	21	732
CCTGTTATTTGTA	0.031	0.004	0.026	39	1452
GGTATTATTGGCG	0.083	0.015	0.064	57	840
ACTAGCATTTGCA	0.03	0.006	0.021	42	1915
CGCGGTATTGAGA	0.019	0.006	0.01	12	1133
CCCGTTATTTGCG	0.027	0.005	0.03	48	1528
CCTGTTGCTTGCG	0.029	0.01	0.016	15	920
CGCGGCATATACG	0.025	0.005	0.021	25	1142
ACCAGTGTTTGGG	0.025	0.003	0.021	20	934
CCTGGCACTAGGG	0.027	0.004	0.031	22	697
CCCGTCGTATATG	0.022	0.009	0.011	17	1602
AGCAGTGCAAGTA	0.023	0.004	0.023	51	2163

GCTATCGTTAACG	0.061	0.01	0.053	75	1334
ACCGTCGTTTGTG	0.025	0.003	0.026	22	825
CGCAGTATAGGGG	0.04	0.012	0.023	18	756
CCTATCACATGTA	0.023	0.002	0.021	59	2749
ACTGTTATTTGGA	0.025	0.004	0.029	40	1330
CGCGGTACAGAGG	0.036	0.008	0.047	42	853
GGCATCACAAAGA	0.074	0.016	0.07	159	2114
AGTAGCACTGAGG	0.022	0.003	0.02	15	752
ACTAGTATTTACA	0.025	0.004	0.023	76	3287
ACCATCATTAACG	0.026	0.002	0.022	93	4059
CCCAGCATTTGCA	0.03	0.002	0.029	74	2494
CCCGGCGCTAAGG	0.02	0.006	0.022	28	1228
CGTAGCACATATG	0.029	0.005	0.025	28	1072
CCTGTTACTGAGG	0.028	0.007	0.02	19	909
GGTGTTACAAATA	0.079	0.008	0.072	128	1659
AGTATCGCTTGTA	0.023	0.009	0.011	11	981
ACCAGCGCTTATG	0.019	0	0.019	33	1699
ACTAGCGCATACG	0.029	0.003	0.032	54	1627
CCTGGTACAGATA	0.024	0.003	0.024	47	1896
CGTAGTACTAGGA	0.021	0.006	0.014	12	817
GCCAGTACATACG	0.058	0.012	0.042	80	1808
ACCATCGCTTGTG	0.027	0.001	0.026	38	1398
CCCAGTACTAGTA	0.024	0.001	0.024	66	2644
AGTATTATTGATA	0.023	0.001	0.023	60	2601
GCTGTCGCAAAGG	0.073	0.007	0.063	40	590
ACTGTCGCTAAGA	0.03	0.007	0.022	37	1639
GGTAGTGCAAGTA	0.055	0.007	0.047	43	874
ACTAGTGCTAATG	0.022	0.001	0.023	46	1928
CCCGTTACAAACA	0.025	0	0.025	117	4570
GGCGGCACTAGTA	0.146	0.029	0.137	140	882
CGTGTCGCTAGGG	0.033	0.013	0.03	13	414
CGTGTCGCTTATA	0.019	0.003	0.017	17	979
GCCGTTGTTTACG	0.057	0.006	0.053	52	931
CGTAGTGTTGGCA	0.022	0.005	0.029	20	660
CCTGTTGTAAGCA	0.022	0.003	0.025	45	1785
GCTATTATATAGA	0.065	0.006	0.061	139	2148
ACTGTCGTTTACG	0.026	0.003	0.024	25	1011
GGTATTGTTGATA	0.099	0.013	0.081	97	1100
CCTAGCACTGGGG	0.052	0.001	0.052	40	732
CCCAGTGCAAAGA	0.026	0.004	0.022	61	2740

CGCGGTATTTGCA	0.022	0.005	0.015	19	1241
GGTATTGTTAGCG	0.107	0.027	0.073	44	562
GCTGTTACTAGCG	0.062	0.006	0.067	50	696
AGTAGTGTAGACG	0.021	0.007	0.012	14	1183
GCCAGCACATATG	0.053	0.004	0.056	97	1643
CGTATCGCATACA	0.028	0.007	0.018	32	1739
GCTATTGTTTAGG	0.072	0.012	0.066	56	787
AGTATCACAGAGA	0.024	0.002	0.026	36	1323
CCTGTCGCTGATA	0.026	0.004	0.02	31	1528
ACCGGCGTAAAGG	0.032	0.006	0.028	23	799
ACTGGTATTAGTA	0.028	0.003	0.025	44	1732
ACTAGTGTAGGGG	0.022	0.006	0.016	15	913
CCCGGCATTAGTA	0.026	0.002	0.022	48	2090
CGCAGTACAGGCA	0.023	0.006	0.02	36	1779
CCCGGTGCTGGGA	0.023	0.001	0.024	21	859
CCTAGTGTTGGTA	0.027	0.003	0.024	30	1212
CCCATTATAGATG	0.026	0.001	0.026	81	2999
GCTATTACAAATA	0.058	0.004	0.053	168	3030
ACCGGTATAAGGA	0.022	0.004	0.016	36	2158
AGTATTATATGTG	0.019	0.003	0.018	31	1655
GGCAGCATATGTA	0.049	0.001	0.048	81	1605
GCCATCGTTGATA	0.051	0.004	0.05	93	1749
ACCAGTACTTGCG	0.023	0.002	0.021	50	2306
GGCGGTACTGGTG	0.069	0.013	0.064	36	524
GCCAGCGCAGGCA	0.053	0.003	0.056	66	1117
CCTATCATAAGTA	0.027	0.001	0.026	92	3493
AGCATCGTTTGTA	0.019	0.004	0.018	34	1865
CGTAGTACATAGG	0.02	0.003	0.023	18	755
GGTATTACAAGCG	0.112	0.012	0.109	126	1031
CGCAGTACTTATG	0.024	0.003	0.024	34	1360
AGTATCACTTGCA	0.022	0.002	0.021	27	1284
CGCAGTACAGACA	0.024	0.003	0.022	55	2407
AGCATTACTTGCG	0.021	0.001	0.02	36	1730
ACTAGCATAAGGA	0.026	0.003	0.022	43	1894
AGCGGTGCTAACG	0.02	0.006	0.012	16	1302
GGTGGCGCAAGCA	0.171	0.029	0.178	120	554
CCTATCGTAGAGA	0.022	0.002	0.021	39	1800
ACTGTCATTAAGA	0.025	0.001	0.024	52	2147
CCTAGCACAGGTA	0.024	0.001	0.023	43	1799
CGTAGTATTAGCG	0.028	0.004	0.03	26	846

AGCGTTACTTATA	0.022	0.002	0.022	48	2094
AGTGTTACATACA	0.027	0.002	0.026	57	2102
GGCAGCGCAAACG	0.059	0.009	0.056	81	1375
CCCAGTGCAAACA	0.023	0.003	0.019	75	3932
ACTATTATATAGG	0.03	0.004	0.025	57	2197
CGTGGCGTTGGCA	0.024	0.008	0.013	8	607
GGCGTTGCATAGG	0.056	0.011	0.064	44	640
GGTGTTGTTGATA	0.075	0.013	0.068	49	667
GCCATTACTTGGG	0.053	0.008	0.057	56	928
CGTGGTACTAATA	0.029	0.002	0.026	37	1371
GCTAGCGCAAGTA	0.06	0.015	0.071	75	987
AGTAGTACAGACG	0.022	0.006	0.014	18	1250
CGTGGTACAGGTA	0.024	0.006	0.025	21	826
CCTGTTATAAATA	0.026	0.006	0.02	74	3559
GCCATTACAAACA	0.051	0.001	0.051	210	3884
GGTATTACTGAGG	0.111	0.022	0.081	57	649
ACTGGCACATGCA	0.024	0.003	0.027	47	1667
CGTAGTGTAAGGG	0.026	0.011	0.012	7	571
GCTGGTACTGGGG	0.121	0.029	0.088	28	289
CCCAGTGCAGGGG	0.027	0.005	0.032	30	914
CGTGGTGCAAACA	0.022	0.005	0.016	23	1424
AGTGGTATATACA	0.023	0.002	0.026	45	1712
AGCATCGTAGATA	0.023	0.003	0.027	78	2768
ACTGTCGCAAGCG	0.027	0.001	0.026	35	1325
AGTAGTATAAACG	0.024	0.002	0.024	57	2297
CCCGTTACAAACG	0.023	0.002	0.021	65	2964
AGCATCGTAAGGA	0.02	0.002	0.017	35	1998
CGCGGCGCTGAGG	0.021	0.006	0.024	16	647
ACTAGTGTTAACA	0.024	0.001	0.025	79	3085
CCTGTCGTTTGGA	0.029	0.005	0.024	24	969
GCTGTTGTTTGCG	0.061	0.007	0.052	27	496
GGTGTCGTTTACG	0.101	0.031	0.067	36	501
AGCAGCGTATATA	0.022	0.004	0.027	65	2369
ACTAGCATTAGGG	0.028	0.004	0.029	27	914
GCCAGTACATAGA	0.052	0.003	0.048	95	1894
CCTGGCATAAAGA	0.025	0.003	0.023	50	2137
CGCATTACATGGG	0.027	0.004	0.021	24	1104
AGTATCATTTATA	0.02	0.004	0.014	33	2341
GGCAGCGTAAGGG	0.061	0.008	0.05	40	762
AGCATTGCTAGGA	0.022	0.002	0.019	29	1533

ACTGTTGCTTAGG	0.029	0.006	0.037	34	877
CCTAGTGCTAGTA	0.022	0.005	0.016	25	1576
CCCATTATAGAGA	0.025	0.002	0.026	84	3183
CCCGGTACATAGA	0.022	0.004	0.02	42	2095
AGCGGCACAAACG	0.021	0.002	0.024	39	1605
GGCAGTGTTTGGA	0.054	0.008	0.063	46	688
GCTGGCGCATATA	0.622	0.054	0.623	862	522
ACCGTCACATATG	0.025	0.002	0.028	73	2547
ACCGGCATAGACA	0.025	0.003	0.021	66	3019
CCCAGTGCTAGCA	0.029	0.003	0.031	67	2114
CGTGGCGCATACG	0.028	0.003	0.024	17	683
CGTGGCGCTAACG	0.03	0.003	0.029	20	681
GGCATCGTAAGCA	0.096	0.024	0.092	170	1684
GCCATCACTTGGA	0.055	0.009	0.063	84	1256
CCCGGTACAGGGG	0.037	0.006	0.029	28	928
GCCGGTATTAGCA	0.08	0.007	0.073	116	1471
CCTATCGCAAACG	0.025	0.003	0.021	50	2277
ACTATCACATAGG	0.029	0	0.03	66	2152
GCCGTCATATGCG	0.058	0.009	0.049	64	1249
AGCGGTATAGGTG	0.022	0.005	0.028	26	887
CCCAGCATAGGTG	0.037	0.004	0.042	66	1522
CGTATTGTAAGCA	0.031	0.004	0.029	49	1634
ACCGTCGCAAGTG	0.029	0.006	0.032	52	1584
GCCAGTGCTTAGG	0.056	0.003	0.06	43	677
GCTAGTGCTAAGA	0.059	0.007	0.065	79	1137
ACTGTTGTAAGTG	0.029	0.009	0.016	20	1220
CGTATTGTTAAGG	0.027	0.003	0.023	23	982
GCTATTGCTTGCA	0.047	0.008	0.05	56	1072
CCCAGCATTAGGA	0.032	0.003	0.028	52	1795
ACCAGCATTAGGA	0.023	0.004	0.018	40	2123
CGTATTGCTAACA	0.021	0.005	0.016	29	1780
AGTGTCATTGGCG	0.02	0.007	0.021	18	833
GCTATTACTGACA	0.057	0.006	0.052	108	1959
CGCATTATATGTG	0.024	0.002	0.024	42	1709
GCTGTTATTAAGA	0.057	0.005	0.054	77	1355
GGTGTCGTAGACG	0.109	0.012	0.092	48	471
CCCGTCGTTTGTG	0.028	0.002	0.028	32	1111
CCCGGTACTAATG	0.027	0.003	0.03	65	2094
GCCAGTACAAGCG	0.057	0.009	0.045	79	1673
ACTGTTGTATATA	0.022	0.004	0.017	36	2081

ACTGTTGTTGAGA	0.034	0.007	0.034	43	1231
AGTATCATATGCA	0.022	0.004	0.026	54	1986
CCTGTCATTGAGA	0.029	0.003	0.03	46	1467
GGCGGTGCATGCG	0.069	0.015	0.059	39	623
ACTGGCACAAGGA	0.028	0.006	0.021	31	1421
ACCGGTACATACA	0.022	0.002	0.019	85	4420
CGTATCATAAATG	0.023	0.003	0.023	53	2261
CCCGGCATTAATA	0.024	0.003	0.02	62	2970
CGCGGTGTAAACG	0.028	0.006	0.021	29	1355
CCCGGTGTAGGCA	0.023	0.003	0.026	39	1463
CGCGGTATAGGTA	0.026	0.003	0.022	28	1228
CCCAGCACTTGCA	0.024	0.002	0.022	58	2521
ACTGTTATTTGTA	0.026	0.003	0.026	44	1637
GGTGGTATTAGGG	0.269	0.049	0.23	100	334
CGTGTTACTAGTA	0.024	0.007	0.015	17	1097
ACCGTCATAAGGA	0.025	0.002	0.023	55	2301
AGTAGCATTAAGG	0.026	0.006	0.019	21	1078
GGCAGCATAAGGA	0.053	0.006	0.051	78	1440
AGCGTCATAGGCG	0.02	0.004	0.015	22	1416
GGTGTTACATAGA	0.067	0.006	0.059	52	824
CCTATCACAAGTA	0.028	0.002	0.025	85	3303
GCCATCGTTGACA	0.046	0.002	0.047	92	1886
AGTGGCACTGGTA	0.025	0.002	0.023	18	748
GCTATTGTTGGGA	0.053	0.009	0.05	44	828
CCCGTTGCAAGCG	0.027	0.002	0.025	42	1633
AGCATCATATACA	0.023	0	0.024	113	4655
AGCGGTATTTGCA	0.02	0.004	0.018	30	1620
CGCGGTGTAGACA	0.023	0.003	0.027	41	1462
AGCATTGTTAGGA	0.022	0.003	0.019	32	1627
GCCGTTACTAGGA	0.055	0.002	0.054	58	1024
CGCATTGTTTATA	0.028	0.001	0.027	55	2007
AGTAGTACTTATA	0.024	0.005	0.021	40	1862
GCTATTGTAGAGG	0.062	0.005	0.057	54	898
CGCAGCGCAGACA	0.028	0.003	0.025	51	2011
CCTGGCGTTTACA	0.029	0.001	0.028	42	1436
GCCGTCGCATGGG	0.042	0.015	0.026	13	489
CCCGTCACTGGTA	0.026	0.002	0.029	52	1766
GCCAGTGCTAAGG	0.045	0.011	0.041	38	894
GGTATTGTTTGCA	0.116	0.03	0.075	69	853
CGCATCGCAAAGA	0.026	0.006	0.018	38	2085

ACTGGTGCAAGCA	0.026	0.006		0.027	49	1776
CGCAGCGCAAATG	0.026	0		0.026	42	1566
CGCATTACTGACG	0.026	0.004		0.022	37	1673
ACCGTCGTTAACG	0.027	0.007		0.023	37	1548
GCTAGTACATACG	0.063	0.012		0.049	69	1337
CCTATTGCATAGA	0.026	0.002		0.024	53	2171
AGCATTATTTGTG	0.019	0.005		0.012	22	1882
ACTGGTACAAATA	0.021	0.003		0.023	85	3636
GCCGTCACTAAGA	0.047	0.003		0.044	73	1605
CGCAGCGCAAATA	0.024	0.002		0.022	51	2291
ACTGGCATTAGGG	0.03	0.008		0.022	14	611
ACCATTGTAGAGA	0.027	0.003		0.024	59	2444
GCTGTTGCTGGGA	0.041	0.008		0.03	15	478
CCTAGCGCTGGTA	0.021	0.006		0.014	18	1307
CCTGGTATTAACG	0.032	0.006		0.025	41	1571
AGTGTTGTAAGGA	0.024	0.004		0.018	19	1015
AGCGGCATTTAGA	0.025	0.004		0.026	31	1142
AGCGGCGCAGATG	0.023	0.001		0.023	22	921
ACTGGTACAAACA	0.021	0		0.02	79	3787
AGCGGCATAAGGG	0.029	0.004		0.025	15	593
GGCATTGTAAGGG	0.077	0.005		0.07	65	864
ACCGTCATAGATG	0.022	0.003		0.021	48	2233
CCTGTCGCATAGG	0.029	0.003		0.025	21	822
CCTAGTATTAAGG	0.021	0.004		0.023	37	1593
GGCAGTACAGACA	0.05	0.005		0.056	114	1926
GCTATCGTAGGCG	0.063	0.004		0.063	66	988
ACCAGTACATGTG	0.023	0.001		0.023	61	2546
CCTATTACTTATG	0.029	0.003		0.027	59	2144
CGCATCATAAATA	0.026	0.002		0.023	108	4542
ACTGGTGCAGACG	0.022	0.002		0.021	25	1159
GCCAGCATAAATA	0.054	0.003		0.051	205	3821
AGTGTCGTAAGCG	0.023	0.001		0.024	20	828
CGCATCACTGAGA	0.025	0.008		0.027	51	1816
GGCATCGCAAGTA	0.08	0.008		0.073	112	1431
CGCATTACTAATG	0.024	0.004		0.022	47	2045
GGCATCATATGCA	0.079	0.014		0.072	146	1873
AGCATTGCATGTG	0.023	0.003		0.026	43	1587
CGTGTTGCAAGGG	0.021	0.003		0.016	8	481
CGCAGTATATATG	0.028	0.003		0.029	57	1906
CGCAGCATTAGGA	0.023	0.005		0.019	24	1246

GCCGGTATAAATA	0.076	0.006	0.071	212	2773
CCTATTACTTGGG	0.028	0.001	0.027	30	1077
AGCAGCACTTACG	0.024	0.001	0.025	45	1735
AGTGTCGCTGGTA	0.031	0.01	0.03	21	678
ACTGGTACAAATG	0.026	0.004	0.027	55	2002
CGTAGTGCAAGTG	0.026	0.005	0.023	17	718
CGTAGCACTGAGG	0.038	0.01	0.035	25	695
AGTGGTATAGACG	0.028	0.001	0.028	24	834
CCCAGCGCAGGGA	0.029	0.001	0.028	40	1390
CCTAGCGCTAGTG	0.032	0.007	0.024	22	899
CCTGTCACAGGCA	0.023	0.001	0.023	41	1704
AGCAGTATTGGTG	0.023	0	0.023	28	1195
ACTATCATAGGGA	0.023	0	0.022	34	1482
AGCAGCACATAGG	0.023	0.004	0.021	32	1502
CGTATTACAAACA	0.021	0.003	0.019	61	3141
CCCAGTATTAGCG	0.021	0.002	0.022	45	1993
CGTATCATTAACA	0.025	0.002	0.025	70	2691
GGTATCACTGACA	0.091	0.017	0.078	100	1184
AGTAGTACAAGTA	0.02	0.001	0.022	43	1952
CGCGGCACTTACA	0.029	0.005	0.025	42	1649
CCCGTTGCTTATG	0.028	0.004	0.025	35	1393
CCCGTCGTATATA	0.023	0.002	0.019	53	2674
CGCAGTGCTGGCG	0.021	0.003	0.017	15	859
CCCGTCGCAGGCA	0.024	0.003	0.021	42	1997
CCTATTGTTTACG	0.029	0.003	0.03	54	1761
CCTATCATATGCA	0.024	0.002	0.022	65	2912
GCTAGTACTGGTA	0.062	0.012	0.047	50	1017
CCTGGCGTTAAGA	0.023	0.005	0.021	30	1382
CCCAGTACAGGTG	0.029	0.005	0.032	54	1608
GCCATCGTTAACG	0.05	0.002	0.051	92	1708
CCTATCATATGGG	0.039	0.01	0.036	47	1262
CCTAGCGTTAGGA	0.022	0.007	0.018	19	1046
CCTGGCGCTGGGA	0.02	0.004	0.025	17	676
ACCAGTATATGTG	0.022	0.001	0.023	58	2459
AGCATTGCATACA	0.024	0.006	0.018	58	3099
ACTGTTGTTAGGG	0.034	0.005	0.027	22	785
GGTAGCATATGCA	0.076	0.006	0.07	71	940
ACCGTCGTTAGTA	0.027	0.012	0.01	16	1518
AGCGGTACTTACG	0.018	0.005	0.01	14	1326
ACCATTATTAGCA	0.024	0	0.025	101	3982

GGCATTACATGCA	0.065	0.012	0.056	106	1779
CCCATTATATAGA	0.022	0.002	0.024	91	3633
CGCATTGTTTACA	0.03	0.005	0.024	50	2047
ACCGTCACTTGCA	0.024	0.003	0.02	55	2712
GCCATCGTTGATG	0.055	0.008	0.045	55	1180
GCCGTCGCAAATA	0.046	0.004	0.05	85	1614
GCCATTACATACA	0.054	0.007	0.055	183	3172
ACCGGCGCAGGCA	0.023	0.004	0.023	30	1289
CCCATCACAGACA	0.026	0.004	0.02	95	4554
GCCATCATTGATA	0.056	0.006	0.047	131	2664
AGTGTTACAGACA	0.022	0.004	0.022	39	1721
CCTGTCACTTGCG	0.023	0.003	0.019	20	1014
ACTGTCACTGGCA	0.024	0.01	0.014	25	1754
CGTATCATTAATA	0.024	0.003	0.022	60	2679
CGTATTGTTTATA	0.025	0.007	0.016	24	1465
GGCGTTATATGTG	0.058	0.013	0.049	40	784
CCTAGCACATGTA	0.023	0.004	0.017	35	2010
GCTGTTACTGACG	0.063	0.005	0.066	62	880
ACCGGCGTATAGA	0.022	0.003	0.019	25	1266
CGTGTTACAGGCA	0.028	0.007	0.017	19	1081
CCTGTCGCTAGTG	0.02	0.002	0.02	18	891
CCTGTTGCTAGTG	0.027	0.011	0.012	10	849
GGCATCGCAAAGA	0.087	0.012	0.071	122	1603
CGCATCGTATGGG	0.026	0.002	0.024	23	948
ACTGTCACAAAGG	0.026	0.001	0.027	44	1578
CCTGTCGTATGGA	0.025	0.008	0.022	23	1040
ACTATCACTTGTG	0.026	0.004	0.03	49	1604
AGTGTCGTTTGCA	0.023	0.003	0.021	17	808
ACCATTATATACG	0.027	0.003	0.023	98	4130
CGCGGTGTTAAGG	0.03	0.01	0.034	26	746
CCCGGTGCAAGCA	0.022	0.001	0.023	51	2182
ACCAGCACTGACG	0.027	0	0.027	67	2374
AGCGGCACAGGCG	0.021	0.008	0.015	13	836
GGCATCGCATACG	0.117	0.007	0.108	161	1327
CCTATCGTTGGGA	0.027	0.001	0.025	27	1057
AGCGTTGCTGATG	0.018	0.005	0.025	27	1071
ACCATCACAAGTA	0.024	0.001	0.023	109	4588
GGTGTCATAGGGA	0.078	0.014	0.075	42	521
GCTATCATATGGG	0.068	0.019	0.057	46	764
AGCAGCATAAAGG	0.02	0.005	0.015	33	2209

CGTGTTACTAACA	0.019	0.001	0.017	32	1849
GGCATCGTATATA	0.116	0.019	0.105	200	1707
GGCATCGCATGCG	0.109	0.007	0.115	114	874
GCCATCGTTGAGA	0.043	0.006	0.039	54	1334
CGCAGTACAGACG	0.026	0.002	0.028	50	1717
ACCGGTGCTGGCG	0.024	0.007	0.015	17	1101
GGTATTACTTATG	0.112	0.019	0.094	95	920
CGCATCACTAATA	0.027	0.002	0.025	76	3012
GGCGTTATATAGA	0.059	0.004	0.054	80	1400
GCCGTCGCTTGTG	0.038	0.012	0.025	14	551
CGCGGTGTTAATA	0.021	0.003	0.017	26	1482
CGTGGCACTGACA	0.034	0.01	0.03	36	1161
GGTAGTGTTTAGA	0.054	0.006	0.06	40	622
CCTATTATAGGGG	0.047	0.003	0.045	51	1087
ACTGGCGTTAATG	0.036	0.013	0.04	24	580
AGTATTACATGGG	0.023	0.004	0.027	26	949
ACTGTCGTATGGG	0.022	0.004	0.019	12	606
ACTGGCGCAAGGA	0.025	0.004	0.022	14	612
CCCAGTACAGACG	0.027	0.004	0.023	62	2671
ACTAGTATTGGCG	0.025	0.001	0.024	40	1630
CCTAGCATTTGCA	0.023	0.006	0.019	32	1667
GCCGTCATTAGCG	0.051	0.002	0.052	66	1203
CCTATTATATACG	0.029	0.003	0.025	77	3004
GGTAGCGCTAGGG	0.079	0.015	0.072	23	298
ACCAGTACATACA	0.022	0.003	0.019	125	6374
CCCGTCACTGGGA	0.031	0.007	0.03	42	1370
AGTGTCACTGGTA	0.025	0.005	0.032	27	826
CGCGGTACAGAGA	0.025	0.003	0.021	27	1269
AGTAGCATATACG	0.027	0.001	0.029	46	1563
AGCAGTACAAAGG	0.021	0.004	0.016	34	2121
GGCAGCGTTTGGA	0.063	0.018	0.039	27	658
CGCAGTACTAAGG	0.026	0.003	0.023	29	1255
CCTGGCACTGAGG	0.026	0.006	0.027	21	757
GGTGGCACTGAGA	0.198	0.042	0.169	100	490
GCCAGCATAGGCG	0.065	0.006	0.06	73	1145
GGCAGTATTGATA	0.055	0.014	0.044	87	1870
AGCGGTGTAAGCA	0.023	0.005	0.017	30	1695
CCCATTACAGACG	0.025	0.002	0.028	88	3055
AGTGGCATTAATG	0.026	0.01	0.025	9	348
GCCATTGTAAGTA	0.047	0.005	0.042	86	1985

AGTATTATAAATA	0.024	0.001	0.024	111	4589
GGTAGTACAGGTA	0.054	0.002	0.057	49	810
ACCAGCACTTACA	0.022	0.001	0.02	102	4932
GCTGTCACTAATG	0.072	0.01	0.066	73	1033
ACTGTTATTAATA	0.022	0.003	0.018	61	3251
CGCGGTGCTGGCG	0.018	0.005	0.011	7	655
AGCATTATAAAGG	0.024	0.001	0.023	67	2841
GCCAGTATATACG	0.046	0.005	0.049	117	2256
GCTATTGCTAACG	0.057	0.006	0.052	76	1381
GCTATCACAGAGA	0.063	0.007	0.066	95	1348
CGTGTTGTAAGCA	0.022	0.004	0.017	20	1155
CGTGGTACTGGCG	0.033	0.009	0.045	23	493
GCCGGTACTGAGA	0.059	0.005	0.061	70	1074
CCCATTATATGGA	0.027	0.003	0.023	60	2598
CGCATTACTAAGG	0.028	0.003	0.026	38	1439
CCTATTACTGACG	0.03	0.004	0.035	71	1985
CGTATCACTGACA	0.03	0.005	0.023	40	1712
GCCGTTATTTGTA	0.054	0.006	0.047	69	1411
GCTGTCATAGGCG	0.079	0.012	0.068	54	740
GCCATCGTTAACA	0.052	0.008	0.061	150	2311
GGCAGCATAGATG	0.049	0.006	0.044	62	1362
GCTGGTATTAGGA	0.144	0	0.144	120	713
AGTAGTGTAGGTA	0.022	0.007	0.018	19	1034
CGCAGTGTATGTA	0.016	0.004	0.011	17	1484
GGCGTTATAGGTA	0.063	0.003	0.065	76	1099
AGCGTCACATACG	0.024	0.005	0.024	50	2032
CCTATTGCATGCG	0.03	0.004	0.027	41	1493
ACTAGCACAGACG	0.027	0.001	0.027	56	2037
AGTAGCATTTGTG	0.015	0.001	0.016	13	819
CGCAGCACTGGTG	0.025	0.005	0.032	27	812
GGCATTGTAAGGA	0.066	0.003	0.065	84	1214
CCCGTCACAGATG	0.025	0.002	0.022	47	2042
AGTAGTGTTTGCG	0.029	0.006	0.02	14	692
GGCGGCACTTGCA	0.117	0.016	0.102	102	902
GCTGTCATTGGGG	0.063	0.007	0.07	31	409
AGCATCATTAGGA	0.022	0.004	0.017	38	2205
AGTGTTATATAGG	0.02	0.006	0.019	21	1063
AGCGTTATTGGCA	0.017	0.001	0.017	29	1650
ACCGGCGTATACG	0.022	0.004	0.016	16	1004
GGCAGCATAAGTA	0.052	0.005	0.051	95	1768

GGTAGCACTTAGA	0.075	0.016	0.077	61	732
GGTATCGTAAATG	0.141	0.015	0.128	136	928
ACCAGCATTGGGG	0.032	0.004	0.037	37	958
GCCATCATAGACA	0.055	0.006	0.048	170	3340
GGCATTGTAAGCA	0.071	0.019	0.055	99	1705
GGTAGTATAGACG	0.072	0.015	0.055	55	941
CGCGGTGCAGGCG	0.028	0.003	0.024	20	798
CCTATTGCTTGGG	0.035	0.004	0.032	27	828
ACCGGCGCATAGG	0.022	0.005	0.015	17	1090
GGCGGCATTAACG	0.565	0.052	0.535	908	788
ACCGGTGCTGACA	0.026	0.002	0.024	52	2097
AGCAGCGCAAGCA	0.022	0.004	0.016	34	2065
ACCGTTATTGATA	0.025	0.002	0.022	69	3003
CCCAGCACTGGGA	0.034	0.008	0.026	36	1339
ACTATCGTAAACG	0.031	0.006	0.023	59	2503
AGCGTCGTATATG	0.024	0.007	0.021	31	1458
CGTAGCACTGACG	0.024	0.002	0.026	24	896
CCTGTTGTTGGTG	0.019	0.003	0.02	17	853
ACTGGCGCTGGGA	0.029	0.007	0.022	10	452
ACTAGCGTAGACA	0.026	0.004	0.022	44	1990
AGTATTGCTTGTG	0.023	0.002	0.023	20	847
ACCATTATAAAGG	0.024	0.001	0.025	102	3969
ACTGGTGTAGACG	0.024	0.003	0.026	28	1031
ACCAGCATAGACG	0.023	0.002	0.021	65	2987
CCTAGTATATACG	0.027	0.007	0.025	58	2271
CCTATCGCAAGCA	0.023	0.004	0.02	54	2599
CCTATCACTTGTG	0.024	0.007	0.015	22	1418
AGCAGCACTAGTA	0.024	0.004	0.029	59	1981
AGTGGTGTTAATA	0.023	0.006	0.018	23	1270
GCTATTGCTGATA	0.052	0.003	0.053	80	1420
CCTGGCATATAGG	0.026	0.007	0.019	18	942
CGTGGCATATAGA	0.025	0.003	0.022	19	847
GGTGTCATTTATG	0.107	0.017	0.094	59	571
AGTGGCATAGGGG	0.011	0.005	0.004	1	237
AGCGTCACAAACG	0.02	0.001	0.019	48	2508
GCCAGCGCTGACA	0.047	0.008	0.041	61	1419
ACCAGCATAGGCA	0.027	0.002	0.027	81	2975
GCCGTCGTAAGCG	0.055	0.009	0.049	45	873
CGCATCATAAGCG	0.027	0.002	0.025	58	2253
AGTAGCGTTTACA	0.026	0.001	0.027	36	1288

GGTGTCACTTGTG	0.086	0.023	0.081	35	398
ACCATTGTTTGCG	0.024	0.002	0.022	36	1567
GGCATTGCTGGGG	0.075	0.008	0.068	36	492
AGTGTTGTAAATA	0.022	0.004	0.026	54	2047
AGCGGCACAAGGA	0.025	0.009	0.033	46	1336
AGCATCGTTTACG	0.023	0.004	0.028	55	1938
GCTGGCATTGGCG	0.844	0.026	0.829	848	175
ACCGGTGCAAGTG	0.027	0.004	0.023	32	1358
CCTATCATTGACA	0.024	0.001	0.024	81	3263
ACCAGTGTTGATG	0.027	0.003	0.023	39	1678
GGTAGTGCATGTG	0.046	0.003	0.047	22	447
CCTATTATTTATA	0.026	0.002	0.024	83	3346
GCTAGCACTTGTA	0.064	0.016	0.045	46	985
CCCGTCACTGATG	0.027	0.006	0.018	33	1802
AGCAGCACAAGCA	0.019	0.003	0.02	55	2639
CGTGTCGCAGATA	0.027	0.005	0.032	35	1055
ACTAGCACATGTG	0.027	0.002	0.024	40	1611
CGCGTTGCTAAGA	0.024	0.009	0.025	32	1239
ACCGGCGTAAATA	0.024	0.003	0.021	48	2278
GCTATTATAGAGA	0.061	0.01	0.048	91	1813
GGCGTCGTAGAGA	0.072	0.018	0.055	49	847
GGTATCGCATAGA	0.096	0.01	0.093	81	792
GCCGTTACAAACA	0.056	0.006	0.06	178	2780
CGCGGTACAAAGA	0.025	0.001	0.025	49	1903
CCTATCGTAAGTA	0.02	0.003	0.019	44	2278
ACCGTCATTTGCA	0.026	0.003	0.026	60	2288
ACTAGCACAAGGA	0.025	0.003	0.028	59	2085
CGCATTGTTGATG	0.021	0.005	0.014	19	1335
GCTGGTGCTTGTG	0.305	0.041	0.263	113	316
CCTATTGCAAGGG	0.029	0.004	0.027	37	1344
GCCGTTATATATG	0.058	0.01	0.044	77	1681
ACTGGCGTATGTA	0.028	0.003	0.03	31	990
CGCGGTGTAAGTG	0.028	0.007	0.02	17	846
ACTAGTATTGAGA	0.029	0.002	0.031	69	2125
GCTATCGTAAACG	0.056	0.008	0.048	81	1620
CCTATTATAGACG	0.026	0.002	0.023	62	2611
CCCATTGTTTATA	0.024	0.001	0.023	64	2755
CCCGTCATAGGTA	0.027	0.003	0.026	54	2020
ACTATCGCTTAGG	0.028	0.003	0.025	31	1223
CCTATTGTTTGGA	0.032	0.006	0.034	46	1300

ACTAGCGTTAGCA	0.024	0.007	0.023	37	1561
GGCAGCACTAATG	0.058	0.008	0.048	67	1316
CCCATCGCTAAGG	0.024	0.007	0.016	30	1807
CCTATCACAGGGA	0.035	0.009	0.029	48	1599
AGCGGCATATAGA	0.024	0.002	0.022	30	1356
AGTAGCATTGACG	0.025	0.003	0.021	22	1011
ACTGGTACATGGA	0.022	0.004	0.027	39	1381
GGTGGTACAAATG	0.1	0.013	0.083	68	752
CCTATTATAGGCA	0.023	0.003	0.027	72	2605
CCTGGCATTTAGA	0.029	0.005	0.027	34	1203
CCCAGCATTAATA	0.026	0.002	0.029	119	4040
CGCGGTGTAAACA	0.024	0	0.024	49	2025
GCCGGTACATAGG	0.071	0.007	0.065	58	832
CCTGGCGTTGACA	0.025	0.005	0.025	42	1644
AGTATCACTGGGG	0.021	0.012	0.015	8	541
GGTAGTGCAGAGA	0.054	0.009	0.05	30	570
ACCGTTACAAGTA	0.024	0.002	0.022	76	3329
ACTGGCATTTGGA	0.032	0.008	0.032	31	940
ACCGTTACATGCG	0.025	0.004	0.02	49	2362
CGCGGCATTAACA	0.025	0.002	0.027	56	2028
CGCAGTATTGAGA	0.031	0.005	0.032	56	1674
CGCGGCGTAAAGG	0.026	0.002	0.028	24	837
ACCGTCGCATACG	0.025	0.003	0.027	54	1923
ACTAGTACATGGA	0.023	0.001	0.022	47	2078
CCTATCGCAGAGA	0.029	0.003	0.026	51	1921
GCCATTATATGTA	0.048	0.002	0.045	132	2819
ACTATCACTTAGG	0.025	0.001	0.024	42	1721
AGCGTTACATAGG	0.021	0.005	0.016	22	1366
CGCAGTATAAACG	0.025	0.001	0.026	70	2663
CCTATCATAAGCA	0.028	0.002	0.025	89	3483
ACTGTCGTAAGCG	0.023	0.001	0.022	23	1013
GCTGTTATAAGTG	0.061	0.011	0.05	53	997
AGTGTTGTAAGTG	0.024	0	0.025	23	890
ACTGTTGTAAGGA	0.03	0.004	0.025	35	1346
GCTGGTGTTAGTA	0.388	0.034	0.353	317	582
AGCATCGTTGACA	0.022	0.003	0.018	48	2656
GCCGTTGTAAGCA	0.051	0.005	0.05	79	1493
GCCATCACATAGG	0.056	0.001	0.054	88	1530
GCTATCATTGACG	0.064	0.005	0.06	90	1422
ACTATTACAGAGA	0.023	0.002	0.025	84	3280

ACCGGCGCATGGG	0.022	0.003	0.023	19	790
CGTGGTATAGAGA	0.025	0.004	0.02	20	975
CGCGTTGCAAATA	0.026	0.001	0.028	58	2032
GCCATTGCTAATA	0.039	0.005	0.045	95	2037
AGTGTTGCTGAGA	0.019	0.002	0.022	18	797
CGCGGTACAAGTA	0.023	0.003	0.021	34	1616
GCTAGTATATGGA	0.066	0.006	0.061	75	1156
AGTGGTGCTTATA	0.024	0.006	0.018	17	954
CCCGGCGCTTATA	0.027	0.006	0.036	61	1641
ACCAGTATATGGG	0.024	0.003	0.021	37	1713
GGTATCACTTGCA	0.106	0.015	0.1	99	892
GCTATTGCATGCG	0.062	0.009	0.052	51	921
GCCGTTATTGACG	0.058	0.005	0.052	75	1365
CCTATCATTTATA	0.027	0.003	0.027	92	3354
GCTATTGCTGGCA	0.052	0.01	0.043	44	975
CCTAGCGCTAGGA	0.026	0.013	0.018	24	1278
CCTGTCGCAAGGA	0.024	0.004	0.018	24	1312
AGCAGTATTAAGA	0.019	0.004	0.018	59	3256
AGTGGCGCATACA	0.026	0.005	0.026	31	1173
CGCATTATATAGG	0.021	0.004	0.019	31	1587
CCCGTCATTGGCG	0.038	0.005	0.042	62	1403
ACTGTCGCTTGGG	0.02	0.004	0.023	15	633
CGTGTTGCATATA	0.025	0.008	0.025	29	1117
GGCGTCACAAAGA	0.059	0.012	0.061	104	1614
ACTAGTATATGTA	0.029	0.004	0.025	69	2703
ACTGGCGCAAATA	0.025	0.004	0.022	30	1305
ACTAGTACAGATG	0.021	0.002	0.024	58	2355
ACTATCATTAATA	0.028	0.003	0.026	111	4174
CCCGGCACTAGCA	0.022	0.002	0.019	43	2231
CCTAGTATTTAGG	0.027	0.004	0.023	27	1136
ACCATCGTTAGGA	0.021	0.003	0.026	48	1784
CCCGTCACTTAGG	0.025	0.005	0.029	39	1319
CGCATTATAGGGG	0.054	0.005	0.053	50	885
AGTGGTGCAAACA	0.024	0.002	0.027	42	1517
AGCGTTACATGCG	0.024	0	0.024	33	1344
GCTATCACTGATG	0.063	0.011	0.075	98	1210
GCTATTGCATACA	0.05	0.005	0.048	87	1716
CCCAGTGTATGCG	0.026	0.005	0.026	42	1554
GCTGGTGTATGTA	0.429	0.053	0.414	414	586
CCTGGCATTTGGA	0.025	0.005	0.018	16	852

ACTATCATTTGGG	0.034	0.004	0.035	45	1253
ACTGGTGCAGACA	0.021	0.003	0.023	47	1979
CCTATCATAAGGG	0.036	0.003	0.031	46	1435
CGCATTGTATGTA	0.025	0.005	0.02	35	1708
CGTATCATTTGTG	0.021	0.003	0.02	19	910
CGTGGTGCTGATG	0.027	0.003	0.03	17	545
GGTGGTATAGACG	0.28	0.036	0.27	226	611
GCTATTGCAAGCG	0.058	0.005	0.056	66	1122
ACTGTTGCTAAGA	0.024	0.001	0.025	46	1762
CGCGGTGTTGAGG	0.026	0.002	0.029	18	604
GGTGGCATAAATA	0.474	0.041	0.47	918	1035
GGCAGCGCTGATG	0.072	0.015	0.093	74	725
GCCGTCGTTAACG	0.053	0.006	0.044	47	1017
GGCAGCGTAAATG	0.053	0.005	0.049	69	1328
GGCATTGCATATG	0.075	0.013	0.059	69	1104
GGCGGTACAAGCG	0.091	0.011	0.092	85	841
GCTGGCATAAGTG	0.859	0.024	0.858	1314	217
AGCATCGTAAATA	0.021	0.001	0.022	89	3946
AGCGGCGCATGTG	0.025	0.005	0.03	24	775
AGTAGTACATACG	0.027	0.004	0.032	51	1520
AGCGTCATAAAGA	0.021	0.003	0.019	55	2862
GGCATTACATAGA	0.055	0.006	0.047	89	1812
GGCGTTGTTGAGG	0.082	0.016	0.06	33	521
GGCGGTATAGATA	0.081	0.01	0.086	123	1308
CGCGTTGCATGTG	0.029	0.004	0.025	21	829
CGTGTTGCTTATG	0.025	0.003	0.024	16	653
CGCGTCACTAGCG	0.021	0.005	0.027	36	1300
GGCAGCGTTAGGA	0.061	0.018	0.051	37	694
ACCGGCATATGCA	0.023	0.004	0.018	43	2315
CCTAGCGTAGATA	0.019	0.004	0.015	30	1920
CCCAGCGTTTACA	0.023	0.001	0.021	59	2691
GCCGTCGCATGGA	0.044	0.007	0.041	28	647
AGCGGTACTGGTG	0.017	0.006	0.016	13	805
GGCGTTGTAGACG	0.057	0.013	0.041	41	947
GGCAGTACAGGGG	0.044	0.012	0.052	35	642
ACTGTCGTTGGCA	0.024	0.004	0.019	20	1022
CGCATTGCTAGTG	0.025	0.005	0.024	26	1053
AGTGGCGCAGACA	0.022	0.002	0.021	22	1025
CCTAGTATTAATA	0.03	0.004	0.025	83	3248
ACCAGCATATATG	0.022	0.001	0.023	73	3115

CCTGTCGCATATA	0.026	0.004	0.021	43	1999
ACCGTTATTTACG	0.024	0.003	0.024	51	2114
CGCGTCGCTAACG	0.023	0.003	0.019	26	1370
CCTGTCATTAACA	0.027	0.003	0.026	72	2711
ACCGTTGTTGGGG	0.026	0.003	0.022	17	752
GGTATTGTATGTA	0.1	0.01	0.088	90	937
CGCATTGTATACG	0.025	0.002	0.022	43	1871
CGTAGCGTTAATA	0.032	0.002	0.035	47	1315
CCCATCACATACG	0.027	0.003	0.028	98	3389
GGCGTCGCTAATA	0.06	0.01	0.048	62	1233
CCTATTGCTAGGG	0.031	0.005	0.025	27	1044
GGTAGTACAGATA	0.063	0.006	0.055	70	1210
ACTGGTACTTATG	0.023	0.002	0.019	26	1319
GGCAGTATTTGTG	0.064	0.006	0.058	56	907
GGTAGTGCAAGCA	0.051	0.012	0.04	33	797
GGCGGTGCAAACG	0.059	0.014	0.048	51	1011
GGCATCACTGAGG	0.079	0.007	0.07	69	910
GGCAGCACATACA	0.044	0.005	0.041	87	2012
GGCAGCACATGTG	0.057	0.004	0.053	57	1010
ACCGTTGTTTGGG	0.023	0.001	0.024	20	812
GCTGTTATAGGTG	0.063	0.013	0.064	50	731
GGCGTTGCTTATG	0.072	0.008	0.063	45	664
AGTGTCATTAGGG	0.019	0.006	0.026	18	674
ACCGGCATAAATA	0.025	0.007	0.018	76	4045
GCCATCGCAGGCA	0.049	0.005	0.044	65	1417
CCCGGTGCTTGTG	0.025	0.004	0.023	18	781
AGCATTGCAAGTG	0.023	0.003	0.019	36	1871
CCCGGCATATAGA	0.027	0.007	0.036	82	2193
CGTGTCACTTAGA	0.027	0.006	0.021	18	831
GGCAGCGTTGATG	0.061	0.011	0.055	49	840
GGTATCATTGACG	0.116	0.013	0.097	88	818
GCTAGCACTGACA	0.066	0.007	0.058	92	1488
GCCAGTACTAAGG	0.042	0.004	0.048	70	1401
GCTATTGTAAATG	0.07	0.01	0.055	102	1738
CCTATCACTAGCA	0.021	0.001	0.021	58	2755
CGCAGCGCATAGG	0.027	0.007	0.032	32	966
GCCAGCATTGACG	0.073	0.005	0.068	102	1391
GGCGGTATATGTG	0.108	0.024	0.091	75	751
GCTGTTACAAGCG	0.069	0.007	0.064	64	934
GCCGGTGTATAGA	0.188	0.051	0.169	196	966

AGTGTTACATGGA	0.028	0.004	0.025	24	951
GGTAGTGCTAATG	0.063	0.013	0.049	34	661
AGCAGCGTAAACG	0.019	0.002	0.018	41	2190
ACTATTGTTAGGA	0.023	0.006	0.019	33	1680
ACCATTACTTGGA	0.023	0.004	0.02	57	2764
ACCGGTGCATGTG	0.025	0.004	0.021	23	1072
GGCATTGCTGGCA	0.061	0.007	0.064	67	982
GCCGTTATTGATA	0.056	0.005	0.051	101	1874
ACTAGTGTATGCA	0.028	0.008	0.017	36	2106
GGCGTCGCAGACA	0.058	0.003	0.056	77	1307
GGTATTATTGAGG	0.11	0.008	0.101	79	704
ACCAGTATTGGCA	0.023	0.002	0.025	74	2834
CCTGTTGCAGGCG	0.032	0.011	0.024	24	965
CCCATTGTTGATG	0.026	0.004	0.023	45	1937
ACCAGCGCATGGA	0.019	0.006	0.013	26	2016
GGTATTACTGACG	0.096	0.024	0.067	77	1068
CCTAGTGCTGAGG	0.025	0.002	0.022	21	932
CCTATTGCAAGCG	0.025	0.003	0.029	53	1801
CGCGTCGCAGGGA	0.031	0.011	0.017	14	830
GCCAGCGTTAAGA	0.055	0.005	0.049	69	1341
AGCGGCACTTATA	0.025	0.005	0.018	29	1577
CCTGTTGTAGGCG	0.023	0.004	0.018	21	1167
AGCAGCATTTGGA	0.024	0.003	0.026	41	1529
AGTATCGCAAAGG	0.028	0.003	0.031	33	1035
CGCGGTACTTGGA	0.023	0.003	0.026	24	910
AGTGTCGCAGATG	0.026	0.005	0.029	22	726
CCCGGCGTTTGGG	0.027	0.001	0.026	17	637
AGTGGTGCTGACA	0.021	0.001	0.019	18	914
GCTGGCACAAATA	0.212	0.021	0.203	413	1625
ACCAGCACAAAGG	0.023	0.004	0.024	72	2876
CGTGGTACTAAGA	0.03	0.01	0.017	17	993
GGTATCATTTATA	0.133	0.015	0.118	183	1362
ACCGGCGCTGAGG	0.025	0.002	0.026	24	904
CGTATTGTAGGTG	0.026	0.005	0.028	22	778
CCCGTCATATGTG	0.022	0	0.022	38	1701
CGTGGTATAAGTA	0.026	0.006	0.021	25	1158
CCTGGCATAGGCA	0.027	0.002	0.025	36	1417
GGCAGTGTAAAGG	0.061	0.008	0.054	61	1070
ACCGTCACTAAGA	0.022	0.002	0.025	80	3185
AGCAGTACATATG	0.021	0.002	0.02	47	2299

GCTGGTATAGAGA	0.151	0.018	0.165	167	844
AGTGTCACTAATA	0.026	0.003	0.029	52	1716
ACCGTCGTTGATA	0.024	0.001	0.022	40	1761
GCCGTTATATGTG	0.052	0.014	0.033	41	1192
CGCGGTGCTAATA	0.03	0.006	0.031	47	1468
ACCGGTATATGGA	0.023	0.001	0.023	44	1868
CCTGGTATAAAGA	0.025	0.002	0.022	46	2083
CGTAGTGCTTGCG	0.024	0.007	0.017	8	463
CGCGGTGTAGGCA	0.018	0.002	0.02	21	1049
GCTAGTGCTTGGG	0.054	0.014	0.053	23	407
GGCGTTACTAACA	0.058	0.01	0.05	102	1946
AGTGTCATATGCA	0.024	0.004	0.021	29	1377
GGTAGTACTAATA	0.063	0.009	0.051	73	1362
CCCGGCATAAGCA	0.024	0.003	0.019	56	2890
CCCATCGCATAGA	0.025	0.005	0.022	57	2554
AGCATCGTATGTG	0.024	0.006	0.022	34	1482
ACTGTTACATGCG	0.029	0.007	0.019	28	1428
CGTGTCGCTTGTG	0.04	0.008	0.039	19	463
GGCAGTGTTAGGG	0.054	0.004	0.049	27	524
GCTAGTATTTGGA	0.065	0.006	0.061	59	905
ACCGTCATAAGCG	0.029	0.004	0.024	56	2301
GCTAGTGCTGGGA	0.053	0.003	0.053	30	535
CGTAGCACTTGCG	0.034	0.002	0.033	21	616
AGCGGCGCAAGCG	0.024	0.003	0.022	22	987
AGCGGTGCTAAGA	0.021	0.006	0.021	28	1287
GGCAGTACAGATA	0.062	0.006	0.057	113	1884
ACCGGTGCTTGGA	0.019	0.004	0.024	28	1133
AGTGGCGCATGGA	0.024	0.002	0.025	14	546
GGTAGCGTAGGCG	0.074	0.006	0.072	35	449
CCCAGCGCAAGCA	0.023	0.001	0.024	80	3207
CCCATTACAAGGA	0.025	0.002	0.027	76	2751
GCCGTTGCTAGTG	0.043	0.004	0.046	31	639
CGTATTGCTTATG	0.028	0.003	0.024	21	863
AGCATTGCTAGTA	0.023	0.004	0.028	56	1913
AGTATCATATGGA	0.024	0.004	0.02	29	1428
CGTATTATATATG	0.029	0.003	0.025	46	1781
ACCGGCGCATACA	0.024	0.004	0.018	33	1759
AGTAGCACTAACA	0.028	0.006	0.02	43	2127
CGCATCACAGGCG	0.024	0.004	0.021	36	1688
GCTGTCGTTAAGA	0.051	0.01	0.037	36	932

GGCGGTGCATGTG	0.066	0.014	0.047	28	569
CCTGTCGCAGACA	0.02	0.003	0.016	34	2046
GCCATTATAAGCG	0.052	0.001	0.052	124	2266
GCCGGCATAGGGG	0.462	0.073	0.439	363	463
CGTATCGTATGGA	0.026	0.003	0.024	24	981
CCTGTCACAAGCG	0.029	0.008	0.02	29	1441
CGTGTTACATATG	0.024	0.002	0.027	29	1048
CGTAGTATTGGTG	0.021	0.005	0.028	17	591
GGCATTGCTTGCG	0.075	0.008	0.063	57	852
CGCATCGCAGAGG	0.028	0.003	0.025	25	985
ACCGGCACAGATG	0.025	0.003	0.021	45	2097
CGCATCGTTAACG	0.026	0.001	0.025	50	1986
CCTGTCGTAAATA	0.024	0.002	0.023	63	2618
GGTATCGCAAATG	0.133	0.009	0.12	119	869
CCCATCACTAGTG	0.028	0.006	0.019	41	2101
CGCATCGTAAGGG	0.028	0.007	0.027	31	1132
CGTATCATTGGCA	0.024	0.002	0.024	34	1364
ACCGGTACTTGTA	0.019	0	0.018	45	2390
CGCGTCACAGACG	0.028	0.001	0.028	47	1659
AGCAGCATTGATA	0.021	0.002	0.022	63	2863
CCCGGCATATATG	0.023	0.004	0.026	54	2037
AGCAGCGCAAAGA	0.025	0.001	0.025	50	1966
CCCGTTATTGGGA	0.022	0.001	0.023	31	1341
GCCGGTGTATAGG	0.381	0.073	0.345	275	523
AGCGGTGCAGGCA	0.02	0.004	0.024	29	1172
AGCGTCGTAAATA	0.019	0.005	0.014	39	2677
ACCGGTGCATAGA	0.022	0.002	0.023	44	1853
CGTGTTGTATGGA	0.018	0.001	0.016	11	660
GGTATCGCATATG	0.134	0.026	0.114	89	695
ACCGGCGCTAAGA	0.026	0.003	0.023	29	1258
CGCAGTGTTTGCA	0.029	0.007	0.02	24	1187
CCTAGCACTGACG	0.036	0.004	0.03	49	1587
CCCAGTGCAGAGA	0.027	0.008	0.028	55	1941
ACCGTCGCAAACG	0.021	0.002	0.023	57	2386
CCTGTTGTAGAGA	0.024	0.003	0.027	40	1440
CGTAGCACTAGCA	0.023	0.005	0.023	26	1114
GGTGTCATTAATA	0.086	0.006	0.08	106	1220
AGCGGTACAAACG	0.026	0.007	0.034	71	2022
ACTAGCGTAAGTA	0.022	0.002	0.024	46	1860
CCTGTTATTTAGA	0.027	0.003	0.027	45	1606

CGCATTGCTAAGA	0.02	0.004	0.016	27	1683
CCCAGCGTTTAGA	0.022	0.005	0.021	38	1798
GCCGGTGCTAGCA	0.064	0.008	0.052	52	940
GCCGGTATAAGCG	0.168	0.027	0.143	194	1158
CGCGGCGTTAACA	0.025	0.002	0.025	40	1556
CGTGTTGTTGATG	0.022	0.001	0.021	14	649
GCTGTCATTTGGG	0.08	0.008	0.07	37	490
CCTAGTACTGACG	0.025	0.009	0.019	31	1624
CCCGTCACTAGTA	0.023	0.002	0.026	59	2245
CGTAGCGTAAGGG	0.025	0.005	0.031	19	594
CGCATTACTGGCA	0.03	0.002	0.03	52	1675
AGTGGTATTTACA	0.02	0.003	0.024	33	1331
CGTATCACTAAGA	0.023	0.008	0.014	22	1575
CCTATCACAGGTG	0.033	0.004	0.029	48	1585
CCTAGTACTAGCA	0.028	0.004	0.032	69	2117
ACTAGCACTGAGG	0.024	0.005	0.022	28	1229
ACTATTGCTTGCG	0.024	0.004	0.023	36	1553
ACTAGTGTTAGGA	0.027	0.003	0.025	36	1422
ACTGGCGCAGACA	0.028	0.005	0.022	23	1043
GCTAGCACAAGCA	0.062	0.007	0.055	85	1467
GCTGGCACTAGCA	0.197	0.015	0.195	204	843
GCTGTTATATGTG	0.062	0.007	0.07	59	788
CGCGGTGTTGGTA	0.034	0.004	0.039	31	758
CGTAGCACATGCA	0.027	0.003	0.023	31	1342
CGTGGCGCAGGCA	0.021	0.007	0.011	8	706
CCCGTCGTTAGGA	0.027	0.005	0.033	42	1243
GGCATCGTTGGCG	0.075	0.016	0.056	56	953
AGCATTACATACG	0.021	0.004	0.015	46	3039
CCCGTTACAAGCA	0.023	0.003	0.021	66	3026
CCCGGTGTATGGG	0.022	0.005	0.025	20	792
CGCGGCATTGACG	0.029	0.002	0.03	32	1038
ACTAGCATAGGTA	0.031	0.004	0.029	60	2000
GGCATTGTTGATA	0.066	0.005	0.06	98	1523
CGTGTCGCATACA	0.022	0.007	0.013	17	1316
GCCGGTATTTACG	0.155	0.022	0.145	185	1088
CGTGTTATAGATA	0.025	0.002	0.022	37	1626
GGTGTTGTAAATG	0.095	0.029	0.065	59	842
CCCGGCGCTGGTG	0.028	0.005	0.022	19	847
GCCAGTATATGGA	0.058	0.004	0.053	90	1621
CGTGTTGCATGGA	0.025	0.009	0.013	9	701

CCTAGCGTAGGTG	0.026	0.007	0.02	18	881
ACCGTCGTAGACA	0.024	0.001	0.024	56	2312
GCTGTCACATAGA	0.057	0.002	0.055	59	1006
ACTATTATAAAGG	0.029	0.004	0.024	79	3190
GGCAGCGCTGGTA	0.054	0.012	0.04	34	817
CCTGTTATTAGTA	0.024	0.006	0.032	56	1703
ACCATTGCAGGTG	0.021	0.002	0.023	42	1749
CCCGGCGCATGCG	0.017	0.002	0.017	24	1395
AGTGGTGCTAACG	0.025	0.006	0.022	19	854
ACCGTCGTAGATG	0.025	0.001	0.026	35	1314
CCTGTCGTATACG	0.026	0.004	0.022	33	1443
AGTGTCGCATGTG	0.022	0.007	0.012	8	641
CGCGTTATTTATG	0.035	0.006	0.04	57	1367
CGTGTTATAGAGG	0.028	0.004	0.025	22	843
CCCGGCATTGGCA	0.029	0.003	0.024	45	1801
ACTATTGTTTATG	0.034	0.006	0.026	52	1953
GCTAGTACTGATG	0.069	0.005	0.07	72	955
GGCGTTGTATACG	0.069	0.003	0.071	72	937
CGTAGCGCTGGGA	0.023	0.012	0.007	4	541
CCTGTTGTTAAGG	0.027	0.003	0.026	32	1187
GCTAGCACTTGGA	0.059	0.009	0.054	38	672
CGTATCATAAGTA	0.022	0.004	0.017	39	2190
AGTAGTATTTGCG	0.024	0.003	0.02	19	933
CGTAGCATATGTA	0.024	0	0.024	32	1306
CCCGGCACAAACA	0.027	0.001	0.029	123	4092
CGTATTGCAAATA	0.03	0.004	0.025	53	2081
CGCGGTATTAATG	0.029	0.002	0.031	44	1359
CCCAGTGCATGGG	0.025	0.005	0.02	20	1002
GCTATTATAAGGG	0.074	0.003	0.073	84	1067
CCCAGTGCTTAGA	0.026	0.004	0.021	37	1757
GCCATCACATGTG	0.056	0.007	0.048	76	1506
CGCAGCATTAATG	0.029	0.001	0.031	60	1877
CGCGTTGTAGGCA	0.02	0.004	0.021	25	1149
ACCAGCGCTGGTA	0.021	0.004	0.015	34	2187
AGCGTTATAGACA	0.022	0.002	0.021	62	2882
CCCAGCGTAGACA	0.022	0.005	0.016	51	3080
CCTATCGTTTGTA	0.021	0.002	0.021	36	1697
CGTATTGCTGGTA	0.029	0.007	0.02	18	881
GCCGTTACTGGCG	0.048	0.004	0.043	43	952
AGCAGCACTAACG	0.019	0.002	0.017	40	2354

ACTAGCGCTAGGA	0.026	0.005	0.02	26	1303
CCTAGCGTTTATG	0.024	0.004	0.023	29	1217
CCCATTGCATGCG	0.026	0.004	0.022	42	1874
ACCAGCGCTAGGG	0.024	0.003	0.023	28	1181
GCCAGTATTTACG	0.05	0.003	0.048	98	1952
GCCGTCGCAGAGA	0.052	0.02	0.025	21	815
AGCGTCATAGGTA	0.023	0.003	0.021	38	1749
CCCATCGCTTGGA	0.022	0.002	0.025	39	1530
CGTATCGTATGGG	0.023	0.002	0.02	15	724
ACCGGCGCTAACG	0.026	0.006	0.023	38	1611
ACTGTCGTTTGCA	0.031	0.005	0.031	36	1111
ACCGTTACTAGTA	0.023	0.002	0.02	61	2923
AGCGTTATATGCG	0.026	0.003	0.022	36	1573
AGCAGCGTTTAGG	0.025	0.003	0.028	27	927
GCCAGTATTAATG	0.049	0.008	0.038	87	2178
CCTAGTGCTAGGA	0.027	0.009	0.017	22	1309
ACCGGCATATGGA	0.026	0.005	0.02	33	1608
AGTGTTGCTGAGG	0.025	0.004	0.023	12	517
AGTATTGTAGACG	0.024	0.003	0.027	39	1429
ACCAGTGTTTATA	0.026	0.002	0.027	71	2553
ACTGGTGCATACA	0.022	0.005	0.017	36	2060
ACTGGCGTTTACA	0.029	0.009	0.016	20	1218
GCTGGTACAGAGA	0.072	0.016	0.056	46	771
AGTGTTACAAAGG	0.021	0.005	0.018	22	1221
GGCGTTACAGACA	0.062	0.01	0.049	85	1639
GGTATTGTTTGGA	0.113	0.024	0.093	57	559
GGTGTTGCAAGTG	0.094	0.005	0.087	46	484
CCCGTTGTAGACA	0.024	0.004	0.025	60	2363
GGCGTCATTAGCG	0.06	0.011	0.048	44	882
GGTGGTATATGTG	0.273	0.061	0.216	143	519
ACCGTTACAAGCG	0.019	0.002	0.017	46	2672
ACTGTCGTTTGGA	0.027	0.002	0.024	20	797
CGCGTCATTTGCA	0.027	0.002	0.028	44	1502
CCCGTTACATGTG	0.024	0.002	0.024	39	1614
CGCGTCACTGGTA	0.027	0.005	0.032	41	1232
CGTATTGTATATG	0.025	0.012	0.017	21	1214
AGCGGTATTAGCG	0.029	0.002	0.026	29	1096
CGCGGCGCATGTG	0.033	0.007	0.038	28	714
GCCATCATTGAGA	0.056	0.002	0.058	124	2009
CCCGGCGTTGGCA	0.019	0.003	0.018	27	1447

ACCATCACTAACA	0.023	0.001	0.023	160	6848
CGCGGCACATATG	0.023	0.006	0.021	29	1337
CCCGGCATAAGTG	0.024	0.001	0.024	42	1702
AGTATTACAAGTA	0.022	0.002	0.025	62	2411
GCTGTTACAGGTA	0.053	0.016	0.036	36	960
CGTAGTGTTAACA	0.027	0.005	0.026	35	1304
AGTATTACTAATA	0.026	0.004	0.02	58	2860
ACTAGCGTAGACG	0.026	0.004	0.022	32	1422
CGTAGCGCAAACA	0.029	0.006	0.021	38	1795
CGTGTTATAAGTA	0.027	0.003	0.031	49	1514
CCCGGCATAAAGG	0.023	0.004	0.018	32	1760
CGCATTACTGGCG	0.021	0	0.021	31	1423
ACCGTCGTATAGG	0.022	0.003	0.025	27	1054
GGTGGTACATGTA	0.083	0.019	0.058	44	709
CCTAGTGTTGACG	0.025	0.005	0.021	33	1515
GGCAGTACTGACA	0.049	0.006	0.046	86	1781
AGTGGTGTAGGCG	0.02	0.004	0.017	9	528
GCCGTTGTATACA	0.049	0.008	0.039	70	1732
ACTGGTATAAGTG	0.025	0.005	0.018	25	1336
GGCGGCATTTGGA	0.324	0.06	0.34	282	548
ACTGTCGCAAGCA	0.02	0.003	0.019	37	1942
AGTATTGCAGGCA	0.019	0.005	0.022	28	1255
GCCGGTGCTTGTG	0.113	0.028	0.1	59	530
CGCGTTGCTGGTA	0.025	0.004	0.029	26	875
CCCGGTATTAACA	0.023	0.004	0.021	67	3187
AGCGTTGTTAATG	0.023	0.007	0.032	51	1527
GCCGGCATAAGTG	0.749	0.027	0.739	1253	443
GCCATCGCATGTG	0.052	0.003	0.048	46	907
ACTATCGTAGAGG	0.028	0.008	0.021	27	1271
CCTGGCACAAGCA	0.022	0.003	0.02	40	1925
CCTAGCGTTGGTG	0.019	0.003	0.02	16	783
CGCATCACAGGTG	0.023	0.001	0.024	33	1323
ACCGTTGTAAGCG	0.018	0.003	0.017	29	1705
CGTGGCGCATGCA	0.023	0.006	0.015	11	732
AGTATCATTGGCG	0.022	0.002	0.019	23	1199
ACTGTCACAAGGA	0.028	0.004	0.022	42	1844
AGTAGTGTTAGGA	0.023	0.003	0.018	15	812
AGCGTTGCTAAGA	0.019	0.004	0.013	21	1579
CGCGGCGCTAGGG	0.026	0.004	0.021	11	515
GCCAGCACAGGCA	0.058	0.004	0.054	100	1740

GCCGGTGCAAGTG	0.112	0.012	0.105	78	665
CGCGGCGTTGGCA	0.021	0.005	0.025	23	901
AGCGTCGTTGGGA	0.022	0.004	0.019	17	864
GGCGGTGTTTGTG	0.069	0.015	0.05	23	441
GCTGGCGCTTATA	0.611	0.041	0.605	724	472
CCTATCATATGTA	0.022	0.002	0.019	58	2945
GCCAGTGTAAGTA	0.045	0.003	0.047	84	1694
AGCAGTACATGGA	0.024	0.003	0.025	45	1786
CGCGTTGCATGGA	0.021	0.005	0.017	16	929
CGCGTCACTTACG	0.022	0.002	0.021	31	1459
ACTAGTGCAGAGG	0.024	0.003	0.019	26	1315
CCCGGTACTGGTG	0.027	0.006	0.019	21	1083
GCCGTCATTGACA	0.049	0.003	0.046	101	2109
ACCGGCATTTGGG	0.023	0.008	0.02	17	843
AGTGTCGCTAAGG	0.025	0.002	0.025	17	651
GGCAGTGCTAGGA	0.052	0.008	0.06	46	723
GCCGTCACAAAGG	0.054	0.005	0.061	84	1293
GGTATTGCAGAGG	0.106	0.024	0.109	61	500
CGCGGCATAAGCG	0.028	0.005	0.027	28	995
CCTGTCGTTTGTA	0.027	0.009	0.015	18	1156
GCCGGCACTAACA	0.102	0.003	0.1	225	2027
ACTGTTACAGGCA	0.023	0.001	0.022	47	2113
GCCAGCATTGGGG	0.094	0.01	0.104	65	562
GCCAGCGCTGAGA	0.053	0.002	0.054	53	937
CGCAGTGCTAATA	0.032	0.006	0.033	62	1818
GCTGTTATATGTA	0.062	0.004	0.065	97	1384
AGCAGCACTGGTA	0.017	0.002	0.018	29	1552
GGCGTTATTAAGG	0.062	0.011	0.069	67	901
GCTAGCGTATACA	0.072	0.01	0.07	104	1386
CCTGTTGCTTACG	0.027	0.003	0.023	28	1191
CGTGGCATTTAGG	0.017	0.011	0.019	7	369
CGCATTACAAATA	0.025	0.003	0.025	101	3891
ACCGTCATTTAGG	0.022	0.003	0.026	40	1518
AGTAGTATATGCA	0.023	0.003	0.026	50	1861
AGTAGCGTTAGGA	0.031	0.014	0.014	11	796
GCCGGCATTAGGG	0.59	0.05	0.525	423	383
GCCGTTACATGGG	0.053	0.01	0.04	31	736
CCCGTTGTAAGGG	0.018	0.004	0.018	22	1215
GCTGTTACAGATG	0.053	0.006	0.046	41	857
ACTGGTACTGATA	0.027	0.005	0.022	48	2162

CCCATCACTGAGA	0.024	0.001	0.025	65	2515
AGCGGTGTTAGCG	0.023	0.007	0.032	29	888
GCTAGCGTTTACG	0.071	0.008	0.079	63	736
ACCGTTACATACG	0.021	0.004	0.02	68	3283
ACTATCATAGACA	0.025	0.003	0.021	77	3537
AGCGTCGCATGCG	0.023	0.005	0.022	25	1115
GGTAGTACTAGCG	0.062	0.009	0.074	49	612
GCTATTGCATATA	0.055	0.004	0.061	103	1596
ACCAGCGTAGGGA	0.023	0.004	0.019	24	1212
GGCAGCGCTGAGA	0.054	0.016	0.043	38	850
AGTATCATTGGGA	0.024	0.009	0.03	30	981
CCTATTGCAGATG	0.031	0.003	0.029	54	1833
GGCATTATATATG	0.064	0.012	0.052	105	1917
AGCGGCGTAAGTA	0.021	0.004	0.02	30	1436
CGCGTCGCATATA	0.025	0.004	0.031	53	1664
GGCGTTGCAAAGA	0.066	0.007	0.056	68	1144
ACTATCGTTTGGG	0.027	0.006	0.034	30	840
AGCAGTGCTAAGG	0.025	0.003	0.024	31	1267
GGCGGTATATGCG	0.128	0.02	0.119	103	761
GGTGTCGTAGAGG	0.107	0.016	0.086	36	384
CGTGGCACTGAGA	0.026	0.002	0.025	19	728
CGCATTGCTGGCG	0.025	0.003	0.022	30	1329
GGTAGTGCTTGGG	0.056	0.014	0.04	11	262
GGTATCATTAGTA	0.108	0.018	0.083	88	971
GCCATTGTATAGG	0.044	0.009	0.033	41	1202
ACCAGTGCAGAGG	0.029	0.003	0.028	50	1707
AGCAGTGCAGAGA	0.02	0.006	0.011	19	1634
GGCATCGTATACG	0.119	0.017	0.097	142	1321
AGTATTATTAATG	0.03	0.003	0.027	64	2332
ACTGTTATAGGCG	0.023	0.01	0.032	43	1322
CGTGGCATATGGG	0.032	0.006	0.04	16	384
CGCGTCACTTATG	0.03	0.007	0.038	48	1223
CGCGGCATTAATA	0.023	0.003	0.026	49	1805
CGCGGCACATGGA	0.031	0.003	0.028	30	1047
GCCGGCATTAACA	0.382	0.035	0.376	976	1618
AGTGTTACTGATA	0.025	0.003	0.027	42	1495
CGCATCACTGAGG	0.027	0.003	0.026	33	1253
GCCAGCGTTGATA	0.054	0.011	0.044	68	1467
CGCATTGTTTGTG	0.026	0.008	0.019	19	984
GCTATTACAGGCG	0.055	0.007	0.047	67	1353

CGTAGTGTTAGGG	0.028	0.004	0.023	10	416
CGTGTCGTTAGCA	0.025	0.01	0.013	12	895
ACTATTACATGCG	0.029	0.003	0.026	63	2396
GGTAGTGCATAGA	0.048	0.01	0.035	26	709
AGCAGTATATAGG	0.024	0.003	0.027	53	1877
CCCAGCACTGATA	0.027	0.001	0.025	79	3020
AGCGTCGCTAGCG	0.017	0.001	0.017	18	1028
AGTATTGTTAATA	0.02	0.002	0.018	46	2443
AGTATCGTATAGG	0.021	0.004	0.022	21	948
CCTGTCGTTGGTA	0.02	0.005	0.019	21	1077
CCCAGTGTTTGTG	0.023	0.006	0.02	24	1157
ACTGTTGTATATG	0.034	0.004	0.028	41	1434
CGCGGCATAGGCA	0.023	0.005	0.022	28	1260
CCTAGTATATGGA	0.027	0.002	0.025	39	1529
AGTAGCACATGGG	0.025	0.008	0.014	9	638
CCTATTGCTTATG	0.026	0.002	0.029	49	1663
CGTAGTATAGATG	0.027	0.002	0.028	33	1132
CGCATTACATGGA	0.022	0.002	0.021	32	1477
CGTAGCACAGAGG	0.026	0.002	0.025	18	689
CCCATCGTTAACG	0.022	0.001	0.02	55	2648
ACTATCGCAGATG	0.025	0.001	0.027	47	1724
CCCAGTGTTTATA	0.027	0.003	0.028	66	2255
GGCATCACATGGG	0.07	0.009	0.06	56	885
AGCGGCACAGACA	0.022	0.001	0.021	46	2100
CGTGGCACTGGTA	0.025	0.008	0.036	24	639
GGTATTGTAGATA	0.105	0.018	0.091	116	1154
CGCAGCGCAGAGA	0.031	0.005	0.026	34	1274
CGCGGCGTAAGCA	0.016	0.004	0.016	22	1318
CCTGTTACAAAGA	0.024	0.004	0.028	63	2202
ACTGGCATATGGA	0.024	0.006	0.017	22	1255
GCCGTCATTGATA	0.052	0.007	0.047	89	1803
GCTAGCACAAACA	0.061	0.004	0.057	149	2464
AGTGGTGTTGATG	0.022	0.004	0.019	11	569
AGTGGTATAAGGA	0.023	0.004	0.021	23	1057
GCCGTTGCAGAGG	0.063	0.012	0.048	33	657
CGTGGTACAGATA	0.025	0.003	0.021	27	1268
GGCAGTGTAAGGA	0.051	0.012	0.038	43	1103
AGCATTATTGGTG	0.022	0.007	0.021	32	1469
CCCAGTATTAGCA	0.029	0.003	0.03	90	2942
GGCAGTGTATAGA	0.062	0.002	0.059	75	1200

GGCAGTGTTAATG	0.053	0.009	0.042	47	1073
CCTGTTGCTAGCA	0.029	0.001	0.031	49	1545
CGTGGCATTAGCA	0.033	0.013	0.021	18	844
AGCAGCGCTAAGA	0.022	0.005	0.021	34	1614
CGTGTCATTTGCG	0.028	0.001	0.028	21	719
CCTGGTGTAAGCA	0.016	0.003	0.017	28	1597
GGCAGTGTTTACG	0.056	0.004	0.06	65	1021
GGCATTATAGATA	0.054	0.002	0.056	151	2530
ACCAGTGCAGGGG	0.025	0.002	0.027	30	1090
GCCATTATTGAGG	0.05	0.008	0.049	77	1480
AGTAGCGCATGGA	0.024	0.006	0.016	13	783
CCTGGCGCAGATA	0.026	0.004	0.022	31	1364
CCTGGTGTTGACA	0.024	0.003	0.022	34	1519
GGTATTGTAAGGA	0.089	0.027	0.057	50	824
AGTATCGCTTGCG	0.022	0.001	0.024	19	780
GGTAGCGTAGGGA	0.072	0.008	0.062	31	466
AGTATTATAAGGG	0.024	0.005	0.027	41	1459
CGCAGTGTATGCG	0.03	0.005	0.037	39	1007
ACCAGCATTAGTG	0.022	0.002	0.02	45	2156
CCCATCACATACA	0.027	0.001	0.027	135	4861
CGTGGCACTTATA	0.023	0.004	0.019	19	993
CGTATCACTAGTA	0.024	0.007	0.014	20	1386
CCTATCACATGCA	0.026	0	0.026	80	2942
ACCAGCGTAGACG	0.023	0.002	0.022	43	1898
GCTATTGCTGATG	0.057	0.008	0.045	45	945
AGCGGTGTATACG	0.024	0.004	0.025	32	1243
GGTGGTACAAACA	0.085	0.004	0.085	119	1282
AGTAGCGCATGCG	0.028	0.006	0.021	16	740
ACTAGCACTTATG	0.023	0.001	0.025	44	1738
CGCAGTACAAGGG	0.024	0.002	0.022	24	1065
GCCATCGTAAGCG	0.056	0.007	0.048	78	1548
CGCGTTGCAAACG	0.025	0.001	0.026	43	1600
ACCGGTGTATATA	0.024	0.003	0.028	64	2198
GCTGGTACAAGTA	0.081	0.016	0.063	83	1235
GGTGTCGCAAAGA	0.083	0.009	0.07	60	793
CCCGTTACTTATA	0.021	0.001	0.022	60	2707
GGCGGTGCAAATG	0.071	0.008	0.081	78	880
GGCGTCGCTTATA	0.056	0.003	0.057	55	915
GGTATTATTAGTG	0.099	0.013	0.081	74	840
GCTATTATAGACG	0.067	0.013	0.056	101	1696

AGCGTTACAAGCA	0.02	0.003	0.016	38	2341
CCCATCGCAAGCG	0.021	0.003	0.023	52	2169
CGCGGTATTTGGG	0.029	0.009	0.035	21	571
CGCGTTATAGGCG	0.021	0.006	0.026	29	1066
AGTAGCGCTTGCA	0.03	0.005	0.024	21	871
ACCAGTATATGCG	0.022	0.002	0.024	62	2514
GCTAGCGTTTGTA	0.052	0.006	0.044	37	810
GCTGTTACTAACG	0.058	0.012	0.053	62	1103
GCCGTCGTAGGTG	0.068	0.014	0.07	37	489
CGCATCACTGACG	0.021	0.005	0.022	39	1732
AGCGTTACAAGCG	0.024	0.004	0.027	45	1601
GCTAGCGTTGGTG	0.075	0.017	0.053	25	449
GCCGGTGCTGATG	0.099	0.011	0.091	67	666
AGCGGTGTTAACA	0.019	0.003	0.018	38	2071
CCTATTACAGACG	0.032	0.004	0.026	66	2439
AGTAGCGCTAAGA	0.023	0.003	0.019	21	1061
CGCAGCGTATAGG	0.03	0.002	0.033	31	918
GCCATTACTTGCA	0.053	0.008	0.042	78	1792
AGCATCGCAGACG	0.026	0.001	0.025	54	2064
CCTGGTGCTTAGA	0.029	0.008	0.025	27	1036
GCCGTTGCATACG	0.047	0.004	0.041	46	1074
ACCATTGCTGGCG	0.021	0.002	0.02	43	2070
AGCGTTACATATA	0.021	0.005	0.02	55	2639
AGTGGCACATGGG	0.032	0.003	0.034	13	371
ACCGGCACAAGGA	0.021	0.001	0.02	44	2162
GCTGTCGTATACG	0.055	0.007	0.051	41	758
CGTATTGCTAGCA	0.02	0.006	0.015	18	1208
CGTATTGTAAATA	0.025	0.005	0.02	51	2484
CCCGTTACAGGTG	0.028	0.002	0.025	37	1415
ACCATTACATAGG	0.023	0.002	0.024	77	3092
CGCGTCGCTAGGG	0.021	0.004	0.025	15	586
AGTAGTATAGGTA	0.022	0.006	0.013	20	1554
CCCAGCACATGGA	0.027	0.007	0.019	38	1968
CCCGTCGCAAGTG	0.03	0.003	0.026	40	1471
GCTATCGCTGGCA	0.056	0.006	0.051	51	955
GGTATTGTTGGGA	0.107	0.021	0.097	56	520
CCCGTCGTAAGGG	0.023	0.004	0.02	22	1074
GCCGTCATAAGGG	0.082	0.014	0.075	64	787
CCCGGCATAGAGG	0.028	0.002	0.027	33	1188
AGTGTTATTGGGA	0.024	0.005	0.026	22	829

ACCATCGCATACA	0.022	0.003	0.019	89	4557
GGCGTCATTGGGG	0.05	0.002	0.053	32	577
GCCGTCGTTTGTG	0.047	0.003	0.051	28	525
ACCATTGCAGGGG	0.027	0.004	0.024	33	1333
GGCGTCGCTGGTA	0.063	0.007	0.054	39	680
ACTGTCATAGGTG	0.034	0.008	0.023	26	1125
GCTAGTGCATACA	0.05	0.006	0.042	73	1667
GCCAGTGTTAGTG	0.057	0.006	0.054	53	925
GCTGGCATAAACA	0.641	0.048	0.616	1822	1136
ACCAGTACAGGGG	0.029	0.007	0.024	29	1174
CGCATCGCTAGGG	0.029	0.002	0.03	23	735
CCTGGCGTATGTA	0.021	0.002	0.023	29	1257
ACTAGCACTGGCA	0.027	0.007	0.024	47	1880
CGTATTGTTAATG	0.028	0.003	0.03	42	1350
CCTATCGCAAGTG	0.026	0.007	0.026	43	1600
CCCGGTGTATACA	0.024	0.006	0.017	37	2149
CCCGGCGTAGATG	0.022	0.006	0.014	19	1324
CCCAGTATAAACA	0.023	0.002	0.021	122	5757
GGCAGCGCAAGCA	0.058	0.011	0.045	64	1354
ACTAGTGCTTGCA	0.02	0.005	0.015	31	2093
ACTGTTACAGGGG	0.026	0.004	0.02	15	728
GGCGGTATAAATG	0.112	0.029	0.084	132	1447
AGTGTTATTGAGG	0.019	0.002	0.021	17	782
AGTGGTACTTAGG	0.017	0.005	0.01	6	595
ACCGTCATTGATG	0.027	0	0.026	51	1889
AGCGTCACAGGGG	0.028	0.003	0.032	29	891
CCCGGTGCAAGTA	0.023	0.004	0.025	47	1806
AGTAGCGTAGACA	0.022	0.007	0.013	21	1604
AGTATTATTTGTG	0.026	0.007	0.035	41	1145
AGTGGCGCTTGGA	0.022	0.008	0.026	12	452
GGCGGCGCATGTA	0.097	0.006	0.104	89	770
ACTAGCACTGGTG	0.022	0.002	0.024	28	1131
ACTGGCACTGGCA	0.025	0.005	0.026	35	1321
GCTATTATAGGCA	0.053	0.005	0.047	84	1688
AGCATCACTGGCG	0.017	0.002	0.014	29	2047
GCCGGTACAAGTA	0.059	0.009	0.068	112	1529
GCCGTCATAAATG	0.05	0.004	0.049	107	2095
CGTGTCACTTGCG	0.031	0.009	0.018	13	720
ACTAGTATAGAGA	0.027	0.002	0.025	63	2414
GCTGGTATTGATG	0.426	0.031	0.415	393	553

ACTAGCGTTTGTA	0.025	0.006	0.029	36	1208
AGTGGCACATATA	0.022	0.003	0.024	33	1319
GGTATCATTGGCA	0.116	0.006	0.11	105	846
GCTATCATAGATA	0.061	0.009	0.06	129	2037
ACTGGCGCATGTA	0.024	0.003	0.021	16	754
GGTATTATTTAGA	0.108	0.01	0.094	125	1205
AGCAGTATTTATA	0.019	0.004	0.014	48	3505
CCCAGTATAAGCG	0.021	0.004	0.016	45	2687
CCCATTACAAGTG	0.028	0.004	0.022	57	2540
CGTATTGTTAACA	0.027	0.002	0.028	55	1906
CGTAGTATTTGGG	0.031	0.002	0.031	16	504
CCCGTCGCTAAGG	0.025	0.003	0.026	36	1357
AGTGGCGCTTGGG	0.021	0.005	0.024	7	290
CCTATCACTAATG	0.028	0.002	0.025	62	2449
ACTAGTGTTGGTG	0.03	0.002	0.031	30	939
GCTAGCATATACA	0.061	0.005	0.054	123	2141
GGTAGCATATGGG	0.063	0.005	0.067	33	461
CGCAGCATTAGCG	0.028	0.005	0.03	38	1224
AGTGTTATATACA	0.023	0.002	0.023	54	2305
ACCATCGCTTATA	0.025	0.001	0.026	95	3511
AGCGGTATTGATG	0.026	0.011	0.017	23	1341
ACCGTCGTATACA	0.023	0.002	0.026	65	2459
ACCAGCACATAGG	0.025	0.001	0.026	61	2306
GCTGGTACATATA	0.075	0.013	0.092	128	1256
GCCGTTACAGATG	0.064	0.01	0.062	83	1264
CGCATTATTGATA	0.023	0.003	0.019	52	2696
CCCATCACTAACA	0.026	0.003	0.022	117	5241
ACTAGTACTAATA	0.024	0.003	0.021	94	4414
GCTAGTGTTAACA	0.051	0.003	0.052	98	1773
CGTGTTGTTAGTG	0.038	0.012	0.033	18	529
CCCGTCACTTGCA	0.024	0.003	0.021	44	2046
GGCGTCGTTAAGA	0.073	0.016	0.056	59	1002
CCTATTACATATA	0.025	0.004	0.02	75	3762
CCCGTCACTAATG	0.026	0.001	0.024	55	2246
GGCGTCGCTTGCA	0.069	0.007	0.061	54	837
GGTGTTGTAAACA	0.082	0.007	0.086	106	1130
GGTGGTATTAAGG	0.293	0.034	0.273	186	496
GGTATCGTAAGCA	0.143	0.023	0.131	150	993
CGCAGCATAAGCA	0.025	0.003	0.027	68	2419
CCTAGTGCAGATA	0.024	0.001	0.024	58	2311

GGTGTCACTAGCG	0.103	0.018	0.089	47	479
CCCGTCGTATACG	0.027	0.005	0.023	44	1889
CCCATCATAAGTG	0.03	0.003	0.026	73	2737
AGTGTTATTAGCG	0.019	0.001	0.018	18	957
CCTAGCGCAGGTG	0.026	0.009	0.028	25	862
CCCATTACTAACA	0.025	0.002	0.024	126	5139
CGTGGCGCAAGTG	0.026	0.006	0.018	9	496
CCCATTACAAGGG	0.029	0.004	0.026	50	1899
CGTAGCGCTGACG	0.024	0.001	0.026	19	712
GGCAGCACTAATA	0.047	0.004	0.043	86	1928
CGCAGCATTGGCA	0.022	0.003	0.023	38	1583
AGCATCGTAAGCA	0.021	0.003	0.021	58	2746
GGCGGCGTTGGGA	0.199	0.045	0.175	81	381
CGCATCATATAGG	0.03	0.003	0.026	45	1670
GGTAGTATTAAGG	0.056	0.009	0.052	44	800
ACCGGCGCATACG	0.023	0.004	0.021	35	1624
AGCAGCGCATAGA	0.025	0.006	0.018	31	1688
AGCGGTGCATGTG	0.021	0.009	0.019	17	855
GCTGGCGTATACA	0.898	0.026	0.877	1799	252
AGTGGTGCAAGTG	0.019	0.004	0.017	11	629
CGCGTCACTAGTA	0.021	0.008	0.014	22	1565
GGTGTCGTAAAGG	0.118	0.033	0.082	45	501
AGTATCGTAGATA	0.026	0.002	0.026	48	1823
GGCGTTGCAAGGA	0.069	0.003	0.064	51	743
GGCGGTACAGATG	0.072	0.014	0.062	56	845
ACCGGTACAAGGG	0.028	0.007	0.032	55	1687
ACTAGCGTTAAGG	0.028	0.008	0.02	21	1026
ACCGTTGCTTATA	0.024	0.001	0.025	58	2299
ACCGGCGTAAACG	0.022	0.003	0.02	26	1263
AGCATCGCAAAGG	0.026	0.003	0.024	46	1867
CGCATCATATGGA	0.023	0.005	0.017	31	1836
CGTATCACAAACA	0.024	0.002	0.023	62	2625
CGCAGCACATGCA	0.023	0.004	0.027	55	1993
CGTGTCATTGGCG	0.016	0.003	0.012	9	760
CCCATCACAAGGG	0.041	0.002	0.039	76	1852
CCTGGTGCAAATG	0.027	0.003	0.029	39	1287
ACTGGTATTGGTA	0.031	0.005	0.039	49	1219
AGTAGTGTAGGGA	0.019	0.005	0.019	17	870
GGTGTTGTTTATA	0.088	0.016	0.108	85	703
GGTAGTACTAGGG	0.067	0.002	0.07	32	428

ACTGTTGTAAGCG	0.034	0.006	0.028	37	1308
ACCAGCGCTAATA	0.02	0.003	0.016	64	3855
CCCGTTACTTGGA	0.026	0	0.025	36	1382
GCCATTATTTGCG	0.053	0.006	0.051	82	1528
GGTGGCATTTACG	0.712	0.056	0.671	735	360
AGTATCGTATGGA	0.022	0.007	0.023	25	1071
CGTATCGTAGATG	0.027	0.001	0.027	31	1124
CCTGGCGTTGGCA	0.03	0.004	0.024	25	1001
ACTGGCACTGGCG	0.032	0.011	0.023	21	882
CCTATCGTAGAGG	0.027	0.005	0.02	27	1302
ACTAGTATTTGTA	0.025	0.002	0.022	46	2038
ACCAGTATATATG	0.024	0.003	0.021	71	3354
CGCGTTGTTTACA	0.026	0.004	0.026	40	1474
CCCAGCACAAGGG	0.038	0.009	0.033	56	1621
CCCGTCGTTGACG	0.019	0.004	0.02	32	1592
AGCGGTACTTAGG	0.018	0.004	0.023	22	915
ACTATTACTGGCA	0.021	0.001	0.02	52	2536
GGCGTCATTTATG	0.068	0.019	0.045	45	966
AGCATTACAAAGA	0.024	0.002	0.021	84	3915
GCTAGCGCTAACG	0.053	0.006	0.048	43	851
GGTATCACTTATG	0.13	0.02	0.128	104	710
ACTGTCACTTGCG	0.021	0	0.021	23	1081
CCTGTTACTGGCA	0.028	0.006	0.031	48	1486
CGCGTTGTAGACA	0.025	0.003	0.021	37	1728
ACTATCGCTGATG	0.029	0.004	0.024	35	1431
GCTAGTGCATATA	0.05	0.002	0.052	79	1426
AGTAGTGCATAGG	0.017	0.002	0.015	12	811
ACTATCATTGACG	0.025	0.003	0.028	65	2272
ACTGTCACAAATG	0.033	0.002	0.033	73	2165
AGTGTTGCAGGGA	0.021	0.003	0.017	10	585
GGTGGTACATGGA	0.093	0.009	0.092	53	521
GGCGGTGTAAACG	0.091	0.02	0.085	99	1060
AGCAGCGTTGACG	0.017	0.003	0.02	31	1507
ACCATCACTGGTG	0.027	0.005	0.03	60	1930
CCCGTTGTATATA	0.023	0.003	0.024	54	2231
GCCGTTACATGCG	0.058	0.009	0.046	52	1083
GGTGTTGTAAATA	0.08	0.018	0.054	64	1113
AGTATCGCAGGCA	0.019	0.005	0.016	20	1241
AGCGTTACAGAGG	0.024	0.005	0.017	20	1160
AGTATTACTTACA	0.025	0.004	0.022	52	2301

GGTATCACAAGTA	0.1	0.027	0.07	84	1108
CCTGGTGCAAATA	0.021	0.003	0.018	42	2347
ACTATTGCTGGGG	0.025	0.002	0.028	26	914
GGCGTCGTTGATA	0.066	0.009	0.054	57	990
GGTAGCATTAAGG	0.072	0.011	0.057	39	647
GCTATCACTGACA	0.057	0.003	0.053	104	1859
CGCATCACAAGTA	0.024	0.001	0.025	67	2628
AGTGTCACTAGCA	0.019	0.005	0.021	25	1162
GCCAGCGTATACG	0.054	0.008	0.044	63	1374
GGCGGCGCAAAGG	0.153	0.025	0.152	115	643
ACCGGTACTGATG	0.021	0.003	0.024	52	2083
ACCGGCGCTTACG	0.019	0.004	0.014	20	1443
GCTATTGCAAACG	0.058	0.009	0.053	91	1623
CCCATCGTTAGGA	0.023	0.003	0.02	36	1793
ACCGGTACTTACG	0.021	0.003	0.018	44	2439
CCTAGTACTGAGG	0.03	0.007	0.021	24	1135
CCCATTGCATGTG	0.022	0.003	0.022	39	1730
ACTGTCATTAAGG	0.026	0.002	0.023	34	1413
GCTGGCGCATAGA	0.618	0.032	0.599	654	438
AGCATTGTATACA	0.02	0.003	0.024	82	3375
CCTGTTGCTGAGA	0.027	0.004	0.025	32	1241
CCCAGTGCAGGTG	0.025	0.004	0.02	23	1116
GGTATCGTTGGTA	0.136	0.037	0.115	78	602
CCTGTCATAAGGA	0.026	0.002	0.023	41	1739
GCCAGCGCAGAGG	0.048	0.011	0.034	22	622
CGTGGCATAGGTG	0.021	0.008	0.013	5	392
CGTGTTATTTATG	0.031	0.005	0.024	23	922
GGCGTCATTAGTG	0.079	0.017	0.057	47	782
CGCGGCGCATGTA	0.019	0.004	0.015	16	1029
CGTGGTATATAGA	0.035	0.007	0.045	44	941
GGCGGTGCTAACA	0.065	0.015	0.063	80	1196
GGTATTACAAAGA	0.08	0.01	0.066	124	1755
ACCATCATTGGTG	0.029	0.004	0.03	63	2050
GGCGTCACATGGA	0.06	0.015	0.056	58	980
CGTGTTACTTATA	0.025	0.006	0.026	37	1392
ACTGGCATAGACA	0.027	0.004	0.028	60	2112
GCCAGCACTGGCG	0.059	0.008	0.051	50	940
CCTGGCACTAGCG	0.027	0.005	0.026	24	907
CCCAGTGTTTACG	0.026	0.001	0.027	49	1778
AGTATCATTAAGA	0.025	0.002	0.022	48	2111

ACTGGCACAAGTG	0.024	0.001	0.022	27	1203
AGCAGTGTAGACA	0.022	0.003	0.019	52	2703
GGCATCGCATACA	0.078	0.006	0.075	144	1780
ACTGGTATTGAGA	0.028	0.009	0.024	34	1393
ACCGGCGTTAAGA	0.027	0	0.027	38	1395
GCTAGCATTTGCG	0.068	0.004	0.066	54	765
GCCGGTATATGCA	0.08	0.009	0.071	125	1638
GCCATCGCTAAGA	0.048	0.005	0.042	62	1431
CCCGTTACTTACA	0.027	0.004	0.021	62	2841
GCTATCACTTAGG	0.063	0.008	0.058	55	886
CCCATCACAAGTG	0.026	0.002	0.029	76	2523
GGCAGTATTGAGG	0.043	0.005	0.041	37	871
ACCATCGTAGACG	0.025	0.003	0.023	56	2399
CGCGTTACTTATA	0.022	0.002	0.019	36	1837
GGTATTGTATATA	0.108	0.012	0.094	144	1382
AGTGTCATATGTA	0.024	0.005	0.03	41	1313
GGTGTCGCTGATG	0.1	0.018	0.105	46	393
AGCATTGCTGAGA	0.019	0.006	0.022	37	1645
GGCGTCGCTGACG	0.054	0.004	0.058	50	811
GGCGGCGCTTGGA	0.08	0.026	0.063	29	429
GGCGGCATTGATA	0.295	0.056	0.291	411	1003
ACCATTGTTAGCG	0.023	0.003	0.02	37	1844
GGCGGTACAGAGA	0.068	0.003	0.064	69	1009
CCTGTTGCATATG	0.028	0.004	0.027	37	1309
CCCGTTGTTAGCA	0.027	0.003	0.023	42	1760
CGCGGCGCATGGA	0.022	0.009	0.014	12	832
CCCATCGCTAACG	0.023	0.003	0.023	64	2699
CCTGTCGCTGGTA	0.021	0.007	0.02	21	1010
CCTATTGCTAAGA	0.027	0.004	0.021	51	2325
GGTATTGTAAACA	0.101	0.004	0.096	182	1706
CCCGGCGTTTACA	0.021	0.003	0.025	50	1959
CCCGTCGCTAATA	0.025	0.005	0.02	49	2447
ACCATCGCTAATA	0.021	0.005	0.015	64	4277
AGCAGCACAAGGG	0.024	0.001	0.023	31	1335
ACCATCATAGGTA	0.025	0.003	0.023	74	3081
GGTGTCGTAAGCA	0.104	0.026	0.07	55	735
CCCAGTACAAGCG	0.032	0.003	0.028	73	2506
GGTAGTACAGACG	0.069	0.007	0.06	52	809
ACCGGCGTTGACA	0.025	0.006	0.025	40	1584
ACCATCATATGTG	0.025	0.002	0.024	68	2809

GGCAGTACTAAGA	0.05	0.003	0.046	75	1548
ACCATTATTAAGA	0.023	0.002	0.026	124	4642
GCCGTTACATGTG	0.054	0.005	0.058	62	1008
ACCGTTACTTAGG	0.021	0.004	0.016	29	1773
CGTGGCGCTAGTA	0.024	0.004	0.029	19	633
AGCATCACTAGGA	0.022	0.004	0.024	45	1869
CCTATTGTTAAGG	0.028	0.008	0.021	33	1543
ACTATCATATGTG	0.029	0.006	0.024	51	2113
CCTATTATTGGGA	0.021	0.004	0.024	36	1465
AGTATTATAGACA	0.024	0.001	0.022	71	3108
CCCGGTGTTAGGG	0.028	0.012	0.045	36	758
AGTATTACTAGTG	0.025	0.005	0.027	35	1281
ACCATCGCAAATG	0.025	0.003	0.022	68	3002
AGCATTGCAGGGG	0.021	0.003	0.024	24	984
CCTAGCACTAAGA	0.027	0.004	0.031	71	2187
CGTGTTGCAGGTG	0.029	0.002	0.027	15	550
CGCGGCATTGGCG	0.021	0.005	0.014	10	706
CCTGTTATTGGCG	0.021	0.001	0.021	24	1135
CGCGGTGTATAGA	0.026	0.005	0.027	30	1064
AGTGTTATTTAGG	0.022	0.008	0.013	11	844
AGTGGTATTAGCG	0.027	0.007	0.021	13	598
ACCAGCACAGGGG	0.025	0.007	0.018	22	1194
GGCAGTGCAAGTA	0.051	0.007	0.054	71	1233
GGTGTTGTAAGCA	0.081	0.009	0.068	56	768
CGTGTTGTTAATG	0.024	0.004	0.02	19	927
CCTGTCATAAGTG	0.021	0.003	0.018	27	1511
GCTATCGCTAGTG	0.057	0.001	0.057	38	632
CGCGTTGCATGCA	0.024	0.002	0.023	33	1379
GGTAGCGTTGGCG	0.069	0.004	0.073	31	395
GGCATCACTTACA	0.066	0.006	0.068	151	2065
CCCATCATTGAGG	0.027	0.003	0.029	53	1748
GCCATTACATGCG	0.052	0.004	0.047	77	1559
CGCGTCGCTAATA	0.024	0.001	0.023	41	1704
CCCAGCATTTGGA	0.027	0.005	0.031	49	1516
AGTGGCACTAGCG	0.02	0.004	0.017	10	569
GGCGTTATTGATG	0.064	0.012	0.061	58	898
CCTATCGTTAACA	0.024	0.001	0.025	77	3001
CGCGTCGTTAATA	0.026	0.003	0.03	52	1676
GCCGGTGTAAGTA	0.167	0.033	0.165	212	1074
AGCAGCGTTGACA	0.024	0.004	0.029	67	2213

GCTATCACATAGA	0.064	0.008	0.063	103	1533
CCCATTATTAGGG	0.034	0.006	0.042	67	1516
GGTGGTGCTAGCG	0.088	0.02	0.067	25	348
GCTAGTATAGATA	0.06	0.003	0.056	115	1923
AGCATCGTAAGCG	0.022	0.003	0.023	45	1943
CCCAGCGCTGACG	0.028	0.002	0.028	50	1750
AGCGGCGCTGGCG	0.018	0.002	0.018	13	699
CGCATTGCAGATG	0.028	0.004	0.023	30	1280
CGCGTTGCAGAGG	0.028	0.003	0.032	27	821
CGTGGCGTAAACA	0.023	0.004	0.018	22	1224
GGCGGTACTTGCA	0.064	0.013	0.049	45	874
GCTATCGCAAATA	0.051	0.004	0.051	99	1840
GCTAGTATAGGTG	0.066	0.008	0.072	67	864
ACTATCGTTTGCA	0.023	0.003	0.027	48	1745
CGCAGCATTTATA	0.023	0.003	0.027	65	2313
AGCATCACATGCA	0.024	0.002	0.021	65	3092
GCCATCGCAGAGG	0.036	0.009	0.03	26	844
GCCGTCATTAACA	0.058	0.006	0.051	140	2603
ACTGGCATTTGGG	0.029	0.003	0.024	15	601
GCCGTCACTTAGA	0.056	0.01	0.044	59	1291
CGCGTTATAGGTG	0.03	0.003	0.028	28	978
CCTGTCGTTTGGG	0.027	0.005	0.028	17	586
ACCAGCGTTTGGA	0.022	0.004	0.018	22	1220
CGCATTATTTACA	0.024	0.001	0.025	74	2906
AGCAGTGTTAGTG	0.014	0.003	0.013	16	1259
CGCGTTATTGGTG	0.024	0.002	0.027	22	807
AGTAGCATTGACA	0.027	0.007	0.018	31	1715
AGTGGTGCAGAGA	0.024	0.007	0.034	27	771
GGCGGTATATAGA	0.075	0.005	0.082	99	1107
CGCGTCATATACG	0.023	0.004	0.018	31	1725
AGCAGTACATATA	0.02	0.002	0.017	59	3396
GCTATTACTAATG	0.067	0.011	0.053	95	1690
AGTGTCATTGGTG	0.034	0.002	0.031	21	646
GCCATCGCTAACA	0.046	0.004	0.051	108	2027
ACTGGTACTTAGA	0.027	0.004	0.032	51	1531
CCCGGTGCTTAGG	0.026	0.006	0.018	15	821
GCCAGCACTGGCA	0.052	0.006	0.053	80	1418
AGCATCGCTAGGA	0.019	0.002	0.017	24	1419
GCCGTCGCAGATA	0.05	0.006	0.058	71	1155
AGCATCACAGATA	0.022	0.004	0.028	95	3292

ACTAGCACTAGTG	0.023	0.001	0.022	34	1487
ACTAGCGTATAGG	0.033	0.009	0.023	24	1028
GGTATCGCAGGGA	0.082	0.012	0.07	41	544
CCTGGTATTAGCA	0.026	0.003	0.022	34	1495
AGTGTTACTGAGA	0.03	0.001	0.031	36	1136
CCTGTTATAAGTG	0.027	0.006	0.021	33	1558
CGTAGTGCATGCG	0.023	0.01	0.01	6	600
GGTGTTATAAGTA	0.083	0.01	0.079	94	1092
GCCAGTGTTGAGG	0.052	0.005	0.051	41	767
CGTATCGCATACG	0.024	0.002	0.025	31	1227
GCTAGCGTAGATA	0.065	0.01	0.052	67	1233
ACTAGCACTTGGA	0.023	0.004	0.02	33	1587
GCCAGTGCTAACA	0.041	0.006	0.044	84	1839
CCTATCACTAGTG	0.027	0.001	0.026	45	1684
ACCGTTGCAAATA	0.023	0.002	0.025	95	3664
CCCATTATAAAGA	0.025	0.005	0.023	110	4676
AGCGTCGTAGGGA	0.018	0.003	0.014	15	1054
ACCGTCGCTAGTG	0.024	0.001	0.026	32	1214
GGCGTCGCAAGTA	0.064	0.015	0.059	63	1005
CGTGGCACATATA	0.025	0.008	0.027	35	1272
AGTAGCATAGAGG	0.016	0.004	0.012	12	994
CGTAGCATAGACA	0.021	0.004	0.018	33	1802
GCCAGCGTTTATA	0.051	0.003	0.055	84	1450
AGCATCATTTGCG	0.021	0.002	0.023	43	1844
GCTAGCACTGATA	0.064	0.002	0.063	94	1403
AGCGTTATATGGG	0.028	0.006	0.026	28	1038
ACCGGCACAAAGA	0.026	0.001	0.025	85	3303
CGCAGTATTTATA	0.027	0.004	0.024	62	2478
AGTAGCGTATATG	0.025	0.003	0.022	23	1004
GCTGGTGTAGGCG	0.702	0.047	0.716	614	243
GCCAGTACAAACG	0.053	0.001	0.055	140	2428
AGTGTCATTAATG	0.027	0	0.027	37	1326
GGCATCGTTAAGG	0.103	0.016	0.095	98	939
GGCAGCACTGGCA	0.051	0.003	0.049	56	1078
GGTGGTGCAAATG	0.101	0.008	0.09	54	545
CGTGTTACATAGA	0.028	0.011	0.018	22	1187
AGTGGCGTTTAGA	0.017	0.003	0.014	8	581
ACTGGCGTTAGCG	0.027	0.007	0.022	9	393
ACTATCATATACG	0.031	0.002	0.034	107	3081
GGCGTTGCAAATG	0.06	0.005	0.054	65	1133

GGCGGCGCTAACG	0.175	0.033	0.168	155	769
CGCGTTGTTAACA	0.023	0.005	0.016	32	1912
GCCGGCATTAATG	0.733	0.031	0.715	1468	586
CGCAGTGCTAAGG	0.026	0.003	0.028	28	965
CCCGTTACTGGTA	0.027	0.001	0.026	45	1683
ACCGTCGCTAACA	0.02	0.003	0.016	53	3243
ACCGGTGCTGACG	0.027	0.003	0.023	35	1491
GCTGTCGCTGGCG	0.049	0.018	0.05	25	474
AGCGTCGCATACG	0.022	0.003	0.019	31	1583
ACTGTTACTGATA	0.024	0.002	0.022	56	2468
ACTGTCATATAGG	0.03	0.004	0.025	34	1322
AGCGTCACTAATA	0.017	0.003	0.017	48	2775
AGCATCATTTGTG	0.021	0	0.021	36	1719
GCTGTTGCTAAGA	0.049	0.012	0.066	59	841
CCCGTCATAAGGA	0.032	0.001	0.033	73	2145
AGCGTTATAGACG	0.02	0.002	0.023	45	1892
CCTGTCACTAGGA	0.028	0.006	0.021	25	1153
GCCAGTGTATGGG	0.037	0.012	0.036	28	751
AGCAGTGTTAGCG	0.024	0.006	0.032	41	1246
ACCGGTATTGAGA	0.024	0.003	0.027	58	2079
AGTATTGCTTATA	0.023	0.004	0.018	31	1677
AGTGTCGTAGGCA	0.02	0.006	0.02	17	834
CGTGTTATATGGA	0.027	0.002	0.024	24	990
ACCGGTATAAACG	0.024	0.002	0.024	69	2864
ACTAGCACTGGGG	0.026	0.006	0.021	17	782
GCTATTACAAGCG	0.059	0.007	0.053	76	1369
GGCAGCATAAGGG	0.047	0.009	0.034	41	1177
ACCGGTATTGACA	0.025	0.002	0.026	77	2917
CCTAGTATTGGCA	0.027	0.005	0.033	52	1519
CGCATCACTGATG	0.025	0.002	0.027	48	1714
CCTATCGCAAGGG	0.026	0.002	0.025	30	1187
CGTGTCATTGGGA	0.028	0.01	0.034	20	570
GCCAGCACAAACG	0.059	0.001	0.06	141	2217
GCCGTTATAGGCA	0.056	0.004	0.058	97	1585
GGCGTTGCTGAGG	0.06	0.019	0.056	33	554
ACCAGCATTAACA	0.022	0	0.022	116	5120
GCTGGTATTGGGA	0.149	0.037	0.136	87	551
ACCAGCGCTAGTG	0.026	0.001	0.025	40	1571
GCCGTTGTAAGTA	0.041	0.004	0.04	58	1378
ACTGGTACATGCA	0.025	0.003	0.021	43	2031

CCCGTTGCTGACA	0.026	0.003	0.028	64	2242
AGTGTTGTAGACG	0.025	0.003	0.022	21	922
CGTGGTATAGGCA	0.026	0.004	0.02	18	886
ACTGTCGCTGGTA	0.027	0.005	0.019	25	1274
GGTAGTGCAGGCG	0.055	0.009	0.05	27	512
ACTGGTACATAGG	0.031	0.005	0.025	29	1142
CCTAGTGTAAGTG	0.028	0.009	0.021	32	1467
GCTGTTACAGGTG	0.074	0.002	0.073	45	568
GCTGGTGCTGATG	0.286	0.051	0.287	173	429
CGCATTATAGACA	0.023	0.002	0.021	73	3456
GGTAGTGCTTGTG	0.065	0.007	0.055	20	345
GCCAGCGCAGGTA	0.05	0.012	0.061	60	923
CGCGGCGCAGGTA	0.021	0.001	0.019	19	992
GCCAGCATAGGGG	0.079	0.006	0.087	64	673
CCTGGTGCATGGG	0.027	0.004	0.022	14	612
CCTGTTACTGAGA	0.029	0.002	0.032	48	1442
CGCGGCACTTAGG	0.022	0.005	0.017	13	741
CCCGGCGCATAGA	0.026	0.003	0.022	35	1531
AGCAGTATAGGCG	0.019	0.003	0.018	33	1754
ACTGTTGTAGACA	0.025	0.001	0.026	57	2116
CGTGGTGTTGATA	0.022	0.006	0.015	11	738
CCCATTGTAGGGG	0.032	0.002	0.031	33	1043
GGCGGTACATAGA	0.058	0.018	0.039	46	1124
CCTAGCATTGACG	0.03	0.002	0.028	43	1519
GGCATTACAAGCG	0.055	0.007	0.049	74	1425
GCCGGCATAAAGG	0.741	0.033	0.742	1356	471
GGCGTTATATGGA	0.049	0.008	0.038	39	983
AGTGTTACAGGTG	0.026	0.007	0.03	25	797
AGCGTTGTAGAGG	0.018	0.002	0.019	18	953
GCTAGTACAAACA	0.057	0.004	0.052	132	2408
ACCAGCGTATGGG	0.024	0.002	0.028	26	917
CCTAGCGCTGGGG	0.027	0.014	0.02	11	528
AGCGGTACTTACA	0.021	0.001	0.021	41	1957
CGCAGCATAGGGA	0.04	0.004	0.04	48	1138
GGCGTTATTTGCA	0.052	0.008	0.042	46	1048
ACCGTCGTAGGCA	0.027	0.005	0.02	33	1601
CCTAGTGTAAGCA	0.03	0.006	0.026	57	2118
CGTGTTACAGATA	0.028	0.006	0.025	39	1496
ACCGTTGCAAAGA	0.021	0.004	0.015	43	2764
CCCGTTACTAGCG	0.03	0.007	0.031	56	1740

AGCGTTGCTTGGG	0.017	0.003	0.019	12	636
CGCAGTATAGAGA	0.025	0.002	0.023	45	1923
GCTAGCGTAAATG	0.083	0.012	0.08	92	1059
GCTATCGCAGGGA	0.044	0.011	0.029	21	711
CGTGTTGCTAATA	0.023	0.005	0.019	24	1235
AGCATTGTAAGGA	0.023	0.005	0.016	35	2155
GCCGTTGTATATA	0.052	0.005	0.045	70	1491
GCTAGCATAAAGA	0.065	0.01	0.067	143	2004
GGCATCGTATGGA	0.093	0.016	0.083	87	962
AGTAGTACAGATA	0.022	0.002	0.024	48	1946
ACTAGCATAGGGA	0.033	0.002	0.035	46	1262
CCCGGCGCAAAGA	0.019	0.003	0.017	36	2098
AGCGGTACAAACA	0.022	0.005	0.023	72	3122
GCCGGCGTAGATA	0.851	0.029	0.856	1812	305
CGCATTACTGATG	0.026	0.001	0.024	39	1568
CGCGGTATAAAGG	0.023	0.006	0.016	20	1245
ACTGGTGCAGAGA	0.026	0.007	0.036	45	1222
CCTAGCACTTGGG	0.039	0.009	0.033	27	789
ACTAGTACTTGGG	0.025	0.001	0.024	27	1112
ACCATTACATGTG	0.024	0.002	0.026	86	3234
AGCGGCGTTGAGG	0.015	0.005	0.014	7	510
AGCATTGTTGGCG	0.022	0.003	0.026	43	1610
ACTATTACAGATG	0.023	0.001	0.024	69	2853
AGTGGCACTGACA	0.02	0.005	0.026	33	1257
GCTATTGTTAATA	0.056	0.008	0.046	103	2139
GGTGGCACTTGCG	0.359	0.06	0.313	157	344
AGTGTTACAAACG	0.023	0.004	0.028	47	1622
ACTGGCACATATA	0.029	0.007	0.025	57	2208
AGCGGTATAGGCA	0.022	0.007	0.012	21	1715
CCTGTTGCTAAGA	0.031	0.007	0.027	45	1593
GGCAGTATTTATG	0.059	0.014	0.04	55	1307
AGCATTGTTTATA	0.02	0	0.02	52	2592
CCCGGTATTTAGA	0.028	0.005	0.025	42	1666
CGCATTGTTGATA	0.028	0.003	0.024	45	1842
AGCAGTACATAGG	0.02	0.001	0.019	33	1663
GCTATTACTTACG	0.063	0.005	0.056	82	1395
GCCGTTATTTATG	0.048	0.006	0.04	57	1360
ACCATTGCTGATG	0.022	0.002	0.022	49	2178
GGCGGCGCTTACG	0.186	0.033	0.177	129	600
CGTAGTACTTGTG	0.032	0.004	0.026	16	592

ACTGGTGCTTACG	0.03	0.005	0.029	28	954
GGCGGTACAGACA	0.071	0.013	0.066	101	1437
CCTGGCGCTTAGG	0.021	0.006	0.028	18	622
GGCGTTACTTAGA	0.064	0.008	0.061	64	983
ACCGGCGTTAAGG	0.019	0.003	0.019	13	663
CGCAGCATATACG	0.026	0.005	0.032	61	1834
GCTAGTGTTAATA	0.055	0.002	0.053	98	1735
GCTGTTGCAGGCA	0.053	0.014	0.045	34	722
GGTGGCATAAGTG	0.707	0.054	0.685	821	377
GCCGTTACTTGCA	0.052	0.011	0.04	52	1257
ACCGGTGTTAGTG	0.027	0.003	0.024	25	1016
GCTAGTACTAACA	0.065	0.01	0.051	106	1962
CCTGTTACTGGGA	0.02	0.001	0.019	19	986
CGCAGTGCTAAGA	0.025	0.005	0.02	29	1401
AGTAGTGTATGCG	0.023	0.003	0.019	17	899
ACTATCACAGAGA	0.024	0.002	0.022	60	2721
GCCATTATTTGTA	0.049	0.007	0.039	86	2116
CGCGTTATATGGG	0.023	0.005	0.027	23	821
GGTAGCGCTGACG	0.069	0.012	0.057	32	526
GCTATCATTAGCA	0.066	0.01	0.053	95	1705
CGCGTCATAGGTA	0.023	0	0.023	34	1419
GGTGGTGTTTAGA	0.106	0.014	0.089	41	420
CCTATCATTGATA	0.022	0.002	0.025	84	3236
CCCATTGCTGATA	0.024	0.001	0.023	63	2699
GGCAGCACATATA	0.052	0.002	0.049	96	1853
CGCGGTGTTGGCG	0.027	0.003	0.026	16	611
GGCAGTATATAGA	0.047	0.003	0.043	83	1826
CGTATCACTGGCG	0.021	0.002	0.022	25	1113
ACCAGTATTTGCA	0.026	0.002	0.023	67	2855
CGCATTATTAAGA	0.027	0.006	0.023	59	2541
CCCGTTGCAAACA	0.022	0.002	0.024	83	3444
CCTATTGTATACA	0.02	0.003	0.019	63	3245
GGCATCATTGGTG	0.079	0.007	0.083	81	894
GCTAGCACATACA	0.065	0.007	0.055	106	1833
GGCAGCGCATAGA	0.062	0.011	0.047	50	1007
GGCATTACTTGTA	0.06	0.005	0.052	76	1373
CGCGTCGCATACG	0.03	0.003	0.031	40	1244
GCCGTTGTTAGCA	0.044	0.006	0.051	63	1165
AGTGGTACAAACG	0.018	0.003	0.021	28	1319
ACCAGCGTTTGGG	0.029	0.004	0.023	20	847

CGCGGTACTAACG	0.021	0.006	0.014	20	1385
GCTATCGTTGGCG	0.047	0.008	0.037	37	972
GGTAGCATTTACG	0.075	0.008	0.068	50	685
CGCGTTACATAGA	0.02	0.006	0.024	41	1654
CCCGGTATAAGGA	0.026	0.006	0.019	36	1828
GGTGTTGTAAACG	0.095	0.013	0.093	75	734
CGTATTACAAATA	0.025	0.004	0.02	60	3016
CCTGTCATTGGTA	0.022	0.002	0.024	30	1241
AGCATTGTAGGTG	0.02	0.005	0.019	25	1326
ACCGGCATAAAGG	0.023	0.002	0.023	42	1793
CCTAGTGTTGGTG	0.022	0.007	0.027	24	868
GCTATCATTAATA	0.061	0.006	0.055	147	2532
CCCATCACTTGTG	0.028	0.005	0.026	48	1783
CGCAGTATTGGGA	0.022	0.006	0.018	20	1089
CCCATCATTTGCA	0.024	0.002	0.024	75	3039
CCTATCGTTGGTA	0.027	0.004	0.022	33	1438
AGCATTGTTGATA	0.02	0.002	0.02	54	2598
GGCGTCGTAAAGG	0.08	0.012	0.073	66	834
AGTGGCATTGACA	0.022	0.008	0.02	18	866
GCCGTCACTAAGG	0.053	0.008	0.062	73	1102
GGCAGTACAAGCA	0.054	0.004	0.05	96	1806
CGCATTGTATGGA	0.027	0.003	0.023	30	1280
GCTATCACTTGCG	0.068	0.004	0.074	69	864
GCCGTTGCTTGTA	0.04	0.017	0.032	27	817
AGTGTTATAAGTA	0.02	0.003	0.019	36	1907
CCTAGTGCAAACA	0.023	0.003	0.022	75	3341
GGCGTTATTAACA	0.057	0.006	0.056	109	1834
GCTGTCATTAATA	0.062	0.003	0.064	124	1810
GCTAGCATTAGTG	0.064	0.007	0.068	66	898
CCTGTTATAGGGA	0.036	0.007	0.029	35	1168
ACCAGCGCTGAGG	0.022	0.004	0.025	34	1342
CCTGTTATTGGTA	0.026	0.004	0.023	31	1337
GCCGTCATTGGTG	0.056	0.01	0.045	38	811
CCTATTGCAGAGA	0.026	0.004	0.022	44	1995
CCTGGCGTTTATG	0.035	0.004	0.035	29	806
AGTGTCGTATGCA	0.026	0.007	0.017	18	1051
CCTATCATATGGA	0.027	0.004	0.022	48	2159
AGCATTGTTTACG	0.025	0.003	0.029	52	1759
GGCGGTGTTTACA	0.076	0.008	0.071	70	912
AGCGGCATAAAGG	0.02	0.004	0.016	11	677

GCCGTCATAAATA	0.058	0.001	0.059	193	3070
CGCGGCGTATACG	0.023	0.003	0.023	23	959
GGTATCATAAATG	0.121	0.01	0.107	151	1256
CCTGTTATAAATG	0.028	0.008	0.017	42	2491
GCCATCGTAGACA	0.052	0.005	0.046	108	2259
GCTGGTATATGTA	0.148	0.021	0.119	137	1012
ACCAGCGCAAAGA	0.019	0	0.019	69	3583
GGTATTACATAGA	0.093	0.017	0.087	122	1276
AGTATCACAGATA	0.024	0.001	0.024	47	1875
AGCGGTGCTTACA	0.022	0.004	0.017	25	1419
GCCAGCGCTGATA	0.053	0.005	0.057	70	1161
GCCGTCGCTTACA	0.056	0.009	0.045	55	1161
GGCGTTATTTACA	0.062	0.009	0.054	89	1558
GCTGGTACAAGCA	0.085	0.013	0.077	100	1206
GGTAGCATAGACA	0.063	0.011	0.059	75	1194
CCTAGTGTAAGTA	0.024	0.002	0.021	44	2072
ACTATCGCAGGTG	0.026	0.003	0.023	28	1166
CCCAGTACTGAGA	0.025	0.003	0.029	66	2217
ACTGGTGTAAATA	0.022	0.002	0.019	45	2283
GGTAGCACAAATA	0.069	0.006	0.061	109	1681
CCCGTTGTAGATG	0.029	0.001	0.031	50	1542
GGTATTACAGAGG	0.103	0.022	0.073	58	735
CGCATCACTTACA	0.023	0.004	0.021	57	2715
GGCGTCATAGATA	0.075	0.017	0.062	97	1460
GGTGGTATTTACA	0.127	0.008	0.116	122	932
AGTGTCGTAGGTG	0.028	0.006	0.037	21	550
GCCGTTGCAGGCA	0.046	0.01	0.032	33	1001
ACCGGCGCATATG	0.026	0.002	0.026	37	1370
GCTGTCGCATGCA	0.066	0	0.066	51	717
AGTGTTGTAAGTA	0.021	0.006	0.02	27	1329
CCTATCATAGGCA	0.024	0.001	0.023	62	2589
GCCGGCGCAAAGA	0.512	0.037	0.487	745	785
GCTGTCACTAACG	0.057	0.005	0.052	65	1177
AGTGGTACTAGGA	0.021	0.006	0.021	17	782
CCCGGTATTAGTA	0.024	0.004	0.024	47	1887
GGTGTCACATGTG	0.081	0.014	0.097	52	485
AGCGGTGTTTGTA	0.019	0.002	0.016	18	1088
CCTGGTGCATACA	0.024	0.003	0.021	41	1878
GGCGGTACTTGTA	0.063	0.007	0.059	56	895
AGCATTGTTTAGA	0.024	0.005	0.018	34	1901

CCTATTGTTAGCG	0.025	0.007	0.017	26	1510
CCCGTCGCTAACA	0.023	0.003	0.027	85	3017
CGCGGTGCTTGCG	0.026	0.003	0.023	16	670
ACCGTTGCTAGTG	0.025	0.004	0.019	25	1308
GCCGTTGCTTACA	0.047	0.005	0.044	59	1272
CGCATCATAAAGG	0.027	0.003	0.026	57	2174
GCCGGTACAAATG	0.069	0.018	0.05	88	1658
GGCATTGTATGTG	0.089	0.008	0.099	95	869
CCTGGTATAAGCG	0.023	0.003	0.026	35	1288
CGCGTTGCTGGGG	0.027	0.004	0.033	17	493
CCTATCACTTGCG	0.026	0.001	0.026	42	1584
CGTAGTGCATAGG	0.021	0.01	0.011	6	532
ACTATTGTTGGGG	0.029	0.007	0.019	18	906
CGCAGCATAGGTA	0.03	0.004	0.034	57	1609
ACCATTGTAGACG	0.03	0.002	0.027	67	2451
GGTGGCGCTGGTG	0.298	0.047	0.288	85	210
GCTATTGTTTGCG	0.062	0.006	0.059	55	874
AGCGTTATTAGTA	0.022	0.003	0.018	40	2133
AGCGGCGTTGATA	0.024	0.008	0.023	32	1331
CGCATTATATACA	0.027	0.002	0.024	88	3606
ACCGGTGCATATG	0.021	0.002	0.022	34	1484
ACCGGCACAGACG	0.025	0.004	0.02	44	2190
ACCATTGCAGGCA	0.023	0.003	0.023	67	2823
ACCGTTACAAGTG	0.021	0.003	0.018	40	2243
AGCGTTGTAGGTA	0.019	0.004	0.015	20	1339
CGTGGTGCATGCA	0.034	0.002	0.033	28	817
AGCATTACTTGGG	0.02	0.006	0.021	26	1187
ACCATTATTAGGG	0.026	0.002	0.029	54	1799
ACTGTCATTGGTA	0.026	0.002	0.024	34	1404
AGTATTACTGAGA	0.024	0.003	0.023	38	1595
CGTATCACAAACG	0.023	0.002	0.022	43	1882
CGCAGCATAAACG	0.025	0.002	0.026	67	2464
GCTGTCATTAACG	0.067	0.014	0.05	64	1208
CGTAGTGCATGCA	0.028	0.01	0.02	17	835
AGCGTTACTTATG	0.018	0.004	0.018	26	1440
CCTATCACAGGTA	0.025	0.002	0.025	58	2249
GCTGTCACTTAGA	0.051	0.004	0.053	46	826
ACTAGCATATACA	0.027	0.002	0.024	90	3644
GCTGTTGCATAGA	0.059	0.005	0.056	48	815
AGCGTCGTAAAGA	0.019	0.001	0.02	40	1981

AGCAGCACAAAGA	0.019	0.005	0.018	51	2800
CGCAGTATTGAGG	0.028	0.004	0.022	25	1110
GGCGTCATATACA	0.066	0.008	0.056	116	1957
AGCATCATTGACG	0.02	0.002	0.021	53	2440
GCCAGCGTTAATG	0.056	0.005	0.05	68	1299
AGTATCATATGTA	0.019	0.005	0.013	25	1938
CGCGTCGTAGGCG	0.025	0	0.026	25	946
GCTATTACAGGGG	0.057	0.016	0.035	28	774
CCTGGCACAGGCG	0.021	0.003	0.024	23	955
CCCGTCATAAATG	0.027	0.004	0.023	68	2898
CGCGGCGCAGACA	0.019	0.005	0.013	21	1625
ACTATCGTTTATA	0.023	0.005	0.017	41	2342
AGCGGTGCTGGTA	0.03	0.006	0.036	34	917
GCTGTTGTATGGA	0.062	0.007	0.052	39	716
ACTGGTACAGGTA	0.026	0.004	0.032	54	1613
ACCGTTATAGACG	0.02	0.002	0.016	40	2412
CGTATCGTAGGGA	0.028	0.001	0.027	24	849
GCCAGTGTAAGTG	0.049	0.004	0.044	51	1099
GCCGTTATTGGCA	0.058	0.007	0.051	76	1407
CCCGTCGTATGTG	0.022	0.002	0.02	26	1265
GGCAGTATATGCG	0.046	0.004	0.042	52	1197
GCCGGTATAGGGA	0.073	0.007	0.068	54	742
GCTAGTATTAAGA	0.056	0.011	0.055	106	1813
CCTATTGTTTGTG	0.029	0.002	0.026	34	1281
CGCAGTATTTGTA	0.029	0.004	0.034	57	1624
ACCGTTGCTGGTG	0.023	0.003	0.02	23	1147
GGCGGCATATATA	0.308	0.06	0.281	472	1210
CGCGTTACTAGTG	0.029	0.011	0.018	19	1017
AGCGGCGCAAATG	0.026	0.003	0.029	39	1307
GCCGGTATAGGCA	0.078	0.009	0.076	113	1374
ACCGTCATTGGGG	0.029	0.008	0.022	18	791
GGCATTGTTAAGG	0.067	0.013	0.061	68	1041
CGCAGCATTTATG	0.023	0.004	0.028	41	1431
AGTAGCACAGAGG	0.026	0.006	0.016	13	775
GCTGGCACATGGG	0.456	0.03	0.461	293	343
GCCGGTGTAGATA	0.179	0.042	0.146	194	1139
AGTAGTATTGAGG	0.028	0.008	0.023	22	915
AGTGGTATTTGGA	0.017	0.007	0.009	6	658
AGCAGTATTAACA	0.02	0.002	0.019	89	4534
ACCAGTATAAGGG	0.024	0.002	0.026	50	1884

CCCAGCGCTAGCG	0.021	0.003	0.023	39	1644
ACTGTCATTGGCG	0.027	0.005	0.021	29	1335
GGTATCGTAAACA	0.146	0.022	0.128	222	1512
GGTAGTACAGATG	0.057	0.011	0.042	34	776
GGCATCGTTGAGA	0.111	0.01	0.104	117	1009
AGCATCGTTGAGG	0.022	0.002	0.019	25	1313
CGCATCATTGGTA	0.021	0.004	0.02	39	1879
AGCATTATTGGCG	0.023	0.001	0.024	44	1817
GGCAGTATTAACG	0.064	0.001	0.063	114	1700
CGTGGTGCAGGGA	0.027	0.007	0.02	10	493
CCCGTTACAGAGG	0.027	0.004	0.024	36	1493
ACTAGCACAAAGA	0.023	0.004	0.018	65	3471
GGCATTGCAAGCG	0.056	0.008	0.046	49	1013
ACCGGCATTAGTG	0.026	0.002	0.024	35	1417
GCCAGCGTTTAGG	0.071	0.003	0.068	52	717
CCCATCATTTGTG	0.024	0.004	0.025	49	1943
CGTGTCACAAATG	0.026	0.005	0.025	32	1240
AGCAGTATATGGG	0.018	0	0.019	25	1324
AGTGGTATATAGA	0.026	0.008	0.027	34	1209
ACCATCACTGGCA	0.021	0.004	0.027	83	2964
ACCGGTGTATGTG	0.026	0.004	0.024	28	1143
GCCGTTGTTGGTA	0.05	0.009	0.037	34	890
CGCGTTATTGACA	0.02	0.001	0.019	40	2104
AGCGTCACAAACA	0.023	0.002	0.023	89	3744
CCCAGCGTTAAGG	0.021	0.003	0.019	30	1576
AGCGGCGTAAATG	0.022	0.007	0.016	18	1101
AGTATTGTAGGGA	0.018	0.004	0.019	22	1110
CCCGGCATTTAGA	0.031	0.004	0.031	57	1789
CGCGGCATAGAGA	0.026	0.006	0.018	24	1281
AGCAGCATTGAGG	0.023	0.002	0.022	32	1401
CGTGTTGCTAATG	0.034	0.009	0.028	24	835
CCCGTTGCTAGGA	0.025	0.004	0.021	27	1239
GGTAGCGTTAAGG	0.078	0.008	0.071	38	501
GCCGGTGCAAACG	0.105	0.008	0.099	137	1241
CGCAGCACTTACG	0.024	0.002	0.022	32	1448
CCTGTCATTAGCA	0.025	0.005	0.019	34	1738
AGCAGTACAGACG	0.018	0.003	0.022	53	2307
GGCGTCGCAAAGA	0.057	0.007	0.066	81	1141
AGCGTCATATGCA	0.024	0.002	0.027	62	2255
ACTAGCGTATGCA	0.025	0.001	0.026	44	1671

GCTGTCGCAAGGG	0.061	0.012	0.058	24	392
GCCAGTGCAGGGG	0.049	0.02	0.041	25	592
ACCATTACAAGCG	0.021	0.001	0.022	84	3752
GGCGGCGTATATG	0.459	0.064	0.471	496	557
ACTAGTGTAGGCG	0.026	0.004	0.025	41	1625
AGCAGCGCATACG	0.025	0	0.026	42	1598
GGTGTCGTTAACA	0.104	0.018	0.089	93	956
AGTGGCATAGATA	0.02	0.002	0.022	22	962
GCCATCGTATGCA	0.048	0.002	0.05	93	1768
AGCAGCATAAATG	0.019	0.001	0.02	63	3054
GGTGTTGTTAACG	0.096	0.006	0.089	58	591
GCTGTCGTTGATA	0.054	0.007	0.058	55	894
CCCAGTATTAATA	0.023	0.002	0.023	103	4304
GGTGGCGCAAGCG	0.3	0.034	0.301	161	373
CGTATTACAGACG	0.026	0.003	0.022	35	1580
CGCGGCACTAATG	0.024	0.004	0.019	25	1286
ACCATCGCTGAGA	0.02	0.001	0.019	48	2480
GCTGTCGTTGGTA	0.064	0.011	0.051	34	634
ACCGGCATAAGTG	0.028	0.008	0.023	39	1652
GGTAGTACTTACG	0.071	0.01	0.071	56	732
CCTAGTACTGGTA	0.025	0.001	0.025	37	1434
CCTATCACTGGCG	0.042	0.009	0.034	50	1402
ACCGTTGTTGGCA	0.031	0.004	0.037	59	1523
CCCATTGCTTGCA	0.024	0.002	0.026	59	2212
GCCAGCACTTGTA	0.05	0.005	0.051	79	1476
AGTAGTACTGATG	0.024	0.003	0.027	30	1076
AGCGTTATTTAGG	0.021	0.005	0.022	25	1126
AGCGGTGCTTGGA	0.023	0.001	0.02	15	720
ACTAGTGTAGGTG	0.025	0	0.025	30	1174
GGCGTCACTTATA	0.054	0.011	0.043	67	1507
GCTATCACAAGCA	0.055	0.005	0.048	82	1621
CCCGTTACTAGTG	0.026	0.003	0.022	33	1447
CCCAGCGTTGAGA	0.023	0.005	0.017	30	1760
AGCGGTACTTGTG	0.018	0.004	0.021	20	919
ACCGGCGTTGGCA	0.024	0.003	0.026	30	1114
AGTGTCGCAAAGG	0.027	0.001	0.027	22	788
GCCGTTACTAACG	0.054	0.01	0.047	77	1572
ACTGTTATAGGTG	0.027	0.004	0.022	26	1136
CGCATCATAGGGG	0.048	0.005	0.042	44	995
ACCAGTACATGCG	0.02	0.004	0.015	44	2894

ACTATCATTGGTG	0.024	0.006	0.017	27	1536
CCTAGCGTAGAGA	0.027	0.001	0.027	42	1520
GCTATTGTATACA	0.057	0.003	0.06	142	2221
GGCAGCATATACG	0.053	0.002	0.054	87	1534
AGCGGCATTGACA	0.022	0.001	0.024	44	1760
GGTATCGCAAGTA	0.099	0.014	0.08	74	850
CGCATTGCAAACA	0.03	0	0.029	89	2966
CGTGGTATTAATG	0.04	0.013	0.025	22	860
GGTGTTACATACG	0.093	0.013	0.076	67	811
ACTATTGCTAGTA	0.027	0.008	0.018	44	2384
CCTATTATTAGGG	0.031	0.006	0.023	29	1233
GCTGTCATAAACG	0.064	0.006	0.057	88	1451
ACCGTTGTTGATA	0.024	0.005	0.028	60	2054
AGTAGCATTTGGA	0.021	0.007	0.011	11	947
GCCAGTACTAGCG	0.06	0.005	0.067	88	1230
CCTGGCGTTGATG	0.024	0.006	0.029	22	744
AGTGTTATTAAGG	0.026	0.005	0.021	24	1128
CGCGTCGTAGAGG	0.025	0.006	0.033	28	814
GGTATCGTATAGG	0.138	0.043	0.109	66	542
GGTATTGCTAGGA	0.068	0.007	0.078	50	588
CCCGTCATATAGG	0.026	0.009	0.019	31	1607
GGTATCATAGGGA	0.093	0.016	0.075	60	735
CCCATTATAAGTA	0.03	0.003	0.026	107	4024
GGTGTTACAAACA	0.074	0.005	0.069	121	1622
ACTATTATTTGCA	0.021	0.007	0.019	52	2669
CCCGTTACTTATG	0.029	0.002	0.03	55	1778
GGCAGCATATGCG	0.049	0.008	0.042	50	1136
ACCAGTGCTAAGA	0.023	0.003	0.019	52	2686
GCCAGCACATGTA	0.051	0.006	0.056	96	1631
CGCAGTGTAAGTA	0.027	0.005	0.02	35	1695
ACCGTTATTAGTG	0.027	0.003	0.023	37	1588
ACCGGCATTAGGG	0.025	0.001	0.023	20	836
CCTATCGCAGGTG	0.028	0.007	0.019	21	1094
ACCAGTACATACG	0.022	0.001	0.02	81	3922
AGTATTATTGAGG	0.022	0.002	0.022	29	1284
GCCGGTACAAACG	0.063	0.006	0.055	92	1596
CGCATTATATACG	0.03	0.004	0.025	61	2402
CCTATTATAGATA	0.024	0.001	0.022	85	3695
AGCGTTATATGTA	0.022	0.003	0.026	59	2229
AGTAGCACTGGCA	0.023	0.003	0.02	22	1085

ACTATTATTGGTA	0.026	0.003	0.026	60	2268
CCCGTTGCTGGTG	0.027	0.009	0.028	28	965
GCTGGTGCTAACG	0.269	0.039	0.247	221	672
CGCGGTATTTGGA	0.021	0.006	0.013	11	849
GCCAGCGTTAGCA	0.056	0.003	0.056	81	1374
GCCGTTATTTACA	0.051	0.002	0.048	112	2205
GGCAGCATTAAGG	0.05	0.011	0.036	39	1042
GGTATCGCTTGGG	0.136	0.033	0.107	43	358
GCCATTACTAGTA	0.052	0.003	0.048	103	2038
GCTATCATATGTG	0.067	0.013	0.056	73	1234
AGTATTGTAAAGG	0.022	0.007	0.032	50	1513
GCCATTACATATG	0.05	0.003	0.053	112	2006
GCCATCACAAGGG	0.051	0.004	0.048	66	1310
CCTATCGTAGACA	0.026	0.003	0.022	57	2498
AGCGGTATTTATG	0.02	0.004	0.015	20	1306
AGTGGTACTGAGA	0.024	0.006	0.016	15	907
AGTATCACAAATG	0.025	0.002	0.026	49	1813
GCCATTGCAGAGA	0.04	0.008	0.029	41	1357
CCCAGTATAGATA	0.025	0.001	0.024	92	3741
CGTGGTGCTGATA	0.025	0.004	0.02	17	844
AGCAGTATTTAGA	0.021	0.002	0.018	43	2308
AGTAGCGCAAACG	0.023	0.004	0.026	35	1290
CGTAGTGTTGACA	0.028	0.004	0.03	31	1011
GGTGTCGTAAGGG	0.114	0.004	0.109	46	376
CCTAGCGCAAATA	0.026	0.002	0.024	76	3149
GGCGTTACATACA	0.062	0.007	0.059	117	1857
CCTGTCGTTGGCA	0.023	0.006	0.02	25	1206
GGCGTTACTTAGG	0.054	0.009	0.042	30	690
CGCAGCATATATG	0.026	0.001	0.027	50	1804
AGTGGTACATGTA	0.017	0.002	0.014	16	1146
GCCGGTGCTTACA	0.055	0.01	0.044	53	1144
ACCATCATTTGGA	0.022	0.002	0.024	57	2285
ACTGGTACTGGTG	0.026	0.009	0.016	15	913
GCTAGCGCAGATA	0.061	0.01	0.047	53	1073
CGTGTCATTTATA	0.031	0.008	0.023	32	1364
ACTAGCGTTTGCA	0.024	0.001	0.025	36	1388
AGTGGCGTAAAGG	0.029	0.003	0.029	11	366
CGCATCGTAAAGG	0.027	0.007	0.028	48	1643
AGTGGCGCATATA	0.019	0.004	0.014	14	1005
CGCATTGCAAATG	0.026	0.006	0.026	50	1862

GCTGGTACTTGTA	0.075	0.003	0.077	64	765
CCCGTCATTGGTG	0.027	0.005	0.024	31	1272
GGCATTACTAGTA	0.058	0.011	0.055	97	1672
CCTGTTACTAGCA	0.023	0.003	0.021	39	1779
GGTGTTATTAAGA	0.085	0.019	0.078	79	932
GCCGTTGCATACA	0.04	0.003	0.041	64	1487
GCCGTTGTAGGCA	0.047	0.002	0.046	55	1140
GGCGTCGTAAACA	0.073	0.015	0.056	103	1725
GGTAGCGCATGGG	0.062	0.009	0.058	18	291
ACCATCGCATGCG	0.025	0.004	0.025	50	1929
AGCGTTACTGATG	0.021	0.002	0.021	32	1457
CGTATTGTAGACG	0.023	0.005	0.023	29	1256
CCCGGCATTAACG	0.025	0.003	0.024	55	2208
AGTGGTGCAAATA	0.02	0.003	0.016	24	1489
GGCAGTATATGCA	0.053	0.006	0.046	85	1759
GCTGTTACAAATA	0.067	0.002	0.066	153	2175
CGTAGTGTAAAGG	0.028	0.004	0.022	18	819
ACTATTGTAAGTA	0.028	0.002	0.028	81	2836
GCCAGCGCAGAGA	0.048	0.013	0.035	38	1044
GCTATTGCAGGGG	0.058	0.014	0.053	34	603
CCCGTTGCTAATA	0.026	0.004	0.02	52	2550
CCTGTTGCTTACA	0.022	0.002	0.023	40	1720
AGCGGCACAGATA	0.019	0.002	0.02	40	1958
AGTGTCACAGGCG	0.026	0.005	0.029	22	743
CGTGTCGCTGACG	0.019	0.007	0.009	7	749
CCCGGTGCTGGTA	0.019	0.001	0.018	22	1227
GGCGGCGCAAGTG	0.193	0.035	0.16	120	629
CGCGTTGTAGGGA	0.024	0.005	0.031	23	723
GGTAGTATATGCG	0.073	0.01	0.058	45	725
CCTATTACATACG	0.024	0.001	0.024	68	2720
GGCAGTGCTTGTA	0.054	0.003	0.054	43	749
AGTGTCATTAGGA	0.017	0.002	0.015	14	941
AGCGGCATAGAGA	0.019	0.005	0.021	27	1262
GCTGTTGCTGACG	0.062	0.01	0.065	49	705
CGCGTTACTGATA	0.026	0.002	0.025	43	1707
CCTAGCGTTAACG	0.023	0.006	0.016	27	1683
GGCATCATAGACG	0.094	0.022	0.068	119	1643
ACTGTTATATAGA	0.026	0.003	0.024	52	2155
CCCAGTATTGGTG	0.023	0.005	0.019	29	1508
AGTGGTATTGATA	0.026	0.004	0.028	37	1270

GGCGTCACAAATG	0.063	0.007	0.053	83	1478
GGCAGTACTGAGA	0.046	0.002	0.049	61	1188
ACTGGTGCTGATG	0.032	0.003	0.029	27	916
CCTGGCGTATAGA	0.028	0.014	0.018	24	1296
CGTATTATAAACG	0.029	0.004	0.029	75	2522
CGCATTGTAGAGA	0.024	0.003	0.023	39	1661
CGCGTCACTGAGA	0.02	0.003	0.017	22	1306
CCTGTCATTAAGG	0.025	0.005	0.026	37	1361
GCCAGTACAGAGG	0.059	0.008	0.053	64	1150
AGCATCGCTGGGA	0.027	0.005	0.02	23	1150
CCCATTACAGGCA	0.024	0.002	0.026	82	3052
GCCAGCGCTAATG	0.054	0.011	0.043	45	1004
GCCAGTATTGGGA	0.049	0.007	0.042	52	1177
CGTGTCGTTAGTG	0.022	0.005	0.025	14	555
ACCAGCATTGGGA	0.027	0.004	0.029	40	1360
GGCGTTGCTGACG	0.058	0.008	0.067	52	725
AGCGGTATAGACA	0.021	0.003	0.018	46	2579
AGTATTACTTGCG	0.022	0.001	0.022	26	1163
GGTATTACATGCA	0.094	0.014	0.082	110	1233
ACTGTTGCTAATA	0.024	0.001	0.023	60	2581
GCTATCGCAAGGA	0.05	0.012	0.068	64	883
CGCGGTGTTTACA	0.028	0.002	0.029	37	1253
GGCAGTGCATGCA	0.045	0.006	0.038	41	1050
CCTAGTGCTGGCA	0.02	0.001	0.02	30	1474
GGCATCGTTTACG	0.119	0.017	0.109	125	1022
CGTGTCGTTTAGA	0.03	0.009	0.019	13	677
AGCGTCATTGGTG	0.024	0.004	0.02	21	1047
CGTAGTACTAATA	0.027	0.008	0.017	30	1711
CGCAGCACTGATA	0.025	0.002	0.027	56	2012
CGCGGCGTTAATG	0.022	0.002	0.022	22	967
CCTAGTGCTGAGA	0.026	0.003	0.022	34	1493
GGCGTCACTTGTA	0.06	0.014	0.048	54	1080
CGCAGTGTAAAGG	0.019	0.003	0.019	25	1280
CCCGTTACAGGCA	0.024	0.004	0.019	44	2293
CGTATCGCTTAGA	0.024	0.004	0.029	29	966
GGTATTACTAATG	0.114	0.021	0.084	106	1162
ACTGTTGCTGGTA	0.023	0.006	0.019	23	1204
GCTAGTATTAGGG	0.068	0.003	0.073	54	688
AGTAGCGCTAAGG	0.024	0.004	0.019	15	756
GGTATTGCATGGA	0.087	0.025	0.061	42	645

ACTGGTGTAAGGG	0.021	0.009	0.023	17	738
CGCGGCATATACA	0.025	0.006	0.023	49	2116
CCTAGCACATGGA	0.026	0.005	0.023	34	1431
ACTGTTGTAAACG	0.03	0.001	0.03	58	1908
GGCATCATTGGCA	0.073	0.005	0.072	115	1487
CCTAGCATAGACA	0.026	0.004	0.027	78	2859
CGTAGTGTAGGCG	0.029	0.007	0.02	12	593
GCCATTGTTTGGA	0.045	0.011	0.034	35	1005
GGTAGCACTGACG	0.07	0.007	0.078	51	601
GGTGGTGTTGATA	0.128	0.024	0.113	69	539
CGCGGTATTGGCA	0.02	0.003	0.021	25	1162
CCTGGTGCAGGCA	0.024	0.009	0.023	30	1258
GCTATCGCAGATG	0.05	0.014	0.043	41	918
AGCGGCGCAAGTA	0.024	0.001	0.023	33	1408
AGCGTCACTTGGA	0.024	0.001	0.024	29	1165
ACTGGTGCTAAGA	0.027	0.005	0.022	33	1459
CCCGGCATAGGCG	0.035	0.005	0.04	61	1463
CCCATCATAGGCG	0.039	0.003	0.041	92	2154
CCTGGCGTATACG	0.026	0.002	0.024	28	1142
GGCATTATTTGTG	0.063	0.006	0.066	77	1084
AGTATCGCTTACA	0.027	0.001	0.026	42	1546
GCTATTGCAAGTA	0.06	0.009	0.053	80	1436
AGTATCACAGGTA	0.024	0.007	0.015	18	1206
CCCGGCGTTAATG	0.027	0.003	0.022	33	1457
CCCGGTGTAGACG	0.026	0.004	0.024	38	1531
AGTGTTGTATGTG	0.025	0.006	0.019	14	719
ACCGTTATAAGGG	0.023	0.003	0.019	27	1414
AGTATCGTTAGTA	0.027	0.005	0.033	45	1334
AGCAGCGTATACA	0.019	0.001	0.017	48	2746
CCCGGTATTTGTG	0.024	0.003	0.027	32	1146
CCTGGCACTAACA	0.03	0.005	0.028	64	2258
CCCGTTGCAAAGG	0.031	0.004	0.027	41	1506
GCCGGCACTTGGA	0.116	0.014	0.102	83	731
GCCAGCGCAAATG	0.053	0.003	0.053	72	1290
GCTGGCGTTGGTA	0.879	0.02	0.857	855	143
AGTGTCACAAATG	0.023	0.003	0.026	36	1333
GCCGGTGTATGTG	0.403	0.074	0.379	326	534
ACCGTCATAGACG	0.023	0.003	0.024	61	2490
CGCAGTATTGATG	0.025	0.009	0.034	53	1491
CGTAGTACTGACG	0.026	0.007	0.017	15	891

ACCATTACATGGG	0.023	0.001	0.024	48	1966
ACTGGCATAGAGA	0.025	0.008	0.015	23	1501
GGCATCATAGATA	0.085	0.01	0.076	189	2306
ACCATCGCAAGCA	0.025	0.002	0.024	101	4105
AGTAGCACTGATG	0.022	0.004	0.019	21	1058
GCTGTTGTATACA	0.058	0.008	0.047	67	1361
AGCGTCACTAAGA	0.021	0.002	0.023	51	2157
GGCGGTGCAAGGA	0.06	0.011	0.063	43	643
CGTGGTATAGAGG	0.029	0.008	0.041	24	564
ACTGGCATTGAGA	0.022	0.001	0.022	29	1270
GCTAGCACAAACG	0.061	0.006	0.052	89	1627
GGTGGCACAAGGG	0.372	0.081	0.332	184	371
CGCAGTGCTTAGG	0.024	0.004	0.024	17	702
GGTGGCGTAGATA	0.373	0.051	0.374	335	560
GCCATTATATAGG	0.056	0.002	0.056	105	1759
ACCATCATATGGG	0.032	0.006	0.03	51	1662
GGCGTCATTTGTA	0.061	0.01	0.059	62	987
AGTGGCATTAACA	0.03	0.001	0.029	29	974
AGCAGCGTTAGGG	0.02	0.005	0.023	20	866
GGCGGTATAAGGG	0.125	0.013	0.107	84	704
CGCATTATAAATA	0.027	0.004	0.022	107	4687
GGCGTCGTATGTA	0.069	0.005	0.066	65	925
GGCAGTATAGATA	0.051	0.005	0.044	106	2307
GCCAGTGCATACA	0.044	0.006	0.051	96	1773
GCTGTTATTGGTG	0.055	0.008	0.05	35	661
CCCAGCATAGATA	0.026	0.002	0.023	90	3747
CGCGTCATAAACA	0.023	0.005	0.02	64	3179
GCTAGCACATGTG	0.07	0.008	0.075	61	750
CCCGGTACATGCA	0.023	0.004	0.022	50	2200
ACTGGCGTAGGCA	0.026	0.005	0.033	32	942
CCCGTCATAGATG	0.025	0.005	0.019	40	2012
ACCATCGCAGGGA	0.026	0.006	0.018	31	1721
CGCATTACAAACA	0.024	0.002	0.027	109	3957
CCCGGTGCTTATA	0.027	0.005	0.029	52	1748
GGCGTCATAGGGG	0.057	0.003	0.056	43	725
ACCGGTATTTGTG	0.028	0.002	0.031	40	1250
AGTAGTGTAAAGG	0.029	0.01	0.043	53	1193
AGTAGTATAAAGA	0.027	0.005	0.028	72	2514
CCCATCGCTTGTG	0.025	0.006	0.019	28	1424
AGTGTTACAAGGA	0.027	0.003	0.031	35	1108

ACCGTCGCTTGTA	0.021	0.002	0.019	32	1634
GGCGGCACATGCA	0.133	0.03	0.099	120	1094
AGTATCGCTTATA	0.023	0.005	0.019	28	1464
GGCAGCACTTGGG	0.047	0.005	0.053	31	559
GCTGTTGTAGGTG	0.051	0.006	0.052	32	587
CGCATTACAGGCG	0.026	0.005	0.033	51	1506
AGCGTCACAGAGA	0.025	0.001	0.026	47	1732
CCTATCGCAGGGA	0.028	0.004	0.024	32	1275
GCTATCATTTGCG	0.055	0.004	0.052	54	980
CCTAGTACTTAGA	0.022	0.004	0.017	32	1839
GGTGTCACATATG	0.094	0.012	0.087	68	718
CCTAGTACAAATG	0.027	0.005	0.024	59	2432
CGTATTATAAGGA	0.025	0.002	0.026	48	1801
ACCAGTACTGGGA	0.022	0.001	0.024	44	1809
GCTAGTGTATGTA	0.056	0.022	0.025	32	1230
CCCGTTGTTAACA	0.023	0.004	0.022	59	2589
AGCGGTATTTGGG	0.021	0.008	0.031	18	571
CGCGGCGCAAGTG	0.026	0.006	0.018	15	826
CGCGTCGTAGGGA	0.026	0.007	0.02	15	731
CGTATCATAGGCG	0.022	0.007	0.025	30	1160
CGTGTCGCAAGTG	0.027	0.005	0.033	24	693
CGTGGTGTTTGGA	0.022	0.013	0.021	9	425
ACCGTCGCAGGTA	0.022	0.002	0.02	36	1809
GCCGGTACTAACA	0.059	0.01	0.066	145	2044
GGTAGCGTAAGGA	0.076	0.011	0.062	38	578
AGTATCGTAAGTA	0.021	0.002	0.018	30	1621
ACCATTATTTAGG	0.028	0.002	0.027	64	2292
AGCATCGCATGTA	0.024	0.001	0.022	46	2034
GGTGGCGTAGGTA	0.364	0.053	0.329	170	347
CGTGTTATTGACA	0.023	0.004	0.023	35	1511
ACTAGCGCTTGGA	0.023	0.004	0.022	26	1157
GGCATTACAGAGA	0.064	0.008	0.052	85	1555
CGTAGTACAGAGA	0.032	0.012	0.015	17	1086
AGTGTCGCTAGTA	0.026	0.002	0.026	22	838
ACCGGCGTATGCG	0.023	0.008	0.015	11	744
CGTAGCGTAAGGA	0.027	0.008	0.027	23	841
CCTAGCGCTGACG	0.023	0.003	0.021	27	1237
GGTATCATATACA	0.126	0.021	0.102	198	1751
GCCATCGTAAATA	0.049	0.002	0.051	154	2839
CCCATCATTGATG	0.027	0.001	0.029	73	2483

GCCGTTGTTTGGA	0.042	0.013	0.033	24	694
AGTATTACTTATG	0.021	0.001	0.021	33	1569
CCTAGTATTTGTG	0.033	0.01	0.039	49	1213
ACCATCGTATAGG	0.031	0.004	0.036	62	1656
ACTGTCACTGAGA	0.023	0.004	0.02	35	1693
GCCGTTATATACA	0.061	0.008	0.057	157	2593
AGTGTTATTTATA	0.022	0.004	0.02	36	1796
CCCGGTGCTAACG	0.025	0.003	0.02	30	1467
AGTGGTATAGAGA	0.022	0.006	0.015	17	1097
CCCAGCGCTGAGA	0.019	0.004	0.014	29	1990
CCCAGCGCATGTG	0.026	0.004	0.021	29	1368
GCTGTTGTTTGTA	0.058	0.013	0.043	32	711
AGTGTCACTTGGA	0.02	0.004	0.017	13	754
GCCATCGTATGGG	0.049	0.011	0.034	30	847
CCCGGTGCAAATG	0.024	0.001	0.025	39	1492
ACCAGCGTTGACG	0.027	0.001	0.026	44	1661
GCTAGCACATAGA	0.056	0.007	0.062	81	1223
GCTGTCGTAGATA	0.054	0.004	0.048	55	1082
AGCGTCGCAAGCA	0.021	0.002	0.02	37	1822
ACTATCGCAGGCG	0.024	0.004	0.022	31	1379
ACTGGTGTAGAGG	0.032	0.005	0.037	28	719
ACCAGCATTGATG	0.024	0.003	0.026	63	2321
ACTAGCATAGATG	0.025	0.002	0.023	45	1899
GGTGTTATAGGCG	0.08	0.017	0.068	41	564
AGTATCGTAGGGG	0.03	0.005	0.032	23	707
CCCGGTATAGGCA	0.027	0.002	0.028	58	2005
GCCAGCGTAGATA	0.058	0.003	0.059	102	1630
GCCAGCATAAACG	0.056	0.006	0.052	147	2665
GCTATTGTTGGTG	0.06	0.008	0.055	43	735
ACCATTACTGGCG	0.025	0.007	0.023	52	2206
GCCAGTGTAAGGG	0.048	0.002	0.046	44	910
GGTATCGTTAAGA	0.156	0.034	0.118	123	922
AGCGGCACTAACG	0.02	0.006	0.016	21	1273
GGTATCACTAACA	0.106	0.012	0.098	154	1417
CGTGGCATTGGCA	0.018	0.003	0.018	13	729
CGTGTCACTAGCG	0.019	0.005	0.019	15	775
CCTGTCGTTAGTA	0.027	0.004	0.022	29	1305
GGCGTTACTAGCA	0.064	0.005	0.058	75	1218
GGTATCGTTGGCG	0.116	0.012	0.109	69	563
AGCATTGCAGACA	0.021	0.002	0.023	70	2932

CCCGGCGTTGGGG	0.029	0.002	0.028	18	616
CCCAGCGTTGATG	0.02	0.004	0.019	33	1684
CGTAGCGCTAACA	0.026	0.003	0.022	32	1413
CGCAGTACTGAGA	0.019	0.001	0.018	29	1565
GCCATCACATGCG	0.045	0.003	0.046	82	1684
AGTGTCATTAGTG	0.024	0.01	0.019	17	864
GGCAGCATTGGGG	0.045	0.005	0.052	38	699
AGCGTTACATGGG	0.019	0.004	0.014	13	924
CCCAGCGTATGTG	0.023	0.005	0.025	39	1491
ACTGTCGCTAACG	0.025	0.003	0.022	36	1586
AGTGTTGCATACG	0.028	0.003	0.03	30	962
GGCGTCGTTGACG	0.091	0.012	0.075	66	812
GCTGTCGTTAGCG	0.053	0.012	0.047	29	592
GGCGTTACTTATA	0.06	0.005	0.065	93	1338
GCTATTATAAATA	0.067	0.007	0.059	232	3689
GCTGTTGTTTACA	0.054	0.006	0.047	50	1003
CGCATTGTTGAGA	0.027	0.002	0.028	42	1483
GGTATCACTTGCG	0.12	0.024	0.09	57	575
GCCATCGCAGAGA	0.047	0.003	0.047	57	1165
AGCGTCGCAAGTA	0.024	0.004	0.02	34	1671
ACCAGCACTGATA	0.021	0.001	0.022	95	4319
GGCGTCGTAAGTG	0.081	0.009	0.076	60	725
CCTATCGTTGAGA	0.024	0.004	0.018	28	1501
CCCGGCATAAATG	0.024	0.002	0.022	55	2498
GCTAGTACAGGGA	0.077	0.011	0.075	64	785
GGCATCACTTATA	0.065	0.009	0.055	109	1880
ACCATTGCATGCG	0.022	0.001	0.024	55	2261
ACTAGCATTGAGG	0.026	0.004	0.03	39	1263
AGCGGCGTTTACG	0.022	0.003	0.026	21	793
GGCAGTACTGGGA	0.058	0.004	0.064	51	747
ACCAGCACTAGGA	0.026	0.004	0.021	51	2337
ACTAGCGCTTATG	0.027	0.004	0.025	33	1272
ACCGTCACTTGCG	0.025	0.005	0.022	36	1594
CCCATTGCATGGG	0.026	0.009	0.039	50	1230
CCCAGTGTAAGCG	0.026	0.003	0.023	43	1835
GGTATCATATACG	0.145	0.022	0.12	139	1017
CGCATCACTAACG	0.026	0.002	0.024	61	2438
CCTATTACTTGTG	0.029	0.003	0.027	42	1538
AGTAGTATAAGTA	0.023	0.002	0.025	57	2265
CGTATCGCTTATG	0.028	0.003	0.032	31	937

ACTGGTGTAAGTG	0.03	0.007	0.024	23	929
GGCATCACTTGCA	0.069	0.008	0.064	103	1509
ACTATCGTAAAGG	0.026	0.002	0.026	49	1842
GCCATTGCAGGCG	0.04	0.009	0.039	54	1347
AGCATTACTAGGA	0.025	0.001	0.025	49	1885
CCCAGCACAGACG	0.039	0.006	0.034	87	2509
GGTGTCGTATGGA	0.085	0.013	0.086	41	435
CGCGTTACATATG	0.025	0.006	0.017	27	1545
GCTATCACAAGGA	0.056	0.002	0.054	71	1245
ACTATTATTTATG	0.031	0.005	0.026	71	2653
AGTGGTATAAAGA	0.022	0	0.022	37	1644
AGTGGCGTAGACA	0.031	0.005	0.033	30	887
CCTAGTACTGATG	0.026	0.007	0.024	37	1500
CGCGGCATTTAGG	0.021	0.005	0.014	9	631
GGTAGCGTTAATA	0.083	0.01	0.073	80	1012
AGCAGTGTATGGG	0.023	0.009	0.036	36	966
CCCGGTGCATATA	0.023	0.002	0.025	52	2013
ACCAGTATAGGGG	0.033	0.014	0.029	33	1109
ACTGGCGTAGGCG	0.021	0.007	0.012	5	408
ACCATTGCTGATA	0.026	0.001	0.027	89	3201
CCTGGTGTAAGGA	0.028	0.003	0.03	33	1085
GGCGTCACTTAGA	0.065	0.01	0.056	58	987
GGTATCGTATAGA	0.143	0.023	0.121	125	904
ACCAGTGCTAGTG	0.019	0.003	0.018	29	1552
AGCGTTGTAGGGG	0.019	0.005	0.015	15	956
GGTAGTGTTAGCG	0.069	0.002	0.072	35	450
CGCATCATTTGTA	0.028	0.001	0.03	60	1940
GGCGGTACTAGCA	0.064	0.012	0.049	53	1033
CCTATTATATAGG	0.025	0.005	0.02	42	2058
ACTAGTGCTAAGA	0.024	0.004	0.019	44	2237
AGTGGTGTTAACG	0.025	0.005	0.022	17	743
CCCATCATTTGGG	0.035	0.005	0.042	51	1162
ACTAGTGTAAACA	0.026	0	0.026	102	3781
AGTAGCGCTGATA	0.026	0.004	0.021	25	1138
CGCGTTACTTGCA	0.025	0.006	0.022	34	1480
CCTAGCATTAAGG	0.024	0.003	0.024	37	1504
AGCAGCGCTTGCG	0.015	0.004	0.02	21	1034
CCCGGCATTTGCG	0.025	0.004	0.028	37	1286
CGTAGCACAGGCG	0.015	0.008	0.009	7	801
AGCATTGCTGGTG	0.024	0.005	0.018	20	1108

GCTGGTACATGGG	0.141	0.018	0.123	54	385
ACCGGCGTATAGG	0.033	0.01	0.029	18	611
AGTGGCGCTTAGG	0.02	0.004	0.023	9	386
ACTAGCATAGACG	0.024	0.006	0.026	55	2026
GGTGGTACTTGGG	0.123	0.02	0.107	29	242
CGCGTCGTAGATA	0.025	0.002	0.023	37	1542
CCTATTGCTAGCG	0.026	0.005	0.025	35	1390
CCCATCATAGGTA	0.03	0.003	0.033	95	2788
GCCATCGCATGCG	0.054	0.005	0.048	53	1042
CGCGTTATTGGTA	0.028	0.003	0.026	33	1260
AGCGTTATAAGTG	0.029	0.008	0.036	65	1736
AGCGTTGTATGTG	0.025	0.005	0.021	22	1042
CCTGGTATTGAGG	0.023	0.004	0.029	23	772
CCCAGTGCTTGGA	0.022	0.007	0.015	19	1223
CGCGGCATATATG	0.023	0.004	0.017	18	1020
ACCAGTATTTAGG	0.021	0.001	0.02	40	1966
ACCATCGTATATA	0.022	0.003	0.019	65	3444
ACTATCACTTACG	0.027	0.001	0.026	66	2482
ACTAGCGTTGGCA	0.031	0.006	0.026	34	1259
GCTGTCGTTAACG	0.051	0.004	0.045	37	777
AGTATCATTGGTG	0.032	0.001	0.031	27	845
CCCAGTACTAATA	0.025	0.001	0.026	110	4183
CGTGGCACAGATG	0.035	0.012	0.018	13	696
CGCATTACAGATA	0.024	0.001	0.023	61	2593
ACTAGCGCATGGG	0.023	0.005	0.021	17	811
ACTGTTGCAGACA	0.025	0.003	0.023	54	2320
CGTAGCACTTATG	0.023	0.002	0.022	19	864
ACCGGTATATGTG	0.027	0.002	0.028	45	1575
CGTATTATTTACA	0.029	0.001	0.028	64	2189
CGCGTTGCTGACG	0.028	0.007	0.026	29	1068
AGTGGTGTAAGTA	0.022	0.003	0.018	19	1019
AGTAGCGTATACA	0.023	0.002	0.021	34	1589
CGCATCGTAAGCG	0.029	0.005	0.024	41	1699
CGCATCATAAGGG	0.033	0.004	0.033	44	1281
CGCATCATTTAGA	0.025	0.001	0.027	54	1982
CCTGTCGCAAGCA	0.028	0.002	0.025	49	1904
GCCGGTACTTGCG	0.07	0.008	0.061	50	764
GCCGTTATTTGCA	0.058	0.005	0.055	86	1491
CGCAGCGTTAAGG	0.022	0.006	0.015	15	962
GCCATTATATGTG	0.056	0.005	0.057	112	1849

CGTATTACAGATA	0.025	0.002	0.023	49	2086
ACCATCATATGCG	0.025	0.003	0.023	70	2983
ACTGGCATTAGTG	0.032	0.003	0.03	28	915
GGTGTTGTTTACG	0.082	0.007	0.088	51	529
CCTGGCATTTACG	0.028	0.007	0.018	23	1223
AGTAGCATTGGGA	0.024	0.004	0.029	26	861
AGCGGCACTGGGA	0.028	0.006	0.022	19	852
GCCATCATAGAGG	0.064	0.013	0.045	61	1280
CCCGTTGCATGGG	0.023	0.001	0.023	22	936
AGTGGTGCAGGGA	0.025	0.004	0.028	14	485
CGTGTTGCTGGCA	0.025	0.004	0.024	17	696
ACCAGCGTTTGTA	0.024	0.001	0.026	44	1660
AGCATCGTATATA	0.023	0.001	0.022	67	3003
ACCGTTGTAAGTA	0.025	0.006	0.018	40	2157
ACCATTGTTAGTA	0.023	0	0.022	57	2504
CGCAGTACAAAGG	0.03	0.006	0.024	38	1565
AGCATCGCAAACA	0.02	0.001	0.019	74	3830
GCTATCGCTTGTG	0.052	0.009	0.048	33	655
CGCATTACTTAGG	0.029	0.003	0.033	39	1147
CGTAGTATAAGTG	0.019	0.004	0.016	19	1187
ACCGTCACTTGTG	0.025	0.003	0.025	38	1492
GGCATCGCTGACG	0.109	0.015	0.091	113	1132
GGTGGTGTTTACA	0.115	0.019	0.09	64	644
ACTGGCATAGAGG	0.025	0.005	0.018	16	859
AGTAGTACAGACA	0.023	0.003	0.02	39	1951
GGCGTTGTTGATA	0.066	0.008	0.06	63	987
CCCAGCACTAGTA	0.018	0.002	0.019	51	2587
ACTAGTACATAGA	0.021	0.002	0.018	56	3014
ACTAGTGCAAACG	0.024	0.003	0.02	54	2636
GCCGGCGCTTATA	0.503	0.041	0.488	624	655
CGCGTTACTTACG	0.021	0.003	0.017	22	1269
ACTGGCGTTGACG	0.035	0.009	0.023	14	598
CCTAGCGCTGAGG	0.019	0.005	0.015	12	812
AGTATTACAAGCA	0.024	0.001	0.023	54	2332
CCTGTTGTAAGGA	0.019	0.001	0.019	27	1412
CCTGGCGTATGCG	0.029	0.004	0.026	22	820
AGTGTCGCTTGGG	0.033	0.011	0.024	10	407
GGTGGCACATGGG	0.412	0.009	0.399	192	289
GCTGTTGCTTGTA	0.047	0.011	0.032	22	663
GGTGGTGTTAGGA	0.092	0.01	0.08	32	370

CGTGGTATTGACA	0.024	0.001	0.025	31	1198
AGCGGTGCATATA	0.02	0.003	0.018	31	1693
GGCGGCGTATACA	0.212	0.038	0.184	236	1044
CCTGTTGTAAATA	0.026	0.002	0.024	64	2634
AGCAGTGTTGGTA	0.023	0.006	0.029	39	1306
CCCAGCGTATACA	0.019	0.003	0.018	58	3201
CGTAGCGTAAACG	0.022	0.003	0.018	22	1202
GGCGGTGCTTGTA	0.064	0.016	0.056	37	629
AGTAGTACAAATG	0.026	0.007	0.02	39	1875
CGCAGTACTGGTA	0.023	0.002	0.023	30	1267
ACTATTGTTAGTA	0.027	0.003	0.029	68	2313
CGCGTTGTTAGGA	0.023	0.003	0.02	18	873
ACTAGTGTTTACA	0.026	0.003	0.022	54	2403
GCTATCATTGGTG	0.057	0.005	0.052	51	934
CCCGTTGTATACG	0.025	0.003	0.025	49	1900
GCCGGTGTTGACA	0.184	0.022	0.169	231	1135
CCCATCACATATG	0.026	0.002	0.029	84	2830
GGCGGTGCAGGGG	0.051	0.013	0.042	17	388
ACCGTTACTTGTG	0.021	0.003	0.019	34	1797
GCCGTCGTATGTG	0.057	0.017	0.042	27	623
AGCATTATTTACG	0.023	0.003	0.027	71	2553
GGCATTGTTGAGA	0.069	0.015	0.059	68	1079
GCCAGCACTAGTG	0.048	0.003	0.043	57	1267
ACTGGTATTAACG	0.025	0.002	0.028	45	1591
AGCGGTATAGGGA	0.022	0.005	0.028	36	1239
GGTATCACAGACG	0.106	0.002	0.103	96	834
CCTATCGCTGACG	0.026	0.005	0.023	38	1650
GCTATTATTTGGG	0.07	0.006	0.072	57	736
GGTGTCGCAAACA	0.089	0.022	0.084	104	1138
GCCATTACAGAGA	0.049	0.012	0.037	71	1860
GCTAGTATTGGCA	0.05	0.01	0.058	70	1137
AGTATCGTTGGCG	0.023	0.005	0.017	17	995
ACCGTTGTTGACA	0.022	0.001	0.021	48	2237
ACTGGTGCATATG	0.026	0.002	0.023	27	1148
CCCAGTACTTGGA	0.024	0.003	0.02	34	1657
CCCATTGTTGGTG	0.031	0.009	0.029	36	1223
ACTGTTATTAGCA	0.023	0.002	0.025	53	2044
AGTAGTACTGATA	0.018	0.003	0.016	28	1669
ACTGGTGCTAGCG	0.025	0.006	0.017	15	871
CGCGTTGTTTAGG	0.027	0.009	0.038	28	707

GGTATTATTTGTA	0.1	0.016	0.082	113	1270
CGCGTCATTGGGA	0.019	0.007	0.011	10	916
ACCATCGCTGATG	0.023	0.001	0.024	50	1996
GGCATCGTTTATG	0.115	0.015	0.1	108	973
ACCATTGCTAGGA	0.025	0.004	0.024	50	2011
GCTGGCGCAGACA	0.595	0.045	0.586	898	635
ACTGGTATAGGTG	0.026	0.005	0.02	18	889
GGTATTGCAGGGG	0.108	0.028	0.092	42	414
AGCGTTACTAGGG	0.028	0.005	0.03	27	880
GGTATCGCTTGCG	0.148	0.007	0.153	79	439
CCTATTACTGAGG	0.026	0.003	0.025	37	1473
ACCAGCATTAATA	0.02	0	0.02	97	4659
AGCGGCGTAGATG	0.021	0.008	0.022	18	808
AGCGTTATTTGCA	0.022	0.006	0.03	58	1857
ACCGTCGCTAGCA	0.023	0.004	0.017	36	2139
AGCAGCGCATATG	0.026	0.003	0.025	38	1502
CGTAGCACAGATA	0.027	0.001	0.026	40	1479
AGCAGTGCTAGCG	0.022	0.003	0.026	34	1252
GCCAGCGTATGGG	0.057	0.015	0.038	25	636
GCCGTCACATGTA	0.048	0.003	0.05	86	1649
CGTAGTACATAGA	0.03	0.007	0.039	47	1143
GGTAGCACTTATA	0.079	0.009	0.066	70	983
GGCGTTATAGATA	0.065	0.002	0.062	108	1626
ACCAGTGTATGGA	0.024	0.002	0.025	41	1609
AGCAGTACATGTG	0.022	0.007	0.013	22	1683
GCTGTCGCAAGGA	0.058	0.007	0.053	32	574
CGTGGCATTTGCG	0.025	0.006	0.031	13	406
CGTGGTGCTAACA	0.028	0.009	0.02	21	1052
CCTGGTACATGCA	0.019	0.005	0.025	39	1512
GGCGTCGCTTACA	0.059	0.006	0.054	62	1078
ACTGGCATTGGCA	0.025	0.004	0.021	28	1293
CGTATTACAAACG	0.026	0.004	0.023	49	2085
CCTAGCACAAAGG	0.029	0.001	0.028	44	1517
GCTAGCGTTGACA	0.063	0.007	0.053	65	1152
AGCAGCGTTGGTA	0.031	0.006	0.037	48	1254
AGCGGCGTATAGA	0.024	0.005	0.017	20	1138
CCTAGCACAGATG	0.029	0.003	0.03	52	1680
CCTGGCACAGGGG	0.047	0.007	0.039	28	694
ACCAGTGCTGGTG	0.02	0.004	0.026	35	1333
GGCATTATTGACA	0.068	0.014	0.051	130	2412

GGTATTGCTGAGA	0.082	0.01	0.073	56	710
ACCAGTATTAATA	0.023	0.002	0.024	133	5495
CCCGTTGCTTGCA	0.022	0.003	0.018	29	1621
ACCGGTGCAAGGG	0.03	0.01	0.026	26	989
GCTGTTGCTGGCA	0.056	0.014	0.044	27	588
AGCATCACAAGCA	0.022	0.002	0.02	70	3383
GCCATCACTAAGA	0.053	0.002	0.053	122	2197
AGTGTTACTTATA	0.019	0.003	0.023	37	1578
CGTAGCATATGTG	0.033	0.009	0.021	17	793
GGTGGTACAAGTG	0.115	0.029	0.102	58	511
AGCATCACAAACA	0.021	0.001	0.02	103	5081
GCTATTATTAACG	0.065	0.007	0.057	122	2011
AGTAGTACTGACA	0.02	0.002	0.018	33	1824
GCTAGTACTTGTA	0.059	0.008	0.048	54	1072
GGCGGTACAAACG	0.084	0.01	0.081	116	1308
CCTATTATAAGGG	0.034	0.007	0.031	49	1519
CGTATTACATAGG	0.021	0.002	0.019	24	1212
ACCGTCACTGGCA	0.022	0.004	0.024	60	2449
CCCGGTACAGGTA	0.026	0.002	0.026	47	1748
GGTGGTATTTATG	0.285	0.067	0.251	188	561
CGCAGTACTAGGA	0.031	0.002	0.03	40	1309
GCCGGTACTAGCG	0.076	0.015	0.058	50	807
AGTATTACTTACG	0.02	0.004	0.015	25	1664
AGCGGTATATGCG	0.02	0.003	0.023	29	1225
ACTAGTACATAGG	0.021	0.003	0.019	36	1899
CGTATCATTGATG	0.029	0.008	0.038	49	1243
AGCAGTGTAGATG	0.02	0.001	0.018	31	1664
GCCGTTACTAATA	0.052	0.006	0.044	101	2196
AGTAGCACTAATG	0.024	0.003	0.026	34	1269
ACCGGTGCTTGCA	0.02	0.004	0.026	43	1641
GCTGGTATAAAGA	0.164	0.01	0.166	284	1427
ACCATTATATGTG	0.025	0.005	0.018	56	2992
ACTATTGTTGACA	0.025	0.005	0.019	54	2778
ACCAGCACTTAGA	0.021	0.001	0.023	75	3215
CCTATCACTTAGG	0.025	0.004	0.02	30	1481
AGCAGCGCAGAGG	0.021	0.001	0.022	22	969
ACCGGTGCAAGCG	0.027	0.004	0.022	33	1497
GGCGTTATTAAGA	0.054	0.007	0.061	89	1374
AGCATTGTAGAGG	0.023	0.004	0.026	39	1455
CGTAGTGCTAGGA	0.025	0.005	0.024	14	561

AGCGGCGTAAGCA	0.018	0.004	0.013	18	1391
AGCGTCACTAGCG	0.021	0.003	0.016	23	1395
GGTGTTGCAGATA	0.079	0.002	0.076	62	751
CCCGGTGTTTGGA	0.022	0.002	0.02	19	924
ACTGTTATTAGTA	0.026	0.002	0.024	49	2001
GGTAGTACATACA	0.064	0.01	0.064	102	1498
GGTAGCGCATACA	0.079	0.014	0.086	87	921
ACTGGCGCTAGTG	0.015	0.008	0.025	16	615
GGTAGCGCATGTG	0.08	0.008	0.075	34	421
GGCGTTGCTTGCG	0.065	0.005	0.072	41	529
CGTGTCGCTAGCA	0.033	0.006	0.035	32	895
GCCGTTACTTGTA	0.059	0.009	0.067	83	1155
CGCGGTACTAGCA	0.024	0.003	0.026	41	1508
ACCGGTGTTAGCG	0.024	0.003	0.023	26	1083
CCCGGTACTTATA	0.025	0.008	0.017	42	2376
CGCAGTGCTTGCA	0.029	0.006	0.022	27	1193
GCCGGTACTTGGA	0.055	0.012	0.058	47	760
CCTGGCATAAAGG	0.023	0.004	0.02	26	1274
ACTAGTGTTTGCG	0.031	0.002	0.029	36	1220
GGCATTATAAGCG	0.065	0.012	0.049	84	1637
GGTAGCGTATGCG	0.089	0.011	0.095	49	468
CGCGGTATTGATG	0.026	0.002	0.024	23	946
AGTAGTATTTGTG	0.018	0.003	0.022	21	943
GCTGTCGTAAATG	0.065	0.009	0.052	51	935
GGCAGTGTAGATA	0.061	0.008	0.058	87	1401
CCTAGCATTAGGG	0.038	0.002	0.038	37	940
GCTATTACAAGGG	0.049	0.007	0.043	42	943
CGTGTTGTTTGTG	0.029	0.005	0.022	11	483
CGTGTCATAGGTA	0.025	0.002	0.023	22	945
AGTGGTGTAGAGA	0.02	0.002	0.02	18	865
GCCATCGTTGGTG	0.05	0.01	0.037	31	806
CGCAGTGCATAGA	0.02	0.004	0.017	22	1280
GGTATTATTTACA	0.118	0.018	0.096	167	1579
CCCGGTATAAGCA	0.025	0.004	0.019	53	2698
GCCGGCGTAGGTA	0.848	0.028	0.839	1232	237
ACCGGTACAAAGG	0.025	0.002	0.027	66	2398
CGCGTCGTAAAGA	0.025	0.009	0.024	41	1699
CCTGGCGTAAAGA	0.023	0.003	0.022	36	1612
GCCATCGTATATG	0.051	0.007	0.054	82	1424
ACTATCGTTGATA	0.024	0.006	0.017	38	2256

AGTGTCACAGATG	0.026	0.003	0.024	23	922
CGCGGTGCTAGTG	0.026	0.008	0.015	10	670
CCCAGTGCAAAGG	0.024	0.006	0.016	31	1855
AGCATTGTTGGTA	0.023	0.003	0.023	39	1621
GCTGGTGTTAGCA	0.4	0.052	0.397	383	581
GGTGGCGCTGACA	0.178	0.022	0.151	102	572
AGCAGTATTTACG	0.023	0.003	0.02	45	2251
GCTGTTGTTGAGA	0.061	0.01	0.047	37	743
GGCGTCGTTAACA	0.078	0.017	0.063	88	1307
CGTGTCGCAAGGA	0.031	0.004	0.025	21	806
GCTATCGCATATG	0.062	0.005	0.062	67	1016
CGTGGTGCATACA	0.021	0.007	0.012	13	1107
ACCGGTGCTAGTA	0.023	0.001	0.022	41	1793
GCCGTTGTTAAGG	0.058	0.001	0.06	54	845
CCTATTACAGATG	0.023	0.004	0.017	39	2273
CCTATTGCTAAGG	0.021	0.003	0.019	31	1605
AGTATTATTTAGA	0.022	0.004	0.017	36	2129
CCCAGTACAAACG	0.028	0.004	0.026	91	3461
GGCAGCGTAGGGA	0.051	0.006	0.049	50	962
AGCAGTGCAGGGA	0.023	0.001	0.024	26	1080
AGCGGTACTAGTG	0.017	0.003	0.015	16	1053
GGCATCGCTTGTA	0.088	0.002	0.089	91	932
CGCATCGTATGCA	0.026	0.004	0.026	50	1862
ACTGTTACTAGCA	0.024	0.002	0.021	50	2291
CGTGGTATTAATA	0.027	0.004	0.027	40	1443
ACCAGTATAGGTG	0.025	0.004	0.029	57	1885
GCCGGCACATGCG	0.198	0.02	0.213	246	911
CCCATCGTTGATA	0.023	0.001	0.022	56	2518
ACTATCGTAAGCG	0.033	0.009	0.026	48	1773
CGCATCGCTTGGA	0.02	0.006	0.018	18	984
GCCGTCATTGAGG	0.057	0.009	0.056	58	987
AGCGTTGCTTAGG	0.022	0.006	0.015	12	799
GGTGGTACTAACA	0.076	0.006	0.071	75	988
GGCAGTGTTAAGG	0.047	0.005	0.042	38	869
GCTGGTACTAGTG	0.121	0.002	0.122	75	540
AGCGGCGTATACA	0.023	0.001	0.022	38	1696
GCCATTACAGATA	0.05	0.001	0.049	139	2698
CCTAGTGTTAAGG	0.025	0.006	0.022	31	1377
AGCAGCATTAGTA	0.024	0.001	0.022	53	2324
AGCGGTACTAAGA	0.021	0.004	0.026	48	1775

ACTATTACATGGG	0.026	0.005	0.02	35	1721
ACTAGCGCATGGA	0.017	0.002	0.021	30	1412
AGCGTCGTTGGTG	0.027	0.001	0.029	23	771
ACTATTACTAGGG	0.021	0.002	0.023	38	1611
ACCGGTGTAAACA	0.019	0.001	0.019	60	3144
CCCATCACTAAGG	0.024	0.003	0.022	51	2281
GCCAGCGTTGGCA	0.048	0.002	0.049	63	1216
GGTATCACTGAGG	0.113	0.003	0.116	65	497
AGCGGTATTAACA	0.021	0.002	0.022	63	2853
GCTGTTGTATGCA	0.053	0.008	0.062	60	914
GGCGTTGCAGGTA	0.053	0.005	0.059	48	760
CCCATTGCAAGGG	0.026	0.001	0.025	37	1465
GCTATTACAGGTG	0.067	0.005	0.063	67	1003
GGCATTATTGGCA	0.057	0.004	0.059	93	1487
ACTATTGCAAGCG	0.029	0.004	0.024	54	2187
ACTGGCATTAACG	0.026	0.004	0.032	47	1429
CGCGTTGTTGGTA	0.023	0.011	0.014	12	860
GCCGTTGTTTATG	0.053	0.012	0.038	33	842
GGCATTGTTAAGA	0.073	0.01	0.065	103	1471
GCCAGTACTTAGA	0.056	0.008	0.058	95	1531
ACTATTATATGCG	0.037	0.005	0.036	84	2273
GGCGGTACTTAGG	0.084	0.017	0.062	37	561
AGTGTCACAGATA	0.021	0.003	0.017	28	1583
CGTAGCACAAGCG	0.027	0.003	0.028	28	956
CCTGTCACAGACG	0.024	0.006	0.019	33	1686
CGTAGTGTATAGA	0.025	0.006	0.017	15	854
AGCGTCGTAGATG	0.02	0.001	0.019	24	1245
GGTATCACAAATG	0.122	0.026	0.086	109	1154
CGCGTTGTTGACA	0.03	0.003	0.033	48	1408
CCCAGTGTAGAGA	0.026	0.002	0.029	53	1788
AGTGGTGTAGACA	0.022	0.004	0.02	23	1137
GGCAGCGTATATA	0.064	0.01	0.064	103	1496
GCTAGTATAGACG	0.059	0.011	0.055	78	1344
CCTGGCATATAGA	0.022	0.004	0.018	29	1609
CGCATCATTTGCG	0.026	0.001	0.026	40	1518
GCTATCGCTAATA	0.05	0.001	0.05	75	1425
GCCAGTGTAGACG	0.048	0.008	0.037	50	1290
CCTGGTGTAAGTA	0.022	0.003	0.02	31	1510
AGTATCGTAGATG	0.02	0.004	0.021	26	1208
AGCAGTACAGGGA	0.025	0.003	0.021	33	1558

CGCAGTATAAGTA	0.028	0.001	0.028	74	2596
AGCGGCGTAGACG	0.028	0.005	0.032	29	890
GGCATTACATAGG	0.075	0.011	0.078	101	1200
GGTATTGCAGGCA	0.071	0.004	0.073	66	835
CGTAGCACTAGGA	0.022	0.003	0.018	16	872
CGTGTCGCATGTA	0.018	0.001	0.017	15	881
ACTGGCGCAGACG	0.021	0.006	0.018	18	979
CGTGGTGTAAATA	0.025	0.002	0.028	42	1443
CCTATCGTTGGGG	0.031	0.013	0.02	15	749
AGCGTTACTTGCG	0.019	0.003	0.023	27	1148
AGTGTTGTAAAGG	0.026	0.007	0.029	29	970
AGTATCACTAGTA	0.025	0.005	0.018	27	1492
CGTGTTGCTGAGG	0.025	0.006	0.028	13	459
AGTGGTGCTAGCA	0.023	0.005	0.018	15	821
AGTAGCGCTTAGA	0.023	0.004	0.024	19	765
GCCATTGTTGGGA	0.035	0.004	0.041	35	824
AGCGGCGTAGGCG	0.026	0.006	0.034	20	568
AGTAGTGTAAGGA	0.025	0.003	0.021	24	1103
CCCGGCGCAAAGG	0.024	0.003	0.025	35	1362
CGCGGTGCAGGTA	0.025	0.005	0.018	17	904
CGCATTATAGGGA	0.029	0.007	0.028	38	1341
ACCGGCACATATA	0.02	0.003	0.017	61	3519
AGTATTGCTAGCA	0.021	0.002	0.021	30	1401
CGTGTCATATGGA	0.024	0.009	0.018	15	831
CCCGTCGCTGAGA	0.028	0.007	0.019	32	1612
GCCGTTGCTAACA	0.045	0.007	0.048	75	1486
GGTAGCGTAAGCA	0.085	0.021	0.063	57	851
GCTGTCACAAACG	0.06	0.001	0.061	81	1253
CGCAGTGCTGGTG	0.024	0.003	0.025	17	658
AGCGTCGTATACA	0.02	0.004	0.015	35	2224
GGCATCGCTGGCG	0.079	0.007	0.075	83	1031
GCCATCATAAGCG	0.056	0.005	0.051	120	2212
AGTGTCACTGAGG	0.022	0.007	0.014	10	690
ACCAGCATTGGTA	0.025	0.004	0.03	73	2337
ACCGTCGCTGGTA	0.022	0.006	0.013	20	1521
CCTAGTATAGGGG	0.036	0.012	0.053	50	902
GCCATCGTATATA	0.05	0.001	0.051	117	2163
CCCATCGCAGATG	0.026	0.004	0.028	58	2031
CGTATTATATGCA	0.026	0.004	0.029	60	2004
GGCAGCGCATGCA	0.058	0.013	0.052	60	1104

ACTGTTATAGAGG	0.029	0.003	0.026	33	1245
GGCGTCGCTGATG	0.08	0.024	0.045	33	694
CGCGTTGCAGATA	0.021	0.006	0.013	20	1557
CGCAGTGCTGGGG	0.02	0.003	0.017	8	455
AGCGTCGCAGGCG	0.025	0.003	0.022	26	1159
AGCATCATATGGG	0.021	0.001	0.02	35	1713
GGCGGCACATGTG	0.268	0.049	0.231	217	724
CCCATCATAGGGA	0.04	0.006	0.046	95	1950
CCCGTCACAGGTG	0.028	0.002	0.03	45	1446
CGTGGCATTGAGA	0.02	0.002	0.02	16	788
CCCAGTGCTTGCA	0.025	0.004	0.031	59	1848
CCTGGTACATAGA	0.023	0.004	0.022	32	1453
ACCGTTGCATGTA	0.025	0.006	0.021	48	2194
CGTGGCATTAGGG	0.021	0.002	0.024	7	285
GGTGGCGCTTAGA	0.158	0.055	0.125	55	385
AGCATCGTAGAGG	0.025	0.006	0.03	46	1473
CCCAGTGTATGCA	0.026	0.003	0.021	47	2159
ACTGTTACTAATG	0.029	0.001	0.029	61	2033
GGCGGCACAAGCA	0.141	0.031	0.126	191	1324
AGTATTACATGCG	0.022	0.002	0.024	33	1329
CGCATCGTATACG	0.029	0.005	0.022	46	2053
AGCATCATAGGTA	0.024	0.001	0.026	70	2637
CGTATCACTAGCG	0.029	0.005	0.024	27	1117
CGTGGTGTAGGCG	0.025	0.008	0.028	13	444
GCCGTCATATGGA	0.057	0.006	0.052	68	1236
AGTATCGCTGAGG	0.029	0.01	0.038	28	703
ACTAGTATTGATG	0.037	0.005	0.032	61	1858
GGTGTCGCAGGTG	0.086	0.014	0.076	31	375
ACCAGTACAGGCA	0.023	0.003	0.021	81	3825
GCCATTGTATAGA	0.046	0.005	0.04	74	1774
CCCGTTACTTGTA	0.026	0.002	0.027	52	1891
GGTGGTACATAGA	0.092	0.028	0.057	44	734
ACCAGCGCAGGCA	0.023	0.005	0.016	47	2836
CCCAGCACAAGCG	0.03	0.003	0.034	87	2463
CCTATTGCTGAGG	0.022	0.006	0.03	36	1184
CGTGGTGCTAGTA	0.025	0.004	0.025	19	729
GCTAGTGCTGACA	0.058	0.002	0.056	76	1273
CCTAGTGCTGATA	0.024	0.001	0.025	48	1883
GCTAGCGCTGGTG	0.061	0.006	0.069	28	377
CCTATTACTTGCA	0.024	0.005	0.017	40	2318

GCCGTCGCTGGGG	0.058	0.011	0.046	16	333
CGCATTACAAGCG	0.023	0.003	0.019	35	1801
CCCAGTATTGGCG	0.022	0.003	0.025	45	1727
CCTATCACTTGGA	0.026	0.002	0.023	39	1692
GGCAGTACTAACA	0.054	0.002	0.051	116	2165
CCTAGTACTAACG	0.026	0.003	0.025	52	2069
CCTAGTACATGTG	0.026	0.002	0.025	35	1388
CGTGTCGCATATG	0.03	0.007	0.022	18	795
CCTGGCACTTACA	0.029	0.008	0.02	37	1848
CGCGGTGCTGGGA	0.025	0.005	0.028	18	626
CGTAGCGCAGACA	0.033	0.007	0.029	37	1248
AGTAGCATAAAGG	0.028	0.007	0.02	30	1458
GGTAGTACTAACA	0.066	0.01	0.059	83	1326
CGTATCACAAATG	0.023	0.002	0.026	47	1782
GCTATTACAGGTA	0.061	0.006	0.058	77	1257
GGCAGCACAAATG	0.045	0.008	0.034	60	1720
GGCAGCGTATGGA	0.061	0.005	0.058	46	753
CGTATTGTATGGA	0.021	0.006	0.012	12	993
CCCAGTGCTTATG	0.027	0.008	0.021	34	1556
GCTATCGCTGATG	0.063	0.007	0.068	56	763
GGCAGCACTAGTG	0.048	0.007	0.042	36	818
CCCAGTGCTTGTG	0.023	0.007	0.015	17	1143
ACCATTATTAGGA	0.024	0.001	0.023	70	2940
GCTAGTGTTTGTA	0.055	0.008	0.047	47	963
GGTATCGCTAAGG	0.129	0.022	0.114	77	599
GCTATCACTAACA	0.049	0.007	0.041	98	2319
GGTGGCATAGGTA	0.478	0.065	0.469	385	436
GGCATCGCAGGGA	0.081	0.006	0.087	78	816
GCCGTTACTTACA	0.054	0.008	0.043	81	1817
CGCAGCATTGGGA	0.026	0.004	0.027	26	922
CGCGGTGTATGCA	0.026	0.003	0.029	32	1054
CGTATTGTATGCA	0.026	0.003	0.029	41	1371
ACTAGTATATGGG	0.029	0.008	0.021	29	1330
GCCATCACTAGGA	0.053	0.002	0.051	83	1545
CCCATCATTAACG	0.024	0.003	0.022	82	3720
AGTAGCACATATG	0.023	0.006	0.019	24	1225
CCTAGCACTGATG	0.027	0.004	0.031	45	1421
CCCATCGCAGAGG	0.023	0.002	0.021	33	1572
ACCGTCGTTTAGA	0.023	0.004	0.026	36	1333
CCCGGCGTAGACG	0.026	0.005	0.018	26	1407

CCCAGTATAAAGG	0.026	0.002	0.027	77	2763
GCCGGTATTGGTG	0.177	0.024	0.179	147	672
ACTGTCGCTTATA	0.02	0	0.02	36	1777
GGTGTTGTTAATA	0.084	0.011	0.069	66	897
AGCATCGTAGGCG	0.025	0.003	0.025	44	1690
CCCGGTACAAAGA	0.022	0.003	0.025	72	2821
AGCAGTACATGGG	0.022	0.005	0.017	21	1238
AGCATTATTGAGA	0.021	0.001	0.02	57	2801
CGCAGTATTGGTG	0.024	0.004	0.023	23	989
ACTGGCGCTGGGG	0.025	0.009	0.019	8	416
CCCGTCACTAGGA	0.022	0.006	0.03	51	1626
GGTGTTACTGGGG	0.069	0.02	0.047	15	304
GGTGTCATAAAGG	0.097	0.015	0.096	82	772
GGCAGTACAAGGG	0.047	0.004	0.043	42	946
GCTAGCATAAGGA	0.07	0.003	0.072	92	1180
GCCGTCACTAATG	0.053	0.007	0.061	97	1485
GGTGGTGCTGATA	0.083	0.014	0.08	51	588
CCCGTCGTTGGTG	0.022	0.006	0.02	19	935
CGTGGTATTGGGA	0.02	0.003	0.017	9	518
AGTGGTATTTATA	0.024	0.003	0.021	30	1403
AGTATCGTTGACG	0.018	0.001	0.02	25	1236
AGTATCGCTGGTA	0.026	0.002	0.024	22	904
CGCGTCGCTGAGA	0.027	0.002	0.025	24	954
GCCATTACTAGGA	0.054	0.007	0.044	68	1479
GGCATCATAAGGG	0.076	0.007	0.069	95	1285
AGCAGCGTAGGTG	0.024	0.005	0.017	19	1078
GCCAGTGCTAGTA	0.044	0.009	0.056	64	1082
CCCATTGTAAGTA	0.027	0.004	0.021	65	2997
CGCGTTACAGGCG	0.018	0.003	0.015	18	1155
CGTATCATAGATA	0.027	0.004	0.022	52	2316
CGTATTATAGGTA	0.02	0.003	0.015	26	1677
CGCAGTACAGGGA	0.023	0.004	0.021	25	1140
AGTAGCGTAAAGG	0.021	0.003	0.022	22	991
CGCATCATAGACG	0.029	0.001	0.03	69	2219
CCTGGTGCTAATA	0.025	0.004	0.019	35	1793
AGTATCGTAGAGG	0.023	0.002	0.026	22	825
CCTATCGCTGATG	0.025	0.004	0.02	29	1416
CGCATCACAAGTG	0.028	0.004	0.023	44	1848
GGTAGCGTAGACA	0.081	0.011	0.071	69	904
ACTGTCACTAGTG	0.028	0.003	0.024	28	1128

ACTAGTGCATAGG	0.024	0.004	0.018	26	1428
CGTAGCATAAGGA	0.023	0.005	0.025	30	1186
CGCAGTACATATG	0.024	0.002	0.023	37	1539
ACCAGCGTAGGCA	0.023	0.004	0.024	49	2013
GCCATTATTTATA	0.049	0.007	0.048	158	3100
GCTAGCGTTAACA	0.057	0.003	0.061	89	1374
AGCGGCGTTAGCG	0.021	0.006	0.021	14	638
AGTATCATAAGCA	0.024	0.003	0.023	51	2193
AGCATCACTGAGA	0.022	0.001	0.02	44	2190
ACTAGTGTATATG	0.033	0.005	0.031	56	1779
ACCATCGTTTACG	0.027	0.002	0.025	56	2196
CGCAGCACAGAGA	0.029	0.005	0.025	43	1646
AGTATTATTGGTA	0.018	0.003	0.018	30	1657
GCCGTCATTTATA	0.049	0.005	0.056	110	1847
AGCGTCACTGGTG	0.021	0.004	0.015	15	1001
CCCAGCGTTAGTA	0.023	0.001	0.022	45	2009
CCTATCACTAGGA	0.026	0.003	0.025	50	1960
ACCGTCGTTAGCA	0.026	0.006	0.017	30	1693
GCCGTCGCTTGCA	0.049	0.004	0.043	38	842
CCTAGTGCAGGCG	0.023	0.007	0.018	22	1185
CCTATTACATGTG	0.034	0.005	0.027	47	1711
CGTAGTACAGACG	0.032	0.01	0.025	27	1035
GCCGGCACTGGCA	0.103	0.008	0.092	113	1109
CGCATCGTTAGTG	0.027	0.005	0.022	28	1237
GGCGTCACTAAGA	0.057	0.01	0.053	72	1289
GGTGGCACATAGG	0.382	0.053	0.41	259	373
GGCATCGCTGATG	0.104	0.005	0.097	101	937
GCTAGTATTGAGG	0.052	0.009	0.052	42	759
CCTAGCGTTTACG	0.021	0.003	0.016	21	1262
AGTGGTATTGGGA	0.022	0.005	0.016	11	688
ACTGGTACAAACG	0.028	0.005	0.023	52	2205
AGTGGTACTGGGA	0.02	0.006	0.016	10	622
GCCGGCATTTGCG	0.741	0.033	0.733	954	348
ACCATTGCTAATG	0.024	0.002	0.02	57	2737
CGTATTACAAAGA	0.029	0.002	0.03	69	2214
ACTATTGCTTGGA	0.025	0.001	0.026	41	1516
CGCGTCATTAATA	0.026	0	0.025	63	2413
AGTAGTATTAGCG	0.018	0.005	0.013	14	1104
CGCGGCGTATAGG	0.023	0.002	0.021	13	604
ACCAGCATTTAGA	0.026	0.004	0.02	58	2836

**CGCATTGCATACG**	0.027	0.004	0.022	38	1680
**ACTGGCATAAATG**	0.024	0.002	0.026	46	1696
**AGTGGTGTTGGCG**	0.027	0.008	0.016	7	427
**CGCGGTGTTTATA**	0.024	0.002	0.025	31	1200
**CCTATCGCATGGG**	0.031	0.011	0.021	20	927
**GCCGTTATAAGGA**	0.057	0.001	0.057	90	1497
**ACTAGTGTAAGCA**	0.025	0.004	0.029	69	2350
**CGCATCGTTTACG**	0.032	0.004	0.032	51	1527
**GGTGGCACTAACG**	0.401	0.054	0.344	331	631
**CGTAGCGCTTATA**	0.023	0.003	0.027	28	1009
**ACTGTTACTTGGA**	0.022	0.006	0.019	28	1423
**AGTATTACAGGTA**	0.021	0.004	0.016	25	1582
**GGTGGTACTAACG**	0.097	0.012	0.084	62	678
**GGTGGTACATGTG**	0.114	0.029	0.104	56	481
**CCTAGTGTAAATG**	0.024	0.001	0.022	53	2318
**ACTATCGCAAGTA**	0.023	0.003	0.02	52	2525
**GGCATTACTAAGG**	0.066	0.011	0.06	80	1260
**AGCAGCGTAGGCA**	0.019	0.006	0.012	21	1696
**GCTGTCACAGACA**	0.06	0.01	0.058	86	1405
**ACTAGCGCAGGTG**	0.019	0.004	0.023	24	1023
**CGTGTCACTTGGG**	0.032	0.008	0.036	16	434
**GGTGGTACTAAGA**	0.081	0.007	0.079	67	779
**AGTATCATAGAGA**	0.022	0.006	0.019	37	1865
**CGTATTGCTTGCG**	0.03	0.005	0.037	27	707
**GCCAGCGTTGGCG**	0.056	0.003	0.052	45	814
**AGCAGTACTGATG**	0.021	0.003	0.022	40	1816
**CGCGTTACAGATG**	0.029	0.005	0.033	45	1299
**GGTATTATTAGGA**	0.107	0.021	0.1	114	1021
**GGTAGTGTAAGGG**	0.058	0.007	0.051	22	409
**AGCGGCACTGAGG**	0.022	0.003	0.018	15	798
**GGTATCATAAATA**	0.132	0.019	0.11	262	2110
**ACCGGCACTAGGG**	0.025	0.004	0.019	22	1120
**AGCATTATTTACA**	0.019	0.002	0.018	73	4043
**CGCAGCGTAAACA**	0.025	0.004	0.026	75	2823
**AGCATCATAGACG**	0.023	0.002	0.022	68	3088
**AGCATCACTAGTG**	0.026	0.005	0.027	52	1874
**CCTGGTACAGGTA**	0.025	0.001	0.024	30	1234
**CCTAGCATAGGTG**	0.038	0	0.039	48	1194
**CGCGGCACATGCG**	0.017	0.006	0.022	23	1031
**ACCATTGTTAGGG**	0.03	0.006	0.036	47	1247

GGTATTGTTTGGG	0.119	0.015	0.116	53	402
AGCGTCGTATGCA	0.022	0.002	0.019	32	1630
GCTATTGCTTGTG	0.058	0.004	0.053	38	680
AGTATCGTAAGCG	0.021	0.004	0.016	20	1262
GGCAGCGTAGGCG	0.057	0.015	0.039	32	790
GGTATCACTTGTG	0.128	0.01	0.114	66	513
ACTATCATAGGCG	0.022	0.004	0.026	56	2100
CCCATTACTTGCG	0.029	0.006	0.021	41	1943
GGCATCGTAGACG	0.116	0.008	0.111	149	1190
CCCATCGCATGGA	0.025	0.002	0.023	45	1948
GGCGGTGCAGGTA	0.057	0.009	0.048	36	711
GCCAGTGCATGTA	0.046	0.008	0.035	41	1146
AGTGGCGCTAGTG	0.029	0.003	0.028	12	410
CCTATTGTATGTA	0.025	0.003	0.029	64	2126
CCCGTTATAAGTG	0.027	0.002	0.03	58	1907
ACTGTCGCTAACA	0.027	0.003	0.023	58	2497
AGTAGTACAAGGG	0.031	0.007	0.034	31	871
ACTGTTACAAGGA	0.023	0.006	0.03	62	1972
CCCGTTGCAGGTA	0.026	0.003	0.023	38	1628
GGTGGTGTTTGGA	0.131	0.025	0.123	37	263
CCCGTCACAAACA	0.024	0.002	0.023	108	4592
AGCAGCACTTGGA	0.027	0.004	0.021	28	1304
CGCAGTACTAAGA	0.027	0.005	0.026	51	1942
CCTATTACAAGTG	0.03	0.007	0.021	46	2113
ACCGTTATATGCG	0.026	0.002	0.028	56	1956
GCCGTCACAGGTG	0.053	0.012	0.057	58	953
GCTGTCACTAGCA	0.064	0.011	0.056	66	1106
GGTGTCATAGGGG	0.061	0.017	0.049	20	388
AGTGTCATAAGCA	0.026	0.005	0.02	33	1602
CCCGTCGCTGACA	0.022	0	0.022	52	2296
ACCGGTATAAATG	0.024	0.005	0.018	50	2782
GCCGTTATATAGG	0.057	0.007	0.051	63	1179
AGTATTATAAGTA	0.024	0.004	0.025	77	2988
AGCAGCGCAAGGA	0.025	0.005	0.018	25	1397
GCCATTGTTAGCG	0.053	0.005	0.048	57	1129
GCTGGTACAGGTA	0.082	0.01	0.068	55	750
GCTAGTGTATGGA	0.058	0.01	0.044	42	905
CCTGTTACAGATG	0.025	0.002	0.028	43	1504
GCTGTTATTAACA	0.059	0.003	0.054	103	1787
ACCATCATAAGGA	0.023	0.002	0.023	73	3074

ACCATCGCTAATG	0.022	0.003	0.02	52	2520
CGTAGCACAAGTA	0.028	0.007	0.021	30	1417
GCTAGTGTTTGCA	0.045	0.007	0.052	60	1090
GCTAGTATTTATA	0.059	0.012	0.046	88	1835
GCTAGCATAAACA	0.058	0.007	0.054	152	2687
CGTATTGCTTATA	0.027	0.003	0.022	30	1321
CCTGTCACTAGCG	0.031	0.005	0.025	31	1205
CGCGTTATAGAGG	0.023	0.006	0.028	30	1057
AGCGTTATTTATG	0.019	0.005	0.026	40	1526
AGTAGTACTAGCG	0.02	0.003	0.017	16	951
GGTATCATAGGCG	0.098	0.02	0.086	65	695
CGCGGTGTTTGTG	0.03	0.004	0.035	23	631
GCTGTTGTTGGTA	0.058	0.008	0.063	45	674
GCCAGTACAAACA	0.048	0.002	0.046	172	3552
CGCAGCGTAGGCA	0.028	0.002	0.025	35	1378
CGCGTCACTGGGA	0.024	0.006	0.028	29	1007
AGTGTTGTTGAGA	0.024	0.011	0.018	17	927
CCCGTTACTTGCG	0.026	0.003	0.026	38	1426
CCTGTCACATACG	0.027	0.005	0.023	40	1669
GGCATTATATGTA	0.065	0.005	0.063	131	1936
GGCAGCGCTTGCG	0.074	0.032	0.06	39	615
GCCGTTACTAGCG	0.055	0.011	0.057	65	1084
GGCATCACTTGGG	0.075	0.013	0.066	52	730
CCTGGTGCATAGA	0.026	0.008	0.017	21	1240
AGCGGTGTATACA	0.022	0.003	0.018	37	2033
CCTGTTATAAAGG	0.026	0.004	0.024	45	1810
GGCGTCGTTGACA	0.08	0.012	0.08	89	1020
CCCGTCGTATGGG	0.024	0.002	0.023	23	974
GGCAGTGCAGAGA	0.05	0.005	0.054	51	890
CGCGTCATATGCG	0.021	0.005	0.025	29	1147
CCCATCATTAGTA	0.03	0.002	0.033	111	3303
CGCGGTACTGAGA	0.016	0.002	0.013	16	1198
GGCAGTGTTAATA	0.055	0.007	0.045	82	1742
GGCGTCGCATACG	0.079	0.004	0.073	75	956
GGCGTCACTGGTA	0.054	0.006	0.047	48	973
CGTGGTACAAAGA	0.024	0.006	0.024	32	1314
CGCGGCGCAAGTA	0.023	0.004	0.017	23	1298
CCTAGTACAGATA	0.026	0.003	0.026	71	2637
AGTGGCGTAGACG	0.023	0.005	0.029	12	395
GGCATTGTTGAGG	0.077	0.019	0.07	55	726

AGTAGTGTTAGTA	0.03	0.001	0.029	36	1210
CCCATTACTTAGG	0.024	0.005	0.021	39	1839
CGCGGCGCTTGCA	0.023	0.003	0.022	23	1035
CCTGGCGCAAACA	0.028	0.007	0.023	50	2153
ACTGGTACTAATG	0.024	0.005	0.023	40	1715
ACTGGCGCTAAGG	0.02	0.013	0.012	8	685
CGTGGTATTGATG	0.026	0.002	0.029	20	674
ACCGGCACATGCG	0.019	0.008	0.017	31	1780
GGCGTCATAAATG	0.071	0.004	0.069	109	1470
ACCGGTATATGTA	0.027	0.003	0.025	63	2445
CGTATTACTGGGG	0.029	0.013	0.015	9	608
CCTGGTACAAGGA	0.028	0.003	0.033	46	1368
ACCATTGTAGACA	0.022	0.003	0.026	100	3759
ACCGGTATTTAGG	0.018	0.001	0.016	20	1219
GGTAGCGCAGGTG	0.068	0.017	0.057	22	364
CGCGTCGCAAGGA	0.021	0.008	0.031	33	1025
CGCATTGCTAGGG	0.022	0.003	0.018	14	778
ACCGTTGTTAGCG	0.024	0.003	0.026	38	1445
GGCAGCACTTAGA	0.05	0.014	0.041	45	1061
GGTGGTACTTAGA	0.079	0.024	0.057	35	577
GCTGTCGCTAAGA	0.056	0.013	0.046	36	750
CCCGGCATTAACA	0.027	0.001	0.028	95	3273
AGTGGTGCTTGCG	0.027	0.005	0.029	13	432
CGTGTTGCAGGGG	0.025	0.005	0.03	11	354
CGTATTGTTAAGA	0.023	0.002	0.026	38	1412
ACTGTCGTATATG	0.03	0.002	0.027	32	1151
CCTGTTACTTGCA	0.024	0.002	0.021	31	1436
ACTATTATATGTG	0.034	0.004	0.029	71	2373
GCTGGCATAAAGG	0.866	0.034	0.863	1357	215
CCCAGCGTAGGTA	0.026	0.004	0.022	40	1810
AGTGTTATTGGTA	0.021	0.002	0.02	22	1064
CCCAGTGCTAATA	0.024	0	0.025	74	2899
CCCAGCGCAGGCG	0.028	0.004	0.025	37	1467
ACTGTCGCTTGTG	0.026	0.008	0.02	16	785
GGCGGCGTAAAGG	0.445	0.039	0.446	444	551
ACCGGTACAAACG	0.022	0.002	0.023	86	3654
GGCATTGTATGCG	0.075	0.014	0.066	71	1011
GGTAGTGCTGACG	0.048	0.013	0.036	22	592
CGTGGCGTAAAGA	0.022	0.003	0.024	22	882
CGTAGCACTAAGG	0.019	0.007	0.016	13	814

GGCAGTACTGATG	0.048	0.008	0.038	45	1137
AGCAGTACAAACG	0.021	0.004	0.016	52	3180
CGTGTCGTAAAGG	0.033	0.002	0.03	24	766
CGCATTACTGAGG	0.023	0.007	0.032	35	1068
CGCGTCGCTGACA	0.023	0.003	0.022	37	1635
CCCGGTGCAAGCG	0.03	0.004	0.025	33	1268
CCTAGTATAGAGA	0.022	0.003	0.026	57	2154
CCCGGCACTTATG	0.03	0.005	0.031	51	1588
AGCAGCATATAGA	0.017	0.001	0.016	43	2668
ACCATTGCAGACA	0.024	0.003	0.02	87	4333
GGTGGCATAAACA	0.472	0.056	0.439	910	1161
GGCGGCGTTAGGA	0.203	0.039	0.17	98	478
GGCGGCATAAGGG	0.404	0.065	0.414	402	570
CCTGGTACAGGGA	0.031	0.002	0.033	29	850
GGTATCGCAAGCG	0.13	0.015	0.111	82	658
AGCAGCACATGTG	0.017	0.001	0.017	26	1502
GGTGTTATTAACG	0.098	0.017	0.077	76	917
CGTGTTATAGACG	0.029	0.001	0.028	33	1142
CGTGTCATAAACG	0.024	0.005	0.027	44	1603
ACTGTCGCTAATA	0.021	0.002	0.021	47	2196
CCTAGCGCATACG	0.029	0.01	0.025	38	1459
GCCGGTATTGAGA	0.073	0.012	0.069	92	1246
GGTGGTACTAGTA	0.087	0.028	0.063	46	681
ACTAGTATAAGTG	0.027	0.004	0.024	49	2026
GCTATCGTAGAGG	0.065	0.007	0.055	45	767
GGCGTCGCATATG	0.063	0.01	0.068	53	730
GCTATCATTAGGA	0.071	0.01	0.057	69	1140
CGTGGTACAGAGG	0.03	0.01	0.035	21	577
AGTGTCATTAATA	0.019	0.002	0.017	35	2054
GCCGTCGCTGAGA	0.051	0.008	0.04	31	739
AGCGGTGCTTATG	0.021	0.001	0.022	21	937
ACCGGCGTTTACG	0.026	0.004	0.021	17	776
CCCAGTATTTGGA	0.026	0.001	0.026	49	1850
CCTAGTACTGGCA	0.02	0.003	0.022	34	1534
GGCAGCACTGAGA	0.054	0.003	0.05	58	1101
CCTGGCACAGACG	0.024	0.002	0.027	39	1429
GCTATCACATGGG	0.074	0.013	0.055	42	716
CCCGTTGCAGGGA	0.02	0.003	0.021	24	1117
ACCAGTGCTGGCG	0.017	0.001	0.016	27	1643
AGCAGCATAGAGG	0.018	0.001	0.017	28	1623

GGTATCGCATGCA	0.112	0.034	0.097	92	861
ACTAGTACTGGTG	0.026	0.004	0.026	34	1277
AGCGGTATAAGCG	0.022	0.003	0.022	33	1436
CGTGGTATTAAGA	0.025	0.003	0.03	32	1048
GGCAGCGTTAACA	0.06	0.004	0.066	117	1660
AGTGGTATTTACG	0.021	0.002	0.02	14	674
GGCAGCGTTTACG	0.049	0.011	0.034	34	967
AGTGGCACAAGCG	0.024	0.003	0.02	14	670
GGTATTGCAGGGA	0.066	0.007	0.056	31	526
CGCGGTATATACG	0.02	0.002	0.019	28	1454
CCCATCATTGGTG	0.03	0.007	0.027	46	1677
GGCATTGTTTGGG	0.071	0.008	0.068	40	548
ACTATCACATGCG	0.024	0.002	0.022	44	1921
AGCATCGCAAATG	0.021	0.001	0.022	57	2560
GGCATTATAGGTA	0.051	0.003	0.055	103	1774
ACTATCACAGACG	0.029	0.005	0.023	59	2537
ACCGTTGTAGAGG	0.03	0.005	0.035	42	1150
GCTATTGCTTGGA	0.057	0.011	0.045	36	770
AGTATCATAAAGG	0.021	0.006	0.016	29	1796
CGCGGTGCAGAGA	0.025	0.004	0.026	28	1058
AGCAGTGTATGTG	0.022	0.006	0.029	37	1242
CCCGGCATTGAGG	0.023	0.001	0.024	29	1164
CGTGGTGCAGACA	0.03	0.01	0.019	21	1059
GGCGGCGCAAATA	0.102	0.021	0.105	149	1269
ACTGTCGTTGAGA	0.021	0.004	0.018	19	1039
CCTGTTGCAAACG	0.03	0.003	0.029	55	1860
ACTATCGTAGGTA	0.025	0.007	0.018	29	1582
GGTGTCATAGAGG	0.098	0.016	0.084	40	437
GGCAGTGTTAACA	0.057	0.006	0.05	91	1744
AGTGGTACAGAGG	0.028	0.006	0.025	17	654
GGTGTCGTATAGA	0.09	0.024	0.055	36	614
CGTAGCGCATACG	0.023	0.009	0.024	22	888
GCTATCGCAAAGG	0.06	0.011	0.072	67	864
GGTGGCACAAATA	0.191	0.022	0.177	264	1227
GCCAGTGTATATG	0.043	0.006	0.036	49	1314
AGTAGTACATATA	0.019	0.003	0.015	33	2161
CCTGGTGCAGACA	0.025	0.006	0.029	55	1831
AGCGGTATTTATA	0.023	0.003	0.02	41	2036
AGTGGCGCATGTG	0.022	0.004	0.026	13	484
AGCAGCACTGACA	0.02	0.002	0.017	47	2710

GCTGGTGCATACG	0.302	0.037	0.313	255	559
ACTGGTGTTTGGG	0.031	0.006	0.029	16	528
GGCAGTGCTGGGA	0.055	0.009	0.061	32	489
ACTGTCGTTAAGA	0.028	0.005	0.026	38	1402
CCCGTTACATGGA	0.025	0.001	0.026	46	1730
AGCGTCATATACG	0.023	0.003	0.019	42	2160
CCTAGCGTAAGCA	0.029	0.004	0.025	51	2016
CGTGTCATATGTG	0.026	0.005	0.033	27	797
CGCAGCGTAGACA	0.028	0.006	0.034	70	1984
GGTATTGTTAATG	0.113	0.014	0.123	119	852
CGCAGTACTAATA	0.023	0.002	0.025	64	2543
ACTAGCGCAAGCG	0.026	0.005	0.021	32	1476
GGCATCGTTAAGA	0.108	0.012	0.106	163	1370
AGTAGCATATATA	0.026	0.002	0.023	54	2303
ACCGTTATTGGTG	0.026	0.006	0.018	25	1373
ACTATCATTTGTG	0.03	0.004	0.029	49	1659
CGCGTTACTAAGG	0.027	0.002	0.025	30	1190
CGTATCGCTTGTA	0.024	0.004	0.026	25	940
GCCAGTGTTTGGA	0.041	0.012	0.028	25	860
CCCAGCGCATATA	0.024	0.004	0.019	62	3215
AGTGGTGTTTGCA	0.023	0.007	0.015	12	787
CCTATTATTAGCA	0.025	0.003	0.025	73	2837
CCTGTTGTAAAGA	0.025	0.001	0.026	51	1942
CCTGTCGCATGGA	0.031	0.006	0.026	27	1029
ACTGGTGTTAAGA	0.026	0.003	0.022	31	1374
AGCGGCATATGCA	0.022	0.001	0.023	33	1408
GGCGGTATTTGTG	0.117	0.025	0.111	77	614
AGCGTTGTTTACG	0.03	0.003	0.027	35	1263
ACTGTCGTTTACA	0.023	0.002	0.022	36	1575
GCTAGCGTATATA	0.064	0.003	0.066	91	1281
GCCGTTATTTGGA	0.056	0.012	0.044	52	1128
AGTGTCACTTGCG	0.022	0.001	0.02	13	639
AGTAGTATATAGA	0.024	0.002	0.023	48	2031
CCTGGCACAGATA	0.025	0.003	0.027	53	1922
GGCAGTGCAAACG	0.039	0.003	0.034	50	1437
GCCGTCATTGGTA	0.051	0	0.052	63	1159
AGTGGTGTAAGCA	0.015	0.001	0.014	15	1085
CCTATTGCTGACA	0.022	0.002	0.019	49	2493
ACCAGCATATGCG	0.024	0.003	0.024	56	2270
GGTAGCACATAGA	0.07	0.011	0.059	56	892

AGTGGCGTAGATA	0.028	0.003	0.03	26	829
ACCGGTGCTTATG	0.025	0.005	0.03	37	1215
ACTAGTGTAAGGG	0.03	0.009	0.018	22	1212
CGCGGCGCAGACG	0.025	0.004	0.02	22	1084
GCTAGCACTTAGG	0.07	0.006	0.069	52	701
AGCGTCACTGACA	0.021	0.002	0.022	53	2307
AGTGTCACAGACG	0.02	0.006	0.016	16	1016
ACCATCATAGGTG	0.031	0.005	0.025	49	1937
CCTGTTGCTGGTA	0.024	0.005	0.017	18	1033
CGCGGTACTAGGG	0.014	0.001	0.015	10	661
GCCGGTATTTGCG	0.157	0.029	0.151	152	853
GCTATTGTAAACA	0.054	0.002	0.053	159	2817
AGTAGCGTTGGCA	0.021	0.007	0.029	25	826
CGTATTACATATA	0.027	0.008	0.016	38	2370
CGTAGTATATATG	0.022	0.005	0.016	20	1268
ACCAGCATAGGCG	0.027	0.003	0.023	43	1813
CGTGGTGTATGCG	0.03	0.003	0.026	13	481
GCTAGCATTTGGA	0.057	0.005	0.064	55	807
AGTGGCGTAGGTG	0.024	0.018	0	0	264
CCTAGTGTTTATA	0.02	0.001	0.02	41	2024
AGTGTTGCTGATA	0.022	0.003	0.023	26	1081
AGCATTACTGGGA	0.019	0.004	0.014	21	1469
CGTGGCATTTGTG	0.022	0.008	0.024	9	363
AGCGGTGCAAATG	0.023	0.007	0.016	24	1474
AGCAGTACAGATG	0.021	0.003	0.017	36	2061
AGTATTACTAAGA	0.023	0.003	0.019	38	2013
CCTAGCGCTTACG	0.018	0.003	0.014	17	1198
CCTATTATATGTG	0.026	0.004	0.02	42	2093
ACTGTCACATATA	0.025	0.002	0.025	74	2939
AGCGTCATTTGTG	0.018	0.005	0.014	16	1119
AGTAGTGCTAAGA	0.025	0.006	0.033	39	1145
GGCGTCATTAGTA	0.072	0.003	0.068	84	1151
AGTATCATATAGG	0.023	0.003	0.022	28	1230
AGTAGCACTTACG	0.023	0.002	0.023	27	1125
ACCAGTGCATGGA	0.022	0.002	0.021	39	1823
CCTAGTGCAGACA	0.022	0.002	0.023	57	2421
AGCAGTGCTGATG	0.022	0.006	0.024	33	1337
GGTATTACAGGCA	0.08	0.02	0.052	61	1107
GCTGTCATTGAGA	0.062	0.004	0.056	59	997
AGTAGTATTGGTG	0.017	0.003	0.022	21	939

GCTAGTATAGATG	0.056	0.01	0.042	59	1356
CGCGGTACTGATA	0.024	0.005	0.021	32	1512
ACCAGTATTAACG	0.022	0.003	0.018	68	3659
CCTGGTGTAAATG	0.022	0.002	0.025	40	1546
AGCAGTGTAGGCG	0.023	0.005	0.017	25	1428
GCCATCGCAGGTA	0.043	0.009	0.037	46	1191
GGTGTTATTGGCG	0.071	0.004	0.072	41	525
GGCGTTGTAGAGA	0.065	0.006	0.07	70	936
CCCGGCGCTTATG	0.023	0.004	0.021	26	1196
GGCATTGTTTGCG	0.077	0.007	0.07	59	789
CCCGTCGTTAATG	0.022	0.001	0.022	38	1685
CGCGTTGTTTATG	0.029	0.006	0.034	32	919
CCCGTTGCTTACA	0.021	0.001	0.02	46	2270
CGTGGTACATATA	0.027	0.006	0.02	28	1350
ACCGGCACAAAGG	0.024	0.003	0.026	53	1957
AGTATTGTAAACA	0.024	0.003	0.02	62	3073
CGTGGCACTTAGG	0.037	0.015	0.016	8	481
AGTGTCGTTTACA	0.021	0.006	0.02	25	1200
AGTAGCGTATGTG	0.026	0.006	0.033	26	751
AGCATTACTAGCG	0.02	0.004	0.016	32	2002
GCCAGTATTTGTG	0.056	0.004	0.051	68	1277
GGTGTCATAAATG	0.107	0.011	0.094	93	894
AGTGTTATAGAGA	0.022	0.001	0.021	33	1507
CGCAGTATTAGGA	0.033	0.005	0.038	55	1394
CGCGTTATTTGGA	0.029	0.003	0.025	26	1032
AGTGGCACTTGCA	0.026	0.006	0.018	15	797
GCCGTCATTTAGA	0.053	0.004	0.05	71	1357
CGCGGTATAAGGG	0.028	0.007	0.019	17	873
CCTGGCACAGATG	0.021	0.004	0.016	20	1209
AGCGGTACTGAGG	0.023	0.004	0.018	17	911
CCTGTCATTGACG	0.034	0.003	0.033	51	1475
ACTATCATAGATG	0.029	0.004	0.025	61	2388
CCTGTCGTAGACA	0.022	0	0.021	43	1995
GGCAGCATTTGTA	0.052	0.008	0.042	57	1289
ACTGGTGTAGGCG	0.021	0.003	0.016	14	853
GCCAGTGCATGGG	0.042	0.004	0.04	25	605
CGTAGCATATAGG	0.025	0.007	0.019	19	959
ACCAGTATTGGGG	0.027	0.006	0.036	39	1051
GCTGGTGCAAACG	0.294	0.038	0.304	290	663
AGTGTCGTTTGCG	0.039	0.002	0.037	21	553

ACCGGTGCAGGTA	0.023	0.004	0.019	30	1585
CCTAGCACTGAGA	0.028	0.001	0.03	55	1777
GCTAGCATTAGGG	0.07	0.014	0.05	29	550
GCCAGCGCAGACA	0.054	0.011	0.053	89	1577
ACCGGTGCTTAGG	0.027	0.007	0.019	19	987
ACTGTTGTTTGGG	0.024	0.005	0.017	11	644
GGCGGTACAGGGA	0.06	0.008	0.052	39	708
GGTGGCATATGCA	0.49	0.04	0.463	498	578
GCCGTTGTAGAGA	0.045	0.008	0.051	60	1117
CCCGTCATATAGA	0.029	0.004	0.035	90	2483
CGCGTCATATGTA	0.027	0.002	0.024	39	1561
CCTGGTACTGACA	0.022	0.006	0.016	29	1757
CGTGGTACTAGCG	0.034	0.011	0.026	16	591
GCTATTGCTAACA	0.057	0.008	0.046	96	1977
CCTAGTGTTTGTG	0.026	0.005	0.024	27	1083
CCTATCGCTGAGG	0.025	0.004	0.02	21	1029
GGCAGTGCATGTG	0.042	0.009	0.036	28	752
CGCAGCGTTAGGG	0.023	0.005	0.019	13	688
GCTGGTACAAACA	0.077	0.004	0.076	141	1716
AGTATTACTGGTG	0.02	0.006	0.023	22	952
ACTAGTATAAGGA	0.022	0.002	0.023	54	2335
CGTAGCGTTGAGA	0.03	0.004	0.036	28	758
GCCGTTATTTGTG	0.056	0.002	0.055	57	982
GGTAGTGCTAACA	0.061	0.006	0.069	72	975
CGTATTACAAGTG	0.025	0.006	0.026	35	1297
AGTGTCGTATGGA	0.024	0.004	0.019	14	730
ACTGGCATAAGTG	0.023	0.005	0.018	21	1128
CGCGGCGTTAAGG	0.028	0.001	0.026	19	715
GCTGTCACTGAGG	0.057	0.002	0.059	39	619
CCCAGCATTGACA	0.027	0.001	0.026	92	3386
AGCAGCGCAAACG	0.024	0.005	0.02	43	2056
CCTAGCATAAGTG	0.022	0.001	0.021	35	1616
GCTAGCGTTTAGG	0.073	0.018	0.05	25	473
CGTAGCGTAGAGA	0.022	0.004	0.016	14	835
CCCAGCGCTTGGA	0.03	0.005	0.027	44	1557
CGTAGCATTTGGA	0.022	0.006	0.02	14	700
GCTGTTGCTTGTG	0.052	0.012	0.039	19	473
CCTGGTATAAGGG	0.034	0.002	0.037	32	844
ACCGGCGTTGGCG	0.031	0.002	0.031	18	562
CGCGTCGCATAGG	0.029	0.003	0.033	32	924

GGCGTTACATGTG	0.056	0.004	0.05	42	796
AGTAGCGTAAATG	0.024	0.005	0.022	31	1405
AGCATCGTATAGA	0.019	0.001	0.019	44	2321
CGTGGCACTGATG	0.032	0.008	0.026	17	631
CCCGGTGCATACA	0.021	0.001	0.02	49	2381
CGCAGCGTATGGA	0.021	0.004	0.025	28	1092
GGTATCGCTTACG	0.144	0.021	0.138	98	613
CGCGGTGTATACA	0.026	0.004	0.023	37	1596
AGCAGCACTTACA	0.019	0.002	0.021	55	2593
AGTATCGTTTGTG	0.033	0.011	0.027	23	844
CCTAGCGTTTGCA	0.02	0.004	0.016	22	1381
ACCAGTGTTAACG	0.026	0.004	0.024	61	2450
ACCGTTATAGGGG	0.03	0.009	0.036	34	913
CCTAGCATTTACA	0.024	0.004	0.023	54	2291
GCCAGTGTTTGTA	0.049	0.012	0.049	58	1118
GGTAGTGTAGACA	0.068	0.013	0.062	64	974
GGCATCATATGGG	0.061	0.008	0.06	68	1061
CCCGTTGTATGCG	0.026	0.002	0.023	34	1433
AGCGGCATATACA	0.021	0.002	0.02	39	1914
AGCATCGTATGTA	0.022	0.001	0.02	44	2196
GGCGGCACAAAGA	0.137	0.013	0.14	203	1252
ACCGGCGCTGACG	0.029	0.001	0.029	39	1315
GCTAGTGTATATG	0.056	0.006	0.048	54	1065
ACCATCGTTAGCG	0.023	0.002	0.021	38	1748
AGTATTGCTTGTA	0.025	0.004	0.02	23	1145
AGTGGTACAGATG	0.031	0.004	0.035	31	852
ACCGTCGTAAGCG	0.023	0.002	0.023	33	1373
CGCATCGTATAGG	0.029	0.006	0.02	26	1258
GGCAGTATATACA	0.06	0.005	0.055	145	2485
CGCGTTACAAGCG	0.019	0.004	0.013	19	1415
AGCGGCGTTAAGA	0.025	0.004	0.026	30	1118
AGTATCATTTGGA	0.025	0.007	0.016	19	1171
GGTAGTACTTGTA	0.058	0.007	0.05	39	744
CGTGGTGTTTGTA	0.02	0.003	0.021	11	509
ACTAGTATAAAGA	0.023	0.001	0.023	83	3599
GGCAGTACTAGTA	0.057	0.004	0.059	86	1380
CGCGTCACTAAGG	0.02	0.001	0.021	25	1140
GCTGTTACTGAGA	0.063	0.001	0.063	64	949
CCTAGTATTGAGG	0.032	0.006	0.038	47	1188
CGCGTTGTATGGA	0.024	0.002	0.023	22	919

AGCGTTGCAGACA	0.024	0.005	0.026	53	2002
CGCATTGTTGGCA	0.025	0.001	0.024	34	1391
GGTGTTATTAATG	0.097	0.012	0.097	99	922
ACCATCGTATGGG	0.026	0.004	0.02	25	1195
CGCGTTGTATGTG	0.027	0.006	0.023	20	848
CGTATTACAGGGA	0.032	0.005	0.027	28	1022
GCTATCACTAATA	0.056	0.008	0.046	103	2146
CCTAGCATTAACG	0.03	0.005	0.024	50	2049
GGCATCGCAGGTG	0.098	0.013	0.079	66	766
GGCGGTATAAACA	0.066	0.013	0.048	107	2114
CGTAGCACTAGTG	0.028	0.007	0.022	17	743
GCCGGTGTTAGCG	0.389	0.075	0.347	314	590
CCTGGCACAAAGG	0.024	0.003	0.021	26	1221
GCTATCGTAGATA	0.057	0.007	0.053	84	1508
AGTGGCATATAGA	0.018	0.003	0.018	13	721
CCTGGCGCATAGA	0.024	0.005	0.029	31	1053
GCCATTATTTGCA	0.048	0.001	0.049	116	2266
CGTATCGCTGGGA	0.019	0.006	0.024	15	612
GCCAGCATTAAGA	0.052	0.007	0.042	101	2307
GGCGGTATATGGG	0.102	0.017	0.091	64	640
ACCGGTATTGGTG	0.028	0.007	0.032	39	1174
GCTAGCATAGGGG	0.07	0.011	0.065	36	518
ACTAGTATTAATG	0.026	0.003	0.022	56	2501
ACTATTGTAGATG	0.024	0.007	0.018	36	2013
CCTGGCGCAGGGA	0.024	0.007	0.017	12	685
ACTGGCATAAAGA	0.027	0.003	0.026	59	2230
CGCGTCGCAAACA	0.022	0.002	0.025	59	2348
GCTGTCATAGAGG	0.071	0.007	0.061	50	765
AGCAGCGTAGAGG	0.024	0.004	0.025	29	1118
ACTGGCACAGGCA	0.022	0.003	0.02	32	1569
CCCAGCATTGGCA	0.032	0.001	0.03	66	2135
GCCGTCGTAAGTA	0.051	0.003	0.052	72	1317
AGCATTATAAGCG	0.024	0.003	0.024	64	2619
GGCGTTGTTGGGA	0.071	0.017	0.058	33	537
GGCATTATTTGGA	0.062	0.001	0.061	83	1276
ACTAGTATAGGGG	0.033	0.007	0.025	24	918
GCCATTGCATGTA	0.036	0.004	0.039	58	1430
ACTATCACTTACA	0.026	0.003	0.022	86	3800
GCTGTTGTTTAGA	0.061	0.005	0.068	54	742
CGCAGCGCTTGCA	0.022	0.002	0.021	27	1273

CCTGTTGTATACA	0.026	0.004	0.022	50	2213
AGCGTTACTGGTA	0.027	0.002	0.023	34	1422
GCCGGTACTAATA	0.062	0.005	0.056	117	1973
AGCGGTACTGATG	0.019	0	0.019	22	1136
ACCGTCGTTGATG	0.024	0.004	0.027	32	1167
ACTGGTGTAAGCA	0.028	0.006	0.031	49	1544
GCTGTCATTGGGA	0.051	0.003	0.049	32	622
GGCATCGTAAGTA	0.087	0.018	0.065	109	1569
CCCATTGTTTGTA	0.022	0.004	0.016	30	1844
ACCGTCACAGATG	0.025	0.003	0.029	65	2166
GCTGGTGCAGATA	0.122	0.014	0.121	105	760
CGCATTGTAGGCA	0.028	0.004	0.022	38	1672
AGCGTTATAGGCA	0.02	0.002	0.022	44	1950
CCCAGTGTTAAGA	0.027	0.003	0.023	48	2069
GCCAGCACTGGTA	0.057	0.004	0.054	71	1254
AGCGTTGCTTATG	0.027	0.005	0.03	33	1069
CCTAGCATTAGTA	0.023	0.001	0.022	40	1762
CGTATTGCAGAGA	0.021	0.001	0.023	26	1115
GCTGGCGCATGTA	0.622	0.052	0.607	687	445
GCTGGTGTAGATG	0.741	0.031	0.749	811	272
GGCGGCATTTATG	0.545	0.065	0.493	619	637
ACCAGTATAGAGA	0.022	0.002	0.023	76	3223
ACTGTTGTATGGG	0.031	0.007	0.04	32	765
CGCATTGCTGAGG	0.023	0.005	0.03	25	811
ACTAGCGTATGTG	0.027	0.006	0.035	36	990
AGCAGTACTTGGG	0.015	0.002	0.015	16	1044
ACTGGTGTTGACG	0.027	0.012	0.019	19	957
GGTGGCGCTAATA	0.158	0.023	0.136	98	625
CGTGTCACTGGGA	0.034	0.011	0.02	12	587
CCTGTTGTTAGGG	0.034	0.01	0.028	21	736
GCCATTACTAACA	0.051	0.004	0.047	161	3270
CGTGTTACATAGG	0.032	0.003	0.032	26	794
AGCGTCATATGTG	0.027	0.004	0.031	44	1392
GGTGTTACATGTA	0.078	0.01	0.071	65	854
GCTGGCGTTAATA	0.872	0.032	0.861	1760	283
GCCATTATATACG	0.054	0.009	0.043	121	2690
CCCAGCGTTAGGG	0.027	0.007	0.018	18	987
CCTAGTACTAGTG	0.023	0.003	0.021	27	1267
ACTATCGCATGGA	0.024	0.006	0.028	48	1669
CGTGTTACATGTA	0.021	0.008	0.032	38	1143

CCTGGTGCTTGTA	0.029	0.001	0.027	30	1070
CGTAGTACAAAGA	0.022	0.004	0.024	39	1583
GGCAGCATATATG	0.05	0.007	0.042	65	1468
CGCATCATAGGCG	0.03	0.004	0.026	41	1522
CGTGGTGTATATG	0.031	0.006	0.023	15	624
AGCATCGCTTGGG	0.022	0.008	0.02	18	875
GCTATCATTGAGG	0.055	0.008	0.044	49	1058
ACCGTTGCAAGGG	0.027	0.004	0.022	30	1332
AGCAGTGTAAGTA	0.021	0.002	0.018	43	2361
CGTGGTACTGAGG	0.024	0.004	0.021	10	472
GCCATCATTTACA	0.052	0.004	0.047	151	3034
GGCAGCGCTGGGG	0.051	0.009	0.043	21	471
GCTAGTATTTGTG	0.06	0.01	0.047	43	880
CGTATTGTTGGTA	0.018	0.008	0.006	6	940
CCTGTTGCTGATA	0.029	0.006	0.028	47	1633
CCTAGTACAAACG	0.029	0.001	0.029	78	2618
CCCATTATTTGGA	0.025	0.004	0.028	59	2055
CGCAGCGCTAGCA	0.022	0.004	0.023	35	1515
GCTAGCACATGGA	0.067	0.006	0.075	68	842
CCTAGTGTTAAGA	0.03	0.002	0.031	63	1946
ACTAGCGTAAACG	0.024	0.006	0.033	63	1873
ACTGGCGTAGGGA	0.021	0.009	0.01	7	697
GCCGGTGCTTGGG	0.12	0.014	0.107	47	392
GCCAGTATTTAGG	0.048	0.003	0.044	55	1183
ACCGGCATATAGG	0.021	0.004	0.016	23	1405
CGTAGCGCATAGA	0.03	0.003	0.027	26	940
CCTGGCGCAGACG	0.018	0.004	0.019	21	1114
AGCATTGCATATG	0.022	0.003	0.024	52	2075
GCCAGCGTTTGCG	0.056	0.018	0.04	34	809
GGCGGTATTGAGG	0.124	0.029	0.1	61	548
ACTGTCATTAGGA	0.027	0.004	0.021	30	1378
GCTGGCGCTAACA	0.621	0.033	0.633	1072	622
AGTGGTATAAGTA	0.023	0.001	0.022	34	1491
CCTAGTATAAGCA	0.025	0.005	0.028	77	2686
CGTGTTGTTAACA	0.029	0.008	0.019	24	1249
CGTGGCACATGTG	0.029	0.013	0.033	20	589
CCCGGTGTTTGCA	0.02	0.001	0.02	26	1291
ACTATCGTATAGG	0.034	0.002	0.032	48	1433
CGCGTTACATGGG	0.021	0.006	0.024	19	768
AGCGGCGTTTGCA	0.023	0.002	0.022	21	952

CCTGGCGTTTAGG	0.022	0.002	0.024	18	720
GGTGGCATAAAGA	0.482	0.057	0.461	706	827
CCCAGCACTGGTG	0.034	0.005	0.038	52	1302
CCTATTGCAAGTA	0.024	0.004	0.024	62	2523
CGTGGTGCTTATG	0.034	0.002	0.031	18	566
CCCGGCGCTTGGG	0.028	0	0.028	20	699
GCCAGTACATGCA	0.056	0.003	0.058	122	1977
CCCAGCGTATATG	0.024	0.004	0.029	58	1942
GGTATTATAGAGG	0.117	0.012	0.108	95	786
ACTATCATTAATG	0.028	0.006	0.022	66	2940
GGCATTATATGGA	0.057	0.004	0.055	85	1454
GGCGTCGCAAGCA	0.07	0.017	0.058	73	1182
AGTAGTACTGGGA	0.026	0.003	0.03	23	749
CCTATTGTTGGCG	0.026	0.002	0.024	33	1330
AGTGGTACTTAGA	0.029	0.003	0.027	25	891
GCCATTACAGGCG	0.053	0.005	0.054	85	1495
GCTGGTACTTATA	0.082	0.006	0.085	93	996
GGTGGTACAGGCA	0.089	0.004	0.084	55	602
CCTATCATTGGGG	0.047	0.008	0.042	38	876
GGCGGTATATATG	0.129	0.005	0.129	142	963
GCCAGCGCAGATG	0.05	0.011	0.034	32	899
CCCGGCGCAGGTG	0.025	0.003	0.028	30	1027
CGTATTACTAGCG	0.026	0.005	0.022	26	1157
GGTGGTATAGACA	0.118	0.023	0.095	97	927
CCTATCACTTAGA	0.025	0.002	0.026	62	2316
ACCGTTGCATATG	0.026	0.005	0.019	37	1920
ACTAGCACAGAGA	0.022	0.003	0.02	51	2546
CGCAGCGCTAGTA	0.022	0.003	0.019	24	1263
CGTAGCGTTTGCA	0.03	0.006	0.023	19	810
CGCAGCACTTGTA	0.021	0.002	0.02	29	1403
ACTGGTACATGCG	0.024	0.004	0.027	34	1230
CGTATTATAAATG	0.026	0.001	0.026	67	2516
GCTGGTACTGATA	0.084	0.003	0.083	92	1018
AGCGGTATTAGCA	0.025	0.003	0.024	44	1823
ACTGGCGTAGATA	0.026	0.004	0.02	26	1246
GCTGGTACTTGGA	0.083	0.002	0.085	51	548
GCCATTACTGGTG	0.049	0.009	0.055	58	1003
GGCGTTACAGGCG	0.066	0.008	0.057	48	801
GCTGTCGCTAATG	0.067	0.008	0.073	48	614
ACTATCACTGGCG	0.029	0.01	0.018	28	1537

GCTGTTATAGGCA	0.065	0.014	0.053	61	1100
AGCATTATAGGCG	0.018	0.001	0.017	34	1976
CGTAGTACAGGGA	0.024	0.01	0.02	16	766
AGCAGCACATATA	0.024	0.003	0.026	83	3071
CGTAGCGCAAATA	0.031	0.004	0.036	56	1516
ACTGTTGTTAGGA	0.026	0.004	0.029	30	1007
ACCAGCGTTTGCA	0.024	0.001	0.025	44	1748
AGCAGCGCTGGGG	0.014	0.002	0.018	13	718
AGTGGTACAAGTG	0.03	0.004	0.025	23	909
AGCAGCATTAGCG	0.021	0.002	0.022	39	1751
CGCGTCGTTGGCG	0.021	0.005	0.026	25	921
CCTAGCACAGGCA	0.023	0.004	0.02	40	1999
ACTAGTGCAGGTG	0.028	0.003	0.024	30	1237
ACTGTTACTGGTA	0.023	0.005	0.017	29	1690
GCTGGTGCTAGTA	0.116	0.01	0.109	76	620
GGCGTCACAAGCA	0.061	0.014	0.043	74	1647
GGTATTATTAAGA	0.103	0.017	0.087	148	1557
GCTGGCATAGGCA	0.649	0.04	0.645	932	513
GGTAGCACTTGTA	0.065	0.018	0.046	35	733
GGCAGCGTATGTA	0.05	0.014	0.037	41	1067
AGCAGTGCAAAGG	0.022	0.001	0.021	35	1606
AGCGTCGCTTGTA	0.019	0.005	0.023	27	1127
CCTAGCGCATGGA	0.021	0.001	0.022	29	1297
AGTAGTGCAGGCA	0.022	0.004	0.016	16	980
CCTATCGTAAACG	0.024	0.005	0.018	49	2648
GGCGTTATTAGCG	0.055	0.013	0.054	50	883
CGTATTACTTGCA	0.028	0.003	0.032	47	1434
GCTATCATAAGGG	0.079	0.007	0.071	69	901
ACCGGCGCTTAGA	0.025	0.002	0.025	24	927
ACCGTCATAAACG	0.025	0.001	0.023	80	3404
AGCGTCACTAGGA	0.024	0.003	0.027	38	1347
ACTATCGTTAGTA	0.025	0.002	0.028	53	1810
CGCGGTACAAATG	0.026	0.002	0.027	44	1591
GGTAGCACATGTA	0.07	0.01	0.074	72	897
CCCGTCATTAGGA	0.023	0.004	0.021	35	1655
GGCGGCGCTTGCA	0.089	0.025	0.065	47	676
CCTGTTGTATGGG	0.029	0.007	0.02	17	850
GCCATTGCAAAGG	0.052	0.005	0.047	67	1361
ACTATCATAAGGA	0.03	0.003	0.032	82	2444
ACCATTGCTTGGA	0.025	0.003	0.022	41	1792

ACTGGTATTAGGA	0.029	0.002	0.028	35	1232
AGCAGCACTTATA	0.024	0.001	0.023	57	2457
GGTGTTATTAACA	0.085	0.004	0.079	117	1358
GGTGGCGCAAGTG	0.301	0.064	0.272	141	377
ACCGTCGCATGCA	0.023	0.001	0.023	52	2245
GCCAGTGTTTATA	0.04	0.001	0.04	65	1569
GGCGGCGCAAGGG	0.161	0.03	0.139	78	485
GCTATCGCAGATA	0.054	0.011	0.051	67	1238
ACCGTTGTTAGCA	0.029	0.004	0.028	49	1731
GGTATCGCAGGCA	0.1	0.023	0.087	79	827
CCTGGTGCAAGGA	0.02	0.005	0.014	16	1156
CGTGTCATTAGCA	0.021	0.001	0.022	25	1128
AGCAGTGCATAGG	0.025	0.005	0.026	31	1175
AGCGTTGTTTAGA	0.02	0.005	0.015	19	1287
ACTGTTGTAAAGA	0.023	0.001	0.022	43	1946
AGTGGTGCAAAGA	0.029	0.004	0.027	31	1118
GCCAGCGTTAGTA	0.047	0.007	0.055	70	1212
AGTGTTATTAAGA	0.018	0.004	0.014	26	1809
GCCATTGCTGAGG	0.045	0.003	0.045	42	884
CCCATTGTTTATG	0.028	0.002	0.025	49	1906
GGTAGCACTTATG	0.08	0.012	0.067	45	628
ACCATTGCTTAGG	0.022	0.004	0.017	29	1672
GGTATTGTAGAGA	0.102	0.022	0.086	88	932
AGTGTCACAAAGG	0.028	0.002	0.025	27	1040
CGCATTACAAGCA	0.025	0.001	0.027	71	2581
GGTAGCACAGAGA	0.071	0.006	0.07	58	772
AGCGGCGTAGATA	0.021	0.004	0.016	22	1376
GGCATCATAAATG	0.103	0.021	0.084	205	2229
CGTAGTGTATGTG	0.024	0.007	0.027	17	608
GCCGGCACTGAGA	0.101	0.003	0.105	119	1009
AGCGTTGTAGGGA	0.024	0.007	0.02	21	1048
CCCAGCATTGGGA	0.038	0.002	0.036	54	1456
CGTGTCGTAGATG	0.023	0.006	0.023	17	726
CCCATTGTATATA	0.025	0.003	0.029	91	3019
GCCGTCACAAATG	0.053	0.008	0.042	84	1898
CGTGTTGCAAGCA	0.026	0.004	0.027	31	1109
CGTAGTATAAAGA	0.025	0.002	0.028	53	1870
ACTAGCATTAGCA	0.028	0.003	0.025	59	2257
CGCATTGTTTATG	0.025	0.004	0.02	28	1376
CCTGTTGTTGAGA	0.023	0.003	0.022	26	1169

CGTAGCGCTGAGG	0.024	0.002	0.022	12	544
GCTGTCATAAATA	0.059	0.008	0.049	114	2226
GCTGGCACAAAGA	0.202	0.029	0.193	266	1113
AGTGGTGCTTAGG	0.03	0.01	0.043	17	382
CCCGGTGCAGGCA	0.024	0.003	0.021	36	1697
GCCGGTGTTGATG	0.415	0.055	0.411	383	550
CCCATTGTTAATG	0.029	0.003	0.031	75	2353
GGCATCACTTGCG	0.072	0.006	0.08	84	970
CCCAGCGTTTGCA	0.025	0.005	0.025	49	1938
GCCGTTGTTTGTA	0.044	0.011	0.028	28	962
AGTGTTATAGATG	0.024	0.004	0.02	25	1249
CCCATCGTAGAGG	0.029	0.004	0.024	37	1530
CCCGTCGTAAATA	0.023	0.002	0.026	88	3274
GGCATTATAGGGG	0.062	0.006	0.061	71	1085
CCCGTTACTGGGA	0.027	0.002	0.027	34	1241
ACCGGCGCAAGTA	0.023	0.006	0.017	26	1460
CGTATTATTTGCA	0.025	0.007	0.031	52	1609
GGTGTCGCAAATG	0.089	0.012	0.073	52	657
AGTATTGTAGACA	0.023	0.003	0.026	58	2212
CGTGGTACTGGGG	0.027	0.013	0.04	13	312
ACTAGCGCATGCG	0.025	0.001	0.024	33	1314
CCTAGCATTGATA	0.026	0.002	0.029	66	2237
ACCGTCATTGGTG	0.026	0.004	0.028	38	1306
CGTGTCACTTGTA	0.016	0.004	0.022	20	871
AGTAGTGCATGGA	0.022	0.005	0.014	12	818
GGTAGTGCAAATA	0.06	0.007	0.052	73	1318
CGTAGCACATGCG	0.028	0.005	0.022	19	841
CGCGGCGCTAGCG	0.023	0.003	0.023	18	756
GGCGTCACTAAGG	0.065	0.003	0.065	57	819
CGTGTCGCTAATG	0.022	0.005	0.019	17	875
ACCGTTGTAGGTA	0.02	0.005	0.013	20	1523
AGTATCGCAAGCA	0.023	0.004	0.019	31	1566
ACCGTCGCAGATG	0.027	0.004	0.029	47	1576
GCTAGTGTTTATA	0.052	0.004	0.048	69	1375
AGCATCATATGGA	0.024	0.001	0.025	59	2316
CGCGTTGCAAGTG	0.024	0.004	0.026	28	1029
GCCAGTGCTTAGA	0.032	0.007	0.042	44	996
ACCATTGTAAGTG	0.025	0.004	0.028	60	2117
ACTGTTACTTAGA	0.026	0.003	0.022	42	1874
ACTATCGCTAATG	0.029	0.006	0.033	65	1916

CCTGGTATTAAGG	0.027	0.002	0.025	28	1099
CCTGGCGTAAACG	0.021	0.003	0.024	35	1444
CCTAGTATATAGG	0.024	0.002	0.026	41	1549
GGCGGTGTTGACA	0.075	0.019	0.063	65	959
ACCGTCATAAGTG	0.023	0.004	0.019	43	2212
CCCATCGTAAGGG	0.038	0.01	0.027	38	1388
AGCATTGTAGATG	0.019	0.001	0.019	37	1938
CGCGGCACAAACA	0.024	0.004	0.018	49	2693
ACCATCATAGGGA	0.032	0.003	0.037	74	1948
GGTAGTATATACA	0.062	0.004	0.065	116	1678
GCTAGTGCATGCA	0.055	0.003	0.051	59	1108
CGCGGCGCTTAGG	0.022	0.007	0.016	10	603
AGCATCGCTTATA	0.023	0.005	0.017	40	2267
AGTGTCGCTAGTG	0.029	0.004	0.029	17	560
GGTGTTGTATGCA	0.086	0.015	0.065	46	659
ACTATTACAAGGA	0.024	0.003	0.023	73	3048
CCCAGCGTAGGGA	0.045	0.005	0.041	54	1254
AGTAGTGTTGATG	0.036	0.008	0.037	33	850
CGTGTTATATAGG	0.026	0.006	0.02	18	867
AGTGTCGCTTGTG	0.032	0.005	0.032	15	457
ACTGGCGCTGGTA	0.025	0.006	0.034	19	537
GGCATCGTAGGTG	0.094	0.008	0.096	86	806
CGTAGTGTAAACA	0.026	0.006	0.022	39	1763
GGCAGCGCTTGTA	0.066	0.009	0.055	47	804
CCTAGCGTATGTG	0.029	0.002	0.032	35	1066
CCCAGTACTAATG	0.027	0.003	0.031	86	2719
CCCATTGCAGAGG	0.025	0.005	0.018	29	1591
AGCATCACTAACA	0.021	0.001	0.022	93	4141
CCCGGTACAGATA	0.025	0.002	0.028	75	2606
GCTAGCGCTTACA	0.07	0.015	0.053	55	985
CGTAGCATAAGCA	0.027	0.003	0.03	47	1537
ACCGGTGCTAGTG	0.029	0.008	0.026	27	1018
GCCATTACTGACG	0.063	0.008	0.053	99	1773
GGCATCGTATGGG	0.107	0.009	0.107	81	676
CCCGTCATAGAGG	0.039	0.004	0.033	52	1530
CCTATCACAGGGG	0.044	0.005	0.049	53	1036
GCCGGTATAAACA	0.077	0.002	0.078	245	2898
CGCGGTATTGGTG	0.023	0.008	0.023	16	666
ACTATTGTATAGG	0.034	0.006	0.043	76	1678
AGCGGCGCTTACA	0.024	0	0.024	35	1448

CCTGGCGTTAGGA	0.023	0.007	0.032	29	877
CGTGGTATTTATG	0.027	0.009	0.021	14	658
GCTGTCACAAGTA	0.066	0.004	0.061	80	1221
CCTGGTGTAGACG	0.028	0.001	0.028	34	1171
AGTGTTATATATG	0.025	0.004	0.029	44	1473
CGCAGTATTAGCA	0.025	0.003	0.028	60	2089
ACTAGCACAGGGA	0.035	0.006	0.032	45	1362
AGCGGCATAGATG	0.032	0.004	0.031	20	635
CGCGTCATAAAGA	0.023	0.001	0.022	49	2154
AGCGGTGCAGACA	0.025	0.003	0.025	43	1655
CGTATCACAAGCA	0.02	0.007	0.012	21	1770
ACCGTTGTTGGCG	0.023	0.003	0.021	37	1739
CCCGGTATTTGCA	0.022	0.002	0.019	33	1744
ACTAGTGCTGACA	0.022	0.001	0.023	61	2540
GCCAGCATTAGCG	0.059	0.003	0.063	96	1434
CGTATCGTAAACG	0.033	0.01	0.021	38	1730
AGCGGCATTGGTA	0.03	0.005	0.029	28	937
ACTATTGTTTGGG	0.028	0.005	0.021	22	1040
CGCGGCATAAAGG	0.027	0.003	0.027	28	1028
GCCATCGTTGGTA	0.052	0.011	0.036	44	1175
AGCGTTATAGGTA	0.025	0.002	0.026	50	1867
GGCGTTGTTAGTA	0.051	0.01	0.04	39	939
ACTAGCGTTGGCG	0.025	0.003	0.022	21	947
CCCGTTGTTAGGA	0.026	0.002	0.027	34	1216
ACCGGTGCTGGCA	0.024	0.002	0.021	35	1610
CGTATCGTTTATG	0.03	0.004	0.024	26	1036
GGTAGCACTGATG	0.066	0.003	0.063	45	675
AGTATTACAGACA	0.022	0.006	0.018	47	2501
CCTAGTGCATATG	0.027	0.004	0.023	36	1559
GCTAGTGCAAGGA	0.052	0.003	0.051	51	952
AGTGTCGTATGTA	0.02	0.002	0.023	23	991
CGTGTTGCTTGTA	0.02	0.003	0.018	13	696
AGCATTGTAGGCA	0.022	0.004	0.016	35	2115
GGCATTGTTAATG	0.08	0.003	0.076	106	1281
AGCGTTATAAGTA	0.023	0.002	0.02	54	2670
AGTGTTGTTAATA	0.023	0.005	0.024	41	1647
GGTATTACAGGGA	0.081	0.007	0.083	68	751
CCTGTTGCAGGCA	0.026	0.004	0.021	31	1442
GGCAGCGTTTGTA	0.059	0.005	0.06	54	847
CCTATTGCTGGGG	0.03	0.008	0.034	28	784

ACTGGTACAGGCG	0.026	0.008	0.024	25	1023
ACCGGTGCAAGGA	0.019	0.003	0.018	30	1623
AGTAGTGCTGGGA	0.027	0.006	0.019	11	558
GCCGTCATTGGCA	0.062	0.005	0.065	94	1350
ACCGGCGCTGGGG	0.032	0.006	0.027	16	573
AGCAGCGCTGGCA	0.02	0.003	0.022	29	1280
GGCGGCATTAATA	0.298	0.051	0.272	515	1378
CCTGGTGCTAATG	0.029	0.008	0.027	30	1064
GGCATCATTGACG	0.072	0.009	0.068	107	1469
GGTAGCACTGGTG	0.045	0.008	0.054	21	369
ACCAGCGTATGCA	0.024	0.005	0.026	64	2372
CCTGTTGCTAATA	0.021	0.004	0.02	40	1977
CGCGGCGTAAATA	0.02	0.006	0.013	24	1796
CCCATTATAGGCA	0.026	0.001	0.025	85	3267
GCTATCATTTACA	0.062	0.003	0.065	141	2039
CCTATCACATGGG	0.041	0.003	0.038	48	1229
GCTGTCGTTTACG	0.052	0.005	0.049	34	657
CGTGTTATTAACA	0.023	0.002	0.023	44	1911
GCCGTCACATAGA	0.052	0.006	0.044	77	1669
ACCATTGCATGGG	0.03	0.005	0.037	60	1576
CGCATTATTGGGA	0.032	0.004	0.034	43	1237
GGTAGCGCTTAGA	0.069	0.014	0.055	30	516
GGCGTCGCTAAGG	0.073	0.01	0.07	49	650
GGCAGCGTTTAGA	0.066	0.012	0.067	64	891
AGCGTTATTGGGA	0.019	0.002	0.015	18	1153
CGCGGCACAGAGA	0.026	0.007	0.017	23	1352
AGCAGTGCAAGCG	0.027	0.002	0.026	41	1541
AGTAGCATATGGG	0.022	0.001	0.022	17	741
ACCATTGTTAAGA	0.025	0.004	0.027	79	2899
CCCGGCGTTAGTG	0.016	0.006	0.017	17	988
CCCATCATAGGTG	0.039	0.005	0.035	66	1816
ACTATTGCTGGGA	0.024	0.007	0.014	21	1437
CGTATCGCAGGGG	0.025	0.006	0.016	9	566
CGTAGCACAGGGA	0.03	0.002	0.031	24	756
ACCGTCATTAGTG	0.023	0.003	0.021	36	1703
ACCGTCACTGACG	0.027	0.005	0.021	43	1991
CCCGGTATTGGTG	0.024	0.001	0.023	25	1069
ACCGTTGTAGGCA	0.025	0.002	0.027	49	1738
ACTATTACAAACG	0.026	0.002	0.027	122	4333
ACTATCGCTAGTG	0.025	0.002	0.026	35	1298

CCTATTGCTGATG	0.024	0.007	0.021	38	1736
GGCGTTACAAGCG	0.06	0.01	0.061	63	971
CCCATCGTAGATG	0.022	0.003	0.017	36	2074
CGCATTACAGATG	0.029	0.004	0.031	53	1651
GCTATCGTAGGTA	0.049	0.012	0.052	58	1053
AGTATTGCATGGA	0.03	0.004	0.034	36	1013
AGTATTGCTGATA	0.023	0.002	0.02	30	1498
CGCATTGTTGGTG	0.025	0.01	0.023	19	808
GCCGTTATAAGTG	0.058	0.006	0.054	81	1420
AGTAGCATAAAGA	0.024	0.007	0.015	33	2222
CCTATTGTTGAGA	0.023	0.005	0.016	29	1752
CGCAGCGTTTGTA	0.029	0.005	0.024	29	1171
CGTAGCACAAATG	0.02	0.007	0.011	16	1430
CGTATCACAAGTG	0.02	0.003	0.015	18	1159
ACTGTCACAGGTG	0.028	0.004	0.025	25	995
CCTGTTGCTAGCG	0.026	0.003	0.028	30	1024
GCCATTATTAGCG	0.051	0.004	0.048	89	1769
AGTAGTACAAGCA	0.021	0.001	0.021	38	1781
CGCAGCACAAAGG	0.024	0.003	0.02	33	1612
CCTGTCGCAAGTA	0.026	0.001	0.027	49	1776
AGCGTCACAGAGG	0.017	0.001	0.016	20	1247
GCCAGCGCATAGG	0.039	0.013	0.028	22	762
GGCATCGCTGGGA	0.079	0.013	0.086	63	673
ACCGTTACTTGCG	0.023	0.005	0.02	37	1818
AGTAGCATTAGGG	0.028	0.002	0.031	22	687
GCTAGCGCATGGG	0.043	0.008	0.044	18	394
ACTGGCGCAAATG	0.026	0.004	0.022	26	1133
CGCAGTACTGATA	0.024	0.004	0.019	37	1927
AGTGGCATAGATG	0.02	0.004	0.015	5	327
CCTGGCGTTGAGG	0.023	0.005	0.021	14	661
AGCGTCACAAGCG	0.022	0.002	0.02	34	1699
AGCGTCGCAGAGA	0.018	0.004	0.013	17	1298
AGCGGTACTTAGA	0.019	0.006	0.024	34	1401
GCCAGCATATATA	0.054	0.004	0.051	154	2868
GGCAGTACTAACG	0.05	0.006	0.048	73	1455
ACTAGTGTATGCG	0.034	0.005	0.036	53	1438
CCCATCATTGAGA	0.027	0.004	0.022	63	2861
CCTGTCATATGTA	0.024	0.002	0.021	40	1862
ACTGGCGCAGATG	0.023	0.001	0.023	20	860
GCTGGCACAGGTA	0.225	0.019	0.234	216	706

ACCATCGTTGGGA	0.021	0.001	0.021	31	1435
AGTAGCGTTGATA	0.025	0.003	0.022	28	1255
CCTGTCGTTTATG	0.031	0.012	0.019	20	1033
ACTATCACTAGGA	0.024	0.004	0.029	65	2167
ACCGGCGCATATA	0.021	0.001	0.02	33	1642
GCTATTACAAGTG	0.061	0.003	0.062	89	1341
ACCAGTACTGGTA	0.021	0.005	0.017	53	2990
CCCGTCACTGAGA	0.027	0.002	0.025	51	1960
GGCATTACTGGGG	0.052	0.011	0.038	27	687
GCTGGTACATACG	0.125	0.01	0.124	125	881
GGCGGTACTTAGA	0.063	0.004	0.061	55	853
GGCATTGCTTATA	0.058	0.012	0.062	88	1333
AGTAGCGCAGATG	0.028	0.002	0.026	22	814
GGCATTATAAAGA	0.058	0.01	0.054	156	2710
CCTGTTGTTGGCA	0.026	0.003	0.023	29	1214
CCCGTTGCAGATG	0.024	0.001	0.023	35	1483
CCTAGCACTGGGA	0.04	0.003	0.037	39	1025
CGTGGTACAAGGG	0.015	0.001	0.014	7	509
GCTGTTACTTAGG	0.063	0.009	0.068	39	536
CGCGGCGTAGACA	0.026	0.005	0.026	38	1439
CCTATTACATGCG	0.025	0.004	0.022	42	1893
GCCAGCACTGACA	0.058	0.005	0.051	117	2169
AGTGGCGCATGGG	0.022	0.008	0.02	7	343
AGTGGTATTGAGA	0.022	0.004	0.019	18	940
CGCGTTACAAATG	0.024	0.004	0.029	60	1988
GGTGGCGCAAAGA	0.151	0.026	0.132	97	636
ACCGTCGCTAGGA	0.025	0.005	0.03	45	1459
CCTGTTGTAAGGG	0.018	0.009	0.012	11	926
CGCAGTGCAGACG	0.026	0.008	0.016	21	1296
GCCGTTGCAAACG	0.049	0.006	0.045	68	1438
CGCGGTGTATGTA	0.022	0.005	0.016	17	1053
AGCGGCACTAATA	0.022	0.004	0.025	56	2202
GGTAGCATTGATA	0.073	0.013	0.075	77	947
AGCATCATAAAGG	0.019	0.002	0.019	55	2837
GGTGGTGTAGAGA	0.107	0.026	0.074	38	474
GGCGGCATTGGTA	0.303	0.06	0.286	275	687
GGCATTGCTTGTG	0.068	0.017	0.05	38	721
ACCGGCGCTGGTG	0.021	0.007	0.011	9	777
AGTATCGCAGATG	0.023	0.002	0.022	25	1093
CCCGGCACAGATG	0.027	0.002	0.025	45	1726

ACCGGTGCAGGCG	0.025	0.004	0.021	26	1203
ACCGTTGCTAACG	0.024	0.003	0.021	46	2186
ACCGGTGTTAAGG	0.031	0.005	0.036	43	1157
CGTAGCGTAGACA	0.021	0	0.021	28	1303
ACCAGTGTTTAGA	0.024	0.004	0.02	36	1802
GCCGTTACTAATG	0.062	0.008	0.051	71	1315
CCCGGTGCATGGA	0.024	0.005	0.017	20	1154
CGCGTTGTAAGGG	0.023	0.009	0.011	9	814
ACTAGTATTTATG	0.027	0.001	0.027	56	1991
AGCGGCATTGAGA	0.021	0.005	0.016	18	1092
GCTAGCACAGACG	0.074	0.01	0.06	69	1074
CCCATTGCAGGCG	0.026	0.004	0.022	41	1865
CCTGGTACAGACG	0.021	0.002	0.019	26	1324
CCTGTCGTAGAGG	0.023	0.006	0.021	19	882
AGTAGTATAGGGG	0.019	0.005	0.012	11	874
CCTAGTATTGATG	0.027	0.004	0.022	36	1614
CGCATCGTAGAGA	0.023	0.003	0.026	44	1625
GCCATCGTATAGA	0.052	0.002	0.05	80	1526
AGTAGCGCTGGCG	0.027	0.01	0.018	14	749
GCTATTGTTAATG	0.057	0.009	0.046	67	1405
CGCGTCACAGATG	0.023	0.003	0.021	30	1376
CGCGGCATTGAGG	0.022	0.007	0.032	23	692
CGCGGTATAAGTG	0.025	0.004	0.031	36	1141
CGCAGTGCTGATA	0.029	0.005	0.027	39	1400
CCCGTTATAAGTA	0.026	0.005	0.022	64	2816
AGTGTTACTGGGG	0.029	0.009	0.019	9	460
GCTGGTACAGGCG	0.096	0.005	0.097	65	604
GGTAGTGTTAACG	0.059	0.012	0.042	33	752
CGTAGCATAAACA	0.025	0.006	0.017	45	2539
GCTGTCATTTAGA	0.052	0.006	0.044	48	1039
ACTATCGCTAGGG	0.026	0.006	0.023	24	1034
GGCGTCGCTAACG	0.075	0.003	0.072	71	911
AGCATCGTAAATG	0.02	0.001	0.021	57	2667
GCCAGCGTTGAGG	0.062	0.005	0.056	42	704
ACCGGCACTGATA	0.024	0.002	0.024	69	2792
ACCATCGTAAGTA	0.022	0.005	0.016	46	2862
ACTGGTGTAAACA	0.021	0.002	0.025	59	2337
ACCAGCGTATATG	0.027	0.002	0.024	44	1805
CGCATCGCTGGCG	0.022	0.005	0.027	41	1482
CGTGGTGTATACG	0.027	0.008	0.017	12	680

CGTGGCACAAACA	0.03	0.001	0.03	52	1685
AGCAGTACTTAGA	0.018	0.005	0.01	21	2014
ACCGGCACTTGGA	0.026	0.004	0.031	49	1522
AGCGGTATTAAGA	0.02	0.003	0.022	46	2036
GGCAGCATATAGG	0.06	0.01	0.069	76	1018
ACCAGTGTTGGTG	0.026	0.003	0.021	24	1109
GCTGTTGCAGAGA	0.052	0.006	0.056	49	823
AGCAGCGCTAATG	0.024	0.004	0.029	47	1582
GCCGTTACTTAGG	0.059	0.01	0.057	48	801
AGCAGTGCATACA	0.023	0.002	0.025	70	2683
CCTGGTACAAAGG	0.024	0.006	0.02	26	1305
ACCAGTATTAAGA	0.021	0.002	0.018	72	3954
CCCAGCATAGGGG	0.036	0.004	0.042	49	1129
ACTATTGCTAGCA	0.022	0.001	0.022	54	2424
CCCGGTGCATACG	0.022	0.005	0.017	26	1462
AGCAGTGCAGACG	0.022	0.003	0.024	43	1778
GCCAGTATTGGCG	0.057	0.008	0.048	67	1337
CGTATCACTTAGG	0.032	0.008	0.024	20	829
CCTAGTGTTGGCG	0.024	0.003	0.026	33	1237
GGCATCGCATGGA	0.08	0.014	0.066	64	900
CGCGTCGTATACG	0.029	0.001	0.03	42	1362
CGCATCGCATGGA	0.025	0.006	0.028	35	1197
AGCGTCGTTTGGA	0.028	0.005	0.029	26	878
CCCGGCATAGACA	0.026	0.003	0.028	90	3155
AGCATTGCTAGTG	0.018	0.001	0.018	25	1379
CCTATCGTTTGGG	0.029	0.005	0.023	20	866
GCCGGCACAAATG	0.212	0.021	0.206	345	1332
GGCAGCGTTTAGG	0.071	0.016	0.058	39	638
GCTAGCACTGATG	0.069	0.01	0.056	57	957
CGTATCACAAGGG	0.035	0.005	0.028	24	844
CCTAGTATTAGCG	0.026	0.004	0.022	33	1447
ACTAGCGTAGGTA	0.024	0.005	0.017	22	1287
CCTATCGTTTGCA	0.028	0.005	0.034	62	1754
GCTGGTGCTTGCA	0.122	0.017	0.102	66	582
CGTATCGTTTGCA	0.025	0.003	0.021	24	1130
CGCGTTATTAATG	0.026	0.003	0.029	46	1558
CCTGGTGCATGCG	0.025	0.007	0.02	18	901
GCTAGCGCTTAGA	0.052	0.001	0.052	40	730
AGTAGCATTAACG	0.024	0.007	0.015	25	1643
AGCGTTGCAAACA	0.02	0.003	0.018	52	2888

GGTGTCATAAGCA	0.093	0.018	0.07	68	898
ACCATCGCTAGGG	0.019	0.002	0.017	22	1296
GGTGGCGCTTGTA	0.155	0.027	0.142	61	370
CCTATTGTTGAGG	0.023	0.007	0.016	21	1297
CCCGGTGTATAGA	0.031	0.01	0.027	40	1455
GGCGGCACAAAGG	0.285	0.04	0.259	290	830
CGCAGCGTAAGCG	0.029	0.006	0.024	31	1272
CGTGTTACTTGTG	0.024	0.007	0.019	12	607
GGTGTCACTGAGA	0.08	0.014	0.071	48	632
CGCGTCGTAAGGA	0.024	0.003	0.025	30	1173
CCCGGTGCTGGTG	0.027	0.008	0.034	24	690
CCCGGCATTAATG	0.026	0.002	0.028	55	1885
ACCGTCATTTACA	0.025	0.002	0.023	80	3414
AGCGGTATTAGTA	0.023	0.002	0.021	38	1765
AGCAGCATAGATG	0.02	0.002	0.02	42	2070
ACTGTCGTAAATA	0.025	0.004	0.029	72	2415
GCTATTATTAAGG	0.063	0.007	0.061	103	1598
GGTGGTGTAAGTA	0.098	0.013	0.092	67	665
CCCGTCATAAGCG	0.024	0.001	0.023	50	2131
GCTGTCACTTATG	0.061	0.015	0.053	46	825
GGCGTCATAAGCG	0.065	0.01	0.056	70	1191
GCCGTCGTTAATA	0.053	0.007	0.045	68	1438
ACCGTTGTTTATG	0.027	0.01	0.023	33	1432
AGCAGTGCTGACA	0.018	0.002	0.015	31	2065
CGTATTGCTGAGG	0.018	0.005	0.011	8	695
AGCAGCGTTTGGG	0.022	0.003	0.025	20	783
CGCGTCGCTTGGA	0.023	0.006	0.013	10	732
GGTGTTACTTGTG	0.08	0.022	0.057	27	449
ACCGGCGTTAGCA	0.023	0.001	0.022	31	1405
CCTGGCGCTAGCG	0.027	0.004	0.033	29	861
GCTGTTGCTTGGA	0.05	0.007	0.052	24	435
CGCATCGTAGAGG	0.028	0.001	0.028	36	1243
AGCATTGCTTATG	0.024	0.003	0.02	35	1691
AGTGTTGCTGGGA	0.019	0.001	0.018	11	603
AGTAGTGTAGGCA	0.021	0	0.02	23	1123
ACTAGCGTATGGA	0.026	0.008	0.031	35	1106
CGCAGCGTTAATA	0.027	0.005	0.027	58	2066
CGCGTTGCTAGCG	0.018	0.007	0.025	25	982
CGTAGTATTAATA	0.023	0.001	0.022	42	1903
CGCGTTATAAAGA	0.027	0.002	0.03	71	2322

CGCATTACTTGCA	0.03	0.003	0.033	64	1847
ACCAGCGTTTACA	0.022	0.003	0.019	50	2523
GGTAGCACATGCA	0.067	0.012	0.071	71	923
ACCGTCGCTAACG	0.024	0	0.024	52	2141
CGCAGCGTAGGTA	0.023	0.009	0.034	41	1154
ACTAGCGCTTGCG	0.027	0.004	0.025	27	1036
ACCGGCATTTAGA	0.022	0.001	0.02	39	1865
GGTAGCGTATAGG	0.087	0.017	0.088	45	464
CGTGGCATAAATG	0.03	0.005	0.025	22	867
AGCGTCACTTATG	0.023	0.002	0.026	36	1371
CGTGGTACAGAGA	0.023	0.004	0.017	15	849
CGCAGTATATGTG	0.018	0.003	0.022	32	1434
AGTATCGTAAGGG	0.021	0.005	0.025	24	933
GGTGGTGCAAGTA	0.073	0.023	0.048	33	649
ACTAGTATATAGA	0.025	0.004	0.025	72	2789
ACTATTGTAAGGG	0.031	0.003	0.03	47	1502
GGTATTATATAGG	0.106	0.02	0.092	93	917
GCCATTGTTAGCA	0.049	0.004	0.047	83	1686
AGCAGTGTATGCA	0.021	0.003	0.025	49	1932
GGCAGTATAAGTA	0.052	0.001	0.05	114	2151
CGCATTGTAGACG	0.023	0.003	0.023	42	1773
CCCATTGTAGGTG	0.025	0.007	0.016	25	1527
GCTATTGCTAGCA	0.051	0.005	0.05	64	1209
AGTATCGTATGCG	0.025	0.001	0.024	24	975
AGTAGTGTAGAGA	0.026	0.004	0.03	37	1198
CCCAGCGTAAAGG	0.024	0.006	0.017	32	1852
AGCGTTGCTAGGA	0.018	0.006	0.012	12	974
GCCGGCGCTGATA	0.499	0.04	0.498	663	669
AGTGGCATTAAGA	0.018	0.003	0.014	11	751
ACCGGTACATGTA	0.022	0	0.022	61	2722
ACCGGCGCAAACA	0.021	0.001	0.02	48	2342
AGTGTCATAAACG	0.022	0.003	0.022	34	1538
ACCGGCGTTGGTA	0.027	0.006	0.021	20	924
ACCGTCATAGGCG	0.026	0.005	0.021	35	1613
GCCATTGTAGAGA	0.05	0.009	0.058	95	1543
CGTAGTACTGGCA	0.021	0.006	0.016	14	872
CGTAGTGTTAGCA	0.022	0.002	0.023	20	848
AGTAGCACAAACG	0.022	0.004	0.02	33	1639
ACCGTCGTTGAGG	0.029	0.009	0.017	15	871
AGTATCATATAGA	0.021	0.004	0.016	32	1986

AGCATTGCAGAGG	0.025	0.004	0.023	32	1377
ACCAGCGTTAGCA	0.021	0.004	0.021	43	2009
CGTGTCACAGGTG	0.022	0.003	0.026	17	638
GGTGGTATAGGTG	0.274	0.035	0.273	134	356
CCTATTGCAAGGA	0.022	0.003	0.017	35	1968
GCCGGTATTAAGG	0.171	0.008	0.174	200	950
GCCAGCGTATATG	0.062	0.003	0.06	71	1121
CCTGTCACTGGTA	0.022	0	0.022	28	1246
ACCGGTGTTTGTG	0.026	0.003	0.024	23	939
ACTGTCGTTAGGA	0.025	0.007	0.016	15	929
ACTGTCGCTTATG	0.025	0.004	0.031	36	1135
GCTGTCGTAAGCA	0.059	0.007	0.063	69	1029
AGTGTCGTAAACG	0.027	0.006	0.021	25	1166
AGTGGCACTGGGA	0.016	0.004	0.011	6	521
CGCGTTGTAGGTG	0.027	0.011	0.03	22	717
GGCGTCGTAAGGG	0.075	0.009	0.062	40	609
ACCATCACATGTA	0.022	0.002	0.02	87	4250
ACCAGTATTTACG	0.023	0	0.023	68	2867
GCTAGTGCTGACG	0.058	0.001	0.057	52	858
CCCAGCACTAAGG	0.026	0.002	0.029	57	1890
CCTGTCACATGTA	0.025	0.001	0.023	41	1719
GCCAGCATTGAGA	0.052	0.011	0.037	67	1735
AGCGGTATATGGG	0.021	0.005	0.025	19	744
GCCATCGTTGGCG	0.044	0.006	0.049	69	1327
AGCGGTGTTTATA	0.026	0.002	0.027	42	1515
CCCAGTACTAGGG	0.024	0.002	0.02	28	1349
GGTATCGCAGGCG	0.107	0.017	0.083	61	678
ACCGGTACATGCA	0.017	0.001	0.018	57	3131
AGCAGCATTAAGA	0.021	0.004	0.019	52	2689
AGTAGCGTTGGTG	0.023	0.006	0.017	9	519
GCTAGCGTAAGGA	0.062	0.009	0.054	47	823
CCCGGCACTTATA	0.02	0.005	0.014	32	2296
AGTATCGCAGGGA	0.026	0.008	0.026	20	753
GCCGTTACAGAGA	0.053	0.01	0.045	65	1377
CGTGTTGCATATG	0.023	0.004	0.018	15	799
CCCATTGCATAGA	0.026	0.002	0.025	62	2432
AGCATCACAGAGG	0.023	0.006	0.015	29	1850
CGCGTTGCAAGGG	0.026	0.006	0.025	20	796
GGTATCGTTGATA	0.136	0.021	0.123	140	999
GCTGGCACTGATA	0.213	0.029	0.205	236	913

GGCGGCGCAGACA	0.096	0.018	0.08	99	1131
GGCAGTGCTGGTG	0.053	0.008	0.041	24	557
AGCGTTATTTAGA	0.019	0.003	0.019	34	1722
GGCGGTGTAAGTA	0.069	0.009	0.072	76	976
ACCGGTATAGGCG	0.024	0.001	0.022	32	1415
CCTGTCACTGAGG	0.025	0.002	0.022	21	927
CCTGTCGTTAACG	0.025	0.003	0.028	42	1473
AGCGTCGTTGGCA	0.024	0.002	0.025	33	1262
CCTATCACAAGTG	0.027	0.004	0.024	53	2150
CGTGTTACATATA	0.021	0.005	0.015	25	1653
GCCATTATAGAGG	0.061	0.012	0.057	95	1576
ACCATCGCTGGGG	0.025	0.008	0.019	18	919
GCCAGCGTTGATG	0.054	0.01	0.058	60	979
GGCGGCACTGAGA	0.131	0.027	0.116	105	801
GCTGTTACATGCG	0.058	0.019	0.031	25	779
CCTAGCATTGGTG	0.019	0.005	0.019	19	959
ACCATCGTTTGGA	0.02	0.004	0.015	22	1479
CGCGTTACAGAGG	0.024	0.001	0.025	26	1003
CGCGTCGCAAATG	0.026	0.002	0.023	33	1410
GCTAGTATATAGG	0.064	0.009	0.052	63	1141
CCCGGTGCTAAGA	0.022	0.002	0.021	36	1663
CGCGTTGCAAAGG	0.028	0.002	0.03	34	1088
CCCGTTGTATGCA	0.023	0.005	0.018	36	1973
CCTAGCACATGTG	0.029	0.008	0.022	29	1279
AGCATTACAAGGA	0.023	0.003	0.023	60	2564
GGCGGCGTAGACA	0.222	0.038	0.212	232	861
AGCAGCGTAAGGA	0.02	0.003	0.016	26	1597
CCCGTTGTTGAGA	0.022	0.005	0.019	27	1360
CCCATTATAGGGA	0.039	0.006	0.045	89	1887
GCTGGTGCAGAGA	0.112	0.003	0.108	70	577
GCTGTCATAAGCA	0.064	0.001	0.066	104	1476
ACTGTCGTTGGTA	0.022	0.006	0.014	13	888
ACTGTTGCAAGTG	0.02	0.002	0.018	22	1218
CGTGTCATAAGTG	0.02	0.006	0.024	25	1023
ACCGGTATAGACA	0.026	0.002	0.024	77	3181
GCTGGCGTAAACA	0.881	0.026	0.873	2376	345
CCCGTCGCTTGTA	0.019	0.001	0.02	31	1543
AGCGTTACTTAGG	0.024	0.003	0.025	26	1021
GCTATCGCAAATG	0.05	0.006	0.041	51	1193
AGTATCGCTTAGA	0.025	0.004	0.019	20	1030

GGCATCATAGGGG	0.052	0.006	0.048	54	1062
CCCATCACAAGCG	0.023	0.003	0.024	67	2768
GCCAGTGTAAAGG	0.046	0.004	0.041	57	1333
CCTGGCACATGCG	0.027	0.004	0.022	24	1065
ACTGGCGCAGGGA	0.029	0.001	0.03	15	485
CGCGGTACTAGTG	0.023	0.004	0.016	14	838
ACCAGTGTAGAGG	0.026	0.003	0.024	36	1460
AGCATTACTAAGG	0.018	0.002	0.015	32	2083
ACTAGTATTGATA	0.022	0.003	0.024	71	2898
GGTAGTATAAACG	0.07	0.013	0.06	84	1317
GGTGGCATTAGCG	0.706	0.057	0.684	622	287
AGCAGCGTTTATA	0.022	0.004	0.025	49	1930
AGTAGCGTTTGCA	0.027	0.005	0.021	18	848
CCTAGTGCTGGTG	0.027	0.006	0.034	30	861
CCCGTCATTAGTA	0.024	0.006	0.015	36	2350
AGCATCACATGCG	0.024	0.002	0.023	47	2001
GCTGGTGCAGACG	0.27	0.019	0.283	215	545
AGTAGTATTTGGG	0.026	0.009	0.038	28	704
ACTGGCGCTGGCA	0.023	0.01	0.011	7	631
CGCAGTGTAGGGG	0.043	0.007	0.039	24	598
GCTATCATTGGCA	0.074	0.009	0.079	108	1256
CCTGTCGTAGGCA	0.02	0.004	0.019	26	1360
CCTGGCATTGACG	0.028	0.005	0.032	40	1218
AGCGTCGCTGGCA	0.023	0.001	0.022	27	1183
CGTGGTGCAAGTA	0.019	0.004	0.022	21	950
GGTAGTATTTGGG	0.059	0.003	0.06	26	410
GCCGTCGTAGACA	0.052	0.006	0.043	65	1440
GCCGTTATATGTA	0.059	0.003	0.062	122	1853
AGCAGTACAAATG	0.021	0.003	0.018	56	3018
AGTGTTATTTGGA	0.015	0.006	0.014	14	1014
GCTGTCGCAGGCG	0.059	0.026	0.05	25	480
GCTGTCATTAACA	0.062	0.004	0.056	115	1932
GGCGGCACTTGGG	0.244	0.028	0.226	119	408
CCTGGCATTGGTA	0.023	0.01	0.024	26	1044
GCTAGCGTAAAGA	0.056	0.009	0.055	73	1260
CGCGTTATAGGTA	0.022	0.003	0.023	36	1511
CGCAGTGCAGGTA	0.024	0.001	0.025	28	1093
GGTATCATAGAGA	0.126	0.029	0.093	108	1053
GCCATTGCTTGTA	0.043	0.011	0.049	56	1091
CGTGTCATATATG	0.03	0.008	0.019	22	1128

ACTGGCGCTTACG	0.032	0.002	0.03	26	854
CGTGTCGTAGGTA	0.022	0.008	0.012	11	882
GCTGGTATATAGG	0.425	0.033	0.418	356	496
GGCAGTGCTAATG	0.053	0.005	0.047	53	1070
AGCGGTGTAAAGG	0.026	0.003	0.028	33	1141
CGTGTTACAAGCG	0.022	0.003	0.017	17	972
CCCGGTACTGATG	0.027	0.004	0.032	53	1628
GGTATTACTTGTA	0.09	0.018	0.072	76	986
AGTATTGCTTGCG	0.025	0.009	0.018	16	872
ACTGTTACTTACG	0.026	0.004	0.028	46	1578
ACTATTACTTGGG	0.026	0.002	0.028	41	1402
CCTAGTGTAGGGA	0.032	0.006	0.032	38	1162
CCTGGTACAGAGA	0.027	0.002	0.024	34	1355
GGTAGCATATAGA	0.062	0.007	0.069	68	918
AGTATTACAAGCG	0.019	0.002	0.016	26	1570
ACTAGCACAGGTG	0.024	0.002	0.025	33	1266
CGCATCATTGGCG	0.026	0.003	0.029	43	1437
GGCGGCATTTACG	0.56	0.055	0.523	716	653
GCCGTCACAAACA	0.054	0.007	0.051	162	2986
GCCATTGTATGGG	0.053	0.009	0.042	39	884
GCTAGTACATAGA	0.059	0.005	0.057	78	1296
CCCATTGTTTACG	0.026	0.001	0.026	54	2029
GGTGTCATTAGGG	0.082	0.02	0.058	21	343
CCCATTACTAGGA	0.028	0.004	0.031	72	2229
AGCAGCATTTAGA	0.021	0.003	0.025	56	2162
CGCATCGCTAAGG	0.028	0.004	0.024	30	1201
CGCATTGTAGAGG	0.027	0.003	0.029	34	1144
CCTAGTGTAGATA	0.02	0.002	0.02	44	2172
ACCGGTATATGCG	0.025	0.006	0.023	36	1554
CGCATCGTAAGCA	0.023	0.003	0.026	63	2401
AGTAGCACAGGCG	0.03	0.002	0.027	25	892
CCTATCATTGGCG	0.026	0.004	0.023	37	1575
ACCATCACTGAGG	0.027	0.001	0.029	62	2077
AGCGGCGTATACG	0.019	0.003	0.021	19	898
GCCAGCACAAGCA	0.054	0.005	0.053	137	2437
CCTGTTGTTGGTA	0.019	0.003	0.018	20	1086
CGTGGTATAAATA	0.025	0.005	0.021	41	1875
GCCGTCATAGGGG	0.092	0.014	0.102	70	617
CGTGTCGTAGGCG	0.018	0.002	0.018	11	613
GGCATCGTTGGTG	0.09	0.01	0.078	55	649

ACCGTTGTATGCG	0.027	0.005	0.02	29	1389
AGTGTTATAGACA	0.024	0.003	0.02	44	2109
GGCGGTGCATAGA	0.049	0.004	0.054	44	765
CCCAGTACTTAGA	0.024	0.005	0.023	54	2300
AGTATTACAGATG	0.023	0	0.023	40	1725
AGCATCGCTAGTG	0.028	0.001	0.028	40	1400
ACTGTCATTAGTG	0.029	0.003	0.024	33	1344
CCCGTCGCTTGGA	0.02	0.001	0.021	25	1183
AGCGTCATTAAGA	0.021	0.006	0.02	46	2225
GCCAGTACAAAGG	0.056	0.002	0.055	91	1577
CGCAGCGCTAATG	0.026	0.003	0.022	31	1366
GCCATTGCTAATG	0.049	0.001	0.049	71	1376
GGTGTCACTAGGA	0.076	0.009	0.065	38	547
CCTGGTACTTGGG	0.026	0.004	0.022	13	585
CCTATCGCTAGTG	0.024	0.003	0.028	35	1228
CCTAGCGTTGGCA	0.026	0.002	0.029	39	1295
CGCGGTATATGGA	0.026	0.007	0.017	20	1129
CGTAGTACAGAGG	0.031	0.009	0.019	14	727
AGTATTGTATGCG	0.025	0	0.025	29	1127
GGCAGTATTGACA	0.05	0.004	0.046	96	2006
GGTAGCGCAAGCG	0.072	0.003	0.07	44	585
GGTGTTGTATGCG	0.077	0.004	0.081	41	465
GCTATCGCTAGCG	0.051	0.01	0.05	42	805
AGTGTTGTAGGGA	0.017	0.007	0.022	15	670
ACTGGTATAAGCG	0.031	0.004	0.027	37	1341
CCCATCGTAAAGA	0.022	0.004	0.016	51	3195
CGCGTTGTTTAGA	0.019	0.002	0.02	20	991
GGCGTCACTGGCG	0.05	0.017	0.035	26	708
GCCAGTGCTAACG	0.044	0.005	0.042	57	1316
CGCGTTACTTGGG	0.024	0.001	0.024	14	573
CGCGGCATATGTA	0.018	0.002	0.021	26	1230
ACCGGTGTATATG	0.029	0.003	0.033	48	1408
CCCAGTGCTAGCG	0.023	0.005	0.016	25	1529
AGTGTTGTATAGA	0.021	0.002	0.02	24	1165
GGTGGTACATATA	0.077	0.015	0.064	66	970
GGTATTATAGGTA	0.088	0.013	0.07	88	1171
CGTGGCATAGGTA	0.022	0.003	0.021	16	743
AGTATTGCTTACG	0.031	0.002	0.03	36	1145
GGCGGCGCAAACG	0.166	0.041	0.142	169	1020
ACCGGCGCAAAGG	0.026	0.002	0.027	36	1292

GCCGGCATAGGTG	0.623	0.042	0.619	651	401
GCTGGTATTTATG	0.449	0.045	0.432	417	549
GCTGGTACATATG	0.105	0.011	0.1	91	817
AGCAGTACAAAGA	0.023	0.002	0.022	72	3237
CCCAGCGCTAAGG	0.028	0.002	0.025	38	1495
GGCGTTATTAATA	0.058	0.001	0.058	119	1938
CGCGTCGCTAAGG	0.021	0.002	0.023	20	861
GGCAGCATTTGGG	0.05	0.011	0.034	25	704
GCCGTTACTGGTA	0.06	0.007	0.053	57	1015
CCTATCACATATG	0.031	0.002	0.028	73	2500
AGTGGCGTTAACA	0.025	0.004	0.022	21	947
GGCGGCGTTAAGA	0.217	0.046	0.192	191	803
GCCATCGTTAATA	0.053	0.003	0.054	128	2262
CCCAGTGCTGGTA	0.024	0.004	0.03	46	1500
CGCGGTATTAGCG	0.022	0.006	0.015	15	1011
GCTAGCGTATGTG	0.086	0.017	0.109	72	587
CCCATCATAAGGG	0.038	0.002	0.038	73	1869
ACCAGTGCTAGGG	0.024	0.002	0.023	30	1261
GCTGTTACTGACA	0.06	0.004	0.064	86	1250
GGCGGTGTTGAGG	0.093	0.015	0.098	49	449
CGTGTTGTTGAGA	0.031	0.005	0.025	18	704
GCCGGTACAGACG	0.074	0.007	0.065	82	1188
ACTGGTATAGGCA	0.025	0.003	0.023	36	1542
CCCGTCATTTACG	0.029	0.004	0.024	48	1962
AGCAGCGCATATA	0.025	0.006	0.024	54	2164
GGCGTTGTTTGCA	0.071	0.004	0.068	55	753
AGCATCACTGGCA	0.019	0.003	0.017	38	2245
GGTAGCATTGACA	0.064	0.004	0.069	79	1072
GGTATTGTTTGCG	0.106	0.01	0.093	57	557
AGTATCATTAACG	0.02	0.004	0.018	37	1966
CCCAGTGCTTGCG	0.022	0.004	0.027	36	1299
ACTATCACAGAGG	0.027	0.003	0.023	44	1853
CCTATCATAGGGG	0.047	0.009	0.048	56	1107
GCCATCATATACA	0.054	0.002	0.051	205	3791
ACCATTATTAATG	0.025	0.002	0.023	100	4194
GCTATCGCATGTA	0.049	0.004	0.044	50	1090
CCTATCGTATACG	0.028	0.001	0.027	57	2075
CCTGTTACTTATG	0.027	0.003	0.028	35	1200
GCCGTCGCAAGCG	0.053	0.011	0.038	31	779
GCCAGTGCTTGCA	0.044	0.007	0.046	56	1159

CGTATTATAGGTG	0.026	0.005	0.034	38	1091
GCCGTTACTGATA	0.049	0.005	0.042	71	1618
ACCATCGCAAACG	0.023	0.003	0.019	69	3526
AGCGTTACAGGGG	0.022	0.004	0.017	13	761
AGTATTATAAAGG	0.025	0.004	0.019	44	2228
AGCGGTGTTAGTA	0.029	0.008	0.021	27	1258
ACCAGCGCTTAGG	0.023	0.005	0.019	24	1209
CCTGTCGTAGGTA	0.023	0.005	0.017	22	1236
GCTGTCGCAAATG	0.061	0.01	0.068	58	799
GGTAGCGTTAGCA	0.072	0.018	0.082	52	581
ACTATTGCAGGGG	0.018	0.004	0.021	23	1082
CGTATTGCTGATA	0.02	0.005	0.018	25	1332
CCTGTTATTTACA	0.025	0.004	0.023	51	2150
ACTGTTGCTTGCG	0.028	0.007	0.019	19	981
ACTATCATTGGCA	0.029	0.003	0.025	56	2202
AGTATTACTGGGA	0.016	0.006	0.012	13	1053
ACCATTACTAGTA	0.025	0.002	0.025	104	3983
CGTGGTATATGCG	0.034	0.006	0.029	18	607
CGTATCGTAGGGG	0.027	0.002	0.024	13	538
AGCAGTGCAAGGG	0.016	0.005	0.015	17	1147
CGTATCGCTAGTA	0.025	0.006	0.027	31	1109
AGCGTTGCAGAGG	0.025	0.006	0.023	23	969
CGCATCGTAGGCG	0.021	0.007	0.015	19	1285
ACTGGCATATACG	0.019	0.004	0.025	36	1404
GCTGGTACTAGGG	0.11	0.02	0.089	35	359
GGTGTCGCTAGCG	0.114	0.02	0.088	40	413
AGCAGCATATGGA	0.019	0.003	0.015	29	1905
CGCGGCGCATAGG	0.021	0.005	0.023	17	711
GGTGTCGTTGACA	0.082	0.008	0.072	55	712
CGCGTTATAAGCA	0.025	0.004	0.02	46	2246
GCTATCACTGGTG	0.06	0.006	0.051	41	762
CCTAGCATTTGGA	0.027	0.006	0.019	23	1187
GGTGTCGTTTGTA	0.096	0.012	0.086	47	497
AGCATTGCTAACG	0.021	0.001	0.021	50	2343
CCTGTTATTAACA	0.025	0.004	0.02	58	2851
CCCGTCATTGAGG	0.022	0.002	0.022	29	1270
CGTGGCATTTACA	0.027	0.001	0.026	30	1122
CCCAGCGCTGATG	0.019	0.002	0.016	26	1581
CCTGTCGCAGGGA	0.016	0.004	0.014	13	909
CCCGGTATAAAGA	0.027	0.005	0.025	72	2785

CGCGGCGTTTACA	0.023	0	0.023	29	1215
ACTGGCGCTTGTA	0.031	0.006	0.023	13	549
GGTAGCGCTGACA	0.075	0.006	0.079	64	742
AGTATCATTGAGG	0.023	0.008	0.015	16	1076
ACTGGCATTTGTA	0.027	0.003	0.023	29	1233
CCCGGCGCTAATG	0.025	0	0.025	37	1433
GGTATTATTTGTG	0.118	0.021	0.102	83	734
CCCGGTGTATATA	0.027	0.003	0.031	61	1889
AGTGGCATAAAGG	0.027	0.008	0.016	5	312
AGCGGTACTTGTA	0.018	0.002	0.016	22	1330
CGTGTCGCTAATA	0.021	0.006	0.013	16	1203
GGCATCGTTTGTA	0.104	0.023	0.084	102	1112
ACCATCGTATAGA	0.025	0.003	0.023	57	2460
GGCAGCACTGACA	0.046	0.004	0.049	86	1679
GGTGTCACTGGTA	0.075	0.007	0.067	43	599
CCCAGTATAAGCA	0.023	0.001	0.024	93	3744
CCCATTACAAACA	0.026	0	0.026	174	6407
AGCGGCACTGATG	0.02	0.002	0.02	19	934
CGCAGCATAGGCG	0.037	0.005	0.037	42	1106
GGCATCGCTTAGA	0.086	0.008	0.097	101	943
CGCAGTGTAGGGA	0.03	0.005	0.038	34	872
CCTATCATTTATG	0.028	0.004	0.023	54	2301
CGCGGCGCAAGGA	0.024	0.005	0.024	22	907
GGCATCATAGGGA	0.054	0.014	0.05	82	1544
GGCGGCACATGTA	0.138	0.006	0.13	156	1043
AGTGGTATAGGTA	0.025	0.005	0.021	21	956
AGTATTGTATGTA	0.025	0.003	0.023	40	1689
ACCGTTATATAGG	0.019	0.003	0.016	30	1855
ACCAGCGTTGGCA	0.026	0.005	0.024	40	1656
AGCAGTGCATGGA	0.018	0.005	0.012	17	1355
AGCATCGCATGGA	0.022	0.002	0.023	32	1359
CCCATTACTGGCG	0.029	0.002	0.032	59	1803
CGCAGCACTAGCA	0.023	0.003	0.025	49	1883
ACCAGCGTATAGG	0.022	0.004	0.016	21	1254
CGTAGCACTGGTG	0.021	0.005	0.017	11	645
GGTAGTACAAAGG	0.058	0.008	0.056	51	864
CCCGGTGTAAGCA	0.025	0.001	0.024	47	1912
GGCGTTGTATACA	0.067	0.009	0.055	86	1468
GCTATTGTATGGA	0.057	0.004	0.052	58	1048
GCTGGTGTTGGCA	0.389	0.063	0.359	274	489

GGTGGCATTGACA	0.468	0.068	0.406	488	713
CCTATTGCTGACG	0.027	0.005	0.021	37	1739
GGCAGCACAGACA	0.046	0.003	0.046	90	1856
GGTGTCATAAATA	0.091	0.004	0.086	144	1522
GGCGTTGCAGGGA	0.068	0.009	0.06	34	532
GCCAGCATAAATG	0.048	0.004	0.054	152	2657
GGCATTGTAAGTA	0.074	0.012	0.073	127	1617
CCCAGCGTTAGGA	0.022	0.001	0.023	35	1470
GCTAGTACAAGGA	0.068	0.006	0.066	78	1106
ACCATCGTTAGTG	0.029	0.001	0.028	46	1623
GGTAGCGCAAATG	0.074	0.011	0.081	69	786
AGTATCGCTAGTA	0.023	0.004	0.019	24	1211
GCTAGTGTATACG	0.065	0.008	0.066	89	1265
CGCATCATATGTA	0.024	0.001	0.024	62	2533
CGTATCACATGTA	0.028	0.002	0.029	43	1431
GGCGGTGTTTGCA	0.073	0.007	0.069	48	646
GGTATTGCAAATG	0.1	0.005	0.1	117	1057
CGTATCGCTTACG	0.031	0.006	0.027	28	1009
ACCATTGCTAGCA	0.023	0.002	0.023	71	2979
CGTATTGTATGTG	0.027	0.005	0.03	28	899
GGCGTCACAGGTG	0.064	0.022	0.049	42	813
CCTGGCACAAGTG	0.026	0.003	0.022	25	1110
CCCATTGTTAGGA	0.024	0.003	0.019	34	1727
GCTATCGTATGCA	0.06	0.004	0.064	88	1277
GGTATCATAGGTG	0.122	0.011	0.115	69	531
CGTAGTGTTGACG	0.022	0.004	0.024	17	683
ACTATCGCTGGCG	0.027	0.001	0.027	34	1249
CGTAGTGCAGACG	0.021	0.007	0.015	11	747
CGTATTATTAATA	0.023	0.005	0.017	54	3069
AGCGTTATAAATG	0.021	0.004	0.016	43	2599
CCCATCACAGAGA	0.021	0.003	0.021	62	2959
GGCGGCGTAGAGA	0.208	0.033	0.195	164	678
CCTGTCGCTGGGG	0.031	0.006	0.025	13	499
AGTGTCGTTGGGG	0.029	0.014	0.037	15	389
AGCGGTATAGAGA	0.019	0.002	0.022	38	1679
AGTGTCACATAGG	0.022	0.007	0.017	14	793
GGTGGTGCAAATA	0.077	0.016	0.06	62	970
ACTAGTGCAAAGA	0.024	0.004	0.022	68	2986
GCCGGCATATAGA	0.391	0.019	0.379	621	1016
AGTATCGTAGGTA	0.021	0.004	0.026	33	1250

AGTATTACAAAGG	0.028	0.005	0.027	50	1810
ACTGTTACTTATG	0.024	0.001	0.026	44	1675
AGCAGCATAGGCA	0.02	0.004	0.017	41	2381
GGTGGTACTGGGG	0.11	0.024	0.125	34	239
ACTAGTGCTTGTG	0.026	0.006	0.02	24	1196
ACCATCGCATATG	0.025	0.005	0.021	53	2417
GCCGGTGTAGGGG	0.299	0.025	0.306	169	384
ACCGGCGCATGCA	0.023	0.003	0.025	36	1385
ACCGTTATTGAGG	0.028	0.005	0.024	34	1409
CCCATTGTAAGCA	0.021	0.004	0.015	46	3044
GCTATCATATACG	0.072	0.013	0.058	108	1762
GCTGGCGCTTACA	0.608	0.045	0.607	845	547
ACTGGTATTAGCA	0.025	0	0.026	46	1741
CGTATCGTTAGGG	0.025	0.003	0.027	17	615
AGTAGTGCTTGGA	0.019	0.003	0.023	15	650
ACTATCGTAGGGG	0.037	0.004	0.041	36	843
AGTGTTATATGCA	0.021	0.001	0.02	31	1538
ACCAGTACTAGTG	0.021	0.002	0.019	47	2463
CGCAGTGTTAGCG	0.023	0.002	0.024	25	996
ACCGGCATATGGG	0.023	0.006	0.016	16	973
CCCATCGCAGGTA	0.023	0.004	0.022	48	2151
ACTGGCGTAGACG	0.03	0.021	0.013	7	548
CCTGGTGTTGGGA	0.024	0.002	0.021	14	662
GGCAGTACATAGG	0.048	0.001	0.048	50	985
ACTAGTGTTTGGG	0.036	0.012	0.026	21	772
CCTGGTGTTTACG	0.025	0.002	0.022	24	1065
GGTGGCGCTTGGG	0.296	0.018	0.302	73	169
ACCAGTACTGGGG	0.029	0.004	0.032	34	1016
GCTATTGTAGACA	0.057	0.008	0.048	96	1889
AGCATTGTTGGTG	0.021	0.002	0.023	28	1175
AGCAGTGCTTACA	0.023	0.005	0.022	43	1957
CCCGTTGCATATA	0.02	0.003	0.023	54	2281
CCTGGCATAAGGA	0.028	0.001	0.027	40	1417
ACTGTTGTTTAGA	0.019	0.004	0.017	22	1271
AGCAGCGCTGGTG	0.02	0.009	0.009	8	887
AGTGTCACTTACA	0.025	0.006	0.02	28	1381
AGCATTATTTGCG	0.026	0.001	0.028	52	1806
GGTGGCACTTGGG	0.401	0.054	0.389	151	237
AGTGTTATATGGA	0.025	0.007	0.014	18	1237
CCCGTTGTTAAGA	0.027	0.004	0.026	50	1879

GCCGTTGTAGATA	0.043	0.003	0.047	77	1552
GCCATCATTTGTA	0.053	0.008	0.042	86	1963
ACCATTACTTGTA	0.02	0.001	0.021	77	3598
CCTAGCACTAACG	0.027	0.005	0.032	62	1897
AGTATCATAAGCG	0.029	0.002	0.027	40	1436
CCCAGCGTTAACA	0.025	0.002	0.028	95	3312
AGTATTGCTAAGA	0.023	0.007	0.015	24	1541
ACTGGTGTAGGTA	0.022	0.002	0.02	21	1052
CGCATCGCTTACA	0.021	0.001	0.019	39	1988
GCCAGTGTTGGTA	0.044	0.009	0.055	61	1042
CGTGGCATAAGGA	0.019	0.004	0.018	15	828
GGTGGTATAAATG	0.272	0.039	0.246	301	924
AGTGGTATATATG	0.027	0.005	0.022	21	932
GGCAGTATTGGTA	0.064	0.002	0.067	87	1209
GCTGGTATAAGCG	0.448	0.042	0.453	477	575
ACTAGCACAGATG	0.027	0.005	0.02	40	1930
AGTATCGCAAAGA	0.022	0.001	0.021	33	1521
CGCGTCGTAGATG	0.018	0.002	0.016	20	1229
AGTAGCGCTGGTG	0.027	0.004	0.033	16	472
GGCGGCACAGGTA	0.128	0.016	0.11	109	878
AGCATTACTGGGG	0.024	0.005	0.029	34	1132
ACCGTCGTTAATA	0.029	0.004	0.025	60	2357
ACCATTGTATGGA	0.021	0.005	0.025	48	1897
GGCGGCATAAGCG	0.547	0.056	0.532	740	650
CCCAGTGTATAGG	0.023	0.003	0.019	27	1393
CGTAGCGCTAGTA	0.027	0.008	0.018	17	946
AGCAGCGTATGTG	0.023	0.004	0.018	22	1217
CCCGGCGCTTGCA	0.027	0.003	0.025	39	1513
CGTATTGCTAATA	0.029	0.002	0.026	44	1648
GCCATTATTAGGA	0.057	0.007	0.049	99	1932
CCTGTTGTTGATG	0.028	0.008	0.016	19	1133
GCTGGCATTAGGA	0.669	0.025	0.666	725	364
GGCAGCGTAAAGG	0.068	0.005	0.065	64	918
ACTGTTGTAAAGG	0.026	0.002	0.023	34	1446
AGCATTACTGGTG	0.027	0	0.027	42	1508
CGTAGTATTTACG	0.026	0.002	0.025	26	1007
GGTGTCACTGGGA	0.098	0.014	0.078	38	449
CGCGTCACAAGTG	0.023	0.002	0.024	35	1414
AGCGGTGCTGGCG	0.025	0.003	0.028	20	686
CCTATTACTGGGA	0.026	0.005	0.033	47	1359

CCTGGTGTATGTG	0.02	0.002	0.019	16	833
CGCGTTGTTAATG	0.021	0.003	0.025	32	1274
AGTGTTATATATA	0.022	0.004	0.018	41	2220
CGTGGTATAAAGA	0.025	0.001	0.025	34	1333
AGCAGTACAAGGG	0.022	0.003	0.018	26	1434
GGTAGCACTAACA	0.057	0.011	0.042	59	1346
CGTAGCGCTAATG	0.022	0.007	0.013	11	824
CCCAGCGTAAGTA	0.02	0.005	0.025	66	2610
AGCATCGCTGGCG	0.018	0.003	0.023	41	1757
AGTGGCGCAAGTG	0.026	0.01	0.025	16	613
AGTATCACTGATA	0.022	0.004	0.016	30	1808
AGTGTTGTATGCA	0.019	0.004	0.014	16	1117
CGTGGTGCTGGTG	0.029	0.014	0.04	15	358
CCCAGTACATAGG	0.022	0.004	0.026	48	1803
CGTATTGCAAGGA	0.024	0.009	0.018	20	1086
ACCGTTACAAAGG	0.025	0.001	0.024	67	2707
GGCGTCGTTAGCA	0.077	0.007	0.082	78	870
AGTGTCGCATAGA	0.021	0.003	0.018	16	856
GCCAGTGTTGGCA	0.063	0.014	0.043	49	1089
ACTGTTGCTTACG	0.028	0.004	0.021	30	1379
ACTGTCGCTAGGG	0.033	0.01	0.024	18	727
GGTGGTATTGACA	0.124	0.017	0.1	89	799
CGCGTCACATATA	0.028	0.002	0.029	65	2195
GGTATTGCAAGTA	0.077	0.013	0.062	65	986
GGTGTTACAGACA	0.074	0.015	0.057	69	1140
ACCGGCACTAGCG	0.024	0.003	0.022	37	1655
AGTGTCGTTTGTG	0.022	0.006	0.015	9	575
CCTGGCGCTTGCG	0.03	0.002	0.033	24	713
CGCAGTGTTTGTG	0.025	0.006	0.028	23	788
CGCAGTGCTTGTG	0.026	0.011	0.012	9	724
ACTGGCACTTGGA	0.023	0.003	0.027	26	923
GCCGTCGCTGATA	0.052	0.01	0.058	60	972
ACTGTTATTGGGA	0.026	0.003	0.023	28	1168
GGCGTCATTTATA	0.072	0.011	0.056	82	1376
CGTGTTACTGATG	0.024	0.005	0.023	19	814
AGCAGTATATGGA	0.026	0.007	0.016	35	2211
GCTGTCGCATACA	0.055	0.014	0.041	44	1032
AGTGTTGTTTACA	0.017	0.003	0.019	22	1164
CGTGTCGTTGGGA	0.026	0.003	0.025	13	499
GCCGGTGCATATG	0.112	0.023	0.093	83	811

GGCGTTACTTACG	0.061	0.01	0.048	47	936
GCCAGTGCTGGTA	0.035	0.007	0.041	39	903
ACTATCACTAATA	0.024	0.003	0.02	93	4482
GCTGTCGTAAAGA	0.047	0.002	0.051	56	1046
CCCAGTGCTTATA	0.02	0.002	0.02	51	2465
ACTGGTACTTACG	0.025	0.002	0.022	35	1525
AGTAGCACATGCG	0.028	0.006	0.019	19	973
CGTGGCACAGACA	0.028	0.009	0.023	31	1321
AGCATTATAGATG	0.02	0.002	0.017	48	2748
GCCAGCACATACA	0.053	0.005	0.054	153	2658
CCCAGTACTGATA	0.029	0.003	0.024	80	3220
AGCGTTACATAGA	0.02	0.003	0.016	32	1943
CCTATCACTAATA	0.023	0.004	0.018	71	3803
CGCGGCATAAGTG	0.025	0.006	0.029	26	860
CGCGGCGTAGGGG	0.037	0.009	0.034	17	479
AGTATCACAAGGA	0.024	0.004	0.02	25	1241
ACCGGCATAGATG	0.026	0.002	0.023	43	1839
CGCGGCATAGATA	0.029	0.003	0.026	43	1634
CGTAGTATTTGCG	0.022	0.01	0.008	6	710
CGTGTCATTTGTA	0.025	0.002	0.025	23	890
ACCGTCGCATGGG	0.024	0.001	0.022	23	1016
CGTGTTATTTGGG	0.026	0.003	0.023	12	511
AGTAGTGTTGAGA	0.024	0.008	0.017	17	1013
GGCGGCGCTGGTA	0.09	0.017	0.105	73	619
ACCAGCATTGAGG	0.022	0.004	0.028	49	1684
AGCAGTACTTGGA	0.021	0.006	0.015	23	1495
GCCAGCGTTAGGA	0.065	0.006	0.06	60	936
CCCATCACTTAGG	0.027	0.003	0.024	43	1742
GGTAGCGTTTATA	0.084	0.016	0.061	48	737
CGCGTTGCATGTA	0.021	0.002	0.018	23	1255
CGTAGTGCTTATA	0.019	0.003	0.016	15	933
ACTGGTACTTAGG	0.024	0.008	0.013	12	941
ACCGTCGTTGGTG	0.02	0.002	0.018	15	803
GCCAGTACTAATG	0.051	0.01	0.038	67	1713
CGCAGTGCTAATG	0.022	0.002	0.022	27	1196
GGCAGCGCTAGCG	0.061	0.009	0.052	41	750
CCTGGTACAGGTG	0.02	0.005	0.014	12	834
AGTGTTGTAGGGG	0.029	0.007	0.019	10	527
CCTGTTGCAGGGA	0.02	0.007	0.026	26	964
GCCAGCGTATATA	0.061	0.01	0.057	103	1696

CGCATCGTTTGGG	0.029	0.008	0.022	17	764
GGCGGCGTATAGA	0.217	0.032	0.21	186	699
GGTGTTATAGGGG	0.067	0.011	0.052	23	420
AGTAGTACAAGCG	0.023	0.005	0.02	25	1234
AGCAGCGTTTGCG	0.021	0.002	0.019	22	1128
AGTGGCACATGTA	0.024	0.006	0.018	16	862
GCTGTTGCATGCA	0.051	0.014	0.039	34	849
GCTAGCGCAGGTG	0.072	0.015	0.062	34	514
GCTGTTATAGGCG	0.072	0.01	0.06	56	874
AGCGTCACTAATG	0.023	0.004	0.028	50	1749
AGTGTTGTTTGGG	0.022	0.005	0.028	12	423
CCTAGCGTTTGTG	0.029	0.006	0.021	20	921
ACCGGTGCTAACG	0.025	0.004	0.019	37	1861
GCCGGTGCTTATA	0.065	0.007	0.055	62	1070
AGCAGCACAGGTG	0.026	0.002	0.025	33	1267
ACTGTTGTAGACG	0.033	0.004	0.037	52	1366
AGTGGTACAGGGA	0.016	0.006	0.008	6	701
ACTGTCACTGGTA	0.024	0.002	0.021	34	1561
AGCGTCGCAGATA	0.02	0.002	0.021	38	1765
AGTGTTATTTGCA	0.021	0	0.021	25	1192
GGTATCGTAAGTG	0.127	0.007	0.118	88	660
GGCGGCGTATGGG	0.419	0.064	0.381	190	309
CCCGGCACATGCA	0.025	0.002	0.025	61	2343
GCTGTTGCTAGTA	0.058	0.01	0.045	35	735
CGCAGTGTTTATA	0.03	0.005	0.028	46	1621
CGCATTACTGAGA	0.028	0.002	0.031	55	1736
GGCGTCGCTGGTG	0.069	0.006	0.059	32	506
GGTGTTACAGGTG	0.094	0.015	0.074	40	499
CGCGTCGCATGTG	0.028	0.007	0.019	17	888
GCCGTTATAAGGG	0.06	0.001	0.06	58	911
ACCGGTATAAAGG	0.022	0.002	0.022	45	2047
GCTGGCGCAAGTA	0.624	0.046	0.622	801	486
AGTATCGTTAACA	0.025	0.005	0.019	44	2306
GCCATTGTTAAGG	0.055	0.005	0.05	64	1217
CGCAGCGTAAACG	0.025	0.002	0.027	53	1894
GGTGGTATATGCG	0.251	0.044	0.219	147	525
CGTGTTATAAAGA	0.03	0.006	0.022	41	1831
CCTATTGTTTGTA	0.023	0.002	0.025	45	1768
AGTGTTGCTAAGG	0.025	0.006	0.017	11	621
ACTGTTGTATGCA	0.028	0.007	0.022	33	1451

ACCGGTGCATAGG	0.023	0.002	0.025	27	1071
CGTGGCGCTTAGG	0.025	0.009	0.016	6	364
CGTATTATTGGTA	0.029	0.007	0.022	29	1312
CGTGTTGCAAGTA	0.027	0.015	0.012	12	987
CCCATTGCAAGTG	0.032	0.003	0.03	60	1933
GGCAGTACTTGCA	0.048	0.009	0.049	61	1190
CCCGTCGTTAGTG	0.018	0.001	0.02	22	1095
GCTAGTACATGCA	0.061	0.007	0.062	88	1328
AGTGGTGCATAGG	0.039	0.009	0.03	19	621
GCCATCATTGGGG	0.088	0.003	0.087	73	763
CCCGTCATTTGCG	0.026	0.002	0.028	43	1479
GGCGTTATAAAGA	0.055	0.004	0.05	96	1819
GGTATCGCAAACA	0.105	0.017	0.094	147	1414
GGTGTCGCTTGTA	0.089	0.017	0.07	35	462
AGTGTCGTAAGCA	0.024	0.009	0.011	14	1222
ACCAGTACTTATG	0.02	0.001	0.021	64	2997
CGTAGCATAGGCA	0.021	0.002	0.018	21	1151
GCTGGTACTGGTA	0.076	0.014	0.087	63	664
GGCAGCACAAACG	0.053	0.007	0.05	89	1697
AGTATTATAAGCG	0.023	0.006	0.025	50	1939
CCTGGCACATAGG	0.028	0.002	0.029	26	861
CCCGGTACTAAGA	0.027	0.003	0.028	64	2229
AGCATTGTTGGGG	0.023	0.009	0.016	15	900
ACCGGTATTAGTA	0.024	0.004	0.021	50	2301
GGTGTTGCAAATA	0.078	0.011	0.067	80	1111
AGCGGCGTTTAGG	0.023	0.011	0.02	10	482
CGTGGCGTAGAGA	0.03	0.008	0.018	11	593
ACCGGCGTAGATG	0.023	0.009	0.015	11	746
AGCATTATAAGGG	0.022	0.002	0.019	40	2025
GGTAGTGCAAACG	0.063	0.012	0.065	57	826
GCTATCATAGACG	0.067	0.007	0.058	94	1529
CCTGGTGCAGGCG	0.025	0.003	0.021	17	799
AGCGGCATTTGCG	0.023	0.005	0.03	15	484
ACTAGCGTTTGGG	0.026	0.006	0.02	12	599
GCTATCGTTGAGA	0.046	0.009	0.034	37	1058
CCCGGTGTTGATG	0.025	0.012	0.028	32	1118
CCTGGCGCTGGGG	0.025	0.005	0.021	9	428
CCTATTGTAAGTG	0.027	0.002	0.025	48	1858
ACTAGTGTTAGCA	0.024	0.003	0.024	45	1823
CGCGGCGCTGGTA	0.018	0.005	0.017	13	766

ACTGTCACAAGTA	0.022	0.001	0.023	58	2435
AGCATCGTTTGTG	0.022	0.005	0.022	27	1181
CGTATCGCATGGG	0.025	0.004	0.02	13	623
GGCGTTATATACG	0.058	0.003	0.057	79	1314
CGCAGCATAGACA	0.023	0.005	0.023	63	2733
CCTGGCATATGTG	0.026	0.007	0.02	21	1005
ACTAGTGCAGGCA	0.022	0.002	0.018	39	2076
CCTATCGCTTACG	0.025	0.005	0.018	30	1604
CCTATCGCTGAGA	0.026	0.004	0.02	34	1626
ACTAGTGTTTAGG	0.033	0.004	0.03	33	1051
GGCGTTGTTAAGG	0.073	0.015	0.07	52	695
AGTGGTACATGGA	0.023	0.001	0.024	20	800
AGCGGCGCTGACG	0.022	0.005	0.016	15	948
GGTGGCATATAGA	0.457	0.065	0.431	449	592
AGCGTTGTTAATA	0.021	0.002	0.019	45	2333
GGCGTTATTAATG	0.059	0.002	0.061	82	1252
GCTGTTGTAGGGG	0.054	0.01	0.064	30	440
ACCAGTGCTTGGG	0.026	0.003	0.022	23	1001
GCTAGTGTATACA	0.05	0.005	0.057	106	1753
GGCATTACTAGGA	0.058	0.01	0.044	56	1205
GCTAGCATAGACA	0.062	0.005	0.056	104	1763
CCTAGTATATGTG	0.022	0.003	0.026	41	1507
CGTGTCGTATAGG	0.023	0.011	0.012	7	578
AGCAGCACAGGCG	0.018	0.003	0.021	32	1469
CCCGGCACATACG	0.024	0.002	0.027	58	2122
CCTGTTGCAGGTG	0.025	0.007	0.032	28	846
AGCGTCATAGGGA	0.018	0.003	0.022	35	1577
GGCGGCATAGGCG	0.508	0.075	0.464	494	571
ACTATTGCAGAGA	0.021	0.001	0.021	50	2321
GGTAGTGTAGAGG	0.068	0.014	0.07	35	463
CCTATCGTTGACA	0.026	0.005	0.021	52	2386
CGCATTGTTTGCG	0.032	0.008	0.024	24	991
ACTGGCACAAGTA	0.024	0.003	0.022	47	2075
GCTGTTACTTGGA	0.062	0.001	0.063	44	652
GCTATTGCAAACA	0.047	0.002	0.046	113	2356
CGTGTTACAGGGA	0.025	0.008	0.015	11	747
AGTGGCGCAGGCG	0.019	0.004	0.015	7	463
GGCAGCATAAACA	0.054	0.009	0.045	142	2983
AGTAGCACATAGA	0.02	0.002	0.022	33	1462
ACCGTCGCATAGG	0.024	0.007	0.024	32	1294

CGTAGCGCTAGGG	0.028	0.002	0.027	12	439
CGTGTTGCAAATA	0.032	0.005	0.029	47	1581
CCCGTTATTGAGA	0.023	0.003	0.02	41	1981
GCCATCGCTAAGG	0.047	0.005	0.045	51	1080
GCTATTATTAATA	0.061	0.004	0.056	175	2956
CGCGGCGCTAATG	0.027	0.005	0.02	19	920
GGTGTCACATGCA	0.08	0.004	0.075	59	726
GGTATTATTAGCG	0.104	0.014	0.091	81	808
CGTGTTGTTGGCA	0.025	0.007	0.036	24	647
AGTAGTATAGACG	0.028	0.001	0.027	39	1427
AGCGTCATTGAGA	0.021	0.004	0.015	29	1847
AGCGGCGCAGGGG	0.023	0.003	0.019	11	574
CGCGGTGCATGCA	0.019	0.001	0.018	21	1161
GGTAGTACTGACG	0.065	0.015	0.063	45	672
CGCGTTACAAACA	0.024	0.004	0.023	76	3220
GGCAGTGCAAATA	0.051	0.006	0.042	81	1842
AGCATCACATAGG	0.019	0.002	0.017	35	2067
CGCATCACTTGCG	0.024	0.002	0.024	34	1404
CCTGGCGTTAATA	0.026	0.002	0.028	51	1764
GGTAGCGCTGGCA	0.061	0.016	0.068	40	546
CCTGTTACTGGGG	0.028	0.003	0.03	18	591
GCCAGTATTAGCG	0.06	0.01	0.074	120	1511
GGTGTCATATAGG	0.097	0.018	0.083	42	467
CCCAGTGCAAACG	0.025	0.001	0.024	66	2685
ACTATTACTAAGA	0.023	0.005	0.016	58	3639
CCCAGTGTAAAGA	0.029	0.007	0.024	67	2764
CGCGTTGCATAGA	0.022	0.005	0.022	27	1195
GGCGTTACTAGGG	0.07	0.006	0.065	43	615
CCCATTACTTATG	0.031	0.002	0.029	74	2497
ACTATTACAGGGA	0.022	0.002	0.024	48	1914
ACTGGCATTGGGA	0.03	0.002	0.031	27	854
ACCGGCACTGGTA	0.02	0.005	0.02	39	1877
CCTAGTATAGGCG	0.028	0.005	0.031	44	1368
AGTATCATAAATG	0.023	0.004	0.018	42	2299
GGTGTCATATATG	0.103	0.022	0.083	63	695
ACTGGTATATGTG	0.028	0.006	0.026	30	1116
GGTGTTATAAACG	0.086	0.005	0.08	101	1155
GCCGTCGCAGACA	0.048	0.005	0.048	63	1250
GGTGTCGTTAATG	0.116	0.01	0.113	75	586
GCTGTTGCTAATG	0.058	0.01	0.044	35	761

CGCATCATAGGGA	0.031	0.002	0.032	45	1363
CCCGTTGTAGACG	0.028	0.004	0.022	40	1749
AGTGTTACTAGCG	0.016	0.002	0.018	16	860
AGCATCATATATA	0.023	0.001	0.023	101	4315
ACTATCGCAAACG	0.027	0.001	0.026	68	2520
GGTATCGCTTAGG	0.115	0.028	0.075	40	490
GCTGTTACTTGCA	0.06	0.003	0.059	63	1013
CCCAGTACTAAGA	0.024	0.003	0.022	70	3108
ACTGGTGTAAATG	0.028	0.005	0.029	41	1366
ACCAGCACTGGCG	0.029	0.001	0.027	46	1630
CCTGTTACTAACA	0.027	0.002	0.025	69	2683
ACCATTATAGAGG	0.025	0.002	0.024	60	2492
GCTGGTATATAGA	0.149	0.011	0.134	166	1070
CCTAGTGCTTACG	0.026	0.003	0.023	34	1441
CGCAGTGTAGATA	0.022	0.006	0.021	39	1853
CGCATTGTAAATG	0.024	0.003	0.028	63	2201
ACCAGCATAAGGG	0.025	0.003	0.026	40	1501
CCCATTGTTGGTA	0.027	0.005	0.028	48	1677
GCTAGCGCTAAGA	0.051	0.006	0.045	41	872
GGTGGTGTATAGA	0.106	0.028	0.089	53	543
CGTGGCGTTTGGG	0.029	0.004	0.03	8	255
CGCGGCACTAAGG	0.03	0.001	0.031	30	926
ACCATTGCTTAGA	0.025	0.003	0.022	55	2430
CGTGTTACTTAGA	0.019	0.003	0.017	17	958
ACTGTTACAGGGA	0.027	0.003	0.023	32	1342
GCCATTGCAAGGA	0.041	0.005	0.048	68	1338
GCCAGCATAAGCG	0.058	0.006	0.055	109	1872
ACTATCGTAAGTG	0.037	0.005	0.032	54	1642
CGCGGTACATAGA	0.025	0.001	0.027	39	1414
CGTAGCATTAGTA	0.021	0.002	0.023	27	1143
CGCAGTATATGGG	0.031	0.008	0.028	28	967
CGCGTCATAGAGA	0.023	0.004	0.02	32	1552
GCTGGTATTGGTG	0.417	0.035	0.424	250	340
GGTATCATTAGCG	0.121	0.018	0.109	81	660
CGTGGTACTGGCA	0.022	0.005	0.017	13	745
AGTATTACTGGGG	0.018	0.003	0.021	15	712
GCCATTGCAGGTA	0.046	0.007	0.038	50	1276
AGTGTCGCTGACG	0.024	0.001	0.024	20	806
ACTAGTGTTGAGG	0.025	0.003	0.02	23	1133
ACCATTGTTAAGG	0.029	0.002	0.026	51	1883

**CCCAGTGCTAGGA**	0.027	0.006	0.027	40	1466
**GCCAGCGTTGAGA**	0.052	0.004	0.047	52	1045
**AGCATTGCTTATA**	0.022	0.001	0.021	52	2418
**CGCAGTGTTAACA**	0.024	0.004	0.026	57	2125
**AGCGGCACTAAGG**	0.019	0.002	0.021	19	869
**CGTGTCACAGACA**	0.025	0.002	0.022	36	1566
**ACCGTTATAGGTG**	0.023	0.004	0.021	34	1621
**CCCATCGCTTGCA**	0.02	0.004	0.021	49	2337
**AGTAGCGCAAGTA**	0.022	0.003	0.018	22	1180
**GCCGTCGCATGCG**	0.064	0.005	0.056	42	702
**CGCGTCATATGGA**	0.023	0.004	0.023	30	1296
**CCTAGCGTTTATA**	0.027	0.003	0.028	52	1836
**CCCAGTGTTGGTA**	0.027	0.005	0.021	33	1569
**CCCATTGTATAGA**	0.025	0.001	0.026	63	2367
**CGCATCGCTGGTG**	0.026	0.003	0.022	19	854
**CCTGGCACTGATA**	0.025	0.003	0.028	47	1616
**GGCGTCACTAATG**	0.061	0.012	0.045	58	1223
**AGCATCGTAGACG**	0.022	0.003	0.019	41	2137
**CGTATCGCAGGTG**	0.026	0.003	0.024	16	659
**GGTAGCATAGAGA**	0.068	0.008	0.068	65	893
**CCCGGTGCAGGGG**	0.025	0.003	0.028	19	661
**CCCATCACTAGGA**	0.022	0.002	0.025	55	2174
**CGCGGCGTTGGGA**	0.018	0.001	0.017	10	579
**GGTGTCACAAACA**	0.098	0.024	0.084	133	1458
**CCCGGTGCAAAGG**	0.027	0.003	0.024	30	1238
**GCTATTGTATAGG**	0.058	0.002	0.056	58	974
**CGTATTGCTAAGA**	0.022	0.002	0.021	26	1191
**GGCATTACTTATA**	0.06	0.005	0.052	115	2090
**CCCAGCGTTGACG**	0.023	0.003	0.023	45	1931
**GCCAGTGTTAGTA**	0.054	0.002	0.055	75	1295
**GCTATCACTTGGA**	0.074	0.014	0.064	58	850
**GCTGGCACTAATA**	0.212	0.008	0.209	305	1151
**ACTATTGCATAGG**	0.024	0.004	0.028	49	1702
**CGTATTGTAGATG**	0.027	0.005	0.021	24	1142
**CCTATTACTTGTA**	0.025	0.003	0.029	63	2084
**CCTAGTGTTTATG**	0.029	0.006	0.02	28	1392
**CCTATCATTGACG**	0.031	0.004	0.025	51	2018
**GCCAGCACATAGA**	0.057	0.007	0.066	120	1691
**ACCGTCGTAAAGG**	0.021	0.004	0.02	27	1337
**GCCAGTATTTAGA**	0.05	0.004	0.048	95	1894

CCCAGCACTGGCG	0.04	0.001	0.038	65	1654
CGTAGCGCTAGGA	0.029	0.008	0.017	10	588
GGCGGCATTGACA	0.269	0.043	0.254	411	1209
ACTGTCACTAGTA	0.026	0.003	0.021	42	1922
GCTATCACTGATA	0.061	0.002	0.06	104	1640
ACTATCATAAACG	0.03	0.006	0.022	81	3569
CGTGGCGCAGGGG	0.021	0.008	0.012	4	320
CCCGTTGTAAGGA	0.017	0.003	0.014	21	1508
CGTATTGCATATG	0.029	0.008	0.02	20	996
GCCGGCACATACG	0.218	0.016	0.219	321	1146
GGTATCATTAACA	0.121	0.021	0.097	191	1769
CCTATTGTATGGA	0.03	0.002	0.03	50	1603
GCCGTCATTAAGA	0.06	0.003	0.056	104	1763
CGCGTCGCAAGCG	0.024	0.005	0.025	30	1193
AGCGGCACTTGCA	0.017	0.001	0.016	22	1312
GCCGGTATTGGCA	0.068	0.003	0.065	86	1235
GGTGTCGCAAGCG	0.084	0.017	0.081	40	454
GGCGTCACTAGGA	0.054	0.004	0.049	46	888
ACCATCGCTTACA	0.023	0.002	0.021	78	3689
GCCAGCGTAGGCA	0.052	0.003	0.048	68	1348
ACCGGTGCATGGA	0.019	0.005	0.026	38	1416
CGCGGTGCATAGG	0.027	0.002	0.028	21	736
GCCAGCGCATACA	0.047	0.001	0.048	87	1710
AGCGGTGTTTATG	0.019	0.002	0.02	21	1044
AGCGTTGCAAAGG	0.031	0.003	0.03	38	1246
GGTATTGTATGTG	0.101	0.015	0.101	70	621
GCTGTCACAAGCA	0.062	0.004	0.057	83	1380
CGTGGTACTGACG	0.022	0.005	0.025	19	737
CGTGGCACATGCA	0.028	0.006	0.02	21	1022
CCCGTTGTTTACA	0.023	0.002	0.025	56	2160
GCTGTTATAGACG	0.054	0.004	0.051	64	1201
ACCGTCGCTAATG	0.026	0.004	0.02	38	1838
ACTGTCACTAGCA	0.025	0.003	0.028	61	2146
GGTAGTGTAGGTG	0.07	0.019	0.079	36	421
CGCAGCGCTGGGA	0.023	0.007	0.02	15	751
ACTGTCATTAGCG	0.027	0.002	0.028	39	1364
ACTGGCATTTATG	0.035	0.002	0.032	38	1134
GCTGTCGTAAACG	0.068	0.009	0.081	85	969
AGCATCATAAATG	0.023	0.003	0.019	79	4019
CCTAGTGTTGATG	0.025	0.003	0.024	33	1349

CGTGGTATTGGCG	0.023	0.008	0.023	13	547
CCCAGCATATGGA	0.027	0.002	0.024	51	2059
AGCATCACTTGCA	0.02	0.003	0.018	43	2296
GCCATTGTTGGTG	0.049	0.007	0.053	46	821
CGTAGCGTTGACA	0.029	0.006	0.034	38	1084
CGCGTCGTAAGCG	0.031	0.005	0.026	32	1214
CGTGTTGTAAAGA	0.025	0.007	0.016	20	1220
ACTATTGCTAAGA	0.025	0.003	0.025	69	2645
CCCAGCGCTAGCA	0.022	0	0.022	54	2457
CGCATTGTTAACA	0.027	0.002	0.028	77	2671
ACTATCACAGGCG	0.021	0.006	0.012	24	1904
ACTGGTATATGCA	0.027	0.002	0.024	45	1810
GGTATTATTGAGA	0.107	0.018	0.092	111	1099
CCTATTGTAAGCA	0.03	0.007	0.023	62	2655
CGTAGTGTATATA	0.026	0.001	0.027	33	1180
ACCGGCGCAAGGA	0.031	0.007	0.026	28	1052
CGCGGTATTTAGA	0.024	0.002	0.026	31	1153
GGCATCATTTACG	0.092	0.01	0.088	146	1506
ACCGTCGTTAAGA	0.027	0.003	0.024	43	1759
CGCATTGCATGCG	0.026	0.002	0.026	32	1219
ACTATCACTGAGG	0.022	0.004	0.016	28	1694
ACTATCGTATGGA	0.03	0.001	0.029	41	1389
CCCAGCATTTGTG	0.024	0.003	0.023	36	1523
GGTGGTGCTAGTG	0.091	0.011	0.101	34	303
AGCATCGCTAGCG	0.027	0.004	0.03	46	1483
ACTAGCACAAACA	0.024	0.001	0.023	114	4904
CCCAGCGTTGGTA	0.025	0.005	0.018	29	1561
GCCAGCGCTTAGG	0.045	0.013	0.036	24	652
GCTGTCATATATA	0.067	0.003	0.065	110	1595
ACTGGTATTAATG	0.032	0.003	0.027	43	1522
CGTAGCATTAATG	0.033	0.005	0.026	33	1235
ACTGTTGCATGCG	0.027	0.002	0.025	30	1165
GGTAGCGCAAATA	0.079	0.005	0.072	80	1028
GCCAGTGCAGAGA	0.048	0.006	0.047	57	1153
AGCAGTGTAGATA	0.026	0.003	0.023	57	2421
ACTGTTGCAAGGA	0.027	0.007	0.018	26	1459
GCTGGCACATGGA	0.214	0.038	0.176	136	637
CGCGTTATTAGGA	0.025	0.006	0.025	32	1241
ACCATCGCTAAGG	0.023	0.005	0.017	32	1887
ACCAGTGTAGACG	0.024	0.005	0.021	45	2103

GGTATTGCTAATA	0.073	0.018	0.063	80	1184
GCTATCGTATACA	0.058	0.005	0.05	88	1659
CCTGTTGCAAAGG	0.024	0.005	0.018	23	1258
CCTAGTACTGGCG	0.031	0.003	0.031	35	1112
GCTAGTATTGATG	0.06	0.001	0.058	73	1186
ACCGGTACTAGTG	0.023	0	0.023	41	1770
GCCGTCGCTAAGA	0.049	0.004	0.044	46	1000
AGTATCGCAGGCG	0.029	0.007	0.038	41	1034
GCTGTCGTTAGTG	0.058	0.016	0.063	32	480
CGCATTGCATAGA	0.027	0.001	0.026	44	1644
CCTGGCACTGGCG	0.028	0.003	0.028	25	854
AGTAGCGTTTGGA	0.025	0.009	0.014	9	650
CGTAGCATTTGTA	0.024	0.011	0.012	12	997
GCTATTATTTGTG	0.05	0.005	0.043	50	1110
GGCATCGCTAGTG	0.109	0.022	0.089	78	795
CGTAGTATAAGCG	0.027	0.001	0.026	29	1095
AGCAGTACAGGCG	0.022	0.004	0.017	27	1595
GCTAGCGTAAAGG	0.058	0.003	0.06	51	793
CGCAGCGTTTATA	0.023	0.001	0.023	39	1623
GGTGTCGCAAGTA	0.082	0.01	0.081	63	713
AGTATCATAAGTA	0.022	0.001	0.021	47	2244
AGTGTTGCTTGGA	0.018	0.005	0.016	10	605
AGCATCATTTGGA	0.021	0.003	0.017	33	1873
AGTGGCACTAACG	0.026	0.009	0.018	14	749
GCCGTCGTTAGCA	0.05	0.003	0.046	52	1071
CGCATTGCAAGTG	0.027	0.003	0.027	35	1247
CGTGGCACATGGG	0.023	0.007	0.032	13	391
GCCGGCGCTTATG	0.736	0.028	0.717	830	328
GCTGTTATATGCA	0.059	0.008	0.048	65	1297
AGTGTTACTGGTA	0.024	0.005	0.022	23	1000
GCTGGTACTAAGG	0.108	0.016	0.126	90	622
GGCGGCATTGAGA	0.285	0.038	0.257	260	753
GGCATTACTGATA	0.054	0.005	0.05	100	1912
AGCGTCATTAACG	0.021	0.003	0.02	45	2192
GGCGGTACAAAGA	0.062	0.009	0.051	74	1385
ACTGTCACTGAGG	0.03	0.006	0.027	29	1031
ACCAGTGCTTGCA	0.024	0.004	0.018	43	2350
GGTAGTATTAATG	0.059	0.004	0.054	62	1086
AGCGTTGTATGTA	0.016	0.002	0.018	30	1596
CCTGGCGTTGAGA	0.02	0.006	0.017	18	1063

GCTATCACAAAGG	0.061	0.011	0.048	70	1394
AGTGGTATTTATG	0.028	0.003	0.024	18	728
AGCAGTGCTTACG	0.022	0.005	0.019	30	1519
GCTGTCACTGACA	0.057	0.007	0.048	64	1283
CCTGTTGCTAGGA	0.026	0.006	0.025	26	995
GGTATCATTAGCA	0.121	0.022	0.106	130	1101
AGTAGTACTTGGA	0.024	0.003	0.026	25	943
GCTGTTATAAGGA	0.066	0.009	0.056	72	1223
ACTGGCGTTGATA	0.03	0.003	0.025	25	966
AGCAGTGTTGGGG	0.016	0.003	0.012	9	714
GCTGTTACAAATG	0.078	0.009	0.067	92	1285
ACCAGTACTTATA	0.022	0.001	0.02	99	4818
GGTATTACTTGTG	0.11	0.01	0.104	75	645
CCTGTCACTGGCA	0.023	0.006	0.016	25	1515
GCTATCATATGGA	0.061	0.005	0.059	75	1194
GGTAGTGTTAGGA	0.06	0.021	0.047	25	504
CCCGGTGCTAGCA	0.023	0.004	0.018	31	1706
CGTGGTATTTGTA	0.026	0.009	0.018	14	760
ACTAGCGCTTGTA	0.019	0.004	0.014	23	1607
GCCAGTATTAACG	0.052	0.004	0.056	135	2268
GGCAGCGCTGGTG	0.059	0.012	0.042	25	570
GCTAGCGTAGGCG	0.083	0.015	0.065	40	580
CCTATCGTTAATA	0.024	0.003	0.025	69	2733
GCCATTACTGGCG	0.05	0.012	0.043	68	1522
CGCGGTGTAGGCG	0.025	0.002	0.026	19	715
CCTGGCACTTACG	0.025	0.003	0.021	26	1207
ACTGTTGCAAAGG	0.023	0.005	0.018	27	1438
CGCATTACTGGGG	0.03	0.008	0.02	15	741
CCCGTTATTAAGG	0.03	0.008	0.038	70	1782
GGTGTTACATACA	0.07	0.01	0.055	71	1212
ACTATCATTTATA	0.027	0.004	0.026	92	3413
CCTGTCGTAGAGA	0.024	0.005	0.03	40	1272
AGTGGTGTTTATA	0.021	0.007	0.015	16	1024
GGTATCACTTATA	0.101	0.004	0.096	122	1153
CGCAGCGTAAGTA	0.025	0.003	0.022	38	1699
GCTGTTGTAAGCA	0.065	0.007	0.072	83	1072
AGCGGCATAAGCA	0.03	0.006	0.021	38	1746
GCCGTCATAGAGG	0.059	0.003	0.061	60	919
CCTGTTACAAGTG	0.024	0.006	0.016	23	1407
AGCGGTGTTAATA	0.022	0.003	0.023	43	1853

CGTGTCACAGGGA	0.025	0.006	0.032	23	698
GCCGGCATTTGTG	0.713	0.042	0.713	823	332
CGCAGCACTGACA	0.027	0.004	0.023	54	2268
GCCGGTGCAAAGG	0.11	0.019	0.115	101	779
GGTATCATAGATA	0.123	0.01	0.12	207	1524
ACCGTTGTTTACG	0.028	0.002	0.031	52	1637
CGCGTCGTATATA	0.025	0.001	0.024	39	1620
GGTGTCACTGGTG	0.086	0.016	0.073	29	367
ACTAGCGCTAATG	0.026	0.001	0.025	41	1597
AGCAGTACTAGGA	0.02	0.001	0.019	34	1740
CCTAGTGTAAAGG	0.023	0.003	0.023	39	1665
GGCGGCATTAGGG	0.461	0.056	0.443	333	418
AGTGGTGTATAGA	0.02	0.003	0.015	13	871
CCCATTACTGATG	0.022	0.002	0.019	49	2485
GCTGGCACAGAGA	0.212	0.024	0.191	184	781
CGTATTATATGTA	0.026	0.005	0.02	40	2003
GGTATTATAAGGG	0.103	0.016	0.08	75	861
CCTGTTGCAGAGA	0.02	0.003	0.02	28	1379
GGCATCGCTGAGG	0.087	0.024	0.055	43	736
GGTGTTATAAACA	0.081	0.014	0.064	120	1746
CCTGGTGTATGTA	0.026	0.002	0.027	34	1205
GCCGTTATTAGGG	0.055	0.005	0.055	43	741
GCCGTCGTTGACG	0.063	0.001	0.063	55	812
GGCAGTACATGCA	0.046	0.005	0.049	78	1501
CGTATTATAAGGG	0.025	0.002	0.025	29	1144
AGTATTGTAGAGG	0.023	0.006	0.022	22	984
GGTGTTATAAAGG	0.1	0.016	0.077	72	859
ACCGGTGCTGATG	0.025	0.008	0.028	36	1254
GGCAGCGCTTATA	0.062	0.012	0.048	54	1062
GGCGTTGCATGGA	0.056	0.008	0.047	33	674
ACTGGCATTTACG	0.032	0.007	0.032	39	1164
CCCATCGTTTAGG	0.026	0.009	0.032	46	1391
ACTATCACTGGTG	0.022	0.003	0.024	38	1572
ACCGTTGTTTGTA	0.02	0.002	0.023	33	1431
GCCAGCGCAAAGG	0.048	0.009	0.059	57	917
AGCAGCGCATGTG	0.015	0.006	0.011	13	1212
CCTATTATTTGCG	0.025	0.002	0.022	38	1670
GGCAGCACTTGCG	0.058	0.005	0.063	55	820
CGCGGTGTTAAGA	0.026	0.002	0.024	25	1026
GCCGTCGTAAGCA	0.062	0.007	0.053	76	1362

**CCCATTGTTGGGG**	0.021	0.003	0.017	16	906
**CGTATTATATAGG**	0.026	0.004	0.031	43	1325
**ACTATTACTTACA**	0.022	0.001	0.021	88	4043
**ACTATTACAAGCG**	0.026	0.002	0.024	66	2725
**AGTAGCGTTTGTA**	0.02	0.011	0.01	9	924
**GCTATTACAGATG**	0.068	0.006	0.06	94	1468
**GGCGTCATTGATA**	0.064	0.011	0.056	82	1383
**CGCATTGTAGGCG**	0.024	0.003	0.023	30	1283
**GGTGGCGCAAGTA**	0.152	0.019	0.148	104	601
**AGCGTCGCATGGG**	0.027	0.006	0.021	17	778
**GGCGTTGCTGAGA**	0.065	0.014	0.084	69	752
**AGCGTTGTATGGA**	0.022	0.004	0.026	32	1180
**CGCGTCGCAAAGA**	0.02	0.004	0.015	24	1612
**CCTGGCATATGGG**	0.026	0.002	0.026	19	706
**CGTGGTGTTAACA**	0.022	0.008	0.016	17	1017
**CGTAGCACTGAGA**	0.016	0.003	0.019	18	918
**AGCATCGTTGGCA**	0.02	0.005	0.018	34	1810
**GCCGGTATAGAGG**	0.171	0.016	0.19	180	767
**CGTAGTACTGAGG**	0.026	0.007	0.024	15	610
**ACTAGTACTAAGA**	0.024	0.002	0.023	78	3259
**GCTGGTACTGGTG**	0.114	0.015	0.104	48	414
**GGCGTCATAGGTA**	0.049	0.006	0.052	62	1136
**GCTGTCGTTGGGA**	0.061	0.003	0.064	30	436
**GGCATCGTATGTA**	0.104	0.026	0.08	109	1248
**GCCATCACAGACG**	0.056	0	0.057	124	2069
**CCCGGTACTAGTG**	0.028	0.001	0.029	39	1313
**CCCATTATTGGTA**	0.026	0.003	0.023	59	2546
**GCCATCGCAAAGA**	0.05	0.005	0.056	102	1732
**CCCAGCACTTGTG**	0.028	0.01	0.023	40	1683
**GGCGGTGTTAGTG**	0.074	0.007	0.069	41	553
**CGTAGTATAGACG**	0.022	0.002	0.019	23	1169
**AGCGGTGCAGAGG**	0.02	0.003	0.016	13	775
**GCTGTCGTATGTA**	0.059	0.003	0.055	49	837
**CGTAGCACTGATA**	0.027	0.007	0.018	23	1232
**ACTGTTGTTGACA**	0.028	0.001	0.029	51	1713
**GCCGTTGTTTGGG**	0.054	0.014	0.047	25	504
**CCTGTCACTAACG**	0.024	0.007	0.019	33	1731
**CCCGTTATTTGGG**	0.029	0.005	0.022	21	933
**CGTGGTGCATATA**	0.027	0.003	0.027	29	1051
**CGTGTCGTAGGGG**	0.04	0.011	0.029	11	369

GGTAGTATTAATA	0.066	0.007	0.058	102	1663
GGCAGTGCAGAGG	0.049	0.01	0.051	36	676
GCCGTTGTATGCA	0.044	0.003	0.043	53	1176
ACTGGTACATGGG	0.026	0.004	0.025	23	882
GGTATTGTTAGGA	0.109	0.025	0.11	76	615
GCCGGCGCTTACA	0.49	0.048	0.478	785	856
ACTGTTGTTGACG	0.024	0.005	0.03	39	1266
CCTGTTATTTGCA	0.026	0.001	0.026	41	1530
GGCGTCGCTAATG	0.069	0.017	0.056	53	888
ACCGGCGTTTAGG	0.024	0.005	0.019	10	505
ACCGTTACTAGTG	0.021	0.001	0.022	44	1938
CGCGGTACATGGG	0.021	0.003	0.025	17	672
ACCATTGTAGAGG	0.03	0.005	0.031	55	1734
GGTAGCGTATATA	0.093	0.019	0.066	65	920
CCCATCATTTGTA	0.028	0.002	0.025	72	2790
ACTATTGCAAATG	0.028	0.003	0.027	87	3148
GCCATTGTAGGTA	0.046	0.004	0.05	70	1335
GGTGGTATTAGTG	0.296	0.059	0.285	155	389
ACCATTGTTAACG	0.028	0.005	0.021	65	2964
GCTATTATAGGCG	0.062	0.001	0.064	95	1391
GGCATTGCTAGCG	0.073	0.004	0.078	73	867
GCCGTCACTTGTA	0.045	0.01	0.043	60	1325
CGTATTACAGGCA	0.025	0.005	0.019	29	1528
CGCGTCGTTTATG	0.026	0.002	0.028	28	959
GCCGGTGCTAACA	0.061	0.01	0.051	75	1383
ACCGGCGCTAGCA	0.024	0.003	0.021	28	1307
AGCGTTACATACA	0.022	0.005	0.018	55	2983
CGTGTCGTAGACG	0.022	0.005	0.028	24	826
GCCAGCACAGACG	0.048	0.006	0.052	90	1637
CCCGGTATTGACG	0.026	0.006	0.022	41	1818
GGTATTGTAAAGG	0.119	0.016	0.103	96	837
AGCAGCATATGCG	0.025	0.003	0.02	37	1776
CGCGTTGTTAAGA	0.028	0.003	0.024	32	1299
AGCGGCGCAGGTA	0.017	0.003	0.014	14	1009
GCTAGTATAAGCA	0.064	0.001	0.063	122	1821
AGCATTATAAGGA	0.02	0.004	0.014	44	3052
CCTAGTGTATGGG	0.031	0.011	0.042	42	952
AGCGGCGCAGGCG	0.021	0.002	0.024	18	745
ACCAGTGTAGGGG	0.022	0.003	0.019	20	1039
GGTAGTATTAGCA	0.053	0.013	0.045	48	1020

GCCGTTGCTTGCA	0.044	0.003	0.046	42	880
CCCAGCATAGGGA	0.047	0.001	0.047	85	1715
GGTGTTACTAAGA	0.068	0.018	0.068	63	859
CGCGGTACTTACG	0.024	0.003	0.022	24	1076
CGTATTGTTGATA	0.025	0.004	0.03	41	1346
ACCGGCACTGGTG	0.032	0.006	0.027	33	1169
GCTGTCACAGATG	0.058	0.007	0.065	62	894
CCTATTGTAAACG	0.022	0.002	0.021	62	2858
CGTGGCACAAGCA	0.026	0.003	0.03	36	1169
CGTAGTGTAGATG	0.021	0.007	0.03	23	741
AGCAGCACAAGTA	0.021	0.001	0.022	61	2725
AGCGTTATAGGGG	0.019	0.005	0.015	18	1204
CCCGTTGTTGATG	0.032	0.005	0.038	51	1294
ACTGTCGTTGGGA	0.028	0.006	0.02	15	717
CGTATTGTTAGTG	0.03	0.003	0.027	21	748
AGTGGCACAGGGG	0.026	0.007	0.033	12	347
AGTGGCACTTAGA	0.022	0.003	0.025	21	828
ACCGGTACTTAGA	0.021	0.002	0.022	52	2278
CGCGTCATAGGCA	0.026	0.007	0.02	32	1577
AGTATCGCTAGGA	0.023	0.008	0.018	17	904
ACTAGTATAGGGA	0.026	0.002	0.026	37	1388
ACCGTCACATAGA	0.024	0.002	0.023	73	3118
ACTATCACATACG	0.025	0.003	0.026	87	3246
CGCGGCGCTTGGG	0.017	0.007	0.008	4	525
ACCAGTATTTGCG	0.023	0.006	0.017	35	2042
GCCATTACTTAGG	0.052	0.004	0.054	69	1214
GGTGGCATAGGCG	0.69	0.065	0.619	553	340
AGCATCGCTGAGG	0.018	0.006	0.01	12	1221
CGTGTTACTTACG	0.03	0.006	0.025	25	973
CGTGGTATTAGCG	0.024	0.002	0.022	13	566
ACCAGCGCTTGGG	0.023	0.007	0.022	21	913
ACCAGCGCAAGGG	0.024	0.004	0.019	25	1287
AGTGTCGCTGGCA	0.024	0.007	0.024	18	747
CCTGTCACAGATG	0.029	0.001	0.03	43	1388
ACTGTTATTTACA	0.025	0.002	0.023	60	2512
ACTAGTACATATG	0.022	0.001	0.023	60	2537
CCCAGCATAAGTG	0.027	0.002	0.026	58	2206
AGCGGCGCAAGGG	0.018	0.005	0.022	16	709
GCCGGCGTAAATG	0.932	0.017	0.932	2154	157
CGCATTACTTGCG	0.03	0.005	0.03	38	1232

CGTGTTATTAGTA	0.031	0.003	0.029	34	1150
CCTGGCGCTTACG	0.025	0.006	0.018	17	953
CGCGGTGTTTAGG	0.024	0.002	0.023	15	626
CCCAGCGTTTGTG	0.019	0.009	0.016	22	1361
ACTGGTACTTGCG	0.019	0.004	0.013	12	904
GGCGTTGCTGACA	0.051	0.002	0.05	55	1055
AGCATCACTGACG	0.021	0.002	0.025	63	2499
CCCAGCATTTATA	0.026	0.003	0.024	78	3195
GGCGGCATATAGA	0.306	0.05	0.28	350	898
ACCAGCATTTACA	0.022	0.002	0.021	85	4040
AGCATTACTTATA	0.023	0.004	0.023	75	3213
GCTATCGCAGGTG	0.061	0.007	0.051	31	577
CCTATCACATAGA	0.024	0.002	0.023	68	2910
GCTGTCATAAAGG	0.067	0.002	0.064	72	1047
GGTGTTGCAAGTA	0.056	0.008	0.046	35	720
GCCATCGCATAGG	0.049	0.004	0.051	51	946
CGCATTACAGGTA	0.024	0.006	0.03	56	1786
GGCGGCGCATGCG	0.191	0.042	0.163	111	568
GCCAGTGCTTGTA	0.049	0.006	0.043	44	973
CGTATCGTATGTG	0.027	0.004	0.024	23	946
GCCATTACAAGGG	0.051	0.005	0.058	83	1340
GCTGGTACATGCG	0.113	0.007	0.118	79	591
ACCGGCACTAGTG	0.021	0.003	0.019	29	1466
CGTATCATAGGTG	0.024	0.004	0.021	22	1043
CCTGGTGTTTGTG	0.025	0.007	0.033	24	711
CCCAGCGTTTGGA	0.031	0.007	0.025	34	1345
ACTGTCGTAAAGG	0.031	0.003	0.027	29	1065
AGCGTCACTTAGA	0.021	0.001	0.023	36	1558
CCTATCACTGAGG	0.023	0.005	0.019	30	1521
ACTGGCGCTAGTA	0.026	0.001	0.026	20	755
AGTGTTACAGGCA	0.022	0.001	0.022	28	1232
AGTAGCATAGACA	0.021	0.004	0.02	40	1974
ACCATCGTATGGA	0.025	0.004	0.022	39	1769
GCTAGTGCAGACG	0.059	0.004	0.064	63	921
GGCGTTGTTTATA	0.066	0.004	0.069	76	1033
CGTAGTACAAATG	0.022	0.005	0.025	34	1341
GGCATCGTATGCA	0.121	0.018	0.101	155	1373
CCTGTCATTTAGA	0.028	0.008	0.016	24	1465
CCCAGTGTTTGCA	0.027	0.005	0.027	47	1719
GCCGGTGTATATA	0.18	0.023	0.182	272	1220

ACTATTATTTGTG	0.029	0.004	0.023	43	1802
GCCATTGTATGGA	0.048	0.002	0.046	59	1227
CGTAGCGCAGGTA	0.029	0.008	0.022	18	807
AGTATCGCATGGA	0.028	0.004	0.027	24	867
GCCGGTGTATGTA	0.181	0.036	0.173	196	939
GGCAGTACAAGCG	0.056	0.005	0.053	69	1235
GCTATTGCATATG	0.051	0.012	0.066	78	1110
GCCGGCATTGGCA	0.382	0.036	0.38	513	836
AGCGTCGCTTATG	0.02	0.003	0.017	19	1104
GGTGTCGCAAGCA	0.078	0.005	0.081	61	695
CGTAGTATATGCA	0.025	0.004	0.023	32	1362
GGTGGTACATATG	0.084	0.008	0.083	58	638
CGTGGCACAGGTA	0.03	0.003	0.026	20	762
GGTATTGCTAATG	0.109	0.006	0.103	84	731
CCCGGTGTTTACG	0.018	0.006	0.01	14	1326
CCTGTCATTTGCG	0.025	0.005	0.02	22	1100
CCTGGTGCTTGCA	0.022	0.006	0.015	18	1187
CCTAGTACAGGTA	0.024	0.004	0.019	34	1800
GGCATCACAGACG	0.083	0.013	0.065	112	1622
GGTATTGTTAAGA	0.109	0.004	0.104	109	937
GGCAGTATATGTA	0.058	0.003	0.056	104	1754
CCTAGCGCAGGCG	0.021	0.001	0.02	21	1006
GGCATCACTAGGG	0.077	0.008	0.069	66	895
GGTGTCGCTTATA	0.081	0.019	0.058	39	630
GGTAGTACTAAGA	0.066	0.007	0.061	63	974
CCTATTACTGATA	0.026	0.001	0.026	80	3020
CCTGTTATAAGCG	0.026	0.003	0.023	39	1650
GGCGTCATAAATA	0.065	0.008	0.074	176	2187
CGCATTATTAAGG	0.021	0.004	0.017	31	1756
GCTAGCATAGATA	0.068	0.002	0.068	128	1766
CCCGGTATATATA	0.026	0.001	0.025	76	2983
ACTAGCGTTAGGA	0.023	0.005	0.02	22	1082
AGCAGCACAGACG	0.024	0.004	0.019	37	1961
CGCGTTGCAAGTA	0.029	0.004	0.029	42	1425
GCCATTGTAAACG	0.05	0.007	0.04	95	2267
GGCATTATAGAGG	0.066	0.005	0.064	86	1249
AGTGTCATTTACA	0.026	0.005	0.02	35	1690
GCTGGTATTGAGA	0.142	0.011	0.151	143	804
ACTGGCATTAATA	0.025	0.004	0.019	47	2368
ACCGGCACTTGGG	0.02	0.004	0.021	21	996

GCCGGTATTGACG	0.165	0.009	0.162	206	1066
GGTATCGCTGGGA	0.098	0.028	0.068	33	449
ACTGTTGTTTGTG	0.029	0.002	0.026	22	820
CGTAGTACTTACG	0.019	0.006	0.011	10	905
GGTGGTGTAAACA	0.099	0.011	0.083	91	1002
GGCGTTACAAGTA	0.063	0	0.063	96	1430
CGCGTCACTTAGA	0.024	0.003	0.022	31	1400
CGCATCGCATGGG	0.029	0.013	0.029	25	838
CCCGGCGTAGGGA	0.025	0.005	0.029	29	960
AGCAGCATTAATA	0.02	0.004	0.023	83	3574
CCTATCATAGGTA	0.026	0.004	0.02	47	2316
CGTATTACAAATG	0.025	0.007	0.021	45	2140
GCTAGCACTTGGG	0.069	0.009	0.067	41	567
ACTGTCGCTTACG	0.023	0.004	0.018	23	1228
ACTGGCGTTTGGA	0.024	0.004	0.018	12	638
CCCGGTGTAAGTG	0.022	0.002	0.02	28	1345
GGTGGCATAGAGG	0.719	0.048	0.721	638	247
CGTAGCACTGACA	0.023	0.004	0.017	25	1445
GCCGGTGCATACA	0.06	0.002	0.062	91	1383
ACTGTTGTTTGTA	0.03	0.007	0.023	30	1272
AGCAGTGCAGACA	0.023	0.002	0.026	62	2358
GCCATTATAGGCG	0.056	0.013	0.042	73	1675
GGTATTGCATATG	0.099	0.013	0.085	70	749
CGCGTTACTGGCA	0.028	0.008	0.022	30	1346
AGTGTCACATGCG	0.031	0.006	0.03	23	756
AGTAGCGCTTAGG	0.025	0.005	0.026	16	600
GCCAGCGCTAGGG	0.059	0.005	0.055	34	584
CCTGTCATATGGG	0.031	0.01	0.022	19	842
CCTGGCGCTGATG	0.024	0.002	0.026	23	873
ACTGGTACTAGCA	0.021	0.003	0.018	37	1975
CGTAGTGTAGGTA	0.024	0.002	0.023	19	807
AGTGTCATAGGCG	0.025	0.003	0.021	18	836
ACCATTATAGGTA	0.023	0.001	0.023	85	3610
GGCATCACTGAGA	0.072	0.005	0.073	110	1394
AGTAGTGCAAGCA	0.022	0.003	0.02	28	1375
GCTATCGTTAATA	0.051	0.006	0.042	81	1841
CGCGGCGTAAGGA	0.028	0.006	0.022	19	851
AGCGTTGCTAACA	0.021	0.003	0.017	39	2219
GGCGGTATTTGTA	0.066	0.004	0.066	62	872
CGTAGCACATGGA	0.017	0.006	0.013	11	867

CCTGGTGTAAGGG	0.025	0.01	0.019	15	793
GCTGGCGCATGCA	0.615	0.042	0.607	715	463
ACTGGTGTTGAGA	0.032	0.004	0.027	25	897
GGCAGTACATAGA	0.052	0.008	0.041	67	1551
GGTGGTGCAAGCA	0.072	0.005	0.066	47	662
GCCAGTATTTGGG	0.055	0.007	0.053	52	936
CGCGTCACTAACG	0.024	0.004	0.029	53	1778
ACTAGCACAGGGG	0.033	0.007	0.024	23	931
GGCGGCGCTTATG	0.172	0.021	0.156	105	566
GCCGGCGTAAGCG	0.936	0.014	0.941	1491	93
GCCAGTGTTAGCG	0.048	0.019	0.027	29	1027
GCCAGTGCATGCA	0.046	0.007	0.039	55	1373
CCCGGCATAAGGA	0.032	0.003	0.033	61	1796
AGCGTTGCTGAGG	0.022	0.005	0.015	13	856
CGTATTGCTTGTG	0.021	0.003	0.018	12	664
AGTATCATTGGCA	0.023	0.006	0.014	20	1405
AGTGGCACAGGGA	0.019	0.002	0.017	11	652
GCTAGTGCTAGGG	0.057	0.011	0.05	25	471
AGTGTTATAGGGA	0.023	0.005	0.031	31	968
GCCATCGTAAGGG	0.058	0.006	0.054	51	902
AGTGTTGCTGGCA	0.027	0.005	0.029	23	761
GGTATCATATAGG	0.135	0.022	0.117	89	672
CGCGTCATTTGTA	0.023	0.003	0.027	38	1367
CGTAGTGCAAGGA	0.023	0.002	0.024	18	732
ACTGTCGCAGAGG	0.024	0.008	0.025	23	894
GGCAGCATAAGTG	0.05	0.012	0.036	45	1207
AGTATCATAGGCG	0.022	0.002	0.022	28	1263
GGTGTTGCTAACG	0.091	0.014	0.077	52	626
CGTAGCATTTATA	0.029	0.003	0.025	37	1421
CGTATCGCAAGGA	0.022	0.001	0.021	21	1003
ACCATCACAGAGG	0.026	0.002	0.025	58	2220
ACCAGTATTGGTG	0.022	0.003	0.025	44	1701
GGCAGTGCAAGTG	0.049	0.006	0.041	36	846
GCCGGCGCAGGTA	0.508	0.047	0.498	515	520
ACTATTGTTGGCG	0.024	0.002	0.024	46	1840
ACCGGCGTTTGTG	0.032	0.006	0.04	21	504
AGTGTTGCTGACG	0.018	0.001	0.016	12	718
CCCGGCGCAGACG	0.026	0.007	0.022	34	1534
AGCGTTACTGGTG	0.019	0.002	0.017	17	967
CCTGTTATAAACA	0.027	0.002	0.024	92	3692

GGTATTGCATATA	0.077	0.003	0.076	97	1185
CCTATTATTGGTG	0.028	0.006	0.023	34	1421
GCCGTTACATATG	0.055	0.005	0.055	74	1277
CCTGGTATTAGCG	0.021	0.009	0.02	19	941
GCTATCATTGATA	0.065	0.006	0.058	118	1934
GCTAGTGTTAAGG	0.056	0.002	0.053	48	857
GCCGGTATAAAGA	0.079	0.005	0.076	158	1934
GGTGGCACAAACG	0.377	0.047	0.367	434	750
AGCAGCGTAAGTA	0.019	0.003	0.018	41	2240
CCCGGCGTAGGGG	0.036	0.007	0.044	33	720
CCCAGCATTAGCG	0.026	0.003	0.023	49	2112
AGCGGCACTTGGA	0.021	0.005	0.022	21	923
ACTGTTACAGACA	0.026	0.003	0.022	68	2991
CCCGGCACAAGGG	0.027	0.003	0.023	30	1248
GCTATCGTTTAGA	0.047	0.006	0.054	56	977
AGTGGCGCTGATA	0.027	0.005	0.021	18	838
CCTAGCACAAGCA	0.026	0.001	0.027	70	2515
ACCGTCGTTTGCG	0.026	0.004	0.022	22	995
GGTAGCGTAGAGG	0.095	0.011	0.083	35	389
GGTGTTGCTAGTA	0.071	0.007	0.061	38	580
GCCAGTGTTTAGG	0.053	0.01	0.066	54	768
CGCGTCATTGAGA	0.024	0.004	0.017	24	1358
CGTGTTGCTTAGG	0.027	0.007	0.026	12	447
CGCATCACTTGCA	0.022	0.004	0.018	38	2017
ACCGGCGTTGGTG	0.033	0.004	0.033	16	467
GCTATTACTAACG	0.059	0.014	0.039	75	1840
AGTATCACATACA	0.023	0.003	0.027	66	2344
AGCGGTACAGGCA	0.017	0.003	0.02	32	1543
GGTAGTGTTAGGG	0.055	0.022	0.03	10	322
GGTAGCGTAAGTA	0.075	0.015	0.063	52	767
CGCAGCGTAGGTG	0.026	0.009	0.015	12	806
CCCATTACAGATA	0.023	0.003	0.027	115	4148
CGCAGTGTAAGGA	0.026	0.001	0.026	33	1238
ACCAGCACTTGCA	0.024	0.004	0.019	64	3377
CGCGTCACATAGA	0.024	0.001	0.023	38	1646
ACCGGCGCAGGGA	0.029	0.008	0.034	29	824
GGTGGTACTAGCA	0.083	0.014	0.081	57	651
GGTATCGTTTGCA	0.121	0.01	0.115	92	707
AGCGGTGCTTAGA	0.021	0.005	0.016	15	950
AGCGTTGCATGTG	0.025	0.003	0.021	23	1047

AGCGTCGCATATA	0.022	0.003	0.024	46	1882
GCCGGTGTAAACG	0.392	0.041	0.392	653	1011
CCCGTTGTATAGG	0.023	0.005	0.029	36	1222
GCCGTCACTAGTA	0.047	0.007	0.038	58	1479
AGTAGTATTTGTA	0.023	0.003	0.022	32	1426
ACCGTCGCTTGTG	0.021	0.005	0.015	17	1115
CGCGGTGTAGAGA	0.028	0.002	0.028	30	1030
CGCGTCACTTGCG	0.022	0.007	0.029	31	1047
ACTGGTATATATG	0.024	0.003	0.02	30	1443
AGTATTATTAGGG	0.023	0.006	0.015	17	1117
CCCGTTACAGGGG	0.049	0.003	0.046	47	972
ACTGTCACTGATG	0.028	0	0.028	39	1353
CCCAGCGTTTGCG	0.019	0.002	0.019	27	1382
CCTGTTATAAACG	0.025	0.004	0.019	48	2423
AGCAGCACAGGCA	0.023	0.005	0.02	42	2090
GGTGTTACAGGGG	0.085	0.002	0.084	33	358
CGCGTCGCAGATA	0.029	0.004	0.025	40	1567
AGCGGTATAGGCG	0.017	0.004	0.012	12	1003
AGTGGCGCTGACA	0.021	0.003	0.022	23	1001
AGCAGTGTTAGGA	0.023	0.004	0.024	32	1280
CCTGTTGCAAGTA	0.029	0.006	0.025	43	1704
AGTGTTACTAAGG	0.018	0.006	0.01	10	992
GGTAGTACAGGCG	0.063	0.011	0.052	31	565
GGCATCACTGGGG	0.071	0.017	0.071	61	804
GGTATCACAGGTA	0.093	0.003	0.096	92	862
AGTGGTGTAAACG	0.021	0.008	0.025	26	1033
CGTATCACTGACG	0.023	0.004	0.018	21	1145
AGCGTCGTTGGTA	0.018	0.003	0.015	17	1116
ACTAGTGCTGGGA	0.019	0.005	0.025	31	1189
ACTAGTATATACG	0.028	0.004	0.024	68	2733
GGCATCATTGACA	0.075	0.002	0.072	165	2129
CGCATTGCATATG	0.026	0.004	0.021	31	1424
AGCGGCGTATGTA	0.024	0.002	0.022	25	1113
CGTGTCGTTGGCG	0.023	0.008	0.013	8	594
CGTATCACTGGCA	0.025	0.004	0.019	22	1155
ACTATTATAAGTA	0.024	0.002	0.025	99	3934
CGCGTCATTAGTG	0.026	0.003	0.022	25	1117
CCTAGCGTAGGGA	0.037	0.011	0.035	33	907
AGTAGTGTAAGCG	0.025	0.007	0.029	32	1075
GCTAGTATTAGTG	0.063	0.007	0.054	59	1038

CCTGGTGCAGAGA	0.023	0.003	0.019	23	1178
GGCGTCGTATGGA	0.065	0.004	0.07	50	665
GCTGTCATAAGTA	0.062	0.009	0.051	74	1382
CGCGGCACAGGTA	0.027	0.002	0.03	35	1136
CGCGTCGCTAGCG	0.023	0.006	0.014	14	975
ACTGGTGTATGTG	0.033	0.005	0.034	28	801
GCCAGCGTAGGTA	0.052	0.003	0.048	60	1190
AGTGTCGTAAGTG	0.028	0.004	0.031	25	787
ACCGGTATTAGGA	0.022	0.005	0.029	50	1694
GCTAGTACTAGTA	0.067	0.006	0.067	87	1214
CCTAGTGCATGTA	0.024	0.005	0.019	35	1766
GCCATCACTAAGG	0.049	0.004	0.046	78	1603
AGTAGTGCATATA	0.024	0.004	0.018	28	1520
GCTGGTATATGCA	0.157	0.005	0.162	201	1036
AGTGTCACTAACA	0.029	0.007	0.019	32	1690
CCTAGTGCAGGCA	0.019	0.004	0.02	34	1690
AGCGGCGTATGGA	0.024	0.006	0.021	19	899
GGCGGTATTAGTG	0.125	0.022	0.11	77	621
GGCAGTACAGAGA	0.046	0.003	0.05	67	1285
CCCGTTGCAGGGG	0.025	0.002	0.023	20	858
AGCGGCGTTTACA	0.015	0	0.015	20	1320
AGTGGCATTAGGA	0.02	0.007	0.027	14	508
ACTAGTACAAGGG	0.025	0.003	0.026	41	1563
CCCAGCATTGAGA	0.028	0.001	0.027	60	2176
CGCAGCACAAGGA	0.018	0.002	0.015	25	1605
GCTGTCATTGAGG	0.058	0.01	0.05	39	734
CCTGGTACTTGTG	0.029	0.004	0.033	27	789
ACTAGCGTAAGCG	0.026	0.012	0.027	33	1200
AGCAGTGTATGTA	0.021	0.002	0.019	36	1877
GGCAGCATAAACG	0.05	0.005	0.057	115	1904
GCCATTATTAATA	0.052	0.004	0.049	204	3924
AGCATTGCATGTA	0.025	0.005	0.018	39	2149
GGTATTGTTAACG	0.109	0.009	0.103	107	934
ACTGTCGTTTAGA	0.019	0.004	0.021	21	997
AGTAGCGTTAGTA	0.023	0.002	0.026	29	1082
GCTATTGCTAATG	0.058	0.012	0.043	54	1205
AGTAGTGCTAGTA	0.02	0.002	0.017	17	972
CGCGGTACTGGTA	0.019	0.005	0.017	18	1015
GCCGTTGTTAACG	0.044	0.001	0.045	62	1322
ACCATCGCTGGGA	0.026	0.005	0.032	53	1600

AGCGTCACTGGGA	0.022	0.001	0.024	25	1038
CGCGGTGTTAGCG	0.031	0.01	0.043	31	685
CGTGGCACAAACG	0.026	0.002	0.025	28	1082
ACTGTCGCAGATG	0.024	0.003	0.026	34	1271
GCTGTTGTTAGCA	0.048	0.012	0.056	55	932
ACTGTTACTAGTG	0.026	0.004	0.026	32	1182
GCCAGTGCTAGGA	0.05	0.014	0.066	58	825
CGTGTTACTTACA	0.023	0.003	0.018	28	1517
AGCGTCGCTGATA	0.019	0.003	0.015	24	1622
GCCGGTACAAGTG	0.075	0.012	0.066	74	1046
GCCGGCACTGGGA	0.093	0.011	0.106	80	672
CGCGGTATTGGCG	0.024	0.008	0.014	12	849
ACTAGTGTTTGCA	0.025	0.006	0.018	29	1593
GCTGTCACATGCG	0.056	0.01	0.063	47	694
GGCATCGTTGATG	0.101	0.007	0.097	107	1000
CCCGGCGCTAGGA	0.02	0.003	0.024	27	1113
CCCATCGTAGGGG	0.046	0.012	0.036	38	1005
ACTAGTATATGCG	0.03	0.009	0.018	32	1762
CCCGTTGCTGATG	0.027	0.004	0.032	44	1339
CGCGTTGTATATG	0.024	0.002	0.027	33	1193
GCCGGTGTTGGCA	0.161	0.03	0.156	156	846
AGCGTCGCTGGTG	0.021	0.005	0.025	19	744
AGCAGCGCTGGGA	0.02	0.003	0.024	21	846
GCTAGTATTAGTA	0.057	0.002	0.054	83	1440
ACCATCGTTTGTA	0.025	0.004	0.019	38	1990
GCCGTTACTGACG	0.051	0.005	0.053	71	1261
GCTGGTGCTGATA	0.142	0.011	0.157	123	661
CGCATTGTTTAGA	0.025	0.005	0.018	25	1378
CGCATTGTTTAGG	0.031	0.001	0.031	32	1017
GGTATCGCTGGCG	0.093	0.032	0.071	50	656
GCCGTTATTAAGA	0.059	0.008	0.05	106	2020
AGCGTTATTAGGA	0.025	0.002	0.026	43	1590
GGTATCGTTAAGG	0.134	0.032	0.089	58	592
CGTGTCGCTGGGA	0.024	0.006	0.032	15	450
ACCGTTACATAGG	0.021	0.003	0.021	47	2198
CGTAGTGCAGACA	0.024	0.002	0.026	31	1143
ACTATCGTTTGGA	0.033	0.006	0.027	34	1217
AGCGTCATTAGTA	0.023	0.001	0.024	45	1866
GCCGTTACTGACA	0.051	0.003	0.054	104	1817
GGCATCATTGGGG	0.054	0.009	0.06	55	866

ACCGGTATTGACG	0.023	0.002	0.022	42	1870
CCTATTGTTGGTG	0.027	0.002	0.026	28	1053
GCCATCGTATGGA	0.054	0.005	0.057	72	1195
CCCGGTGCTGGCG	0.024	0.002	0.023	24	1004
ACTGTTATTGATA	0.023	0.002	0.02	46	2253
AGCATTATTTGCA	0.024	0.002	0.022	65	2911
AGCAGCGCAAACA	0.021	0.002	0.022	70	3056
GCCAGCGTTTATG	0.06	0.004	0.062	65	980
CGCATTACATGTA	0.027	0.002	0.028	65	2217
AGCGGTATTGATA	0.016	0.002	0.014	28	2011
GGCATCGTAGAGA	0.108	0.014	0.093	128	1252
AGTGGCACTAATA	0.023	0.004	0.018	26	1419
CGTGTTGCAGGGA	0.021	0.013	0.014	8	578
CGTGGTACTTACA	0.026	0.006	0.019	22	1126
CCTGGTGTATGGA	0.024	0.007	0.014	13	893
CGCAGCGTAAGGG	0.035	0.004	0.04	34	816
AGCGGTATAGATA	0.022	0.001	0.02	49	2365
AGTGTCACTGGGA	0.021	0.008	0.032	22	671
CGCGTCATTTGTG	0.026	0.007	0.035	34	926
AGCATCACTTGGA	0.019	0.003	0.021	35	1637
GCCATTACTGGCA	0.054	0.004	0.06	106	1660
GCTAGCACATGCA	0.063	0.005	0.06	81	1265
GGCATCGCTAACG	0.105	0.02	0.085	126	1357
CGCATTGTAAATA	0.025	0.002	0.027	91	3237
ACTAGTACTGGTA	0.023	0.005	0.017	37	2174
CCCAGCGTATAGG	0.024	0.004	0.026	41	1518
ACCGGTATTGATA	0.023	0.001	0.024	68	2723
GGCGGTACAAGTA	0.063	0.005	0.057	70	1166
CCCGGTGCAAACA	0.023	0.001	0.022	67	3038
CGTAGTGCAGGTG	0.03	0.004	0.029	14	467
GCCGGTGCAGACA	0.063	0.002	0.061	85	1307
ACCAGTATTGACG	0.019	0.003	0.014	40	2758
GGCATCACATGTA	0.07	0.019	0.054	91	1583
ACTAGCGTATATA	0.029	0.007	0.021	49	2231
GGTAGTATTGATG	0.064	0.003	0.059	48	760
CGCGGCACTTGCG	0.023	0.003	0.02	16	781
CCTAGTATAAGGA	0.026	0.004	0.021	44	2023
CCTGTCACATGGA	0.032	0.007	0.025	33	1289
GCCGTCATTAGTA	0.049	0.012	0.04	69	1669
AGCGGCGCTTGGA	0.015	0.007	0.025	18	706

CCCAGTGTAGGTA	0.024	0.004	0.02	34	1679
GCTAGCACATATG	0.062	0.004	0.065	75	1077
AGTAGTGCAGACA	0.024	0.006	0.017	25	1417
ACCAGTACTGGCA	0.021	0.002	0.021	70	3206
CGTGGCGTTTGTG	0.04	0.013	0.024	8	324
ACTGGTATTGGTG	0.023	0.008	0.016	13	785
ACTGTCGTAGGCA	0.027	0.003	0.025	32	1249
CGTATCGCATATA	0.022	0.001	0.023	36	1540
ACTATCGTATGTG	0.033	0.004	0.029	44	1454
GCTATTACTGGGA	0.05	0.003	0.046	41	844
GCCATTACTAGTG	0.045	0.007	0.035	52	1436
GCCAGTACTGACA	0.049	0.009	0.043	103	2308
CCTGTCGTAAGCA	0.021	0.002	0.019	36	1884
GCTGGTGCTGGCG	0.218	0.032	0.214	117	431
GGCAGCGTTGGCG	0.048	0.012	0.033	24	702
GCTGGTACTTAGA	0.071	0.01	0.057	45	743
CGCGGCATTAGTA	0.021	0.001	0.019	24	1269
GCTAGCGTTAGTA	0.049	0.009	0.049	44	861
ACCGTCGCTAAGA	0.024	0.004	0.018	37	2045
GGCAGTGTATGTG	0.06	0.013	0.058	47	758
CGCGTTACAAACG	0.022	0.005	0.015	32	2096
GGCGGTGCAAAGG	0.064	0.007	0.055	39	674
CGCATCACTGGTA	0.028	0.003	0.025	43	1708
CGTGTCACAAGCG	0.024	0.005	0.024	22	911
CCCGGCATTTGTA	0.027	0.004	0.022	38	1724
GGCATTACATGTA	0.063	0.006	0.055	94	1602
GGCGGCACTTGCG	0.28	0.066	0.261	212	600
CCTGTTATATGCA	0.027	0.002	0.03	62	1986
GGCGGTGCTAGCG	0.065	0.018	0.078	44	517
GGTAGCGTATGGG	0.077	0.018	0.075	27	331
GGTAGTATTGAGA	0.056	0.003	0.059	52	827
ACCATCGTAAAGG	0.02	0.002	0.023	52	2200
ACTGTCGCATACG	0.02	0.002	0.017	26	1474
CGCATCGTAGACA	0.026	0.003	0.027	75	2678
GCCGTTACTTACG	0.053	0.009	0.056	70	1183
GGTATCGCTGGTG	0.11	0.005	0.105	51	435
GCCGTTGCTAATG	0.048	0.007	0.044	44	950
AGTATCGCTTATG	0.025	0.005	0.019	20	1010
AGTAGTATAGATG	0.024	0.003	0.023	37	1562
GCTGTTACTGGTG	0.06	0.007	0.067	35	485

GGCGGCGCTTAGA	0.095	0.016	0.1	68	609
GCCGGCGCTAACA	0.513	0.045	0.521	988	910
GGTGGTATAGAGA	0.129	0.028	0.141	101	614
GCCAGTATAGATA	0.054	0.006	0.047	146	2988
ACCGGCGCTTATG	0.025	0.004	0.021	22	1036
AGTGGCGCTTGCA	0.024	0.004	0.028	18	636
GCTAGTGCAGACA	0.061	0.006	0.053	79	1424
GGCGGCACAAGCG	0.253	0.034	0.222	255	896
CCCGTCATTGGGG	0.025	0.001	0.025	23	899
ACTGTCACATGGG	0.027	0.003	0.03	26	840
CGTGGCATTTGCA	0.02	0.004	0.015	11	704
GCCGTTGCAGGTA	0.053	0.006	0.048	44	864
GCTGTTGTAGAGG	0.059	0.018	0.035	24	654
AGCAGCGTTAACG	0.021	0.002	0.018	34	1848
GCCGGCGCAAGGA	0.503	0.032	0.497	519	525
CCTGTTGTAGACA	0.027	0.001	0.026	53	1988
CCTGGTACAAATA	0.027	0.004	0.032	91	2784
CCTAGCATTGGGG	0.044	0.008	0.054	41	712
ACCAGCGTAGGGG	0.024	0.009	0.025	20	770
GCCGTCGCAGGGA	0.048	0.006	0.041	26	615
GGCATTGCTAGTA	0.058	0.007	0.056	66	1116
GGTAGCATTGGTG	0.053	0.01	0.054	24	418
CGTGTCATTTATG	0.031	0.002	0.032	31	945
CCTGTTGTAGGTG	0.02	0.006	0.014	13	929
AGTAGCGCTAGTA	0.024	0.013	0.017	15	894
CGTGGTACTTGTA	0.022	0.003	0.024	19	776
GGTAGTACTTATA	0.059	0.023	0.028	30	1054
GGCGGTGCAAACA	0.05	0.002	0.047	74	1502
CCCATTACAAATG	0.026	0.002	0.023	90	3830
AGCAGTGCAGGCA	0.021	0.004	0.018	30	1681
ACCGTCATTAACG	0.022	0.005	0.021	62	2883
GGCATTGTAGATA	0.071	0.016	0.058	108	1770
GCCATCACTTAGG	0.049	0.007	0.041	50	1158
CGTGTTGTATGTA	0.026	0.004	0.021	18	842
CCCGGTATATGTG	0.02	0.004	0.022	32	1453
AGCGTTGTAAGTA	0.022	0.003	0.026	50	1853
GCTAGCGTTAGGA	0.071	0.014	0.051	36	669
AGCGTCGCTAACA	0.019	0.003	0.017	39	2263
CCCGTCATTAATG	0.026	0.004	0.026	62	2336
ACCAGTGTTTGCG	0.022	0.003	0.025	35	1385

CCTATTATTGAGA	0.025	0.003	0.021	48	2285
CCTGGTATTAGGG	0.035	0.006	0.027	18	659
ACTGTCACTGATA	0.024	0.004	0.02	45	2241
GCCATTACTTAGA	0.049	0.006	0.041	79	1827
GGCATCATATAGA	0.088	0.009	0.087	170	1795
AGCGTTGTTAACA	0.023	0.005	0.03	75	2422
GGCAGTGTATGCA	0.054	0.01	0.045	55	1158
GCCATTGTAGGGA	0.052	0.01	0.053	61	1089
ACTGGCGTAAACA	0.024	0.006	0.019	35	1805
CGTGTCACAGATG	0.021	0.002	0.019	18	914
ACTATCATATGCG	0.029	0.002	0.031	69	2127
ACTGGCATTTGCG	0.03	0.003	0.034	31	870
CCTGTCATAAATA	0.024	0.002	0.021	74	3409
CCCAGCGTAGAGA	0.018	0.003	0.014	28	1918
CCTGTCGCTGAGG	0.021	0.004	0.021	15	690
AGCGGCACTGACG	0.023	0.006	0.018	20	1065
GGTGTTATATGGA	0.084	0.024	0.051	40	744
CCCAGTGTATGTG	0.026	0.003	0.028	39	1345
AGTGTTGCAAGCA	0.022	0.002	0.02	24	1204
GGTAGCACAAAGA	0.072	0.012	0.058	73	1179
CCTGGTGTTGATA	0.024	0.008	0.014	19	1299
GGCATCACTAATA	0.07	0.012	0.061	157	2403
GGCGTCACAAGCG	0.057	0.004	0.057	65	1067
GCCATTGCAGATG	0.056	0.005	0.05	64	1209
GCCGTCATTGAGA	0.05	0.007	0.04	61	1448
CCCATCGTTAACA	0.023	0.002	0.025	96	3695
CGCGGTATAGGGA	0.028	0.006	0.024	21	869
ACTGGTGTATGGG	0.021	0.007	0.016	10	615
ACCGGTGTAAGCG	0.02	0.009	0.011	17	1509
ACTGTTATTGACG	0.034	0.008	0.026	44	1675
GGCGTCGTTAGGG	0.059	0.003	0.058	31	507
CGCGTCGCAGGCG	0.025	0.009	0.013	13	1007
GCTGTTACAAAGA	0.058	0.01	0.046	72	1497
CGCGGCGTATATG	0.028	0.005	0.021	19	898
CGTGTTGCAAGGA	0.027	0.006	0.022	18	798
CGCGGTGCAGATA	0.021	0.004	0.016	22	1352
GCTGTCGTTTAGA	0.056	0.003	0.052	34	620
ACCATTATTTGCA	0.023	0.002	0.021	76	3548
GGTATCGTTTAGG	0.141	0.012	0.129	70	474
GGCGGTGTTGGGG	0.075	0.014	0.059	19	301

AGCAGCGTAGAGA	0.022	0.004	0.022	37	1668
AGTAGCACTGAGA	0.024	0.002	0.027	30	1098
CCTATTGCAGATA	0.022	0.004	0.016	45	2685
GCCGTCACAAATA	0.055	0.005	0.055	166	2846
GCTGGCGTTAGCG	0.937	0.011	0.934	1023	72
ACTGTTGCATGTA	0.022	0.001	0.021	36	1705
ACCGGCATTTGTG	0.022	0.003	0.02	25	1207
CCCAGTGCTAGGG	0.026	0.005	0.024	23	947
ACTGGTACAGAGG	0.023	0.004	0.021	23	1087
CCTAGTGCAGGGG	0.024	0.003	0.025	20	765
CCTGGTGTAAGTG	0.026	0.007	0.022	24	1079
CGTAGCATTGGCG	0.023	0.008	0.015	11	700
CCTGTTACAGATA	0.03	0.003	0.029	65	2159
CGTAGCGTAAAGG	0.037	0.003	0.035	31	862
CGTGGCGCAGGTG	0.026	0.011	0.041	15	355
AGCAGTATATGCG	0.023	0.001	0.022	44	1931
CGCGTTGCAAGCA	0.025	0.005	0.021	34	1601
ACCGTTATTGGGA	0.023	0.002	0.024	36	1444
AGCGGCATATATA	0.024	0.005	0.02	38	1877
CCTGGCGTTGGGG	0.027	0.002	0.025	12	476
CGCGGTGTAAGTA	0.026	0.003	0.023	30	1279
GCCGTTACAGAGG	0.049	0.009	0.038	40	1003
CGTGTTATTTGCG	0.02	0.003	0.018	14	755
GGCATTGTTAGTA	0.065	0.008	0.056	74	1251
GCCGGTGTAAAGA	0.173	0.037	0.164	233	1189
GCTAGCATTTGTA	0.066	0.004	0.067	76	1051
CCTAGCATAAGGA	0.028	0.001	0.028	54	1851
CGTAGCGCAAAGG	0.024	0.004	0.02	16	775
AGTGTCACTGACA	0.024	0.009	0.018	25	1395
CCCGTCGTAGGCA	0.022	0.003	0.024	46	1895
GGCATTGTTGGCA	0.073	0.015	0.058	66	1075
CCCAGTGCAAGCG	0.026	0.004	0.024	47	1897
GGCGTTGCATGTG	0.072	0.011	0.069	44	592
ACCGTCGTAAGGA	0.023	0.004	0.019	28	1442
GCTGTTACTTGCG	0.064	0.01	0.076	51	623
AGTAGTGCTTATA	0.018	0.005	0.024	30	1225
GCCAGCATTTGGA	0.052	0.003	0.05	62	1183
CGCAGCGTAAGGA	0.025	0.007	0.017	22	1271
ACTATCATTTACA	0.026	0.003	0.024	86	3543
CGCAGCACTGAGA	0.023	0.001	0.022	34	1495

AGTGTTGTATAGG	0.021	0.007	0.013	10	764
CCTGTTACTGGCG	0.031	0.009	0.034	34	971
ACTAGTATTAGCG	0.023	0.004	0.025	44	1746
ACTATTGCATAGA	0.025	0.003	0.021	55	2580
CCTGGTACTTAGG	0.035	0.006	0.026	21	775
CGTGGTACTAGGG	0.027	0.008	0.037	16	415
AGTAGTGTTAGTG	0.026	0.007	0.017	13	749
ACTGGCATAGGTG	0.031	0.005	0.037	33	858
GGCGTTGCTTATA	0.059	0.003	0.058	64	1033
CGTATCGTAGGTA	0.025	0.005	0.026	31	1180
CCCATTGTTGAGG	0.025	0.001	0.027	37	1330
GGTATCACTAGGA	0.101	0.015	0.094	74	710
CCTGGTGTTGGCG	0.029	0.005	0.035	29	805
AGCAGTGTATGGA	0.028	0.004	0.031	43	1347
CGTGGTGTATAGG	0.033	0.004	0.038	19	485
CGCGGTACATACA	0.023	0.001	0.023	52	2171
AGTATTACAGACG	0.024	0.001	0.026	47	1767
CGTATCATTGGTG	0.029	0.007	0.021	18	853
CGCGGTACAGGTA	0.022	0.006	0.025	31	1234
GGTGTCGCTTAGA	0.08	0.015	0.091	49	492
AGCGGCGTTGAGA	0.023	0.009	0.019	18	942
GCTGTCACTTATA	0.055	0.006	0.047	60	1214
AGCATTACTGGTA	0.025	0.003	0.029	58	1927
CCCGGCGCATACA	0.024	0.002	0.025	66	2611
GCCGTTGTTGAGA	0.061	0.006	0.065	64	928
GGCAGTACAAACG	0.051	0.006	0.043	83	1829
GCCGTCGCAAGTA	0.049	0.009	0.059	66	1048
CGTGGTGTTTGCA	0.024	0.008	0.018	11	616
GCCGTCATTTATG	0.057	0.009	0.044	61	1315
AGCGTCATAAGGG	0.023	0.006	0.014	21	1439
AGCGTTATAAATA	0.022	0.002	0.025	111	4321
CGTATTGCATATA	0.022	0.005	0.026	42	1605
CCCGTTATATATG	0.024	0.003	0.022	51	2315
GGTATTGCAGATA	0.071	0.014	0.053	62	1099
CGTAGCATATATA	0.027	0.005	0.02	37	1771
AGTGTCATTGACG	0.026	0.005	0.024	27	1111
CGCGTTGTTAGTA	0.024	0.004	0.029	35	1152
ACCATCGTATGTG	0.022	0.002	0.023	39	1665
CGCATCGCATACA	0.025	0.003	0.022	57	2577
CCTATTGTTTGCA	0.028	0.003	0.032	57	1720

CGTATTGTATGTA	0.022	0.004	0.02	26	1300
GCCAGTATAGGCG	0.044	0.003	0.04	61	1466
GCTGGTGCTGGCA	0.141	0.013	0.134	80	515
CCTAGTACAGGCG	0.026	0.002	0.023	32	1334
AGTGTTGTAAGCG	0.022	0.005	0.018	16	882
AGTGTCACATACG	0.027	0.001	0.028	34	1179
CCTGTCACTTGGG	0.027	0.004	0.025	17	669
CGCATTACTAGGG	0.024	0.003	0.025	24	953
ACCGGCATAGAGA	0.021	0.001	0.019	42	2121
CGCGGTGTTGACA	0.021	0.002	0.021	27	1276
CGCGGCACTGACG	0.023	0.002	0.026	32	1209
ACCGTTGTATAGG	0.032	0.004	0.031	41	1267
GCCGGCATAAGCA	0.384	0.018	0.382	849	1375
CGTGGCATTAACA	0.021	0.004	0.024	32	1300
ACCGTCATTAGTA	0.021	0.001	0.021	55	2604
GCTATTATAAATG	0.066	0.005	0.06	171	2680
AGCAGCGCTTGGA	0.023	0.003	0.027	28	994
ACCGTTGCATGCG	0.021	0.004	0.026	42	1560
CCCGGTGCAGAGA	0.021	0.006	0.02	32	1539
GGCGTTATTGACG	0.065	0.014	0.055	56	957
GGCGGCATAGACG	0.548	0.07	0.508	778	754
CGTATCGCAGATG	0.035	0.004	0.038	37	929
AGCATTATTAGTA	0.025	0	0.025	83	3283
CGCGGTGTAAATG	0.02	0.004	0.02	25	1235
GCCGTCACAAGTA	0.056	0.005	0.061	120	1834
GCCGTCGTTAATG	0.059	0.001	0.061	59	905
ACCATCATAGACG	0.025	0.002	0.023	82	3539
CGCATCGTAGGTA	0.024	0.001	0.024	37	1521
GCTGGTGCAAAGG	0.272	0.021	0.275	210	554
AGCGTCGCTAGTA	0.018	0.001	0.019	25	1261
ACTGGCACTAAGG	0.027	0.001	0.027	30	1073
GCTAGTGTAAGGG	0.052	0.004	0.057	46	764
AGCATTGCTTACA	0.025	0.003	0.022	54	2428
CCTGGCACATGGG	0.022	0.009	0.021	15	713
CGTGTCGCATAGA	0.021	0.007	0.016	14	855
ACCATTGTAGGGA	0.021	0.003	0.018	33	1765
ACTGTCATTGACG	0.026	0.004	0.024	42	1682
ACCAGCGTTAACG	0.026	0.002	0.026	56	2119
AGCGTTGTTTGTA	0.022	0.001	0.022	29	1290
CCCATCGTAGACA	0.025	0.006	0.017	62	3484

CCCAGCGCAAAGG	0.029	0	0.029	50	1647
CGTGTCGTAAGCG	0.026	0.009	0.013	11	812
CCCGGCGTTGATG	0.022	0.003	0.026	30	1134
GCTGTTGTAGACG	0.065	0.006	0.063	57	841
CGCGGCACTAATA	0.027	0.003	0.024	47	1951
CGCGGCACTGGTA	0.021	0.003	0.017	18	1014
GCTAGTGCATGTG	0.056	0.006	0.052	40	734
GGCAGTGTTTGCG	0.058	0.006	0.058	47	768
GCCAGCATTGATG	0.057	0.009	0.045	73	1534
AGCGTCACATGCG	0.021	0.001	0.019	27	1389
CGTGGCATAAGTA	0.025	0.003	0.026	27	1027
CCTAGTGTATGGA	0.023	0.005	0.017	23	1297
AGCAGCGTAAGCA	0.024	0.001	0.022	52	2282
CGCGGTATATACA	0.024	0.003	0.02	46	2239
AGCATTATTTGGG	0.02	0.003	0.023	30	1293
GCTAGCACAAGTA	0.056	0.004	0.057	87	1447
CCCGGCGCAAGTA	0.029	0.001	0.03	63	2069
GGTGTTGTAGACG	0.085	0.008	0.078	45	535
AGTATTGCTAATG	0.023	0.004	0.029	43	1462
ACCATTGTTTGGA	0.027	0.002	0.029	48	1600
GGTGGTGCATGCA	0.077	0.008	0.086	52	550
GCCAGTACTGAGA	0.047	0.003	0.048	80	1602
CGTGTTACAAATG	0.024	0.001	0.022	32	1414
ACTAGTATTTGCG	0.03	0.003	0.029	43	1418
CGCGTTATATGTG	0.023	0.001	0.024	30	1214
GCTATTACTTATA	0.059	0.004	0.059	119	1900
CGCGGCACTAAGA	0.022	0.002	0.025	37	1464
AGTATCGTTTGGA	0.02	0.005	0.019	15	785
GGCGGTACTAAGG	0.085	0.014	0.07	57	757
GGCATCATTTGTA	0.074	0.007	0.071	114	1484
CCCAGTGTTTGGG	0.025	0.002	0.027	25	908
ACTGGCATAGACG	0.028	0.001	0.029	39	1295
GCTATTGTTGACA	0.058	0.014	0.046	83	1741
ACCGGCATAGGCG	0.026	0.006	0.024	31	1288
GGCATCGTTAGCG	0.121	0.014	0.106	107	900
CCCGGTACAAGTG	0.026	0.006	0.018	30	1673
AGTGGCACTAGGA	0.03	0.003	0.026	19	723
GCTGGTGTATAGA	0.393	0.035	0.399	386	581
GGTGTTGCAGAGG	0.076	0.001	0.076	31	378
AGTGGTGTTGGCA	0.02	0.005	0.022	15	669

CCTGTTACAAGCG	0.027	0.006	0.022	34	1493
GGCAGCGCTAGGG	0.05	0.018	0.024	13	533
CCTGGCGCTAAGA	0.026	0.003	0.03	33	1085
GCCAGCACTAGCG	0.053	0.003	0.056	76	1272
CGTGGCATTAGGA	0.022	0.008	0.014	9	644
ACTGTTACTTGTA	0.025	0.002	0.027	50	1808
CCCGGCGCAGATG	0.029	0.002	0.028	39	1372
ACTGTCACTAACG	0.03	0.002	0.027	54	1951
CGTGTCGTTGACA	0.026	0.005	0.022	24	1060
CCTGTTGCTGATG	0.03	0.011	0.02	21	1021
GGTGTTGCAAATG	0.069	0.011	0.055	43	744
GGCATCATTTGCG	0.083	0.009	0.087	103	1082
GGCGTCATTAACA	0.065	0.014	0.052	110	1994
AGCAGTATATAGA	0.024	0.003	0.023	73	3065
AGCATCACTGGGA	0.021	0.001	0.02	30	1468
GCCATCGTAGGCG	0.048	0.009	0.037	50	1285
ACTGTCATATAGA	0.027	0.008	0.017	36	2058
GGCAGTACTTGTG	0.06	0.014	0.048	41	819
AGTGTCATATAGA	0.025	0.002	0.028	40	1382
CGTAGCATTGATA	0.024	0.001	0.023	33	1399
ACTAGCGCTAAGA	0.021	0.002	0.02	43	2107
ACCGTCGTTGAGA	0.021	0.003	0.02	27	1329
CCCGTTGCAAACG	0.024	0.001	0.025	60	2299
CGTATCGTATACG	0.028	0.005	0.022	32	1411
CGTATCGCAAAGA	0.023	0.001	0.022	33	1486
AGCGGCGCATACA	0.022	0.003	0.024	43	1737
GCCGTTACAAGGA	0.054	0.008	0.05	67	1272
ACTATCGCTGGTA	0.025	0.004	0.019	33	1667
CCCGGCATTTGGG	0.025	0.004	0.025	20	796
ACCGTTGTTAACG	0.026	0.005	0.031	64	1997
AGTGTCACATGGG	0.026	0.007	0.033	18	533
CGCAGTATTAACG	0.025	0.001	0.025	54	2099
CGCATCACTAAGA	0.023	0.002	0.026	61	2293
ACCGTCGCTTAGG	0.02	0.004	0.025	27	1074
GGCGGTATTTACG	0.122	0.012	0.123	130	930
AGTAGTGCAGGTG	0.023	0.006	0.029	20	679
CCTATTACAGGGA	0.026	0.005	0.022	33	1500
AGTGGTACTGGTG	0.018	0.007	0.015	8	539
AGTGGCATAGGCA	0.027	0.007	0.029	19	636
GGCAGTACTAATG	0.06	0.008	0.063	93	1387

GGCAGTGCAGATG	0.049	0.004	0.043	39	868
GGTAGTACAGAGG	0.063	0.008	0.059	36	570
CGTAGTATAAGCA	0.023	0.003	0.019	31	1614
GGTAGTACAAACA	0.065	0.009	0.054	104	1811
CCTAGCACAGGCG	0.031	0.002	0.029	38	1293
CGCGGTATTGACG	0.022	0.012	0.015	17	1109
GGCAGCGCAAGTA	0.056	0.002	0.055	69	1186
CGCATTATTTAGA	0.025	0.003	0.025	54	2073
AGCATCACTTAGA	0.027	0.003	0.031	70	2206
CGCAGCGTATACG	0.024	0.007	0.017	25	1479
AGTATCATTTGTA	0.024	0.004	0.02	31	1541
GGTAGCACAAAGG	0.065	0.007	0.055	39	674
GCCGTTATAGGGA	0.064	0.006	0.059	54	860
ACTATTGTAGGTG	0.034	0.001	0.035	49	1363
GGCAGCATTAATG	0.058	0.003	0.054	88	1532
CCCATTACAGGGA	0.031	0.001	0.032	63	1894
CGTGGCGTATAGG	0.017	0.003	0.014	6	416
GGCGTTATTAGGG	0.053	0.01	0.045	29	611
GCCATTGCTTAGA	0.047	0.009	0.047	57	1148
CCTGGCATTAGCG	0.024	0.001	0.023	24	1022
GGTAGTATATATG	0.071	0.014	0.055	58	1000
CCCGGTGTAAGGA	0.023	0.003	0.018	25	1351
GCTATCGTTAAGA	0.052	0.004	0.047	65	1328
ACTATTACAGAGG	0.022	0.003	0.019	44	2263
GCTGGTGCATGGA	0.112	0.005	0.106	60	507
GCTGGTGTTTGTG	0.741	0.028	0.739	525	185
GGCGGCGCAAACA	0.094	0.019	0.076	124	1498
GGTATTATTTACG	0.114	0.024	0.097	116	1075
CCTAGCATAGAGG	0.036	0.006	0.033	44	1302
GCTGTTATAGACA	0.065	0.004	0.059	107	1696
AGCAGCACTGGTG	0.025	0.002	0.028	32	1109
CGTGGCGTATGGA	0.024	0.004	0.029	14	464
CCTGTTACTTACA	0.029	0.002	0.028	58	2008
GCCATTATTTGGA	0.06	0.006	0.068	125	1718
GGTGTCACTGATG	0.097	0.008	0.086	54	571
GGCGGCGTTAGGG	0.45	0.083	0.413	215	306
CGTGTTACTAATA	0.022	0.005	0.015	27	1723
AGTAGTATATGGA	0.027	0.003	0.031	42	1309
CGCATCGCAAATA	0.025	0.004	0.02	59	2918
GCTGTTACTTGTA	0.063	0.006	0.061	56	864

CCCGGTATTAATG	0.025	0.003	0.022	46	2049
CCTAGTGCTAGTG	0.026	0.004	0.028	31	1071
ACTATCGCAGACG	0.023	0.004	0.026	50	1842
CGCGGTATAGGTG	0.02	0.003	0.018	15	831
ACCGTTGCAGACG	0.022	0.002	0.022	46	2021
GGCGGCATTTATA	0.309	0.04	0.296	430	1025
CGCAGTGTTAATG	0.031	0.003	0.033	47	1364
CGTGGTACAAATG	0.024	0.008	0.025	27	1073
GCCATTACTTATG	0.047	0.003	0.048	84	1655
ACTATCATAGGTG	0.03	0.006	0.022	36	1606
CCCGGTACTTGTA	0.026	0.007	0.018	30	1641
CGCGTCACAGGCA	0.026	0.005	0.019	32	1625
CCTGTTGTATGTA	0.023	0.001	0.022	31	1400
CGTATTGCATGCG	0.025	0.011	0.015	13	850
CGCATTACAAGGA	0.025	0.005	0.028	50	1729
ACTGTTGCTTAGA	0.023	0.005	0.021	31	1422
GGTGTTGTATGTG	0.092	0.03	0.076	31	378
GCCGTCGTTTGGA	0.05	0.004	0.047	32	652
GCCATCACAGGCG	0.049	0.011	0.048	90	1786
CCCGTTGCTAGTA	0.027	0.001	0.028	48	1697
CGTATCGTTTAGA	0.023	0.001	0.023	25	1080
ACCGTCACTGGTG	0.029	0.008	0.028	40	1407
ACCGTTGCAGGTG	0.027	0.006	0.023	31	1300
AGCGTCGTTAGGA	0.017	0.004	0.021	22	1033
CGTGTCATAAGGA	0.02	0	0.02	24	1180
AGCGGTGCTAATG	0.02	0.002	0.02	23	1139
CGTGTCATTTGCA	0.019	0.006	0.01	11	1044
AGTAGTACTGAGG	0.024	0.004	0.024	20	823
CCCAGTGCATGCA	0.024	0.004	0.027	63	2309
CCTGTCACTAGTA	0.023	0.002	0.023	38	1608
ACTGGTGCAAGTA	0.026	0.001	0.024	42	1684
CGTGTTACAAAGG	0.024	0.005	0.019	21	1078
GCCATCACTGACG	0.061	0.006	0.067	120	1683
CGTGTCGTTAACG	0.024	0.003	0.024	23	951
GGCATCGCTAGTA	0.079	0.005	0.083	103	1142
GGCGGTGCATGCA	0.054	0.014	0.035	30	838
GCCAGTGCAAGTA	0.044	0.001	0.042	64	1458
AGTAGCACAGACA	0.026	0.002	0.028	53	1830
GGTAGTGCTAGTG	0.073	0.02	0.049	22	427
GGCGGCGTAGGTA	0.196	0.015	0.193	148	617

CGTGTTATTAGGA	0.026	0.001	0.026	22	840
CGCGTCGTAGACA	0.026	0.004	0.027	48	1707
CGCGGTATTTAGG	0.022	0.004	0.023	20	834
GGTAGTGCATATA	0.057	0.011	0.055	53	912
GCTATCACATATG	0.068	0.012	0.052	82	1495
CCCAGTGTAGGTG	0.022	0.005	0.029	36	1205
ACTGGTGTATATA	0.02	0.001	0.019	33	1733
CGCAGTATTTACG	0.022	0.006	0.017	29	1654
AGCAGTATAGATG	0.02	0.003	0.018	43	2334
GGCAGCACTAGTA	0.048	0.009	0.036	48	1298
CGTATTGCAGACG	0.029	0.011	0.022	25	1110
CGCGTCATTAAGG	0.025	0.005	0.025	31	1188
GGTAGCATAGAGG	0.064	0.008	0.064	41	599
GGCGGCATAGAGG	0.494	0.055	0.479	453	492
CCCGTCACTTGCG	0.028	0.004	0.022	32	1393
CGTATTATAGGGA	0.032	0.008	0.021	27	1285
AGTATCACTGGGA	0.026	0.002	0.028	24	839
AGTGGTGTATACA	0.024	0.001	0.023	32	1387
ACTAGTACAGGCG	0.024	0.001	0.023	38	1614
CCCGTTGTATAGA	0.021	0.005	0.022	38	1662
GGCGGTACAAGGG	0.064	0.017	0.041	28	659
ACTATCACTTGCG	0.024	0.007	0.017	32	1834
ACTGTTACAAGCA	0.025	0.007	0.016	47	2957
GCTATCACAAATG	0.065	0.002	0.066	121	1726
AGCATTGCAGAGA	0.021	0.005	0.019	36	1867
GCCATTACAGGGG	0.046	0.008	0.056	52	876
GCCATCGTATACG	0.053	0.009	0.044	81	1766
CGCATTACAGAGA	0.023	0.004	0.024	48	1943
ACCGGTATAAGCA	0.024	0.004	0.019	58	3054
GCCAGCACTGATG	0.056	0.005	0.052	68	1245
GGCGGTACATATG	0.07	0.02	0.044	41	899
GCCGTTATTGACA	0.056	0.007	0.065	148	2129
CGTGGCGCAAGTA	0.027	0.005	0.033	26	768
ACTGTCGCATATA	0.027	0.001	0.026	57	2110
AGTGTCATAAAGA	0.023	0.004	0.019	37	1898
GCCATTGTTGACG	0.068	0.005	0.071	105	1382
GCCGTCACATGCG	0.053	0.004	0.055	68	1179
AGCAGCATAGGTG	0.019	0.002	0.018	29	1577
CGCGTCATTTATA	0.024	0.005	0.021	42	1933
CGTGTTATATGTG	0.034	0.006	0.027	26	945

GGTGGTACTTGTA	0.072	0.019	0.046	27	565
ACTGTTATTAGTG	0.026	0.002	0.024	31	1256
GCTGGTATAAGCA	0.158	0.004	0.156	219	1186
CCCAGTGTAAGGG	0.024	0.003	0.021	29	1371
GGCGTCACAGGGG	0.06	0.015	0.051	36	669
GGCGTCACTAATA	0.059	0.007	0.055	101	1719
GGTGGTGTAAATA	0.103	0.011	0.088	101	1052
GGCAGTACTTAGG	0.051	0.005	0.044	35	753
CGTGTCGTAAGTA	0.026	0.002	0.025	30	1153
CCCGTCGCTAATG	0.021	0.001	0.023	39	1647
AGCAGCACAGGGA	0.018	0.004	0.021	30	1370
CCTGGTATTAACA	0.022	0.002	0.02	47	2330
ACTGGCGTTGATG	0.026	0.008	0.016	8	492
ACCGGCACTAAGA	0.027	0.002	0.028	81	2806
AGCGTTGCAGGGA	0.013	0.003	0.013	12	918
GCTAGTGCAGATG	0.056	0.011	0.069	62	841
ACCATTGCAAATG	0.025	0.002	0.022	82	3611
AGCGGTACATAGA	0.02	0.001	0.021	34	1607
GCTAGTGTTTATG	0.062	0.006	0.071	71	935
CCTAGTACAGAGG	0.028	0.004	0.033	45	1328
ACTGGCGCTGATG	0.025	0.005	0.028	21	720
CGTATTACTTAGG	0.03	0.003	0.027	25	891
AGCATTGCTTGCG	0.02	0.004	0.026	34	1299
CGTGTCGCTAGTA	0.03	0.006	0.028	23	786
CGCAGCACAGACG	0.021	0.004	0.019	34	1711
GCCAGTGTATACA	0.04	0.004	0.035	76	2073
CGCATTGTATATG	0.023	0.002	0.025	42	1623
GGCATTGTAGGCG	0.064	0.001	0.064	66	961
GGCGTCACTGAGA	0.064	0.007	0.055	63	1079
AGTAGTACAGGTA	0.024	0.005	0.018	24	1292
CCTAGTGCTTAGG	0.028	0.003	0.032	31	952
ACTGGCGCAGGTG	0.025	0.006	0.021	13	597
GCCATTGTTGAGG	0.059	0.01	0.048	45	891
CGTATTGTAAAGA	0.025	0.004	0.028	55	1877
CCCGGCACATGTG	0.031	0.001	0.03	43	1398
GCTGGTACTGGCA	0.082	0.008	0.072	59	762
CGCGTCGCTTAGA	0.025	0.006	0.029	30	998
GCCGTCGCTAATG	0.052	0.015	0.035	30	827
AGCGGCACTAACA	0.021	0.003	0.022	48	2149
GGCGGCATATGGA	0.284	0.034	0.263	261	730

ACCATTGCATACA	0.023	0.002	0.024	107	4373
CCCAGCATTAGTA	0.027	0.001	0.026	71	2693
CCTGTCGTTTAGA	0.02	0.003	0.022	26	1150
AGCGGCGCTAGTG	0.026	0.007	0.032	23	704
ACTATTGTTAACG	0.03	0.005	0.027	69	2483
AGTGTCGCATAGG	0.031	0.004	0.027	17	611
CCTAGCACATATG	0.026	0.001	0.024	42	1710
GGTGTTGTTTAGG	0.094	0.024	0.066	26	368
CGTGGTGTTAGGG	0.026	0.001	0.026	8	302
AGCATTACAGGTA	0.021	0.001	0.021	51	2360
AGCGGTGCTTAGG	0.018	0.004	0.014	10	690
ACTGGTATTTATG	0.022	0.001	0.021	26	1229
GCTGGTGCTGGGG	0.277	0.034	0.271	80	215
GCCGTTGTATGCG	0.06	0.005	0.067	57	800
AGTGTTATAGGCG	0.021	0.005	0.02	18	890
CCCGTCACAGGTA	0.028	0.003	0.027	58	2125
AGTATTGCAAGCG	0.02	0.003	0.02	24	1183
ACCGGTACAGGCA	0.023	0.001	0.021	60	2787
AGCGTCGTTAAGA	0.026	0.003	0.022	37	1619
CGTGTTGCTAGCA	0.02	0.006	0.027	25	895
AGTGTTGCTGATG	0.021	0.003	0.024	17	684
CGCAGCGTTGGGG	0.036	0.004	0.03	15	485
ACCGGCGTTTACA	0.026	0.005	0.019	31	1587
AGCGTCATTGGGG	0.022	0.005	0.022	22	977
AGTGGTATATGGG	0.03	0.009	0.04	19	457
CCCGGCGCTTGCG	0.025	0.002	0.025	27	1069
GGTATCGCTTATG	0.133	0.033	0.109	75	614
CGCATCACTTATG	0.027	0.009	0.035	60	1637
CGTAGCATAAACG	0.023	0.005	0.016	26	1582
GCCATTGCTGGCG	0.041	0.006	0.05	72	1364
AGCGGTGTTTACG	0.026	0.007	0.023	25	1080
GCTGTTACAAGTG	0.065	0.008	0.058	52	842
CCTATTGCTTAGG	0.029	0.009	0.018	22	1179
GGTGTTATATACA	0.08	0.005	0.074	105	1319
GCCGGCGCTGAGA	0.511	0.05	0.523	532	485
AGCGGTACTAGGG	0.021	0.005	0.014	11	787
GGCGTTATTTAGA	0.063	0.003	0.06	70	1095
AGCGGCATATGGA	0.023	0.008	0.011	11	972
AGCAGTGTATAGA	0.025	0.001	0.026	51	1931
GCTAGCATAGACG	0.06	0.009	0.062	83	1254

AGTGTCACAAGGA	0.019	0	0.018	19	1012
GCTAGCACTTGTG	0.062	0.01	0.06	44	690
ACCGTCGTTAATG	0.021	0.003	0.018	26	1418
AGCGTTACAGGTG	0.02	0.007	0.02	24	1194
AGCATTGCTGGCA	0.021	0.001	0.023	40	1707
AGTGGTGTATATG	0.028	0.008	0.019	14	705
GCCATTATTTAGG	0.053	0.002	0.055	87	1508
CGCGTTGCATAGG	0.014	0.002	0.011	10	874
GCCGGTATAGGGG	0.115	0.011	0.124	72	508
GCTGTCACATGCA	0.061	0.005	0.059	68	1075
CCCGTTGTTGGCA	0.019	0.002	0.017	25	1421
ACTAGTACTTATA	0.022	0.002	0.025	91	3503
GGTATCGCTAACG	0.138	0.016	0.134	120	778
GGTAGTGCTGACA	0.042	0.005	0.038	32	807
CGTATCGCAGAGA	0.021	0.002	0.024	25	1031
GGTGTTATTGGGG	0.105	0.006	0.099	33	302
GGCGGCGTTTGGG	0.44	0.06	0.405	167	245
AGCGGTGTTGATG	0.024	0	0.024	22	876
CGCATCACTTAGG	0.025	0.007	0.022	25	1096
AGCGTTGTTTGCA	0.016	0.005	0.021	27	1251
GCCGTTGTAGAGG	0.047	0.006	0.04	31	748
ACTGGTGTTTAGG	0.018	0.002	0.016	9	564
CCTATTGCTTACA	0.028	0.002	0.03	78	2537
GCTGGTATAAGTA	0.149	0.025	0.12	168	1230
CGCGGCGTTGAGA	0.021	0.004	0.022	19	845
ACTATCGCTGGCA	0.026	0.002	0.027	52	1890
GCTGTCGTAGACA	0.05	0.001	0.049	57	1108
ACCGGTGCTTGCG	0.024	0.002	0.022	23	1010
AGCGTTGCAAACG	0.021	0.006	0.019	38	1986
AGTAGTGCAGGGG	0.022	0.007	0.013	6	468
AGTGGTGTTTACG	0.016	0.007	0.008	5	587
AGCGTTACAAATG	0.019	0.002	0.02	46	2236
CCTGGTATATGTG	0.032	0.004	0.033	33	970
GCTATTGCTAGGG	0.057	0.007	0.048	29	574
AGCGTCGTATGTA	0.019	0.003	0.023	36	1539
CCTGGTATTGGTG	0.019	0.007	0.015	11	738
CGTGTTGTTGGTA	0.019	0.006	0.011	7	649
GGTATCATTTACA	0.116	0.004	0.114	168	1300
GGCGTCGCAAGGG	0.068	0.005	0.065	39	561
ACCATTACAAAGG	0.022	0.002	0.019	80	4029

GGTGTTGCTTGTA	0.072	0.013	0.054	26	453
ACCGGTGTTAGCA	0.026	0.002	0.026	45	1702
ACCAGTATTTGTG	0.026	0.006	0.018	38	2034
AGCGGCGCAAAGG	0.026	0.006	0.024	23	928
GCTGTCATTTATA	0.056	0.013	0.043	64	1426
CGCATTGCAGGCG	0.027	0.002	0.029	39	1303
ACCAGCATTTGCG	0.019	0.003	0.016	32	1976
GCTAGCACAGGGG	0.066	0.023	0.042	21	481
GGCAGCATTAGGG	0.047	0.005	0.039	38	927
ACTGTCGTAGATG	0.035	0.004	0.029	32	1076
GGCGTTACTGGGG	0.065	0.017	0.051	25	462
CGCAGTACAGGTG	0.029	0.012	0.014	15	1029
CCTATTATATAGA	0.023	0.002	0.025	80	3069
GCTGGTGCATAGG	0.272	0.026	0.238	133	427
GCTAGTGTTGAGG	0.076	0.007	0.067	46	637
GCCGGCATTTATG	0.733	0.049	0.74	1133	398
GCCAGTGTAGGGG	0.046	0.013	0.033	22	648
AGCAGCACTGGGA	0.02	0.003	0.016	19	1145
CGCGGCACAAGCG	0.022	0.003	0.021	25	1167
CGTAGTATTGACA	0.024	0.004	0.028	45	1554
CGTGTTACTGAGG	0.03	0.006	0.037	25	647
CGTGTTGTTTATA	0.021	0	0.022	23	1042
CCCGGCACTGGCA	0.022	0.002	0.025	42	1668
GCCGGTACAGATA	0.059	0.008	0.054	93	1615
AGTATTGCAAATG	0.024	0.007	0.015	25	1675
CGTGGCGTATGGG	0.031	0.018	0.023	8	345
CGTAGTATTGGTA	0.032	0.007	0.036	35	939
CCTGTCACTTATA	0.023	0	0.023	45	1923
CCCATTACTTATA	0.027	0.002	0.027	105	3730
AGTGTCGTTTAGA	0.022	0.004	0.019	14	737
GGCGGCGTTGACA	0.191	0.02	0.191	190	803
GCCGGCACAAATA	0.1	0.018	0.089	226	2316
CGCGTCGCTGGGA	0.026	0.018	0.013	10	763
AGCGTTATTAGCA	0.017	0.001	0.018	36	1997
AGTAGCACAAGCA	0.021	0.005	0.02	35	1687
AGCGTCATTTATG	0.02	0.004	0.019	28	1452
ACTGGTGCTAATG	0.02	0.001	0.019	23	1209
AGTAGCACTGATA	0.022	0.004	0.021	34	1560
AGTGGCACATAGA	0.024	0.001	0.023	24	1032
CGTATTGCTGGTG	0.021	0.005	0.015	10	655

GGTGGTGCTAGTA	0.079	0.015	0.078	42	496
AGTGGTATTTGTA	0.021	0.001	0.023	23	991
AGCGTCGCAAGCG	0.024	0.004	0.023	29	1247
CGTAGCACAGATG	0.026	0.008	0.027	26	955
GGCGTTGCATGTA	0.056	0.002	0.054	48	837
GGCGTTACATGCG	0.066	0.007	0.059	53	841
GGCGTTACAAGCA	0.063	0.008	0.074	115	1443
CCCGTCACTGGCA	0.028	0.006	0.019	39	1983
CGTATCATTTATA	0.024	0.004	0.021	45	2147
ACTGTTGCTAATG	0.022	0.004	0.022	37	1640
CGTATTGTTTACA	0.03	0.006	0.023	35	1492
AGTGGCGCATGCA	0.027	0.008	0.015	14	896
CCCATTGCTGGTA	0.026	0.001	0.025	46	1786
ACTGTTACATAGG	0.03	0.005	0.024	35	1416
ACCATCACAAGGG	0.025	0.003	0.027	51	1857
ACTGTTGTATAGA	0.026	0.003	0.03	47	1533
AGTGGCGTATAGA	0.021	0.001	0.021	14	661
CGCGTTATAAGTA	0.024	0.002	0.022	49	2201
AGCATTACTTACG	0.022	0.002	0.02	50	2404
GGTGGCACTTGGA	0.176	0.035	0.145	73	429
CGTGGCATTTGGA	0.032	0.013	0.018	9	481
ACCGTTGCTGACA	0.021	0.002	0.024	64	2643
CCCGGCGCTAGGG	0.021	0.004	0.016	13	785
GGCGGCGTATAGG	0.452	0.058	0.433	283	371
AGTGGCACTAAGA	0.023	0.001	0.024	26	1051
CGTGTCGCAGGCA	0.018	0.001	0.017	15	851
GCTGTTACTGGCG	0.047	0.01	0.033	26	754
CCTAGCGTTGAGA	0.031	0.003	0.034	45	1292
CGTGGCATAAACG	0.024	0.003	0.028	23	800
GCCGGCATAAAGA	0.384	0.039	0.384	864	1384
CCCGTCGTAGAGG	0.021	0.001	0.023	27	1163
AGTAGTGCTAGGA	0.021	0.009	0.02	15	753
CCTGGTGCTGGTA	0.024	0.003	0.02	18	882
ACTATCGCTGACG	0.028	0.009	0.021	37	1716
GCTGGCACTAAGA	0.221	0.021	0.219	259	926
AGTGTCATTTGGG	0.023	0.009	0.018	10	546
CGCGTTGCTTAGA	0.022	0.003	0.024	23	947
GGTGTCATTAAGG	0.1	0.007	0.095	55	523
AGTATCGTATATG	0.024	0.004	0.021	29	1362
GCTATCGCTGGCG	0.049	0.006	0.048	48	943

GGCGTCATATGTG	0.065	0.004	0.069	64	860
ACTGTCACTTATA	0.019	0.002	0.019	47	2364
GGCAGTATATATA	0.06	0.01	0.05	126	2399
CGCATCGCTTACG	0.024	0.006	0.029	44	1471
CCCATTATTTATG	0.027	0.004	0.024	69	2769
GCTAGTACTTGGA	0.058	0.007	0.06	55	868
CCCGTTGTTTACG	0.021	0.004	0.018	27	1512
CGTGTCATTTAGA	0.027	0.004	0.023	25	1056
GGTAGTGCTAGCA	0.056	0.001	0.056	40	675
CCTAGCGCTTGGA	0.021	0.002	0.023	25	1056
CGTGGCATAGGCG	0.032	0.001	0.031	14	441
ACTAGTGTAAGGA	0.026	0.003	0.024	43	1755
CGCATCACAAACG	0.026	0.002	0.029	89	2975
CCCGTCGTAGGCG	0.02	0.003	0.017	24	1353
CCTGGTATTGGCG	0.025	0.003	0.029	26	870
ACTAGTGCTTGTA	0.022	0.005	0.019	34	1790
GGTAGTGTAAGTG	0.059	0.004	0.059	41	650
AGCGTCACATGCA	0.022	0.004	0.026	51	1940
CCCGGTACAGGTG	0.027	0.006	0.034	42	1178
CCTGGCGCAAGTG	0.019	0.005	0.013	13	955
CGTGTTGCTGGCG	0.017	0.007	0.009	5	550
GGTATTGTTTAGG	0.114	0.014	0.096	52	492
CGTGTCACTAACA	0.033	0.008	0.027	44	1603
CGCAGTACAAATA	0.023	0.001	0.022	73	3227
GGTGTTATTGGCA	0.085	0.02	0.057	43	713
CGTGTCACTTACG	0.025	0.005	0.031	29	911
CCCAGCGTAGGGG	0.045	0.003	0.047	42	849
ACTAGTGCATGGG	0.024	0.003	0.026	28	1062
GCCGTTGTTAATA	0.048	0.007	0.041	69	1620
CGCGGTGCAAGCA	0.024	0.005	0.023	33	1391
AGTATCGTAAACA	0.017	0.003	0.018	49	2731
GGCGGCGTTAATA	0.218	0.026	0.22	246	871
AGTAGCGCAGGCA	0.023	0.003	0.027	26	950
GGTATTGCTTACG	0.104	0.008	0.093	73	712
AGTGTTATATACG	0.019	0.004	0.014	22	1538
CCTGGCACTGATG	0.027	0.005	0.021	24	1100
CGCGGTGTTAACG	0.019	0.006	0.021	23	1079
GGTGGTGTTAGCG	0.196	0.026	0.192	88	370
GGTAGTATAGAGG	0.062	0.009	0.05	38	715
GGTGTTGCTGGTG	0.08	0.015	0.062	19	286

GGTGGCGCATGTA	0.157	0.02	0.168	96	476
GCCAGCGCTGGCG	0.052	0.017	0.05	40	760
CGTATCATTGAGG	0.023	0.008	0.012	12	1016
CCCAGTGTTGATG	0.029	0.008	0.024	40	1629
CCCAGTGTAGGGA	0.031	0.005	0.025	32	1263
GCTGTCATTAGGA	0.055	0.007	0.052	46	838
CCTGGTGCATGTG	0.024	0.003	0.024	20	799
GCTGTCGTTGATG	0.061	0.005	0.054	32	558
GGTGTTACTGACA	0.082	0.009	0.08	88	1006
GCTGTTATAAACA	0.06	0.007	0.051	126	2369
CGTAGTATTTATG	0.026	0.005	0.021	22	1032
GCCATTACATAGG	0.054	0.007	0.063	99	1479
GGCAGCGTATAGG	0.058	0.014	0.048	38	761
CGTGGTACAGATG	0.026	0.005	0.02	16	780
CGCATCGTTGGTA	0.023	0.003	0.019	26	1370
GGTATTACTAACA	0.082	0.014	0.078	154	1812
GGCAGCACTAAGG	0.051	0.008	0.041	43	1018
GGTGTCACTTATG	0.109	0.013	0.097	61	567
ACTAGCACTGGCG	0.028	0.001	0.029	36	1197
ACTGTTGTTGATG	0.027	0.004	0.03	36	1181
CGTGTCATAAAGG	0.025	0.003	0.028	33	1129
GCCAGCGTAAAGA	0.062	0.013	0.045	78	1657
CGCAGCATTGGCG	0.032	0.012	0.025	28	1075
CCCGTCGCAGATA	0.024	0.003	0.027	66	2408
GGCGTCGTTTAGA	0.08	0.014	0.071	56	737
GCCGGCACAAGTA	0.101	0.017	0.091	158	1569
ACTGTCGTTAGCA	0.022	0.003	0.019	27	1375
CCTGTTACATGTG	0.025	0.004	0.019	23	1191
CCTGGTACATGGA	0.02	0.007	0.011	12	1047
AGTGGTGCTAATG	0.026	0.005	0.023	18	752
ACCATCATTAGTG	0.025	0.003	0.021	56	2591
CGCGTCGCTAGTA	0.027	0.005	0.02	24	1157
GCCAGCGCTTATG	0.053	0.004	0.048	43	850
GGTAGTATTTGTA	0.071	0.008	0.061	53	821
AGCGTCGTAGGCG	0.022	0.008	0.026	30	1128
GGCGTCGCTTGTA	0.059	0.009	0.059	46	736
CCTGGCGCTTAGA	0.024	0.005	0.017	16	922
CCCATCGTAAACG	0.024	0.001	0.025	89	3458
GCTAGTGCAGGTG	0.054	0.006	0.045	27	578
AGTGGCGTTTGTA	0.021	0.003	0.019	11	576

CGCAGTGCTTGGA	0.023	0.003	0.019	15	789
GCTGTCACTTGTA	0.044	0.01	0.05	43	823
AGCATTGTTGACG	0.026	0.005	0.03	56	1811
GGCGTCACTGATA	0.073	0.012	0.06	89	1397
ACTATCATTGGTA	0.028	0.004	0.024	50	2009
AGTAGCACAGGCA	0.03	0.004	0.024	28	1137
GCCGGCGCAGGCA	0.501	0.031	0.487	619	652
CCCATCGCAGGGG	0.041	0.001	0.042	45	1025
GCTGTCACAAGGG	0.056	0.009	0.048	32	638
GGCAGTACTAGCA	0.054	0.01	0.04	59	1404
CGCAGTATAAGCA	0.021	0.004	0.018	49	2745
GCTGTTGTTTATA	0.058	0.006	0.052	59	1071
GCCATTACTGGTA	0.064	0.005	0.057	91	1495
AGCGTCGTTTGCG	0.021	0.003	0.021	19	906
AGTGGTGTAAGCG	0.013	0.001	0.014	10	702
CCTGGTATAAATG	0.026	0.004	0.025	46	1815
GCCATCACTGACA	0.05	0.004	0.045	124	2614
GGTGTCATTAACA	0.093	0.01	0.083	111	1223
GCTATCGCAAGTA	0.055	0.005	0.061	77	1190
GGTAGTGTTTATG	0.074	0.013	0.058	36	586
AGCGTCACATATA	0.021	0.003	0.018	47	2625
GCTAGCATTTGCA	0.065	0.007	0.065	76	1093
GCTAGTACTGGCA	0.059	0.005	0.064	69	1010
CCTGTTGTTTATA	0.028	0.005	0.024	42	1693
CGCGTCACTTGCA	0.024	0.002	0.021	33	1511
AGTATTGCTTAGA	0.021	0.005	0.027	33	1182
ACCGTTGTAAGGA	0.025	0.006	0.03	50	1625
CGCGTCGCAAATA	0.022	0.002	0.019	44	2241
CGCGGCACTTACG	0.022	0.009	0.012	14	1119
GCTATCGTAGGGA	0.052	0.004	0.047	37	754
CCTATCATTGGGA	0.034	0.004	0.03	42	1339
CGCAGCACAGGTG	0.023	0.006	0.016	17	1058
CGCGTTACTAACA	0.022	0.002	0.023	61	2596
GCTGGCGCTGGCA	0.61	0.039	0.586	574	406
ACCAGTGCTAGCA	0.021	0.003	0.02	54	2651
CCCGGTGCTAGCG	0.024	0.003	0.028	30	1048
ACTAGCACAGAGG	0.027	0.001	0.028	37	1271
ACTAGCATTTAGG	0.027	0.003	0.026	34	1260
AGCGGCACATGGA	0.017	0.005	0.017	19	1094
AGCAGCACATGGG	0.022	0.007	0.017	18	1070

GCCGTTGCTGACA	0.047	0.005	0.043	55	1219
GGCATTGTTTACA	0.067	0.006	0.063	104	1554
CGCGGCGCAAGCA	0.02	0.004	0.02	29	1430
GCCGTCGCAAAGA	0.043	0.004	0.037	44	1145
GCCGTTGTTGGCG	0.043	0.001	0.044	42	904
ACTAGCGCAGGCA	0.022	0.002	0.019	40	2026
GGTGTTGCAAACA	0.072	0.019	0.047	59	1192
ACTAGCGTATATG	0.029	0.003	0.024	35	1411
ACCATCGTAGGGG	0.034	0.009	0.032	35	1075
GGTAGTGTATGGA	0.087	0.018	0.112	54	430
CGCGGCGCTTGTA	0.018	0.002	0.018	17	916
CCTGGCGTTGGGA	0.024	0.007	0.028	19	657
GCTGGTGTAGACA	0.416	0.029	0.413	547	777
GGTGGCATTAAGA	0.47	0.052	0.466	519	595
AGTGGCACAAGTG	0.024	0.007	0.024	14	574
GGTGTCATTAGCA	0.086	0.02	0.07	54	719
CGCGTCGTTGACA	0.024	0.006	0.018	27	1474
CGTAGCATAAGTA	0.024	0.006	0.02	32	1570
GGCATCATATAGG	0.086	0.008	0.075	104	1281
GGTGGTACATGGG	0.096	0.018	0.075	30	368
AGCGGTGCAGATG	0.023	0.005	0.021	22	1007
GCCGGCGCTAATA	0.477	0.046	0.485	807	857
CGTAGTGTAGGCA	0.021	0.01	0.012	9	756
ACCATTGTTTACA	0.026	0.004	0.026	84	3190
CGTATTATTAGGG	0.028	0.002	0.025	23	898
ACTGTTGCAAGCA	0.026	0.003	0.024	49	1992
GGTAGCGCTTGCG	0.058	0.01	0.05	23	436
GGTAGTGTTGGGG	0.074	0.014	0.072	20	259
CCCGTCGTTTGCG	0.023	0.005	0.026	30	1143
GGTGTCGTAGAGA	0.098	0.013	0.086	52	551
GGCGGTATTAGCG	0.109	0.018	0.095	81	769
CGTATTGTAAATG	0.033	0.002	0.031	51	1599
CCTGTCATTTAGG	0.018	0.006	0.018	19	1013
AGTGGCGTAAACA	0.024	0.003	0.022	27	1216
GGTATCACTTAGA	0.118	0.017	0.102	95	840
GGCAGCGCATAGG	0.062	0.018	0.06	47	737
GGTATCGCTAATA	0.107	0.019	0.091	121	1208
CGCGGCATAAGCA	0.026	0.002	0.028	47	1657
CGTAGTGCAAGCA	0.026	0.002	0.024	24	969
CGCGGTATTGGTA	0.025	0.003	0.023	26	1099

GCTATTATTTGCG	0.072	0.012	0.065	76	1086
ACTGGTGCTGAGA	0.021	0.006	0.02	23	1140
GCCATCACTTATG	0.054	0.002	0.053	95	1708
ACTATTGCAGGGA	0.024	0.004	0.021	34	1624
GGCATTGCTAGGG	0.08	0.003	0.083	58	638
GGTATCATTGGGA	0.111	0.025	0.08	60	688
AGCAGCATAGGTA	0.022	0.002	0.024	53	2132
ACCGGCGCTTGCG	0.026	0.001	0.027	26	953
GCTGGTATAAATG	0.455	0.059	0.414	645	914
GGCGTTGCTAACA	0.059	0.005	0.057	86	1415
GGCAGCACTGATA	0.047	0.009	0.036	57	1514
CCTGTTACTAGGA	0.026	0.003	0.027	34	1214
AGTATTGTATGGG	0.023	0.002	0.024	20	814
AGTGTTATAGGTA	0.023	0.003	0.019	25	1267
GGTAGTGTAAACG	0.058	0.008	0.065	63	909
GGTATCATTTGGG	0.12	0.03	0.116	53	405
GGTGTCACAAGTA	0.081	0.006	0.073	68	860
GCCATCGCTGACA	0.045	0.005	0.038	67	1700
GCTAGTATTTACG	0.061	0.01	0.073	100	1262
CGTATTACATAGA	0.024	0.001	0.023	41	1708
AGTATTGTTTGTA	0.018	0.003	0.014	19	1350
CGTGTTACAAGTG	0.02	0.002	0.021	20	940
AGCAGCATATAGG	0.019	0.003	0.019	35	1798
CCCGGCGCAGGTA	0.024	0.007	0.022	34	1485
AGCGTTATTGATG	0.021	0.002	0.019	30	1577
ACCAGCGCTTGTA	0.016	0.002	0.019	44	2313
GCCATCACAGGTG	0.048	0.004	0.049	67	1290
ACTATCGCATGTA	0.024	0.005	0.027	62	2233
GGCAGCGCATGTG	0.064	0.005	0.059	47	753
GGTATTATAAAGA	0.108	0.009	0.106	231	1947
CGCAGTACAGGTA	0.022	0	0.021	34	1550
CCTGTCATAGATA	0.024	0.004	0.023	54	2320
CGCATTGCTAACG	0.025	0.004	0.022	36	1619
AGTATTGTTGAGG	0.032	0.007	0.025	24	948
GGCATTGTAAATG	0.086	0.008	0.079	140	1635
ACTGTCGTAAGGA	0.023	0.001	0.021	24	1098
GGTATTATTTATG	0.113	0.012	0.105	119	1018
AGTGTTGCATGTA	0.024	0.006	0.019	19	989
CCCGGTGCAGGCG	0.026	0.005	0.019	22	1112
GCTGTCGTAGGCG	0.063	0.006	0.06	31	487

AGTATTACATAGG	0.024	0.007	0.026	36	1373
AGCGGTGCAGACG	0.026	0.007	0.017	19	1127
CGTGTCACTGGGG	0.029	0.006	0.03	13	423
AGCATCACAGAGA	0.025	0.004	0.021	53	2459
GCTATTACTTACA	0.062	0.004	0.058	122	1978
AGCGTCGCTGAGG	0.021	0.003	0.024	20	798
GCTAGCGCATATA	0.07	0.017	0.047	59	1199
ACTATCATAAGTA	0.026	0.001	0.027	87	3179
CGCAGCGTATATG	0.023	0.003	0.02	26	1297
ACTAGCGTTGGTG	0.028	0.009	0.039	28	698
ACTGTCGTAGACG	0.031	0.004	0.034	37	1049
GCTAGCACAAATG	0.056	0.005	0.055	79	1360
GGTGTTACTGAGG	0.084	0.02	0.067	35	485
GGTGTCATAAGGG	0.092	0.022	0.062	32	482
CCCGTCATTGGCA	0.023	0.003	0.02	42	2077
ACTATTATTTGGA	0.03	0.006	0.025	51	1953
GGTAGCACATATA	0.071	0.014	0.052	66	1210
ACCGTCATTGAGG	0.023	0.006	0.016	23	1453
GGTAGCGCTAGTA	0.085	0.019	0.063	41	609
CCTAGCACTAGCG	0.019	0.003	0.023	30	1288
GGCGTCGCTGATA	0.064	0.002	0.064	67	980
CGCGTCATTTGGG	0.03	0.013	0.012	8	677
CCCAGCGTTGGCG	0.024	0.005	0.021	30	1377
CCTAGCATATAGA	0.024	0.003	0.024	48	1969
GGTAGCGCTTGGG	0.076	0.012	0.067	20	280
CCTGTCATAGAGA	0.028	0.004	0.029	51	1728
CCCGGTATTGATG	0.021	0.002	0.021	33	1549
GCCGGTGTTTATG	0.397	0.038	0.39	394	617
ACCGTTATTAAGG	0.029	0.003	0.033	66	1918
AGTGGTACATACG	0.021	0.004	0.016	16	1013
GGCAGCACATGGA	0.051	0.006	0.045	43	917
CGTGGTGCAAAGG	0.027	0.007	0.033	21	621
CCCAGCATAAGGA	0.031	0.002	0.031	74	2338
CGCGTCATTAGGG	0.022	0.004	0.026	19	707
CGTAGTACATGTA	0.023	0.004	0.019	22	1135
AGCGTCATTGATA	0.026	0.002	0.028	68	2338
ACTGGTGCAAGGG	0.024	0.003	0.021	16	754
AGCAGCGTTAGGA	0.023	0.005	0.026	34	1295
CGTAGTATTAACA	0.025	0.002	0.022	47	2070
AGCGGCGTAAGGG	0.028	0	0.028	17	595

CCCGGCGCTGAGA	0.026	0.008	0.016	23	1382
ACTATCATTAGTA	0.025	0.003	0.024	64	2598
CCTAGTACTAAGG	0.029	0.003	0.026	37	1394
CGCGGCGTAGGTA	0.027	0.007	0.024	22	892
AGCAGTGCTAGCA	0.025	0.002	0.023	40	1697
AGCAGTGCTGGCA	0.015	0.003	0.012	18	1440
CCTGTCGTTAAGG	0.025	0.006	0.021	22	1019
GCCAGCGCTGGTG	0.059	0.011	0.074	43	542
GCTAGCACAAGCG	0.071	0.008	0.065	74	1065
AGCAGCGTTGAGA	0.023	0.005	0.027	39	1414
GGCGGCGCATACA	0.108	0.017	0.088	110	1136
GCCATCACATGGG	0.057	0.009	0.044	49	1058
ACTGTCGCTGGCA	0.018	0.002	0.019	24	1262
CGTAGTGCTTAGA	0.027	0.001	0.028	20	700
ACCGGCGCTTGTG	0.022	0.005	0.028	22	755
AGTAGCGTAGACG	0.028	0.006	0.034	36	1038
CCTGGCATTGATA	0.023	0.005	0.025	42	1652
GGCGGTACTTGGA	0.065	0.014	0.076	55	664
CGCATCGCTAAGA	0.031	0.002	0.032	56	1693
AGTATCACTAGCA	0.023	0.004	0.017	26	1493
CCCGTCATTTATG	0.022	0.006	0.014	26	1795
ACCATCACTTGTG	0.024	0.005	0.025	57	2251
GCCGTCGTAGGGA	0.05	0.013	0.035	25	690
GCCATTGTTTAGA	0.043	0.009	0.034	49	1393
GGTGTTACTTATA	0.076	0.007	0.073	78	988
GCTATCGCAAGGG	0.051	0.006	0.043	30	663
ACTAGCGTATGTA	0.027	0.01	0.014	22	1561
AGCAGCACAGATG	0.023	0.005	0.024	45	1865
AGTGTCGCAGGTA	0.02	0.003	0.023	18	769
GGCGGTGTTGGCG	0.077	0.012	0.093	49	477
CGTGTTACATGGG	0.022	0.005	0.018	11	605
AGCGGTATATATA	0.019	0.002	0.017	44	2570
GGTGTTGTAAGCG	0.079	0.011	0.064	38	555
GCTGTTGCAAACG	0.06	0.01	0.059	64	1015
GGCGTTGTTAGCG	0.066	0.008	0.054	37	650
CCCGGTGCTAGGG	0.023	0.004	0.021	15	713
AGCATTATTAGGG	0.027	0.003	0.022	35	1552
GGCATTATATGGG	0.068	0.014	0.054	59	1035
ACCGGTATAAGTA	0.022	0.005	0.016	47	2907
ACCATCGTTGGGG	0.022	0.009	0.034	32	907

CGTGTCGCTGATA	0.021	0.001	0.02	18	880
GCTGTTGCTGAGG	0.061	0.012	0.045	22	472
GCTAGTACTAAGA	0.063	0.005	0.057	88	1461
ACCGGCGCAAAGA	0.02	0.002	0.021	34	1589
CGTATCGTATATA	0.024	0.002	0.027	51	1812
GGCGGTGTATACG	0.083	0.017	0.07	64	855
ACTGGCACATAGA	0.027	0.005	0.031	51	1596
ACTATTGCAGGCA	0.019	0.003	0.016	38	2412
GCTAGCGTTTGTG	0.082	0.019	0.058	32	521
CGTATTATAAGTA	0.025	0.003	0.024	59	2379
GGTGTCGTTGGTG	0.085	0.029	0.045	11	232
GCCATCGCATGGG	0.045	0.006	0.05	37	702
ACCGGTGCTTGGG	0.026	0.007	0.018	13	716
GGCATTGCTTACA	0.058	0.004	0.056	86	1453
AGTGTCATTTACG	0.029	0.008	0.019	21	1074
GGTAGTATATAGA	0.073	0.01	0.069	88	1189
ACCGTCGCTGGCG	0.023	0.006	0.022	28	1226
CGTGTTGTATAGG	0.021	0.004	0.024	14	572
GGCGGTGCTTGCA	0.066	0.02	0.063	45	665
GGTAGCGTATGTG	0.081	0.004	0.085	41	442
CGCGGCATTGAGA	0.024	0.002	0.024	28	1121
ACCGGTATAAAGA	0.023	0.002	0.024	80	3254
GCTGTCGTTGGTG	0.058	0.011	0.059	24	380
ACTGGTGCATACG	0.024	0.007	0.015	20	1291
CCTGGTATAGGTG	0.029	0.004	0.029	25	830
AGTGTTGCTTAGG	0.02	0.005	0.02	11	541
CCCAGTGCAAGTG	0.026	0.003	0.023	39	1625
GGCGTTACTAGCG	0.062	0.005	0.068	56	762
CGCGTTACTGACA	0.022	0.005	0.014	31	2112
GCTGTTACTTGTG	0.054	0.005	0.049	32	619
AGCGTTGTTTGTG	0.026	0.004	0.022	19	854
ACTGGTATTAGGG	0.033	0.007	0.039	28	696
AGCGTCATAAGCA	0.022	0.001	0.022	60	2719
AGCGGCACTAGTG	0.02	0.001	0.018	14	744
GCCAGTGTAAATA	0.045	0.004	0.05	133	2526
CCTATCGCATACG	0.028	0.003	0.029	56	1869
GGTGTCACAGACA	0.079	0.013	0.063	70	1042
GGTGTCGCATGTG	0.099	0.021	0.07	31	410
ACCATTGCTGACG	0.028	0.002	0.026	64	2435
GCTAGTGTAAAGG	0.068	0.007	0.065	85	1225

CGCGGTGCTGGGG	0.025	0.009	0.013	6	474
CCCGGCATTGACA	0.026	0.002	0.025	69	2692
AGCATTGCATAGA	0.02	0.005	0.019	41	2149
AGTGGCGTTTGCG	0.031	0.013	0.029	8	272
ACTGTTGTTAGTA	0.029	0.008	0.018	25	1340
CGCAGTATAGACA	0.025	0.002	0.023	65	2782
GGCAGTATAGGGA	0.048	0.004	0.043	54	1208
GCTAGTATTTATG	0.058	0.012	0.049	65	1266
CCCGTCGTTGGGA	0.024	0.005	0.017	20	1139
CCTATTGCATGGA	0.028	0.013	0.021	33	1531
AGCATTGTTAGTA	0.023	0.003	0.027	57	2060
CCCATTGTTTAGA	0.026	0.003	0.023	44	1848
AGCGGTACAGATG	0.019	0.004	0.021	29	1370
CCTGTTACTAGTA	0.028	0.007	0.019	32	1628
CCCGGTGTAAAGG	0.026	0.002	0.024	34	1372
CGCGTCGCTAGGA	0.022	0.003	0.02	18	876
AGTATTATTTGTA	0.024	0.004	0.019	38	1913
GCTGGTGCTTATA	0.13	0.017	0.107	98	818
CGCATTACTTATG	0.026	0.004	0.021	34	1570
GCCAGTACTAGTG	0.05	0.006	0.047	57	1160
AGCAGTACAGGTG	0.021	0.003	0.025	36	1425
ACCATTATTAGCG	0.023	0.001	0.021	61	2838
CGTGTTACTAGCG	0.027	0.002	0.026	21	802
CGCATCGCAAACG	0.027	0.004	0.032	72	2182
AGCGGCGTAAAGA	0.019	0.003	0.021	33	1514
GGTGTTGCATGGG	0.092	0.014	0.11	38	309
AGCGTCATTGGCA	0.022	0.004	0.015	26	1659
AGTGTCACAAGTG	0.025	0.003	0.021	19	880
ACCATCGCAGAGA	0.023	0.002	0.023	64	2743
CCTGGTATAGAGA	0.02	0.008	0.011	17	1491
ACCGTCATTAAGG	0.026	0.006	0.021	42	1932
AGTGTTGCAAGGG	0.026	0.008	0.026	15	559
GCCGTCGCTGATG	0.049	0.006	0.057	40	662
AGTGTTACTGATG	0.026	0.005	0.024	22	907
GCTGGTGTTTACA	0.413	0.035	0.374	420	702
CGTGGTACATATG	0.021	0.007	0.011	10	864
CCCGGTGTTGGGG	0.026	0.006	0.029	17	575
ACTGGCATAAACG	0.027	0.002	0.026	51	1882
ACCGTCGTTAGTG	0.028	0.003	0.03	28	903
AGTATCACATATA	0.02	0.004	0.015	34	2187

CGCAGTGTATACG	0.033	0.004	0.033	49	1419
GCCGTTGCTGATA	0.034	0.004	0.034	39	1118
CGCGTTACATAGG	0.025	0.004	0.022	23	1013
GCCGGCACTTGGG	0.226	0.023	0.204	121	471
GCTGGTGCTGACA	0.114	0.008	0.103	89	772
GCTATTATTGACA	0.065	0.008	0.054	133	2309
ACTGGCGCATAGG	0.025	0.009	0.014	10	690
CGTGGCACAGACG	0.026	0.002	0.023	20	845
GGCAGTATAAATA	0.056	0.007	0.051	173	3249
ACCGTTATAGATG	0.022	0.002	0.019	44	2250
CCCAGCGTTTGTA	0.025	0.003	0.026	45	1697
GGCGTCGTATGCA	0.081	0.015	0.064	60	884
AGTGTTATTGGGG	0.028	0.003	0.031	17	523
GCTGGTATTTGGG	0.45	0.055	0.445	221	276
ACCGGCGCTAGGA	0.018	0.003	0.023	21	894
CCCAGCACTTAGA	0.025	0.003	0.021	49	2251
CGTATTGTAAGCG	0.026	0.003	0.022	25	1107
AGCGTCGCAAAGA	0.021	0.004	0.02	38	1828
CGCGGTGCATGCG	0.023	0.003	0.027	23	839
GCCAGCACAGAGG	0.058	0.012	0.041	43	996
AGCAGTGCATGTG	0.027	0.003	0.022	28	1219
ACCGGCACTAGGA	0.026	0.002	0.025	45	1766
GGTAGCGTTTACA	0.087	0.013	0.078	71	844
GCTGGCACTGATG	0.461	0.028	0.434	382	499
ACCAGCGCATAGA	0.023	0.002	0.02	57	2765
GCTATCACATACA	0.055	0.008	0.047	113	2267
GGCATTATAAGGG	0.054	0.003	0.055	66	1138
CGCAGTGCTAGGA	0.027	0.006	0.024	22	901
AGCGTCATATGCG	0.024	0.002	0.024	38	1520
GCTAGTGTAAATG	0.059	0.001	0.058	95	1533
AGTGTCGTTGGTG	0.025	0.009	0.027	15	542
AGTGGCACAAGTA	0.022	0	0.021	25	1144
GCTAGCGCTAGCG	0.065	0.008	0.054	31	540
ACCGTCACAGACG	0.02	0.003	0.016	39	2389
ACCATCGCAAGTG	0.025	0.002	0.027	57	2085
GCCATTGTTAGTA	0.056	0.002	0.054	92	1600
CGTGTTATAAGCA	0.022	0.002	0.022	35	1563
GCCGGTGCTGGCA	0.051	0.003	0.05	42	797
ACTAGTATTGGTA	0.028	0.002	0.031	60	1874
CGCGTCGTTAAGA	0.02	0.002	0.022	30	1343

GGTGTCACTTACA	0.078	0.011	0.067	65	899
GCCGGCGTAAACG	0.934	0.007	0.936	2280	155
GCTAGCGTAGGTA	0.07	0.003	0.071	60	788
CGCGTCGCTGGTA	0.024	0.004	0.029	28	937
ACCGGCACAAGCA	0.025	0.004	0.022	77	3387
CCTGGCACTTATA	0.028	0.004	0.029	49	1616
ACCGGTGTAGGGA	0.022	0.005	0.016	17	1044
GGTATTGTTTATA	0.109	0.013	0.094	114	1098
CGCAGCGTAGACG	0.026	0.003	0.027	39	1397
GCCGGCGTAGGCG	0.931	0.011	0.936	1275	87
GGTGTTATTAGTG	0.081	0.033	0.054	34	593
AGTAGCACTGGTG	0.022	0.003	0.022	15	675
CCTGGTGTAGATG	0.025	0.007	0.015	17	1115
ACTGGCACAAGCG	0.025	0.003	0.029	38	1283
GCTAGTATTGACG	0.056	0.005	0.05	65	1238
AGTGGCGCTTACA	0.02	0.006	0.015	14	925
ACTGGTGTAAACG	0.025	0.003	0.025	38	1496
CGCGGCACTGGGA	0.027	0.003	0.029	20	675
GCCGGCACATACA	0.104	0.003	0.1	215	1939
CCCGGCACTAGTG	0.025	0.005	0.02	27	1321
ACTATCATTAGCA	0.028	0.005	0.022	61	2733
AGCGGCACAAGCG	0.027	0.003	0.025	29	1121
CGTAGTGCTGATA	0.024	0.004	0.018	17	941
GGTATTGCAGGCG	0.081	0.023	0.073	51	649
AGCAGTGTAGGTG	0.019	0.001	0.02	23	1110
GCCGGTATAAAGG	0.159	0.012	0.16	205	1080
AGCGGCACAGGGA	0.019	0.002	0.016	15	939
CGCAGCGCATAGA	0.027	0.006	0.021	28	1303
AGCGGCATTTGGA	0.025	0.003	0.024	18	717
ACCGGCATATGCG	0.023	0.002	0.023	37	1555
AGCAGCATTTGTA	0.018	0.003	0.017	36	2029
GCTGTTACTTATA	0.063	0.006	0.054	75	1315
ACCGGTATTTATG	0.025	0.004	0.028	48	1646
ACTGGTGTTAAGG	0.02	0.003	0.022	18	812
ACCATTGTTTAGG	0.021	0.002	0.021	33	1520
GCTGTTGCTAACG	0.052	0.002	0.051	47	872
ACCGTTATATATG	0.022	0.004	0.022	56	2476
CCCGTTACAAAGG	0.024	0.005	0.018	38	2128
GCTGGTGCATATA	0.14	0.031	0.14	135	826
CCCGGTATATAGG	0.027	0.004	0.029	44	1497

CCCAGCGCTAGGG	0.023	0.005	0.023	24	1019
GGCAGCATTGATA	0.06	0.011	0.052	86	1564
AGCGTTGCATAGG	0.024	0.005	0.031	33	1026
CCTGTTGCTGGCA	0.023	0.002	0.025	30	1178
CGTGGTATTTGGG	0.029	0.009	0.019	7	361
CCTGTTATTGGCA	0.026	0.001	0.027	38	1363
GGTGTCGCATGGA	0.081	0.022	0.092	42	417
GGTATCACATATG	0.127	0.022	0.111	114	909
AGTGGCACATGCA	0.022	0.004	0.017	17	995
ACTGGCGTTAGGA	0.034	0.003	0.035	23	638
CCTGTTACAAATG	0.026	0.005	0.02	43	2149
GGCATTGTATATG	0.074	0.01	0.061	78	1191
GCTGTCACTGGGA	0.051	0.006	0.045	25	525
CGCGTTGCAGGCA	0.025	0.007	0.016	19	1160
ACCGTCATAAAGG	0.021	0.002	0.02	46	2258
GGCGTTACAGGTA	0.067	0.012	0.073	80	1015
ACTGTCGCTGACA	0.022	0.002	0.024	46	1851
CGCATTGTAGACA	0.024	0.004	0.025	61	2405
CGCATTACTGGTA	0.028	0.002	0.026	40	1507
CCTATTATTTGGA	0.027	0.005	0.02	35	1702
CGTAGCGTTTGTG	0.033	0.003	0.028	16	550
GCTGGCGTAAGTA	0.875	0.025	0.868	1444	219
ACCGTTACTGATA	0.023	0.002	0.02	74	3668
CGTAGTACATGGA	0.019	0.004	0.025	24	930
ACCAGCGCAGGTG	0.024	0.001	0.023	31	1304
AGCGGCGCTGAGA	0.024	0.008	0.012	12	1000
ACCGTCACAGAGG	0.024	0.004	0.018	31	1672
AGCATTGTATGGA	0.02	0.003	0.017	27	1550
GGTAGTGTAGGGA	0.048	0.008	0.039	23	561
GCTGTCGCTAGGA	0.048	0.009	0.042	21	478
CGTGTCGTATACA	0.02	0.002	0.022	31	1390
AGTGTTATTGACG	0.032	0.003	0.035	42	1150
ACCAGCGTAGAGA	0.026	0	0.026	49	1864
GCCGTCACAGATA	0.055	0.001	0.055	112	1923
CCCGGTGTTGGTA	0.026	0.002	0.027	28	1022
ACTGTCGCTTAGG	0.03	0.008	0.023	22	921
CGCGTCACTGATA	0.025	0.003	0.021	39	1792
GGTAGTACATATG	0.072	0.008	0.074	63	789
CCTATCATTAGGA	0.022	0.004	0.018	37	1996
GGCATTATTTACA	0.057	0.005	0.053	133	2382

AGTATCGTTAACG	0.022	0.005	0.016	26	1588
CGCGGTGTAAGCA	0.022	0.002	0.022	28	1265
ACCGGTGTATAGA	0.025	0.002	0.025	43	1681
CGCAGTGCTAGGG	0.021	0.01	0.007	4	585
CGTATTGTAAGGG	0.029	0.012	0.022	19	840
GCCGTTGCAGGTG	0.033	0.007	0.03	20	653
CGCGTCACAAGCA	0.025	0.006	0.019	42	2132
GCTAGTGCAAAGG	0.044	0.008	0.037	36	937
CGTAGCGCTTGTG	0.026	0.004	0.021	12	556
CCCGGTGCTAGTG	0.025	0.002	0.021	21	965
CGCAGTGTATATG	0.028	0.007	0.032	43	1285
GCCGTTACTTATG	0.045	0.006	0.037	42	1080
CCCAGTGCATGTG	0.03	0.005	0.026	36	1349
AGCGGTGCTGACA	0.017	0.001	0.018	28	1506
GGTAGCGTTAATG	0.077	0.01	0.073	57	721
CGTGTCACAGGGG	0.033	0.008	0.025	12	462
CCCAGTGTTTAGG	0.023	0.004	0.029	32	1062
GCTGTCACTTGCG	0.056	0.011	0.041	28	660
AGTATTACTTGGA	0.024	0.003	0.025	31	1223
GCTGGTATTAGCG	0.446	0.035	0.432	365	479
GCCGGCGTAGACG	0.927	0.017	0.914	1690	160
CGTATTACTTAGA	0.022	0.003	0.021	30	1383
AGCGGTACATATA	0.02	0.004	0.023	52	2248
GCCGGCACTGATA	0.106	0.009	0.101	157	1404
AGCATTACATGGG	0.02	0.004	0.015	22	1425
GGCAGTGTATACA	0.059	0.003	0.063	113	1690
ACTAGCGTTTGGA	0.026	0.006	0.032	31	931
ACTAGTGTATGGA	0.029	0.004	0.033	47	1393
CGCATTGTAAGTG	0.028	0.005	0.027	40	1421
CGTATTACTTGCG	0.021	0.006	0.013	13	958
GCCAGTATAAATG	0.051	0.005	0.045	131	2804
AGCATCGTTGGTA	0.027	0.002	0.026	43	1617
CGCGGTGTTTACG	0.028	0.009	0.02	17	818
CGCAGTGTTTGGA	0.021	0.009	0.015	12	810
AGCGTCATTTGTA	0.028	0.007	0.019	33	1705
ACTATTACTTGCG	0.025	0.002	0.025	50	1944
CCCAGCATTTGGG	0.033	0.006	0.025	26	1002
GGCATCGCATATA	0.085	0.012	0.071	120	1559
GGTGTTACAAATG	0.09	0.017	0.078	84	990
GCTAGTGCAAACA	0.058	0.007	0.059	124	1970

ACCGTCATAGGGG	0.035	0.004	0.034	31	891
AGCGTTGTTGAGA	0.024	0.009	0.036	47	1247
CCTGGTATAAGTG	0.029	0.004	0.029	38	1270
CGCGGCGTTTAGA	0.023	0.006	0.016	13	810
CGCGTTATAAGGG	0.024	0.006	0.017	17	974
AGTATTGTTAAGG	0.022	0.002	0.023	28	1165
AGCGGTATTTAGG	0.023	0.006	0.03	29	939
CCCATTACTTGGA	0.026	0.006	0.021	40	1872
GGCAGCGTTTATA	0.068	0.002	0.066	93	1312
CGTAGTACTTATA	0.024	0.002	0.025	34	1301
CGCGGTACAAGCA	0.025	0.005	0.017	31	1769
CGTGTCACAAGGA	0.023	0.004	0.017	16	924
CCTATCACTGGGA	0.033	0.007	0.04	56	1354
AGCAGCGTAGACA	0.021	0.001	0.019	49	2501
GCTATCGCATGGG	0.06	0.014	0.073	37	472
GCTGGCGCAAGCG	0.83	0.017	0.827	915	191
CGTGTTGCTGACA	0.021	0.005	0.022	24	1070
GGCATCATTGGGA	0.071	0.001	0.072	77	999
ACTATTGTTTGTA	0.026	0.004	0.021	39	1855
CGCGTCGCAGGTA	0.025	0.003	0.028	31	1080
GCCGTCACATGTG	0.056	0.006	0.049	54	1046
GCCAGCGCTTGTA	0.055	0.011	0.048	45	883
CCTGTTACTTACG	0.025	0.006	0.021	28	1318
GGTGGTATATGGG	0.295	0.068	0.29	131	321
CGCAGCGTTAACG	0.027	0.003	0.023	36	1534
CCTAGTGCTGGGA	0.025	0.001	0.025	24	946
CGCATCGTTTACA	0.024	0.003	0.021	45	2131
CGTATTATAAGTG	0.023	0.004	0.019	32	1630
CGTGTCACATAGA	0.025	0.002	0.024	25	1038
CGTAGCACAAAGA	0.027	0.003	0.03	48	1561
GCCGGTGTAGGTA	0.176	0.024	0.168	161	797
CCTGGTACTAGGA	0.025	0.005	0.021	21	978
GGTGGTACAAGCA	0.084	0.009	0.077	71	851
CGCGTTGTTGGGA	0.022	0.007	0.029	20	662
GCCGGTACTTGGG	0.083	0.01	0.092	56	554
GCCAGCACAGGTG	0.048	0.005	0.051	55	1021
GGCGGCATAGATA	0.291	0.058	0.25	394	1179
CGTATCACATGCA	0.018	0	0.018	28	1530
GCTGTTGCATATG	0.061	0.01	0.051	39	727
GGTATTGTAGGGA	0.092	0.035	0.057	41	680

CCCGGCGCTGATG	0.027	0.006	0.031	38	1169
AGCAGTGCATGTA	0.02	0.004	0.023	45	1874
CGTGTCGTAAACG	0.029	0.001	0.028	37	1264
GGCGTTATTGGCA	0.06	0.006	0.069	80	1085
CGCGGTATTGAGG	0.02	0.007	0.015	12	770
CGCATTATATGGA	0.03	0	0.029	55	1810
CCTAGCATTGATG	0.029	0.008	0.018	28	1500
ACTGTCGTAGGCG	0.027	0.009	0.015	12	781
GGCAGTATAGGCA	0.063	0.009	0.061	98	1516
GCCATTACATACG	0.054	0.005	0.05	117	2207
CGCATTGTAGGGG	0.037	0.014	0.028	22	769
GCTATTACAAAGA	0.055	0.003	0.059	140	2226
CCTATCGTTTACG	0.033	0.006	0.027	49	1761
GGCATCGCTAAGG	0.107	0.008	0.103	96	836
GGTATCATTAAGG	0.147	0.012	0.139	123	759
AGTATTGTAGGTG	0.021	0.002	0.021	21	974
ACTGGTGCTAACG	0.028	0.007	0.023	31	1305
AGTAGTGTTTAGA	0.026	0.004	0.025	27	1043
CGTATTGTATAGA	0.029	0.002	0.028	38	1325
CCCGGCGCAGGGG	0.028	0.011	0.019	13	679
GCTGTTACAAAGG	0.063	0.01	0.065	66	955
CCTAGCGCAGGTA	0.017	0.003	0.02	31	1493
AGCAGTGTTGGGA	0.017	0.002	0.015	15	990
GGCATTATTTGGG	0.063	0.014	0.044	38	817
CCCGGCGTTGAGG	0.027	0.001	0.029	24	818
GCTGTCGCTTACA	0.051	0.004	0.057	56	927
GCCAGCACTTGTG	0.056	0.006	0.063	64	946
GGCATCGTTAGGA	0.106	0.023	0.077	83	991
CGCAGTGCAAGGA	0.036	0.002	0.038	47	1182
CCTGTTGTAGAGG	0.027	0.007	0.033	34	991
ACCAGTATAGACG	0.022	0.002	0.024	78	3161
GGCGGCATAAACG	0.546	0.066	0.511	1139	1089
CCCGTTATATGCA	0.026	0.004	0.028	79	2706
CGTAGCGCATGCG	0.022	0.003	0.02	15	717
ACCATTACTTGCG	0.023	0.001	0.021	57	2624
CCCGTCACAGGGG	0.043	0.011	0.051	54	1015
ACTATCGTTTAGG	0.031	0.005	0.026	31	1173
GGTATCACAGATA	0.106	0.018	0.094	134	1284
AGTATCGCAAGGA	0.023	0.008	0.034	36	1035
GCTATTGTAAGCA	0.057	0.005	0.057	102	1694

AGTATTGTTTGCA	0.022	0.007	0.02	26	1307
AGTGGCATAGGCG	0.016	0.009	0.004	1	245
GGTGTCACAGACG	0.089	0.02	0.062	42	635
GCTAGCGCTAACA	0.059	0.005	0.052	68	1230
ACCGGCACTAGCA	0.023	0.002	0.025	73	2818
CCCAGTGCAAGGG	0.023	0.002	0.022	30	1319
GCTGTCACAGATA	0.063	0.007	0.054	72	1267
GGTATTATATACA	0.105	0.017	0.093	211	2070
ACTAGTGTATGGG	0.026	0.005	0.024	23	939
CGTGGTGCATGTG	0.023	0.008	0.021	11	516
AGCGTTATTAAGA	0.023	0.005	0.019	47	2454
GCTGTTACATGCA	0.053	0.007	0.046	57	1179
GCTGGCGTAAATG	0.947	0.011	0.935	1754	121
CCTGTTGTTAATG	0.025	0.003	0.026	42	1581
AGTGGCACAGACG	0.017	0.001	0.016	12	740
GCTAGCGCAAGGG	0.06	0.009	0.061	31	479
CGTAGTGCAGGGA	0.016	0.004	0.021	11	503
GGCGGTATAGGTA	0.091	0.011	0.089	89	912
GGTAGCACATGTG	0.068	0.014	0.05	30	573
CGCAGTGTAAGGG	0.027	0.006	0.025	22	856
CCCGTTGCTAGGG	0.026	0.007	0.018	18	960
ACCGTCGCAGGCA	0.025	0.002	0.023	46	1977
ACTAGCACATATG	0.025	0.004	0.029	63	2080
GGCGGTGCAGACG	0.05	0.005	0.048	38	752
GGTGGTACATGCA	0.086	0.003	0.082	62	691
GGTGTCATTAGGA	0.08	0.007	0.07	41	544
AGTAGCACATGCA	0.024	0.004	0.022	33	1446
GGTATTGCAGACG	0.091	0.014	0.107	90	754
ACCGTCACTTGGA	0.02	0.003	0.022	41	1849
CGCGTCACTAGCA	0.023	0.009	0.015	26	1743
ACCAGCGTTAGTG	0.026	0.003	0.022	27	1192
CGTATCGCTTACA	0.023	0.002	0.025	35	1374
GGTATCGCAGACA	0.112	0.017	0.089	103	1055
GCCGTTGTATAGG	0.051	0.009	0.057	48	787
GCTGGTGCTAGCA	0.122	0.012	0.134	102	659
CCCAGCGTATACG	0.021	0.002	0.019	42	2201
GGTATCGTATGGG	0.133	0.022	0.127	61	421
CCCGGCGCTTACG	0.024	0.003	0.024	33	1336
GGTGTTATAGATA	0.078	0.011	0.063	84	1247
AGCATCGCTTATG	0.026	0.002	0.023	39	1632

GCTGTTACAAACG	0.072	0.002	0.075	110	1351
CCCATCGCATAGG	0.03	0.008	0.02	32	1586
AGCGGTGTTGACG	0.022	0.005	0.019	21	1062
CGCATTGCTTATA	0.025	0.003	0.022	36	1633
GCCATTATAAAGG	0.053	0.004	0.05	123	2330
CCCAGCACAGAGG	0.028	0.004	0.033	54	1592
AGCATTGTATGGG	0.027	0.009	0.04	49	1188
CGTATCGCTTGGA	0.028	0.003	0.032	24	717
CCTGGTGTTAGTA	0.025	0.005	0.031	36	1121
CGCATTGCTTGGG	0.029	0.004	0.032	22	657
GGTAGTGTATGTG	0.063	0.007	0.063	34	505
AGCAGCACTTGGG	0.022	0.002	0.024	21	841
GGCGGTGCTGATG	0.068	0.008	0.056	34	571
GGTGGTACTGACA	0.075	0.016	0.059	52	826
ACCGGCACATGGG	0.021	0.005	0.013	15	1111
GGCAGCGTATATG	0.068	0.018	0.046	53	1096
ACCGTCACAGGGA	0.03	0.001	0.029	44	1451
AGCAGTGTTGAGA	0.019	0.002	0.018	29	1540
GGCGGCGTAAACG	0.441	0.072	0.402	570	849
GGTAGCGTATAGA	0.072	0.012	0.06	43	675
CGCAGCGCTTGTG	0.022	0.004	0.019	14	730
GGTAGCGTAAGCG	0.085	0.004	0.081	48	543
CCTGTTACATGGG	0.027	0.005	0.02	18	887
AGCGGTGTAGGCA	0.02	0.002	0.021	28	1279
AGTGTTGTTTGCG	0.025	0.003	0.028	17	597
CGTATCGTAGAGA	0.024	0.007	0.017	21	1221
AGCATTGTTTGGA	0.017	0.005	0.024	33	1340
GCTGTCGCTTGCA	0.057	0.009	0.057	38	632
CCTGTCGCATGTA	0.027	0.005	0.029	42	1406
GCTAGCGTAAACA	0.073	0.003	0.072	149	1907
AGTGGCACAGGCA	0.025	0.004	0.03	28	912
AGTAGTATTTACA	0.023	0.003	0.027	58	2080
GCCATTACTGGGG	0.06	0.012	0.058	46	748
CCCGTTGCTAAGA	0.024	0.006	0.024	45	1850
CGCGTTACTTGTG	0.028	0.007	0.019	17	891
GGTGTTATTGGGA	0.077	0.015	0.069	31	417
GCCGGTATTAACG	0.169	0.028	0.162	269	1396
AGCGTTGCAGGTA	0.019	0.004	0.014	18	1270
AGTATCGCTAATA	0.027	0.006	0.024	44	1789
CCCATCGTATATG	0.024	0.004	0.024	55	2230

GCCGTCGCTTACG	0.051	0.014	0.062	55	826
GGCATTGTTAGCA	0.074	0.007	0.085	121	1308
GCTAGCACTGACG	0.063	0.002	0.065	69	996
CCCATCGCAAGGA	0.024	0.002	0.024	54	2220
ACCGTCGCTTACG	0.022	0.001	0.022	38	1678
CGTGTTGCTGGTA	0.023	0.005	0.021	12	568
CGCATCGCTTGGG	0.029	0.007	0.026	18	673
AGCAGTGTTAACA	0.028	0.004	0.023	65	2802
CGTATCACTTAGA	0.026	0.003	0.024	28	1142
CCTGGCGCAGGTA	0.027	0.002	0.024	22	914
GCTGTCGCAGACG	0.064	0.02	0.089	61	625
GCCATTACTTACG	0.054	0.009	0.043	82	1808
ACTAGCGTTGACA	0.026	0.005	0.024	46	1857
GCTGGTGTTGGTA	0.436	0.051	0.441	296	375
AGCGTCGTTTGGG	0.024	0.004	0.017	11	621
GGCGGCGTAAGCA	0.201	0.017	0.201	228	908
GGCAGTATTGGCG	0.058	0.01	0.044	41	895
GGTGGTACTAAGG	0.111	0.024	0.101	52	463
GCTGTCATTTGTG	0.068	0.013	0.05	33	628
AGCAGCGCAAGCG	0.02	0.004	0.016	24	1453
GGCGTCGTATGGG	0.081	0.015	0.084	45	490
CGCATTACTTACA	0.023	0.003	0.026	67	2533
CGCAGTACTGATG	0.033	0.004	0.031	41	1287
AGCGTTACTGGGG	0.021	0.005	0.018	13	726
GCTGGTGTTGGGG	0.731	0.053	0.712	346	140
GCCATCGCTGATA	0.046	0.006	0.041	59	1396
AGCATCGTATATG	0.023	0.004	0.024	51	2067
GGCGGCATTGGGA	0.257	0.061	0.245	169	521
ACTGTCGCAGGTG	0.027	0.005	0.021	18	854
AGTATTGTATAGG	0.029	0.006	0.023	28	1164
AGTAGCACTTGCG	0.021	0.002	0.019	16	848
CGCAGTACTAGGG	0.027	0.004	0.028	22	778
CCTGGCACTGGTA	0.027	0.004	0.023	25	1084
CGCGGTGTTAGGG	0.026	0.004	0.02	11	526
GCCAGTGTTTGGG	0.045	0.002	0.046	28	577
ACTGGTACAGGGG	0.031	0.009	0.022	16	724
CCTGTTATTAGGA	0.03	0.003	0.027	34	1224
AGCATTGTAGGGA	0.023	0.002	0.022	35	1578
CCCGTCATATATA	0.029	0.002	0.027	94	3412
GGCATTATTGATG	0.061	0.007	0.051	79	1457

CGCATTGTTGACG	0.027	0.002	0.026	38	1433
GGTGTTACAGACG	0.085	0.009	0.075	61	747
GGTGTTATAGATG	0.094	0.022	0.067	54	752
CCTAGTGCATGCG	0.026	0.003	0.024	32	1293
ACTGGTGTTTGTA	0.028	0.007	0.026	25	955
AGCGTCGCTTAGA	0.019	0.003	0.018	21	1178
ACCGGTGTAAGGG	0.025	0.002	0.022	25	1099
GGCATTATTAGCA	0.057	0.005	0.051	97	1812
CCCGTCACTAGCG	0.025	0.002	0.027	49	1796
GGCGGTGCAAGCG	0.065	0.01	0.051	37	686
GCTGTCGCTTGTA	0.058	0.006	0.062	33	503
CGTGGTATTTAGA	0.019	0.009	0.008	7	846
GGCAGTGCTTGCA	0.053	0.017	0.051	46	856
GCTATTACTTGGG	0.063	0.007	0.058	42	680
AGCAGCATAAGCA	0.02	0.002	0.018	58	3100
GCTATCGTTAACA	0.059	0.004	0.054	102	1801
CCTATCGCATATG	0.024	0.004	0.018	32	1707
AGCATTGCTGAGG	0.023	0.006	0.022	30	1318
GCCGTCATTGACG	0.076	0.019	0.049	69	1337
AGTGGTGCATATA	0.024	0.008	0.036	43	1167
CCTGGCGCATATA	0.023	0.005	0.017	25	1484
GCCGGTACATATG	0.079	0.008	0.069	90	1211
GGCAGCGTTAGGG	0.061	0.011	0.058	32	524
CGCAGCATAAATA	0.024	0.001	0.023	83	3478
ACCGTTGTATGGG	0.028	0.005	0.033	33	965
CGTGTTATTGGTG	0.03	0.015	0.009	6	647
CGCAGCACTTATA	0.024	0.004	0.02	40	1980
GGCAGTGCTTGGA	0.053	0.008	0.053	35	626
GGTAGTATAGGCA	0.072	0.023	0.045	46	975
ACCATCATAAAGG	0.027	0.001	0.026	91	3380
GCTGTCGTTAATA	0.066	0.017	0.046	57	1181
CCTGTCGCAGGTA	0.022	0.003	0.026	31	1166
GGCATCGTATATG	0.12	0.011	0.111	154	1238
GCTAGTATTAAGG	0.056	0.002	0.057	67	1108
CCCGTCATATGCG	0.02	0.004	0.015	28	1852
CGCAGCGCTGGCG	0.023	0.008	0.011	12	1059
AGTATTACAAATG	0.023	0.002	0.02	49	2415
CGCGTCACATGTA	0.023	0.003	0.022	37	1679
ACCATCGCAGAGG	0.027	0.004	0.023	36	1549
ACCGGCACTTAGA	0.025	0.004	0.021	45	2063

AGTGGTATAGGTG	0.019	0.008	0.011	7	625
GCTAGCACTAGTG	0.066	0.005	0.074	55	692
CCTGGTGCAGGTG	0.017	0.003	0.016	11	690
AGTAGTACTGACG	0.027	0.003	0.025	31	1198
CGCAGTGCATATA	0.025	0.008	0.018	32	1744
ACCAGCATTAGCA	0.025	0	0.025	84	3320
AGTATCGTTGGTA	0.028	0.007	0.023	23	979
GGCAGCGTTAGCA	0.065	0.01	0.057	66	1090
AGTGGTGCAAGGA	0.022	0.003	0.026	19	723
AGTGGCACTGGCG	0.028	0.003	0.023	10	421
CGCAGTGTTGGGG	0.026	0.008	0.019	9	463
AGCAGTGTAAGCG	0.02	0.001	0.022	37	1676
CCTGTCGTAAGCG	0.022	0.003	0.019	24	1242
CGTGTTATTAACG	0.029	0.006	0.02	27	1325
AGTGTCATTGGGA	0.015	0.002	0.013	9	681
GGCAGCACAAGGA	0.051	0.005	0.045	54	1140
GCTAGTGCTTACA	0.053	0.007	0.049	68	1325
GGTGGCACAAGCG	0.406	0.06	0.378	304	501
ACCAGCGCAGGGA	0.022	0.002	0.02	31	1528
CCCGTCACTTGGG	0.024	0.005	0.018	17	945
AGTATCACAGATG	0.025	0.009	0.026	33	1253
CGTATCGTTAGCA	0.024	0.003	0.029	39	1315
GGCAGCACTAACA	0.048	0.004	0.054	119	2090
GCCAGTGCAGAGG	0.042	0.001	0.041	36	846
GCTAGCATTGACG	0.068	0.007	0.074	84	1044
AGTGTTATTAGTA	0.022	0.004	0.022	33	1484
GGCGTTGCAAGTA	0.057	0.014	0.044	55	1186
CGTGTCACTGGCG	0.024	0.005	0.027	20	712
GCCAGCGCTTGGG	0.048	0.011	0.043	23	511
AGTAGTATATGTG	0.023	0.001	0.024	30	1197
CCTGTTATTAATA	0.024	0.005	0.018	51	2834
CGTAGCGTTAGCG	0.022	0.012	0.04	26	628
GGTGGTGCTGAGA	0.067	0.018	0.045	20	426
ACTGGTGCTGATA	0.028	0.005	0.022	37	1640
ACTGGTGCATAGG	0.03	0.008	0.021	18	852
CGCAGCGCAGATG	0.037	0.006	0.039	46	1130
CGTAGTGCATACA	0.03	0.003	0.026	33	1225
GGCATTATAAATA	0.059	0.006	0.052	201	3691
GGTAGTGTTAACA	0.061	0.004	0.065	75	1079
ACCGGTATTGGCA	0.03	0.006	0.029	60	1979

CGTATCGCAGAGG	0.03	0.014	0.019	15	764
GCTGTTATTGAGA	0.055	0.009	0.044	50	1077
CGCATTATTAGTG	0.023	0.006	0.02	31	1529
GGTATCGTAAGGA	0.148	0.041	0.127	103	705
AGTATCATTGAGA	0.026	0.005	0.025	41	1583
GGTAGTACAGGCA	0.059	0.014	0.043	34	756
ACTAGCATTGGTG	0.032	0.004	0.036	43	1143
CCTGGCGTAGATA	0.028	0.005	0.022	37	1668
CGTGTCGTTGATA	0.023	0.004	0.018	19	1015
CGTATTGTTGGTG	0.022	0.006	0.02	13	641
GCTGGCATATACA	0.662	0.049	0.651	1400	751
GGCGGCACAAATA	0.128	0.019	0.131	263	1742
ACCGGTGTTAGTA	0.025	0.005	0.022	33	1441
GCTGGTGTTAACG	0.741	0.037	0.743	933	322
ACCGTTATTTGGG	0.019	0.004	0.014	16	1113
ACTAGTGTAGGCA	0.026	0.004	0.025	46	1794
CCCGTTGCTGGCA	0.024	0.002	0.021	35	1595
GGCGTCACTTGCA	0.071	0.006	0.062	73	1109
ACTAGCACTAGCG	0.021	0.002	0.018	31	1689
GCTATTATTGATG	0.073	0.004	0.068	109	1500
CCCGGTATAGGTA	0.022	0.005	0.015	28	1786
GCTGTCACTGACG	0.054	0.005	0.052	50	905
CGCGTCGCATACA	0.023	0.003	0.021	41	1893
GCTATCACTTACA	0.057	0.002	0.058	112	1824
GGTGGTACAGGGA	0.09	0.015	0.081	40	453
CCCATCGTTGGGA	0.025	0.002	0.028	37	1301
GCTAGCATATGGG	0.064	0.004	0.059	40	638
CCCATCGCTAGGA	0.025	0.002	0.027	57	2059
GGTGGTACTGGGA	0.07	0.007	0.065	27	388
GCCATTACATGGG	0.052	0.009	0.041	46	1085
CGCGGCGTTGAGG	0.022	0.013	0.009	5	527
GGTAGTGCATACG	0.046	0.004	0.045	34	729
GCCGGTGTTAATG	0.412	0.063	0.384	446	716
CGCATTGCAAGTA	0.021	0.001	0.023	46	1956
AGTATCATAGACG	0.026	0.002	0.024	42	1678
GGCAGCGTTAATG	0.061	0.007	0.052	55	1006
AGTGTTGTATATG	0.025	0.003	0.024	24	962
AGCAGCGTAGGCG	0.019	0.004	0.02	26	1269
AGCATTACAAGCG	0.022	0.001	0.021	53	2459
GCTGTTGTTGGGA	0.058	0.005	0.061	29	446

GCTATCATTTAGA	0.062	0.009	0.056	86	1457
CGTATTACATACG	0.029	0.006	0.021	35	1669
AGCGGCATTTATG	0.02	0.005	0.021	13	607
ACCAGCGCTTGGA	0.025	0.002	0.027	48	1761
GGCGTTATTGGGG	0.068	0.015	0.048	26	513
CGTGGTGTAGAGA	0.02	0.002	0.018	13	703
GCTGTTGCATATA	0.047	0.006	0.039	43	1067
ACTGTCATTTGGA	0.026	0.002	0.023	26	1087
CGTGTCACTGACG	0.023	0.009	0.021	19	899
AGCATTGCATGCA	0.021	0	0.021	47	2220
GCTGGTACAAAGA	0.079	0.005	0.083	112	1242
ACCGTCGCAGACG	0.025	0.002	0.025	46	1798
GCTAGTACAGAGG	0.068	0.014	0.061	55	846
AGCAGTACTTGCA	0.022	0.007	0.016	33	2068
CGCAGTATTAGTG	0.021	0.002	0.019	24	1231
ACTGGTGTATGTA	0.029	0.003	0.028	36	1243
GGCGTCATTGGGA	0.071	0.022	0.045	35	743
GCCAGCACTTACG	0.051	0.002	0.052	84	1539
CCTAGCACTGAGG	0.028	0.002	0.026	27	1021
CGCAGCGTAAAGA	0.027	0.001	0.026	50	1864
GCCGGTGTTTATA	0.191	0.033	0.175	220	1039
AGTGGCGTAGAGA	0.024	0.006	0.023	16	689
GGCGGCACTTAGG	0.264	0.045	0.233	170	561
CGTAGTACTGATG	0.02	0.003	0.024	21	854
GCTGTTGCATGTA	0.062	0.009	0.056	46	777
CCCATTACATAGG	0.022	0.001	0.022	50	2190
GGTGTCGCTTAGG	0.114	0.027	0.094	34	329
CGCATCACTAGCA	0.024	0	0.023	55	2307
CCTAGTATTGACG	0.025	0.002	0.027	48	1752
AGTAGCACTAAGG	0.022	0.006	0.014	14	961
CGCGTCACTGGTG	0.022	0.004	0.017	14	810
GGTAGCATTGACG	0.058	0.008	0.058	38	615
AGCGGCGCTGATG	0.02	0.003	0.019	16	836
CCCATCACTTGCA	0.024	0.002	0.022	64	2897
GGCAGTATTGAGA	0.055	0.009	0.05	73	1400
ACCATTGTTGGGA	0.023	0.005	0.017	25	1449
CGCATCGTTAGCA	0.023	0.003	0.02	36	1803
GGCATTGTTAGGA	0.065	0.011	0.069	67	904
GGTGGTGCAGGCG	0.081	0.029	0.06	21	331
GGTAGTGCATATG	0.058	0.003	0.053	35	627

GGCAGTATAGACG	0.052	0.007	0.043	68	1529
AGCGGCACAAATG	0.021	0.004	0.016	25	1548
CGCGGCGCAAATA	0.023	0.004	0.018	35	1938
GCTGTTGTTGACA	0.053	0.01	0.043	47	1053
CCTATCACAAGCA	0.026	0.003	0.024	87	3538
ACTGTTGCAGAGG	0.021	0	0.021	22	1005
GCTATTGTTAAGA	0.059	0.002	0.06	96	1498
AGTATCACTAGGG	0.027	0.001	0.028	22	775
CGTAGTATAAAGG	0.024	0.005	0.021	25	1167
CCTATTATATATG	0.026	0.003	0.026	79	2927
GGCAGCATTAAGA	0.052	0.006	0.051	87	1628
AGTGTTACTGACG	0.02	0.004	0.015	16	1024
GGTATTATTAACA	0.097	0.014	0.081	186	2105
CGTATTACAGATG	0.031	0.004	0.029	42	1384
CCTATCGTTAAGA	0.027	0.009	0.021	45	2054
GCCGGTACTGGCA	0.06	0.01	0.05	58	1104
AGTATTGCTTAGG	0.017	0.003	0.014	11	795
CGCGTTGCAAGGA	0.025	0.006	0.018	21	1119
GCCGGCACTTACG	0.203	0.02	0.193	231	964
AGCAGCATAGGCG	0.023	0.006	0.017	28	1655
GGTGTCATAAACA	0.084	0.012	0.075	121	1496
CCTGTCATATAGG	0.028	0.007	0.035	48	1309
GCTGGCATTGGTA	0.635	0.037	0.617	690	428
AGCAGCACTTGCG	0.022	0.003	0.023	31	1311
ACCGTTGCAGACA	0.021	0.003	0.025	76	2986
CCCGTCGTTTAGG	0.02	0.01	0.033	33	964
CGTAGTGTAGGTG	0.029	0.008	0.018	8	445
ACCGTTGTTAGGA	0.023	0.004	0.029	38	1288
GCCGTTGCTTATG	0.051	0.003	0.054	45	785
GGCAGCGTAAGTA	0.064	0.012	0.056	79	1328
CCTGTTGTTTGCA	0.029	0.001	0.032	41	1259
AGTAGTATAGGGA	0.015	0.002	0.015	18	1183
CGTGGCGTTAGTA	0.026	0.008	0.029	18	605
GGCGTCATAGGGA	0.06	0.01	0.052	55	1001
CGTATTGCTGATG	0.025	0.004	0.019	18	926
GGTATTGTAAGCA	0.094	0.012	0.08	97	1123
GGTGGCGTTTGCG	0.651	0.069	0.615	343	215
ACTATTGTAGACA	0.03	0.004	0.027	90	3215
ACCAGTGTATGGG	0.03	0.003	0.029	33	1095
ACTGGCATAAGTA	0.028	0.004	0.026	52	1957

CGTAGCGCTTGTA	0.034	0.01	0.038	29	727
CCCGTTGTTGGTA	0.029	0.006	0.03	38	1214
GCCAGTATTAAGA	0.055	0.005	0.053	133	2377
ACTGTCACTAGCG	0.025	0.003	0.03	38	1238
ACCGTCGCTGGTG	0.025	0.008	0.013	13	989
CGCATCGTTTATA	0.025	0.003	0.021	41	1940
GCCGTTGCTTAGA	0.048	0.005	0.055	47	807
GGTGTTATAAAGA	0.088	0.012	0.077	100	1203
AGTGTTACTAACG	0.027	0.005	0.033	46	1354
GCTAGTGTAGGCA	0.052	0.005	0.045	51	1074
CCTGGCACAAAGA	0.031	0.004	0.033	63	1875
CCTAGCATAAGGG	0.041	0.003	0.038	46	1180
GGCGGCACAAGGG	0.291	0.028	0.289	231	569
CGCGGCGCAGATG	0.028	0.003	0.026	26	963
GGTATTACAGGTG	0.093	0.005	0.087	63	660
GGTATTGTAAATA	0.107	0.016	0.094	180	1736
GCCGGTGCATGGA	0.063	0.01	0.077	51	612
GGCATTATTGACG	0.063	0.006	0.062	97	1474
GCCAGTATAAGTG	0.049	0.004	0.05	93	1761
GGTGGCACTGGGA	0.191	0.039	0.171	74	358
CCTGGTGTAGGGA	0.027	0.004	0.023	19	800
GCCATTGCTAGTA	0.044	0.005	0.04	51	1223
CGCAGCGTATGTA	0.024	0.003	0.02	27	1357
CCTGGCATTTAGG	0.028	0.006	0.019	16	806
CCCGTTGCTGGTA	0.021	0.004	0.026	36	1371
GGTGGCACTAACA	0.192	0.019	0.183	222	988
CGTAGTGTAAACG	0.025	0.007	0.017	19	1110
ACTAGTGTATACG	0.031	0.003	0.026	53	1967
GCCGGTGCAAATG	0.116	0.012	0.113	133	1049
GCTATCGTTAGTG	0.046	0.009	0.033	30	881
ACTATTGTTGAGA	0.028	0.001	0.028	55	1926
GGCATCATTTACA	0.088	0.009	0.079	181	2124
CCTGGTACTAAGA	0.025	0.003	0.021	33	1537
GGCGTTGCTTAGG	0.077	0.003	0.075	42	515
GCTATTATTGGTA	0.051	0.007	0.041	61	1437
GGCAGCACTTGGA	0.047	0.002	0.049	42	816
GGCGTTATTTGGG	0.049	0.005	0.042	24	545
GGTAGTGCAGACG	0.062	0.012	0.047	32	647
AGTATTACAGAGA	0.028	0.001	0.029	54	1802
AGTGTCATTTGCA	0.021	0.007	0.021	24	1135

AGCGGCGTTGGGA	0.023	0.005	0.03	20	642
GCTGGCATATATG	0.867	0.03	0.878	1557	216
CGTGGCACTTATG	0.026	0.007	0.031	19	597
GGCAGTGTATATG	0.051	0.006	0.057	63	1050
GGTAGCGCAAAGA	0.078	0.011	0.073	66	839
GCCAGTGCAAATG	0.049	0.005	0.045	70	1474
GCCGGTGCAAGGG	0.094	0.013	0.076	45	550
AGTAGTGTAAGCA	0.02	0.003	0.019	29	1518
AGTATTGCAAACG	0.024	0.002	0.022	38	1697
GGTGTCATTGACA	0.095	0.016	0.074	78	972
GCTAGTACTTATG	0.058	0.005	0.065	65	936
AGCGGCGTAGGTG	0.025	0.006	0.021	12	551
CCTAGCGTTAGCG	0.027	0.007	0.025	26	1032
CCCGTTGCAAGGG	0.026	0.007	0.03	37	1201
CGTAGTGTTTACA	0.028	0.003	0.024	25	1023
CGCAGCGTTTGTG	0.026	0.004	0.026	21	788
GCCAGTGTTGGGA	0.041	0.014	0.027	22	791
AGTGGTGCAGATG	0.024	0.007	0.014	10	683
GGCATTGCTAACA	0.055	0.005	0.048	95	1872
CGTGTCGCAGAGG	0.032	0.009	0.041	24	565
GGCGGTGTAGGGG	0.063	0.022	0.061	29	448
GCTAGTGCTTACG	0.057	0.011	0.044	42	923
ACTGGCGCAAGCA	0.025	0.004	0.022	21	946
AGCATCGCTGACA	0.023	0.002	0.021	51	2412
CGCAGCGCAGGCG	0.027	0.008	0.02	22	1078
GCCAGCGCTAGGA	0.046	0.007	0.036	32	845
GCCGTCGCATGTA	0.057	0	0.057	51	851
CCTGGCATAGATG	0.021	0.001	0.02	27	1347
CGTGTTATTAGCG	0.025	0.003	0.029	26	857
GCTGGCATAAGCA	0.633	0.028	0.628	1234	730
CGTAGCGCATGTA	0.025	0.004	0.021	19	894
GGTGTTACAGGCA	0.084	0.017	0.06	53	828
AGCGTCGCATGGA	0.026	0.007	0.025	26	1012
AGCGTCATTTACG	0.022	0.003	0.024	44	1800
AGTGTCGTAAATA	0.022	0.002	0.02	38	1893
GCTGTCATTGGCA	0.062	0.01	0.052	53	973
AGTAGTGCTGATG	0.024	0.006	0.028	24	831
AGTATCACAGGCG	0.029	0.004	0.024	26	1048
ACCGTCGTAAGTG	0.03	0.004	0.026	33	1223
AGTGGTGTTGGGA	0.028	0.008	0.039	18	443

GGCATTATTTATA	0.063	0.006	0.056	139	2359
CCTGTTATAAAGA	0.024	0.002	0.022	59	2642
AGCAGCGCTGGTA	0.028	0.004	0.031	35	1102
CCCAGCATTGGTG	0.025	0.004	0.025	34	1312
CCTGTCGCAAACA	0.026	0.007	0.025	68	2693
GGCGGCATAAAGA	0.296	0.058	0.26	447	1274
CCTGGCATTAACA	0.024	0.002	0.023	50	2172
CCCGGCACTAACG	0.029	0.003	0.032	78	2352
GGCGTCACAAGGA	0.069	0.012	0.052	58	1056
CCTATTGTAGGGG	0.041	0.002	0.04	38	920
GGTGTCACAAGGG	0.1	0.03	0.08	38	435
ACCGTTATTTAGG	0.026	0.004	0.022	34	1540
AGCGTTGCAAATA	0.023	0.002	0.022	60	2633
GGTATTGCTAACA	0.079	0.01	0.067	101	1407
AGCGTCGTTTAGG	0.026	0.008	0.018	15	823
GCTATCGTAGAGA	0.048	0.011	0.039	47	1160
GGTGGTGTATGGA	0.105	0.036	0.059	26	415
GCCAGCATATGCG	0.062	0.006	0.055	91	1551
GCTATTGCAAAGG	0.045	0.006	0.053	65	1157
GCCGGCACAGGCG	0.202	0.005	0.198	209	847
CGTGGTATTAGTA	0.018	0.002	0.021	21	959
CCTATCACTAGCG	0.029	0.005	0.022	43	1871
GGCATCGTAGGGG	0.071	0.009	0.064	55	810
GCCATTGCAGGTG	0.057	0.002	0.056	49	830
AGTGTCATAAGGG	0.02	0.004	0.015	12	774
AGTGGTGTAGATG	0.021	0.008	0.009	7	741
GGTGGTGCTGACA	0.075	0.019	0.064	40	585
GGTAGTATTTACG	0.059	0.011	0.065	58	838
GCTAGTGCAAATG	0.058	0.007	0.068	89	1227
GCTATTGCAAGGA	0.044	0.009	0.031	36	1126
CCTGGTGCAAAGG	0.024	0.002	0.022	21	922
AGTATCGCTGGGG	0.021	0.002	0.02	10	489
CGCAGCATTAAGG	0.029	0.004	0.03	39	1270
GGTGGCATTAACG	0.721	0.045	0.691	966	432
CCTATCGCTGACA	0.025	0.004	0.02	47	2312
GGCATTACAGAGG	0.065	0.006	0.062	70	1065
AGTGGTACTGATG	0.036	0.01	0.027	22	807
GCTGTTACTGAGG	0.067	0.009	0.064	44	647
CGCATTGCTAGCA	0.022	0.007	0.021	34	1576
CGTGGCGCAAAGG	0.019	0.004	0.013	8	616

CCTGGCACTGACG	0.027	0.008	0.016	21	1287
AGCGGTGCTTGTG	0.021	0.008	0.029	19	645
CGTAGTGTAAGTG	0.02	0.003	0.017	13	745
CGCAGCATAGACG	0.028	0.003	0.028	51	1785
CGTAGCGTTAGGG	0.03	0.003	0.032	15	460
ACCATTGCTTGGG	0.027	0.006	0.019	25	1260
CGTGTCACATACA	0.025	0.006	0.022	37	1676
ACTGTCGTAAATG	0.031	0.005	0.037	55	1436
GGTAGTACAAATA	0.065	0.011	0.052	92	1672
CGTATCGTAAACA	0.024	0.003	0.02	53	2610
AGTGGTGCTTAGA	0.027	0.004	0.021	14	658
GGCGGCACTAGCA	0.143	0.02	0.14	165	1015
CGCGTTGCTAGTA	0.029	0.001	0.029	34	1141
GGCAGTGTAAGTA	0.052	0.004	0.057	83	1371
GCCGGCACAGGCA	0.109	0.011	0.108	145	1201
GCTATCACATACG	0.064	0.007	0.056	92	1563
CGCGGTATTTGTG	0.032	0.005	0.037	31	802
AGTGTTGTTTGGA	0.015	0.004	0.021	14	652
GGTGGCACTTATA	0.204	0.039	0.188	156	676
ACTAGCATAGGCG	0.023	0.002	0.025	36	1423
CGCAGCGCAAGTG	0.021	0.002	0.02	23	1123
CGCGGCGTTTGTG	0.03	0.006	0.022	12	530
CCCGTTGTAAGCG	0.025	0.003	0.028	46	1621
AGCGTCGTAGAGG	0.022	0.001	0.024	24	987
CCCAGCGCTAGTG	0.026	0.005	0.023	32	1342
ACTGTCACTAAGG	0.03	0.008	0.021	29	1330
AGTAGCATTTGGG	0.023	0.006	0.014	9	621
CCTGGCACAGGGA	0.041	0.007	0.031	31	956
GGCAGCGCTAATG	0.058	0.006	0.065	72	1031
CCTGGTATTTGTG	0.017	0.007	0.013	11	854
CCCGTTGCATGTA	0.025	0.007	0.026	47	1796
GCTGTCACTGATA	0.049	0.01	0.04	53	1257
CCTATCGTATGCA	0.027	0.001	0.028	60	2057
AGTGGTATAAAGG	0.024	0.006	0.022	19	843
AGCAGTGCTTGGA	0.02	0.002	0.023	25	1071
GCTATTGCTTACA	0.052	0.005	0.05	80	1529
CGTAGCGCTTGGA	0.024	0.009	0.024	13	532
GCCATTGTTGATG	0.049	0.007	0.059	74	1190
CGTAGCGTTAGTG	0.024	0.005	0.027	16	586
CCCGTCGTAGATA	0.024	0.002	0.024	60	2392

GGTGTTACTAACA	0.066	0.019	0.049	69	1349
GCCGGTGTTGGCG	0.302	0.06	0.266	214	591
CCTGTCGCTGGGA	0.027	0.008	0.017	14	822
AGTGTTGTATACA	0.023	0.005	0.026	42	1588
GCTGTCACTAGTG	0.06	0.018	0.043	32	719
CGCATCGCTGAGG	0.024	0.004	0.02	19	941
GCTAGTACATGCG	0.057	0.009	0.058	60	969
CGTGGCGCATAGA	0.023	0.004	0.018	12	667
GGTGTCATAAGTG	0.089	0.01	0.078	53	623
AGCATCGCATGCA	0.026	0.002	0.025	54	2140
ACTGGTACAGAGA	0.021	0.001	0.019	36	1812
AGTGTCATAGGCA	0.024	0.003	0.02	25	1201
ACCGTCACAAGGG	0.024	0.001	0.022	31	1347
AGTGTTGTTGGCA	0.016	0.002	0.017	14	823
GCCGTCGCAGGCA	0.05	0.009	0.05	52	987
AGCATTACATGTG	0.02	0.005	0.013	26	1970
ACTAGCGCATAGA	0.025	0.001	0.023	47	1964
CCCAGTACTTGCG	0.022	0.001	0.023	40	1708
CCTGTTGTAAAGG	0.027	0.005	0.029	40	1319
CCCGGTATTAGGG	0.027	0.003	0.031	32	999
ACTGGTACTAGCG	0.027	0.002	0.028	34	1196
GGCATCACATGTG	0.076	0.01	0.076	96	1175
CCTAGCGCTAGGG	0.02	0.002	0.019	12	627
GGCATTATATGTG	0.06	0.009	0.048	68	1344
GGTAGCGTATACG	0.089	0.012	0.082	65	728
GCTATCGTATGTA	0.053	0.002	0.056	75	1269
GGTGTTATTTGGA	0.083	0.014	0.068	40	550
CCTGTCGCAAAGG	0.025	0.003	0.026	29	1091
GCCATTACATGTA	0.055	0.006	0.047	107	2151
GGCAGTGTAGGGA	0.039	0.005	0.045	41	875
ACCGGTGTTTAGA	0.031	0.003	0.027	37	1345
CGTATCGCTGGGG	0.024	0.004	0.029	13	436
AGTATCATTGGTA	0.025	0.003	0.026	38	1411
GCTATCGTTAGCA	0.049	0.004	0.044	57	1226
CGTGGTATTGGCA	0.017	0.007	0.011	8	735
CCCGGCATTGGTA	0.03	0.003	0.027	42	1534
AGTGTTGCAAACG	0.027	0.005	0.024	28	1132
CGTGGTACTAATG	0.026	0.003	0.023	21	900
AGTATCACATAGA	0.023	0.001	0.024	38	1555
GGTGGTGTATACA	0.111	0.032	0.107	88	737

AGTAGCATTAGCG	0.028	0.01	0.021	21	999
GCTGGTATTGGGG	0.435	0.049	0.401	157	235
ACTAGTACTGGCA	0.024	0.001	0.023	51	2185
GCCGGTACAGACA	0.061	0.007	0.059	118	1880
CGTAGCGTTAAGA	0.026	0.009	0.037	35	914
GCTGTCACTTGCA	0.053	0.004	0.056	52	883
ACCGGTGTTTGCG	0.028	0.01	0.033	32	935
GGCAGCACTTATG	0.049	0.006	0.047	54	1094
CGTATTACTAGTG	0.022	0.007	0.012	13	1055
ACTGTCATAGAGG	0.029	0.006	0.026	30	1117
GCCGGCACTGATG	0.199	0.018	0.174	202	957
CGTATCACATGCG	0.025	0.004	0.02	22	1074
GGTGTCATTTGGG	0.095	0.03	0.065	23	330
CCTGTCATTTGTG	0.028	0.004	0.022	23	1006
CGTGTTGTTGGTG	0.023	0.009	0.033	15	446
CCCGTCATAAGTG	0.028	0.001	0.027	52	1908
CGTGTCACATGTG	0.025	0.002	0.022	17	759
GGTGGTACAAATA	0.077	0.012	0.064	91	1323
GGTGTCACATGCG	0.097	0.019	0.074	42	529
GCTAGTACAGAGA	0.06	0.005	0.065	85	1213
ACCGTCATAAGTA	0.027	0.005	0.022	72	3203
GCCATTGCAAACA	0.047	0.001	0.048	137	2700
GCTAGTGCATGCG	0.045	0.003	0.041	32	740
GGTGGTGTATATA	0.109	0.023	0.087	68	716
ACTATCGTTAACA	0.028	0.002	0.029	90	2990
GCCAGTACTAGTA	0.05	0.004	0.054	96	1694
GGTATCATATGTG	0.129	0.01	0.114	92	713
ACCGTCACTAGTG	0.027	0.006	0.036	63	1677
GGCATTGTTGGGG	0.069	0.006	0.062	34	518
CCCGGTACTAAGG	0.026	0.002	0.025	37	1470
AGCGTTGTTGATA	0.024	0.003	0.021	35	1659
ACTGGCACTGGTG	0.039	0.005	0.037	30	780
GCCATCATAAGTG	0.058	0.007	0.056	122	2070
CCTGTCATTAGTA	0.03	0.004	0.024	41	1636
GGCGGTGTATACA	0.071	0.013	0.067	86	1195
ACTATCGCAGGTA	0.025	0.005	0.019	38	1967
CCTGGCGCAGGCA	0.028	0.004	0.031	37	1168
GCCGGCGCATACG	0.744	0.027	0.746	1162	396
ACTAGTATTAACG	0.026	0.003	0.023	59	2526
GCCGGTGTTAGGA	0.191	0.015	0.17	135	661

CGTAGCATTAAGG	0.023	0.001	0.023	23	961
GGCGTTGCTTGGA	0.055	0.014	0.072	41	525
GGCGTCATATGGA	0.062	0.012	0.05	51	962
CCTGGTGCTGGCA	0.028	0.005	0.031	34	1072
GGTAGTACATGCA	0.07	0.006	0.062	61	916
AGTAGCACATGGA	0.021	0.007	0.016	16	984
CCCAGCGTATGGA	0.024	0.001	0.025	42	1618
AGCGTTACTAGTG	0.019	0.004	0.021	27	1266
ACTGGCGTTAGCA	0.025	0.006	0.016	16	964
CGCGTTGTAGGTA	0.022	0.002	0.023	24	1042
GGCATTGTATACA	0.074	0.019	0.065	138	1976
CGCAGTGCTGGGA	0.026	0.006	0.033	25	744
AGTATCGCTTGTG	0.021	0.008	0.019	15	775
AGTAGCGTTGAGG	0.025	0.008	0.018	12	648
GCTAGTGTTGGTG	0.074	0.021	0.053	37	667
GGTGGTATTGATG	0.283	0.034	0.263	182	509
CGTGGCGTTAATA	0.025	0	0.024	24	959
ACTGGCACTTACG	0.026	0.004	0.021	26	1191
ACCGGCACTTAGG	0.023	0.002	0.021	27	1275
AGCATTATATGGG	0.025	0.003	0.023	38	1604
AGCAGTACTTAGG	0.02	0.004	0.025	34	1328
AGTAGTGTTGGTG	0.024	0.006	0.027	15	531
CGTGGTGCTTATA	0.023	0.006	0.016	13	822
GGCATTGTATGTA	0.067	0.003	0.07	100	1330
GGTATCGCTTGCA	0.101	0.014	0.089	67	683
ACTGGCACAGGCG	0.028	0.006	0.026	25	952
CCTAGTGCAAGCG	0.025	0	0.025	38	1488
CCCATTGCAGGGA	0.027	0.004	0.023	39	1632
AGTAGCGCTAGGG	0.028	0.013	0.042	21	483
GGTATTGCTAGCG	0.097	0.027	0.072	46	589
CGTGGCATTGGTG	0.029	0.018	0.003	1	325
AGCGGTATTGGCG	0.027	0.006	0.026	21	786
CCTATTATATATA	0.025	0.002	0.026	115	4311
AGCGTTACTGAGG	0.02	0.005	0.017	18	1067
AGCGTCACTAGTG	0.025	0.007	0.029	35	1165
GCTGGTATTAGTA	0.169	0.013	0.175	184	868
ACCATCATTGGGA	0.024	0.002	0.022	40	1803
GCCGTTGCAGATA	0.046	0.011	0.04	55	1334
CGTATTACTGGTA	0.023	0.005	0.019	23	1177
CCTAGTATTTGCG	0.032	0.004	0.037	47	1233

**CCTATTGTTAAGA**	0.026	0.001	0.028	70	2411
**GCCGTCACTAACA**	0.054	0.002	0.05	131	2472
**CCCGGTACAAACG**	0.025	0.002	0.027	75	2686
**AGCGGCATTGACG**	0.021	0.008	0.012	8	685
**GGCAGCATTGAGA**	0.053	0.005	0.047	61	1236
**CCTATTGTAGGCG**	0.024	0.002	0.027	42	1513
**AGTATTGCTAGTA**	0.024	0.003	0.021	27	1251
**AGTATCATTTACA**	0.023	0.002	0.022	52	2287
**CGTGGTACAAGCG**	0.024	0.004	0.029	21	715
**GCCGGTACAGGGG**	0.063	0.005	0.069	39	529
**ACCGGTACTGGTG**	0.02	0.004	0.014	21	1475
**CGCATCGTTAAGG**	0.028	0.001	0.027	37	1327
**GGTAGCACAGATG**	0.065	0.003	0.06	43	668
**GCTGGTACTTACA**	0.077	0.007	0.079	95	1105
**ACTAGTGTAAATA**	0.024	0.002	0.025	99	3897
**ACCGTTATTAGGA**	0.024	0.005	0.024	45	1799
**CGCGTTACTGGGA**	0.026	0.003	0.021	19	870
**ACCGGCACAGGTG**	0.027	0.007	0.02	29	1432
**GGCGGCGCAGGCA**	0.096	0.009	0.09	74	749
**GCCATCATTTGTG**	0.056	0.006	0.063	98	1453
**GGCATCACTTGTG**	0.086	0.018	0.074	78	974
**ACTGTCGCTAGTA**	0.02	0.004	0.014	20	1379
**CCCGTCATAGGTG**	0.038	0.004	0.033	45	1337
**AGCATCGTTTAGG**	0.021	0.002	0.019	25	1266
**GCCGTTGTTGGGA**	0.04	0.009	0.028	20	688
**GCTGGTATATATG**	0.439	0.04	0.426	519	699
**AGTAGTACAAACA**	0.022	0.002	0.019	57	3000
**ACCGGTGTAGAGA**	0.019	0.002	0.019	30	1589
**CCTAGCGCATGCA**	0.023	0.003	0.02	41	1995
**GCTGGCACTTATG**	0.449	0.029	0.451	405	493
**AGTGTCACTGAGA**	0.022	0.007	0.024	25	1024
**GGCAGTATAAACG**	0.054	0.009	0.042	93	2141
**GGTAGTATATATA**	0.074	0.015	0.055	90	1554
**GCCATTGTAGATG**	0.048	0.006	0.044	69	1494
**CGCAGCACAAGTG**	0.021	0.002	0.018	27	1489
**CGCAGCGTTTGGA**	0.021	0.003	0.021	18	833
**GGTGTTACTTGGA**	0.077	0.015	0.055	29	494
**CGTGGCGCTGAGA**	0.031	0.007	0.022	12	542
**GCTGTTGCAGATG**	0.059	0.004	0.064	46	673
**CGTAGCACTAATG**	0.027	0.001	0.025	28	1091

CGTGTCGTAGATA	0.024	0.004	0.021	24	1103
AGTGGCACTGGGG	0.033	0.014	0.014	4	290
GGTGGCACTAATA	0.199	0.035	0.173	182	872
ACTGGTATTTGTA	0.021	0.004	0.024	36	1451
ACCGGCGCAGGGG	0.026	0.004	0.024	16	647
GCTAGTGTAAGCA	0.057	0.003	0.053	83	1473
GCTAGTGTTTGTG	0.055	0.007	0.057	41	674
ACCGTCGCATGGA	0.02	0.004	0.026	38	1437
AGCGGCGTATGCG	0.018	0.005	0.019	14	706
AGTGGCATTTGGA	0.019	0.005	0.018	7	379
GCTGTTGCAGACG	0.057	0.006	0.057	48	788
AGTATCATATATG	0.023	0.004	0.019	37	1890
CGCATCGTATAGA	0.023	0.003	0.021	40	1845
GGTATTATATGGA	0.112	0.02	0.085	94	1013
ACCATCGCTAGTA	0.021	0.002	0.02	56	2805
CCCGGTGTTAGCA	0.025	0.004	0.021	34	1551
GCCGTCGCAGAGG	0.056	0.01	0.067	36	505
CCTATTGCTGGCG	0.026	0.004	0.026	35	1337
AGTATTATAGAGA	0.024	0.001	0.025	57	2218
AGCAGTGTTGATA	0.021	0.003	0.017	33	1907
ACTATTGTAGGGA	0.03	0.004	0.03	49	1607
GCCGTCACAGGTA	0.055	0.001	0.055	76	1302
CCCGTTACAGAGA	0.027	0.006	0.025	58	2272
CGTATCGCTAGTG	0.034	0.002	0.031	25	783
CGCGGTATAAATA	0.028	0.002	0.031	88	2786
GCCGGCACTAATA	0.109	0.007	0.1	204	1831
ACCGGTGCATGCA	0.023	0.001	0.024	52	2073
AGCAGTACTAGCG	0.024	0.001	0.025	42	1658
GCTGTCACATGTA	0.068	0.01	0.078	85	1005
CCTGGCATTTGCA	0.026	0.004	0.023	31	1332
CCCAGTACTAGTG	0.031	0.001	0.032	61	1823
CCCAGTACTAGCG	0.025	0.007	0.031	62	1963
AGCAGCGCTTAGG	0.021	0.002	0.023	23	982
AGTGGTGCTGGGA	0.028	0.006	0.027	12	438
GGCGTTACTAACG	0.068	0.006	0.061	78	1194
CCCGTCACTAAGG	0.027	0.002	0.026	44	1623
GGTGGTGTTGGCG	0.177	0.038	0.226	75	257
CCTGGTGTATGCG	0.025	0.002	0.027	25	890
GCTAGCATATGCA	0.06	0.007	0.05	74	1395
AGTAGCGCAAAGA	0.021	0.004	0.02	27	1300

GCCGGTGCTGAGA	0.055	0.006	0.046	37	761
CGTGTTATAAAGG	0.027	0.002	0.029	35	1154
CGCATTGTTTGTA	0.021	0.003	0.024	32	1315
GCTGGTGTAAAGA	0.414	0.027	0.415	520	732
AGTAGTGTAGATA	0.023	0.005	0.021	34	1617
CCTAGTACTGGGA	0.025	0.006	0.034	36	1025
AGTAGTGTATAGA	0.025	0.003	0.022	28	1273
ACCATCGTAGGTG	0.025	0.006	0.018	26	1400
GGTGGTGCTAGCA	0.071	0.001	0.07	37	491
ACTGTCGTTAATG	0.026	0.002	0.023	29	1214
ACTGGCGTATAGA	0.027	0.003	0.029	29	958
CCTAGCGTTTACA	0.023	0.003	0.019	38	1924
GGCATTGCAAGCA	0.062	0.009	0.054	83	1457
ACCGTCATTTGTA	0.022	0.003	0.02	45	2203
GGCAGTGTTGATA	0.065	0.008	0.075	102	1249
GGCGTCACAAGTA	0.055	0.002	0.055	87	1507
GGTATCGTAGGCA	0.115	0.013	0.125	111	774
CGTAGCACAAACA	0.025	0.002	0.023	55	2318
AGTAGTGCAGATA	0.023	0.004	0.021	30	1369
AGCGTCGTTAGCA	0.024	0.004	0.029	47	1580
GGCAGCATTTAGA	0.051	0.006	0.043	55	1237
ACCATTGCAAGGA	0.022	0.002	0.021	56	2617
GCTAGTGTTGACG	0.059	0.008	0.049	51	981
CGTGGCGTTGGTG	0.016	0.005	0.013	4	305
GGTAGTGTATGGG	0.06	0.012	0.048	19	373
AGCGTCGTTGGGG	0.015	0.006	0.014	8	582
GGCGTTGCATATA	0.066	0.015	0.048	59	1172
GGCAGCGTAAGCA	0.063	0.007	0.056	80	1358
GGCAGCACTAGGG	0.053	0.005	0.047	33	664
AGTAGTGCTGATA	0.021	0.003	0.025	31	1198
AGCGGTATTAGGG	0.021	0.009	0.012	9	766
CCCAGTGTTGAGA	0.031	0.007	0.024	38	1535
GCCATCGCTTGCG	0.048	0.009	0.055	51	880
GGTATCATTGATA	0.128	0.024	0.106	151	1279
GCTAGCGTTTGGG	0.099	0.008	0.104	42	361
GCCGGTGTTTGCA	0.171	0.028	0.146	146	851
CGTATCATAAGGA	0.029	0.004	0.024	41	1635
AGCGGCGTTAACG	0.027	0.006	0.023	22	944
GGTAGTACATGGA	0.053	0.008	0.043	34	748
GCCAGCGCTAACG	0.056	0.013	0.051	63	1177

GGTATCATTAAGA	0.125	0.004	0.12	170	1243
GCTGGTACATAGG	0.107	0.012	0.119	80	590
GCCAGTATAGAGG	0.055	0.002	0.052	74	1355
GCTATCACTGAGG	0.067	0.013	0.07	64	855
CGCGGCATTAACG	0.024	0.009	0.02	24	1196
AGTGGCATTGGCA	0.018	0.005	0.017	10	569
CCCAGTGCTTAGG	0.021	0.002	0.02	22	1098
GCCGGTACATGTG	0.079	0.015	0.072	71	913
ACTGTCGTTTATG	0.023	0.012	0.03	31	1002
CCCGTCGCTAGTG	0.026	0.007	0.017	20	1138
GGCGGTGTTTAGG	0.095	0.013	0.077	39	469
ACCATTGCTGGGA	0.024	0.004	0.019	33	1726
CGCGGTATTAGGA	0.027	0.003	0.025	26	998
AGCGTTGCAGAGA	0.015	0.003	0.011	15	1319
AGTGGTACATATG	0.025	0.002	0.023	22	944
CGTGTCACATGGA	0.028	0.006	0.019	15	756
AGCAGTGCATGGG	0.018	0.002	0.018	16	893
CGTGTTACATACG	0.024	0.006	0.032	40	1192
AGTAGTGTAAGTA	0.02	0.001	0.018	29	1557
GCCATCATTTATA	0.052	0.002	0.05	153	2893
ACTGTTATTTGGG	0.036	0.009	0.022	20	875
AGTAGTGTTTATA	0.024	0.004	0.019	27	1368
GCCAGTGCTTATG	0.046	0.003	0.043	43	960
CCCGTCGTTTGGG	0.026	0.006	0.035	26	723
GGCGGCATTAAGA	0.3	0.051	0.28	381	979
CCTGTTATTAACG	0.031	0.006	0.032	64	1932
GCCATCGCAGGCG	0.043	0.004	0.04	52	1238
GCCGGCACAAGTG	0.2	0.014	0.206	248	954
CGCAGCGTAAATG	0.025	0.003	0.025	45	1746
GCCGGTGCATAGA	0.06	0.004	0.061	59	912
GGCGTTACTTGTG	0.061	0.015	0.06	42	661
CGCAGCGTTGGCA	0.025	0.004	0.021	26	1184
AGCGGCGCATGTA	0.016	0.001	0.018	21	1161
ACCGTTGCTGATA	0.023	0.001	0.025	58	2287
CGCGTTACATGCG	0.021	0.006	0.014	17	1164
GGCGGCATTAATG	0.548	0.055	0.516	855	801
AGTAGTACATGCA	0.021	0.003	0.017	27	1536
ACTAGCATATACG	0.022	0.003	0.025	59	2283
ACCAGCGTTGGTG	0.025	0.003	0.023	21	906
CCTGGCGTTAGTA	0.022	0.004	0.017	20	1175

GCCAGCACTTGCA	0.051	0.004	0.047	76	1534
CGCGTTGCTTGCG	0.028	0.007	0.036	27	713
AGTAGTGCAGGCG	0.024	0.008	0.032	25	768
GGTATCGTTTATG	0.149	0.042	0.107	79	658
GCTGTTGTTAATG	0.062	0.001	0.061	61	944
AGTAGTGTTTGTG	0.025	0.005	0.025	19	734
CGCGTTATAGACA	0.026	0.002	0.024	60	2446
GGTGGCGTAGAGA	0.378	0.04	0.352	199	367
ACCGGTACAGAGA	0.023	0.002	0.021	57	2718
CGTGTCGTTTATA	0.025	0.004	0.025	25	956
CGTAGTACTTGCA	0.027	0.001	0.028	26	893
AGCGTTGCAAATG	0.023	0.002	0.026	45	1710
GGCATTACATGGG	0.067	0.021	0.054	53	920
AGTATTGCTGGCG	0.024	0.003	0.022	22	995
ACTATTATTAGGG	0.029	0.002	0.029	44	1480
GGCGTCATATATG	0.07	0.011	0.054	70	1219
CGTGGCGCAAATG	0.029	0.004	0.029	21	701
GCTGTTGCTTAGA	0.046	0.002	0.048	32	628
CCTAGCACTGATA	0.022	0.004	0.027	65	2361
CGTGGTGTTAAGG	0.032	0.005	0.03	17	542
AGCGGCATAGAGG	0.024	0.004	0.023	13	549
GGTGGCACATGCA	0.192	0.023	0.181	149	672
GGCATTGCTAACG	0.068	0.003	0.066	90	1271
GCCAGTGTTGGTG	0.046	0.01	0.036	26	688
GCCATTGCTTGCG	0.049	0.008	0.038	34	871
ACCGTCACTAACG	0.024	0.003	0.021	61	2896
AGTAGCGCAAATG	0.025	0.004	0.029	34	1146
GCTAGCGCTGATA	0.062	0.01	0.056	54	908
AGTGTTGTTGAGG	0.023	0.002	0.021	12	570
CCTATTATAAGTA	0.028	0.001	0.028	99	3488
CGCAGTGCAAGCG	0.018	0.003	0.013	17	1253
GGCAGCACAGGGA	0.047	0.003	0.042	42	967
CCCAGCATTTACG	0.027	0.005	0.025	63	2490
CGCATCGCTGACG	0.028	0.003	0.031	46	1425
GGTAGTACTGATG	0.058	0.007	0.048	39	766
ACTATTGCAAGGA	0.027	0.002	0.024	52	2126
GGCATCACAGAGG	0.092	0.016	0.083	93	1030
GGCATCGTTAGGG	0.114	0.024	0.086	58	614
CGTGGCATTGGGA	0.03	0.009	0.03	16	522
CGCAGCGCAAGGG	0.022	0.002	0.023	21	903

GCTGGCGCAAGCA	0.629	0.038	0.638	906	514
GCCGGCGCAAATG	0.756	0.036	0.726	1204	455
GCTAGTGTAGAGA	0.055	0.007	0.046	56	1157
AGCAGTGTTTGTG	0.022	0.002	0.021	21	983
GCCGTTGTAGGGA	0.059	0.005	0.052	39	704
ACTAGCGTTAGTA	0.03	0.006	0.032	48	1468
GGCATCATTGAGG	0.095	0.019	0.078	84	989
GCCGGCGCTAGTA	0.487	0.026	0.488	563	590
GCCGTCATTAGGA	0.053	0.007	0.048	61	1215
GCTAGTGTAGGGA	0.06	0.006	0.065	53	762
AGCGTTACTAGCG	0.023	0.008	0.026	34	1253
CCTATCATTGGTA	0.029	0.005	0.023	50	2161
CGCGTCGCTAGCA	0.023	0.008	0.022	29	1284
AGTAGCGCTAGCG	0.022	0.004	0.022	14	612
GCCGTCGTTTATA	0.051	0.005	0.045	57	1216
CCCAGTGTTGGGG	0.025	0.003	0.03	24	771
CCCAGCGCATATG	0.023	0.003	0.02	39	1902
ACTGTTACTTGCG	0.025	0.001	0.023	29	1221
GGTAGTACTAGGA	0.067	0.013	0.057	39	642
CGCGTTGTTAGCG	0.022	0.004	0.026	25	919
GGCGTCACATACG	0.057	0.006	0.049	65	1273
CGTATTATTGGGG	0.033	0.009	0.027	19	686
GGCGTCGCAAACG	0.081	0.018	0.057	72	1196
GCTAGCATTTAGA	0.063	0.006	0.068	84	1160
ACTGGCATAAGGA	0.029	0.004	0.023	31	1324
GCTGTTGTATAGA	0.054	0.004	0.049	48	934
CCCGTCACAAGGA	0.025	0.004	0.022	49	2155
ACTGTCATAAAGG	0.033	0.008	0.023	41	1769
CCTATCATATAGA	0.026	0.004	0.02	59	2876
CCTAGCGTATATG	0.026	0.006	0.026	38	1437
ACTGTTGTAGATG	0.031	0.004	0.027	38	1359
CGTGGTGCAAGTG	0.025	0.006	0.022	13	585
CGTATCGTTTGCG	0.024	0.006	0.025	22	861
CCTGGTACTGGTG	0.03	0.007	0.022	17	770
AGCATTATTGGGG	0.015	0.002	0.013	16	1190
CGCGTTGTTTGCA	0.022	0.004	0.026	28	1035
GCTGTCGCATGTA	0.059	0.005	0.066	50	709
CGTGTTATTGAGG	0.025	0.008	0.025	16	616
AGCAGCGTTTGGA	0.026	0.003	0.031	34	1075
GCCGTTGCATGCG	0.045	0.005	0.041	34	793

GGTGTTGTTGACA	0.083	0.015	0.062	49	741
ACCGGTGTTGATA	0.025	0.001	0.024	43	1776
CCTAGTGCTGATG	0.026	0.009	0.021	27	1290
AGTAGCGTAAGCA	0.022	0.004	0.018	25	1402
CGCGGCATATGCG	0.023	0.004	0.018	15	821
GCCAGTACTTAGG	0.062	0.001	0.064	67	988
CCTGGTGCTGAGG	0.026	0.013	0.016	10	602
CGTATCACAAATA	0.025	0.003	0.025	69	2658
CGCATTGTTGGGG	0.025	0.007	0.018	10	553
CCCGGCACAGACG	0.028	0.005	0.024	50	2056
CGCGGTGCATGTA	0.024	0.006	0.032	33	1006
CGCAGCGTAAGTG	0.023	0.004	0.018	23	1249
GGTAGCATATGTG	0.078	0.016	0.055	38	652
GCTATCGTTTATG	0.059	0.003	0.058	61	987
CCCGTCATAAAGG	0.026	0.003	0.029	61	2031
GCCGTCGCTTAGA	0.048	0.008	0.038	32	804
ACTGTTACATAGA	0.025	0.002	0.024	55	2259
GGTAGCACTAGTA	0.062	0.014	0.057	51	846
GGCATCGCAGAGG	0.101	0.012	0.107	90	752
AGCAGTGCATGCA	0.021	0.002	0.019	37	1865
GGCGGTACAAGCA	0.066	0.012	0.051	67	1240
CGTGGCACATATG	0.024	0.003	0.021	16	756
CCTATTGCTTGGA	0.026	0.003	0.028	35	1217
CCCGTTGTTGAGG	0.029	0	0.028	32	1092
GGCGTCGTAAGGA	0.081	0.019	0.08	66	759
CCTATTGCTAACG	0.025	0.004	0.021	49	2313
CGCAGCACTAGTG	0.022	0.002	0.021	24	1105
GGTATCGTTGGTG	0.126	0.018	0.115	57	437
AGCGGTACAAGGA	0.023	0.004	0.025	38	1505
GCCGGCGCAGACA	0.51	0.056	0.528	936	837
CCTGGTATTTACG	0.023	0.007	0.02	25	1221
CGTGTTACAGGCG	0.021	0.001	0.022	17	764
GGTAGCACTGGTA	0.054	0.009	0.041	28	647
ACTGGCGTAAGGA	0.021	0.003	0.016	15	901
GCCGGCGCATATA	0.498	0.033	0.473	732	816
CCTGGTACATACG	0.025	0.004	0.029	44	1496
ACTGTCGCTAATG	0.025	0.002	0.022	32	1424
ACCGGCGCAGGTA	0.029	0.004	0.023	25	1063
CCTGGCGCTGATA	0.023	0.004	0.023	29	1227
CCTGTTACATGCG	0.026	0.005	0.023	30	1298

AGCGGTATTGGGG	0.027	0.003	0.024	15	620
GCCATCACAAGCA	0.056	0.008	0.047	132	2677
GGCGTCACTAACG	0.06	0.007	0.056	79	1321
AGTATCACATGCA	0.023	0.001	0.024	37	1497
CGTGTTATTTGGA	0.024	0.004	0.02	14	700
CGTAGCGTATACG	0.026	0.001	0.027	25	905
GGTATCGCTTGTG	0.128	0.029	0.088	42	434
GCCATCGCAAGTA	0.041	0.006	0.033	57	1651
AGTGTCGCAAGCG	0.027	0.002	0.026	21	795
AGCGTTGTATATG	0.027	0.004	0.022	31	1359
ACCGTTATTTATA	0.025	0.005	0.025	79	3089
AGCGGTGCAAATA	0.025	0.001	0.024	55	2241
CCCATCGTATGTA	0.022	0.004	0.016	39	2422
CCTGGTGTTTAGG	0.023	0.004	0.028	20	690
CGTGGCATTAACG	0.026	0.005	0.018	14	748
AGTGTTGCATGCG	0.033	0.009	0.026	18	676
GGCATTACAAGGG	0.069	0.016	0.057	67	1118
GGTAGCGCAGGCA	0.069	0.015	0.066	43	613
CCCATTGCTTACG	0.024	0.002	0.024	54	2179
AGTGTTACAAGGG	0.017	0.005	0.012	10	840
AGTGGTGCTGAGA	0.024	0.003	0.02	13	643
CGCAGCATAAAGG	0.024	0.005	0.029	48	1617
CCTAGCACTAGCA	0.023	0.002	0.022	46	2056
GGTAGTGTATAGA	0.059	0.009	0.061	54	834
CGCATTACAGACA	0.022	0.002	0.024	66	2634
AGTGGTACTAACG	0.024	0.004	0.023	25	1043
GCTAGTGCTTGGA	0.054	0.011	0.047	29	590
CCCGGTGTTTGTA	0.026	0.005	0.032	37	1113
GGTGGTGCTGGCA	0.076	0.017	0.058	28	452
CGCGGTGTAAGGA	0.022	0.002	0.023	23	964
CGTGGCATAGATG	0.026	0.002	0.028	16	548
ACTATTATATATA	0.028	0	0.028	141	4915
GGTAGTGCTGGTA	0.054	0.002	0.057	30	493
GGTGGTGCAAACA	0.074	0.017	0.052	51	931
GGCAGCGTAGATA	0.054	0.011	0.039	56	1363
GCCATCGCAAATG	0.054	0.009	0.06	103	1608
CCCATTATTTACG	0.031	0.002	0.03	91	2893
AGCGTTACTAACG	0.023	0.003	0.022	46	2056
AGCGGTATAGGTA	0.019	0.003	0.023	38	1583
GCCGTTGTTAATG	0.052	0.005	0.054	63	1108

CCCAGTGCTGACG	0.022	0.003	0.024	42	1738
GGCGTCACTGGGA	0.063	0.013	0.045	35	735
CGCGGCGTTTGGA	0.018	0.006	0.023	14	598
CCTGGCGTTAGGG	0.024	0.011	0.016	8	494
CGTAGTGCAAATA	0.026	0.001	0.027	39	1425
CCCAGTGTTGAGG	0.022	0.003	0.026	32	1186
GGCAGTACTGAGG	0.048	0.002	0.05	38	728
GCTATCGTAAAGA	0.046	0.005	0.052	86	1561
CCTGTTGCTGGGA	0.024	0.002	0.022	19	852
AGCGGTACAGAGG	0.016	0.006	0.014	15	1057
CCTGGCGCTGACA	0.028	0.001	0.029	42	1397
GCTATTACAGGCA	0.064	0.003	0.061	96	1487
ACTGTCGCATGTG	0.022	0.001	0.022	21	949
AGCAGTATAGATA	0.019	0.003	0.02	76	3798
CCCGGTATAAATG	0.025	0.001	0.027	70	2570
CGTATTACTAATG	0.027	0.001	0.026	44	1630
CCCGGCGTAGACA	0.021	0.005	0.015	36	2415
AGCATCGCATGGG	0.028	0.004	0.033	37	1083
ACTGGCATAAGCA	0.029	0.006	0.036	78	2094
GCTGGTGTAGACG	0.735	0.035	0.725	840	319
ACTGGTACTGAGG	0.026	0.002	0.028	27	935
GCTATCGCAAACG	0.057	0.002	0.055	79	1368
GCCATTATTAGCA	0.06	0.006	0.056	152	2563
CCCGGCATTGATA	0.03	0.005	0.031	74	2290
AGTATCGTAAGCA	0.02	0.004	0.02	34	1708
AGTGGTGTTGAGA	0.025	0	0.025	19	741
AGTGGCGCATATG	0.025	0.004	0.023	13	557
CCCAGTGTTAGGA	0.023	0.009	0.016	22	1363
CCTGGCATTTGGG	0.028	0.006	0.023	13	561
CCTGTCACAAACG	0.03	0.004	0.025	49	1942
GCTATCATTTGGA	0.057	0.004	0.057	64	1065
GCCAGCACTAGTA	0.049	0.005	0.042	77	1746
CCCGTTATTGGCG	0.035	0.003	0.035	50	1372
GGCATTGTATAGG	0.073	0.003	0.069	74	1002
ACTGTTACTTGCA	0.023	0.007	0.016	29	1807
ACTGTCACTTAGG	0.022	0.003	0.018	18	983
CCTAGTACTGGGG	0.04	0.013	0.059	44	707
ACTGTTACAGATG	0.027	0.004	0.023	37	1594
GGTGGCGTTAGCA	0.377	0.02	0.373	232	390
CGCATCGTTTGGA	0.022	0.005	0.015	16	1068

GGTGTCGTAGATG	0.103	0.019	0.086	50	534
CGTGGCACTGGCA	0.032	0.005	0.025	19	755
GCTAGCGCAAGCA	0.057	0.006	0.048	54	1071
GCCGGCACATGTG	0.22	0.022	0.213	222	818
GGTATTGCTAAGA	0.069	0.002	0.068	66	903
CGCAGTGCAGATG	0.032	0.002	0.032	38	1166
CGCAGCATTGAGG	0.026	0.001	0.025	26	1007
GCTAGTATTAGCG	0.061	0.014	0.049	55	1057
GCCATTGCTTACG	0.051	0.006	0.043	56	1236
CCCGGTGTTAGTA	0.025	0.005	0.018	27	1436
GGTATTACTGGTG	0.107	0.017	0.084	57	618
GGTGGTATTAGCA	0.125	0.029	0.12	90	660
GGCGTCATAAAGG	0.066	0.013	0.058	68	1109
GGCAGTGCAGGCG	0.035	0.005	0.028	24	829
ACCATCGCAAGTA	0.024	0.002	0.021	73	3370
CCTGTTATTTACG	0.025	0.004	0.024	39	1602
ACCAGTGTTTATG	0.02	0.003	0.024	41	1691
CCCGGTGTAGATA	0.02	0.001	0.02	33	1659
AGTGTCATTGGCA	0.023	0.003	0.018	20	1071
CCCATCGTAGGGA	0.033	0.006	0.027	41	1477
GGCGGTACTGGGA	0.068	0.016	0.059	41	650
CGCGTTGCTAGGA	0.015	0.003	0.015	14	901
GCTAGTATATGGG	0.061	0.004	0.066	52	741
GGTGTTGTTGACG	0.095	0.012	0.099	51	464
GCTGTTGCAAATG	0.055	0.005	0.056	55	935
ACCATTATATGCG	0.025	0.004	0.019	59	3001
GGTATCATTGAGA	0.136	0.018	0.114	118	915
GGCAGCGCAAGGG	0.059	0.02	0.049	35	683
GCCGTTGTAGACA	0.043	0.008	0.038	61	1538
AGTGTCGTTGATA	0.017	0.003	0.013	15	1148
ACCAGTACTAGGG	0.022	0.004	0.027	48	1739
AGCAGCGTTGGGA	0.022	0.004	0.017	16	937
ACCGGCATTGGCA	0.023	0.004	0.018	33	1807
GCTAGTATTGGGA	0.056	0.005	0.053	44	790
GGCAGCGCTAACG	0.06	0.004	0.056	70	1171
CCCGGTGCAGGTA	0.02	0.001	0.022	33	1491
GCCAGCGCTGGGA	0.05	0.014	0.053	37	660
GCTGGCACTTAGA	0.205	0.021	0.2	171	684
CCCGTCATTTGTG	0.019	0.004	0.014	18	1277
AGCATCACTTGTG	0.024	0.002	0.02	33	1591

GCTATTGCAGACG	0.059	0.009		0.053	67	1202
ACCGTTGTTAGGG	0.021	0.002		0.022	20	907
GCTATCATAGAGA	0.066	0.004		0.063	104	1545
AGTGGTACATGCG	0.023	0.006		0.031	21	648
GCTAGTGTTGGGG	0.056	0.008		0.061	30	458
CGTAGTACTGGGG	0.034	0.017		0.058	21	341
CGTAGTACTAAGG	0.028	0.005		0.023	21	878
ACTGTCGCTAGCA	0.022	0		0.021	35	1598
GGCGGTGTTAGGA	0.07	0.005		0.064	39	570
GGTATTGTATAGA	0.111	0.012		0.101	105	930
GCCGGCACAGGTG	0.201	0.012		0.205	183	708
CCTAGCACTTAGA	0.029	0.001		0.028	48	1697
ACTATCGCTGGGG	0.02	0.008		0.025	19	753
GGCGTTGCTAGTG	0.084	0.028		0.046	27	563
CGCGTCATAAAGG	0.027	0.01		0.017	25	1487
CGTGGTGCTGACG	0.025	0.008		0.027	16	571
AGCGGTACTGGGA	0.022	0.006		0.014	13	928
GGTGTTGCAAACG	0.094	0.016		0.077	61	732
CGTGGTGCAGAGG	0.027	0.011		0.013	6	445
CGCGTCGTATGCG	0.026	0.008		0.015	15	996
AGTAGTATTAGTG	0.022	0.007		0.025	27	1052
GCTGGTGCATGCG	0.279	0.014		0.267	173	476
GGTAGCGCAAGGG	0.087	0.008		0.089	32	327
CCTGTCGCAGATA	0.024	0.003		0.019	33	1718
GGCGGTATTTACA	0.063	0.015		0.043	61	1350
GCTATTGTTAGTA	0.054	0.002		0.056	75	1263
GGTATCACTTACA	0.102	0.009		0.094	123	1179
GGCGGCACTAGCG	0.255	0.051		0.234	218	712
GCCATTGCTAACG	0.052	0.007		0.046	78	1601
GCCAGCGTAAGTA	0.059	0.011		0.06	104	1634
CGCAGCGCTTAGG	0.024	0.001		0.024	18	744
CGCAGTATTTACA	0.024	0.004		0.02	48	2389
GCCATCACAAATA	0.05	0.004		0.047	187	3782
CGCGGCACATGCA	0.022	0.002		0.024	37	1481
CGTATCACAGGCA	0.026	0.008		0.019	26	1339
GGTGTTATTTGGG	0.082	0.02		0.062	27	411
ACCATTGTATAGG	0.025	0.002		0.028	53	1809
CGCGTTATAAATG	0.026	0.001		0.027	59	2110
AGTGTCACTTATA	0.02	0.002		0.021	29	1384
AGTGTCGCTTGTA	0.022	0.006		0.019	14	725

GGTGTCGCTTACA	0.093	0.024	0.06	43	669
GGCAGCGCAGAGA	0.065	0.009	0.065	60	860
CGTGTCATTGAGA	0.026	0.003	0.021	21	993
AGCATTGCATGGA	0.022	0.005	0.024	39	1607
CCTGGTGTAGGGG	0.036	0.01	0.023	12	503
AGTATCATAGGTA	0.022	0.003	0.025	44	1707
CGTAGCACTGGGG	0.028	0.009	0.025	11	426
GGTGGCATAGGCA	0.481	0.039	0.451	430	524
ACCGTTACTGAGG	0.021	0.002	0.021	38	1749
ACTGGTGTTTGTG	0.02	0.002	0.018	12	667
GGTGGCGTTTACA	0.387	0.05	0.385	288	461
AGTAGCACTGGTA	0.02	0.003	0.019	18	937
AGCGGCACTGGTA	0.018	0.006	0.017	19	1094
AGTGTTACTTACG	0.025	0.011	0.014	16	1099
AGTAGTGCTAGTG	0.028	0.004	0.026	17	633
GGCAGCGTTGGGA	0.05	0.002	0.053	35	631
GGTGTCGTTAGCG	0.095	0.008	0.087	35	367
GCTGTCACAGGGA	0.064	0.002	0.062	45	685
CGCGGTATTAAGA	0.03	0.003	0.028	46	1590
AGTAGTGTATGTA	0.03	0.005	0.031	39	1223
CGTGTCGTTAATA	0.026	0.004	0.032	42	1285
ACCAGTACTTACG	0.023	0.002	0.021	69	3240
AGTATTGCTAGGG	0.028	0	0.029	20	680
CCTGGCATAGGCG	0.037	0.011	0.034	34	975
GGCATTGTAGGCA	0.067	0.01	0.069	95	1288
ACCGGCGTAGAGG	0.023	0.005	0.017	10	579
AGTAGCATTGATA	0.025	0.005	0.02	34	1652
GCCGTTATAGATG	0.056	0.003	0.06	90	1415
CGTGTCGTATAGA	0.028	0.004	0.029	26	884
GCTATCATAAAGG	0.065	0.003	0.065	113	1633
CCTGTCGTTTGTG	0.032	0.005	0.026	19	700
GCCGTTGTTGGTG	0.043	0.015	0.028	19	649
AGTGGTGTTTATG	0.013	0.002	0.013	8	605
CGCAGTACAAGTG	0.026	0.003	0.029	46	1545
ACCGGCACTGGGA	0.024	0.007	0.017	21	1193
CCTGGCGTAGGCA	0.023	0.004	0.018	22	1207
GGCATTGCAGACG	0.072	0.001	0.071	93	1213
CGCGTCACTAGGA	0.023	0.005	0.018	20	1111
CGCGTTGCAGGCG	0.027	0.003	0.024	23	921
ACTGGCGCATATG	0.029	0.008	0.018	16	871

ACCAGTGTATATG	0.028	0.002	0.03	62	2010
AGTATCGTTAATG	0.021	0.007	0.018	28	1486
AGTATTGCAGGTA	0.025	0.003	0.02	25	1223
GCCAGCGTTTGCA	0.049	0	0.049	60	1170
CCCATTGCTTGCG	0.025	0.006	0.019	31	1560
CCCGTTACATATG	0.032	0.003	0.028	63	2166
CGCAGTGTTGGGA	0.017	0.003	0.012	9	739
CGTGGCACTTGTG	0.019	0.003	0.018	9	491
CGCGTTGTATGCG	0.02	0.004	0.016	16	960
GCTATTATAAGGA	0.059	0.005	0.053	99	1776
AGCATCATTTGGG	0.024	0.005	0.021	29	1364
CGCGTTGTTTGGG	0.022	0.005	0.022	13	567
CCTGGTATAGGTA	0.027	0.001	0.029	40	1357
CCCAGTATTTAGG	0.023	0.002	0.025	44	1689
CCTGGTATAAGCA	0.027	0.001	0.027	54	1917
AGTATCACTTGTA	0.024	0.008	0.019	23	1207
ACTGTCACTTACG	0.02	0.004	0.023	33	1391
CCCGTCGCTGATA	0.027	0.006	0.023	50	2109
GCTGTTGCATGGG	0.049	0.004	0.045	19	399
GGCATCACAAACA	0.07	0.013	0.057	184	3035
ACCATTGTAGGTG	0.025	0.003	0.022	33	1476
CCTGGCACTTAGG	0.026	0.007	0.024	19	764
ACTATCGTTAGGA	0.028	0.004	0.028	39	1359
GGCATTGCATGGA	0.058	0.003	0.054	54	946
ACTGTTACTGGCG	0.021	0.004	0.021	24	1122
CCCAGTATTTATG	0.026	0.003	0.028	68	2364
AGCGGCGTAAGTG	0.019	0.006	0.026	20	764
CCTGGTGTAGGCA	0.027	0.008	0.022	28	1235
GGTGGTGCATAGA	0.078	0.012	0.067	36	500
CGTATCATTAAGA	0.027	0.004	0.03	61	1969
GGTATTACAGATG	0.101	0.027	0.083	92	1013
ACTGTCGCAAGGG	0.03	0.01	0.02	18	886
CGCGTCATAGAGG	0.027	0.002	0.029	30	1017
CCTGTTGTATACG	0.028	0.004	0.029	44	1491
CGCAGCACAAAGA	0.024	0.002	0.022	52	2345
GCCAGCATAGGCA	0.051	0.006	0.046	92	1923
GCCGTCACAGATG	0.046	0.013	0.04	55	1337
CCTAGCGCTGAGA	0.019	0.003	0.017	24	1414
GGTGGTGTATGCA	0.103	0.017	0.082	47	524
AGTGTTGTTAGCA	0.024	0.005	0.017	18	1052

GCCGGCGTTAGCA	0.857	0.024	0.861	1445	233
GCCATCGTTTGCA	0.049	0.009	0.046	68	1411
AGCAGTACTGGGG	0.023	0.002	0.022	20	879
GCCGGCACAAACG	0.219	0.021	0.226	431	1474
CCCAGTACATGGA	0.027	0.005	0.019	42	2191
AGCAGCGCTAACA	0.02	0.003	0.018	46	2492
AGTATTACTAAGG	0.025	0.003	0.023	34	1457
CCCAGCGCAGATG	0.023	0.003	0.019	36	1845
AGTGTCGCAAATG	0.022	0.006	0.016	18	1082
AGCGGCATTTGCA	0.021	0.003	0.021	25	1139
CGCGGCGTTGATA	0.029	0.003	0.028	32	1098
CGCGGTGCTAGTA	0.021	0.002	0.018	19	1013
GCTGTCACAAGTG	0.065	0.002	0.067	61	848
ACTGGTGTTAGTA	0.025	0.002	0.027	31	1132
GGTAGCGCAGATG	0.085	0.01	0.077	48	572
CCCATTATAAGGA	0.025	0.003	0.026	83	3152
CGTATCGTTGACG	0.029	0.006	0.023	27	1137
CGTATCGTTAGTG	0.022	0.006	0.014	13	886
GGCGGCGTTAGTA	0.221	0.025	0.193	141	591
CCTGGCGCATGTA	0.022	0.007	0.014	15	1077
CGCGGTACTAAGG	0.022	0.002	0.02	20	986
AGTAGCGCAGATA	0.027	0.003	0.025	33	1294
ACTATCGTTAGGG	0.025	0.003	0.022	24	1043
AGTATTATAGACG	0.022	0.005	0.017	34	1995
ACCAGTGCAAAGG	0.024	0.003	0.027	57	2054
GGCGGTGTAGATA	0.077	0.007	0.076	85	1027
ACTATCGTAGGTG	0.026	0.005	0.023	28	1207
CCCAGTACAAGGG	0.026	0.004	0.032	55	1653
CGTGGCACATACG	0.024	0.006	0.017	14	812
AGTATCACAGAGG	0.03	0.008	0.021	20	934
CGTAGCACTTGGG	0.028	0.01	0.038	20	504
AGTGGCACAGAGA	0.021	0.008	0.011	10	925
GGTGTCGCTTACG	0.098	0.011	0.1	55	493
CCTGTCATTGATG	0.023	0.003	0.027	41	1480
GCCGGCACTTGCA	0.106	0.006	0.113	141	1105
GGTAGTGTTGATG	0.056	0.003	0.061	35	543
ACTAGTACTTAGA	0.021	0.002	0.02	52	2510
GCCGGTGCTGGGG	0.095	0.026	0.122	50	361
GGTAGCGTTAACA	0.077	0.011	0.067	69	961
CCTATTGTATGGG	0.025	0.004	0.019	24	1213

CGCGTCATTTGGA	0.022	0.002	0.019	18	923
GGCATCGTAGGGA	0.083	0.015	0.065	66	949
ACCATCGTTGAGA	0.023	0.003	0.022	47	2077
ACCGTTACTGGTG	0.026	0.005	0.03	46	1499
AGTGTTATTAGTG	0.024	0.001	0.025	25	977
CCCGGCGTAGAGA	0.027	0.004	0.023	36	1552
AGTGTTACTGGCG	0.02	0.009	0.009	8	840
GGCGGTACAAAGG	0.086	0.012	0.073	66	842
ACTGTTACATACG	0.028	0.003	0.032	67	2015
CGCAGTGTTAAGA	0.024	0.004	0.023	34	1455
CGCAGTGCTTGCG	0.025	0.008	0.023	19	806
CCTATTATTTGGG	0.026	0.005	0.021	27	1246
CGTAGCGTAGGGA	0.035	0.005	0.029	16	535
AGCGTCATATAGA	0.021	0.002	0.019	40	2095
ACTGGTGTTGGCG	0.025	0.007	0.014	12	819
AGTGTTGCAAGGA	0.021	0.008	0.022	19	854
AGTAGCACTGACG	0.024	0.004	0.021	23	1081
CGTGGCACAAAGG	0.025	0.006	0.017	13	752
GGCGGCGCTTACA	0.106	0.015	0.093	99	962
CGCATCACAAATG	0.03	0.003	0.033	91	2678
CGTATCGTTGGCG	0.021	0.001	0.019	18	910
CCTATCACTGGGG	0.049	0.006	0.044	43	927
CCTGTTGCATATA	0.029	0.001	0.03	58	1880
CGCGTTATTGAGG	0.02	0.002	0.017	16	921
GGTAGTGTTGACA	0.056	0.011	0.045	37	793
GCTATCGCAGAGG	0.051	0.009	0.043	32	707
GGTGGTGTAGACA	0.113	0.009	0.103	76	661
AGTAGTACTGGCA	0.013	0.002	0.012	13	1096
CCCGGTACTGGGA	0.025	0.003	0.025	29	1145
GCCGGTATTGGTA	0.076	0.02	0.051	57	1069
AGCAGTATAGGTG	0.024	0.003	0.024	39	1555
GCTGGCGTTAGGA	0.882	0.023	0.874	939	135
AGTAGCACTAATA	0.022	0.002	0.02	40	2009
AGCGTCGTATGGA	0.025	0.004	0.02	23	1137
CGCGGTACAAGTG	0.022	0.003	0.025	28	1079
CGCATTGTATGGG	0.029	0.003	0.025	23	892
ACTATCATATGGG	0.031	0.003	0.03	39	1266
GGCAGTGTAAGCA	0.054	0.005	0.053	83	1493
AGTGGCGCTAGGA	0.022	0.01	0.014	7	499
GCCGTCATAGAGA	0.061	0.008	0.071	120	1569

CCTATCGTAGATA	0.03	0.004	0.025	62	2439
GGTAGTGTAAGGA	0.056	0.006	0.058	39	637
ACCGTTGCTTGTA	0.024	0.002	0.026	46	1733
CCTAGTGCAAAGA	0.027	0.003	0.025	57	2255
GCTATTGCAGATG	0.059	0.005	0.053	55	985
CGTGTTGCAGATA	0.027	0.006	0.026	28	1069
CGTGTCATTAAGG	0.027	0.003	0.028	24	824
CGTATCACTTGTA	0.027	0.006	0.019	23	1172
GCTAGTACTTGCA	0.062	0.01	0.055	62	1066
GCCGGCATTTGTA	0.41	0.015	0.421	571	786
GGCGGTGCTTGGG	0.067	0.022	0.044	14	304
GGTGGTACAAGCG	0.099	0.033	0.078	48	568
CCTGGCGCTGGTA	0.023	0.003	0.019	17	880
GCTAGCGTTAATA	0.062	0.003	0.057	84	1389
GCTAGTGTTTAGG	0.058	0.013	0.041	27	635
GGTAGCGTATATG	0.079	0.021	0.06	41	645
GGTGGCACTAAGG	0.392	0.061	0.344	244	466
CCCGTTACTAATG	0.027	0.003	0.031	72	2243
CGTAGCATTTGCG	0.017	0.005	0.012	9	715
AGTAGCGCTGACA	0.03	0.003	0.027	34	1215
CGCAGCATTTGCG	0.026	0.002	0.027	32	1165
GCTGGTATTTACG	0.436	0.039	0.433	486	636
GGCATCATAGGTG	0.065	0.017	0.051	64	1201
GGTGTTACTTGCG	0.086	0.006	0.095	45	431
CCCGTTACAAGGA	0.026	0.003	0.021	47	2187
ACTGTTGCTTATA	0.019	0.002	0.019	38	1984
GGTATTGTATAGG	0.112	0.003	0.116	81	618
CGTGTTACTTGCA	0.03	0.006	0.037	38	987
GGTAGCGTAAACG	0.074	0.005	0.067	56	786
CGTGGTGTTAGCA	0.029	0.012	0.04	27	648
AGTATTACATGGA	0.021	0.002	0.023	33	1399
ACTAGCACTAATA	0.025	0.001	0.024	98	3919
AGTGTTGCATGGG	0.026	0.006	0.03	14	460
CCTGGCATATGCG	0.027	0.003	0.024	25	1010
GGCGGCGCAAGCG	0.169	0.03	0.177	130	603
GCTATCGCATACA	0.057	0.016	0.036	56	1488
GGCATCGTAGGCG	0.079	0.017	0.055	59	1021
CGCAGTACTGGGA	0.021	0.01	0.014	14	971
AGTGTTGCTTACG	0.032	0.01	0.04	31	743
CGCGTCATTAGCA	0.023	0.003	0.026	44	1632

CCTAGCACTTATA	0.022	0.005		0.018	41	2290
GCCGTTGCTAGCA	0.051	0.005		0.044	48	1048
CGTGGTGTTGAGG	0.021	0.005		0.014	5	356
AGTATCACAGGCA	0.029	0.002		0.029	40	1354
GGCGGCATTAGCA	0.304	0.055		0.274	333	882
GGCGGCACAGGGA	0.133	0.018		0.133	102	667
CGCAGCGCAGGCA	0.029	0.003		0.028	37	1304
GCTAGCGCTTGTG	0.045	0.01		0.053	24	428
CGCGGCATTTACA	0.023	0.003		0.019	31	1580
CCTGGCACTGAGA	0.024	0.005		0.018	23	1278
GCTGTCACTGATG	0.057	0.01		0.052	44	804
CGTGTCATTAGGA	0.02	0		0.02	17	829
CCTATCGTATATG	0.021	0.004		0.016	30	1895
CGTGTCATAAGGG	0.015	0.005		0.014	10	729
AGTGGCACAAGGG	0.026	0.005		0.028	13	459
CCTAGCGTTGACG	0.025	0.003		0.024	35	1398
CCCAGCGCAAGTG	0.025	0.005		0.018	32	1710
ACTATTGCATGCG	0.024	0.005		0.029	53	1797
CCTGGCATAAGTA	0.022	0.003		0.025	43	1688
GGTATCGTAGACA	0.135	0.021		0.113	147	1149
CCTAGCACATGGG	0.047	0.01		0.043	42	938
AGCATCATTAGTG	0.021	0.002		0.024	46	1894
GGTATCGTAGGTA	0.13	0.023		0.101	79	707
CCCATCGCTTGCG	0.025	0.006		0.017	28	1580
AGTGTCACAAACG	0.026	0.002		0.025	35	1387
CGCGGCGTTAGCG	0.028	0.005		0.022	14	615
CGCATCACTGGTG	0.033	0.003		0.036	44	1180
GGTGTTGTTTGTG	0.079	0.02		0.056	21	355
CGTAGCGCAGGCA	0.018	0.004		0.023	18	782
CCTGGCACAAGCG	0.026	0.001		0.024	31	1250
GGCGGTGTTGGTA	0.064	0.006		0.061	41	628
GGCGTTATTTATG	0.062	0.013		0.05	53	1015
GGCGTCGTAAATG	0.087	0.017		0.082	109	1224
CCTGTTATTGGGG	0.048	0.009		0.056	39	659
GGCGGCGTTGAGA	0.194	0.027		0.16	119	626
CCTAGTGCATACG	0.026	0.003		0.022	41	1783
ACTAGCACTAGGA	0.024	0.003		0.024	45	1864
AGCAGCATAAACA	0.021	0.003		0.024	119	4801
AGTATTACAGGCG	0.024	0.002		0.022	28	1260
AGTAGCGTAGGTG	0.022	0.005		0.024	14	574

AGTATCACAGACG	0.022	0.002	0.019	28	1416
ACTGGTGCTGGGG	0.021	0.005	0.013	6	454
GGCAGTATTTAGA	0.056	0.012	0.04	62	1479
CGCATTGTAAGTA	0.023	0.002	0.025	55	2165
AGTAGCGTTAATA	0.025	0.004	0.02	35	1701
CCTAGCGTAGGTA	0.03	0.012	0.018	25	1383
GCTATTATTAAGA	0.061	0.003	0.057	137	2275
CGCGTCACATGCG	0.021	0.008	0.017	21	1226
AGTATCGTAGAGA	0.022	0.002	0.024	32	1322
CGCAGTATTAAGG	0.023	0.007	0.031	45	1402
GCCGGTGTTGATA	0.177	0.033	0.15	191	1079
ACTATTGCTAAGG	0.026	0.002	0.025	46	1817
GGCATTACTGGCG	0.07	0.007	0.059	65	1029
GGCATTGCATACA	0.061	0.006	0.057	105	1743
CCTGTCATATACG	0.028	0.008	0.023	46	1978
GGTATCACAGAGG	0.13	0.055	0.074	53	661
CCTGGCACTTGCG	0.028	0.007	0.025	22	871
GGTAGTGTTAATA	0.053	0.012	0.057	63	1039
GGCGGTGCAGATG	0.062	0.015	0.046	31	641
GCTATCACTTAGA	0.064	0.004	0.067	87	1216
CCCATTGCAAGCA	0.025	0.004	0.021	69	3145
AGCGTCGCAGGCA	0.025	0.002	0.026	39	1464
CGCGTCACAGGGG	0.022	0.003	0.026	19	714
ACCGTCGCAGGTG	0.023	0.005	0.026	33	1224
GGTATCACTGATA	0.092	0.013	0.074	95	1190
GCCATTGCAAGTG	0.052	0.002	0.055	65	1123
ACTGTTGCTAGTA	0.022	0.002	0.02	31	1538
GCCAGTGTATACG	0.047	0.006	0.044	68	1462
GCTATTACAGACA	0.056	0.004	0.051	118	2206
GGCAGTACTTATA	0.052	0.004	0.056	103	1747
GCCGGTATTAGCG	0.166	0.011	0.164	185	942
GGTGGCATTGAGG	0.717	0.045	0.708	505	208
CCCATCGCTGACG	0.026	0.005	0.026	53	1986
GGCATTGTTTAGG	0.096	0.017	0.082	66	735
GGTGGTACAGATA	0.086	0.014	0.069	61	826
ACTGTTATTGGGG	0.032	0.005	0.032	21	631
CGTATTGCATACG	0.033	0.003	0.029	35	1167
GCTGGTGCTTGTA	0.134	0.024	0.132	84	554
AGCAGCGCTTGGG	0.022	0.005	0.017	12	713
ACTAGTGTTGGCA	0.022	0.005	0.019	29	1476

CCCAGTGCAGAGG	0.025	0.002	0.025	32	1263
GGTAGTATATGGG	0.065	0.015	0.046	28	576
CGCGGCGCTAAGG	0.023	0.001	0.024	19	776
ACTGGTGTTAATG	0.03	0.006	0.035	42	1148
GGCGTCGCTAGGA	0.059	0.01	0.046	33	678
GGCGTCGTAGACG	0.075	0.012	0.06	52	817
AGCATCACAGGTG	0.025	0.002	0.023	39	1694
GCCATTGTTGGTA	0.048	0.003	0.05	65	1237
AGTAGCGCTGACG	0.024	0.002	0.021	17	805
CGTGTTACATGGA	0.02	0.007	0.013	13	963
GCTGGTATAGACA	0.15	0.008	0.149	236	1345
GCCATCGTAGAGA	0.048	0.004	0.053	85	1517
GCCAGCACTAGCA	0.054	0.011	0.051	98	1826
ACTATTACAGGGG	0.026	0.003	0.024	26	1060
GCTATTATAGACA	0.062	0.003	0.066	191	2683
AGTATTACTTGGG	0.022	0.002	0.019	16	841
AGTATCGCATACG	0.024	0.003	0.022	28	1271
AGCGGCATAAATA	0.02	0.004	0.015	37	2388
ACTGGTATAAGGG	0.029	0.01	0.029	28	948
CCCGTTGCATATG	0.026	0.001	0.027	44	1581
CGCGTTGTTAACG	0.021	0.002	0.022	29	1277
GCCGGTGCTTACG	0.1	0.02	0.084	74	807
AGTAGTGTATATG	0.024	0.003	0.029	34	1150
AGCATCGCTGAGA	0.022	0.002	0.026	44	1662
GGTAGCGCTGGCG	0.085	0.028	0.059	27	429
AGTATTACAGGCA	0.023	0.001	0.024	42	1739
AGTAGCGCTTACA	0.025	0.003	0.021	25	1190
CGCGTTACTGAGG	0.027	0.006	0.036	34	922
CCTGGCATTAGTA	0.024	0.007	0.017	25	1440
CGCGGCGTAAGTA	0.025	0.005	0.031	39	1237
CCTGTTGTATAGG	0.026	0.005	0.028	31	1094
CGTATTATTGGCA	0.029	0.004	0.03	44	1446
GGCGGCACTGACA	0.12	0.026	0.106	153	1286
GCTAGCGCATGGA	0.065	0.004	0.06	39	607
GCCATCGTTTGCG	0.051	0.005	0.049	55	1067
CCCGGCGTAAATA	0.02	0	0.02	62	3114
CGTAGCACTGGCA	0.023	0.007	0.014	13	919
GGCAGCATAAGCA	0.05	0.01	0.036	75	1988
GGTGGCGTTTATG	0.663	0.049	0.636	460	263
GGTATTACAAATG	0.112	0.01	0.123	193	1376

GGCGGCACTTAGA	0.135	0.025	0.123	119	849
GGTGTCGTTTATA	0.077	0.015	0.061	41	631
GCCGGTACTGACA	0.057	0.011	0.059	102	1621
AGCAGCGTAGACG	0.019	0.002	0.022	38	1685
GGCATTGTTTACG	0.074	0.004	0.074	86	1072
CGCAGCGCTAACG	0.028	0.005	0.021	31	1429
GCTATTGCATGCA	0.06	0.002	0.06	87	1355
ACTAGTATAAAGG	0.023	0.002	0.022	54	2450
CGTGGTACAGACG	0.023	0.008	0.016	13	820
CGCGGTACTTGCA	0.021	0.002	0.02	25	1197
CGCGGCACAGGCA	0.024	0.006	0.021	31	1476
GCCATCACAAACG	0.046	0.006	0.042	123	2800
CGCGTCGCATGGG	0.026	0.006	0.02	13	625
AGTGTCGCTGACA	0.026	0.006	0.018	21	1171
CGTATTGTTGGCG	0.026	0.007	0.024	19	773
CCCGTCGCATGGA	0.018	0.003	0.014	20	1369
CGTAGCGTTGACG	0.025	0.006	0.033	29	858
GGTAGTATTGGCA	0.062	0.007	0.059	49	786
CGCAGCGCATGGA	0.026	0.006	0.034	37	1061
CCTAGCATATGCG	0.025	0.005	0.021	31	1457
GGTGTCGTATATA	0.091	0.019	0.088	81	835
GGTAGTACTGGCG	0.058	0.005	0.064	35	508
GCCAGTGTTTACG	0.046	0.006	0.039	50	1245
CGTGTCATTGAGG	0.021	0.007	0.014	10	700
ACCGGCGTTAGGA	0.023	0.005	0.019	18	910
ACTATTACTAGTG	0.029	0.004	0.024	54	2223
CCCAGCGCTAGGA	0.025	0.004	0.03	52	1677
GGTATCACTTGGG	0.135	0.038	0.126	56	387
AGCGGCATTGGGA	0.015	0.005	0.008	5	660
AGTAGCATATGGA	0.026	0.005	0.019	22	1147
ACTATCATATGTA	0.029	0.003	0.032	92	2805
CGTATTATATGTG	0.022	0.004	0.026	35	1313
AGCGGTGTAGATA	0.022	0.006	0.017	29	1680
CCCAGTATAGAGG	0.025	0.006	0.032	58	1743
CGCAGTATAAGGG	0.023	0.004	0.023	28	1183
GCTGTCGCTAATA	0.063	0.008	0.063	67	992
ACTATCGCTTGTG	0.031	0.004	0.025	30	1164
CCCAGTATTTGTG	0.02	0.002	0.02	35	1751
CGTGTCGCAGATG	0.021	0.004	0.015	12	771
CCTGGTATAGACA	0.027	0.003	0.023	49	2086

ACTGGTGCTTGCA	0.023	0.004	0.02	24	1204
CCCGTCGTTGGCG	0.023	0.003	0.027	31	1128
GCTGTCGTTAGGG	0.055	0.011	0.041	15	353
CCCGGTATTTATG	0.026	0.009	0.018	30	1638
GGCAGCATAAGCG	0.058	0.007	0.063	89	1317
CGTAGTGTTAAGA	0.023	0.005	0.017	17	975
CCTAGTGCAAACG	0.029	0.007	0.02	45	2231
GGCGTTATTTACG	0.059	0.007	0.052	55	1004
CCTGGTATATACA	0.022	0.006	0.015	37	2373
CGTATTGTTAGTA	0.019	0.003	0.017	21	1196
CGTGTTATATGTA	0.024	0.003	0.024	32	1306
CCTATCGTAGGCG	0.026	0.002	0.027	41	1488
GCTATCGTAAACA	0.056	0.008	0.044	106	2284
CCTATCGTTTGGA	0.03	0.005	0.024	28	1127
GGTGTTGTTTACA	0.073	0.01	0.068	53	729
ACCGGCGTAGACG	0.023	0.004	0.018	16	886
AGCGGCACTAGGG	0.017	0.006	0.009	6	643
GGCAGTACAAAGA	0.053	0.007	0.06	127	1974
CGTAGCGCAGATG	0.026	0.006	0.019	15	761
GCTGTCGTAAGGG	0.069	0.001	0.07	34	455
CCCGTCGCTGGGA	0.024	0.009	0.022	26	1158
CCTGGCGTTAGCG	0.02	0.007	0.023	20	832
AGTGGCATATGCA	0.027	0.004	0.022	17	756
GGTAGCACATGCG	0.074	0.009	0.061	43	658
GGTAGTGTTAAGA	0.065	0.012	0.054	42	735
GGCATCATTTGGG	0.089	0.019	0.079	69	807
CCTGTCGTTGAGG	0.025	0.011	0.01	8	827
CCTATTGTATGCA	0.022	0.004	0.019	43	2262
GGCGGTGCTAGTA	0.064	0.008	0.065	53	768
GGCATTACAGGTG	0.059	0.006	0.051	56	1048
CCCATCGTTTGGG	0.017	0.003	0.013	14	1024
CCCAGCGTTGGGA	0.022	0.005	0.028	31	1089
GGCGTCGCTGGGA	0.052	0.014	0.072	43	554
CGTGTCACTGATG	0.026	0.009	0.023	18	764
GCTATTGTAAGCG	0.051	0.003	0.051	66	1228
CCTGTCATTTACG	0.023	0.005	0.017	27	1577
CGCGTTATATATG	0.025	0.006	0.018	29	1616
GCCAGCGCAGGCG	0.042	0.009	0.032	27	804
CCCGTCACATGCG	0.025	0.006	0.023	43	1843
ACTATTATTTAGA	0.031	0.005	0.029	87	2873

CCTGGTGTATAGG	0.025	0.007	0.017	15	880
CCTGGTGCTAAGA	0.026	0.004	0.024	32	1314
GGTGTTATATGTA	0.077	0.023	0.058	61	985
GGTGTCACTTGGG	0.1	0.012	0.096	32	301
CGTGGTATATGGA	0.021	0.005	0.025	18	689
ACCGGCATAAGGG	0.023	0.004	0.023	25	1071
GGTGTCGTTTAGA	0.094	0.009	0.082	41	460
GGTATCATATGCG	0.125	0.014	0.121	91	661
GCCATTGCTGGTA	0.044	0.008	0.033	34	1010
CCCAGTGTTAATG	0.025	0.001	0.023	49	2071
AGCAGCACAAGCG	0.024	0.004	0.023	45	1885
CGCGGCATTAGTG	0.022	0.006	0.028	21	727
GCTATTATTAATG	0.067	0.005	0.065	139	1993
CGTGTCACTTATG	0.023	0.004	0.019	16	823
CGTAGCGCTGGTA	0.026	0.006	0.024	16	661
CGTAGTGTAAATG	0.024	0.003	0.027	31	1115
ACTAGCGTAGGGG	0.022	0.012	0.01	6	609
CGCGGTACAGATA	0.025	0.002	0.023	41	1751
AGCGTCGTAAGGA	0.027	0.006	0.019	26	1328
ACTGGCGTATATA	0.03	0.002	0.03	43	1385
GGTATCATAAGGA	0.123	0.02	0.128	140	957
ACCGGTGTAGATA	0.026	0.004	0.021	43	2041
CCTATTACTAGGG	0.026	0.003	0.022	28	1218
GCTATCATTGGGG	0.066	0.012	0.056	34	568
GCCATCATTAGGG	0.072	0.004	0.074	74	922
GCTGTCGCAGGGG	0.041	0.018	0.045	12	252
GGTGGTATTTACG	0.272	0.052	0.227	169	577
ACTGGCGCATATA	0.019	0.003	0.023	26	1083
CGTGGTGTTGGGG	0.022	0.013	0.026	6	223
CCTGTCACTGAGA	0.023	0.004	0.024	37	1478
CGCATCGTAAGTG	0.025	0.002	0.023	37	1539
ACTAGTGTAAGTA	0.024	0.004	0.02	48	2364
CGCATCACAAGGA	0.022	0.002	0.026	50	1877
CCTAGTGCTTGCG	0.028	0.003	0.029	27	899
CGTGTTGCATAGG	0.026	0.007	0.032	19	581
CCTAGCGTATGCG	0.025	0.003	0.022	26	1155
CCCGGCACAGAGG	0.029	0.001	0.03	40	1310
ACCATTGCATAGG	0.024	0.001	0.024	50	2010
CCTGTTGCATGGG	0.033	0.003	0.037	28	727
CCTGGTGTTAGCG	0.026	0.006	0.018	16	896

ACTGTCGCAAATG	0.03	0.003	0.028	50	1767
AGTGGTACTAGCG	0.017	0.005	0.015	10	672
GCCGTTATTTGGG	0.045	0.008	0.043	31	689
GCTAGTACAAGCG	0.064	0.007	0.055	62	1069
ACTGTCGCTAAGG	0.021	0.001	0.022	25	1090
GCTATCACTGACG	0.065	0.008	0.055	78	1334
GGCGTCATATGCG	0.068	0.005	0.062	59	889
CGCAGCGTTTAGA	0.022	0.008	0.011	12	1128
AGTGTTACTTATG	0.025	0.006	0.028	28	970
CGTAGCGTAAATA	0.028	0.005	0.022	36	1584
AGCAGTGTAGACG	0.022	0.003	0.018	31	1720
GCCGGCACTTACA	0.103	0.005	0.109	206	1691
CCCAGTACTGGTA	0.025	0.003	0.02	46	2221
AGCGTTGCAAAGA	0.026	0.001	0.024	50	2006
AGCGGCACTAATG	0.025	0.002	0.024	31	1253
GCTGTCATAGACA	0.064	0.008	0.052	84	1522
AGTGGCGTAGGCA	0.018	0.003	0.014	9	618
AGCGGCGCATAGA	0.022	0.002	0.019	21	1084
CCTGTCGCATATG	0.028	0.005	0.033	42	1213
AGCAGTACAGGTA	0.024	0.001	0.023	48	2053
CCCGGTGCAGACG	0.025	0.006	0.029	42	1422
ACCAGTGTTGGGA	0.026	0.004	0.021	26	1233
CCTGTTACATAGA	0.023	0.005	0.017	31	1761
CGCGGTACAGGGA	0.024	0.004	0.029	28	922
GGCATTGTTTAGA	0.068	0.005	0.06	74	1154
AGTAGTACTTGCG	0.022	0.004	0.022	18	807
CGTAGCGTATAGG	0.027	0.009	0.024	17	694
GGTAGTGCTAGCG	0.056	0.015	0.035	19	527
CGCGGTGTAGAGG	0.025	0.007	0.015	10	636
CGTATTGCAAGCA	0.028	0.004	0.024	38	1574
GCCGGTGTTGACG	0.401	0.059	0.41	422	608
GCCGGCACAGGGA	0.112	0.014	0.095	89	846
CCTGGTATAAATA	0.032	0.002	0.036	107	2898
GCTGGCGCAAATA	0.646	0.037	0.638	1291	734
AGTAGTGCAGATG	0.023	0.003	0.019	17	892
ACCGTCACTGGTA	0.022	0.002	0.024	54	2241
CGTATTGCATAGG	0.026	0.006	0.019	16	841
ACCGGCACATGGA	0.026	0	0.026	48	1833
GCCGGCATTAGTG	0.746	0.03	0.746	988	336
AGCGGTACTAGCG	0.021	0.005	0.02	21	1027

GGCGTCACTTAGG	0.058	0.007	0.05	36	680
CCTAGCGTTGATA	0.022	0.003	0.02	34	1702
ACCGGTACAAGTG	0.022	0.002	0.02	46	2216
AGTGTCGTTGGGA	0.014	0.004	0.017	10	573
GGTAGCGCAGACG	0.084	0.01	0.083	53	589
CGCGTTGCTAAGG	0.025	0.004	0.03	27	881
CCTGTTGCATAGG	0.026	0.006	0.03	30	966
GCCAGCGCAGGTG	0.056	0.011	0.071	48	631
GCCATTGCTTGGG	0.051	0.006	0.057	36	595
GCTGTCGTATATA	0.057	0.009	0.053	63	1125
GGTAGTACAAGCG	0.062	0.014	0.047	37	749
AGTGGCGCTAATG	0.021	0.003	0.02	13	636
GCTAGTGTTAATG	0.062	0.007	0.052	68	1241
GCCGGCGCATACA	0.501	0.041	0.498	939	945
ACCGGTGTTGATG	0.03	0.002	0.028	35	1198
AGTATTGCAGATG	0.024	0.003	0.022	28	1221
AGCGGTGTTGGGG	0.03	0.018	0.056	29	492
CGTAGCGCTTGCG	0.032	0.01	0.023	12	515
ACTGGCGTAAGCG	0.03	0.003	0.025	14	539
AGCGTTACTAGGA	0.023	0.002	0.022	30	1353
GGCGTCACAAAGG	0.061	0.001	0.06	67	1054
GGCATCGTATACA	0.102	0.022	0.084	184	2017
GGTAGCATAAGCA	0.065	0.013	0.048	55	1088
CGCGTTGCAGAGA	0.024	0.004	0.019	23	1180
CGTGGTGTTAATA	0.026	0.002	0.028	28	966
CGCGGCGCATGCG	0.028	0.007	0.019	17	880
CCTGGCATTAGGA	0.024	0.002	0.021	22	1042
CCCGTCGTTAACA	0.022	0.004	0.027	80	2923
GCTAGCGTTAGGG	0.072	0.008	0.081	43	485
CGTGTTGCAGGCG	0.028	0.003	0.025	17	654
AGTATCATTTAGA	0.02	0.004	0.018	31	1703
AGTATTGTTGATA	0.023	0.004	0.018	34	1831
CGCGTCATTGAGG	0.03	0.01	0.044	39	845
AGTATTGCTGACA	0.023	0.004	0.027	47	1667
CCCATCATTAGTG	0.025	0.008	0.014	33	2273
AGTATCATAGGTG	0.024	0.006	0.017	19	1105
GCTATCGTAAGTG	0.074	0.01	0.061	65	1007
AGTGTTGCTTGCG	0.029	0.009	0.028	16	563
CCTGGCGCTTGTA	0.019	0.005	0.018	17	946
AGCGGTGCATACA	0.027	0.008	0.018	33	1815

CCTATTATTTGTA	0.028	0.002	0.026	63	2377
AGCGGCGTTGACG	0.027	0.008	0.022	18	802
ACCGTTATTTGCG	0.024	0.001	0.023	33	1422
ACTGTTGTAGGTA	0.028	0.001	0.028	35	1225
GGCATTACTTGGG	0.062	0.009	0.062	45	676
CGTGTTGTATAGA	0.023	0.008	0.035	31	863
CGCATTACATATG	0.026	0.005	0.034	66	1894
GGCAGTGCTAAGG	0.05	0.012	0.05	42	803
AGCAGCGCTGGCG	0.025	0.007	0.022	24	1080
CGTGTCGTAGGCA	0.021	0.003	0.024	22	897
CGCATTGCAGACG	0.026	0.009	0.014	21	1507
GCCGTCATAAAGG	0.065	0.005	0.058	87	1424
ACTATCGTAGGGA	0.026	0.001	0.027	35	1241
AGCAGTGTTAGCA	0.022	0.005	0.022	42	1848
CGTAGCACATGGG	0.018	0.005	0.018	10	539
GCCGGCACTAATG	0.198	0.017	0.188	261	1124
GCCAGTGTTGACA	0.054	0.005	0.048	81	1621
CGTAGTGTAAGGA	0.021	0.003	0.018	15	830
CCCGTTACTGACG	0.03	0.002	0.031	61	1878
AGCGTTGCTTGCA	0.02	0.004	0.014	19	1299
GGTATTACTTACA	0.095	0.015	0.086	133	1411
GGTGTTGCTTGGA	0.058	0.011	0.043	17	379
GGTGGCATATGGG	0.711	0.048	0.689	440	199
CCTGGTACAAGCG	0.03	0.002	0.027	36	1280
GGTAGCATATACA	0.073	0.004	0.071	104	1361
GCCGTCGCATACG	0.05	0.009	0.037	39	1014
GGCGGTGTTAAGG	0.091	0.015	0.071	48	626
CGTGGTACAGGCA	0.023	0.007	0.014	12	864
GGCAGTGCTTATA	0.044	0.005	0.051	59	1108
CCCGGTATTAGTG	0.02	0.001	0.021	28	1334
CGCATTGTTTGGG	0.028	0.005	0.035	25	696
GGTGTCGTTAAGA	0.092	0.013	0.079	57	663
AGCGTTATAAAGA	0.023	0.002	0.02	62	3053
GCCGTTGCTTACG	0.053	0.015	0.033	31	922
AGTAGCACAAGTA	0.026	0.003	0.027	47	1709
AGCGGTATTTACG	0.025	0.007	0.016	22	1366
GCTAGTGCAAGTG	0.055	0.009	0.042	36	817
CCCGGTATTAGCG	0.026	0.001	0.025	37	1428
ACCATTGCTGGTG	0.02	0.003	0.024	39	1577
GGTAGTATAAGCG	0.067	0.008	0.062	55	836

GGTGTTGCATGCA	0.07	0.019	0.081	60	682
GCCGGCGTTTACG	0.925	0.021	0.927	1469	116
GCCATCATATGCG	0.054	0.007	0.047	84	1698
ACTGTTATAGGTA	0.025	0.005	0.019	36	1875
CGTATTGCTAGTA	0.018	0.002	0.019	21	1095
GGTAGCGCTGGTA	0.083	0.036	0.06	30	469
CGCGTCGTATGGA	0.019	0.005	0.026	25	952
GCCGTTACAGGCA	0.054	0.003	0.055	80	1367
GGTAGTGCTGGCG	0.045	0.01	0.059	28	444
GGCAGCGTTGACG	0.068	0.002	0.065	68	973
AGTATCGTTGGGG	0.027	0.004	0.029	16	539
GCTATCACTTGTA	0.056	0.012	0.044	57	1248
CGTGGCATATATG	0.023	0.011	0.039	26	633
CGCAGCATAGAGA	0.025	0.001	0.024	43	1786
CCTGTTGCTTGGA	0.019	0.008	0.018	16	865
GCCGGTGCAGGGA	0.058	0.011	0.048	34	672
GCCATTACAGGTG	0.058	0.001	0.057	71	1165
ACTGTCGTAGAGA	0.024	0.005	0.018	21	1160
ACTAGCGTTTGTG	0.04	0.004	0.035	28	764
AGCGGTGTATAGA	0.023	0.001	0.025	34	1334
CCCGGCGCTGATA	0.025	0.004	0.031	56	1764
AGCAGCATTAGGG	0.019	0.005	0.017	25	1451
GGCGTCACTTACG	0.075	0.012	0.064	71	1031
GCTAGTGTATGCG	0.058	0.007	0.055	54	919
GCCGTCACAAGTG	0.057	0.005	0.051	66	1229
CGTGTCGCAAATA	0.028	0.004	0.034	52	1500
GGCGTTGTATAGG	0.083	0.011	0.069	48	652
CGCGGCACAAGTA	0.024	0.006	0.02	35	1695
ACTAGTACTAGTG	0.024	0.004	0.019	33	1740
AGTATCACTGAGA	0.029	0.005	0.028	38	1314
ACTAGTACATGGG	0.022	0.003	0.017	23	1328
CCCAGCACTTGGA	0.028	0.002	0.025	43	1675
AGTGTCACATGCA	0.019	0.007	0.028	36	1256
GCTATTGTATGTG	0.062	0.006	0.07	69	921
CCCGGCGTATACG	0.023	0.002	0.022	34	1543
CCCGTTATATAGG	0.026	0.006	0.019	34	1735
CGCAGTGTTTGTA	0.029	0.003	0.024	29	1170
CCCGGCGCTTAGG	0.017	0.001	0.016	15	912
AGTGTCGCTAACG	0.023	0.002	0.021	22	1019
GGTGGCACATACA	0.208	0.047	0.193	217	908

GGTGGCATTTATA	0.482	0.05	0.457	574	682
AGTGTCATTTATA	0.025	0.003	0.021	34	1623
CGCGTCGCTAATG	0.025	0.004	0.021	24	1130
GGCATTGCATGTA	0.054	0.008	0.043	54	1205
ACTGTTGTTTACG	0.029	0.007	0.024	30	1246
CGTGGCACAGAGG	0.015	0.005	0.019	10	526
GCTAGTGTTGATG	0.067	0.002	0.064	61	890
ACTGGCACAAAGA	0.025	0.004	0.021	42	1984
GGTAGCGCTTGTA	0.057	0.004	0.052	30	548
GGTGTCATTAACG	0.101	0.013	0.088	71	736
AGCAGCGCAGACG	0.024	0.005	0.019	30	1521
CCTAGCACTGGTG	0.029	0.004	0.024	23	945
CCTATTGCTAGGA	0.025	0.003	0.022	36	1590
CGCGTTGCATGGG	0.021	0.005	0.028	18	636
ACCGTCATTTGGA	0.026	0.004	0.023	40	1717
CCTAGCACAAGGA	0.03	0.003	0.027	50	1773
GGTAGCATAAACG	0.078	0.013	0.06	73	1140
GGCAGTGCTGGTA	0.036	0.002	0.034	27	760
GCTAGTGTTTGCG	0.05	0.008	0.045	34	719
CGCGTCGTTAGGG	0.021	0.005	0.013	8	587
GCTGTCGCATACG	0.052	0.016	0.03	23	749
CGCGTTACATACA	0.022	0.003	0.026	66	2497
CCTAGTGTTTAGA	0.026	0.003	0.03	45	1438
GGTAGCACTAATG	0.076	0.005	0.075	64	790
CCCATCGCTTACG	0.024	0.002	0.024	51	2033
ACTAGCGCATGTG	0.026	0.006	0.033	40	1180
CGTATTATTAAGA	0.026	0.001	0.026	57	2111
CCTGTTATTAGGG	0.026	0.006	0.02	18	869
ACCGGCGTTGATG	0.022	0.004	0.027	20	726
AGTGGTACAGGCA	0.025	0.002	0.022	22	983
GGCGTCACTAGCA	0.067	0.013	0.054	72	1251
AGTGGTGTAGGTG	0.02	0.002	0.022	10	444
GGTAGTGTAAGCA	0.062	0.011	0.046	42	864
ACTATTGTAAGTG	0.034	0.007	0.029	56	1859
ACTATTGTTGGTA	0.024	0.003	0.028	48	1658
CGTGTTGTTGAGG	0.02	0.014	0.026	12	448
AGTAGTATTAGCA	0.023	0.003	0.019	32	1655
GGCGTCATTGGTG	0.056	0.01	0.045	31	651
GCTATTGCTTAGG	0.066	0.009	0.067	50	694
CCTGTTACAGAGG	0.026	0.004	0.031	32	994

GGCATCGCAAATG	0.1	0.006	0.099	170	1542
GGTAGTGCAAGGA	0.053	0.017	0.051	30	556
CGTGTTGTTAGTA	0.028	0.006	0.022	19	860
GGCATCACATACG	0.089	0.009	0.086	161	1710
CGCGGTACATATA	0.022	0.004	0.019	38	1917
AGCAGTACAGACA	0.019	0.001	0.019	65	3350
ACTGGCACAGATA	0.032	0.003	0.028	60	2058
CGCATCATTAGCA	0.026	0.002	0.024	61	2479
ACCGTCATAGGTG	0.033	0.008	0.044	61	1323
AGTGTCGCTAGCA	0.023	0.003	0.025	22	851
CGCGGCGCATACA	0.022	0.004	0.017	27	1585
CCCGTCACATGGG	0.034	0.002	0.037	44	1149
CCCATTATATAGG	0.026	0.003	0.023	58	2448
ACTATCACTAGGG	0.029	0.007	0.038	50	1273
AGTGTCGTAGGTA	0.016	0.008	0.013	11	859
CCTGGTGTATAGA	0.023	0.006	0.015	19	1264
GCTGGTATATACG	0.448	0.049	0.435	543	704
ACTATTGCTTACA	0.024	0.002	0.023	71	3018
ACCGTTGTTAGTG	0.027	0.004	0.029	35	1178
GCTAGTATAGGCG	0.068	0.008	0.065	72	1035
GGCGTTATAGGGG	0.052	0.005	0.058	46	745
ACTAGCGCTAACG	0.022	0.003	0.019	32	1669
CGCAGCACTAAGA	0.021	0.004	0.022	42	1855
GCTGTTGCAAGGG	0.053	0.003	0.057	30	493
GCCAGCGTAAGGG	0.068	0.01	0.079	59	684
CCTAGCGCTTGGG	0.026	0.006	0.036	21	570
GCCGTCGTATGCA	0.055	0.006	0.055	66	1137
GCCGGCATTTATA	0.398	0.037	0.386	745	1184
GGCGGTACTAAGA	0.064	0.006	0.057	67	1101
CCCAGCGTTTGGG	0.021	0.002	0.018	17	912
CGTGGCGTAAGGA	0.027	0.004	0.02	12	583
CGCGTCGCATGGA	0.025	0.001	0.024	23	919
GGTAGTGCAGGTA	0.044	0.002	0.042	28	634
CGTATCACTAATG	0.031	0.008	0.021	31	1419
AGCAGTGCTAGTG	0.018	0.002	0.017	20	1191
ACCATCGTTAATG	0.024	0.001	0.024	57	2326
GGCGTTGTTAACA	0.065	0.002	0.065	97	1406
CGCAGTGCTAGCG	0.022	0.007	0.022	22	957
GGCATCGCAGAGA	0.084	0.013	0.069	84	1130
GCCAGTGTTGAGA	0.049	0.005	0.041	48	1111

GGTGGTATAAACG	0.276	0.046	0.252	282	837
GGTGGTGTTAACA	0.114	0.004	0.112	96	762
GGTGTTACAAAGG	0.087	0.012	0.072	58	746
CCTGTTGTTGGGG	0.029	0.005	0.029	17	560
GCCGTTACATAGA	0.057	0.008	0.062	101	1517
GGTAGTATTTGCA	0.064	0.009	0.054	52	903
ACTGGTGCTTGGA	0.02	0.009	0.008	7	922
GCTATTATATGTG	0.062	0.007	0.052	75	1357
ACTAGTATAGGCG	0.021	0.004	0.016	26	1611
ACCGGTGCAAGCA	0.02	0.001	0.019	48	2452
GGTGTCGCTGAGA	0.073	0.025	0.059	31	494
ACCGGCATTAGGA	0.024	0.002	0.027	41	1501
CGTGTCATAGGCG	0.021	0.001	0.02	17	852
CCTATTACAAGCG	0.024	0.002	0.024	57	2318
GGCGTTGTTAAGA	0.063	0.003	0.065	72	1039
GCTGTTGCAAAGA	0.045	0.003	0.047	51	1034
AGCAGCACTAGTG	0.019	0.003	0.024	35	1449
AGCGTTATATAGG	0.021	0.002	0.02	29	1445
AGTAGTGTAAATG	0.022	0.001	0.02	31	1502
CCTATTACATGGG	0.028	0.004	0.026	33	1218
AGTATTGCAGGCG	0.022	0.004	0.026	28	1063
ACTGGCGTATACA	0.028	0.004	0.023	35	1485
CGTGGTATTTAGG	0.022	0.006	0.028	12	415
AGCGTCACTAGTA	0.019	0.003	0.019	35	1764
GCCGGCACAGATA	0.109	0.018	0.114	206	1596
CCCATTATTTGCA	0.022	0.002	0.024	72	2965
GGTAGTGTTGGTG	0.069	0.031	0.049	18	352
AGCGTTGTAAGCG	0.019	0.005	0.017	25	1445
AGTAGTGTAGACA	0.02	0.004	0.016	26	1617
CCCAGTATATATG	0.031	0.004	0.03	87	2826
ACCGGTGTATGGG	0.023	0.007	0.016	14	858
GGTAGCGCAAGGA	0.068	0.015	0.048	33	661
GGCAGTATAGGCG	0.051	0.012	0.044	48	1053
AGTGGCATTAGTG	0.019	0.011	0.01	2	205
ACCGTCGCTAGCG	0.023	0.004	0.016	24	1431
CCTAGTGCTTGCA	0.024	0.004	0.02	31	1537
GGTATTGTATGCA	0.095	0.009	0.089	92	940
ACTAGCGTTGGGA	0.031	0.006	0.032	28	843
AGTAGTATTGATG	0.021	0.002	0.019	25	1269
GCCGGCGTTAACA	0.858	0.021	0.84	2241	427

AGCGTTGTATGGG	0.023	0.004	0.018	15	802
GGTGTTGTTGAGA	0.103	0.012	0.101	55	488
AGTGTTACTTAGA	0.021	0.008	0.011	13	1163
ACCGGTACTAAGG	0.023	0.007	0.017	34	2024
AGTATCGCTTACG	0.023	0.008	0.03	34	1085
AGCGTCATTAGCG	0.023	0.007	0.015	22	1411
CCTGTCGTATGTG	0.027	0.004	0.023	24	1021
ACCGTCGTAGGTA	0.026	0.004	0.023	35	1458
CCTGGTACTGAGA	0.029	0.001	0.028	35	1223
AGTGGCACAGATA	0.023	0.005	0.028	35	1204
CGTAGCGCTTGCA	0.028	0.007	0.026	22	814
CGCGTTGCTTGGG	0.024	0.007	0.015	8	538
AGCATTGTTTGGG	0.024	0.006	0.018	17	918
GCTAGCATTAATG	0.07	0.01	0.068	97	1331
CGTGGTGTTAACG	0.029	0.008	0.019	13	687
ACCATCGCTGGCG	0.027	0.003	0.023	37	1585
AGCGTCATTGGCG	0.021	0.002	0.022	26	1137
GGCATCACTGGGA	0.065	0.007	0.056	53	898
GGTGTCGTTGATA	0.084	0.022	0.057	46	763
GCCAGCGTAAGCG	0.05	0.008	0.039	46	1131
CCTAGCACTTAGG	0.032	0.006	0.026	27	1025
GGTGGCATTGGGG	0.658	0.074	0.597	308	208
GCTGGTGTATACG	0.746	0.038	0.73	915	338
CCTGGCACTGGGG	0.04	0.004	0.043	25	556
GCCGTCATTTGCA	0.064	0.015	0.049	76	1462
CCTATTACTGGTG	0.026	0.006	0.023	29	1234
AGCGGTACTGACA	0.022	0.002	0.022	45	2004
AGTGTTACAGAGA	0.029	0.011	0.029	36	1213
GCTGTTATAGATG	0.066	0.006	0.057	62	1018
GCTGTTACAGGGA	0.063	0.009	0.051	36	673
CGTGTTACTAGGA	0.022	0.005	0.019	15	782
GCCGTTGCAAGGA	0.045	0.007	0.039	39	957
ACTGGTATAGAGA	0.025	0.003	0.02	34	1644
CGTGTCGCAGGTA	0.037	0.013	0.037	27	705
AGTAGTACATGTG	0.024	0.003	0.027	28	1008
CGCATTATTGGTG	0.023	0.005	0.018	21	1140
CCCAGCATTGGTA	0.028	0.003	0.026	53	1982
GGTATTGCATACG	0.097	0.012	0.079	78	904
AGTAGTGCATACG	0.025	0.003	0.024	26	1043
CCCGTCATTGACG	0.032	0.003	0.028	54	1895

CCTGGCATTTATG	0.03	0.006	0.026	29	1073
GGTGGTACTGACG	0.09	0.006	0.089	53	543
AGTAGTACTAAGG	0.024	0.004	0.021	24	1100
ACTATCGTAAGTA	0.026	0.001	0.024	55	2191
ACTAGCGTTGATA	0.031	0.004	0.029	50	1697
ACTAGTATTGGGG	0.024	0.004	0.02	16	804
GCCAGCATATGGA	0.054	0.007	0.051	75	1398
GCCATCACAGGGG	0.07	0.005	0.065	62	894
AGCGGTATAGATG	0.018	0.002	0.019	27	1410
GGTGGCGCTGACG	0.288	0.044	0.262	137	385
ACCATCACTAGGA	0.024	0.002	0.026	71	2647
CGTAGCGTTGATG	0.021	0.006	0.028	19	648
ACTATCGTATGGG	0.027	0.005	0.032	30	897
GGCGTTGCTTAGA	0.053	0.008	0.044	31	673
CCCGTCGCAAGGA	0.026	0.001	0.027	50	1813
GCCGTTACATGTA	0.057	0.008	0.048	78	1549
AGCGTCGCAGAGG	0.023	0.008	0.015	16	1019
CGTAGTGCTAAGA	0.022	0.005	0.022	20	909
GGTGTTGTTTGGG	0.089	0.015	0.089	26	265
GGCGTCGTAAATA	0.067	0.006	0.059	104	1645
GCCGTTACTGAGA	0.058	0.007	0.056	72	1225
CCTGGTGCAGAGG	0.03	0.006	0.023	18	757
CCCATTGCTAGGG	0.021	0.002	0.017	23	1310
GGCGTCGTAAGTA	0.065	0.014	0.046	49	1025
GGTGGCACTTATG	0.385	0.041	0.354	252	459
CGCGTCATTAATG	0.023	0.006	0.017	28	1598
CCTAGCGCAGACG	0.022	0.002	0.026	40	1510
CGTGGTATATACA	0.03	0.004	0.026	38	1451
GGCAGCATATAGA	0.058	0.016	0.038	64	1607
CGTGGTACTAGTA	0.023	0.005	0.02	17	847
AGTGGTATATGCG	0.032	0.004	0.028	19	667
AGTATTGTTGAGA	0.022	0.004	0.016	21	1265
AGCATCGCTTAGG	0.019	0.005	0.013	16	1180
GCCGTTATTGGTG	0.057	0.008	0.058	50	817
CGCGTTATAGGGA	0.028	0.002	0.031	31	954
ACTATCACAGGTG	0.025	0.003	0.023	41	1715
GGCGGTGCATGGA	0.042	0.015	0.027	16	582
AGTGTTGTAAAGA	0.023	0.002	0.02	30	1462
CCTGTCACTAAGG	0.022	0.003	0.019	22	1111
ACTAGTGTTAAGA	0.027	0.004	0.025	54	2111

AGTGTCGCATATA	0.025	0.005	0.019	25	1261
CGTATCACAGAGG	0.028	0.004	0.024	22	879
GCTATCACTGGGA	0.062	0.016	0.041	34	795
ACTAGCGCAGGTA	0.021	0.003	0.021	37	1729
GCTGTTGTATGTG	0.075	0.007	0.082	57	636
AGCGGTGTTAACG	0.025	0.002	0.026	36	1325
ACCGGTATTAGCG	0.02	0.005	0.025	39	1536
CCCAGTGTATGGA	0.019	0.003	0.02	30	1477
AGCAGCGCTAGGA	0.023	0.003	0.024	27	1085
AGTAGCGCAGACG	0.016	0.001	0.018	16	889
GCTGTTACTTATG	0.058	0.004	0.061	55	844
ACCGGCGTTTGCG	0.023	0.008	0.017	11	623
CCCGTCACTTATG	0.023	0.004	0.022	39	1767
AGCAGCATAAGTG	0.02	0.005	0.015	31	2047
ACTGGTGCTAGCA	0.023	0.004	0.019	30	1555
GGTGGTGCAGAGA	0.08	0.015	0.059	32	508
CGCGGTATTGGGG	0.029	0.003	0.034	17	489
CGTAGCGCAGGGG	0.027	0.017	0.014	6	410
GCTGTTATTGGCA	0.057	0.01	0.059	60	956
CCCAGCATATGGG	0.045	0.005	0.052	74	1351
AGCAGCGTTAAGG	0.019	0.005	0.013	17	1275
ACCGGTACTTGTG	0.021	0.004	0.024	39	1589
CGTGTCGTTTGTG	0.023	0.004	0.029	16	543
GCCGTTACTAGTG	0.053	0.007	0.043	44	974
GCCATCATAGATG	0.054	0.007	0.05	121	2292
AGCGTCGCATGTA	0.023	0.004	0.018	27	1489
CGCGGCGCTAACG	0.022	0.001	0.021	24	1102
CGTAGCGTTTAGG	0.027	0.007	0.03	16	520
GCCGGCGCTTAGA	0.493	0.045	0.481	464	500
CCTGGTGTTTATG	0.026	0.005	0.02	19	939
AGTGGTATTAATG	0.021	0.001	0.019	20	1006
GCTAGCGCTTGGA	0.062	0.012	0.077	43	512
AGCGGCATATACG	0.016	0.006	0.015	12	769
CCCGTTACTAGGG	0.028	0.003	0.024	27	1089
CGTAGTGCAGGGG	0.027	0.005	0.029	12	407
CGTGGTATTTGTG	0.016	0.003	0.014	7	498
GCCAGTGCAAACA	0.045	0.009	0.055	135	2304
GGTAGCGCTGAGA	0.107	0.017	0.108	56	462
AGTAGCATAAGGA	0.023	0.002	0.025	37	1438
CGCGGCGTTAGTG	0.02	0.002	0.018	11	585

GGCGGTGTTGAGA	0.065	0.014	0.047	31	635
ACTGGCGCAAACG	0.03	0.005	0.023	30	1272
GGCGGTGCAAGTA	0.06	0.002	0.057	53	877
GGTGTTGTTTGCA	0.076	0.024	0.044	24	519
AGTAGTGTAAAGA	0.019	0.004	0.013	22	1731
AGTATCACAGGGG	0.026	0.006	0.019	14	704
GGTGGCGTATGTG	0.656	0.043	0.6	406	271
GCCATTGCTGGGA	0.052	0.002	0.054	46	809
CGCGTCGTTTACG	0.021	0.006	0.015	17	1135
GGTGGCGTATGCA	0.379	0.058	0.348	243	456
GCCAGTATTGGTA	0.052	0.003	0.056	94	1591
AGTAGCACTTGTG	0.029	0.011	0.019	15	756
GCTGGCATTAAGA	0.641	0.049	0.62	957	586
GGCAGCGCAGGGG	0.08	0.008	0.083	51	560
GCCATCGTTTACG	0.062	0.011	0.06	84	1307
CCTGTCGCTTGTG	0.024	0.005	0.021	15	698
ACTGTCGCTGATA	0.024	0.003	0.021	36	1653
CGCAGTACAAATG	0.027	0.003	0.023	51	2138
CCCATTACTGGGA	0.033	0.001	0.032	51	1539
GGTATTATTTATA	0.104	0.014	0.097	192	1795
GCCGGCGTAGGGA	0.806	0.012	0.79	876	233
CGCGGCATTTGTA	0.024	0.003	0.02	25	1210
ACTGTCACAGGCA	0.023	0.004	0.018	36	1999
GGCAGCGTTGGGG	0.051	0.014	0.043	23	509
AGCGTTGTTTGGA	0.02	0.008	0.015	14	915
GGTGGTACTAGGA	0.081	0.024	0.062	30	456
CCTGGCACTTGTG	0.028	0.004	0.031	27	843
GGCGTTGCTTACA	0.055	0.009	0.055	62	1062
AGTGTCGTAAGTA	0.028	0.007	0.021	25	1160
ACTGGCGCTAATA	0.021	0.005	0.015	16	1062
CGTGGTACTGACA	0.021	0.001	0.022	26	1152
GGCGGCATATGCG	0.553	0.075	0.518	605	564
AGTATCGTTAAGG	0.026	0.004	0.031	34	1048
CGTGTTGTTAATA	0.025	0.004	0.028	36	1248
CCTGTCACTGATA	0.024	0.004	0.018	35	1922
GGTGTCACTGAGG	0.099	0.014	0.114	49	381
CGCGGTGCAGGCA	0.018	0.004	0.017	20	1173
ACCGTTGCAAGCG	0.023	0.006	0.016	31	1871
GCCGTTGCTTGTG	0.044	0.017	0.062	32	483
ACTGGTACAGGTG	0.027	0.008	0.024	25	997

GCTGTCGCAGATG	0.058	0.011	0.067	44	608
GCTGTCACTAAGA	0.051	0.005	0.052	60	1097
ACCAGCGCAGGGG	0.024	0.006	0.015	13	844
AGCGGTGTATGGG	0.024	0.004	0.019	11	576
GCCGGTATAGGTA	0.071	0.006	0.079	108	1258
GCTAGCACAAAGG	0.065	0.006	0.058	65	1062
GCTGTTGTTGAGG	0.06	0.008	0.05	29	548
GGCAGTATTGGCA	0.046	0.006	0.044	60	1301
CGCGTCACAGGTG	0.024	0.004	0.019	18	940
ACTAGCGTAAGGG	0.036	0.009	0.034	32	898
GGTGGTATTTAGA	0.132	0.008	0.126	80	557
GCCATTATAAGGG	0.054	0.007	0.051	75	1408
GGCATTATTAGCG	0.07	0.006	0.072	97	1257
AGCATTGTATGTG	0.024	0.004	0.022	34	1518
CCCATTGCAAAGG	0.025	0.003	0.02	45	2177
CCCATCGCTAGTA	0.025	0.007	0.015	38	2414
GCTAGCGTATGTA	0.051	0.004	0.046	47	972
ACTGGTGCAGATG	0.028	0.008	0.02	23	1104
AGCAGTGTTGGTG	0.024	0.008	0.015	14	926
AGTGTTATTGGTG	0.025	0.002	0.025	18	709
ACCAGCATTTGGA	0.022	0.004	0.018	36	1932
CCTGGTGCTAACA	0.025	0.002	0.024	46	1897
ACCGTTACAGGTG	0.022	0.002	0.026	43	1623
GGCATTACAAGGA	0.061	0.013	0.045	72	1531
GGTGTCGCTAGCA	0.079	0.005	0.076	48	581
GCTATCACTAGCA	0.054	0.008	0.048	82	1616
GGTGGCGTTAGTG	0.656	0.041	0.667	364	182
CCCGGCGTATAGG	0.027	0.003	0.03	30	973
GGTGGCACAAGTG	0.4	0.031	0.386	277	440
GGTGGTACTTGTG	0.101	0.023	0.09	38	386
CGTATCGTTAGCG	0.021	0.005	0.019	17	883
CGTATCGTAAAGG	0.021	0.005	0.015	18	1179
GGTGTTACTGAGA	0.065	0.013	0.053	38	676
GCCGGTGCTGACG	0.111	0.016	0.091	82	818
GCTATCATAAGTG	0.062	0.004	0.061	91	1396
CGCAGCACAGATA	0.021	0.004	0.017	37	2183
CCTGGTGTTAATA	0.023	0.001	0.022	40	1745
CCTGTCATAGGTG	0.025	0.002	0.025	27	1046
ACCATCGCTTAGG	0.022	0.001	0.021	31	1434
GGTATTGCATGGG	0.082	0.019	0.097	49	454

AGCATCGTAGAGA	0.02	0.006	0.022	47	2055
CGCGGCGCATATA	0.026	0.002	0.025	35	1385
GCTGTTACAGAGG	0.061	0.014	0.041	27	629
AGCGGTGCAAGTG	0.019	0.001	0.019	18	933
CGTGGTACTAGGA	0.026	0.011	0.018	14	744
GCTGTCACTTACG	0.067	0.007	0.061	57	885
AGTAGCACAGGTG	0.029	0.005	0.035	26	727
ACTATCACAGGCA	0.024	0.002	0.022	60	2717
CGTGGTGTAAGGA	0.017	0.004	0.022	15	655
GCTGTCACAGAGA	0.056	0.006	0.048	46	909
GGTGTTATATGCG	0.087	0.018	0.076	48	585
GGCATTGTTTGGA	0.077	0.008	0.087	73	769
ACCGTCGCTGATG	0.023	0.002	0.023	33	1375
CGCGGTGTTAACA	0.021	0.003	0.023	37	1579
AGTAGCGCAAGGA	0.026	0.004	0.031	29	910
ACCGTCATAGGGA	0.027	0.003	0.024	33	1343
AGTAGTGCATGTA	0.028	0.006	0.032	36	1075
GCTAGTACTTATA	0.061	0.007	0.071	116	1517
CGTAGTGCAAAGG	0.023	0.005	0.026	19	702
GGCGTCGCAAGCG	0.075	0.012	0.059	56	894
CGCAGCGTATGGG	0.03	0.011	0.043	30	675
CGCGGCATAGACA	0.023	0	0.022	45	1991
GGCAGCATTAGGA	0.048	0.013	0.032	35	1054
GGTGGCGTTAGTA	0.395	0.044	0.348	209	392
GCTAGTATATATA	0.058	0.006	0.056	135	2282
AGCGTCATTAGGG	0.025	0.002	0.022	23	1001
GGCGGCGTAGGGG	0.24	0.045	0.268	145	397
CGTATCGCTAGCA	0.027	0.004	0.027	35	1262
GGTAGCACTGGGG	0.069	0.004	0.067	24	332
ACCGTCGTAGAGA	0.026	0.002	0.024	35	1445
CCTAGTACAGAGA	0.027	0.002	0.025	49	1943
ACCGGCGTAGGCG	0.026	0.006	0.029	19	632
CGCGTTGTTGATG	0.028	0.006	0.02	19	934
GGCATCATATGCG	0.082	0.007	0.075	108	1334
GGCGTCGCTGAGA	0.051	0.003	0.051	40	742
GGCAGTGTATGTA	0.051	0.001	0.052	65	1183
ACCGTTATTGGGG	0.025	0.006	0.032	27	819
CGTGTTGTTTGGA	0.03	0.011	0.022	12	525
CGTAGCATAGATG	0.025	0.003	0.03	30	986
GGCATTGCTTACG	0.069	0.005	0.071	81	1062

CGTGGCGCTAGTG	0.021	0.002	0.023	11	471
GCCGTTACAGGCG	0.062	0.007	0.051	54	1000
ACTGTTGTTTGGA	0.031	0.01	0.018	16	879
GGTATTATAAGCG	0.115	0.019	0.101	119	1065
CGTGGCGCATATG	0.024	0.001	0.024	14	565
AGTATCGCAAACA	0.022	0.002	0.024	57	2326
GGCATTGCTGGGA	0.061	0.013	0.054	34	594
CCCAGCGCTAACG	0.024	0.007	0.014	32	2183
GGTGGTATTTATA	0.143	0.027	0.137	122	769
GCCGTTGCTAACG	0.052	0.006	0.06	69	1074
CGCAGTGCTAGCA	0.026	0.004	0.024	33	1325
GCTAGCACTAGCA	0.064	0.004	0.061	81	1257
CGTAGTGCAGGTA	0.021	0.008	0.012	8	671
GCTGTCATAAGGG	0.063	0.01	0.061	43	667
CGCAGTGCTGAGG	0.029	0.011	0.023	17	719
GCTGGCACTTGCA	0.212	0.009	0.206	186	719
GGCGGCACTAACA	0.14	0.03	0.12	215	1575
GGCGGCATATAGG	0.509	0.081	0.475	540	597
AGCGTCGTAGAGA	0.021	0.001	0.021	30	1373
ACCAGCGCTGGGG	0.02	0.003	0.025	21	836
GCTGTTGCATACA	0.049	0.006	0.041	50	1179
AGTATTGTAAGGA	0.022	0.002	0.02	30	1453
CGTGGTGTTAAGA	0.024	0.004	0.023	19	802
AGTAGCGCAAGTG	0.031	0.007	0.033	28	822
CCCGTTGTTTGTG	0.035	0.003	0.037	40	1047
GGCGTTACAAAGA	0.063	0.008	0.055	99	1685
GGTGGCACTTACA	0.201	0.052	0.149	136	776
AGTGGTGTTGGTA	0.025	0.002	0.026	16	609
CGTATTGCAAGGG	0.026	0.006	0.034	26	734
CCTGGTGTATGGG	0.018	0.001	0.018	12	656
GCCGTCGCATGCA	0.047	0.007	0.037	41	1056
ACCGGCATTTGCG	0.024	0.004	0.018	22	1231
GGCATTGCATAGG	0.08	0.011	0.065	62	898
GGCGGCATAAGTG	0.561	0.073	0.515	663	625
AGTAGCGCAAACA	0.026	0.004	0.029	54	1801
GCTGGCGTAGATG	0.942	0.016	0.938	1218	80
GGCGGCATAGGTA	0.302	0.038	0.297	317	749
ACTGTCACATGCG	0.023	0.001	0.022	31	1385
GGTGGTGTAAATG	0.218	0.057	0.202	173	685
GCTGGTGCAGAGG	0.275	0.03	0.234	114	373

ACCATTACTGGTA	0.022	0.002	0.02	64	3154
GCCGGTGCTAGTA	0.06	0.013	0.041	38	884
CGTAGCGTTGGCA	0.026	0.007	0.028	22	756
CGTGTTGCTAGGG	0.025	0.008	0.028	11	388
AGCGTCGCTAGGA	0.019	0.002	0.022	23	1036
CGCGGCACATGTG	0.024	0.007	0.014	14	971
GGTAGTGTATGCG	0.066	0.015	0.059	32	515
CGTAGCGCTGGGG	0.016	0.006	0.024	8	328
AGTAGCATTTAGA	0.023	0.005	0.017	24	1402
AGCGGCATAGGTG	0.02	0.002	0.018	9	485
ACCGTTACATGCA	0.019	0.002	0.02	71	3522
GGTAGCGCATGTA	0.078	0.02	0.079	52	605
AGCGGCGCTTGTA	0.021	0.003	0.022	21	948
ACTATTGTTAATA	0.024	0.001	0.024	88	3639
AGCATCATTGAGG	0.023	0.003	0.026	43	1643
CGTAGTACAAAGG	0.028	0.003	0.032	34	1033
AGTATCATATGTG	0.022	0.003	0.024	31	1236
CGCGGTGCATGGA	0.022	0.001	0.021	17	794
GCCATTGCTGAGA	0.045	0.004	0.038	48	1199
CCTGTTGTAGATG	0.019	0.004	0.025	35	1354
ACTGGCATTTAGG	0.024	0.004	0.022	16	723
GCCATCACTGATG	0.056	0.002	0.054	99	1720
GCCGGCATATGTG	0.72	0.037	0.715	1098	437
ACTAGTATTGGTG	0.027	0.003	0.023	30	1258
CGCGTCGCTGGCA	0.025	0.004	0.019	22	1107
GCCAGTGTAGGTG	0.053	0.005	0.047	41	833
ACTGGCATAGATA	0.022	0.003	0.026	54	1997
CCTGTTGTAAGTG	0.026	0.006	0.019	24	1265
GGTGTTATATATG	0.092	0.014	0.088	85	876
CGTATCACAGGGA	0.021	0.002	0.018	17	918
CGTGTTATTTAGG	0.029	0.008	0.018	13	703
AGTAGTACATAGA	0.02	0.004	0.014	22	1584
GCCATCGCAGGGA	0.046	0.008	0.038	34	856
AGTGTTATTAGGA	0.02	0.005	0.014	15	1079
GCCGTCACATACG	0.056	0.004	0.054	89	1559
CCTATCGTTAGTA	0.034	0.002	0.032	58	1728
CGTGGCGTAAGTA	0.03	0.004	0.027	22	791
CGTGTTGTAGGCA	0.021	0.003	0.017	14	791
ACTGGCACTTAGG	0.026	0.004	0.024	19	787
CCTGGCGCTGACG	0.028	0.005	0.024	26	1044

CGTAGCATAGGGA	0.032	0.011	0.017	13	737
GCTAGCACTTAGA	0.056	0.01	0.068	75	1030
AGTAGCGTTGGGA	0.018	0.004	0.019	11	582
GCTGGCATAGATA	0.642	0.042	0.646	1237	677
CCTGGTGCAGGTA	0.027	0.009	0.018	21	1120
CGCATTGCAAAGG	0.023	0.002	0.026	38	1431
ACTGGTGCTTGGG	0.024	0.009	0.013	7	531
GCTAGTGTTAAGA	0.057	0.002	0.058	79	1289
ACTGTCGTTTGTG	0.025	0.009	0.014	9	644
CCTGTTGCATACG	0.021	0.001	0.019	30	1567
ACTATCGTATATA	0.027	0.003	0.023	69	2923
GGCGGTATTGATA	0.067	0.018	0.058	80	1298
ACTGTCACTTAGA	0.025	0.008	0.018	33	1781
GGCGTTGCTTGTG	0.057	0.003	0.055	29	502
GGTGGCATAAGTA	0.474	0.061	0.453	567	686
ACTGGTGCAAATA	0.025	0.003	0.021	56	2643
CGTAGTGTAGACA	0.032	0.005	0.037	44	1151
GGCAGTATATGGA	0.053	0.012	0.06	84	1305
ACCATCACATGGA	0.024	0.005	0.021	58	2741
GCTATCGTATACG	0.068	0.012	0.085	114	1234
CCTGTTGTTAGTG	0.023	0.004	0.022	22	978
GGCAGCGCAGGGA	0.057	0.011	0.046	32	670
GGCGGCGCTGATA	0.101	0.017	0.089	80	816
ACTATCACAAGCG	0.024	0.001	0.023	61	2632
AGCGTCATTTGCG	0.02	0.005	0.017	20	1158
CCTATCATTAAGA	0.028	0.002	0.026	82	3061
CGTGGTGTATAGA	0.022	0.012	0.014	10	727
CCTAGCGTAGGCG	0.021	0.005	0.019	20	1043
CGTATCATTTACG	0.028	0.005	0.024	37	1507
AGTAGTACAAAGA	0.023	0.002	0.025	51	1999
CCTGGTGCTGGGA	0.025	0.003	0.021	16	756
GCCGGTGCTTGCG	0.1	0.019	0.092	56	550
CGTATCGCTGACA	0.027	0.003	0.023	34	1425
CCCGTTGCAAGTA	0.027	0.005	0.02	43	2070
ACCGGCGTAAGTG	0.023	0.001	0.022	16	713
CGTATCGTAAGCA	0.023	0.003	0.019	34	1750
CCTGGTGTAGGTG	0.022	0.007	0.013	11	808
GGTATCGTATGTG	0.147	0.025	0.114	75	583
AGCGGTACATACG	0.024	0.003	0.027	44	1609
CGCGTTACAGAGA	0.021	0.006	0.016	24	1439

ACCGGCACTGGCG	0.022	0.007	0.012	16	1290
GCTATTGTTAACA	0.059	0.002	0.057	137	2260
GCCATCATTGATG	0.057	0.006	0.059	115	1849
CGCGGTGCAAATG	0.027	0.004	0.027	35	1264
GCCATTACAAGTA	0.048	0.006	0.055	153	2632
GGCAGTACTTGGA	0.056	0.017	0.056	52	874
CGTAGCGTAGATA	0.027	0.003	0.023	29	1249
CCTGGCGTATATG	0.022	0.001	0.022	22	964
AGTAGCGTAAGGA	0.021	0.007	0.014	14	968
CGCGGTGTTGAGA	0.023	0.003	0.025	22	870
GCCGTCGTAGAGG	0.057	0.013	0.05	34	644
GGCAGCGCAGGTG	0.057	0.01	0.042	29	655
GCCGTTGTTTATA	0.049	0.003	0.044	67	1439
AGTGGCACTTGGA	0.022	0.001	0.021	12	559
GCTAGCGCAAACG	0.06	0.004	0.065	69	991
GGCATCGTTTAGG	0.104	0.025	0.079	62	722
CGTAGTATAGGCA	0.029	0.008	0.02	24	1151
ACTGGTATTTAGG	0.022	0.004	0.016	15	903
CCTAGTGTAGGCG	0.022	0.005	0.015	20	1293
GGTAGTATATGGA	0.067	0.008	0.072	57	730
CCCGGCGCAGGCG	0.022	0.007	0.032	37	1126
CCCAGTGCTAACG	0.023	0.002	0.024	54	2224
CCCATTGTTTGCA	0.026	0.006	0.018	39	2092
AGTGTCGCAGGCG	0.017	0.007	0.009	7	734
AGCGGCATAAAGA	0.023	0.004	0.018	34	1807
GCTAGCGTTAGCA	0.059	0.002	0.058	61	982
CCTGGCGCAAGGA	0.021	0.005	0.018	20	1095
GCTAGCATAAGCG	0.064	0.004	0.063	76	1127
AGTATTATTGGCG	0.024	0.004	0.029	40	1327
CGTGGCACTAACG	0.021	0.002	0.019	17	870
ACTGGCGCTTATG	0.03	0.007	0.024	18	730
ACTGTTATAAAGA	0.025	0.001	0.025	77	3056
CCCGGCATAGAGA	0.026	0.004	0.025	51	2029
AGCAGTGTTAATA	0.026	0.003	0.022	60	2702
CCTAGCATATGGG	0.033	0.006	0.036	37	986
AGCATTGCATAGG	0.021	0.005	0.016	24	1464
AGTATCGTATGGG	0.024	0.002	0.024	18	745
CCTGTTATTAGCG	0.026	0.01	0.012	17	1348
AGCGGCACTTGGG	0.025	0.005	0.029	15	494
CGTGGTATAAGCA	0.021	0.006	0.015	18	1202

GGTAGCATTGGGA	0.064	0.007	0.063	35	524
CCTAGTGTAGAGG	0.026	0.006	0.023	28	1176
GCTATCACATGTA	0.06	0.005	0.054	84	1475
AGCGGTATTAACG	0.015	0.002	0.012	21	1666
GCCGTCGCTAAGG	0.039	0.011	0.026	17	631
AGCGGCACTAGCG	0.016	0.003	0.017	16	903
CGTGTCGTAGAGG	0.034	0.007	0.024	15	602
ACTGGCGCTAGGG	0.031	0.004	0.029	14	477
AGCAGCGTTAGTG	0.019	0.005	0.026	29	1076
GGCAGTGCATATA	0.043	0.004	0.045	63	1349
ACCGGTGTTGACA	0.025	0.005	0.028	57	1969
CCTATCACTGAGA	0.023	0.006	0.016	38	2282
CGCAGTATTAATG	0.028	0.003	0.025	52	1989
CCTGTCATATGTG	0.026	0.007	0.021	27	1283
GGCGGCGCTAGCG	0.175	0.018	0.157	108	578
AGTATCGCTAGTG	0.026	0.005	0.019	16	813
AGCATTACAGGGA	0.02	0.003	0.017	31	1789
GCCGGCGTAAACA	0.858	0.03	0.853	2768	476
CCTGGTGTATACA	0.022	0.004	0.024	47	1881
ACTATTATTGAGG	0.029	0.001	0.029	55	1814
GGTATCATTTAGA	0.127	0.038	0.078	85	1005
GGTGTTGCTGGGA	0.072	0.009	0.065	22	319
GGTGTTACAAGTG	0.079	0.012	0.068	48	663
CCCAGCATAAGTA	0.025	0.002	0.024	84	3389
GGCAGCACTGACG	0.053	0.011	0.038	46	1157
AGTATTGTTTAGA	0.023	0.003	0.025	36	1386
GCTGGCATATGTG	0.869	0.029	0.886	1138	146
GGTGGTATAAGGA	0.117	0.03	0.089	62	638
ACTGGTGCTAGGG	0.028	0.008	0.019	11	574
CGTAGCGTAAACA	0.026	0.007	0.02	36	1784
GCCGGTATTGGCG	0.149	0.018	0.135	122	779
GCCAGTACTTGTA	0.05	0.007	0.041	68	1588
CCCATCATATATG	0.026	0.003	0.029	91	3049
CCTATCATTAGGG	0.039	0.01	0.034	45	1274
GCCAGTATTAATA	0.054	0.006	0.05	193	3630
GCCATCACTGAGA	0.048	0.009	0.041	71	1677
CCCGGCGTTGGTG	0.023	0.009	0.025	19	729
CGCGGCGCAGGCG	0.025	0.01	0.01	8	758
ACCGGTGTTAGGG	0.023	0.006	0.032	28	846
GGCGTCGCAGGTG	0.078	0.025	0.047	29	585

GCCATTGCAGACG	0.051	0.006	0.046	69	1439
CGCATTGTTAGGG	0.028	0.001	0.026	23	848
CGTAGCATATACG	0.02	0.002	0.019	25	1301
GCTAGTACTGACA	0.056	0.003	0.053	81	1461
CGTAGCACATGTG	0.021	0.001	0.023	18	778
GCTGTTACAGGCA	0.067	0.017	0.044	49	1061
AGCGTTGTAGATA	0.029	0.005	0.023	47	1980
ACCGGTACTTAGG	0.021	0.001	0.022	36	1587
CCTAGTGCTTGTA	0.023	0.001	0.022	32	1412
CGTGTCGTATGGG	0.037	0.011	0.033	16	471
GGCATCATTTGGA	0.083	0.01	0.072	84	1079
GCCGGCGCAAATA	0.504	0.038	0.506	1062	1035
AGCGGTACTAAGG	0.02	0.002	0.02	24	1172
CCTAGTGCTAGCG	0.022	0.004	0.023	26	1086
CGTAGCGCAAATG	0.028	0.004	0.023	24	1036
ACTAGTGCATGTG	0.028	0.002	0.029	44	1451
AGCAGTATTAGGG	0.022	0.001	0.02	28	1347
GCTATTGCTTACG	0.056	0.006	0.047	48	974
GCTAGCGTATAGA	0.061	0.009	0.049	49	942
GCTGTTGTAAATA	0.053	0.008	0.043	76	1699
GGTGGTGCTTATA	0.078	0.015	0.074	43	539
CCTAGTGTTAGCA	0.023	0.003	0.024	45	1815
CGTGTCGTTAGTA	0.016	0.005	0.01	8	827
CGTATTGTATATA	0.026	0.002	0.025	47	1823
GGCGTTGTAGATA	0.063	0.002	0.061	77	1193
AGTGTTACATGTA	0.025	0.003	0.022	31	1393
GGCATTGTAGGGG	0.066	0.017	0.044	37	802
CGCAGTGCAGAGG	0.022	0.008	0.031	25	778
CGCGGCGCTAGTG	0.027	0.009	0.026	18	676
GGTAGCACTTAGG	0.058	0.013	0.05	24	455
AGCGGCGTTAGTA	0.022	0.003	0.021	22	1034
GCTGTCATTTAGG	0.062	0.003	0.059	43	685
AGTGTCGCAGGCA	0.021	0.005	0.018	16	880
CGTAGCATTGGGG	0.036	0.01	0.023	10	434
GGCGTCATTGAGA	0.061	0.01	0.048	50	992
AGTGGTACTAGGG	0.026	0.005	0.032	16	483
ACTGTCGCTGATG	0.023	0.004	0.028	31	1077
GGCAGTGTAAATA	0.055	0.006	0.05	111	2118
CGCAGTGTATGTG	0.027	0.005	0.032	30	896
CGTGTTATTGATG	0.025	0.002	0.023	22	916

GGCGTTGCAGGCA	0.053	0.004	0.052	47	849
GCCGGCACTTAGA	0.106	0.009	0.097	110	1027
CGTGTCGCAAACG	0.025	0.005	0.017	20	1129
CGCATCACATAGG	0.029	0.002	0.028	42	1443
AGCATTACAGATG	0.023	0.004	0.018	47	2594
CGTGTCACTTAGG	0.024	0.004	0.029	17	570
CGCATTGCAGGGG	0.031	0.007	0.035	25	685
ACTAGTGCAAGCG	0.028	0.004	0.029	52	1760
GGCAGCACAGGCA	0.053	0.007	0.044	63	1372
CCTGTCGTATGCA	0.02	0.003	0.017	27	1538
CCCAGTATTTGCG	0.029	0.004	0.026	51	1877
ACTGTCGTTAAGG	0.022	0.006	0.019	17	881
CCTATTGTTAGTG	0.019	0.004	0.025	35	1376
GGCAGTGTTAGTA	0.061	0.014	0.054	62	1081
CGCGTTACATGCA	0.022	0.002	0.024	42	1736
ACTAGCGTAGATG	0.028	0.002	0.03	36	1184
ACTAGTACAAACG	0.024	0.004	0.02	73	3493
AGTAGCATAGAGA	0.028	0.007	0.025	39	1509
GCTATCGCTTGCG	0.062	0.018	0.044	32	693
AGTGGTGTAAGTG	0.028	0.011	0.037	23	601
AGCGTCATAAGCG	0.024	0.001	0.023	42	1793
CGCGGCGTATGGA	0.026	0.01	0.04	31	744
GGTGTCATAGATA	0.076	0.009	0.068	75	1029
GCCGGCATAAACG	0.743	0.03	0.75	2003	668
GCCAGCATTGGTG	0.052	0.007	0.046	54	1112
GGCATTATTTATG	0.058	0.007	0.053	87	1565
CCCGTTGCAGGTG	0.024	0.003	0.024	26	1073
GCCATTGTATATG	0.051	0.006	0.047	71	1455
CCTAGCGCAAAGA	0.017	0.005	0.011	25	2251
GCCGTTACTGAGG	0.058	0.008	0.047	41	840
CGTGGCATTTATG	0.024	0.01	0.011	6	533
CGTGTTGTAGGGG	0.028	0.005	0.036	13	353
ACTGTTGCTGGTG	0.02	0.005	0.016	14	853
GGCAGCGTTTGCA	0.062	0.006	0.053	51	910
AGTGTCGCTTGCA	0.025	0.006	0.032	25	763
AGTGTTGCTTGTA	0.02	0.006	0.029	23	784
GGTAGTGCTAGGG	0.044	0.01	0.042	13	300
CCTGGTGCTTGTG	0.02	0.002	0.019	11	579
GCTGTCATTTGTA	0.063	0.005	0.061	59	910
GGTATTGTAAGTA	0.088	0.007	0.078	93	1094

AGCGGCGCATATA	0.024	0.004	0.02	31	1533
CCTGGTGCTTAGG	0.025	0.008	0.029	18	595
GGTGGCGTTTGGG	0.655	0.062	0.615	252	158
GCCGGCACATATG	0.217	0.023	0.211	298	1117
CCTGGCACAGAGG	0.03	0.003	0.027	25	902
AGCGGCGCTAAGG	0.021	0.002	0.024	19	768
ACTATTGTTGGCA	0.025	0.005	0.017	33	1863
GCCGTCGCAAGGA	0.045	0.009	0.052	48	869
GCCATTACAGAGG	0.051	0.005	0.058	86	1406
AGCGTCACAGACA	0.019	0.003	0.022	57	2593
AGCGTCGCTTGTG	0.018	0.008	0.008	7	916
CGTAGTGCTGAGA	0.033	0.005	0.03	20	647
CCTGTCACATAGA	0.027	0.003	0.03	50	1601
ACTGGCATAGGGG	0.025	0.008	0.025	14	549
GGCATTGCTTGGA	0.057	0.005	0.053	39	698
AGTGGCATTGATG	0.034	0.01	0.02	6	298
AGCAGCGTTGGTG	0.024	0.006	0.015	14	900
GCTATCGCATACG	0.059	0.002	0.059	65	1035
CCCGGCGCAGGGA	0.026	0.005	0.022	25	1088
CGCAGTACATACA	0.026	0.002	0.026	73	2723
CCTGTCACAAGGA	0.026	0.001	0.028	45	1576
ACCGTTGTAAGGG	0.022	0.003	0.019	24	1255
GCTATCACTGGCA	0.055	0.005	0.055	71	1218
ACTATTGTAGGTA	0.028	0.002	0.03	60	1963
CGCGTTGTAAAGA	0.028	0.006	0.02	35	1716
GCTATTACTAAGG	0.069	0.002	0.072	95	1229
GCCATCATATGTG	0.049	0.007	0.057	97	1598
ACCAGCATAGGGG	0.028	0.004	0.024	26	1064
GGTGGCGTTGGTG	0.632	0.06	0.65	297	160
GGTAGTGCATGCG	0.052	0.009	0.04	19	459
GGCGTCACATGCA	0.068	0.007	0.058	76	1231
AGTATCACTTAGG	0.025	0.005	0.018	17	914
GGTGGCGTAAGCG	0.665	0.05	0.621	545	333
CGTGGTATAAACG	0.021	0.006	0.013	16	1203
GGCATCGCAAGCG	0.1	0.02	0.074	91	1131
GCTGGCGCAAACA	0.622	0.043	0.633	1236	717
ACTGGTACTGATG	0.025	0.003	0.021	27	1282
ACTATCGTTGAGG	0.027	0.005	0.034	39	1099
GCTGTTACATGTA	0.065	0.007	0.06	68	1057
GGTAGCGCTAATG	0.076	0.015	0.076	51	620

CCTGGTGCATGGA	0.023	0.001	0.021	21	957
GCTGGTACAGGGG	0.11	0.007	0.11	48	387
GCTGTTGTAAAGG	0.061	0.006	0.069	66	884
CGCGTCGCTTGTA	0.025	0.001	0.026	29	1068
GCCGGTACAGGCA	0.062	0.003	0.065	84	1203
CGTAGCATTTAGG	0.027	0.005	0.031	23	724
CCTAGCGCAAGGA	0.018	0.001	0.019	30	1557
ACCGGCGTATGGG	0.026	0.003	0.027	13	473
GGTATCGTAGGGG	0.094	0.007	0.092	45	446
ACCAGTGTAGGCG	0.024	0.008	0.018	37	2025
CGCGGTGTATATA	0.024	0.001	0.024	34	1406
GCCATTGTTTACG	0.055	0.002	0.057	81	1338
GCCAGTACTGGTA	0.048	0.011	0.033	46	1342
CGTATCATTGAGA	0.026	0.002	0.023	35	1496
AGTGTTGTAAACA	0.023	0.003	0.02	41	1993
ACCGTCGTATATG	0.029	0.003	0.032	47	1423
AGCATTGCAAGTA	0.025	0.004	0.021	53	2430
GGTATTATTAATG	0.11	0.018	0.087	132	1381
CGTATTGCATGGA	0.024	0.002	0.026	23	848
GCTGTTATTAGCA	0.067	0.006	0.063	81	1204
ACTGTTATATAGG	0.027	0.006	0.019	26	1351
AGCGGTGTATAGG	0.02	0.002	0.019	16	826
AGCGTCGTTGAGG	0.018	0.004	0.017	15	846
CGTGTTGCTGATA	0.026	0	0.026	27	994
CCCAGCGCAGAGA	0.024	0.003	0.025	60	2300
AGTGGTGCTTGTG	0.022	0.008	0.023	10	418
AGTATCATTTAGG	0.023	0.004	0.023	26	1100
GGCGTCGCTTACG	0.072	0.002	0.07	58	776
CGTGGCGTTTGCG	0.028	0.011	0.013	5	367
GGCGTTGTAGAGG	0.067	0.008	0.056	38	643
GCCGTTGCTGGCA	0.04	0.002	0.037	34	875
GCCGGCATATGGG	0.593	0.039	0.585	543	385
GGCAGCGTATGTG	0.064	0.005	0.065	55	790
GCCGTCGTAAACA	0.057	0.006	0.055	114	1977
CGTGTCGTTAAGG	0.021	0.006	0.017	11	655
AGTGGTATTTGGG	0.022	0.005	0.022	8	364
GGCGTCGTTTGCG	0.08	0.012	0.065	42	601
AGTATTATAGGCA	0.023	0.001	0.023	47	2021
AGCGTTGTATACG	0.022	0.004	0.02	31	1556
GCCGGCATATGGA	0.379	0.027	0.37	465	791

GCTATCATATGCG	0.065	0.008	0.058	77	1261
ACTGGCATTAATG	0.026	0.002	0.029	41	1382
GCTAGCGCTTGTA	0.056	0.009	0.045	31	656
GCTGTCACTGAGA	0.056	0.004	0.055	51	882
ACTAGTGCAGAGA	0.02	0.004	0.018	38	2092
GCCAGCATAGAGG	0.07	0.008	0.066	71	1000
GCCGGTGTTGAGA	0.171	0.021	0.166	151	759
GGCATTGCTGAGA	0.049	0.005	0.046	50	1046
CCCGGTATTAAGG	0.024	0.007	0.018	30	1608
CGTGGCACTTAGA	0.022	0.01	0.013	10	741
CGCGTCATAGACG	0.022	0.006	0.015	24	1571
GCTGTTGTTGGCG	0.058	0.016	0.061	47	719
GGTAGCACTGGCG	0.048	0.006	0.053	28	497
CCTGTTGCATGCG	0.028	0.01	0.016	17	1038
ACCGGCGCTTGTA	0.028	0.002	0.026	26	965
GGCGGCGCTGGGA	0.11	0.045	0.062	28	427
ACCAGTGCATAGG	0.023	0.001	0.025	44	1717
CCTAGCGCATGCG	0.023	0.001	0.024	27	1107
GCTATTGCATGGA	0.045	0.006	0.041	42	983
GGCGTCGTAGAGG	0.06	0.02	0.038	27	677
ACCGGTGTTTACG	0.025	0.005	0.019	25	1325
GCCATCGTTTATA	0.052	0.003	0.055	96	1662
ACCATTATATGTA	0.025	0.005	0.029	121	4123
CCTATCGCTGGCG	0.027	0.005	0.02	24	1151
AGTGGTACTAAGA	0.032	0.007	0.025	30	1191
AGTGTTGTATGGG	0.024	0.009	0.023	12	516
CGCGGTACTGACG	0.025	0.006	0.019	23	1215
ACCAGTGTTGGCA	0.024	0.002	0.022	40	1803
GGCGGCGCTAAGA	0.112	0.023	0.092	78	773
GGTGGTGTAAACG	0.227	0.031	0.22	162	576
GGTGGCGCAAATA	0.154	0.027	0.13	129	863
GGCATTGTAAGCG	0.081	0.006	0.077	100	1202
CGTAGTATTAATG	0.024	0.004	0.027	33	1195
AGCGGTGTAGACG	0.016	0.003	0.016	20	1198
GGCGTCATTTACA	0.062	0.002	0.06	99	1551
ACTGTCATAAGTA	0.024	0.001	0.025	60	2329
CGTGTCACTGGCA	0.027	0.005	0.032	27	825
GGCATTACTTGCG	0.061	0.015	0.041	42	993
ACTGTTGTAGAGA	0.031	0.009	0.019	25	1319
CCTAGCACTAAGG	0.025	0.004	0.022	30	1363

CGCAGCACTGGTA	0.029	0.006	0.025	31	1220
CGTGTCATAAGCG	0.023	0.003	0.023	24	1028
GCTGTCGTAGGTA	0.061	0.007	0.052	41	752
GGCGTCGTTAGTG	0.072	0.016	0.065	41	593
GCTAGCGTTAGTG	0.071	0.008	0.061	39	601
GCCGTTGCAAGCA	0.047	0.006	0.039	53	1309
GGCAGCGTTAATA	0.061	0.011	0.055	92	1575
GGCGGCGTTTGCA	0.223	0.033	0.178	126	580
AGTATCGCTTGCA	0.021	0.001	0.021	22	1041
CGTGGTGTTTATG	0.023	0.003	0.021	12	566
GGTGTTGTTAGGA	0.077	0.025	0.064	30	438
CCCATCGTTTGGA	0.02	0.004	0.015	21	1401
AGCATCGCTAGCA	0.022	0.003	0.026	55	2078
CGTATTGCATAGA	0.023	0.007	0.02	24	1184
CGTAGCACAAACG	0.021	0	0.02	32	1547
CGTAGCATTGGGA	0.026	0.006	0.035	25	695
AGTGTCACTTAGA	0.026	0.006	0.024	25	1020
CGCGTCATAGGGA	0.034	0.004	0.037	38	976
GCTATTGTAAGGA	0.051	0.005	0.044	58	1275
AGCGTTACTGGCG	0.021	0.001	0.022	24	1061
CCCATCACTGATG	0.034	0.007	0.028	70	2450
ACTGTTATATGTA	0.026	0.002	0.027	62	2207
CGCGGCATAAGGG	0.03	0.002	0.032	23	699
GGTGTCACTGGCA	0.087	0.007	0.084	53	576
AGTGTTACAAGTA	0.025	0.005	0.032	56	1702
GGCGGTATATAGG	0.118	0.017	0.103	96	838
ACCATCATTTGGG	0.025	0.002	0.027	37	1354
CCTGGTGCAGATA	0.023	0.003	0.025	38	1509
AGCGGCATAAACG	0.024	0	0.023	23	968
GGCGTTGTTTAGG	0.068	0.009	0.079	48	563
GGTAGCACAAGTA	0.074	0.005	0.078	87	1034
GCTATCATTTGTA	0.054	0.004	0.05	78	1470
CCCGTCGCAGGGA	0.025	0.006	0.028	38	1302
CGCATCGTATGGA	0.024	0.001	0.026	37	1393
GGCGGCATATGGG	0.461	0.056	0.413	330	469
GGCGGCACTGAGG	0.27	0.057	0.22	160	567
CGTGTTGCTGAGA	0.018	0.006	0.01	7	691
AGCGTCATAGATA	0.021	0.002	0.023	61	2583
GCCAGTACTGGGA	0.046	0.001	0.045	48	1009
AGCGTTGCTGGGG	0.02	0.005	0.025	13	515

GGTATTATTAAGG	0.112	0.002	0.114	113	878
AGTGTCGCTTAGG	0.02	0.003	0.024	13	534
GGTGGTATTAATG	0.265	0.028	0.248	224	678
GCTAGTACATGTG	0.063	0.015	0.078	73	865
ACCGTCGCTAGTA	0.019	0.003	0.022	42	1865
GGCATCACTGACA	0.059	0.004	0.055	115	1967
CCCGGCGCTTAGA	0.024	0.007	0.018	23	1238
GCTGGCACAAGCG	0.45	0.028	0.49	485	504
CCTAGTGCAAGGG	0.031	0.002	0.029	33	1109
CCCGGTGCAGACA	0.022	0	0.023	56	2402
GCCGGCGTTGACA	0.851	0.027	0.844	1806	335
CGTATTGCAGGCA	0.025	0.007	0.016	19	1158
GCCAGCACTGGGA	0.053	0.007	0.043	41	914
ACCATCGCATACG	0.025	0.004	0.031	80	2481
GCTGGTATAGGTG	0.418	0.022	0.393	304	469
CCTGGTGCTGGTG	0.024	0.006	0.032	18	537
GGTGTTGCAGACA	0.072	0.014	0.054	50	881
CGTGTCGCATATA	0.025	0.011	0.019	21	1104
GCTAGTGTTGAGA	0.048	0.004	0.05	52	992
CCTGGTACTTGCA	0.026	0.001	0.025	36	1387
CGCAGCGCTTGGG	0.027	0.002	0.024	15	612
ACCGTTGCTGGTA	0.02	0.002	0.02	33	1600
CCTAGTACTAGGG	0.023	0.002	0.024	22	898
GCTGTTGTATGCG	0.073	0.018	0.051	37	691
GGCATCACTAGGA	0.077	0.007	0.08	98	1120
CCTGGCACTTAGA	0.027	0.003	0.023	27	1137
GGTGTCATTGGGG	0.076	0.023	0.108	35	290
CGTGTCATAGGGA	0.016	0.003	0.017	12	696
CGTGTTATATATA	0.025	0.004	0.019	36	1824
AGTAGTGCTGACG	0.022	0.002	0.02	17	825
CGTATTGCAGGGG	0.031	0.007	0.029	15	500
GCCGGCACAAGGA	0.103	0.009	0.116	135	1032
AGCGGTACAGGCG	0.026	0.007	0.022	23	1006
GCTATTGTATATA	0.052	0.001	0.053	116	2075
CGTGGCGTTGACA	0.024	0.001	0.025	24	923
CGCGTTACAGGTA	0.021	0.004	0.022	31	1370
GGCGTTATTTGTA	0.056	0.003	0.06	68	1064
AGCAGCGTAAAGG	0.02	0.002	0.019	33	1670
GGCAGTATAGATG	0.055	0.005	0.057	87	1438
CCCGTTGCATGTG	0.027	0.007	0.028	32	1123

AGTGTCACAAGTA	0.023	0.005	0.023	32	1355
AGTATTACATATA	0.021	0.002	0.018	53	2841
GCCGTCGTATGCG	0.048	0.004	0.051	40	746
CCCAGCATTTAGG	0.021	0.001	0.023	38	1605
ACCGTTGCTGAGG	0.028	0.005	0.031	38	1177
ACCAGCGCTAATG	0.023	0.001	0.022	49	2178
CGTGGCGCATAGG	0.019	0.006	0.01	5	480
GGCAGTGCTGATG	0.052	0.012	0.036	32	859
AGCAGTACTGAGA	0.023	0.002	0.025	47	1857
CGTGTTGCAAAGG	0.032	0.007	0.034	25	721
GCCATTATTTGTG	0.049	0.003	0.045	71	1516
CGTATTATTAACA	0.021	0.001	0.02	62	3000
GGCATTGCTAGGA	0.063	0.002	0.061	52	804
GGCGGCACATGGG	0.266	0.054	0.213	134	496
CGTGGTGCAGAGA	0.024	0.001	0.023	17	724
CGTGTTGCTAACG	0.022	0.002	0.022	21	937
CGTGGCGCATGGA	0.032	0.015	0.021	10	476
CGCGGTGCAGATG	0.023	0.004	0.027	24	876
GGCATCACTTGGA	0.06	0.011	0.066	73	1034
GGTGGCATTAGTA	0.492	0.059	0.502	509	504
GCCGTCGCTGACA	0.048	0.008	0.04	50	1200
GGTAGTGCATGGA	0.044	0.008	0.036	18	484
GCTGTCATAAGGA	0.057	0.004	0.061	70	1070
GGCAGCGCTAGGA	0.065	0.008	0.068	53	728
CGTGTTGTAGACG	0.029	0.004	0.026	23	853
GCCATTGCTAGCG	0.037	0.01	0.026	29	1067
CGTGGCGCAGACG	0.016	0.007	0.01	7	716
CGTAGCGTAGGTG	0.025	0.009	0.033	18	529
GCTGTTACTGGCA	0.059	0.004	0.065	54	779
CCCGGCGTATGTA	0.024	0.003	0.026	46	1716
CGCGTCACTGACG	0.024	0.001	0.025	35	1389
AGCATTGCATATA	0.023	0.003	0.026	74	2776
ACCGTCGTATGCG	0.027	0.002	0.027	32	1150
CCTGGCACAAACA	0.025	0.003	0.021	60	2745
GCTAGCATAAGGG	0.079	0.005	0.072	56	717
CCTGGCATTGGCG	0.034	0.003	0.03	26	855
CGTATCATTTGGG	0.025	0.007	0.015	11	707
CCCGGCACTGACG	0.031	0.002	0.032	63	1895
CCTGGTACATGGG	0.025	0.004	0.019	14	719
CGCATCGCTTATG	0.027	0.006	0.034	44	1259

AGTATTGCATGGG	0.02	0.005	0.013	10	738
CCTGTTGTATGTG	0.027	0.005	0.026	26	978
GGCGTCATTTACG	0.075	0.015	0.073	80	1009
CGTGTTGTTTACA	0.023	0.003	0.021	20	934
ACCGTCGTAAGGG	0.021	0.002	0.022	21	931
GCCGTCGTTAGCG	0.063	0.007	0.061	43	658
CCTGTTATTGATA	0.025	0.006	0.018	38	2034
CCTAGCGTTAATG	0.028	0.008	0.027	43	1572
GCCGTTACAGGTA	0.058	0.014	0.076	97	1182
GCTATCGTATGGA	0.06	0.007	0.05	45	858
GGTATCATAAAGA	0.132	0.015	0.115	195	1497
CCTATCGTTGATG	0.028	0.005	0.03	43	1396
CGCGGTGCTTATA	0.021	0.003	0.019	22	1149
CGTGGCGCTTGGG	0.015	0.002	0.018	5	276
GGTGTTATAGGTA	0.078	0.016	0.064	52	760
ACTGGCGTAGAGA	0.024	0.012	0.024	23	929
AGTGGTATTTAGA	0.027	0	0.027	28	1016
CGTGGTACTAGTG	0.023	0.001	0.024	14	567
AGCATCGTTAGTA	0.02	0.003	0.023	52	2186
GGCAGCATTTACG	0.056	0.003	0.052	72	1311
CGTGTTGCTGACG	0.029	0.003	0.033	25	743
ACCGTTGCTGGGG	0.024	0.004	0.03	25	801
GGCGTTATTGAGA	0.06	0.011	0.046	51	1068
CCTGTCATTGGGG	0.048	0.017	0.047	31	634
AGTATTATTAGGA	0.022	0.005	0.017	29	1697
GCCATCGTTGGCA	0.05	0.006	0.044	60	1316
CGCGGCATTGATA	0.026	0.002	0.024	36	1454
CGCGGTGTTGATA	0.026	0.001	0.025	30	1171
GCTGTCGCTTATA	0.053	0.012	0.037	30	773
GCTATCGTATGCG	0.057	0.003	0.056	53	895
CCTGGCATTTGTG	0.025	0.004	0.025	20	777
GCTGTCATATGCG	0.069	0.01	0.066	62	872
CGTATCGCTTGTG	0.027	0.004	0.027	17	617
GCTGGTGTTAAGA	0.412	0.051	0.403	438	649
ACTGGCACAGAGA	0.022	0.006	0.017	26	1473
GGCAGTGCTGAGA	0.053	0.009	0.06	52	819
GCTGTCGCTAAGG	0.062	0.003	0.062	27	409
GGCAGCGTTAACG	0.062	0.009	0.05	58	1110
AGTAGTACTTGTG	0.027	0.003	0.024	21	837
ACTATTGCTAGTG	0.028	0.001	0.028	48	1689

CGCATCGTAGGTG	0.026	0.004	0.03	33	1068
CCCATTGTTTAGG	0.022	0.003	0.026	36	1334
GGCGGTGTTAACG	0.091	0.016	0.071	67	871
GGTATCGTTGACG	0.151	0.011	0.143	109	653
ACCGGTGTAGACG	0.026	0.007	0.015	23	1465
CCTATCGCTGGTG	0.026	0.001	0.027	26	936
AGTGGTACAAGCG	0.019	0.004	0.015	13	850
AGCGGCGCAGGCA	0.022	0.007	0.017	21	1249
AGTGTCATTGAGA	0.022	0.002	0.023	28	1165
ACCGGCGTAAGTA	0.024	0.008	0.014	21	1510
ACCGGTATTGATG	0.022	0.002	0.02	32	1578
ACCAGCGTAGAGG	0.025	0.003	0.028	34	1200
ACTGGTGTTGGTA	0.031	0.002	0.029	26	875
AGTGTTGCTTGTG	0.023	0.005	0.029	18	598
CGTATCATAAGGG	0.022	0.003	0.025	29	1123
GCTATCGCAAGCG	0.059	0.008	0.048	50	987
AGTGTTGCAGGTA	0.019	0.004	0.025	21	816
GGCGGCATAGGGA	0.167	0.032	0.144	148	880
GCCGGTGTTAGTG	0.403	0.062	0.418	340	474
CCTGGCATAAGGG	0.037	0.006	0.032	32	958
CCCGGCGTAGGCG	0.022	0.006	0.022	25	1132
GCTAGTGCAGAGG	0.061	0.009	0.049	32	626
AGCAGTGCTGGTG	0.023	0.001	0.021	20	927
AGTATCGTTGATA	0.026	0.007	0.016	27	1661
GCTATCATTAGTG	0.076	0.014	0.068	88	1198
AGTGTTATAAATG	0.025	0.003	0.021	42	1977
CGCAGCATAAGGA	0.026	0.002	0.023	35	1468
AGTAGTACAGAGG	0.028	0.004	0.033	32	941
GCTATTGCAAGGG	0.053	0.003	0.051	41	760
CGTATTACTTGGA	0.028	0.004	0.026	26	967
CCCGTCGTTGACA	0.021	0.003	0.019	48	2417
GCTAGCGCAGATG	0.062	0.004	0.061	40	616
ACTAGCGCTTACG	0.023	0.005	0.022	32	1398
GGCGGTACTTATG	0.071	0.019	0.064	54	790
CGCATCATTGGTG	0.028	0.013	0.046	54	1132
CGCGTCGTAGGTA	0.022	0.001	0.021	23	1062
AGTGTTGTTTGTA	0.018	0.003	0.014	11	777
AGTGTTGTTTGTG	0.025	0.01	0.022	13	569
CGCGTCACTAGGG	0.031	0.004	0.033	27	783
GCTATCGTAAATG	0.055	0.007	0.053	87	1547

CCCGGTACTTAGA	0.031	0.005	0.038	65	1659
GCCAGCACATGGA	0.057	0.013	0.044	61	1336
GCTGTCGTATAGG	0.064	0.014	0.046	27	555
GGTGGTGCTGATG	0.095	0.029	0.063	25	374
ACCGGCGCAAGGG	0.029	0.006	0.03	25	802
GCTAGCACAAAGA	0.066	0.006	0.058	100	1627
CCTGTCGCTTGCA	0.024	0.002	0.025	33	1294
CCTAGTGCATGGG	0.022	0.004	0.017	16	925
CGTGGTGTATACA	0.027	0.005	0.022	24	1071
AGCGGCGCTAACG	0.024	0.004	0.028	34	1177
AGTATTGTTGATG	0.021	0.003	0.017	20	1144
CGCGTCACTAGTG	0.029	0.003	0.028	30	1041
CGTGGTGTTAATG	0.024	0.004	0.024	16	659
GCTATTGCAGGCA	0.052	0.004	0.058	74	1202
CGCAGTGTTTATG	0.026	0.002	0.023	27	1148
GGTATCATAGGGG	0.084	0.009	0.072	42	545
ACCGTCGCTTATG	0.025	0.003	0.03	43	1414
CCTGGCGCATATG	0.022	0.004	0.02	19	933
CCCGTCATAAAGA	0.025	0.003	0.022	71	3209
ACTAGCGTTGGGG	0.032	0.01	0.02	11	535
GCTAGCATATGCG	0.068	0.004	0.074	74	931
GCCGTCGTTAAGG	0.056	0.006	0.052	41	748
CCTGTTGCAGAGG	0.018	0.004	0.021	21	958
CGTGGTATTAGCA	0.022	0.001	0.02	19	909
AGCGTTATTGGTA	0.018	0.002	0.016	25	1561
CCCGTTATTTACA	0.028	0.005	0.023	68	2937
ACCGGTACTGGTA	0.021	0.003	0.021	47	2145
ACTAGCGTTAACG	0.023	0.005	0.021	34	1569
AGTAGTGTTAGCG	0.022	0.008	0.013	12	879
CGTAGCGCAGACG	0.028	0.011	0.015	13	863
CGTGGCATAAAGG	0.025	0.003	0.028	16	564
AGTATCATTAATG	0.02	0.004	0.015	28	1866
CCCAGCGCTGGTG	0.024	0.005	0.019	22	1160
GGCGGCGCAAATG	0.185	0.044	0.158	154	823
GCCATCATAGGGA	0.078	0.011	0.062	80	1203
GGTGTTACTTGCA	0.076	0.008	0.073	57	728
GGCAGTGCTAAGA	0.051	0.002	0.05	56	1061
AGCATTGCAGGTA	0.021	0.004	0.023	43	1800
GGCAGCACTTAGG	0.047	0.008	0.038	33	836
GCCGTTGCTGGTG	0.054	0.007	0.046	25	516

CGTATCACTTGGG	0.028	0.006	0.036	21	556
GGCGTTGTAGGCA	0.065	0.005	0.063	59	882
CCTGTCATTGGCG	0.024	0.002	0.027	29	1031
AGTAGTGTAAACA	0.025	0.004	0.023	57	2394
GCTGTTATATAGA	0.068	0.01	0.06	81	1268
CCCGGCATTTGTG	0.024	0.002	0.026	31	1139
ACTAGCGCTGGGG	0.036	0.014	0.022	13	591
GGCAGCACTAGGA	0.047	0.009	0.042	45	1016
AGCAGCGCTGATG	0.021	0.004	0.022	27	1214
CGCGGTGCAGGTG	0.023	0.004	0.017	11	636
CCTGTTACTGACG	0.026	0.005	0.023	32	1348
CGTAGCGCTAATA	0.021	0.004	0.027	36	1322
CGTGTCGTTAGGA	0.015	0.001	0.015	10	651
ACCGGTACATGGG	0.025	0.003	0.029	39	1292
GCTGTCGCAAGCG	0.054	0.012	0.037	24	617
GCTAGTACTAATA	0.066	0.01	0.056	120	2025
AGCAGTGCTGGGA	0.018	0.003	0.015	15	1019
GGCGTCGTTAGGA	0.087	0.01	0.094	64	617
GGTGTTATTTAGG	0.094	0.017	0.084	47	510
GGCGTTGCAAATA	0.059	0.008	0.05	76	1437
AGCAGTACTTGTG	0.018	0.001	0.019	27	1381
CCTGGCGCTAGTG	0.027	0.005	0.031	24	746
CCCAGCGTAGACG	0.02	0.004	0.021	47	2151
CGTGGCATTGACA	0.027	0.006	0.02	21	1055
GGTGGCACAAAGG	0.402	0.057	0.377	316	523
GGCGTTGCTAGCA	0.055	0.008	0.065	58	831
CGTAGTGTATGTA	0.026	0.004	0.024	22	894
GCTGTTGCAAGTA	0.06	0.001	0.062	58	885
ACTGTTACTTGGG	0.028	0.003	0.025	21	835
GGTGTCACAAATG	0.105	0.02	0.088	79	821
GGTGTTACTTACG	0.095	0.023	0.091	66	656
GCTGGCACATAGG	0.479	0.027	0.468	384	436
GGTATTGTATACG	0.109	0.024	0.088	84	867
CGCGTCACTGATG	0.024	0.001	0.024	30	1229
ACCGGTGTAGGCA	0.021	0.001	0.02	32	1572
CCCGGCGTAGATA	0.025	0.003	0.02	42	2013
GGTATCGTTAGGG	0.119	0.018	0.096	42	394
CGCGTCGCTTAGG	0.021	0.002	0.024	18	746
GCTGTTGCAGGTG	0.052	0.002	0.053	26	462
GGCGTCGTAAGCA	0.071	0.006	0.062	77	1157

GGCAGCATTAGCG	0.067	0.005	0.061	66	1022
GGCGTCGCATAGG	0.073	0.017	0.05	33	622
GGTGTCACTAGTG	0.096	0.014	0.09	47	478
GGCGGTATTTATA	0.081	0.018	0.059	83	1328
GGCAGCACTGGTA	0.054	0.01	0.052	58	1057
ACCAGCGTAGGTA	0.022	0.003	0.023	41	1724
GGCGGCATTTGTG	0.568	0.067	0.568	536	408
CGTAGCGTAGAGG	0.022	0.001	0.021	13	620
GCCAGTACTGATA	0.048	0.005	0.041	90	2085
AGTATTATAGATG	0.022	0.006	0.019	39	2052
CCTGGTACTGACG	0.029	0.005	0.025	30	1187
CGCGTTACATGTA	0.022	0.005	0.015	26	1658
CCCGTTGCTTGTG	0.028	0.004	0.028	30	1053
AGTGGCACTAGGG	0.021	0.007	0.015	6	383
AGTATCGTTGGCA	0.018	0.002	0.019	22	1137
AGTAGTGTTAGGG	0.022	0.008	0.028	17	599
GGTGGCGCTAATG	0.308	0.052	0.266	162	446
AGTAGCATATATG	0.028	0.009	0.028	42	1437
CGCATTACTAGCG	0.027	0.002	0.03	46	1483
GCCATTGCAAATG	0.051	0.011	0.036	66	1746
GGTATCACTGGTA	0.106	0.021	0.085	68	736
GGCGGTGTTAGCA	0.068	0.006	0.07	57	757
GCCGTTGTAGGCG	0.043	0.002	0.044	48	1031
CGTATCACTAGTG	0.022	0.006	0.027	26	937
GCCGGCGCTGACG	0.749	0.033	0.742	987	344
GGCAGCATAGGGA	0.057	0.008	0.05	67	1273
GCTGTTGTAGAGA	0.054	0.006	0.058	54	879
AGTGGCGCTAGCA	0.025	0.001	0.026	20	764
GCTATCACTGGTA	0.056	0.012	0.048	56	1111
CCTGGTGTTGACG	0.024	0.005	0.019	19	984
CGTGTTACAGAGG	0.024	0.004	0.02	15	722
CCCATTGTTAGCA	0.027	0.003	0.025	56	2195
GCTGGTGTAAGCA	0.401	0.066	0.36	441	785
GGTGTCATAAAGA	0.087	0.011	0.074	89	1117
ACCATCGTTAGGG	0.024	0.005	0.017	21	1217
GCTAGTGTTTACA	0.048	0.002	0.049	70	1368
GCCAGTGCAAACG	0.057	0.007	0.067	113	1578
CGTGTCGTTGGTA	0.03	0.006	0.022	15	669
ACTGTCGCTTACA	0.023	0.006	0.016	31	1880
CGCGGCGTTTATA	0.021	0.001	0.022	24	1062

GGTGTCGTAGGCA	0.099	0.014	0.084	48	521
GGTAGTGCTAAGA	0.048	0.013	0.029	24	801
AGTGTCATATGCG	0.029	0.006	0.022	20	902
ACCGTTATTTATG	0.024	0.006	0.016	33	2015
AGCATTGCTGGTA	0.022	0.002	0.02	32	1563
GGCATCGTTTGGA	0.116	0.018	0.093	84	817
GCTAGCATATATG	0.064	0.004	0.069	91	1236
AGTATTGCAGGGA	0.034	0.009	0.028	25	863
CGTAGTGTTAATG	0.027	0.007	0.024	21	867
CCCGGCGTAAAGA	0.021	0.003	0.021	46	2121
CCTGTTACTAGTG	0.029	0.004	0.026	29	1106
GCCGGTGCTTGTA	0.07	0.017	0.057	45	744
CCCGGTATTTGTA	0.03	0.005	0.037	64	1682
GGCGGCGCTGGGG	0.174	0.023	0.157	57	307
CCTGTTGTTTATG	0.024	0.004	0.021	27	1243
GGTATTGTAGGGG	0.07	0.028	0.048	24	473
AGTAGCGTATGGG	0.014	0.004	0.013	7	549
CCCATCACAGGTG	0.03	0.005	0.024	43	1756
AGCGTCACAGATG	0.026	0.003	0.028	46	1597
GGCGGCGTTAGCA	0.208	0.046	0.176	148	695
ACCATTACAGGGG	0.024	0.004	0.03	41	1306
GCCGTTGTAAGTG	0.041	0.004	0.039	39	971
AGTGGCATTAGTA	0.027	0.01	0.017	11	644
CGCGGCATTAATG	0.03	0.01	0.04	43	1023
CCCGTCACAAAGG	0.028	0.004	0.027	55	2020
AGTGGCATTGGGA	0.02	0.013	0.005	2	393
CCTATTATAGGGA	0.037	0.009	0.047	69	1407
AGTGGTGCAGAGG	0.021	0.006	0.017	8	474
ACTGGCACTGGGA	0.027	0.003	0.032	26	795
GGCGGCACTAGTG	0.277	0.036	0.253	201	592
CCTGGTGTTAGGA	0.02	0.004	0.014	13	898
GGTATTGTTGGTA	0.088	0.014	0.072	51	656
GGCATCGCATATG	0.11	0.018	0.099	129	1179
GCTGTCGTTGAGG	0.056	0.017	0.032	13	396
AGCAGCATTAAGG	0.02	0.001	0.021	37	1697
CCTAGTATTAGGG	0.032	0.007	0.024	23	938
GGTAGCATTGGGG	0.072	0.012	0.06	28	438
CGTAGCGTAGGCA	0.028	0.003	0.024	21	862
GCCAGTGCATATG	0.052	0.007	0.046	51	1051
GGTAGCGCAGATA	0.074	0.004	0.07	60	802

CGTAGCATTGAGG	0.031	0.003	0.028	22	759
CCCATCATAAAGG	0.035	0.006	0.026	76	2812
CGCAGTGCTAGTG	0.022	0.001	0.021	18	852
CGCATCGTTGGCA	0.026	0.004	0.021	32	1498
GGTGGTACAGGTA	0.071	0.005	0.073	50	637
GGCGTCACAGACG	0.069	0.005	0.071	88	1154
CCCGGCATTTACA	0.023	0.006	0.016	44	2693
GCCATTACAGGGA	0.047	0.006	0.039	54	1337
GGCGGTGTTAGGG	0.085	0.015	0.066	27	385
CCCAGCGTTGGTG	0.025	0.007	0.022	26	1154
GGCGGTACAGGGG	0.08	0.019	0.084	35	381
GGTATTGCTAAGG	0.096	0.017	0.072	50	640
CGTGTCACATGTA	0.03	0.004	0.031	33	1045
AGTGTCGTAGATA	0.022	0.003	0.02	26	1244
CCCGGTGCATAGG	0.029	0.006	0.037	35	911
GCTATCGCTGAGG	0.065	0.001	0.066	43	609
ACTGGCACTGATG	0.026	0.001	0.026	31	1146
GCCGTCACTTGCG	0.046	0.007	0.041	43	998
AGTGGCGCTGGTA	0.028	0.012	0.011	6	526
CCCGTCGCATGGG	0.027	0.005	0.021	20	913
GGCGTCACATGCG	0.061	0.005	0.055	52	900
GCCGTTACTTGGA	0.047	0.004	0.052	51	935
AGTAGTGCAAATG	0.022	0.004	0.028	37	1274
GGCGGTGCTAGTG	0.077	0.014	0.062	38	579
GGTATCACAAGCG	0.125	0.011	0.112	92	732
AGTGTTGTTGGTA	0.018	0.006	0.013	10	739
ACCGTTGTAGACA	0.022	0.002	0.02	52	2572
GCTGTTGTTGACG	0.069	0.014	0.052	42	761
CGCAGCATAAGTG	0.02	0.004	0.016	24	1517
GGTGTTATTGGTG	0.096	0.009	0.085	36	388
AGCGTTGTAGACG	0.025	0.002	0.022	34	1479
GCTAGCGCTGACG	0.065	0.011	0.077	56	671
GCCGGCGTTAAGG	0.939	0.017	0.938	1235	81
GGTAGTACTAGCA	0.066	0.018	0.05	44	832
GCTATTATATGGG	0.066	0.013	0.053	53	952
GCTGGTGCTAACA	0.115	0.011	0.122	133	953
AGTAGCATTAACA	0.021	0.003	0.017	43	2421
GCCGTTGCTAGCG	0.047	0.005	0.041	33	769
GCTATTGCTAAGG	0.048	0.017	0.057	52	855
AGCGGCACAAGTA	0.022	0.003	0.02	35	1741

**CGTATCATTTAGG**	0.025	0.002	0.024	26	1044
**GCTGGCGCTAAGA**	0.64	0.019	0.633	754	438
**GGCGTCGCTAGCG**	0.065	0.03	0.033	22	647
**CCTAGTACAAAGG**	0.026	0.002	0.029	53	1787
**GCTGGCGCTTGCA**	0.612	0.052	0.605	624	407
**CGTGGTATAGGGG**	0.013	0.004	0.019	7	362
**CGTGGCACTAGGA**	0.029	0.003	0.024	17	682
**GGCATCACAGGTG**	0.089	0.014	0.073	79	1008
**GCTGGCGCTGATG**	0.816	0.031	0.805	782	189
**AGCGTCACTGGCG**	0.025	0.007	0.018	22	1199
**GCTAGCGTTAGCG**	0.086	0.022	0.063	43	644
**GGTGTCACTTGCA**	0.072	0.009	0.074	49	614
**AGTGGTGTAAGGA**	0.02	0.002	0.018	14	746
**GGCGTTACAGGTG**	0.051	0.007	0.041	30	697
**CGCGGCACTAGGG**	0.019	0.004	0.014	9	630
**CGCGTTGCAAATG**	0.03	0.002	0.03	46	1484
**ACTGGTATAGAGG**	0.025	0.002	0.028	28	971
**CGCGTTGTAAACG**	0.023	0.003	0.02	34	1692
**GCCGGCATTGAGA**	0.393	0.012	0.39	566	884
**GGTGTTACTAATG**	0.086	0.01	0.074	65	810
**GCCAGTGTAAGCG**	0.05	0.006	0.047	61	1237
**ACTAGCGTAAATG**	0.028	0.001	0.028	50	1751
**GGCGTTATAGGGA**	0.058	0.018	0.037	36	949
**CGTATTACTTATG**	0.023	0.001	0.021	29	1322
**GGTGTCGTTTACA**	0.079	0.007	0.08	61	701
**CGTGTTGTTAGGG**	0.025	0.006	0.028	12	420
**CGTAGTATTAGGA**	0.019	0.006	0.022	21	918
**GGCGGTATAGACG**	0.117	0.014	0.114	121	941
**CCCGGTATATGTA**	0.025	0.003	0.025	54	2072
**ACTAGCACTGGGA**	0.029	0.001	0.03	34	1084
**GGCATTACAGGGA**	0.06	0.005	0.055	64	1096
**CGTGGCACATACA**	0.024	0.001	0.023	31	1315
**GGTAGTGTATACA**	0.062	0.001	0.06	63	986
**AGCGGCGCAAGTG**	0.03	0.007	0.023	21	912
**ACTGTCGTTGGGG**	0.036	0.004	0.033	16	462
**AGTGGTATTGGCG**	0.024	0.009	0.034	18	506
**GCCAGTGTTAGCA**	0.055	0.007	0.046	68	1400
**GCCATTATTGGCG**	0.058	0.004	0.061	106	1635
**GGTAGCGCTTATA**	0.067	0.014	0.057	42	700
**CGTAGCGCAAGGG**	0.022	0.009	0.033	18	524

AGTGGCGCAGAGA	0.026	0.007	0.021	14	654
CGTAGCGCTGGCA	0.024	0.005	0.023	18	763
ACTGGCACTAGCA	0.027	0.004	0.03	51	1647
CCCGTCATTTATA	0.025	0.002	0.027	74	2665
AGCAGCGCAGGTA	0.021	0.006	0.015	22	1428
AGTGGTGTATGGG	0.023	0.001	0.024	10	407
GCCGTTATTGGGA	0.05	0.004	0.051	49	915
AGTATTGTTAACG	0.025	0.003	0.029	49	1670
ACCGTCGTTAGCG	0.025	0.004	0.021	25	1174
GCCGGTATATATA	0.079	0.005	0.076	165	2002
GCTAGTGCTAGTG	0.052	0.005	0.046	30	625
GGCGGTACTAGTG	0.087	0.007	0.08	52	597
ACCGGTGCATACA	0.021	0.002	0.023	67	2801
GGCGGTGTTAGTA	0.073	0.018	0.055	40	685
AGTGTCACTGATA	0.028	0.005	0.021	29	1328
CGTGTCGTTGATG	0.026	0.006	0.02	13	640
AGTGGTGCAGGCA	0.023	0.001	0.023	18	756
CGTGGCGCATGTG	0.023	0.002	0.026	12	456
CGTGTTACTGATA	0.023	0.005	0.03	39	1277
GCTGTTACAAGGG	0.057	0.011	0.066	48	684
ACTAGCATTTGGG	0.03	0.001	0.03	25	796
GCCATTGCAGGCA	0.042	0.005	0.045	68	1427
GGTATTGCTTGCG	0.115	0.026	0.107	54	449
GCCGTCATATGGG	0.07	0.01	0.062	48	729
CGCGTTATATGTA	0.022	0.001	0.023	42	1779
CGCGGCGTTGGGG	0.021	0.004	0.023	9	384
GCTATTACTGGCA	0.058	0.002	0.058	78	1259
AGCATCGTTAATA	0.021	0.002	0.02	66	3263
GCCATTACTGAGG	0.056	0.007	0.049	63	1210
GCTAGTGCAAGCA	0.055	0.004	0.052	75	1372
ACCAGTGTTGAGA	0.027	0.002	0.028	50	1765
GCCGTTGTAGGTA	0.051	0.01	0.039	42	1039
CGCAGCGCAGGTA	0.021	0.005	0.027	33	1210
GGCGTCATTTGTG	0.067	0.015	0.052	38	693
GCCGTTGTAGGTG	0.055	0.005	0.05	36	690
GGTATTGCTTATG	0.094	0.013	0.076	53	646
CCTGGTATTTGCG	0.023	0.005	0.018	16	886
GGTGGTGTTGAGG	0.191	0.02	0.182	57	256
AGTGTTATTTGTA	0.025	0.003	0.024	30	1227
GCCATCGTTTACA	0.046	0.005	0.042	84	1896

AGTGTCACTTGTA	0.026	0.011	0.014	15	1023
CCCGGCACAGGTG	0.029	0.003	0.033	43	1267
GGCGTCATAGAGA	0.059	0.012	0.048	61	1221
AGTAGCGTTGGCG	0.021	0.006	0.02	14	689
GGCATTACAGGGG	0.05	0.013	0.047	37	755
GGCGTTATTTATA	0.063	0.016	0.047	74	1490
CCTGTCGTATAGG	0.019	0.002	0.022	22	977
CCCGTTGTTTGCA	0.028	0.004	0.024	36	1473
ACTGGCACTGACG	0.032	0.003	0.029	40	1324
GGCATTGCAGGGG	0.061	0.006	0.07	41	547
CGTAGCGTTGATA	0.025	0.007	0.02	22	1052
GCCGTTGCTGAGG	0.05	0.013	0.032	22	661
GCTGGCACTGGGG	0.46	0.051	0.46	209	245
ACTGGTGTAGGCA	0.025	0.006	0.017	20	1158
CCCGGTGTAAATG	0.026	0.004	0.026	50	1873
AGCATCATAGGTG	0.018	0.003	0.016	32	1943
CGTATCATTGGGG	0.02	0.006	0.024	15	599
CCTGGCGTTGACG	0.027	0.011	0.014	14	954
ACTGTTACATGTG	0.026	0.002	0.028	41	1406
AGTGGTGTTAGCA	0.018	0.001	0.018	15	817
CGCGGTGCTTGGA	0.02	0.004	0.014	10	680
GGTGTCGTATGCG	0.102	0.007	0.098	39	357
CGCAGTGCAGGTG	0.027	0.007	0.02	17	836
CGCGTTGCAAACA	0.023	0.001	0.023	55	2385
ACTAGTGCAGATG	0.023	0.002	0.025	44	1692
GGTGGCATTAGGA	0.469	0.075	0.398	280	424
ACTAGCGTAGGCA	0.026	0.009	0.017	24	1391
GGTAGCATAAGGA	0.069	0.01	0.055	50	855
CCTAGTATTAGGA	0.025	0.001	0.024	36	1494
GCTGGTACTAACG	0.108	0.018	0.102	95	834
CCTGGTGCAGGGA	0.028	0.004	0.032	27	827
GCTGTTGTTGGCA	0.056	0.01	0.048	36	719
GGCGGCGCATAGA	0.111	0.031	0.088	72	748
GGTAGTGCAGGCA	0.047	0.009	0.051	34	629
GCTAGCGCAAGGA	0.059	0.008	0.05	38	723
GCCGGTACAAGCG	0.073	0.011	0.062	74	1113
CGTATCGTTAATG	0.025	0.001	0.025	33	1296
AGCGTCGCTAGGG	0.024	0.008	0.018	12	669
CCCATCGTTAAGG	0.023	0.004	0.021	37	1698
ACTGTCGTAGAGG	0.025	0.001	0.023	17	723

CCTGGTGTTAAGG	0.021	0.002	0.023	22	920
GGCGGTGCTGATA	0.045	0.008	0.053	45	798
CCTAGCACAGGTG	0.028	0.005	0.031	35	1089
ACTAGCACAGGCG	0.026	0.004	0.033	47	1397
GGTGGTGCAAGTG	0.094	0.027	0.064	26	382
ACCGGTATAGGTG	0.026	0.006	0.034	43	1228
GCCGTTACAAGCG	0.063	0.004	0.063	85	1262
GCCAGCGTTAACG	0.064	0.006	0.055	77	1326
AGCGGTACTAATA	0.021	0.004	0.015	37	2381
GCCGGCGCAAGCA	0.49	0.041	0.466	783	899
AGTATTGCATGCA	0.025	0.006	0.018	25	1371
GGCAGCGTAGACG	0.067	0.008	0.065	74	1062
CGTAGCGTTGGGG	0.02	0.002	0.021	8	366
GCCGGTGTTTGTA	0.176	0.023	0.171	146	709
ACCGTCGCAGGCG	0.021	0.003	0.021	30	1392
GCCAGTGTATAGA	0.042	0.008	0.051	79	1479
CGCAGTGTAGGCG	0.025	0.004	0.03	29	934
GCTGGTGTTTGTA	0.427	0.054	0.413	321	457
ACTGGCACTTATA	0.022	0.001	0.023	43	1794
GGCGTTATTTGCG	0.049	0.008	0.041	30	696
CGTGTCATATGGG	0.028	0.003	0.03	19	621
GCTGTCGTAGGGA	0.06	0.014	0.041	25	592
GCCAGTGTAGGGA	0.054	0.005	0.053	50	901
GCCAGTATATGCG	0.054	0.002	0.053	84	1502
GGTAGCGCTTAGG	0.084	0.003	0.08	29	333
GCCGGTACAAAGG	0.066	0.007	0.064	74	1086
GGTGTCGCTGGTA	0.077	0.005	0.071	34	445
GGTGTTATATGCA	0.081	0.011	0.066	59	837
CGTATCACAAGCG	0.026	0.005	0.029	36	1198
CCCGTTGTTGGTG	0.025	0.004	0.03	29	929
ACCGGTGTTAATA	0.022	0.002	0.022	54	2438
GGTGTTATAGGGA	0.065	0.007	0.075	44	541
GCCGGTGCAAACA	0.066	0.013	0.055	99	1689
GCTAGCACTAACA	0.06	0.002	0.058	120	1946
CGTGGTGCTTACG	0.026	0.002	0.029	16	544
AGCGTCGTATGCG	0.021	0.002	0.02	23	1137
GCTGTTGTAAGGG	0.064	0.002	0.061	41	630
GGTATCGCAAGGG	0.137	0.041	0.132	64	422
CGCAGTACTTGGA	0.023	0.003	0.019	20	1032
GCCATTGTATACG	0.054	0.007	0.047	83	1681

GCCAGTGCTGGCA	0.044	0.01	0.031	35	1087
CCTAGTACTTGCA	0.024	0.005	0.018	34	1848
GCCGGCGCATGCA	0.5	0.032	0.469	645	729
ACCGGCATAGGCA	0.024	0.003	0.021	44	2036
ACCGTTGCTTGGG	0.021	0.008	0.032	29	864
ACTGGTATAAAGG	0.025	0.004	0.023	35	1471
CGCGGTGCAAAGA	0.018	0.004	0.017	24	1402
ACTATCACATGGG	0.028	0.005	0.027	36	1301
ACTATCGCTTGTA	0.022	0.003	0.02	37	1843
CGTGGTACAAGCA	0.026	0.005	0.025	32	1263
ACTGGTGTATAGA	0.022	0.007	0.032	42	1279
GCCATTGTTAGGG	0.042	0.008	0.035	28	773
AGTGTTGCATGTG	0.021	0.008	0.011	8	688
GGCGTTGTATATA	0.064	0.011	0.055	78	1334
GCCGGCACAAACA	0.102	0.006	0.105	284	2429
GCCGTCGTAAATG	0.054	0.007	0.063	78	1159
CGTGTCGCTTACA	0.023	0.004	0.027	29	1056
GGCATTACTGAGA	0.054	0.006	0.046	69	1428
GGCATCGTAAAGA	0.11	0.011	0.099	197	1789
CGCATCGTTTGCA	0.024	0.004	0.019	31	1600
CGCATTGTAGGTA	0.024	0.009	0.018	27	1509
ACTGTTACAAAGG	0.031	0.007	0.023	43	1811
AGTGTTACTTAGG	0.023	0.006	0.015	12	800
GCTGTTACTAGTA	0.063	0.017	0.043	48	1071
ACTAGTGCAAGGG	0.025	0.003	0.028	36	1270
GCTATTGTTGAGG	0.059	0.011	0.049	43	826
CCCGGTGTTGGCG	0.023	0.001	0.023	23	999
GCCGTTGTAGATG	0.053	0.002	0.05	53	1000
GGTGGTGTTGAGA	0.105	0.013	0.106	55	463
CCTGGCGTTAGTG	0.031	0.009	0.028	20	705
CGTAGTGTAGGGA	0.024	0.004	0.025	16	632
CCCAGTACTGGGA	0.028	0.005	0.026	37	1371
CGCGGCGCTGGCG	0.025	0.003	0.024	18	739
GGTGTCGTTTGGA	0.087	0.016	0.084	33	361
ACCATCATAAGCG	0.027	0	0.027	97	3483
CGCGGCACTGAGG	0.018	0.006	0.011	8	737
GCCATCGCTGAGA	0.043	0.006	0.044	52	1131
GGCATCGCTTGCG	0.086	0.018	0.063	54	799
GCCATCATTGGGA	0.059	0.011	0.066	82	1157
GGTGGCATTAACA	0.486	0.054	0.444	743	931

CCCGGCACTGAGA	0.021	0.003	0.018	32	1706
GCCAGTATTTGCA	0.05	0.007	0.055	109	1881
AGTATTGTTGGGA	0.023	0.004	0.029	26	872
GGCGGCGCTAGTG	0.194	0.049	0.176	99	462
GCTGTTATTGGCG	0.058	0.011	0.043	35	784
GCCATTGCATATA	0.046	0.003	0.045	84	1766
GGTAGCGCTGATG	0.078	0.008	0.068	32	436
AGTGGTGCTTGGG	0.018	0.006	0.023	7	297
CCCGGCGTATGGA	0.018	0.004	0.018	23	1282
AGTGTTGCTAAGA	0.021	0.003	0.021	22	1031
ACTATCATTAGCG	0.034	0.007	0.026	56	2089
GCTGTCGCTTACG	0.066	0.008	0.069	46	622
AGCGTTGTTGGTG	0.027	0.001	0.028	22	759
CCCGGTACATAGG	0.022	0.005	0.029	42	1399
CGCGTCGTTTGGA	0.022	0.001	0.023	17	735
GGTGTCGTAAGTG	0.108	0.008	0.099	56	508
AGTGTTGCAGACG	0.018	0.006	0.015	13	855
CCCGGCGTATATA	0.022	0.002	0.021	51	2324
AGCGGTACTTGGA	0.015	0.002	0.014	14	1009
GCCATTGCATACG	0.051	0.007	0.042	64	1477
CCCAGTGCTTGTA	0.026	0.002	0.029	50	1690
ACTGGTGCAGGGA	0.022	0.005	0.019	17	897
GCTGTCGCAGGTA	0.059	0.005	0.052	27	496
CCCAGTGCTGATG	0.027	0.001	0.029	44	1467
GGCGGCGCTGACG	0.175	0.046	0.127	98	673
GCTGGCATTAATG	0.869	0.022	0.87	1664	248
GCCATTGCTAGGA	0.042	0.003	0.039	42	1039
CGCAGCGCAGACG	0.031	0.001	0.03	41	1324
CGCGGTGCAAGTG	0.025	0.005	0.02	18	883
GCTGGCACATGTA	0.206	0.021	0.2	196	784
CGCGGTACATGTG	0.025	0.006	0.017	16	931
CCTGTTACAAGTA	0.027	0.003	0.024	52	2083
GCTGTTGCAAAGG	0.057	0.008	0.047	35	713
GCCAGCACTTAGG	0.059	0.006	0.053	58	1036
GGCGGTACAAGTG	0.072	0.01	0.075	61	757
GGTGGTATAAAGA	0.148	0.028	0.14	154	943
AGTGGCGCTGGGA	0.033	0.008	0.022	9	409
GGCGGTACTTGCG	0.061	0.019	0.046	31	636
AGTGGCGTTAAGG	0.013	0.008	0.024	8	328
AGCGTTATTGGGG	0.024	0.004	0.018	15	802

CGTGTTATATGCA	0.025	0.004	0.019	25	1291
CGCATCACATGGG	0.03	0.008	0.023	29	1218
GGCGGTGTTGATG	0.082	0.009	0.09	59	597
CGTGTCACAAGTA	0.025	0.003	0.022	26	1183
CCCAGTATAGGTG	0.03	0.004	0.024	39	1597
ACTGGTATTTGCG	0.034	0.005	0.039	37	905
AGCGTCACAAAGA	0.021	0.006	0.016	41	2575
GGCGTCACATATG	0.065	0.005	0.064	75	1100
GCCATCGCAAGCG	0.053	0.008	0.043	55	1231
CCCGGTGTTGAGA	0.025	0.008	0.017	20	1190
CGCGTCACAGGCG	0.019	0.007	0.013	16	1249
CCTATTGCTTGTG	0.024	0.003	0.026	31	1142
CGTATTGTAGGTA	0.024	0.003	0.019	22	1122
CGTGGTATTTGCA	0.027	0.005	0.02	16	774
GGTGTCACAGAGG	0.11	0.02	0.093	44	429
CGTAGCATTGGTG	0.02	0.004	0.022	15	659
GGTGTCATTTGGA	0.084	0.013	0.084	39	425
GGCAGCGCTGAGG	0.066	0.015	0.051	32	592
GCTGTCGTAAGTA	0.056	0.011	0.043	45	1003
CGCGGCACTGAGA	0.025	0.005	0.019	22	1166
GGCAGCGTTGAGA	0.059	0.013	0.044	41	886
GGTGGTGCTTACG	0.088	0.018	0.063	27	401
GGCAGCGCTTACA	0.051	0.005	0.057	82	1345
ACCGTCGTTGGCG	0.024	0.003	0.026	25	938
GGCGTTACTGAGA	0.055	0.007	0.047	48	969
AGCAGTGCTAGGG	0.021	0.002	0.017	15	850
GGCGTTGCTAACG	0.072	0.011	0.057	59	970
ACCAGCGTTAGCG	0.03	0.006	0.036	50	1332
GGTGTTGCTTACA	0.061	0.01	0.047	36	726
GCTGTCATTGATG	0.071	0.007	0.065	58	839
CGCAGTATTGGTA	0.022	0.002	0.021	32	1501
ACTATCGTTGGTG	0.029	0.004	0.023	22	933
GCCAGCGCTAAGG	0.047	0.008	0.058	46	749
CGCATCGTTGGGA	0.027	0.001	0.028	30	1025
AGTGTTGTAAGCA	0.027	0.006	0.035	47	1313
CGTGGTATAAAGG	0.018	0.003	0.014	12	831
GGCATCACTTAGA	0.07	0.016	0.061	87	1329
CGTAGCACAGGCA	0.026	0.01	0.013	14	1061
AGCGTCGTTTACG	0.015	0.004	0.019	24	1224
GGTATTATATGTG	0.124	0.016	0.108	121	1000

CGTGGTATTGGTA	0.027	0.007	0.023	17	734
ACCGTTGCAAGTG	0.024	0.001	0.023	41	1733
GGTATTATATAGA	0.102	0.013	0.1	166	1497
GGCGTTATTAGTA	0.057	0.009	0.046	62	1283
AGTATCACTTGTG	0.03	0.008	0.041	35	827
GCCGTCGCAGATG	0.049	0.009	0.041	30	695
GCCGGCGCTGGTA	0.508	0.052	0.533	483	423
GCCATTGTTTATA	0.056	0.004	0.053	109	1937
CGTAGCATAAAGG	0.029	0.005	0.023	26	1113
CGTAGCATTTATG	0.025	0.006	0.023	24	998
GGCGGTGCTGACG	0.066	0.004	0.065	45	649
ACTGGCACATGGG	0.036	0.006	0.028	22	763
GGCATTGTAAACG	0.077	0.016	0.067	117	1633
ACCAGCGTTTGTG	0.024	0.01	0.012	14	1126
GCCAGCACAGGCG	0.053	0.007	0.061	79	1206
GGTGTCATAGGTA	0.087	0.005	0.08	57	657
CCCAGTGTTTGCG	0.024	0.004	0.021	30	1370
CGTAGCATAAGGG	0.023	0.003	0.019	14	704
GGCGTTGTTTACG	0.075	0.008	0.074	59	742
GCTGGCGCTGGCG	0.802	0.037	0.788	569	153
GCCGGTATTTGGG	0.146	0.032	0.107	71	595
AGTAGCACTGGGG	0.017	0.004	0.013	7	533
CGCGTCGTAGACG	0.025	0.003	0.023	28	1179
GGTATCGTATGCG	0.149	0.014	0.133	78	508
CGCAGTGTTTACG	0.022	0.005	0.017	20	1181
GGTGTTGCTTGTG	0.13	0.009	0.116	41	311
CCTAGTGTAAGGG	0.022	0.005	0.017	20	1162
CGTAGCGTTAGTA	0.024	0.003	0.024	21	851
GCCAGCGCATACG	0.056	0.012	0.044	54	1163
CCCGTCGTAGGGA	0.026	0.005	0.021	25	1189
GCCATCACTGGTA	0.049	0.004	0.049	78	1517
GGCGTCATTGAGG	0.066	0.014	0.07	51	677
GCCATCACTAGGG	0.061	0.008	0.068	67	919
CGCATTGTAAACG	0.028	0.003	0.024	56	2305
AGCATCACAAATG	0.023	0.003	0.027	94	3409
GGCATTACTTACG	0.067	0.015	0.048	72	1441
ACTGTTATAAGTG	0.025	0.003	0.024	42	1677
ACCGTCACAAAGG	0.023	0.001	0.024	58	2332
GCCGTCGTAAGTG	0.041	0.004	0.041	37	858
ACCGTTATAAGCG	0.025	0.006	0.024	55	2200

GGTGTTACAGAGA	0.083	0.023	0.076	62	755
AGTATCGTTAGCG	0.025	0.005	0.032	33	1003
ACCGGTGTTTATG	0.023	0.001	0.024	29	1201
GGCGGCGCTGGCG	0.146	0.036	0.13	72	482
GGCAGTGCAAGCG	0.048	0.008	0.038	37	927
CCTGGCACAGACA	0.027	0.002	0.027	58	2080
GGTATTGTTGATG	0.119	0.025	0.092	69	681
ACTGGCATTTGTG	0.028	0.001	0.029	23	776
AGCAGTGTTTGTA	0.023	0.001	0.025	39	1522
GGCATCGCAAGGA	0.073	0.014	0.057	64	1059
CGTGTTGTTAGCA	0.024	0.007	0.015	13	856
GGCGTTACTTGTA	0.056	0.017	0.043	46	1033
AGTGTCATAGATG	0.021	0.002	0.024	27	1097
CGTGTTGTTTGCG	0.029	0.009	0.023	12	500
GCTGTCATTTGCG	0.059	0.015	0.038	28	703
AGCAGTACTGAGG	0.022	0.003	0.025	33	1291
CCTGTTGTTTGGA	0.023	0.002	0.02	18	876
AGCAGCGCAGAGA	0.023	0.004	0.025	37	1439
AGTGTCACAGAGG	0.03	0.007	0.022	16	709
CGTAGTGTTGGCG	0.02	0.004	0.015	9	573
AGTAGTGCAGGTA	0.018	0.001	0.017	17	969
GGCATCGTTAGCA	0.103	0.001	0.104	149	1277
CCTATTATTGACG	0.026	0.002	0.025	55	2182
CGCAGTACTTGCA	0.025	0.002	0.024	38	1551
AGTGTCACAGGCA	0.019	0.005	0.024	26	1067
GGTGTTGTTGGTA	0.077	0.012	0.061	31	481
GGTAGCATTTGTG	0.069	0.011	0.055	26	447
CGTATCGCTGACG	0.04	0.02	0.019	18	916
AGTATCACTTAGA	0.027	0.004	0.022	29	1289
GCCGTCATTGATG	0.056	0.005	0.059	79	1256
GGCATTGTTTATA	0.074	0.005	0.067	106	1482
GGCATCACTAGTA	0.065	0.012	0.052	84	1546
GCCAGCACTAAGA	0.052	0.005	0.058	122	1984
GCTAGCACATACG	0.064	0.008	0.054	68	1201
GCTATTGTAGGGG	0.06	0.011	0.054	37	644
CGTGGTGTAAACG	0.024	0.004	0.022	20	874
GCTAGTGCTTGTG	0.06	0.006	0.051	32	592
GCTATCATTGAGA	0.053	0.004	0.05	82	1553
CGTATTGTAGATA	0.024	0.004	0.029	49	1627
GCTGGTGCAAACA	0.121	0.016	0.131	178	1180

ACTGTTGTAGGCG	0.024	0.004	0.022	28	1237
GGCAGTACATACA	0.057	0.007	0.052	117	2150
ACTATCGTAGACG	0.027	0.001	0.026	50	1871
GGCGGTGCATGTA	0.061	0.008	0.051	41	761
ACCGTTATATGCA	0.026	0.004	0.019	56	2820
GCTATCATAGGGA	0.07	0.004	0.075	70	864
GCTGTTATATGCG	0.068	0.009	0.055	46	788
CCCAGTACTAGGA	0.025	0.005	0.027	55	2011
ACCGGTATTGGCG	0.024	0.004	0.019	25	1293
GCTAGTATAAGGG	0.066	0.011	0.055	53	902
GGCAGCATATGTG	0.052	0.003	0.055	59	1009
GGTGGTATTGACG	0.26	0.02	0.242	162	508
GCTGGCACTGGGA	0.205	0.015	0.185	106	466
CGCGTTACTGATG	0.023	0.001	0.022	25	1130
ACTGTCATAAGGG	0.023	0.004	0.02	22	1079
CGCAGCGCTGACG	0.021	0.003	0.018	23	1277
CCCGGTGTTTGTG	0.02	0.009	0.009	8	857
GGCAGCATAGAGG	0.057	0.007	0.047	55	1109
GGCGGTGCAAGTG	0.069	0.01	0.076	50	608
CGTGGTACATGTA	0.023	0.006	0.019	19	1005
GCCAGCGCTTAGA	0.054	0.004	0.06	56	877
CGCAGTACTAGTG	0.025	0.004	0.031	37	1159
CGCATCGCAAGTG	0.026	0.002	0.027	40	1436
CCTGTTGCATGTA	0.022	0.004	0.024	35	1439
GCTATTACTGGCG	0.058	0.002	0.056	76	1275
AGCAGTGCTGGCG	0.022	0.003	0.023	26	1113
ACTGTTGCTGATG	0.025	0.003	0.024	30	1220
ACTGTTGTTTACA	0.027	0.004	0.028	51	1800
AGCAGTACTTGCG	0.021	0.003	0.018	26	1396
CCCAGCACTAGCG	0.03	0.004	0.026	50	1906
CGTGGCATAGGGG	0.035	0.003	0.032	11	331
CGTAGTGCTGGTA	0.024	0.004	0.019	13	674
GGTGGCATTGGGA	0.483	0.046	0.43	239	317
GGTGGTACATGCG	0.108	0.016	0.085	43	460
CGTGTTGTTAACG	0.028	0.007	0.02	19	923
CGTGGTGCTAGCA	0.03	0.001	0.03	22	713
AGCGTCACTAAGG	0.026	0.003	0.024	32	1321
AGTAGCGCTAGCA	0.023	0.007	0.018	18	989
CCTGTTGCTGGCG	0.023	0.005	0.017	15	894
GGTGGTGCTTAGA	0.063	0.004	0.06	29	451

GGCGTTACTGGTG	0.056	0.021	0.05	30	569
GCTGTCACTAACA	0.056	0.002	0.054	93	1619
GGCGGTATAAGCA	0.08	0.009	0.074	113	1407
GGCGTCGTAGGTG	0.073	0.014	0.065	39	562
GGTGGCATATGTA	0.511	0.056	0.473	500	557
CCCGTTGTAAGTG	0.026	0.007	0.021	32	1469
GCCATCGCTGACG	0.058	0.002	0.061	77	1195
CGCGGCGTTGATG	0.022	0.007	0.032	24	730
CGCGTCGCAGAGG	0.027	0.006	0.021	17	803
CGTGGTACAAACG	0.02	0.004	0.015	17	1129
CCTGGTGCAGACG	0.028	0.006	0.035	39	1074
CGTAGTGTTAACG	0.016	0.005	0.011	10	877
CCTAGTATTAAGA	0.027	0	0.027	63	2276
CGCGGCGTTAGCA	0.016	0.003	0.017	19	1081
AGCAGCGTTTGTG	0.021	0.002	0.023	23	961
GCTAGCGTATAGG	0.069	0.013	0.065	40	575
GGTATCACTTGTA	0.087	0.015	0.068	60	821
GCCAGCATTTAGA	0.056	0.004	0.051	99	1844
ACTGGTGCAAGTG	0.027	0.002	0.024	24	970
CCCATCGCATGGG	0.022	0.005	0.015	19	1287
AGCAGTATTTGCG	0.022	0.001	0.022	36	1570
GCCAGCGCTTGCG	0.054	0.007	0.047	36	736
GCCATTGTAAATG	0.045	0.001	0.047	105	2123
GGTAGCGTAAATA	0.075	0.009	0.082	104	1162
CCCAGTGTAGATA	0.023	0.003	0.028	73	2560
GGTAGCATTTGGG	0.094	0.027	0.069	24	324
GGCAGTGTTAGTG	0.055	0.016	0.033	27	784
AGTATCATAAGTG	0.023	0.002	0.02	32	1537
CCCGGCATAGGGG	0.034	0.009	0.044	42	907
GCCAGCGCTTGGA	0.06	0.013	0.066	54	760
GGCAGTATATAGG	0.057	0.012	0.039	52	1266
CGTAGCGCATATA	0.025	0.002	0.025	31	1197
GGCATTGTTGACG	0.084	0.001	0.086	99	1055
GGCGTCGTATACG	0.067	0.006	0.062	62	946
GGTATCGCAGATG	0.144	0.008	0.143	109	651
ACCGTTGTTAGTA	0.027	0.006	0.021	36	1697
CGTGGCATATGGA	0.021	0.005	0.02	13	643
AGTGTCATTGATG	0.027	0.003	0.026	27	1006
AGCATTACTTAGA	0.022	0.002	0.021	51	2329
AGTGGCGTATGGG	0.04	0.011	0.036	7	188

CGTATCGCTAGGG	0.017	0.003	0.02	12	578
CGCGTCGCAAGTA	0.024	0.007	0.025	40	1555
AGTAGCATTGGCA	0.029	0.002	0.027	33	1189
CGCAGTATAGGGA	0.03	0.004	0.03	35	1125
AGCGTCGCAAGTG	0.023	0.001	0.022	26	1166
GGCGGCATTTGTA	0.289	0.057	0.257	268	775
AGTGGCACTAGTG	0.023	0.006	0.03	14	448
ACCATCGCTGAGG	0.02	0.005	0.019	27	1413
ACTATCGTTAACG	0.028	0.004	0.025	54	2096
GCTGGCACTGGTG	0.459	0.041	0.476	303	334
GGTATTGTTGAGG	0.111	0.014	0.119	62	459
CGTGGTGCTAATG	0.023	0.004	0.018	11	603
AGCAGCGTTGGCG	0.023	0.002	0.025	27	1075
GGTGTTACTGGCG	0.071	0.013	0.065	32	464
ACTAGCGTAAGTG	0.028	0.002	0.028	32	1117
GGTGGCACAGACA	0.2	0.033	0.192	217	911
GCCGGTGTATACA	0.175	0.026	0.165	288	1459
CGTAGCACTAACG	0.021	0.001	0.023	28	1195
AGTAGCGTAAGGG	0.022	0.007	0.015	10	676
GCTGGTACAGAGG	0.111	0.008	0.101	55	492
ACCGGTGCTAGCG	0.023	0.002	0.02	25	1239
GCTAGTACTTACG	0.057	0.006	0.049	58	1135
ACTGTCGCATACA	0.024	0.002	0.024	58	2348
CCCGGTGTTTATA	0.026	0.007	0.016	28	1676
GGCATTGCAAAGG	0.07	0.008	0.07	84	1120
GCTAGCATTAACA	0.058	0.007	0.053	117	2102
AGCGTCGTTAATA	0.026	0	0.026	57	2136
CCTGGTATATAGG	0.03	0.003	0.03	32	1028
ACCGTCGTTTATG	0.028	0.007	0.021	25	1180
CGCGTCATAGGGG	0.044	0.009	0.056	42	702
CGCATTACTGGGA	0.021	0.004	0.026	30	1121
CGCGTCGTTGGCA	0.025	0.003	0.021	22	1027
CGCGTCGTATACA	0.02	0.004	0.015	31	1997
GGTGTTATATAGG	0.073	0.013	0.055	38	654
ACCGGCACAGGGG	0.028	0.003	0.028	25	855
CGCGGCATTTATG	0.023	0.009	0.011	10	864
ACCGGTGTTGGGA	0.024	0.01	0.016	15	923
AGTGGTACTAACA	0.026	0.003	0.026	43	1603
ACTGTTGCTTGTA	0.021	0.002	0.023	31	1330
CGCAGCGTTGAGG	0.025	0.007	0.025	18	700

CGCGGCGCATGCA	0.026	0.008	0.018	21	1177
GCCGGCACTGAGG	0.207	0.029	0.2	168	672
CGTGGTGTTAGGA	0.027	0.002	0.028	15	512
CCTGGCGCTGGCG	0.026	0.005	0.019	14	725
ACCAGTGCTAGGA	0.021	0.002	0.022	43	1931
CGCATCGCATGTG	0.032	0.006	0.026	29	1087
GGTAGTACATGTA	0.062	0.01	0.05	50	956
AGTGGCGTTAATA	0.019	0.005	0.012	12	969
AGCGTTGCTAATG	0.027	0.002	0.029	41	1369
CGTGTCACAGAGA	0.024	0.002	0.023	24	998
GGTAGTGCTTATA	0.063	0.009	0.063	52	779
AGTGTTGCAAATG	0.022	0.002	0.023	27	1152
GGCGTCGTTTATG	0.076	0.008	0.077	54	647
GCCAGTGTAGGCA	0.044	0.008	0.034	48	1379
AGCGGTACAAGCG	0.023	0.001	0.023	31	1303
GGTATTGTTGGCG	0.091	0.006	0.088	61	630
CCCATTGCTTGGA	0.027	0.004	0.027	39	1417
AGTGTCATAGGTA	0.018	0.004	0.011	13	1132
GCCAGTGTTAGGA	0.051	0.017	0.042	41	930
GCTGGTATAGGGG	0.224	0.037	0.186	91	398
ACCGTTACATGGG	0.021	0.002	0.021	34	1580
CGCGTCACTGGGG	0.026	0.004	0.029	16	545
CCCATCGTAAGTG	0.024	0.003	0.02	42	2034
GGCGGCACAGGGG	0.247	0.031	0.219	126	449
ACCGGCATAAATG	0.025	0.004	0.026	65	2449
CCTAGCGTATAGG	0.022	0.003	0.024	27	1080
AGCGGCGCAGACA	0.019	0	0.02	33	1642
GCCGGTGCTAGCG	0.096	0.016	0.087	62	651
GCTGGTATAAACG	0.436	0.018	0.431	688	909
CGTGGTACAAGTA	0.026	0.007	0.035	43	1196
CCTGTCGTATAGA	0.024	0.002	0.024	35	1398
CCTGTTGCTAACG	0.021	0.003	0.017	27	1551
AGTGGCGTTTGCA	0.015	0.005	0.013	8	591
GCTAGCGTTAATG	0.074	0.005	0.077	74	883
GCTAGCATTGAGG	0.061	0.009	0.059	49	779
GGTATTGCTTGTA	0.073	0.014	0.056	41	685
CGCATTGCATGGG	0.027	0.003	0.023	21	880
AGCATCACTAGTA	0.021	0.002	0.019	49	2507
GCTAGTATATAGA	0.056	0.006	0.049	86	1684
GGTGTCACTGGCG	0.079	0.005	0.072	35	449

AGTAGTGTTTGCA	0.025	0.012	0.042	44	1012
AGCGTCATAGACG	0.021	0.007	0.015	29	1881
GGCAGCGCAAAGA	0.056	0.005	0.05	67	1285
AGTATCGCTTAGG	0.021	0.007	0.012	9	728
CGCATCGTATGTA	0.02	0.001	0.019	35	1809
AGTAGTGCTTACG	0.023	0.003	0.019	16	818
GGTGTCATATACG	0.096	0.021	0.066	53	750
CGCATCATTGATA	0.025	0.004	0.019	53	2666
GCTGTCATTAAGA	0.064	0.007	0.059	83	1329
GGCGTTGTTTGTA	0.066	0.011	0.057	43	711
GGCGGTACTTACG	0.089	0.014	0.069	61	817
ACTATTGCTGGCA	0.022	0.001	0.023	47	2030
GGTGTCATATAGA	0.093	0.002	0.091	73	732
GGTATTACTTGGG	0.098	0.012	0.085	48	517
AGTGGTATAAGGG	0.026	0.004	0.029	19	637
CGTATCATTAGTA	0.027	0.005	0.019	33	1685
CCTATTATTGGGG	0.038	0.005	0.032	31	946
AGCGGTGTAAGTA	0.024	0.007	0.017	28	1648
AGTGTCGCTTATG	0.024	0.005	0.026	20	760
CGTAGTATAGGTG	0.024	0.004	0.02	16	792
ACTGTTGTAGGGA	0.028	0.003	0.026	25	921
AGTGTTGCTGGCG	0.02	0.006	0.027	16	574
ACTGTCGTAGGTA	0.028	0.002	0.028	31	1095
GCTGTTATAGGGG	0.071	0.009	0.078	39	461
AGCGTTGCTGGCG	0.022	0.004	0.017	17	1010
CGTAGCGTAGGGG	0.03	0.019	0.007	3	413
CGCGGTGCAGGGG	0.025	0.003	0.026	13	493
ACCAGTGCTTACG	0.021	0.002	0.018	40	2128
AGTAGCGTTTGTG	0.023	0.003	0.02	12	594
CGTATTGTAGGGG	0.039	0.01	0.03	17	542
GCTGGCACAGACA	0.217	0.024	0.209	308	1165
AGCAGTGTTTGGG	0.027	0.008	0.017	15	845
ACCGGCGCTAGCG	0.026	0.003	0.022	24	1065
ACTAGCACAAAGG	0.025	0.003	0.03	59	1934
ACCGGCGCTGGTA	0.026	0.005	0.023	21	910
GGCATTATTTGCG	0.059	0.008	0.07	83	1095
GGCGTCACAGAGG	0.06	0.004	0.065	51	738
ACTGGTGTTGAGG	0.019	0.004	0.023	16	676
AGCGGTACATAGG	0.014	0.001	0.015	15	1004
CGTATTGTAGAGG	0.029	0.011	0.019	16	813

AGTATTGCAAAGG	0.028	0	0.028	37	1286
CGTGGCGTTGGCG	0.016	0.005	0.01	4	386
GCCAGTGTTTGCA	0.049	0.01	0.035	47	1302
CCTGTTGTATAGA	0.024	0.001	0.024	39	1560
ACCGGTACTGGGG	0.026	0.003	0.024	21	842
GCCGTCGCTGGCG	0.057	0.003	0.054	35	611
CGCGTTACAGACA	0.021	0.002	0.024	54	2183
GGCAGCGCAGGCA	0.054	0.015	0.045	46	981
ACTGTTGCTGACA	0.022	0.002	0.025	49	1878
GGTGGCATTGATG	0.718	0.043	0.681	719	337
CGCATCATTAGCG	0.022	0.009	0.03	51	1656
ACTGGCACAGGGA	0.021	0.008	0.009	9	946
GGTGTTGCAGAGA	0.062	0.008	0.07	43	569
AGCATCGTTGATG	0.023	0.005	0.02	34	1664
AGCATTGTTTAGG	0.02	0.001	0.019	25	1285
GCTGTCGTAGGGG	0.064	0.022	0.08	30	347
CCCAGTGCTGGGG	0.024	0.001	0.023	19	812
AGCGTCACTTGGG	0.015	0.001	0.013	11	844
CGTGGTGCATGGA	0.025	0.005	0.028	15	526
CCCGGTATAGGTG	0.027	0.001	0.028	35	1223
AGCGTTGCTTGGA	0.025	0.006	0.027	22	808
ACTGTTGTTTGCG	0.03	0.005	0.034	30	865
GGCATTATTTAGA	0.059	0	0.059	104	1654
GCTGTCGCTGAGA	0.051	0.002	0.051	28	522
CGCGGTGTTGGTG	0.026	0.01	0.037	19	491
GGCGTCATATATA	0.073	0.01	0.067	118	1652
CCCATCGCTGGTA	0.025	0.004	0.02	40	1941
AGTGGCGCATGTA	0.025	0.006	0.033	26	762
GCTGGTACTAGGA	0.079	0.014	0.063	44	652
ACTGTTACTAAGA	0.025	0.002	0.022	52	2366
CGTGTCACTGGTG	0.016	0.005	0.009	5	549
GCCAGTACAGATG	0.05	0.004	0.045	75	1607
ACTGGCGCATGTG	0.027	0.01	0.024	17	703
CGTATCGCTTGCG	0.027	0.002	0.025	20	791
AGTGTTACTTGGG	0.015	0.008	0.009	5	530
CGTGTCGCATGGA	0.024	0.006	0.026	17	634
GGTATCATATAGA	0.144	0.025	0.143	200	1194
CCCGTCACAAACG	0.027	0.003	0.025	79	3111
GGCAGCGTAAGCG	0.059	0.01	0.054	57	1005
AGTGGCGCTAATA	0.026	0.004	0.02	20	993

GCTATTGTAGGTA	0.052	0.011	0.047	59	1196
CGCGGCGTAGGCA	0.03	0.013	0.011	12	1035
GGCGGTATAGGTG	0.126	0.024	0.143	106	636
GGTGTCATATGGA	0.09	0.012	0.094	63	609
CGTGGTGCTAAGA	0.016	0.005	0.01	8	766
ACTGTTACTAAGG	0.026	0.008	0.014	22	1496
GCTAGCGTAAGTA	0.065	0.008	0.057	71	1166
GCTATTACTAGCG	0.059	0.003	0.056	67	1125
ACTATCGCATGGG	0.029	0.004	0.025	28	1078
ACTGGCGTTTAGG	0.031	0.006	0.038	15	384
ACCATCGTTTAGA	0.022	0.003	0.022	46	2074
CCTAGTATAGATG	0.022	0.002	0.02	38	1895
AGCAGCACTGAGG	0.016	0.001	0.018	21	1176
CCTGGTATTGACA	0.024	0.003	0.026	48	1830
GGCGTTATAGGTG	0.061	0.011	0.052	41	747
GCCGGTGTTTGGA	0.166	0.024	0.138	91	570
GGTGGCATATGTG	0.721	0.052	0.695	639	280
CCTAGCGTTGGCG	0.018	0.003	0.017	17	989
AGTGGCATTTAGA	0.021	0.004	0.025	15	579
CCTGTTGCATAGA	0.021	0.004	0.015	23	1476
CCCGGCACTAAGA	0.025	0.002	0.023	52	2245
AGCGTTGCAGGTG	0.027	0.004	0.032	29	866
GCCGGCGCATGTA	0.486	0.037	0.463	551	638
CGCGTCACAGATA	0.023	0.002	0.021	45	2070
CCTATCGTATAGA	0.026	0.003	0.023	47	2023
GGCATTGCAGGCA	0.052	0.003	0.048	56	1108
GGCAGCGTAAGTG	0.059	0.015	0.043	43	968
CGCAGCGTAGGGA	0.043	0.002	0.041	35	823
AGTAGCACTTGGA	0.026	0.009	0.013	10	775
AGTATTACAGAGG	0.028	0.005	0.02	26	1281
CCTGGCACTAAGG	0.022	0.008	0.013	12	933
CGTATCACTTGCG	0.027	0.007	0.018	16	856
GGTAGTATTGACG	0.075	0.009	0.07	58	770
AGCAGTATTAAGG	0.024	0.003	0.023	46	1921
CGCGGCGTTGACG	0.029	0.005	0.023	21	883
CGTAGTATTAGCA	0.03	0.01	0.025	31	1204
AGTAGCGCATATA	0.026	0.004	0.024	33	1324
CCCGTCATAGGCG	0.035	0.006	0.04	64	1546
CCCAGCACTGGGG	0.037	0.008	0.046	48	990
GGTATTATAGGGA	0.079	0.006	0.072	71	918

AGTGGTACTGAGG	0.032	0.012	0.024	14	576
CGTGTCGCTTGCG	0.021	0.013	0.015	9	589
GGCGTTATATGGG	0.059	0.005	0.065	45	643
GCCAGCACATGGG	0.062	0.01	0.052	40	730
CGCGTTGTTAAGG	0.018	0.002	0.015	15	960
AGCATTACTTATG	0.026	0.003	0.024	56	2292
CCTATCGTATGGG	0.031	0.007	0.021	22	1038
GCCAGCGCAGGGA	0.045	0.006	0.04	28	675
GGTATCACATGGA	0.087	0.012	0.076	58	704
GGTGGTGCTGACG	0.108	0.046	0.067	31	430
GGTAGCGTAGGCA	0.075	0.016	0.054	37	647
AGTGGTATTAGTA	0.022	0.001	0.024	26	1080
GCTGGTACTGACA	0.085	0.009	0.082	98	1104
CGTATCATTAGCG	0.025	0.006	0.022	29	1288
GCCAGTGCAGGCA	0.044	0.01	0.033	43	1263
ACTGGTACTAAGG	0.031	0.004	0.028	38	1326
CCTAGTGCAGAGG	0.022	0.004	0.018	19	1035
AGCGTTGCTGGTG	0.021	0.008	0.02	16	784
CGTAGCATAGGTA	0.027	0.007	0.024	27	1106
GCCGTTATAGGTG	0.057	0.01	0.043	42	927
CCTATTACAGAGG	0.027	0.002	0.029	49	1640
GCTATCGCTAAGG	0.067	0.013	0.05	40	753
GGTGTTGCTAAGA	0.067	0.006	0.07	51	673
CGCGTTGTAGGGG	0.039	0.016	0.018	10	551
GGTGTTACTAGTG	0.082	0.013	0.064	34	494
GCTAGTACAGGTG	0.064	0.009	0.061	49	756
CGCGTCGCTAGTG	0.019	0.005	0.012	11	877
GGCGGTACTAATG	0.085	0.019	0.064	68	1000
CCTGGCATAGGGG	0.047	0.005	0.046	33	677
GGTGTCACATAGA	0.075	0.011	0.066	57	808
GCCATTGTAGAGG	0.059	0.015	0.039	42	1041
GGTGTTGTAAAGG	0.095	0.006	0.09	52	524
CCTATTGCTAGTG	0.027	0.002	0.03	42	1378
CCTGGTACATGCG	0.026	0.005	0.029	29	980
ACCGTTGTAGAGA	0.028	0.006	0.022	39	1766
ACCAGTGTTGGGG	0.026	0.003	0.025	22	867
AGCATTATTGAGG	0.017	0.005	0.011	19	1751
CCCAGTGTAGAGG	0.024	0.005	0.029	39	1326
GCTGTCGCTAGCA	0.06	0.009	0.056	42	705
GGTGTTGTTAGCA	0.105	0.03	0.073	47	594

ACTGGTGTTAGGA	0.031	0.009	0.039	37	900
AGCAGCGCAGGCG	0.022	0.006	0.015	17	1145
GGCGGCGTAAGTG	0.449	0.037	0.416	394	552
GGTAGCATAAATG	0.071	0.015	0.05	59	1127
GCCGTTGCAGGCG	0.047	0.004	0.05	54	1035
GCCGTTGCATATG	0.049	0.007	0.04	38	907
AGTGTCACTAATG	0.025	0.004	0.02	23	1133
AGCATCGTTAGGA	0.023	0.002	0.022	33	1478
GGTGGTGTATGGG	0.181	0.027	0.18	58	265
GCCGGCGTTAATG	0.934	0.021	0.922	1621	138
AGTGGTGTTAACA	0.023	0.001	0.024	33	1315
AGCAGTGCTGAGG	0.021	0.006	0.025	25	973
CCCGGTGTTTGCG	0.028	0.008	0.029	29	962
AGCGTTATAAGCG	0.021	0.002	0.022	41	1810
GCTATCACTTGTG	0.057	0.013	0.065	62	899
GGTAGCGTTTAGA	0.074	0.008	0.081	45	511
GCCGTCATTTGTG	0.061	0.007	0.051	50	925
GCTATCGCTGGGG	0.048	0.009	0.048	23	453
GCCGTTGCTTATA	0.049	0.008	0.041	47	1099
GGTAGTGCAAGTG	0.055	0.013	0.052	31	569
ACTGGTGCTAGTG	0.032	0.001	0.031	26	819
AGTAGCGCTGGGA	0.024	0.003	0.027	14	504
ACTGGCGCAGAGG	0.038	0.002	0.036	20	540
AGTGTCGTTAACA	0.024	0.008	0.017	28	1651
GCCGGCACTAGGA	0.122	0.014	0.132	141	931
CGCAGCGTAGATG	0.029	0.002	0.027	33	1179
CGTGGCGCTTACG	0.027	0.006	0.019	11	578
AGCGGTGCTAGTA	0.023	0	0.023	28	1194
AGCGGCATTAAGA	0.018	0.004	0.013	19	1451
GCCGTCACAGGGG	0.067	0.007	0.07	43	574
ACCGGCGCATGCG	0.024	0.005	0.023	26	1128
CCCAGTACTAAGG	0.027	0.003	0.025	54	2080
GGCATCGTTAATG	0.108	0.007	0.101	143	1279
GCCGGCATTAGGA	0.384	0.033	0.398	467	705
GCTGGTGCAGGGA	0.138	0.029	0.146	70	411
CGCGGCGTAAAGA	0.03	0.003	0.027	37	1340
CCTATCGCTAACA	0.025	0.003	0.022	67	3012
CGCGGCATTAAGG	0.02	0.006	0.012	10	854
AGCAGTGCATACG	0.017	0.002	0.017	31	1846
GGTAGCGTTAGTG	0.07	0.015	0.059	26	411

CGTATTACTGATG	0.034	0.005	0.031	40	1257
GCTATTATAAGTA	0.057	0.008	0.047	117	2376
CCTGTTACATACG	0.026	0.002	0.024	41	1686
GGCGGTACAGACG	0.076	0.021	0.08	78	894
CGTAGTACAGGCG	0.023	0.009	0.016	12	736
GGCATCACAGGGA	0.069	0.012	0.053	61	1100
GGTGGTGTTGGTA	0.103	0.036	0.076	30	363
GGTGGTGTTAGGG	0.206	0.03	0.216	70	254
ACTGGTGCAAAGG	0.023	0.003	0.021	23	1075
AGTGTCATTTGTG	0.023	0.009	0.034	26	732
AGCATCATTGATG	0.023	0.005	0.022	52	2308
CCCAGCGCATAGG	0.019	0.002	0.017	24	1360
CCTGTTGCAAAGA	0.026	0.002	0.024	48	1990
CGTGGTGTTTAGA	0.022	0.008	0.015	8	539
ACTAGCATATGCG	0.027	0.002	0.028	48	1686
AGCGTTACAAGGA	0.022	0.005	0.02	35	1707
GGCGTCGCAGAGG	0.095	0.014	0.076	47	572
GGCATTACTGGGA	0.058	0.003	0.054	56	973
ACTAGCGTAGAGG	0.027	0.006	0.033	32	924
GGCGTTATAGATG	0.062	0.01	0.047	56	1131
CGTATCGCAAGCG	0.026	0.008	0.015	16	1087
GGCAGTGCTTGTG	0.045	0.005	0.043	28	619
AGCATCGCAGAGG	0.022	0.001	0.023	29	1256
CGTAGTATAGAGG	0.031	0.004	0.028	23	805
ACCGTCGCATATG	0.028	0.004	0.024	43	1755
GGTGTTGTTGGGA	0.074	0.024	0.071	25	328
CCTAGCGCATGTA	0.024	0.003	0.023	40	1731
ACCAGTGCTTAGA	0.022	0.004	0.026	56	2131
GGCGTTGCAAGCA	0.056	0.005	0.05	61	1156
CCCGGCATATACG	0.025	0.006	0.018	41	2224
CGCGTCGCATGCA	0.026	0.001	0.025	35	1385
GCCATCATTGGCA	0.056	0.004	0.057	117	1943
ACTATTGTAGGCG	0.026	0.006	0.022	41	1861
AGCGTCACATAGA	0.02	0.002	0.024	46	1906
AGTGGCACAGGTA	0.02	0.001	0.021	17	782
GCCGGTGCTTAGG	0.108	0.015	0.127	65	445
GGTATTACAGACA	0.086	0.011	0.071	123	1604
GGTATCGCTTAGA	0.09	0.008	0.1	73	658
CGTATTGCAAAGG	0.026	0.004	0.031	37	1143
GCTGTTATTGATA	0.068	0.007	0.059	90	1433

AGTAGTGTAAGTG	0.023	0.007	0.014	15	1050
ACTGGTACTGGGA	0.024	0.012	0.009	9	968
CCTGTTACTTGTG	0.036	0.007	0.039	36	892
ACTAGTACTGGCG	0.024	0.002	0.021	29	1381
ACTGTCGTATGTG	0.033	0.004	0.029	24	812
GCTGTCGCAGGGA	0.036	0.004	0.036	18	477
GCCGTTATTGGCG	0.057	0.003	0.053	55	976
CCTGGCGCTAGGA	0.027	0.004	0.027	22	804
AGCGGCGTTAGGG	0.019	0.006	0.012	5	424
CCCGGCGCTTGGA	0.031	0.002	0.033	34	999
GGCGGCGCAAAGA	0.097	0.017	0.09	94	946
ACTAGCGCTGAGG	0.028	0.003	0.025	23	908
CGCGGTGCTGGCA	0.023	0.003	0.028	27	944
GGTATTACAAGTA	0.083	0.019	0.078	118	1397
CGTATCATTAGGG	0.03	0.006	0.038	34	853
GCCATCATATGGA	0.058	0.006	0.051	91	1682
CGTGGTGCTTGGG	0.016	0.005	0.013	4	311
CCTGGCATATGGA	0.02	0.003	0.02	22	1097
CCCGGTATATGGA	0.025	0.005	0.028	46	1601
GGTAGTACTAGTG	0.073	0.018	0.047	28	566
CGTATTGTAAGGA	0.026	0.003	0.027	32	1155
AGTAGTGTTTACG	0.028	0.004	0.032	32	970
GCCGTTGTTTGCA	0.048	0.007	0.04	42	998
CGTATTATTAGGA	0.023	0.007	0.018	26	1419
GCTAGCGCTAAGG	0.067	0.006	0.076	45	544
ACTAGTGTAAAGA	0.023	0.001	0.023	65	2820
GCTATTGTTAACG	0.062	0.001	0.062	101	1539
GGTGTTACTAAGG	0.091	0.015	0.077	49	589
GGTAGCACTTGCG	0.079	0.011	0.067	35	485
AGCGTCGCTTAGG	0.027	0.007	0.021	17	811
CGTAGTGTTAGGA	0.022	0.011	0.02	12	597
GGCATTACAGACG	0.06	0.004	0.063	104	1538
ACTAGTACTTGCA	0.023	0.002	0.022	57	2583
AGTAGTGCATACA	0.024	0.005	0.02	33	1604
ACTGTTGCAGGGG	0.029	0.006	0.035	24	653
CGTGGCGCATGTA	0.024	0.012	0.041	26	606
GCTAGTACTAGGG	0.052	0.004	0.05	32	607
GCTAGCGCAAATG	0.064	0.008	0.055	53	915
CCCGGCGTTGGCG	0.021	0.007	0.022	21	947
GCTAGTACTGGTG	0.063	0.005	0.06	42	653

AGTGGCATTTGGG	0.021	0.016	0.039	6	149
GCCGTCGCTAGCA	0.052	0.015	0.034	37	1049
CGTAGTGCTGGTG	0.024	0.013	0.029	13	441
GCTAGTGCTAAGG	0.056	0.005	0.058	44	713
CGTAGTGTTTGCA	0.023	0.006	0.017	12	696
AGCGGTACAGAGA	0.024	0.003	0.025	38	1497
AGTGGCATAGAGG	0.02	0.007	0.03	8	257
GCTGGTGCTTATG	0.286	0.044	0.304	182	417
ACTGTCACAGGGA	0.028	0.006	0.021	23	1078
CGTGGTGTTGGCA	0.027	0.008	0.027	15	538
AGTATCACAGGTG	0.026	0.007	0.017	15	882
CGCATTGCTGGCA	0.022	0.001	0.022	30	1320
GGTATCATATATG	0.121	0.015	0.1	106	957
CGCGGCGCATACG	0.022	0.003	0.021	26	1208
GGTAGCACAGGTG	0.074	0.015	0.073	35	445
GCTGTTATATATA	0.061	0.005	0.059	117	1858
ACCATCGCATGTG	0.026	0.004	0.02	37	1822
GGTATCGTTTGGG	0.119	0.018	0.1	40	360
CGCATTATAAGGG	0.027	0.01	0.013	18	1405
GCTGGTGCTAAGG	0.294	0.051	0.321	177	375
CCCGTCGCAAATA	0.021	0.002	0.019	62	3283
GCTGTCACTAGTA	0.063	0.003	0.06	65	1026
AGCAGTGCTGACG	0.022	0.003	0.018	27	1515
GGCGGTATTGATG	0.133	0.041	0.1	91	823
GCCAGTGCATGGA	0.039	0.004	0.034	31	882
CCCGTCGTTGATA	0.02	0.001	0.02	43	2124
GGTGGCACTAATG	0.394	0.047	0.358	332	596
CGTATTATATAGA	0.028	0.004	0.022	46	2029
GCTAGCGTTGAGA	0.057	0.007	0.054	43	755
CGCATTGTTTGGA	0.026	0.004	0.032	37	1137
CGCGGCGTTAGGG	0.018	0.002	0.02	9	451
ACTGGCATATGCA	0.027	0.004	0.032	54	1654
GGTAGCGCAAACA	0.062	0.015	0.043	54	1194
CGTGGTGCAAGCG	0.028	0.012	0.044	28	608
GGCAGCGCTTGCA	0.056	0.005	0.061	59	903
GCTGTCACAGGTA	0.065	0.006	0.057	57	936
CGTAGTATATAGA	0.025	0.003	0.022	29	1293
GGCGTTGCTTACG	0.059	0.012	0.052	42	764
GGTGTTATAGACA	0.083	0.01	0.073	95	1208
GGCGGCGTTGACG	0.428	0.039	0.401	378	565

CGTGTCGCATACG	0.031	0.002	0.028	26	887
AGTGGCATATGTA	0.026	0.009	0.014	10	725
GCTAGTATTTGCG	0.069	0.006	0.061	56	868
GCTGGCGTAGATA	0.874	0.035	0.873	1550	226
CGTGGTACAGGGA	0.027	0.015	0.046	27	559
GGTGTTGTTGGCA	0.088	0.005	0.085	40	433
GGCGGTGCTGGGG	0.067	0.008	0.059	22	349
AGTATCACTAAGG	0.029	0.003	0.027	32	1157
GGCGGCGCATGGA	0.098	0.015	0.092	55	546
GCCAGCGCTAGCG	0.045	0.006	0.052	42	762
CGCATCACAGGTA	0.024	0	0.024	46	1868
GGTATCATTGACA	0.126	0.019	0.131	192	1273
ACTAGCACATGGG	0.024	0.004	0.018	18	973
CCTGGCACATGCA	0.026	0.005	0.033	52	1501
ACCGTCGTAGGTG	0.029	0.003	0.025	23	911
AGTGTCGTTAGTG	0.034	0.003	0.029	20	659
CGCGGCGTTTGCG	0.016	0.007	0.014	10	698
CGTAGTGCTGGGA	0.021	0.001	0.02	10	497
GGCATTGCAAACG	0.074	0.005	0.067	114	1599
CGCGTTGTAAAGG	0.028	0.008	0.02	23	1152
GCCAGCGTAGGCG	0.055	0.005	0.051	48	892
AGTGGTGCTTACG	0.02	0.003	0.016	11	662
CGTGTTGTAAGCG	0.027	0.008	0.021	16	752
ACCAGTGCAGGTG	0.022	0.003	0.02	29	1427
GCTATCACAAGTG	0.063	0.006	0.064	78	1149
GCTGGTACAAATG	0.115	0.011	0.1	125	1123
CGCAGTACTGGTG	0.023	0.004	0.018	17	902
GCTGGTGCTAATG	0.293	0.022	0.266	200	551
GCCAGCACTTACA	0.056	0.009	0.068	159	2189
CGTATTGTAAGTA	0.022	0.008	0.015	24	1560
GGTAGTATTAGTA	0.069	0.014	0.058	62	1006
CGTATCACAAGGA	0.026	0.005	0.02	25	1202
GGCATTATTGAGG	0.069	0.015	0.048	51	1004
ACCGGCGCTAAGG	0.019	0.001	0.019	20	1032
CGTGTCACTGAGG	0.022	0.007	0.021	13	595
AGTGTCGCAGACG	0.028	0.006	0.031	27	841
GGTAGCGTTGAGG	0.078	0.014	0.068	26	358
CGTATTGCTGGGG	0.029	0.008	0.018	9	501
GGCAGCGCTAGTA	0.054	0.007	0.053	55	976
AGTAGCGTTAGGG	0.029	0.002	0.032	18	539

AGCGTTGTTGGTA	0.026	0.004	0.031	33	1045
AGCGGCGCAGACG	0.022	0.005	0.018	20	1091
AGTGGTACATGCA	0.025	0.002	0.023	26	1116
GGCGTCACTTGGA	0.076	0.01	0.065	49	707
GGCGTCATAGGCG	0.054	0.013	0.04	38	902
GCCAGTATTTGGA	0.047	0.005	0.053	74	1327
GCCGTTGCTGAGA	0.059	0.008	0.047	42	845
CCCAGCGTTAGTG	0.026	0.003	0.027	41	1454
GGTGGCATTGAGA	0.465	0.07	0.423	352	480
CGTGGCATATACA	0.024	0.008	0.012	16	1315
GCTAGCACTGGTA	0.052	0.007	0.045	41	874
GGTATCGCAGATA	0.108	0.013	0.111	112	899
CCCAGCGTAGATA	0.025	0.001	0.025	68	2608
ACCGGTGCTGAGG	0.024	0.003	0.028	27	954
GCCGGTGTAAGGA	0.174	0.034	0.151	151	851
AGCGGTGTAGGGA	0.018	0.004	0.02	21	1048
CGCATCACAGAGA	0.023	0.001	0.023	50	2153
CGTAGCACTTACA	0.026	0.002	0.025	37	1467
GGTGTCGCATGTA	0.093	0.006	0.09	54	548
GGTATCGCATGTA	0.088	0.021	0.066	58	823
CCCGGCACATAGG	0.024	0.006	0.025	39	1525
AGTAGCATAAGCG	0.023	0.001	0.025	33	1298
GGTGGCATAGACG	0.725	0.046	0.732	920	336
GCCGGTATATGCG	0.162	0.021	0.158	185	983
GGTAGCACAGGTA	0.061	0.006	0.054	38	670
CGTAGTACTTATG	0.02	0.003	0.018	15	800
CGCGTTGCATATG	0.025	0.002	0.024	26	1078
GCTGGTGTATGCG	0.742	0.061	0.743	635	220
CCCGGTACAAGTA	0.024	0.003	0.021	53	2417
GGTGGCGCATATG	0.329	0.033	0.315	162	352
ACCGTCGTTAGGA	0.023	0.005	0.02	23	1148
AGTGGTGCTGGCG	0.024	0.008	0.022	10	446
CGCAGTGTTGGTG	0.02	0.002	0.022	16	714
GGCGTCGTTGAGG	0.065	0.004	0.065	37	528
ACCGGCACTTACG	0.023	0.003	0.024	50	2016
GCCATCGCTTAGA	0.055	0.002	0.054	59	1041
GCTATCGCTTATG	0.052	0.002	0.052	48	868
GCCAGCATAGGGA	0.085	0.01	0.073	84	1073
AGCAGCGTTTGCA	0.021	0.003	0.023	36	1503
CCCAGTATATGGG	0.029	0.001	0.027	40	1423

GCTGTTGCAGATA	0.056	0.005	0.053	55	987
GGTGTCGCTGATA	0.085	0.004	0.089	64	654
CGCGGCACAGGTG	0.032	0.007	0.024	19	786
ACCATCACTTAGG	0.025	0.003	0.022	48	2159
GGTAGTATTGGGG	0.063	0.004	0.063	27	404
CGTGGTATTTACG	0.022	0.003	0.02	14	689
ACTGTCGCAGATA	0.022	0.002	0.021	41	1940
GGTGGTGTAAGCA	0.108	0.022	0.094	68	654
CGCGGCGTAGGGA	0.037	0.008	0.025	18	690
CCCGTTATATGGA	0.022	0.003	0.02	39	1915
GGCGGTGCTTACA	0.06	0.012	0.075	74	909
GCTATCGCAAGTG	0.051	0.019	0.037	32	837
AGCAGCGTAAATG	0.022	0.002	0.021	48	2187
ACCGGTATTTGGG	0.023	0.009	0.011	10	933
GGCGTTGCAGGTG	0.056	0.008	0.055	33	562
GGTAGTGCTAAGG	0.048	0.01	0.038	20	504
CCCGTTACTTGGG	0.029	0.007	0.024	25	1004
AGCAGTGTTTGCG	0.02	0.009	0.024	28	1142
CCTGTTGCTAGTA	0.026	0.002	0.028	42	1440
CCTGTTGCTAGGG	0.023	0.009	0.014	10	691
AGTGGCACATACA	0.024	0.002	0.022	33	1474
AGTAGCACAAGGG	0.026	0.004	0.023	18	750
AGTGGTGTAAAGG	0.032	0.011	0.041	30	697
AGCGGTATTTGCG	0.022	0.002	0.025	24	949
GGCGTTGCTGGCA	0.058	0.012	0.041	32	742
GGTGGCGCTAACG	0.303	0.059	0.282	165	420
GGCAGTGCTAGCA	0.055	0.005	0.051	51	946
AGCGGTACTAGGA	0.025	0.006	0.018	21	1169
AGCGTTGTAAGTG	0.027	0.003	0.023	29	1254
AGTGTCATATGTG	0.023	0.006	0.021	20	927
AGTATCACTGATG	0.029	0.001	0.03	38	1237
ACTATTATTAGCG	0.028	0.002	0.029	63	2113
CGTGTTACAAGGA	0.027	0.004	0.023	25	1061
GCTAGCGCATACG	0.062	0.011	0.048	40	794
CGCGTCACAAGCG	0.03	0.004	0.026	39	1459
GGCGGCGTTTAGA	0.198	0.066	0.159	102	539
AGCGGCGTATGGG	0.026	0.002	0.028	15	519
AGTGTTATTTAGA	0.023	0.001	0.025	33	1285
ACTATCGCTGAGA	0.029	0.006	0.031	60	1871
GCCAGTGTTAGGG	0.048	0.007	0.053	40	715

**CGTGGTGTAAAGG**	0.024	0.012	0.04	25	597
**CCTAGTGCAAAGG**	0.027	0.002	0.025	38	1496
**GCCATTGTATATA**	0.045	0.005	0.051	119	2198
**CCCAGCGCTGGGG**	0.035	0.002	0.033	25	735
**GCCGTCGTATACG**	0.054	0.01	0.045	48	1026
**CCTGGCGCATGGG**	0.027	0.013	0.012	7	567
**GCCGTTACAAAGG**	0.068	0.005	0.062	84	1281
**GCTGGCGCAGACG**	0.814	0.04	0.779	925	262
**GCCGGCGCATGCG**	0.75	0.028	0.735	869	314
**GGCGTCACAGGCA**	0.062	0.013	0.046	59	1222
**GGTGTTATTTATA**	0.081	0.001	0.079	92	1067
**GCCAGTGCAGATG**	0.052	0.008	0.06	63	990
**CCTGGCGCTTGTG**	0.017	0.003	0.013	8	614
**CGTAGTGCAAAGA**	0.022	0.004	0.026	29	1098
**GCCGTCATAAAGA**	0.059	0.002	0.057	131	2170
**CCCGTCGTTGGGG**	0.026	0.005	0.028	19	657
**CCTGTTGTAGGGA**	0.022	0.008	0.023	22	928
**AGTGGCGCAAACA**	0.026	0.003	0.023	31	1339
**CGCGTTATTTATA**	0.025	0.007	0.034	68	1944
**AGTGGCGCTAGTA**	0.025	0.01	0.013	9	697
**CCCGTCGCAGAGG**	0.026	0.006	0.018	22	1171
**GGCAGTGTAGGTA**	0.053	0.007	0.048	44	876
**AGTGTCGTTGACG**	0.028	0.008	0.024	21	848
**CCTGTCGTTGATG**	0.021	0.007	0.015	15	995
**GGCGTTATTAGCA**	0.056	0.003	0.058	77	1256
**CCTGTCGCAGACG**	0.022	0.003	0.025	33	1288
**GGCAGTACATATG**	0.06	0.005	0.055	74	1262
**GGTGTCACATAGG**	0.082	0.01	0.068	35	483
**GGCGGTGTTAATG**	0.082	0.021	0.085	72	775
**GCTGTTACTTGGG**	0.072	0.003	0.074	33	412
**AGTATCACAAACA**	0.022	0.004	0.019	52	2747
**GGTGGCGTTGGCA**	0.396	0.075	0.377	191	315
**AGTGGTACTTGGA**	0.028	0.004	0.022	16	703
**CCTATCGTTGGCG**	0.026	0.008	0.018	26	1425
**CGTATTATTTGCG**	0.026	0.003	0.023	24	1027
**GCTAGTGTTTAGA**	0.058	0.011	0.052	52	956
**CGTGTCACTAAGG**	0.022	0.002	0.023	18	771
**ACTATCGCTAAGG**	0.027	0.003	0.022	32	1433
**GCCAGTGCAGGTA**	0.047	0.006	0.04	42	996
**CGTGTTGTAGACA**	0.021	0.005	0.016	20	1265

GGCAGTGTAGGTG	0.063	0.011	0.056	39	661
AGTAGTACTAGGA	0.025	0.006	0.019	22	1120
AGCAGCATTGGTG	0.021	0.003	0.024	30	1244
GGTATTGTAAGTG	0.099	0.013	0.09	79	794
GGCAGTGCATAGG	0.05	0.004	0.055	40	687
CGCGGCATTAGGA	0.021	0.002	0.018	17	922
GCTGGCACAGGCA	0.212	0.017	0.214	227	836
GGCGGCGCTGACA	0.102	0.015	0.093	99	968
ACCAGCGTTTGCG	0.022	0.002	0.024	30	1245
CGCAGTGTTGACG	0.03	0.008	0.023	29	1226
AGTGTCGTTGACA	0.022	0.001	0.023	30	1262
GCCAGCACTTGGG	0.05	0.002	0.05	40	756
GCTGGCATTAACG	0.868	0.029	0.849	1684	300
CCCGTCATTGATG	0.024	0.003	0.02	34	1689
GCCAGCGTATAGA	0.059	0.006	0.05	69	1299
GCTATCGTAAGCA	0.061	0.006	0.053	86	1548
AGCGGCGTTTATG	0.023	0.003	0.023	16	678
AGTGGTATTTGTG	0.016	0.008	0.011	6	564
CCCATTGCAGATA	0.023	0.002	0.024	77	3168
GGTATCATAAACG	0.128	0.017	0.106	149	1262
CGTGGTATAGGCG	0.026	0.005	0.033	18	528
CGCATCGCTGAGA	0.019	0.003	0.016	20	1259
CGTGGCGCTGATA	0.033	0.004	0.028	20	689
GCTGTCGTTGGGG	0.077	0.009	0.076	24	291
AGCGGTGTAGGGG	0.018	0.009	0.007	6	847
CGCGGTGCTTATG	0.025	0.008	0.024	19	772
CCCGTTGCATACG	0.022	0.002	0.024	48	1933
CCCAGTGTTTGGA	0.026	0.003	0.023	30	1264
AGTGGTATAGGCA	0.025	0.006	0.029	28	938
GGTATTACTTGCG	0.105	0.009	0.116	84	643
GCTGGTATAGGTA	0.164	0.003	0.16	156	819
AGCGTCGTAGGTA	0.019	0.002	0.016	20	1254
CGTAGTGCAGATA	0.025	0.003	0.026	27	1021
AGTGGTATTAACG	0.023	0.003	0.022	22	1000
ACTAGCGCTTGGG	0.019	0.002	0.02	15	750
GGCGTTACTAAGA	0.067	0.013	0.053	71	1272
GCTGTCGTTTGCG	0.065	0.01	0.069	31	417
CGTGTCGCTGGTG	0.023	0.005	0.016	8	490
GCTGGCGCATAGG	0.817	0.023	0.801	661	164
CGTATCGTATACA	0.025	0.006	0.026	53	1997

CCCGTCGTAGGGG	0.04	0.004	0.043	31	694
GGTGGCACATATG	0.391	0.083	0.333	259	519
CGTGTCACATGGG	0.022	0.009	0.02	13	644
GCCAGCGCAAAGA	0.056	0.003	0.052	75	1358
CGCGGTATTAGTG	0.02	0.006	0.026	23	875
CGTGTCACTGGTA	0.022	0.007	0.013	10	749
GGTGTTATTGACA	0.086	0.013	0.068	76	1035
ACTATTGTTTGCG	0.026	0.003	0.025	35	1384
ACTAGTGCTTGGA	0.023	0.001	0.023	33	1380
GGCAGCGTATAGA	0.061	0.014	0.045	52	1112
GGTGTCATTAAGA	0.088	0.027	0.062	61	923
AGCGGCGTAGGCA	0.025	0.002	0.024	25	1035
CGTGTCGCAGGTG	0.036	0.006	0.03	16	525
CCCAGTGTAAACG	0.026	0.008	0.017	47	2687
CGTATCGTATAGG	0.029	0.014	0.024	22	891
CCTAGTACTGGTG	0.02	0.001	0.021	21	988
CCTGTCGCTGATG	0.023	0.01	0.01	10	996
GCCGGCATTTACG	0.723	0.031	0.717	1214	480
CCCAGTGCTGGCA	0.023	0.002	0.02	35	1693
CGCGTTGCAGGTG	0.029	0.003	0.026	20	735
AGTGTTGCAGAGG	0.026	0.008	0.02	11	550
GCCGGCATATGTA	0.383	0.039	0.384	677	1085
CGCATCGCAAGGA	0.024	0.005	0.017	24	1372
GGCGGCACAGATA	0.134	0.026	0.115	164	1263
CCTATCGCATAGG	0.025	0.01	0.011	15	1307
AGCGGCACAAGCA	0.025	0.003	0.026	51	1904
CCTAGTATTGGTA	0.028	0.006	0.033	53	1541
GGTGGTGCAGGTA	0.062	0.017	0.04	20	479
ACTGTTGTATACG	0.033	0.003	0.033	53	1572
AGCATCACAGGGA	0.022	0.006	0.015	25	1692
GCTAGTACTTAGA	0.062	0.007	0.063	73	1094
CCCGGCGTTGGGA	0.019	0.004	0.019	18	934
CCCATCGTTAGGG	0.027	0.005	0.022	25	1110
AGCATCGCTGGGG	0.023	0.007	0.013	12	881
CCCGTTACAGACG	0.024	0.003	0.028	65	2289
CGTATCACAGAGA	0.023	0.006	0.019	24	1265
GGCATCGCTAATA	0.083	0.011	0.07	129	1727
GGCAGCATAGACG	0.056	0.008	0.054	80	1408
CGTGGTGCAAATG	0.027	0.004	0.024	18	738
CCCGTTGCTGGGA	0.031	0.005	0.024	24	968

GGCGTCACAGGGA	0.073	0.01	0.065	57	815
AGTATTATTAAGG	0.025	0.004	0.024	44	1799
AGTATTGCAAGCA	0.021	0.003	0.022	39	1710
CGCGGTGCATAGA	0.02	0.002	0.017	18	1024
GGCGTTATAAGGA	0.057	0.008	0.048	61	1204
GGCGTTACAGAGG	0.049	0.005	0.045	32	676
CGCGGTGCTAAGA	0.024	0.002	0.025	30	1168
GGTGGCGCAGGTG	0.286	0.058	0.292	108	262
CGCGGTGTAGGGG	0.033	0.009	0.031	14	434
ACTAGCGTTAGTG	0.033	0.007	0.033	29	862
GCTGGTATATGTG	0.452	0.03	0.459	391	461
CGTGGTGTTGGTA	0.027	0.009	0.039	19	469
CGCGTTGTTTGGA	0.026	0.005	0.032	23	695
GCTGGCGTATGGA	0.881	0.029	0.871	880	130
CCTGGCACTTGGA	0.026	0.007	0.03	28	916
AGTATTGTATACA	0.026	0.001	0.027	68	2435
CCTAGCGCATGGG	0.035	0.004	0.03	21	677
GCTGTTGTTAATA	0.056	0.007	0.046	65	1348
AGCGGCACTGGTG	0.023	0.002	0.024	16	639
GCCGTCATAAGGA	0.054	0.006	0.048	70	1399
GGCATCGCTTGTG	0.098	0.013	0.079	56	651
CGCGGTGTTTGTA	0.025	0.009	0.031	26	809
CCTATCGCAGATG	0.025	0.004	0.02	31	1551
CGTATTGTTGAGA	0.033	0.009	0.02	21	1050
GGTGGCATTGGTG	0.702	0.041	0.681	474	222
GCCGGCATTTACA	0.399	0.033	0.41	897	1291
CCCATCATAGGGG	0.039	0.005	0.039	54	1326
CGTGTTACAAGTA	0.027	0.003	0.022	33	1441
GGTAGCGTTGATG	0.081	0.01	0.087	45	474
GGCAGCGCTAGTG	0.068	0.02	0.057	42	700
GCTGTTGCTTGGG	0.053	0.021	0.033	13	376
GCTATCGTTAATG	0.062	0.002	0.063	80	1188
AGCGGCATTTGTA	0.021	0.005	0.028	31	1077
GGTATTGCAGGTA	0.057	0.006	0.058	42	685
AGTGTTGTATATA	0.021	0.003	0.021	34	1611
AGCAGCATTGACG	0.022	0	0.021	40	1855
CGTATTGCTTAGA	0.023	0.011	0.038	37	932
AGCGTCGTTAGCG	0.02	0.004	0.021	24	1120
GCTAGTGCTAACG	0.058	0.001	0.056	68	1139
GGTAGCGTATGGA	0.088	0.022	0.095	51	486

AGTAGCGTATACG	0.018	0.003	0.014	16	1094
CCTATCGCTTAGG	0.033	0.002	0.035	38	1060
GCCGTTGTAAAGG	0.051	0.003	0.048	54	1075
CGCGTCGTTAATG	0.027	0.006	0.019	24	1248
GCCATCGCAAGGA	0.051	0.007	0.041	50	1155
GCTGTTATTAGTA	0.06	0.002	0.06	77	1198
GCCAGTATAGGGA	0.058	0.003	0.061	62	951
AGTGGCATTTGTA	0.02	0.008	0.008	5	585
GGTATTGTTTAGA	0.104	0.021	0.098	85	780
CGCAGCGTTGGCG	0.032	0.006	0.026	23	869
CGTGGCATTAGCG	0.029	0.009	0.018	8	442
CGTATCACTGGGG	0.027	0.003	0.024	13	538
GCTGGTACTTATG	0.122	0.004	0.127	88	603
AGTGTCATAAGTG	0.03	0.004	0.033	37	1073
CGCGTCGTTAGCA	0.026	0.002	0.023	31	1293
GCCGTCGTATGGA	0.055	0.012	0.071	56	732
ACTGGTGCAGGGG	0.028	0.003	0.026	15	564
GCTAGTATATGCG	0.059	0.009	0.05	59	1129
GCTGTCATTAGCG	0.068	0.009	0.059	49	778
GCCAGTGTATGCG	0.04	0.002	0.038	40	1012
AGTAGCGCAGGGA	0.027	0.002	0.029	18	594
GGTAGCACTTGTG	0.07	0.014	0.054	27	476
CCCATCGCTAGTG	0.025	0.001	0.024	39	1592
CGCGGTGTATGGA	0.023	0.004	0.029	24	817
GCTGTCGCTAGTG	0.048	0.01	0.033	15	433
CGTGTTGCTAGTA	0.022	0.001	0.023	18	759
GCTGTTGCTGGCG	0.046	0.01	0.049	33	637
AGTGGCACAAGGA	0.018	0.006	0.014	12	862
GGCGGCGTTTGTG	0.441	0.027	0.418	250	348
GGTGTTGTTTGGA	0.083	0.026	0.096	39	366
GCTGGTGCATATG	0.296	0.022	0.308	240	540
GCCGTCACTAGTG	0.058	0.011	0.053	55	987
CGCGTTACTTAGA	0.023	0.003	0.02	28	1393
GCCGGCGCTGGCG	0.722	0.019	0.711	609	248
GGTATCGTTGAGA	0.162	0.005	0.159	124	656
CGTGTTATATGGG	0.027	0.001	0.028	20	682
ACCGGCGTAGGGA	0.027	0.004	0.021	18	836
CGCATTGCTGATG	0.025	0.006	0.022	25	1131
AGCAGTACTAGTG	0.021	0.006	0.014	23	1578
CGCATCGTAGGGG	0.037	0.003	0.038	29	732

GCCGTCATAGGGA	0.079	0.003	0.075	70	867
GGCGTTGTATGGG	0.078	0.018	0.058	32	523
ACTGGCACTGACA	0.028	0.004	0.026	54	2051
CGTGTTGTAAGTA	0.026	0.003	0.029	32	1062
GGTATCGCATGTG	0.13	0.028	0.117	65	491
CGCAGTGCAGGCG	0.024	0.002	0.023	24	1017
GGCGTCGTTTGGG	0.098	0.018	0.091	36	361
GCCGGTGTTGGTG	0.414	0.073	0.412	235	336
CCCGTCGTTGGTA	0.022	0.001	0.023	31	1332
CGCGGCACAGATA	0.025	0.002	0.027	51	1839
CGTAGCGCATACA	0.026	0.007	0.033	45	1335
GCTAGTATATACG	0.063	0.006	0.056	93	1576
AGCGGTATTGGTA	0.025	0.006	0.031	39	1230
GCCGTCATAAGCG	0.055	0	0.055	86	1487
CCTGTCGTTTAGG	0.032	0.003	0.035	24	670
GCTAGTATTTAGA	0.066	0.009	0.068	92	1255
GGCATCGCTTAGG	0.102	0.015	0.093	72	699
CGTATTGCTAGGG	0.025	0.008	0.019	10	525
GGCGTTACATATA	0.062	0.009	0.051	93	1729
CCCGGTGCTAAGG	0.024	0.004	0.025	24	927
CGCAGTGCTGATG	0.024	0.003	0.024	25	1007
AGTATTACATGTA	0.019	0.002	0.017	34	2010
GCTAGTACTAAGG	0.059	0.009	0.049	50	973
CGCGGTATAGGGG	0.043	0.007	0.049	29	558
GCTAGTGCTAGGA	0.048	0.006	0.05	39	737
CCTAGTGTTTGCG	0.019	0.004	0.019	21	1102
ACCGTCACAGGCA	0.019	0.003	0.02	62	3066
CCCGGTACTTGGG	0.026	0.01	0.012	11	878
GGTATTGCTTACA	0.085	0.002	0.082	82	915
AGTGTCGCATGCG	0.025	0.008	0.018	13	716
ACTATCATAGGGG	0.042	0.013	0.025	26	1029
GCTATTATTGATA	0.065	0.001	0.064	148	2152
ACTGGCGTATGGG	0.031	0.011	0.021	8	380
GGCGTCACAGAGA	0.061	0.014	0.044	51	1100
GGTGTTACAAGGG	0.079	0.009	0.07	37	493
CCCGGCGTTAAGG	0.024	0.001	0.024	25	1008
GGTGGTGCTAATG	0.081	0.006	0.077	33	396
CGCGTCATAGGTG	0.022	0.001	0.021	20	944
CGTGGTGCAGATA	0.028	0.006	0.034	32	903
CGCGGTATATGTG	0.016	0.004	0.015	15	1017

GCCATCGTTAGTA	0.046	0.01	0.044	66	1449
AGTGGCATTAACG	0.02	0.004	0.015	6	388
AGTAGCATTTAGG	0.025	0.01	0.025	23	907
ACTGGCGCTGGCG	0.026	0.003	0.028	17	585
GGTGGTATAGGGA	0.12	0.035	0.085	44	474
GGCGGTATTTGCA	0.076	0.014	0.057	56	932
CCTGGTACATATG	0.025	0.002	0.023	28	1202
AGTGTTGCTAGCG	0.025	0.002	0.023	16	682
GCTGGTATTAACG	0.441	0.039	0.438	591	758
AGTGTCACTAACG	0.026	0.003	0.03	37	1216
CGTGTTACTGGGA	0.029	0.013	0.018	12	637
GGTGGTGTAAAGA	0.11	0.008	0.11	80	646
GCTGGCATAAATG	0.86	0.027	0.845	1929	354
GCTATCGTTGGTG	0.055	0.003	0.053	34	610
CGTAGTACTTGGA	0.032	0.003	0.028	20	684
AGTGGTATTGGCA	0.031	0.002	0.03	26	828
GGTAGCATTAATG	0.078	0.007	0.069	63	847
ACTATTATTGATG	0.024	0	0.024	61	2467
GCCGTTATATGCA	0.061	0.002	0.059	111	1775
GCCAGTGCAGACG	0.053	0.019	0.027	35	1270
GCTATCGCTTAGG	0.057	0.012	0.043	27	607
AGTAGTACTAGTG	0.018	0.005	0.019	17	889
CGTGGCGTAGGCA	0.03	0.012	0.046	30	618
GCTAGTATAGGCA	0.058	0.001	0.057	79	1304
GCCGGCATTGGGG	0.452	0.056	0.383	224	361
CGTATTGCTTACA	0.025	0.006	0.017	24	1384
GGTGTCGCAGGCA	0.075	0.007	0.07	37	494
GGTAGTACTTGCG	0.053	0.006	0.055	29	497
GGTGGCATAGATG	0.732	0.046	0.699	837	360
GGCATTGCTTGTA	0.055	0.006	0.058	60	969
GGTATTGCTGATA	0.072	0.013	0.062	57	869
GGTAGCGTAGATG	0.101	0.015	0.08	52	600
GCTGTCATTGGTG	0.086	0.011	0.101	63	563
CCCGGCACATGGG	0.029	0.006	0.021	22	1002
ACTGGTGTTGGTG	0.031	0.005	0.033	20	590
CGCATTATTAACG	0.027	0.002	0.029	73	2488
CGTGTTGTAGAGA	0.02	0.01	0.006	5	774
GCTGGTGTAGAGA	0.403	0.063	0.432	361	475
AGTAGCATTGGCG	0.022	0.003	0.022	18	810
CGCATTATAAGTG	0.02	0.003	0.02	40	1947

AGCGTTACTTGTA	0.018	0.002	0.018	29	1562
AGTAGCGCATGGG	0.021	0.007	0.03	15	478
GCTAGTGTTAGCG	0.069	0.017	0.053	43	773
GGCGGCATAAAGG	0.55	0.084	0.537	807	695
GGTATCACATGGG	0.107	0.014	0.09	49	498
ACTGTCATAGACG	0.028	0.002	0.03	53	1687
ACTGGTGCTGGGA	0.023	0.003	0.019	15	793
AGTGTCGCATACG	0.031	0.004	0.033	31	912
GCCGGTATATGGG	0.133	0.015	0.129	99	666
CGTATTATAAGCG	0.024	0.004	0.024	38	1555
GCTAGTGTTAGGG	0.044	0.006	0.044	28	612
ACTAGCGTTTACG	0.033	0.005	0.032	39	1190
GGTAGCGTTTGGA	0.104	0.036	0.06	26	409
AGTGTTATTAGGG	0.024	0.013	0.006	4	708
CGTGTCATAGAGA	0.028	0.002	0.03	35	1144
CGTGGCATAAATA	0.026	0.001	0.026	44	1627
GCTGTCGCTGGTA	0.048	0.002	0.05	28	533
GGCATCGCAGGGG	0.102	0.027	0.064	41	599
CGTATCGCTGAGG	0.022	0.006	0.031	20	619
GGTATCGTTTACG	0.155	0.021	0.128	103	702
CGTGTCGCAGACG	0.025	0.002	0.027	25	910
CGTGTCGCAAGCG	0.03	0.01	0.041	34	787
GCCATCATATACG	0.055	0.007	0.052	148	2701
GGCAGCACATACG	0.056	0.006	0.049	65	1252
CGTGGTGCATGTA	0.024	0.011	0.011	8	741
GGTATTGCTGATG	0.11	0.011	0.115	82	631
ACTATCGTTTATG	0.022	0.007	0.013	20	1533
GCTGGTGCAGGCG	0.231	0.029	0.237	131	421
GGTATCGTAGAGA	0.135	0.036	0.091	87	864
GGTATTATAAGGA	0.098	0.011	0.091	130	1306
GGCAGCATTGAGG	0.062	0.002	0.062	54	812
GCTGTTACATGGA	0.064	0.008	0.062	53	798
GCCGTTGTATGGA	0.041	0.011	0.048	42	831
CGTGGTATAGACG	0.029	0.005	0.028	24	834
GGTAGTGTTTGTA	0.059	0.007	0.052	31	568
GCCGTTGCAAGGG	0.046	0.004	0.05	37	698
AGTGGCGTTAACG	0.028	0.006	0.019	9	454
AGCGGTGCATGCA	0.025	0.003	0.03	41	1323
GGTGTCGCAGGGG	0.075	0.009	0.069	18	244
CGTGGTATTAACG	0.024	0.006	0.021	19	888

GGCGGTACATGGG	0.072	0.017	0.056	32	535
ACCGTCGTTTAGG	0.021	0.001	0.021	18	850
CCTGTTGCAGACA	0.023	0.004	0.019	40	2118
CGCAGCGCTTGGA	0.021	0.004	0.026	21	792
GCCAGCACAGATG	0.048	0.004	0.045	71	1522
CGTAGTGCTGATG	0.02	0.005	0.024	15	604
GGTGTTATAAATA	0.085	0.006	0.079	153	1783
GGCGGTGTAAGGG	0.092	0.029	0.061	32	492
GGTATTACATATG	0.106	0.012	0.089	113	1151
CCCGGCGCTAGCG	0.022	0.008	0.025	34	1302
ACTGGTGTATATG	0.023	0.006	0.025	30	1157
CGTGGTGTTGGGA	0.029	0.007	0.019	8	405
GGTATCGTAGGTG	0.12	0.023	0.117	60	454
ACTATCATATGCA	0.031	0.002	0.029	82	2758
GGTGTTACTAGGA	0.093	0.012	0.086	55	582
GCTGGTACAGGTG	0.117	0.022	0.095	57	544
GCCGGTGCTAGGG	0.125	0.021	0.096	47	442
GGTAGTACATGTG	0.062	0.008	0.054	35	616
AGCGTTGCTAGCA	0.022	0.005	0.016	23	1425
CCTATTGCAGGCG	0.024	0.004	0.02	30	1501
ACCAGTGTAGGTG	0.021	0.003	0.018	24	1303
CGTGTCGCTGAGA	0.024	0.001	0.022	17	743
ACCGGCGCTGATA	0.025	0.005	0.03	39	1253
GGTGTCACTTGGA	0.084	0.013	0.074	38	475
CCCGTCGTATGTA	0.02	0.004	0.016	29	1811
AGCGTTGCAAGCA	0.024	0.002	0.027	46	1665
CGCGTTACTAGGA	0.022	0.006	0.016	18	1131
GCCATTGCATGCG	0.044	0.004	0.039	47	1144
GCTAGCGTTGATG	0.085	0.01	0.073	52	663
GCCGGCACTTATG	0.226	0.016	0.224	248	857
CCTGTTACAGGGA	0.027	0.006	0.032	34	1041
AGTAGTGCAAGTA	0.022	0.006	0.015	20	1285
GCCGGCACTAACG	0.207	0.025	0.174	274	1298
ACTGTCATTAGGG	0.024	0.003	0.021	19	893
GCCATTGCTGATG	0.047	0.003	0.044	49	1063
ACCGTCATTTAGA	0.02	0.003	0.024	54	2233
GGTGTTGTTAACA	0.079	0.009	0.083	85	933
ACCGGCGCTGACA	0.024	0.005	0.018	29	1583
GGCGGCGCATATA	0.084	0.009	0.076	84	1015
GGTGGCGTTAACA	0.378	0.047	0.37	364	621

GGCGGTGTAGAGA	0.054	0.016	0.039	31	767
CGCGGTGTTAGTG	0.025	0.002	0.022	14	623
GGTATCATATGGG	0.12	0.012	0.103	57	496
GCCAGCATTAGGA	0.06	0.01	0.047	64	1298
CGTATTGCAGAGG	0.024	0.006	0.018	14	784
GCTAGCGTTTATG	0.084	0.009	0.081	59	667
GGTGTTGCTGACA	0.084	0.023	0.06	45	707
GGCGTTGCATGGG	0.067	0.007	0.068	36	495
CGCGGCGCAAGCG	0.021	0.005	0.025	24	955
GCTGGTACAAGCG	0.112	0.015	0.111	91	727
GCTAGTACAAAGG	0.065	0.008	0.076	92	1125
GCCAGTGCTTGGA	0.034	0.003	0.038	28	708
CCTGTCGTTGGGA	0.024	0.009	0.012	10	800
AGTGTTACTGAGG	0.029	0.002	0.032	24	735
CCTATTGTTGATG	0.033	0.004	0.031	55	1744
CCCAGCGCAGAGG	0.026	0.002	0.029	36	1210
CGCGGTATAGACA	0.023	0.005	0.02	39	1938
AGCGTCGTTAACG	0.023	0.006	0.026	42	1590
CCCGGTGTTAACG	0.027	0.006	0.026	45	1668
ACTGGTATTTGCA	0.025	0.001	0.024	34	1385
CCCATTACATGGG	0.029	0.005	0.025	38	1513
GCTATCGTTTGGA	0.054	0.01	0.041	31	719
CGTATTGTAGGCG	0.025	0.007	0.015	14	890
GGTATTGTAAGGG	0.116	0.008	0.107	66	552
GCTGGCATTAGCA	0.664	0.04	0.656	973	510
GGTAGTGTTTATA	0.065	0.019	0.046	41	849
GCCGGTGTATGGA	0.18	0.027	0.194	155	642
CGTATTGTTAGGG	0.029	0.001	0.028	18	621
CGTGTCACAGACG	0.024	0	0.024	26	1066
CCCGGTATTGGCG	0.026	0.001	0.026	32	1210
GGCGGCACAGAGA	0.135	0.019	0.115	121	928
CCTGTCGCAGGCA	0.017	0.005	0.01	14	1411
GGTGGCACAAGGA	0.191	0.031	0.178	122	564
CGTAGTGCTGACG	0.022	0.005	0.029	19	643
GCTGTCATTAGCA	0.051	0.003	0.051	61	1133
GGTGTCGCATACG	0.099	0.005	0.107	76	637
GGTGTCGTTAGCA	0.101	0.011	0.108	67	553
GGTATCATTGGCG	0.101	0.02	0.082	63	706
CGCGTTATTTGTA	0.022	0.005	0.021	30	1389
GGTGTTGCAAGCA	0.079	0.001	0.079	64	747

CGCGTCGCATGTA	0.026	0.005	0.021	27	1264
CCCATCGTTTGCG	0.03	0.002	0.027	44	1560
GGCGTTGCTAATG	0.061	0.002	0.063	56	837
CCTAGCGTTGATG	0.028	0.004	0.022	27	1192
GGTGGTGCATACA	0.069	0.013	0.056	47	798
CCCGTTGCAGAGG	0.029	0.002	0.027	30	1097
ACTAGTACTAGGG	0.027	0.002	0.027	35	1247
GCTGTCGTTGGCG	0.069	0.017	0.07	36	476
GGCGTCATTAGGG	0.078	0.018	0.053	37	665
AGCAGTGTAAGTG	0.018	0.004	0.018	29	1595
GCTATCGTAAGGG	0.06	0.003	0.06	47	741
GCTGTTGTAGGGA	0.056	0.013	0.051	32	594
AGCGGTACATGTA	0.02	0.002	0.017	28	1613
CGCGGCATATAGA	0.023	0.003	0.019	28	1440
ACCATTGTAAGGG	0.023	0.006	0.015	27	1793
GCTGTTGCTAGGA	0.048	0.005	0.048	29	579
GGTGGTGTTAAGA	0.104	0.02	0.092	50	496
CGCATTATTAGGG	0.031	0.008	0.042	46	1049
GGCGGCGCTTAGG	0.172	0.035	0.145	72	425
AGTAGCGCAAGGG	0.029	0.009	0.023	14	601
GGTGTTGCAAGGA	0.087	0.021	0.076	41	502
GCTGTTACTAGGA	0.063	0.008	0.052	45	824
GGTATCGCTGATA	0.104	0.014	0.098	96	888
AGCAGTGTTAGTA	0.022	0.003	0.018	31	1728
GCCGGCATAAATG	0.743	0.028	0.738	1873	664
GCTATTGTATGTA	0.047	0.006	0.04	60	1426
GCTGTTACTAAGA	0.059	0.008	0.051	64	1192
GCCATTACTGACA	0.049	0.008	0.039	104	2531
GGTAGTATAGATA	0.06	0.014	0.054	79	1394
AGTGTTGTTGGGG	0.028	0.004	0.027	10	363
CGTATCGTTTGTA	0.031	0.007	0.04	42	1019
GCTATCATTGGCG	0.059	0.004	0.056	77	1302
CGCAGCGCATGGG	0.034	0.014	0.017	13	770
CCCGTTACTTGTG	0.026	0.002	0.023	29	1249
GCTATCGCTGGTA	0.043	0.007	0.044	34	747
CGCGTTGTTAGTG	0.024	0.008	0.014	11	775
CGTATCGCAGACG	0.027	0.003	0.023	27	1138
AGTGGTACTGGTA	0.018	0.002	0.016	12	759
GCCGGCACATAGA	0.109	0.01	0.113	155	1213
CCCGTCGCATGCA	0.021	0.005	0.014	31	2211

CGTGGCATAGAGA	0.026	0.003	0.021	18	824
CGTGTCGCAGGGG	0.02	0.006	0.016	8	492
GCTAGCACTAGCG	0.058	0.013	0.04	35	851
CGTGTCGTTTGCG	0.024	0.004	0.029	16	528
AGTGTCACTTGGG	0.027	0.006	0.035	19	521
ACCGTTGCAGGGG	0.026	0.004	0.021	21	967
GGCGGTACTAGCG	0.071	0.013	0.057	40	657
CGTGTCGCAAGGG	0.025	0.006	0.018	10	538
CGTGTCGCAGACA	0.025	0.008	0.029	33	1120
GGTATCATAAGCA	0.13	0.019	0.104	153	1314
GCCATCGCAGGGG	0.053	0.008	0.049	33	641
AGTGTTGTAAACG	0.021	0.006	0.018	25	1396
AGTATTGCTGACG	0.022	0.004	0.017	20	1189
GGTAGCATAGACG	0.075	0.01	0.074	62	780
GCCGTCGTAAACG	0.053	0.009	0.048	61	1202
GGTGTCGTTGATG	0.113	0.012	0.1	51	459
CCTATCGTTTAGG	0.031	0.009	0.018	21	1116
CCTGGCGCAAATG	0.026	0.002	0.029	39	1300
GGTGTTGTAGGGG	0.073	0.024	0.042	16	367
GGTAGTGCTGGGG	0.057	0.014	0.073	19	242
CCTGGTACTGGGG	0.028	0.009	0.019	9	475
AGTATTGCTTGCA	0.021	0.008	0.015	17	1147
GCCGGCATAGGCA	0.384	0.035	0.383	645	1039
CGTGTTACAGGGG	0.027	0.006	0.021	11	517
GCCGGCGCTTGCA	0.497	0.047	0.494	550	563
ACCATCGTAAGGG	0.028	0.004	0.025	38	1486
GCTAGTGTTGGCG	0.054	0.012	0.038	38	951
AGCAGTATAAGCG	0.018	0.004	0.013	31	2350
CGTATCGTAAGTG	0.03	0.003	0.026	33	1232
CGCAGCACATACG	0.026	0.001	0.025	49	1873
GCTGTTATAAGGG	0.068	0.011	0.053	41	735
CGTAGTGCAAGGG	0.025	0.013	0.012	7	564
CGCGGTACTAGTA	0.024	0.006	0.016	22	1345
GCCGTCGCAAGGG	0.048	0.016	0.067	38	531
GGTGGTACTTAGG	0.102	0.012	0.087	31	324
CCCGTCGTATGCA	0.026	0.002	0.028	59	2054
GCTATCACTTACG	0.061	0.005	0.059	83	1322
GCCATCACTGGGG	0.059	0.009	0.062	49	737
CGTGTCACTAAGA	0.026	0.011	0.016	20	1198
CGTATTGTTGACG	0.03	0.003	0.027	27	981

AGCGGCGCTTGCG	0.018	0.004	0.013	10	756
AGCGTCACTGAGG	0.021	0.006	0.013	14	1093
ACCAGTATTAGGG	0.029	0.003	0.028	45	1569
GGCAGCACTAGCG	0.056	0.007	0.055	55	944
CGTAGCACTAAGA	0.026	0.008	0.023	27	1171
GCTAGCGCAGGGA	0.056	0.004	0.05	29	554
GGTAGCATAGGTG	0.069	0.019	0.042	25	566
GCTGTCGCTGACG	0.062	0.012	0.06	35	544
GCTGTCATTAATG	0.063	0.008	0.051	65	1201
GGCGGTGTTTGTA	0.057	0.007	0.061	42	643
GGCATTACTGACG	0.06	0.003	0.056	85	1434
GCCGGCGCTTGGG	0.735	0.029	0.756	510	165
GGTAGCATTGGTA	0.056	0.008	0.053	35	631
GGCAGCGCTGATA	0.052	0.01	0.04	49	1170
CGTGTCACTTATA	0.025	0.003	0.021	26	1205
GGTGTCGCTGAGG	0.089	0.014	0.101	36	320
CGTATCACTGAGA	0.021	0.003	0.018	21	1174
GGCGGCATAGACA	0.305	0.054	0.276	478	1251
AGTGGTGTAGGGG	0.026	0.005	0.029	10	340
AGCGTTGCATGCG	0.025	0.004	0.023	26	1117
GGCAGTACTGGTG	0.047	0.007	0.05	38	723
GCCATCGTTTAGG	0.06	0.008	0.052	50	913
GGCGGTATTGGGA	0.061	0.005	0.056	37	626
AGTAGCGTATGTA	0.019	0.006	0.011	14	1204
GGCGTTGTAGGTG	0.072	0.013	0.058	36	586
ACCATTGCTTACA	0.021	0.002	0.023	88	3672
GCTGGTGCAGGGG	0.236	0.029	0.24	87	276
CCCAGTATAGGCG	0.031	0.004	0.025	50	1915
CGTAGCGTTTACA	0.021	0.009	0.009	10	1090
GCTATCGTTGGCA	0.061	0.001	0.062	63	952
CGTGTCATTTAGG	0.027	0.006	0.025	17	676
AGCAGTATTTGGG	0.019	0.004	0.019	22	1108
GCTATTGTAGGTG	0.053	0.004	0.057	49	806
GCTATCGTTTGTA	0.063	0.013	0.047	51	1039
CGCAGCATTAAGA	0.025	0.002	0.022	47	2074
CCTGGTACTAGCG	0.023	0.003	0.027	26	938
GCTAGCGTAGATG	0.076	0	0.076	62	750
AGTGGCACAGATG	0.027	0.007	0.036	23	608
GGTAGTACTAGTA	0.06	0.005	0.055	54	931
AGTGTTATATAGA	0.021	0.005	0.021	35	1648

CGCATTATTAGCG	0.028	0.008	0.025	40	1590
CCTGGCACAGAGA	0.026	0.005	0.019	26	1312
GGTAGCGCTTATG	0.081	0.008	0.079	42	489
GCTGGTATTTAGG	0.445	0.053	0.435	283	368
CGTGGTACTAGCA	0.029	0.005	0.036	35	929
CCCGTTGTAGGCG	0.02	0.003	0.017	23	1301
CCTGGTACTGGTA	0.021	0.006	0.013	14	1087
GGTGTTGCATAGG	0.117	0.011	0.102	48	424
GGCGGCGCAGGGG	0.144	0.016	0.14	59	361
CGTATCACTGAGG	0.028	0.003	0.026	21	792
GCTGGCGTAGGGA	0.859	0.032	0.856	752	127
GGTAGTGCTTACG	0.059	0.006	0.052	30	550
CCCAGCGCATGGG	0.025	0.005	0.03	35	1136
GGTGGCGTTGACA	0.389	0.049	0.371	282	479
CGCGTTGCTGGTG	0.018	0.005	0.012	8	675
GGCGGTGCATGGG	0.058	0.011	0.05	21	400
CCTAGTATTTGGA	0.024	0.002	0.026	35	1308
GGTGTCATTTATA	0.085	0.017	0.064	64	938
GCTGGTGCTGGTG	0.258	0.021	0.263	108	302
GCTGGCATAGGTG	0.868	0.024	0.856	853	143
GCTGTCGCTTAGA	0.058	0.014	0.055	35	599
GGTAGTGTTTGCG	0.064	0.016	0.055	25	433
ACTGTTACAAGCG	0.024	0.001	0.025	43	1671
GCCGGCGCAGATA	0.481	0.038	0.453	680	822
CGCGGTGCAGACG	0.024	0.003	0.022	24	1076
AGTAGTGTATGGG	0.024	0.002	0.023	15	630
CGTGTCATATGCG	0.028	0.001	0.029	23	784
CGTGGCGTTAAGA	0.029	0.003	0.029	21	712
ACTGTCATTGGGA	0.027	0.005	0.031	35	1095
CCTGGTGTAGACA	0.023	0.002	0.021	35	1597
AGCGTTGCTGACA	0.026	0.008	0.019	35	1777
ACTGTTGCATATA	0.027	0.003	0.022	53	2305
GCCATTGTATGCG	0.051	0.004	0.056	70	1182
CGTGTTGTAAACG	0.027	0.004	0.032	35	1059
CGCGTTGCTGGCG	0.02	0.004	0.014	13	899
CCCGTCACTAGTG	0.025	0.003	0.027	43	1528
GGCGTTGTTGACG	0.065	0.004	0.062	54	815
GCTGTTGCTTAGG	0.067	0.021	0.053	27	484
CGCGGCACTTGTG	0.025	0.003	0.028	21	729
GCCGGTATATACA	0.07	0.004	0.07	168	2215

AGTAGTACTGGTA	0.025	0.005	0.023	25	1081
CGTGTCATAGGTG	0.024	0.005	0.031	21	663
AGTGTCACAGGTG	0.031	0.006	0.022	14	617
GGTGTCACTGGGG	0.077	0.009	0.064	20	291
GGTAGTGTTTGCA	0.061	0.013	0.044	27	590
AGTGGCGTAAGTA	0.028	0.014	0.021	17	796
GGCATTGTTTGCA	0.072	0.007	0.063	77	1137
AGCGGCACATATG	0.025	0.009	0.013	15	1127
CGTGTCATATAGG	0.035	0.004	0.031	26	817
GCTAGCGTATGGG	0.083	0.005	0.088	39	406
CCTGTTGTTTAGG	0.024	0.004	0.02	19	910
CGTAGCGCAAGTG	0.025	0.007	0.014	11	755
GCCATCGTAGGTA	0.048	0.003	0.047	63	1279
CGTGGCACTAATG	0.021	0.003	0.024	18	727
CGCGTCACTGGCA	0.019	0.006	0.023	33	1426
AGTAGTGCAGGGA	0.028	0.007	0.037	26	676
GGCGTTGTAAGCG	0.062	0.008	0.052	46	844
ACTAGCATTGGCG	0.029	0.005	0.035	49	1343
ACTGGCGCTTGCG	0.032	0.008	0.042	26	589
AGCGGTGCAGGTG	0.022	0.003	0.022	16	716
CCTATTGTAGGGA	0.026	0.003	0.025	34	1310
GCTGTTACTGGGG	0.062	0.011	0.053	24	432
AGTGTTACAGATG	0.023	0.006	0.023	26	1125
GCCAGCGTTTACG	0.065	0.005	0.072	77	996
CGTAGTGCTTGGA	0.025	0.009	0.019	9	473
AGTATTATTTGGG	0.02	0.008	0.023	23	998
GGTGGCATTTGTG	0.716	0.057	0.675	498	240
CGCGGCGTTTAGG	0.036	0.003	0.037	19	501
CGTGGCGTAGAGG	0.028	0.011	0.015	6	397
GGTGGTGCTGGTA	0.072	0.006	0.076	27	328
GCCGGCGTTGGCA	0.853	0.031	0.858	1218	201
CCTGGCACAAATG	0.031	0.003	0.026	42	1558
AGTGTTGCATAGA	0.02	0.008	0.027	27	967
CGTATCGCTAACG	0.026	0.002	0.024	29	1164
GGTATTGCAAACG	0.105	0.024	0.089	104	1071
CGTATCGCAGGCG	0.025	0.004	0.02	20	982
ACCGGTGTAAGGA	0.018	0.007	0.01	15	1498
GCCAGTGCAAAGG	0.05	0.002	0.049	54	1055
GGTAGTACAGAGA	0.066	0.006	0.058	52	837
GCCAGTATTGGGG	0.076	0.007	0.085	62	671

CGTGGTATTGGGG	0.022	0.008	0.029	9	301
AGCGGCACTAGCA	0.019	0.005	0.014	23	1570
CGCAGCGCAGGGG	0.024	0.004	0.022	14	614
GCTGTTACATACG	0.06	0.004	0.054	62	1081
GCTATCGCAGGCA	0.055	0.003	0.056	61	1023
ACCGGTGTTTGGA	0.021	0.006	0.017	18	1012
GGCGGTACAGGTA	0.067	0.021	0.061	61	939
CGTGTTATTTGCA	0.028	0.008	0.02	22	1071
CGCAGCACTTGCA	0.02	0.004	0.015	25	1638
AGCAGTGCTTAGA	0.022	0.002	0.024	35	1450
ACTGGCGCAAGCG	0.027	0.006	0.032	31	928
AGCAGCATTTGCA	0.02	0.004	0.014	29	2092
CCTATCGCTTATG	0.024	0.003	0.02	29	1401
GGCAGCACAGGCG	0.054	0.016	0.032	33	999
CGCATCGCAGGGA	0.023	0.001	0.023	25	1086
CGCGTCATTGATA	0.025	0.001	0.024	45	1855
CGCGTTACTAGGG	0.03	0.003	0.034	26	748
CGCGTTGTTTATA	0.029	0.005	0.032	44	1335
CGTGGCGCTGGCG	0.025	0.012	0.022	10	451
CCTGGTATTTATG	0.024	0.002	0.023	27	1138
AGTGGTATAGGGG	0.025	0.007	0.034	17	477
AGTGGCATAAGGG	0.02	0.009	0.009	2	228
AGCGGTGCATGTA	0.026	0.003	0.025	31	1202
GGTATCGTAGGGA	0.106	0.01	0.098	63	578
GCCAGCGTTAGGG	0.045	0.008	0.044	29	629
CGTAGTATATGGA	0.023	0.003	0.026	25	955
GCTAGTGTAGATA	0.055	0.006	0.054	85	1482
ACTGGCACAGACG	0.025	0.003	0.028	41	1410
CCCGGTGTAGGTA	0.019	0.003	0.019	24	1243
GGCATCACTGGTG	0.084	0.007	0.079	75	872
ACTGTTGCTTGGG	0.024	0.002	0.021	14	651
AGCATTGTTAGTG	0.022	0.002	0.021	30	1422
GCCATTGCTTGTG	0.046	0.007	0.036	32	859
CGTGGCGTTGATG	0.025	0.005	0.021	9	428
GCCGGTGTAAGGG	0.366	0.06	0.338	263	514
ACTAGCGTAGATA	0.025	0.004	0.019	39	1972
CGTAGTGTTGAGG	0.019	0.008	0.01	5	493
CGCATCGCAAAGG	0.029	0.002	0.026	40	1487
CGTGTTATAAGTG	0.021	0.004	0.026	28	1050
AGTATCACTTATG	0.025	0.004	0.019	24	1228

GGCATCGTTTGGG	0.127	0.026	0.117	68	514
AGTGTCACAGAGA	0.028	0.008	0.034	39	1101
AGTGTCATTGATA	0.02	0.003	0.016	24	1480
ACTAGCGCTGGTG	0.027	0.003	0.025	21	810
GGCGTCGCAGATG	0.067	0.01	0.054	43	753
ACTAGCGTTGAGG	0.027	0.005	0.021	18	835
ACCGGCGTAGGTG	0.026	0.01	0.017	9	518
GCTGTTACAGACA	0.058	0.008	0.056	88	1478
GCCATCGCTGGTA	0.053	0.008	0.042	45	1017
AGCGTCATTGATG	0.028	0.007	0.019	30	1522
GCCGTTGTTGACG	0.053	0.005	0.05	50	950
AGCGGTGTATGCA	0.021	0.003	0.017	24	1368
CGTGTCGTTAGCG	0.02	0.012	0.003	2	607
GCCAGCGCATGTA	0.054	0.005	0.06	74	1158
CCTGTCACAAAGG	0.031	0.004	0.031	45	1422
GGTATTACTGGCG	0.075	0.024	0.048	44	875
CCCGTCGTTTACG	0.022	0.005	0.021	34	1555
CGTGTTGTTGGCG	0.024	0.001	0.024	14	562
CCTGGTATTTAGG	0.025	0.004	0.026	22	813
GGCATTGTTAGCG	0.089	0.014	0.074	70	879
GGTAGTACTGGTA	0.069	0.003	0.066	47	661
AGTATTGTTAGCG	0.023	0.003	0.023	23	980
GCTGGTATTGGCG	0.391	0.027	0.363	240	422
AGCAGTACTAGCA	0.02	0.004	0.023	56	2397
AGCATCACTGGGG	0.02	0.003	0.017	23	1302
CGCGGCATAGGCG	0.023	0.007	0.028	24	838
ACTGGTGCATGCG	0.019	0.008	0.025	23	912
AGTGGTGCTGAGG	0.022	0.006	0.028	12	422
GCTATCGTTTACG	0.061	0.007	0.057	63	1048
GGCAGCATATGCA	0.059	0.008	0.053	87	1564
GCCGTTATAAAGG	0.063	0.005	0.068	114	1553
GGTGGTGCTTGTG	0.092	0.014	0.084	24	263
AGTAGTATATACA	0.021	0.004	0.016	43	2590
CCTGTTGTTTACG	0.025	0.007	0.03	41	1346
AGTGTCGTTAGCA	0.027	0.008	0.035	33	919
AGTGGTATAAGCA	0.02	0.001	0.02	30	1436
GCTATCACAGGCG	0.055	0.001	0.056	74	1237
CGCAGCGTTGATA	0.025	0.004	0.025	37	1421
CGTGTTGCTTACA	0.028	0.001	0.026	29	1077
AGTAGTGTATAGG	0.023	0.006	0.016	15	925

AGCGTCACAGGTG	0.02	0.005	0.027	32	1173
AGTGTTACTGGCA	0.02	0.003	0.021	21	980
GGCGTCACTGGTG	0.057	0.004	0.053	36	641
GGCGTTGTAAGGG	0.07	0.012	0.055	38	659
GGTAGCATATGCG	0.071	0.014	0.058	38	621
GCTGGTGTTGGGA	0.397	0.054	0.398	218	330
GCTGGTGCAGGTA	0.105	0.008	0.095	63	597
AGCGTTACAAGGG	0.019	0.004	0.013	15	1138
ACTGGCACTAACA	0.027	0.002	0.028	69	2379
GCCGGTATTTGTG	0.146	0.015	0.146	132	770
CGTGGTATATGTA	0.029	0.005	0.022	22	982
GGTAGTGTAAATG	0.052	0.008	0.05	49	930
GGCGTCGTTTGTG	0.104	0.014	0.096	52	489
GGTAGTATTTATG	0.073	0.013	0.056	49	833
GGCGGTATTGACG	0.106	0.022	0.119	111	823
AGTGGCGCAAGGG	0.023	0.01	0.014	6	415
GGCATCATAAGTG	0.094	0.014	0.078	120	1415
GCTGGTATTAGTG	0.423	0.028	0.446	331	411
GCTATCGCTGGTG	0.053	0.008	0.044	28	609
GGCGTTGCTAGGA	0.064	0.004	0.063	40	595
GCCGGCATTGACA	0.371	0.039	0.369	836	1431
CGCAGCGCTGAGA	0.026	0.006	0.018	20	1066
GGCAGCACAAGTG	0.055	0.005	0.053	64	1150
AGCATCGCAGGGG	0.023	0.002	0.025	28	1101
GGCGTCGTAGGTA	0.066	0.005	0.061	51	781
GCCAGCACTGGGG	0.064	0.013	0.064	38	557
GCTAGCACTAGGG	0.059	0.003	0.062	39	587
CCTGTTGTTTACA	0.024	0.002	0.022	39	1774
AGTAGTGCTGGCG	0.032	0.006	0.027	17	606
CGTGTTACTAGCA	0.025	0.008	0.016	19	1150
CGTATCATTAGTG	0.033	0.007	0.028	31	1074
CCTGTCGCATGCG	0.019	0.003	0.015	15	958
AGTGGTGCATAGA	0.032	0.004	0.026	23	877
CGTGTTGCTTGGA	0.026	0.003	0.022	11	479
CCCGTCACAGGGA	0.041	0.01	0.028	44	1503
GGCGGCACATGCG	0.265	0.052	0.22	189	671
AGCAGCGTAGGTA	0.019	0.002	0.018	27	1484
CGTGTCGCATGCA	0.028	0.005	0.021	19	900
ACTGTTGTTAGCA	0.027	0.003	0.024	37	1508
GCTATCATTAAGG	0.066	0.01	0.054	74	1288

ACTGTCGCAAAGG	0.024	0.001	0.025	32	1269
AGTAGCATATAGA	0.028	0.002	0.028	47	1643
ACCAGCGCTGGCG	0.019	0.002	0.017	24	1399
GCTAGTGTAGGGG	0.064	0.016	0.051	29	544
GGCAGTGTAGGCA	0.049	0.01	0.042	47	1067
AGTGTCGCAAGGG	0.023	0.006	0.015	8	532
GGTAGTGCTTATG	0.058	0.009	0.047	24	489
AGTGGTGCAAAGG	0.026	0.008	0.017	11	648
AGTATCACTAGCG	0.027	0.007	0.02	22	1060
ACCGGTGCATGCG	0.022	0.002	0.024	32	1313
AGCGGTGCAGGTA	0.023	0	0.023	24	1033
CGCATCACAGGGG	0.041	0.005	0.038	35	894
CCCATTACTGGCA	0.026	0.002	0.023	62	2606
CCTAGCATTAGTG	0.031	0.008	0.026	32	1200
AGTGTTGCTAACG	0.023	0.005	0.017	17	978
GCTATTACATGCG	0.059	0.003	0.056	71	1197
CGTAGTGTTGAGA	0.027	0.012	0.02	15	722
CGCGTCGTTGGTG	0.025	0.007	0.016	12	718
CGCGTCGCTTGTG	0.03	0.004	0.031	23	713
CCTGTCATAGGTA	0.025	0.004	0.023	36	1502
GGCGGCATTGGCA	0.27	0.052	0.25	257	773
CGCATCACAGAGG	0.029	0.006	0.028	40	1379
CCCGGTGTAGGCG	0.025	0.006	0.02	23	1154
GCCGGCACAAAGA	0.104	0.008	0.114	210	1640
CGCGGTGCAAGGA	0.023	0.004	0.018	19	1064
AGTGTCATAAATG	0.032	0.009	0.022	37	1651
CGTGTCACAAGGG	0.03	0.001	0.03	20	640
ACTGTCGTATGCG	0.029	0.002	0.026	25	921
GGCATTGCATAGA	0.058	0.006	0.059	73	1165
CGTAGTGCATATG	0.033	0.002	0.033	23	666
GGTATTGCAAACA	0.082	0.012	0.077	132	1586
CGTATCATATGCG	0.026	0.004	0.022	30	1310
CGCATTGCAAGGA	0.02	0.002	0.022	33	1452
GGCAGCACTAAGA	0.044	0.001	0.044	68	1470
AGCATCGCTGGTA	0.02	0.004	0.024	35	1431
GCTAGTGCAGGGG	0.062	0.018	0.05	29	555
GGTATTGTTGACG	0.104	0.012	0.091	72	719
ACTATCGTATGCA	0.028	0.001	0.027	54	1934
GCTGGTATAGATG	0.436	0.047	0.454	493	593
ACTGTTATATGGA	0.027	0.005	0.023	36	1545

CGTAGTACTGGTA	0.026	0.006	0.034	31	882
GCCGGTGCTAGGA	0.072	0.008	0.079	54	628
GGCGTCATTGGCA	0.07	0.007	0.06	59	926
GGCGTTGTAAATG	0.079	0.005	0.073	93	1179
GCTGGCATTTATG	0.874	0.022	0.874	1326	192
GGTATCGCAGGGG	0.128	0.007	0.122	53	383
ACCATCACTAGGG	0.029	0.001	0.028	42	1436
GGTGGCGCTGGCG	0.285	0.051	0.31	104	231
ACTGGCGTTGAGG	0.031	0.013	0.013	5	375
ACCGGTATTGAGG	0.022	0.005	0.018	23	1276
GCCGTCGCAAAGG	0.055	0.005	0.048	39	774
CGTAGTGTATGGG	0.023	0.007	0.025	12	463
GCCGGCATAGACG	0.727	0.046	0.731	1402	515
AGTGGTGTATGTA	0.021	0.003	0.017	14	815
CGCATTGTTAATG	0.026	0.002	0.024	42	1703
CGTAGCGTATGTG	0.026	0.001	0.027	19	686
GCTAGTGCTGGCA	0.054	0.001	0.053	49	873
CGCAGCACTGGCG	0.021	0.002	0.023	24	1039
CCTAGCACAGAGG	0.031	0.005	0.031	37	1149
ACCGGCACAGGCG	0.024	0.004	0.02	31	1554
CCCGGTGCTGATA	0.021	0.003	0.02	35	1757
ACTGGCGTTGGCG	0.018	0.002	0.015	6	395
AGTGTCATAGGTG	0.025	0.005	0.02	14	695
GCCGGTGTTAGGG	0.374	0.068	0.331	197	398
CGTAGTGCTTAGG	0.021	0.005	0.023	11	464
CCTGGCGTAAGCG	0.021	0.007	0.015	16	1031
AGTGGCACTAACA	0.026	0.004	0.03	47	1539
CGTGTCACTTGGA	0.024	0.004	0.021	14	652
GGTGGTGCAGGGG	0.09	0.024	0.065	15	216
GCTGTTATTTAGA	0.064	0.004	0.058	69	1128
CCTATCGCTAGGA	0.025	0.003	0.021	31	1473
CGCGGCGTTTGCA	0.024	0.004	0.028	25	862
CGCGGTGCTGAGG	0.013	0.002	0.016	9	544
GGTATTATTTAGG	0.112	0.005	0.109	98	797
AGTAGCGCAGGTA	0.025	0.008	0.014	13	938
CGTGGCGCATGGG	0.034	0.009	0.025	9	344
GGCATCGCTGACA	0.08	0.004	0.077	129	1539
CCCGGCACTGATG	0.027	0.005	0.024	37	1487
GCTAGCGCATGCA	0.053	0.003	0.05	45	862
GCCGTCACAAACG	0.052	0.007	0.043	90	1996

CGTGTCACTTGTG	0.022	0.003	0.026	16	610
CCTGTTACAAGGG	0.026	0.007	0.027	26	955
GGTATTGTAGAGG	0.106	0.014	0.118	75	563
GGTAGCATTAACA	0.065	0.001	0.063	90	1339
GGCAGCATAGATA	0.057	0.007	0.048	92	1814
AGTGGCATTGGCG	0.027	0.01	0.015	3	192
CGCATCGCTGATA	0.02	0.004	0.017	29	1719
CGCATTGTTGGCG	0.025	0.003	0.025	28	1098
ACTAGCATTGGGA	0.025	0.007	0.015	17	1094
GGTATCACAAGGA	0.105	0.011	0.092	86	850
AGTGTCGCTTACG	0.025	0.011	0.021	16	733
GCTGGCACTGAGG	0.437	0.035	0.43	275	364
CGTGGTATTGGTG	0.022	0.005	0.023	10	432
GCTATCATTAGGG	0.079	0.009	0.086	66	702
GGCGGTGCATATG	0.062	0.006	0.066	46	654
GCTGTTATATGGG	0.063	0.014	0.067	43	596
ACTGGTACTTGTG	0.024	0.004	0.02	19	911
CCCAGCACTTGGG	0.037	0.006	0.037	41	1054
CCCGTTGTATGGA	0.021	0.004	0.016	21	1331
AGTGGTGTTAATG	0.031	0.012	0.018	14	750
CGCAGTGCAGAGA	0.026	0.002	0.028	33	1151
GCCGGCATATATG	0.737	0.039	0.743	1423	492
GCCGTTACTAAGG	0.056	0.011	0.043	48	1077
GGCGTTGTAGATG	0.07	0.014	0.054	51	899
ACCGGTGCTAGGG	0.023	0.006	0.031	26	805
ACTGTCGTTGATG	0.022	0.003	0.019	19	989
AGTAGTACTAGGG	0.021	0.001	0.02	14	701
GGTGGCACATGGA	0.202	0.029	0.219	126	450
GCTGTTGTTTATG	0.061	0.01	0.058	41	670
CGTGGTGTTGGTG	0.022	0.004	0.017	6	351
GCTGGCGCATGCG	0.824	0.034	0.828	773	161
AGTAGCATAGGGA	0.025	0.008	0.027	34	1221
GGCAGTATTGGGA	0.058	0.006	0.062	64	969
ACTGGTATTTGGG	0.029	0.003	0.032	21	629
AGTGTTATTTATG	0.024	0.002	0.025	32	1249
GGTAGTATTGGTG	0.068	0.01	0.08	43	495
GCCAGCACTTGGA	0.052	0.006	0.054	58	1007
GCTGTTGCATGTG	0.071	0.015	0.059	35	559
AGTGGTATTAGGA	0.025	0.009	0.025	21	808
GGCAGCACAGAGG	0.053	0.004	0.047	44	895

ACTGTTGTTTGCA	0.028	0.004	0.027	33	1204
ACTGTTGCTGATA	0.025	0.002	0.022	40	1753
GGCGGTATATACG	0.132	0.033	0.114	142	1103
ACTGTCGTAAGGG	0.023	0.001	0.023	16	670
GCCGTCACTGGGA	0.054	0.009	0.064	60	878
AGCATCGTATACG	0.025	0.001	0.023	54	2283
GCTAGTGTTTGGA	0.052	0.01	0.039	29	723
AGTATCGTATGTG	0.018	0.002	0.015	16	1018
GGTATCACTGATG	0.137	0.013	0.13	111	745
CCTAGCATATGTG	0.024	0.002	0.021	30	1387
GGTGGTATATGCA	0.115	0.017	0.1	80	717
GGTGTTGTTAAGG	0.092	0.012	0.084	45	489
ACTGTCGTTAACA	0.028	0.004	0.024	53	2117
AGTAGCATTTGCG	0.024	0.005	0.025	21	834
GCTATCATTTACG	0.071	0.012	0.057	95	1564
CGCAGCGTAGGGG	0.038	0.002	0.035	21	573
CCTGTCGTAGGTG	0.023	0.003	0.018	17	916
GCTAGCGCTGATG	0.076	0.008	0.073	49	618
CCCAGCGCTAATG	0.023	0.002	0.024	47	1886
CCCAGTACTTGTG	0.026	0.002	0.025	39	1530
GCTATCGTTGACA	0.057	0.005	0.056	90	1519
AGTATCACTGACG	0.029	0.003	0.032	39	1193
GGTGGCACTGGTA	0.208	0.047	0.175	101	475
ACCGGCACATAGG	0.022	0.004	0.018	29	1576
CCTAGCACTTATG	0.029	0.013	0.018	26	1453
GCTGGTACTTGGG	0.114	0.017	0.09	35	355
GGTAGTGCTGAGA	0.042	0.005	0.047	27	551
GCTAGTGCATGGG	0.06	0.017	0.036	20	542
GCTAGTACTGGGG	0.07	0.011	0.085	41	442
CCTGTCACTTGGA	0.025	0.006	0.017	19	1067
GCTGTCGCAAGTG	0.056	0.007	0.046	24	499
GCTGTCGCTAACG	0.056	0.014	0.041	32	749
CGTATCGCTTATA	0.025	0.003	0.023	33	1378
AGTGGTATTGGTG	0.023	0.014	0.009	4	451
CGTGTTGTAGATG	0.019	0.004	0.024	19	762
GGTAGCATTAGTA	0.064	0.004	0.06	53	827
CGCGGTACTTGTA	0.022	0.008	0.019	22	1128
AGCAGTGTTAGGG	0.02	0.004	0.024	23	922
ACCATCACATAGG	0.023	0.003	0.027	74	2641
AGCGTTGTTTATG	0.02	0.007	0.019	22	1154

CGTAGCGCAGATA	0.024	0.002	0.026	29	1089
AGTGGTGTTAGTG	0.027	0.004	0.023	11	477
AGTAGTGCTAAGG	0.023	0.008	0.016	12	740
GGCAGTGTATGGA	0.051	0.01	0.037	33	849
AGCGGCGCTGGTA	0.021	0.005	0.028	24	840
GGCAGCGTAGGGG	0.05	0.01	0.04	28	680
GGTGGCGCTGGGA	0.168	0.024	0.182	54	242
CCCGTCGCTGGGG	0.026	0.005	0.025	18	711
CGTAGTGTAGAGA	0.034	0.004	0.037	31	803
GCTAGTATTAATA	0.066	0.005	0.059	150	2388
GGTGTCGCTAGGA	0.075	0.003	0.074	31	387
ACTAGTATAGAGG	0.024	0.007	0.016	25	1532
GGCGTTGCAAACG	0.063	0.008	0.053	69	1228
AGCGGCACATGTA	0.021	0.004	0.015	23	1506
GGTAGCATATATA	0.074	0.002	0.075	105	1293
GCTGTTGCAGGGA	0.048	0.005	0.042	18	414
ACCGTCGTAGGCG	0.023	0.002	0.023	27	1128
GCCGTCGCTTAGG	0.063	0.012	0.047	24	492
GCCGGTGCTGAGG	0.11	0.012	0.114	63	490
CCTGGTGCAAACG	0.022	0.003	0.024	36	1443
AGTGTTGCATACA	0.02	0.006	0.014	21	1497
GCCATCACTAGTG	0.047	0.003	0.044	67	1444
GGTGTTGTATGGG	0.081	0.015	0.06	19	298
GCTATTGTTGACG	0.059	0.006	0.055	63	1080
AGTGGCGTTGACA	0.02	0.002	0.019	16	820
GGTGGCATAGAGA	0.479	0.066	0.468	478	544
CGCGGCACTGATG	0.024	0.003	0.021	21	966
AGTGTTGTAGAGG	0.024	0.003	0.025	17	675
GGTATCGTAAACG	0.151	0.024	0.119	136	1007
CCCGTTATAGGGG	0.051	0.006	0.043	43	951
AGTGTTACTAGGA	0.024	0.002	0.025	23	890
AGCAGCGCTTGTA	0.027	0.004	0.022	29	1304
CGTGTCGCTGATG	0.022	0.005	0.016	10	630
ACTGTTGCTAGGA	0.021	0.004	0.025	30	1177
CGTGTTGTTGACA	0.022	0.001	0.023	25	1043
GGTATCGTAAATA	0.134	0.01	0.139	264	1637
GGCATTGCTGGTA	0.052	0.016	0.036	32	860
AGTGGTGCATGTA	0.02	0.004	0.024	20	818
GGTGTCGCATGCA	0.083	0.014	0.081	58	656
AGTGGTACATAGG	0.027	0.006	0.026	19	708

CCTATTACAAGGA	0.025	0.002	0.024	59	2448
CCTAGTACATGGG	0.031	0.007	0.028	29	1015
GGCGGCACATGGA	0.134	0.022	0.118	100	745
GGCATTACATGTG	0.059	0.008	0.048	57	1136
CGTGTCGCTTGCA	0.027	0.002	0.03	23	735
ACTGTTGCTAAGG	0.025	0.005	0.021	25	1144
ACTGGCACTTGGG	0.021	0.002	0.023	15	641
GCCAGCGCAAGGG	0.056	0.012	0.042	32	733
CCTGTCATTTGGG	0.024	0.001	0.024	18	728
GGTGGTACTTACG	0.125	0.052	0.103	59	515
GGTAGCATTGGCA	0.066	0.02	0.06	42	657
CCTGGTACAGATG	0.023	0.004	0.023	28	1216
CGTAGCACAGACG	0.026	0.001	0.024	27	1093
GCTGGCACATGTG	0.447	0.042	0.437	355	458
GGCGGTATAGGCG	0.115	0.033	0.087	71	749
GCTGGTGTTTAGA	0.422	0.055	0.433	325	426
GCTGTCGCATGCG	0.049	0.005	0.043	23	517
CCCAGTGTTAACA	0.026	0.002	0.026	84	3161
GCTAGTGCAAAGA	0.053	0.008	0.056	73	1230
CGTATTGTAGGGA	0.027	0.009	0.027	21	771
GGCATCACTAAGG	0.089	0.01	0.086	115	1229
GGTGTCGTAGGGA	0.091	0.022	0.062	23	346
CCCGGTACAGAGA	0.026	0.005	0.025	50	1971
ACTGTCGTTTGGG	0.025	0.007	0.018	10	554
GGTGTCGTTGGGA	0.072	0.027	0.042	14	319
ACTAGCATATGGG	0.031	0.006	0.039	40	995
CCCGGTATTTGGA	0.029	0.002	0.031	36	1114
AGTAGTACAGGGA	0.028	0.001	0.03	29	933
AGCGGCGCTTGCA	0.024	0.007	0.031	34	1068
CCCGGCGTATGCA	0.022	0.003	0.018	35	1911
AGCGTCGTTTAGA	0.026	0.002	0.025	30	1164
GCTGTTGTATGTA	0.056	0.006	0.048	51	1002
AGTATCACTGGTG	0.033	0.007	0.022	17	739
CGTAGTACAGGTA	0.028	0.008	0.02	21	1038
GGCAGCACAAGCG	0.058	0.008	0.047	57	1168
CCTGGTGTTGGTG	0.028	0.01	0.019	12	631
GCCGTCGCATATG	0.042	0.006	0.049	38	745
GCCATCGCTGGCG	0.038	0.001	0.038	48	1218
GCCATCGTTGGGA	0.05	0.008	0.044	41	890
GCTAGTGCATGGA	0.067	0.014	0.086	74	785

GGTGTTGCTAACA	0.067	0.015	0.068	64	878
CCCGGCACTGGCG	0.036	0.007	0.026	33	1221
GCTAGTATTGGGG	0.068	0.004	0.065	33	478
AGTGTCATTGGGG	0.03	0.009	0.039	20	491
CCCGGTGCTGACG	0.026	0.001	0.026	33	1246
GCCATCGTTAGCG	0.052	0.005	0.047	56	1145
CCTAGTATTTACG	0.026	0.001	0.027	46	1682
GGTGTCACAAACG	0.099	0.015	0.083	76	837
CGTAGTATTGGCA	0.029	0.008	0.017	17	976
CCTGTCATTGGGA	0.028	0.005	0.021	20	912
CCCGTTGCTGAGG	0.028	0.003	0.032	33	1003
CGTAGCGTTGGTG	0.029	0.009	0.021	10	459
CCTGTTATATAGG	0.028	0.007	0.019	28	1436
GGTGGCATTTGGA	0.49	0.064	0.479	290	316
GCCGGTGTTTAGA	0.169	0.01	0.178	165	760
GGTGTTGCAGGTA	0.075	0.013	0.056	32	538
ACTGGTGCAGGTA	0.025	0.003	0.029	36	1188
GGTATTACAAACG	0.099	0.016	0.083	136	1494
ACCAGCACTTAGG	0.026	0.004	0.02	41	1985
AGCATCGCAAGTA	0.024	0.003	0.02	49	2444
CGCATCGCTTGTG	0.023	0.004	0.026	26	964
AGTAGCACATACG	0.029	0.006	0.022	30	1344
GCTAGCGCATGTA	0.052	0.005	0.058	48	780
GCTATCGCAGAGA	0.053	0.004	0.05	49	932
CGCGTCGCAGGTG	0.03	0.003	0.029	24	816
GCTGGCGTTAACA	0.885	0.024	0.889	1952	243
ACTAGCGCTTATA	0.023	0.002	0.022	51	2292
CGCGTTGTATGGG	0.031	0.005	0.024	15	620
GGCAGTACAGGTG	0.045	0.008	0.034	31	880
CCCGGTGTATGTG	0.025	0.006	0.018	19	1054
AGTGGCGCATGCG	0.02	0.007	0.018	9	483
AGCGGTGCTAGTG	0.014	0	0.014	11	801
AGTGTCATTAGCA	0.018	0.003	0.014	19	1342
GGCAGTGCAAAGG	0.041	0.003	0.037	35	919
CCCGTTGTTGGGA	0.025	0.002	0.027	24	877
CCCAGCACATGTG	0.028	0.003	0.027	51	1839
CCTGGCATATATA	0.024	0.004	0.029	67	2219
AGTGGCACTGATG	0.021	0.004	0.022	12	542
CGCATCGCTAGTA	0.027	0.006	0.025	39	1548
AGCGGTGTAGAGA	0.021	0.002	0.018	23	1234

AGCGTTGTTAGCG	0.022	0.007	0.015	16	1076
ACTGTTATTGAGA	0.03	0.004	0.027	49	1737
CGCATTATTGGGG	0.034	0.01	0.031	24	754
CGCATCGCTAGCG	0.023	0.001	0.025	31	1230
ACTGTCACTTGGA	0.026	0.006	0.034	45	1280
CGCAGCATAAGGG	0.043	0.007	0.051	55	1018
AGTGGCGCTGATG	0.027	0.008	0.017	10	577
AGTAGCACTTACA	0.026	0.004	0.024	41	1647
GGCATCGTTTATA	0.105	0.019	0.096	153	1445
CGCGTCGTAAGTA	0.028	0.002	0.031	49	1548
CGCAGTACAGATG	0.027	0.005	0.02	30	1462
AGCGGCATTAGTG	0.017	0.006	0.008	4	498
ACCGGCACTTGTG	0.023	0.007	0.018	24	1324
GGTGGCGTTTGCA	0.389	0.032	0.397	216	328
GCCAGCGTTAAGG	0.062	0.007	0.052	49	892
CGCATCACTTGTA	0.026	0.002	0.029	53	1799
CGTATTGCTTGTA	0.026	0.004	0.031	32	993
AGCAGTGCAGGTG	0.019	0.001	0.019	20	1035
GGCATCGTTAGTA	0.099	0.008	0.1	135	1210
GCCAGTACTTGCG	0.052	0.001	0.053	56	993
GCCAGTGCTTGCG	0.044	0.003	0.045	35	741
GCCGGTATTAGTG	0.162	0.003	0.159	170	898
CGTGGTATTTATA	0.028	0.003	0.027	32	1147
CCCGTTGCTTAGG	0.023	0.008	0.014	14	960
AGTGGCGTTAGTA	0.027	0.005	0.023	16	685
GGTAGTGCTTAGG	0.066	0.017	0.054	23	401
GGCAGTATTGATG	0.052	0.004	0.055	67	1143
CGTAGCGCTGATG	0.025	0.008	0.02	14	683
GCTAGTGCTTGTA	0.048	0.003	0.047	43	868
CGCGGCACTAGTG	0.019	0.002	0.016	13	815
GCTATTGTAGGCA	0.059	0.005	0.058	76	1244
CCCAGTGTTAGTG	0.021	0.008	0.011	16	1377
CGCGTCGTAAAGG	0.023	0	0.023	26	1127
GGCATTATTGGTG	0.057	0.003	0.058	57	925
AGCGGTACAAGTG	0.02	0.003	0.018	23	1275
CGTAGCATATGGG	0.021	0.011	0.012	8	671
GGTAGCGCTGATA	0.072	0.011	0.075	53	649
GGCAGTGCATATG	0.064	0.016	0.087	84	882
GGTGTTGCTAGCA	0.063	0.003	0.058	34	548
CCTGTCGTTTATA	0.023	0.002	0.022	36	1623

ACTAGTGCTGATG	0.024	0.003	0.021	34	1580
ACTGGTATAAGGA	0.028	0.001	0.03	45	1460
GGCAGCACAAAGA	0.05	0.002	0.05	87	1662
ACCGGCGTTGATA	0.026	0.006	0.022	34	1512
AGTAGTGCTTATG	0.025	0.008	0.021	17	809
GCTAGTACTGACG	0.054	0.005	0.048	52	1037
ACTGTTGCAAGGG	0.023	0.002	0.02	20	967
GCTGGTGCTTAGG	0.306	0.035	0.312	129	285
GCCGTTACTAGTA	0.052	0.002	0.055	80	1386
GCTGTTACTAGTG	0.056	0.007	0.046	34	699
AGCGGTGCAGGCG	0.02	0.003	0.018	17	911
GCCGTTGCATAGG	0.052	0.004	0.052	40	733
CCCGGTGTTGGTG	0.022	0.006	0.016	13	809
GGCAGTACATGGA	0.055	0.007	0.064	75	1091
GGTATCGTAAAGG	0.133	0.011	0.146	124	727
CGCAGTACTAGCG	0.023	0.002	0.026	30	1115
GGCAGCGTTAAGA	0.058	0.003	0.057	73	1212
CGCGGTATAGAGG	0.026	0.005	0.023	21	888
CCTAGCGTTTGGG	0.021	0.002	0.019	13	659
CGCGTCGTTTGTA	0.022	0.006	0.016	16	983
GGCAGCGCTAACA	0.06	0.012	0.047	77	1566
GGTGTTATTAGGG	0.078	0.011	0.08	40	461
GGCAGCGTTTGGG	0.059	0.007	0.057	28	459
GGTATCATTTGCG	0.138	0.004	0.143	105	627
CCTGGTGTATGCA	0.02	0.003	0.016	21	1253
CGCGGCATAGGGG	0.044	0.004	0.039	22	549
GCCGGCATAAGTA	0.396	0.039	0.375	779	1297
CGTATCGTAGGCG	0.026	0.011	0.016	16	965
CGTGGTGTAGGTG	0.025	0.001	0.027	11	400
CCTGGCACTGGGA	0.029	0.005	0.035	28	780
GCTGTTATTGACA	0.06	0.005	0.067	107	1492
CGCGGTACTTATG	0.032	0.007	0.023	24	1022
GCCGTCGTTGAGA	0.047	0.004	0.044	39	854
CCCAGTGTTAGGG	0.021	0.002	0.022	22	973
ACTGGTGCTGGTG	0.035	0.005	0.038	24	610
GGCGGCACTGGTG	0.26	0.057	0.211	147	549
GGTGGCGCAGAGA	0.15	0.033	0.156	75	407
ACTATCGCAAGGG	0.023	0.002	0.02	26	1248
ACTGGCGTAGGTG	0.025	0.004	0.023	9	377
AGCAGCATTTGTG	0.02	0.003	0.018	25	1346

ACTAGTACAGGTG	0.018	0.004	0.024	40	1636
CCCGTCATTTGGG	0.032	0.004	0.027	25	892
GGCGGTATTTGGG	0.128	0.02	0.113	52	410
ACCGTCGCAAGGG	0.022	0.008	0.014	16	1139
GGCGGCATAGGTG	0.471	0.062	0.437	435	560
GCTAGCGTTAAGG	0.077	0.016	0.072	49	629
GGTAGCGCATAGG	0.073	0.019	0.047	21	427
ACTGTTACTAGGG	0.026	0.005	0.02	18	900
AGTGGCGCTAAGA	0.026	0.003	0.026	21	784
GCCAGCGTAAACG	0.066	0.004	0.066	112	1575
GCTAGCATTGGTA	0.064	0.01	0.049	50	964
GCTGGCATAGGGA	0.475	0.044	0.449	366	450
GGTGGTGCAGGCA	0.083	0.01	0.081	39	442
GGCGGTATTGGTG	0.119	0.006	0.117	65	490
CGCATTATAGGTG	0.028	0.002	0.026	35	1336
GGCATCGCATGGG	0.098	0.019	0.085	64	685
GCTGTTATAAAGA	0.062	0.004	0.063	116	1731
AGTGGTGCTAGTG	0.022	0.002	0.025	13	503
CGCGGTGCATATG	0.027	0.002	0.026	26	966
GCTATCACTAGTG	0.06	0.01	0.047	47	952
ACTGTTGCATAGG	0.031	0.004	0.029	32	1086
GCCGTTACAGACG	0.059	0.008	0.049	72	1398
GGCGGCGTTGGGG	0.376	0.055	0.377	155	256
CCTGGCACAAGGG	0.029	0.01	0.022	19	834
AGTAGCATTAGTG	0.024	0.003	0.021	21	995
GCTGTCGTAGAGA	0.059	0.011	0.059	48	765
GGTATTACTAGTA	0.09	0.018	0.072	94	1208
CGCGTTATTTGCG	0.02	0.005	0.016	16	1014
GCCAGTACAGGGA	0.055	0.011	0.05	61	1169
CGTGTTGTATATG	0.023	0.004	0.028	23	786
CGTAGCATTAGCG	0.028	0.003	0.026	23	862
GGTGGCACAAGTA	0.175	0.018	0.176	171	799
GCTGTCACTAGGA	0.055	0.001	0.055	39	666
ACCGTTGCTAAGG	0.022	0.003	0.019	30	1564
CGCGGCGTTAGGA	0.023	0.009	0.014	11	759
CGTAGTATTTGTA	0.023	0.005	0.025	25	975
AGTAGCGTATAGA	0.024	0.002	0.027	31	1128
GCCGGTGTTTGGG	0.375	0.061	0.341	168	325
ACTAGTGCAAATG	0.024	0.004	0.025	64	2460
GCCGGCGCAGAGA	0.488	0.048	0.507	592	576

GCCAGCGTTTGGG	0.052	0.005	0.047	28	565
ACTGGCACTTATG	0.029	0.005	0.033	40	1166
GGCAGCGTAGGTG	0.061	0.01	0.055	40	688
CGCGGCATTGGTG	0.028	0.003	0.027	17	603
CGCATCGCAAGGG	0.028	0.011	0.014	15	1078
AGTGTTATTTACG	0.021	0.005	0.024	28	1140
CGCGGTGTATACG	0.022	0.005	0.019	20	1043
ACTGGTGTTGGGG	0.035	0.007	0.043	20	447
GGTAGTATTGGCG	0.054	0.007	0.046	27	563
CCTATTGCTGAGA	0.025	0.002	0.023	40	1673
GCTATCGCATAGG	0.059	0.006	0.068	54	746
ACCGTTATTAGGG	0.026	0.002	0.028	34	1180
GCTGTCGCTTGTG	0.076	0.01	0.08	30	346
GCTGGCGCTGACG	0.813	0.037	0.789	836	223
GGTATCATAGATG	0.138	0.017	0.132	125	824
GCTATTGTAAAGG	0.06	0.008	0.051	76	1423
GCCAGTGCTGGTG	0.051	0.007	0.043	27	601
CGTAGCGTAGGCG	0.019	0.006	0.024	18	735
CCCGTTATTAGCG	0.028	0.004	0.025	43	1700
CGTGTCGCATAGG	0.033	0.007	0.039	23	566
ACTATTATTGGTG	0.027	0.002	0.03	48	1542
CCCGGTACAAATG	0.024	0.003	0.027	69	2517
GGTGGCACTGGCG	0.377	0.028	0.377	181	299
AGTAGTGCAGAGG	0.023	0.003	0.02	13	645
AGTGTTGTTAGCG	0.027	0.001	0.028	19	662
CGTGTCGTAGGTG	0.021	0.003	0.019	10	507
CGTAGTACTAACG	0.024	0.001	0.024	29	1170
CCCGGTACTTGGA	0.024	0.003	0.027	35	1260
GGTAGTATATAGG	0.064	0.019	0.048	35	693
AGTGGTGCTGATA	0.029	0.005	0.029	27	909
CCTAGCGCTGGTG	0.023	0.002	0.024	19	760
ACTGGCACTAGTG	0.025	0.002	0.025	25	973
GCTAGCGCTAGGA	0.06	0.01	0.05	28	527
CCCGTTGTAGGTG	0.031	0.007	0.022	25	1117
CCTGGTGCTTACG	0.032	0.005	0.027	24	858
GCTATTATAAGTG	0.059	0.005	0.052	87	1600
AGTATTGTTTGCG	0.028	0.011	0.016	15	910
AGCGGCATAGGCA	0.024	0.001	0.025	31	1196
CCTGTTGCATGGA	0.029	0.002	0.027	29	1053
ACTGTTATTTAGA	0.027	0.003	0.024	46	1865

AGTGGTGCAGACG	0.031	0.009	0.019	14	737
ACCAGCGTAAGTG	0.022	0.002	0.023	37	1607
GGTAGTATTAGGA	0.054	0.005	0.056	43	731
GGCATCGCTAATG	0.106	0.022	0.083	112	1237
CGCAGTATAGGTG	0.026	0.002	0.025	28	1080
AGCGGTGCAAGCA	0.02	0.004	0.017	28	1605
ACTGGTGTAGGGA	0.025	0.007	0.034	28	789
AGTAGTACTGGCG	0.022	0.005	0.02	18	877
GGCGGCATTTAGG	0.56	0.06	0.559	514	405
CGCGTCGCAAGGG	0.025	0.002	0.023	20	851
GCCGTTACTGGGG	0.058	0.004	0.062	37	558
GCCATTGTTGAGA	0.046	0.004	0.045	60	1269
CGCAGTATATGTA	0.03	0.008	0.019	42	2213
AGCGGCATTTACG	0.024	0.007	0.034	22	629
CCCGGTACAAGGG	0.025	0.007	0.016	20	1231
CCTATTACTTGCG	0.03	0.002	0.032	48	1456
ACTGGTGCAGAGG	0.033	0.006	0.032	25	749
CCTAGCGCAGGGA	0.025	0.004	0.021	23	1073
GCCGGCGTTGGTG	0.922	0.015	0.926	870	70
CCTGTTACATGCA	0.023	0.004	0.02	38	1834
GCTGGCGCTTAGA	0.619	0.027	0.617	593	368
GGCATCGCAGATG	0.11	0.028	0.08	93	1075
AGTAGCACAAATG	0.025	0.004	0.019	32	1673
CGCGTCGTTGGTA	0.021	0.004	0.02	20	976
CGTGTCATATATA	0.025	0.002	0.022	35	1557
GCTGTCGCAGATA	0.057	0.005	0.051	45	842
CGCGGCGCAAGGG	0.023	0.007	0.015	11	745
GGCAGTGTTTATG	0.061	0.009	0.048	45	891
GCCGGTGCATGGG	0.132	0.049	0.11	50	404
AGCGGTATTGGTG	0.024	0.007	0.019	14	737
AGTATCACTAATA	0.024	0.002	0.023	51	2206
AGCGTTGTTGGCG	0.014	0.001	0.015	17	1084
GGTATTATATGCA	0.11	0.013	0.092	145	1435
GGTAGCACAGGCA	0.072	0.007	0.078	63	742
GGTGTTGTTGATG	0.093	0.009	0.08	42	480
CCTGGTACTTATA	0.028	0.007	0.026	46	1708
GGTATTACTAGGA	0.089	0.025	0.069	63	850
AGTAGCGCAGAGG	0.016	0.005	0.009	6	657
CCCAGTATTAGGG	0.03	0.002	0.032	42	1287
GCTAGTATTTGCA	0.057	0.007	0.067	97	1354

GCTGTCATTAGTA	0.057	0.01	0.05	55	1053
AGTGTTGCTGGTG	0.023	0.003	0.019	10	507
GGTGTCATTTGCA	0.098	0.013	0.089	67	684
CGTGTTGTTAAGA	0.025	0.008	0.016	16	981
GCCGGCATAGGGA	0.208	0.032	0.189	166	713
GCTGTCGTTTATG	0.074	0.007	0.083	53	584
CGTAGTGTTAAGG	0.03	0.012	0.034	23	654
GGTAGTGCTAGTA	0.051	0.013	0.037	24	633
AGTAGTATTTAGA	0.018	0.002	0.016	25	1526
CCTGTTATAAGGG	0.031	0.005	0.036	40	1077
AGTGTCGTTTATA	0.021	0.002	0.02	24	1176
GCTAGCACTGGGG	0.066	0.012	0.071	32	421
GGCAGCGCATGCG	0.061	0.004	0.063	50	744
ACTGGCGCAAGTG	0.022	0.003	0.018	16	879
GGCGGTATATGGA	0.079	0.009	0.076	70	847
CCTGGTGTTAGGG	0.027	0.006	0.02	13	636
CGTGGTACAGGCG	0.025	0.007	0.02	11	552
GCCGGTGCATACG	0.117	0.015	0.104	109	935
GCCGGCACTGGTG	0.225	0.012	0.21	175	658
GGCGTTATAAGGG	0.07	0.001	0.07	59	778
GCCATCATTGACG	0.062	0.005	0.058	105	1721
CGTATTACTAGTA	0.025	0.002	0.028	45	1564
GCTATCGTAAGGA	0.062	0.01	0.057	63	1040
GCTAGCGCAAGCG	0.061	0.005	0.055	39	668
AGTAGCACTAGGA	0.025	0	0.025	26	1003
GCTGTTGCAAGCA	0.059	0.001	0.06	66	1041
GGCGTCACAGGTA	0.068	0.009	0.057	67	1110
CGTATCACATGGA	0.015	0.005	0.009	9	1029
GCCGGCATTAACG	0.743	0.032	0.749	1590	534
AGTGGCGTTTGGA	0.029	0.004	0.025	10	393
GGTGTCGCAAGGG	0.088	0.007	0.092	34	336
GGCATTGTTAGTG	0.085	0.015	0.07	63	836
ACTGTCATAAGCG	0.026	0.001	0.026	42	1568
GGTGGTGTATGTG	0.194	0.068	0.143	58	347
GCTGGTGCAAGCA	0.112	0.01	0.126	115	798
AGTAGTATTAAGG	0.023	0.006	0.015	20	1307
CGCGGTGTAAAGG	0.021	0.007	0.018	17	915
GGCATTGCTGATA	0.052	0.006	0.051	68	1255
ACCGTTGTAGGTG	0.028	0.003	0.024	26	1060
GGTATCACAAACG	0.128	0.013	0.118	144	1081

CCTGTTGTTAGCA	0.027	0.002	0.024	35	1420
AGTGGCGCAAACG	0.031	0.002	0.034	28	798
AGCAGTGTTGACG	0.023	0.004	0.025	37	1467
CCTAGTGCTAGGG	0.026	0.003	0.022	19	846
GGTGTTATTAGCA	0.075	0.018	0.062	57	861
GGTATTATTGGGG	0.098	0.025	0.063	34	503
AGTGTCGCTGAGG	0.013	0.007	0.023	13	551
CGTGGCACAGAGA	0.027	0.004	0.02	17	815
CGCAGCACAGGGG	0.034	0.003	0.033	25	728
CCCGGCGTTTGGA	0.025	0.003	0.021	21	959
AGCGTCATATAGG	0.024	0.001	0.024	33	1343
ACTGTTACAGACG	0.025	0.006	0.017	32	1897
CGCATTGCTAGCG	0.027	0.002	0.029	35	1179
GGTAGTATAGATG	0.058	0.009	0.047	39	795
ACCGTCACTGGCG	0.032	0.003	0.036	52	1404
ACTGGCGCTAATG	0.021	0.004	0.021	19	882
ACCGGTACATGTG	0.02	0.002	0.018	35	1959
AGTGTCACTGGCG	0.016	0.001	0.017	12	677
GGCAGTGTTGGCA	0.054	0.011	0.039	40	989
CGTGTTGCTTAGA	0.03	0.011	0.033	25	734
CGTGTCACATAGG	0.023	0.006	0.014	11	756
GGCATTGTATGCA	0.07	0.005	0.064	90	1320
AGTGTCGTTTATG	0.02	0.003	0.024	18	722
CGCGGTACATAGG	0.022	0.001	0.021	20	925
GCCGGTACAGAGA	0.048	0.005	0.041	52	1209
CGTAGTGCAAGTA	0.025	0.006	0.03	31	1004
GGTAGTGTATATG	0.06	0.003	0.057	42	701
GCCATTGCAGATA	0.047	0.007	0.052	101	1854
GGTAGCGCTTGCA	0.07	0.012	0.057	34	559
ACTGGCGTTAAGA	0.031	0.005	0.027	29	1055
GGTATCGCAGGTA	0.098	0.027	0.08	62	712
GGTGGTATTTGTG	0.254	0.05	0.248	129	392
GGTGGTATTAGTA	0.124	0.014	0.112	86	679
GCTGTCGTTAATG	0.06	0.018	0.086	75	802
GCTGTCACTGGCA	0.055	0.008	0.051	48	890
CGTGGCACTTGTA	0.034	0.009	0.026	19	714
GCTAGTGCATACG	0.055	0.009	0.044	48	1046
ACTGTTACATGGG	0.025	0.003	0.026	26	992
AGTATTGCTGGTG	0.022	0.003	0.024	16	662
AGTATCGCTGGTG	0.024	0.007	0.014	9	622

CGTGGCATTGGTA	0.03	0.003	0.026	16	589
CGCATTGCAGAGA	0.022	0.007	0.029	43	1421
CCTGGTGTAAAGG	0.035	0.009	0.038	43	1093
CCTAGCATTAGCA	0.028	0.002	0.028	57	2005
GGTGGCATAAGCG	0.706	0.044	0.704	801	337
CCTAGCACAGGGA	0.038	0.01	0.052	65	1182
GCTGTCGTTGACA	0.059	0.01	0.057	60	993
CGCAGCGTTGAGA	0.025	0.008	0.014	16	1102
GCTGGCATAGACA	0.654	0.037	0.658	1394	723
GCTAGCGCTGAGA	0.057	0.02	0.043	30	665
GGCGGCACTTGTG	0.266	0.039	0.266	177	489
AGTGTCGCATGGA	0.025	0.006	0.018	11	617
GGTAGCGTTGGTA	0.088	0.005	0.09	42	426
GCTATCGTTGGGG	0.07	0.013	0.063	30	449
CCTAGCACTTGGA	0.029	0.006	0.02	25	1195
GGCGGCACTAATA	0.145	0.039	0.112	183	1445
ACTGTTACTGGCA	0.021	0.004	0.021	36	1695
GCCGTCACTGAGA	0.055	0.004	0.056	73	1239
AGTATTGCTAAGG	0.031	0.006	0.038	40	1016
CGCGTCGTAGGCA	0.03	0.005	0.024	30	1222
AGTGGCATATGCG	0.022	0.002	0.022	6	268
GGTATCACAGATG	0.129	0.018	0.131	120	798
GCTGGCATATATA	0.654	0.038	0.652	1464	781
GCCGGTGTTGGGA	0.166	0.031	0.203	120	472
GCTGTCATAGATG	0.056	0.004	0.053	56	992
AGTGTCGCAAAGA	0.029	0.004	0.033	39	1133
AGTATTGTTGGTA	0.023	0.004	0.02	23	1112
AGCAGCGTATGGA	0.021	0.005	0.016	21	1311
GGCGGCGTATGCG	0.443	0.079	0.428	347	463
GCTGTCGTAAGCG	0.052	0.003	0.055	39	671
GGTGGTGCAGATA	0.062	0.017	0.043	30	672
CGTATTATAGGCG	0.025	0.003	0.029	36	1193
CGTGTCATTGGTG	0.018	0.004	0.019	12	625
CGCGGTGCTAAGG	0.025	0.002	0.022	16	712
CGCGGTATATGCG	0.021	0.002	0.021	24	1110
GCTATTGTTTGGA	0.062	0.011	0.063	56	835
CGTAGCACAGGTA	0.028	0.004	0.027	27	970
CCTGGCGTATAGG	0.014	0.004	0.019	15	761
CGCGGCGTATGCG	0.03	0.002	0.034	27	772
GCCGTCATAGGCG	0.057	0.004	0.063	68	1008

CGTAGCGCTTGGG	0.029	0.004	0.03	10	323
CGCGGTGTAAAGA	0.023	0.003	0.024	35	1423
ACTGTTGCTTGGA	0.029	0.01	0.015	16	1019
CGCGGTGCAAAGG	0.026	0.006	0.019	18	914
CGCGGCGTAGATG	0.028	0.004	0.027	23	832
CGTATTATTGATG	0.022	0.001	0.024	36	1476
GCCGGCATATAGG	0.725	0.043	0.723	1095	420
GGTGGTATTAACG	0.284	0.046	0.257	224	647
CCTAGTGCAAGTG	0.028	0.005	0.02	30	1443
AGTAGCATTGATG	0.025	0.011	0.017	20	1187
AGCGTTACAGGGA	0.025	0.002	0.024	30	1203
CCTATCGCAGGTA	0.029	0.002	0.026	43	1633
GCCAGCGTTAGCG	0.07	0.005	0.063	57	847
AGTGTTATAGGTG	0.03	0.001	0.028	24	831
AGCGTTGCAGGGG	0.022	0.007	0.022	16	702
GCTGGCACAGGGG	0.444	0.051	0.473	211	235
CCCAGCGTAAGGG	0.031	0.002	0.03	40	1286
AGTGTTGTTTATA	0.024	0.002	0.021	27	1232
GGTGTTACAGGTA	0.075	0.024	0.047	37	742
GGCGGCATTAACA	0.305	0.039	0.287	534	1324
GGTGTTATATATA	0.086	0.02	0.064	85	1253
AGCATCACTGGTG	0.03	0.004	0.025	37	1449
ACTGGCGCAGGGG	0.028	0.004	0.023	11	477
GGCGGCGTTGATA	0.198	0.026	0.175	173	814
GCCGGCACAGGGG	0.197	0.015	0.188	114	493
CGCAGCATATAGG	0.021	0	0.021	27	1279
AGTGGCGCAAGGA	0.022	0.001	0.021	14	659
GGCAGTGTTGAGG	0.056	0.005	0.049	34	657
AGCATTGCTGGGG	0.018	0.005	0.012	11	886
AGTGTTACAGGGG	0.02	0.003	0.016	10	600
ACTGGTATTTACG	0.025	0.003	0.021	28	1326
ACTGTCACTGGGA	0.025	0.006	0.02	20	987
GGTAGCGCTGAGG	0.07	0.015	0.06	22	344
CGTGTTACTAAGG	0.019	0.006	0.013	11	823
CGTGTTACTTATG	0.023	0.007	0.014	12	867
CGCGGCGCTTGTG	0.025	0.005	0.033	21	613
AGTGGCGTATGCA	0.024	0.004	0.026	17	645
GCTGTTGTAGGTA	0.058	0.005	0.053	43	769
GCTGTTGTTTGTG	0.056	0.011	0.047	24	488
CGTGTCGCATGGG	0.034	0.006	0.025	12	467

CGCGTCATAAGTA	0.023	0.004	0.028	56	1956
ACTGGTGTTGGGA	0.022	0.003	0.026	16	609
GGCGTCACAAGGG	0.071	0.015	0.065	54	780
CGTGTCATTAGGG	0.032	0.003	0.029	16	534
GGTATTGCTTGGA	0.085	0.012	0.085	47	506
AGTGGCATATACG	0.021	0.007	0.015	6	383
CCTGTCGCTTGGA	0.024	0.005	0.031	27	837
GCTGGTATTTAGA	0.16	0.017	0.158	154	818
GCTAGCGCAGACG	0.063	0.002	0.063	53	791
GGCAGTGCATGTA	0.051	0.009	0.043	43	954
GCTGGTGTTAACA	0.431	0.027	0.452	677	820
GCCGGTACTAGGG	0.063	0.013	0.044	30	651
GGCATCGTAAGTG	0.116	0.029	0.09	104	1058
CCTGGTGTTTGGA	0.018	0.008	0.007	5	694
ACTGGCGTAAGTA	0.029	0.003	0.026	32	1207
GGTGTTGCAGGTG	0.086	0.013	0.087	28	295
GCCGTTACAAGCA	0.054	0.006	0.059	112	1801
ACTGGTATATAGA	0.025	0.005	0.028	49	1724
GCTAGTGCAAGCG	0.049	0.004	0.052	50	908
GCCGTCGCTAGGG	0.048	0.013	0.03	15	493
GGTAGTGTTGATA	0.061	0.005	0.063	54	800
GGCGTCATTTGCA	0.071	0.02	0.046	57	1187
GGCAGCGCTTATG	0.056	0.005	0.057	51	848
AGTGGTACTTGCG	0.022	0.008	0.011	6	527
CCTGTCACTGATG	0.026	0.005	0.032	41	1259
AGCGGCGCATACG	0.017	0	0.017	19	1077
CGTGTTGTATACG	0.023	0.006	0.014	13	939
AGTGGTGCAGACA	0.021	0.006	0.028	32	1097
AGTAGTATAGAGG	0.025	0.01	0.024	28	1127
GCCGGCGTTTATA	0.851	0.042	0.845	1552	284
CGCGGCATTAGCG	0.031	0.009	0.026	20	738
CGCGTTGCTAGGG	0.027	0.005	0.032	19	583
GGTGTCGCATATA	0.081	0.026	0.051	44	812
GGCGGCGTATATA	0.227	0.039	0.203	243	955
ACCGGCGCAGGTG	0.029	0.005	0.036	33	888
GGTATCGTTGAGG	0.146	0.009	0.141	83	506
GCTATTACAGGGA	0.059	0.014	0.044	46	1011
CCCATTGTTGGGA	0.022	0	0.023	31	1330
GGTAGTGCAAGCG	0.063	0.017	0.044	27	580
GGCGGCATATATG	0.546	0.045	0.512	809	771

CGTGGCGTATACG	0.023	0.005	0.017	11	624
GGTGTCGCTAATG	0.107	0.012	0.107	63	524
CCCGGTGTTTGGG	0.021	0.009	0.009	6	661
GCTGGCATTGGGG	0.796	0.041	0.788	487	131
GGCGGTATTAACG	0.116	0.023	0.113	144	1125
CCTATCATTGAGG	0.027	0.001	0.027	43	1522
GCTAGTGCTGAGA	0.045	0.002	0.045	39	825
GCTGGTGCTAGTG	0.272	0.019	0.255	131	382
ACTGTTATAAGCA	0.025	0.001	0.027	68	2495
AGCGGTACATGTG	0.019	0.004	0.022	23	1033
CCTGGTACAGGCG	0.03	0.008	0.019	18	947
GGTGTTACTAGTA	0.07	0.01	0.058	47	762
CGTGGTATAAGGG	0.024	0.001	0.024	13	533
ACTGTCACTGACG	0.031	0.005	0.027	41	1477
GGTGTCGTTTGGG	0.088	0.014	0.078	20	238
AGTAGCACAGGGA	0.021	0.006	0.013	12	882
AGTAGTGCTAGCG	0.02	0.006	0.015	11	724
AGCGGCATTTGGG	0.02	0.013	0.003	1	334
CGCATCGCAGGCG	0.026	0.007	0.02	30	1468
GCTGGTATAAGTG	0.439	0.046	0.43	460	610
GCTATCGCTAATG	0.057	0.002	0.055	57	980
GGTATTGCTTAGA	0.081	0.005	0.075	60	744
CGTGGTGCTAATA	0.021	0.003	0.018	19	1053
CCTAGTGTATATG	0.023	0.001	0.023	38	1639
GGTATCGTAGACG	0.158	0.048	0.118	102	760
GGTGTTGTAAGGA	0.093	0.019	0.076	46	557
GGTAGTGCTTAGA	0.057	0.018	0.035	20	553
AGCGGCATTTAGG	0.018	0.007	0.009	4	429
AGTGGCATTGAGG	0.021	0.007	0.027	6	215
GGCGTTGCAGAGA	0.058	0.01	0.05	46	866
AGTAGTACTGGGG	0.026	0.003	0.028	16	559
GCTAGCATTAAGA	0.06	0.005	0.056	90	1504
CGCATCGTTGAGG	0.022	0.005	0.02	21	1040
CGTATTATAAAGG	0.025	0.005	0.018	35	1878
CGTAGCGTTAGGA	0.027	0.008	0.027	19	673
CCTATCGTTTGTG	0.028	0.004	0.024	28	1135
GGCGGCACTAGGA	0.128	0.022	0.106	84	708
AGTAGTGTAGAGG	0.023	0.008	0.012	9	765
CCTGGCATTAAGG	0.022	0.004	0.018	19	1061
CGTGGTGCTTAGG	0.023	0.008	0.024	9	368

CGTGGCGTTAGCA	0.029	0.006	0.022	15	661
AGTGTTGCAGGTG	0.022	0.002	0.024	14	575
CGCGGTGCTGATA	0.026	0.003	0.026	31	1157
GGTGTTGCTGATA	0.076	0.007	0.067	48	672
GGTGTCATTTAGA	0.103	0.024	0.077	52	625
GCCAGTGTTAAGG	0.046	0.014	0.05	53	999
GGTGTCACAGATG	0.093	0.011	0.098	64	590
ACTGGTACAAGGG	0.028	0.006	0.035	37	1016
CGTATCACTAGCA	0.029	0.001	0.027	40	1424
AGTGGTATAAACG	0.023	0.002	0.02	26	1246
GGTATCACAAAGG	0.129	0.017	0.118	118	885
GCTGTTGCAGGGG	0.054	0.003	0.057	24	396
AGTGGCGCTGGCA	0.02	0.001	0.02	13	651
GCTGTTGCTGGTG	0.048	0.015	0.069	30	404
GCTAGCGCATGCG	0.047	0.008	0.052	32	587
CGTAGCATTTGTG	0.028	0.003	0.03	21	685
GCCAGCGTAGGGA	0.058	0.005	0.06	47	739
GGCGTCATATACG	0.062	0.012	0.052	68	1243
GCCGGCATAGAGG	0.641	0.024	0.645	764	420
GCCAGCGCAGACG	0.053	0.005	0.047	51	1043
GGTGGCACTAGGG	0.398	0.084	0.352	162	298
CGTATTACTTACG	0.024	0.003	0.02	29	1418
CGTAGTGCTAGGG	0.023	0.007	0.015	6	383
ACCGGTGTATGGA	0.023	0.003	0.024	30	1202
ACCATTGCATGTG	0.024	0.003	0.022	47	2066
GCTGTTGTAAATG	0.07	0.009	0.065	80	1157
GGTATCGTTAGCA	0.134	0.019	0.112	105	832
CCCATCGTAGGCG	0.022	0.005	0.027	50	1793
AGTGTCGTTAAGG	0.021	0.007	0.025	21	824
CCTGTTATTTGGA	0.028	0.012	0.027	30	1072
CCTAGCGTTAGTA	0.018	0.004	0.017	27	1570
GGTGTTGCTAGGA	0.067	0.002	0.064	33	481
GCTGGTGTAGGCA	0.388	0.036	0.388	384	605
AGTGGTATTAAGG	0.022	0.007	0.013	9	709
CGCATCATTAGGA	0.022	0.004	0.023	41	1730
CCTGTCGCAGGGG	0.025	0.01	0.012	7	588
GGTGTTATATAGA	0.078	0.002	0.077	81	965
AGCGGTGTAGGCG	0.021	0.008	0.02	19	909
GGTGTTGCATGTA	0.071	0.012	0.056	37	626
AGTGGCATTTGTG	0.023	0.001	0.023	5	216

CGTGGTATTAACA	0.027	0.001	0.025	38	1488
CGTAGTGTTTGGA	0.032	0.005	0.035	19	525
ACCGTCATTGGGA	0.021	0.005	0.029	45	1513
CGTGTTGTAGATA	0.024	0.004	0.022	27	1180
GGTATTACATGGA	0.088	0.007	0.078	74	873
GGTGGTACAAGTA	0.077	0.002	0.074	72	903
CGCAGTGTTAGTA	0.023	0.003	0.028	38	1323
CGTAGCGTTTGCG	0.03	0.011	0.042	23	527
CGTATCGTAAATG	0.03	0.004	0.026	42	1574
GGTAGTGTATAGG	0.071	0.022	0.046	24	503
ACTAGTGCTGGGG	0.027	0.002	0.024	19	762
GGCGTCGTTAGTA	0.065	0.006	0.064	58	843
GGCGTTACTTGCA	0.06	0.013	0.042	45	1030
CGCAGCGCTTAGA	0.029	0.004	0.023	26	1124
GGTATCACTGAGA	0.095	0.015	0.083	72	799
AGCGGCGCTTACG	0.022	0.003	0.019	19	1003
GGCGGTACATAGG	0.075	0.009	0.073	50	636
CGCAGTGCAAGGG	0.021	0.003	0.017	14	816
AGTGTCATTTGTA	0.029	0.01	0.02	22	1056
CCCGTCGCTGGCG	0.025	0.002	0.023	28	1177
CGTGGTGTATGGA	0.028	0.008	0.031	17	532
AGTGGCACTTGCG	0.017	0.003	0.019	8	416
CCTAGTGTTAGGG	0.026	0.004	0.021	21	973
GGTGGCACAAACA	0.192	0.03	0.186	286	1250
CGCAGTGCATGTG	0.027	0	0.027	25	906
CGCAGCGCTAGTG	0.021	0.006	0.028	26	888
GGTGGTACATACG	0.094	0.01	0.096	73	688
GCCGGCGCTAACG	0.748	0.029	0.73	1140	422
GGCGGTGTAGGCG	0.081	0.001	0.079	47	548
CCTGGCGCTGAGG	0.031	0.008	0.024	15	621
GGCAGCGCTGGGA	0.056	0.007	0.063	36	539
GCCGTTATTTAGA	0.054	0.006	0.047	69	1407
GGTGTCATTGGTA	0.093	0.007	0.084	49	536
AGTGGTACAAGGA	0.022	0.004	0.026	27	997
CGTGGTACTTGTG	0.026	0.01	0.012	6	514
CGTGTCGTTGGGG	0.03	0.011	0.045	16	342
GGTGTCGCTGGTG	0.096	0.007	0.086	28	299
AGCGTCGCAAACG	0.027	0.002	0.026	50	1878
CGTGGTGCTAAGG	0.026	0.006	0.022	11	480
CCCGTCGCATAGG	0.025	0.003	0.024	30	1221

GCTGGCATTTATA	0.648	0.043	0.641	1187	665
CGCAGCATAGGGG	0.044	0.007	0.036	30	815
GCCAGTGCTTACA	0.045	0.002	0.042	69	1561
ACCGTTGCTTGGA	0.02	0.001	0.019	25	1265
GGTGGTATTGAGA	0.125	0.011	0.118	83	618
GCTATCGCTTACG	0.051	0.008	0.062	58	883
GGTGTCATAGGTG	0.091	0.008	0.086	44	469
AGCGTCGCTGGCG	0.027	0.003	0.027	29	1032
GGTGGTACTGATG	0.106	0.031	0.084	45	493
GGTAGTGTTTGTG	0.052	0.009	0.064	27	396
GGTGGTGCATGCG	0.116	0.027	0.098	42	385
GGCAGTATTTACG	0.053	0.006	0.048	66	1311
ACTGTTGTAGATA	0.032	0.001	0.032	64	1959
GCCATCGCTAGCG	0.055	0.005	0.053	56	1010
ACTGGCATAAAGG	0.03	0.004	0.026	32	1218
GCCGGCACTGACG	0.198	0.017	0.191	239	1011
GCCAGCGTTTAGA	0.047	0.007	0.039	45	1106
GGCGGCGTTTGCG	0.461	0.017	0.458	286	338
AGTGTCGCTAGGA	0.021	0.006	0.016	11	667
GCCGGCGTATGCA	0.848	0.031	0.858	1558	258
GCCAGCGCATATG	0.049	0.013	0.033	35	1018
GGCGTTGTAGGTA	0.061	0.007	0.055	49	837
CGTGGTGTTTACA	0.03	0.005	0.022	19	831
GGCATCACAGGGG	0.073	0.009	0.061	64	988
AGCATCGTAGGTG	0.022	0.004	0.016	23	1400
GGCGTCGTAAACG	0.075	0.015	0.072	92	1185
CGTATCGCTGGCA	0.024	0.005	0.028	27	946
AGTGTCGCAAACG	0.022	0.007	0.018	22	1198
CGTGGTGCAAGGG	0.028	0.008	0.032	15	448
GCTGGTGCTTAGA	0.121	0.016	0.13	75	503
CGCGGTGCTTAGG	0.023	0.004	0.021	13	608
GCTAGCGCTGGGG	0.05	0.016	0.049	16	311
ACCGGCGTTTGGA	0.022	0.006	0.013	11	831
CCTGTCGTAGGGA	0.028	0.001	0.026	22	826
GGCAGTGCATGCG	0.05	0.007	0.046	37	763
CCCAGCATTGGGG	0.038	0.005	0.035	36	983
ACTGTTGTTGGTG	0.031	0.008	0.028	24	836
GGTGGCACTTACG	0.391	0.036	0.364	264	462
CGCATCGTTAGGG	0.026	0.004	0.021	18	859
CGTGGCGCAAGCA	0.026	0.003	0.022	20	888

CCCATTGCAGGGG	0.029	0.008	0.027	31	1137
GCCGGCACAGATG	0.193	0.04	0.19	238	1014
GCTGGCGCAGATG	0.81	0.05	0.793	857	224
ACTGGCGCAGAGA	0.032	0.004	0.028	19	670
AGTAGTACAAAGG	0.025	0.004	0.026	36	1348
ACCGGCGCAGAGA	0.024	0.004	0.029	30	1004
GGTAGTGTATACG	0.062	0.004	0.057	42	696
CCCGTCATTGGGA	0.03	0.003	0.03	39	1250
CGCGGCGCTTATA	0.02	0.001	0.021	27	1240
GGTGTCGCAGGGA	0.071	0.005	0.066	28	395
GCTAGCGCAGGTA	0.063	0.004	0.062	44	669
CGCGGTGTATGTG	0.02	0.003	0.024	17	706
AGCGGCATAAGCG	0.019	0.007	0.02	14	688
GCCGTCATTGGCG	0.06	0.004	0.063	66	978
AGTAGTACTTGGG	0.02	0.003	0.019	12	616
ACTGGTACTAGGG	0.029	0.004	0.024	22	882
GGCATCGCAAGTG	0.098	0.015	0.084	90	982
GGCATTGCATACG	0.077	0.021	0.077	110	1325
GCCGTTGTAAGGG	0.057	0.006	0.054	41	725
CGTGGCGTTAACA	0.024	0.004	0.024	24	968
GCTATCGTTTAGG	0.055	0.01	0.053	43	774
ACCATCATAAGGG	0.031	0.002	0.033	71	2057
CGTATCGTAGGCA	0.023	0.006	0.024	29	1170
GCCGTCGCAGGTA	0.043	0.002	0.04	32	760
CGCGTTGTATAGG	0.029	0.007	0.023	20	859
AGTGTCGCTGGGG	0.02	0.009	0.025	9	346
GGTAGCATTAGGA	0.065	0.016	0.048	33	661
CGTAGCGCTGGCG	0.021	0.005	0.028	20	685
ACTGTCGCTGGGG	0.025	0.004	0.024	15	598
GGCGTTGCATACG	0.07	0.008	0.073	68	868
CGTGTCGCTTACG	0.034	0.014	0.019	15	782
AGTGGTGTTAGGG	0.024	0.003	0.028	11	388
GGCGGTGTAAAGG	0.085	0.008	0.086	70	747
GCTGTTATTGGGG	0.042	0.006	0.034	15	428
CCCGGCATATGGG	0.031	0.006	0.023	23	992
CGTAGTATAAGGA	0.021	0.004	0.026	32	1190
AGTGGTACAGACG	0.026	0.01	0.024	22	900
AGTGGCATAGGGA	0.023	0.002	0.023	12	521
AGCAGTGCAGGGG	0.021	0.002	0.023	18	778
AGTGTTGTTTGCA	0.022	0.004	0.016	15	895

GGTGTTACTGGTG	0.085	0.027	0.098	42	387
GGCAGTGCAGGGA	0.052	0.008	0.061	38	587
GCTGGCGCATACG	0.813	0.04	0.803	1052	258
GCTGTTACATATG	0.064	0.007	0.067	68	946
CGTAGTGCTGAGG	0.025	0.004	0.028	14	481
CCTAGCGTAAGGG	0.026	0.005	0.028	25	874
CGTAGTGTATGCG	0.029	0.005	0.022	13	573
GCCGGCGTTGGGA	0.831	0.025	0.828	802	167
ACTGGTGCAGGTG	0.024	0.011	0.012	9	745
CGCAGTGTAGGTA	0.031	0.001	0.033	39	1136
AGTGTCGTATAGA	0.024	0.004	0.019	20	1039
GCTATCGTTAGCG	0.065	0.008	0.057	55	903
AGCGGCGCAGAGG	0.023	0.006	0.027	20	708
GGCGTTATAAAGG	0.068	0.009	0.056	71	1208
GGTGTCGTATGCA	0.106	0.027	0.092	62	610
CCTAGTGTAGAGA	0.022	0.006	0.016	27	1634
GGTGGTGTAGAGG	0.2	0.035	0.228	89	302
GCCATCGCAAGTG	0.052	0.004	0.05	61	1159
ACTGGCATTGGGG	0.031	0.007	0.037	19	498
CGTAGCGTTTATA	0.023	0.004	0.028	29	1005
GGTGTCACAAGTG	0.082	0.018	0.06	41	637
CCCAGTATTAGTG	0.028	0.001	0.028	56	1943
GCCGTCGTAGGGG	0.058	0.006	0.052	25	453
AGTATTGTTGGGG	0.035	0.004	0.04	24	579
GGCGTCACTTGCG	0.069	0.003	0.072	54	700
CGTGGCACTGACG	0.023	0.005	0.016	12	759
GCTAGTACTTGCG	0.057	0.004	0.058	49	795
CGTGTCGTTGAGA	0.024	0.006	0.025	20	770
CGTGTTACTGGTA	0.028	0.003	0.032	30	914
CGTAGTATTTAGG	0.037	0.004	0.032	23	704
AGTATTGCTTATG	0.021	0.003	0.02	22	1088
GCCGTTGCTGATG	0.04	0.003	0.044	36	781
GCTATTGTTTGGG	0.054	0.001	0.052	31	560
GCTAGCGCTTATG	0.07	0.009	0.061	40	615
GGCGTCGCTGGCG	0.066	0.004	0.07	48	636
ACTGTTGTAAGGG	0.032	0.006	0.028	27	938
CGTGGCGCTGGCA	0.025	0.007	0.016	9	569
GCCGGCGCTGGGA	0.468	0.063	0.488	349	366
AGTGGCATAGAGA	0.032	0.009	0.02	13	640
GGTATCATTGGTG	0.094	0.014	0.079	46	534

GGCAGTGTTTGCA	0.046	0.006	0.054	57	992
AGTGGTACTTATA	0.022	0.002	0.025	32	1259
CGCGTCGCAGGCA	0.029	0.004	0.025	33	1308
CGCGGTGCTGGTG	0.022	0.01	0.011	6	548
GGTGGTGTTAATG	0.224	0.034	0.243	147	458
GCTAGCGTTGAGG	0.077	0.01	0.063	33	494
GCCGTCGCTAACG	0.05	0.01	0.056	59	1002
CGTAGCGTAAGTG	0.032	0.008	0.023	18	782
GCCGTCGTAAGGA	0.047	0.005	0.05	47	888
GCTGTTGCTGACA	0.046	0.004	0.044	45	978
CGTAGCGCATGGA	0.023	0.005	0.016	11	660
GCTGTTGTTAAGA	0.049	0.006	0.055	58	1005
GCCGTCGTAAGGG	0.046	0.003	0.044	29	632
GGCAGCGCTTGGA	0.052	0.008	0.05	32	610
CGTGTCATTAGTG	0.022	0.002	0.019	15	757
CGTATCATTTGGA	0.025	0.003	0.027	29	1053
GGTAGTGTATGTA	0.058	0.002	0.055	41	709
CGCATTATTGAGG	0.023	0.004	0.029	40	1359
CGCGTTGTAAGTA	0.032	0.011	0.037	57	1478
CCCGTTACTTAGG	0.026	0.001	0.026	36	1358
GGTGGTGTATAGG	0.215	0.003	0.214	69	254
CCTGGCATAAGCG	0.032	0.012	0.027	35	1285
CGTATCACTGATG	0.026	0.001	0.028	31	1078
GGTAGTATATGTA	0.07	0.01	0.063	76	1137
ACCGGTATAGGCA	0.02	0.004	0.016	39	2352
ACTGGCATTGACG	0.028	0.002	0.028	34	1174
GGCGGTATATGCA	0.08	0.014	0.061	72	1117
GCTGGCGCTAGTA	0.599	0.041	0.582	566	406
GGTATTGCTAGTA	0.069	0.006	0.061	49	757
AGTAGTACAAGGA	0.021	0.001	0.021	29	1358
GCCATTGCTAGTG	0.049	0.003	0.052	50	903
AGCGGCGCTAGTA	0.026	0.006	0.02	23	1118
GGTAGTACTTAGA	0.066	0.008	0.056	44	743
GGTAGTATTTGTG	0.061	0.011	0.047	28	573
GCCATTGTTGATA	0.05	0.005	0.049	94	1805
ACCATTATAGGGG	0.027	0.002	0.029	38	1285
CCTGTCACAGGCG	0.026	0	0.026	32	1197
GGCGGCACAAGGA	0.145	0.023	0.139	140	868
GGCGTTACTGATG	0.058	0.012	0.042	41	933
CGTGTCGCTTGGA	0.027	0.007	0.032	15	453

AGTGTCATATAGG	0.029	0.006	0.022	22	962
ACTGGCGCTGAGG	0.031	0.005	0.028	16	558
ACTAGCATAGGGG	0.027	0.005	0.024	20	811
GGCATCACATAGG	0.092	0.004	0.091	116	1164
GCTGGTGCATGTG	0.272	0.02	0.255	131	383
CGTATCATTGGGA	0.025	0.005	0.026	25	952
ACTAGCGCAGACG	0.027	0.004	0.021	34	1562
GCCGGTATATAGG	0.161	0.029	0.163	175	898
CCTATTACAAGGG	0.027	0.004	0.023	36	1543
GGTGGTGCATGTA	0.069	0.014	0.061	38	587
AGTGGCACAAAGG	0.029	0.014	0.016	11	656
GCTATCGCTTATA	0.053	0.005	0.046	60	1240
GCTGTCGCTTAGG	0.06	0.014	0.04	15	359
GCTGGTGCTGAGG	0.288	0.035	0.28	112	288
GGTATCGCTTACA	0.095	0.025	0.082	88	981
CGTGGCGTAAGTG	0.023	0.004	0.026	14	518
CGTGTCGTTAATG	0.03	0.003	0.029	27	889
CGCGTTGTTGACG	0.029	0.002	0.032	32	973
CGTATCGTAAGCG	0.028	0.006	0.02	25	1243
GGTAGTGTTTAGG	0.064	0.014	0.051	24	443
CGTGGTACTGAGA	0.025	0.007	0.023	19	825
CGTAGTACTAACA	0.022	0.001	0.021	40	1898
GGCGTTACTAAGG	0.058	0.003	0.054	49	854
GGTATTATAAGCA	0.093	0.011	0.082	147	1643
CGCATCGTAGATG	0.025	0.003	0.022	34	1526
ACTATTACTGGGG	0.021	0.003	0.017	17	964
GGTAGTACAAACG	0.056	0.01	0.042	51	1156
AGTATTATATATG	0.024	0	0.024	54	2224
CCTAGCATTGGCG	0.029	0.008	0.018	19	1032
GGCAGCGTTTGCG	0.055	0.015	0.035	25	697
GCTATCGTTAGGG	0.054	0.003	0.053	35	628
GCTAGTATAGAGG	0.071	0.003	0.068	63	869
CGTGGCGCTTGTA	0.014	0.003	0.01	6	605
CCCATCACATGGG	0.035	0.001	0.037	54	1421
CGTGGTGCTTAGA	0.023	0.003	0.022	12	540
CGTATCATAAACG	0.026	0.001	0.024	56	2259
ACTGGCGCTGGTG	0.017	0.007	0.017	10	564
CGCGGTGCTTACG	0.028	0.003	0.024	21	856
GGTGGTACTAGCG	0.106	0.028	0.094	46	442
AGTGTTGTTGGTG	0.019	0.005	0.014	7	491

GCTATCGTATGTG	0.059	0.006	0.053	47	845
GGCGGCGTAGATA	0.195	0.036	0.189	199	856
GCTGGCACAAAGG	0.429	0.03	0.421	443	609
GGCAGTGCATGGG	0.058	0.003	0.055	29	497
CCTAGTGTTGATA	0.026	0.001	0.027	50	1820
ACTGGTATTAGTG	0.031	0.004	0.027	30	1066
GGCGTTACTGAGG	0.069	0.019	0.049	35	682
CGTATTATTTGTG	0.027	0.004	0.024	28	1132
CCCGTTGTATGGG	0.025	0.003	0.02	20	961
GCCGGCACTTATA	0.108	0.005	0.114	180	1400
AGCGTTGTATAGA	0.022	0.003	0.019	32	1613
GCCATTACAAGGA	0.058	0.003	0.06	112	1757
ACCGGCGCTGGCG	0.025	0.001	0.023	22	921
GCCGTTGCATGGA	0.043	0.002	0.044	31	681
GGCAGCATTAGTG	0.051	0.004	0.046	45	933
CGCGGTACAGATG	0.024	0.002	0.021	26	1199
CGTAGCACATGTA	0.024	0.002	0.025	28	1094
CGCGGCATAGATG	0.03	0.005	0.023	22	919
AGTGTCGTTGAGG	0.017	0.007	0.019	12	604
GCCGGTGTTAAGA	0.169	0.023	0.173	221	1060
CGCAGCATTTGGA	0.021	0.002	0.024	26	1079
AGTGTTGTTGATG	0.027	0.005	0.021	17	784
GCTATCACAGGTG	0.065	0.01	0.055	47	814
GGTAGCATATAGG	0.073	0.006	0.066	39	550
CCTGGCACTGGTG	0.037	0.009	0.05	35	659
CGCATCACTAGGA	0.022	0.001	0.02	32	1561
GGTGGCGCAAACG	0.309	0.029	0.278	214	556
ACTGTCGTAGGGG	0.039	0.013	0.022	13	577
CCTAGTACTTACG	0.024	0.004	0.03	51	1643
GGTGTTACATGCG	0.104	0.033	0.058	34	549
GCTAGCATAGGCG	0.065	0.015	0.068	60	821
AGTGGTATATACG	0.032	0.003	0.029	29	965
GCCGTTGCAGGGA	0.043	0.004	0.048	33	652
GGTATCGTTAATG	0.137	0.012	0.144	140	830
GCCGTCACATAGG	0.046	0.003	0.042	44	997
CCTGGCATTAGTG	0.027	0.007	0.019	18	929
AGTGTTACAGAGG	0.028	0.005	0.022	18	792
AGTGTTGCTAGGG	0.029	0.001	0.029	13	434
AGTATTGCATATG	0.023	0.002	0.022	29	1270
AGTGGCATTGGTG	0.031	0.006	0.037	7	183

GCCGTCGCTAATA	0.049	0.007	0.042	55	1265
CGTATTACATGGG	0.028	0.005	0.031	25	794
AGCGGCATTGATG	0.026	0.011	0.012	7	592
AGCAGTGTATACG	0.021	0.001	0.019	40	2037
CGTATTGTTTACG	0.023	0.006	0.031	32	1005
GCTGGCACTAGGA	0.218	0.007	0.222	169	592
ACTGTCGCAAGGA	0.023	0.002	0.023	31	1302
AGTATCATTGATG	0.03	0.001	0.029	44	1486
GCTGGCACTAGGG	0.48	0.048	0.453	258	311
GGCATTGCTTAGG	0.083	0.009	0.085	59	633
AGTAGTGTTGACA	0.022	0.001	0.023	34	1423
GGTGTTGTTGAGG	0.098	0.023	0.068	27	368
AGTGGTACAGAGA	0.021	0.002	0.02	22	1091
GCTGGCATTAGGG	0.844	0.018	0.843	727	135
GGTAGCATTAGTG	0.079	0.023	0.049	31	600
CGCGTCGTTAGGA	0.022	0.005	0.024	22	910
CCTGTTGTAAATG	0.025	0.004	0.024	45	1811
AGTGTTACTAGTG	0.024	0.008	0.013	12	902
GCCGGCATTAAGG	0.731	0.04	0.712	1110	450
CGCGGTGCAAGGG	0.028	0.001	0.028	19	655
CGCAGCATTAGGG	0.034	0.007	0.042	34	784
CGCAGCACAAGGG	0.027	0.008	0.022	24	1090
ACCGGCGCAGGCG	0.019	0.003	0.023	25	1064
ACTGGTGCAAGCG	0.023	0.005	0.018	20	1108
GCTGGTACTTGTG	0.096	0.013	0.078	39	460
GCCGTTACAGGTG	0.056	0.01	0.052	47	861
CGTGTTGTAAGGA	0.023	0.003	0.02	16	784
GGCATCGTTGATA	0.107	0.016	0.098	146	1351
GCTATCGCAGGGG	0.056	0.022	0.057	30	493
GGCGGTATAGAGG	0.111	0.033	0.097	76	708
CGTGTCACAGGCG	0.029	0.004	0.03	25	811
AGTAGCACTAGTA	0.021	0.005	0.014	18	1305
GCTGTCATTAAGG	0.071	0.005	0.065	55	791
GGTAGCACTGATA	0.062	0.006	0.054	55	958
GGCGTCATATGTA	0.069	0.012	0.052	70	1266
AGTGTTGTTGGGA	0.016	0.004	0.022	13	586
CGCGTCGTTTGGG	0.02	0.003	0.017	11	646
CGTGGCGTTTAGG	0.023	0.008	0.013	4	316
AGTGGTGCTGATG	0.024	0.008	0.029	17	568
CGTGGCGCAAACG	0.026	0.006	0.034	29	818

GCCGTCGCTTGCG	0.05	0.01	0.05	31	589
CCTATTGCTAGCA	0.023	0.004	0.024	51	2092
GGTGTCGTTGGCG	0.073	0.008	0.068	31	423
CCTGGCACATATG	0.026	0.003	0.03	41	1331
CGTATTGCATGTG	0.028	0.005	0.032	26	784
CGTGGCACATAGA	0.023	0.009	0.017	16	924
AGCAGTGTTAAGG	0.027	0.002	0.025	36	1402
CGCGGTGTAGATA	0.029	0.003	0.025	33	1267
CCTGGCGTAAGTG	0.019	0.006	0.01	9	857
ACCAGCGTTAAGG	0.022	0.001	0.024	35	1427
GGTGGCACAAGCA	0.205	0.045	0.168	153	760
GCTAGTGCTGATG	0.048	0.004	0.051	39	720
AGTATCGCAAGTG	0.024	0.004	0.018	18	982
GGCATCATTTATG	0.085	0.007	0.075	122	1506
GGTGTTATAGGCA	0.084	0.015	0.064	56	813
CGTATTGCAGATG	0.029	0.009	0.03	31	987
ACTAGTGTTGGGA	0.029	0.002	0.03	32	1037
GGCAGTATTGGTG	0.062	0.004	0.057	49	813
AGTGGTGCAAACG	0.026	0.003	0.022	23	1013
GCCGGCGCAGACG	0.763	0.035	0.748	1059	356
GCTATTGCATGGG	0.05	0.01	0.039	27	674
CGCGGCATAGACG	0.028	0.001	0.03	37	1205
GCTGGCATATGCA	0.663	0.032	0.673	1032	501
AGTGGCGTTTATA	0.029	0.008	0.031	23	722
GGCGGCGTAGAGG	0.435	0.034	0.402	268	398
GGTATCACTAATG	0.124	0.014	0.114	114	888
CGTAGCGTTAACG	0.03	0.008	0.038	39	999
GCCAGTGTTTGTG	0.046	0.007	0.037	29	748
CCCGTCACTGGGG	0.041	0.003	0.044	40	867
GGCAGCACAGACG	0.051	0.007	0.041	56	1304
GGTGTCGTTGAGA	0.091	0.014	0.08	41	473
GGTGGCGTAAACG	0.677	0.043	0.644	800	442
CGCGTCATTTGCG	0.02	0.002	0.019	20	1019
GCCGTTGTTTGCG	0.061	0.016	0.039	30	736
CGTATTGCTGAGA	0.029	0.003	0.025	23	892
CCTAGTACAGGGG	0.042	0.007	0.032	29	866
ACCGGTGTAGGGG	0.02	0.004	0.016	11	695
CCTGTCGCTTAGG	0.025	0.006	0.024	17	697
CCTAGCATTTAGG	0.033	0.005	0.035	42	1150
CGTGGCGTTGGGA	0.023	0.004	0.019	7	367

AGTAGCGCATAGG	0.019	0.002	0.021	14	648
CGTATTATTTGGG	0.029	0.003	0.027	21	766
GCTGTTATAGGTA	0.059	0.001	0.059	64	1024
GCTAGCATTGGTG	0.063	0.008	0.067	47	659
AGTGTCGCTAAGA	0.031	0.004	0.034	34	979
GCCGGTATTAGTA	0.077	0.01	0.089	134	1370
GGTGTCACTGACG	0.089	0.008	0.078	56	659
CGTGGTGCTGGGA	0.033	0.008	0.024	10	411
CGTAGTACATATG	0.027	0.002	0.029	32	1082
GCCGTCGTTGGGG	0.062	0.003	0.06	24	374
GGTGGCGCAGATA	0.146	0.024	0.114	77	599
ACTGTCGCAGGGG	0.025	0.005	0.022	15	660
CCCAGTGTATATG	0.031	0.004	0.026	52	1940
GGCAGCATAGGCA	0.05	0.005	0.044	64	1392
AGTAGCATATGTA	0.023	0.005	0.018	29	1605
CGTAGTATTGGGG	0.025	0.008	0.014	6	437
GGTGGCATTAATG	0.711	0.042	0.678	933	444
CGCGTCGTAAGCA	0.024	0.005	0.017	31	1765
AGTGGCATAAGCA	0.025	0.006	0.017	16	920
CGTATTGTTTGTA	0.022	0.002	0.022	25	1122
GGTGGCACAGGTA	0.185	0.025	0.171	127	617
ACCAGTGCTTGGA	0.02	0.004	0.023	35	1505
GCCATCGCTGGGA	0.055	0.006	0.049	38	734
GCTGGCGCAGGTA	0.611	0.046	0.616	567	354
CGTAGTGCTAAGG	0.022	0.002	0.021	12	561
GGTAGCGCTAGCA	0.07	0.012	0.074	53	665
GGCGGTGCTTATG	0.059	0.015	0.044	27	584
GGCGTTGTTGGTA	0.055	0.008	0.044	32	698
GCCGTTATTTACG	0.062	0.01	0.054	79	1386
GCTGGTGTTAGTG	0.746	0.049	0.706	547	228
GGTAGCGCAGGCG	0.07	0.003	0.067	32	447
ACCGTCGCTTAGA	0.02	0.004	0.015	23	1528
CCCATCATTAGGG	0.04	0.004	0.046	66	1360
GCTGGCACTAGTG	0.473	0.042	0.477	336	368
AGTGGTGTTTAGG	0.025	0.008	0.014	6	433
CGTAGCACTTAGA	0.023	0.007	0.021	21	980
AGTATTGCATGCG	0.031	0.005	0.039	42	1043
GCCGGCGCAAGTA	0.501	0.056	0.472	656	734
GCCGGCGCAGGCG	0.729	0.038	0.724	816	311
CGTGTTGCTTGCA	0.026	0.008	0.015	12	769

AGTGGTGTTGATA	0.028	0.009	0.021	21	969
GGTGGTGCTAACG	0.088	0.017	0.104	60	518
AGCAGCACTGGGG	0.024	0.007	0.033	35	1023
AGTGGCGCTTGTG	0.024	0.006	0.028	10	352
GGTATTGTTAGTG	0.093	0.012	0.1	72	645
CGCAGCACATGGG	0.03	0.004	0.024	22	881
CGCATCGCTAGCA	0.021	0.001	0.022	40	1762
GGTGGCGCTTGCG	0.324	0.087	0.289	99	244
ACCGGTGCATATA	0.026	0.005	0.025	64	2491
AGTGTTACATGGG	0.021	0.003	0.016	11	662
CCTGGCATTTGTA	0.028	0.003	0.025	33	1269
AGTGTCACAAACA	0.026	0.002	0.029	65	2208
GGCGGCGCAAGTA	0.093	0.008	0.092	94	930
GCTAGCGTTGGTA	0.067	0.002	0.065	48	691
CGCGTTATTGATG	0.029	0.004	0.031	39	1238
CCTATCACAGAGG	0.028	0.004	0.026	45	1717
CCTGTTGTAGGTA	0.02	0.002	0.017	22	1276
AGCATTACTGATG	0.02	0.001	0.02	46	2250
GGTAGCACAGGGG	0.045	0	0.044	19	408
CCCGGCACTTAGG	0.03	0.005	0.029	34	1131
CGCATTGCTGGTA	0.023	0.001	0.022	24	1049
CGTGTCGCTAGCG	0.023	0	0.023	15	625
ACCGTTGCTTAGG	0.02	0.002	0.017	19	1076
GCTAGTATTTGGG	0.056	0.015	0.044	30	652
CCTGTCGCTGGCG	0.028	0.006	0.022	18	797
AGCGGTATAGGGG	0.019	0.005	0.015	15	982
CCCATTGCTGGTG	0.018	0.003	0.017	21	1237
GGTGGTGTAAGGG	0.185	0.038	0.184	76	336
GGCGGCACTGGTA	0.12	0.006	0.112	97	766
GCTGTCATTTACG	0.06	0.003	0.056	57	967
GCCAGTACTGGGG	0.049	0.008	0.057	38	631
AGCGGCACATAGG	0.027	0.007	0.021	18	850
AGTAGTGTTGGGG	0.025	0.011	0.021	9	419
AGTGTCGCTTGGA	0.026	0.006	0.017	10	571
AGTGGTATAAGTG	0.03	0.003	0.031	24	754
CCTGTTACAGGCG	0.027	0.003	0.025	30	1148
GCTGGCGCAGAGA	0.621	0.052	0.614	652	410
GGTATTGCAAGCA	0.081	0.014	0.077	82	983
CCTGGCGTAGACA	0.023	0.002	0.021	41	1910
CGTAGTACAAGCA	0.029	0.004	0.034	48	1359

GGCATTGTTGGGA	0.083	0.016	0.067	50	698
CCTGTCACTAATG	0.021	0.001	0.022	35	1578
GCTAGTGTATGTG	0.056	0.005	0.059	54	855
AGCAGCACATATG	0.022	0.002	0.02	41	2012
CGTGTCATTTGTG	0.021	0.005	0.016	10	630
CGCGTTGCAGGTA	0.019	0.004	0.019	22	1120
CGCGGCACATAGG	0.031	0.004	0.026	25	921
AGTAGCACAGGGG	0.016	0.005	0.009	6	641
GCTAGTACAAGTG	0.072	0.018	0.057	59	976
GGTGGCGCTAGTG	0.322	0.05	0.318	120	257
GGTGGCGCTAGGG	0.285	0.052	0.28	82	211
GGTAGTGCTAGGA	0.048	0.011	0.046	21	439
ACTGGTATATGGG	0.025	0.004	0.02	16	768
AGTGGCGTTGATG	0.022	0.004	0.017	5	285
AGTGTTGTTAATG	0.025	0.011	0.012	12	1013
CGCGGTGTAAATA	0.026	0	0.027	53	1916
CGCATCGTTGATA	0.022	0.004	0.022	44	1936
ACTGTCGCATAGG	0.024	0.006	0.017	16	923
CGTGGTGTTTACG	0.025	0.007	0.015	9	585
GGTATCATTTACG	0.119	0.008	0.109	101	823
AGTGGTGCTGGCA	0.019	0.009	0.007	5	712
ACTGGCGTTGAGA	0.032	0.004	0.035	28	772
ACTGGTGTTAGCG	0.031	0.003	0.033	28	817
GGTATCACAGGTG	0.119	0.019	0.1	69	622
GCCGGTGCAGGGG	0.109	0.013	0.103	50	435
CCTGGTATTAATG	0.025	0.004	0.02	29	1409
AGCGTTACTAGCA	0.023	0.003	0.019	37	1926
GCTGGCGTTGATG	0.948	0.01	0.948	1142	63
ACTGGTGCTGACG	0.024	0.006	0.021	24	1103
GGTAGCGTTAGTA	0.083	0.019	0.056	34	570
AGTAGTGTTGAGG	0.022	0.002	0.02	14	693
GGTAGTGTAGAGA	0.074	0.004	0.07	52	691
GGTGGCACAGAGG	0.407	0.036	0.409	250	361
GCTGTCGCATATA	0.058	0.005	0.051	55	1026
CGTGGTACTGATA	0.027	0.002	0.027	28	1015
GGCAGTGCAGGTG	0.048	0.004	0.047	32	649
CGTATCGTAAGGG	0.023	0.001	0.022	21	914
GCTATCGCTGACG	0.058	0.013	0.046	42	873
CGTGGCGCAAGGA	0.032	0.003	0.03	18	592
CGCAGTGCATGGG	0.03	0.011	0.022	17	743

GCTGTTACAGACG	0.059	0.006	0.058	60	975
GGTGTTATAGAGA	0.075	0.008	0.072	63	809
AGTGTCATAGGGA	0.016	0.003	0.017	15	882
GGCGGTGCAGGGA	0.055	0.012	0.058	30	491
ACCGGCATTGGGG	0.024	0.003	0.026	18	676
AGTGGCGCTGAGG	0.021	0.014	0.008	3	356
CGTGTTGTAGGTA	0.017	0.006	0.025	19	747
AGTAGCATTTACG	0.022	0.009	0.016	20	1260
CGTGGTGCTTGCA	0.021	0.011	0.005	3	585
AGTGTCGTATGCG	0.026	0.005	0.024	17	680
GGTGTCACTTAGG	0.107	0.018	0.091	40	401
CCTGGTGCTGACG	0.023	0.005	0.021	21	961
GCCGGCGTAGGCA	0.859	0.026	0.838	1417	274
GGCATTGCTGATG	0.078	0.006	0.077	80	957
CGTATCGCATATG	0.023	0.002	0.022	23	1030
CCTAGCGCTTAGA	0.024	0.002	0.027	39	1421
ACTGGCGTATGGA	0.02	0.006	0.026	19	700
ACCGGTGTTGGTG	0.02	0.001	0.02	17	850
CCTGTTGTAGATA	0.026	0.001	0.028	53	1855
CCTGGTACAAAGA	0.024	0.001	0.022	49	2152
GCCGGCGTATACA	0.857	0.023	0.854	2180	374
GGTGTCACATGGA	0.066	0.008	0.063	39	583
GGTGGCGTATGGA	0.402	0.041	0.379	184	302
CGTGGCATTTACG	0.022	0.005	0.027	16	575
AGTGGTGCATGGG	0.028	0.002	0.03	12	394
AGTAGCGCTTGGG	0.036	0.001	0.037	14	363
GGCAGTGTTTGGG	0.042	0.013	0.032	19	580
CGTGGCGTTTACA	0.027	0.005	0.02	15	747
CCTGGTGCTAGCG	0.026	0.005	0.02	16	804
GGTAGCGTTGGGA	0.078	0.015	0.06	22	346
AGCGTCATTTGCA	0.025	0.003	0.023	42	1765
AGTAGTGTATGTG	0.021	0.001	0.02	17	835
GCCGTCGCAAGTG	0.051	0.005	0.046	35	721
GGCGTTACAGATA	0.06	0.009	0.056	94	1588
GCTGGCACTTGTG	0.465	0.038	0.463	291	337
GCTGGCATAAACG	0.853	0.025	0.847	1972	355
AGCGGCATTGGGG	0.017	0.006	0.014	6	432
GGTGTCGCAGGCG	0.074	0.019	0.076	38	465
CGCGGTGTTGGGG	0.035	0.002	0.037	15	394
AGCGGCGTTAAGG	0.02	0.002	0.023	14	600

AGCGGTATTAAGG	0.021	0.004	0.016	20	1243
CCCGGTGCTAGTA	0.029	0.009	0.024	37	1477
GCTGGTACTTGCA	0.08	0.012	0.074	65	808
CCTGTTGCTTGCA	0.022	0.002	0.019	25	1275
GGTGTCGCAAAGG	0.128	0.027	0.096	55	517
ACTGGTATTGACG	0.032	0.007	0.035	45	1242
CGTGTTGTAAAGG	0.025	0.003	0.024	20	808
GGTGGCACATGCG	0.389	0.029	0.363	232	407
GGTATTACTAGTG	0.106	0.017	0.095	76	720
GGTGGTATTTGCG	0.277	0.034	0.303	157	362
GGTGTCATAAGTA	0.092	0.016	0.083	82	906
GGTATCACATACG	0.128	0.019	0.112	128	1011
CGCGGTGTTTAGA	0.022	0.002	0.021	18	859
CCCGTTGTATATG	0.024	0.002	0.025	41	1629
CGTGTCGCAGGGA	0.024	0.004	0.029	18	600
AGCGTTACAGGCG	0.018	0.002	0.016	19	1193
GGTGTTGCATAGA	0.084	0.015	0.071	46	602
AGTAGTGTTTATG	0.027	0.009	0.04	39	942
CCTATCGCTTGTA	0.024	0.003	0.021	32	1524
CCTAGTGCATGTG	0.023	0.004	0.026	30	1139
CCTGTTATTGGTG	0.019	0.006	0.015	15	956
ACTGGTACTGAGA	0.019	0.004	0.017	29	1643
GGTGTTACAGGCG	0.101	0.022	0.092	54	533
GCCGGCACTAGCG	0.211	0.03	0.213	223	826
CGTGGCACATGGA	0.028	0.005	0.023	16	675
GCCGTCACTGGCG	0.059	0.014	0.053	54	973
GCCAGCATAGGTG	0.071	0.01	0.074	75	940
CGCGGTACTTATA	0.024	0.002	0.022	34	1500
CGTGGTGTAAGGG	0.023	0.009	0.028	13	456
GGCGTCGCTGAGG	0.075	0.016	0.08	46	531
GCCGGTGTTTGCG	0.398	0.04	0.359	256	457
GGTGGTACTTGCG	0.1	0.031	0.07	28	370
CCTGGCATTGGGG	0.044	0.01	0.056	34	575
CGTAGTGCAGGCA	0.02	0.004	0.015	11	743
GCTGGCGTTAGCA	0.884	0.022	0.879	1272	175
GGTATTGCTGACA	0.07	0.001	0.07	73	972
CGTGGCGCTAAGG	0.023	0.01	0.018	9	497
AGTGGCACTTAGG	0.025	0.008	0.023	9	375
ACTATTATAGGGA	0.032	0.002	0.03	54	1762
AGTATTACTGAGG	0.019	0.005	0.013	15	1099

GGTATTATAGGCA	0.093	0.018	0.068	91	1240
GCTGGTATTTGTG	0.452	0.02	0.459	308	363
CGCAGTACTTGGG	0.03	0.006	0.022	16	698
GGTGTCGCATGGG	0.114	0.028	0.115	37	284
GGCAGCGCTGGCG	0.073	0.005	0.078	55	649
GGCAGTGTAGAGA	0.049	0.003	0.045	48	1030
GGTAGCATAGGCA	0.061	0.012	0.045	38	803
AGTGGTACAAATG	0.024	0.001	0.025	33	1263
GGTATTACATAGG	0.1	0.015	0.114	105	819
GCTATCACATGCG	0.059	0.012	0.066	78	1102
CGTATTATTGATA	0.028	0.007	0.019	42	2206
GCTAGCATAGGCA	0.066	0.005	0.063	88	1313
GCCGTTGCTGGGA	0.052	0.002	0.052	28	511
GGCAGCACTGGGA	0.045	0.011	0.041	31	732
CCTATCGCTAGGG	0.027	0.007	0.034	33	925
CCTAGCGTTGGGG	0.024	0.003	0.019	11	571
GCTGTCGCTAACA	0.05	0.005	0.044	52	1131
GCTATTGTAGGGA	0.07	0.01	0.062	58	871
AGCAGTGCTTGGG	0.013	0.001	0.012	10	803
GCCGTTGTTAGGA	0.042	0.008	0.051	42	784
GGCGGTATTTGCG	0.122	0.03	0.099	71	644
CGTAGTGTTAATA	0.024	0.003	0.02	25	1207
CGTATTGCTAGGA	0.028	0.006	0.023	20	840
ACCGGCACAGGGA	0.025	0.002	0.027	36	1275
ACTGTCATAGATG	0.022	0.003	0.018	30	1636
CGCAGTGTTAAGG	0.033	0.011	0.048	49	965
CGTATCGCATAGG	0.021	0.01	0.008	7	846
CGTGTCACATGCA	0.017	0.007	0.007	8	1152
AGCGGTATAGAGG	0.025	0.001	0.024	27	1091
GGTATCATTGGTA	0.124	0.011	0.109	101	827
ACTGTTGCATATG	0.024	0.006	0.016	24	1484
GGCGGTGTAGGTA	0.075	0.01	0.062	48	723
CGTGTTGCTTACG	0.026	0.002	0.029	20	673
GCCGGCATTGGTG	0.716	0.035	0.709	830	341
CGCGTTACTTAGG	0.024	0.006	0.028	25	882
CGCAGTGTAGAGG	0.029	0.011	0.044	41	890
CGTATCGTATGCG	0.027	0.006	0.019	19	1007
CCTGGCACAAGGA	0.023	0.004	0.02	28	1403
CGCGGCATTGACA	0.021	0.002	0.019	33	1744
GCCGGCACATGGA	0.105	0.016	0.086	86	919

GGCGTTGTATGGA	0.068	0.01	0.054	44	774
CCTGTCGTTGACG	0.025	0.003	0.025	31	1190
GCCATCACAGGGA	0.052	0.005	0.046	61	1279
CGTGGCACAGGCA	0.018	0.003	0.018	15	832
GCCGGCGTTTAGG	0.93	0.016	0.928	992	77
CCTGGCGTTTGGA	0.028	0.01	0.022	17	760
GGTGGTATTTGTA	0.135	0.034	0.133	86	561
GGTGTTGCTTGCA	0.052	0.006	0.043	25	553
GCCGTTACTTGGG	0.051	0.008	0.047	30	612
GCCGGTGCAGAGA	0.057	0.009	0.053	47	841
GGTGTCGCTTGGG	0.082	0.011	0.079	21	244
ACTGTTATTGGTG	0.035	0.006	0.029	29	967
AGTGGCGTATAGG	0.023	0.011	0.035	11	305
ACCGTTATAGGCG	0.02	0.002	0.017	30	1697
CCTGGCATTAATG	0.029	0.004	0.033	45	1300
GGCGGCACAGGTG	0.254	0.047	0.224	172	597
GGCATCGTTTGTG	0.118	0.019	0.097	79	735
GCTAGTGCTAGTA	0.05	0.005	0.049	48	925
GGCGGTACTAGGG	0.066	0.012	0.061	29	447
CCTAGCGTAAGTA	0.022	0.003	0.026	51	1919
GGCGTTGCAAGTG	0.068	0.022	0.049	38	732
CGTAGTATAGGGG	0.028	0.005	0.035	19	531
CGTGGTGCAGGTA	0.024	0.004	0.026	17	625
GCCGTTATATGGG	0.064	0.008	0.062	54	814
CCTGGCGTTAGCA	0.019	0.006	0.015	19	1278
GGTGTTACAAGCA	0.07	0.005	0.065	75	1076
CCTGGCGCTTATG	0.03	0.002	0.031	27	852
CGCGTCGTTGGGG	0.028	0.008	0.028	12	417
CGTGTCGCAAAGA	0.025	0.009	0.017	19	1074
AGCGGCGCTAGGA	0.019	0.001	0.021	17	809
AGCATCGCTTGCG	0.021	0.001	0.022	29	1291
ACTGGCACATGGA	0.022	0.004	0.028	32	1124
AGTGTCGCTAATA	0.027	0.002	0.027	37	1324
GGCATTACTGGTG	0.052	0.004	0.047	43	865
GCCGTCGTAGATG	0.051	0.01	0.066	60	853
GCTGGCATATGCG	0.864	0.038	0.881	1147	155
GGCGTCGTAGGGG	0.053	0.015	0.032	22	660
GGCGGCATTTGGG	0.54	0.052	0.516	337	316
CGTGGCATTTGTA	0.03	0.006	0.037	24	630
GGCGGTACTTGGG	0.073	0.02	0.054	24	419

AGTAGCGCTTGTA	0.031	0.014	0.014	12	825
GCTATCGTTTGCA	0.057	0.009	0.044	52	1142
CGCATCACTGGGG	0.035	0.003	0.039	32	797
ACTGGCGTAGGGG	0.024	0.007	0.022	8	364
CGTATTGTTGAGG	0.018	0.009	0.008	6	739
CGCAGCGCTTGCG	0.024	0.002	0.021	20	912
GGTATCGTTAGCG	0.134	0.011	0.123	75	536
CGCAGCGTTAAGA	0.025	0.003	0.029	46	1544
AGTAGCACTTATA	0.019	0.003	0.023	39	1624
CGTAGTATTGACG	0.026	0.005	0.021	20	936
CCCGGTATTTAGG	0.028	0.006	0.024	30	1221
GCTGGTACTGGGA	0.07	0.023	0.039	21	520
CCTGGTGTTAGTG	0.021	0.002	0.018	15	811
GCTATTACTTAGA	0.05	0.004	0.045	62	1307
GCTAGCGTTGGCG	0.069	0.005	0.064	41	604
GGCAGCATTTGGA	0.052	0.01	0.039	34	830
GCTAGTGCAAGGG	0.059	0.005	0.059	43	680
GGCATCGCTAGGG	0.103	0.015	0.088	65	676
CCTAGCGCATGTG	0.031	0.009	0.021	22	1005
GCCGTTACTGGTG	0.053	0.004	0.048	38	751
AGCGGTGTTTGGG	0.024	0.009	0.023	12	503
CCTAGCGCAAGGG	0.024	0.007	0.015	13	865
CGTGGCGCTAATA	0.029	0.004	0.026	24	915
GGCGGCGTAAATA	0.22	0.031	0.223	363	1264
CGTGGCGCAAGGG	0.028	0.004	0.026	10	370
ACCGGCGTAAGGG	0.017	0.006	0.01	6	595
GCTAGCGTAGAGG	0.088	0.016	0.067	41	571
GGCGGTGTAAGCG	0.092	0.01	0.083	64	711
GGCGGTGTTTGCG	0.086	0.038	0.074	41	511
AGTAGTACAGGTG	0.017	0.001	0.017	15	850
AGTATCGTTGGTG	0.025	0.003	0.024	18	746
GGTGTTGCAAGGG	0.086	0.01	0.085	35	376
CGTAGCACAGGGG	0.037	0.011	0.021	12	550
AGCAGCGCTTATG	0.022	0.001	0.021	26	1210
GCTGTTGTTTGGA	0.041	0.007	0.046	27	556
CGTGTTGCATACA	0.029	0.006	0.02	27	1351
AGTGGTACTGGCA	0.018	0.003	0.022	17	770
GCCGGCATAAGGA	0.368	0.037	0.349	517	966
GCTAGCACAGATG	0.059	0.01	0.048	52	1024
GGTGGTGCATATA	0.062	0.018	0.046	31	638

GGTATTATAAGTG	0.112	0.011	0.104	123	1064
CGTGTTGTATGCA	0.022	0.004	0.019	18	927
GGCAGTACATGGG	0.06	0.006	0.068	51	704
AGTGGCATTTACG	0.034	0.009	0.042	12	271
CGTAGCGCAAGTA	0.025	0.001	0.024	25	999
AGCGGTGTAGGTG	0.017	0.003	0.012	9	743
CGCAGCACTTAGG	0.021	0.002	0.022	22	999
CGTAGCGCTAGCG	0.024	0.004	0.024	15	603
AGTAGCACAAGCG	0.019	0.007	0.01	11	1095
ACCGGCATAGGGG	0.029	0.005	0.029	26	877
GCTGTCACAAGGA	0.052	0.005	0.056	56	937
CGTGGTGCTTACA	0.027	0.008	0.017	16	899
GCTGGCACTTGGA	0.195	0.016	0.181	124	561
GGTGGCGCTAGTA	0.181	0.027	0.153	82	455
GCTAGCGTAGGGG	0.077	0.016	0.055	22	380
GCTAGCGCAGGGG	0.075	0.028	0.037	13	340
GCTGTTGTTAGTA	0.064	0.007	0.064	62	902
GCCAGTATTAGGA	0.055	0.002	0.053	89	1585
CGTGTCGCAAGCA	0.024	0.005	0.02	23	1108
GGTATTGTAAAGA	0.113	0.025	0.086	126	1345
GGTGGCGCTTGTG	0.299	0.031	0.319	115	246
CCCATTGCTGGGG	0.026	0.008	0.024	23	932
AGCGGTACTGGGG	0.021	0.002	0.022	13	566
CGCGGCGCTAATA	0.022	0.004	0.017	27	1532
AGTGTTACTGACA	0.024	0.003	0.028	43	1508
GCTAGCGCTGGCG	0.053	0	0.053	28	504
CGCGGTGCTAGCG	0.025	0.005	0.018	14	768
AGTGTTGCTAGTG	0.024	0.007	0.033	20	579
GCTGTTGCTTATG	0.061	0.001	0.061	39	599
ACTGGTATTTGGA	0.033	0.007	0.031	35	1097
GGTAGCATTAAGA	0.068	0.001	0.069	72	965
GGTAGCGTAAATG	0.087	0.017	0.078	76	899
ACTAGTGTATAGG	0.027	0.002	0.029	39	1315
GGCGTTGCTGATG	0.07	0.009	0.063	44	653
GGTGTCGCTGACG	0.098	0.016	0.087	55	575
AGTGGTGTTGAGG	0.028	0.004	0.023	10	418
AGTAGCATTAGGA	0.019	0.004	0.024	27	1111
GGTATTACTGGGA	0.094	0.016	0.072	50	641
ACTATTGCTTGGG	0.022	0.001	0.021	23	1080
GGCGGTGTAGACA	0.065	0.006	0.065	80	1144

CGCGGCGCAGGGG	0.024	0.005	0.031	16	507
GCTAGTACTTGGG	0.058	0.009	0.069	38	511
GCTGGTGCTGACG	0.278	0.029	0.24	158	499
CCTGTTATTGAGG	0.021	0.002	0.021	22	1043
GCCGTCATATGTG	0.057	0.007	0.06	73	1146
GCCGTTGTTAGTG	0.051	0.004	0.056	45	765
ACTAGCGTTTGCG	0.035	0	0.035	31	866
GGCATCGTTGACA	0.107	0.013	0.097	170	1580
AGTGGTATTAGCA	0.021	0.003	0.02	23	1141
CGTGTCACTAGGG	0.027	0.005	0.031	17	534
GCCGTTGTATAGA	0.049	0.006	0.04	52	1234
ACCGGTGTAAAGG	0.022	0.001	0.022	35	1527
GCTGGCGCTAACG	0.819	0.036	0.818	1107	247
GGCAGTGTTAGGA	0.046	0.004	0.041	34	797
GGCGGCGTAAGTA	0.209	0.029	0.222	238	834
CCCGTTATAGAGG	0.023	0.005	0.018	27	1492
ACTGGCATTTGCA	0.027	0.005	0.02	26	1286
GCTGTTGTAGGCA	0.058	0.006	0.064	50	736
ACTGGCGCAAAGG	0.03	0.004	0.024	19	767
CGTGTTGCAGATG	0.023	0.01	0.026	18	678
CCTAGCGCAGATG	0.026	0.001	0.027	34	1224
GCTGGTACATAGA	0.084	0.004	0.078	77	904
AGTGTTGTTTATG	0.02	0.003	0.018	14	775
GCCAGCGCATGCG	0.052	0.002	0.05	47	893
GGTATCGTTAATA	0.144	0.036	0.114	163	1270
GGTATCGTAAGGG	0.137	0.019	0.112	66	525
GGTGTCACAAAGG	0.089	0.011	0.098	67	617
GGTAGCACAAGGA	0.069	0.006	0.069	58	778
CGCAGCACAAACG	0.023	0.003	0.019	43	2246
ACCGGTGTTAGGA	0.023	0.003	0.024	28	1163
AGTGGTACATGGG	0.028	0.01	0.034	16	450
GCCAGCACAGAGA	0.045	0.009	0.05	85	1599
GGTAGTGTTAGTG	0.067	0.008	0.059	30	475
GCTAGTACTAACG	0.063	0.01	0.058	79	1291
GGTATCGTTTGTA	0.145	0.017	0.126	100	692
GGTGGTGTTTGTA	0.111	0.007	0.101	42	374
GGTGGTGCAAGGG	0.079	0.012	0.077	24	288
GGTATTATTGACG	0.113	0.012	0.103	114	989
ACTGTCATAGGGG	0.035	0.008	0.032	22	658
CGCGGTACATATG	0.023	0.004	0.02	27	1297

GCCGGCGTTGAGG	0.931	0.014	0.927	1018	80
CGTAGTGCAAGCG	0.029	0.005	0.023	16	688
CGTGTCGTATGTA	0.018	0.003	0.022	20	893
CGCAGCGCTGATG	0.023	0.003	0.02	21	1050
CGTGTCATTTACG	0.024	0.003	0.024	25	1015
GGCAGCATTTGCG	0.057	0.003	0.054	55	969
GGCGGTACTTACA	0.067	0.018	0.055	71	1222
ACTAGCGCTAGGG	0.024	0.002	0.023	19	803
CGTGGTGCTTGTG	0.022	0.005	0.015	6	398
GGTAGCACTAGGG	0.06	0.005	0.064	28	407
CGTGGTGCAAGGA	0.024	0.003	0.025	17	656
GGTGGCACAGAGA	0.212	0.037	0.217	167	601
GCTAGCATATGTG	0.066	0.011	0.061	61	933
GGCGTTGCAGATA	0.068	0.011	0.055	65	1118
GGCGTTATTGGTG	0.072	0.014	0.053	39	692
ACCGTTACTGGCA	0.02	0.003	0.016	44	2701
GCCGTTGCAAGCG	0.039	0.006	0.04	42	998
GGTGGCGCAGATG	0.317	0.03	0.3	172	402
ACCAGCGTATGCG	0.028	0.001	0.027	42	1504
AGTGTTGCAGACA	0.019	0.001	0.018	24	1314
CCCGTCGCATGTG	0.026	0.005	0.023	27	1167
GGTGGCACTGACA	0.191	0.038	0.165	164	828
GGTGGCGCTAAGG	0.291	0.025	0.302	119	275
AGCATTGCTAGCG	0.022	0.002	0.023	35	1487
GGTGGCGCTTATA	0.147	0.007	0.148	88	507
CGCGTCGCAGGGG	0.025	0.004	0.019	11	554
CCTGGTGCATACG	0.023	0.008	0.031	35	1108
GGCGTTGCTGATA	0.054	0.018	0.053	55	974
GCTATCGCTTGGA	0.058	0.016	0.077	55	661
CGTATCGCATGCG	0.027	0.006	0.023	21	907
GCCGTCACAAGGG	0.059	0.012	0.072	67	868
CGTAGTATATAGG	0.024	0.007	0.018	16	878
AGTGTTGCAAAGG	0.024	0.003	0.024	20	824
CGTATCACTTGTG	0.016	0.006	0.008	7	818
GGTAGTGCTGGCA	0.063	0.008	0.052	30	545
ACTGGTGTTTACA	0.028	0.006	0.02	31	1530
GGCGTCGTTTACA	0.077	0.013	0.059	70	1119
CCTATCGCATGTA	0.028	0.005	0.028	56	1909
GGTAGCGTTAGGA	0.073	0.006	0.072	36	466
AGTAGTGTAGGGG	0.029	0.02	0.02	14	670

CGTAGTGTTTACG	0.022	0.002	0.024	16	649
CGCGGTGCTGAGA	0.02	0.005	0.027	23	837
AGTGGTATTAAGA	0.021	0.001	0.022	29	1283
GGCGGTGTTTATG	0.081	0.006	0.074	53	663
GCTGGCATAGATG	0.866	0.029	0.871	1283	190
AGCGGCGTTGGCA	0.025	0.005	0.021	19	900
GCTGTTGCTAGTG	0.051	0.009	0.046	25	522
GCTGTTGCAGGCG	0.055	0.011	0.053	38	683
CGTGTCATTGACG	0.022	0.005	0.03	28	917
CGTAGTACTTGTA	0.031	0.005	0.028	27	931
CGCGGCGCAGATA	0.027	0.007	0.019	24	1249
GGCGTCACAGACA	0.057	0.005	0.059	108	1719
GCTGTCGCATAGG	0.055	0.007	0.064	28	409
GGCATCGCATGTA	0.086	0.009	0.081	106	1203
CGCAGTACATGTG	0.026	0.006	0.017	20	1147
GCTAGTGTAGGTG	0.07	0.01	0.073	58	735
CGCAGCACAGATG	0.026	0.003	0.022	32	1411
GCCAGCGTAGAGG	0.072	0.014	0.059	45	719
GCTATCGTTAAGG	0.06	0.009	0.07	73	973
CGCGGTGTATGGG	0.025	0.007	0.018	11	597
GGCATCGTTGAGG	0.108	0.006	0.099	77	700
GCTGGTGCATAGA	0.127	0.007	0.12	92	672
GGTATTGTATGCG	0.098	0.004	0.093	65	635
CGCGTCGCAAACG	0.022	0.005	0.02	36	1760
CGTGTTGCAGGTA	0.023	0.005	0.019	14	725
GCTATCACTGAGA	0.054	0.003	0.049	64	1233
CGTGTCGCTGGCA	0.022	0.007	0.016	13	825
CGTATCACTAGGG	0.027	0.005	0.03	21	680
GCTGGCATTGATA	0.626	0.047	0.615	1080	675
GGCGGCACTTGGA	0.134	0.006	0.127	83	569
CGTGGCGCAGAGA	0.023	0.008	0.014	9	634
GCCAGTGTATGCA	0.046	0.004	0.048	75	1495
ACTGGTGCTGGCG	0.028	0.005	0.022	17	755
GCCGGCACTGGTA	0.117	0.013	0.128	137	936
AGCAGCACATAGA	0.02	0.007	0.01	23	2176
AGCGTTATATGGA	0.024	0.003	0.02	35	1685
CCTAGCGTTTGGA	0.026	0.005	0.022	21	942
CGTGTTGCAGAGA	0.023	0.006	0.028	23	791
GGTAGCGCATGCA	0.071	0.015	0.07	50	668
CCTGGTGCTGAGA	0.024	0.004	0.03	31	1013

CGTGTCGTATGCA	0.022	0.007	0.032	30	922
ACCAGCGTTGGCG	0.027	0.005	0.033	39	1156
ACCGTCGCAGGGG	0.017	0.001	0.018	14	775
GGCAGCACTTGTG	0.055	0.013	0.049	39	752
GGCATTATTGGCG	0.064	0.008	0.053	62	1113
GGTAGCGTAAAGG	0.081	0.002	0.078	50	593
CGTAGCATTTACG	0.022	0.006	0.014	15	1031
GCCAGCGTAGGGG	0.056	0.008	0.046	24	503
GGCATCGCTTGGG	0.1	0.004	0.103	58	505
AGTAGCGCAGGCG	0.023	0.003	0.02	14	703
GGTATTGCTGGGA	0.09	0.015	0.076	35	428
GGCATTGTATACG	0.085	0.014	0.068	98	1345
GCTGGCATTTGGA	0.648	0.045	0.609	549	353
CCCGGTGCTGGGG	0.028	0.013	0.016	9	560
GCCGGCGCAAAGG	0.755	0.041	0.737	931	333
GCTAGCGTTTGCA	0.065	0.006	0.064	52	760
CGCAGCACATAGG	0.025	0.002	0.025	30	1154
AGCGGCGTTTGTA	0.019	0.001	0.021	20	933
GCCGTTGCTGGTA	0.045	0.011	0.036	28	760
GGTGTTGTTTAGA	0.082	0.014	0.077	45	543
GGTATTGCAGGTG	0.089	0.023	0.066	38	538
GCTAGTGCATGTA	0.044	0.009	0.031	35	1080
AGTGGTACTTATG	0.028	0.011	0.018	16	873
AGCGTTACTAAGG	0.025	0.004	0.029	41	1354
CGTGGTGCAGACG	0.039	0.01	0.028	19	666
GGTGGCGTTTACG	0.674	0.05	0.644	533	295
CGCATTGCTAAGG	0.022	0.004	0.017	21	1203
CGCGGTATAGAGA	0.019	0	0.019	27	1384
GGCGGTACAGGCA	0.064	0.004	0.068	70	956
GGCGTTACTGGTA	0.063	0.005	0.066	63	897
GGCATCGTAGATA	0.115	0.024	0.094	170	1648
CGCGTCACTTGGA	0.021	0.005	0.014	14	979
CCTGGTGCATAGG	0.031	0.011	0.03	22	702
GGTGGTATATATA	0.127	0.022	0.116	141	1073
GCTGGTGTAAGTA	0.401	0.04	0.417	485	678
GGCGTCGTATACA	0.074	0.007	0.074	108	1344
GGTATCGTTGGGG	0.125	0.024	0.117	39	295
GGTGTCACTTGTA	0.071	0.016	0.051	33	620
CCCGGTGCATGGG	0.021	0.004	0.028	22	772
CGCAGCGTTAGCG	0.025	0.002	0.026	26	957

CGTATCGTTTGTG	0.021	0.009	0.014	10	730
GGTGTTGCTAGCG	0.069	0.009	0.077	31	371
AGCGGTGCAAGCG	0.024	0.008	0.018	19	1055
GCTAGCATAGGGA	0.069	0.013	0.062	54	812
CGTGGTGCTGACA	0.022	0.004	0.028	25	879
CCTGGCGTAAGGG	0.025	0.005	0.017	11	627
CGCAGTGCTTACG	0.028	0.008	0.022	26	1159
AGCGGTGCTTGGG	0.028	0.005	0.021	10	459
AGTGTCACTAGCG	0.024	0.003	0.02	15	736
AGTGGCACATGTG	0.028	0.007	0.02	9	435
CGTAGCGTTGGCG	0.024	0.008	0.024	15	611
CGTGGTGTTAGCG	0.029	0.009	0.031	14	441
GGTAGCGTTAGCG	0.068	0.011	0.067	30	420
CGTATTGCAGACA	0.024	0.001	0.024	39	1618
AGTATCACTGGCG	0.013	0.001	0.011	13	1175
GGTAGTATTTAGA	0.062	0.005	0.063	63	941
GCTATTACAGATA	0.062	0.003	0.058	124	2003
GGTGGCATTTGCG	0.715	0.069	0.664	501	253
GCCAGCATTTGTA	0.044	0.006	0.036	64	1691
AGTGGTGCTGGGG	0.03	0.002	0.031	10	308
GGTATCACTAGCA	0.099	0.02	0.08	89	1025
CGTGTTACAGATG	0.021	0.008	0.015	16	1042
AGTGTTGTTGATA	0.019	0.003	0.021	25	1150
CGCGGTACTGGGA	0.031	0.006	0.03	25	810
CCCAGTACTTAGG	0.025	0.003	0.029	45	1491
CGCGTTGTTGAGG	0.023	0.008	0.02	15	731
GGCGTTATTAGTG	0.062	0.004	0.057	54	898
GGTGGTGCAAACG	0.094	0.015	0.091	62	623
CGTAGCATTAGGA	0.025	0.007	0.026	22	813
GGCGGCATTTGCG	0.553	0.072	0.544	485	407
ACCGGCGTAGGGG	0.032	0.011	0.027	14	499
AGTAGCACTTAGA	0.023	0.004	0.022	25	1130
GGTATTACTAGCA	0.081	0.023	0.061	71	1089
CGTGGTACTAAGG	0.023	0.011	0.01	7	680
CCTGTTGCTGGGG	0.03	0.002	0.028	15	529
CGTGTTATAGGCA	0.019	0	0.019	22	1160
AGCAGCGTATGGG	0.018	0.008	0.014	12	869
GGCGTTGTAAGTG	0.073	0.004	0.071	60	785
CCTGTTGCTGAGG	0.027	0.005	0.034	26	748
CGCAGTACTGACA	0.022	0.002	0.02	43	2156

ACCAGCGCTTGTG	0.027	0.003	0.027	36	1290
GCCATTATTGGTA	0.056	0.006	0.057	111	1853
GGCAGTGTTGGGG	0.06	0.009	0.052	23	418
CCTGTCGTTTGCG	0.021	0.003	0.017	15	891
AGCGGTACTAACA	0.021	0.003	0.023	59	2485
CCTGGTACTGGCG	0.027	0.005	0.025	21	826
CGCGTTGTTGGTG	0.027	0.005	0.029	18	607
GGTGTTGCTAAGG	0.086	0.013	0.068	30	410
GCTGTTGCTTACG	0.065	0.008	0.054	39	683
GCCGTCGCTTGGG	0.049	0.007	0.056	22	373
GGTAGCGCTTGTG	0.076	0.015	0.089	33	337
GGTGGTACATAGG	0.115	0.027	0.108	58	479
AGTGGCATTAATA	0.026	0.008	0.033	35	1014
CGCGTCACATGTG	0.023	0.001	0.022	26	1141
AGTGTCGTATGGG	0.026	0.004	0.03	17	549
GCTGTTGCATACG	0.06	0.004	0.058	52	847
CCCGGCACAGGGG	0.039	0.008	0.037	36	940
AGCGGCATATGTG	0.022	0.005	0.026	14	518
GCCGTTGTATGTA	0.044	0.003	0.047	58	1183
GCTGTTGTTAAGG	0.065	0.005	0.067	48	671
CCTAGCACAAGTG	0.031	0.004	0.031	47	1470
GCTGGCACTGGTA	0.226	0.031	0.249	199	600
GCTGTCGCTAGTA	0.066	0.008	0.057	35	582
AGTGTTGTAGGCG	0.019	0.008	0.01	7	701
AGTAGTGCTTGGG	0.027	0.004	0.023	9	376
GGCATCACTAAGA	0.071	0.006	0.062	116	1747
CGTAGTACTGGTG	0.015	0.004	0.016	9	562
CGCGTCATTGATG	0.026	0.006	0.019	24	1217
ACTGGTGCATGGA	0.025	0.003	0.027	27	959
CGTGTCATTTGGA	0.026	0.012	0.011	8	701
ACTGGCGTAAATG	0.029	0.006	0.037	30	780
AGTGTTATATGTG	0.024	0.008	0.025	26	1019
GCCAGTACATGCG	0.049	0.006	0.042	60	1363
AGCGGTGTTTAGA	0.019	0.001	0.017	18	1028
AGCGGCGTAGACA	0.021	0.004	0.019	32	1660
CGTATCGCTGGTG	0.032	0.003	0.03	17	551
GGCGTTATTTAGG	0.063	0.01	0.059	47	755
GCCGGTACTGGCG	0.076	0.006	0.083	71	782
AGCGGCATAAGTG	0.021	0.003	0.018	12	669
CCCAGCACATAGG	0.028	0.001	0.027	49	1794

AGCGTCGCAGGGA	0.025	0.002	0.023	22	944
ACTGGCGCATGGG	0.028	0.003	0.029	14	467
CCTGGTGTAGGCG	0.025	0.005	0.031	27	858
GCTAGTGCTGGTG	0.062	0.012	0.045	23	491
AGCGGTGTATGCG	0.021	0.004	0.026	25	930
CCTGTCGCATGCA	0.024	0.002	0.021	35	1608
CGCGTCATAGATG	0.023	0.001	0.023	34	1422
ACTGGCGTTTGGG	0.021	0.013	0.004	1	255
GCCAGTGTTAACG	0.05	0.008	0.043	67	1495
GGTGGCGCAAGGG	0.268	0.045	0.265	88	244
CGTGGCGCAGAGG	0.028	0.016	0.013	6	444
GCTATCATAGGCA	0.061	0.016	0.043	69	1542
GGCATTGCTTGGG	0.067	0.019	0.057	32	526
CCTAGCGTAGATG	0.023	0.003	0.024	34	1378
GCCGGCGCTTGTA	0.499	0.039	0.484	465	496
AGTAGTATATGGG	0.022	0.008	0.016	14	871
GGCGGTGCTTAGG	0.052	0.03	0.035	16	435
CCTAGCGTTGGGA	0.027	0.002	0.027	26	929
AGTATCGCATGGG	0.019	0.005	0.013	9	659
CCTGGCGTTAATG	0.027	0.006	0.022	23	1006
AGCAGCGCTAACG	0.022	0.003	0.023	39	1663
GCCATCGCTAATA	0.044	0.007	0.035	75	2052
CCTAGCGTAAAGG	0.022	0.002	0.02	29	1426
AGTAGTGCTGACA	0.03	0.004	0.035	47	1280
CCTGGCGCAGGGG	0.02	0.002	0.02	10	488
CCTGGCATTTGCG	0.025	0.005	0.022	19	836
GGTAGTACTGGCA	0.056	0.013	0.047	35	706
GCTGGCATTTACG	0.87	0.02	0.865	1313	205
CGTGGCGTTTATG	0.015	0.003	0.02	9	451
CCCATCGCTTGGG	0.033	0.006	0.025	25	978
CGTGGCATAGATA	0.023	0.003	0.02	22	1067
GCTGTTATTGATG	0.066	0.014	0.051	52	973
AGTGGCGTAAGCG	0.019	0.003	0.021	8	375
GCCGGCATTAAGA	0.396	0.017	0.387	738	1168
AGTATCACTGGCA	0.023	0.007	0.016	20	1197
GGTGTCATAAGCG	0.088	0.022	0.073	50	638
GGTGTCACAGGTG	0.057	0.005	0.051	24	449
GGTAGCGTTGGGG	0.08	0.011	0.065	19	273
AGTGTCGCATGTA	0.026	0.004	0.025	24	951
GCCGGCGCTAGGA	0.489	0.052	0.455	379	454

CGCGGCATTTGCA	0.019	0.003	0.02	23	1153
GCCGTCGCAAGCA	0.046	0.007	0.038	48	1227
AGTGGCGCTAGCG	0.03	0.011	0.015	7	456
GGTAGCACATAGG	0.052	0.017	0.031	20	621
GGTAGTGTTAGTA	0.058	0.019	0.044	29	636
GCTGTCGTAAGTG	0.07	0.008	0.059	39	622
GCTATTACATAGG	0.065	0.006	0.057	69	1140
GGTATTACATGTG	0.104	0.009	0.1	87	786
GGTATCGTTAGTA	0.137	0.018	0.117	107	808
CCTGTTGTTAGCG	0.028	0.004	0.024	26	1045
CGTGGCGTAGATG	0.024	0.009	0.023	12	515
GCTGGTGTAGAGG	0.749	0.065	0.737	553	197
GGCAGCGCATATG	0.061	0.008	0.05	51	968
GGTGGCGCTTGCA	0.186	0.05	0.164	82	417
GCTGTTACTAAGG	0.056	0.01	0.047	38	769
CGTGGTACAAATA	0.025	0.007	0.018	32	1773
CCTGTTGCTTGGG	0.029	0.011	0.015	9	588
GCTAGTACAGGGG	0.06	0.003	0.064	33	485
CCTGTCATAGGGA	0.039	0.007	0.043	50	1122
GGCGGCGTTTGTA	0.194	0.027	0.193	122	511
CGCAGTATTTGGA	0.027	0.003	0.028	36	1228
AGTGGCGCAGAGG	0.019	0.005	0.019	8	414
GCCGGTGCTGGTA	0.058	0.011	0.042	27	612
ACTATCGCTTATA	0.031	0.005	0.029	75	2533
GGCGGCACTGGGA	0.132	0.034	0.093	68	666
AGTGGTGCTGACG	0.03	0.011	0.016	10	618
GCTGTCGCAAGTA	0.049	0.001	0.049	40	782
CCTGGTATTGGGA	0.03	0.002	0.031	25	776
ACCGGCGTAGGCA	0.019	0.003	0.018	23	1266
GCTGTTATAGGGA	0.063	0.022	0.056	44	741
GGTGTCGTTGGCA	0.084	0.005	0.086	42	446
CGTGTCGTTAGGG	0.022	0.002	0.019	8	414
GGCGTCGTAGGGA	0.05	0.006	0.051	38	710
AGCAGCGCTTGCA	0.024	0.008	0.034	49	1385
GGCATCGCTTACG	0.122	0.022	0.092	104	1032
GGTATTATATGCG	0.115	0.029	0.09	90	911
CGTGTTGCTTATA	0.019	0.003	0.016	15	930
CGCGTTGTAGATA	0.025	0.005	0.019	29	1533
ACTGGTGCATGTG	0.022	0.009	0.025	22	866
GCTGGCATTTGTA	0.63	0.048	0.635	786	451

GGTATCGTATACA	0.142	0.024	0.13	191	1283
AGTATCGCATGCG	0.024	0.005	0.018	18	978
GGTAGCGCATGGA	0.069	0.011	0.058	26	426
GCCGTTGCTAAGG	0.043	0.007	0.033	26	763
GGCGTTACTTGCG	0.067	0.008	0.064	46	674
AGTATCACAAGTG	0.024	0.007	0.019	22	1155
AGTAGCGCAGACA	0.025	0.001	0.026	38	1429
AGTGGCGTTTACG	0.017	0.011	0.006	2	360
GCCATCGCTTGTG	0.066	0.007	0.06	47	740
ACTGGCGTAAGCA	0.025	0.005	0.022	28	1220
CCTGGTGCTTATA	0.021	0.003	0.017	25	1452
CGTAGTACTTAGG	0.033	0.013	0.016	10	624
GCTATTGTTAGGA	0.049	0.01	0.035	38	1048
CCTGGTGCTGGCG	0.04	0.016	0.031	21	661
GGCAGTGCTAGGG	0.047	0.002	0.047	25	510
ACTGTCGTATGGA	0.027	0.007	0.036	33	875
GCTGTTACTGGTA	0.064	0.003	0.059	47	748
GGTGTCACTAAGA	0.079	0.022	0.071	65	856
AGCATCACTAGGG	0.027	0.004	0.029	41	1390
GGTGGTGCTAAGG	0.112	0.01	0.097	38	352
AGTAGTATTAGGG	0.024	0.008	0.021	17	782
CGTAGCGTTAACA	0.02	0.002	0.022	31	1372
CCCGTTGCTAAGG	0.023	0.004	0.023	30	1259
CCTGTCATATGCG	0.028	0.004	0.024	32	1320
CGTAGTATATGCG	0.019	0.004	0.023	21	896
AGTATTGCTGAGG	0.023	0.003	0.026	21	781
ACTGTCGTTAGTA	0.023	0.001	0.025	30	1185
CCTGGCGCATGTG	0.024	0.006	0.028	23	807
GCTAGTGCAGAGA	0.049	0.005	0.051	53	987
GCCAGTGTAAGCA	0.046	0.001	0.046	88	1820
CCTGTTGTAGGGG	0.04	0.007	0.037	25	653
GCCGGTGCAAGTA	0.064	0.004	0.059	66	1053
GGTGGCGCATGGA	0.159	0.038	0.133	49	320
GCTAGCGTTGACG	0.073	0.008	0.077	68	810
GCTGGTGCAAGGG	0.279	0.018	0.256	122	355
GCCGGCATTTAGG	0.735	0.036	0.728	848	317
GGCGTCGCTTAGG	0.085	0.025	0.091	48	482
AGCGGCGCTGGGA	0.021	0.003	0.018	12	668
CGTAGCATTAGGG	0.028	0.001	0.027	15	547
GGTAGCGTAGACG	0.074	0.012	0.075	50	613

GGCGTTGCTGGTG	0.056	0.018	0.047	23	465
AGTGGCGTATGTA	0.02	0.008	0.03	21	680
GGCGGCATAAATG	0.565	0.05	0.552	1092	888
GCTGGCATTGATG	0.869	0.017	0.863	1187	188
AGTGTCGCAGAGG	0.023	0.005	0.026	15	573
GCTGGCACAAGCA	0.21	0.019	0.212	285	1060
GCCGTCGTAAATA	0.052	0.004	0.047	88	1772
AGTGGTGTATAGG	0.025	0.014	0.014	8	552
GCTATTGCAGAGG	0.057	0.009	0.066	55	781
GCCGTTATAGACG	0.057	0.008	0.046	76	1575
ACCGGTGCTTAGA	0.023	0.004	0.02	31	1505
AGTGGCACAAACG	0.026	0.004	0.03	31	1007
GCTGGCGTAGGTG	0.947	0.013	0.941	915	57
GGTAGTGCTGGTG	0.047	0.011	0.061	22	339
CGTAGCGTATGCA	0.021	0.002	0.018	18	985
CGCAGCGCAAGGA	0.025	0.002	0.027	34	1206
CGTATTGCAGGCG	0.028	0.006	0.027	27	969
GGCATTGCAGGTA	0.051	0.003	0.056	59	1004
GGTGGTGTTGGTG	0.206	0.05	0.136	42	267
AGTAGTGCAAGGG	0.023	0.008	0.014	9	637
GCCGTTGTTGAGG	0.053	0.01	0.046	34	712
GGCAGTGCTGGCG	0.045	0.007	0.041	34	804
CGCGTTACTGGTG	0.021	0.003	0.025	21	836
GGCGTTGCTAATA	0.058	0.013	0.053	71	1276
AGTGGCATAAGCG	0.033	0.004	0.027	8	288
GGCGGCATTGGTG	0.525	0.078	0.499	405	406
AGTGGTGCTAGGA	0.028	0.011	0.037	23	597
ACCGGCGTAAATG	0.034	0.001	0.033	36	1063
CCCATCACATGGA	0.027	0.005	0.022	52	2362
GGCGGTACTGACG	0.076	0.006	0.072	65	841
ACCGGCATTGATG	0.026	0.002	0.023	37	1550
GGCGGCGTTAACG	0.47	0.052	0.476	496	547
ACTGGTGCTTGCG	0.03	0.002	0.032	24	734
AGTATCACTTGGA	0.022	0.007	0.024	22	905
CGTAGTACTAGCG	0.02	0.004	0.025	19	749
GGTATTATTTGGG	0.108	0.015	0.088	50	518
CCCGGCATTGGCG	0.023	0.003	0.02	24	1190
GGTAGCATATATG	0.074	0.011	0.068	65	885
CGCGGCACAGGGA	0.025	0.003	0.021	19	895
GGCGGTGCAGATA	0.044	0.004	0.049	51	989

GCTGGTACAGGGA	0.078	0.005	0.085	53	569
AGTAGCGTTAAGG	0.026	0.006	0.017	14	810
GGCGTTGTTTGTG	0.07	0.012	0.071	42	553
CGTAGTACATGCG	0.023	0.001	0.022	17	766
GGCGGTACTAGTA	0.069	0.004	0.064	64	941
GGTATCATTAATG	0.12	0.016	0.112	117	929
AGCAGCACTTATG	0.021	0.003	0.02	33	1607
AGTAGTGCTGGTG	0.018	0.003	0.022	11	496
GGTATTGCTAACG	0.099	0.02	0.085	82	886
CGCGGTACAAACA	0.025	0.001	0.026	75	2759
AGTAGCGTAAGTG	0.021	0.001	0.021	18	843
GGCATCATTAGCG	0.097	0.008	0.094	124	1196
CGTGTTGTAAACA	0.018	0.002	0.017	29	1674
ACCGGCGCAGAGG	0.023	0.004	0.02	19	923
AGCGGTACTTGCG	0.019	0.004	0.015	15	1010
AGTGTTGCTTGGG	0.029	0.011	0.021	8	373
CGTGGCACAAATA	0.025	0.003	0.029	49	1663
CGCGTCACAGAGG	0.028	0.002	0.025	27	1038
CGTGGTACATGGA	0.026	0.008	0.015	11	733
GGTAGCACATGGG	0.075	0.008	0.084	38	415
AGTATCGTTGGGA	0.022	0.001	0.021	17	802
CGTGGCACTAGCG	0.022	0.009	0.014	8	569
GGTAGCATAGGGG	0.045	0.011	0.031	17	534
GGTATCATTAGGG	0.122	0.011	0.11	57	462
GCCAGTGCTGAGA	0.033	0.005	0.026	26	983
CGTGTCACTAGCA	0.019	0.003	0.021	22	1050
AGCGGTGCTGAGA	0.02	0.005	0.017	18	1038
GGTGGCACTTAGG	0.385	0.047	0.341	156	302
ACCAGTACTTGGG	0.018	0	0.018	29	1552
GGCATCGCTGGGG	0.087	0.016	0.065	40	579
GGTAGTGCATAGG	0.063	0.002	0.065	31	446
CGTGGTACTTAGG	0.029	0.004	0.031	17	526
GCCGGCATTGAGG	0.724	0.051	0.748	920	310
CGCGTCGTATAGA	0.024	0.006	0.021	29	1368
GCTGGCACAAACA	0.213	0.018	0.209	431	1629
CCTGGCATTAGGG	0.031	0.009	0.028	19	651
CGTGTCGTTGACG	0.027	0.008	0.037	31	806
CCTGGCGTAGAGA	0.024	0.007	0.015	17	1107
CCTATCGCTGGGA	0.025	0.001	0.024	29	1174
GGCGGTGCTAGGA	0.056	0.006	0.064	37	538

AGCAGTATTGGGG	0.024	0.008	0.033	33	977
GCCGGTGTAGATG	0.391	0.053	0.374	366	613
CCTGTTGTAAGCG	0.03	0.008	0.02	27	1307
CGTGGCATTAGTG	0.026	0.006	0.027	11	393
GCTGGTATTGACG	0.446	0.052	0.432	450	591
GCCAGTACTGAGG	0.054	0.005	0.059	63	1007
AGTAGCGCAAAGG	0.023	0.002	0.024	23	933
CCTGTTGTTTGTA	0.02	0.002	0.021	23	1098
GGTAGCATAGGCG	0.049	0.014	0.029	18	599
GGCGTCGCTTGTG	0.087	0.011	0.071	40	520
GCTGGCACTAATG	0.44	0.028	0.442	517	652
ACTAGTGTTAAGG	0.031	0.005	0.035	54	1506
CGTATTACTAACG	0.019	0.001	0.018	30	1655
CGCGGCGCTTAGA	0.022	0.004	0.02	18	865
AGCATCGTTTGGG	0.019	0.007	0.014	14	992
CGTGGCGCTAACA	0.03	0.004	0.028	30	1058
AGCGTCATTTGGG	0.022	0.004	0.023	18	782
ACTGGCGCTAACG	0.027	0.006	0.02	21	1037
CGCGTTACTTGTA	0.024	0.003	0.027	37	1331
GGTATTGCAAAGG	0.096	0.017	0.073	64	807
CCCGGCACTGGTG	0.02	0.004	0.014	16	1113
AGTATTGCAAGTG	0.031	0.006	0.032	36	1081
ACTGTCGTATGTA	0.03	0.004	0.031	38	1171
ACCATTGTTTGGG	0.024	0.001	0.026	29	1071
CGTGGCGTTGAGA	0.037	0.009	0.027	15	531
CGTGGCACTAGTA	0.033	0.009	0.021	19	870
AGTGGCGCAGGTG	0.023	0.011	0.039	17	420
GGCGGCGTTGGCG	0.398	0.045	0.396	257	392
ACTGTCATAGGGA	0.029	0.003	0.027	30	1068
GGCATCGCTGGTG	0.099	0.007	0.09	62	630
CCCGGCACTGGTA	0.023	0.005	0.017	27	1549
AGTAGTATAGGCG	0.029	0.007	0.02	21	1042
GGTAGCGCTAGGA	0.062	0.014	0.045	21	448
CGCAGTACTTACG	0.02	0.002	0.022	32	1408
GGCGGCGTAAGGA	0.184	0.039	0.164	128	652
GCCGGTGCATGTG	0.108	0.021	0.126	83	574
ACTGGCATAGGGA	0.033	0.006	0.025	23	903
GCTAGCATATAGA	0.072	0.014	0.055	84	1448
CGTATTATTAGCG	0.023	0.005	0.019	24	1213
CCTGGCGCTAGGG	0.021	0.01	0.016	9	558

GGTAGCACTAGTG	0.072	0.011	0.058	32	518
GCTGGTACATGTG	0.099	0.017	0.076	51	620
GGTGTCGCTGACA	0.084	0.028	0.068	55	753
GCCGGCATTGGGA	0.333	0.047	0.289	260	639
GGCGTCGTATATG	0.068	0.009	0.055	49	838
GGTATTGCTGGTA	0.082	0.016	0.07	44	584
GGTAGCACTTGCA	0.063	0.006	0.059	42	666
GGCGGTGCTTATA	0.043	0.009	0.044	38	835
GGTGGTGCAAAGA	0.075	0.009	0.085	62	666
GGCGGTGTAGGTG	0.099	0.016	0.08	40	463
GGCGGTGCTTGTG	0.065	0.012	0.079	34	397
ACTAGTGCTAACA	0.026	0.001	0.025	84	3337
GCTAGCACAGGTG	0.066	0.006	0.062	50	754
GCTGGCGCTAATG	0.817	0.036	0.798	896	227
CGTGTTGCTGATG	0.037	0.007	0.03	20	649
AGTGGTACTTGGG	0.018	0.009	0.015	7	449
CGTATTACTGGCG	0.032	0.006	0.039	48	1190
GCTGGCGTAGACA	0.878	0.019	0.878	1729	241
AGTGTCATTAAGG	0.026	0.004	0.028	26	895
GCTGGCATTGAGG	0.863	0.018	0.863	882	140
AGTGGCGTTGGGA	0.021	0.006	0.018	7	379
AGTAGCGTTGACG	0.02	0.004	0.019	18	918
AGTGTCATTTAGG	0.016	0.001	0.017	13	772
GGTGGCGTTTGTA	0.382	0.055	0.355	188	342
AGTGGTGCTTACA	0.022	0.002	0.022	22	996
CGTGGTATAGGTA	0.018	0.004	0.02	17	833
GCCGTCATTTGGA	0.06	0.006	0.056	59	1001
AGTGTCGTTAACG	0.018	0.005	0.026	28	1058
ACTGTCATTGAGA	0.027	0.002	0.028	47	1656
GGCGTTGCTGGTA	0.066	0.009	0.056	35	588
GGTGGCACAAATG	0.407	0.059	0.373	414	697
GGCAGTGCAAGCA	0.054	0.008	0.047	63	1281
CGTAGCGTTAATG	0.024	0.005	0.022	19	851
CCTATTACTGGCG	0.029	0.009	0.022	31	1400
GGTGTTATTGAGG	0.098	0.01	0.101	50	446
GGCGTCGTTTATA	0.076	0.015	0.061	68	1039
ACTGGTATTGGGA	0.031	0.008	0.022	19	845
CGTAGCATTGAGA	0.022	0.005	0.029	30	1006
ACCGTCGTTGGGG	0.025	0.005	0.018	10	538
CCTGTTGTTTGTG	0.024	0.001	0.026	22	827

GGTGGTATTGGTG	0.268	0.068	0.219	95	339
GGCAGCATTGGCG	0.052	0.008	0.046	39	808
GGCAGCGCTAATA	0.06	0.001	0.06	91	1414
CGCAGCGCAGAGG	0.023	0.006	0.031	27	841
ACCGGTGTTGACG	0.026	0.002	0.026	33	1242
CGCGGTATAAGTA	0.026	0.002	0.029	53	1780
GGCGGTGCAGGTG	0.058	0.009	0.071	38	494
AGTAGTGTTAAGG	0.035	0.01	0.027	26	941
GGCGGCACAAGTG	0.276	0.049	0.239	231	737
CCCGTCGCAGGTG	0.026	0.001	0.027	30	1068
GGTGGCGCTAGCA	0.14	0.019	0.142	77	464
CGTGTCGCTGAGG	0.031	0.005	0.025	13	516
GCTGTCACAGGCG	0.056	0.003	0.052	42	769
CCCGGTGTTGGGA	0.02	0.006	0.018	15	823
CGTGGCATAGAGG	0.022	0.009	0.011	5	436
AGTGGCGTTAAGA	0.022	0.005	0.017	12	698
CGCGTTATATGGA	0.022	0.002	0.025	34	1346
GGTAGTGTAAACA	0.062	0.003	0.06	82	1279
GGTATTATAGACG	0.12	0.016	0.115	149	1146
AGCGTCGTTTGTA	0.022	0.003	0.021	25	1158
GGTGGCACTAGGA	0.182	0.046	0.164	85	434
GGCGGTGCAGACA	0.053	0.012	0.047	52	1055
CGCGGTGCTAGGG	0.022	0.001	0.024	13	528
GCTGTTGTTAGGG	0.074	0.012	0.059	27	434
GGTGGTGTATACG	0.209	0.022	0.205	129	499
GGTGTCACATGTA	0.078	0.001	0.078	60	714
CGTGGCGTTGACG	0.031	0.007	0.032	16	483
AGTGGTGCATGCG	0.035	0.006	0.033	19	552
GCTGGCGCAGGCA	0.609	0.044	0.613	695	439
CCTAGCACATAGG	0.031	0.011	0.019	25	1281
GGTATCGCTAGGG	0.136	0.046	0.086	33	349
CGCATCGTTAGGA	0.029	0.005	0.033	45	1315
GCCGTTGTAGGGG	0.05	0.005	0.057	29	482
AGTATCGTAGGTG	0.024	0.004	0.019	16	832
GCTAGTATATGTG	0.058	0.003	0.06	66	1039
CGCGGCGCAGGGA	0.024	0.001	0.023	16	689
CGTGTTGCTTGTG	0.023	0.006	0.027	14	500
CGTGTTGTTTAGA	0.019	0.004	0.023	18	749
CGTGTTATTGGCG	0.02	0.006	0.018	14	781
GGTAGTGCTGAGG	0.073	0.01	0.065	26	374

GGTGTTGCATGGA	0.077	0.022	0.066	35	494
GGCAGTACAGGGA	0.052	0.007	0.043	43	950
CCTATTATAGGTG	0.031	0.007	0.02	33	1590
CGCGGTATATAGG	0.019	0.005	0.013	13	973
CGTAGCGCTTATG	0.022	0.004	0.017	12	691
GGTGTCGCATGCG	0.079	0.005	0.072	36	465
AGCGTTGTTAAGG	0.023	0.002	0.021	24	1106
GGCAGCGCAAATG	0.051	0.001	0.05	67	1275
CCTGGCGTATGTG	0.033	0.009	0.028	21	728
AGTATTACTGGCA	0.022	0	0.022	31	1381
GCCATCGTAGGGG	0.054	0.001	0.053	41	731
AGTGTCGTTAGGA	0.03	0.005	0.031	21	662
GGCGGCGCAGAGA	0.103	0.007	0.102	72	637
CCTGTCGTATGGG	0.023	0.003	0.026	20	744
GGCGTTACTTATG	0.059	0.01	0.065	62	891
CGTAGTGCTTGGG	0.021	0.008	0.032	10	301
CGTAGTATTAGGG	0.022	0.006	0.015	9	608
GCTAGTGCTGGGG	0.056	0.006	0.052	23	421
GGCGGTACTGGTA	0.053	0.004	0.058	49	798
CGTGGCACATGTA	0.024	0.005	0.022	20	891
AGTGGTATATGGA	0.026	0.003	0.027	24	871
ACCATCACATGGG	0.025	0.007	0.025	39	1494
AGCATTGCTTGGG	0.016	0	0.017	15	886
GGTAGCATTTATG	0.092	0.012	0.078	61	717
GGTGGTGCAAAGG	0.118	0.027	0.111	48	384
CGCGTTATTAGGG	0.022	0.011	0.013	10	782
GGTGGCGCAGGCG	0.293	0.042	0.261	109	308
GCTGGCGTTTGGA	0.873	0.026	0.866	753	117
CGTATCGTATATG	0.03	0.003	0.026	34	1272
GGTGGCACTAGTG	0.369	0.07	0.335	203	403
CGCGTTACTGGGG	0.027	0.01	0.014	9	614
CCTGGTGCAAGGG	0.028	0.009	0.023	16	683
CGTGTTGTTGGGG	0.015	0.004	0.011	4	362
AGTGTCGTAGGCG	0.026	0.003	0.023	14	608
AGTGGCGTTTGGG	0.021	0.008	0.03	6	195
GGCATCGTTGGGG	0.089	0.015	0.084	52	569
GGTGGCACTAGCA	0.189	0.033	0.179	131	599
CGCGTCGTTTAGA	0.024	0.005	0.025	27	1033
AGTGTTGCTTGCA	0.024	0.005	0.017	14	800
GCTGTTATTTGTG	0.062	0.009	0.051	38	708

AGCGTTGTTGGGG	0.02	0.006	0.028	17	586
GGTGTTGTATATG	0.097	0.023	0.074	51	640
AGCGGCGCTGATA	0.022	0	0.023	30	1291
AGTGGCGTTGGCA	0.028	0.006	0.025	14	548
AGTGTCGTATACG	0.024	0.006	0.025	27	1052
CGTGGCATATGCG	0.025	0.012	0.022	11	493
GCTGTCGCTGGTG	0.065	0.015	0.045	17	357
CCTAGCATAGAGA	0.023	0.001	0.022	41	1805
GGTGTCGTATACA	0.109	0.009	0.102	97	855
AGTGGCATTGAGA	0.025	0.008	0.015	8	541
GGCATTGCTTATG	0.069	0.003	0.069	70	938
CGTGGTATTGATA	0.026	0.002	0.029	33	1109
GGTAGTACAGGTG	0.068	0.013	0.061	34	525
AGTGGTGCTAAGA	0.022	0.002	0.024	22	912
AGCGTCATTAAGG	0.027	0.003	0.023	32	1378
AGTGGCATTGACG	0.03	0.017	0.042	13	299
GGTAGTGCAGATG	0.07	0.007	0.077	45	537
CCTGTCGCAGGTG	0.025	0.004	0.02	17	828
GCTGTCGCTGACA	0.046	0.003	0.05	47	895
GGTGTTGCTTATA	0.076	0.017	0.071	53	689
GGTGGCGCAGGTA	0.16	0.013	0.171	79	382
GCTGGCGTTAGTA	0.89	0.027	0.889	1085	136
GGTGGTGTAGGGG	0.121	0.038	0.076	26	315
GCCAGTGTTTAGA	0.043	0.003	0.047	58	1184
GGCGGTGCTGGGA	0.059	0.016	0.044	23	501
AGTGTTATTGGCA	0.023	0.002	0.023	26	1115
GCCGGTGTTTACA	0.162	0.043	0.125	173	1210
AGCGTTGCTAAGG	0.022	0.004	0.017	18	1067
AGTGTCACAGGTA	0.025	0.007	0.017	16	931
CGTGGCGCTAGCG	0.02	0.008	0.02	10	485
GGCGGCACAAACG	0.266	0.045	0.235	385	1253
CCTGTTACAGGGG	0.049	0.013	0.03	22	712
GGTGGTATAAGCG	0.285	0.031	0.282	210	535
GGTAGCATAGATA	0.07	0.014	0.056	71	1194
CGTGTTGCTTGGG	0.029	0.007	0.022	8	354
CGTGGTGTTAGTG	0.03	0.011	0.039	16	399
GCCGTTGCATGTG	0.047	0.004	0.049	38	731
GCTGGCATAGGCG	0.849	0.04	0.859	1004	165
GCTGTTGCAGAGG	0.062	0.003	0.066	36	511
GGTGTCGCTTATG	0.104	0.016	0.083	40	440

GCTAGTACATGGA	0.071	0.002	0.073	72	917
GCCATCGTAAGTG	0.053	0.01	0.039	56	1377
GGTAGTATAAGGG	0.069	0.005	0.063	44	653
AGTGTTGCAGAGA	0.019	0.003	0.015	14	907
AGCGTCGCTGAGA	0.02	0.001	0.019	25	1308
AGTGGTATTTGCG	0.015	0.001	0.014	8	544
CGTGTTACTAGGG	0.024	0.001	0.022	13	575
GGTGGTGCATATG	0.113	0.014	0.102	53	465
GCCGGCGCATGGG	0.749	0.035	0.714	595	238
ACTAGCGTTAGCG	0.033	0.004	0.036	36	967
GGCAGTACTAGGG	0.058	0.006	0.05	41	780
AGCGGTATAAGGG	0.026	0.005	0.019	21	1060
CCTGGCGCAGGTG	0.025	0.005	0.028	19	668
AGTGGTACTAGTG	0.025	0.004	0.021	14	646
AGCGGCGCAGAGA	0.024	0.004	0.023	23	978
GGCGTTACAAAGG	0.07	0.001	0.071	72	943
AGTGTTACTGGGA	0.024	0.006	0.028	20	692
CGCGTCACAGGGA	0.029	0.007	0.035	35	979
CGTAGTATTAGTG	0.024	0.007	0.034	29	822
GCTGGCGTAAGCA	0.878	0.034	0.883	1542	205
GGTGGCGCTTAGG	0.332	0.067	0.319	120	256
GGTGTCATTTGTA	0.086	0.02	0.058	38	618
CGTGTTGTATGCG	0.027	0.003	0.029	18	610
CGCATCACTAGCG	0.023	0.002	0.021	36	1690
CGTGGCATTGAGG	0.024	0	0.024	9	371
AGCGGCGTTGATG	0.021	0.009	0.029	21	692
CCTGTCGTTGGTG	0.029	0.001	0.028	20	687
GGCGTCACTTGGG	0.074	0.009	0.086	51	543
GGTGTTGTATACA	0.083	0.024	0.074	71	889
CGCGGCGTTGGCG	0.02	0.001	0.019	12	618
AGCGGCGCTAAGA	0.019	0.006	0.025	31	1207
GGTGGTGCAAGCG	0.09	0.008	0.083	38	422
ACTAGTGTTGGTA	0.02	0.003	0.019	26	1359
CGCGTTGTAAGTG	0.021	0	0.021	22	1015
GCTGGTACAAGTG	0.128	0.01	0.138	104	648
AGTGGCGCTTAGA	0.024	0.01	0.013	8	585
GCTAGTGCTAGCA	0.047	0.007	0.043	46	1025
CCTGTTGCATGTG	0.025	0.006	0.032	34	1032
GCTGTCATATGTG	0.062	0.006	0.054	45	795
AGCGGTACTTGGG	0.026	0.005	0.029	19	626

GGTAGCATTTGGA	0.088	0.011	0.073	45	574
GGTGTTATTTATG	0.104	0.024	0.118	100	745
GGTATCACTGGCG	0.099	0.017	0.08	63	725
CCTAGTGTTGAGA	0.022	0.002	0.02	28	1389
GCTATCGCATGCG	0.05	0.003	0.046	37	766
GGTAGTACTGATA	0.055	0.003	0.059	64	1018
GGTATCGTAGATG	0.142	0.008	0.134	103	665
CGTGGTATTTGCG	0.029	0.01	0.015	8	516
GCCAGTGTTGGCG	0.044	0.003	0.048	55	1086
AGTGGTACTTACG	0.03	0.009	0.043	34	757
CGTAGTGCTTACG	0.027	0.003	0.023	14	584
GGCATCGTAAGGG	0.105	0.012	0.09	88	893
GCTGTTGTAGATG	0.054	0.003	0.051	44	823
CGTGGCGTAGGTG	0.026	0.005	0.03	12	387
GGCATCATAGGTA	0.084	0.004	0.088	151	1560
ACCATCGCATAGG	0.027	0.007	0.018	31	1688
CGTATTGCTGGCA	0.021	0.005	0.023	22	939
GGCAGTACATGCG	0.057	0.002	0.053	61	1084
GCTAGTACTGGGA	0.068	0.009	0.056	39	658
AGTAGTGCATGTG	0.022	0.004	0.021	15	704
GGTATCGCAGACG	0.142	0.029	0.12	93	679
CGTGGCGCAGGCG	0.031	0.006	0.023	12	512
AGCGGCGCATATG	0.024	0.003	0.023	25	1060
GCCAGCACATAGG	0.056	0.007	0.053	66	1185
GGCATCGTTTAGA	0.104	0.011	0.097	115	1068
CGTGGTGCTAGTG	0.028	0.002	0.024	10	402
CGTGGTACTGGTA	0.021	0.003	0.019	13	672
GCCGTCGCTGAGG	0.042	0.016	0.044	24	526
GGCGTTACTGGGA	0.059	0.008	0.052	39	704
GGCATCGTAGGTA	0.09	0.007	0.083	100	1106
GGTAGCGCTAAGG	0.07	0.016	0.05	25	472
GCTATTGCAAAGA	0.049	0.006	0.042	74	1686
GCCGGCACAGACA	0.109	0.003	0.112	222	1758
CGCGTTGCTGAGA	0.02	0.004	0.022	22	985
CCCGTCGCAAGGG	0.021	0.002	0.019	23	1168
CGTGTCGCTTGTA	0.019	0.004	0.014	10	700
GCTGGTACATGCA	0.086	0.001	0.088	92	959
GCCATCGTAAAGG	0.058	0.007	0.048	72	1423
AGTGTCACAGGGG	0.029	0.007	0.029	15	496
GGTATCGCTAGCA	0.096	0.008	0.102	90	793

GGTAGCGCTAATA	0.073	0.001	0.074	71	894
CGCAGTACATGCA	0.023	0.002	0.022	40	1804
CGCGTCATTAACG	0.027	0.007	0.017	30	1728
AGTATCGCTTGGA	0.026	0.004	0.02	16	772
GGTGGCGCTTATG	0.324	0.04	0.301	143	332
GCTAGCGCATAGG	0.053	0.016	0.03	16	512
CCTGGTGCTAAGG	0.024	0.007	0.021	17	805
CCTAGTGTTTGGA	0.02	0.007	0.018	20	1076
GGTATCGCTGACG	0.135	0.029	0.108	88	729
GGCAGCATTTGTG	0.055	0.001	0.054	48	839
GCTGGCATTGAGA	0.64	0.033	0.65	792	426
GGCAGCACTGGTG	0.046	0.007	0.039	28	696
CGCATCATTGGGG	0.039	0.003	0.036	30	804
GCTGGCGTTTGCG	0.94	0.013	0.937	835	56
GGTGGTGCATACG	0.08	0.017	0.066	36	507
CGCATCGCAGAGA	0.027	0.006	0.029	45	1516
CGTGTCGCTTGGG	0.031	0.008	0.039	14	348
CGTGTCGTATATG	0.025	0.005	0.019	16	822
GGCGTTGTTAATG	0.08	0.017	0.081	76	868
GGCGGTATAGAGA	0.072	0.004	0.068	74	1015
GGTGGTGTTTATA	0.096	0.013	0.086	59	625
CGCGGTACTAGCG	0.027	0.002	0.027	26	943
GCTAGTACTAGTG	0.065	0.011	0.052	46	843
GCCAGTGCTGAGG	0.046	0.004	0.051	37	682
AGCGGCACAGGGG	0.028	0.012	0.044	26	562
GGCGTTGTTGGTG	0.071	0.009	0.073	38	483
AGCGGTACATGGA	0.024	0.007	0.016	21	1264
CGTAGCACTAGGG	0.021	0.005	0.026	15	556
AGCGTCGCTGGGG	0.019	0.003	0.021	13	602
GGTGTCATAGGCG	0.094	0.028	0.096	53	500
GGCAGTGCTAGCG	0.052	0.012	0.053	41	734
AGTGTCGTAAGGA	0.018	0.001	0.018	15	838
GGTGTCGCAGATA	0.075	0.016	0.06	45	710
GGCAGTGTTTAGG	0.057	0.007	0.048	33	650
GCTGGCATTTAGA	0.644	0.022	0.649	811	439
AGTGTCATTTGGA	0.023	0.004	0.017	13	752
CGCGTTGTTGGCG	0.021	0.003	0.022	19	835
GCTGGCGCTGAGA	0.624	0.043	0.658	574	298
GGCAGTGTTGGGA	0.062	0.017	0.049	34	657
GGTGTCGTTGGGG	0.113	0.024	0.081	20	226

AGTGGCGCAGGCA	0.02	0.001	0.018	13	690
GGTGTCATATGGG	0.079	0.02	0.092	33	326
GGCGGTACATGCG	0.071	0.01	0.074	59	739
ACTGGCACAGGGG	0.024	0.005	0.017	11	622
GGTATCGTATGGA	0.124	0.009	0.127	89	612
CGTGTCATTTGGG	0.028	0.004	0.023	12	514
GGTATTGCTTGCA	0.079	0.018	0.063	51	763
GGTAGTGCTTACA	0.063	0.002	0.06	53	825
CGTGTTGTTTGTA	0.029	0.008	0.025	17	674
AGTGTTGCTTATG	0.021	0.009	0.009	7	746
GCCGTCGCTTATG	0.055	0.013	0.04	27	655
GCTGGCACTGACG	0.446	0.059	0.429	444	591
CCTAGTGTTTAGG	0.028	0.004	0.024	26	1050
CGTGTCGCTAGTG	0.03	0.007	0.033	19	558
AGTGGTGCTTATG	0.031	0.008	0.026	14	530
GCTATTATTTAGG	0.068	0.008	0.062	73	1106
CGTGGCATTAATG	0.022	0.004	0.028	18	628
CGTATCACTTGCA	0.024	0.003	0.02	23	1134
GCCATTGCTAGCA	0.044	0.006	0.037	55	1419
AGCGGTGCTAGGG	0.02	0.009	0.008	5	623
GCCATCGTTTGGA	0.047	0.004	0.045	45	962
CGTATTGCTAGCG	0.026	0.004	0.02	16	775
GCTGTTATTGGGA	0.063	0.009	0.056	42	709
ACTGGCGTTTACG	0.03	0.005	0.023	12	512
GGTGTTGCAAAGA	0.065	0.017	0.044	39	842
AGTGTCGCATGCA	0.025	0.006	0.026	26	963
GCTGTCGCAAAGA	0.064	0.012	0.053	48	865
GGCGGTGTATGCA	0.063	0.008	0.055	53	918
GGTGTCACTTACG	0.096	0.013	0.088	59	614
AGTAGCATAGACG	0.024	0.002	0.027	38	1377
CCCGGTACTGGGG	0.041	0.007	0.034	25	717
CGTATTATTGGTG	0.022	0.004	0.018	17	952
ACTGTCGCAGGGA	0.026	0.002	0.028	28	956
GCCGGCACTGGCG	0.188	0.008	0.176	155	726
GGTGGTGCATGGG	0.106	0.027	0.076	19	231
CCTGGTGCTAGGG	0.032	0.005	0.034	18	516
GGTGGTGTTTAGG	0.192	0.046	0.239	85	270
AGCGTCGCAGGGG	0.032	0.007	0.037	27	698
GGTAGCGCAAGCA	0.07	0.008	0.06	50	781
AGTGGCGCTTGCG	0.015	0.009	0.016	7	444

CGCGTTGCTGAGG	0.025	0.005	0.024	16	657
CGTGGCGTTTATA	0.024	0.007	0.016	12	720
GGTGTCATTGGGA	0.11	0.007	0.104	50	430
ACCGGCGCTTGGG	0.028	0.006	0.02	13	622
CGCGGCACTGGCG	0.022	0.003	0.02	17	837
GCCGGTGTAAGCG	0.396	0.05	0.391	423	660
CCCGTCGTATGGA	0.022	0.002	0.021	29	1328
GGTGGCGTTTAGG	0.642	0.068	0.638	354	201
CGCATTATTTGTG	0.028	0.01	0.019	25	1314
CGCGTTATTGGGG	0.032	0.007	0.024	13	533
CCTGGCGCAAAGA	0.025	0.007	0.017	25	1487
GGTGGCGCTGGGG	0.323	0.061	0.38	93	152
GGTAGTATAGGCG	0.046	0.004	0.044	30	657
GCCGTCATTTGGG	0.058	0.01	0.05	31	586
ACTGTTGTTGGCA	0.022	0.003	0.025	29	1145
GGTAGTATTTGCG	0.06	0.01	0.06	37	579
AGTGTCGCATGGG	0.013	0.004	0.018	9	500
GGTGGTACTAGGG	0.101	0.033	0.1	31	278
CGCAGTGTTGAGG	0.02	0.003	0.016	13	782
GCCGGTGTTGGTA	0.166	0.018	0.147	119	692
AGTGGTGTAGGGA	0.021	0.006	0.021	13	595
CGTGTTACAGAGA	0.023	0.003	0.02	22	1074
CGTAGTGCATGTA	0.016	0.006	0.014	11	788
AGTGTCACAAGCG	0.03	0.003	0.029	29	960
CGTGGCGCTGATG	0.028	0.015	0.011	5	465
AGTGTTGTAAGGG	0.023	0.003	0.027	20	714
GGCAGTGTTGGTG	0.058	0.01	0.066	41	580
CGTGGTATTGAGA	0.018	0.002	0.019	14	716
GGTAGCGCAGGGG	0.073	0.013	0.073	21	268
GGCGGTATTAGGG	0.111	0.021	0.088	57	588
GGTGTTGTTAGTG	0.089	0.014	0.071	32	418
CGCAGTGTTAGGA	0.029	0.002	0.031	32	1001
GCCATTATAGGGG	0.085	0.008	0.075	62	769
GGCGTCGTTAAGG	0.074	0.013	0.058	40	651
CCCAGTGCTTGGG	0.024	0.005	0.027	23	830
CGCGTCGTTGGGA	0.024	0.005	0.03	21	669
GGTGGTATTAATA	0.135	0.026	0.122	148	1067
CGTGGCGCAGATG	0.022	0.006	0.014	8	574
GGTATCACTAGCG	0.115	0.017	0.091	64	643
ACTGGCGTTGGTG	0.027	0.003	0.026	9	339

GCTGGCACAAGTG	0.449	0.055	0.43	427	565
GGTGTCGTTTGTG	0.116	0.016	0.136	46	293
GCTGGTACAAAGG	0.12	0.016	0.106	90	762
AGTATCGTAAAGG	0.029	0.005	0.027	38	1360
GGCGTCATTTAGA	0.068	0.016	0.052	59	1085
GCTGGCGTATGCA	0.883	0.028	0.877	1362	191
GCCGGTGTATGCG	0.387	0.06	0.377	343	568
GGTATTGCATGTG	0.09	0.027	0.054	34	591
AGTGGTGCTTGGA	0.017	0.005	0.01	5	506
AGCGTCGCTAGTG	0.027	0.006	0.025	25	972
CGCAGTGCTGGTA	0.026	0.003	0.023	22	931
GGCGTTGCTAGGG	0.052	0.01	0.039	18	444
GCCGGCATATGCG	0.725	0.023	0.728	1115	417
CCCAGCGTTTAGG	0.021	0.005	0.013	16	1201
AGTGGTATAGATG	0.022	0.002	0.02	18	862
GCCGGTACTTGTA	0.061	0.004	0.062	73	1096
GGTGGTGTTGGGA	0.114	0.052	0.077	22	265
GGTAGCACTTGGA	0.066	0.022	0.047	24	490
CCTGTTGCAAGGA	0.021	0.006	0.016	20	1252
CGTAGCGCTGATA	0.031	0.012	0.015	15	1017
GCTATCACAAGGG	0.067	0.007	0.059	59	948
GGTGTCACAAGCA	0.087	0.02	0.067	68	947
AGTGTTGCATAGG	0.026	0.011	0.01	7	666
GGCATTGTAAACA	0.076	0.012	0.061	168	2584
ACTAGCGTTGAGA	0.026	0.002	0.029	40	1351
GCCATCGCTTGGG	0.053	0.006	0.058	34	552
GCCGGCGCAAGTG	0.743	0.026	0.746	918	313
ACCGGCGTAGAGA	0.026	0.001	0.025	30	1174
GGCGTCGCATAGA	0.064	0.004	0.065	58	828
AGCGGTGCTTGCG	0.016	0.004	0.018	13	705
AGCATCACTTGGG	0.022	0.004	0.026	30	1141
GCCGTCGTTAGTG	0.065	0.018	0.045	29	610
CGTAGTGCATGTG	0.032	0.006	0.024	14	559
CCTGGCGTAGGTA	0.023	0.003	0.02	22	1100
GGTAGCATTAGGG	0.063	0.012	0.064	33	479
GGTATCACAGGCG	0.085	0.003	0.088	67	698
CGCGTTGCTAATG	0.024	0.007	0.017	20	1142
ACTGTTGCTGGGA	0.025	0.003	0.022	21	948
GGCGGTGTATGGG	0.089	0.03	0.07	32	427
ACTAGCGTTGATG	0.034	0.004	0.036	38	1028

GGTGTCGTAGGGG	0.07	0.013	0.065	23	329
GCCGGCGCAGGGG	0.725	0.034	0.711	477	194
GGTGGCGTTGACG	0.66	0.054	0.637	467	266
AGTAGCGTTGATG	0.026	0.006	0.034	28	798
GCTGTTGTTGGGG	0.08	0.016	0.102	39	342
GCTGTCGCTTATG	0.052	0.009	0.053	29	521
GCTGTTACTGGGA	0.054	0.004	0.055	37	634
GCCAGCGTTTGGA	0.043	0.004	0.041	37	860
AGTGGCATAAAGA	0.018	0.005	0.014	14	1010
GCTGTTGCTGGTA	0.05	0.006	0.045	31	656
GCTAGCGTATATG	0.08	0.007	0.08	71	815
AGTGGCATAAATA	0.029	0.006	0.022	31	1374
GCCGGCGTATGTG	0.934	0.012	0.918	1179	105
CGTGTTATTGGTA	0.025	0.003	0.026	23	858
ACCGGTGTTGAGG	0.026	0.002	0.028	25	867
AGTAGTGCAAAGA	0.027	0.004	0.032	46	1408
AGTGGCACAGAGG	0.018	0.002	0.017	7	394
CGTATTGTAGGCA	0.027	0.006	0.02	24	1188
GGCGGTGTATATG	0.084	0.011	0.079	61	716
GGTGGCACTGGCA	0.16	0.011	0.157	100	536
AGTGTCACATAGA	0.022	0.003	0.024	29	1171
GGCGGCGTAAGCG	0.457	0.063	0.435	398	516
CGCATCGCTTGCA	0.021	0.003	0.018	27	1444
GGCGGCGCAAGGA	0.09	0.02	0.089	67	684
GGCGGCGCAGGCG	0.189	0.04	0.181	108	488
GGTATTGCAAGGA	0.081	0.013	0.084	65	708
ACTGGCGTTGGGG	0.03	0.017	0.019	6	302
AGCGTCGCTGATG	0.023	0.004	0.028	32	1101
GGCATTGCAGAGA	0.058	0.01	0.053	63	1127
GGTGTTGCAGGGA	0.089	0.031	0.056	24	403
GGTAGCGTTGATA	0.087	0.009	0.083	61	676
GGCGGTGCTTACG	0.056	0.008	0.067	48	669
GGCAGTATTTGCG	0.049	0.016	0.031	31	968
GGTATCGCAAAGA	0.097	0.007	0.087	92	970
CGTATCGCTGGTA	0.021	0.005	0.023	20	842
ACTGTTGCTGACG	0.022	0.005	0.029	40	1355
GCTAGCATTGGGG	0.072	0.006	0.068	31	425
CGCGTCGTTTGTG	0.021	0.005	0.027	19	682
CGCGTTATAAGCG	0.023	0.006	0.03	43	1376
GCTGGCGTTGACG	0.95	0.013	0.948	1260	69

AGTAGCGCATGTG	0.023	0.005	0.016	11	678
CGCGGTACTTGCG	0.023	0.004	0.018	15	822
CGTGGCATTAAGA	0.029	0.006	0.021	21	986
GGCGTTACATGGG	0.054	0.006	0.047	32	650
CGTGTTATTAGTG	0.026	0.005	0.02	16	774
GGCAGTGCAGGCA	0.051	0.003	0.047	45	904
AGTGGCGCAAAGA	0.018	0.006	0.009	9	957
GGCGGTGCTGACA	0.053	0.006	0.048	50	993
GGCGTTGTTGGCG	0.062	0.011	0.047	32	645
AGCGTCGCATAGG	0.021	0	0.02	18	863
CGTGTTGTTTGCA	0.025	0.007	0.027	19	686
GGTGGCGTATAGG	0.671	0.065	0.616	406	253
CCTGTCACATGGG	0.031	0.006	0.028	24	833
CCTGGTATAGAGG	0.02	0.007	0.028	27	922
CGTAGTGCAGAGG	0.038	0.013	0.027	14	507
GGCAGCGCTAAGG	0.053	0.004	0.048	37	733
GGTGTCGCTAAGG	0.099	0.014	0.08	37	424
AGCGGCGCTGGTG	0.023	0.005	0.03	19	612
GCCGGTGTAGGTG	0.405	0.071	0.402	296	440
GGTGGCATAAGGA	0.476	0.052	0.464	441	509
GGTAGTACATAGG	0.06	0.001	0.061	40	617
AGTGGCGTTGAGG	0.019	0.006	0.02	5	241
ACTGTCGCTGAGA	0.02	0.001	0.02	26	1278
CGTATCACTGGTG	0.025	0.005	0.023	16	694
GGCAGCGTTAGTA	0.056	0.009	0.065	69	999
CGCGTCGCTTATA	0.022	0.008	0.013	18	1364
ACTGGCGTATGCG	0.036	0.008	0.044	21	455
GGTATTACAGGGG	0.105	0.02	0.09	55	555
GCCGGCGTAAAGA	0.848	0.034	0.85	1818	321
GCTGGTGTAAGCG	0.753	0.037	0.752	819	270
GGTATTGTAGGTA	0.095	0.004	0.095	78	739
AGTATTGCAGAGG	0.021	0.003	0.02	17	833
ACTGTCGCATGGG	0.028	0.008	0.026	20	764
AGTGTCGTAGGGG	0.019	0.003	0.021	11	508
GGCGGCGCAGGGA	0.101	0.015	0.095	51	488
CCTGGTGCTAGTG	0.025	0.004	0.026	19	698
AGTAGTGTTAGCA	0.024	0.006	0.021	26	1203
GCCAGCACTTAGA	0.049	0.002	0.046	73	1507
AGCGGTACTGAGA	0.024	0.003	0.028	38	1343
CGCAGCGCATGTG	0.024	0.002	0.026	26	990

AGTGGTATTGATG	0.02	0.009	0.019	13	672
CGTGGCATATAGG	0.02	0.013	0.027	13	464
CCTGGCGTAGAGG	0.024	0.005	0.017	12	704
GCCGTTACATGGA	0.047	0.003	0.046	56	1167
CGCGGTACTGAGG	0.022	0.001	0.02	16	790
CGCGGTGCTTGTA	0.023	0.009	0.019	16	848
AGTGTCACTAGGG	0.021	0.006	0.016	9	571
GCTGTTGCTGATG	0.044	0.004	0.038	27	685
GGTATTACTGGTA	0.079	0.001	0.077	71	849
GCCGGCGTAAGTA	0.844	0.033	0.846	1747	318
AGTGGTGCATGTG	0.028	0.01	0.021	11	523
GCTAGTGCTTATG	0.063	0.005	0.065	56	799
GGTGGTGCTTGTA	0.075	0.007	0.073	33	420
CGTAGTGTTTGCG	0.024	0.002	0.02	11	526
ACTGGTATTGGGG	0.023	0.007	0.018	10	553
CGTAGTATTGAGG	0.02	0.002	0.018	13	697
GCCATTGTTAGTG	0.051	0.001	0.05	56	1061
CCTATCGCAGGGG	0.035	0.005	0.042	33	753
CGTAGTATATGTG	0.023	0.005	0.028	24	825
AGTGGCGTAAGCA	0.025	0.002	0.025	21	824
CGTGGCGTAGGGG	0.026	0.014	0.021	5	233
GGTAGCGTTTAGG	0.097	0.014	0.098	36	333
GGTGTCGCTTGTG	0.098	0.022	0.091	30	301
CGTGGCATTTAGA	0.028	0.011	0.018	13	702
GGTGGCGTAAGCA	0.363	0.034	0.359	299	533
AGTAGTGTATGGA	0.019	0.005	0.025	23	915
GCTGTTGCTGAGA	0.049	0.012	0.036	25	668
GGCGGCACTTACG	0.256	0.047	0.234	246	806
AGTGTCGTTTGGG	0.022	0.006	0.015	6	407
GGTGGTGCTTACA	0.088	0.009	0.085	56	606
GGCGGCGTAGGCG	0.422	0.048	0.416	316	443
GCCGGCGTTAGGA	0.852	0.015	0.842	992	186
GGCGGTGTTTAGA	0.068	0.008	0.075	49	607
GGTGGCGTTAATG	0.689	0.047	0.675	679	327
GGCGTCATTGGTA	0.065	0.012	0.058	58	941
AGTATCGCTTGGG	0.021	0.004	0.025	15	579
GGTAGCACTAGCG	0.072	0.013	0.057	35	577
AGTGTCATAGAGG	0.033	0.016	0.011	9	794
CGTGTTATAGGTG	0.02	0.003	0.018	14	748
CGTGGCACAAGGG	0.023	0.008	0.017	9	527

GGCAGTATTGGGG	0.053	0.023	0.047	33	662
GGTGTTACAGAGG	0.082	0.022	0.09	57	577
GGCGTCACTTACA	0.055	0.004	0.049	78	1503
GGTATTACAGACG	0.093	0.016	0.077	89	1063
CGTAGTGCTTATG	0.028	0.008	0.02	12	602
GGTGGTACTGAGG	0.113	0.017	0.111	46	368
AGTGGCGTAAACG	0.021	0.008	0.032	18	549
GGTGGCGTAGGGG	0.516	0.1	0.447	202	250
CGTATTGTTTGTG	0.027	0.01	0.015	11	723
GGTGTTGTAGGCA	0.075	0.01	0.065	41	589
GCCGGCGCTAAGA	0.492	0.053	0.508	620	600
GCTGGCGCTTGTA	0.6	0.05	0.582	560	403
AGTGTCGTAGAGG	0.022	0.008	0.014	9	646
CGCAGTACTTGTA	0.023	0.003	0.022	33	1436
GGCATCGTTGGGA	0.096	0.015	0.093	74	718
CGTGGCGTAAGGG	0.021	0.004	0.016	6	362
AGTGGCACAGGTG	0.021	0.001	0.02	9	447
ACCGGCGCTAGGG	0.03	0.009	0.041	31	716
GCTGTTATATACG	0.063	0.008	0.057	72	1196
AGCGGTGCTGGTG	0.02	0.001	0.019	12	631
GGCAGCGCTTGTG	0.049	0.01	0.048	32	632
GGCGTTACTAATG	0.064	0.004	0.06	69	1087
AGTGTCGTTAGTA	0.024	0.007	0.02	18	864
CGTAGTGCTAGTG	0.028	0.008	0.027	14	508
GGTAGTATTTGGA	0.057	0.005	0.051	34	639
AGCGTCGTAGGTG	0.022	0.007	0.03	29	932
GGTAGTGCATGGG	0.05	0.008	0.044	15	324
AGCGTTGCTGGGA	0.017	0.006	0.009	7	787
GGCGTCGTTAGCG	0.087	0.013	0.1	65	584
CCTGGCGTTTGCG	0.016	0.001	0.017	11	648
CGTGGCGCTGGTA	0.032	0.008	0.037	20	522
GGCGGCGTAGATG	0.455	0.077	0.431	386	509
GCTAGTGCTTAGA	0.052	0.005	0.059	55	882
ACTGTCACTGGGG	0.026	0.003	0.023	15	626
CGTGTTACTGACG	0.028	0.001	0.029	30	991
CCTGGTATAGGCG	0.029	0.005	0.022	22	962
AGTGGCGCATACG	0.026	0.01	0.016	11	690
GGTGGTACAGATG	0.116	0.017	0.096	58	548
GCTATCGTAGGGG	0.058	0.008	0.069	35	473
CGCAGTGCTTGGG	0.022	0.008	0.027	16	566

CCCGGTGCTTAGA	0.022	0.006	0.031	39	1223
GGTGGCGTAAAGA	0.395	0.067	0.374	301	504
GGCGGCGTTTGGA	0.225	0.03	0.192	97	408
GGTATTGCATGTA	0.078	0.005	0.075	70	865
CCCGTTATAGGTG	0.03	0.001	0.031	41	1269
GGTGGCGCTGAGA	0.156	0.018	0.131	60	398
AGTATTGCTGGGG	0.02	0.001	0.022	11	498
GGCGGTGTTAATA	0.079	0.018	0.069	92	1235
CGTGTTGCAGACG	0.025	0.005	0.026	23	865
CGTATCACATGGG	0.02	0.004	0.022	16	697
ACTGGCATAGGCG	0.028	0.002	0.025	23	895
GCCGTTGCTGGGG	0.048	0.008	0.038	17	434
CGCGGTGCATACG	0.02	0.004	0.022	24	1071
GGTGGCGTAGGTG	0.686	0.041	0.703	426	180
GGCGTTATTTGTG	0.064	0.012	0.055	42	715
CGTGTCGCTGGGG	0.011	0.004	0.007	3	401
AGTGGCATATATG	0.032	0.012	0.037	14	360
GGTGTCGTATATG	0.097	0.007	0.1	63	567
CCTGTTGTTGGGA	0.02	0.001	0.019	15	777
GGTAGCATTTGCG	0.08	0.024	0.064	38	558
GGTAGCGTTTGCA	0.072	0.02	0.058	36	587
GGCGGTGTATAGG	0.083	0.005	0.089	54	550
CGTAGCGCTAGTG	0.03	0.001	0.03	17	559
CGTGTTACAGACG	0.028	0.001	0.029	29	979
GCCGTCACTTGTG	0.063	0.015	0.041	37	860
GGTGTTGTAAGGG	0.079	0.011	0.074	32	403
GCCAGTGTTTGCG	0.052	0.008	0.063	58	864
AGTGTTGTAGGCA	0.028	0	0.028	28	989
AGTGGTGTTAGCG	0.029	0.005	0.032	17	521
CCTGGCGTATGGG	0.024	0.008	0.014	8	545
GCTGGTATTAAGG	0.463	0.02	0.448	425	524
GCTGGTGCTAGCG	0.298	0.045	0.287	163	404
GCTATCGCAGACG	0.062	0.005	0.069	69	936
GGTGGCATTTATG	0.722	0.051	0.73	791	292
GGCGGTATTAGTA	0.074	0.024	0.066	79	1126
AGTGTCATTTATG	0.025	0.003	0.027	27	978
GGTGGCGTTGAGG	0.651	0.047	0.664	350	177
ACTGTCGCTGGCG	0.024	0.004	0.029	25	851
CGTGTTACTTGGA	0.031	0.009	0.031	23	730
CCTAGTACAAGGG	0.032	0.01	0.03	37	1179

GCCATTGTAGGCG	0.043	0.008	0.032	44	1321
GCTGGCGTTGAGA	0.881	0.02	0.868	1022	155
GGTGTTATTGACG	0.098	0.023	0.11	84	680
GCTAGTATAGGGA	0.074	0.016	0.067	60	839
GCTGGTATTGAGG	0.433	0.042	0.425	269	364
CGCGTCATATGGG	0.025	0.003	0.027	21	759
CCCGTTGTTGGCG	0.021	0.003	0.019	24	1273
GGCAGTGCAGACG	0.047	0.015	0.037	38	998
GGTATCGCTGAGG	0.131	0.022	0.114	57	445
GGTGTTATAAGTG	0.085	0.024	0.062	49	740
GGTATCGTTGGGA	0.131	0.022	0.12	62	456
GGCATCGCAAGGG	0.095	0.028	0.071	66	864
GCCAGTGTATAGG	0.047	0.009	0.039	40	997
AGTATCGCAGGGG	0.038	0.005	0.032	18	553
AGCGGCACAGGTA	0.021	0.002	0.02	27	1315
GGTGTTGCTTACG	0.07	0.011	0.055	29	501
AGCGGTGCTGATA	0.02	0.004	0.014	19	1345
CGTGGTATTAGGG	0.032	0.007	0.035	15	413
ACCGTTGTTGATG	0.028	0.004	0.023	34	1445
AGTGGTGCTAATA	0.022	0.01	0.013	15	1170
GGTATTACTTACG	0.114	0.011	0.108	109	897
CGTGTTGTTTATG	0.02	0.002	0.021	13	594
GGCAGCACATAGG	0.057	0.008	0.046	41	857
CGCGGCGTAAGCG	0.031	0.011	0.019	17	879
GGTGGCACTGACG	0.394	0.035	0.387	301	477
CGCGTCACATGGG	0.02	0.005	0.015	12	805
GCTAGCACTGGCG	0.056	0.006	0.05	42	794
AGTGGCATATGGG	0.021	0.01	0.019	4	206
GGTATCACAAGCA	0.095	0.009	0.085	113	1216
GGTGGTATTTGGG	0.264	0.058	0.183	69	308
GGTGTCACTAACG	0.096	0.023	0.074	56	697
CGTGTTGCAAACA	0.026	0.002	0.023	37	1582
GGTGGCGTAGACG	0.67	0.059	0.635	582	334
GGTAGCATTAGCA	0.07	0.018	0.051	51	941
AGTGGTGTAAGGG	0.036	0.006	0.036	18	486
AGTGTCGCAGAGA	0.023	0.003	0.026	22	828
GGTGTCATTTAGG	0.125	0.018	0.105	49	416
CGCAGCGCTGAGG	0.02	0.003	0.018	14	770
GCTATCACTGGGG	0.067	0.005	0.06	34	530
GCCGTTGCAAGTA	0.049	0.003	0.046	56	1164

GGTATCGCAAAGG	0.139	0.031	0.095	71	678
AGTGGCGCATAGG	0.019	0.003	0.015	7	450
AGCGGCGCTAATG	0.028	0.002	0.025	25	994
AGTGGCACTGGTG	0.021	0.005	0.019	8	410
GGTAGCATTAACG	0.073	0.007	0.082	82	912
CCTAGCGTAGAGG	0.03	0.001	0.031	32	989
GGCGGTGTTTATA	0.065	0.006	0.056	51	855
CGTATTGCTGGGA	0.018	0.005	0.013	9	695
AGCGTTACTTGGG	0.02	0.004	0.021	17	798
AGTGTCGTTAGGG	0.023	0.003	0.019	9	468
GGTATCGCATGGG	0.135	0.019	0.116	54	410
CGTGGTGTATGGG	0.024	0.009	0.017	6	351
GGTGTCATTAGTG	0.084	0.014	0.069	38	515
GCCGGCGTTTGGG	0.934	0.005	0.94	739	47
AGTGGCGTATGTG	0.026	0.002	0.023	7	292
GGTGGTACTAATG	0.114	0.018	0.091	57	569
GGTGGTGCTTGCA	0.057	0.009	0.045	22	466
CCCGTCGCTTAGG	0.019	0.006	0.019	19	961
CCCAGCGTTGAGG	0.023	0.002	0.022	28	1247
GGTGGCGTAGGGA	0.332	0.044	0.286	116	290
CGTGTCGTAAGGG	0.024	0.001	0.023	13	548
GCTGGCATAGGTA	0.65	0.044	0.621	800	488
CGTGTTGTTTGGG	0.029	0.007	0.02	8	388
GCCATCGCAAGGG	0.037	0.007	0.044	40	870
AGTGTCGTTGAGA	0.03	0.017	0.017	14	795
CGTAGCACTAGCG	0.024	0.007	0.017	13	752
GGTGTTGCTGGTA	0.073	0.008	0.08	37	426
GCCGGTACTTGTG	0.072	0.003	0.075	62	766
GCTGGCGCAAACG	0.82	0.032	0.808	1336	317
GGTGGCATATACG	0.718	0.037	0.716	998	396
ACCAGTGTAAGGG	0.026	0.001	0.025	39	1517
ACTATCGTTTGTG	0.028	0.005	0.025	27	1064
ACCGGTATTTGCG	0.02	0.004	0.015	19	1250
GCTGGCATAGACG	0.851	0.025	0.832	1431	288
CGTATTATTGGGA	0.03	0.011	0.044	43	941
AGCGTCGTTTGTG	0.025	0.005	0.018	16	874
GGTGGCATAAAGG	0.71	0.053	0.676	852	409
GCCGTCGTAGAGA	0.05	0.01	0.04	39	939
GGTAGTGCTTGGA	0.058	0.01	0.046	19	398
GGTAGCATTTAGG	0.082	0.026	0.067	35	491

ACTGGCGTAAGTG	0.033	0.002	0.034	17	489
GGCGGTATTTATG	0.126	0.008	0.116	108	824
GCTGGCGTATAGA	0.874	0.018	0.857	1228	205
ACCGGCGTTGGGA	0.022	0.006	0.02	15	734
GGCGGTACAGGCG	0.066	0.003	0.065	40	577
GCTAGTACTGAGG	0.059	0.008	0.061	43	658
GGTATCACAAAGA	0.096	0.02	0.097	148	1377
GGTGGCGCTGGCA	0.142	0.025	0.112	50	396
AGTGGTGTTGGGG	0.022	0.01	0.013	4	301
GGTGTTATTAGCG	0.094	0.022	0.087	55	578
CGCAGCGCTGGTG	0.028	0.007	0.021	16	753
GCCGTTATTGGGG	0.074	0.003	0.076	40	487
CCCAGTATTTGGG	0.019	0.002	0.016	21	1275
GGCAGCGTTGAGG	0.07	0.012	0.06	39	608
GGTGGTACAAGGG	0.137	0.017	0.151	70	394
GCCGGCATTTGGG	0.717	0.059	0.711	662	269
GGCAGTGTTTGTG	0.061	0.01	0.071	47	611
CGTGTCATAGATG	0.027	0.002	0.025	26	1011
GCTGGCGTATGTA	0.882	0.027	0.884	1262	166
GGTAGCGTTGGTG	0.077	0.025	0.047	18	362
GCCGGCATATACG	0.739	0.027	0.733	1534	558
GGTGTCACAGGGG	0.086	0.027	0.054	18	318
GGCGTCGCTTATG	0.078	0.008	0.073	55	698
GGCGGCGCATGCA	0.096	0.01	0.083	76	837
AGTGGTGTTGACG	0.027	0.006	0.032	19	583
GCCATTACTTGGA	0.048	0.006	0.044	57	1247
GGTGTTGCAGGGG	0.062	0.019	0.042	12	273
ACCGGTATAGGGA	0.023	0.006	0.025	29	1152
CGTATCACAGGGG	0.023	0.006	0.027	18	638
AGCGGCGTTGGTG	0.022	0.003	0.02	10	497
AGCGGTGTTTGCG	0.017	0.003	0.015	11	739
GGTATCATAGAGG	0.133	0.019	0.126	86	599
GGCATTGCTGGCG	0.055	0.012	0.039	43	1050
GGTGGTACAGAGA	0.09	0.021	0.062	44	664
GCTAGCACAGGGA	0.062	0.007	0.052	44	803
GCTGGTATATGGG	0.457	0.039	0.454	275	331
CGCGGTACTGGTG	0.022	0.008	0.016	11	662
GGCGGTATAAGCG	0.125	0.035	0.093	92	901
GCTATCACATAGG	0.064	0.006	0.059	67	1077
GGTAGTACAAGTG	0.077	0.019	0.065	52	748

GGTGGCATTTAGA	0.474	0.046	0.455	412	493
CGTAGCGCTAAGG	0.023	0.002	0.023	15	624
AGTATTACATGTG	0.021	0.003	0.017	25	1432
CGTAGTATAGAGA	0.027	0.002	0.029	35	1154
GGTGTCGTTGACG	0.123	0.016	0.111	56	447
GGCGTCGCTAGGG	0.069	0.017	0.05	26	489
GCTAGCATTTGTG	0.065	0.003	0.065	55	794
GCTGTTATTAGTG	0.065	0.006	0.071	60	782
CCCGTCGCAGGGG	0.023	0.001	0.022	18	791
GCCATCGCTTAGG	0.047	0.004	0.052	39	709
CGCAGTGTTAGGG	0.03	0.01	0.03	20	638
GGCAGCATTGGGA	0.044	0.006	0.038	37	945
GCTAGCACTAGGA	0.056	0.01	0.042	36	823
GGTAGTATAAGTA	0.072	0.01	0.059	84	1338
GCCGGCATTTGGA	0.377	0.021	0.375	408	681
GGCAGCGCAGGCG	0.061	0.009	0.048	38	746
GGTGGTGCTTGGG	0.095	0.044	0.068	15	207
GCCGTTGTTAGGG	0.057	0.008	0.055	31	534
ACCGGCGTATGGA	0.03	0.009	0.019	19	959
GCTGTCGTATGTG	0.063	0.02	0.068	39	531
GGCATTGCTGGTG	0.066	0.01	0.056	35	591
GGCGTCACTGAGG	0.058	0.016	0.064	46	674
GGCAGCACAGGGG	0.045	0.003	0.046	42	866
CGCGGCATTGGTA	0.026	0.006	0.021	22	1020
GGCGTCGCAGATA	0.066	0.017	0.055	68	1161
GGTGGCGTATATA	0.399	0.064	0.342	338	650
GGTGGCGTTAATA	0.388	0.061	0.348	336	630
GGCAGTGTTGAGA	0.051	0.002	0.048	48	951
GGCAGCACAAGTA	0.055	0.004	0.052	87	1587
AGTGGCGCTGGGG	0.013	0.007	0.015	4	257
AGTAGTACATGCG	0.023	0.011	0.008	8	1046
ACTGTTGCAGGTG	0.031	0.009	0.019	20	1011
GGCGGTGCTAAGA	0.065	0.007	0.062	56	845
GCCGGCGCTTAGG	0.745	0.035	0.734	559	203
AGTATTATTAGTG	0.027	0.003	0.022	33	1435
GCCAGTGCTGGGA	0.039	0.004	0.045	31	660
GCTAGCACATAGG	0.066	0.009	0.077	65	780
CGTAGTACATGGG	0.027	0.007	0.018	11	585
CCCGGTGCTTGCG	0.026	0.002	0.023	21	881
CGCAGTGTTGGTA	0.017	0.004	0.019	19	1005

ACTGGCATATGTG	0.026	0.005	0.024	24	993
CCCGGTACAGGCG	0.022	0.007	0.015	21	1372
CGCGTTGTTAGGG	0.027	0.004	0.022	14	622
CGTGGCGTTTAGA	0.02	0.001	0.019	10	511
GGTGGCACAGATG	0.407	0.046	0.387	276	438
GGTGGTGTTAGCA	0.099	0.034	0.074	41	510
GCTGTCGCTGGCA	0.061	0.01	0.066	44	618
CCTGTCACATGCG	0.03	0.001	0.029	34	1156
ACCGTCGTTAAGG	0.027	0.006	0.02	21	1052
GGTAGTATTGATA	0.072	0.005	0.076	97	1171
GGCATTGTTAGGG	0.089	0.011	0.082	56	627
CGTGTTGCAGGCA	0.021	0.002	0.023	19	807
ACCAGTGCTGGCA	0.023	0.005	0.017	37	2159
GGTGTTACTGACG	0.085	0.019	0.084	67	735
GCTGTCGCTGATG	0.063	0.004	0.064	34	501
AGTGGCACTAAGG	0.024	0.003	0.02	11	534
CGTGTCATTAGCG	0.026	0.002	0.029	23	773
GCCGGCACTAGGG	0.202	0.014	0.189	128	550
AGTGTTACATGCA	0.028	0.002	0.027	37	1350
GGTATCGCTTGGA	0.11	0.023	0.078	46	542
CGTAGTACATGTG	0.029	0.009	0.037	27	710
AGTGTTGCAAGTG	0.023	0.003	0.027	23	820
CCTGTCGCAAGGG	0.023	0.005	0.03	25	802
AGTGGCGTTGGTA	0.026	0.005	0.028	15	513
CGTGTCATAGATA	0.025	0.006	0.02	31	1518
CGTAGCACAAGGG	0.023	0.003	0.027	18	649
CGTAGCGTTTAGA	0.027	0.007	0.024	18	741
AGCGGCATTAGCG	0.018	0.002	0.021	11	522
CGTGGTGTAGGGG	0.033	0.006	0.026	9	341
CGCGGCGCTGGTG	0.024	0.005	0.017	10	566
CGTAGTGTTGATG	0.037	0.003	0.041	26	607
GGTGGTGCTTAGG	0.101	0.026	0.067	20	280
GCTGGCACATACG	0.46	0.034	0.436	514	665
GCTGGCACTTACA	0.225	0.019	0.227	292	994
GGTGTTATTAAGG	0.113	0.017	0.114	81	629
GGTAGTATTAGGG	0.056	0.007	0.05	25	476
GCTGTTGCATGCG	0.064	0.003	0.068	45	614
ACTGGTGTTAGTG	0.019	0.003	0.022	18	790
GGTATTGCTAGGG	0.093	0.03	0.06	27	423
GGTAGTGTAGATG	0.067	0.005	0.06	40	628

GCTATCGCAGGTA	0.056	0.005	0.053	48	851
GCCGGTATTTGCA	0.078	0.004	0.082	117	1316
ACCGTCGCATGTG	0.024	0.007	0.016	21	1328
CCTAGTGTTGGGA	0.026	0.007	0.016	16	1003
CCTGGCACTAGCA	0.028	0.004	0.031	47	1455
AGCAGCGCTGAGA	0.026	0.001	0.027	34	1211
CGCAGCGTTTAGG	0.025	0.003	0.023	18	768
GCTAGTGTTGGTA	0.052	0.008	0.053	47	834
GCTAGTACATGGG	0.057	0.007	0.048	30	598
CGTGTCACTAGTG	0.026	0.008	0.018	12	650
CGTGGCGTAGGGA	0.034	0.003	0.037	16	422
CGTGGCGCATATA	0.022	0.003	0.024	22	904
GCTAGCGCAGACA	0.061	0.005	0.054	66	1163
GCTGGTACTGACG	0.108	0.003	0.104	77	661
AGTATTGCTTGGA	0.022	0.003	0.018	15	831
GGTGTTACAAGTA	0.081	0.017	0.061	66	1010
GGCGTCGCTTGCG	0.067	0.019	0.05	33	622
GGTAGCGCATACG	0.071	0.016	0.083	59	654
CGTGTTGCTTGCG	0.043	0.003	0.047	26	525
CGCGGCATTAGGG	0.02	0.01	0.034	18	515
GCTGGTGCTGAGA	0.104	0.006	0.1	57	514
GGTGTTGCTGACG	0.084	0.009	0.094	48	463
CGCGTTATATAGG	0.024	0.003	0.019	23	1178
GGTATCACATGTG	0.122	0.026	0.129	94	633
ACCGGCGCTTAGG	0.029	0.008	0.024	21	849
GCTAGCGTTTGGA	0.065	0.008	0.055	34	586
AGCATCGCTTGTG	0.026	0.006	0.022	27	1224
AGCGGCGTTAGGA	0.022	0.003	0.026	21	800
AGTATTACTAGCG	0.026	0.003	0.028	37	1286
GGTGGCATTGACG	0.702	0.04	0.703	769	325
CCTAGCGCTGGCG	0.03	0.006	0.035	29	796
GCCGTTGCTTGGG	0.045	0.007	0.038	18	460
GCCAGCGTTGGGA	0.07	0.004	0.065	51	732
AGTATCACAGGGA	0.026	0.007	0.036	36	956
GGTGGTATATATG	0.269	0.064	0.256	220	638
GGCGGTATTGGGG	0.119	0.028	0.081	33	372
GGTGGCACTAGCG	0.389	0.072	0.362	220	387
GCTGTCGTTAAGG	0.048	0.005	0.051	31	579
AGTATCACAAGGG	0.029	0.002	0.031	31	971
AGTGGCGCATAGA	0.016	0.002	0.015	12	763

AGCGTTATTGGCG	0.019	0.007	0.018	22	1168
CCTGGTATAGGGG	0.042	0.007	0.033	22	646
AGTATCGCATAGG	0.024	0.004	0.017	15	848
GGTATCATTTAGG	0.127	0.017	0.103	73	633
GGTATTGTTGGGG	0.095	0.022	0.069	28	377
CGCGGCACAGGGG	0.03	0.005	0.034	21	605
CCTGGCGTAGGCG	0.024	0.003	0.026	21	781
GCTGGTATTGGCA	0.156	0.007	0.146	130	758
AGTAGTATATAGG	0.021	0.006	0.019	22	1167
CCTGGCATAGGTG	0.031	0.007	0.022	20	891
GCTGTCGTTTGTG	0.069	0.006	0.073	34	431
GCTGGTGTATGGA	0.432	0.023	0.431	302	398
AGCGTCACTTGCG	0.02	0.006	0.019	23	1178
ACTGTCGCTTGCG	0.021	0.005	0.015	15	963
GCCGGCGCATATG	0.765	0.03	0.738	926	329
GCTGGCACTTACG	0.451	0.034	0.467	482	550
GCTGTCATATGGA	0.071	0.019	0.047	43	871
GCTGTTACATAGG	0.061	0.005	0.058	45	730
GGTATTACAAGGG	0.106	0.013	0.113	92	725
ACTGGCATAAGCG	0.028	0.006	0.021	25	1161
ACTGGCGCTGAGA	0.03	0.006	0.036	25	675
GGTGGTGCAGGGA	0.068	0.018	0.045	14	299
ACTGGCGCATGCG	0.023	0.003	0.021	15	698
GCCGGTATTTAGG	0.18	0.024	0.146	131	768
AGCATCGTATGGG	0.022	0.003	0.019	24	1218
GGCGTTACAGGGA	0.075	0.003	0.074	56	699
GCTAGCACTGAGG	0.063	0.015	0.048	34	676
GGTGGTGTTTACG	0.219	0.042	0.2	92	369
GCTGGTGCTTGGA	0.121	0.017	0.127	54	370
CGTATTGTTTGCG	0.023	0.009	0.017	12	678
GGCGTCGTTTAGG	0.077	0.022	0.049	28	539
CGTGTCGCTAAGG	0.025	0.006	0.02	13	626
AGTGGCGTTTATG	0.027	0.006	0.025	9	348
CGTGTTGCAAGCG	0.024	0.008	0.029	23	760
CGTGGTGCTAGCG	0.022	0.009	0.029	14	467
GCCGTCGTTAGGG	0.044	0.004	0.04	20	485
AGTGGTACAGGCG	0.023	0.01	0.011	7	650
CGTAGCGCTAACG	0.026	0.002	0.023	22	944
GGTGGTGTAGGTG	0.196	0.067	0.157	55	295
AGTGGTGCATACG	0.026	0.008	0.023	19	813

AGTATTGCATAGG	0.025	0.009	0.017	16	914
GCTGGCGTAGGTA	0.881	0.027	0.877	1074	151
AGCGGTGTATGGA	0.022	0.004	0.022	22	996
GCTATCACAGGGA	0.062	0.005	0.057	58	958
GCTGGCGTAAACG	0.942	0.006	0.946	1820	103
GGTATCACTGACG	0.128	0.018	0.116	100	763
AGTGGCATTAGCG	0.029	0.006	0.029	7	236
CCCGTCGTTTAGA	0.021	0.002	0.019	28	1473
GGTGTTGCTGAGG	0.085	0.009	0.073	23	292
AGTGGCGTTGACG	0.024	0.012	0.008	3	364
AGTATCGCTAGGG	0.025	0.007	0.018	12	644
CGCATTGTAGGTG	0.026	0.001	0.027	27	977
GGCGGCGCTAGTA	0.103	0.008	0.096	74	696
CGCGGCATAGGGA	0.03	0.004	0.026	23	847
CCCATTGTTGACG	0.023	0.003	0.021	44	2033
GCTGTTACTAGGG	0.069	0.016	0.05	28	533
GCCGGCGTTAATA	0.853	0.032	0.847	2086	378
ACTGGCGCTTGGG	0.035	0.008	0.031	13	409
GGCAGCGCTTAGA	0.06	0.012	0.048	38	760
GGTATCACAGGGA	0.081	0.016	0.063	43	636
AGTGGTGCTTGTA	0.021	0.001	0.021	14	638
GCTGGTGTTGAGA	0.399	0.035	0.412	343	490
GCCGGCGTAGATG	0.928	0.018	0.931	1451	107
GCTAGCGCATGTG	0.05	0.016	0.027	15	543
CCTAGTATAGAGG	0.026	0.004	0.021	28	1321
GGTGTCGCTAAGA	0.086	0.014	0.078	51	602
GCTGTCGCAGAGA	0.062	0.018	0.046	31	640
AGCGGCATATATG	0.019	0.004	0.016	11	667
AGTAGTGCAAGCG	0.02	0.003	0.016	15	912
GCTGGTATAGATA	0.159	0.013	0.167	258	1289
CGTATTATATGGG	0.033	0.01	0.041	40	937
AGCGGCGCAAGGA	0.019	0.002	0.018	20	1093
GCTGTCGCAGAGG	0.041	0.008	0.034	15	429
GGTGGTGCTGGTG	0.083	0.018	0.07	18	239
GGCGGCGCAGGTG	0.171	0.032	0.155	83	451
GCCAGCACTTGCG	0.05	0.006	0.045	50	1053
CCTAGCGCTGGGA	0.024	0.004	0.02	18	895
GCCGTTGTTAGTA	0.047	0.009	0.055	62	1061
CCCGGTGTAAGGG	0.022	0.005	0.022	21	936
GGCGTCGTATAGG	0.075	0.008	0.078	51	599

GGCGTCGCAGGGG	0.073	0.01	0.065	32	457
AGTATCATTTGGG	0.026	0.005	0.019	16	813
CGTGTTGTTGGGA	0.033	0.002	0.035	15	411
GGCGTCGCTGGGG	0.063	0.007	0.072	32	413
AGTGGCGTATACG	0.02	0.006	0.02	9	440
GGTGGCATTGATA	0.467	0.057	0.433	497	652
CGCAGTGTTTGCG	0.027	0.004	0.022	19	838
AGTAGCACAGACG	0.026	0.003	0.023	30	1272
GGTAGTGTTGAGG	0.068	0.017	0.054	22	384
GGTATCACTAGTG	0.125	0.036	0.102	66	578
CGTGGTACAGGGG	0.033	0.004	0.029	10	337
GCCGGCGTAAATA	0.846	0.032	0.842	2658	497
CGCGGTATTAGGG	0.023	0.007	0.031	21	652
GGTATCGCTGGGG	0.167	0.01	0.182	57	257
AGTATTGCTGGGA	0.024	0.006	0.018	14	764
CGCGTTGCAGATG	0.027	0.006	0.028	29	1023
CGTGGCGCAGACA	0.026	0.006	0.022	23	1014
GGTGGTATTGGTA	0.114	0.018	0.122	67	483
ACTGGCGCAGGTA	0.02	0.005	0.014	9	621
GGTGGCATATGCG	0.713	0.051	0.683	706	328
GCTAGTGTAGGCG	0.059	0.006	0.052	52	956
GGCGGCGCTGAGA	0.099	0.018	0.085	58	624
ACCGGCACTTATG	0.026	0.004	0.032	57	1751
CCCGGTATTGAGA	0.024	0.01	0.011	19	1693
CGTGGCATTAGTA	0.025	0.003	0.028	23	786
GGTATCGTAGGCG	0.133	0.019	0.108	72	594
GGTGGCACTAGTA	0.198	0.048	0.137	102	644
GGTGGCACATGTG	0.363	0.041	0.335	202	401
GGTGGCATTTACA	0.48	0.065	0.442	533	674
GGCAGTACTGGGG	0.058	0.009	0.05	31	595
GCCGTCGTTGGGA	0.043	0.008	0.037	24	633
AGTAGTATTTAGG	0.022	0.003	0.018	18	977
GGTAGTACTTAGG	0.07	0.006	0.061	31	478
GCCGTCATTAGGG	0.059	0.011	0.054	38	662
GGTGTTGCATGTG	0.087	0.022	0.061	26	400
GGTGTTGTTAATG	0.086	0.012	0.086	56	598
GGCGGCGCTTGCG	0.166	0.044	0.154	89	489
GCTAGCGCTTGCG	0.056	0.011	0.043	22	487
AGCGGCGTTTGGG	0.016	0.004	0.02	8	386
GCTGGTACTTAGG	0.124	0.022	0.145	68	402

GGCGGTGCTAGGG	0.063	0.013	0.046	17	353
GCCGGTACTTGCA	0.059	0.011	0.059	69	1103
GGTGTTACAGGGA	0.067	0.01	0.056	34	572
AGTGTTGCAAGCG	0.032	0.003	0.033	29	838
CGCGTTGCTTGCA	0.026	0.001	0.028	31	1084
GCCGTTACAGGGG	0.055	0.005	0.049	33	641
CGTGGCGTAGACG	0.025	0.005	0.021	12	551
ACTGGCGTTGGGA	0.025	0.01	0.031	18	554
AGTAGCATTAGCA	0.022	0.002	0.021	33	1527
CGTATTGCTAATG	0.027	0.001	0.029	35	1174
ACCGTCACAGGGG	0.021	0.003	0.02	21	1013
GGTGTCATTTGTG	0.091	0.013	0.075	34	419
CGTGGCACTAGTG	0.03	0.004	0.026	14	531
GGTATTGCTGGTG	0.09	0.01	0.075	33	407
CGCAGTACAGGGG	0.026	0.007	0.016	11	698
AGTATTGTTGGCG	0.018	0.004	0.014	15	1073
GCTGGTACTAGTA	0.078	0.017	0.057	53	882
AGTGTTGCTAGCA	0.024	0.002	0.025	27	1033
AGTGGCGCAGACG	0.027	0.005	0.027	18	660
CCTGGCACTAGTG	0.031	0.005	0.028	27	937
CCTGGTGCTGGGG	0.022	0.007	0.021	10	461
GGCGGTGCAGGCG	0.055	0.008	0.049	32	617
GGCGGTGTAGAGG	0.08	0.009	0.081	43	489
GGTGTCGTTAAGG	0.093	0.014	0.087	44	463
GCTGTCGCTTGGG	0.057	0.021	0.034	10	286
AGCGGCGTAGGGA	0.02	0.01	0.029	26	857
AGTGTCGTAGGGA	0.023	0.004	0.018	10	547
CGCGGTGCATGGG	0.034	0.007	0.031	18	554
GGTAGTGCTTGTA	0.05	0.005	0.056	30	502
GGTGTTACTGGGA	0.085	0.033	0.078	44	520
GGCAGCATTGGTG	0.048	0.005	0.053	39	690
GGTGGTGTAAGGA	0.101	0.023	0.103	53	462
GCCGTTGCTGGCG	0.043	0.001	0.043	43	962
ACTGGTGTTGATG	0.023	0.001	0.023	21	877
GGTGGCGCTGGTA	0.178	0.022	0.181	71	321
GCCAGCGCATGGA	0.052	0.006	0.043	38	842
AGTAGTGTTGGTA	0.016	0.002	0.014	13	931
ACTGGCGCTAGCG	0.031	0.003	0.026	18	671
GCCGGCGCATAGA	0.505	0.026	0.476	576	633
GGCGTTGCTAGCG	0.065	0.006	0.057	38	632

AGCGTCACTTGTG	0.017	0.006	0.016	17	1021
GCCAGCGTATAGG	0.057	0.01	0.058	54	876
GGCGTCGCTAGTG	0.074	0.019	0.079	47	548
GGCGGCATTGGCG	0.51	0.047	0.483	409	438
GGCGTTACAGGCA	0.065	0.018	0.045	55	1175
ACTGTCATATGGG	0.027	0.006	0.028	26	915
AGTATCGCAAGGG	0.02	0.005	0.027	23	827
ACTGTCGTTGGTG	0.024	0.005	0.021	12	561
GGCGGCATTGGGG	0.348	0.019	0.353	229	419
GCTGGCGCTAGCA	0.605	0.062	0.599	704	471
GGCGGCACTTGTA	0.14	0.027	0.11	102	825
AGTAGCGCTGAGG	0.025	0.008	0.032	17	518
CCTGGTACATGTG	0.028	0.002	0.025	25	956
GCTATTGCAGGGA	0.041	0.004	0.042	34	781
AGTGGCATTAGGG	0.031	0.005	0.032	6	183
GGCGGTATAAAGG	0.132	0.014	0.128	149	1019
CGTAGCATTAGTG	0.022	0.004	0.025	21	811
CCTGGCGTTGGTG	0.028	0.008	0.029	16	545
CGTAGCGCTAGCA	0.031	0.001	0.031	28	863
GGTGGTATTGGCA	0.126	0.021	0.135	80	512
GGTGGTATTGAGG	0.283	0.057	0.234	115	376
AGTAGTGCTGGGG	0.029	0.011	0.02	8	398
GCCGGCATAGATG	0.727	0.043	0.744	1335	460
GGCGGTGTTTGGG	0.085	0.006	0.078	29	345
GGTGTTACTAGGG	0.096	0.009	0.108	49	406
AGTAGCGCATAGA	0.028	0.007	0.023	23	972
AGCGGCGCATGGG	0.019	0.006	0.012	7	557
GGTAGTGTAGGTA	0.059	0.006	0.053	33	592
CCTGTTACTTGGG	0.028	0.009	0.04	27	655
GCCATCGCTAGGG	0.048	0.011	0.04	28	673
GGTATTACTTGGA	0.088	0.008	0.078	58	690
CGCGGCGCTGGGG	0.027	0.005	0.032	15	461
GCTGGCACAAGGA	0.218	0.019	0.233	215	709
AGTATCATAGAGG	0.02	0.004	0.02	24	1190
CGTGGCATAAGGG	0.015	0.002	0.017	8	461
AGTGGTGCTAGTA	0.027	0.007	0.017	13	774
GGTGGCGCTAGGA	0.159	0.034	0.121	41	298
GCTGGTACTGATG	0.119	0.002	0.12	81	596
GGTGTTGTTAAGA	0.078	0.018	0.065	49	701
GGTGTCACTAAGG	0.1	0.009	0.091	50	502

ACTGGTGCAGGCG	0.025	0.006	0.03	26	828
CGTGGTGTTTGTG	0.027	0.005	0.027	10	364
GGTAGCGTTTATG	0.08	0.025	0.057	32	528
AGTGTCGCTAATG	0.021	0.001	0.02	18	869
ACCGGCGTTGAGA	0.027	0.005	0.035	38	1054
ACTGGTACTTGGA	0.026	0.002	0.023	28	1167
AGTGTCACTTAGG	0.027	0.006	0.03	21	676
AGCGGCACTGGGG	0.008	0.003	0.008	4	468
ACCGGTGCAGGGG	0.026	0.004	0.021	16	764
GGCATCGCTTGGA	0.084	0.015	0.068	57	778
GGTAGTACTGAGG	0.057	0.004	0.051	29	542
CGTATTGTTTGCA	0.025	0.001	0.025	27	1049
ACTGTTACTGGGG	0.022	0.006	0.03	20	636
GGCGTTGCAAGCG	0.064	0.011	0.052	47	861
GCCGTCGTATGGG	0.037	0.011	0.038	19	479
CGTGGCGTTAGGG	0.019	0.013	0.02	7	335
CGTGGTGTATGTG	0.025	0.011	0.022	10	439
GGTAGCGCTTGGA	0.073	0.008	0.063	22	328
CCTAGCGCTAACG	0.035	0.003	0.032	51	1519
AGTAGTATATGCG	0.026	0.006	0.026	31	1169
ACTGTCGTTAGGG	0.03	0.006	0.024	14	568
GGTGTCGTTGGTA	0.087	0.014	0.084	40	438
GGTGGTACAGACA	0.07	0.022	0.058	57	931
GGTATTACTAGGG	0.113	0.017	0.114	77	598
GGTGGTGTAAAGG	0.19	0.003	0.186	114	500
CCTGGCGCTGGCA	0.031	0.008	0.025	24	931
GGTGGCATTTAGG	0.708	0.048	0.683	529	245
GGTAGCGCTAGCG	0.073	0.011	0.059	30	478
CGTGGTGTTAGTA	0.029	0.01	0.023	17	718
CGTGGTGTATGTA	0.022	0.005	0.019	13	670
GCCGGCATAGGTA	0.391	0.04	0.394	564	869
CGCGGTGTAGGTG	0.029	0.004	0.024	15	609
GCTGGTGTTTAGG	0.752	0.057	0.766	534	163
GGCATCATTAGTG	0.083	0.002	0.085	105	1129
GCTGGCACTGGCA	0.208	0.031	0.21	174	655
AGCGGCGTTTGGA	0.019	0.003	0.015	11	732
GCCGTCGTAGGTA	0.051	0.011	0.045	41	866
AGTGGCATAAGTG	0.026	0.012	0.028	9	308
GCTAGCATTAACG	0.062	0.009	0.052	76	1398
GGCAGTGCTTGGG	0.053	0.005	0.058	26	424

GGCAGTGCTTAGA	0.051	0.008	0.042	36	817
GCTAGCATATGGA	0.064	0.002	0.067	71	986
GCTGGCGTAGAGG	0.946	0.011	0.939	867	56
GCTATTGTTGGGG	0.067	0.019	0.061	33	509
GGTGGTATATAGG	0.276	0.036	0.267	164	451
AGTATCATAGATG	0.025	0.003	0.025	40	1550
GCCGTCGCTGGTA	0.045	0.018	0.07	49	654
CCCGGCGTAAGCG	0.023	0.005	0.018	25	1391
AGTATCGCTGGGA	0.023	0.008	0.017	12	707
GGCGTCGTTGGCG	0.075	0.007	0.076	50	608
GCTGGTGTAGGTG	0.726	0.054	0.725	459	174
GGTAGCACAGGGA	0.07	0.006	0.072	42	538
GGTATTGTATATG	0.107	0.015	0.086	82	876
GGTGTTGTATACG	0.094	0.024	0.066	49	698
GGTGTCGTAAGGA	0.097	0.026	0.094	53	512
GCTGGTGTAGGTA	0.403	0.054	0.374	288	482
GCCGTTGTATGTG	0.05	0.008	0.045	37	787
GGTAGTATTTACA	0.068	0.011	0.062	80	1202
GCTGGCGCTTACG	0.809	0.032	0.808	824	196
AGTGGCATAGACG	0.019	0.006	0.021	7	320
GGTGTCGTATGGG	0.096	0.01	0.092	31	305
ACTATCGCAGAGG	0.027	0.004	0.024	30	1197
GCTGGCGCAAGGA	0.644	0.053	0.621	599	365
GGCGTCGCAAGGA	0.048	0.008	0.043	37	830
AGTGTTGTTTACG	0.03	0.004	0.027	22	797
GCTGTTGCAAGCG	0.065	0.016	0.086	67	715
AGTGTTGTATGCG	0.023	0.004	0.022	17	771
GCTGGCGTTGAGG	0.946	0.012	0.946	876	50
GGCAGTATTTAGG	0.053	0.002	0.056	55	929
GGCAGTGCTGGCA	0.048	0.011	0.037	30	790
AGTGGTGCTTGCA	0.022	0.005	0.019	13	664
GCTAGTGCAGGCG	0.059	0.008	0.055	49	850
GGTATCGCTAGTA	0.102	0.013	0.094	77	740
GGTAGTGCAGGGG	0.051	0.006	0.047	16	326
GGTGTTATTTGTG	0.081	0.027	0.05	26	490
CGTGGTACAGGTG	0.018	0.006	0.009	5	529
GCTAGTGCTTGCG	0.05	0.007	0.043	28	621
CGCGGCGTAAGGG	0.024	0.004	0.029	17	572
GGTGTTGTAGGTG	0.084	0.007	0.09	34	343
AGTGTCGCTAGGG	0.025	0.017	0.044	20	431

CGTAGTACTGGCG	0.027	0.011	0.014	10	682
CCTGTCGTTAGCG	0.022	0.008	0.022	23	1014
ACTGGCGCTGATA	0.024	0.001	0.022	21	940
GGTGTCGCAGAGG	0.092	0.01	0.086	34	360
CGTGGCACTAGCA	0.031	0.008	0.024	23	925
GCCAGTGCTTGTG	0.047	0.016	0.05	35	666
CGCGTCGCTGGGG	0.016	0.004	0.011	5	460
GGTGGTATAAGGG	0.273	0.059	0.23	132	442
AGCGTCGTATAGG	0.02	0.007	0.02	21	1043
GCTAGCATTGGGA	0.069	0.01	0.059	42	671
GGTGGCGTAAGTG	0.673	0.038	0.669	484	239
GCTGTCGTATGCG	0.059	0.013	0.065	39	562
AGTGGCATAAATG	0.026	0.005	0.031	13	400
GCCGGCATAAGCG	0.734	0.033	0.718	1290	506
GGCGGCACTGATG	0.268	0.049	0.223	200	698
GGTAGTGTTTACA	0.052	0.003	0.053	50	890
CGCGGCATATAGG	0.022	0.004	0.025	20	783
AGTGTTGTTGGCG	0.02	0.002	0.019	13	654
AGCGGCACTTAGG	0.013	0.004	0.007	5	662
GGTAGCGTTTGCG	0.069	0.018	0.043	18	398
CGTAGTACAGGGG	0.024	0.01	0.016	8	478
GGTGGCATAAGCA	0.491	0.048	0.488	619	650
GGTATTGCAGATG	0.097	0.009	0.089	72	737
AGTGGTGCATGGA	0.028	0.002	0.025	16	624
CGTGGTGTTGATG	0.024	0.008	0.014	7	494
GGTAGCATAAGGG	0.079	0.02	0.091	57	568
GCTGGTGCAGATG	0.278	0.029	0.294	205	493
GCTGGCGCTTGTG	0.804	0.041	0.771	570	169
GCCGGCGTAGAGA	0.852	0.026	0.842	1235	231
ACTGTCACAGGGG	0.035	0.004	0.04	31	744
CGTATTGTTTGGG	0.038	0.006	0.033	17	491
ACTATCGCATAGG	0.023	0.004	0.019	26	1333
GGTGTTGTTAGCG	0.087	0.018	0.064	29	425
CCTGGTGCATATG	0.02	0.002	0.021	21	1003
GGTGGCGCATAGA	0.151	0.017	0.167	100	498
CCCATTGTTAGGG	0.025	0.004	0.019	23	1167
CGCGTCACTTGGG	0.021	0.003	0.017	12	693
GCCGTCGTTTAGG	0.059	0.002	0.061	35	543
GCTATTACATGGG	0.054	0.009	0.043	37	819
GCTAGCATTGAGA	0.056	0.014	0.049	54	1046

GGTGTCATATGTG	0.11	0.036	0.101	60	537
GCTGTTGCTAGGG	0.072	0.017	0.053	24	430
GCTGTTGTAGGCG	0.049	0.001	0.051	39	730
GGTAGTATTTAGG	0.071	0.005	0.078	42	499
GCTGGTGTTGGCG	0.668	0.047	0.685	470	216
AGTGGCGTTAGGA	0.018	0.006	0.011	5	464
GGTAGCATTGGCG	0.062	0.008	0.07	33	438
GGTGGTGCTGGGG	0.104	0.015	0.124	26	183
GGCAGCGTAGATG	0.06	0.003	0.061	60	931
ACTGGCGCTTAGG	0.029	0.006	0.024	13	532
CCTGGTACAGGGG	0.036	0.002	0.038	24	608
GGTATTATAGGGG	0.07	0.014	0.051	37	685
AGTGGCGCTTACG	0.025	0.009	0.013	8	595
CCTGGTACTTGCG	0.032	0.006	0.028	24	839
AGTGTTGCTGGGG	0.019	0.001	0.019	8	421
GCCAGTGTATGTG	0.051	0.004	0.048	49	964
ACTGGCGTTAGGG	0.026	0.008	0.023	8	338
GGCGGCATTGATG	0.538	0.073	0.5	626	625
GCTGGCATTAGTG	0.863	0.021	0.865	990	154
CGTGGCATTTGGG	0.021	0.006	0.029	9	297
AGTAGTACATATG	0.03	0.006	0.037	53	1387
GGCGGCGTTAGTG	0.465	0.062	0.45	313	383
GGTGGCGCAAATG	0.315	0.044	0.277	196	512
GCTGGCGTATATA	0.883	0.029	0.882	1683	226
GCTGGCATATACG	0.856	0.023	0.848	1629	291
GCTGGCGCAGATA	0.599	0.055	0.62	892	546
AGTATCGTTTGCG	0.024	0.007	0.028	23	803
GCTGGCACAGGCG	0.443	0.036	0.437	328	422
GGTGTTACAGATG	0.087	0.013	0.085	67	718
GGTAGCATTTAGA	0.068	0.015	0.064	55	806
CGTGGTGTAGGCA	0.028	0.002	0.025	17	668
GGTATTGCTTATA	0.071	0.008	0.061	62	948
CGTAGTGCTAGCA	0.025	0.008	0.016	14	855
AGTAGTGCTGAGG	0.021	0.005	0.024	15	617
GGTGTCGTAAGCG	0.096	0.023	0.112	62	494
ACCAGCGTTGGGG	0.03	0.007	0.038	29	732
GGTAGCGTTTGTA	0.098	0.026	0.077	42	502
CCTGTTGCTAAGG	0.025	0.002	0.025	26	999
GCTATTATTGAGG	0.056	0.01	0.05	58	1108
GGCGTCGTTGGTA	0.074	0.035	0.063	45	672

GCTGTCGTAGGCA	0.063	0.008	0.058	45	731
GCTGTCACTAATA	0.058	0.006	0.053	89	1584
GCTGTCGCATGGA	0.052	0.011	0.036	19	507
GGCGTTGTTAGGG	0.058	0.003	0.056	26	442
CGTAGCGTTTGGG	0.03	0.002	0.033	13	384
CCTGTCGCAGGCG	0.03	0.006	0.036	32	861
GGTAGCGTTTGTG	0.076	0.018	0.052	21	380
GCCGGCGTAGACA	0.857	0.029	0.847	1972	356
CGTGGTGTTTAGG	0.021	0.002	0.02	8	397
GGTGGTATAGATG	0.278	0.068	0.241	191	600
CGTAGCGCAGGTG	0.027	0.006	0.035	20	545
CGTGGTGCTAGGG	0.03	0.011	0.036	13	344
AGCGGCGTATGCA	0.027	0.011	0.037	46	1210
GCCGGTACTGGGA	0.06	0.008	0.061	52	804
GGTGGCGTAAGGA	0.381	0.054	0.363	201	352
AGTGTCGCTGATG	0.022	0.002	0.02	15	723
CGTGTTACTGGCA	0.021	0.003	0.016	16	965
GGTGTCATATGCG	0.095	0.018	0.076	42	508
GCTGGCGTATATG	0.945	0.009	0.944	1390	82
CGCGGTGCTTGGG	0.025	0.003	0.025	11	433
CGTGGCACTGGGA	0.02	0.006	0.018	9	479
GCTGGCACTGGCG	0.426	0.041	0.394	272	418
CCTAGTGTTTGGG	0.026	0.008	0.036	27	716
GGTGTCGCTTGGA	0.087	0.028	0.053	20	358
GCCGTCGTTAGGA	0.049	0.01	0.056	47	788
GGTGTTGCTTGCG	0.073	0.018	0.049	17	330
ACCGTTGTTTGTG	0.027	0.003	0.022	24	1056
GGTGGCACTGGTG	0.39	0.081	0.345	152	289
GGTGTTACTAGCG	0.091	0.006	0.083	50	556
CCTGGTGTATATG	0.031	0.005	0.028	30	1053
GGCGGCGTAAGGG	0.412	0.032	0.412	265	378
GGTAGCGTAGGGG	0.068	0.004	0.063	25	375
GGCAGCACTGGCG	0.059	0.011	0.047	43	869
GGCGTTACAGGGG	0.056	0.005	0.055	31	533
AGTGGCGTATGCG	0.024	0.01	0.021	8	366
GGTGGTGCATGGA	0.076	0.008	0.081	32	364
AGTAGCGCTTATG	0.027	0.002	0.026	21	793
GGCGGCGTTAGCG	0.48	0.081	0.472	375	419
GGTGGTATTGGCG	0.248	0.054	0.208	106	403
GCCGGTGCATGCG	0.101	0.006	0.109	80	654

GGCGTCGTTGGGA	0.076	0.006	0.084	43	469
GCTGGCATATGTA	0.644	0.038	0.62	939	575
AGTGGCACTAGTA	0.026	0.003	0.031	30	939
AGTGTCGCAAGTG	0.021	0.006	0.013	8	629
GGTAGTACTGGGG	0.062	0.019	0.046	16	331
CGCGGCGTTTGGG	0.038	0.007	0.04	17	410
AGCGTTGTAGGTG	0.025	0.005	0.026	24	901
CGCGTCGTTTAGG	0.034	0.002	0.037	29	755
GCTAGCATTTAGG	0.057	0.008	0.047	39	792
GGTAGCACAAGCG	0.07	0.014	0.056	41	694
GCTGGTGTATATG	0.751	0.055	0.753	892	293
GGTATTGCATAGG	0.106	0.014	0.086	59	628
GCCAGCATTAGGG	0.088	0.003	0.09	69	698
GGTATCGCATGGA	0.103	0.006	0.11	71	577
GGTGGCGCTGATA	0.168	0.014	0.15	82	464
GGTAGCACTGGGA	0.067	0.011	0.079	41	475
CGTGGTATTGAGG	0.025	0.006	0.033	16	470
CGTGGCACAGGGA	0.02	0.004	0.02	12	596
AGTGGCACTTACG	0.027	0.006	0.018	12	640
GGTGTTGCAGATG	0.067	0.009	0.06	34	536
CCTGGTACTGAGG	0.031	0.003	0.029	25	839
ACTAGTGTTGGGG	0.028	0.001	0.027	20	713
GCTGGCGCTTATG	0.822	0.018	0.809	712	168
GGTGGTGCAGACG	0.095	0.017	0.088	46	476
CGTATCACTTGGA	0.03	0.012	0.017	14	826
AGTGTCGCTGATA	0.024	0.002	0.027	28	1015
AGCATTGCTTGGA	0.02	0.003	0.022	28	1255
ACTATCGCTTGGG	0.022	0.004	0.02	18	881
AGCGGCATTAAGG	0.021	0.004	0.019	11	582
GGTGGCGTTAACG	0.669	0.053	0.647	610	333
CCTATCGCTTGTG	0.028	0.005	0.035	37	1033
GCCGTCGCTGGCA	0.047	0.009	0.05	42	790
GCCGGTGTTTGTG	0.39	0.042	0.359	234	417
GGTGGCATTAAGG	0.705	0.036	0.697	743	323
GCCATTGCAGGGG	0.042	0.007	0.033	24	698
CCTAGCGTAGGGG	0.037	0.006	0.032	22	665
CGTGTTACATGCG	0.027	0.005	0.023	20	836
CGCAGTATTTGGG	0.023	0.004	0.023	19	812
GGTGTTGTATAGG	0.095	0.003	0.092	44	433
CGTAGCGTATGGG	0.017	0.002	0.017	8	467

GGTGTTGCAAAGG	0.101	0.021	0.075	46	569
AGCGGCGCAGGGA	0.019	0.007	0.029	22	745
GCTGGCATATGGA	0.632	0.037	0.617	662	411
GGCGGCGCAGATG	0.183	0.04	0.178	122	565
CGCAGCATAGAGG	0.023	0.001	0.024	25	1037
GCTGTTGTTTACG	0.066	0.01	0.077	63	750
GCTGGTACAAGGG	0.099	0.01	0.107	59	493
GGTGGTGTATGTA	0.105	0.005	0.101	57	510
GGTGTTACATGGA	0.077	0.024	0.062	42	633
GGTGGCGCAGAGG	0.283	0.024	0.282	109	277
GGTGTTGCTAGTG	0.078	0.009	0.091	38	379
GCTGGTGTTAATA	0.419	0.051	0.414	598	847
GGTGGTACTAGTG	0.091	0.024	0.089	40	409
CGCAGCGCAGGTG	0.022	0.005	0.017	15	854
CGTAGTACAGATG	0.031	0.002	0.029	28	923
GCCGGTATTGGGG	0.141	0.024	0.161	79	412
GGTGTTGTTAGGG	0.075	0.019	0.079	28	325
GGTGTTGTAGAGA	0.075	0.011	0.069	41	557
AGTGGCACTTGTG	0.026	0.008	0.021	9	423
GCTGGTGCAAGTA	0.118	0.016	0.119	102	755
CGTATCGTTGGGA	0.031	0.016	0.018	14	760
GGTATTACTGGGG	0.107	0.019	0.096	46	434
ACTATCACAGGGG	0.027	0.009	0.025	26	995
GGTGGTGTAGGGA	0.124	0.032	0.11	46	371
GCCGGCGCTGGGG	0.733	0.02	0.727	434	163
AGCGGCGCAAACG	0.025	0.005	0.023	32	1380
GCCATCGCTGAGG	0.051	0.005	0.044	35	754
CGTATTGCAGGTG	0.031	0.005	0.024	17	677
GGCGGTACAAATG	0.072	0.01	0.069	89	1192
GGTATTGCAGAGA	0.07	0.008	0.067	55	760
CGTGGCACTTGCG	0.034	0.004	0.03	14	448
CGCAGTGTTAGTG	0.022	0.003	0.022	20	869
GCTGTCGTTTGGG	0.083	0.011	0.067	25	348
ACTGTTGCTGAGG	0.024	0.006	0.031	27	842
GGTATCGTTGATG	0.154	0.021	0.124	90	634
CGTGGCGTAAACG	0.024	0.006	0.022	19	836
AGTGGTGTATGCG	0.021	0.011	0.032	18	547
GGTAGTACAGGGG	0.056	0.01	0.046	18	374
GGTAGTATTGGTA	0.063	0.006	0.056	43	725
GCTGGCGCAGGGA	0.625	0.051	0.61	438	280

CGTGGCACAGGGG	0.018	0.007	0.009	3	344
AGCGGCGTTAGCA	0.03	0.004	0.025	28	1085
GCTAGCACTTATG	0.068	0.008	0.078	76	902
GCTGGCATAGGGG	0.735	0.048	0.706	587	245
CGTAGCATAGGGG	0.037	0.009	0.049	24	462
GCTGGCATAAGGG	0.82	0.036	0.799	848	213
CGTGGCGTATGCG	0.024	0.014	0.043	21	467
AGCGGTGTTTGTG	0.022	0.003	0.026	19	716
AGTGGTGTTGGTG	0.015	0.005	0.011	4	348
GGTGTCGCTTGCG	0.093	0.03	0.061	23	354
GCCGGCGCAGATG	0.746	0.041	0.733	906	330
GGCGGCGTTGATG	0.454	0.055	0.442	363	459
GGTGGTACTTGCA	0.088	0.004	0.094	58	559
CGCAGCATTGGTG	0.028	0.005	0.023	21	890
ACTGGCGCTAGGA	0.033	0.003	0.032	16	477
CGTAGCATAAGCG	0.026	0.004	0.021	23	1056
AGTGGCGCAAAGG	0.027	0.008	0.021	13	594
CGTGGTGCAGGCA	0.027	0.002	0.028	21	736
CGTAGCACTGGGA	0.02	0.005	0.027	16	586
AGTGTCGCAGGTG	0.029	0.01	0.023	12	514
CGTGGCGCTGGTG	0.036	0.004	0.041	15	355
CGTAGTGTTGGGG	0.021	0.012	0.014	4	279
CGTGGCGTTGGTA	0.021	0.003	0.017	8	469
GGCGTCATTGGCG	0.066	0.01	0.053	43	776
GCTGGCGCTTGGG	0.8	0.012	0.787	407	110
GGTGGCATAAATG	0.715	0.053	0.703	1210	510
GGTGGTATAGAGG	0.267	0.057	0.257	148	428
GCTGGTGTATGTG	0.746	0.05	0.756	617	199
GCCGGTACATGCG	0.076	0.002	0.076	73	888
GCTGTTGCAGACA	0.047	0.004	0.042	48	1087
GGCGGTATTAAGG	0.119	0.029	0.093	79	770
CCTAGTATATGCG	0.025	0.002	0.027	44	1569
GGTGTTGCTGGCA	0.074	0.007	0.065	33	475
GCTGGTACAGACG	0.111	0.014	0.116	97	739
CGTGGTGCATAGG	0.028	0.003	0.026	13	491
GCCGTCGCAGGCG	0.055	0.013	0.057	37	608
CGTGTTATAGGGA	0.027	0.01	0.028	21	723
AGTGTCGTTGATG	0.027	0.005	0.022	17	769
CGTGGTATTTGGA	0.024	0.007	0.029	16	541
GGTATCACTGGGG	0.134	0.038	0.158	67	357

GGTGGTGTTGACG	0.192	0.044	0.178	81	375
CCTGGTACTAATG	0.028	0.009	0.021	28	1286
CGTGGCGCTTAGA	0.019	0.005	0.026	15	563
AGTGTTATTTGGG	0.023	0.005	0.027	17	613
GCTGGTGCAGGTG	0.278	0.045	0.251	108	323
ACTGGCGTAGATG	0.025	0.002	0.027	14	500
CCCGGCGCTGGGG	0.021	0.005	0.02	13	639
CGTGGTACTTATG	0.028	0.006	0.02	13	627
GGTGTCACATGGG	0.095	0.023	0.082	29	323
GCTAGCGCAAAGG	0.056	0.011	0.046	33	686
CGTGTCGTTTGGA	0.016	0.006	0.012	6	511
GGCGGCGCTGGTG	0.163	0.033	0.157	72	388
GCTGGCATTTGTG	0.858	0.031	0.84	863	164
GCTAGCGCAGGCG	0.069	0.013	0.052	32	580
CGTATTGCTAGTG	0.026	0.011	0.017	14	823
GGTGGCGTTTAGA	0.38	0.053	0.348	187	350
GGTGGCGTATACA	0.398	0.055	0.394	373	573
GGCGGTGTATGGA	0.064	0.019	0.038	24	613
GCTGTCACTTAGG	0.066	0.014	0.058	36	587
AGTATCGTTGAGG	0.019	0.003	0.022	18	788
GGTGTTGCTTAGA	0.074	0.024	0.053	29	519
GGTGGTGTTGGCA	0.099	0.018	0.117	54	408
GCTGGCACTAAGG	0.484	0.023	0.474	432	479
GGTAGCATTTGTA	0.056	0.009	0.054	43	752
GGTAGCGTAAGTG	0.08	0.016	0.075	44	540
GGCAGTGTAAGGG	0.065	0.011	0.052	38	699
CCCGTTGCTGGGG	0.019	0.006	0.016	12	730
ACTGTCACAGATG	0.026	0.006	0.025	39	1511
GGTGTTGTTGGTG	0.064	0.006	0.055	20	344
GCTATCACATGGA	0.068	0.003	0.072	77	997
GGTGGCACTGATG	0.39	0.068	0.348	251	471
GCTGGTATAGAGG	0.451	0.036	0.426	336	452
GCTGGTACATGGA	0.087	0.01	0.087	66	690
CGTGGTGCTAGGA	0.02	0.003	0.019	9	477
CGTGGTACATAGG	0.028	0.009	0.015	9	589
AGTATTACTTGTG	0.027	0.006	0.034	39	1097
GCTGTCGTTGGCA	0.053	0.01	0.055	38	655
AGTGTCATTGAGG	0.032	0.006	0.025	20	793
CGTGTCACAGGTA	0.02	0.001	0.019	17	863
GGTGTTATAGGTG	0.094	0.023	0.069	35	473

GGTAGCGCTTACA	0.085	0.021	0.075	62	762
GCTGGCGCAAAGG	0.819	0.032	0.825	836	177
GGTGGTGTAGGTA	0.101	0.007	0.106	50	420
GCCGGTGCAAGGA	0.054	0.004	0.057	47	779
CGTGGCGTTTGTA	0.022	0.002	0.019	10	511
GGTGGTATAAAGG	0.277	0.039	0.254	208	610
GGTGGTGCTTATG	0.091	0.017	0.072	27	346
GGCGTCGCTGGCA	0.066	0.006	0.057	52	853
GGTGGCATATATG	0.715	0.049	0.693	949	420
CCTAGCGTTAGGG	0.028	0.007	0.037	25	650
ACTGGCGTAAGGG	0.036	0.008	0.032	11	337
CCCGTTATTAGGG	0.028	0	0.028	31	1060
CGTGTTATTGGGA	0.025	0.009	0.028	19	649
GGTGTCACTTAGA	0.081	0.024	0.051	35	647
AGTAGCACTTGGG	0.02	0.007	0.011	6	555
AGTAGTGCTTGCG	0.021	0.005	0.025	15	578
GCCGGCGCAAGCG	0.76	0.045	0.763	1025	319
GCCGGCGTAGAGG	0.928	0.016	0.939	1045	68
GGTGTCGCTTGCA	0.088	0.024	0.054	29	504
GCCAGCGCTGGCA	0.048	0.001	0.047	50	1008
AGCGGCGTTGGCG	0.019	0.008	0.019	13	664
AGTGGCATTTATG	0.021	0.006	0.025	7	268
GCCGGTGCAGGCG	0.089	0.015	0.093	75	734
GCCGGCGTATATG	0.927	0.016	0.919	1630	143
GGTGGCGTTTATA	0.38	0.03	0.357	278	501
CGCAGTGCTAGTA	0.024	0.003	0.021	27	1271
GCTGGCGTTTAGA	0.899	0.024	0.903	1074	116
GGCATTGCAGGGA	0.054	0.004	0.059	51	820
GGCGTTGTTTACA	0.055	0.009	0.048	60	1201
GCTGGCATTAGCG	0.841	0.032	0.843	1049	195
CGTGGTGCTTGTA	0.022	0.007	0.012	7	591
ACTGGCGCTTGTG	0.025	0.003	0.029	18	595
GCTGGTGCAAGCG	0.281	0.036	0.255	171	500
CGCGGCATTTGTG	0.016	0.009	0.029	18	609
GGCGTTACAAGTG	0.063	0.002	0.065	69	995
GGTAGTACTAAGG	0.058	0.005	0.051	33	608
CCTGGCGTATGGA	0.024	0.002	0.023	20	849
GGTGTTGTAAGTG	0.086	0.01	0.078	42	498
CGTGTCGCATGCG	0.024	0.005	0.018	13	725
AGTGGTGCAGGTA	0.023	0.009	0.012	8	684

AGTGGTATTAGTG	0.025	0.006	0.027	15	543
GGTAGCGCAGAGA	0.061	0.012	0.069	45	603
CGCGGTGTTGACG	0.024	0.004	0.018	17	916
GCTGGCACTTGCG	0.435	0.047	0.41	284	409
GGTGGTATTTGGA	0.122	0.005	0.122	59	424
GGTGTCGTTAGGG	0.106	0.015	0.095	31	295
GGTAGCGTTAGGG	0.076	0.019	0.057	19	313
GGCATTGCAAGTG	0.07	0.011	0.055	62	1061
CGTGGCGCTTGGA	0.027	0.01	0.018	8	438
CGCGGTGTTTGCG	0.028	0.004	0.025	16	636
GGCGGTGCTGGTG	0.066	0.019	0.044	17	370
GCCGGCGTATAGA	0.849	0.032	0.837	1424	278
CCTGGTGCTTGGA	0.027	0.007	0.024	19	784
AGTGTTGTTAGTG	0.025	0.007	0.034	22	621
CGCAGTGTTTGGG	0.029	0.003	0.025	16	612
GCTGGCGCTGATA	0.628	0.038	0.616	721	449
GGTGTTACTGGTA	0.088	0.011	0.073	47	597
GGTGGCGTAAAGG	0.663	0.049	0.649	538	291
AGCGGCGTAGAGG	0.019	0.002	0.018	11	612
GGTATTGTTAGGG	0.099	0.026	0.077	40	482
GCTATCGTTTGGG	0.066	0.006	0.075	42	521
CGTGGCGCAAGCG	0.025	0.009	0.019	11	555
GGTGTTGCTAATG	0.084	0.009	0.086	46	492
CGTGGTACATGCG	0.024	0.009	0.012	8	654
GGTAGCACTAAGG	0.054	0.008	0.061	38	585
GGTGTCGCTGGGA	0.073	0.012	0.065	26	371
CGTGGCATTGGCG	0.023	0.009	0.027	12	425
GGTATTGCTTGTG	0.089	0.019	0.064	35	511
GCTGGTGCAAGTG	0.296	0.037	0.303	204	470
CGCAGCGCTGGGG	0.028	0.002	0.03	14	450
GCTGTCGTTTGGA	0.056	0.012	0.073	38	480
GGTAGTGTAGGCG	0.068	0.004	0.07	36	480
CGCGTCGTAGGGG	0.037	0.015	0.018	10	543
CGTGGCACAAATG	0.029	0.007	0.02	21	1014
GGCGTTGTTGGGG	0.072	0.025	0.041	16	378
CGCAGTGTAGGTG	0.024	0.007	0.017	13	745
ACTGTCGCTGGTG	0.027	0.006	0.019	14	739
CGTAGTGTTGGGA	0.031	0.005	0.033	17	503
GCTGGTACTAGCA	0.073	0.013	0.081	79	896
GGCGTCGCATGCG	0.075	0.007	0.065	52	748

GCTGGCGTTAAGG	0.943	0.011	0.943	1109	67
GGCGGCGCTGGCA	0.086	0.021	0.11	82	664
CCTATCGTTGGTG	0.027	0.001	0.028	30	1056
AGTGTCGCAGGGG	0.023	0.007	0.023	10	434
GGCGGTGCTAATG	0.064	0.003	0.067	50	691
AGTGGCGCTGACG	0.021	0.007	0.014	9	625
CGTAGCGCTGGTG	0.025	0.003	0.025	12	477
GGTATTGTAGGCG	0.091	0.021	0.111	78	625
CGTGGCGTTGGGG	0.023	0.001	0.023	6	258
GCTAGCGCTGAGG	0.071	0.011	0.06	26	406
GGTGGTGCATGTG	0.114	0.007	0.122	47	339
GGTGGCATTAGTG	0.713	0.023	0.692	627	279
GGCGGCGTTTATG	0.455	0.068	0.43	336	446
GCTGGCATTGGCA	0.642	0.041	0.604	733	481
CGTAGTGTAGACG	0.029	0.004	0.026	21	785
CGTGTTATTGGGG	0.024	0.01	0.01	5	491
GCTAGTACTTAGG	0.068	0.009	0.059	42	664
GGTGTCATTGAGA	0.09	0.018	0.074	54	673
GCTAGCGTTGGGA	0.057	0.008	0.051	28	516
GCTGGCATTTAGG	0.873	0.027	0.881	918	124
CGCGTTGCTTGTG	0.025	0.007	0.016	12	736
GGTGTCATTGAGG	0.085	0.014	0.074	35	437
AGTGGCATTTAGG	0.029	0.009	0.039	7	174
GGCGGTGCAAGGG	0.061	0.019	0.066	36	506
CGTATCGCAAGTG	0.025	0.005	0.031	31	973
GCTGGCGCTTGCG	0.817	0.026	0.81	648	152
GCTGGCATATAGG	0.869	0.033	0.858	1133	187
GGTGTTACTTAGA	0.076	0.004	0.081	62	704
GCCGTCGTTGATG	0.056	0.007	0.049	40	771
AGTGGTATTGGGG	0.015	0.001	0.017	6	350
GCTATCGCTAGGA	0.051	0.006	0.048	36	714
GGCAGTGTTGACG	0.051	0.003	0.05	50	950
GCTGGCACAGAGG	0.442	0.027	0.443	353	443
GCTGGCGCAAGGG	0.817	0.04	0.815	643	146
CCCGGCATTAGGA	0.025	0.003	0.022	32	1450
CGTATCACTTATG	0.029	0.004	0.031	35	1110
CGTGGCATTTATA	0.023	0.002	0.02	19	937
GCCAGCGCTGAGG	0.053	0.01	0.057	42	689
GCTGGCACAGACG	0.444	0.03	0.438	481	616
GCTGTCGTATATG	0.066	0.011	0.055	40	684

GCCGGCGCTGAGG	0.753	0.048	0.738	622	221
GCTGGTGTTAGGA	0.422	0.052	0.394	265	407
AGTGGCATATGTG	0.025	0.011	0.011	3	277
GGCGGCGCTAGGG	0.184	0.014	0.165	78	396
CGTGGTGTTGGCG	0.028	0.006	0.037	16	422
GGTGGCGTAGAGG	0.67	0.056	0.688	413	187
GCCGGCGCTGATG	0.762	0.044	0.755	830	270
GCTGGCGTAAAGA	0.886	0.023	0.874	1618	234
GGTGGTATAAGTG	0.27	0.025	0.239	162	516
GCTGGCACTAGCG	0.462	0.041	0.443	347	436
CGTGTTGCTAAGG	0.032	0.008	0.026	15	569
AGTGTCGTTTAGG	0.026	0.004	0.021	12	567
GGTGTCATTGGTG	0.092	0.021	0.077	33	396
AGTGTTGTAGGTG	0.021	0.01	0.033	22	649
GCCATCGCTGGTG	0.05	0.005	0.044	34	732
AGCGTTGCTTGCG	0.018	0.004	0.024	21	851
AGTGGCACTTGGG	0.011	0	0.01	3	289
GGTAGCACTGAGG	0.069	0.022	0.1	47	423
CGTAGTGCATACG	0.025	0.009	0.023	18	780
AGTATTGTTTAGG	0.026	0.006	0.019	18	939
CCTAGCGCTAAGG	0.03	0.001	0.031	31	970
CGCGGTGTAGATG	0.023	0.005	0.021	18	840
GGCGGCGTTGGTG	0.425	0.066	0.422	237	324
GGTGGCATTAGGG	0.709	0.028	0.705	468	196
GCCGTCGCTGGTG	0.057	0.003	0.061	30	459
GGTATTGTAGGTG	0.1	0.013	0.084	50	543
AGTGGTATTTAGG	0.03	0.013	0.043	23	508
GGCAGTACATACG	0.053	0.008	0.054	90	1587
AGCGGCACTTACG	0.025	0.005	0.022	24	1080
AGTGGTGTTTGCG	0.023	0.003	0.021	10	469
GGTGGTGTTTGCG	0.207	0.06	0.135	48	307
AGTGGCGCTAGGG	0.026	0.002	0.023	8	335
GCCGTCGTTGGTG	0.062	0.017	0.07	37	489
GCTGGCGCTAGTG	0.81	0.021	0.818	635	141
GGTGTCGTAGGTG	0.106	0.035	0.085	34	364
GCCATTGCAGAGG	0.059	0.008	0.048	48	947
GGTGTCATTGGCG	0.092	0.024	0.065	33	471
AGTAGTGCTTAGG	0.016	0.004	0.019	11	583
AGTGGCGCTTGTA	0.014	0.001	0.016	10	631
GGCATTGTTGGCG	0.067	0.016	0.046	44	922

CGCGTCGTAAACG	0.031	0.004	0.036	62	1665
CGTGGCGCTGGGG	0.016	0.008	0.007	2	289
GGTGTCGCTGGGG	0.112	0.047	0.126	31	215
AGCGGCATATAGG	0.014	0.007	0.023	13	548
GGCGTCGTTGGTG	0.072	0.016	0.065	31	449
GCCGGCGTTGATA	0.861	0.019	0.858	1632	270
AGTGTCACTGGGG	0.025	0.003	0.021	10	469
GGTGTTACTAACG	0.063	0.015	0.054	47	816
CGTGGTACATGGG	0.019	0.006	0.012	6	511
CGTGGCGCTTATA	0.028	0.003	0.028	22	772
GGTGGCGCTTACG	0.283	0.047	0.237	116	373
GGCAGTGCAAGGG	0.066	0.006	0.069	48	644
GGTGTTGTAAAGA	0.078	0.015	0.069	64	870
AGTGGCATTTGCA	0.019	0.007	0.012	7	576
GGCGGTGCTTGCG	0.057	0.004	0.063	31	459
GGCGGTACAGGTG	0.076	0.026	0.039	23	567
GGTGTCGTATAGG	0.128	0.018	0.104	48	413
GGTATCGTTTGTG	0.14	0.024	0.107	51	427
GGTGTCGTTGAGG	0.084	0.013	0.084	32	350
ACTGGCGTTAGTG	0.029	0.009	0.034	15	420
ACCGTCACTAGGG	0.024	0.004	0.018	20	1080
GCTGGCATAAGGA	0.628	0.057	0.616	815	508
GCCGGCGTTTGCA	0.851	0.021	0.842	1269	239
AGTAGCGCTTGGA	0.03	0.005	0.022	13	566
GGCGGCGTTGAGG	0.447	0.045	0.443	253	318
GCTGGCGCAAATG	0.825	0.03	0.817	1167	262
GGCATCGTTAGTG	0.103	0.016	0.081	72	814
GCTGGTGCTAGGG	0.298	0.039	0.29	116	284
CGCATTGCTTAGG	0.022	0.008	0.012	12	964
GGTATTGTAGACG	0.105	0.012	0.089	84	861
GGTAGCGCTAGTG	0.071	0.003	0.067	30	418
ACTGGTGTAGGGG	0.023	0.009	0.011	6	533
AGTGTCGTAAAGG	0.027	0.007	0.036	35	933
CGTGTTATTAGGG	0.027	0.013	0.022	13	579
GCTATTGCTGGGA	0.056	0.006	0.064	47	689
GGTGGTACTGGCG	0.113	0.002	0.111	36	288
CGCGGTACTGGGG	0.022	0.006	0.015	8	542
CGTATTGTTTAGG	0.03	0.005	0.027	20	720
AGTGGCGTAGATG	0.025	0.004	0.026	9	333
GCTGTCACAGGTG	0.056	0.016	0.065	41	593

ACTGGCGCATGGA	0.024	0.005	0.018	9	497
GGTGGCGTATACG	0.694	0.035	0.671	676	331
GCCATTGTAGACG	0.049	0.005	0.044	73	1596
CGTGGTACTTGCG	0.025	0.008	0.036	18	488
GGTATTGCTGGCA	0.076	0.017	0.057	44	726
CGTGGCGCTGAGG	0.02	0.005	0.013	5	376
CGCAGCGCTAGGG	0.021	0.006	0.027	18	658
GGTGGTGTATATG	0.21	0.014	0.199	107	431
CCCAGTATAGGGG	0.044	0.005	0.044	53	1156
GGCGGTGCATAGG	0.067	0.01	0.078	44	517
GCCGGCATTGGCG	0.691	0.051	0.716	858	340
CGTGGCACTGGGG	0.023	0.007	0.027	9	322
GCCGGCGTTTGCG	0.93	0.02	0.922	1023	86
GGTAGCGTTTGGG	0.087	0.016	0.072	20	257
GCTGTTATTGAGG	0.066	0.016	0.046	35	725
GGTGTCGCTGGCG	0.089	0.005	0.086	37	392
GGTGGCGCTAGCG	0.315	0.044	0.304	123	281
GGTGGTATATGGA	0.138	0.025	0.131	71	473
GGCGGCGCTGAGG	0.165	0.039	0.128	65	444
GGTGTTGCTAGGG	0.082	0.018	0.071	22	286
CGTAGTGCATGGG	0.032	0.01	0.023	10	428
GGTAGCGTAAGGG	0.069	0.003	0.065	29	418
GGTGGTGCTAGGA	0.068	0.004	0.069	30	402
AGTGGCGCTGGCG	0.021	0.006	0.027	11	396
GGCGTTGCTGGGA	0.049	0.007	0.041	23	534
GGTGTCGCAAGGA	0.077	0.01	0.073	37	471
GCTAGCGCTTAGG	0.059	0.009	0.049	21	411
CGTGGCGTATATG	0.018	0.007	0.024	13	522
GCCGTTATAGGGG	0.089	0.002	0.086	56	595
CGTATCGCTTAGG	0.032	0.006	0.028	18	635
ACCGGTGCTGGTG	0.022	0.003	0.023	21	884
GCCGGTACTGGGG	0.063	0.007	0.056	28	468
AGTGGCGTAGGCG	0.024	0.005	0.018	5	270
CGCGGCGTAGGTG	0.028	0.004	0.025	14	548
CCTGTTACTTAGG	0.019	0.006	0.02	18	903
AGTGGCATATAGG	0.019	0.013	0.004	1	265
CCTGGCGCTTGGA	0.016	0.006	0.017	12	694
GCCGGCGTAGGGG	0.887	0.016	0.882	762	102
GGTGTCATTTGCG	0.113	0.019	0.101	52	462
AGTGGCATTTGCG	0.025	0.016	0.017	4	227

GGTGTCGCATATG	0.117	0.016	0.095	57	546
GGTATCGTTTGGA	0.147	0.028	0.119	75	556
CGTGGCGCTGGGA	0.021	0.005	0.018	7	378
ACTGGCGTATATG	0.037	0.003	0.035	20	555
CCTGGCGCTGGTG	0.021	0.01	0.032	19	572
GGTGTCGTTTGCA	0.1	0.017	0.08	41	474
CCTGGTGTTTGGG	0.015	0.001	0.013	7	513
GCTGTCACTTGGA	0.059	0.01	0.047	34	695
GGTGGTGCAGAGG	0.098	0.006	0.094	31	299
GGCGGCACTGGGG	0.208	0.047	0.163	80	412
AGTGGCATAAACG	0.025	0.003	0.029	13	442
GCTGGTGTTTGGA	0.395	0.058	0.387	212	336
GGTGGCACAGGCG	0.374	0.06	0.366	247	427
GCTGTCACTTGTG	0.062	0.012	0.075	47	576
AGTGTCGTTGGTA	0.021	0.003	0.019	14	724
GCTGGTGCAGACA	0.116	0.012	0.1	101	909
GCTGGCGCTGGTG	0.814	0.031	0.793	488	127
GGTGTTGCTGGGG	0.067	0.028	0.028	7	239
AGTAGCATTGGTG	0.024	0.004	0.028	18	623
GGTGGCGCATGCA	0.156	0.031	0.128	72	490
GGTATCGCTAGCG	0.112	0.013	0.095	58	554
GCTGGCATAAGCG	0.863	0.026	0.877	1341	188
GCCGGCGCTTACG	0.743	0.043	0.72	803	312
GCCGGCGTTGGTA	0.854	0.025	0.833	1055	211
CGTGGCATTGATA	0.031	0.004	0.027	26	951
CGTGGCGTTGAGG	0.029	0.01	0.023	7	291
GGTAGCGCATATG	0.079	0.016	0.065	37	528
CGTAGTGCTGGCG	0.02	0.005	0.014	7	496
GCTGTTGCTTGCG	0.067	0.003	0.065	33	477
GGTGGTGCTGAGG	0.079	0.022	0.048	13	256
GGTAGTGTAGGGG	0.07	0.006	0.071	35	455
GGTAGTATTAGTG	0.059	0.003	0.056	40	674
CGTGGCACTGAGG	0.032	0.013	0.016	7	427
GGTGGCGCATACG	0.316	0.012	0.31	200	446
GCTGGCACAAGGG	0.458	0.033	0.429	337	448
CGTGGTGCTGGTA	0.024	0.006	0.02	11	534
GGTGTCACTAGGG	0.092	0.005	0.089	35	359
GGTGGTGCTAGGG	0.084	0.017	0.068	17	233
GGCAGTGCAGGGG	0.035	0.005	0.04	20	479
GCTGGCGTAGACG	0.943	0.019	0.944	1407	83

GCTGTTGTTGGTG	0.061	0.017	0.06	27	423
GGTGTCGCTAGTG	0.11	0.035	0.078	31	365
GCCGGCGTATACG	0.935	0.01	0.935	1894	132
GGTGTTACTTGGG	0.084	0.017	0.077	31	371
GGTGTTGCTGATG	0.085	0.008	0.076	37	453
GCTGGCGCTGGTA	0.598	0.043	0.611	511	326
GGCAGCGTTAGCG	0.068	0.008	0.067	50	699
AGTGGTGCTAGGG	0.024	0.002	0.026	9	336
GGTGGCGTTTGGA	0.385	0.041	0.404	162	239
GGTGGCGTTGATA	0.381	0.042	0.346	239	451
GGTGGTGCTTGGA	0.076	0.004	0.074	28	351
GGTGGCGCTGAGG	0.295	0.07	0.252	79	235
GGCGGTGTTGGTG	0.093	0.011	0.082	31	348
CGTGGCATATGTG	0.024	0.003	0.026	12	449
CCTGGCGTAGGGG	0.033	0.007	0.032	14	427
GGTAGTGTAAGCG	0.062	0.012	0.076	53	645
CGTATTGCAGGTA	0.022	0.004	0.017	18	1069
AGTGGCGTTTGTG	0.031	0.012	0.023	6	254
GGTAGCGCAGAGG	0.092	0.004	0.098	43	398
GGTGTCGTTAGTG	0.11	0.02	0.094	34	326
AGCAGTGCTTAGG	0.02	0.005	0.015	15	1005
CGTAGTGTTGGTA	0.03	0.001	0.03	19	609
CGTGGCGTTAATG	0.025	0.002	0.028	15	516
GCTGTCGCTGGGG	0.051	0.008	0.041	12	282
AGCGGTGCTGGGG	0.017	0.004	0.023	10	425
GCTGGTGCTGGTA	0.123	0.025	0.143	80	478
AGTGGCGTTGGCG	0.027	0.006	0.018	4	215
GGTGTTACAAGGA	0.072	0.012	0.068	51	698
GCTGGCGTTGGGA	0.89	0.032	0.901	720	79
GGTGGTGTAGGCG	0.203	0.031	0.201	84	333
GGTGGCATATAGG	0.709	0.061	0.678	654	311
GCTGTCACTGGGG	0.054	0.005	0.061	27	416
GGTATTGTATGGG	0.101	0.03	0.076	37	449
CGTGGTGCAGGGG	0.024	0.015	0.03	10	328
GGTGGTACAGGTG	0.115	0.013	0.119	48	354
GGCGGCATTAGTG	0.55	0.053	0.538	556	477
CGTATTGCTTGGG	0.031	0.006	0.025	12	470
CGTGTTGTAGGGA	0.02	0.004	0.026	15	567
GGTGTTGTAGATG	0.083	0.006	0.074	44	552
CGTAGTGCTGGGG	0.018	0.003	0.021	6	274

GGTGTCGTATGTG	0.118	0.012	0.102	51	447
CGCGGCGCTTGGA	0.023	0.005	0.026	17	626
GGCGGCGTATACG	0.455	0.034	0.415	458	645
AGTGGTATTAGGG	0.02	0.002	0.023	10	431
GGTGTCGTTAGTA	0.103	0.02	0.095	52	497
ACTGGTGCATGGG	0.029	0.008	0.023	14	606
GCTGGTACAGATG	0.121	0.023	0.109	86	703
GGCGTTGTATGTG	0.074	0.009	0.072	47	603
GCCGTCGTTTGCG	0.062	0.004	0.056	37	619
ACTGTCATTGGGG	0.038	0.008	0.033	22	642
GGTGGTACTGGTG	0.112	0.027	0.084	30	326
GGTATCGCTGATG	0.138	0.005	0.143	103	615
CGTGGCATAGGGA	0.02	0.004	0.02	11	533
GCTGGTGTTTATG	0.764	0.038	0.739	740	261
GCTATCACATGTG	0.064	0.011	0.067	78	1088
AGTAGCGTTTGGG	0.022	0.003	0.026	13	485
CGCGGTGTTGGCA	0.027	0.005	0.022	19	858
GGTAGCGCTGGGG	0.088	0.007	0.08	22	253
CGTGGCATTGATG	0.028	0.003	0.029	16	533
CCTGGTATTGGGG	0.025	0.004	0.03	15	490
GGTGGTGTTAGTG	0.178	0.018	0.202	69	273
ACTGGTGCTGAGG	0.024	0.007	0.022	14	614
CGCGGCGTAAGTG	0.019	0.005	0.016	13	811
GGTGGCGCTGATG	0.336	0.037	0.347	162	305
CGTGGCGTATGTG	0.028	0.007	0.036	15	396
GGTAGTGTTGACG	0.069	0.018	0.058	39	632
GGTAGTGCATGCA	0.056	0.012	0.042	27	622
GCCGGCGTATGGA	0.85	0.035	0.848	1101	198
CGTAGTGTAGGGG	0.037	0.006	0.03	13	418
CGTGTCATTGGGG	0.018	0.007	0.023	10	419
GCTAGCGTTGGGG	0.082	0.022	0.11	39	314
GCTGGTGTTGGTG	0.728	0.023	0.739	456	161
CGCGGTGTTTGGG	0.018	0.009	0.005	2	382
GCTGTCGCTTGCG	0.037	0.007	0.031	14	434
GCTGGCGTTGGTG	0.943	0.021	0.918	696	62
GCTGGCGTATACG	0.944	0.01	0.95	1520	80
GGCAGCGCAGGTA	0.047	0.005	0.049	46	893
GCTGGCGTTGATA	0.887	0.022	0.888	1403	177
GGTGGTATTAGCG	0.269	0.052	0.224	130	450
GGTGGCGCATGCG	0.295	0.035	0.262	119	335

**GGTATCATTGGGG**	0.11	0.011	0.096	41	386
**GGTGGCATAGGGG**	0.589	0.074	0.518	353	328
**GCCGGCGTAAGGA**	0.852	0.034	0.843	1245	232
**CGTGTTACTTAGG**	0.027	0.006	0.032	20	605
**GCTGTCACATGGG**	0.051	0.005	0.055	28	483
**ACTGTTGTAGAGG**	0.027	0.008	0.023	23	959
**GCCGGCGTTAAGA**	0.862	0.025	0.842	1476	278
**AGTGGCATTAAGG**	0.031	0.017	0.006	2	306
**ACTGGCGTTTGCG**	0.035	0.008	0.038	16	403
**CGCAGTATTGGGG**	0.035	0.004	0.03	19	616
**GCTGGTGTTGACG**	0.744	0.047	0.735	775	279
**GGTGGCACAGGGA**	0.161	0.016	0.154	72	397
**GCTAGCGCTTGGG**	0.06	0.001	0.061	19	295
**GGTGGCGCATAGG**	0.31	0.027	0.31	136	303
**GGTGGCACTGGGG**	0.363	0.061	0.338	128	251
**GGCGTCGCAAAGG**	0.068	0.012	0.052	42	767
**GGTGGTGTTAACG**	0.226	0.029	0.198	123	498
**AGCGTCACTGGGG**	0.021	0.001	0.021	15	698
**GGTGTTGCTTAGG**	0.094	0.004	0.099	36	326
**GGTAGCGTTAAGA**	0.091	0.022	0.068	47	646
**CGTGGCGTTAAGG**	0.026	0.002	0.023	10	419
**GGTGTCGCAAACG**	0.111	0.015	0.1	85	762
**CGCGGCGTATGGG**	0.025	0.003	0.023	12	504
**GGTGGCGTATGGG**	0.667	0.058	0.665	352	177
**CGTGGTGCATGGG**	0.023	0.003	0.023	8	333
**GGTGGTACAGGGG**	0.097	0.011	0.093	27	262
**GCCGGCGTTTGTA**	0.848	0.035	0.844	1117	207
**GGTGGCGTAAATG**	0.688	0.059	0.672	820	400
**AGTGGCACTGAGG**	0.019	0.004	0.013	6	445
**GCTGGTGTTAGCG**	0.743	0.04	0.731	632	233
**CGTAGTGTTGGTG**	0.026	0.001	0.025	10	386
**GGCGGCACTAGGG**	0.265	0.064	0.244	163	504
**CGTGTCGCAGGCG**	0.029	0.004	0.025	17	650
**GGTGGCGTTGGGG**	0.646	0.037	0.617	238	148
**CGTATTGTTGGGA**	0.02	0.008	0.016	11	687
**GCCGGTGTTGGGG**	0.376	0.08	0.339	162	316
**GCTAGTATAGGGG**	0.073	0.001	0.072	45	577
**GGTGGCACTGAGG**	0.376	0.054	0.355	198	360
**GCTAGCGCTAGGG**	0.07	0.008	0.069	32	430
**GCTGGTGTTTGGG**	0.74	0.061	0.755	376	122

GGTGTTATATACG	0.1	0.009	0.093	87	853
GCTGGCGTAGAGA	0.869	0.03	0.862	1078	173
ACTGGTGTTAGGG	0.027	0.003	0.031	18	564
GCCGGCACTAGTG	0.213	0.032	0.198	201	813
GGTAGTGTTAAGG	0.06	0.01	0.047	26	526
GGTGGCATTTGGG	0.693	0.05	0.639	383	216
GCCGTCGTTGGTA	0.048	0.008	0.054	44	769
GGTGGCGTAGATG	0.666	0.063	0.652	537	287
GCTGGTGTAGGGG	0.678	0.064	0.61	304	194
GCTGGTGTATAGG	0.735	0.045	0.718	633	249
CGTAGCACTTGTG	0.025	0.004	0.023	14	601
GGTGTTGTAGAGG	0.08	0.01	0.08	34	392
CGTGTTGCTGGTG	0.03	0.01	0.043	22	484
GGTGTCGTTTATG	0.098	0.011	0.083	39	430
CGTAGTGCTTGCA	0.029	0.01	0.017	12	707
GCTGGCGTTTACA	0.879	0.021	0.881	1449	195
GCTGGTATTAGGG	0.42	0.041	0.398	254	384
GCCGGCGCTAGCG	0.739	0.04	0.699	748	322
AGTGTTACTTGTG	0.02	0.003	0.022	18	805
GCCGGCGCTGGTG	0.734	0.06	0.731	611	225
AGTGGTGCTGGTA	0.024	0.007	0.014	9	630
GCCGTCGCAGGGG	0.047	0.005	0.04	16	381
CGTGGCGCATGCG	0.031	0.007	0.024	13	527
GCTGGTATAGGCG	0.425	0.05	0.417	349	487
GGCGGCGCTAATG	0.198	0.047	0.223	188	656
GCCAGCGTTGGGG	0.066	0.015	0.05	25	474
AGCGGCATTGGTG	0.031	0.009	0.044	17	370
GCCGTCGTTGAGG	0.067	0.007	0.062	35	534
GCTGGTGTATGGG	0.718	0.043	0.738	422	150
GGTAGTACTTGGG	0.081	0.02	0.073	31	392
GCTGGCATTTGGG	0.875	0.034	0.862	669	107
GGTGGCGTAAGGG	0.656	0.027	0.665	435	219
GGTGGTGTTGGGG	0.167	0.025	0.168	37	183
GCTGGTGTTGATG	0.742	0.04	0.703	631	266
GCTGGCGTTGGCA	0.873	0.025	0.895	1071	125
CGTGGCACTGGCG	0.026	0.01	0.026	13	494
CCTGGTATTTGGG	0.03	0.004	0.025	16	634
GCCGGCGCAAGGG	0.749	0.029	0.746	655	223
GGCGGCGCTTGGG	0.156	0.049	0.126	51	353
AGTGGCGCAGGGG	0.028	0.006	0.035	11	302

GGTGGCGTAGGCG	0.67	0.029	0.677	418	199
GGTGTCGCAGGTA	0.081	0.024	0.052	28	508
GCTAGCGCTGGGA	0.062	0.011	0.049	25	487
GCTGTCGCTAGGG	0.047	0.013	0.049	17	330
ACCGTCATTAGGG	0.026	0.009	0.03	33	1049
GGTGGTGCAGGTG	0.09	0.012	0.075	22	270
AGTGTTGCAGGGG	0.031	0.012	0.038	16	401
GGCGGCATAGATG	0.551	0.055	0.522	708	648
GCTGGCGTATAGG	0.944	0.014	0.934	1032	73
GCTGGTGTTTGCG	0.73	0.055	0.685	505	232
CGTGGTATAGGTG	0.027	0.004	0.026	14	535
GCCGGTGCAGGCA	0.066	0.004	0.062	60	905
GCTGTCGCTTGGA	0.046	0.002	0.044	20	431
GCTATCGCTAGGG	0.045	0.009	0.032	16	486
GGTGGCATAAGGG	0.713	0.037	0.694	564	249
GCTGGTGTAAACG	0.757	0.034	0.739	1209	428
GGCGTTGCTTGGG	0.07	0.013	0.052	22	399
GGCAGTATAGGGG	0.051	0.016	0.058	53	867
AGTAGCGCAGGTG	0.028	0.007	0.037	22	565
GGTGGCGTTAGCG	0.702	0.059	0.712	445	180
GCCGGCGCTAATG	0.753	0.039	0.753	972	318
AGTGTCGCTGGGA	0.024	0.009	0.037	19	501
GCCGGCGTTAGTG	0.916	0.017	0.915	1113	104
GGTATTGCTTGGG	0.109	0.01	0.099	35	319
AGTGGCGTAAGGG	0.019	0.004	0.018	5	268
GCCGGCGTAAGTG	0.937	0.015	0.932	1385	101
GCCGGCGTAAGCA	0.843	0.029	0.846	1793	327
CGTGGTGCTGGCG	0.03	0.006	0.024	10	410
AGTGGCGTATATG	0.026	0.011	0.013	5	393
GGTGGCACAGGGG	0.426	0.027	0.419	182	252
GCCGGCGTAGGTG	0.928	0.016	0.922	1044	88
GCTGGCGTTGACA	0.886	0.017	0.88	1462	200
AGTGGTACTGGGG	0.024	0.014	0.044	16	347
GGTATTGCTAGTG	0.099	0.019	0.099	61	556
GCCGGCGTTTAGA	0.855	0.019	0.835	1186	234
GGTGGCATAGGTG	0.709	0.038	0.673	521	253
GCCGGCGCTAGGG	0.749	0.043	0.748	573	193
GCCGGCATTAGCG	0.726	0.039	0.725	1040	395
GGTGGCGCATGTG	0.317	0.02	0.298	123	290
GCCGGCGTAAAGG	0.928	0.012	0.937	1503	101

GCTGGTGTTAAGG	0.745	0.039	0.731	662	244
GCTGGTGTTAATG	0.752	0.05	0.756	922	298
GCTGGCGTTAAGA	0.883	0.017	0.888	1344	170
GCCGGCGCTAAGG	0.743	0.044	0.731	786	289
GCTGGCGCATATG	0.828	0.029	0.822	936	202
AGTGGCGTTAGGG	0.01	0.011	0.005	1	216
GGTGTCGCATAGG	0.096	0.029	0.06	26	405
AGTGGCGTAGAGG	0.015	0.004	0.015	4	266
GGCAGTGCTGGGG	0.049	0.006	0.056	24	408
GGTGGTGTTTGCA	0.116	0.017	0.092	39	383
GCCGGCGTATGGG	0.931	0.01	0.935	888	62
AGCGGCATAGGCG	0.021	0.003	0.017	10	570
GCTGGCGTTTGTA	0.883	0.026	0.875	945	135
GGTGGCGTTGAGA	0.375	0.048	0.382	187	303
AGTGGTATAGGGA	0.019	0.002	0.019	14	725
AGTGGCATAGGTG	0.024	0.012	0.04	9	215
GGTATTGCTGGGG	0.092	0.024	0.059	18	287
GCTGGCATTTGCG	0.86	0.024	0.866	946	147
GCTATCGCTTGGG	0.045	0.019	0.019	8	424
GGTAGTGTTGGCG	0.044	0.009	0.037	17	438
GGTGGTGTTTGTG	0.194	0.037	0.201	74	294
GGTGGCGTATATG	0.663	0.047	0.664	582	294
GCTGGCGTTAATG	0.942	0.021	0.931	1451	107
GGTGTTGCTTGGG	0.094	0.009	0.087	24	253
GGTATCGCAGGTG	0.115	0.016	0.092	47	463
GGTGGCATTGGCG	0.678	0.045	0.659	457	236
GCTGGCGCTAAGG	0.799	0.023	0.783	634	176
GCCGGCGTAAGGG	0.925	0.009	0.927	1087	85
GCTGGCGCATGGG	0.835	0.031	0.848	559	100
GCTGGCGTTTGCA	0.89	0.025	0.874	1034	149
GGTGGTGTTTGGG	0.205	0.039	0.219	56	200
GCTGGCGTAGGCG	0.942	0.011	0.949	964	52
GGTGTTGCAGGCA	0.084	0.022	0.054	33	577
GGTGTTGTTGGGG	0.1	0.021	0.099	29	264
GCTGGTACTGAGG	0.107	0.014	0.094	46	442
GCCGGCATAAGGG	0.507	0.051	0.489	495	518
GCTGTCGCAGGTG	0.059	0.007	0.059	26	418
GCTGGTGCTTGGG	0.281	0.037	0.284	88	222
GCTGGCGCATGTG	0.823	0.033	0.825	729	155
GCTGGCGCAGGTG	0.81	0.044	0.793	606	158

GCTGGCGTAAGTG	0.94	0.02	0.949	1222	66
GCTGGCATAGAGG	0.847	0.03	0.816	876	197
GCCGGCGTTGACG	0.929	0.018	0.93	1695	127
GCCGGCGTTTATG	0.929	0.016	0.931	1303	96
GGTGGCGTTGGCG	0.637	0.015	0.625	339	203
GCCGGCGTTAGGG	0.93	0.024	0.902	879	96
GGTGGCGTTGGGA	0.37	0.047	0.399	132	199
GCTGGCGTTTACG	0.946	0.007	0.949	1191	64
CGTGTTGCTGGGG	0.017	0.011	0.016	5	299
GCTGGCGCTAGGG	0.818	0.038	0.789	476	127
GCTGGCGTTGGCG	0.933	0.021	0.924	830	68
GCTGGCGTTTATA	0.876	0.024	0.876	1283	181
GGTGGCGCAGGGG	0.276	0.026	0.239	68	216
GCTGGCGCTAGCG	0.821	0.031	0.802	687	170
GGTGGCGTTAGGA	0.392	0.077	0.334	147	293
AGTGGCGTTTAGG	0.032	0.024	0.005	1	212
GCCGGCGTTGAGA	0.863	0.026	0.847	1242	224
GCCGGCGTTAACG	0.932	0.011	0.939	1879	123
GCTGGCGCAGAGG	0.812	0.014	0.815	602	137
GCTGGCGCAGGGG	0.823	0.034	0.817	464	104
GCCGGCGCTTGCG	0.746	0.049	0.733	677	247
GCTGGCGTTAACG	0.949	0.013	0.947	1595	90
GCTGGCGCTGGGG	0.807	0.057	0.793	364	95
GCTGGCGTTAGTG	0.949	0.012	0.95	935	49
GCTGGTGTTGAGG	0.748	0.049	0.721	494	191
GCCGGCGTTGGCG	0.926	0.01	0.921	1001	86
GCCGGCGTTTGGA	0.859	0.035	0.847	904	163
GCTGGCGTATGGG	0.937	0.022	0.919	708	62
GCTGGCGCAGGCG	0.81	0.035	0.812	704	163
GCTGGCGTAAGCG	0.944	0.019	0.941	1265	80
GCCGGCGTTGATG	0.922	0.01	0.917	1323	120
GGTGGCGTTTGTG	0.675	0.037	0.677	361	172
GCTGGCGTAAGGG	0.947	0.018	0.94	916	58
GGTGGTGCTTGCG	0.085	0.024	0.067	18	249
GGCGGCGTAGGTG	0.438	0.065	0.439	274	350
AGTGGCGTTGGTG	0.03	0.01	0.042	9	207
GCTGGTGTTAGGG	0.746	0.039	0.702	424	180
GCTGGCGTTTAGG	0.959	0.006	0.961	817	33
GCTGGCGCTGAGG	0.835	0.033	0.854	638	109
GCCGGCGTTGGGG	0.915	0.023	0.914	640	60

GCCGGCGTTTGTG	0.936	0.015	0.933	970	70
GCTGGCGTTGGGG	0.953	0.002	0.951	598	31
GCCGGCGTATAGG	0.929	0.019	0.913	1130	108
GCTGGCGTATGTG	0.945	0.016	0.945	1069	62
GCTGGCGTAGGGG	0.913	0.023	0.896	551	64
GCTGGCGCTTAGG	0.837	0.023	0.833	600	120
GCCGGCGTTAGCG	0.939	0.008	0.942	1298	80
GCTGGCGTTTGGG	0.945	0.021	0.942	585	36
GCTGGCGTTAGGG	0.936	0.018	0.943	705	43
GCTGGCGTTTGTG	0.952	0.018	0.965	755	27

Replicate 2			Replicate 3		
fitness	cleaved	uncleaved	fitness	cleaved	uncleaved
0.031	55	1701	0.023	29	1206
0.016	16	1001	0.024	20	815
0.014	7	509	0.037	17	446
0.027	35	1251	0.029	30	996
0.021	35	1607	0.03	45	1453
0.021	48	2194	0.023	44	1898
0.02	42	2015	0.025	44	1702
0.03	88	2882	0.021	51	2391
0.026	95	3542	0.03	90	2927
0.029	75	2551	0.031	67	2084
0.021	28	1298	0.03	36	1149
0.034	213	6004	0.03	148	4803
0.03	54	1770	0.02	29	1434
0.023	32	1386	0.028	35	1220
0.033	59	1737	0.057	86	1411
0.055	67	1152	0.056	53	899
0.026	54	1998	0.027	46	1669
0.024	56	2313	0.028	62	2164
0.029	55	1858	0.03	52	1666
0.025	50	1936	0.031	51	1621
0.024	47	1944	0.029	44	1468
0.018	9	483	0.023	11	458
0.02	35	1694	0.025	36	1415
0.031	29	912	0.018	13	713
0.022	123	5492	0.025	116	4555
0.017	20	1188	0.02	21	1052
0.061	160	2478	0.052	117	2134
0.102	114	1007	0.112	105	831
0.009	9	977	0.023	19	817
0.027	68	2474	0.023	52	2181
0.058	142	2309	0.083	159	1760
0.053	129	2313	0.058	121	1957
0.023	106	4559	0.026	102	3783
0.024	112	4629	0.019	78	4101
0.025	60	2319	0.021	41	1958
0.022	40	1820	0.029	44	1465
0.017	41	2315	0.024	52	2122
0.019	21	1066	0.024	22	911

0.013	19	1421	0.022	28	1220
0.058	147	2374	0.049	100	1958
0.05	52	988	0.057	56	922
0.025	71	2752	0.023	52	2163
0.092	64	630	0.121	77	558
0.017	18	1065	0.021	21	1001
0.022	16	716	0.031	19	590
0.414	365	516	0.551	528	431
0.022	70	3142	0.022	59	2585
0.018	10	559	0.019	9	454
0.05	115	2165	0.057	103	1718
0.024	30	1225	0.026	25	953
0.026	72	2716	0.026	66	2487
0.077	151	1814	0.07	105	1390
0.02	26	1274	0.03	35	1128
0.061	108	1661	0.063	88	1303
0.067	74	1025	0.063	56	831
0.02	69	3297	0.028	84	2941
0.027	36	1277	0.022	24	1061
0.034	36	1036	0.027	27	989
0.023	42	1773	0.022	31	1355
0.021	57	2679	0.021	48	2211
0.016	28	1701	0.021	32	1458
0.018	19	1063	0.021	18	841
0.018	13	715	0.014	9	648
0.014	26	1816	0.026	41	1554
0.023	35	1476	0.022	26	1148
0.023	82	3474	0.024	73	3015
0.072	87	1119	0.062	63	958
0.027	29	1049	0.04	39	937
0.065	50	719	0.054	34	599
0.024	24	986	0.034	29	826
0.1	142	1285	0.116	133	1013
0.055	65	1115	0.064	67	972
0.028	89	3124	0.026	74	2791
0.01	9	903	0.03	24	773
0.057	78	1298	0.05	57	1085
0.059	100	1605	0.074	101	1255
0.02	52	2510	0.023	50	2083
0.041	72	1669	0.043	66	1475

0.021	84	3954	0.02	68	3404
0.028	127	4415	0.02	75	3616
0.026	66	2504	0.023	50	2151
0.022	49	2229	0.025	44	1728
0.016	100	6110	0.025	137	5387
0.021	45	2129	0.024	42	1691
0.033	77	2270	0.026	51	1875
0.026	104	3839	0.025	82	3148
0.055	128	2190	0.062	121	1829
0.068	100	1368	0.091	108	1083
0.063	97	1455	0.064	77	1129
0.019	63	3172	0.019	54	2765
0.029	66	2179	0.03	57	1821
0.053	120	2123	0.057	108	1798
0.022	94	4133	0.021	82	3749
0.025	137	5400	0.027	129	4627
0.02	27	1329	0.022	23	1038
0.026	128	4794	0.022	92	4152
0.026	123	4608	0.018	77	4126
0.065	77	1111	0.09	89	905
0.021	63	2925	0.03	76	2446
0.03	70	2231	0.031	58	1840
0.02	57	2826	0.028	65	2267
0.018	31	1676	0.023	34	1438
0.028	115	4002	0.024	82	3379
0.031	29	899	0.014	10	694
0.019	21	1111	0.022	22	994
0.028	35	1217	0.024	25	1022
0.066	74	1046	0.049	45	879
0.052	94	1728	0.058	82	1324
0.029	55	1813	0.023	36	1530
0.05	86	1628	0.053	83	1488
0.026	63	2396	0.033	65	1920
0.031	52	1624	0.032	43	1317
0.029	122	4021	0.028	101	3492
0.027	51	1873	0.022	35	1546
0.019	23	1216	0.03	33	1069
0.029	65	2197	0.031	53	1647
0.018	26	1437	0.037	43	1129
0.063	80	1196	0.082	94	1053

0.021	53	2510	0.024	47	1903
0.051	74	1378	0.07	86	1135
0.028	82	2825	0.023	53	2239
0.027	150	5378	0.025	128	4943
0.046	46	946	0.066	57	811
0.025	46	1791	0.03	46	1486
0.018	67	3749	0.029	102	3446
0.025	49	1900	0.033	51	1517
0.022	37	1608	0.029	41	1381
0.029	97	3297	0.022	64	2885
0.026	72	2719	0.022	52	2344
0.026	56	2069	0.028	57	1960
0.022	19	856	0.03	19	619
0.155	161	875	0.197	199	809
0.024	95	3939	0.026	89	3327
0.026	77	2891	0.021	51	2399
0.108	152	1253	0.105	122	1044
0.034	45	1283	0.028	31	1093
0.021	108	4963	0.02	95	4600
0.199	178	718	0.229	186	626
0.025	25	976	0.031	26	809
0.024	107	4364	0.025	92	3657
0.03	95	3030	0.03	80	2565
0.063	56	829	0.051	37	690
0.02	62	3111	0.024	69	2780
0.028	55	1903	0.024	38	1574
0.092	141	1389	0.084	100	1093
0.051	65	1207	0.06	63	986
0.065	90	1285	0.061	70	1085
0.029	154	5176	0.023	102	4422
0.029	79	2647	0.024	61	2438
0.024	32	1323	0.025	29	1115
0.04	19	452	0.02	8	397
0.022	23	1002	0.02	15	752
0.034	32	911	0.027	22	789
0.022	74	3328	0.024	67	2708
0.021	53	2444	0.026	63	2320
0.02	16	771	0.034	25	701
0.026	59	2222	0.03	58	1874
0.024	99	4078	0.026	90	3343

0.075	51	631	0.123	70	497
0.017	28	1589	0.034	45	1268
0.049	116	2266	0.047	100	2016
0.022	25	1121	0.022	21	929
0.015	25	1624	0.026	36	1329
0.027	34	1228	0.025	32	1247
0.019	101	5134	0.026	120	4548
0.027	46	1680	0.025	37	1434
0.055	108	1846	0.049	81	1573
0.025	78	3068	0.027	72	2595
0.025	97	3747	0.027	87	3120
0.025	71	2811	0.019	47	2412
0.02	56	2697	0.025	64	2468
0.012	21	1737	0.031	49	1541
0.023	99	4178	0.023	78	3388
0.075	114	1407	0.06	71	1116
0.022	59	2609	0.03	68	2232
0.03	41	1334	0.059	63	1004
0.034	49	1402	0.022	26	1167
0.04	67	1606	0.052	74	1350
0.027	75	2734	0.03	69	2256
0.028	58	1999	0.029	46	1534
0.026	52	1986	0.035	57	1589
0.025	25	963	0.033	27	780
0.033	69	2028	0.021	38	1755
0.02	116	5649	0.021	95	4447
0.084	66	722	0.098	65	598
0.029	50	1662	0.03	48	1529
0.026	38	1428	0.017	23	1327
0.021	47	2243	0.02	40	1934
0.03	37	1179	0.031	34	1072
0.029	83	2822	0.027	66	2373
0.053	66	1174	0.09	90	914
0.028	69	2429	0.027	61	2197
0.029	86	2887	0.028	71	2429
0.026	91	3446	0.025	70	2689
0.064	105	1530	0.079	104	1206
0.018	32	1713	0.024	36	1469
0.024	41	1678	0.021	28	1304
0.022	36	1590	0.024	33	1324

0.024	43	1727	0.025	38	1505
0.023	65	2725	0.019	45	2385
0.074	80	995	0.107	89	745
0.024	64	2562	0.029	63	2129
0.028	105	3650	0.02	59	2920
0.026	85	3174	0.023	63	2687
0.024	43	1742	0.022	36	1568
0.019	47	2390	0.024	52	2083
0.161	62	322	0.23	74	248
0.024	119	4921	0.027	111	4071
0.02	90	4427	0.021	81	3763
0.077	289	3482	0.098	315	2908
0.048	59	1166	0.054	51	890
0.027	22	785	0.018	11	607
0.021	43	1967	0.023	39	1690
0.022	64	2791	0.025	68	2619
0.025	67	2583	0.017	41	2435
0.023	30	1294	0.027	27	989
0.02	47	2258	0.024	48	1958
0.09	68	691	0.092	57	560
0.023	53	2214	0.028	52	1787
0.026	38	1444	0.022	28	1264
0.055	92	1580	0.047	65	1311
0.021	51	2372	0.027	56	2023
0.025	53	2061	0.027	51	1811
0.029	76	2514	0.023	50	2087
0.028	53	1835	0.028	42	1480
0.016	12	741	0.025	18	704
0.08	171	1955	0.078	135	1605
0.022	30	1352	0.034	42	1187
0.016	55	3344	0.024	68	2794
0.016	48	2933	0.019	51	2575
0.044	76	1650	0.053	73	1310
0.02	26	1252	0.025	25	960
0.02	52	2512	0.021	44	2049
0.057	116	1919	0.052	93	1690
0.019	62	3118	0.018	48	2573
0.027	60	2188	0.025	46	1772
0.034	38	1069	0.026	28	1032
0.031	18	559	0.056	25	425

0.02	35	1699	0.016	23	1419
0.026	181	6871	0.026	154	5827
0.045	65	1392	0.052	68	1244
0.022	110	4894	0.022	89	3882
0.021	37	1728	0.021	32	1520
0.028	36	1241	0.029	29	981
0.114	99	767	0.149	106	604
0.03	125	4065	0.019	69	3537
0.047	177	3585	0.054	168	2931
0.024	87	3604	0.033	103	2982
0.019	88	4463	0.022	86	3794
0.03	49	1605	0.024	33	1336
0.024	87	3569	0.025	77	2991
0.03	138	4490	0.023	87	3719
0.031	36	1132	0.035	37	1028
0.031	41	1294	0.032	40	1223
0.03	57	1874	0.034	56	1586
0.032	94	2874	0.029	71	2357
0.081	71	810	0.087	66	692
0.073	75	950	0.078	74	873
0.022	88	3855	0.021	71	3333
0.026	39	1485	0.019	26	1366
0.171	129	627	0.195	114	472
0.021	58	2684	0.033	76	2195
0.026	51	1919	0.024	37	1497
0.017	25	1469	0.031	37	1159
0.058	143	2303	0.06	124	1959
0.018	17	937	0.038	33	844
0.057	70	1159	0.051	49	905
0.02	54	2656	0.024	55	2242
0.023	29	1232	0.023	23	995
0.026	54	2014	0.028	50	1732
0.027	72	2597	0.027	64	2309
0.027	47	1722	0.017	25	1479
0.029	62	2084	0.019	33	1745
0.023	42	1803	0.017	27	1533
0.023	48	2063	0.02	35	1709
0.059	152	2436	0.068	150	2048
0.029	73	2449	0.029	62	2079
0.021	30	1369	0.032	41	1238

0.027	82	2950	0.03	79	2574
0.03	14	454	0.022	9	405
0.032	87	2622	0.023	51	2154
0.023	42	1805	0.024	40	1595
0.026	105	3860	0.024	84	3476
0.023	88	3773	0.029	102	3449
0.051	49	917	0.052	39	711
0.016	44	2678	0.019	45	2376
0.015	21	1374	0.02	21	1041
0.026	57	2103	0.027	49	1778
0.028	69	2437	0.023	45	1934
0.039	95	2325	0.027	52	1889
0.027	49	1771	0.03	51	1641
0.022	37	1654	0.028	38	1336
0.021	45	2134	0.018	33	1798
0.021	40	1893	0.024	38	1520
0.023	53	2245	0.023	45	1942
0.026	43	1620	0.019	26	1360
0.057	86	1431	0.063	74	1107
0.029	58	1932	0.025	42	1662
0.613	1290	813	0.698	1564	678
0.083	76	836	0.15	111	630
0.024	64	2654	0.028	67	2341
0.022	64	2911	0.021	56	2605
0.064	161	2371	0.067	142	1991
0.025	56	2157	0.017	32	1852
0.023	73	3105	0.024	63	2533
0.046	40	825	0.044	32	703
0.022	65	2943	0.018	44	2346
0.027	52	1857	0.019	31	1577
0.095	56	531	0.116	64	486
0.031	70	2225	0.029	51	1723
0.027	58	2114	0.029	51	1731
0.048	141	2810	0.042	105	2378
0.036	43	1137	0.05	49	939
0.025	53	2061	0.024	42	1741
0.021	57	2611	0.029	63	2146
0.03	41	1307	0.018	20	1107
0.067	87	1215	0.074	90	1133
0.023	28	1184	0.022	21	924

0.03	31	997	0.021	19	874
0.025	69	2702	0.024	58	2334
0.074	135	1686	0.052	80	1455
0.058	116	1870	0.052	86	1578
0.025	41	1622	0.026	38	1419
0.065	92	1326	0.059	71	1136
0.031	69	2168	0.025	47	1861
0.026	77	2877	0.024	60	2426
0.05	61	1162	0.056	57	966
0.023	15	628	0.032	16	486
0.06	86	1357	0.072	96	1237
0.028	30	1036	0.026	24	892
0.024	37	1507	0.024	34	1381
0.072	83	1069	0.071	67	880
0.025	25	964	0.029	21	715
0.029	76	2590	0.024	54	2207
0.028	88	3053	0.033	87	2564
0.062	87	1315	0.083	100	1112
0.026	42	1576	0.038	55	1378
0.028	59	2057	0.032	56	1708
0.031	40	1244	0.028	29	999
0.083	82	909	0.113	114	895
0.031	165	5134	0.031	143	4498
0.036	41	1105	0.029	27	911
0.023	73	3036	0.023	63	2636
0.021	79	3701	0.03	99	3170
0.023	55	2373	0.023	50	2093
0.016	29	1769	0.023	36	1525
0.025	75	2933	0.021	54	2573
0.021	22	1017	0.026	24	910
0.022	62	2788	0.022	45	2045
0.052	105	1916	0.041	74	1750
0.025	42	1667	0.032	44	1337
0.027	126	4586	0.028	113	3866
0.028	100	3476	0.03	92	3010
0.032	29	869	0.024	18	733
0.029	39	1312	0.036	42	1122
0.023	19	791	0.03	23	744
0.058	116	1870	0.06	102	1592
0.016	24	1504	0.025	33	1290

0.026	55	2021	0.026	50	1892
0.012	12	986	0.016	13	791
0.052	93	1697	0.076	115	1401
0.02	31	1520	0.029	37	1250
0.027	67	2420	0.032	64	1929
0.049	61	1174	0.065	62	892
0.042	48	1089	0.042	38	860
0.023	39	1642	0.028	40	1388
0.027	111	4071	0.025	89	3496
0.018	21	1131	0.034	36	1036
0.023	74	3103	0.025	68	2619
0.048	73	1457	0.058	76	1230
0.019	33	1721	0.019	26	1372
0.026	33	1259	0.028	32	1107
0.02	27	1323	0.015	17	1099
0.024	17	691	0.024	13	523
0.027	78	2760	0.023	55	2383
0.078	170	2005	0.085	153	1652
0.022	59	2605	0.027	62	2242
0.446	731	909	0.556	895	716
0.321	242	513	0.463	328	380
0.02	14	684	0.025	15	583
0.032	52	1550	0.031	48	1503
0.056	90	1506	0.046	61	1260
0.056	49	830	0.052	43	778
0.03	53	1690	0.028	39	1365
0.024	69	2851	0.014	32	2303
0.025	134	5192	0.025	122	4703
0.09	91	916	0.116	103	787
0.06	47	735	0.066	44	618
0.125	184	1289	0.16	198	1041
0.085	65	702	0.095	58	555
0.024	48	1987	0.03	46	1513
0.021	42	1999	0.03	53	1692
0.021	18	859	0.03	22	719
0.026	70	2604	0.026	59	2189
0.023	46	1961	0.022	40	1812
0.046	96	2002	0.06	108	1688
0.024	27	1081	0.038	38	974
0.015	53	3376	0.019	57	2989

0.023	25	1070	0.02	17	851
0.032	62	1872	0.04	61	1478
0.023	66	2799	0.023	54	2339
0.027	56	2054	0.016	29	1785
0.029	83	2773	0.027	66	2375
0.024	49	2021	0.031	47	1492
0.025	66	2601	0.027	58	2128
0.08	164	1886	0.102	187	1648
0.051	52	971	0.055	50	856
0.028	58	2005	0.029	54	1816
0.024	41	1676	0.023	32	1362
0.023	44	1861	0.024	41	1684
0.025	95	3753	0.039	118	2917
0.022	34	1536	0.028	38	1312
0.024	51	2116	0.033	64	1847
0.019	51	2680	0.026	64	2373
0.048	119	2339	0.047	96	1935
0.062	59	890	0.074	63	787
0.026	37	1363	0.026	31	1174
0.055	126	2153	0.063	123	1829
0.048	27	541	0.026	12	449
0.025	17	664	0.018	10	549
0.068	161	2211	0.075	148	1825
0.05	86	1641	0.06	93	1469
0.03	59	1929	0.034	58	1672
0.022	40	1792	0.021	30	1429
0.014	12	818	0.014	9	614
0.032	53	1583	0.032	42	1288
0.13	98	657	0.174	111	526
0.061	55	844	0.059	47	751
0.081	69	778	0.122	80	574
0.024	84	3400	0.025	77	2981
0.019	49	2574	0.023	50	2101
0.055	45	770	0.079	57	662
0.051	135	2536	0.044	101	2176
0.05	145	2739	0.059	133	2130
0.035	30	837	0.032	22	666
0.022	39	1761	0.034	53	1511
0.06	113	1776	0.063	94	1402
0.021	93	4381	0.024	93	3708

0.062	200	3050	0.045	127	2671
0.034	98	2806	0.025	64	2479
0.022	49	2211	0.029	58	1935
0.05	46	879	0.066	55	783
0.042	91	2071	0.059	116	1841
0.052	109	1980	0.065	111	1591
0.068	65	890	0.086	66	698
0.028	66	2305	0.026	54	2046
0.021	132	6137	0.022	121	5355
0.03	46	1478	0.021	27	1229
0.022	63	2862	0.018	45	2504
0.101	114	1019	0.108	100	825
0.017	17	974	0.028	23	799
0.358	551	990	0.484	836	890
0.023	53	2236	0.023	42	1772
0.022	65	2857	0.024	62	2526
0.021	59	2752	0.023	53	2221
0.057	83	1380	0.064	78	1135
0.033	49	1432	0.031	42	1333
0.024	35	1410	0.025	31	1223
0.057	102	1698	0.069	97	1312
0.02	62	3059	0.025	60	2376
0.028	76	2605	0.033	76	2208
0.074	75	934	0.064	56	815
0.064	72	1051	0.049	45	868
0.024	62	2544	0.015	35	2225
0.046	38	796	0.044	32	689
0.024	93	3818	0.032	101	3097
0.021	55	2519	0.028	61	2089
0.025	46	1793	0.029	49	1636
0.023	50	2116	0.027	53	1892
0.027	49	1763	0.025	41	1591
0.038	27	685	0.024	13	531
0.013	12	918	0.028	20	695
0.066	88	1252	0.051	56	1051
0.021	19	904	0.018	14	753
0.054	80	1404	0.066	75	1057
0.025	35	1387	0.013	16	1178
0.028	43	1475	0.023	28	1179
0.03	40	1315	0.028	28	983

0.019	60	3100	0.015	41	2683
0.021	90	4259	0.024	94	3746
0.024	37	1528	0.021	29	1380
0.049	78	1514	0.052	69	1253
0.034	129	3695	0.033	118	3480
0.027	30	1082	0.028	25	867
0.026	56	2110	0.03	50	1642
0.033	110	3201	0.031	90	2827
0.022	28	1228	0.023	25	1056
0.015	14	909	0.033	26	765
0.027	41	1479	0.02	27	1307
0.033	34	1007	0.035	29	790
0.022	17	759	0.029	20	673
0.027	58	2129	0.023	43	1834
0.064	122	1771	0.063	102	1530
0.103	122	1059	0.086	81	862
0.026	55	2022	0.028	49	1692
0.021	146	6670	0.022	132	5904
0.024	41	1668	0.023	35	1476
0.027	44	1583	0.02	27	1296
0.023	89	3754	0.021	76	3465
0.031	44	1354	0.035	40	1111
0.028	42	1468	0.034	45	1283
0.065	32	459	0.044	17	368
0.024	138	5527	0.029	145	4898
0.028	56	1946	0.02	36	1753
0.019	29	1510	0.024	30	1223
0.028	28	989	0.043	41	916
0.019	34	1764	0.022	33	1492
0.023	40	1685	0.022	32	1414
0.036	70	1885	0.031	54	1672
0.038	33	845	0.041	29	670
0.024	47	1947	0.031	54	1716
0.026	96	3587	0.024	79	3217
0.019	26	1344	0.021	26	1190
0.033	179	5297	0.035	163	4531
0.024	58	2375	0.02	39	1933
0.025	74	2938	0.026	70	2621
0.025	44	1736	0.028	39	1362
0.019	88	4519	0.025	93	3676

0.025	99	3887	0.024	80	3252
0.025	67	2612	0.024	53	2125
0.026	40	1520	0.021	26	1196
0.027	58	2058	0.036	62	1683
0.033	53	1556	0.017	22	1297
0.025	120	4667	0.024	100	4021
0.027	27	974	0.034	26	746
0.345	364	691	0.493	566	582
0.024	40	1646	0.024	35	1402
0.018	29	1576	0.033	44	1309
0.027	43	1521	0.026	35	1311
0.027	77	2745	0.023	54	2304
0.051	219	4068	0.057	210	3470
0.016	51	3146	0.024	62	2552
0.125	141	983	0.173	153	732
0.048	95	1905	0.053	84	1507
0.063	109	1620	0.044	65	1423
0.041	51	1201	0.018	19	1011
0.03	38	1230	0.022	22	956
0.023	24	1001	0.023	21	883
0.06	126	1957	0.066	113	1590
0.022	27	1195	0.025	25	968
0.024	144	5789	0.022	115	5152
0.028	81	2788	0.027	74	2649
0.025	39	1495	0.021	26	1221
0.027	71	2593	0.036	84	2222
0.018	49	2604	0.03	67	2198
0.02	40	1996	0.022	42	1868
0.059	83	1330	0.061	74	1134
0.017	16	902	0.028	22	756
0.021	78	3704	0.028	94	3249
0.121	133	967	0.135	126	810
0.027	37	1324	0.018	21	1172
0.059	63	1010	0.049	44	861
0.026	73	2732	0.029	66	2173
0.02	11	528	0.034	13	372
0.026	63	2338	0.023	48	2054
0.02	54	2708	0.025	57	2268
0.021	82	3767	0.023	77	3246
0.023	31	1327	0.021	23	1067

0.028	70	2391	0.028	63	2214
0.034	88	2519	0.033	77	2268
0.03	83	2700	0.033	78	2295
0.032	31	924	0.039	34	847
0.032	64	1944	0.034	61	1753
0.023	50	2109	0.027	44	1608
0.02	62	3109	0.026	72	2722
0.024	68	2714	0.023	52	2206
0.023	29	1213	0.026	26	986
0.025	60	2361	0.021	40	1903
0.046	98	2049	0.042	74	1700
0.024	61	2446	0.029	63	2095
0.027	89	3153	0.02	54	2638
0.041	55	1274	0.045	51	1089
0.025	38	1501	0.022	30	1321
0.066	77	1085	0.071	69	898
0.028	65	2274	0.03	58	1847
0.025	65	2543	0.03	64	2095
0.036	51	1364	0.028	33	1160
0.023	77	3217	0.026	70	2602
0.059	105	1676	0.06	93	1450
0.022	101	4465	0.028	107	3651
0.038	93	2369	0.02	40	1915
0.086	85	898	0.094	75	720
0.019	34	1731	0.029	43	1437
0.036	47	1253	0.041	47	1104
0.027	32	1164	0.021	24	1144
0.02	30	1491	0.024	29	1172
0.021	30	1368	0.023	25	1076
0.018	35	1963	0.026	44	1667
0.028	50	1767	0.02	29	1406
0.026	33	1243	0.026	29	1095
0.28	161	415	0.39	180	282
0.385	260	415	0.57	503	380
0.024	48	1942	0.026	40	1528
0.015	22	1444	0.032	35	1044
0.027	69	2469	0.02	44	2184
0.026	16	596	0.024	11	445
0.027	54	1971	0.029	49	1637
0.02	58	2883	0.021	55	2513

0.024	47	1932	0.019	31	1599
0.027	36	1307	0.027	28	1013
0.077	109	1314	0.108	120	993
0.02	129	6256	0.021	119	5522
0.03	58	1860	0.022	36	1572
0.797	846	215	0.862	1091	174
0.019	20	1049	0.028	27	935
0.024	48	1958	0.016	27	1655
0.063	106	1585	0.051	77	1427
0.028	62	2171	0.027	46	1666
0.024	43	1738	0.028	40	1410
0.692	656	292	0.814	908	207
0.017	27	1534	0.016	21	1290
0.054	43	746	0.073	50	635
0.022	60	2729	0.024	61	2447
0.025	53	2102	0.027	48	1740
0.022	89	3994	0.018	68	3612
0.027	45	1631	0.02	32	1547
0.028	52	1822	0.029	46	1542
0.05	124	2361	0.054	113	1988
0.024	102	4234	0.027	93	3361
0.012	32	2529	0.025	56	2229
0.058	95	1555	0.055	81	1405
0.032	55	1662	0.039	55	1350
0.023	48	2085	0.036	68	1809
0.027	113	4000	0.028	97	3361
0.017	20	1149	0.024	25	1035
0.017	44	2615	0.017	36	2117
0.034	55	1572	0.046	67	1380
0.023	61	2649	0.027	58	2118
0.033	80	2321	0.022	46	2005
0.024	49	2017	0.023	41	1766
0.02	47	2253	0.025	51	2017
0.023	53	2297	0.022	44	1928
0.033	43	1258	0.028	38	1298
0.023	110	4677	0.025	106	4054
0.022	137	5953	0.02	109	5221
0.066	83	1170	0.048	50	993
0.018	34	1877	0.024	42	1695
0.027	40	1426	0.025	30	1186

0.022	45	2036	0.028	49	1691
0.029	71	2352	0.021	47	2197
0.15	74	420	0.231	98	326
0.025	186	7155	0.027	164	5924
0.03	44	1423	0.029	36	1188
0.021	23	1057	0.031	28	877
0.017	30	1720	0.022	32	1438
0.027	48	1708	0.024	37	1500
0.027	37	1342	0.02	23	1107
0.051	72	1348	0.065	77	1116
0.027	123	4390	0.024	95	3903
0.055	128	2209	0.05	100	1891
0.059	163	2621	0.048	116	2316
0.024	23	948	0.017	16	910
0.019	24	1226	0.028	28	964
0.07	71	943	0.072	60	768
0.021	59	2701	0.024	55	2261
0.028	35	1215	0.028	25	870
0.023	15	639	0.015	8	536
0.025	31	1187	0.024	24	970
0.028	93	3183	0.023	59	2542
0.026	23	845	0.043	32	705
0.06	119	1865	0.057	93	1543
0.016	19	1135	0.028	29	1012
0.03	54	1756	0.022	33	1447
0.025	152	5893	0.024	130	5202
0.084	219	2376	0.107	239	1987
0.036	64	1727	0.019	30	1579
0.018	37	2000	0.016	27	1626
0.023	86	3695	0.03	96	3142
0.065	118	1704	0.049	76	1479
0.03	53	1739	0.025	38	1473
0.019	27	1409	0.042	56	1262
0.069	51	685	0.071	44	580
0.025	33	1272	0.036	46	1220
0.022	61	2772	0.025	55	2160
0.02	99	4748	0.023	94	4029
0.026	81	2982	0.029	82	2724
0.023	57	2428	0.026	48	1810
0.021	29	1346	0.031	36	1113

0.032	113	3449	0.029	93	3103
0.028	66	2251	0.03	58	1883
0.028	50	1761	0.019	29	1532
0.02	72	3533	0.022	65	2925
0.06	161	2514	0.068	151	2059
0.026	39	1470	0.018	22	1224
0.016	7	421	0.019	8	403
0.053	78	1390	0.068	84	1160
0.034	57	1621	0.037	55	1423
0.061	75	1149	0.046	48	986
0.026	81	3046	0.019	51	2666
0.067	45	624	0.085	40	431
0.025	40	1579	0.033	47	1399
0.022	65	2934	0.023	58	2451
0.052	158	2905	0.064	147	2140
0.056	86	1453	0.075	98	1217
0.067	108	1516	0.034	49	1396
0.037	71	1852	0.038	62	1572
0.022	42	1870	0.023	38	1600
0.031	57	1791	0.032	52	1573
0.017	21	1223	0.029	31	1056
0.121	115	835	0.162	121	628
0.027	98	3579	0.023	75	3181
0.026	76	2838	0.027	63	2257
0.033	51	1487	0.02	27	1300
0.019	55	2838	0.025	65	2493
0.02	31	1535	0.025	30	1164
0.031	37	1142	0.014	12	850
0.055	63	1090	0.065	62	890
0.032	83	2545	0.023	54	2299
0.018	34	1866	0.023	41	1735
0.026	47	1735	0.014	20	1444
0.029	39	1324	0.023	26	1120
0.031	87	2687	0.024	57	2286
0.031	92	2918	0.024	61	2504
0.063	78	1163	0.059	63	1011
0.027	38	1370	0.039	44	1097
0.021	70	3208	0.023	68	2912
0.129	202	1360	0.131	171	1139
0.026	49	1849	0.02	33	1600

0.03	49	1566	0.029	40	1322
0.019	41	2079	0.025	47	1847
0.024	106	4354	0.024	88	3579
0.022	87	3788	0.026	86	3235
0.054	66	1163	0.042	43	969
0.021	64	2964	0.024	56	2270
0.032	121	3677	0.02	62	3107
0.032	62	1885	0.032	46	1398
0.022	58	2612	0.029	62	2051
0.041	92	2175	0.059	111	1784
0.033	80	2352	0.029	58	1923
0.021	56	2643	0.026	58	2132
0.018	25	1402	0.021	26	1188
0.112	136	1083	0.122	133	959
0.025	36	1406	0.044	55	1205
0.021	24	1094	0.025	24	926
0.05	64	1217	0.046	52	1069
0.017	72	4250	0.026	97	3676
0.024	80	3261	0.028	78	2745
0.02	38	1826	0.018	26	1447
0.015	20	1346	0.02	25	1195
0.024	76	3137	0.02	55	2666
0.024	83	3387	0.032	93	2820
0.024	39	1599	0.021	29	1357
0.088	74	766	0.11	75	604
0.025	34	1304	0.017	21	1204
0.023	79	3381	0.021	67	3064
0.019	33	1701	0.016	24	1513
0.029	79	2658	0.03	66	2144
0.023	58	2486	0.022	47	2060
0.019	84	4284	0.021	80	3820
0.014	22	1601	0.022	29	1303
0.018	47	2508	0.029	62	2046
0.024	65	2674	0.022	55	2449
0.042	39	879	0.044	35	763
0.02	33	1599	0.03	39	1250
0.019	40	2082	0.023	40	1705
0.019	35	1852	0.023	38	1585
0.025	37	1419	0.028	38	1313
0.014	34	2324	0.029	58	1958

0.024	75	3031	0.012	33	2633
0.029	64	2172	0.034	56	1584
0.028	69	2382	0.031	68	2111
0.021	47	2177	0.025	50	1940
0.047	141	2852	0.06	143	2247
0.024	44	1794	0.025	39	1530
0.051	76	1409	0.05	60	1136
0.021	72	3333	0.032	95	2909
0.031	42	1327	0.036	41	1097
0.026	84	3137	0.024	65	2641
0.027	81	2896	0.02	53	2626
0.016	11	690	0.031	20	632
0.071	146	1914	0.076	128	1557
0.075	169	2078	0.061	109	1683
0.014	7	478	0.015	6	394
0.031	76	2377	0.025	55	2169
0.025	56	2208	0.03	62	2027
0.022	43	1924	0.027	46	1688
0.025	45	1730	0.031	44	1366
0.026	38	1428	0.032	37	1125
0.024	99	4025	0.019	68	3524
0.053	124	2202	0.07	133	1780
0.025	61	2356	0.03	60	1925
0.021	46	2103	0.018	33	1752
0.019	61	3106	0.019	49	2573
0.027	44	1565	0.026	30	1145
0.025	70	2693	0.02	42	2053
0.029	60	2006	0.03	52	1667
0.047	94	1900	0.048	80	1601
0.049	113	2175	0.058	108	1758
0.027	57	2046	0.028	50	1764
0.026	38	1399	0.02	23	1135
0.053	104	1869	0.04	70	1678
0.021	44	2046	0.025	41	1607
0.035	41	1141	0.024	23	924
0.032	53	1619	0.033	51	1475
0.029	63	2107	0.029	57	1917
0.017	37	2101	0.02	36	1773
0.079	114	1331	0.09	110	1113
0.027	95	3472	0.021	68	3135

0.018	41	2178	0.021	40	1869
0.032	58	1779	0.026	40	1479
0.04	26	620	0.027	14	498
0.022	101	4591	0.02	78	3783
0.082	89	997	0.071	63	823
0.029	72	2410	0.027	55	1950
0.025	59	2263	0.025	51	1973
0.022	69	3129	0.025	64	2496
0.022	40	1757	0.026	42	1584
0.09	90	909	0.083	68	750
0.029	71	2388	0.023	49	2074
0.027	48	1735	0.023	38	1586
0.027	74	2663	0.028	61	2152
0.026	118	4391	0.025	93	3650
0.031	69	2178	0.021	41	1936
0.051	53	988	0.051	48	899
0.019	82	4125	0.027	102	3666
0.022	69	3052	0.024	65	2618
0.024	54	2200	0.024	45	1813
0.023	71	3068	0.032	79	2398
0.061	80	1237	0.045	53	1132
0.024	54	2186	0.025	52	2016
0.029	41	1385	0.027	33	1195
0.021	50	2300	0.023	45	1920
0.021	41	1922	0.034	59	1653
0.018	22	1223	0.019	21	1102
0.024	52	2145	0.039	74	1813
0.02	40	1944	0.026	46	1744
0.022	29	1283	0.018	20	1122
0.02	34	1657	0.023	32	1387
0.022	46	2026	0.012	20	1716
0.022	23	1005	0.021	19	889
0.026	125	4685	0.023	92	3881
0.019	83	4313	0.028	106	3632
0.022	43	1928	0.025	40	1537
0.029	23	763	0.018	12	668
0.053	122	2180	0.055	107	1832
0.032	70	2092	0.031	58	1791
0.021	37	1698	0.03	43	1388
0.016	38	2303	0.024	47	1893

0.032	55	1669	0.017	27	1553
0.062	136	2046	0.045	89	1868
0.077	100	1201	0.079	88	1022
0.067	84	1175	0.066	71	1004
0.022	99	4472	0.027	105	3829
0.027	42	1516	0.028	42	1439
0.02	18	861	0.021	15	716
0.022	23	1006	0.029	26	859
0.033	39	1130	0.031	30	935
0.026	62	2293	0.025	48	1908
0.362	1163	2054	0.436	1435	1856
0.08	66	758	0.107	76	631
0.021	109	5020	0.022	100	4416
0.069	94	1259	0.061	73	1120
0.024	61	2447	0.024	52	2088
0.027	79	2881	0.022	56	2470
0.033	59	1726	0.032	49	1475
0.031	29	906	0.031	24	751
0.042	48	1100	0.058	57	928
0.023	35	1459	0.025	31	1217
0.075	117	1453	0.068	93	1270
0.026	32	1198	0.015	14	925
0.027	144	5117	0.03	134	4369
0.061	85	1312	0.042	54	1219
0.03	65	2137	0.019	36	1872
0.024	38	1577	0.021	26	1206
0.026	64	2393	0.021	43	1982
0.047	94	1899	0.045	77	1639
0.029	58	1933	0.029	45	1520
0.027	25	893	0.025	19	730
0.018	78	4282	0.023	87	3706
0.022	66	2956	0.022	53	2341
0.029	94	3178	0.028	77	2634
0.032	43	1317	0.028	34	1160
0.026	26	979	0.026	21	796
0.023	56	2343	0.028	55	1922
0.02	42	2104	0.029	52	1718
0.03	45	1459	0.036	39	1056
0.029	92	3064	0.026	68	2562
0.034	22	630	0.039	23	568

0.015	27	1793	0.033	50	1479
0.078	43	510	0.051	22	410
0.027	48	1706	0.026	37	1362
0.034	54	1536	0.023	31	1318
0.07	53	700	0.101	60	537
0.021	111	5156	0.025	111	4397
0.023	65	2755	0.025	61	2348
0.025	120	4635	0.035	142	3918
0.025	75	2931	0.017	42	2393
0.07	97	1280	0.103	112	972
0.055	99	1696	0.074	108	1342
0.04	40	952	0.027	23	835
0.031	37	1150	0.033	29	854
0.024	46	1832	0.024	37	1493
0.027	43	1525	0.023	31	1315
0.03	118	3764	0.025	78	3050
0.071	75	977	0.061	52	800
0.027	54	1927	0.031	48	1495
0.028	22	768	0.032	23	688
0.044	46	1006	0.045	45	954
0.016	19	1182	0.039	37	917
0.021	57	2720	0.028	68	2341
0.027	45	1623	0.028	41	1398
0.065	189	2719	0.068	159	2189
0.026	42	1548	0.029	37	1219
0.103	211	1847	0.103	169	1473
0.024	51	2117	0.023	44	1837
0.019	20	1059	0.018	15	839
0.023	29	1259	0.026	29	1080
0.027	43	1540	0.02	28	1343
0.026	77	2854	0.028	67	2347
0.058	175	2843	0.06	154	2398
0.029	62	2050	0.022	37	1655
0.025	65	2569	0.029	63	2116
0.027	64	2289	0.031	60	1894
0.031	78	2409	0.024	52	2130
0.066	183	2610	0.039	92	2248
0.028	30	1023	0.025	23	906
0.024	47	1919	0.016	26	1565
0.025	16	622	0.037	19	497

0.068	52	718	0.07	44	589
0.057	97	1604	0.063	87	1299
0.017	89	5025	0.027	120	4336
0.026	64	2432	0.03	67	2165
0.055	63	1077	0.068	55	750
0.046	61	1254	0.054	59	1030
0.035	59	1606	0.026	37	1365
0.03	50	1603	0.03	41	1342
0.023	18	762	0.031	20	633
0.041	100	2352	0.074	149	1862
0.033	145	4304	0.038	149	3785
0.026	61	2286	0.026	52	1974
0.095	44	420	0.118	54	404
0.118	54	403	0.097	38	355
0.019	26	1322	0.021	24	1106
0.016	30	1862	0.028	47	1606
0.062	123	1853	0.05	84	1591
0.024	163	6660	0.026	154	5681
0.025	45	1739	0.027	41	1495
0.03	219	7033	0.031	187	5798
0.064	179	2617	0.093	217	2110
0.023	136	5876	0.024	125	5146
0.022	102	4519	0.024	93	3743
0.064	72	1055	0.056	50	837
0.032	87	2601	0.024	52	2109
0.073	125	1593	0.057	90	1484
0.025	65	2486	0.021	46	2131
0.019	77	3971	0.026	92	3387
0.035	33	897	0.037	33	852
0.027	108	3947	0.019	73	3716
0.063	74	1102	0.052	56	1026
0.027	59	2089	0.029	55	1818
0.68	1209	568	0.79	1655	439
0.026	58	2200	0.021	39	1822
0.029	105	3462	0.025	71	2798
0.031	72	2250	0.025	52	2060
0.024	93	3705	0.027	84	3069
0.025	51	1975	0.023	40	1710
0.025	34	1302	0.023	26	1118
0.086	100	1061	0.116	116	888

0.037	63	1643	0.03	42	1378
0.02	86	4292	0.026	95	3627
0.069	64	858	0.061	40	619
0.024	78	3107	0.027	66	2416
0.02	25	1207	0.021	21	958
0.022	143	6414	0.025	137	5382
0.063	111	1662	0.065	92	1321
0.024	75	3087	0.022	57	2570
0.061	93	1440	0.054	66	1146
0.061	58	888	0.067	58	809
0.021	93	4410	0.032	119	3618
0.02	32	1607	0.027	39	1430
0.021	148	6991	0.027	158	5725
0.023	48	1997	0.035	69	1904
0.03	115	3694	0.026	92	3427
0.06	219	3432	0.052	157	2872
0.027	65	2377	0.025	56	2210
0.032	68	2068	0.025	42	1668
0.037	77	2004	0.026	52	1919
0.049	120	2318	0.054	113	1965
0.064	119	1744	0.059	96	1530
0.023	50	2083	0.027	46	1684
0.023	48	2072	0.025	43	1673
0.022	42	1831	0.025	40	1556
0.025	68	2645	0.031	66	2048
0.023	120	5019	0.022	97	4368
0.056	78	1324	0.089	102	1046
0.016	86	5421	0.025	118	4657
0.027	64	2347	0.033	70	2064
0.019	31	1573	0.03	42	1347
0.027	27	974	0.017	15	847
0.024	138	5713	0.023	117	4924
0.023	66	2774	0.026	62	2313
0.024	76	3107	0.028	71	2487
0.019	34	1747	0.022	31	1356
0.02	14	687	0.022	13	582
0.018	50	2683	0.028	67	2284
0.021	36	1664	0.016	24	1483
0.037	30	772	0.018	11	597
0.024	31	1287	0.021	24	1098

0.026	86	3213	0.023	64	2734
0.064	54	792	0.059	43	683
0.023	48	2020	0.019	33	1732
0.025	24	950	0.025	22	874
0.019	28	1470	0.027	38	1370
0.02	29	1387	0.021	25	1191
0.022	79	3449	0.027	85	3031
0.027	103	3679	0.028	97	3343
0.052	91	1666	0.066	92	1298
0.027	119	4268	0.034	129	3626
0.032	37	1117	0.039	42	1047
0.021	83	3807	0.026	85	3231
0.03	62	2006	0.026	46	1729
0.021	75	3544	0.027	84	3057
0.026	45	1710	0.024	36	1460
0.016	43	2570	0.028	61	2098
0.022	28	1252	0.02	22	1070
0.036	50	1346	0.034	43	1227
0.029	53	1756	0.024	37	1513
0.025	28	1089	0.027	24	881
0.023	27	1156	0.026	26	987
0.076	60	730	0.07	48	640
0.026	73	2711	0.029	70	2361
0.051	64	1203	0.064	69	1001
0.067	33	459	0.037	13	342
0.024	72	2936	0.029	74	2503
0.061	167	2574	0.057	133	2210
0.027	31	1126	0.03	28	918
0.024	54	2178	0.026	47	1734
0.021	49	2312	0.014	31	2173
0.024	53	2117	0.032	58	1755
0.021	52	2385	0.025	54	2087
0.028	70	2392	0.025	53	2091
0.034	92	2588	0.035	73	2021
0.02	57	2860	0.018	47	2610
0.029	34	1158	0.022	23	1003
0.027	56	2050	0.022	39	1740
0.023	47	1979	0.021	35	1661
0.023	39	1694	0.03	38	1214
0.02	35	1687	0.028	40	1376

0.027	66	2345	0.033	68	1986
0.023	57	2425	0.025	53	2108
0.023	43	1868	0.02	35	1717
0.025	102	3974	0.022	75	3302
0.098	176	1628	0.088	129	1339
0.024	86	3480	0.02	62	3116
0.023	33	1426	0.02	23	1120
0.03	75	2394	0.029	56	1845
0.019	25	1281	0.022	24	1057
0.023	69	2905	0.02	49	2385
0.068	58	793	0.082	61	683
0.023	89	3753	0.028	89	3078
0.031	44	1357	0.023	29	1247
0.022	76	3302	0.025	69	2641
0.022	64	2902	0.029	67	2208
0.025	57	2253	0.025	50	1985
0.061	85	1312	0.04	48	1139
0.018	49	2743	0.02	46	2299
0.131	134	887	0.157	143	770
0.019	70	3558	0.02	60	2979
0.015	44	2846	0.024	63	2592
0.076	120	1454	0.087	110	1159
0.061	212	3242	0.072	204	2620
0.02	117	5767	0.026	128	4741
0.161	223	1165	0.143	166	994
0.028	36	1241	0.029	29	986
0.055	118	2034	0.045	78	1668
0.023	28	1177	0.02	24	1155
0.026	81	3024	0.023	58	2512
0.019	47	2421	0.024	50	2059
0.024	92	3757	0.019	58	3022
0.028	95	3261	0.032	86	2624
0.058	76	1242	0.049	54	1052
0.019	40	2068	0.02	33	1650
0.021	18	831	0.026	18	665
0.041	59	1365	0.036	45	1206
0.023	73	3105	0.021	59	2707
0.026	44	1663	0.023	31	1299
0.112	89	704	0.142	104	628
0.013	35	2575	0.028	60	2068

0.016	18	1099	0.025	25	968
0.051	81	1513	0.062	86	1294
0.06	50	782	0.059	44	704
0.058	137	2220	0.062	124	1877
0.017	30	1780	0.026	44	1669
0.019	65	3406	0.025	72	2863
0.03	16	517	0.017	7	405
0.032	41	1250	0.033	36	1045
0.023	64	2691	0.023	58	2503
0.034	75	2128	0.036	73	1969
0.044	62	1350	0.058	73	1188
0.135	82	526	0.237	123	396
0.021	44	2087	0.023	41	1773
0.051	51	942	0.033	29	843
0.025	71	2775	0.026	64	2369
0.026	45	1708	0.025	38	1498
0.057	177	2944	0.052	138	2513
0.023	72	3124	0.031	84	2666
0.028	27	934	0.021	19	898
0.046	59	1234	0.028	34	1186
0.028	77	2659	0.029	68	2253
0.027	51	1812	0.023	38	1627
0.031	76	2337	0.022	46	2022
0.035	33	898	0.035	28	768
0.067	132	1840	0.049	84	1635
0.055	136	2340	0.044	86	1864
0.021	65	3026	0.031	78	2450
0.088	179	1859	0.135	224	1435
0.189	314	1347	0.254	390	1148
0.034	49	1378	0.043	51	1141
0.062	161	2434	0.062	140	2106
0.024	49	1954	0.028	48	1679
0.024	106	4392	0.022	85	3701
0.018	21	1123	0.028	29	1001
0.021	48	2187	0.024	45	1825
0.02	66	3200	0.025	65	2544
0.025	73	2822	0.026	63	2397
0.021	115	5269	0.025	118	4626
0.023	68	2882	0.023	54	2296
0.027	32	1147	0.03	28	920

0.02	45	2164	0.026	42	1584
0.025	75	2944	0.027	65	2385
0.025	64	2500	0.028	61	2117
0.032	49	1470	0.024	32	1293
0.016	13	824	0.021	16	736
0.016	35	2134	0.028	51	1800
0.025	123	4853	0.025	107	4215
0.023	21	879	0.024	16	664
0.03	42	1373	0.036	47	1253
0.057	153	2515	0.048	106	2110
0.022	56	2534	0.026	56	2098
0.02	77	3753	0.029	93	3116
0.033	60	1772	0.031	49	1532
0.021	128	5973	0.018	93	5155
0.017	43	2418	0.024	52	2118
0.025	29	1151	0.021	21	969
0.023	53	2241	0.028	51	1768
0.026	95	3581	0.019	55	2892
0.024	71	2902	0.032	78	2349
0.059	57	907	0.045	34	729
0.027	132	4804	0.026	105	3971
0.018	22	1232	0.018	19	1049
0.02	28	1392	0.029	36	1220
0.021	79	3756	0.031	110	3421
0.016	28	1772	0.034	57	1614
0.022	69	3086	0.02	55	2680
0.019	78	4022	0.021	75	3454
0.046	50	1035	0.053	52	938
0.02	29	1440	0.03	39	1251
0.029	55	1842	0.019	30	1511
0.085	104	1113	0.087	82	863
0.02	27	1321	0.035	42	1143
0.025	105	4046	0.032	111	3333
0.026	103	3817	0.023	79	3312
0.025	27	1073	0.016	15	896
0.054	73	1278	0.047	57	1146
0.014	33	2283	0.024	51	2118
0.03	35	1125	0.039	39	956
0.062	52	787	0.097	69	639
0.029	95	3205	0.026	78	2967

0.05	46	867	0.06	50	777
0.021	41	1955	0.015	23	1544
0.021	29	1383	0.027	33	1189
0.607	521	338	0.7	613	263
0.022	82	3697	0.029	94	3126
0.02	59	2844	0.024	58	2315
0.064	231	3383	0.075	215	2656
0.023	52	2176	0.02	39	1897
0.056	41	691	0.047	29	583
0.031	50	1543	0.03	37	1183
0.029	37	1225	0.03	33	1054
0.023	144	6135	0.026	146	5480
0.026	40	1491	0.038	48	1224
0.021	54	2549	0.021	48	2277
0.02	33	1650	0.028	37	1272
0.018	71	3860	0.027	88	3155
0.027	88	3174	0.027	80	2869
0.016	46	2807	0.028	69	2367
0.023	34	1418	0.028	33	1139
0.072	90	1166	0.07	65	864
0.023	23	981	0.036	28	741
0.024	80	3229	0.024	69	2773
0.024	54	2189	0.018	34	1848
0.042	25	577	0.075	37	455
0.022	57	2544	0.028	64	2182
0.053	94	1678	0.05	78	1497
0.062	196	2941	0.048	123	2424
0.025	46	1790	0.032	48	1471
0.027	78	2809	0.028	74	2557
0.021	84	3889	0.022	73	3208
0.023	45	1883	0.019	33	1679
0.02	29	1420	0.026	34	1299
0.025	39	1500	0.028	34	1165
0.027	46	1636	0.021	32	1465
0.03	91	2981	0.03	81	2612
0.069	56	753	0.031	21	650
0.032	35	1067	0.031	31	985
0.05	131	2514	0.049	105	2031
0.02	55	2633	0.033	75	2191
0.099	77	703	0.136	84	535

0.023	37	1557	0.021	29	1322
0.028	25	853	0.022	16	709
0.025	30	1173	0.026	29	1068
0.083	60	660	0.091	59	589
0.03	67	2183	0.033	64	1900
0.025	73	2891	0.034	90	2584
0.027	62	2220	0.022	43	1885
0.029	72	2452	0.026	54	2032
0.022	43	1896	0.024	37	1520
0.09	127	1288	0.125	144	1006
0.026	70	2592	0.022	48	2120
0.021	78	3676	0.027	89	3189
0.079	67	784	0.081	57	650
0.03	54	1772	0.034	51	1460
0.057	139	2314	0.057	114	1893
0.025	62	2427	0.021	40	1902
0.018	35	1864	0.031	49	1544
0.053	58	1042	0.068	67	916
0.022	54	2363	0.02	42	2012
0.051	45	845	0.058	44	721
0.028	54	1888	0.025	37	1468
0.021	47	2175	0.016	33	2052
0.03	69	2237	0.026	58	2176
0.022	41	1839	0.023	31	1346
0.023	24	1034	0.028	25	884
0.026	17	638	0.018	11	599
0.048	145	2869	0.062	153	2334
0.063	95	1423	0.073	86	1085
0.062	185	2789	0.061	146	2252
0.017	33	1945	0.032	54	1609
0.023	19	801	0.031	20	624
0.018	70	3800	0.019	63	3267
0.02	16	766	0.019	12	615
0.04	81	1965	0.047	84	1688
0.028	87	3050	0.025	68	2678
0.027	109	3910	0.028	92	3181
0.023	36	1526	0.02	27	1295
0.021	92	4337	0.021	84	3865
0.023	25	1044	0.033	33	956
0.019	19	958	0.025	18	702

0.012	14	1201	0.014	13	928
0.111	181	1451	0.179	242	1112
0.196	198	814	0.236	201	652
0.027	118	4316	0.031	113	3511
0.066	42	596	0.068	37	504
0.053	49	869	0.063	49	729
0.068	119	1622	0.08	112	1288
0.032	50	1492	0.022	26	1154
0.021	21	964	0.022	19	829
0.027	41	1482	0.019	25	1322
0.024	17	688	0.028	16	551
0.061	73	1130	0.06	60	934
0.028	45	1537	0.023	28	1167
0.066	78	1108	0.065	67	963
0.023	71	2973	0.027	68	2455
0.018	17	906	0.03	25	813
0.017	23	1311	0.029	30	1015
0.036	63	1696	0.028	40	1387
0.032	60	1822	0.027	44	1564
0.027	60	2166	0.021	38	1780
0.06	103	1616	0.075	114	1415
0.017	28	1593	0.025	34	1350
0.027	51	1805	0.025	38	1479
0.015	41	2655	0.019	47	2386
0.02	83	4003	0.021	70	3211
0.021	51	2348	0.025	50	1917
0.047	74	1494	0.078	101	1196
0.028	69	2422	0.017	39	2245
0.154	360	1981	0.17	340	1662
0.021	134	6150	0.023	127	5335
0.024	17	697	0.027	15	543
0.039	40	977	0.035	29	800
0.02	48	2335	0.022	45	1975
0.025	25	971	0.042	37	852
0.028	66	2289	0.023	47	1959
0.031	106	3364	0.024	71	2895
0.032	67	2015	0.04	70	1691
0.024	78	3185	0.029	81	2701
0.023	59	2500	0.033	72	2130
0.024	54	2238	0.027	56	2055

0.019	27	1408	0.023	26	1105
0.033	49	1423	0.026	36	1347
0.02	21	1008	0.022	18	817
0.028	15	512	0.019	9	470
0.02	76	3747	0.022	76	3346
0.018	16	851	0.028	22	763
0.435	397	516	0.546	473	393
0.072	111	1433	0.057	73	1216
0.025	71	2726	0.025	61	2424
0.02	62	3030	0.026	61	2286
0.024	29	1178	0.023	25	1044
0.028	90	3166	0.021	56	2631
0.023	133	5670	0.021	105	4954
0.02	40	1978	0.03	48	1537
0.024	83	3334	0.031	83	2611
0.038	42	1071	0.024	23	950
0.031	42	1319	0.021	24	1101
0.021	59	2779	0.021	57	2596
0.083	102	1122	0.086	87	922
0.075	133	1647	0.093	140	1363
0.021	33	1518	0.02	27	1355
0.021	44	2091	0.022	42	1842
0.082	140	1558	0.052	77	1394
0.027	127	4640	0.023	93	3883
0.022	30	1311	0.023	26	1102
0.042	41	927	0.031	23	730
0.069	169	2270	0.066	131	1845
0.05	129	2448	0.063	138	2044
0.03	92	2966	0.025	67	2588
0.023	43	1788	0.029	43	1415
0.031	71	2256	0.026	47	1728
0.02	62	3073	0.018	50	2673
0.069	128	1717	0.065	98	1407
0.022	113	5124	0.023	104	4439
0.029	43	1460	0.036	45	1222
0.026	157	5807	0.021	102	4858
0.017	23	1306	0.018	20	1115
0.046	53	1092	0.067	68	947
0.024	72	2976	0.022	55	2471
0.024	28	1148	0.019	20	1027

0.024	42	1685	0.028	43	1487
0.017	33	1948	0.028	50	1740
0.033	40	1176	0.014	15	1061
0.025	26	1033	0.035	32	889
0.025	68	2620	0.028	63	2148
0.023	50	2145	0.013	25	1902
0.027	46	1635	0.035	48	1341
0.1	77	694	0.094	60	581
0.025	40	1546	0.027	39	1398
0.08	138	1595	0.101	140	1243
0.034	24	681	0.016	11	661
0.054	64	1132	0.037	36	947
0.064	117	1712	0.081	113	1276
0.017	38	2229	0.027	52	1871
0.026	41	1509	0.031	39	1220
0.032	46	1387	0.013	16	1198
0.026	35	1305	0.03	33	1061
0.024	63	2573	0.021	50	2305
0.086	50	529	0.083	36	399
0.028	32	1091	0.024	23	938
0.025	47	1817	0.02	32	1592
0.02	31	1486	0.017	21	1226
0.022	18	817	0.042	29	666
0.019	38	2009	0.031	52	1610
0.019	58	2983	0.03	75	2444
0.03	117	3743	0.025	84	3317
0.025	99	3936	0.023	76	3244
0.021	72	3307	0.026	73	2774
0.027	36	1295	0.026	28	1039
0.12	106	777	0.172	128	615
0.016	41	2591	0.022	48	2097
0.075	77	948	0.077	66	792
0.059	171	2729	0.052	117	2138
0.071	89	1164	0.118	130	973
0.02	62	3035	0.026	66	2468
0.023	65	2804	0.023	54	2313
0.026	85	3145	0.022	63	2818
0.02	40	1916	0.022	37	1621
0.023	82	3482	0.02	55	2653
0.021	41	1870	0.03	48	1558

0.018	65	3450	0.023	69	2933
0.026	44	1644	0.022	29	1316
0.024	110	4406	0.03	124	3958
0.023	131	5557	0.025	115	4416
0.021	43	2012	0.025	43	1650
0.027	103	3735	0.024	82	3359
0.023	53	2296	0.022	44	1920
0.023	106	4465	0.025	91	3609
0.027	92	3318	0.026	76	2850
0.039	33	806	0.047	29	590
0.029	60	1997	0.02	36	1753
0.034	51	1438	0.019	23	1193
0.028	65	2231	0.016	30	1844
0.026	36	1329	0.028	32	1121
0.05	102	1949	0.049	87	1698
0.025	31	1232	0.024	23	937
0.014	21	1430	0.022	27	1227
0.021	44	2054	0.023	41	1743
0.02	18	872	0.036	25	678
0.016	24	1459	0.016	20	1204
0.024	31	1246	0.027	26	937
0.023	68	2894	0.024	60	2401
0.018	44	2383	0.023	44	1883
0.024	39	1590	0.03	42	1338
0.029	30	1013	0.021	17	796
0.024	67	2768	0.025	59	2287
0.032	65	1996	0.038	69	1747
0.016	43	2687	0.021	50	2311
0.049	196	3810	0.053	187	3314
0.02	34	1693	0.026	39	1457
0.022	57	2506	0.026	60	2259
0.022	29	1315	0.02	24	1179
0.063	93	1387	0.061	67	1026
0.021	82	3869	0.03	103	3350
0.027	29	1048	0.022	22	967
0.042	40	918	0.04	30	720
0.019	59	3011	0.023	59	2549
0.028	43	1485	0.03	41	1320
0.024	88	3575	0.019	61	3123
0.028	125	4375	0.02	81	3966

0.028	38	1324	0.023	24	1026
0.057	97	1607	0.053	77	1368
0.028	59	2046	0.026	45	1668
0.077	151	1812	0.092	166	1645
0.015	44	2989	0.027	71	2569
0.025	91	3617	0.021	64	2969
0.027	70	2497	0.035	77	2122
0.023	86	3610	0.02	59	2950
0.026	69	2572	0.029	66	2181
0.025	43	1658	0.025	37	1472
0.025	60	2332	0.023	43	1854
0.054	148	2582	0.06	134	2112
0.031	87	2738	0.024	58	2312
0.028	89	3139	0.032	86	2612
0.068	62	851	0.114	87	676
0.022	85	3841	0.024	81	3236
0.023	62	2596	0.021	46	2153
0.066	90	1269	0.065	76	1101
0.016	43	2564	0.02	44	2109
0.035	38	1033	0.02	19	942
0.024	46	1880	0.03	51	1677
0.016	53	3189	0.027	73	2586
0.025	85	3355	0.023	69	2877
0.69	595	267	0.787	924	250
0.025	37	1454	0.03	37	1187
0.056	114	1938	0.059	96	1541
0.034	27	770	0.03	19	614
0.018	35	1959	0.021	32	1480
0.177	177	821	0.261	229	649
0.074	62	778	0.049	30	582
0.024	54	2232	0.02	38	1831
0.02	25	1224	0.031	33	1047
0.025	72	2772	0.035	89	2485
0.025	37	1440	0.029	37	1219
0.063	200	2968	0.055	147	2513
0.029	95	3197	0.027	75	2689
0.022	88	3842	0.016	52	3170
0.022	26	1164	0.019	21	1061
0.06	99	1539	0.044	57	1236
0.034	61	1714	0.028	45	1535

0.055	127	2201	0.065	124	1783
0.023	14	607	0.021	12	572
0.022	110	4957	0.027	116	4110
0.018	21	1167	0.025	29	1119
0.021	47	2172	0.024	47	1942
0.054	114	2013	0.057	104	1720
0.056	54	912	0.034	29	830
0.027	22	787	0.02	14	695
0.023	82	3471	0.026	78	2969
0.025	25	967	0.023	16	686
0.029	135	4539	0.024	85	3503
0.025	133	5277	0.025	116	4462
0.104	94	806	0.146	124	724
0.014	18	1279	0.023	24	1003
0.176	183	856	0.266	263	726
0.049	72	1394	0.057	75	1241
0.026	42	1586	0.035	48	1319
0.026	57	2137	0.032	60	1818
0.024	88	3558	0.023	74	3079
0.026	60	2259	0.026	48	1802
0.026	93	3422	0.023	71	2965
0.21	342	1284	0.267	395	1084
0.02	71	3436	0.019	56	2919
0.049	34	658	0.056	32	543
0.018	33	1775	0.02	30	1485
0.021	18	823	0.025	16	613
0.03	37	1214	0.018	18	970
0.023	74	3133	0.03	79	2596
0.029	63	2133	0.028	51	1762
0.021	67	3145	0.024	66	2732
0.027	32	1158	0.027	26	933
0.023	50	2093	0.028	45	1585
0.04	33	784	0.07	50	662
0.02	59	2927	0.022	56	2458
0.02	54	2692	0.024	57	2352
0.02	24	1155	0.022	21	947
0.091	93	925	0.103	88	765
0.032	94	2818	0.027	64	2301
0.021	48	2214	0.027	47	1673
0.076	73	886	0.037	31	815

0.026	86	3184	0.025	69	2677
0.069	158	2148	0.062	115	1742
0.026	75	2834	0.033	82	2437
0.019	110	5648	0.025	124	4863
0.017	25	1470	0.029	38	1271
0.02	36	1803	0.025	40	1569
0.021	57	2676	0.031	68	2124
0.019	32	1671	0.021	30	1433
0.035	43	1170	0.049	50	966
0.021	47	2198	0.025	48	1862
0.031	66	2079	0.031	52	1637
0.03	43	1408	0.026	33	1257
0.027	85	3042	0.022	57	2549
0.022	36	1631	0.019	28	1408
0.03	43	1402	0.032	38	1150
0.062	163	2455	0.055	117	2019
0.026	97	3620	0.022	76	3395
0.062	65	989	0.077	61	732
0.028	45	1540	0.025	37	1441
0.09	151	1527	0.109	144	1182
0.02	36	1768	0.022	30	1329
0.043	47	1051	0.047	48	979
0.029	73	2415	0.031	62	1922
0.113	135	1063	0.129	113	761
0.029	87	2907	0.026	66	2429
0.023	26	1125	0.019	18	953
0.077	67	806	0.089	69	708
0.03	76	2467	0.031	62	1925
0.017	16	946	0.032	27	804
0.067	207	2892	0.074	180	2245
0.033	66	1941	0.021	33	1535
0.062	210	3175	0.04	118	2801
0.032	59	1811	0.026	39	1475
0.026	82	3105	0.024	68	2744
0.031	86	2692	0.029	65	2155
0.067	87	1204	0.07	74	981
0.019	18	916	0.018	14	756
0.023	47	2028	0.022	37	1636
0.03	70	2247	0.026	49	1868
0.019	33	1738	0.023	34	1465

0.031	70	2211	0.027	55	1963
0.032	63	1931	0.036	60	1608
0.024	18	727	0.013	9	658
0.041	103	2400	0.046	97	1997
0.026	71	2670	0.024	53	2175
0.047	83	1675	0.055	72	1249
0.017	39	2246	0.024	48	1913
0.025	72	2836	0.029	74	2481
0.026	72	2684	0.025	61	2390
0.017	53	3050	0.027	78	2768
0.028	75	2596	0.034	74	2090
0.056	38	642	0.06	37	576
0.031	70	2183	0.032	62	1867
0.065	179	2575	0.069	151	2039
0.029	35	1155	0.028	26	907
0.023	12	509	0.017	8	466
0.435	332	432	0.557	489	389
0.025	33	1308	0.018	21	1148
0.021	49	2338	0.017	34	1946
0.019	33	1676	0.024	39	1585
0.021	133	6199	0.024	132	5472
0.056	127	2152	0.056	107	1803
0.024	16	643	0.027	14	499
0.026	146	5409	0.028	123	4263
0.138	167	1045	0.158	142	759
0.019	52	2711	0.024	58	2382
0.057	166	2766	0.045	110	2342
0.088	104	1074	0.095	89	847
0.018	41	2252	0.019	36	1887
0.024	95	3903	0.021	77	3539
0.018	30	1642	0.027	34	1242
0.088	93	961	0.073	61	778
0.054	107	1889	0.065	108	1543
0.056	127	2131	0.049	89	1711
0.014	19	1299	0.023	25	1076
0.03	69	2255	0.027	58	2078
0.02	25	1245	0.032	34	1016
0.02	20	962	0.029	25	830
0.059	88	1405	0.037	47	1237
0.056	53	891	0.065	48	691

0.018	24	1341	0.021	26	1224
0.025	148	5854	0.026	136	5027
0.055	72	1241	0.063	76	1132
0.019	29	1514	0.014	21	1456
0.037	41	1082	0.03	30	956
0.068	54	735	0.053	37	655
0.027	38	1367	0.031	38	1194
0.02	37	1832	0.042	69	1556
0.021	51	2420	0.028	59	2085
0.075	80	986	0.129	123	829
0.019	58	3020	0.023	60	2530
0.068	141	1931	0.08	138	1586
0.036	106	2833	0.03	79	2553
0.021	42	1913	0.03	52	1655
0.054	139	2430	0.044	98	2116
0.038	53	1345	0.026	30	1111
0.024	76	3092	0.025	72	2779
0.029	59	1949	0.019	34	1731
0.027	67	2406	0.031	63	2001
0.026	25	921	0.03	26	836
0.062	67	1018	0.047	46	929
0.015	23	1551	0.02	25	1240
0.03	88	2814	0.025	62	2396
0.029	47	1554	0.026	33	1249
0.032	41	1258	0.023	23	992
0.059	59	934	0.056	48	814
0.057	75	1236	0.059	61	981
0.044	25	544	0.086	46	491
0.047	45	922	0.056	40	678
0.029	52	1768	0.029	46	1535
0.028	73	2509	0.025	53	2076
0.06	110	1726	0.061	97	1483
0.026	116	4362	0.028	116	4031
0.025	41	1602	0.027	44	1564
0.065	221	3180	0.064	173	2545
0.062	126	1922	0.066	109	1543
0.027	29	1041	0.027	24	868
0.055	78	1339	0.058	67	1090
0.115	152	1171	0.144	170	1011
0.019	13	675	0.022	14	624

0.027	34	1220	0.023	24	1039
0.019	89	4594	0.022	91	3978
0.022	49	2180	0.021	38	1808
0.021	31	1447	0.026	31	1181
0.076	128	1552	0.095	130	1239
0.026	89	3301	0.03	82	2662
0.022	33	1497	0.023	29	1214
0.016	32	1947	0.032	55	1641
0.066	72	1026	0.059	54	867
0.019	29	1520	0.024	31	1251
0.028	56	1950	0.025	44	1698
0.235	380	1234	0.354	541	989
0.022	148	6729	0.028	170	5930
0.077	71	852	0.067	55	765
0.03	56	1831	0.025	39	1532
0.031	74	2346	0.023	46	1934
0.049	22	427	0.091	39	388
0.08	191	2207	0.084	178	1946
0.052	151	2754	0.051	121	2232
0.03	30	956	0.021	17	780
0.023	69	2949	0.021	54	2477
0.041	50	1170	0.038	41	1028
0.026	51	1884	0.025	40	1584
0.021	52	2437	0.024	47	1951
0.029	73	2441	0.028	59	2041
0.029	65	2142	0.019	37	1898
0.018	11	600	0.015	8	517
0.022	23	1042	0.034	30	846
0.021	64	2989	0.022	58	2542
0.025	56	2190	0.034	67	1879
0.022	56	2509	0.025	54	2128
0.026	83	3094	0.019	51	2635
0.029	56	1881	0.033	56	1617
0.023	113	4853	0.023	100	4167
0.414	531	751	0.457	597	709
0.061	145	2248	0.059	109	1739
0.061	95	1468	0.056	69	1163
0.026	48	1770	0.03	44	1416
0.029	41	1380	0.033	36	1070
0.068	62	845	0.075	55	677

0.024	62	2495	0.019	42	2150
0.026	78	2878	0.023	56	2427
0.015	12	810	0.034	23	651
0.024	21	840	0.018	14	763
0.025	65	2531	0.022	51	2250
0.02	48	2336	0.022	43	1903
0.024	83	3371	0.022	62	2737
0.027	115	4215	0.027	99	3570
0.026	49	1868	0.031	52	1626
0.028	92	3137	0.023	62	2663
0.025	115	4560	0.023	85	3684
0.025	148	5698	0.029	144	4803
0.031	53	1661	0.034	48	1354
0.022	65	2941	0.027	66	2369
0.07	162	2140	0.059	117	1872
0.199	132	531	0.25	170	510
0.044	65	1429	0.044	55	1200
0.022	39	1762	0.038	62	1571
0.029	20	662	0.03	18	587
0.018	20	1065	0.024	24	964
0.061	101	1557	0.07	84	1118
0.045	39	835	0.024	17	702
0.028	60	2055	0.026	50	1909
0.032	78	2330	0.028	54	1907
0.022	46	2031	0.031	59	1843
0.027	58	2064	0.028	48	1662
0.016	13	800	0.018	12	661
0.04	43	1027	0.048	40	798
0.018	26	1410	0.038	47	1189
0.02	100	4837	0.023	100	4227
0.02	38	1910	0.027	50	1780
0.025	86	3380	0.025	73	2878
0.024	71	2885	0.018	46	2466
0.033	58	1683	0.029	46	1551
0.021	37	1763	0.023	38	1599
0.052	151	2771	0.064	161	2343
0.061	118	1824	0.06	107	1666
0.075	61	754	0.058	38	612
0.028	60	2087	0.025	50	1929
0.02	118	5647	0.024	121	4958

0.031	26	811	0.027	19	696
0.025	49	1879	0.022	36	1611
0.029	88	2989	0.024	61	2505
0.025	31	1207	0.03	30	978
0.015	26	1685	0.023	31	1316
0.06	81	1271	0.07	82	1090
0.03	23	747	0.018	11	609
0.023	23	980	0.023	20	837
0.028	82	2869	0.025	58	2244
0.026	25	939	0.035	30	826
0.028	102	3597	0.024	75	3040
0.021	35	1604	0.027	38	1373
0.018	38	2020	0.031	51	1618
0.027	57	2019	0.032	61	1843
0.025	105	4159	0.027	99	3613
0.029	77	2564	0.019	43	2164
0.035	69	1892	0.017	30	1690
0.025	101	4007	0.021	75	3417
0.026	52	1977	0.031	53	1667
0.019	60	3092	0.02	54	2687
0.063	190	2822	0.061	151	2314
0.024	41	1646	0.027	39	1387
0.064	36	527	0.044	22	476
0.025	92	3561	0.027	89	3253
0.024	20	797	0.019	13	676
0.018	14	750	0.014	9	627
0.024	106	4236	0.026	96	3611
0.017	23	1314	0.022	24	1090
0.058	174	2838	0.053	130	2331
0.066	90	1272	0.054	62	1083
0.022	53	2386	0.025	55	2120
0.019	42	2210	0.017	37	2095
0.031	86	2678	0.03	75	2421
0.025	76	3017	0.029	76	2539
0.061	52	804	0.053	34	604
0.06	97	1509	0.062	79	1201
0.018	72	3978	0.024	86	3572
0.033	29	848	0.025	20	794
0.028	68	2378	0.027	60	2129
0.025	88	3425	0.024	67	2742

0.028	101	3571	0.027	93	3320
0.026	34	1254	0.041	45	1046
0.025	35	1385	0.028	29	990
0.015	11	709	0.02	14	674
0.072	62	803	0.06	39	606
0.066	107	1510	0.064	90	1327
0.027	48	1719	0.021	31	1445
0.021	59	2767	0.034	82	2358
0.022	61	2713	0.024	60	2485
0.024	63	2560	0.03	70	2271
0.02	58	2820	0.027	65	2384
0.024	47	1915	0.023	39	1687
0.025	56	2147	0.027	47	1675
0.334	419	835	0.464	655	758
0.034	34	958	0.024	19	768
0.032	62	1854	0.035	54	1500
0.037	32	824	0.084	66	722
0.011	12	1075	0.025	25	977
0.063	68	1005	0.037	31	799
0.018	28	1516	0.027	34	1234
0.104	106	918	0.092	77	759
0.021	84	3898	0.024	75	3011
0.025	63	2426	0.035	72	1981
0.061	86	1320	0.054	62	1085
0.028	58	2027	0.033	59	1734
0.017	53	3141	0.023	64	2694
0.031	73	2281	0.026	54	2024
0.027	73	2642	0.022	55	2467
0.035	46	1286	0.023	26	1116
0.028	130	4454	0.03	113	3642
0.027	17	613	0.044	25	539
0.041	31	732	0.028	19	664
0.02	119	5797	0.02	99	4858
0.026	40	1495	0.029	39	1306
0.018	39	2121	0.025	43	1694
0.041	50	1155	0.058	56	909
0.021	118	5487	0.022	102	4532
0.018	31	1711	0.025	37	1432
0.057	207	3418	0.044	133	2856
0.031	58	1784	0.028	46	1593

0.024	68	2817	0.027	62	2194
0.028	35	1232	0.037	44	1137
0.023	44	1897	0.023	38	1606
0.029	76	2523	0.024	51	2046
0.025	44	1737	0.024	34	1410
0.022	82	3622	0.022	72	3129
0.021	29	1357	0.031	38	1190
0.019	16	805	0.031	22	694
0.079	58	676	0.075	56	691
0.011	11	1026	0.027	25	896
0.021	37	1743	0.018	26	1437
0.022	14	634	0.024	13	523
0.056	122	2061	0.077	131	1564
0.027	111	4010	0.029	102	3393
0.031	128	3955	0.028	99	3464
0.066	64	912	0.063	49	728
0.024	58	2366	0.022	44	1924
0.027	96	3481	0.028	83	2866
0.02	68	3300	0.021	60	2832
0.018	50	2741	0.031	73	2280
0.257	599	1734	0.377	954	1576
0.024	41	1646	0.029	43	1437
0.044	27	593	0.041	21	488
0.051	58	1082	0.039	35	870
0.061	129	2003	0.05	86	1618
0.024	55	2193	0.034	65	1860
0.069	145	1943	0.076	137	1672
0.027	48	1715	0.024	35	1446
0.025	71	2738	0.027	67	2435
0.02	54	2653	0.016	37	2230
0.031	98	3015	0.028	73	2517
0.033	73	2119	0.022	39	1751
0.035	52	1448	0.032	39	1198
0.026	38	1433	0.028	33	1152
0.046	37	775	0.043	31	698
0.025	52	2048	0.029	46	1520
0.027	57	2081	0.031	52	1629
0.023	60	2514	0.029	69	2280
0.141	246	1500	0.194	307	1276
0.021	34	1609	0.02	24	1203

0.026	49	1860	0.031	51	1592
0.054	60	1048	0.05	51	977
0.029	64	2115	0.033	62	1828
0.018	56	3009	0.02	55	2703
0.033	79	2296	0.029	58	1924
0.025	58	2252	0.02	38	1866
0.031	41	1293	0.027	31	1113
0.024	29	1175	0.04	37	897
0.024	59	2372	0.027	55	1958
0.023	54	2253	0.028	51	1763
0.024	128	5191	0.025	111	4309
0.016	14	883	0.032	28	855
0.023	46	1973	0.018	33	1765
0.021	22	1022	0.03	26	829
0.023	19	802	0.016	12	718
0.039	46	1137	0.03	31	996
0.05	68	1293	0.065	77	1106
0.021	74	3447	0.033	105	3065
0.026	101	3833	0.031	105	3243
0.018	11	586	0.029	16	528
0.025	66	2523	0.026	54	2020
0.026	52	1939	0.026	41	1509
0.025	88	3501	0.031	87	2716
0.014	7	507	0.029	15	502
0.022	44	1914	0.019	32	1683
0.042	47	1062	0.069	67	903
0.027	66	2379	0.027	54	1963
0.024	29	1189	0.024	26	1073
0.024	88	3521	0.022	64	2846
0.022	40	1767	0.034	53	1507
0.031	52	1639	0.023	30	1299
0.058	111	1798	0.058	98	1590
0.108	150	1236	0.114	129	1003
0.02	55	2754	0.019	46	2362
0.026	66	2502	0.037	78	2006
0.03	107	3457	0.021	62	2858
0.074	67	835	0.046	38	791
0.053	102	1813	0.055	82	1398
0.047	67	1357	0.049	55	1060
0.027	74	2682	0.024	58	2390

0.161	143	747	0.252	193	573
0.021	37	1689	0.027	37	1332
0.023	15	649	0.021	10	469
0.024	71	2827	0.016	37	2313
0.015	16	1043	0.027	23	821
0.027	52	1866	0.019	29	1486
0.017	24	1368	0.026	31	1144
0.03	56	1803	0.02	29	1416
0.085	66	712	0.069	46	618
0.025	178	6908	0.025	157	6036
0.027	113	4028	0.029	105	3531
0.033	29	860	0.022	16	703
0.023	52	2212	0.017	31	1755
0.026	112	4246	0.024	88	3543
0.027	87	3093	0.028	75	2640
0.023	43	1850	0.015	22	1445
0.058	131	2112	0.08	164	1884
0.055	77	1331	0.057	62	1032
0.031	40	1242	0.033	29	860
0.023	40	1690	0.034	51	1430
0.017	54	3104	0.022	60	2668
0.028	62	2179	0.028	56	1938
0.072	119	1527	0.069	96	1291
0.06	59	926	0.059	46	736
0.025	75	2927	0.019	48	2428
0.026	31	1145	0.027	26	932
0.059	57	901	0.06	49	769
0.025	35	1347	0.025	30	1193
0.027	62	2248	0.024	48	1981
0.06	59	927	0.055	49	836
0.045	24	506	0.073	34	433
0.02	47	2312	0.025	48	1836
0.022	20	887	0.032	23	706
0.018	23	1235	0.029	32	1068
0.021	52	2429	0.028	57	1972
0.067	144	2019	0.073	130	1663
0.037	58	1520	0.042	57	1290
0.022	146	6438	0.023	134	5602
0.018	48	2583	0.024	49	1975
0.021	72	3424	0.026	75	2841

0.076	65	790	0.091	58	580
0.021	56	2557	0.018	36	2003
0.037	98	2561	0.022	51	2253
0.03	42	1354	0.034	36	1029
0.016	44	2684	0.033	79	2292
0.02	45	2194	0.032	58	1752
0.052	47	854	0.049	35	679
0.026	44	1666	0.029	40	1348
0.062	104	1568	0.08	108	1238
0.024	169	6859	0.027	162	5917
0.016	25	1532	0.019	25	1294
0.022	37	1623	0.031	44	1380
0.063	89	1321	0.053	58	1028
0.171	158	766	0.247	198	604
0.024	22	898	0.027	22	794
0.026	134	5094	0.022	97	4355
0.029	31	1030	0.021	20	923
0.026	28	1054	0.024	23	928
0.028	50	1763	0.025	34	1314
0.025	59	2348	0.024	51	2089
0.022	71	3182	0.027	72	2579
0.024	73	2942	0.021	54	2513
0.016	24	1520	0.028	39	1357
0.028	48	1672	0.036	53	1429
0.021	53	2489	0.022	51	2220
0.022	59	2646	0.022	48	2144
0.021	74	3387	0.027	77	2810
0.027	40	1453	0.043	58	1293
0.027	52	1886	0.024	37	1480
0.027	44	1560	0.03	42	1381
0.021	47	2144	0.024	43	1738
0.032	37	1128	0.029	28	934
0.02	7	348	0.043	13	288
0.057	121	2008	0.057	105	1723
0.019	37	1903	0.028	44	1547
0.012	12	1004	0.037	31	816
0.066	122	1732	0.057	98	1629
0.021	40	1849	0.03	49	1609
0.031	61	1915	0.029	45	1498
0.028	69	2391	0.022	48	2101

0.027	126	4608	0.024	96	3883
0.035	118	3239	0.029	81	2737
0.024	46	1843	0.022	37	1675
0.023	44	1844	0.017	26	1511
0.025	98	3875	0.023	83	3587
0.021	57	2598	0.027	59	2156
0.018	27	1503	0.021	28	1330
0.024	46	1859	0.02	34	1644
0.021	27	1275	0.025	25	995
0.023	94	3925	0.021	79	3602
0.029	64	2113	0.028	50	1733
0.374	540	902	0.47	681	768
0.018	54	2876	0.018	44	2359
0.052	159	2912	0.055	149	2574
0.031	48	1506	0.034	46	1320
0.022	89	3939	0.029	96	3197
0.022	103	4661	0.024	104	4172
0.019	17	872	0.014	12	872
0.023	101	4249	0.022	87	3894
0.033	58	1678	0.024	36	1488
0.023	60	2557	0.021	47	2165
0.03	54	1760	0.014	21	1525
0.029	43	1458	0.019	24	1269
0.062	54	823	0.068	55	750
0.052	55	1010	0.056	58	973
0.025	55	2189	0.023	45	1879
0.029	33	1114	0.027	28	1005
0.02	35	1692	0.037	53	1382
0.018	44	2423	0.034	68	1925
0.025	48	1872	0.02	31	1545
0.023	48	2031	0.032	56	1695
0.024	46	1873	0.022	39	1716
0.442	618	780	0.574	835	619
0.022	43	1921	0.031	51	1594
0.045	79	1667	0.048	79	1554
0.033	68	2016	0.028	50	1753
0.12	298	2179	0.155	337	1840
0.021	38	1810	0.03	42	1348
0.023	54	2331	0.03	62	2002
0.032	71	2142	0.035	66	1824

0.026	43	1596	0.036	50	1331
0.018	13	709	0.034	21	595
0.023	82	3462	0.024	72	2917
0.024	34	1373	0.017	21	1196
0.021	59	2704	0.021	48	2281
0.035	69	1928	0.02	32	1607
0.079	39	455	0.078	33	389
0.025	62	2385	0.032	66	2000
0.026	56	2140	0.019	35	1802
0.025	14	555	0.031	13	401
0.033	58	1684	0.024	34	1375
0.028	64	2202	0.029	57	1940
0.018	48	2614	0.02	44	2144
0.023	67	2823	0.026	65	2463
0.028	50	1766	0.033	49	1435
0.028	106	3682	0.026	86	3234
0.029	27	917	0.031	24	756
0.036	15	402	0.031	11	339
0.018	31	1658	0.029	44	1493
0.071	59	776	0.06	46	724
0.022	52	2323	0.022	44	1923
0.025	47	1839	0.023	41	1705
0.023	85	3598	0.025	86	3371
0.022	94	4112	0.025	89	3441
0.025	37	1455	0.02	25	1216
0.085	119	1281	0.103	124	1083
0.066	53	746	0.072	45	583
0.024	14	578	0.028	15	523
0.021	15	690	0.035	22	610
0.033	55	1596	0.019	28	1461
0.014	16	1136	0.029	30	1000
0.026	33	1215	0.023	24	1029
0.028	135	4624	0.023	95	3976
0.04	39	936	0.059	51	808
0.041	40	927	0.083	64	709
0.017	26	1489	0.025	34	1333
0.031	129	4026	0.028	101	3490
0.021	50	2299	0.02	43	2064
0.025	97	3791	0.02	67	3236
0.026	47	1786	0.019	30	1523

0.025	104	4003	0.027	97	3504
0.056	170	2844	0.055	137	2347
0.035	55	1519	0.021	29	1340
0.026	49	1826	0.026	43	1585
0.061	169	2582	0.056	121	2026
0.044	81	1746	0.049	75	1442
0.006	4	717	0.028	17	581
0.034	23	662	0.029	16	535
0.022	50	2213	0.028	57	1967
0.045	47	992	0.063	55	825
0.023	33	1392	0.029	32	1083
0.022	41	1858	0.024	40	1633
0.026	54	1986	0.026	42	1549
0.024	71	2906	0.033	84	2457
0.022	40	1749	0.018	28	1526
0.029	48	1586	0.032	41	1252
0.025	19	735	0.034	20	564
0.024	46	1847	0.028	48	1685
0.029	117	3859	0.026	97	3685
0.038	55	1378	0.042	57	1291
0.029	44	1462	0.028	34	1191
0.021	46	2152	0.018	37	1985
0.053	35	621	0.062	32	487
0.023	52	2208	0.038	69	1752
0.024	45	1849	0.026	43	1611
0.015	31	2100	0.021	37	1757
0.018	31	1681	0.021	29	1385
0.025	32	1253	0.025	29	1149
0.029	160	5437	0.025	111	4342
0.029	50	1645	0.027	40	1462
0.029	62	2074	0.028	49	1672
0.026	38	1442	0.025	32	1269
0.027	38	1379	0.033	42	1216
0.034	25	718	0.03	18	592
0.02	81	3878	0.029	105	3504
0.024	50	2072	0.019	36	1840
0.024	42	1728	0.028	41	1445
0.337	525	1035	0.427	681	915
0.026	51	1939	0.023	41	1731
0.505	864	847	0.646	1264	693

0.022	73	3184	0.027	75	2686
0.089	177	1811	0.093	168	1647
0.024	133	5435	0.019	88	4544
0.019	39	1986	0.027	45	1619
0.023	29	1233	0.026	27	996
0.017	52	3065	0.017	47	2712
0.023	31	1292	0.018	19	1047
0.03	55	1763	0.036	59	1559
0.038	54	1375	0.024	30	1217
0.069	144	1933	0.061	107	1641
0.021	52	2401	0.024	51	2037
0.025	44	1706	0.025	34	1328
0.081	147	1675	0.09	140	1413
0.07	84	1114	0.079	77	898
0.069	64	860	0.072	58	745
0.026	43	1590	0.025	35	1362
0.019	22	1157	0.028	31	1074
0.027	89	3232	0.023	69	2871
0.063	62	915	0.082	64	712
0.051	221	4149	0.057	208	3416
0.079	217	2526	0.114	250	1939
0.054	44	773	0.057	43	705
0.148	207	1195	0.183	207	924
0.069	157	2128	0.09	165	1664
0.021	43	1976	0.025	41	1592
0.059	40	633	0.052	29	526
0.056	205	3432	0.051	153	2839
0.026	40	1487	0.023	30	1269
0.022	125	5499	0.025	123	4737
0.03	83	2716	0.024	63	2521
0.031	65	2057	0.03	58	1851
0.025	48	1899	0.037	61	1585
0.026	50	1896	0.014	25	1700
0.021	60	2789	0.022	51	2264
0.024	48	1933	0.024	42	1694
0.023	51	2124	0.024	40	1639
0.066	102	1453	0.09	116	1174
0.024	30	1220	0.041	40	924
0.03	61	1968	0.025	42	1658
0.026	37	1400	0.019	22	1115

0.022	60	2661	0.027	66	2387
0.028	106	3705	0.033	101	2936
0.023	63	2654	0.02	44	2167
0.031	73	2248	0.033	68	1982
0.029	105	3482	0.038	120	3047
0.022	50	2256	0.02	41	1972
0.021	53	2477	0.025	56	2209
0.027	39	1425	0.016	23	1421
0.111	215	1721	0.153	237	1314
0.079	153	1784	0.083	131	1451
0.061	225	3444	0.059	185	2940
0.025	43	1685	0.041	57	1332
0.02	39	1907	0.03	50	1642
0.024	81	3313	0.02	57	2844
0.028	40	1395	0.018	20	1117
0.022	65	2832	0.02	42	2069
0.062	68	1025	0.053	51	909
0.051	56	1034	0.073	63	805
0.053	70	1246	0.047	50	1014
0.027	58	2072	0.028	53	1848
0.029	46	1522	0.018	24	1278
0.015	18	1186	0.021	22	1034
0.051	55	1027	0.074	62	776
0.057	80	1329	0.071	83	1092
0.023	46	1942	0.022	37	1626
0.024	172	7082	0.024	152	6229
0.025	68	2637	0.029	61	2071
0.037	42	1088	0.039	42	1027
0.024	108	4338	0.028	108	3741
0.046	29	606	0.084	43	469
0.023	62	2685	0.023	54	2305
0.026	65	2443	0.027	57	2060
0.026	88	3335	0.027	79	2822
0.021	23	1070	0.027	24	851
0.035	92	2517	0.028	60	2049
0.024	11	450	0.034	15	428
0.024	71	2850	0.022	54	2354
0.027	73	2618	0.028	64	2260
0.021	61	2786	0.027	66	2390
0.031	106	3266	0.029	86	2876

0.055	61	1050	0.055	44	762
0.026	34	1296	0.027	30	1097
0.024	35	1444	0.033	43	1246
0.012	21	1694	0.03	48	1566
0.109	175	1432	0.126	167	1157
0.025	34	1327	0.03	35	1145
0.024	25	1023	0.037	33	862
0.022	108	4836	0.021	86	4064
0.029	40	1335	0.021	25	1161
0.055	79	1345	0.057	68	1128
0.036	38	1020	0.019	18	918
0.026	64	2439	0.028	59	2030
0.056	84	1421	0.071	98	1277
0.031	165	5182	0.026	121	4488
0.029	106	3599	0.024	78	3203
0.034	59	1663	0.019	28	1430
0.028	75	2568	0.03	67	2202
0.024	81	3287	0.03	80	2598
0.02	13	627	0.034	19	534
0.032	59	1772	0.022	32	1398
0.025	58	2266	0.024	50	2060
0.022	190	8577	0.025	181	7142
0.076	222	2697	0.072	172	2232
0.022	35	1581	0.016	23	1393
0.03	27	887	0.013	10	765
0.027	46	1676	0.021	28	1314
0.068	60	828	0.047	38	778
0.023	64	2690	0.022	55	2442
0.034	29	819	0.038	31	786
0.049	54	1041	0.051	50	934
0.038	29	730	0.015	9	589
0.026	50	1889	0.022	35	1561
0.025	38	1509	0.022	26	1159
0.027	26	922	0.025	20	791
0.065	97	1392	0.062	78	1182
0.016	11	664	0.038	20	500
0.113	202	1579	0.172	256	1236
0.024	56	2295	0.022	45	1984
0.056	292	4914	0.056	246	4160
0.027	81	2974	0.025	68	2676

0.029	72	2381	0.035	68	1867
0.061	128	1980	0.05	94	1769
0.115	137	1053	0.148	138	794
0.025	87	3355	0.031	91	2833
0.026	35	1329	0.026	30	1133
0.021	23	1052	0.036	31	819
0.028	41	1426	0.023	28	1207
0.021	74	3460	0.02	63	3167
0.053	114	2028	0.059	104	1671
0.064	232	3416	0.045	141	2962
0.698	576	249	0.818	761	169
0.028	91	3207	0.025	72	2797
0.026	58	2175	0.024	49	2024
0.017	43	2438	0.024	52	2113
0.025	154	5989	0.02	102	5030
0.024	44	1765	0.029	44	1461
0.018	23	1230	0.029	30	1006
0.381	573	930	0.443	608	766
0.023	66	2807	0.03	72	2332
0.026	91	3400	0.033	91	2689
0.025	31	1208	0.02	24	1196
0.028	31	1082	0.025	25	982
0.025	76	2945	0.025	58	2305
0.052	61	1111	0.054	52	912
0.022	35	1551	0.031	41	1267
0.028	18	624	0.038	25	634
0.026	91	3470	0.026	82	3130
0.026	70	2647	0.029	73	2444
0.026	60	2270	0.022	43	1872
0.072	61	787	0.063	42	623
0.022	48	2133	0.018	32	1757
0.025	21	823	0.042	31	707
0.035	95	2641	0.026	65	2422
0.022	21	921	0.033	28	809
0.026	98	3660	0.026	87	3201
0.025	62	2443	0.023	49	2082
0.031	69	2170	0.024	47	1913
0.029	28	951	0.027	24	878
0.048	93	1836	0.046	79	1621
0.123	121	865	0.162	142	737

0.052	96	1742	0.045	73	1564
0.031	47	1489	0.022	28	1263
0.019	78	3933	0.027	92	3269
0.024	107	4337	0.022	81	3558
0.027	158	5683	0.024	119	4875
0.02	44	2196	0.026	46	1748
0.022	52	2317	0.023	50	2111
0.033	47	1382	0.037	42	1085
0.022	110	4908	0.026	111	4166
0.025	25	983	0.02	17	828
0.021	29	1381	0.026	34	1251
0.013	10	774	0.017	11	648
0.026	28	1061	0.018	18	959
0.058	147	2376	0.06	133	2092
0.024	22	905	0.024	19	781
0.055	85	1456	0.051	64	1192
0.032	37	1125	0.026	28	1070
0.072	58	748	0.06	41	643
0.066	53	756	0.062	43	649
0.048	62	1226	0.057	59	983
0.108	94	779	0.151	119	667
0.028	69	2392	0.03	59	1909
0.216	225	818	0.331	322	651
0.055	61	1053	0.053	53	942
0.088	181	1878	0.082	138	1543
0.022	34	1517	0.017	23	1293
0.723	1106	423	0.819	1556	345
0.08	186	2153	0.095	186	1762
0.022	39	1773	0.028	38	1340
0.02	55	2739	0.026	65	2415
0.024	69	2837	0.024	60	2453
0.023	41	1748	0.017	27	1541
0.023	71	3065	0.024	63	2606
0.02	77	3752	0.026	76	2805
0.024	111	4443	0.03	118	3815
0.032	186	5647	0.029	134	4566
0.024	61	2438	0.02	41	2018
0.023	81	3405	0.032	91	2772
0.028	71	2445	0.026	59	2203
0.024	71	2909	0.028	75	2573

0.054	54	950	0.073	65	831
0.026	141	5288	0.021	103	4774
0.049	81	1567	0.078	108	1276
0.054	55	957	0.048	41	809
0.029	38	1269	0.02	22	1088
0.028	54	1889	0.024	41	1637
0.028	15	528	0.032	12	368
0.021	34	1579	0.031	43	1355
0.025	75	2972	0.019	47	2472
0.033	70	2076	0.024	40	1660
0.024	31	1242	0.011	12	1069
0.025	47	1856	0.028	47	1624
0.024	25	1034	0.014	12	829
0.077	51	615	0.072	44	567
0.026	53	1993	0.03	55	1771
0.029	32	1081	0.034	31	873
0.038	54	1374	0.017	22	1290
0.025	19	748	0.016	11	675
0.03	52	1661	0.03	43	1370
0.039	38	939	0.014	10	705
0.023	59	2535	0.024	53	2150
0.023	60	2548	0.026	60	2221
0.021	157	7366	0.022	143	6365
0.027	129	4565	0.039	153	3731
0.049	42	816	0.084	57	621
0.082	45	507	0.121	56	408
0.024	52	2102	0.023	43	1823
0.023	43	1823	0.023	35	1510
0.022	57	2492	0.029	63	2073
0.017	23	1303	0.03	32	1031
0.024	50	2044	0.024	42	1745
0.029	39	1313	0.021	23	1097
0.016	22	1347	0.025	31	1231
0.017	18	1026	0.017	15	889
0.02	29	1403	0.032	42	1291
0.024	97	3930	0.021	76	3490
0.029	30	999	0.016	14	885
0.026	25	920	0.028	22	762
0.019	17	878	0.022	16	712
0.073	84	1072	0.062	59	892

0.052	46	845	0.052	43	787
0.029	37	1250	0.017	17	970
0.052	33	602	0.032	17	515
0.018	25	1349	0.023	26	1100
0.019	57	2905	0.026	65	2444
0.016	51	3164	0.026	71	2636
0.026	53	2008	0.025	47	1806
0.063	178	2667	0.061	149	2292
0.024	82	3275	0.027	80	2926
0.106	66	555	0.168	79	390
0.024	85	3492	0.025	82	3190
0.061	78	1191	0.064	68	995
0.018	28	1529	0.014	19	1307
0.027	51	1840	0.023	35	1502
0.017	25	1483	0.042	49	1123
0.021	41	1879	0.021	36	1663
0.021	79	3698	0.017	58	3324
0.02	20	978	0.026	22	831
0.088	98	1014	0.082	68	759
0.032	36	1096	0.024	24	968
0.028	86	2961	0.021	54	2504
0.863	1036	165	0.904	1439	152
0.057	163	2675	0.052	122	2239
0.044	44	946	0.049	42	808
0.023	64	2674	0.025	60	2308
0.019	50	2644	0.026	59	2249
0.03	71	2304	0.021	40	1869
0.022	28	1245	0.023	25	1080
0.021	51	2347	0.034	67	1925
0.027	79	2890	0.025	65	2523
0.029	78	2621	0.022	50	2253
0.03	88	2823	0.026	65	2402
0.028	101	3461	0.026	77	2908
0.018	20	1082	0.03	23	733
0.02	36	1806	0.024	38	1558
0.019	41	2089	0.025	44	1748
0.025	79	3142	0.023	61	2592
0.025	137	5362	0.021	95	4382
0.058	107	1748	0.059	86	1372
0.024	64	2659	0.029	66	2243

0.02	18	874	0.037	26	686
0.026	58	2189	0.024	47	1891
0.023	40	1672	0.036	50	1358
0.028	54	1891	0.026	45	1718
0.022	27	1218	0.024	24	988
0.024	51	2109	0.024	41	1639
0.057	164	2695	0.043	97	2162
0.02	46	2284	0.029	58	1914
0.013	10	762	0.027	20	724
0.022	59	2635	0.024	54	2197
0.02	60	2902	0.022	56	2527
0.033	79	2327	0.029	55	1839
0.023	54	2260	0.026	53	1974
0.025	96	3768	0.033	100	2976
0.067	156	2158	0.056	99	1659
0.021	33	1521	0.024	29	1204
0.023	48	2009	0.033	55	1600
0.055	133	2299	0.059	128	2034
0.032	43	1299	0.047	51	1032
0.079	98	1150	0.095	109	1039
0.02	42	2060	0.028	45	1555
0.023	36	1530	0.022	30	1328
0.228	224	758	0.232	213	706
0.018	41	2283	0.026	53	1969
0.024	58	2314	0.026	48	1828
0.026	36	1354	0.022	25	1091
0.026	64	2369	0.021	41	1939
0.119	171	1264	0.185	236	1038
0.013	9	682	0.019	10	523
0.038	68	1725	0.046	74	1519
0.018	47	2556	0.034	82	2306
0.032	65	1969	0.023	34	1462
0.056	114	1906	0.052	87	1585
0.047	34	690	0.06	38	598
0.026	33	1221	0.025	27	1041
0.024	93	3716	0.029	98	3335
0.022	112	5086	0.022	103	4556
0.104	143	1234	0.114	135	1049
0.023	42	1812	0.044	67	1470
0.018	83	4508	0.021	80	3674

0.054	144	2504	0.059	132	2093
0.027	115	4188	0.022	86	3801
0.028	59	2031	0.032	56	1719
0.026	67	2544	0.023	52	2181
0.026	62	2327	0.022	44	1942
0.029	76	2562	0.021	49	2320
0.027	44	1588	0.033	45	1308
0.077	84	1002	0.075	74	914
0.024	69	2858	0.025	59	2284
0.025	58	2276	0.026	53	2018
0.017	26	1522	0.029	38	1263
0.021	67	3053	0.027	74	2709
0.017	34	1936	0.019	31	1617
0.017	19	1126	0.025	25	967
0.024	108	4357	0.023	85	3565
0.026	39	1455	0.021	28	1284
0.018	47	2635	0.028	67	2344
0.027	30	1066	0.027	28	991
0.028	32	1128	0.027	28	994
0.025	51	1981	0.021	37	1752
0.024	38	1537	0.022	31	1381
0.074	100	1243	0.119	139	1027
0.076	61	740	0.074	48	598
0.026	96	3616	0.022	74	3322
0.02	19	911	0.018	16	885
0.068	188	2559	0.066	160	2256
0.057	142	2364	0.052	114	2064
0.023	118	5019	0.031	132	4137
0.024	87	3470	0.029	89	2973
0.02	39	1884	0.021	34	1622
0.026	21	786	0.021	13	608
0.023	39	1622	0.031	47	1458
0.03	35	1135	0.035	36	997
0.077	61	729	0.075	57	698
0.028	54	1863	0.027	42	1541
0.024	75	3033	0.025	66	2588
0.053	57	1023	0.049	45	867
0.016	21	1270	0.04	40	965
0.027	81	2973	0.035	92	2523
0.063	111	1663	0.074	113	1414

0.025	88	3427	0.025	73	2826
0.124	101	715	0.128	86	587
0.073	139	1753	0.089	151	1538
0.027	56	1990	0.023	43	1862
0.027	64	2343	0.028	61	2150
0.026	97	3633	0.025	85	3257
0.041	75	1763	0.044	71	1557
0.023	77	3231	0.018	53	2814
0.026	69	2597	0.024	55	2262
0.023	73	3035	0.016	42	2582
0.043	62	1378	0.059	74	1183
0.019	30	1531	0.026	38	1443
0.046	73	1505	0.073	99	1261
0.032	180	5467	0.028	130	4490
0.017	26	1547	0.026	38	1408
0.026	129	4741	0.024	105	4272
0.063	108	1603	0.046	63	1306
0.034	45	1298	0.016	18	1110
0.023	29	1239	0.038	38	963
0.019	23	1202	0.034	31	884
0.023	25	1053	0.021	21	972
0.032	94	2877	0.022	56	2513
0.055	79	1345	0.073	88	1117
0.028	36	1270	0.03	35	1127
0.023	33	1376	0.022	26	1160
0.023	104	4477	0.026	94	3562
0.027	59	2109	0.026	49	1811
0.03	49	1606	0.023	32	1356
0.021	42	1918	0.025	40	1538
0.062	89	1346	0.062	69	1044
0.018	33	1802	0.024	41	1670
0.023	70	2912	0.029	68	2258
0.022	83	3725	0.028	92	3235
0.025	41	1578	0.025	35	1353
0.02	55	2685	0.025	55	2182
0.032	49	1473	0.023	29	1214
0.029	53	1753	0.031	47	1474
0.03	89	2882	0.022	53	2388
0.083	125	1388	0.043	56	1238
0.024	25	1037	0.018	16	862

0.037	38	985	0.025	23	887
0.024	39	1588	0.019	27	1378
0.017	28	1648	0.019	27	1392
0.014	16	1102	0.02	20	967
0.027	64	2279	0.025	48	1870
0.032	12	368	0.024	8	322
0.025	25	978	0.02	17	828
0.037	76	1992	0.034	57	1636
0.066	141	1982	0.059	104	1662
0.033	54	1590	0.024	32	1290
0.078	47	556	0.032	19	567
0.025	63	2449	0.032	68	2082
0.024	35	1406	0.036	40	1071
0.052	85	1547	0.041	55	1300
0.022	43	1932	0.023	38	1643
0.017	40	2306	0.025	52	2039
0.02	87	4200	0.022	83	3714
0.048	189	3740	0.043	136	3009
0.099	129	1168	0.139	171	1058
0.023	20	853	0.016	12	759
0.024	45	1858	0.015	25	1622
0.021	50	2300	0.023	47	2014
0.058	55	892	0.076	62	759
0.021	57	2663	0.025	59	2335
0.07	66	872	0.062	57	857
0.022	102	4520	0.029	111	3770
0.162	122	630	0.238	178	570
0.023	40	1687	0.027	39	1400
0.031	63	1985	0.022	35	1573
0.061	23	354	0.07	22	294
0.019	69	3615	0.028	87	3054
0.024	56	2250	0.03	62	2024
0.032	22	664	0.041	23	537
0.026	66	2508	0.02	42	2067
0.025	82	3135	0.027	76	2729
0.023	103	4387	0.03	122	3949
0.031	144	4440	0.025	100	3956
0.022	24	1049	0.024	21	866
0.066	64	901	0.038	30	769
0.082	89	997	0.095	96	911

0.023	19	816	0.039	29	717
0.028	118	4157	0.023	82	3539
0.023	68	2928	0.023	60	2517
0.022	127	5743	0.026	125	4641
0.054	94	1653	0.05	74	1408
0.023	32	1364	0.026	28	1034
0.018	20	1079	0.023	23	984
0.057	155	2569	0.07	167	2215
0.025	59	2310	0.025	48	1902
0.036	63	1708	0.031	46	1439
0.017	28	1660	0.025	37	1446
0.055	88	1518	0.04	56	1339
0.014	16	1159	0.013	13	983
0.021	87	4089	0.021	75	3426
0.058	105	1720	0.059	88	1414
0.021	37	1703	0.042	59	1349
0.029	57	1921	0.018	29	1581
0.065	57	817	0.087	65	684
0.025	63	2469	0.024	51	2068
0.022	21	923	0.018	14	778
0.024	53	2116	0.025	43	1672
0.023	63	2711	0.029	70	2386
0.024	42	1674	0.019	29	1516
0.023	34	1452	0.022	30	1344
0.019	62	3196	0.022	59	2660
0.013	15	1167	0.036	36	977
0.064	124	1805	0.046	75	1553
0.031	52	1608	0.024	37	1510
0.037	57	1497	0.021	29	1331
0.019	53	2811	0.022	55	2430
0.026	88	3251	0.027	78	2769
0.016	24	1499	0.028	34	1194
0.027	56	2050	0.02	33	1618
0.024	58	2350	0.031	61	1916
0.028	74	2578	0.025	55	2178
0.019	30	1588	0.035	42	1162
0.026	51	1944	0.029	47	1561
0.023	35	1498	0.017	21	1198
0.02	47	2347	0.025	49	1951
0.025	48	1900	0.027	44	1589

0.025	55	2106	0.018	31	1738
0.035	55	1529	0.035	44	1229
0.052	126	2295	0.054	104	1833
0.086	66	704	0.082	60	671
0.029	40	1353	0.02	26	1260
0.019	62	3207	0.026	74	2766
0.022	172	7632	0.025	163	6477
0.065	138	1995	0.054	96	1678
0.028	88	3026	0.023	59	2490
0.019	22	1133	0.022	23	1026
0.026	38	1409	0.016	20	1194
0.026	35	1327	0.032	33	1007
0.024	24	989	0.03	24	783
0.029	49	1665	0.025	37	1414
0.036	72	1946	0.019	31	1621
0.054	122	2123	0.055	105	1814
0.017	44	2518	0.02	43	2077
0.026	39	1487	0.025	32	1269
0.023	51	2202	0.026	47	1775
0.025	39	1548	0.02	26	1299
0.054	63	1111	0.063	63	940
0.017	33	1874	0.024	35	1441
0.057	116	1930	0.049	80	1549
0.033	62	1804	0.025	40	1564
0.024	57	2331	0.03	64	2091
0.019	76	4002	0.029	100	3384
0.029	84	2810	0.038	88	2257
0.034	27	774	0.038	27	690
0.071	68	890	0.056	44	739
0.026	25	935	0.009	7	750
0.056	95	1614	0.093	135	1319
0.024	29	1193	0.023	22	926
0.027	58	2117	0.011	21	1827
0.047	78	1565	0.045	61	1309
0.063	30	445	0.09	39	392
0.027	40	1436	0.029	36	1198
0.028	70	2417	0.02	43	2159
0.021	40	1872	0.026	40	1492
0.021	99	4518	0.022	86	3813
0.02	30	1442	0.034	37	1067

0.029	58	1963	0.02	36	1753
0.019	22	1136	0.023	22	934
0.04	46	1110	0.028	24	840
0.019	41	2082	0.025	43	1681
0.03	57	1859	0.031	52	1637
0.019	22	1134	0.023	23	987
0.037	87	2275	0.026	51	1881
0.023	113	4703	0.029	114	3881
0.072	112	1447	0.075	96	1186
0.026	52	1912	0.02	34	1629
0.064	78	1139	0.07	68	898
0.022	58	2596	0.027	61	2181
0.02	70	3410	0.024	74	3034
0.043	86	1933	0.05	86	1623
0.068	37	507	0.095	45	431
0.064	132	1926	0.05	92	1732
0.025	69	2723	0.025	56	2177
0.085	79	853	0.078	59	700
0.025	54	2132	0.02	39	1886
0.017	47	2639	0.03	70	2238
0.025	26	1016	0.033	32	951
0.022	35	1542	0.027	33	1208
0.026	42	1602	0.026	34	1279
0.063	108	1616	0.063	94	1400
0.028	104	3596	0.033	108	3161
0.033	63	1841	0.032	51	1558
0.029	98	3246	0.027	73	2593
0.616	963	600	0.659	1115	576
0.026	27	1012	0.031	24	760
0.021	36	1662	0.023	33	1380
0.019	26	1316	0.021	21	968
0.023	53	2257	0.025	46	1778
0.021	32	1511	0.029	40	1316
0.041	54	1272	0.049	62	1211
0.023	30	1298	0.033	39	1152
0.068	122	1681	0.056	87	1473
0.062	254	3874	0.058	202	3278
0.028	63	2224	0.028	54	1860
0.027	42	1493	0.014	19	1311
0.026	41	1545	0.025	33	1268

0.022	79	3540	0.028	88	3091
0.057	41	683	0.093	57	554
0.067	119	1663	0.049	72	1403
0.055	136	2346	0.053	106	1881
0.024	66	2655	0.024	53	2200
0.022	49	2185	0.031	62	1946
0.106	174	1460	0.105	127	1087
0.023	129	5378	0.023	114	4799
0.064	149	2192	0.059	121	1941
0.019	22	1122	0.024	22	896
0.021	49	2308	0.026	51	1901
0.026	142	5378	0.027	134	4876
0.018	28	1509	0.029	37	1220
0.029	59	1956	0.029	50	1681
0.022	56	2440	0.025	54	2104
0.02	87	4327	0.023	90	3899
0.029	17	562	0.029	14	471
0.023	80	3386	0.021	60	2809
0.033	95	2823	0.022	52	2273
0.026	48	1772	0.023	34	1446
0.091	75	746	0.097	59	552
0.023	40	1729	0.026	38	1437
0.459	477	563	0.573	645	481
0.02	48	2348	0.026	52	1979
0.026	46	1728	0.027	39	1400
0.026	79	2982	0.022	59	2663
0.02	22	1074	0.028	26	910
0.068	147	2019	0.068	114	1567
0.029	38	1252	0.027	28	1001
0.027	141	5029	0.028	121	4271
0.055	41	698	0.058	34	549
0.019	54	2719	0.017	39	2317
0.025	104	4112	0.031	105	3311
0.044	73	1593	0.053	78	1399
0.021	54	2524	0.021	45	2147
0.016	12	752	0.044	28	610
0.015	24	1545	0.025	33	1265
0.027	92	3372	0.023	74	3114
0.022	42	1899	0.03	55	1769
0.02	18	895	0.017	12	710

0.035	54	1475	0.016	21	1293
0.06	57	895	0.06	42	659
0.025	106	4129	0.025	94	3602
0.065	82	1178	0.041	41	968
0.029	57	1911	0.025	41	1624
0.05	75	1421	0.043	56	1233
0.029	184	6119	0.025	135	5225
0.02	51	2465	0.025	51	1986
0.027	62	2255	0.022	38	1670
0.02	113	5408	0.022	103	4670
0.024	53	2186	0.022	41	1856
0.028	133	4672	0.022	95	4179
0.02	35	1695	0.022	32	1399
0.015	20	1325	0.034	36	1022
0.022	51	2293	0.027	53	1930
0.023	23	993	0.024	20	826
0.035	32	888	0.039	34	842
0.028	60	2053	0.025	46	1764
0.017	27	1518	0.021	27	1274
0.063	88	1301	0.065	80	1147
0.032	58	1742	0.027	43	1554
0.053	245	4340	0.047	179	3658
0.078	93	1099	0.067	65	898
0.021	27	1234	0.026	28	1056
0.026	190	7004	0.027	157	5743
0.084	52	565	0.091	48	481
0.037	27	705	0.037	21	549
0.022	26	1134	0.017	17	968
0.02	50	2407	0.021	43	2022
0.031	48	1486	0.029	37	1258
0.053	141	2505	0.042	95	2155
0.024	95	3790	0.022	70	3137
0.011	6	530	0.028	12	413
0.019	45	2317	0.022	47	2132
0.108	182	1503	0.111	146	1164
0.034	71	1996	0.019	36	1902
0.704	643	271	0.804	876	213
0.055	48	821	0.044	36	780
0.024	40	1612	0.03	45	1458
0.022	40	1790	0.032	52	1563

0.055	18	311	0.119	35	258
0.027	57	2061	0.025	48	1844
0.021	70	3292	0.024	73	2907
0.04	92	2232	0.037	75	1946
0.02	49	2383	0.028	58	2002
0.061	109	1685	0.087	129	1359
0.061	160	2452	0.048	106	2099
0.03	120	3939	0.024	86	3480
0.058	69	1129	0.058	59	955
0.022	36	1600	0.027	39	1402
0.024	38	1557	0.023	31	1291
0.015	18	1203	0.027	28	998
0.023	71	3083	0.031	84	2638
0.033	44	1306	0.027	31	1120
0.05	54	1017	0.059	56	891
0.016	26	1584	0.026	37	1362
0.023	55	2377	0.027	57	2018
0.064	92	1347	0.05	59	1123
0.023	61	2536	0.024	56	2241
0.056	106	1795	0.05	80	1531
0.027	36	1306	0.031	32	986
0.024	46	1877	0.019	30	1561
0.055	160	2754	0.049	125	2429
0.033	22	644	0.039	23	562
0.027	34	1216	0.037	37	973
0.083	54	600	0.089	46	470
0.102	99	868	0.106	93	784
0.022	53	2333	0.029	55	1841
0.019	27	1417	0.026	30	1107
0.031	38	1200	0.035	35	971
0.069	82	1100	0.061	64	987
0.026	61	2319	0.026	51	1908
0.029	58	1911	0.021	37	1716
0.062	100	1505	0.083	104	1145
0.033	52	1517	0.031	38	1193
0.03	103	3314	0.028	78	2688
0.021	95	4364	0.035	135	3710
0.058	141	2304	0.05	104	1957
0.022	46	2008	0.037	63	1645
0.063	111	1652	0.055	79	1356

0.016	38	2346	0.026	54	1984
0.037	98	2555	0.026	55	2052
0.063	73	1084	0.095	75	711
0.028	97	3371	0.028	85	2935
0.029	101	3382	0.03	91	2947
0.028	80	2796	0.029	76	2566
0.075	43	528	0.053	23	411
0.028	48	1680	0.023	32	1382
0.072	52	671	0.067	43	598
0.025	76	2988	0.027	66	2394
0.085	47	509	0.103	52	452
0.022	82	3724	0.021	65	3075
0.056	130	2174	0.059	123	1947
0.114	277	2163	0.167	329	1642
0.015	10	636	0.032	17	515
0.028	129	4491	0.028	107	3757
0.047	59	1206	0.064	73	1060
0.065	161	2306	0.058	115	1862
0.14	64	392	0.229	106	356
0.018	15	804	0.029	22	735
0.031	47	1468	0.027	31	1127
0.026	84	3115	0.033	89	2572
0.04	35	846	0.055	39	664
0.038	74	1890	0.02	31	1549
0.377	496	818	0.47	574	646
0.03	73	2366	0.032	59	1786
0.019	29	1489	0.035	46	1275
0.034	77	2155	0.02	36	1792
0.054	148	2609	0.052	115	2088
0.028	46	1612	0.022	28	1249
0.023	60	2580	0.038	89	2253
0.135	162	1037	0.194	180	749
0.026	106	3931	0.032	106	3246
0.035	70	1928	0.018	31	1700
0.029	94	3118	0.025	66	2610
0.03	24	789	0.021	14	650
0.022	52	2263	0.019	35	1789
0.058	113	1844	0.062	102	1545
0.026	79	2916	0.026	67	2520
0.034	48	1374	0.031	39	1208

0.03	107	3423	0.029	93	3125
0.026	27	1007	0.019	17	869
0.026	40	1509	0.021	28	1301
0.026	72	2746	0.023	57	2450
0.023	67	2867	0.02	53	2592
0.023	16	673	0.033	19	565
0.015	13	864	0.024	17	704
0.019	46	2369	0.025	56	2142
0.049	76	1475	0.049	64	1253
0.03	82	2658	0.019	47	2372
0.078	72	856	0.12	89	652
0.024	64	2638	0.028	61	2135
0.031	124	3865	0.026	89	3336
0.023	41	1718	0.024	33	1341
0.019	87	4522	0.019	75	3931
0.034	30	840	0.024	18	730
0.027	92	3368	0.028	82	2809
0.027	71	2550	0.032	70	2148
0.019	24	1269	0.017	20	1168
0.028	63	2166	0.029	57	1928
0.024	80	3299	0.022	57	2582
0.029	61	2067	0.026	48	1820
0.021	58	2769	0.023	56	2393
0.022	59	2643	0.026	64	2354
0.019	36	1895	0.021	33	1505
0.025	117	4510	0.028	110	3792
0.025	75	2877	0.024	61	2488
0.023	46	1913	0.033	57	1654
0.036	83	2193	0.03	59	1904
0.018	34	1816	0.03	55	1753
0.033	55	1595	0.027	37	1315
0.025	109	4252	0.029	114	3796
0.024	43	1722	0.03	44	1412
0.06	87	1362	0.052	67	1223
0.019	61	3139	0.021	54	2535
0.085	38	407	0.07	27	356
0.032	28	837	0.046	30	617
0.029	38	1256	0.018	19	1009
0.184	173	766	0.258	218	626
0.028	135	4719	0.028	107	3736

0.026	36	1339	0.026	31	1172
0.028	84	2865	0.034	81	2309
0.022	25	1103	0.037	37	976
0.024	54	2193	0.027	54	1955
0.02	46	2218	0.023	42	1786
0.026	57	2129	0.023	43	1793
0.025	58	2222	0.019	36	1818
0.027	34	1220	0.028	30	1032
0.036	35	929	0.035	32	882
0.027	126	4618	0.027	117	4214
0.016	34	2089	0.03	52	1655
0.067	64	890	0.066	52	733
0.019	75	3887	0.024	83	3415
0.027	149	5396	0.028	130	4546
0.013	7	537	0.01	5	489
0.023	57	2416	0.023	45	1920
0.023	40	1707	0.029	43	1447
0.029	59	1979	0.033	66	1929
0.033	75	2226	0.039	74	1847
0.027	35	1247	0.012	13	1080
0.024	90	3606	0.027	89	3149
0.024	59	2386	0.017	36	2067
0.038	52	1314	0.045	57	1207
0.026	53	1971	0.025	40	1589
0.026	145	5455	0.024	122	4998
0.016	23	1457	0.035	47	1284
0.068	94	1293	0.05	62	1169
0.03	87	2793	0.024	57	2293
0.049	29	563	0.052	26	478
0.017	17	1007	0.029	26	857
0.02	75	3609	0.023	76	3191
0.141	146	887	0.218	232	833
0.024	54	2179	0.024	42	1732
0.044	55	1191	0.045	48	1023
0.018	64	3489	0.023	69	2977
0.016	14	837	0.024	17	684
0.027	66	2405	0.024	47	1926
0.021	80	3777	0.021	71	3310
0.023	92	3849	0.025	92	3526
0.02	53	2582	0.023	49	2084

0.033	33	980	0.021	19	871
0.033	39	1157	0.037	35	916
0.043	100	2216	0.04	81	1950
0.025	39	1540	0.025	31	1220
0.021	41	1932	0.027	49	1791
0.018	18	966	0.021	19	886
0.026	30	1114	0.032	35	1059
0.024	35	1397	0.019	24	1261
0.021	28	1275	0.029	32	1057
0.022	32	1426	0.022	28	1233
0.016	17	1042	0.025	22	843
0.024	212	8734	0.02	162	7859
0.022	65	2904	0.02	55	2639
0.018	5	266	0	0	237
0.025	97	3746	0.03	97	3189
0.027	90	3289	0.024	71	2868
0.054	90	1586	0.058	79	1278
0.025	48	1888	0.026	41	1508
0.027	32	1147	0.028	29	1005
0.02	21	1046	0.029	26	871
0.03	61	1978	0.021	39	1790
0.05	66	1245	0.061	68	1048
0.02	30	1453	0.037	41	1071
0.054	75	1326	0.04	49	1177
0.067	40	557	0.092	55	545
0.015	10	662	0.029	16	544
0.024	118	4866	0.022	93	4157
0.024	44	1752	0.029	40	1363
0.03	57	1835	0.033	49	1454
0.061	94	1457	0.098	146	1348
0.024	123	4977	0.025	117	4655
0.028	36	1245	0.029	31	1054
0.027	45	1604	0.021	29	1346
0.019	58	3075	0.031	83	2621
0.028	82	2797	0.021	53	2457
0.027	61	2224	0.026	50	1843
0.05	136	2577	0.052	122	2205
0.02	95	4569	0.027	109	3912
0.051	86	1591	0.045	65	1376
0.021	41	1918	0.025	35	1379

0.028	60	2064	0.029	52	1731
0.044	45	985	0.062	58	873
0.025	104	4040	0.031	106	3363
0.062	55	836	0.072	55	713
0.063	85	1264	0.044	50	1090
0.021	32	1466	0.04	50	1198
0.028	43	1515	0.023	27	1166
0.015	15	1010	0.03	29	945
0.033	36	1068	0.025	25	986
0.02	50	2471	0.027	60	2146
0.03	42	1338	0.021	23	1089
0.02	116	5575	0.024	117	4743
0.016	19	1185	0.02	19	951
0.016	10	635	0.014	8	578
0.024	62	2570	0.035	80	2174
0.021	39	1805	0.023	34	1454
0.067	90	1251	0.076	84	1026
0.016	13	819	0.02	13	651
0.035	83	2286	0.024	52	2122
0.027	72	2560	0.026	60	2222
0.049	24	467	0.028	9	318
0.024	29	1161	0.026	28	1070
0.023	23	996	0.027	23	832
0.027	26	933	0.02	16	780
0.024	58	2348	0.032	56	1707
0.028	39	1379	0.031	39	1224
0.021	41	1946	0.02	34	1626
0.025	53	2092	0.03	54	1766
0.031	62	1930	0.024	39	1599
0.03	51	1649	0.02	29	1436
0.034	21	590	0.031	15	474
0.02	37	1782	0.028	39	1360
0.063	43	644	0.064	38	558
0.049	104	2015	0.045	83	1759
0.025	55	2147	0.026	47	1793
0.062	193	2900	0.047	121	2473
0.031	28	866	0.018	13	704
0.028	37	1289	0.026	30	1140
0.068	36	493	0.104	44	378
0.029	31	1044	0.014	12	872

0.017	15	876	0.027	19	681
0.028	42	1432	0.023	27	1153
0.022	61	2744	0.019	44	2233
0.027	87	3177	0.024	65	2605
0.026	56	2073	0.029	54	1824
0.019	19	968	0.025	22	845
0.025	68	2602	0.014	32	2259
0.024	47	1909	0.017	26	1545
0.029	51	1724	0.016	26	1628
0.045	69	1459	0.044	60	1316
0.022	52	2358	0.026	55	2065
0.028	60	2112	0.02	37	1815
0.024	91	3692	0.035	105	2895
0.02	54	2601	0.016	40	2386
0.081	110	1256	0.093	104	1014
0.03	77	2519	0.028	62	2170
0.019	21	1096	0.024	23	936
0.026	22	819	0.024	16	641
0.064	161	2355	0.06	131	2041
0.068	97	1334	0.081	101	1144
0.018	23	1263	0.027	27	981
0.037	49	1263	0.034	33	942
0.027	23	820	0.032	25	754
0.028	73	2499	0.026	61	2279
0.03	33	1079	0.029	28	930
0.024	30	1223	0.022	23	1046
0.025	42	1641	0.026	33	1229
0.025	40	1559	0.028	37	1272
0.016	17	1052	0.017	17	964
0.048	83	1641	0.058	87	1407
0.015	19	1224	0.035	36	993
0.028	31	1069	0.039	36	897
0.02	25	1220	0.021	23	1094
0.064	167	2442	0.084	192	2093
0.026	49	1850	0.023	36	1544
0.072	47	606	0.094	54	523
0.028	74	2570	0.022	45	2034
0.104	165	1422	0.121	163	1188
0.028	47	1635	0.022	32	1411
0.022	64	2854	0.024	55	2268

0.024	84	3436	0.032	94	2831
0.024	47	1935	0.035	59	1647
0.246	363	1112	0.35	537	997
0.025	34	1349	0.021	25	1138
0.017	72	4278	0.028	97	3332
0.03	53	1694	0.029	44	1495
0.057	51	842	0.056	40	677
0.024	75	3052	0.031	91	2840
0.066	66	941	0.038	30	768
0.057	49	815	0.073	55	696
0.023	44	1874	0.028	43	1475
0.02	25	1216	0.032	33	1008
0.02	42	2032	0.025	47	1814
0.019	35	1841	0.019	30	1514
0.034	21	599	0.028	15	527
0.028	27	924	0.041	31	718
0.024	58	2374	0.022	47	2095
0.031	66	2056	0.025	44	1689
0.06	94	1463	0.039	50	1221
0.022	45	1980	0.017	29	1679
0.026	16	591	0.029	17	574
0.053	76	1359	0.053	58	1044
0.055	59	1017	0.051	41	765
0.061	243	3769	0.056	182	3062
0.02	38	1900	0.03	48	1568
0.026	43	1637	0.029	39	1328
0.084	70	765	0.1	74	667
0.026	135	5012	0.028	117	4051
0.025	25	969	0.026	22	816
0.019	86	4534	0.028	110	3843
0.021	63	2992	0.027	70	2566
0.025	87	3363	0.026	82	3104
0.02	101	4832	0.019	80	4220
0.024	48	1912	0.016	23	1408
0.028	41	1439	0.016	21	1256
0.088	110	1141	0.077	88	1058
0.034	35	992	0.026	24	907
0.032	36	1075	0.022	21	950
0.058	108	1739	0.054	86	1501
0.02	47	2253	0.033	62	1820

0.019	36	1885	0.025	41	1618
0.019	14	737	0.021	13	604
0.016	43	2701	0.021	47	2151
0.034	46	1310	0.019	23	1172
0.03	107	3470	0.025	71	2784
0.048	66	1299	0.059	74	1176
0.026	39	1472	0.027	36	1280
0.175	92	433	0.235	111	362
0.057	48	791	0.09	63	637
0.05	119	2244	0.066	119	1695
0.021	32	1492	0.023	30	1247
0.113	99	780	0.121	94	684
0.028	46	1606	0.031	47	1490
0.031	39	1209	0.024	25	1027
0.022	54	2402	0.027	58	2126
0.025	56	2213	0.029	55	1830
0.083	177	1957	0.116	197	1501
0.043	76	1687	0.058	88	1430
0.023	30	1281	0.035	38	1044
0.019	45	2346	0.029	58	1939
0.022	52	2359	0.036	74	2000
0.023	28	1214	0.023	25	1076
0.027	43	1555	0.03	37	1196
0.023	75	3156	0.021	55	2592
0.022	47	2068	0.029	50	1697
0.053	57	1027	0.064	56	818
0.02	37	1769	0.026	38	1415
0.031	59	1872	0.018	26	1396
0.056	93	1559	0.047	68	1370
0.024	69	2822	0.025	57	2264
0.027	41	1465	0.027	35	1281
0.024	27	1102	0.031	32	1016
0.022	36	1605	0.027	33	1204
0.031	64	2034	0.027	49	1768
0.076	86	1050	0.048	50	986
0.017	20	1155	0.048	50	994
0.033	89	2627	0.021	48	2261
0.04	76	1811	0.041	62	1439
0.074	43	535	0.073	38	480
0.276	241	632	0.435	355	462

0.064	82	1200	0.066	72	1016
0.024	91	3723	0.027	89	3251
0.021	63	2933	0.032	79	2381
0.035	51	1410	0.041	52	1207
0.023	118	4979	0.018	80	4314
0.018	37	2002	0.026	43	1620
0.021	21	968	0.015	13	833
0.018	13	721	0.02	12	577
0.024	41	1655	0.025	37	1446
0.021	105	4932	0.023	97	4185
0.048	54	1069	0.041	36	847
0.021	84	3876	0.021	67	3077
0.064	80	1165	0.048	47	939
0.066	68	958	0.065	55	796
0.028	18	636	0.035	20	548
0.031	113	3485	0.026	77	2895
0.369	305	522	0.449	319	391
0.044	44	961	0.066	51	717
0.024	96	3968	0.02	68	3406
0.023	45	1930	0.016	27	1626
0.06	30	467	0.048	19	380
0.025	50	1984	0.024	41	1661
0.029	87	2894	0.02	51	2543
0.09	64	648	0.11	58	467
0.023	124	5243	0.027	125	4431
0.018	27	1481	0.022	29	1304
0.024	30	1228	0.026	25	937
0.018	27	1438	0.022	32	1424
0.024	45	1848	0.03	48	1572
0.029	64	2173	0.025	50	1926
0.02	70	3356	0.019	51	2662
0.024	40	1600	0.032	41	1230
0.059	93	1478	0.048	68	1357
0.042	82	1864	0.025	46	1787
0.067	157	2173	0.06	119	1879
0.063	55	818	0.046	32	664
0.023	33	1429	0.027	36	1282
0.057	92	1529	0.056	77	1305
0.013	9	660	0.017	9	530
0.023	49	2106	0.023	43	1845

0.028	70	2451	0.025	57	2204
0.019	61	3077	0.034	93	2638
0.025	48	1876	0.016	25	1558
0.027	58	2117	0.028	48	1680
0.037	86	2208	0.033	65	1911
0.049	57	1110	0.071	66	860
0.066	57	804	0.05	36	679
0.019	21	1057	0.035	35	960
0.024	27	1102	0.04	38	908
0.026	36	1342	0.023	28	1174
0.015	18	1153	0.014	14	967
0.017	57	3335	0.034	98	2787
0.028	55	1915	0.029	50	1694
0.036	77	2088	0.029	52	1763
0.027	51	1843	0.028	48	1642
0.024	50	2032	0.02	34	1662
0.026	57	2148	0.028	53	1860
0.032	26	782	0.068	46	633
0.017	40	2354	0.021	38	1802
0.087	38	400	0.121	42	304
0.081	120	1363	0.071	85	1112
0.024	36	1477	0.027	36	1314
0.033	61	1784	0.028	46	1571
0.026	43	1635	0.027	38	1370
0.023	38	1616	0.021	30	1411
0.025	49	1924	0.032	51	1520
0.023	61	2603	0.029	67	2249
0.026	62	2334	0.026	50	1899
0.013	15	1162	0.032	30	913
0.053	46	821	0.05	36	686
0.022	14	627	0.035	19	526
0.024	27	1099	0.018	18	985
0.02	68	3363	0.02	62	3089
0.027	39	1428	0.025	31	1229
0.07	135	1791	0.052	81	1470
0.031	77	2441	0.035	81	2254
0.027	29	1047	0.028	24	831
0.024	56	2254	0.028	51	1769
0.023	42	1792	0.023	39	1691
0.026	80	2964	0.021	55	2570

0.021	50	2281	0.029	53	1803
0.038	46	1151	0.032	31	947
0.021	25	1193	0.027	30	1085
0.021	33	1513	0.025	31	1188
0.055	113	1928	0.075	124	1530
0.024	104	4254	0.024	89	3560
0.024	50	2048	0.028	54	1892
0.032	60	1795	0.02	32	1545
0.026	26	967	0.029	25	845
0.032	106	3191	0.027	71	2545
0.073	105	1342	0.068	79	1078
0.021	28	1307	0.035	39	1061
0.053	123	2196	0.051	105	1939
0.026	57	2166	0.033	57	1674
0.162	84	434	0.242	104	326
0.034	70	1999	0.027	47	1696
0.051	46	856	0.063	44	659
0.017	24	1420	0.027	33	1196
0.019	65	3328	0.02	55	2761
0.025	53	2099	0.028	48	1675
0.022	135	6037	0.025	130	5042
0.024	101	4133	0.025	96	3738
0.063	223	3343	0.075	220	2700
0.027	50	1829	0.028	46	1599
0.057	38	626	0.074	37	465
0.022	59	2641	0.018	36	2004
0.027	86	3155	0.03	84	2704
0.054	41	725	0.043	30	668
0.029	39	1311	0.02	22	1095
0.111	239	1910	0.118	203	1524
0.059	108	1711	0.056	84	1424
0.031	70	2213	0.029	54	1798
0.021	20	925	0.018	17	924
0.056	153	2601	0.074	166	2073
0.053	59	1054	0.091	84	839
0.027	84	3062	0.026	67	2485
0.029	41	1382	0.024	29	1169
0.015	15	987	0.042	31	700
0.059	101	1617	0.048	67	1329
0.023	35	1513	0.022	29	1297

0.023	55	2365	0.025	51	1975
0.02	63	3085	0.03	80	2631
0.026	87	3215	0.021	57	2623
0.028	109	3848	0.029	97	3305
0.032	86	2633	0.034	81	2290
0.024	103	4243	0.015	55	3625
0.019	108	5475	0.021	101	4613
0.042	46	1058	0.058	54	880
0.023	36	1535	0.026	35	1302
0.022	67	2918	0.019	49	2502
0.03	26	851	0.024	21	841
0.025	80	3093	0.027	76	2700
0.029	46	1522	0.032	45	1371
0.024	63	2614	0.022	49	2159
0.057	54	889	0.076	61	739
0.06	95	1490	0.056	77	1308
0.033	72	2111	0.024	40	1649
0.073	74	935	0.059	48	759
0.019	59	3102	0.021	53	2488
0.087	129	1353	0.101	152	1346
0.022	53	2366	0.028	53	1817
0.035	85	2330	0.026	52	1917
0.064	87	1272	0.068	80	1088
0.067	131	1838	0.063	102	1515
0.024	41	1679	0.024	38	1546
0.02	43	2134	0.031	59	1823
0.025	84	3287	0.023	66	2832
0.02	29	1392	0.029	33	1101
0.052	54	983	0.061	48	738
0.06	73	1149	0.039	36	892
0.023	53	2297	0.018	39	2102
0.028	69	2387	0.024	47	1928
0.018	39	2154	0.023	41	1757
0.037	19	495	0.021	10	459
0.022	22	960	0.041	30	698
0.022	53	2372	0.015	31	2080
0.022	82	3576	0.023	77	3254
0.033	57	1673	0.025	38	1472
0.028	78	2747	0.018	46	2476
0.023	30	1247	0.036	42	1129

0.028	68	2391	0.019	37	1940
0.022	45	2016	0.037	67	1745
0.062	233	3499	0.055	168	2886
0.061	143	2198	0.068	120	1657
0.026	23	862	0.026	20	742
0.053	188	3380	0.055	165	2820
0.022	74	3338	0.033	88	2604
0.019	47	2423	0.02	42	2051
0.084	77	840	0.107	88	737
0.027	88	3146	0.022	58	2637
0.025	88	3438	0.031	98	3062
0.065	116	1660	0.057	88	1458
0.015	19	1277	0.035	41	1145
0.02	43	2159	0.027	55	1998
0.033	58	1695	0.033	53	1557
0.019	33	1697	0.025	38	1466
0.032	38	1156	0.036	36	973
0.03	66	2166	0.022	43	1881
0.022	40	1753	0.018	27	1473
0.029	59	1980	0.025	42	1649
0.027	11	394	0.042	13	295
0.09	69	695	0.102	67	589
0.03	97	3153	0.035	87	2416
0.021	64	2982	0.024	61	2523
0.025	63	2410	0.025	55	2104
0.023	24	1036	0.025	22	846
0.031	91	2857	0.025	64	2450
0.031	45	1414	0.03	39	1252
0.024	59	2421	0.025	55	2104
0.054	150	2629	0.058	142	2309
0.028	38	1305	0.029	40	1328
0.025	39	1501	0.026	32	1187
0.021	84	3913	0.018	61	3267
0.027	25	913	0.018	14	756
0.019	57	2876	0.022	54	2445
0.07	88	1168	0.073	80	1011
0.026	95	3560	0.023	74	3150
0.027	35	1274	0.013	13	1009
0.024	27	1121	0.026	27	1013
0.059	131	2071	0.052	104	1882

0.034	66	1863	0.023	38	1609
0.031	46	1455	0.027	37	1332
0.068	118	1615	0.058	84	1360
0.05	108	2065	0.053	96	1725
0.029	103	3428	0.027	76	2720
0.031	75	2340	0.029	62	2096
0.03	37	1201	0.032	29	882
0.024	62	2562	0.025	60	2366
0.023	27	1144	0.023	23	972
0.034	40	1133	0.035	33	915
0.028	32	1095	0.021	22	1021
0.024	68	2766	0.019	44	2320
0.022	79	3457	0.027	75	2688
0.059	50	800	0.052	37	676
0.017	30	1748	0.016	24	1517
0.021	43	2018	0.025	44	1699
0.024	54	2176	0.027	54	1930
0.03	84	2761	0.032	79	2353
0.086	99	1057	0.125	122	855
0.029	83	2798	0.03	71	2272
0.023	37	1538	0.021	26	1190
0.025	54	2095	0.025	42	1661
0.04	68	1636	0.026	38	1439
0.063	116	1715	0.073	109	1393
0.029	91	3096	0.025	71	2821
0.057	139	2312	0.065	127	1833
0.033	79	2306	0.025	52	2060
0.022	54	2359	0.026	53	1964
0.022	19	852	0.033	26	758
0.02	51	2540	0.025	50	1922
0.023	45	1949	0.03	52	1664
0.024	31	1250	0.03	32	1032
0.018	64	3522	0.027	80	2925
0.03	26	848	0.027	21	745
0.022	98	4380	0.025	97	3768
0.025	58	2304	0.023	50	2080
0.021	67	3186	0.027	78	2842
0.065	99	1431	0.078	97	1153
0.027	27	967	0.021	17	775
0.027	51	1849	0.025	40	1547

0.094	176	1695	0.14	224	1371
0.025	43	1709	0.018	26	1390
0.019	21	1087	0.025	24	929
0.022	44	1983	0.026	46	1704
0.028	76	2608	0.032	70	2152
0.025	49	1881	0.028	44	1539
0.023	26	1113	0.029	28	922
0.028	22	759	0.018	14	771
0.026	83	3122	0.022	64	2860
0.027	48	1754	0.029	38	1286
0.027	51	1840	0.027	47	1665
0.02	19	931	0.018	15	814
0.033	68	1969	0.025	44	1723
0.028	43	1488	0.025	33	1271
0.026	48	1793	0.03	51	1677
0.024	64	2564	0.02	46	2275
0.054	106	1870	0.05	87	1647
0.061	111	1703	0.059	89	1408
0.027	47	1665	0.031	44	1376
0.08	67	775	0.086	69	730
0.612	907	575	0.671	1075	528
0.052	74	1360	0.069	81	1094
0.087	115	1212	0.08	92	1054
0.052	117	2124	0.043	76	1699
0.024	47	1886	0.033	58	1690
0.028	104	3583	0.021	64	2971
0.021	45	2150	0.022	42	1833
0.024	67	2714	0.027	66	2406
0.027	17	613	0.029	16	528
0.024	72	2927	0.023	67	2787
0.047	77	1557	0.049	68	1310
0.06	196	3062	0.06	165	2595
0.023	84	3638	0.029	92	3077
0.022	50	2254	0.025	50	1957
0.067	92	1280	0.066	69	979
0.028	60	2068	0.022	42	1892
0.019	44	2308	0.026	52	1965
0.021	62	2841	0.024	57	2344
0.033	83	2402	0.03	63	2011
0.024	45	1793	0.035	56	1553

0.022	26	1138	0.023	22	940
0.321	266	562	0.488	399	418
0.018	46	2453	0.024	50	2054
0.115	163	1255	0.181	203	919
0.047	42	861	0.063	52	771
0.041	47	1107	0.039	39	971
0.023	29	1227	0.031	32	1017
0.023	45	1937	0.025	44	1693
0.017	33	1950	0.026	44	1677
0.016	10	622	0.027	13	465
0.033	37	1076	0.034	33	926
0.074	57	709	0.073	50	639
0.025	57	2223	0.035	63	1738
0.033	130	3834	0.026	86	3161
0.027	67	2425	0.028	54	1875
0.025	55	2176	0.028	50	1713
0.022	69	3096	0.029	77	2544
0.022	25	1087	0.025	25	978
0.019	25	1264	0.036	35	936
0.026	26	970	0.027	24	858
0.102	68	600	0.113	62	488
0.027	43	1558	0.02	26	1270
0.05	54	1030	0.096	92	870
0.439	410	525	0.564	496	384
0.048	45	899	0.064	56	823
0.081	115	1306	0.112	145	1153
0.059	80	1274	0.057	68	1131
0.057	67	1112	0.054	52	915
0.025	18	716	0.042	24	553
0.019	31	1639	0.032	43	1301
0.022	39	1739	0.018	27	1491
0.12	151	1112	0.166	170	857
0.021	67	3150	0.031	87	2718
0.028	35	1198	0.024	25	1017
0.027	72	2582	0.02	43	2118
0.043	50	1117	0.052	55	998
0.024	37	1521	0.021	29	1358
0.017	19	1113	0.029	28	940
0.088	271	2805	0.124	332	2345
0.021	52	2416	0.019	43	2248

0.032	62	1867	0.032	54	1646
0.056	54	917	0.05	37	704
0.025	60	2362	0.024	55	2239
0.018	17	946	0.024	22	889
0.019	35	1826	0.027	41	1491
0.025	41	1580	0.029	36	1191
0.017	96	5402	0.019	92	4658
0.023	66	2848	0.022	56	2492
0.021	24	1134	0.029	29	986
0.025	47	1867	0.025	46	1779
0.344	212	405	0.517	344	322
0.06	156	2435	0.044	99	2136
0.065	97	1385	0.093	122	1189
0.047	43	877	0.036	29	777
0.033	71	2113	0.017	30	1697
0.03	45	1431	0.039	49	1208
0.027	44	1588	0.027	41	1477
0.018	25	1331	0.015	17	1092
0.022	36	1589	0.019	26	1351
0.017	19	1116	0.024	22	910
0.043	53	1167	0.051	50	929
0.024	73	2992	0.023	57	2388
0.027	45	1597	0.033	48	1406
0.023	33	1372	0.029	32	1088
0.026	41	1522	0.026	36	1336
0.035	59	1636	0.024	31	1263
0.025	59	2323	0.031	64	2028
0.048	121	2380	0.055	117	2013
0.059	100	1600	0.049	71	1368
0.022	41	1843	0.033	49	1447
0.016	26	1574	0.022	30	1306
0.025	69	2653	0.031	71	2217
0.028	38	1298	0.026	28	1033
0.027	86	3050	0.021	56	2575
0.028	57	1974	0.021	33	1515
0.021	98	4599	0.027	114	4125
0.02	26	1244	0.028	33	1134
0.02	32	1529	0.037	46	1210
0.022	42	1825	0.027	45	1601
0.027	27	987	0.022	18	787

0.014	11	783	0.048	34	670
0.024	23	935	0.027	23	840
0.051	114	2131	0.049	100	1951
0.02	33	1637	0.022	31	1388
0.071	171	2245	0.053	104	1853
0.029	92	3120	0.034	97	2772
0.052	73	1324	0.07	81	1075
0.021	43	2002	0.027	46	1638
0.071	102	1340	0.08	102	1181
0.034	41	1179	0.031	30	937
0.07	121	1608	0.051	79	1480
0.022	45	2019	0.022	41	1794
0.02	41	1960	0.022	38	1714
0.025	30	1187	0.028	29	991
0.017	34	2014	0.027	49	1739
0.028	38	1311	0.023	26	1089
0.057	98	1634	0.069	99	1340
0.028	221	7764	0.026	190	7052
0.016	60	3656	0.024	76	3077
0.027	57	2034	0.029	52	1769
0.023	47	1975	0.027	48	1754
0.026	35	1323	0.021	24	1103
0.073	147	1872	0.075	127	1571
0.041	65	1519	0.063	82	1219
0.026	37	1372	0.024	27	1101
0.016	46	2748	0.018	44	2385
0.024	48	1957	0.023	37	1574
0.025	69	2734	0.023	53	2277
0.024	23	923	0.017	14	788
0.024	98	3949	0.025	86	3343
0.031	44	1357	0.018	22	1171
0.02	32	1578	0.029	35	1181
0.026	63	2390	0.03	57	1863
0.204	129	502	0.384	192	308
0.031	93	2896	0.022	55	2418
0.083	151	1666	0.093	136	1324
0.081	137	1544	0.095	141	1336
0.03	47	1533	0.028	34	1189
0.036	63	1708	0.024	34	1377
0.034	53	1512	0.035	48	1337

0.023	73	3160	0.032	91	2780
0.049	153	2939	0.059	159	2525
0.03	49	1609	0.025	37	1436
0.021	71	3364	0.025	74	2879
0.023	50	2099	0.027	50	1803
0.078	111	1315	0.065	80	1142
0.019	83	4398	0.028	113	3900
0.068	44	599	0.069	35	471
0.021	58	2660	0.028	62	2129
0.062	193	2902	0.069	176	2369
0.024	100	4080	0.027	95	3469
0.024	80	3266	0.023	66	2757
0.016	28	1691	0.03	41	1348
0.024	47	1939	0.034	58	1660
0.019	44	2329	0.029	57	1914
0.03	91	2935	0.033	89	2576
0.059	172	2762	0.05	120	2264
0.029	28	940	0.027	21	769
0.053	63	1130	0.062	61	924
0.02	33	1650	0.023	33	1420
0.022	48	2115	0.022	37	1608
0.023	76	3287	0.023	72	3039
0.091	110	1101	0.07	73	970
0.03	46	1476	0.026	37	1367
0.019	35	1786	0.027	36	1288
0.026	59	2234	0.025	49	1931
0.031	24	756	0.023	14	592
0.104	82	705	0.114	81	631
0.02	24	1180	0.022	21	927
0.059	79	1261	0.059	61	968
0.025	111	4304	0.024	90	3664
0.022	74	3223	0.028	79	2745
0.027	56	1997	0.032	58	1772
0.029	30	998	0.021	17	782
0.155	319	1742	0.206	413	1596
0.051	68	1271	0.048	58	1142
0.054	123	2170	0.046	84	1733
0.024	87	3565	0.019	59	2982
0.024	47	1924	0.021	34	1590
0.031	44	1392	0.025	29	1144

0.023	57	2376	0.022	45	1993
0.021	47	2187	0.022	39	1732
0.049	110	2135	0.055	96	1656
0.022	47	2073	0.027	45	1597
0.054	153	2700	0.075	179	2209
0.028	36	1247	0.019	20	1057
0.014	23	1633	0.018	28	1550
0.064	48	704	0.074	50	622
0.028	102	3491	0.026	82	3045
0.015	38	2434	0.025	52	2002
0.025	66	2558	0.029	60	2022
0.026	29	1099	0.028	27	933
0.032	115	3518	0.03	95	3049
0.02	21	1023	0.028	22	754
0.029	52	1765	0.03	44	1412
0.053	35	631	0.053	32	574
0.024	29	1198	0.023	27	1138
0.023	78	3305	0.027	82	2985
0.067	64	895	0.056	45	760
0.019	41	2106	0.023	42	1761
0.023	63	2706	0.019	44	2269
0.016	21	1259	0.022	23	1040
0.028	62	2161	0.035	65	1789
0.022	36	1623	0.023	31	1325
0.029	34	1136	0.04	35	851
0.014	11	753	0.02	13	637
0.02	61	3031	0.019	51	2581
0.028	68	2356	0.028	54	1907
0.023	45	1889	0.031	54	1672
0.134	173	1117	0.112	116	922
0.027	42	1521	0.035	44	1210
0.024	64	2589	0.026	60	2209
0.021	45	2054	0.035	57	1563
0.023	60	2575	0.016	34	2137
0.06	30	466	0.067	27	378
0.019	31	1593	0.015	21	1350
0.106	111	936	0.163	152	783
0.022	119	5195	0.029	133	4434
0.019	65	3274	0.019	62	3118
0.611	1474	938	0.716	1781	705

0.024	49	1983	0.022	40	1793
0.025	22	844	0.017	13	760
0.024	48	1990	0.023	40	1715
0.026	39	1462	0.03	38	1219
0.027	39	1388	0.02	22	1084
0.026	107	4063	0.027	104	3684
0.02	79	3964	0.022	82	3654
0.027	29	1048	0.03	26	847
0.026	87	3267	0.022	55	2490
0.023	78	3310	0.023	65	2715
0.026	107	4033	0.028	97	3401
0.019	29	1513	0.035	45	1243
0.029	36	1186	0.037	37	965
0.021	34	1567	0.028	38	1330
0.026	106	3915	0.025	87	3362
0.021	39	1829	0.017	26	1512
0.08	80	915	0.055	48	819
0.024	36	1484	0.031	40	1266
0.037	33	848	0.022	17	766
0.024	34	1358	0.023	27	1138
0.022	103	4503	0.029	115	3878
0.055	137	2346	0.061	132	2027
0.051	51	945	0.046	37	763
0.02	23	1114	0.025	23	895
0.041	99	2300	0.03	64	2076
0.026	42	1596	0.029	40	1316
0.073	91	1151	0.066	66	933
0.03	21	672	0.029	17	572
0.018	32	1712	0.029	46	1523
0.022	39	1769	0.022	34	1504
0.077	63	751	0.12	75	552
0.061	101	1543	0.05	66	1257
0.024	32	1296	0.025	27	1067
0.02	24	1171	0.017	16	937
0.059	65	1030	0.068	65	893
0.039	28	686	0.059	32	506
0.087	51	533	0.099	48	438
0.058	61	988	0.04	30	712
0.067	81	1126	0.062	65	978
0.062	201	3024	0.057	154	2533

0.017	31	1790	0.031	49	1547
0.023	44	1871	0.019	29	1518
0.027	62	2256	0.025	50	1977
0.017	19	1086	0.017	16	906
0.03	54	1740	0.036	54	1433
0.107	92	765	0.131	85	565
0.038	76	1936	0.034	55	1544
0.018	20	1093	0.029	29	960
0.012	23	1876	0.032	53	1628
0.026	44	1619	0.039	54	1337
0.022	59	2644	0.024	54	2225
0.063	121	1810	0.054	86	1510
0.02	70	3440	0.021	63	2963
0.025	47	1866	0.021	35	1602
0.025	50	1983	0.02	33	1605
0.025	25	969	0.032	26	783
0.021	54	2469	0.024	59	2409
0.065	81	1173	0.065	63	910
0.092	160	1579	0.126	182	1257
0.026	76	2823	0.027	68	2409
0.021	11	524	0.014	6	437
0.058	143	2332	0.055	115	1995
0.022	32	1393	0.023	27	1145
0.022	33	1447	0.031	38	1194
0.058	174	2843	0.06	159	2492
0.021	58	2728	0.019	41	2142
0.026	56	2068	0.027	48	1715
0.108	73	606	0.138	79	495
0.024	32	1303	0.026	26	964
0.019	34	1774	0.023	38	1587
0.026	33	1254	0.029	31	1033
0.024	51	2115	0.035	61	1697
0.024	62	2558	0.023	55	2307
0.018	26	1413	0.023	29	1208
0.023	47	1982	0.025	39	1550
0.054	61	1071	0.058	55	888
0.095	244	2323	0.107	237	1985
0.02	80	3983	0.014	47	3230
0.033	45	1299	0.04	47	1142
0.023	34	1424	0.025	29	1132

0.029	79	2658	0.03	69	2244
0.042	60	1364	0.022	25	1108
0.081	199	2271	0.098	215	1984
0.104	116	1001	0.126	120	834
0.054	176	3079	0.065	177	2538
0.018	60	3197	0.022	62	2819
0.023	97	4098	0.029	97	3249
0.031	34	1079	0.033	34	981
0.021	35	1596	0.022	32	1452
0.032	49	1500	0.024	34	1378
0.034	64	1818	0.024	37	1488
0.055	48	820	0.084	62	678
0.028	25	877	0.023	19	819
0.026	71	2669	0.022	55	2429
0.026	49	1808	0.023	37	1589
0.025	95	3661	0.024	75	3064
0.15	134	760	0.233	179	589
0.019	29	1499	0.015	18	1184
0.021	33	1574	0.024	32	1328
0.018	44	2375	0.026	53	2019
0.026	62	2327	0.027	56	2017
0.031	41	1266	0.031	40	1249
0.021	88	4024	0.024	86	3510
0.015	10	666	0.034	20	570
0.028	29	1025	0.021	19	890
0.025	88	3501	0.024	77	3174
0.027	60	2140	0.024	46	1846
0.033	52	1503	0.026	33	1235
0.027	108	3926	0.021	71	3391
0.031	55	1737	0.028	39	1366
0.023	27	1131	0.022	25	1103
0.025	59	2256	0.019	41	2148
0.024	48	1992	0.023	41	1707
0.075	71	871	0.108	87	717
0.025	88	3390	0.031	93	2939
0.018	24	1289	0.023	27	1123
0.05	127	2407	0.06	121	1903
0.02	45	2157	0.019	36	1864
0.02	26	1267	0.023	23	985
0.027	40	1462	0.033	40	1190

0.089	63	648	0.081	50	566
0.02	40	1923	0.031	52	1605
0.024	22	909	0.017	15	849
0.021	45	2062	0.029	58	1938
0.078	135	1600	0.063	87	1284
0.412	329	470	0.526	408	367
0.066	136	1939	0.047	83	1670
0.201	257	1023	0.231	255	847
0.019	36	1861	0.024	36	1479
0.062	67	1009	0.069	59	795
0.065	157	2263	0.06	121	1908
0.054	81	1427	0.066	78	1099
0.024	41	1696	0.023	33	1386
0.019	18	950	0.025	21	829
0.078	58	686	0.083	59	654
0.029	58	1915	0.023	40	1692
0.032	48	1445	0.021	25	1174
0.024	55	2220	0.028	55	1897
0.028	87	3040	0.033	77	2270
0.026	55	2092	0.02	37	1859
0.024	20	814	0.028	18	624
0.027	44	1590	0.029	37	1230
0.052	126	2279	0.055	114	1973
0.024	68	2791	0.029	67	2274
0.029	34	1147	0.025	24	934
0.016	29	1752	0.021	30	1392
0.019	90	4720	0.027	109	3999
0.021	58	2756	0.03	74	2374
0.019	22	1136	0.018	17	910
0.047	87	1764	0.044	68	1487
0.028	49	1719	0.022	31	1369
0.026	46	1708	0.034	49	1373
0.028	90	3152	0.032	83	2550
0.02	33	1640	0.027	41	1487
0.019	30	1566	0.028	37	1289
0.056	146	2440	0.041	89	2086
0.132	89	585	0.172	89	427
0.054	204	3595	0.054	168	2932
0.023	46	1950	0.034	54	1535
0.049	52	1020	0.059	57	904

0.051	59	1106	0.048	44	874
0.023	25	1057	0.03	27	872
0.07	160	2112	0.092	169	1660
0.373	411	690	0.463	497	576
0.025	146	5813	0.026	132	4863
0.047	65	1313	0.054	60	1048
0.052	158	2885	0.06	148	2334
0.078	120	1419	0.047	58	1169
0.019	20	1049	0.027	24	861
0.022	64	2811	0.029	74	2521
0.019	21	1102	0.012	11	901
0.358	667	1194	0.451	832	1012
0.02	13	647	0.012	8	640
0.027	59	2132	0.029	54	1829
0.025	21	814	0.022	15	656
0.055	91	1555	0.065	88	1267
0.042	43	987	0.055	45	778
0.026	15	557	0.027	12	434
0.067	100	1399	0.057	74	1222
0.026	36	1334	0.02	23	1127
0.053	90	1623	0.068	96	1310
0.028	107	3725	0.028	90	3140
0.021	14	662	0.017	8	469
0.016	33	2060	0.023	39	1641
0.03	59	1887	0.024	41	1673
0.025	42	1672	0.033	50	1447
0.019	104	5242	0.021	98	4670
0.078	53	623	0.079	45	526
0.026	42	1591	0.029	42	1389
0.053	44	785	0.058	42	676
0.064	104	1514	0.066	87	1231
0.024	108	4317	0.026	101	3780
0.019	13	682	0.04	24	571
0.006	4	665	0.026	15	573
0.032	98	2974	0.02	55	2730
0.033	55	1587	0.027	34	1227
0.027	48	1758	0.027	42	1527
0.027	69	2475	0.032	68	2028
0.025	80	3126	0.029	76	2539
0.024	38	1525	0.034	37	1063

0.248	261	791	0.334	318	633
0.019	29	1532	0.026	33	1242
0.079	139	1611	0.076	117	1414
0.021	52	2476	0.037	76	1991
0.028	103	3622	0.023	69	2993
0.079	80	931	0.075	75	919
0.081	69	786	0.067	50	691
0.025	55	2142	0.026	50	1842
0.112	91	725	0.165	119	603
0.051	66	1225	0.054	61	1059
0.026	49	1814	0.02	29	1422
0.433	368	481	0.538	454	390
0.025	67	2657	0.021	48	2251
0.019	50	2625	0.025	61	2391
0.028	72	2495	0.025	51	1977
0.026	55	2064	0.031	57	1778
0.028	60	2051	0.036	68	1838
0.025	66	2565	0.031	65	2027
0.03	87	2782	0.026	59	2239
0.071	68	886	0.07	55	728
0.032	77	2362	0.028	56	1946
0.019	9	476	0.031	14	431
0.027	71	2528	0.02	46	2207
0.022	102	4488	0.025	99	3843
0.121	43	311	0.13	39	261
0.023	20	854	0.029	26	871
0.019	79	4133	0.019	74	3817
0.021	27	1237	0.024	24	980
0.024	44	1768	0.042	65	1490
0.023	57	2425	0.036	74	1986
0.022	32	1399	0.023	27	1147
0.025	150	5821	0.026	133	4962
0.053	84	1504	0.042	57	1294
0.022	44	1923	0.021	36	1657
0.019	24	1258	0.025	31	1185
0.025	92	3549	0.026	82	3129
0.039	69	1700	0.028	44	1507
0.028	62	2164	0.026	47	1758
0.058	114	1839	0.058	92	1484
0.021	36	1647	0.034	49	1375

0.023	42	1756	0.033	58	1707
0.024	53	2201	0.03	63	2019
0.076	85	1040	0.065	63	912
0.024	71	2834	0.023	54	2344
0.03	69	2254	0.022	43	1875
0.032	44	1332	0.028	37	1283
0.02	57	2821	0.031	71	2227
0.049	35	676	0.064	44	642
0.022	30	1321	0.033	36	1069
0.039	117	2905	0.029	78	2650
0.026	46	1718	0.033	49	1429
0.031	30	927	0.027	20	725
0.255	356	1040	0.321	461	977
0.024	48	1979	0.027	46	1686
0.018	44	2446	0.028	57	2002
0.029	81	2742	0.03	73	2389
0.054	97	1706	0.059	87	1395
0.022	14	636	0.026	17	636
0.07	132	1750	0.057	92	1534
0.024	22	887	0.029	22	748
0.028	40	1414	0.021	23	1090
0.053	85	1526	0.048	63	1237
0.063	51	755	0.081	51	577
0.02	29	1437	0.023	29	1251
0.023	58	2423	0.025	55	2129
0.025	43	1660	0.014	21	1471
0.024	120	4955	0.022	95	4132
0.024	13	534	0.007	3	419
0.055	65	1117	0.068	71	966
0.029	68	2269	0.03	61	1998
0.021	48	2293	0.014	26	1870
0.02	15	742	0.03	22	708
0.016	39	2365	0.028	57	1984
0.03	113	3604	0.025	80	3184
0.024	44	1757	0.021	33	1523
0.028	51	1776	0.032	52	1553
0.021	59	2812	0.023	56	2333
0.022	75	3285	0.021	60	2745
0.058	75	1209	0.063	65	965
0.048	56	1122	0.051	54	996

0.027	109	3856	0.023	82	3486
0.031	71	2249	0.03	62	2039
0.086	98	1042	0.108	97	805
0.021	29	1332	0.025	28	1076
0.018	43	2314	0.026	56	2132
0.02	32	1606	0.026	37	1365
0.022	24	1073	0.019	18	914
0.032	65	1981	0.044	77	1669
0.021	42	1934	0.023	38	1611
0.026	60	2271	0.042	78	1766
0.022	74	3228	0.021	61	2844
0.03	79	2523	0.026	61	2280
0.062	92	1389	0.06	77	1208
0.021	177	8261	0.021	155	7214
0.03	125	4000	0.029	97	3277
0.055	46	783	0.068	43	591
0.051	54	1014	0.051	43	803
0.022	14	627	0.03	17	558
0.028	48	1693	0.026	34	1295
0.047	111	2238	0.049	99	1930
0.05	81	1546	0.051	68	1267
0.023	61	2581	0.032	66	2025
0.067	115	1598	0.063	92	1380
0.028	63	2194	0.018	35	1936
0.02	51	2558	0.019	42	2158
0.026	24	914	0.014	12	863
0.023	37	1580	0.026	34	1297
0.027	82	2920	0.032	81	2451
0.019	48	2504	0.025	53	2064
0.033	62	1826	0.034	46	1313
0.021	32	1471	0.031	40	1243
0.027	49	1735	0.034	47	1345
0.021	17	785	0.024	18	717
0.05	142	2705	0.078	182	2150
0.025	106	4074	0.019	65	3270
0.114	139	1081	0.165	152	767
0.052	103	1859	0.054	86	1507
0.018	36	1996	0.022	38	1663
0.064	65	949	0.07	54	718
0.022	47	2069	0.019	36	1834

0.086	108	1151	0.064	72	1051
0.023	22	919	0.021	16	743
0.028	101	3492	0.027	84	3042
0.036	28	755	0.022	15	655
0.02	38	1834	0.03	45	1453
0.04	81	1956	0.038	68	1737
0.018	48	2567	0.019	46	2354
0.016	10	610	0.028	14	482
0.015	30	1955	0.022	38	1728
0.03	33	1083	0.02	20	984
0.024	18	746	0.019	11	579
0.024	59	2440	0.027	54	1976
0.041	36	833	0.055	43	735
0.026	79	2966	0.023	59	2548
0.025	34	1344	0.032	40	1202
0.02	16	782	0.029	19	646
0.025	61	2336	0.032	60	1825
0.024	39	1590	0.03	43	1387
0.225	197	678	0.308	249	560
0.021	33	1553	0.02	27	1349
0.026	81	3037	0.026	72	2649
0.021	28	1325	0.031	37	1138
0.024	22	878	0.032	25	753
0.026	84	3112	0.03	80	2621
0.022	40	1739	0.028	43	1512
0.639	450	254	0.678	486	231
0.032	27	829	0.016	11	692
0.022	55	2469	0.022	46	2060
0.076	30	365	0.059	19	301
0.067	138	1927	0.058	94	1526
0.027	60	2139	0.021	39	1845
0.025	31	1203	0.02	20	977
0.021	27	1230	0.034	40	1151
0.02	29	1425	0.022	27	1228
0.028	34	1198	0.032	34	1044
0.023	39	1651	0.033	45	1307
0.062	114	1729	0.063	87	1300
0.031	54	1712	0.021	30	1399
0.027	99	3513	0.02	60	2954
0.035	39	1082	0.031	30	929

0.016	22	1368	0.023	26	1091
0.023	39	1654	0.029	49	1662
0.022	9	392	0.044	16	344
0.032	86	2621	0.015	30	1975
0.027	20	711	0.018	11	595
0.064	82	1204	0.081	84	953
0.337	403	793	0.465	658	756
0.031	51	1608	0.027	39	1383
0.022	54	2442	0.023	48	2080
0.032	50	1501	0.034	44	1263
0.028	53	1874	0.033	51	1497
0.027	37	1314	0.027	29	1050
0.027	33	1183	0.026	25	945
0.078	56	664	0.053	35	626
0.018	19	1020	0.023	22	923
0.02	34	1671	0.029	40	1326
0.136	248	1579	0.185	314	1382
0.063	47	695	0.075	47	578
0.031	61	1878	0.023	37	1550
0.096	41	386	0.12	42	307
0.02	30	1457	0.017	23	1329
0.011	10	911	0.024	18	733
0.021	56	2557	0.024	53	2198
0.088	75	775	0.102	72	633
0.026	39	1488	0.026	34	1286
0.029	108	3668	0.032	97	2891
0.05	40	764	0.065	44	633
0.017	38	2234	0.018	34	1897
0.024	136	5650	0.026	127	4703
0.058	111	1809	0.072	124	1588
0.018	8	432	0.034	14	395
0.018	40	2137	0.025	49	1922
0.027	56	2013	0.021	36	1639
0.029	28	924	0.038	33	841
0.025	79	3094	0.026	70	2655
0.02	14	680	0.03	19	607
0.117	249	1871	0.141	254	1545
0.025	65	2500	0.028	53	1852
0.07	91	1215	0.068	68	938
0.064	137	1989	0.064	116	1684

0.326	452	933	0.471	698	785
0.081	55	623	0.108	58	480
0.02	39	1912	0.019	28	1475
0.063	118	1741	0.044	70	1536
0.027	51	1842	0.026	40	1513
0.046	69	1444	0.042	47	1084
0.028	65	2269	0.032	60	1807
0.061	82	1269	0.069	70	947
0.024	62	2488	0.031	64	1981
0.066	45	639	0.055	29	494
0.06	124	1927	0.074	117	1473
0.031	108	3432	0.027	82	2939
0.025	86	3311	0.021	60	2758
0.029	86	2859	0.022	55	2450
0.022	29	1313	0.043	49	1092
0.018	27	1471	0.022	27	1211
0.101	169	1498	0.16	233	1224
0.033	45	1299	0.025	29	1132
0.025	34	1315	0.026	29	1090
0.024	37	1528	0.037	49	1280
0.019	28	1446	0.022	29	1286
0.029	67	2251	0.032	61	1875
0.07	183	2435	0.096	206	1937
0.025	48	1878	0.021	35	1606
0.019	49	2550	0.026	59	2184
0.025	20	796	0.015	11	711
0.102	65	574	0.151	90	505
0.062	27	406	0.073	27	341
0.021	22	1034	0.023	21	909
0.083	95	1052	0.105	107	915
0.019	53	2752	0.023	55	2333
0.02	23	1100	0.039	33	823
0.02	36	1734	0.042	67	1544
0.019	25	1299	0.029	33	1116
0.049	54	1037	0.084	88	959
0.021	39	1823	0.017	28	1642
0.022	97	4285	0.026	93	3525
0.053	104	1872	0.055	91	1575
0.055	75	1288	0.052	57	1031
0.026	42	1585	0.028	37	1293

0.036	53	1415	0.035	44	1228
0.027	80	2933	0.028	69	2379
0.03	42	1345	0.027	32	1133
0.025	38	1505	0.022	28	1231
0.02	40	1948	0.033	54	1603
0.028	20	703	0.036	24	645
0.126	63	436	0.258	119	343
0.062	70	1065	0.052	51	923
0.04	16	385	0.027	10	362
0.017	31	1771	0.029	45	1496
0.028	33	1138	0.038	38	973
0.019	25	1293	0.023	25	1060
0.023	137	5814	0.026	140	5169
0.024	88	3578	0.023	67	2909
0.028	100	3434	0.03	95	3096
0.022	28	1254	0.034	35	1005
0.026	50	1909	0.029	51	1691
0.021	114	5240	0.025	113	4409
0.018	12	654	0.034	21	589
0.027	98	3530	0.022	72	3193
0.023	30	1265	0.025	30	1152
0.064	88	1293	0.046	52	1068
0.022	50	2231	0.037	69	1802
0.032	128	3871	0.032	110	3367
0.026	52	1938	0.029	51	1697
0.121	83	603	0.138	75	470
0.017	50	2862	0.025	62	2416
0.095	52	493	0.14	72	444
0.02	42	2110	0.025	46	1765
0.025	68	2606	0.034	72	2044
0.029	31	1054	0.039	37	901
0.026	61	2242	0.034	67	1889
0.017	31	1747	0.024	38	1549
0.072	113	1461	0.099	126	1142
0.027	71	2516	0.031	71	2200
0.028	140	4851	0.025	107	4205
0.057	69	1140	0.05	50	959
0.024	117	4806	0.016	69	4350
0.016	22	1367	0.038	40	1006
0.033	38	1100	0.035	37	1020

0.033	74	2148	0.021	40	1842
0.025	49	1909	0.025	38	1512
0.038	102	2577	0.034	77	2207
0.025	26	995	0.016	13	798
0.018	27	1478	0.023	29	1221
0.011	8	708	0.018	10	536
0.052	107	1953	0.046	77	1595
0.051	135	2509	0.053	123	2178
0.021	92	4307	0.023	86	3683
0.032	40	1217	0.016	18	1132
0.027	32	1136	0.018	18	959
0.023	30	1301	0.023	23	975
0.042	88	2032	0.048	87	1717
0.059	133	2123	0.046	92	1887
0.05	49	923	0.075	61	753
0.074	79	994	0.063	55	820
0.022	49	2218	0.024	47	1881
0.035	36	992	0.028	21	728
0.028	66	2297	0.024	47	1927
0.047	81	1647	0.063	91	1359
0.023	46	1949	0.03	51	1643
0.021	50	2369	0.025	53	2057
0.024	52	2076	0.019	36	1845
0.023	88	3742	0.023	72	3118
0.046	131	2745	0.054	126	2190
0.035	48	1325	0.034	37	1047
0.026	89	3324	0.028	81	2766
0.033	46	1356	0.037	46	1191
0.022	59	2641	0.028	66	2291
0.026	107	4044	0.02	66	3220
0.022	58	2622	0.03	66	2165
0.029	35	1184	0.02	21	1051
0.021	53	2479	0.032	65	1979
0.021	10	462	0.019	7	355
0.088	73	754	0.079	60	701
0.021	27	1285	0.026	29	1093
0.083	150	1665	0.083	125	1386
0.079	151	1751	0.065	104	1504
0.175	110	519	0.24	138	437
0.027	46	1688	0.02	29	1431

0.028	52	1835	0.035	57	1574
0.025	120	4655	0.024	95	3917
0.018	18	991	0.018	16	852
0.049	77	1498	0.052	72	1311
0.018	16	865	0.01	7	694
0.019	29	1463	0.022	29	1272
0.024	62	2525	0.023	54	2288
0.116	74	564	0.141	66	401
0.025	20	786	0.016	11	694
0.067	48	671	0.056	31	524
0.058	149	2406	0.076	157	1897
0.047	43	873	0.124	96	676
0.057	98	1616	0.05	69	1309
0.029	13	428	0.022	8	357
0.02	59	2937	0.032	74	2267
0.058	145	2353	0.061	127	1947
0.022	41	1842	0.027	45	1596
0.031	24	754	0.026	16	590
0.446	666	827	0.571	924	694
0.017	28	1573	0.022	30	1348
0.112	108	855	0.117	91	685
0.023	44	1867	0.021	35	1669
0.06	67	1044	0.05	48	920
0.018	76	4208	0.027	96	3399
0.072	118	1516	0.054	75	1319
0.026	54	2047	0.023	46	1931
0.022	52	2314	0.029	61	2063
0.054	66	1153	0.053	56	1001
0.06	98	1542	0.061	80	1237
0.026	26	973	0.036	31	830
0.03	45	1436	0.029	37	1238
0.017	9	525	0.016	7	441
0.103	73	638	0.125	74	516
0.052	115	2077	0.057	103	1702
0.021	47	2149	0.023	42	1792
0.022	73	3178	0.022	61	2709
0.034	60	1684	0.037	57	1487
0.089	108	1106	0.108	115	946
0.025	100	3957	0.027	88	3220
0.022	22	956	0.029	24	799

0.028	55	1877	0.024	36	1437
0.036	49	1329	0.029	32	1067
0.021	126	5904	0.022	112	5079
0.028	27	928	0.03	25	819
0.027	53	1944	0.018	27	1504
0.03	32	1032	0.031	27	856
0.033	60	1741	0.026	37	1388
0.017	29	1695	0.034	51	1445
0.018	65	3492	0.027	82	2928
0.034	59	1674	0.036	52	1394
0.019	40	2038	0.031	55	1731
0.027	97	3476	0.023	73	3081
0.034	45	1298	0.031	36	1131
0.177	162	755	0.165	116	587
0.347	612	1152	0.432	733	963
0.017	19	1115	0.028	29	1016
0.023	55	2292	0.033	65	1879
0.018	37	2036	0.021	37	1724
0.025	31	1211	0.018	18	994
0.029	48	1584	0.033	44	1300
0.051	66	1224	0.047	54	1085
0.06	68	1057	0.044	40	871
0.029	44	1482	0.031	39	1228
0.047	104	2132	0.055	95	1626
0.021	23	1062	0.02	17	849
0.025	51	2004	0.036	65	1737
0.023	88	3796	0.023	77	3324
0.025	48	1851	0.026	43	1605
0.065	65	937	0.083	68	755
0.02	31	1503	0.022	29	1292
0.02	59	2822	0.023	62	2642
0.054	50	868	0.049	36	693
0.016	28	1764	0.02	27	1292
0.022	29	1303	0.024	28	1139
0.018	34	1875	0.021	33	1518
0.016	16	974	0.043	32	720
0.029	64	2134	0.023	43	1823
0.021	80	3674	0.022	77	3348
0.018	25	1387	0.032	40	1203
0.033	68	1992	0.025	40	1588

0.025	51	1950	0.025	44	1747
0.028	72	2525	0.019	47	2408
0.028	101	3448	0.033	96	2839
0.023	64	2672	0.022	51	2263
0.027	49	1793	0.023	35	1478
0.055	77	1334	0.062	74	1126
0.031	91	2806	0.02	47	2282
0.017	21	1185	0.022	22	959
0.029	47	1588	0.034	42	1196
0.028	77	2722	0.03	70	2247
0.022	129	5628	0.029	149	4957
0.025	38	1495	0.028	35	1229
0.027	57	2091	0.024	43	1727
0.02	55	2631	0.022	50	2210
0.023	44	1847	0.031	44	1390
0.03	47	1494	0.031	41	1283
0.023	17	731	0.03	19	611
0.024	65	2672	0.033	76	2215
0.031	36	1123	0.029	29	961
0.02	28	1389	0.03	36	1153
0.025	27	1041	0.029	25	823
0.045	49	1038	0.05	43	813
0.022	102	4625	0.018	69	3805
0.026	81	3013	0.028	76	2605
0.024	33	1358	0.033	44	1304
0.026	61	2328	0.021	44	2017
0.019	31	1578	0.024	33	1323
0.052	127	2332	0.051	108	2019
0.023	84	3630	0.031	101	3119
0.027	73	2595	0.024	49	1957
0.018	85	4540	0.02	79	3779
0.024	56	2276	0.029	60	2006
0.054	180	3144	0.076	196	2383
0.023	32	1366	0.015	16	1037
0.055	112	1932	0.044	72	1574
0.029	34	1120	0.051	52	966
0.023	33	1372	0.023	25	1057
0.029	39	1327	0.036	45	1210
0.044	68	1463	0.052	67	1226
0.024	89	3664	0.02	61	2984

0.02	26	1275	0.022	25	1092
0.025	175	6900	0.025	156	6024
0.03	120	3910	0.029	98	3299
0.019	25	1297	0.03	34	1090
0.025	25	964	0.031	26	814
0.02	56	2683	0.02	43	2153
0.054	62	1094	0.053	51	907
0.02	47	2291	0.027	51	1832
0.019	69	3614	0.032	101	3043
0.029	80	2699	0.025	68	2641
0.017	20	1173	0.025	25	987
0.025	56	2153	0.018	34	1838
0.07	105	1400	0.06	77	1198
0.025	58	2251	0.024	48	1942
0.023	31	1303	0.031	32	990
0.021	25	1163	0.034	36	1021
0.021	24	1142	0.031	29	917
0.024	46	1844	0.03	54	1724
0.022	42	1846	0.035	59	1635
0.027	51	1873	0.029	46	1527
0.028	23	809	0.026	20	735
0.064	71	1030	0.067	56	785
0.018	17	907	0.039	31	760
0.028	44	1504	0.026	36	1328
0.033	47	1359	0.025	29	1112
0.026	57	2142	0.025	49	1884
0.029	61	2063	0.034	61	1725
0.065	156	2230	0.063	117	1752
0.067	104	1450	0.071	93	1211
0.06	52	812	0.051	37	692
0.022	33	1455	0.029	36	1205
0.071	177	2328	0.064	139	2031
0.028	32	1097	0.033	34	1009
0.056	247	4196	0.056	196	3296
0.027	67	2390	0.02	46	2282
0.039	51	1267	0.027	30	1090
0.021	44	2052	0.025	44	1703
0.019	46	2439	0.02	40	1939
0.018	21	1131	0.029	28	931
0.062	40	603	0.056	31	525

0.058	242	3900	0.051	168	3122
0.029	46	1539	0.025	33	1289
0.027	133	4879	0.026	116	4294
0.022	39	1738	0.029	46	1554
0.032	38	1145	0.022	22	997
0.027	59	2130	0.031	57	1800
0.033	56	1617	0.025	35	1373
0.033	72	2098	0.02	39	1870
0.359	240	429	0.502	372	369
0.024	61	2486	0.02	43	2126
0.036	49	1327	0.026	30	1104
0.02	52	2542	0.028	61	2124
0.028	55	1897	0.021	34	1622
0.028	33	1147	0.026	25	948
0.024	38	1522	0.032	39	1168
0.022	35	1556	0.031	44	1375
0.026	106	3977	0.021	76	3527
0.022	54	2412	0.02	42	2050
0.026	57	2112	0.026	47	1768
0.022	74	3269	0.028	80	2748
0.033	62	1798	0.036	56	1496
0.02	20	965	0.026	21	781
0.015	29	1942	0.026	45	1664
0.065	135	1929	0.047	84	1696
0.027	33	1192	0.031	30	940
0.039	41	1003	0.05	44	835
0.016	68	4297	0.019	72	3724
0.067	60	831	0.065	52	747
0.03	50	1623	0.019	28	1464
0.025	130	5089	0.025	115	4453
0.062	87	1309	0.054	65	1133
0.019	12	635	0.022	11	498
0.032	46	1384	0.032	42	1284
0.021	62	2828	0.025	62	2414
0.02	28	1378	0.026	34	1253
0.077	73	876	0.078	59	694
0.138	95	594	0.171	107	517
0.031	31	955	0.019	14	739
0.018	19	1054	0.017	13	773
0.021	66	3042	0.027	75	2690

0.131	114	755	0.127	90	619
0.029	50	1704	0.024	37	1515
0.023	107	4519	0.019	77	3951
0.022	48	2128	0.019	34	1724
0.024	55	2258	0.018	41	2257
0.026	33	1255	0.033	34	997
0.024	40	1632	0.025	31	1204
0.031	66	2045	0.021	39	1797
0.021	79	3663	0.023	75	3153
0.054	145	2536	0.038	83	2080
0.026	104	3918	0.025	89	3506
0.049	57	1112	0.057	54	899
0.023	74	3116	0.027	72	2637
0.023	33	1376	0.027	34	1218
0.025	115	4455	0.023	90	3825
0.028	52	1812	0.025	39	1500
0.022	13	566	0.034	19	535
0.08	70	807	0.092	70	689
0.029	99	3326	0.022	63	2823
0.024	51	2118	0.031	54	1709
0.026	52	1944	0.018	33	1775
0.11	162	1314	0.136	150	955
0.023	47	2022	0.015	26	1754
0.029	56	1908	0.04	65	1560
0.021	33	1563	0.032	42	1255
0.028	58	1987	0.024	42	1695
0.02	55	2683	0.027	62	2256
0.024	32	1293	0.024	29	1205
0.026	47	1787	0.033	54	1587
0.022	35	1567	0.024	34	1395
0.025	105	4046	0.023	80	3459
0.024	37	1531	0.018	23	1271
0.024	95	3859	0.027	86	3050
0.027	138	4921	0.026	118	4350
0.058	88	1423	0.065	84	1202
0.028	75	2638	0.025	52	2019
0.043	29	651	0.019	10	530
0.216	169	613	0.253	182	538
0.034	32	899	0.035	30	816
0.025	79	3094	0.026	72	2731

0.052	25	455	0.081	34	387
0.027	36	1299	0.033	38	1098
0.028	96	3341	0.03	96	3142
0.026	66	2457	0.03	65	2096
0.067	79	1102	0.061	59	908
0.068	103	1421	0.064	83	1208
0.015	19	1224	0.022	23	1036
0.027	30	1091	0.027	25	905
0.02	24	1193	0.02	21	1019
0.028	92	3252	0.032	78	2398
0.024	21	845	0.024	17	702
0.021	30	1410	0.02	24	1181
0.026	100	3737	0.024	85	3480
0.065	52	753	0.072	51	658
0.092	100	986	0.064	55	806
0.036	16	429	0.032	11	336
0.021	39	1787	0.024	36	1449
0.021	78	3595	0.022	69	3030
0.023	35	1504	0.019	24	1251
0.01	4	391	0.015	6	383
0.018	17	942	0.035	27	751
0.019	23	1169	0.023	21	894
0.017	35	2014	0.026	44	1678
0.027	64	2266	0.023	45	1936
0.029	127	4184	0.034	115	3292
0.023	69	2876	0.025	60	2383
0.024	57	2271	0.027	49	1777
0.028	53	1821	0.022	35	1523
0.024	57	2336	0.021	45	2079
0.019	56	2838	0.024	58	2371
0.097	202	1870	0.115	201	1549
0.028	47	1628	0.015	21	1408
0.03	96	3067	0.035	91	2533
0.07	67	888	0.099	85	774
0.031	44	1364	0.039	51	1257
0.022	69	3009	0.027	70	2542
0.021	32	1513	0.031	40	1235
0.02	45	2192	0.028	56	1912
0.028	23	799	0.026	17	640
0.021	22	1031	0.028	29	995

0.028	54	1852	0.014	22	1582
0.049	79	1539	0.064	79	1162
0.022	76	3367	0.024	75	3019
0.032	130	3957	0.03	103	3324
0.018	18	976	0.018	12	646
0.026	169	6455	0.026	148	5635
0.02	97	4677	0.021	88	4014
0.024	45	1858	0.025	40	1562
0.027	131	4806	0.03	124	3980
0.023	88	3714	0.028	85	2904
0.097	58	541	0.1	54	487
0.02	58	2773	0.022	57	2593
0.033	86	2494	0.028	59	2014
0.028	78	2664	0.023	50	2121
0.061	257	3934	0.068	237	3236
0.027	116	4137	0.021	79	3736
0.035	46	1255	0.024	26	1068
0.017	30	1728	0.021	30	1430
0.064	80	1170	0.097	106	987
0.02	79	3777	0.022	71	3124
0.029	46	1518	0.028	39	1344
0.031	62	1946	0.026	46	1735
0.025	48	1862	0.02	32	1556
0.019	32	1611	0.027	37	1310
0.019	24	1220	0.027	28	992
0.032	25	754	0.055	37	640
0.063	48	712	0.038	22	552
0.02	64	3187	0.026	71	2673
0.039	30	743	0.02	14	681
0.02	30	1463	0.026	29	1077
0.058	112	1817	0.045	68	1427
0.075	182	2258	0.096	185	1752
0.025	218	8670	0.023	181	7663
0.016	16	1007	0.027	23	839
0.028	34	1185	0.021	22	1020
0.052	104	1879	0.05	72	1354
0.084	83	909	0.117	98	743
0.058	109	1763	0.063	98	1457
0.069	91	1235	0.059	64	1028
0.025	70	2715	0.031	67	2109

0.07	76	1006	0.099	87	795
0.021	50	2314	0.029	53	1791
0.023	117	4927	0.025	110	4376
0.026	50	1899	0.019	30	1588
0.022	28	1266	0.039	46	1138
0.023	10	434	0.027	10	360
0.021	49	2331	0.026	56	2115
0.053	109	1945	0.052	92	1670
0.032	44	1320	0.02	23	1115
0.023	36	1515	0.033	43	1263
0.471	604	678	0.541	720	611
0.031	128	4027	0.031	102	3237
0.027	22	799	0.031	21	663
0.023	29	1255	0.019	22	1108
0.024	29	1163	0.031	28	881
0.036	68	1829	0.03	49	1594
0.026	50	1909	0.023	34	1441
0.023	43	1840	0.028	43	1492
0.022	33	1480	0.037	41	1074
0.023	101	4284	0.019	72	3640
0.024	60	2485	0.023	52	2204
0.016	16	954	0.029	22	732
0.027	79	2892	0.024	58	2400
0.032	27	812	0.022	17	749
0.063	66	986	0.046	37	776
0.028	82	2841	0.024	64	2608
0.022	66	2945	0.02	48	2382
0.027	65	2357	0.034	68	1954
0.03	54	1762	0.031	45	1393
0.019	35	1837	0.026	43	1618
0.054	53	932	0.055	46	789
0.048	68	1355	0.057	62	1018
0.079	115	1342	0.063	79	1185
0.021	27	1241	0.018	20	1115
0.027	110	4002	0.03	105	3438
0.027	38	1364	0.028	32	1114
0.061	197	3041	0.053	150	2654
0.058	53	864	0.051	38	712
0.028	95	3291	0.021	62	2881
0.022	48	2100	0.023	42	1774

0.027	49	1742	0.026	39	1488
0.172	57	275	0.375	122	203
0.053	29	523	0.042	19	438
0.028	90	3106	0.018	49	2618
0.038	65	1637	0.05	70	1343
0.056	78	1322	0.051	58	1084
0.028	52	1785	0.026	42	1578
0.029	84	2779	0.029	73	2426
0.019	32	1650	0.02	29	1439
0.032	27	828	0.025	18	689
0.036	44	1163	0.031	30	935
0.027	60	2153	0.035	61	1677
0.025	110	4229	0.029	111	3694
0.021	33	1532	0.013	16	1215
0.012	15	1263	0.022	24	1087
0.025	38	1472	0.025	29	1128
0.06	111	1749	0.068	103	1418
0.029	63	2108	0.026	48	1801
0.027	60	2134	0.019	33	1736
0.495	601	612	0.643	905	502
0.054	92	1610	0.076	109	1325
0.033	26	769	0.032	22	668
0.028	38	1326	0.047	57	1156
0.022	53	2382	0.022	44	1986
0.027	56	2023	0.025	46	1770
0.024	86	3440	0.019	61	3146
0.096	95	898	0.139	120	746
0.024	57	2312	0.024	50	2075
0.028	35	1225	0.025	27	1039
0.021	44	2040	0.023	37	1601
0.021	13	600	0.037	16	422
0.026	31	1169	0.012	12	974
0.014	27	1972	0.018	31	1645
0.028	76	2645	0.026	63	2364
0.029	104	3422	0.026	82	3034
0.016	41	2461	0.026	56	2095
0.015	31	2007	0.028	48	1643
0.024	39	1558	0.028	40	1407
0.026	85	3241	0.026	76	2849
0.022	23	1025	0.024	22	895

0.041	41	967	0.052	49	886
0.029	74	2452	0.031	68	2148
0.02	57	2755	0.022	52	2265
0.021	21	972	0.035	30	817
0.024	106	4226	0.022	89	3870
0.019	38	1947	0.022	34	1510
0.08	58	670	0.106	62	525
0.022	32	1429	0.022	27	1181
0.019	34	1743	0.029	42	1398
0.045	66	1394	0.056	66	1112
0.03	23	738	0.02	13	635
0.024	17	679	0.023	13	548
0.022	38	1684	0.032	46	1411
0.02	62	3005	0.021	56	2675
0.03	77	2501	0.026	58	2203
0.022	99	4351	0.025	94	3624
0.06	26	408	0.069	21	284
0.018	42	2314	0.025	50	1945
0.019	43	2278	0.027	57	2075
0.046	95	1978	0.067	117	1633
0.032	37	1116	0.019	17	899
0.023	81	3503	0.028	78	2687
0.257	315	909	0.383	469	755
0.105	75	638	0.091	54	537
0.026	19	702	0.028	18	628
0.063	117	1732	0.073	114	1440
0.027	131	4647	0.026	109	4148
0.024	41	1633	0.02	29	1388
0.015	11	709	0.035	21	582
0.031	71	2255	0.025	48	1909
0.028	62	2179	0.018	35	1870
0.085	71	762	0.099	61	558
0.018	39	2078	0.023	39	1639
0.024	15	619	0.023	11	458
0.023	60	2580	0.032	68	2044
0.023	29	1228	0.043	52	1148
0.025	24	935	0.028	21	742
0.023	41	1726	0.03	41	1342
0.05	235	4496	0.052	198	3635
0.084	76	824	0.144	101	601

0.025	40	1589	0.02	27	1319
0.029	99	3291	0.024	69	2815
0.024	99	3995	0.027	94	3336
0.02	74	3672	0.02	62	3030
0.111	69	555	0.093	43	421
0.023	30	1255	0.023	28	1205
0.024	22	907	0.023	18	748
0.021	12	547	0.037	19	497
0.044	43	938	0.061	49	750
0.014	14	953	0.015	13	829
0.02	26	1289	0.024	25	1023
0.021	46	2138	0.019	35	1834
0.017	24	1427	0.034	42	1205
0.018	54	2885	0.017	41	2377
0.043	81	1798	0.059	95	1505
0.025	87	3452	0.026	84	3188
0.027	53	1925	0.016	26	1564
0.02	28	1384	0.029	36	1185
0.024	52	2082	0.016	29	1765
0.029	143	4721	0.028	118	4124
0.037	150	3906	0.03	112	3565
0.049	153	2970	0.05	131	2514
0.019	14	737	0.031	20	626
0.023	42	1746	0.029	46	1536
0.016	49	3045	0.02	59	2842
0.34	378	733	0.539	615	526
0.026	92	3474	0.032	95	2856
0.027	57	2038	0.026	51	1945
0.027	58	2129	0.018	35	1861
0.024	62	2491	0.025	55	2160
0.03	32	1030	0.019	17	892
0.024	41	1676	0.021	29	1375
0.021	40	1840	0.023	37	1551
0.022	115	5214	0.025	118	4582
0.058	66	1072	0.071	70	922
0.109	125	1025	0.131	122	806
0.065	116	1679	0.074	116	1443
0.057	158	2606	0.067	158	2189
0.055	75	1301	0.052	57	1042
0.03	86	2826	0.027	68	2471

0.023	24	1038	0.039	33	820
0.025	32	1246	0.036	42	1141
0.844	1278	236	0.88	1702	233
0.407	411	598	0.51	522	502
0.05	77	1471	0.061	80	1236
0.032	66	1965	0.03	53	1736
0.024	69	2860	0.025	65	2541
0.071	67	883	0.074	58	730
0.063	93	1392	0.06	66	1027
0.019	35	1760	0.025	35	1370
0.028	51	1769	0.02	28	1361
0.019	23	1215	0.031	31	956
0.019	33	1749	0.022	33	1490
0.053	118	2092	0.058	109	1762
0.024	54	2183	0.02	36	1789
0.032	15	453	0.022	9	406
0.033	28	826	0.034	25	714
0.071	89	1169	0.048	46	908
0.028	36	1262	0.037	40	1041
0.017	13	767	0.024	14	574
0.021	44	2082	0.023	40	1729
0.025	46	1816	0.032	46	1407
0.026	38	1424	0.029	38	1278
0.052	121	2212	0.063	126	1865
0.056	77	1299	0.063	65	974
0.07	51	676	0.081	52	587
0.045	99	2099	0.058	108	1766
0.069	83	1118	0.068	59	807
0.029	52	1754	0.03	47	1510
0.018	55	2993	0.024	63	2521
0.07	49	650	0.073	44	561
0.028	103	3532	0.026	74	2744
0.048	85	1670	0.047	70	1428
0.029	44	1451	0.027	33	1202
0.061	102	1559	0.072	96	1232
0.026	21	800	0.016	11	692
0.059	78	1244	0.061	74	1137
0.026	34	1295	0.02	24	1205
0.025	20	783	0.032	20	601
0.024	98	3960	0.021	72	3352

0.045	36	765	0.028	19	659
0.017	47	2799	0.02	48	2309
0.063	210	3150	0.083	238	2628
0.033	54	1562	0.029	40	1345
0.018	14	786	0.035	27	742
0.022	44	1986	0.021	34	1586
0.034	107	3053	0.026	68	2565
0.022	29	1274	0.033	37	1069
0.052	258	4687	0.046	192	3968
0.026	69	2561	0.03	65	2071
0.022	77	3472	0.021	64	3055
0.026	23	847	0.028	22	759
0.023	46	1958	0.025	43	1682
0.023	159	6703	0.02	123	5900
0.123	164	1170	0.142	166	1002
0.022	74	3249	0.021	57	2619
0.054	64	1132	0.041	45	1041
0.027	31	1113	0.012	12	1024
0.034	83	2377	0.021	45	2067
0.021	55	2519	0.025	53	2036
0.025	29	1117	0.023	22	952
0.04	41	972	0.041	39	913
0.03	47	1514	0.041	54	1267
0.025	39	1541	0.026	36	1329
0.141	111	676	0.159	106	560
0.061	51	780	0.066	46	655
0.022	24	1071	0.035	28	772
0.021	55	2613	0.02	46	2275
0.043	91	2037	0.042	80	1830
0.02	24	1167	0.024	25	998
0.071	61	796	0.054	39	678
0.04	30	724	0.071	45	592
0.026	24	906	0.026	22	826
0.085	101	1085	0.097	97	900
0.029	75	2487	0.027	61	2182
0.022	62	2714	0.028	67	2348
0.027	60	2180	0.025	44	1710
0.054	71	1242	0.056	62	1038
0.018	14	779	0.02	14	697
0.062	76	1150	0.052	49	893

0.051	103	1925	0.047	84	1712
0.067	53	735	0.053	38	684
0.022	20	894	0.01	8	800
0.051	38	706	0.056	33	553
0.027	77	2816	0.025	67	2662
0.119	118	874	0.102	79	696
0.045	70	1477	0.021	27	1243
0.059	71	1131	0.074	65	810
0.029	36	1222	0.031	35	1107
0.026	101	3855	0.027	88	3144
0.018	66	3545	0.023	71	3055
0.029	46	1516	0.026	33	1258
0.021	26	1206	0.031	28	885
0.207	290	1109	0.234	278	909
0.071	62	808	0.082	56	630
0.084	73	793	0.11	77	621
0.025	37	1463	0.014	16	1140
0.05	232	4439	0.057	210	3458
0.082	56	631	0.101	62	551
0.029	97	3242	0.03	79	2591
0.029	19	635	0.034	20	567
0.024	26	1077	0.023	23	981
0.027	64	2292	0.023	44	1874
0.024	59	2369	0.026	54	2034
0.023	24	998	0.024	21	843
0.029	84	2775	0.029	66	2235
0.016	54	3228	0.018	46	2561
0.029	39	1284	0.02	23	1130
0.021	36	1645	0.025	35	1378
0.024	41	1675	0.018	24	1347
0.025	67	2620	0.021	47	2228
0.031	16	498	0.036	16	432
0.064	128	1866	0.086	153	1617
0.028	63	2153	0.03	56	1806
0.028	89	3048	0.028	81	2802
0.054	50	880	0.051	37	689
0.024	85	3528	0.028	82	2840
0.026	89	3362	0.029	93	3141
0.025	26	1032	0.029	27	890
0.024	69	2749	0.029	68	2243

0.027	71	2559	0.026	54	2050
0.028	107	3783	0.023	75	3205
0.023	91	3804	0.022	72	3187
0.022	112	5040	0.019	90	4544
0.1	144	1293	0.154	174	956
0.019	61	3176	0.023	65	2770
0.019	34	1762	0.029	44	1470
0.022	74	3240	0.019	54	2725
0.018	52	2891	0.026	63	2326
0.091	148	1475	0.086	120	1273
0.026	49	1845	0.027	44	1573
0.042	33	758	0.068	44	603
0.023	32	1334	0.036	44	1163
0.401	283	422	0.556	385	308
0.043	117	2583	0.053	117	2094
0.021	38	1798	0.03	45	1447
0.021	131	6121	0.02	109	5261
0.025	77	3023	0.025	65	2557
0.023	93	3873	0.024	83	3363
0.038	76	1942	0.048	82	1634
0.021	22	1014	0.023	18	753
0.03	56	1799	0.03	45	1479
0.019	38	1938	0.028	43	1519
0.022	101	4544	0.018	70	3787
0.021	33	1522	0.016	21	1290
0.04	33	786	0.035	23	635
0.035	45	1253	0.022	23	1002
0.023	24	1005	0.016	13	814
0.018	44	2405	0.023	45	1901
0.015	16	1033	0.033	28	818
0.019	34	1787	0.029	41	1372
0.022	52	2264	0.023	42	1792
0.026	30	1134	0.017	17	1010
0.093	139	1351	0.131	176	1164
0.021	48	2243	0.024	42	1740
0.03	41	1341	0.025	28	1114
0.026	25	942	0.035	28	780
0.042	65	1493	0.062	82	1247
0.022	14	621	0.026	15	569
0.061	137	2123	0.036	71	1875

0.025	83	3184	0.025	68	2629
0.026	84	3150	0.026	66	2510
0.018	49	2722	0.022	52	2333
0.02	33	1588	0.034	51	1438
0.025	16	614	0.012	7	558
0.027	128	4595	0.025	101	3934
0.084	52	564	0.095	47	450
0.059	65	1046	0.088	77	801
0.01	12	1195	0.026	27	1031
0.056	319	5392	0.053	253	4493
0.027	71	2569	0.015	32	2058
0.019	44	2252	0.023	47	2022
0.019	19	978	0.03	26	841
0.024	62	2541	0.025	52	2007
0.859	819	134	0.886	1012	130
0.022	69	3125	0.029	83	2806
0.021	154	7022	0.021	129	6017
0.018	50	2654	0.017	40	2332
0.064	181	2642	0.054	119	2070
0.052	55	997	0.059	49	776
0.054	96	1694	0.051	78	1456
0.031	78	2424	0.023	43	1845
0.024	57	2275	0.026	50	1883
0.107	67	561	0.104	57	489
0.024	49	2015	0.023	39	1635
0.114	92	713	0.094	61	586
0.02	27	1337	0.039	48	1185
0.02	40	1949	0.022	36	1623
0.025	83	3186	0.03	81	2652
0.027	70	2565	0.032	67	2027
0.026	72	2737	0.025	64	2459
0.018	41	2176	0.022	42	1868
0.03	32	1021	0.033	28	831
0.022	28	1239	0.034	36	1027
0.026	102	3863	0.021	71	3332
0.034	15	432	0.078	34	401
0.031	15	469	0.03	12	387
0.045	94	2004	0.055	92	1584
0.023	16	666	0.029	17	564
0.024	113	4535	0.032	127	3880

0.026	55	2024	0.03	51	1627
0.015	29	1962	0.019	32	1692
0.016	44	2665	0.025	57	2216
0.03	40	1292	0.025	27	1045
0.022	35	1551	0.019	25	1264
0.093	74	719	0.144	93	553
0.02	33	1596	0.033	46	1331
0.024	54	2203	0.017	33	1869
0.027	72	2601	0.025	55	2134
0.072	131	1697	0.084	123	1350
0.017	35	1994	0.019	32	1666
0.012	13	1033	0.024	22	878
0.02	60	2876	0.029	74	2474
0.065	118	1686	0.063	93	1378
0.02	34	1655	0.035	46	1265
0.025	114	4486	0.029	126	4185
0.023	76	3253	0.02	52	2575
0.029	43	1435	0.029	38	1268
0.062	206	3111	0.058	163	2641
0.023	24	1030	0.025	22	861
0.025	83	3248	0.026	77	2931
0.02	89	4321	0.025	97	3805
0.054	176	3105	0.051	143	2649
0.023	54	2265	0.021	45	2051
0.02	27	1323	0.021	25	1138
0.041	99	2331	0.045	89	1886
0.024	39	1574	0.029	39	1317
0.022	57	2497	0.018	40	2201
0.019	71	3608	0.024	75	3021
0.013	22	1625	0.031	42	1314
0.022	36	1567	0.023	32	1363
0.046	124	2544	0.047	112	2264
0.025	46	1784	0.024	39	1586
0.02	14	696	0.01	6	586
0.023	38	1586	0.021	31	1424
0.02	38	1846	0.024	38	1579
0.03	96	3074	0.025	76	2913
0.053	50	890	0.046	35	722
0.051	105	1958	0.04	71	1693
0.023	79	3429	0.029	89	2969

0.03	20	638	0.016	9	555
0.027	115	4195	0.023	79	3347
0.024	61	2523	0.033	77	2282
0.024	79	3213	0.023	67	2872
0.03	37	1183	0.022	24	1049
0.017	65	3672	0.022	70	3173
0.026	87	3321	0.021	63	2896
0.024	22	878	0.036	26	696
0.023	34	1419	0.02	26	1293
0.021	64	2933	0.018	49	2673
0.064	85	1237	0.091	96	957
0.021	69	3283	0.024	70	2869
0.032	53	1585	0.021	31	1443
0.03	21	671	0.028	18	614
0.019	65	3423	0.021	64	3032
0.021	66	3147	0.028	80	2787
0.02	36	1738	0.024	35	1436
0.024	34	1379	0.022	28	1241
0.029	59	1972	0.023	41	1714
0.033	37	1095	0.026	27	1028
0.024	35	1453	0.026	30	1142
0.082	136	1526	0.084	115	1258
0.024	156	6352	0.019	107	5648
0.019	28	1435	0.037	40	1046
0.024	29	1180	0.027	25	900
0.026	75	2767	0.027	65	2356
0.026	102	3857	0.032	107	3196
0.034	42	1211	0.025	26	1010
0.113	103	812	0.155	133	724
0.136	85	539	0.249	126	381
0.021	62	2926	0.018	49	2621
0.026	24	909	0.034	23	658
0.019	45	2376	0.029	55	1858
0.026	48	1767	0.032	53	1628
0.023	46	1956	0.027	47	1666
0.052	42	762	0.065	42	601
0.017	11	645	0.022	12	531
0.028	64	2251	0.024	47	1943
0.022	33	1492	0.034	40	1141
0.063	114	1693	0.054	86	1506

0.029	67	2221	0.026	49	1830
0.022	53	2363	0.034	69	1969
0.021	39	1789	0.026	38	1441
0.023	114	4866	0.023	91	3784
0.027	26	937	0.033	27	783
0.026	79	3004	0.027	72	2558
0.032	49	1497	0.031	40	1258
0.058	95	1530	0.059	84	1341
0.032	34	1036	0.033	28	816
0.033	109	3222	0.028	80	2806
0.031	38	1183	0.045	42	897
0.02	80	3949	0.021	72	3300
0.022	55	2474	0.026	59	2188
0.054	66	1158	0.029	29	981
0.02	18	882	0.017	13	750
0.033	80	2381	0.025	54	2141
0.016	41	2506	0.023	50	2103
0.031	84	2632	0.028	66	2326
0.021	29	1381	0.02	24	1185
0.04	69	1675	0.034	57	1608
0.048	53	1058	0.076	71	868
0.061	222	3411	0.048	145	2845
0.022	29	1266	0.018	19	1059
0.078	67	790	0.107	75	623
0.022	61	2703	0.023	53	2217
0.061	93	1427	0.042	50	1152
0.027	80	2857	0.023	58	2437
0.184	168	744	0.25	200	600
0.039	50	1224	0.054	55	966
0.023	110	4629	0.02	83	4100
0.019	46	2386	0.022	48	2102
0.031	26	806	0.037	27	699
0.025	71	2778	0.025	65	2501
0.02	56	2757	0.022	53	2304
0.076	162	1971	0.053	90	1599
0.02	31	1513	0.032	39	1174
0.025	93	3561	0.027	77	2822
0.033	46	1337	0.02	23	1146
0.066	111	1560	0.091	118	1178
0.029	34	1149	0.033	31	921

0.024	32	1292	0.021	24	1094
0.015	9	595	0.02	11	531
0.03	42	1348	0.035	46	1262
0.127	99	683	0.119	76	564
0.023	80	3469	0.022	67	2930
0.025	71	2754	0.025	59	2307
0.03	50	1628	0.029	41	1354
0.027	58	2070	0.027	53	1939
0.019	25	1306	0.032	37	1108
0.025	68	2676	0.019	44	2244
0.066	135	1918	0.061	106	1643
0.028	94	3273	0.022	63	2833
0.026	114	4196	0.027	92	3327
0.023	22	921	0.039	32	798
0.023	42	1808	0.026	38	1420
0.017	39	2315	0.024	52	2074
0.027	82	2973	0.029	74	2466
0.027	102	3733	0.027	80	2906
0.025	58	2269	0.021	46	2139
0.026	33	1225	0.023	23	967
0.027	83	2993	0.025	65	2530
0.03	164	5314	0.023	121	5070
0.067	140	1937	0.061	109	1687
0.02	19	910	0.019	14	709
0.023	132	5599	0.031	155	4798
0.012	16	1331	0.029	32	1080
0.056	186	3124	0.052	139	2558
0.035	60	1646	0.025	34	1337
0.076	99	1198	0.098	113	1036
0.033	21	612	0.07	37	492
0.082	97	1092	0.093	96	938
0.023	34	1439	0.027	33	1204
0.024	32	1300	0.024	30	1220
0.023	45	1944	0.025	43	1644
0.055	158	2726	0.048	110	2172
0.027	90	3301	0.024	67	2694
0.026	98	3645	0.021	72	3302
0.357	641	1157	0.434	776	1012
0.077	83	1000	0.048	48	956
0.029	74	2513	0.028	55	1893

0.03	25	799	0.024	19	770
0.025	80	3077	0.024	65	2612
0.024	190	7674	0.024	160	6591
0.028	61	2114	0.025	47	1810
0.016	11	657	0.012	7	569
0.076	63	764	0.106	74	622
0.048	51	1022	0.065	56	805
0.026	33	1260	0.029	30	991
0.022	28	1260	0.028	28	977
0.045	44	939	0.066	56	797
0.02	66	3310	0.02	58	2818
0.034	42	1202	0.029	31	1024
0.021	40	1834	0.025	36	1421
0.018	31	1655	0.025	36	1391
0.032	52	1557	0.025	29	1152
0.031	83	2570	0.026	61	2253
0.023	92	3975	0.027	95	3469
0.063	131	1959	0.061	105	1629
0.031	34	1053	0.022	20	877
0.018	44	2366	0.023	45	1922
0.024	19	775	0.031	18	568
0.056	80	1336	0.053	64	1140
0.026	34	1278	0.022	26	1136
0.036	33	896	0.028	25	867
0.018	51	2759	0.029	65	2181
0.049	146	2846	0.05	126	2396
0.029	48	1604	0.037	48	1245
0.026	64	2425	0.03	60	1924
0.023	25	1084	0.02	17	821
0.026	47	1750	0.037	62	1625
0.022	16	725	0.038	28	707
0.062	66	1004	0.052	47	850
0.028	44	1532	0.02	28	1362
0.031	84	2612	0.034	71	2030
0.024	178	7159	0.023	150	6415
0.027	81	2925	0.035	82	2242
0.027	41	1461	0.032	41	1243
0.064	72	1053	0.069	58	787
0.022	75	3259	0.016	48	2913
0.027	52	1873	0.02	34	1633

0.024	91	3767	0.026	88	3287
0.012	22	1746	0.027	39	1394
0.081	99	1119	0.129	123	834
0.025	104	4000	0.022	78	3398
0.02	52	2516	0.02	46	2204
0.021	53	2442	0.02	48	2321
0.025	64	2493	0.016	35	2181
0.025	62	2379	0.02	40	1994
0.03	81	2659	0.023	51	2131
0.025	61	2420	0.018	38	2113
0.114	127	983	0.219	214	761
0.288	136	336	0.297	137	325
0.136	187	1192	0.189	247	1063
0.026	64	2392	0.024	44	1788
0.022	68	3022	0.019	52	2629
0.065	100	1443	0.089	108	1104
0.016	18	1074	0.024	22	894
0.017	59	3371	0.018	52	2762
0.026	102	3838	0.02	67	3213
0.022	45	1958	0.03	51	1674
0.019	64	3347	0.027	88	3115
0.067	69	957	0.057	49	818
0.028	43	1499	0.021	28	1325
0.05	179	3366	0.055	163	2801
0.023	27	1171	0.025	24	939
0.022	34	1495	0.023	28	1210
0.016	24	1440	0.022	25	1114
0.02	27	1293	0.024	28	1118
0.029	55	1829	0.024	34	1411
0.074	140	1754	0.058	82	1338
0.023	62	2674	0.018	44	2365
0.086	44	468	0.118	49	366
0.026	30	1137	0.034	28	798
0.031	29	914	0.026	23	867
0.03	52	1700	0.023	35	1502
0.019	79	4093	0.024	90	3687
0.103	134	1167	0.137	146	916
0.028	38	1303	0.031	36	1131
0.024	57	2340	0.021	41	1942
0.027	58	2104	0.02	35	1699

0.024	17	681	0.031	19	598
0.026	44	1677	0.033	51	1477
0.024	46	1867	0.018	27	1477
0.02	41	1994	0.022	35	1562
0.023	63	2736	0.019	49	2468
0.021	58	2673	0.024	53	2188
0.048	47	930	0.082	71	791
0.026	30	1111	0.036	30	796
0.028	44	1532	0.025	38	1471
0.029	32	1061	0.041	36	839
0.019	23	1176	0.024	22	905
0.034	97	2743	0.029	77	2586
0.024	35	1445	0.031	41	1287
0.028	30	1051	0.031	29	908
0.025	22	860	0.02	14	687
0.061	62	956	0.06	50	783
0.02	69	3318	0.019	53	2731
0.023	68	2919	0.03	76	2445
0.052	85	1537	0.06	80	1244
0.017	33	1928	0.032	49	1506
0.077	110	1318	0.092	103	1016
0.03	95	3074	0.026	72	2667
0.063	56	834	0.072	52	672
0.018	41	2236	0.023	46	1960
0.056	56	941	0.062	60	915
0.061	142	2187	0.062	125	1883
0.025	64	2526	0.017	36	2133
0.021	28	1279	0.029	32	1081
0.058	170	2760	0.049	116	2236
0.024	66	2704	0.025	58	2282
0.019	25	1315	0.033	34	993
0.02	65	3193	0.023	66	2793
0.03	26	852	0.018	15	823
0.143	151	908	0.155	127	691
0.02	38	1908	0.021	34	1620
0.024	33	1321	0.027	32	1156
0.023	34	1427	0.027	32	1139
0.025	40	1586	0.031	39	1211
0.019	26	1361	0.018	21	1179
0.041	69	1631	0.014	21	1443

0.049	107	2085	0.06	112	1766
0.02	124	5991	0.024	128	5154
0.033	63	1835	0.022	38	1682
0.047	55	1110	0.024	25	1005
0.023	128	5403	0.022	107	4653
0.025	24	940	0.028	23	797
0.041	46	1068	0.038	37	935
0.029	54	1789	0.019	29	1523
0.019	23	1200	0.024	26	1043
0.03	64	2038	0.029	51	1713
0.069	62	833	0.075	56	693
0.024	14	576	0.022	11	485
0.037	22	565	0.054	26	459
0.026	49	1811	0.023	37	1597
0.137	184	1160	0.183	212	949
0.068	148	2026	0.063	108	1601
0.076	123	1501	0.094	119	1150
0.062	177	2671	0.05	124	2359
0.028	61	2112	0.029	54	1781
0.024	82	3369	0.024	71	2856
0.058	54	884	0.076	62	758
0.017	22	1245	0.026	25	954
0.047	37	756	0.019	13	660
0.023	63	2696	0.027	59	2146
0.028	140	4868	0.02	85	4106
0.067	29	401	0.081	32	361
0.025	62	2419	0.021	43	2035
0.029	88	2913	0.027	66	2423
0.029	71	2412	0.024	49	2013
0.02	79	3967	0.019	66	3465
0.029	39	1308	0.034	39	1104
0.023	37	1571	0.024	36	1442
0.03	58	1893	0.029	43	1429
0.033	43	1254	0.02	20	998
0.025	50	1917	0.026	42	1568
0.073	198	2532	0.056	129	2179
0.032	81	2437	0.018	36	1970
0.053	112	2021	0.063	112	1666
0.04	88	2111	0.047	89	1802
0.028	32	1120	0.036	30	810

0.064	187	2733	0.076	184	2229
0.019	28	1413	0.019	24	1238
0.051	105	1963	0.064	110	1612
0.04	34	826	0.022	16	723
0.024	56	2242	0.026	49	1817
0.027	35	1274	0.033	39	1138
0.022	72	3133	0.028	70	2431
0.031	67	2103	0.022	39	1725
0.04	34	821	0.049	39	749
0.064	67	983	0.084	73	798
0.057	79	1299	0.072	90	1154
0.03	31	1001	0.019	15	785
0.055	48	819	0.093	68	665
0.031	60	1906	0.024	37	1517
0.018	45	2525	0.014	29	2115
0.026	26	962	0.014	11	788
0.023	38	1610	0.028	38	1326
0.026	43	1581	0.024	31	1265
0.022	32	1441	0.029	36	1215
0.02	46	2248	0.028	56	1972
0.019	16	821	0.031	22	678
0.035	59	1639	0.03	43	1400
0.07	45	602	0.051	24	446
0.018	50	2785	0.019	42	2218
0.07	78	1031	0.055	55	954
0.25	629	1887	0.362	923	1629
0.021	43	2044	0.025	45	1750
0.019	96	4955	0.024	101	4059
0.268	192	525	0.337	230	453
0.018	24	1292	0.025	28	1109
0.02	40	1938	0.019	33	1691
0.03	76	2494	0.021	47	2226
0.018	29	1565	0.02	26	1252
0.027	184	6571	0.03	175	5688
0.029	68	2246	0.029	54	1777
0.024	147	6024	0.03	151	4963
0.068	137	1864	0.083	137	1507
0.03	79	2581	0.026	62	2280
0.022	41	1831	0.027	43	1549
0.063	87	1297	0.062	63	948

0.043	35	777	0.06	40	622
0.068	90	1237	0.068	80	1092
0.019	17	867	0.022	17	766
0.091	112	1123	0.087	87	917
0.021	47	2194	0.03	54	1730
0.057	70	1155	0.118	125	937
0.024	33	1367	0.028	33	1143
0.022	63	2809	0.023	54	2333
0.025	22	872	0.025	19	736
0.028	123	4287	0.024	96	3947
0.021	68	3123	0.027	80	2833
0.024	131	5226	0.026	122	4614
0.028	30	1030	0.025	23	903
0.062	138	2088	0.073	115	1461
0.027	132	4812	0.029	108	3643
0.021	82	3789	0.022	71	3163
0.023	44	1891	0.032	49	1504
0.021	36	1699	0.035	52	1432
0.023	79	3422	0.019	57	2963
0.021	73	3443	0.029	84	2805
0.022	59	2622	0.018	40	2173
0.026	60	2224	0.023	46	1977
0.026	37	1392	0.02	25	1204
0.03	43	1382	0.022	27	1220
0.062	150	2259	0.071	143	1865
0.024	65	2590	0.026	58	2173
0.193	116	486	0.239	118	376
0.026	66	2514	0.027	52	1906
0.021	51	2378	0.027	52	1859
0.042	97	2211	0.032	60	1826
0.023	36	1509	0.024	29	1197
0.031	67	2122	0.035	65	1776
0.033	39	1137	0.028	29	997
0.022	60	2614	0.027	63	2238
0.022	39	1755	0.028	41	1407
0.03	107	3464	0.02	60	2868
0.031	155	4798	0.026	104	3898
0.023	93	3931	0.024	80	3307
0.022	53	2315	0.024	49	1992
0.074	48	604	0.066	37	520

0.025	33	1313	0.033	38	1121
0.033	43	1247	0.028	30	1053
0.053	75	1331	0.037	46	1211
0.024	104	4321	0.027	104	3783
0.025	44	1717	0.022	30	1323
0.022	51	2288	0.022	45	1967
0.053	100	1781	0.058	82	1320
0.025	33	1286	0.024	28	1122
0.026	52	1931	0.025	46	1801
0.077	58	694	0.073	50	633
0.043	33	740	0.079	56	657
0.027	55	1962	0.034	56	1587
0.053	78	1396	0.053	65	1153
0.022	31	1378	0.018	23	1226
0.02	66	3184	0.019	55	2783
0.063	154	2300	0.069	143	1943
0.029	70	2332	0.023	48	2046
0.03	123	4021	0.032	107	3211
0.045	87	1827	0.038	61	1556
0.058	98	1588	0.052	68	1248
0.011	5	452	0.012	5	419
0.023	79	3406	0.025	68	2617
0.062	140	2107	0.066	121	1711
0.019	47	2405	0.034	66	1886
0.025	109	4293	0.02	76	3751
0.03	67	2138	0.033	67	1971
0.109	90	739	0.161	117	610
0.029	82	2757	0.022	51	2217
0.036	137	3717	0.033	111	3281
0.023	41	1771	0.034	54	1556
0.019	48	2544	0.023	50	2148
0.018	37	2014	0.016	29	1755
0.063	132	1956	0.067	113	1572
0.024	91	3750	0.019	59	3092
0.027	78	2801	0.036	92	2472
0.023	84	3555	0.023	74	3108
0.022	18	804	0.037	25	653
0.061	87	1333	0.079	89	1043
0.022	55	2500	0.026	51	1937
0.034	59	1683	0.028	38	1310

0.025	74	2874	0.034	75	2123
0.032	53	1616	0.022	33	1454
0.055	102	1738	0.049	81	1579
0.041	46	1086	0.041	35	813
0.025	75	2944	0.035	87	2381
0.025	39	1541	0.031	41	1293
0.021	52	2381	0.023	45	1945
0.016	15	922	0.02	16	776
0.023	70	2932	0.032	81	2472
0.024	64	2555	0.034	74	2112
0.027	40	1420	0.024	31	1261
0.02	17	815	0.03	20	647
0.024	28	1154	0.022	24	1082
0.019	85	4445	0.025	94	3686
0.028	50	1707	0.028	43	1476
0.026	95	3595	0.024	76	3033
0.018	68	3796	0.018	62	3292
0.131	87	578	0.159	97	513
0.026	77	2862	0.019	48	2506
0.06	98	1545	0.07	90	1201
0.105	185	1583	0.095	142	1358
0.046	50	1028	0.078	70	824
0.017	43	2472	0.025	54	2110
0.068	58	789	0.085	57	616
0.035	89	2446	0.025	53	2033
0.026	110	4176	0.028	98	3371
0.028	22	750	0.024	15	617
0.033	41	1207	0.031	29	905
0.024	81	3336	0.032	84	2571
0.016	56	3536	0.02	65	3141
0.024	82	3328	0.029	86	2890
0.034	88	2523	0.019	38	1917
0.027	49	1794	0.019	28	1451
0.061	65	1005	0.053	53	946
0.025	48	1894	0.024	41	1645
0.031	25	779	0.015	10	663
0.019	48	2445	0.026	59	2168
0.025	43	1706	0.026	40	1513
0.066	130	1851	0.072	119	1540
0.032	47	1428	0.031	37	1164

0.018	38	2019	0.019	31	1599
0.018	20	1112	0.028	25	861
0.024	64	2582	0.028	69	2380
0.021	83	3885	0.028	88	3035
0.016	18	1085	0.017	15	859
0.022	45	2026	0.033	59	1731
0.016	52	3104	0.024	67	2714
0.02	29	1445	0.019	23	1159
0.026	32	1181	0.019	18	930
0.028	28	978	0.024	21	841
0.018	8	430	0.026	11	410
0.017	27	1577	0.025	34	1328
0.027	52	1842	0.027	45	1624
0.026	33	1246	0.021	23	1089
0.023	47	2035	0.027	46	1638
0.028	66	2326	0.028	56	1914
0.057	60	995	0.079	65	760
0.026	40	1510	0.037	46	1202
0.021	42	1927	0.032	55	1641
0.058	48	785	0.088	63	651
0.024	62	2515	0.024	58	2326
0.081	74	842	0.105	86	731
0.038	24	614	0.025	14	552
0.034	39	1110	0.036	36	975
0.055	43	742	0.08	51	584
0.067	132	1833	0.061	100	1551
0.026	25	924	0.03	24	774
0.026	92	3438	0.023	71	2953
0.022	63	2775	0.032	75	2266
0.055	144	2488	0.038	80	2053
0.019	47	2410	0.024	50	2044
0.022	60	2623	0.03	69	2227
0.022	62	2805	0.026	64	2421
0.042	58	1308	0.055	64	1097
0.1	84	756	0.148	106	608
0.02	25	1242	0.026	29	1102
0.027	57	2019	0.021	41	1947
0.023	48	2085	0.025	45	1779
0.052	58	1055	0.043	42	925
0.025	48	1847	0.02	32	1530

0.024	117	4785	0.023	100	4197
0.02	42	2046	0.029	53	1755
0.032	53	1601	0.023	31	1328
0.016	33	1982	0.025	45	1740
0.03	49	1574	0.026	37	1362
0.029	38	1286	0.039	44	1097
0.021	30	1412	0.029	33	1124
0.022	53	2339	0.027	55	1946
0.02	17	851	0.033	20	592
0.06	79	1245	0.056	67	1137
0.035	51	1404	0.03	33	1079
0.016	15	937	0.018	14	766
0.028	93	3201	0.027	76	2718
0.019	57	2932	0.024	60	2449
0.03	28	898	0.021	14	649
0.022	77	3371	0.024	67	2770
0.084	56	612	0.066	43	611
0.018	69	3669	0.019	60	3178
0.016	29	1764	0.023	35	1493
0.022	35	1563	0.037	48	1258
0.031	46	1415	0.027	31	1097
0.029	57	1918	0.027	44	1611
0.017	44	2495	0.026	62	2284
0.026	35	1321	0.024	27	1081
0.021	33	1523	0.041	54	1267
0.014	35	2465	0.019	43	2164
0.066	128	1797	0.06	103	1615
0.023	56	2352	0.028	53	1840
0.031	52	1622	0.032	46	1393
0.011	12	1035	0.034	31	894
0.068	47	644	0.051	27	500
0.123	224	1591	0.132	187	1232
0.066	65	927	0.064	49	715
0.103	167	1449	0.146	216	1264
0.02	34	1672	0.026	40	1485
0.02	22	1061	0.021	19	874
0.022	106	4694	0.026	110	4068
0.025	79	3055	0.035	90	2515
0.026	38	1411	0.033	41	1199
0.021	16	750	0.014	9	641

0.055	69	1197	0.062	67	1019
0.029	63	2124	0.022	42	1899
0.024	26	1052	0.028	27	931
0.017	24	1423	0.029	34	1119
0.022	56	2526	0.025	53	2074
0.053	35	624	0.042	27	609
0.025	128	4946	0.031	131	4074
0.024	43	1760	0.028	43	1486
0.059	103	1640	0.083	124	1362
0.022	102	4595	0.029	113	3781
0.018	26	1391	0.034	43	1221
0.02	69	3340	0.019	59	3078
0.029	48	1595	0.029	42	1421
0.037	48	1257	0.039	41	999
0.032	72	2181	0.027	54	1947
0.059	45	720	0.079	52	606
0.021	43	1958	0.039	61	1490
0.018	34	1825	0.027	38	1368
0.028	25	866	0.021	16	735
0.025	62	2380	0.024	50	2076
0.023	47	2029	0.026	46	1720
0.021	64	2999	0.032	84	2560
0.059	100	1591	0.057	78	1289
0.023	73	3087	0.027	76	2701
0.02	69	3460	0.025	81	3215
0.049	54	1049	0.035	34	930
0.061	118	1817	0.057	90	1501
0.019	55	2865	0.034	79	2276
0.031	85	2696	0.034	88	2470
0.019	33	1700	0.024	35	1403
0.024	112	4478	0.025	106	4055
0.024	73	3027	0.029	77	2601
0.034	32	923	0.027	21	765
0.028	115	4025	0.026	91	3351
0.023	89	3750	0.024	81	3269
0.024	51	2078	0.028	46	1613
0.026	107	3954	0.028	91	3193
0.095	105	998	0.143	141	847
0.023	51	2181	0.026	50	1907
0.024	66	2695	0.023	56	2371

0.02	50	2425	0.026	59	2254
0.024	42	1740	0.035	51	1392
0.02	33	1591	0.031	41	1287
0.026	34	1298	0.035	43	1189
0.024	26	1050	0.021	17	803
0.018	36	1967	0.018	29	1578
0.031	57	1754	0.027	41	1484
0.025	24	954	0.025	19	740
0.022	58	2557	0.029	62	2056
0.032	78	2396	0.023	47	1967
0.03	75	2460	0.022	48	2148
0.02	24	1187	0.02	21	1014
0.032	76	2318	0.044	87	1881
0.082	71	796	0.115	94	724
0.028	142	4883	0.027	113	4132
0.021	43	1980	0.018	33	1815
0.065	143	2058	0.064	113	1662
0.118	93	698	0.103	70	608
0.052	84	1536	0.044	64	1376
0.023	93	3889	0.027	94	3421
0.03	44	1427	0.034	42	1189
0.06	93	1449	0.057	65	1077
0.037	51	1336	0.029	35	1192
0.026	91	3400	0.03	90	2959
0.026	92	3451	0.03	97	3190
0.025	53	2102	0.018	34	1861
0.019	61	3167	0.018	52	2884
0.095	82	778	0.134	85	551
0.053	87	1568	0.057	79	1316
0.025	66	2603	0.02	41	2040
0.05	21	398	0.06	20	315
0.019	61	3130	0.031	85	2645
0.031	76	2393	0.024	51	2042
0.086	49	522	0.106	53	445
0.027	68	2434	0.033	73	2122
0.027	61	2168	0.023	48	2056
0.023	44	1855	0.024	41	1660
0.053	33	591	0.057	35	574
0.027	103	3723	0.023	76	3222
0.022	77	3464	0.022	64	2869

0.022	44	1943	0.029	48	1586
0.021	31	1451	0.023	30	1275
0.066	61	857	0.06	43	668
0.025	69	2678	0.029	62	2098
0.033	78	2317	0.023	48	2032
0.026	40	1503	0.017	22	1302
0.017	19	1108	0.017	15	883
0.034	75	2119	0.02	35	1717
0.026	26	968	0.03	27	870
0.077	142	1697	0.078	119	1405
0.019	21	1097	0.014	13	927
0.029	76	2584	0.024	52	2103
0.046	68	1420	0.056	69	1154
0.022	74	3319	0.023	70	2968
0.02	18	900	0.047	33	674
0.066	86	1217	0.081	87	988
0.027	32	1150	0.023	21	900
0.072	89	1148	0.09	89	905
0.02	26	1277	0.033	38	1127
0.38	559	912	0.426	628	845
0.017	62	3554	0.02	59	2945
0.022	95	4258	0.021	76	3566
0.018	25	1330	0.021	26	1229
0.05	154	2956	0.057	139	2315
0.021	57	2701	0.038	83	2117
0.397	222	337	0.543	285	240
0.057	49	814	0.063	46	689
0.023	48	2007	0.027	45	1616
0.019	49	2517	0.023	48	2002
0.033	73	2164	0.036	64	1726
0.061	80	1230	0.056	60	1008
0.077	43	515	0.065	31	449
0.065	73	1042	0.065	60	860
0.02	47	2294	0.03	57	1869
0.025	57	2190	0.025	44	1735
0.025	32	1257	0.028	32	1120
0.025	41	1571	0.02	27	1296
0.024	55	2190	0.024	46	1889
0.019	96	5046	0.024	105	4263
0.025	44	1695	0.03	44	1437

0.029	58	1915	0.028	46	1580
0.019	30	1589	0.029	38	1292
0.064	115	1681	0.063	97	1440
0.054	158	2745	0.043	111	2489
0.062	80	1220	0.05	59	1113
0.03	57	1867	0.024	41	1682
0.055	83	1421	0.041	54	1272
0.024	72	2950	0.025	63	2507
0.021	68	3129	0.021	59	2731
0.025	121	4629	0.023	93	4003
0.05	77	1462	0.055	74	1263
0.025	20	777	0.043	32	704
0.049	60	1177	0.049	59	1149
0.032	76	2288	0.027	53	1918
0.101	112	998	0.116	113	862
0.027	60	2145	0.024	40	1648
0.024	91	3738	0.023	76	3272
0.028	45	1561	0.014	19	1375
0.024	69	2762	0.026	65	2411
0.024	39	1581	0.027	41	1488
0.264	537	1500	0.383	742	1193
0.029	53	1779	0.026	40	1508
0.142	200	1210	0.206	286	1104
0.019	26	1370	0.034	39	1122
0.04	45	1086	0.073	57	720
0.016	20	1225	0.031	34	1060
0.024	67	2734	0.027	64	2336
0.03	46	1511	0.03	38	1211
0.025	72	2780	0.028	70	2442
0.019	31	1604	0.028	43	1492
0.072	75	973	0.052	48	872
0.024	39	1569	0.02	28	1348
0.03	44	1437	0.033	40	1177
0.021	83	3823	0.024	80	3226
0.185	163	717	0.232	184	608
0.024	37	1512	0.027	36	1293
0.018	30	1611	0.029	41	1350
0.026	77	2879	0.02	48	2388
0.025	81	3184	0.021	61	2798
0.022	62	2776	0.036	83	2241

0.064	35	510	0.092	35	346
0.026	31	1141	0.032	31	935
0.028	54	1903	0.029	52	1732
0.022	62	2770	0.026	59	2204
0.009	4	422	0.032	11	328
0.022	37	1634	0.021	29	1344
0.026	50	1899	0.026	41	1512
0.046	82	1702	0.03	45	1469
0.025	33	1305	0.025	32	1234
0.02	21	1040	0.022	18	784
0.062	141	2142	0.049	90	1754
0.013	8	588	0.03	17	551
0.033	66	1951	0.032	52	1565
0.019	16	846	0.009	7	750
0.019	23	1212	0.027	27	974
0.019	32	1661	0.015	22	1410
0.06	63	989	0.064	61	895
0.027	94	3433	0.025	71	2748
0.022	31	1375	0.035	39	1083
0.021	53	2437	0.022	51	2223
0.046	38	791	0.04	33	784
0.025	44	1731	0.031	46	1422
0.023	15	633	0.014	7	506
0.038	65	1635	0.034	53	1486
0.045	77	1630	0.065	90	1298
0.021	96	4492	0.021	82	3740
0.025	28	1077	0.031	27	850
0.81	1701	400	0.886	2294	296
0.019	117	5973	0.025	125	4970
0.018	43	2282	0.028	53	1811
0.034	74	2115	0.025	43	1683
0.017	25	1440	0.025	32	1227
0.031	37	1171	0.021	22	1043
0.026	17	629	0.034	19	536
0.023	132	5702	0.027	140	4964
0.027	104	3678	0.032	109	3335
0.028	63	2216	0.023	43	1823
0.023	39	1672	0.031	45	1400
0.03	51	1639	0.028	38	1299
0.056	48	810	0.075	58	715

0.025	64	2485	0.028	55	1937
0.066	195	2753	0.06	143	2232
0.057	165	2734	0.057	129	2143
0.053	69	1242	0.053	62	1105
0.02	27	1323	0.028	32	1107
0.028	51	1783	0.043	68	1514
0.104	76	652	0.12	71	519
0.026	42	1583	0.024	32	1301
0.025	65	2519	0.024	49	2024
0.02	18	900	0.025	20	781
0.035	65	1818	0.032	45	1380
0.03	31	989	0.034	31	878
0.075	155	1917	0.084	132	1444
0.066	59	830	0.076	52	632
0.026	22	839	0.021	15	695
0.068	50	684	0.081	57	648
0.022	17	740	0.021	13	614
0.023	35	1475	0.018	24	1343
0.056	129	2184	0.05	97	1861
0.023	84	3631	0.03	87	2847
0.024	79	3164	0.025	69	2706
0.029	75	2522	0.026	54	2051
0.044	9	195	0.043	6	135
0.073	111	1405	0.097	134	1247
0.017	66	3784	0.022	74	3262
0.07	75	993	0.088	71	736
0.087	145	1523	0.075	100	1227
0.025	51	1998	0.028	48	1650
0.016	27	1656	0.023	33	1375
0.036	42	1119	0.024	24	977
0.031	35	1109	0.04	38	923
0.025	53	2094	0.027	54	1966
0.016	13	775	0.016	13	800
0.021	57	2706	0.024	55	2229
0.022	110	4913	0.032	133	4052
0.023	69	2879	0.031	69	2175
0.358	910	1632	0.462	1208	1409
0.076	74	895	0.12	97	709
0.085	105	1133	0.057	57	935
0.021	30	1394	0.029	33	1098

0.023	36	1538	0.038	51	1289
0.049	67	1310	0.048	51	1022
0.055	135	2326	0.062	126	1902
0.031	34	1075	0.016	14	844
0.019	33	1734	0.034	50	1419
0.093	130	1275	0.122	136	979
0.02	35	1749	0.025	37	1426
0.03	37	1204	0.022	26	1139
0.015	10	665	0.037	20	522
0.089	64	655	0.21	138	518
0.079	125	1454	0.067	83	1159
0.027	81	2939	0.018	48	2584
0.02	43	2069	0.026	47	1745
0.027	27	974	0.018	16	874
0.024	82	3284	0.026	79	2903
0.017	18	1069	0.03	28	902
0.026	47	1773	0.021	32	1494
0.063	122	1828	0.062	96	1441
0.021	46	2168	0.029	56	1850
0.023	61	2566	0.024	54	2207
0.018	40	2160	0.022	41	1789
0.081	62	706	0.103	67	583
0.026	93	3443	0.029	92	3084
0.023	18	748	0.02	13	633
0.028	20	705	0.031	18	565
0.16	229	1205	0.152	198	1108
0.025	86	3415	0.026	74	2754
0.02	17	831	0.02	13	652
0.038	68	1743	0.029	44	1469
0.036	56	1480	0.028	37	1307
0.062	96	1450	0.057	79	1307
0.066	123	1732	0.075	120	1487
0.016	15	934	0.024	19	784
0.147	124	722	0.183	124	552
0.026	38	1435	0.012	15	1225
0.01	6	612	0.014	7	494
0.024	44	1773	0.027	37	1338
0.047	139	2792	0.061	147	2251
0.029	100	3398	0.028	83	2846
0.087	118	1244	0.09	99	995

0.025	58	2251	0.028	57	1960
0.021	37	1701	0.024	35	1454
0.021	84	3876	0.019	67	3399
0.027	90	3268	0.025	72	2822
0.028	68	2393	0.023	49	2112
0.121	111	803	0.163	132	680
0.021	41	1948	0.026	41	1545
0.035	48	1322	0.02	24	1205
0.056	169	2875	0.058	147	2408
0.025	64	2514	0.022	47	2074
0.025	59	2309	0.021	39	1853
0.025	56	2202	0.035	61	1678
0.068	54	739	0.08	61	702
0.027	55	1949	0.03	48	1575
0.023	33	1413	0.031	38	1207
0.353	822	1506	0.445	1002	1248
0.026	36	1365	0.02	24	1171
0.023	65	2735	0.021	51	2356
0.084	93	1010	0.057	54	887
0.029	54	1834	0.027	38	1364
0.026	34	1287	0.019	21	1074
0.02	36	1747	0.022	35	1584
0.021	32	1477	0.014	20	1361
0.028	30	1033	0.027	28	1008
0.025	50	1911	0.025	43	1690
0.021	23	1086	0.019	18	951
0.076	50	609	0.058	31	508
0.027	32	1147	0.017	17	956
0.03	69	2234	0.027	45	1643
0.064	93	1358	0.043	53	1192
0.026	28	1047	0.025	24	924
0.021	91	4263	0.025	95	3692
0.02	36	1763	0.026	36	1345
0.047	56	1139	0.065	64	919
0.026	46	1711	0.02	31	1523
0.041	41	953	0.044	37	804
0.07	77	1026	0.042	38	870
0.022	62	2707	0.019	46	2408
0.028	39	1372	0.036	47	1251
0.019	73	3740	0.021	64	2998

0.079	92	1070	0.068	66	902
0.026	40	1482	0.014	19	1381
0.048	132	2625	0.043	91	2029
0.07	66	882	0.065	55	792
0.024	61	2488	0.034	74	2074
0.027	66	2394	0.023	49	2074
0.032	73	2218	0.026	50	1855
0.021	50	2344	0.02	43	2152
0.021	45	2145	0.023	43	1805
0.057	63	1042	0.071	57	742
0.038	78	1989	0.023	43	1805
0.028	20	704	0.033	19	555
0.027	84	3049	0.021	57	2604
0.023	106	4500	0.021	79	3769
0.032	44	1314	0.024	27	1102
0.03	53	1699	0.028	40	1373
0.032	24	717	0.027	17	607
0.06	35	545	0.071	36	473
0.026	71	2617	0.033	70	2052
0.042	57	1310	0.041	49	1139
0.057	65	1085	0.056	57	961
0.015	32	2037	0.023	38	1628
0.021	33	1504	0.018	26	1400
0.023	111	4743	0.023	92	3930
0.024	72	2890	0.024	58	2373
0.018	36	1927	0.026	41	1531
0.032	56	1714	0.026	35	1297
0.026	84	3170	0.021	55	2573
0.027	48	1732	0.026	40	1509
0.022	69	3041	0.023	59	2488
0.085	66	711	0.136	92	586
0.059	73	1174	0.058	59	961
0.027	33	1197	0.036	32	864
0.021	61	2886	0.027	71	2593
0.026	82	3086	0.034	83	2394
0.028	44	1508	0.021	27	1256
0.018	17	938	0.031	24	762
0.03	63	2047	0.018	34	1822
0.031	35	1093	0.025	23	892
0.022	160	7240	0.026	171	6325

0.023	57	2443	0.024	53	2114
0.053	85	1512	0.057	75	1232
0.018	24	1340	0.016	18	1076
0.022	95	4233	0.022	88	3926
0.021	36	1639	0.021	30	1366
0.021	79	3656	0.026	82	3113
0.024	70	2883	0.025	62	2411
0.023	75	3141	0.03	88	2865
0.05	144	2714	0.056	129	2192
0.014	33	2268	0.028	53	1854
0.025	45	1765	0.031	52	1618
0.017	19	1113	0.019	18	951
0.019	78	4025	0.019	65	3408
0.024	57	2310	0.028	60	2097
0.014	25	1753	0.02	31	1552
0.073	76	972	0.098	89	815
0.026	90	3386	0.024	68	2818
0.015	15	955	0.033	29	856
0.082	78	870	0.128	109	742
0.028	87	3018	0.026	67	2514
0.04	34	819	0.035	28	763
0.025	35	1345	0.031	32	1013
0.02	176	8523	0.022	169	7467
0.948	1086	60	0.959	1392	59
0.029	33	1099	0.027	29	1047
0.022	42	1894	0.023	32	1384
0.042	71	1618	0.052	70	1281
0.031	50	1586	0.021	27	1254
0.026	72	2719	0.024	58	2322
0.071	39	512	0.088	40	416
0.056	184	3096	0.047	129	2603
0.023	66	2845	0.029	80	2641
0.018	46	2540	0.027	59	2095
0.024	91	3631	0.022	64	2887
0.056	81	1374	0.07	78	1031
0.025	62	2461	0.024	49	1955
0.022	24	1092	0.031	29	903
0.028	51	1744	0.022	34	1519
0.025	38	1467	0.02	27	1293
0.03	26	854	0.037	26	683

0.027	100	3658	0.025	81	3096
0.146	147	861	0.189	168	722
0.032	42	1273	0.021	26	1241
0.068	160	2190	0.048	98	1928
0.024	60	2412	0.019	38	1950
0.032	60	1842	0.023	40	1675
0.027	65	2362	0.029	63	2098
0.018	43	2350	0.028	55	1918
0.026	64	2434	0.029	62	2077
0.03	66	2098	0.033	55	1624
0.066	115	1638	0.049	75	1454
0.058	82	1329	0.05	58	1098
0.024	49	1995	0.025	44	1702
0.111	108	867	0.105	94	799
0.027	43	1528	0.028	38	1307
0.057	130	2141	0.04	75	1799
0.031	32	1005	0.032	24	725
0.026	37	1385	0.018	21	1168
0.025	54	2137	0.022	42	1827
0.037	23	597	0.023	13	549
0.007	4	540	0.019	10	520
0.048	75	1482	0.063	85	1264
0.02	39	1905	0.024	39	1559
0.534	882	771	0.689	1230	555
0.029	101	3335	0.026	81	2987
0.032	65	1997	0.026	44	1646
0.638	302	171	0.713	361	145
0.057	169	2780	0.048	123	2418
0.02	33	1652	0.019	28	1446
0.028	93	3268	0.025	77	2953
0.095	50	474	0.097	47	436
0.065	75	1079	0.071	67	877
0.063	84	1243	0.048	61	1198
0.024	65	2685	0.031	73	2267
0.081	111	1254	0.095	108	1034
0.027	90	3298	0.027	77	2765
0.091	107	1070	0.093	88	858
0.019	27	1403	0.033	38	1130
0.022	67	3029	0.031	83	2600
0.054	51	900	0.072	60	776

0.022	34	1536	0.02	29	1439
0.07	255	3402	0.056	165	2785
0.029	77	2550	0.025	55	2124
0.031	41	1295	0.034	40	1142
0.028	42	1477	0.028	32	1116
0.018	29	1542	0.028	35	1195
0.039	87	2135	0.032	61	1869
0.081	58	658	0.109	75	612
0.03	41	1341	0.023	24	1035
0.021	51	2374	0.035	70	1942
0.06	75	1179	0.049	47	908
0.017	23	1314	0.033	40	1172
0.025	61	2427	0.014	32	2261
0.02	51	2536	0.021	46	2112
0.028	50	1744	0.024	38	1547
0.027	44	1572	0.029	42	1386
0.022	131	5949	0.019	97	5142
0.079	48	558	0.057	31	517
0.018	19	1015	0.025	21	822
0.02	30	1469	0.023	27	1125
0.025	37	1457	0.027	32	1162
0.052	120	2179	0.062	116	1760
0.022	51	2317	0.027	55	1968
0.059	82	1303	0.072	88	1134
0.03	51	1644	0.041	64	1493
0.076	66	797	0.052	40	730
0.619	779	480	0.703	1036	437
0.02	42	2028	0.024	41	1689
0.025	32	1227	0.028	30	1023
0.031	45	1412	0.038	43	1086
0.072	89	1152	0.058	58	935
0.021	48	2232	0.025	48	1908
0.055	112	1932	0.054	90	1569
0.064	175	2548	0.054	115	2000
0.02	45	2168	0.025	51	1998
0.023	74	3075	0.028	78	2663
0.054	182	3207	0.058	170	2742
0.047	127	2561	0.048	118	2338
0.028	139	4849	0.023	97	4085
0.028	34	1172	0.015	15	998

0.05	74	1398	0.056	70	1190
0.052	36	655	0.07	40	531
0.024	61	2517	0.027	61	2238
0.031	61	1918	0.02	30	1502
0.055	106	1820	0.048	79	1552
0.25	323	970	0.348	453	849
0.025	37	1461	0.031	41	1283
0.067	241	3339	0.064	194	2834
0.027	68	2414	0.025	57	2246
0.027	42	1533	0.027	33	1190
0.012	15	1266	0.022	23	1010
0.015	18	1163	0.02	20	993
0.022	34	1521	0.03	39	1262
0.026	33	1251	0.023	25	1049
0.027	111	4008	0.021	70	3297
0.031	40	1268	0.021	24	1113
0.026	64	2362	0.021	44	2025
0.027	33	1209	0.02	20	992
0.029	37	1243	0.021	25	1138
0.026	50	1887	0.03	55	1797
0.581	913	659	0.693	1240	549
0.023	49	2127	0.023	43	1867
0.054	90	1584	0.039	52	1273
0.019	28	1458	0.022	28	1270
0.017	21	1188	0.029	32	1071
0.024	85	3514	0.024	74	3026
0.034	35	992	0.034	31	869
0.018	45	2478	0.022	46	2014
0.073	63	799	0.092	65	644
0.026	22	812	0.028	18	627
0.058	58	944	0.062	51	767
0.026	39	1440	0.024	33	1326
0.026	51	1907	0.024	42	1683
0.382	443	718	0.498	579	583
0.026	47	1790	0.023	36	1564
0.026	158	5876	0.034	169	4832
0.03	19	605	0.029	17	566
0.052	94	1720	0.047	72	1472
0.021	52	2456	0.03	57	1825
0.059	102	1627	0.056	87	1456

0.035	35	969	0.022	16	708
0.023	62	2604	0.028	63	2220
0.024	68	2803	0.027	61	2235
0.026	36	1328	0.017	18	1021
0.028	111	3917	0.025	83	3210
0.027	19	697	0.036	20	533
0.091	185	1855	0.113	187	1470
0.03	64	2092	0.022	38	1667
0.05	58	1092	0.054	50	880
0.273	229	610	0.389	340	535
0.022	63	2784	0.033	77	2283
0.058	61	989	0.041	37	870
0.028	35	1201	0.028	29	1007
0.019	43	2234	0.024	45	1820
0.198	328	1331	0.254	374	1096
0.018	35	1866	0.02	31	1495
0.026	33	1252	0.03	35	1120
0.032	43	1304	0.039	39	970
0.017	17	1003	0.023	22	924
0.057	41	679	0.059	42	673
0.062	148	2231	0.063	125	1850
0.028	54	1847	0.029	43	1448
0.019	29	1489	0.025	33	1290
0.183	173	770	0.235	209	681
0.065	67	960	0.035	30	825
0.021	67	3053	0.019	52	2753
0.249	380	1144	0.367	581	1003
0.059	89	1415	0.044	58	1253
0.048	118	2335	0.043	88	1948
0.025	72	2851	0.021	51	2392
0.051	79	1481	0.048	65	1280
0.031	68	2094	0.022	43	1924
0.056	68	1142	0.048	43	856
0.048	44	869	0.056	43	722
0.014	28	1926	0.016	26	1647
0.025	39	1507	0.023	31	1305
0.02	35	1679	0.023	30	1252
0.025	104	4016	0.033	112	3240
0.019	64	3393	0.025	71	2753
0.059	216	3423	0.049	154	3009

0.028	37	1270	0.021	24	1108
0.088	71	733	0.095	72	683
0.022	35	1561	0.027	35	1260
0.026	131	4872	0.024	109	4382
0.018	44	2349	0.031	63	1963
0.053	81	1455	0.078	94	1110
0.018	21	1161	0.026	26	969
0.024	53	2111	0.024	47	1912
0.026	13	483	0.026	12	450
0.028	47	1632	0.032	44	1341
0.021	57	2598	0.027	61	2199
0.024	50	2050	0.017	32	1821
0.025	39	1502	0.021	26	1191
0.027	39	1429	0.017	20	1137
0.066	83	1169	0.072	73	938
0.027	52	1900	0.031	49	1554
0.097	105	974	0.131	123	819
0.018	52	2765	0.025	59	2276
0.063	116	1714	0.053	78	1407
0.063	147	2203	0.073	145	1840
0.02	34	1649	0.028	40	1377
0.032	59	1807	0.022	33	1443
0.028	122	4305	0.03	101	3305
0.023	19	822	0.015	10	636
0.07	90	1188	0.08	78	898
0.019	43	2207	0.018	35	1869
0.017	19	1113	0.039	36	893
0.022	27	1178	0.024	24	968
0.05	170	3248	0.05	140	2686
0.606	458	298	0.714	574	230
0.029	55	1838	0.018	30	1642
0.018	20	1105	0.02	18	902
0.019	32	1678	0.016	22	1355
0.053	155	2797	0.042	102	2301
0.016	19	1201	0.027	30	1101
0.052	132	2421	0.06	126	1990
0.03	20	640	0.017	10	588
0.1	94	842	0.099	64	582
0.027	63	2304	0.025	54	2074
0.057	123	2032	0.067	118	1650

0.157	207	1113	0.209	240	910
0.037	22	574	0.046	26	536
0.068	73	998	0.071	65	850
0.019	59	2998	0.026	66	2480
0.018	59	3132	0.024	61	2481
0.026	95	3522	0.028	85	2911
0.029	70	2313	0.03	61	1992
0.03	64	2094	0.027	48	1725
0.022	53	2313	0.028	59	2044
0.018	90	4815	0.022	93	4171
0.031	39	1216	0.018	19	1016
0.03	49	1602	0.024	32	1284
0.016	58	3672	0.027	77	2811
0.06	142	2210	0.066	129	1822
0.024	90	3724	0.029	94	3179
0.038	43	1080	0.073	68	863
0.023	74	3178	0.025	68	2618
0.023	53	2246	0.028	54	1905
0.025	39	1548	0.025	32	1271
0.067	87	1203	0.063	64	954
0.023	47	1997	0.026	46	1718
0.018	24	1300	0.019	21	1078
0.021	46	2112	0.033	62	1811
0.035	16	446	0.03	13	422
0.018	60	3338	0.027	76	2689
0.042	104	2370	0.042	88	1989
0.03	94	3092	0.025	67	2638
0.031	42	1301	0.027	30	1069
0.079	70	812	0.08	55	632
0.022	41	1797	0.023	40	1678
0.025	100	3856	0.022	76	3305
0.05	53	997	0.046	43	895
0.022	30	1317	0.021	26	1194
0.014	16	1095	0.024	23	920
0.057	103	1705	0.065	104	1504
0.029	42	1390	0.021	23	1074
0.055	102	1755	0.06	87	1365
0.053	110	1954	0.054	98	1723
0.025	115	4548	0.023	94	4018
0.027	29	1038	0.021	20	925

0.029	55	1836	0.022	35	1569
0.027	36	1282	0.033	35	1014
0.021	171	7826	0.021	145	6889
0.021	19	902	0.021	18	827
0.019	13	664	0.023	15	636
0.026	75	2839	0.021	51	2430
0.024	72	2887	0.026	66	2496
0.068	75	1022	0.082	74	827
0.027	43	1539	0.027	35	1258
0.031	26	822	0.035	28	767
0.138	224	1397	0.187	263	1140
0.017	53	3038	0.023	63	2695
0.023	37	1551	0.017	22	1303
0.069	210	2840	0.068	165	2258
0.03	53	1710	0.018	28	1509
0.063	54	802	0.076	46	563
0.022	49	2165	0.026	45	1669
0.093	92	901	0.073	59	747
0.043	35	771	0.041	31	730
0.036	36	974	0.036	30	798
0.023	29	1214	0.023	24	1015
0.021	99	4629	0.023	87	3720
0.025	43	1657	0.027	34	1211
0.025	37	1429	0.023	27	1156
0.025	49	1920	0.021	34	1612
0.024	64	2611	0.029	67	2207
0.026	37	1371	0.03	41	1314
0.021	80	3819	0.026	83	3085
0.032	61	1861	0.041	68	1605
0.022	61	2752	0.022	55	2434
0.023	29	1250	0.03	30	967
0.028	80	2783	0.026	63	2348
0.057	43	714	0.071	52	680
0.029	11	362	0.017	6	338
0.027	43	1571	0.021	29	1340
0.023	46	1962	0.027	44	1595
0.031	27	845	0.023	18	754
0.024	56	2240	0.015	30	1954
0.023	21	906	0.03	22	705
0.027	76	2733	0.028	72	2463

0.06	41	638	0.111	66	528
0.054	152	2653	0.068	148	2042
0.072	48	621	0.138	86	536
0.022	16	727	0.017	10	568
0.065	151	2171	0.065	131	1871
0.017	11	621	0.041	20	467
0.067	75	1041	0.048	41	822
0.018	22	1199	0.02	21	1013
0.024	63	2594	0.029	59	1974
0.056	89	1502	0.057	73	1205
0.023	27	1155	0.026	23	857
0.058	51	829	0.071	57	747
0.091	139	1383	0.098	121	1120
0.022	71	3178	0.021	61	2813
0.044	112	2408	0.05	102	1929
0.065	63	899	0.049	40	781
0.03	36	1162	0.025	26	994
0.021	32	1477	0.024	34	1358
0.025	78	3089	0.022	56	2512
0.031	70	2219	0.024	51	2062
0.018	12	664	0.035	20	556
0.02	38	1834	0.025	38	1506
0.019	41	2071	0.019	32	1610
0.098	235	2161	0.117	227	1705
0.02	24	1170	0.021	20	923
0.024	64	2588	0.022	46	2037
0.022	36	1577	0.023	34	1424
0.066	71	999	0.036	34	923
0.023	56	2348	0.035	76	2076
0.017	22	1258	0.03	31	1018
0.02	25	1232	0.032	33	1014
0.022	46	2060	0.027	43	1540
0.026	69	2633	0.03	70	2240
0.027	68	2438	0.032	73	2175
0.054	150	2618	0.048	118	2343
0.023	24	1039	0.034	31	874
0.022	78	3526	0.031	100	3109
0.019	20	1017	0.021	18	835
0.032	39	1197	0.024	22	914
0.061	77	1183	0.051	50	935

0.128	89	609	0.124	61	432
0.024	37	1503	0.028	37	1262
0.098	79	728	0.105	66	562
0.028	35	1206	0.027	26	928
0.026	82	3058	0.028	72	2522
0.035	24	664	0.025	14	556
0.013	9	659	0.009	5	570
0.026	36	1361	0.039	47	1152
0.022	25	1101	0.02	16	804
0.02	91	4354	0.024	90	3716
0.024	40	1604	0.026	37	1399
0.06	94	1473	0.095	123	1171
0.024	74	3014	0.021	53	2464
0.018	25	1333	0.031	36	1109
0.02	25	1222	0.027	28	1014
0.035	75	2055	0.026	48	1772
0.022	54	2382	0.024	49	2015
0.018	30	1648	0.017	25	1489
0.018	19	1050	0.035	26	718
0.023	46	1938	0.027	49	1757
0.022	62	2751	0.022	51	2262
0.02	86	4133	0.029	104	3425
0.024	33	1326	0.031	36	1113
0.026	73	2773	0.018	40	2135
0.019	15	777	0.033	22	653
0.023	37	1607	0.026	37	1372
0.023	38	1649	0.018	25	1329
0.055	151	2578	0.057	129	2142
0.022	73	3239	0.022	61	2735
0.058	53	854	0.037	27	696
0.02	84	4067	0.032	114	3440
0.023	66	2852	0.029	64	2171
0.1	61	552	0.109	56	460
0.03	62	2038	0.02	36	1808
0.024	32	1317	0.02	24	1177
0.023	49	2096	0.026	52	1965
0.032	70	2100	0.024	49	1977
0.02	39	1927	0.023	37	1541
0.022	41	1802	0.02	33	1579
0.02	19	921	0.032	25	762

0.025	24	949	0.024	21	869
0.045	82	1721	0.036	54	1465
0.028	50	1767	0.024	36	1443
0.024	33	1368	0.022	24	1084
0.029	68	2302	0.02	39	1891
0.027	37	1347	0.027	29	1034
0.021	22	1026	0.036	31	824
0.027	60	2128	0.019	32	1634
0.027	73	2667	0.018	43	2396
0.021	60	2755	0.03	67	2183
0.073	49	625	0.078	46	546
0.058	111	1817	0.075	124	1536
0.018	41	2215	0.024	41	1679
0.017	27	1602	0.013	18	1396
0.023	42	1768	0.013	20	1514
0.023	63	2656	0.02	45	2206
0.026	97	3615	0.024	79	3213
0.022	68	3032	0.026	73	2731
0.818	1427	318	0.878	1938	270
0.027	52	1856	0.027	41	1505
0.092	63	622	0.096	57	536
0.065	58	830	0.066	52	734
0.387	391	620	0.525	588	532
0.022	62	2777	0.023	53	2296
0.019	28	1415	0.02	24	1173
0.033	116	3354	0.028	80	2812
0.055	99	1709	0.068	101	1391
0.019	33	1725	0.023	34	1421
0.058	145	2371	0.061	118	1819
0.016	18	1122	0.024	23	929
0.023	25	1057	0.035	33	910
0.063	151	2238	0.063	125	1855
0.016	32	1914	0.018	31	1692
0.025	55	2146	0.03	56	1781
0.037	51	1311	0.037	46	1204
0.051	78	1448	0.087	111	1166
0.022	28	1272	0.03	33	1078
0.023	35	1469	0.025	32	1230
0.023	138	5851	0.024	116	4690
0.025	30	1189	0.026	27	1020

0.033	40	1173	0.031	35	1090
0.025	46	1777	0.024	39	1580
0.026	25	941	0.023	17	716
0.019	38	1953	0.017	29	1699
0.035	64	1775	0.046	72	1480
0.023	71	3046	0.03	79	2518
0.03	101	3319	0.027	79	2847
0.02	67	3284	0.022	64	2823
0.025	34	1318	0.039	46	1148
0.028	30	1027	0.02	18	869
0.072	169	2172	0.068	135	1861
0.026	43	1613	0.032	46	1389
0.023	40	1673	0.02	28	1340
0.07	43	569	0.042	20	458
0.018	13	711	0.025	15	592
0.027	62	2224	0.026	48	1824
0.071	67	881	0.065	47	678
0.089	83	846	0.119	91	676
0.023	44	1874	0.019	32	1666
0.074	66	828	0.065	49	705
0.02	35	1689	0.024	34	1408
0.026	34	1255	0.028	29	1004
0.031	31	960	0.038	29	743
0.052	89	1633	0.076	112	1366
0.029	52	1739	0.034	53	1512
0.03	41	1323	0.03	34	1105
0.064	124	1818	0.07	107	1428
0.019	15	794	0.038	27	676
0.019	69	3608	0.032	100	2992
0.023	68	2861	0.022	52	2352
0.023	73	3084	0.023	64	2729
0.022	31	1383	0.039	51	1241
0.022	25	1117	0.028	26	913
0.067	92	1281	0.066	74	1049
0.02	46	2204	0.025	51	2004
0.03	43	1407	0.03	37	1217
0.019	24	1229	0.017	19	1116
0.035	70	1950	0.03	52	1679
0.019	35	1792	0.026	41	1565
0.021	24	1133	0.032	33	983

0.064	48	706	0.057	32	531
0.027	111	4021	0.023	77	3325
0.023	33	1417	0.028	32	1119
0.278	172	446	0.44	329	418
0.054	127	2225	0.059	116	1847
0.031	64	1977	0.038	62	1561
0.023	40	1720	0.027	42	1509
0.028	38	1314	0.035	36	986
0.042	36	826	0.078	58	689
0.026	111	4215	0.026	101	3754
0.024	54	2194	0.025	48	1845
0.101	68	603	0.105	53	453
0.024	48	1932	0.023	38	1620
0.022	37	1662	0.034	47	1340
0.072	30	385	0.104	41	352
0.021	34	1602	0.027	37	1325
0.069	94	1274	0.077	84	1002
0.024	158	6514	0.02	110	5307
0.026	43	1604	0.038	52	1328
0.025	21	806	0.041	30	710
0.02	20	983	0.024	19	757
0.045	21	444	0.026	10	382
0.068	95	1304	0.04	53	1276
0.023	78	3266	0.031	87	2690
0.032	35	1058	0.013	11	854
0.023	46	1957	0.025	44	1730
0.026	71	2679	0.033	73	2164
0.027	63	2273	0.025	52	1999
0.015	18	1190	0.022	21	919
0.024	85	3477	0.013	41	3003
0.018	53	2877	0.022	52	2310
0.151	168	948	0.182	196	882
0.024	66	2665	0.023	56	2381
0.025	100	3899	0.023	77	3209
0.026	83	3118	0.027	66	2414
0.023	37	1557	0.02	28	1381
0.076	128	1552	0.102	138	1214
0.023	53	2302	0.018	37	1988
0.034	34	980	0.026	22	826
0.027	26	920	0.037	29	750

0.071	68	887	0.085	64	686
0.064	72	1058	0.075	71	878
0.029	62	2101	0.026	44	1639
0.022	32	1399	0.022	28	1218
0.025	36	1382	0.021	27	1240
0.017	28	1663	0.018	27	1467
0.022	39	1719	0.029	44	1494
0.102	129	1131	0.152	173	968
0.015	20	1324	0.025	29	1126
0.075	152	1872	0.067	114	1594
0.022	26	1162	0.026	22	835
0.026	79	2973	0.024	62	2558
0.03	115	3763	0.033	105	3118
0.031	70	2207	0.035	74	2051
0.071	36	471	0.071	28	366
0.023	65	2720	0.02	48	2360
0.062	54	818	0.042	28	646
0.021	44	2084	0.027	49	1764
0.027	48	1734	0.03	41	1329
0.025	42	1622	0.024	34	1363
0.022	22	985	0.02	17	841
0.027	64	2309	0.026	51	1875
0.024	32	1287	0.027	31	1106
0.016	15	952	0.023	18	762
0.03	77	2499	0.027	58	2058
0.022	28	1248	0.032	32	955
0.036	50	1326	0.029	32	1062
0.028	23	799	0.007	5	683
0.031	63	1991	0.029	56	1851
0.03	61	1984	0.02	32	1606
0.019	24	1272	0.012	13	1092
0.024	157	6453	0.023	131	5514
0.023	54	2317	0.021	43	1979
0.046	32	663	0.052	29	530
0.024	48	1961	0.024	41	1652
0.03	35	1148	0.036	35	936
0.026	79	2928	0.032	83	2513
0.022	52	2274	0.028	61	2098
0.029	22	736	0.024	15	623
0.063	69	1021	0.056	48	810

0.026	58	2175	0.024	43	1731
0.016	22	1337	0.021	25	1185
0.022	41	1793	0.026	43	1596
0.026	39	1449	0.037	48	1250
0.019	73	3858	0.024	84	3381
0.025	65	2551	0.026	55	2090
0.029	17	568	0.05	24	457
0.038	40	1005	0.032	24	719
0.022	36	1621	0.04	52	1239
0.035	75	2075	0.025	44	1747
0.03	68	2163	0.023	43	1815
0.02	62	2997	0.016	44	2626
0.024	94	3812	0.021	69	3222
0.027	27	976	0.03	23	742
0.019	21	1079	0.033	29	842
0.026	28	1031	0.033	27	791
0.025	103	4039	0.028	106	3701
0.03	76	2451	0.023	49	2116
0.014	8	577	0.024	13	535
0.027	73	2595	0.033	71	2078
0.024	72	2922	0.024	61	2452
0.034	65	1875	0.023	36	1545
0.054	109	1921	0.048	78	1544
0.055	51	884	0.057	43	716
0.031	30	928	0.019	14	714
0.02	80	3995	0.017	58	3374
0.023	112	4772	0.029	125	4185
0.027	49	1742	0.023	35	1461
0.027	96	3421	0.017	52	2959
0.023	47	2017	0.02	34	1705
0.013	16	1259	0.025	26	1028
0.031	43	1350	0.036	45	1204
0.033	30	875	0.022	18	790
0.021	24	1127	0.025	25	995
0.027	22	803	0.028	21	732
0.023	36	1504	0.027	36	1310
0.431	613	810	0.575	893	659
0.101	73	650	0.143	82	490
0.016	23	1416	0.011	14	1317
0.043	25	563	0.028	15	514

0.031	97	3038	0.027	70	2477
0.021	32	1472	0.018	24	1284
0.11	320	2588	0.134	323	2093
0.03	28	891	0.029	22	738
0.034	53	1486	0.02	25	1212
0.026	89	3379	0.032	103	3152
0.022	24	1054	0.016	15	951
0.023	152	6317	0.023	130	5577
0.029	82	2723	0.027	60	2131
0.069	93	1260	0.053	56	996
0.031	22	681	0.023	14	600
0.028	73	2548	0.019	42	2178
0.022	39	1754	0.025	39	1530
0.024	24	997	0.026	20	743
0.019	23	1218	0.022	24	1077
0.062	66	998	0.064	54	793
0.022	64	2857	0.021	56	2576
0.021	39	1840	0.029	46	1514
0.025	109	4248	0.017	60	3404
0.051	81	1511	0.064	90	1327
0.03	41	1324	0.025	30	1169
0.034	23	644	0.044	21	453
0.019	27	1381	0.022	26	1148
0.029	63	2142	0.021	43	1960
0.024	67	2708	0.029	69	2309
0.131	174	1152	0.143	150	897
0.023	36	1553	0.025	34	1318
0.027	68	2491	0.029	65	2145
0.048	87	1720	0.049	77	1485
0.025	35	1365	0.017	20	1142
0.033	33	982	0.015	12	813
0.017	46	2636	0.025	58	2271
0.018	40	2225	0.027	54	1919
0.026	25	933	0.022	20	869
0.028	49	1726	0.033	48	1396
0.136	158	1000	0.211	242	904
0.025	44	1729	0.035	51	1408
0.024	24	976	0.02	16	795
0.023	44	1879	0.025	41	1570
0.029	26	870	0.03	22	722

0.02	32	1530	0.037	54	1403
0.026	41	1514	0.026	34	1280
0.016	26	1567	0.02	25	1249
0.022	45	2009	0.036	61	1634
0.029	42	1413	0.025	34	1304
0.103	207	1804	0.116	193	1474
0.022	21	939	0.022	18	811
0.069	50	675	0.086	53	561
0.025	51	2030	0.024	44	1777
0.02	69	3330	0.025	77	2994
0.021	76	3555	0.03	90	2932
0.022	13	574	0.033	15	436
0.326	370	765	0.439	539	689
0.025	26	1023	0.018	17	910
0.049	63	1218	0.069	68	917
0.037	48	1255	0.018	20	1076
0.048	114	2256	0.054	107	1890
0.022	70	3049	0.028	81	2769
0.063	103	1526	0.073	89	1125
0.02	88	4379	0.024	91	3745
0.084	122	1338	0.092	109	1075
0.027	43	1564	0.029	39	1289
0.053	62	1108	0.05	46	875
0.014	24	1690	0.033	48	1397
0.057	76	1256	0.075	78	961
0.023	73	3058	0.024	67	2716
0.031	45	1430	0.025	31	1213
0.028	51	1752	0.029	45	1503
0.023	14	584	0.018	10	534
0.021	28	1304	0.02	23	1119
0.027	142	5107	0.024	111	4514
0.021	41	1937	0.025	47	1863
0.026	55	2055	0.025	47	1797
0.035	37	1011	0.034	27	771
0.029	93	3141	0.029	77	2619
0.029	135	4536	0.027	110	3925
0.029	46	1536	0.023	28	1179
0.031	49	1530	0.026	33	1251
0.026	53	1990	0.023	38	1644
0.029	18	613	0.015	7	456

0.021	74	3462	0.018	59	3157
0.024	129	5272	0.023	110	4630
0.024	100	4139	0.02	76	3727
0.019	19	983	0.026	23	871
0.028	45	1585	0.021	29	1344
0.025	43	1706	0.029	39	1312
0.026	65	2459	0.023	48	2030
0.023	62	2599	0.037	83	2168
0.048	59	1177	0.078	78	928
0.021	27	1281	0.018	20	1102
0.026	105	3871	0.021	76	3595
0.086	113	1203	0.086	89	949
0.021	51	2416	0.037	72	1900
0.066	109	1543	0.049	63	1223
0.025	55	2127	0.021	37	1739
0.014	10	686	0.024	14	579
0.088	90	935	0.09	68	691
0.026	42	1588	0.022	29	1285
0.021	58	2731	0.024	61	2475
0.108	76	628	0.146	87	507
0.031	30	931	0.024	18	730
0.023	158	6852	0.026	149	5643
0.059	137	2196	0.058	113	1846
0.019	15	760	0.036	22	583
0.076	156	1894	0.068	103	1413
0.02	16	798	0.03	20	637
0.018	25	1367	0.033	40	1157
0.022	81	3658	0.026	87	3300
0.023	34	1422	0.037	47	1225
0.03	28	898	0.034	26	733
0.03	107	3415	0.03	92	2941
0.019	20	1061	0.03	27	884
0.018	36	2020	0.028	51	1753
0.03	39	1277	0.027	28	994
0.023	36	1536	0.013	18	1317
0.031	32	1000	0.022	20	884
0.019	11	561	0.014	6	434
0.064	91	1335	0.074	82	1027
0.02	36	1749	0.025	35	1356
0.055	175	3036	0.07	185	2449

0.056	101	1692	0.058	82	1340
0.024	39	1583	0.021	29	1358
0.062	102	1552	0.056	80	1358
0.039	48	1184	0.044	48	1046
0.03	91	2990	0.024	66	2632
0.016	34	2126	0.025	42	1638
0.023	77	3254	0.026	77	2887
0.019	37	1898	0.026	45	1685
0.03	35	1128	0.02	19	934
0.022	14	618	0.022	13	581
0.022	87	3908	0.025	85	3351
0.029	51	1686	0.021	29	1359
0.06	107	1672	0.06	88	1379
0.017	6	352	0.032	8	241
0.018	83	4484	0.021	87	4044
0.023	43	1847	0.024	39	1574
0.025	39	1522	0.039	56	1376
0.019	18	951	0.016	12	742
0.075	54	667	0.09	66	668
0.037	32	825	0.019	14	704
0.022	30	1309	0.021	26	1189
0.028	47	1662	0.039	57	1414
0.058	83	1345	0.042	48	1108
0.062	55	836	0.036	29	779
0.023	59	2509	0.025	55	2187
0.02	68	3290	0.022	61	2760
0.024	53	2152	0.019	37	1948
0.023	21	898	0.018	14	756
0.027	42	1533	0.023	33	1423
0.025	27	1058	0.023	23	960
0.022	45	2022	0.021	39	1793
0.024	41	1702	0.026	36	1360
0.024	87	3476	0.023	70	3015
0.068	102	1399	0.058	72	1160
0.034	44	1233	0.035	40	1100
0.022	56	2482	0.025	53	2076
0.023	43	1787	0.024	36	1468
0.022	50	2213	0.026	50	1839
0.058	60	968	0.067	65	901
0.025	19	730	0.029	20	658

0.02	47	2271	0.028	55	1891
0.018	12	659	0.038	22	558
0.025	93	3588	0.026	83	3154
0.023	157	6668	0.021	118	5591
0.034	72	2072	0.027	49	1791
0.031	59	1832	0.015	25	1618
0.018	26	1385	0.029	40	1354
0.024	48	1938	0.032	53	1606
0.032	24	715	0.04	27	645
0.022	47	2059	0.031	59	1848
0.023	51	2143	0.022	36	1597
0.023	61	2614	0.024	54	2243
0.028	63	2177	0.029	57	1917
0.024	49	2012	0.033	58	1716
0.023	54	2297	0.027	54	1947
0.028	97	3416	0.02	64	3071
0.022	43	1913	0.026	43	1616
0.015	25	1660	0.027	39	1426
0.022	76	3323	0.019	58	3019
0.034	42	1179	0.026	25	920
0.021	30	1420	0.029	40	1331
0.028	61	2116	0.022	40	1778
0.021	60	2832	0.02	53	2612
0.046	78	1630	0.046	68	1409
0.054	42	729	0.061	38	580
0.013	13	950	0.026	24	901
0.048	81	1590	0.062	88	1329
0.024	118	4757	0.022	91	3969
0.019	35	1773	0.028	43	1471
0.028	146	5081	0.025	117	4549
0.027	32	1169	0.028	28	970
0.063	179	2681	0.056	139	2336
0.034	34	952	0.024	22	891
0.028	33	1127	0.024	24	985
0.045	36	763	0.043	28	622
0.026	53	1963	0.027	49	1772
0.059	67	1077	0.069	58	778
0.024	51	2035	0.019	32	1638
0.023	46	1960	0.035	64	1783
0.059	76	1213	0.054	59	1030

0.021	21	996	0.025	23	891
0.022	98	4373	0.023	88	3675
0.024	34	1368	0.027	27	987
0.031	68	2103	0.035	58	1579
0.028	36	1267	0.022	20	909
0.087	81	854	0.098	83	761
0.078	102	1200	0.062	64	974
0.022	22	1001	0.014	11	787
0.023	45	1932	0.026	41	1531
0.029	87	2899	0.021	49	2303
0.025	82	3156	0.022	63	2761
0.024	104	4276	0.028	107	3734
0.021	21	975	0.024	19	763
0.022	55	2487	0.026	53	1982
0.023	134	5612	0.03	140	4605
0.066	36	508	0.068	30	408
0.019	27	1402	0.035	42	1157
0.023	57	2416	0.023	48	2081
0.026	78	2946	0.024	57	2334
0.025	21	815	0.023	18	765
0.059	85	1348	0.048	54	1071
0.029	57	1905	0.025	41	1602
0.057	143	2382	0.06	134	2114
0.025	34	1326	0.017	18	1023
0.082	112	1257	0.083	95	1051
0.066	73	1040	0.079	82	961
0.02	45	2179	0.023	46	1966
0.018	59	3166	0.02	54	2607
0.02	69	3426	0.023	76	3186
0.023	22	922	0.023	18	777
0.038	87	2182	0.029	58	1961
0.02	105	5181	0.028	125	4323
0.032	61	1854	0.025	37	1464
0.029	67	2234	0.038	78	1948
0.025	66	2526	0.022	52	2365
0.02	34	1651	0.023	33	1380
0.021	36	1705	0.032	50	1503
0.024	67	2745	0.025	62	2425
0.025	23	912	0.026	19	720
0.028	71	2487	0.025	49	1945

0.029	61	2061	0.025	48	1864
0.02	51	2540	0.02	41	1975
0.022	45	2035	0.028	44	1536
0.018	37	2011	0.024	43	1719
0.03	62	2007	0.038	65	1647
0.027	38	1392	0.046	56	1159
0.044	71	1527	0.049	64	1238
0.023	33	1385	0.027	29	1048
0.025	61	2396	0.027	59	2104
0.054	51	889	0.057	46	764
0.025	66	2584	0.022	53	2372
0.065	66	951	0.07	59	785
0.027	37	1348	0.026	31	1171
0.016	65	4126	0.027	96	3485
0.028	55	1915	0.031	52	1642
0.022	64	2853	0.03	77	2486
0.062	135	2057	0.048	92	1805
0.027	33	1193	0.02	19	936
0.025	37	1426	0.03	39	1246
0.229	222	746	0.228	220	747
0.013	27	1988	0.024	43	1732
0.018	35	1880	0.029	46	1538
0.024	65	2603	0.031	72	2261
0.024	53	2141	0.03	55	1808
0.026	82	3132	0.025	63	2497
0.056	68	1157	0.071	60	783
0.064	94	1383	0.074	89	1108
0.029	42	1413	0.02	24	1180
0.029	105	3532	0.032	100	2995
0.066	67	955	0.075	63	774
0.349	319	595	0.459	394	464
0.021	16	746	0.025	17	667
0.07	81	1074	0.089	84	859
0.019	12	636	0.026	14	521
0.022	116	5095	0.023	103	4413
0.023	60	2578	0.026	58	2211
0.024	121	4912	0.021	91	4236
0.056	117	1971	0.051	89	1658
0.029	75	2543	0.028	67	2304
0.023	33	1427	0.034	45	1274

0.017	30	1774	0.02	32	1578
0.078	121	1437	0.099	128	1167
0.024	77	3109	0.026	73	2708
0.022	36	1611	0.031	43	1331
0.023	39	1670	0.034	50	1410
0.05	56	1057	0.041	37	859
0.022	45	1963	0.024	41	1690
0.032	88	2692	0.024	55	2196
0.022	46	2072	0.027	48	1755
0.017	25	1433	0.037	45	1186
0.023	102	4307	0.028	100	3475
0.024	24	968	0.014	10	723
0.024	64	2624	0.018	38	2021
0.059	76	1204	0.044	41	896
0.029	125	4119	0.02	70	3345
0.019	44	2286	0.015	31	2026
0.02	19	929	0.022	20	886
0.023	56	2379	0.024	48	1923
0.025	145	5749	0.028	148	5046
0.049	39	760	0.076	50	605
0.097	70	655	0.115	73	564
0.025	43	1646	0.029	45	1485
0.031	35	1080	0.025	22	860
0.02	43	2114	0.024	41	1633
0.038	51	1297	0.031	36	1112
0.022	86	3772	0.018	59	3207
0.024	43	1777	0.03	44	1438
0.025	59	2301	0.02	38	1900
0.018	61	3369	0.023	70	2924
0.024	15	619	0.022	12	537
0.021	38	1788	0.031	50	1548
0.023	78	3318	0.03	86	2818
0.014	12	873	0.022	16	705
0.018	22	1214	0.028	30	1042
0.058	54	881	0.057	48	791
0.017	30	1786	0.023	34	1453
0.058	52	841	0.066	49	690
0.023	46	1951	0.022	34	1542
0.019	56	2831	0.029	71	2403
0.041	59	1363	0.054	67	1177

0.023	69	2972	0.028	76	2677
0.054	141	2487	0.057	135	2244
0.039	23	565	0.037	18	474
0.021	34	1552	0.03	38	1246
0.024	64	2564	0.027	61	2187
0.068	141	1930	0.049	85	1635
0.026	24	907	0.038	27	685
0.026	65	2395	0.026	51	1924
0.049	56	1081	0.05	47	886
0.027	115	4178	0.024	85	3434
0.067	42	585	0.085	46	496
0.026	35	1321	0.032	34	1044
0.023	27	1124	0.034	31	882
0.025	39	1494	0.022	28	1250
0.022	61	2758	0.023	58	2488
0.016	16	966	0.033	28	822
0.019	31	1601	0.021	26	1240
0.013	21	1551	0.024	33	1314
0.012	15	1285	0.031	33	1048
0.023	62	2614	0.026	55	2056
0.022	50	2231	0.019	40	2060
0.015	23	1470	0.023	26	1113
0.021	21	970	0.041	35	815
0.063	91	1343	0.085	103	1103
0.049	59	1134	0.064	65	955
0.695	660	290	0.808	966	229
0.022	40	1765	0.02	28	1349
0.029	51	1705	0.024	37	1525
0.019	16	835	0.028	21	733
0.021	27	1269	0.033	35	1029
0.029	41	1384	0.035	43	1175
0.021	72	3297	0.015	45	2868
0.031	50	1579	0.029	45	1488
0.022	45	2016	0.022	38	1698
0.03	42	1352	0.024	28	1137
0.119	114	845	0.173	137	655
0.588	896	627	0.695	1108	487
0.036	87	2337	0.035	70	1913
0.022	39	1760	0.028	43	1485
0.023	67	2812	0.03	77	2471

0.022	55	2435	0.025	50	1973
0.049	35	685	0.042	27	620
0.02	56	2773	0.018	45	2461
0.016	23	1434	0.027	32	1175
0.023	28	1193	0.024	26	1054
0.024	21	865	0.023	17	717
0.054	77	1356	0.056	70	1187
0.021	52	2437	0.022	48	2111
0.02	31	1509	0.027	36	1286
0.021	43	2045	0.021	38	1813
0.025	73	2854	0.027	64	2306
0.021	21	984	0.038	31	777
0.061	55	852	0.077	55	656
0.018	30	1634	0.021	26	1240
0.022	60	2668	0.023	54	2318
0.257	249	721	0.31	295	656
0.022	66	2988	0.023	61	2566
0.072	144	1853	0.111	184	1478
0.019	61	3098	0.02	54	2590
0.028	142	5014	0.025	110	4342
0.018	42	2253	0.026	46	1747
0.024	58	2409	0.024	48	1994
0.06	86	1353	0.079	104	1210
0.025	79	3090	0.028	75	2592
0.065	111	1599	0.064	83	1215
0.057	46	761	0.058	39	636
0.024	63	2600	0.031	69	2154
0.02	33	1613	0.03	44	1417
0.028	45	1575	0.017	24	1425
0.018	25	1335	0.022	27	1204
0.025	23	911	0.039	31	755
0.028	98	3451	0.031	95	3016
0.023	31	1321	0.028	31	1077
0.028	63	2205	0.033	63	1870
0.024	51	2033	0.025	46	1812
0.028	44	1524	0.026	35	1311
0.079	48	559	0.063	32	474
0.018	17	909	0.029	21	711
0.065	88	1276	0.06	67	1054
0.048	98	1941	0.046	79	1657

0.065	52	744	0.063	44	650
0.014	31	2113	0.029	51	1728
0.027	70	2540	0.021	51	2356
0.014	16	1147	0.024	22	914
0.064	46	668	0.058	34	557
0.063	89	1319	0.059	70	1108
0.025	37	1423	0.027	32	1152
0.014	10	719	0.03	21	672
0.024	123	5083	0.021	96	4371
0.021	51	2324	0.023	51	2184
0.02	24	1155	0.023	22	926
0.026	25	955	0.035	29	788
0.015	15	1005	0.026	23	847
0.059	54	868	0.078	63	742
0.027	38	1377	0.021	27	1247
0.021	68	3137	0.025	67	2636
0.026	128	4845	0.022	92	4094
0.02	58	2842	0.024	60	2390
0.024	29	1176	0.028	29	1001
0.024	62	2524	0.023	55	2369
0.024	46	1892	0.026	39	1463
0.036	95	2509	0.047	104	2103
0.056	44	738	0.056	33	554
0.044	74	1615	0.05	73	1387
0.035	34	934	0.043	39	871
0.022	36	1628	0.025	34	1334
0.069	52	697	0.048	27	530
0.02	19	923	0.023	16	682
0.027	82	2973	0.02	51	2511
0.017	25	1470	0.021	28	1285
0.027	27	991	0.026	22	809
0.068	55	750	0.098	65	595
0.016	30	1820	0.031	49	1542
0.337	168	331	0.457	266	316
0.016	11	673	0.028	17	596
0.021	61	2813	0.031	79	2488
0.018	32	1758	0.034	52	1497
0.014	10	688	0.028	17	597
0.024	41	1671	0.034	44	1244
0.058	104	1693	0.064	90	1323

0.027	24	861	0.032	21	635
0.026	67	2503	0.019	45	2338
0.03	36	1154	0.029	30	1008
0.017	9	513	0.02	9	437
0.02	25	1208	0.021	22	1015
0.025	45	1791	0.031	48	1525
0.063	127	1875	0.057	101	1665
0.024	57	2315	0.029	58	1956
0.061	68	1055	0.046	45	929
0.03	47	1528	0.032	47	1402
0.014	21	1502	0.024	33	1333
0.046	20	417	0.059	21	337
0.022	47	2112	0.023	44	1887
0.033	35	1029	0.024	20	830
0.02	29	1447	0.019	24	1264
0.124	85	601	0.154	101	555
0.023	34	1426	0.04	47	1133
0.028	118	4137	0.023	88	3660
0.023	68	2949	0.02	50	2459
0.028	51	1783	0.021	31	1473
0.05	89	1694	0.048	74	1456
0.027	35	1272	0.043	50	1122
0.018	25	1369	0.028	32	1101
0.029	53	1749	0.021	33	1513
0.026	50	1870	0.024	40	1631
0.038	15	376	0.039	14	345
0.018	59	3243	0.028	74	2596
0.072	125	1613	0.052	81	1468
0.026	50	1888	0.033	51	1507
0.031	29	917	0.037	28	739
0.029	38	1259	0.023	27	1128
0.025	30	1174	0.031	31	984
0.025	106	4070	0.025	97	3740
0.068	131	1784	0.056	91	1537
0.021	72	3277	0.03	82	2696
0.163	130	667	0.218	171	612
0.029	41	1379	0.038	42	1075
0.028	44	1543	0.035	44	1218
0.034	51	1470	0.027	35	1258
0.022	107	4679	0.027	110	3982

0.039	60	1494	0.028	34	1166
0.022	21	916	0.027	21	744
0.021	24	1116	0.026	27	1003
0.021	69	3222	0.028	78	2718
0.02	20	962	0.027	21	761
0.018	47	2596	0.022	49	2176
0.023	31	1298	0.031	38	1201
0.063	127	1905	0.075	130	1598
0.02	46	2236	0.019	35	1825
0.021	55	2618	0.026	60	2284
0.024	56	2232	0.02	35	1734
0.594	403	275	0.738	574	204
0.02	31	1500	0.019	23	1219
0.073	23	290	0.049	13	253
0.014	32	2175	0.024	44	1781
0.415	663	934	0.49	768	800
0.023	44	1876	0.03	47	1524
0.054	86	1504	0.054	68	1187
0.075	143	1760	0.11	167	1351
0.061	78	1199	0.064	69	1008
0.02	28	1355	0.015	18	1217
0.028	116	4057	0.026	96	3622
0.016	18	1142	0.042	43	979
0.028	82	2895	0.028	75	2652
0.021	75	3453	0.032	102	3110
0.02	10	486	0.055	22	376
0.03	58	1848	0.031	49	1513
0.031	68	2136	0.027	50	1773
0.026	81	3032	0.024	69	2772
0.064	49	719	0.074	52	652
0.023	20	865	0.007	5	667
0.035	35	952	0.025	21	821
0.029	92	3123	0.027	72	2573
0.027	65	2308	0.024	47	1912
0.025	51	1997	0.016	27	1658
0.055	70	1210	0.057	59	971
0.025	75	2985	0.028	68	2357
0.045	26	547	0.056	30	504
0.028	21	726	0.016	10	620
0.061	73	1128	0.052	53	969

0.02	62	3030	0.018	46	2542
0.024	87	3474	0.028	88	3038
0.023	48	2027	0.022	39	1774
0.054	51	897	0.086	64	684
0.024	74	2968	0.025	64	2516
0.021	25	1192	0.02	20	987
0.025	84	3221	0.025	63	2476
0.047	67	1368	0.062	70	1057
0.574	788	584	0.672	1028	501
0.025	31	1224	0.021	21	960
0.027	140	5126	0.02	94	4553
0.058	79	1284	0.077	83	990
0.027	72	2554	0.03	73	2336
0.027	61	2217	0.028	58	1999
0.13	146	974	0.133	115	752
0.023	65	2791	0.029	65	2203
0.038	56	1427	0.029	35	1175
0.062	102	1537	0.056	73	1237
0.04	51	1237	0.034	41	1153
0.014	11	800	0.031	19	590
0.023	59	2559	0.02	44	2131
0.068	128	1759	0.058	88	1428
0.018	61	3299	0.025	75	2905
0.028	57	1967	0.029	46	1546
0.106	200	1684	0.103	151	1309
0.062	61	922	0.096	69	649
0.025	82	3169	0.026	73	2754
0.09	51	518	0.13	66	440
0.022	37	1677	0.024	34	1379
0.03	42	1342	0.037	40	1044
0.067	53	733	0.07	52	687
0.048	83	1650	0.054	80	1394
0.022	40	1744	0.033	55	1605
0.024	65	2643	0.022	51	2276
0.029	104	3443	0.022	68	3026
0.032	86	2616	0.037	94	2434
0.163	185	953	0.188	166	718
0.03	72	2364	0.03	69	2242
0.018	42	2238	0.024	45	1822
0.048	85	1678	0.061	91	1398

0.017	43	2473	0.03	62	2018
0.027	66	2405	0.023	53	2240
0.031	56	1757	0.032	49	1491
0.02	17	851	0.02	15	734
0.028	31	1084	0.026	27	995
0.023	33	1412	0.031	37	1166
0.087	58	607	0.095	50	474
0.026	21	799	0.019	13	676
0.026	22	824	0.029	20	662
0.041	49	1132	0.02	17	825
0.032	61	1829	0.027	47	1695
0.033	29	846	0.047	38	770
0.036	56	1516	0.028	42	1450
0.034	43	1237	0.027	32	1149
0.022	53	2371	0.02	42	2043
0.016	40	2441	0.02	43	2087
0.023	36	1546	0.035	43	1203
0.029	47	1601	0.035	46	1257
0.025	20	796	0.026	17	626
0.02	28	1381	0.02	22	1087
0.022	58	2623	0.019	43	2181
0.024	34	1404	0.025	29	1117
0.07	112	1486	0.047	61	1250
0.022	57	2563	0.028	63	2185
0.021	50	2327	0.03	62	1983
0.032	79	2384	0.027	59	2163
0.016	10	626	0.041	19	447
0.022	64	2830	0.025	66	2573
0.025	92	3559	0.027	93	3322
0.028	79	2776	0.035	76	2115
0.029	74	2520	0.03	71	2277
0.027	21	753	0.022	15	661
0.1	151	1354	0.116	141	1074
0.019	38	1920	0.031	56	1760
0.027	54	1976	0.039	62	1528
0.026	49	1814	0.028	42	1481
0.042	40	916	0.043	33	727
0.029	66	2215	0.022	48	2118
0.027	67	2432	0.026	59	2252
0.026	114	4214	0.022	79	3442

0.04	18	429	0.027	11	394
0.053	91	1612	0.055	75	1291
0.023	96	4017	0.026	99	3666
0.052	58	1059	0.07	64	846
0.018	39	2148	0.028	52	1783
0.02	45	2260	0.026	50	1888
0.021	40	1871	0.031	49	1548
0.058	77	1248	0.078	85	1001
0.03	41	1305	0.029	34	1153
0.054	79	1371	0.056	66	1117
0.031	39	1233	0.021	24	1094
0.051	134	2502	0.064	137	2011
0.019	23	1165	0.029	30	1008
0.075	92	1140	0.107	112	932
0.029	68	2316	0.041	86	2027
0.024	115	4594	0.023	90	3907
0.025	56	2211	0.031	52	1651
0.02	57	2734	0.024	56	2319
0.072	72	934	0.107	85	710
0.019	27	1362	0.03	35	1132
0.128	211	1438	0.183	254	1137
0.064	80	1175	0.058	63	1025
0.053	51	919	0.073	56	708
0.076	65	794	0.133	99	644
0.024	96	3889	0.026	89	3386
0.09	128	1302	0.111	128	1030
0.018	17	904	0.04	29	689
0.026	78	2924	0.027	66	2405
0.021	21	989	0.027	25	890
0.027	22	792	0.012	9	720
0.031	49	1513	0.033	41	1199
0.023	65	2733	0.022	52	2361
0.06	113	1785	0.093	149	1459
0.066	55	775	0.055	37	641
0.034	66	1850	0.029	51	1735
0.037	42	1103	0.019	19	973
0.026	51	1937	0.023	34	1462
0.015	29	1863	0.034	51	1454
0.06	83	1295	0.085	91	984
0.02	28	1363	0.039	40	989

0.02	30	1487	0.029	40	1316
0.018	28	1507	0.022	28	1228
0.02	36	1759	0.032	46	1401
0.111	123	987	0.145	138	816
0.032	25	760	0.032	22	671
0.054	75	1302	0.053	63	1121
0.045	97	2074	0.053	93	1657
0.023	54	2251	0.03	60	1961
0.924	1195	98	0.959	1649	71
0.018	31	1655	0.019	27	1365
0.025	73	2807	0.026	60	2282
0.02	30	1457	0.025	30	1165
0.018	18	988	0.023	22	923
0.046	105	2167	0.032	60	1792
0.025	46	1789	0.025	34	1349
0.022	132	5781	0.023	118	5024
0.019	15	792	0.03	21	674
0.025	36	1399	0.02	22	1074
0.028	139	4774	0.035	138	3779
0.056	257	4307	0.049	186	3646
0.054	54	941	0.072	57	739
0.025	43	1689	0.031	47	1486
0.053	31	556	0.042	20	455
0.021	24	1138	0.026	25	954
0.016	19	1179	0.019	19	981
0.105	139	1183	0.148	160	919
0.025	39	1540	0.02	28	1369
0.435	1002	1302	0.54	1375	1172
0.027	48	1704	0.029	43	1464
0.019	56	2821	0.026	66	2432
0.02	65	3144	0.027	72	2617
0.022	60	2687	0.016	36	2180
0.046	46	964	0.04	33	798
0.02	37	1842	0.024	40	1621
0.052	25	456	0.058	23	375
0.052	42	772	0.064	44	643
0.024	65	2616	0.023	55	2334
0.02	19	953	0.038	27	678
0.02	19	908	0.029	21	708
0.025	48	1887	0.038	58	1480

0.02	43	2087	0.021	35	1601
0.042	72	1636	0.048	73	1439
0.026	55	2064	0.022	38	1710
0.025	92	3581	0.023	72	3029
0.029	75	2512	0.034	75	2136
0.024	71	2872	0.031	82	2568
0.017	21	1197	0.037	37	953
0.022	34	1511	0.03	38	1209
0.026	37	1365	0.03	36	1180
0.022	48	2097	0.026	49	1827
0.032	95	2833	0.023	59	2479
0.021	49	2289	0.032	58	1778
0.025	26	1014	0.02	17	819
0.018	28	1564	0.02	26	1247
0.023	45	1925	0.022	35	1591
0.031	50	1581	0.019	25	1316
0.028	68	2391	0.036	65	1762
0.021	59	2765	0.026	62	2319
0.028	54	1851	0.031	52	1615
0.02	45	2168	0.023	43	1818
0.059	82	1308	0.058	75	1220
0.024	31	1239	0.027	30	1092
0.025	45	1752	0.03	42	1374
0.057	78	1285	0.064	71	1047
0.034	129	3619	0.022	75	3287
0.013	29	2280	0.025	51	1955
0.018	15	806	0.025	16	628
0.102	169	1481	0.138	178	1116
0.021	44	2066	0.022	38	1668
0.075	56	687	0.067	41	575
0.02	23	1110	0.028	26	913
0.023	121	5129	0.026	112	4238
0.023	36	1551	0.022	28	1245
0.034	24	680	0.025	15	584
0.02	52	2577	0.026	54	2022
0.024	83	3387	0.021	58	2758
0.019	18	941	0.018	14	782
0.031	57	1800	0.025	40	1576
0.018	61	3411	0.028	81	2837
0.07	231	3071	0.061	167	2558

0.023	120	5004	0.022	95	4260
0.024	72	2904	0.026	63	2334
0.022	86	3767	0.034	118	3390
0.039	44	1086	0.073	71	901
0.022	39	1704	0.022	34	1494
0.02	60	2922	0.023	61	2538
0.02	16	798	0.026	18	668
0.032	107	3246	0.028	88	3008
0.036	26	695	0.022	13	589
0.102	53	469	0.13	57	380
0.022	28	1262	0.025	28	1073
0.025	11	423	0.01	4	381
0.029	32	1079	0.032	31	951
0.018	35	1867	0.022	35	1566
0.03	98	3182	0.027	81	2877
0.099	81	735	0.081	57	649
0.027	130	4690	0.022	96	4183
0.02	81	3949	0.026	93	3528
0.024	60	2447	0.036	75	1990
0.018	34	1875	0.035	55	1507
0.026	55	2099	0.027	47	1668
0.021	40	1835	0.029	47	1599
0.067	85	1191	0.08	94	1078
0.042	55	1242	0.047	52	1055
0.059	116	1856	0.079	129	1506
0.036	48	1273	0.034	39	1102
0.053	92	1644	0.061	88	1347
0.018	32	1784	0.019	28	1408
0.023	44	1845	0.025	41	1608
0.021	43	2006	0.026	49	1814
0.047	66	1326	0.074	86	1082
0.025	36	1420	0.04	50	1209
0.024	71	2902	0.02	52	2589
0.068	70	955	0.079	63	737
0.044	100	2184	0.036	75	2016
0.036	84	2244	0.024	48	1937
0.02	23	1139	0.025	23	914
0.03	43	1403	0.031	37	1153
0.033	53	1573	0.026	36	1323
0.019	42	2175	0.021	37	1748

0.023	27	1163	0.016	16	982
0.043	78	1723	0.061	96	1485
0.028	66	2320	0.023	42	1786
0.034	39	1093	0.024	23	934
0.023	61	2591	0.02	45	2214
0.028	41	1432	0.025	32	1233
0.026	92	3476	0.02	57	2788
0.062	221	3361	0.051	150	2804
0.063	69	1023	0.077	73	881
0.024	51	2078	0.029	50	1703
0.024	36	1445	0.028	33	1155
0.07	68	898	0.081	67	759
0.021	33	1573	0.027	39	1394
0.027	54	1942	0.031	57	1780
0.03	32	1044	0.034	32	923
0.03	52	1676	0.035	46	1272
0.027	120	4328	0.021	81	3771
0.019	29	1527	0.027	37	1340
0.029	100	3355	0.028	82	2809
0.066	101	1418	0.091	114	1134
0.031	83	2591	0.023	54	2342
0.024	54	2231	0.028	53	1834
0.027	95	3380	0.026	78	2875
0.027	36	1313	0.029	33	1115
0.023	58	2460	0.026	56	2103
0.021	59	2776	0.025	64	2545
0.023	40	1722	0.037	56	1463
0.038	37	933	0.029	24	800
0.022	59	2684	0.031	70	2212
0.069	61	825	0.103	83	724
0.03	64	2072	0.029	53	1759
0.021	40	1824	0.029	50	1704
0.026	81	2991	0.025	66	2627
0.021	14	652	0.029	16	527
0.024	50	2030	0.014	24	1662
0.073	96	1215	0.059	66	1048
0.024	190	7788	0.023	162	6905
0.067	51	705	0.068	41	561
0.023	130	5552	0.027	131	4792
0.074	151	1883	0.091	152	1517

0.027	25	891	0.034	24	678
0.034	66	1871	0.029	48	1606
0.019	42	2120	0.023	39	1689
0.025	21	836	0.016	12	743
0.033	37	1082	0.027	26	925
0.062	227	3411	0.058	164	2666
0.071	78	1027	0.043	37	819
0.025	45	1743	0.027	40	1454
0.048	81	1605	0.069	96	1297
0.043	138	3098	0.059	147	2329
0.075	169	2082	0.072	135	1750
0.013	16	1199	0.031	32	1005
0.024	40	1603	0.024	29	1182
0.186	119	521	0.256	156	453
0.043	43	967	0.057	51	840
0.017	67	3815	0.023	73	3087
0.121	76	550	0.109	63	516
0.03	27	871	0.018	15	835
0.024	71	2832	0.029	66	2197
0.023	54	2298	0.031	64	2004
0.085	97	1049	0.098	80	739
0.027	44	1560	0.025	32	1262
0.025	75	2980	0.027	72	2598
0.026	39	1437	0.027	34	1219
0.056	53	897	0.057	42	701
0.018	22	1196	0.011	11	1017
0.021	41	1866	0.028	45	1557
0.109	118	960	0.146	120	704
0.066	136	1910	0.065	100	1445
0.022	15	680	0.028	16	548
0.025	25	977	0.04	33	794
0.027	65	2363	0.026	52	1941
0.025	23	890	0.019	14	713
0.026	65	2465	0.02	43	2134
0.026	102	3854	0.031	105	3235
0.026	18	678	0.025	16	612
0.019	31	1622	0.028	41	1421
0.023	29	1243	0.036	39	1044
0.024	16	647	0.027	15	542
0.03	24	779	0.013	10	753

0.028	44	1528	0.04	52	1251
0.02	73	3583	0.023	73	3083
0.016	14	860	0.024	19	769
0.03	70	2274	0.027	53	1908
0.48	541	585	0.634	812	469
0.035	49	1335	0.023	28	1203
0.154	78	427	0.193	81	339
0.033	45	1306	0.038	39	1000
0.024	51	2043	0.03	55	1787
0.033	53	1534	0.043	61	1363
0.029	70	2305	0.03	66	2114
0.019	16	807	0.019	13	674
0.034	80	2277	0.023	46	1930
0.036	26	694	0.063	41	612
0.102	172	1517	0.107	153	1279
0.028	93	3197	0.033	90	2641
0.014	32	2181	0.025	48	1835
0.056	108	1834	0.066	92	1308
0.024	58	2318	0.023	46	1960
0.022	36	1600	0.024	33	1358
0.029	33	1094	0.032	32	961
0.016	31	1870	0.025	38	1501
0.024	61	2441	0.018	36	2021
0.03	32	1026	0.034	30	849
0.061	85	1316	0.041	49	1151
0.032	30	898	0.023	20	839
0.028	86	2986	0.032	89	2721
0.032	98	2945	0.029	75	2533
0.034	54	1543	0.027	37	1346
0.034	24	683	0.023	16	669
0.023	79	3358	0.026	78	2959
0.024	39	1615	0.024	34	1360
0.021	26	1222	0.031	36	1116
0.02	50	2501	0.029	66	2214
0.04	28	675	0.033	20	587
0.025	28	1110	0.044	40	867
0.02	33	1653	0.027	42	1486
0.029	63	2111	0.02	34	1701
0.017	35	1987	0.018	27	1512
0.022	80	3545	0.024	77	3133

0.02	21	1012	0.026	23	875
0.026	57	2111	0.022	41	1827
0.045	49	1038	0.07	66	883
0.021	32	1496	0.023	30	1281
0.02	46	2199	0.023	49	2056
0.021	54	2561	0.021	48	2191
0.026	73	2725	0.03	69	2260
0.022	20	874	0.028	21	726
0.443	656	826	0.546	860	714
0.026	16	606	0.016	9	558
0.028	24	838	0.043	29	641
0.026	52	1971	0.026	48	1772
0.016	12	724	0.029	20	670
0.02	34	1632	0.02	28	1405
0.027	37	1324	0.032	36	1101
0.051	80	1480	0.093	123	1197
0.024	66	2681	0.022	52	2288
0.032	69	2069	0.027	49	1748
0.058	46	744	0.059	38	609
0.072	92	1181	0.092	95	938
0.022	20	883	0.035	25	696
0.027	31	1114	0.022	20	910
0.022	63	2821	0.037	88	2316
0.04	41	980	0.088	71	739
0.056	99	1681	0.057	93	1540
0.045	82	1723	0.062	91	1385
0.022	28	1240	0.027	29	1035
0.02	45	2192	0.022	38	1715
0.029	53	1798	0.033	55	1602
0.027	25	917	0.021	16	763
0.031	86	2696	0.02	46	2216
0.024	21	871	0.031	21	666
0.029	112	3739	0.028	91	3131
0.034	50	1400	0.022	26	1147
0.036	74	1971	0.029	52	1761
0.03	134	4344	0.023	84	3520
0.054	46	799	0.073	50	633
0.024	56	2235	0.024	42	1743
0.012	13	1048	0.016	14	858
0.06	105	1651	0.046	69	1430

0.019	44	2220	0.032	61	1822
0.03	50	1621	0.035	55	1501
0.025	86	3327	0.02	62	2983
0.027	58	2115	0.032	59	1804
0.041	58	1346	0.061	71	1097
0.063	88	1313	0.09	102	1031
0.034	59	1656	0.04	56	1350
0.019	42	2203	0.021	43	2000
0.055	69	1189	0.083	92	1016
0.016	15	906	0.022	18	796
0.025	49	1874	0.022	37	1683
0.027	75	2750	0.033	77	2252
0.025	176	6743	0.023	135	5621
0.03	60	1925	0.026	43	1636
0.024	58	2334	0.025	51	2008
0.025	49	1877	0.026	47	1755
0.056	184	3093	0.056	153	2573
0.081	52	592	0.069	36	487
0.023	31	1297	0.03	34	1114
0.021	20	913	0.028	23	786
0.023	28	1213	0.026	27	1019
0.027	93	3351	0.025	81	3133
0.031	79	2465	0.03	65	2078
0.02	55	2704	0.032	73	2203
0.018	51	2827	0.024	61	2449
0.112	203	1612	0.162	240	1238
0.014	21	1481	0.02	26	1264
0.023	21	901	0.026	20	753
0.221	298	1051	0.226	295	1010
0.068	99	1366	0.079	96	1120
0.077	77	920	0.065	57	816
0.026	45	1690	0.035	55	1509
0.024	63	2509	0.018	38	2100
0.031	94	2959	0.021	56	2628
0.032	38	1167	0.031	34	1073
0.047	48	971	0.067	59	816
0.03	43	1391	0.019	26	1321
0.023	101	4288	0.024	84	3373
0.022	26	1134	0.019	20	1028
0.02	39	1897	0.025	41	1595

0.026	21	799	0.024	17	699
0.026	68	2571	0.035	77	2103
0.028	95	3252	0.03	87	2823
0.023	32	1377	0.022	24	1078
0.014	16	1105	0.029	26	880
0.023	49	2049	0.034	57	1640
0.028	87	3023	0.031	78	2404
0.066	67	942	0.046	36	744
0.028	22	759	0.03	23	732
0.049	45	870	0.067	51	712
0.03	93	2995	0.03	75	2435
0.257	389	1127	0.344	484	923
0.022	23	1002	0.013	10	768
0.03	120	3902	0.028	103	3512
0.024	51	2031	0.027	49	1773
0.042	31	699	0.102	61	538
0.021	50	2350	0.028	58	2010
0.02	39	1868	0.022	35	1525
0.026	37	1410	0.022	26	1143
0.087	92	965	0.146	117	687
0.017	25	1405	0.024	30	1201
0.059	37	585	0.029	15	510
0.023	36	1520	0.021	28	1301
0.057	68	1129	0.07	67	884
0.085	158	1706	0.086	137	1455
0.063	114	1692	0.082	114	1268
0.025	48	1872	0.023	34	1440
0.058	88	1424	0.076	91	1100
0.024	34	1362	0.022	25	1088
0.065	65	935	0.072	63	809
0.019	15	792	0.043	29	650
0.02	26	1274	0.023	30	1256
0.076	62	751	0.066	44	622
0.046	76	1564	0.036	51	1381
0.029	32	1065	0.031	26	820
0.06	66	1031	0.035	32	881
0.015	24	1610	0.03	41	1347
0.025	27	1048	0.03	25	818
0.031	24	754	0.022	15	682
0.027	81	2974	0.029	71	2396

0.021	29	1321	0.023	24	1036
0.269	355	964	0.324	378	790
0.029	30	994	0.033	25	727
0.043	67	1504	0.055	72	1238
0.073	92	1162	0.057	67	1099
0.016	47	2833	0.018	41	2198
0.05	116	2218	0.071	134	1749
0.029	62	2066	0.024	44	1761
0.027	32	1152	0.022	20	870
0.023	58	2497	0.025	59	2275
0.016	30	1880	0.018	28	1500
0.022	77	3354	0.023	65	2801
0.028	32	1105	0.043	39	877
0.024	103	4133	0.019	73	3745
0.024	44	1752	0.023	36	1515
0.064	84	1236	0.062	70	1060
0.026	60	2232	0.023	43	1864
0.059	230	3688	0.046	141	2931
0.022	57	2482	0.019	40	2064
0.028	65	2236	0.027	53	1879
0.017	46	2625	0.026	60	2261
0.035	61	1688	0.031	48	1503
0.014	28	1950	0.016	26	1552
0.025	113	4484	0.025	100	3847
0.018	44	2351	0.027	57	2032
0.044	30	658	0.057	31	509
0.027	80	2923	0.027	69	2485
0.027	18	652	0.033	19	563
0.033	52	1528	0.028	39	1342
0.023	68	2857	0.016	39	2372
0.046	67	1396	0.036	44	1191
0.023	39	1621	0.027	38	1355
0.022	96	4275	0.022	81	3620
0.031	52	1631	0.031	44	1359
0.024	47	1882	0.019	33	1727
0.023	49	2079	0.02	33	1656
0.025	88	3370	0.027	80	2857
0.024	37	1529	0.029	35	1184
0.075	96	1183	0.072	79	1012
0.049	102	1983	0.064	114	1660

0.067	61	849	0.062	44	664
0.023	102	4343	0.022	82	3692
0.064	68	996	0.07	61	809
0.024	155	6303	0.021	116	5300
0.053	81	1437	0.04	53	1270
0.028	87	3024	0.023	61	2588
0.024	75	3051	0.03	80	2558
0.05	104	1968	0.049	85	1643
0.02	18	874	0.039	27	660
0.097	180	1684	0.133	214	1392
0.049	38	735	0.052	39	707
0.023	75	3212	0.026	74	2776
0.031	31	962	0.03	27	882
0.026	70	2660	0.023	54	2282
0.026	39	1460	0.028	34	1200
0.054	99	1743	0.06	89	1394
0.023	65	2784	0.031	74	2325
0.033	57	1685	0.032	45	1381
0.02	21	1024	0.017	15	879
0.059	56	892	0.048	38	746
0.019	32	1617	0.025	35	1368
0.023	52	2197	0.027	50	1770
0.022	20	869	0.026	20	760
0.023	71	3008	0.022	59	2633
0.098	85	786	0.173	128	610
0.022	70	3091	0.03	80	2550
0.023	48	2033	0.024	47	1929
0.028	35	1220	0.03	36	1160
0.028	99	3462	0.032	96	2897
0.034	61	1709	0.024	35	1396
0.062	66	1004	0.064	56	814
0.029	51	1691	0.024	33	1370
0.083	88	975	0.141	140	854
0.014	25	1743	0.029	42	1392
0.017	21	1180	0.022	24	1059
0.025	22	873	0.013	10	767
0.061	77	1177	0.074	75	942
0.026	58	2134	0.026	44	1631
0.025	29	1121	0.019	18	914
0.027	34	1231	0.026	28	1033

0.015	26	1665	0.028	41	1444
0.021	157	7192	0.021	130	6044
0.027	42	1514	0.022	28	1251
0.367	254	439	0.483	345	369
0.059	102	1626	0.067	102	1423
0.051	125	2341	0.07	155	2056
0.022	47	2129	0.025	50	1922
0.022	38	1666	0.036	53	1433
0.027	25	888	0.046	33	688
0.263	448	1253	0.36	602	1068
0.024	66	2735	0.029	70	2342
0.059	35	554	0.046	24	495
0.052	61	1118	0.064	60	883
0.08	106	1218	0.088	96	993
0.094	41	396	0.088	35	364
0.026	81	3073	0.017	47	2712
0.453	436	526	0.537	467	402
0.084	98	1075	0.064	57	828
0.06	80	1244	0.078	83	982
0.037	33	851	0.06	48	758
0.02	40	1928	0.03	52	1707
0.06	175	2759	0.063	153	2259
0.028	32	1120	0.035	38	1040
0.025	87	3354	0.029	91	3045
0.034	49	1375	0.035	38	1034
0.026	67	2543	0.022	48	2165
0.067	86	1194	0.05	58	1093
0.024	49	2027	0.02	33	1621
0.067	61	846	0.053	42	746
0.02	21	1053	0.025	25	994
0.03	47	1530	0.022	28	1273
0.028	76	2594	0.031	73	2286
0.021	46	2122	0.015	27	1734
0.02	55	2760	0.026	62	2365
0.158	319	1701	0.185	351	1542
0.029	41	1372	0.015	20	1316
0.031	113	3570	0.024	79	3242
0.035	69	1905	0.04	73	1735
0.034	53	1513	0.019	25	1313
0.032	39	1187	0.031	30	929

0.059	185	2952	0.063	169	2521
0.033	52	1516	0.023	28	1185
0.033	88	2577	0.032	72	2184
0.033	90	2617	0.022	53	2408
0.049	31	601	0.037	19	498
0.165	115	584	0.208	125	477
0.025	25	974	0.023	19	825
0.024	140	5693	0.031	157	4977
0.021	73	3413	0.035	108	2978
0.019	20	1046	0.036	33	884
0.021	81	3751	0.023	77	3235
0.014	29	1995	0.021	37	1727
0.084	27	295	0.085	25	268
0.028	18	622	0.024	15	610
0.022	55	2444	0.024	52	2160
0.023	66	2772	0.025	60	2378
0.027	64	2314	0.028	52	1782
0.02	45	2207	0.038	74	1879
0.024	44	1787	0.034	57	1609
0.104	348	3013	0.129	347	2336
0.019	38	1979	0.027	45	1620
0.017	23	1296	0.025	28	1086
0.016	72	4384	0.019	70	3577
0.062	75	1142	0.075	76	943
0.066	180	2559	0.063	146	2155
0.051	54	999	0.043	37	824
0.022	19	834	0.01	7	704
0.027	106	3830	0.022	69	3137
0.026	55	2094	0.024	47	1884
0.023	27	1153	0.022	23	1040
0.026	38	1451	0.026	31	1164
0.066	82	1165	0.094	102	984
0.032	53	1619	0.032	47	1410
0.024	37	1526	0.028	37	1273
0.022	94	4120	0.024	78	3234
0.021	26	1184	0.027	27	983
0.024	66	2707	0.018	48	2590
0.062	143	2151	0.077	140	1688
0.022	32	1446	0.03	36	1179
0.026	57	2147	0.022	43	1872

0.016	25	1501	0.019	25	1264
0.026	51	1884	0.02	34	1694
0.04	62	1490	0.066	82	1169
0.025	41	1569	0.024	34	1394
0.024	30	1197	0.041	43	996
0.098	83	767	0.096	62	582
0.069	82	1102	0.074	71	892
0.023	35	1460	0.022	32	1449
0.026	75	2789	0.017	42	2422
0.017	20	1165	0.021	17	812
0.03	113	3655	0.026	83	3057
0.023	118	4934	0.018	77	4253
0.024	58	2399	0.019	42	2120
0.025	69	2662	0.042	90	2058
0.021	67	3081	0.022	62	2805
0.018	35	1948	0.032	52	1574
0.025	31	1234	0.015	17	1080
0.026	33	1213	0.023	24	1025
0.028	41	1418	0.024	28	1134
0.026	39	1471	0.024	29	1183
0.026	64	2361	0.026	51	1902
0.019	31	1575	0.025	33	1266
0.059	80	1266	0.073	88	1116
0.017	20	1123	0.035	32	895
0.021	97	4477	0.026	108	4023
0.024	56	2260	0.025	48	1906
0.035	45	1240	0.023	27	1130
0.059	73	1174	0.071	72	938
0.025	24	921	0.031	25	793
0.035	60	1670	0.017	25	1412
0.021	38	1805	0.02	32	1568
0.023	77	3239	0.025	76	2978
0.019	31	1579	0.025	34	1344
0.029	29	957	0.033	26	762
0.026	53	2024	0.017	31	1792
0.024	41	1642	0.028	39	1350
0.079	234	2717	0.097	253	2353
0.022	70	3096	0.029	75	2483
0.033	29	850	0.029	23	784
0.032	60	1789	0.037	56	1452

0.019	52	2643	0.024	60	2425
0.019	52	2616	0.033	70	2026
0.023	45	1935	0.026	42	1561
0.024	33	1328	0.022	24	1091
0.051	119	2226	0.056	106	1798
0.021	56	2660	0.024	59	2370
0.023	28	1209	0.028	28	977
0.027	128	4641	0.031	128	3970
0.062	63	948	0.069	59	792
0.068	226	3077	0.099	263	2401
0.02	7	348	0.073	23	290
0.068	51	704	0.072	41	525
0.022	97	4398	0.017	65	3736
0.02	28	1395	0.022	25	1113
0.025	46	1814	0.031	49	1517
0.03	45	1450	0.043	50	1116
0.064	82	1190	0.076	80	971
0.027	24	872	0.033	24	713
0.066	228	3216	0.07	197	2608
0.023	47	2002	0.019	35	1798
0.029	51	1687	0.03	43	1381
0.031	37	1167	0.034	33	935
0.02	82	3921	0.025	84	3320
0.028	35	1194	0.019	20	1023
0.031	87	2730	0.034	81	2304
0.018	15	820	0.016	11	657
0.019	42	2148	0.027	47	1665
0.029	41	1360	0.039	46	1136
0.022	55	2502	0.025	50	1923
0.02	27	1343	0.027	33	1171
0.031	101	3172	0.026	74	2805
0.022	52	2324	0.026	54	2060
0.036	59	1580	0.027	35	1265
0.062	74	1120	0.052	55	1003
0.019	21	1070	0.025	22	869
0.052	162	2963	0.056	142	2387
0.024	42	1742	0.026	37	1413
0.013	26	1986	0.025	40	1539
0.021	86	3956	0.024	80	3318
0.017	18	1036	0.03	28	909

0.027	24	859	0.035	25	681
0.066	69	970	0.059	53	851
0.021	82	3887	0.028	95	3295
0.175	214	1010	0.266	273	753
0.025	101	4020	0.024	82	3357
0.027	78	2844	0.025	63	2506
0.022	63	2852	0.02	50	2412
0.03	81	2659	0.025	59	2264
0.04	53	1280	0.063	75	1108
0.041	62	1464	0.023	30	1284
0.023	23	973	0.028	24	823
0.026	139	5136	0.031	148	4620
0.053	58	1028	0.066	65	923
0.024	51	2081	0.037	68	1779
0.023	30	1281	0.026	31	1170
0.03	84	2699	0.029	65	2156
0.084	38	412	0.073	27	341
0.045	104	2187	0.056	111	1887
0.022	48	2143	0.026	50	1840
0.025	16	624	0.018	9	483
0.053	35	631	0.038	22	555
0.034	34	965	0.017	14	834
0.086	110	1163	0.063	65	973
0.02	27	1337	0.025	29	1135
0.029	82	2710	0.027	67	2458
0.019	37	1921	0.018	30	1614
0.033	39	1144	0.028	28	970
0.028	69	2423	0.032	71	2141
0.016	37	2228	0.024	47	1930
0.025	62	2375	0.03	66	2101
0.023	31	1301	0.029	29	968
0.071	121	1591	0.057	79	1305
0.044	35	754	0.083	50	549
0.024	18	732	0.028	17	596
0.027	40	1445	0.034	44	1250
0.027	26	923	0.028	20	707
0.033	38	1110	0.036	34	922
0.053	71	1257	0.057	65	1079
0.028	44	1553	0.032	42	1285
0.091	161	1612	0.112	171	1354

0.023	48	2055	0.031	55	1732
0.013	10	736	0.027	16	578
0.03	39	1258	0.025	30	1164
0.021	38	1778	0.026	40	1489
0.024	34	1358	0.026	29	1092
0.031	40	1268	0.032	35	1070
0.029	36	1225	0.022	24	1049
0.054	84	1461	0.044	57	1233
0.022	46	2014	0.031	58	1795
0.024	37	1497	0.023	31	1342
0.023	41	1747	0.025	36	1397
0.025	24	920	0.01	8	821
0.028	77	2703	0.028	67	2353
0.06	51	794	0.059	45	717
0.018	16	864	0.028	21	718
0.024	31	1258	0.025	27	1069
0.017	79	4515	0.026	102	3776
0.04	52	1259	0.062	65	979
0.02	55	2725	0.017	41	2400
0.023	47	2007	0.018	32	1738
0.024	23	928	0.036	30	803
0.021	34	1612	0.026	39	1433
0.04	81	1953	0.054	96	1689
0.03	61	1967	0.027	50	1827
0.938	1058	70	0.964	1543	57
0.026	37	1409	0.023	29	1210
0.068	114	1569	0.069	87	1168
0.019	47	2443	0.018	37	2049
0.022	42	1874	0.033	50	1487
0.021	70	3270	0.024	64	2590
0.031	95	2972	0.034	80	2286
0.027	51	1866	0.024	41	1638
0.055	96	1635	0.055	88	1507
0.014	9	645	0.023	12	519
0.023	51	2170	0.024	44	1812
0.035	112	3075	0.026	74	2738
0.075	129	1580	0.057	80	1321
0.04	32	771	0.031	22	695
0.029	71	2371	0.024	53	2178
0.024	117	4684	0.024	94	3784

0.024	66	2740	0.022	55	2399
0.028	68	2350	0.025	53	2064
0.023	46	1942	0.025	41	1594
0.14	121	744	0.158	128	682
0.029	49	1634	0.027	42	1512
0.023	17	737	0.016	10	627
0.022	40	1800	0.027	40	1420
0.026	23	861	0.023	17	716
0.019	76	3904	0.021	73	3428
0.062	142	2147	0.046	87	1798
0.028	43	1518	0.022	29	1271
0.027	39	1402	0.019	23	1161
0.052	51	923	0.064	49	712
0.02	25	1229	0.02	19	937
0.02	29	1442	0.02	24	1161
0.025	92	3569	0.027	79	2830
0.027	56	1989	0.035	65	1767
0.02	51	2536	0.03	60	1957
0.024	53	2131	0.031	61	1931
0.073	88	1121	0.076	78	952
0.056	68	1151	0.053	59	1045
0.025	51	1986	0.029	47	1599
0.017	27	1567	0.028	36	1231
0.031	42	1292	0.033	37	1085
0.062	61	919	0.066	62	871
0.034	39	1092	0.03	26	848
0.023	46	1943	0.029	49	1621
0.022	35	1547	0.023	33	1394
0.022	45	2001	0.027	47	1685
0.023	33	1412	0.03	35	1124
0.03	142	4535	0.025	105	4175
0.024	46	1848	0.025	42	1623
0.035	56	1553	0.033	44	1292
0.027	61	2218	0.032	57	1749
0.024	69	2833	0.019	44	2293
0.029	23	760	0.028	19	670
0.024	87	3482	0.034	101	2861
0.022	25	1102	0.037	37	959
0.022	27	1178	0.014	14	965
0.454	728	875	0.52	899	831

0.229	312	1053	0.338	456	895
0.029	104	3527	0.025	75	2870
0.047	48	965	0.059	51	813
0.02	102	4948	0.023	94	4009
0.021	48	2275	0.029	57	1898
0.072	97	1247	0.069	87	1165
0.027	66	2396	0.021	47	2172
0.017	12	692	0.029	18	599
0.025	13	498	0.044	20	435
0.026	32	1180	0.029	25	842
0.025	155	6146	0.024	138	5505
0.019	25	1319	0.026	29	1068
0.022	85	3813	0.027	91	3274
0.021	37	1763	0.024	36	1491
0.024	23	934	0.033	28	821
0.045	75	1584	0.071	92	1200
0.061	45	688	0.055	37	630
0.035	72	1977	0.034	64	1818
0.023	70	2918	0.032	83	2488
0.019	23	1161	0.021	19	890
0.024	63	2548	0.018	37	2045
0.02	79	3829	0.021	72	3338
0.033	128	3766	0.031	103	3234
0.059	50	800	0.069	51	689
0.06	115	1791	0.043	68	1529
0.046	55	1132	0.057	56	928
0.03	73	2355	0.025	55	2115
0.019	29	1479	0.024	30	1195
0.024	75	3023	0.025	67	2563
0.029	26	864	0.052	39	708
0.023	35	1513	0.025	35	1362
0.031	56	1748	0.032	50	1492
0.025	44	1737	0.026	40	1491
0.023	54	2344	0.035	65	1813
0.025	18	697	0.018	11	617
0.024	51	2039	0.019	35	1812
0.022	36	1600	0.032	43	1300
0.021	15	693	0.031	18	561
0.026	64	2375	0.027	55	1977
0.015	16	1038	0.023	20	847

0.063	39	582	0.094	41	397
0.026	21	800	0.019	14	726
0.018	32	1788	0.034	57	1624
0.029	17	567	0.04	22	524
0.059	70	1120	0.067	67	933
0.067	98	1359	0.073	98	1238
0.034	49	1413	0.037	45	1167
0.064	94	1370	0.05	68	1284
0.027	53	1897	0.031	51	1580
0.024	43	1750	0.039	57	1390
0.02	48	2332	0.02	39	1867
0.018	8	429	0.026	9	338
0.025	60	2379	0.019	38	1987
0.062	81	1235	0.052	58	1066
0.019	32	1669	0.026	37	1392
0.062	92	1381	0.072	95	1228
0.021	33	1564	0.026	39	1451
0.047	53	1067	0.085	84	910
0.046	49	1019	0.052	49	894
0.026	124	4575	0.028	108	3800
0.024	26	1055	0.019	16	848
0.027	42	1511	0.015	20	1346
0.035	79	2149	0.034	63	1781
0.056	74	1245	0.076	93	1127
0.054	98	1714	0.053	87	1546
0.03	31	1014	0.032	26	797
0.022	29	1263	0.026	24	905
0.068	55	749	0.094	62	596
0.02	81	3923	0.016	57	3410
0.023	45	1905	0.023	37	1599
0.022	27	1219	0.028	30	1031
0.024	77	3139	0.027	69	2517
0.061	149	2295	0.051	96	1802
0.053	108	1918	0.049	76	1485
0.029	49	1659	0.019	29	1477
0.021	31	1425	0.035	50	1388
0.026	43	1618	0.031	40	1264
0.021	58	2718	0.016	39	2345
0.026	51	1892	0.026	40	1471
0.033	46	1338	0.032	39	1175

0.02	22	1064	0.022	19	834
0.024	66	2662	0.026	65	2428
0.034	21	596	0.03	17	554
0.026	44	1674	0.023	34	1467
0.022	23	1023	0.017	13	773
0.099	91	826	0.131	95	628
0.02	62	2967	0.029	74	2490
0.024	59	2445	0.029	65	2146
0.029	70	2342	0.034	67	1912
0.029	43	1453	0.023	31	1306
0.019	8	410	0.019	6	318
0.079	32	371	0.1	37	332
0.019	34	1768	0.034	51	1462
0.024	40	1615	0.028	40	1412
0.025	51	1988	0.028	49	1704
0.034	79	2250	0.027	54	1965
0.017	48	2789	0.024	55	2276
0.025	22	872	0.024	19	766
0.021	57	2655	0.019	41	2117
0.026	79	2972	0.02	50	2414
0.039	45	1115	0.051	52	975
0.034	102	2890	0.033	79	2284
0.017	26	1476	0.037	44	1144
0.023	38	1600	0.024	29	1168
0.017	64	3675	0.019	58	3014
0.044	88	1911	0.046	77	1594
0.023	42	1778	0.029	44	1489
0.025	60	2367	0.02	39	1925
0.032	76	2276	0.025	52	2069
0.067	68	946	0.098	82	751
0.028	39	1340	0.032	37	1137
0.024	63	2611	0.029	72	2453
0.11	141	1145	0.15	183	1036
0.052	113	2070	0.054	106	1860
0.059	178	2847	0.053	135	2394
0.027	70	2563	0.019	43	2180
0.017	23	1341	0.02	23	1138
0.03	49	1603	0.033	40	1187
0.021	24	1111	0.033	33	975
0.022	29	1303	0.046	50	1045

0.022	43	1874	0.042	61	1408
0.034	69	1968	0.028	48	1666
0.025	97	3712	0.016	54	3314
0.046	45	928	0.056	49	822
0.022	26	1168	0.018	16	866
0.02	37	1859	0.015	26	1701
0.031	26	805	0.011	7	618
0.031	98	3097	0.031	93	2881
0.081	120	1364	0.07	81	1076
0.025	54	2111	0.023	42	1810
0.053	146	2620	0.049	120	2308
0.058	125	2041	0.054	103	1788
0.127	82	562	0.157	97	522
0.02	44	2162	0.02	33	1655
0.029	84	2861	0.03	74	2388
0.025	65	2532	0.027	59	2139
0.024	63	2606	0.019	46	2374
0.058	64	1041	0.088	78	808
0.019	43	2211	0.022	42	1827
0.019	38	1993	0.023	41	1736
0.023	88	3749	0.031	102	3141
0.023	50	2171	0.028	54	1885
0.053	53	939	0.069	54	724
0.058	109	1763	0.057	85	1409
0.059	93	1481	0.066	87	1239
0.02	48	2345	0.021	43	1981
0.071	50	656	0.045	30	634
0.058	45	732	0.075	56	690
0.023	39	1652	0.027	39	1397
0.02	30	1444	0.027	38	1351
0.028	55	1943	0.02	33	1607
0.026	37	1393	0.023	27	1145
0.018	50	2677	0.02	45	2197
0.099	169	1543	0.128	161	1100
0.018	27	1480	0.023	29	1249
0.023	131	5519	0.02	98	4855
0.026	18	687	0.024	14	563
0.059	285	4585	0.057	228	3765
0.033	24	707	0.021	12	565
0.026	55	2060	0.036	63	1665

0.022	37	1634	0.041	64	1486
0.03	74	2412	0.03	62	1994
0.071	82	1073	0.097	96	895
0.027	70	2486	0.024	51	2080
0.026	35	1315	0.024	27	1091
0.024	59	2424	0.018	41	2196
0.026	75	2859	0.027	66	2358
0.028	22	773	0.04	27	647
0.024	36	1477	0.03	37	1188
0.029	119	3939	0.027	93	3297
0.016	21	1296	0.015	17	1138
0.033	67	1992	0.033	56	1638
0.019	63	3192	0.023	62	2651
0.017	44	2540	0.019	43	2223
0.021	107	4942	0.019	87	4415
0.033	47	1362	0.035	37	1010
0.027	55	1963	0.022	38	1722
0.025	65	2546	0.02	47	2253
0.017	18	1059	0.043	39	869
0.025	81	3134	0.021	52	2399
0.026	31	1146	0.02	20	982
0.022	35	1564	0.026	37	1365
0.06	68	1074	0.098	93	859
0.025	52	1991	0.028	46	1582
0.059	95	1517	0.057	79	1318
0.027	37	1313	0.016	21	1263
0.067	28	393	0.053	16	285
0.077	62	742	0.077	51	614
0.027	33	1190	0.026	28	1062
0.021	84	3860	0.022	73	3274
0.03	35	1137	0.017	16	910
0.024	49	2025	0.018	33	1831
0.021	51	2326	0.037	81	2098
0.018	23	1239	0.035	43	1179
0.028	86	2951	0.02	51	2522
0.016	44	2779	0.023	54	2271
0.017	45	2553	0.024	55	2197
0.022	50	2195	0.027	52	1876
0.025	57	2212	0.025	48	1885
0.025	48	1836	0.036	58	1548

0.056	48	813	0.044	31	671
0.025	53	2091	0.025	48	1845
0.015	52	3373	0.029	85	2880
0.018	31	1685	0.013	17	1273
0.046	31	645	0.064	35	512
0.021	10	460	0.041	18	416
0.02	73	3611	0.02	63	3013
0.035	41	1136	0.025	25	974
0.024	103	4262	0.02	79	3829
0.025	58	2279	0.031	62	1969
0.06	124	1960	0.075	121	1496
0.017	30	1687	0.021	30	1393
0.02	34	1686	0.025	35	1377
0.028	22	776	0.027	19	690
0.063	63	935	0.117	99	750
0.035	57	1584	0.022	29	1280
0.03	30	980	0.031	27	849
0.047	41	828	0.063	50	745
0.063	90	1350	0.076	82	1000
0.023	50	2144	0.027	49	1745
0.121	93	673	0.143	94	563
0.019	16	840	0.021	15	697
0.06	83	1308	0.052	61	1121
0.021	29	1346	0.024	30	1197
0.021	26	1207	0.025	28	1080
0.02	43	2139	0.023	40	1726
0.026	44	1617	0.029	43	1442
0.064	44	645	0.078	41	483
0.029	66	2172	0.028	52	1808
0.035	128	3541	0.036	110	2937
0.022	41	1782	0.029	43	1448
0.113	74	579	0.137	80	505
0.025	36	1410	0.035	43	1171
0.028	78	2723	0.027	63	2241
0.053	92	1646	0.064	88	1290
0.102	90	789	0.111	82	657
0.023	75	3232	0.021	60	2754
0.019	23	1163	0.032	36	1104
0.019	91	4657	0.025	102	3924
0.044	56	1231	0.065	68	986

0.067	51	711	0.06	39	609
0.028	36	1255	0.032	34	1015
0.055	75	1295	0.042	51	1171
0.067	55	771	0.079	49	573
0.017	38	2200	0.022	42	1834
0.111	237	1901	0.097	166	1544
0.016	22	1358	0.032	34	1045
0.019	59	2975	0.024	62	2489
0.072	95	1222	0.061	67	1032
0.04	87	2072	0.052	92	1690
0.024	62	2482	0.024	50	2022
0.018	30	1593	0.025	35	1392
0.052	92	1661	0.041	57	1350
0.018	29	1551	0.026	32	1194
0.027	24	859	0.04	29	700
0.024	35	1407	0.028	36	1239
0.032	44	1319	0.014	16	1120
0.022	73	3301	0.026	75	2851
0.025	26	1032	0.027	26	922
0.046	72	1484	0.06	77	1200
0.03	139	4504	0.035	136	3774
0.065	78	1122	0.058	57	920
0.052	54	981	0.059	59	945
0.052	43	786	0.048	36	709
0.06	85	1331	0.065	78	1118
0.022	44	1998	0.032	55	1682
0.045	61	1297	0.054	62	1080
0.014	12	833	0.037	27	695
0.034	49	1392	0.037	43	1106
0.051	74	1367	0.051	65	1207
0.054	84	1466	0.074	97	1217
0.082	43	480	0.069	32	431
0.032	24	733	0.029	17	568
0.027	35	1252	0.023	27	1148
0.057	77	1284	0.074	86	1082
0.022	46	2009	0.016	30	1807
0.027	56	1995	0.024	42	1682
0.066	99	1394	0.093	119	1164
0.064	93	1367	0.057	65	1081
0.024	44	1769	0.018	29	1548

0.029	79	2683	0.024	55	2205
0.024	21	863	0.029	21	711
0.017	18	1046	0.023	21	903
0.023	34	1463	0.027	32	1167
0.033	44	1305	0.034	36	1034
0.024	26	1055	0.023	20	846
0.026	96	3639	0.024	75	3083
0.034	38	1067	0.033	34	984
0.084	67	727	0.067	40	553
0.032	65	1980	0.02	33	1612
0.031	59	1846	0.025	39	1535
0.021	126	5965	0.025	125	4888
0.032	78	2327	0.023	46	1961
0.056	82	1376	0.064	75	1093
0.031	19	599	0.028	14	492
0.033	103	3023	0.035	86	2405
0.032	18	550	0.024	9	370
0.054	138	2432	0.05	114	2161
0.022	47	2081	0.028	50	1705
0.018	35	1947	0.022	37	1650
0.019	8	421	0.011	4	346
0.017	29	1633	0.028	42	1442
0.048	101	2001	0.057	103	1699
0.023	35	1474	0.023	27	1135
0.021	29	1343	0.018	19	1035
0.034	49	1410	0.026	31	1149
0.05	51	975	0.052	47	858
0.027	88	3173	0.021	55	2609
0.012	15	1212	0.03	29	942
0.026	57	2109	0.024	39	1612
0.032	49	1467	0.023	31	1318
0.059	41	659	0.072	47	603
0.601	1145	760	0.698	1413	611
0.061	125	1921	0.072	118	1520
0.021	37	1721	0.024	33	1341
0.019	20	1028	0.02	19	916
0.025	61	2349	0.025	46	1780
0.022	50	2212	0.022	41	1861
0.022	69	3033	0.025	66	2620
0.026	125	4760	0.029	123	4131

0.109	167	1368	0.121	158	1143
0.022	48	2103	0.023	46	1980
0.021	46	2120	0.016	29	1742
0.023	51	2175	0.028	49	1717
0.027	53	1905	0.022	37	1654
0.026	122	4591	0.024	97	3935
0.026	63	2399	0.025	52	2005
0.017	27	1540	0.025	33	1305
0.027	28	998	0.024	22	907
0.027	13	467	0.048	20	399
0.071	129	1697	0.084	126	1372
0.031	91	2892	0.035	85	2372
0.016	43	2583	0.019	43	2233
0.025	42	1650	0.025	34	1306
0.063	148	2201	0.057	112	1847
0.036	104	2819	0.023	59	2537
0.027	57	2050	0.024	45	1847
0.057	43	713	0.08	51	588
0.017	23	1330	0.025	27	1054
0.019	35	1839	0.025	42	1628
0.024	66	2654	0.033	78	2303
0.023	92	3988	0.022	79	3497
0.025	60	2371	0.027	57	2079
0.091	120	1192	0.112	108	859
0.069	102	1383	0.065	81	1160
0.184	215	951	0.238	250	799
0.024	78	3210	0.027	73	2671
0.028	76	2592	0.028	57	1974
0.023	17	714	0.032	22	659
0.015	22	1410	0.029	39	1291
0.02	45	2234	0.024	48	1991
0.018	42	2318	0.024	46	1897
0.028	28	972	0.033	29	861
0.026	99	3735	0.028	82	2889
0.017	30	1760	0.03	46	1486
0.026	91	3409	0.022	63	2744
0.024	64	2617	0.031	72	2219
0.022	82	3722	0.023	75	3249
0.019	42	2194	0.028	51	1784
0.025	57	2238	0.03	58	1868

0.028	101	3538	0.02	64	3217
0.02	15	737	0.015	10	638
0.031	29	893	0.026	17	643
0.03	31	1008	0.035	26	727
0.02	62	3047	0.025	67	2593
0.062	77	1170	0.031	33	1043
0.066	61	860	0.069	61	827
0.839	1930	371	0.89	2642	328
0.026	37	1377	0.019	23	1199
0.062	74	1117	0.072	74	959
0.02	23	1116	0.023	20	846
0.021	35	1626	0.027	35	1271
0.02	29	1391	0.022	25	1132
0.029	106	3586	0.025	74	2934
0.023	21	910	0.034	27	771
0.071	99	1297	0.055	70	1205
0.036	54	1454	0.026	38	1428
0.02	30	1445	0.032	40	1193
0.026	64	2404	0.017	35	2047
0.016	41	2587	0.025	57	2258
0.099	69	628	0.109	67	549
0.473	435	485	0.627	655	389
0.023	43	1793	0.027	42	1536
0.022	39	1709	0.019	28	1445
0.022	97	4389	0.023	89	3744
0.09	106	1075	0.112	108	858
0.069	78	1055	0.089	87	887
0.018	15	840	0.024	15	618
0.027	83	3013	0.026	75	2776
0.021	87	3981	0.02	70	3385
0.107	179	1494	0.124	188	1327
0.03	52	1690	0.026	38	1443
0.018	46	2558	0.021	47	2173
0.659	693	358	0.772	916	271
0.009	6	641	0.024	13	522
0.058	71	1163	0.067	71	990
0.026	49	1825	0.026	41	1553
0.023	29	1247	0.02	22	1102
0.02	46	2301	0.026	56	2061
0.055	155	2653	0.049	108	2104

0.022	45	2036	0.03	52	1697
0.09	102	1032	0.132	124	817
0.023	50	2122	0.034	61	1738
0.021	89	4125	0.023	79	3383
0.637	1247	712	0.707	1487	615
0.027	11	400	0.025	9	354
0.05	93	1756	0.056	83	1388
0.023	28	1185	0.023	22	954
0.057	72	1187	0.044	49	1075
0.021	51	2323	0.028	56	1976
0.022	31	1394	0.017	22	1311
0.027	49	1754	0.028	44	1542
0.031	71	2242	0.026	48	1801
0.027	102	3724	0.03	94	3090
0.022	46	2091	0.027	45	1599
0.022	28	1219	0.022	23	1021
0.029	33	1091	0.035	32	877
0.029	46	1562	0.025	35	1371
0.025	71	2799	0.028	70	2408
0.034	81	2304	0.034	68	1954
0.027	32	1166	0.032	31	931
0.027	25	898	0.019	14	722
0.023	59	2479	0.018	36	1956
0.085	135	1455	0.103	134	1162
0.021	81	3687	0.024	79	3187
0.026	36	1336	0.022	27	1184
0.021	42	1963	0.02	38	1834
0.066	156	2210	0.053	100	1783
0.023	44	1831	0.02	28	1393
0.018	38	2033	0.03	53	1710
0.022	116	5149	0.022	99	4496
0.064	72	1057	0.044	47	1029
0.021	86	4079	0.023	79	3287
0.025	97	3761	0.027	91	3273
0.057	73	1213	0.055	56	965
0.02	69	3359	0.022	68	2981
0.065	83	1188	0.066	75	1058
0.031	72	2257	0.022	44	2000
0.02	12	574	0.026	14	518
0.026	42	1591	0.042	56	1274

0.02	22	1072	0.02	19	939
0.069	69	926	0.093	81	792
0.012	6	483	0.034	16	453
0.02	17	817	0.026	18	671
0.019	28	1482	0.028	35	1211
0.032	27	830	0.067	46	639
0.027	35	1244	0.028	29	1013
0.026	93	3468	0.017	50	2898
0.019	31	1644	0.028	42	1483
0.054	108	1884	0.072	116	1500
0.027	45	1649	0.03	43	1383
0.021	27	1244	0.035	40	1107
0.686	1284	587	0.796	1819	465
0.029	61	2015	0.021	37	1706
0.12	120	883	0.168	154	762
0.024	45	1795	0.028	44	1543
0.025	18	690	0.036	22	595
0.031	52	1623	0.026	36	1366
0.026	30	1146	0.028	27	932
0.053	93	1676	0.059	87	1393
0.022	51	2273	0.023	41	1738
0.021	40	1836	0.027	42	1494
0.013	18	1321	0.015	15	999
0.096	85	802	0.092	64	631
0.032	47	1442	0.039	47	1150
0.018	22	1185	0.027	28	994
0.185	173	763	0.215	168	615
0.025	55	2104	0.027	54	1977
0.031	16	492	0.032	13	390
0.031	67	2127	0.037	72	1862
0.029	56	1893	0.031	52	1644
0.025	38	1462	0.024	28	1137
0.034	38	1081	0.03	30	969
0.025	82	3141	0.029	74	2449
0.022	61	2652	0.02	45	2153
0.019	17	869	0.02	13	634
0.024	31	1245	0.019	21	1074
0.058	176	2868	0.057	148	2450
0.081	177	2005	0.099	172	1569
0.025	20	776	0.02	13	633

0.035	48	1336	0.031	39	1201
0.029	48	1629	0.022	30	1349
0.021	48	2186	0.033	61	1798
0.048	46	917	0.061	49	755
0.019	21	1095	0.027	29	1044
0.075	75	926	0.063	54	798
0.025	62	2385	0.023	48	2075
0.029	52	1711	0.027	36	1309
0.032	47	1418	0.021	24	1093
0.104	148	1275	0.091	112	1119
0.052	70	1274	0.072	83	1077
0.029	46	1562	0.023	36	1515
0.022	35	1557	0.035	48	1306
0.022	36	1589	0.026	35	1313
0.066	164	2316	0.052	114	2089
0.021	180	8338	0.022	156	7029
0.026	46	1729	0.028	42	1446
0.024	68	2754	0.025	58	2276
0.029	66	2175	0.026	52	1986
0.09	55	556	0.06	28	442
0.025	60	2341	0.033	67	1971
0.024	74	2967	0.026	68	2579
0.052	135	2478	0.055	115	1990
0.02	53	2662	0.022	53	2322
0.023	22	936	0.019	16	809
0.024	43	1745	0.025	38	1510
0.019	15	786	0.027	18	661
0.024	45	1793	0.028	40	1386
0.025	58	2260	0.029	58	1947
0.077	56	671	0.092	51	501
0.06	55	868	0.065	54	777
0.052	59	1069	0.066	70	986
0.051	62	1142	0.047	46	926
0.023	53	2299	0.02	40	1941
0.021	81	3746	0.026	81	3042
0.015	12	767	0.032	23	689
0.026	60	2255	0.03	58	1900
0.029	56	1886	0.031	50	1585
0.05	93	1773	0.057	85	1417
0.024	21	838	0.023	18	750

0.052	56	1023	0.05	42	791
0.032	33	1009	0.027	21	754
0.023	18	749	0.029	20	670
0.016	71	4248	0.023	83	3529
0.023	42	1794	0.023	38	1586
0.022	38	1694	0.022	34	1500
0.022	39	1727	0.03	41	1339
0.033	69	2048	0.024	43	1786
0.015	51	3352	0.023	69	2941
0.029	64	2153	0.027	59	2105
0.025	50	1924	0.03	49	1567
0.077	49	587	0.053	27	480
0.025	50	1948	0.028	48	1692
0.019	40	2108	0.02	37	1789
0.028	58	2019	0.039	61	1517
0.023	66	2845	0.026	60	2259
0.103	129	1127	0.117	134	1015
0.056	60	1014	0.059	57	903
0.021	15	686	0.051	29	537
0.061	177	2725	0.054	132	2326
0.03	46	1501	0.036	49	1297
0.032	58	1728	0.024	35	1429
0.016	31	1911	0.019	33	1705
0.031	127	4035	0.02	72	3551
0.1	251	2252	0.127	254	1752
0.027	33	1211	0.035	42	1153
0.018	45	2505	0.024	52	2078
0.027	18	660	0.024	15	622
0.088	124	1280	0.12	127	928
0.024	91	3690	0.029	84	2815
0.02	46	2227	0.021	42	1964
0.021	42	1970	0.024	39	1616
0.026	51	1886	0.027	42	1511
0.028	32	1126	0.026	26	978
0.021	71	3383	0.028	79	2772
0.024	51	2046	0.027	45	1617
0.029	72	2374	0.025	54	2082
0.025	40	1549	0.03	43	1403
0.026	53	1950	0.033	52	1521
0.022	32	1422	0.019	23	1213

0.023	64	2679	0.029	71	2354
0.025	11	435	0.018	7	383
0.018	55	2992	0.019	51	2642
0.023	44	1862	0.021	35	1624
0.028	38	1343	0.037	41	1070
0.025	113	4402	0.026	104	3841
0.024	31	1248	0.021	23	1053
0.362	359	632	0.47	476	537
0.023	86	3630	0.029	91	3031
0.029	50	1647	0.027	39	1409
0.038	78	1965	0.047	82	1665
0.912	1401	136	0.951	1814	93
0.023	28	1181	0.023	23	994
0.021	38	1765	0.027	38	1382
0.022	60	2657	0.021	51	2432
0.026	54	2018	0.026	45	1690
0.029	23	758	0.027	20	726
0.031	62	1925	0.026	46	1711
0.07	97	1296	0.055	64	1106
0.03	24	788	0.025	17	667
0.029	59	1993	0.028	46	1584
0.023	42	1749	0.026	42	1544
0.017	26	1460	0.02	26	1299
0.023	37	1548	0.03	37	1210
0.024	66	2667	0.026	62	2337
0.026	17	638	0.019	11	568
0.029	82	2784	0.024	53	2155
0.021	60	2852	0.019	50	2587
0.042	63	1430	0.033	48	1385
0.024	38	1578	0.019	23	1194
0.024	70	2859	0.028	69	2378
0.069	77	1035	0.094	91	878
0.026	26	967	0.03	23	740
0.021	39	1835	0.026	40	1485
0.028	66	2328	0.027	53	1933
0.057	64	1050	0.057	53	876
0.022	87	3925	0.03	103	3312
0.018	23	1226	0.024	25	1024
0.05	54	1033	0.044	37	796
0.028	77	2626	0.033	75	2228

0.07	76	1013	0.081	73	830
0.02	87	4301	0.017	66	3775
0.031	24	755	0.032	22	657
0.027	50	1769	0.028	41	1410
0.022	40	1746	0.021	34	1574
0.055	89	1525	0.059	85	1365
0.024	97	4030	0.032	112	3342
0.024	78	3165	0.021	57	2711
0.419	612	850	0.563	895	695
0.026	90	3414	0.024	80	3196
0.066	118	1682	0.059	90	1447
0.02	18	882	0.018	14	754
0.024	30	1242	0.024	24	969
0.028	42	1464	0.026	31	1182
0.315	174	378	0.449	243	298
0.067	101	1399	0.077	82	987
0.06	108	1703	0.066	100	1414
0.025	48	1854	0.019	33	1680
0.026	61	2284	0.019	41	2121
0.025	35	1372	0.026	32	1202
0.03	59	1931	0.028	47	1662
0.021	34	1568	0.031	45	1429
0.069	174	2352	0.119	255	1895
0.024	25	1005	0.026	21	776
0.02	74	3638	0.02	68	3353
0.055	81	1400	0.076	95	1161
0.027	39	1412	0.031	36	1144
0.05	78	1483	0.058	74	1198
0.021	52	2454	0.031	66	2040
0.022	23	1034	0.015	13	828
0.022	53	2399	0.028	57	1982
0.068	163	2222	0.069	135	1818
0.022	34	1500	0.019	24	1209
0.019	23	1185	0.032	30	911
0.02	36	1765	0.022	36	1591
0.027	56	2040	0.028	50	1721
0.027	101	3707	0.02	64	3144
0.066	62	881	0.072	67	859
0.031	50	1588	0.027	37	1317
0.021	21	962	0.02	18	891

0.061	145	2237	0.043	86	1905
0.026	85	3243	0.025	65	2491
0.025	34	1352	0.02	27	1352
0.022	16	722	0.032	20	596
0.131	76	505	0.21	120	451
0.051	60	1118	0.066	66	941
0.058	78	1262	0.081	93	1056
0.019	28	1454	0.018	26	1406
0.032	74	2273	0.025	53	2034
0.025	45	1764	0.019	27	1430
0.044	82	1778	0.037	57	1495
0.029	70	2373	0.02	38	1854
0.024	89	3658	0.025	83	3304
0.034	27	758	0.032	20	613
0.047	32	653	0.05	32	603
0.027	35	1282	0.022	27	1175
0.022	35	1574	0.023	32	1378
0.052	50	916	0.071	62	814
0.07	124	1651	0.052	76	1382
0.026	44	1655	0.022	35	1534
0.024	41	1674	0.031	43	1353
0.038	58	1488	0.028	36	1228
0.022	42	1907	0.018	29	1600
0.082	117	1309	0.089	111	1141
0.022	49	2208	0.02	39	1875
0.02	35	1747	0.017	23	1351
0.064	89	1310	0.073	80	1022
0.027	100	3586	0.023	76	3206
0.022	20	872	0.019	12	629
0.033	25	734	0.034	23	649
0.029	49	1631	0.033	46	1347
0.029	44	1470	0.027	32	1171
0.022	63	2824	0.028	70	2389
0.025	60	2337	0.024	46	1860
0.021	53	2487	0.025	56	2146
0.024	19	768	0.035	22	611
0.018	57	3135	0.03	84	2725
0.026	33	1240	0.023	24	1039
0.016	19	1153	0.022	19	854
0.027	44	1597	0.038	51	1275

0.047	56	1147	0.072	76	985
0.022	38	1716	0.029	40	1316
0.018	43	2408	0.025	47	1850
0.022	83	3657	0.029	90	2968
0.025	58	2292	0.024	46	1849
0.029	58	1960	0.026	42	1601
0.03	42	1354	0.024	28	1115
0.022	97	4272	0.023	89	3758
0.146	123	718	0.215	150	547
0.027	45	1609	0.021	35	1598
0.017	15	870	0.019	15	777
0.098	95	877	0.148	138	796
0.019	144	7284	0.022	149	6523
0.02	54	2596	0.021	52	2377
0.027	44	1587	0.032	40	1204
0.036	54	1465	0.034	45	1285
0.059	111	1772	0.07	106	1413
0.058	225	3653	0.052	164	2996
0.071	63	823	0.061	48	733
0.404	332	489	0.507	458	446
0.078	76	893	0.083	65	716
0.022	23	1011	0.032	27	818
0.022	24	1078	0.03	27	885
0.031	50	1580	0.032	44	1313
0.118	70	524	0.108	48	395
0.023	64	2730	0.022	53	2321
0.03	140	4492	0.024	88	3522
0.024	41	1688	0.029	43	1438
0.02	64	3205	0.022	58	2618
0.019	81	4251	0.032	112	3439
0.021	22	1025	0.037	34	886
0.018	28	1502	0.026	33	1254
0.06	114	1787	0.085	131	1414
0.032	31	944	0.026	23	864
0.015	47	3028	0.025	63	2449
0.026	54	1988	0.015	27	1774
0.062	79	1190	0.057	56	932
0.024	24	997	0.033	27	786
0.035	42	1172	0.034	32	901
0.023	75	3122	0.017	45	2650

0.048	30	590	0.09	53	538
0.027	114	4175	0.025	93	3555
0.034	86	2464	0.029	66	2178
0.025	32	1225	0.03	31	1003
0.069	111	1493	0.066	81	1155
0.063	185	2748	0.063	165	2465
0.647	509	278	0.774	722	211
0.025	33	1293	0.016	18	1092
0.047	31	628	0.086	46	491
0.02	81	4007	0.028	99	3491
0.05	54	1035	0.07	57	761
0.028	28	972	0.029	26	878
0.016	26	1557	0.029	35	1159
0.011	10	876	0.026	21	776
0.023	48	2081	0.023	44	1840
0.075	54	663	0.066	41	584
0.053	59	1057	0.047	42	855
0.024	71	2942	0.02	51	2444
0.026	68	2554	0.024	57	2319
0.052	63	1153	0.055	54	922
0.018	24	1285	0.028	29	1021
0.026	48	1804	0.02	33	1580
0.026	86	3200	0.027	67	2451
0.03	95	3021	0.026	67	2546
0.021	62	2959	0.022	58	2565
0.024	46	1852	0.025	40	1581
0.025	36	1387	0.029	37	1237
0.031	61	1887	0.026	45	1662
0.034	40	1154	0.023	22	919
0.155	327	1789	0.223	465	1624
0.034	41	1176	0.037	40	1050
0.067	51	713	0.069	43	582
0.037	50	1316	0.068	68	931
0.027	54	1910	0.022	39	1697
0.024	16	663	0.021	14	643
0.024	79	3201	0.031	90	2795
0.059	49	780	0.039	27	661
0.041	32	754	0.019	15	773
0.016	19	1150	0.029	31	1040
0.047	62	1251	0.038	47	1181

0.024	52	2079	0.029	50	1655
0.023	19	805	0.019	13	687
0.069	104	1396	0.084	95	1034
0.049	73	1403	0.035	43	1175
0.102	54	476	0.106	56	472
0.039	42	1033	0.056	55	928
0.023	51	2179	0.036	74	1997
0.071	82	1073	0.05	44	839
0.02	45	2206	0.016	35	2152
0.406	442	647	0.533	598	524
0.024	90	3667	0.029	88	2973
0.023	18	772	0.011	8	701
0.024	65	2629	0.025	56	2197
0.024	139	5609	0.021	106	4847
0.035	49	1333	0.047	56	1143
0.023	23	977	0.021	20	916
0.028	46	1593	0.029	47	1573
0.024	72	2969	0.026	72	2667
0.026	152	5637	0.021	104	4925
0.02	30	1503	0.024	32	1307
0.027	34	1211	0.03	35	1121
0.068	87	1201	0.067	59	827
0.024	39	1555	0.024	36	1468
0.028	17	597	0.021	11	511
0.024	119	4770	0.026	110	4058
0.021	55	2582	0.018	42	2249
0.025	75	2903	0.027	67	2454
0.037	53	1372	0.034	42	1180
0.064	108	1590	0.057	82	1362
0.341	244	472	0.46	310	364
0.025	34	1310	0.028	35	1208
0.032	63	1897	0.025	40	1533
0.057	67	1116	0.056	55	919
0.023	11	458	0.016	6	362
0.026	82	3057	0.036	97	2590
0.024	42	1701	0.028	40	1388
0.018	23	1233	0.034	32	916
0.022	59	2601	0.025	53	2109
0.027	53	1907	0.019	35	1770
0.027	93	3349	0.027	83	2961

0.067	59	827	0.064	45	663
0.091	159	1596	0.125	166	1164
0.024	30	1224	0.033	33	972
0.027	154	5543	0.027	129	4597
0.029	142	4792	0.029	127	4225
0.034	98	2789	0.044	109	2396
0.081	85	959	0.059	49	785
0.063	56	835	0.086	63	672
0.033	10	297	0.029	8	266
0.018	21	1154	0.027	28	992
0.055	55	938	0.069	57	773
0.076	161	1953	0.096	156	1465
0.038	61	1527	0.042	55	1260
0.022	23	1040	0.024	22	904
0.017	36	2052	0.026	47	1774
0.026	57	2146	0.025	46	1827
0.035	53	1442	0.023	31	1335
0.019	30	1573	0.014	18	1292
0.027	63	2276	0.025	46	1782
0.045	63	1332	0.061	66	1024
0.015	16	1072	0.015	14	892
0.025	20	786	0.029	19	637
0.03	47	1515	0.027	38	1354
0.066	88	1242	0.063	72	1069
0.026	21	782	0.033	22	654
0.025	45	1775	0.019	27	1406
0.025	49	1911	0.027	43	1568
0.074	106	1325	0.079	91	1065
0.016	32	1982	0.028	45	1578
0.026	91	3389	0.032	98	2932
0.02	31	1505	0.033	42	1232
0.099	198	1793	0.124	194	1375
0.022	18	801	0.019	14	741
0.021	58	2713	0.033	76	2234
0.707	692	287	0.819	989	219
0.094	91	872	0.139	125	772
0.028	51	1790	0.03	51	1637
0.021	23	1095	0.028	29	1003
0.019	40	2067	0.027	51	1836
0.028	84	2902	0.019	45	2278

0.025	27	1066	0.032	32	963
0.022	140	6224	0.02	99	4911
0.052	54	984	0.034	28	803
0.025	80	3147	0.023	65	2813
0.021	26	1211	0.036	39	1044
0.062	62	932	0.071	61	799
0.023	37	1592	0.028	38	1319
0.027	34	1231	0.024	28	1128
0.064	77	1134	0.096	103	968
0.023	47	2029	0.032	55	1681
0.056	212	3552	0.062	201	3035
0.029	133	4501	0.025	96	3784
0.074	64	804	0.084	54	587
0.019	29	1497	0.031	38	1182
0.027	29	1057	0.029	25	835
0.022	66	2930	0.03	74	2424
0.023	48	2084	0.028	51	1747
0.014	13	931	0.032	26	787
0.027	62	2255	0.029	61	2041
0.051	67	1237	0.052	53	961
0.028	98	3457	0.03	98	3164
0.066	68	965	0.09	73	736
0.025	52	2010	0.024	43	1769
0.222	258	906	0.371	470	796
0.056	48	803	0.044	31	678
0.027	68	2488	0.021	47	2223
0.035	35	972	0.03	26	842
0.046	71	1475	0.067	89	1248
0.027	47	1697	0.025	37	1436
0.028	37	1284	0.033	35	1019
0.079	52	604	0.08	38	438
0.078	78	926	0.143	111	664
0.065	44	632	0.104	56	484
0.033	72	2098	0.03	57	1851
0.076	72	872	0.043	31	690
0.025	42	1649	0.024	37	1488
0.053	57	1019	0.04	36	869
0.09	65	655	0.07	45	599
0.073	86	1098	0.06	62	975
0.024	113	4567	0.028	106	3746

0.019	31	1568	0.024	28	1146
0.024	46	1904	0.02	34	1701
0.058	100	1625	0.041	57	1339
0.022	41	1782	0.018	26	1442
0.021	58	2768	0.021	49	2325
0.414	496	701	0.467	550	628
0.021	43	2017	0.036	62	1653
0.019	50	2547	0.024	52	2111
0.063	87	1284	0.056	66	1116
0.018	61	3402	0.022	74	3329
0.07	122	1619	0.079	118	1374
0.018	32	1729	0.026	40	1472
0.022	30	1323	0.028	29	1020
0.037	37	976	0.051	43	796
0.014	30	2050	0.026	48	1771
0.11	98	794	0.119	87	645
0.055	64	1108	0.039	41	1021
0.049	84	1641	0.079	115	1336
0.025	25	980	0.023	21	884
0.025	83	3267	0.025	67	2643
0.022	56	2460	0.031	67	2118
0.023	92	3952	0.029	102	3430
0.067	93	1285	0.063	70	1047
0.079	83	965	0.088	77	795
0.021	54	2505	0.029	65	2145
0.029	59	1998	0.028	45	1580
0.12	139	1019	0.167	181	902
0.024	48	1949	0.011	20	1759
0.025	111	4270	0.027	100	3545
0.017	24	1350	0.022	25	1093
0.059	93	1475	0.049	71	1371
0.027	53	1888	0.023	40	1691
0.026	69	2553	0.02	42	2069
0.073	89	1137	0.101	105	930
0.027	49	1748	0.025	35	1351
0.03	104	3367	0.028	79	2701
0.02	32	1606	0.024	32	1290
0.08	69	794	0.049	34	663
0.015	21	1358	0.035	40	1088
0.048	85	1669	0.061	89	1380

0.026	54	2042	0.017	32	1846
0.073	125	1598	0.053	83	1480
0.02	12	583	0.034	18	515
0.02	31	1499	0.023	30	1259
0.028	66	2267	0.034	66	1858
0.023	27	1138	0.014	13	887
0.056	64	1074	0.115	109	836
0.019	27	1412	0.026	29	1066
0.065	51	735	0.07	46	614
0.016	10	601	0.016	9	539
0.03	31	1016	0.03	22	723
0.018	63	3452	0.022	61	2706
0.046	39	801	0.071	42	550
0.028	39	1344	0.021	23	1077
0.021	47	2217	0.026	52	1965
0.027	80	2867	0.03	78	2548
0.054	47	820	0.066	45	642
0.015	26	1734	0.021	30	1431
0.026	99	3747	0.022	75	3330
0.061	55	841	0.09	62	626
0.028	38	1336	0.032	39	1165
0.011	8	710	0.032	19	583
0.02	42	2090	0.031	56	1778
0.073	45	571	0.081	43	491
0.024	49	1962	0.024	37	1508
0.023	66	2767	0.022	55	2473
0.02	72	3550	0.026	84	3150
0.016	18	1086	0.02	17	814
0.027	33	1168	0.028	28	956
0.02	28	1404	0.035	42	1143
0.024	50	2003	0.03	53	1693
0.022	66	2942	0.017	45	2623
0.024	28	1117	0.038	36	900
0.026	21	779	0.022	14	617
0.024	29	1168	0.013	12	941
0.049	119	2304	0.059	120	1904
0.02	26	1260	0.024	27	1090
0.027	83	2955	0.022	58	2598
0.017	62	3625	0.016	49	3089
0.038	54	1352	0.05	64	1228

0.064	94	1380	0.062	73	1102
0.022	54	2355	0.021	42	1915
0.059	220	3494	0.058	190	3101
0.054	76	1344	0.058	67	1080
0.118	121	907	0.126	114	792
0.027	27	964	0.016	12	744
0.032	103	3125	0.031	87	2763
0.021	73	3408	0.028	86	2962
0.031	21	665	0.022	13	570
0.059	105	1664	0.05	67	1264
0.027	28	1021	0.024	20	803
0.024	53	2122	0.03	50	1629
0.058	138	2235	0.041	82	1905
0.078	81	954	0.104	92	793
0.092	63	621	0.103	55	478
0.05	93	1764	0.044	66	1423
0.076	46	556	0.069	38	511
0.031	45	1417	0.022	28	1225
0.016	20	1252	0.032	34	1032
0.053	201	3592	0.06	184	2870
0.024	40	1636	0.021	29	1350
0.057	72	1183	0.058	62	1007
0.025	14	542	0.031	14	445
0.024	36	1452	0.017	18	1033
0.022	21	952	0.02	17	824
0.017	52	2952	0.022	56	2443
0.058	38	614	0.091	55	552
0.024	39	1562	0.024	32	1309
0.024	51	2033	0.025	44	1749
0.022	35	1589	0.034	48	1382
0.02	26	1304	0.03	35	1137
0.057	199	3281	0.058	164	2648
0.025	48	1860	0.023	37	1549
0.023	76	3230	0.021	61	2898
0.019	43	2164	0.025	47	1819
0.024	61	2507	0.019	41	2084
0.024	55	2229	0.023	48	2011
0.032	123	3665	0.033	96	2821
0.027	57	2085	0.027	50	1815
0.064	82	1209	0.064	70	1029

0.023	46	1916	0.017	28	1598
0.014	14	994	0.037	31	803
0.07	168	2234	0.072	151	1958
0.023	44	1863	0.017	26	1502
0.085	41	442	0.124	53	374
0.021	70	3189	0.027	76	2705
0.026	76	2886	0.023	56	2327
0.029	31	1055	0.016	17	1055
0.035	41	1132	0.015	14	918
0.02	13	642	0.017	9	509
0.026	27	1012	0.04	38	918
0.023	22	918	0.024	19	772
0.073	137	1737	0.097	151	1398
0.04	102	2436	0.05	105	1975
0.026	99	3738	0.026	80	3010
0.063	53	790	0.059	41	655
0.017	24	1352	0.02	26	1253
0.028	90	3165	0.025	72	2829
0.023	41	1709	0.026	35	1308
0.02	57	2840	0.023	59	2473
0.108	220	1819	0.109	197	1613
0.02	33	1628	0.024	35	1398
0.068	117	1594	0.083	124	1362
0.023	65	2815	0.029	68	2311
0.043	58	1302	0.055	59	1007
0.07	48	635	0.059	36	579
0.069	42	566	0.048	26	512
0.048	64	1275	0.049	66	1273
0.033	96	2832	0.024	55	2238
0.059	138	2209	0.057	117	1935
0.024	42	1735	0.036	50	1338
0.025	51	1978	0.026	45	1702
0.047	32	652	0.079	43	503
0.063	66	988	0.044	38	832
0.02	42	2056	0.025	46	1788
0.023	20	865	0.023	17	727
0.023	17	732	0.017	10	585
0.02	17	821	0.029	22	732
0.165	108	546	0.207	122	468
0.136	191	1210	0.178	206	953

0.031	60	1871	0.026	42	1605
0.025	59	2262	0.02	41	1976
0.025	119	4557	0.023	91	3800
0.019	22	1140	0.039	35	872
0.023	27	1128	0.028	28	967
0.019	36	1821	0.027	45	1640
0.092	122	1208	0.096	103	967
0.02	50	2453	0.025	55	2113
0.031	57	1796	0.03	49	1601
0.069	96	1305	0.076	85	1039
0.023	45	1934	0.028	42	1466
0.018	18	1005	0.024	20	819
0.056	64	1078	0.066	64	904
0.026	23	872	0.034	24	678
0.051	85	1597	0.05	67	1283
0.024	81	3308	0.022	62	2783
0.076	295	3609	0.099	335	3043
0.032	69	2055	0.027	47	1696
0.025	62	2448	0.025	48	1894
0.078	182	2146	0.098	185	1710
0.026	115	4245	0.023	84	3505
0.027	53	1883	0.021	34	1593
0.034	48	1361	0.033	40	1157
0.03	51	1672	0.027	39	1424
0.02	22	1102	0.017	14	800
0.087	89	929	0.085	72	778
0.052	40	732	0.045	28	592
0.032	55	1656	0.023	36	1563
0.028	55	1884	0.029	47	1596
0.024	29	1193	0.026	30	1115
0.021	15	706	0.031	16	492
0.024	67	2703	0.03	69	2251
0.019	19	982	0.032	27	828
0.023	37	1560	0.018	27	1485
0.022	87	3880	0.028	99	3447
0.027	73	2587	0.03	70	2300
0.023	30	1257	0.013	15	1135
0.024	61	2443	0.03	60	1968
0.024	49	1953	0.021	37	1706
0.159	88	464	0.246	113	346

0.018	14	776	0.024	16	663
0.052	92	1683	0.047	76	1550
0.06	101	1570	0.064	90	1318
0.075	85	1043	0.072	64	821
0.026	108	4067	0.026	95	3502
0.024	49	1991	0.042	72	1653
0.031	109	3438	0.021	65	2969
0.022	28	1229	0.023	26	1113
0.074	80	996	0.088	76	790
0.024	44	1762	0.032	45	1373
0.023	34	1420	0.022	24	1065
0.032	31	942	0.02	16	799
0.021	31	1445	0.02	23	1154
0.028	73	2493	0.029	63	2142
0.024	32	1305	0.032	33	1011
0.021	89	4149	0.022	80	3575
0.061	100	1543	0.047	66	1333
0.025	54	2093	0.029	48	1633
0.05	150	2822	0.049	127	2463
0.019	21	1097	0.022	17	772
0.186	85	373	0.302	110	254
0.056	54	911	0.062	52	782
0.023	42	1798	0.028	45	1588
0.027	31	1126	0.016	16	962
0.05	59	1117	0.063	62	927
0.022	35	1556	0.026	39	1466
0.052	55	1004	0.056	47	794
0.03	20	642	0.023	12	518
0.064	99	1445	0.088	108	1116
0.019	19	965	0.03	22	711
0.037	42	1090	0.032	33	984
0.054	111	1927	0.053	91	1617
0.081	37	419	0.133	57	371
0.08	56	642	0.082	45	504
0.027	68	2470	0.018	38	2121
0.06	69	1080	0.034	33	950
0.029	91	3017	0.03	82	2645
0.096	64	603	0.076	39	473
0.038	112	2802	0.022	57	2499
0.056	69	1174	0.047	47	950

0.023	62	2676	0.03	75	2434
0.072	92	1178	0.061	68	1042
0.06	253	3946	0.058	221	3587
0.06	59	921	0.063	53	784
0.028	91	3108	0.028	82	2800
0.075	75	928	0.072	66	850
0.023	39	1645	0.027	38	1367
0.031	30	945	0.021	20	929
0.012	12	1016	0.016	13	814
0.021	91	4215	0.02	77	3778
0.029	79	2678	0.028	64	2196
0.042	49	1130	0.052	56	1018
0.013	28	2069	0.017	32	1808
0.018	48	2624	0.031	68	2123
0.033	31	903	0.026	22	813
0.026	25	940	0.019	17	862
0.021	26	1223	0.022	25	1087
0.081	89	1007	0.119	103	763
0.035	25	691	0.023	16	669
0.019	29	1482	0.02	25	1228
0.024	70	2874	0.025	59	2333
0.033	29	850	0.024	16	640
0.02	39	1870	0.022	37	1624
0.031	33	1043	0.024	19	773
0.013	26	1959	0.031	51	1570
0.019	29	1476	0.031	38	1181
0.061	61	946	0.067	59	826
0.024	35	1444	0.029	36	1222
0.058	90	1452	0.072	92	1182
0.023	20	842	0.017	15	856
0.026	89	3348	0.021	63	2896
0.026	88	3350	0.026	77	2907
0.029	130	4397	0.028	96	3375
0.429	458	610	0.516	589	553
0.112	122	964	0.168	173	859
0.072	69	893	0.046	39	817
0.032	54	1613	0.025	35	1349
0.022	125	5510	0.017	80	4657
0.158	157	838	0.177	149	694
0.55	1072	878	0.678	1406	668

0.057	84	1393	0.075	84	1037
0.054	90	1582	0.074	102	1275
0.062	40	605	0.066	35	494
0.046	34	698	0.059	38	602
0.024	25	1000	0.029	30	1007
0.064	141	2047	0.055	104	1791
0.041	73	1709	0.058	88	1436
0.018	26	1386	0.033	37	1098
0.024	135	5443	0.026	122	4622
0.027	20	726	0.021	14	643
0.016	27	1692	0.026	41	1513
0.019	64	3260	0.026	72	2740
0.026	134	5117	0.029	136	4630
0.022	55	2433	0.023	51	2185
0.019	41	2143	0.032	62	1876
0.022	114	5185	0.02	93	4470
0.064	130	1916	0.091	143	1426
0.024	77	3134	0.036	93	2470
0.027	43	1557	0.03	40	1274
0.028	26	896	0.025	19	729
0.051	74	1369	0.043	50	1107
0.035	20	550	0.027	13	470
0.034	38	1093	0.024	23	919
0.044	26	565	0.021	10	477
0.026	31	1169	0.025	25	994
0.045	31	657	0.023	12	520
0.024	40	1662	0.022	31	1402
0.024	54	2171	0.021	43	1989
0.029	52	1738	0.022	31	1352
0.029	27	889	0.02	15	724
0.023	45	1913	0.018	28	1497
0.031	90	2852	0.041	100	2354
0.026	92	3412	0.019	56	2915
0.059	59	936	0.029	22	746
0.028	80	2757	0.02	45	2261
0.019	70	3649	0.024	75	3058
0.022	16	715	0.019	12	605
0.02	27	1297	0.02	23	1141
0.025	76	2980	0.018	45	2435
0.041	28	656	0.019	11	569

0.024	46	1892	0.015	25	1637
0.029	36	1210	0.029	31	1045
0.065	104	1489	0.071	95	1246
0.025	50	1958	0.031	60	1863
0.061	97	1491	0.057	76	1256
0.025	41	1625	0.034	49	1401
0.023	27	1149	0.044	42	919
0.02	22	1068	0.021	18	859
0.026	52	1984	0.024	39	1575
0.019	23	1165	0.028	27	940
0.054	67	1182	0.067	71	986
0.019	69	3546	0.021	63	2930
0.065	72	1031	0.044	40	874
0.02	28	1395	0.028	31	1086
0.014	4	288	0.009	2	233
0.024	64	2616	0.021	44	2077
0.02	58	2816	0.022	57	2496
0.031	26	811	0.056	43	728
0.025	57	2248	0.029	54	1827
0.053	160	2839	0.047	122	2465
0.027	38	1376	0.026	32	1199
0.028	35	1219	0.022	23	1034
0.027	43	1566	0.027	40	1447
0.106	140	1177	0.138	164	1025
0.029	38	1288	0.027	29	1030
0.026	61	2302	0.019	39	2053
0.061	105	1614	0.058	83	1346
0.035	44	1231	0.029	34	1142
0.093	61	592	0.14	73	448
0.062	87	1325	0.069	87	1171
0.024	62	2516	0.024	52	2159
0.059	120	1931	0.046	73	1519
0.024	82	3340	0.027	87	3143
0.02	97	4720	0.023	103	4338
0.024	30	1214	0.019	21	1079
0.026	23	855	0.024	19	765
0.067	126	1756	0.063	101	1492
0.021	108	4951	0.026	121	4499
0.067	44	615	0.049	28	543
0.027	33	1190	0.027	28	991

0.031	50	1552	0.03	41	1345
0.029	95	3190	0.021	58	2709
0.065	101	1447	0.058	78	1278
0.078	93	1098	0.088	84	875
0.064	121	1775	0.053	83	1496
0.023	37	1604	0.027	34	1214
0.026	29	1105	0.027	30	1068
0.025	61	2384	0.028	58	1993
0.024	53	2150	0.027	48	1760
0.025	85	3291	0.025	78	3048
0.085	136	1455	0.088	118	1219
0.358	487	873	0.43	580	769
0.03	71	2324	0.022	45	2009
0.065	95	1370	0.067	79	1105
0.025	89	3443	0.023	74	3112
0.032	87	2670	0.024	58	2341
0.022	51	2276	0.028	55	1935
0.067	78	1094	0.053	46	814
0.018	45	2395	0.027	56	1997
0.023	34	1440	0.023	27	1157
0.031	65	2058	0.034	61	1742
0.027	37	1339	0.032	44	1324
0.046	43	899	0.041	32	749
0.021	91	4343	0.024	90	3585
0.027	60	2158	0.028	52	1783
0.023	85	3566	0.019	56	2886
0.06	54	843	0.07	46	612
0.045	67	1430	0.068	81	1111
0.099	131	1187	0.093	100	973
0.054	57	992	0.063	49	727
0.025	38	1494	0.025	32	1229
0.617	484	300	0.717	606	239
0.029	27	907	0.02	15	738
0.061	82	1257	0.091	90	901
0.029	12	408	0.019	6	308
0.05	60	1148	0.058	54	877
0.027	74	2705	0.028	66	2309
0.033	67	1994	0.027	46	1627
0.055	146	2488	0.054	122	2130
0.028	52	1823	0.021	33	1517

0.044	63	1382	0.048	55	1081
0.024	85	3423	0.015	44	2906
0.058	66	1071	0.107	105	872
0.025	51	1950	0.017	29	1698
0.019	49	2527	0.02	45	2151
0.05	57	1084	0.043	40	880
0.032	60	1818	0.03	48	1561
0.022	58	2639	0.03	72	2299
0.029	75	2507	0.034	75	2123
0.135	224	1434	0.187	290	1261
0.028	59	2020	0.025	42	1641
0.082	105	1176	0.087	90	939
0.028	109	3781	0.034	106	2997
0.026	66	2447	0.024	55	2283
0.093	147	1441	0.137	178	1117
0.153	231	1279	0.192	242	1018
0.016	29	1731	0.034	49	1377
0.057	33	541	0.117	53	401
0.036	62	1680	0.024	38	1565
0.026	75	2784	0.023	54	2266
0.024	37	1505	0.016	21	1272
0.069	130	1751	0.071	103	1340
0.023	28	1185	0.03	28	908
0.024	66	2635	0.026	60	2215
0.049	33	636	0.053	29	521
0.052	46	831	0.075	56	694
0.056	131	2218	0.055	104	1800
0.016	59	3654	0.02	63	3059
0.093	72	705	0.091	68	676
0.067	99	1372	0.062	74	1112
0.032	61	1834	0.028	45	1558
0.023	66	2810	0.027	65	2356
0.023	17	716	0.02	11	552
0.076	75	917	0.076	62	749
0.032	51	1554	0.027	37	1318
0.041	37	855	0.047	38	763
0.025	53	2074	0.024	41	1675
0.06	119	1851	0.055	91	1561
0.066	154	2174	0.063	124	1829
0.061	66	1014	0.045	40	843

0.068	85	1171	0.075	70	862
0.03	23	749	0.031	19	592
0.025	81	3212	0.027	76	2752
0.031	67	2109	0.031	53	1656
0.027	103	3693	0.03	105	3397
0.064	86	1268	0.044	51	1109
0.018	39	2148	0.017	33	1962
0.078	110	1308	0.103	113	983
0.015	20	1322	0.027	31	1129
0.025	55	2143	0.025	47	1865
0.029	69	2349	0.031	66	2032
0.029	92	3080	0.031	90	2809
0.013	15	1105	0.025	25	966
0.028	25	871	0.041	25	590
0.053	121	2172	0.055	118	2028
0.021	63	2921	0.022	60	2646
0.028	52	1798	0.026	39	1485
0.018	39	2124	0.029	53	1786
0.019	31	1620	0.022	26	1171
0.066	111	1580	0.059	85	1364
0.073	115	1466	0.063	80	1190
0.052	152	2789	0.049	118	2298
0.031	55	1725	0.027	37	1321
0.026	81	3094	0.027	79	2852
0.023	90	3787	0.02	62	3086
0.017	33	1901	0.026	44	1679
0.021	87	4125	0.032	114	3408
0.047	56	1148	0.055	58	994
0.017	29	1680	0.028	40	1411
0.073	75	949	0.092	81	804
0.072	111	1428	0.054	68	1192
0.024	94	3760	0.028	89	3061
0.13	146	976	0.135	131	838
0.024	69	2793	0.024	55	2280
0.024	65	2637	0.026	54	2019
0.029	61	2025	0.018	31	1680
0.092	90	891	0.13	113	753
0.019	56	2832	0.031	77	2371
0.029	55	1822	0.034	52	1477
0.054	83	1461	0.056	75	1255

0.08	139	1591	0.063	85	1262
0.027	50	1787	0.03	45	1472
0.011	14	1213	0.02	18	902
0.031	29	919	0.025	19	736
0.023	32	1341	0.029	34	1119
0.067	68	950	0.072	56	727
0.121	88	638	0.155	95	519
0.024	47	1887	0.024	39	1599
0.032	36	1085	0.033	32	940
0.019	75	3868	0.023	81	3440
0.034	55	1568	0.019	26	1308
0.024	26	1080	0.035	31	867
0.02	48	2294	0.022	44	1958
0.071	99	1292	0.059	68	1094
0.024	19	758	0.024	17	694
0.028	50	1728	0.017	27	1524
0.053	72	1280	0.062	64	965
0.023	53	2277	0.033	68	1991
0.026	32	1220	0.014	17	1216
0.095	171	1626	0.14	220	1350
0.019	35	1822	0.027	44	1607
0.016	21	1330	0.017	19	1111
0.013	20	1499	0.04	55	1333
0.027	59	2160	0.025	46	1764
0.022	17	750	0.02	15	750
0.026	25	920	0.031	24	757
0.026	57	2160	0.027	50	1823
0.025	85	3349	0.019	57	2924
0.038	43	1081	0.04	37	884
0.025	23	889	0.024	19	759
0.018	23	1241	0.028	27	941
0.017	26	1517	0.008	10	1278
0.021	79	3625	0.036	116	3081
0.166	120	603	0.218	139	500
0.029	94	3152	0.027	74	2668
0.02	72	3542	0.025	74	2928
0.055	64	1092	0.056	53	899
0.03	53	1728	0.033	48	1388
0.028	69	2419	0.019	43	2173
0.064	68	995	0.082	73	820

0.026	16	604	0.015	8	534
0.029	22	747	0.037	22	565
0.025	22	860	0.025	21	815
0.074	103	1288	0.084	94	1030
0.026	29	1076	0.023	20	863
0.019	21	1101	0.024	21	857
0.028	106	3716	0.025	81	3178
0.039	85	2090	0.026	46	1728
0.024	30	1212	0.02	24	1183
0.049	23	449	0.06	25	392
0.027	32	1162	0.024	27	1121
0.028	129	4434	0.021	83	3836
0.025	48	1906	0.037	60	1577
0.029	36	1212	0.028	31	1062
0.016	47	2976	0.024	64	2590
0.017	14	831	0.038	28	704
0.019	43	2220	0.022	42	1892
0.026	151	5597	0.028	131	4630
0.029	31	1049	0.025	23	909
0.065	51	730	0.074	52	654
0.067	73	1015	0.049	41	799
0.048	122	2407	0.052	104	1889
0.022	71	3180	0.027	70	2550
0.027	76	2779	0.03	71	2308
0.018	46	2544	0.022	47	2090
0.035	80	2196	0.052	98	1773
0.045	45	950	0.063	48	715
0.031	47	1464	0.015	19	1232
0.026	62	2328	0.017	35	1984
0.027	38	1363	0.026	29	1071
0.026	114	4260	0.034	124	3533
0.048	59	1182	0.052	51	923
0.064	55	804	0.09	65	659
0.023	75	3200	0.023	62	2577
0.027	30	1092	0.013	13	955
0.038	51	1297	0.026	27	1027
0.024	17	688	0.018	12	641
0.028	47	1615	0.036	50	1332
0.023	25	1065	0.022	22	991
0.021	30	1399	0.023	28	1177

0.013	23	1743	0.022	31	1377
0.027	47	1677	0.029	42	1394
0.019	26	1370	0.033	44	1283
0.022	37	1616	0.028	39	1363
0.019	34	1746	0.025	38	1485
0.173	186	890	0.255	266	777
0.024	18	730	0.03	20	651
0.02	83	4101	0.02	70	3383
0.174	170	805	0.22	193	684
0.077	66	795	0.097	69	645
0.031	55	1748	0.019	34	1742
0.026	29	1107	0.036	30	795
0.026	35	1317	0.019	21	1104
0.028	52	1786	0.029	45	1520
0.049	114	2228	0.077	152	1824
0.029	45	1529	0.025	32	1227
0.02	34	1696	0.022	32	1455
0.022	22	970	0.017	13	768
0.027	25	905	0.021	17	782
0.022	51	2310	0.023	48	2073
0.079	155	1810	0.1	169	1529
0.021	53	2515	0.025	52	1993
0.025	65	2568	0.028	64	2200
0.058	77	1244	0.053	58	1039
0.022	79	3558	0.028	90	3145
0.086	108	1154	0.058	62	1004
0.03	59	1933	0.026	44	1665
0.029	108	3575	0.024	70	2894
0.021	16	746	0.016	11	679
0.016	18	1122	0.022	21	947
0.035	34	938	0.033	28	832
0.049	44	861	0.031	26	808
0.021	53	2486	0.015	34	2274
0.02	91	4404	0.021	84	3940
0.073	41	518	0.07	31	414
0.39	393	614	0.508	451	437
0.026	65	2458	0.024	52	2108
0.03	22	705	0.044	29	630
0.134	249	1606	0.171	262	1270
0.032	119	3644	0.023	68	2932

0.028	55	1934	0.032	52	1585
0.018	15	835	0.029	19	645
0.049	80	1543	0.056	67	1129
0.045	49	1028	0.047	43	869
0.03	72	2333	0.032	70	2150
0.027	26	944	0.036	31	827
0.063	89	1317	0.076	85	1036
0.064	80	1174	0.056	54	911
0.024	55	2251	0.033	62	1806
0.019	29	1481	0.017	22	1277
0.027	97	3502	0.025	83	3179
0.033	36	1065	0.03	31	996
0.026	89	3388	0.023	67	2885
0.056	101	1718	0.04	62	1502
0.069	46	618	0.054	28	493
0.067	175	2427	0.059	118	1885
0.027	48	1730	0.023	35	1480
0.061	35	536	0.052	26	473
0.032	78	2378	0.024	47	1927
0.056	86	1442	0.054	74	1307
0.021	27	1229	0.019	22	1133
0.038	45	1141	0.033	33	961
0.033	54	1597	0.027	37	1309
0.025	78	3028	0.026	71	2679
0.021	48	2224	0.023	47	2015
0.09	144	1451	0.098	125	1156
0.021	131	6038	0.02	107	5343
0.097	75	700	0.126	83	578
0.022	16	724	0.041	26	611
0.022	41	1819	0.032	48	1468
0.057	101	1659	0.066	95	1344
0.022	43	1892	0.017	28	1601
0.02	27	1321	0.023	26	1101
0.022	61	2739	0.021	49	2266
0.031	34	1048	0.02	18	868
0.016	26	1629	0.028	36	1259
0.02	24	1196	0.036	34	920
0.023	32	1382	0.035	45	1226
0.024	38	1562	0.022	31	1387
0.022	30	1350	0.022	23	1022

0.026	61	2330	0.03	60	1944
0.02	37	1854	0.018	29	1550
0.028	57	1995	0.022	40	1743
0.029	84	2775	0.026	68	2533
0.057	52	853	0.09	75	759
0.018	16	886	0.026	20	738
0.127	165	1138	0.166	201	1012
0.014	16	1154	0.019	19	965
0.019	16	821	0.028	21	716
0.027	70	2478	0.023	50	2133
0.192	96	403	0.249	112	337
0.033	49	1433	0.032	38	1136
0.07	120	1588	0.105	156	1323
0.025	49	1885	0.025	41	1598
0.025	63	2411	0.035	74	2050
0.042	42	969	0.061	51	784
0.032	87	2603	0.025	56	2184
0.019	24	1257	0.021	23	1065
0.018	44	2401	0.022	44	1974
0.025	40	1584	0.03	39	1257
0.132	125	819	0.144	119	705
0.024	88	3536	0.02	67	3231
0.029	36	1211	0.021	20	955
0.022	31	1379	0.016	20	1225
0.023	67	2806	0.024	60	2439
0.023	25	1044	0.018	16	895
0.02	30	1487	0.026	33	1230
0.016	48	2926	0.02	51	2444
0.16	172	903	0.19	205	875
0.06	35	545	0.098	44	407
0.061	50	775	0.047	35	714
0.034	104	2977	0.037	103	2677
0.009	7	752	0.017	10	562
0.027	34	1215	0.023	24	1028
0.129	120	812	0.155	127	694
0.09	53	537	0.068	33	454
0.065	75	1078	0.055	51	880
0.037	71	1829	0.034	59	1668
0.031	67	2071	0.03	58	1867
0.022	57	2486	0.035	79	2169

0.022	43	1908	0.029	52	1715
0.026	19	714	0.026	15	571
0.027	28	1021	0.028	26	888
0.026	57	2110	0.031	57	1784
0.022	31	1361	0.038	47	1192
0.024	13	531	0.041	19	450
0.019	35	1765	0.018	29	1603
0.025	61	2349	0.024	53	2112
0.023	34	1421	0.021	26	1231
0.06	104	1620	0.056	86	1455
0.047	59	1193	0.049	56	1089
0.07	87	1154	0.05	50	945
0.124	74	525	0.166	85	428
0.023	50	2093	0.025	43	1647
0.024	33	1369	0.029	33	1106
0.028	59	2030	0.02	34	1655
0.023	36	1557	0.021	27	1261
0.058	74	1207	0.063	71	1063
0.025	60	2323	0.026	51	1922
0.023	33	1432	0.033	42	1249
0.041	54	1268	0.068	71	972
0.05	88	1685	0.04	60	1440
0.026	93	3417	0.037	114	2995
0.861	1159	187	0.888	1497	189
0.108	134	1109	0.16	153	802
0.023	54	2268	0.022	42	1830
0.088	235	2443	0.09	215	2168
0.148	142	820	0.147	120	698
0.024	63	2510	0.024	54	2184
0.031	26	806	0.061	41	633
0.077	53	637	0.057	29	479
0.021	34	1600	0.027	36	1284
0.034	50	1416	0.021	28	1311
0.025	88	3386	0.029	91	3081
0.03	36	1160	0.048	48	950
0.036	96	2537	0.024	53	2194
0.021	49	2272	0.026	50	1838
0.036	27	717	0.025	14	543
0.023	16	668	0.033	20	586
0.026	56	2119	0.029	58	1921

0.018	14	759	0.026	17	630
0.056	51	859	0.075	52	640
0.029	74	2442	0.022	45	1994
0.067	52	720	0.052	30	544
0.096	95	893	0.134	127	823
0.019	82	4303	0.026	95	3626
0.034	45	1294	0.023	25	1058
0.047	66	1333	0.055	68	1159
0.028	46	1626	0.037	54	1402
0.031	25	774	0.019	13	675
0.117	85	642	0.133	93	604
0.029	34	1136	0.031	27	841
0.372	438	740	0.486	551	583
0.029	56	1891	0.021	34	1570
0.028	42	1470	0.028	34	1166
0.044	64	1379	0.032	43	1288
0.021	47	2181	0.025	48	1866
0.024	105	4206	0.03	112	3582
0.017	32	1819	0.027	44	1563
0.063	49	726	0.07	53	707
0.028	50	1724	0.021	32	1511
0.025	48	1904	0.031	56	1725
0.037	61	1590	0.037	55	1450
0.024	47	1927	0.021	37	1709
0.07	77	1016	0.075	72	885
0.019	32	1686	0.041	63	1490
0.106	81	683	0.154	95	520
0.036	32	850	0.016	12	743
0.075	55	675	0.105	63	538
0.053	51	903	0.108	79	654
0.023	42	1804	0.033	57	1667
0.013	22	1728	0.016	21	1312
0.022	14	616	0.046	23	475
0.021	51	2385	0.028	53	1874
0.016	24	1463	0.021	28	1319
0.064	94	1384	0.056	64	1077
0.021	27	1257	0.017	19	1081
0.033	59	1722	0.028	43	1477
0.029	29	958	0.03	25	811
0.019	28	1413	0.029	36	1219

0.03	64	2094	0.024	40	1599
0.017	30	1715	0.022	34	1530
0.207	271	1036	0.244	305	943
0.024	49	1987	0.022	39	1719
0.023	37	1541	0.033	46	1364
0.021	25	1142	0.023	22	915
0.018	37	2077	0.021	38	1756
0.022	61	2733	0.029	65	2144
0.026	32	1218	0.016	17	1053
0.018	22	1171	0.026	27	1026
0.052	37	672	0.057	32	531
0.036	37	986	0.027	25	888
0.065	89	1289	0.058	67	1082
0.046	58	1210	0.031	35	1081
0.022	53	2341	0.028	56	1949
0.033	72	2124	0.028	52	1805
0.019	15	794	0.016	10	633
0.062	128	1927	0.062	110	1652
0.033	70	2044	0.037	57	1478
0.021	149	7014	0.022	133	5996
0.031	78	2462	0.03	64	2079
0.025	49	1931	0.022	40	1740
0.016	8	503	0.027	12	427
0.124	298	2107	0.143	299	1792
0.027	60	2181	0.03	56	1808
0.025	74	2834	0.021	51	2420
0.023	35	1480	0.019	23	1165
0.02	84	4091	0.019	69	3657
0.098	65	596	0.074	40	502
0.012	41	3345	0.028	81	2797
0.029	51	1729	0.032	48	1442
0.025	30	1158	0.027	27	974
0.024	19	783	0.025	16	620
0.08	171	1973	0.083	155	1716
0.028	57	2007	0.023	41	1732
0.025	69	2740	0.023	55	2345
0.036	35	938	0.025	21	834
0.187	268	1166	0.238	330	1056
0.029	79	2626	0.026	63	2399
0.056	86	1461	0.063	84	1250

0.026	87	3212	0.019	51	2590
0.032	39	1199	0.027	30	1077
0.026	26	980	0.029	25	840
0.086	55	581	0.057	34	562
0.023	33	1381	0.016	18	1089
0.057	42	693	0.077	50	600
0.027	47	1683	0.028	45	1574
0.029	62	2080	0.034	58	1641
0.02	67	3347	0.018	54	2875
0.02	44	2165	0.024	43	1719
0.027	68	2481	0.02	45	2260
0.023	36	1555	0.033	44	1309
0.071	142	1845	0.082	126	1413
0.028	45	1580	0.026	36	1372
0.022	43	1886	0.03	48	1528
0.025	33	1298	0.035	38	1055
0.057	50	829	0.06	44	684
0.247	100	305	0.408	159	231
0.031	45	1423	0.028	36	1243
0.023	63	2659	0.022	54	2350
0.028	70	2439	0.026	54	2011
0.023	62	2582	0.023	49	2096
0.028	45	1555	0.032	41	1245
0.607	827	536	0.683	1016	471
0.056	64	1089	0.068	70	956
0.062	107	1627	0.053	83	1485
0.021	24	1142	0.01	10	1022
0.017	24	1363	0.019	25	1264
0.057	41	675	0.095	57	541
0.029	84	2803	0.027	62	2255
0.032	35	1042	0.029	27	893
0.025	68	2668	0.025	51	2020
0.018	57	3046	0.028	74	2558
0.029	21	711	0.021	12	569
0.018	21	1143	0.018	18	956
0.619	773	476	0.719	1139	446
0.026	31	1178	0.028	29	1010
0.026	35	1334	0.023	27	1149
0.032	63	1880	0.02	32	1592
0.03	77	2494	0.018	37	2032

0.042	92	2115	0.053	98	1757
0.023	39	1686	0.022	29	1318
0.015	21	1338	0.019	26	1326
0.024	92	3689	0.021	67	3164
0.022	31	1372	0.03	36	1176
0.023	46	1915	0.03	51	1676
0.024	70	2811	0.025	62	2465
0.018	31	1677	0.025	37	1421
0.086	119	1267	0.117	148	1117
0.248	232	704	0.375	374	624
0.029	54	1831	0.018	28	1565
0.03	77	2459	0.026	53	2002
0.063	158	2367	0.051	115	2120
0.031	48	1509	0.03	40	1279
0.021	47	2223	0.024	46	1845
0.027	108	3937	0.019	67	3533
0.051	39	731	0.071	44	579
0.024	37	1512	0.021	26	1203
0.044	32	700	0.077	48	574
0.035	48	1333	0.035	40	1106
0.053	67	1199	0.066	76	1067
0.029	38	1269	0.024	28	1131
0.079	113	1319	0.095	106	1005
0.024	60	2397	0.026	54	2012
0.057	84	1401	0.084	94	1025
0.024	41	1694	0.035	52	1414
0.052	112	2030	0.056	100	1696
0.031	111	3515	0.023	72	3128
0.061	73	1129	0.051	54	1003
0.031	23	713	0.033	21	607
0.022	103	4579	0.02	79	3937
0.033	25	727	0.038	28	711
0.021	64	2998	0.025	65	2579
0.05	41	781	0.076	48	585
0.027	58	2064	0.017	32	1879
0.023	46	1982	0.027	45	1607
0.025	68	2623	0.031	70	2200
0.025	26	1032	0.033	32	936
0.059	145	2332	0.062	126	1914
0.02	16	782	0.028	17	589

0.066	45	634	0.083	44	486
0.024	30	1216	0.03	31	1003
0.051	205	3796	0.041	135	3193
0.027	30	1090	0.019	18	914
0.017	16	953	0.02	17	819
0.024	24	996	0.029	24	797
0.031	58	1838	0.027	40	1455
0.022	44	1974	0.031	52	1649
0.089	122	1245	0.085	98	1057
0.023	21	878	0.05	38	720
0.246	282	863	0.368	416	713
0.018	29	1590	0.021	28	1295
0.027	42	1531	0.023	35	1455
0.026	73	2684	0.038	91	2278
0.029	78	2567	0.026	57	2159
0.019	63	3237	0.021	61	2851
0.017	69	4006	0.021	73	3467
0.069	136	1823	0.088	141	1453
0.05	81	1528	0.036	49	1325
0.027	63	2283	0.025	52	2044
0.052	87	1591	0.052	78	1431
0.026	56	2082	0.024	42	1698
0.022	33	1475	0.028	36	1253
0.069	85	1148	0.08	79	913
0.015	16	1032	0.03	28	912
0.027	39	1393	0.025	30	1185
0.028	54	1888	0.031	50	1548
0.05	84	1596	0.061	88	1344
0.025	60	2335	0.024	49	2009
0.1	85	761	0.117	80	604
0.022	45	2031	0.028	52	1822
0.033	35	1027	0.025	22	860
0.031	81	2519	0.029	68	2316
0.03	80	2628	0.023	58	2419
0.016	20	1263	0.024	25	1003
0.081	122	1383	0.083	100	1098
0.019	44	2328	0.031	61	1928
0.121	84	609	0.117	67	508
0.061	43	664	0.067	40	558
0.048	74	1481	0.049	67	1307

0.062	75	1138	0.085	85	910
0.112	115	916	0.151	125	702
0.023	63	2630	0.026	57	2152
0.031	45	1408	0.023	29	1248
0.03	54	1740	0.03	44	1410
0.026	67	2483	0.019	43	2271
0.02	30	1487	0.029	35	1193
0.027	41	1483	0.032	40	1196
0.025	32	1270	0.022	24	1091
0.049	99	1929	0.075	120	1481
0.095	133	1269	0.161	190	993
0.1	64	573	0.182	110	496
0.053	204	3678	0.052	159	2913
0.032	111	3380	0.021	61	2879
0.037	47	1228	0.019	21	1114
0.062	55	830	0.067	59	824
0.023	37	1592	0.022	31	1377
0.021	69	3199	0.023	74	3156
0.023	24	1016	0.028	25	873
0.025	127	4876	0.025	110	4332
0.018	23	1268	0.019	20	1027
0.026	66	2444	0.022	50	2207
0.072	46	589	0.09	42	427
0.026	76	2857	0.022	51	2278
0.022	32	1423	0.024	27	1101
0.028	70	2471	0.024	50	2050
0.06	67	1052	0.07	66	882
0.018	14	764	0.025	18	690
0.028	26	900	0.026	20	735
0.065	50	717	0.062	44	670
0.021	57	2654	0.02	47	2279
0.023	74	3108	0.023	62	2588
0.026	52	1977	0.031	50	1578
0.017	16	946	0.033	24	700
0.024	14	578	0.015	8	518
0.024	60	2400	0.023	43	1805
0.049	74	1438	0.057	69	1137
0.028	144	4951	0.024	101	4159
0.018	22	1197	0.018	18	965
0.02	32	1584	0.022	29	1279

0.078	80	950	0.105	89	760
0.023	51	2121	0.02	35	1723
0.039	54	1330	0.04	49	1176
0.025	51	2006	0.026	48	1781
0.024	48	1921	0.024	39	1596
0.064	65	947	0.035	33	918
0.018	32	1753	0.026	41	1540
0.09	64	646	0.115	79	608
0.03	51	1645	0.027	40	1461
0.022	13	583	0.032	17	515
0.023	28	1170	0.024	26	1045
0.025	51	2012	0.026	43	1606
0.015	15	972	0.026	20	763
0.02	59	2858	0.024	58	2384
0.021	24	1142	0.021	22	1016
0.034	36	1028	0.026	24	895
0.029	49	1617	0.031	43	1355
0.031	32	992	0.021	19	874
0.021	23	1071	0.029	27	890
0.036	19	508	0.027	11	403
0.014	36	2475	0.019	41	2149
0.025	26	1024	0.032	30	910
0.02	27	1320	0.033	37	1093
0.044	54	1162	0.037	38	998
0.019	52	2692	0.025	59	2320
0.054	53	936	0.072	63	811
0.027	55	1947	0.027	43	1565
0.023	86	3724	0.024	77	3124
0.029	40	1336	0.022	26	1131
0.029	60	1988	0.029	51	1707
0.018	74	3938	0.019	63	3256
0.025	54	2102	0.024	46	1865
0.036	47	1260	0.033	37	1101
0.023	75	3161	0.031	86	2711
0.082	96	1071	0.119	108	797
0.2	156	624	0.299	220	517
0.024	71	2884	0.021	53	2469
0.033	34	1003	0.027	22	789
0.061	142	2186	0.064	118	1725
0.053	52	936	0.04	33	797

0.034	19	533	0.016	7	433
0.029	77	2614	0.029	66	2203
0.023	42	1799	0.022	37	1641
0.027	54	1964	0.029	49	1665
0.013	8	593	0.04	19	460
0.035	60	1669	0.03	44	1439
0.018	75	4013	0.021	69	3159
0.064	64	929	0.072	66	845
0.108	146	1202	0.149	170	974
0.021	41	1926	0.033	51	1508
0.024	20	811	0.035	25	690
0.081	119	1349	0.099	121	1103
0.082	104	1162	0.072	73	936
0.022	61	2720	0.019	47	2449
0.028	38	1326	0.029	34	1127
0.028	93	3257	0.024	70	2822
0.069	72	966	0.055	48	823
0.029	42	1395	0.042	51	1157
0.09	36	365	0.076	24	293
0.028	57	2005	0.028	50	1763
0.229	129	435	0.302	136	315
0.022	19	853	0.022	16	718
0.031	28	889	0.032	23	700
0.029	33	1114	0.029	27	910
0.036	44	1181	0.02	20	956
0.027	63	2254	0.028	54	1874
0.065	80	1151	0.054	59	1040
0.05	35	672	0.032	22	672
0.022	18	785	0.013	8	590
0.02	25	1237	0.042	48	1084
0.025	56	2156	0.029	53	1790
0.037	58	1511	0.022	28	1219
0.103	122	1058	0.145	160	947
0.061	80	1225	0.08	84	966
0.028	116	4057	0.022	75	3311
0.028	56	1980	0.021	37	1692
0.036	21	562	0.054	25	435
0.033	47	1398	0.021	27	1271
0.03	32	1026	0.019	16	835
0.046	67	1391	0.05	60	1133

0.026	64	2425	0.029	57	1920
0.061	52	795	0.052	33	599
0.023	78	3252	0.028	78	2739
0.03	50	1630	0.019	27	1390
0.02	68	3282	0.024	69	2817
0.035	48	1310	0.036	38	1031
0.028	37	1286	0.022	23	1033
0.03	55	1763	0.037	51	1344
0.063	79	1176	0.08	78	903
0.075	57	704	0.085	53	571
0.025	70	2759	0.018	43	2352
0.131	76	502	0.097	50	464
0.07	55	733	0.081	54	609
0.03	42	1372	0.015	17	1086
0.024	40	1599	0.026	37	1390
0.017	13	732	0.004	3	701
0.019	27	1390	0.03	38	1236
0.028	43	1475	0.026	31	1170
0.058	106	1714	0.053	81	1441
0.025	36	1422	0.025	31	1198
0.018	84	4485	0.019	71	3746
0.04	18	428	0.102	40	352
0.06	54	844	0.043	34	766
0.018	21	1138	0.034	33	939
0.02	31	1554	0.025	33	1267
0.019	58	2992	0.029	74	2512
0.339	305	594	0.477	495	542
0.027	48	1724	0.024	35	1409
0.019	33	1709	0.022	32	1408
0.094	68	658	0.156	86	467
0.024	122	5014	0.024	112	4601
0.027	31	1125	0.024	27	1087
0.051	122	2279	0.05	102	1936
0.023	28	1167	0.03	34	1098
0.022	44	1921	0.027	41	1455
0.03	63	2048	0.028	56	1914
0.026	72	2700	0.028	71	2486
0.336	154	304	0.494	248	254
0.022	37	1652	0.03	40	1272
0.055	96	1649	0.063	84	1256

0.369	288	493	0.482	399	428
0.02	23	1145	0.018	18	966
0.028	153	5391	0.03	147	4726
0.019	44	2235	0.026	50	1887
0.026	38	1446	0.022	27	1192
0.019	34	1785	0.02	33	1618
0.044	80	1721	0.066	96	1350
0.022	19	829	0.024	17	679
0.022	45	1986	0.023	36	1546
0.023	45	1887	0.018	28	1512
0.028	60	2104	0.026	46	1748
0.021	22	1016	0.022	20	874
0.037	40	1034	0.025	22	868
0.061	63	966	0.048	41	813
0.026	77	2897	0.03	70	2291
0.026	32	1196	0.038	40	1004
0.033	22	641	0.026	15	555
0.023	83	3605	0.018	58	3113
0.024	56	2307	0.028	57	1958
0.019	17	888	0.016	12	749
0.021	17	783	0.036	26	694
0.03	59	1895	0.023	40	1672
0.052	92	1675	0.066	103	1451
0.021	15	696	0.031	17	535
0.046	78	1604	0.066	90	1280
0.078	150	1763	0.049	75	1446
0.015	20	1275	0.025	28	1105
0.026	23	869	0.031	25	777
0.036	97	2620	0.022	55	2444
0.019	36	1878	0.019	29	1481
0.034	41	1181	0.026	26	983
0.035	50	1398	0.033	40	1174
0.044	58	1257	0.052	53	959
0.016	46	2761	0.032	81	2440
0.025	52	2027	0.032	57	1730
0.025	46	1773	0.037	54	1417
0.02	38	1821	0.025	41	1576
0.032	17	515	0.043	20	449
0.027	56	2050	0.026	48	1771
0.044	76	1650	0.072	102	1307

0.03	48	1541	0.028	35	1199
0.016	10	624	0.015	8	525
0.026	127	4770	0.026	108	4064
0.027	70	2530	0.026	60	2290
0.063	84	1257	0.074	75	943
0.026	55	2081	0.03	54	1769
0.027	84	3033	0.033	83	2402
0.02	56	2769	0.016	40	2496
0.026	51	1929	0.022	38	1668
0.061	83	1275	0.059	78	1247
0.026	91	3405	0.032	95	2902
0.072	75	966	0.074	65	811
0.023	63	2703	0.038	94	2359
0.028	59	2044	0.014	26	1787
0.027	91	3319	0.022	68	2995
0.027	37	1321	0.022	23	1016
0.022	59	2605	0.024	55	2226
0.108	64	526	0.127	58	400
0.017	33	1906	0.017	29	1642
0.058	149	2415	0.055	119	2051
0.024	69	2800	0.026	61	2313
0.111	204	1639	0.128	209	1421
0.057	96	1577	0.041	63	1461
0.028	42	1446	0.033	42	1217
0.031	39	1227	0.028	29	1012
0.027	77	2822	0.027	68	2405
0.018	24	1347	0.03	37	1179
0.078	38	449	0.125	52	363
0.032	44	1316	0.023	28	1186
0.021	40	1847	0.034	51	1449
0.023	27	1173	0.03	30	971
0.049	89	1729	0.054	81	1427
0.056	109	1854	0.057	94	1556
0.052	56	1019	0.058	49	803
0.027	107	3920	0.033	111	3223
0.025	46	1772	0.025	36	1429
0.03	132	4316	0.022	88	3843
0.034	85	2442	0.025	50	1956
0.058	107	1724	0.063	92	1372
0.062	147	2219	0.066	124	1759

0.023	27	1161	0.01	10	1007
0.027	70	2559	0.025	54	2070
0.025	17	673	0.024	15	612
0.03	66	2151	0.028	51	1743
0.019	36	1817	0.028	44	1522
0.049	40	773	0.067	45	629
0.301	323	751	0.369	384	658
0.038	44	1129	0.033	36	1063
0.021	59	2719	0.029	65	2205
0.019	25	1306	0.02	23	1131
0.086	99	1047	0.087	81	855
0.025	28	1072	0.018	17	931
0.024	70	2811	0.027	64	2343
0.049	34	659	0.027	17	608
0.023	51	2142	0.025	44	1741
0.012	23	1880	0.024	39	1607
0.027	47	1693	0.03	49	1570
0.019	18	935	0.016	11	678
0.019	12	615	0.055	29	501
0.032	86	2632	0.025	57	2214
0.023	81	3461	0.025	76	2959
0.025	65	2530	0.023	48	2056
0.063	165	2468	0.046	109	2238
0.042	90	2060	0.054	101	1778
0.023	31	1334	0.019	22	1137
0.022	49	2217	0.019	40	2026
0.026	83	3084	0.03	81	2599
0.03	61	1996	0.034	67	1889
0.03	47	1512	0.031	43	1338
0.074	200	2497	0.085	188	2032
0.025	37	1439	0.029	35	1179
0.031	102	3237	0.031	88	2797
0.063	52	769	0.076	57	696
0.021	51	2372	0.024	47	1882
0.061	121	1857	0.057	92	1518
0.042	78	1798	0.05	76	1436
0.029	76	2522	0.03	61	1970
0.026	31	1149	0.015	15	954
0.068	76	1041	0.062	60	901
0.109	52	427	0.135	49	314

0.029	42	1426	0.022	31	1391
0.027	62	2267	0.02	39	1954
0.026	41	1536	0.019	26	1315
0.02	56	2744	0.023	57	2441
0.021	31	1423	0.023	27	1134
0.051	91	1702	0.069	106	1435
0.11	76	612	0.149	86	490
0.018	26	1453	0.02	26	1269
0.031	98	3044	0.027	72	2610
0.058	107	1753	0.065	95	1375
0.028	29	996	0.015	15	969
0.019	48	2481	0.024	52	2088
0.05	71	1356	0.042	51	1177
0.055	84	1457	0.045	59	1238
0.334	552	1100	0.413	711	1010
0.043	39	876	0.032	22	669
0.023	68	2905	0.021	49	2246
0.036	45	1213	0.024	27	1087
0.018	12	665	0.038	21	531
0.037	28	736	0.025	16	617
0.025	17	658	0.032	20	601
0.024	26	1065	0.031	26	807
0.027	69	2474	0.026	51	1912
0.025	50	1976	0.033	56	1619
0.078	92	1090	0.076	76	920
0.02	25	1201	0.026	29	1086
0.021	81	3701	0.023	72	3043
0.023	53	2224	0.015	28	1825
0.026	111	4163	0.023	78	3386
0.079	44	516	0.11	59	475
0.034	53	1520	0.027	35	1253
0.021	66	3061	0.021	55	2576
0.031	64	1968	0.026	51	1896
0.026	42	1566	0.026	35	1332
0.015	21	1412	0.026	31	1174
0.025	26	1017	0.022	20	887
0.026	56	2078	0.03	60	1916
0.025	23	881	0.062	45	679
0.066	70	993	0.078	71	842
0.03	100	3289	0.027	78	2847

0.032	36	1094	0.025	21	825
0.039	107	2620	0.037	79	2053
0.032	54	1660	0.025	40	1556
0.047	29	592	0.045	25	526
0.243	201	627	0.333	274	548
0.015	13	874	0.032	25	746
0.028	57	1944	0.02	37	1794
0.03	16	513	0.033	15	442
0.019	22	1141	0.015	15	1011
0.022	72	3219	0.026	72	2666
0.066	200	2837	0.072	165	2140
0.03	37	1179	0.031	35	1095
0.019	67	3489	0.02	59	2914
0.025	60	2367	0.03	63	2028
0.064	95	1384	0.087	101	1065
0.04	19	460	0.035	14	388
0.051	184	3442	0.037	107	2763
0.026	61	2286	0.034	66	1904
0.028	54	1908	0.028	45	1584
0.018	70	3830	0.019	61	3108
0.024	25	1028	0.024	21	846
0.04	40	972	0.046	36	747
0.019	43	2228	0.021	42	1922
0.022	71	3085	0.023	62	2646
0.024	29	1175	0.028	30	1050
0.021	143	6730	0.028	167	5752
0.023	42	1775	0.029	45	1513
0.025	72	2789	0.024	53	2176
0.023	29	1242	0.018	19	1045
0.027	52	1860	0.034	55	1546
0.017	23	1323	0.027	29	1037
0.02	37	1789	0.02	29	1432
0.029	63	2084	0.021	34	1622
0.075	97	1203	0.064	66	967
0.029	100	3294	0.038	110	2789
0.026	27	1030	0.016	16	1013
0.018	19	1047	0.017	16	914
0.027	32	1168	0.017	15	884
0.021	22	1043	0.032	30	903
0.029	52	1756	0.021	38	1750

0.036	20	537	0.032	17	515
0.048	63	1253	0.06	67	1056
0.037	54	1396	0.074	83	1046
0.03	66	2135	0.025	45	1767
0.021	50	2355	0.023	50	2109
0.023	63	2720	0.022	53	2315
0.031	80	2496	0.033	68	2001
0.024	28	1133	0.024	25	1004
0.021	64	2953	0.022	57	2494
0.061	88	1347	0.057	62	1029
0.065	79	1144	0.053	60	1078
0.082	129	1445	0.075	97	1201
0.055	65	1112	0.085	83	890
0.024	148	5923	0.029	148	5028
0.027	54	1958	0.024	37	1532
0.031	50	1576	0.028	42	1438
0.034	41	1172	0.031	34	1052
0.029	77	2600	0.025	58	2290
0.024	14	558	0.012	5	423
0.02	91	4541	0.025	97	3817
0.036	80	2125	0.032	55	1647
0.028	59	2055	0.027	48	1707
0.018	28	1551	0.026	33	1246
0.143	76	454	0.154	76	417
0.055	75	1293	0.066	66	931
0.043	18	397	0.073	24	306
0.023	80	3409	0.021	65	3061
0.024	45	1801	0.023	38	1641
0.019	28	1484	0.021	27	1246
0.025	52	2025	0.028	52	1825
0.079	89	1044	0.096	91	854
0.029	49	1663	0.025	36	1409
0.023	70	2985	0.027	67	2421
0.03	64	2060	0.024	42	1706
0.032	34	1027	0.036	35	947
0.028	39	1345	0.032	42	1262
0.029	24	795	0.014	9	642
0.024	27	1113	0.013	12	929
0.028	33	1125	0.037	36	940
0.022	32	1448	0.036	46	1238

0.019	33	1677	0.025	35	1352
0.029	53	1805	0.036	56	1493
0.027	32	1166	0.02	19	944
0.018	33	1772	0.02	30	1488
0.019	40	2041	0.027	47	1718
0.032	63	1879	0.015	25	1676
0.1	220	1986	0.12	209	1527
0.106	74	621	0.159	99	524
0.082	69	775	0.082	55	614
0.026	45	1655	0.021	30	1389
0.024	39	1597	0.019	27	1411
0.023	58	2473	0.03	71	2266
0.078	90	1065	0.057	59	968
0.023	35	1492	0.027	35	1238
0.024	48	1935	0.021	33	1572
0.036	29	787	0.03	22	720
0.05	60	1151	0.084	73	799
0.045	38	813	0.106	82	694
0.034	70	1994	0.028	48	1640
0.018	19	1066	0.03	28	920
0.06	180	2800	0.061	143	2187
0.028	27	931	0.016	13	805
0.132	202	1331	0.168	208	1030
0.026	18	666	0.033	19	563
0.03	60	1933	0.031	50	1547
0.023	57	2442	0.023	45	1898
0.023	21	884	0.045	37	791
0.101	101	903	0.123	98	700
0.057	76	1249	0.061	63	962
0.024	24	993	0.014	12	821
0.03	57	1867	0.025	44	1700
0.021	44	2092	0.024	44	1792
0.021	51	2403	0.02	39	1961
0.061	57	872	0.096	69	648
0.076	99	1199	0.072	82	1062
0.029	35	1163	0.027	32	1133
0.021	62	2905	0.018	43	2356
0.021	22	1008	0.032	27	810
0.03	71	2290	0.032	64	1922
0.028	45	1575	0.03	39	1259

0.034	46	1327	0.022	25	1088
0.029	149	4988	0.029	124	4209
0.021	38	1793	0.029	42	1416
0.019	42	2114	0.023	40	1735
0.6	372	248	0.692	450	200
0.061	59	916	0.038	33	831
0.058	137	2219	0.065	121	1739
0.019	37	1871	0.016	25	1530
0.032	90	2717	0.023	58	2474
0.028	31	1092	0.022	21	955
0.022	52	2283	0.029	57	1916
0.023	32	1387	0.025	31	1219
0.019	28	1480	0.026	31	1168
0.026	77	2857	0.03	78	2552
0.019	62	3123	0.026	72	2729
0.073	89	1129	0.074	78	980
0.016	23	1381	0.019	24	1260
0.031	58	1813	0.031	48	1503
0.018	14	782	0.027	18	655
0.025	37	1452	0.036	42	1141
0.091	142	1421	0.093	125	1222
0.065	68	973	0.059	50	792
0.022	49	2174	0.022	42	1900
0.023	71	2962	0.029	77	2553
0.014	15	1073	0.029	24	806
0.032	137	4207	0.023	87	3637
0.028	70	2414	0.021	43	2045
0.072	123	1584	0.067	96	1329
0.02	81	3903	0.029	99	3345
0.02	60	2902	0.018	49	2602
0.025	67	2575	0.022	52	2300
0.02	55	2684	0.03	76	2458
0.024	77	3160	0.029	69	2321
0.026	58	2185	0.03	49	1568
0.023	34	1432	0.017	20	1137
0.021	57	2619	0.024	53	2192
0.066	56	798	0.108	80	662
0.032	42	1266	0.026	28	1050
0.028	66	2279	0.025	48	1873
0.03	46	1466	0.016	20	1260

0.024	82	3394	0.03	89	2918
0.062	74	1113	0.064	65	953
0.026	60	2216	0.026	55	2027
0.07	180	2393	0.093	193	1885
0.041	30	707	0.037	23	605
0.022	42	1903	0.034	55	1553
0.058	35	570	0.063	38	570
0.028	25	874	0.015	10	650
0.192	269	1133	0.224	286	988
0.024	67	2761	0.031	74	2301
0.024	41	1679	0.024	33	1341
0.093	49	479	0.1	51	459
0.032	29	881	0.027	19	697
0.061	108	1671	0.046	70	1441
0.063	60	895	0.073	61	775
0.022	65	2859	0.026	68	2543
0.021	45	2146	0.024	49	1959
0.015	15	1003	0.027	21	763
0.402	571	848	0.508	742	718
0.022	23	1044	0.018	16	851
0.031	63	1954	0.023	36	1521
0.022	16	716	0.028	17	598
0.018	30	1607	0.026	34	1282
0.025	34	1341	0.024	29	1169
0.018	37	1976	0.015	25	1681
0.348	924	1729	0.429	1213	1616
0.017	30	1701	0.026	38	1443
0.019	17	880	0.023	17	721
0.028	57	1976	0.031	60	1889
0.017	24	1379	0.023	26	1102
0.066	95	1347	0.075	82	1016
0.054	57	991	0.088	68	707
0.038	58	1487	0.029	39	1314
0.041	64	1512	0.036	49	1300
0.029	106	3560	0.027	89	3214
0.02	37	1856	0.028	45	1588
0.021	121	5623	0.02	99	4936
0.074	65	816	0.072	54	700
0.026	59	2187	0.023	42	1747
0.025	135	5161	0.027	117	4257

0.134	153	991	0.162	142	734
0.037	73	1926	0.031	51	1598
0.02	49	2367	0.023	47	1969
0.023	42	1804	0.027	41	1452
0.076	88	1077	0.097	83	776
0.016	28	1706	0.026	40	1521
0.122	143	1031	0.134	139	897
0.038	54	1355	0.059	66	1058
0.022	16	699	0.01	6	597
0.022	65	2907	0.022	58	2535
0.052	68	1236	0.044	46	996
0.017	37	2121	0.035	61	1687
0.018	82	4573	0.022	87	3911
0.028	67	2293	0.024	49	2026
0.018	37	2005	0.021	34	1619
0.023	40	1701	0.032	44	1348
0.06	89	1399	0.075	107	1329
0.185	220	968	0.242	262	821
0.027	24	871	0.021	13	610
0.079	94	1093	0.092	96	953
0.042	58	1333	0.071	88	1158
0.367	941	1620	0.439	1102	1407
0.02	25	1200	0.022	25	1113
0.051	79	1485	0.079	100	1158
0.028	64	2233	0.028	49	1697
0.023	32	1364	0.024	28	1117
0.066	53	750	0.046	31	641
0.023	24	999	0.017	14	833
0.017	60	3387	0.026	70	2606
0.023	56	2430	0.025	52	2017
0.022	31	1360	0.022	25	1095
0.024	32	1316	0.03	36	1167
0.094	102	983	0.121	104	757
0.062	35	534	0.114	50	390
0.024	34	1405	0.032	37	1120
0.025	24	943	0.023	18	749
0.028	57	1996	0.031	58	1828
0.023	52	2194	0.034	61	1713
0.032	91	2733	0.035	82	2272
0.02	50	2466	0.027	51	1864

0.015	17	1153	0.023	22	944
0.014	27	1888	0.019	34	1798
0.023	14	607	0.052	26	477
0.031	55	1695	0.026	37	1409
0.025	65	2551	0.028	61	2093
0.025	88	3454	0.03	95	3093
0.071	123	1600	0.07	103	1367
0.056	78	1316	0.061	69	1054
0.031	46	1444	0.035	43	1192
0.097	139	1292	0.114	139	1078
0.028	49	1701	0.017	24	1422
0.079	79	916	0.08	64	733
0.026	55	2043	0.022	43	1888
0.03	40	1276	0.024	26	1061
0.056	111	1860	0.065	112	1611
0.044	49	1068	0.065	57	817
0.028	48	1672	0.024	37	1515
0.031	66	2043	0.028	52	1790
0.067	76	1056	0.044	42	921
0.028	51	1752	0.025	38	1465
0.02	20	996	0.025	23	895
0.025	36	1423	0.023	25	1078
0.016	24	1503	0.021	28	1291
0.025	97	3727	0.029	99	3292
0.021	81	3846	0.025	81	3184
0.023	76	3178	0.021	61	2798
0.028	28	988	0.019	17	860
0.032	17	518	0.043	20	447
0.068	66	901	0.109	89	730
0.02	47	2357	0.028	60	2113
0.023	33	1392	0.027	31	1138
0.026	39	1434	0.018	21	1154
0.029	41	1374	0.022	26	1152
0.101	229	2031	0.14	256	1575
0.031	48	1496	0.035	42	1162
0.022	66	2995	0.029	74	2438
0.32	211	448	0.41	267	384
0.093	207	2026	0.139	245	1516
0.027	58	2116	0.035	61	1703
0.021	90	4214	0.024	86	3569

0.029	86	2846	0.023	55	2365
0.019	10	507	0.018	8	427
0.019	20	1011	0.024	19	789
0.02	52	2508	0.028	64	2200
0.022	48	2116	0.024	41	1674
0.025	89	3489	0.028	87	3002
0.055	67	1158	0.059	63	998
0.019	24	1266	0.026	28	1057
0.081	62	703	0.1	64	573
0.025	19	748	0.037	25	658
0.018	38	2053	0.022	40	1810
0.014	12	816	0.026	21	786
0.068	123	1679	0.06	89	1403
0.03	45	1444	0.012	16	1269
0.059	83	1322	0.049	56	1094
0.025	51	1963	0.025	42	1634
0.025	51	1970	0.019	30	1517
0.031	108	3357	0.021	65	3016
0.024	37	1524	0.025	33	1308
0.02	31	1533	0.027	36	1315
0.018	22	1224	0.029	31	1042
0.221	253	892	0.322	346	728
0.025	27	1049	0.038	36	917
0.058	206	3370	0.054	164	2862
0.054	73	1291	0.081	89	1015
0.03	112	3622	0.026	84	3141
0.052	45	824	0.053	40	714
0.052	59	1076	0.048	49	963
0.019	32	1682	0.024	35	1440
0.034	42	1189	0.039	42	1033
0.027	39	1388	0.018	22	1194
0.027	97	3454	0.028	81	2840
0.12	149	1088	0.163	173	889
0.024	28	1147	0.043	42	941
0.024	33	1325	0.023	26	1099
0.065	141	2014	0.058	100	1617
0.029	75	2508	0.026	52	1981
0.036	33	887	0.045	36	770
0.018	32	1728	0.026	38	1421
0.033	42	1224	0.028	29	1024

0.059	155	2453	0.071	153	1989
0.032	55	1681	0.031	45	1387
0.059	100	1589	0.062	89	1350
0.059	114	1834	0.062	104	1567
0.021	64	2982	0.029	72	2390
0.078	103	1219	0.096	103	973
0.021	34	1621	0.032	49	1484
0.022	19	862	0.024	18	725
0.021	58	2749	0.018	41	2245
0.026	73	2710	0.023	54	2339
0.028	42	1462	0.029	38	1280
0.038	55	1398	0.024	30	1215
0.042	25	564	0.024	13	518
0.06	61	950	0.088	71	737
0.027	36	1303	0.026	28	1060
0.025	57	2264	0.027	57	2065
0.02	38	1831	0.023	34	1420
0.062	136	2070	0.055	98	1698
0.028	17	595	0.039	22	544
0.021	17	783	0.03	21	671
0.079	61	709	0.091	55	551
0.026	59	2193	0.017	30	1708
0.06	217	3427	0.04	123	2976
0.033	33	977	0.026	20	741
0.029	57	1924	0.027	48	1730
0.07	68	907	0.091	70	701
0.032	34	1013	0.035	32	876
0.023	65	2718	0.02	48	2388
0.019	23	1165	0.028	27	953
0.023	39	1685	0.022	33	1476
0.016	17	1068	0.027	23	823
0.019	21	1068	0.024	21	856
0.023	58	2506	0.023	49	2053
0.034	41	1151	0.034	34	963
0.059	78	1233	0.051	51	944
0.017	25	1455	0.019	22	1114
0.042	44	998	0.058	46	749
0.031	110	3449	0.033	101	2948
0.025	60	2386	0.03	70	2249
0.056	111	1858	0.063	104	1544

0.018	43	2284	0.02	39	1906
0.025	62	2389	0.022	43	1922
0.023	88	3813	0.024	73	2953
0.143	148	886	0.146	111	651
0.05	96	1837	0.045	74	1575
0.02	64	3089	0.029	75	2548
0.025	55	2186	0.019	36	1871
0.032	34	1044	0.026	24	886
0.064	76	1108	0.064	70	1027
0.071	137	1786	0.073	108	1373
0.023	55	2370	0.026	54	2019
0.07	209	2788	0.081	205	2322
0.023	72	3036	0.022	52	2349
0.02	28	1384	0.02	24	1177
0.019	34	1795	0.018	28	1562
0.03	69	2221	0.031	56	1734
0.019	18	927	0.036	31	841
0.015	25	1663	0.038	61	1540
0.02	27	1303	0.025	32	1250
0.02	68	3309	0.027	78	2850
0.015	22	1415	0.013	15	1128
0.023	54	2286	0.03	58	1871
0.059	78	1243	0.065	67	961
0.171	120	582	0.238	162	518
0.021	32	1482	0.021	28	1310
0.059	129	2072	0.065	123	1766
0.02	25	1246	0.026	25	937
0.022	29	1287	0.03	36	1182
0.031	34	1073	0.021	20	940
0.067	64	886	0.073	51	650
0.024	34	1377	0.017	21	1191
0.03	74	2403	0.027	53	1942
0.09	263	2652	0.127	308	2117
0.034	28	789	0.024	16	656
0.038	23	584	0.021	10	477
0.033	46	1364	0.025	28	1112
0.061	111	1705	0.071	104	1365
0.048	83	1640	0.064	86	1261
0.024	33	1343	0.021	24	1138
0.026	55	2049	0.028	46	1606

0.058	81	1319	0.054	67	1183
0.057	37	607	0.113	61	477
0.041	146	3377	0.051	150	2766
0.026	76	2899	0.021	55	2519
0.072	125	1615	0.061	93	1442
0.022	23	1041	0.022	19	859
0.056	53	900	0.046	39	802
0.022	58	2634	0.022	49	2183
0.027	44	1602	0.025	32	1254
0.062	131	1979	0.05	95	1798
0.024	77	3131	0.029	73	2437
0.022	10	455	0.025	8	312
0.023	28	1216	0.028	29	1021
0.022	55	2486	0.02	41	2015
0.023	64	2762	0.029	61	2068
0.142	102	614	0.154	93	512
0.021	15	692	0.016	10	611
0.03	43	1400	0.022	27	1174
0.028	91	3141	0.02	55	2733
0.021	27	1286	0.025	27	1048
0.058	88	1421	0.054	71	1248
0.021	35	1625	0.03	42	1350
0.062	118	1782	0.067	109	1520
0.034	47	1336	0.024	27	1115
0.017	34	1948	0.021	37	1695
0.021	31	1415	0.015	19	1212
0.025	61	2423	0.026	52	1922
0.022	59	2595	0.026	56	2111
0.115	64	491	0.194	96	399
0.036	20	538	0.039	17	423
0.14	254	1566	0.204	338	1321
0.051	96	1786	0.032	53	1580
0.053	46	820	0.05	40	754
0.025	20	778	0.017	10	566
0.023	58	2460	0.029	65	2212
0.143	126	753	0.15	113	641
0.027	70	2530	0.025	56	2171
0.063	137	2036	0.059	102	1634
0.06	97	1520	0.069	96	1301
0.029	72	2437	0.025	58	2283

0.031	17	532	0.016	8	482
0.191	253	1071	0.251	326	975
0.147	105	610	0.153	82	454
0.027	70	2547	0.032	74	2252
0.023	25	1041	0.034	30	864
0.029	84	2775	0.027	66	2359
0.023	63	2620	0.024	57	2350
0.034	41	1156	0.037	38	999
0.03	70	2274	0.021	42	1982
0.03	30	977	0.028	25	875
0.026	20	742	0.026	17	635
0.027	37	1322	0.029	32	1058
0.023	39	1640	0.031	37	1170
0.017	15	882	0.025	18	709
0.062	86	1309	0.044	51	1108
0.274	274	727	0.333	282	564
0.024	52	2111	0.024	40	1599
0.053	54	966	0.052	41	744
0.023	38	1642	0.031	45	1415
0.062	122	1858	0.059	103	1644
0.032	54	1638	0.026	37	1411
0.026	61	2330	0.023	46	1920
0.02	44	2154	0.033	58	1704
0.054	106	1853	0.067	109	1513
0.051	94	1752	0.04	63	1529
0.142	251	1518	0.202	308	1214
0.157	116	621	0.238	142	454
0.021	68	3146	0.028	74	2610
0.02	49	2345	0.04	77	1830
0.034	102	2934	0.029	75	2475
0.024	37	1525	0.022	29	1293
0.055	113	1926	0.041	69	1596
0.022	33	1435	0.027	33	1170
0.077	83	999	0.078	69	814
0.055	106	1806	0.059	102	1640
0.028	65	2279	0.033	65	1892
0.019	23	1158	0.031	28	871
0.032	53	1602	0.022	28	1245
0.103	126	1094	0.16	183	961
0.025	39	1525	0.024	31	1268

0.02	115	5647	0.024	115	4766
0.024	82	3334	0.027	75	2751
0.046	73	1524	0.058	77	1247
0.063	54	804	0.101	73	649
0.026	69	2569	0.024	50	2070
0.029	112	3742	0.033	111	3235
0.023	61	2620	0.034	75	2112
0.024	34	1385	0.035	42	1144
0.027	30	1090	0.027	25	913
0.023	41	1746	0.033	48	1394
0.028	62	2126	0.025	45	1746
0.028	59	2071	0.031	51	1603
0.025	71	2730	0.021	54	2464
0.021	15	714	0.031	19	603
0.033	30	887	0.043	32	717
0.014	17	1173	0.017	17	965
0.048	43	852	0.075	52	640
0.04	68	1639	0.06	75	1175
0.052	47	851	0.056	41	688
0.02	22	1089	0.032	28	856
0.03	94	3011	0.025	72	2832
0.028	47	1649	0.019	28	1422
0.118	204	1520	0.178	256	1186
0.027	56	2042	0.036	64	1706
0.021	38	1807	0.017	28	1589
0.028	62	2178	0.021	39	1802
0.024	35	1438	0.033	43	1257
0.023	46	1961	0.034	59	1689
0.052	34	618	0.054	28	491
0.019	59	3019	0.023	60	2596
0.03	74	2419	0.026	57	2119
0.065	75	1083	0.052	51	929
0.019	42	2181	0.017	34	1963
0.029	38	1255	0.02	22	1074
0.028	66	2298	0.024	46	1872
0.024	81	3249	0.021	62	2906
0.02	43	2112	0.027	51	1837
0.087	93	981	0.124	124	879
0.029	63	2104	0.027	48	1731
0.023	34	1456	0.027	33	1187

0.056	162	2708	0.051	131	2435
0.027	36	1320	0.023	26	1091
0.06	103	1621	0.062	92	1399
0.058	46	751	0.083	59	650
0.025	25	995	0.028	23	794
0.022	46	2035	0.022	39	1705
0.029	52	1742	0.039	61	1508
0.022	74	3363	0.026	73	2700
0.045	49	1048	0.031	30	932
0.023	66	2821	0.027	67	2378
0.026	17	637	0.043	22	491
0.021	58	2665	0.026	62	2288
0.085	60	650	0.047	28	568
0.023	38	1599	0.023	32	1374
0.029	32	1082	0.021	19	887
0.054	169	2944	0.05	129	2461
0.029	53	1753	0.025	41	1594
0.029	110	3635	0.026	80	3007
0.03	52	1681	0.023	36	1531
0.022	33	1497	0.023	29	1216
0.043	31	698	0.069	43	577
0.025	57	2266	0.029	55	1819
0.021	70	3257	0.018	52	2792
0.017	42	2489	0.027	59	2157
0.107	89	741	0.13	89	595
0.021	72	3280	0.023	63	2723
0.023	37	1543	0.03	41	1333
0.123	75	534	0.126	70	485
0.019	33	1665	0.027	37	1356
0.073	71	901	0.118	93	698
0.028	63	2169	0.035	69	1929
0.024	48	1935	0.03	49	1579
0.019	73	3705	0.021	68	3240
0.014	22	1534	0.021	25	1177
0.595	485	330	0.661	640	328
0.052	46	834	0.083	59	654
0.023	23	975	0.022	21	915
0.029	60	1993	0.026	41	1551
0.026	138	5129	0.023	105	4523
0.034	70	2017	0.023	44	1909

0.028	53	1848	0.026	44	1665
0.022	29	1307	0.037	39	1006
0.195	222	917	0.249	266	802
0.023	48	2022	0.036	63	1697
0.02	76	3707	0.027	79	2855
0.046	132	2736	0.067	158	2201
0.214	268	986	0.316	372	805
0.068	80	1091	0.058	53	859
0.024	42	1699	0.025	40	1564
0.028	69	2383	0.029	63	2096
0.073	108	1364	0.062	74	1116
0.027	42	1527	0.031	43	1329
0.058	77	1244	0.071	79	1036
0.034	31	868	0.042	30	678
0.027	21	751	0.034	21	602
0.057	55	912	0.081	71	803
0.027	132	4802	0.026	110	4114
0.092	94	933	0.112	94	746
0.037	43	1112	0.043	49	1093
0.019	42	2183	0.016	30	1815
0.027	43	1553	0.019	27	1360
0.024	23	944	0.027	23	838
0.018	28	1495	0.025	33	1279
0.044	44	964	0.038	32	801
0.049	60	1164	0.054	60	1044
0.021	27	1234	0.019	19	1001
0.023	18	781	0.023	17	708
0.018	24	1342	0.022	27	1176
0.049	52	999	0.071	65	851
0.063	86	1289	0.057	65	1066
0.029	62	2094	0.033	63	1822
0.025	13	502	0.026	11	407
0.027	33	1197	0.019	23	1180
0.022	25	1093	0.024	24	985
0.02	27	1292	0.035	42	1151
0.02	36	1769	0.032	50	1491
0.018	20	1100	0.029	26	863
0.026	25	932	0.035	30	816
0.06	70	1088	0.054	55	962
0.046	78	1633	0.045	64	1354

0.025	21	836	0.034	26	730
0.029	49	1665	0.03	47	1498
0.082	93	1047	0.072	66	854
0.026	35	1291	0.022	26	1155
0.022	55	2418	0.023	48	2010
0.023	102	4277	0.026	95	3578
0.065	148	2139	0.045	91	1923
0.38	268	438	0.48	393	425
0.024	42	1685	0.025	37	1439
0.062	69	1045	0.073	65	828
0.022	54	2456	0.021	42	2002
0.022	92	4029	0.025	89	3410
0.077	44	527	0.123	56	398
0.06	49	765	0.065	51	731
0.029	28	925	0.021	19	868
0.019	73	3703	0.018	60	3251
0.022	35	1527	0.021	30	1411
0.14	114	700	0.171	107	519
0.069	57	773	0.08	59	681
0.095	112	1064	0.061	56	855
0.026	27	1026	0.034	30	860
0.103	101	880	0.148	130	746
0.037	41	1059	0.045	46	966
0.021	30	1404	0.022	26	1143
0.031	21	659	0.022	13	589
0.027	49	1745	0.037	60	1581
0.035	21	573	0.067	28	389
0.023	38	1647	0.022	38	1680
0.024	98	3909	0.025	92	3638
0.019	35	1779	0.025	39	1543
0.024	31	1249	0.026	30	1142
0.024	86	3455	0.03	90	2891
0.02	93	4662	0.027	102	3616
0.06	90	1411	0.078	110	1294
0.017	29	1670	0.017	27	1555
0.08	100	1146	0.076	78	945
0.149	125	715	0.18	132	600
0.051	88	1629	0.064	89	1303
0.341	266	514	0.467	315	359
0.058	147	2408	0.048	107	2105

0.028	31	1094	0.033	28	828
0.021	42	1999	0.026	43	1641
0.025	40	1534	0.03	38	1223
0.023	55	2314	0.024	44	1798
0.019	25	1306	0.029	28	954
0.022	53	2306	0.021	44	2041
0.019	37	1908	0.024	42	1740
0.038	42	1071	0.043	42	946
0.024	89	3565	0.023	71	3046
0.021	97	4596	0.023	94	3937
0.045	65	1394	0.059	73	1174
0.021	41	1953	0.028	44	1528
0.059	112	1801	0.048	80	1586
0.021	26	1207	0.031	34	1074
0.032	54	1638	0.028	39	1351
0.025	74	2927	0.019	53	2667
0.033	18	522	0.026	10	368
0.023	23	977	0.026	24	899
0.023	36	1560	0.025	32	1249
0.049	87	1678	0.051	73	1358
0.019	34	1724	0.023	35	1475
0.016	63	3919	0.028	100	3421
0.027	41	1497	0.021	29	1330
0.041	46	1079	0.034	34	966
0.163	278	1429	0.14	213	1312
0.07	32	426	0.061	23	355
0.026	32	1176	0.033	30	883
0.025	44	1741	0.017	29	1638
0.059	48	764	0.083	58	639
0.021	42	1940	0.026	44	1619
0.03	24	776	0.029	22	738
0.026	118	4367	0.031	120	3785
0.019	41	2136	0.026	44	1651
0.027	70	2554	0.022	47	2135
0.028	27	953	0.036	28	746
0.071	79	1033	0.099	96	875
0.021	40	1890	0.015	24	1584
0.019	41	2094	0.018	33	1803
0.031	44	1364	0.026	31	1144
0.029	50	1689	0.023	34	1432

0.022	34	1488	0.028	35	1218
0.027	30	1064	0.025	22	849
0.023	80	3338	0.025	74	2844
0.029	67	2274	0.03	61	1999
0.03	41	1305	0.024	26	1076
0.025	40	1566	0.026	35	1332
0.076	114	1382	0.071	84	1097
0.059	79	1256	0.051	61	1146
0.021	30	1409	0.024	28	1118
0.026	86	3252	0.028	79	2731
0.089	47	482	0.032	13	390
0.02	37	1852	0.02	33	1578
0.024	57	2312	0.019	37	1905
0.059	103	1643	0.094	143	1378
0.021	43	2021	0.025	41	1574
0.027	89	3260	0.019	58	2942
0.088	75	780	0.085	52	561
0.025	35	1350	0.024	32	1279
0.029	44	1478	0.023	28	1206
0.019	29	1496	0.027	34	1210
0.036	46	1248	0.022	26	1135
0.095	52	494	0.115	53	406
0.086	72	769	0.106	83	702
0.024	84	3359	0.022	64	2851
0.091	66	659	0.105	60	514
0.029	44	1466	0.028	36	1257
0.05	56	1055	0.046	40	838
0.027	29	1031	0.026	21	797
0.037	35	915	0.047	38	775
0.06	49	761	0.045	32	682
0.022	42	1850	0.018	30	1617
0.112	101	802	0.138	90	563
0.065	166	2376	0.069	158	2116
0.026	23	877	0.042	28	641
0.019	34	1771	0.021	32	1490
0.051	38	705	0.057	32	533
0.033	29	862	0.017	13	730
0.028	58	1978	0.025	45	1789
0.023	80	3341	0.027	79	2822
0.028	36	1264	0.02	23	1133

0.155	143	777	0.206	178	684
0.028	52	1799	0.025	39	1543
0.095	124	1179	0.113	126	992
0.018	53	2941	0.03	82	2688
0.019	80	4177	0.026	87	3311
0.02	57	2728	0.035	81	2226
0.019	41	2140	0.02	37	1840
0.03	45	1440	0.034	41	1157
0.018	46	2450	0.018	37	1986
0.018	27	1508	0.02	27	1333
0.071	88	1152	0.063	70	1043
0.064	128	1874	0.066	109	1555
0.033	66	1948	0.022	45	2021
0.023	80	3351	0.024	70	2894
0.053	83	1471	0.065	80	1150
0.013	23	1752	0.027	38	1374
0.019	49	2501	0.03	60	1923
0.022	41	1817	0.027	44	1580
0.019	24	1218	0.028	28	975
0.016	16	960	0.025	19	751
0.085	138	1489	0.123	149	1066
0.019	50	2528	0.022	50	2226
0.019	33	1721	0.015	23	1478
0.026	27	1013	0.031	25	791
0.028	64	2209	0.031	58	1788
0.052	103	1859	0.058	96	1562
0.018	22	1198	0.02	20	992
0.054	74	1293	0.055	57	978
0.03	54	1745	0.022	34	1498
0.03	57	1868	0.033	55	1587
0.049	117	2265	0.069	132	1775
0.024	50	2029	0.039	70	1730
0.02	23	1110	0.026	24	908
0.015	27	1723	0.015	22	1461
0.034	26	740	0.028	17	598
0.187	168	730	0.301	232	540
0.032	80	2406	0.027	58	2109
0.057	87	1437	0.047	62	1248
0.177	95	442	0.239	111	353
0.02	24	1176	0.026	27	1013

0.021	46	2125	0.032	55	1677
0.026	57	2175	0.035	71	1968
0.021	27	1231	0.022	23	1004
0.031	49	1535	0.025	34	1347
0.021	75	3491	0.023	67	2858
0.068	38	520	0.045	22	469
0.021	29	1359	0.036	46	1215
0.029	76	2543	0.025	50	1986
0.037	66	1734	0.042	59	1356
0.025	18	688	0.02	13	642
0.013	19	1442	0.026	34	1284
0.056	32	540	0.039	21	513
0.096	111	1049	0.116	122	932
0.036	71	1902	0.05	85	1622
0.054	110	1915	0.056	103	1738
0.031	60	1855	0.031	46	1443
0.016	12	718	0.024	15	620
0.028	100	3474	0.025	74	2874
0.019	77	4047	0.024	87	3609
0.038	48	1216	0.033	33	963
0.02	20	959	0.02	16	791
0.032	44	1324	0.022	27	1185
0.018	30	1653	0.034	47	1335
0.024	87	3508	0.023	73	3051
0.042	48	1095	0.035	35	964
0.022	50	2201	0.023	44	1911
0.023	58	2460	0.027	60	2184
0.059	73	1154	0.059	60	958
0.031	71	2224	0.017	31	1797
0.026	62	2295	0.022	41	1851
0.019	23	1168	0.029	30	999
0.016	7	440	0.024	11	438
0.026	42	1581	0.027	35	1258
0.024	100	4072	0.027	98	3492
0.065	147	2125	0.05	99	1863
0.027	66	2358	0.023	47	1988
0.028	53	1871	0.03	47	1519
0.028	31	1083	0.03	31	1009
0.069	66	889	0.074	61	768
0.083	71	785	0.058	41	672

0.037	54	1402	0.021	25	1160
0.032	42	1291	0.034	46	1307
0.06	133	2071	0.09	143	1447
0.054	53	921	0.055	44	756
0.194	338	1401	0.231	368	1223
0.022	35	1545	0.027	39	1386
0.02	33	1624	0.016	24	1450
0.017	37	2141	0.028	50	1747
0.023	101	4285	0.026	107	3959
0.026	27	1020	0.023	21	873
0.027	40	1433	0.019	24	1260
0.022	29	1304	0.028	31	1090
0.024	46	1900	0.024	42	1705
0.06	104	1639	0.055	81	1383
0.051	146	2700	0.04	102	2448
0.026	47	1747	0.038	56	1435
0.074	44	549	0.076	37	452
0.071	109	1418	0.075	97	1200
0.022	35	1567	0.029	39	1321
0.025	43	1700	0.018	25	1401
0.026	74	2784	0.025	61	2371
0.029	62	2079	0.027	52	1856
0.041	39	914	0.042	30	679
0.02	23	1106	0.029	27	899
0.036	71	1882	0.027	47	1697
0.026	92	3383	0.029	89	2938
0.016	26	1650	0.035	50	1388
0.035	39	1063	0.026	24	909
0.079	133	1553	0.054	74	1301
0.022	55	2401	0.026	57	2135
0.024	41	1703	0.026	37	1391
0.019	18	946	0.033	27	793
0.02	38	1832	0.02	31	1492
0.029	23	768	0.019	13	683
0.034	88	2511	0.033	77	2246
0.02	60	2990	0.025	68	2695
0.03	30	973	0.028	25	880
0.019	24	1207	0.028	29	991
0.028	41	1439	0.023	28	1210
0.028	35	1227	0.023	25	1064

0.038	47	1182	0.015	15	989
0.061	43	667	0.084	50	543
0.052	60	1085	0.065	63	911
0.023	24	1017	0.014	13	912
0.016	24	1511	0.037	52	1364
0.024	56	2313	0.033	67	1978
0.868	2278	345	0.905	3001	315
0.034	43	1208	0.031	32	1006
0.029	87	2867	0.029	69	2337
0.015	8	526	0.055	22	381
0.053	56	999	0.072	57	740
0.026	58	2201	0.036	62	1653
0.025	57	2184	0.028	56	1912
0.02	39	1930	0.021	35	1636
0.022	51	2242	0.028	53	1870
0.023	64	2680	0.024	55	2273
0.025	20	776	0.042	26	589
0.027	124	4386	0.019	75	3809
0.024	13	518	0.028	13	444
0.033	35	1023	0.031	28	890
0.025	91	3596	0.03	97	3101
0.025	105	4082	0.032	120	3627
0.031	98	3062	0.032	82	2461
0.06	76	1201	0.038	37	947
0.017	22	1261	0.031	33	1017
0.054	73	1279	0.055	66	1127
0.028	80	2785	0.033	72	2124
0.027	44	1597	0.017	25	1469
0.018	51	2769	0.023	58	2436
0.025	44	1729	0.026	41	1522
0.017	42	2383	0.029	57	1882
0.027	34	1247	0.028	29	1012
0.061	143	2188	0.1	202	1810
0.024	43	1773	0.026	39	1483
0.022	44	1947	0.021	35	1659
0.024	57	2333	0.02	44	2131
0.07	80	1057	0.07	63	840
0.053	91	1610	0.049	72	1406
0.024	35	1453	0.038	48	1215
0.025	47	1856	0.019	28	1463

0.022	17	751	0.03	18	578
0.069	108	1448	0.071	88	1155
0.026	31	1169	0.021	19	896
0.053	35	631	0.046	26	540
0.022	34	1513	0.022	29	1299
0.128	132	902	0.166	150	754
0.095	56	536	0.095	47	449
0.027	69	2499	0.021	50	2315
0.021	82	3879	0.021	64	2985
0.02	42	2027	0.026	42	1602
0.026	31	1163	0.031	31	954
0.095	73	698	0.133	89	578
0.032	56	1678	0.021	30	1411
0.019	26	1351	0.028	34	1177
0.042	54	1236	0.038	41	1047
0.013	31	2388	0.03	61	1952
0.021	38	1763	0.02	30	1503
0.021	58	2677	0.022	52	2335
0.032	54	1639	0.028	38	1298
0.065	150	2172	0.067	123	1713
0.031	73	2270	0.019	37	1963
0.024	89	3652	0.026	84	3107
0.018	13	713	0.027	17	613
0.03	45	1457	0.016	19	1163
0.09	60	610	0.126	78	543
0.029	55	1813	0.035	57	1575
0.028	28	986	0.029	25	825
0.074	140	1758	0.054	89	1564
0.072	43	556	0.08	45	519
0.035	31	852	0.027	17	614
0.039	94	2311	0.035	72	1968
0.067	191	2657	0.059	144	2278
0.094	127	1229	0.089	100	1020
0.03	143	4563	0.025	97	3796
0.02	42	2087	0.024	44	1812
0.019	17	899	0.027	21	766
0.028	45	1591	0.021	29	1356
0.051	56	1038	0.055	51	881
0.015	8	532	0.027	14	504
0.015	25	1600	0.037	49	1273

0.027	51	1824	0.023	35	1464
0.061	121	1858	0.07	116	1538
0.028	45	1588	0.025	38	1453
0.034	105	2985	0.037	97	2513
0.024	84	3395	0.026	68	2581
0.024	34	1380	0.018	22	1228
0.018	29	1601	0.023	33	1401
0.025	68	2667	0.026	56	2134
0.019	32	1637	0.033	48	1413
0.02	19	921	0.007	5	693
0.062	79	1191	0.056	57	965
0.04	50	1203	0.022	23	1006
0.024	21	848	0.024	18	719
0.061	98	1513	0.061	81	1250
0.016	21	1288	0.015	18	1150
0.066	87	1228	0.065	70	1008
0.026	61	2324	0.022	41	1839
0.028	45	1560	0.032	42	1279
0.025	38	1498	0.028	33	1156
0.076	44	537	0.127	70	482
0.027	69	2532	0.032	76	2265
0.061	110	1693	0.07	112	1483
0.022	72	3141	0.03	82	2647
0.021	26	1204	0.03	29	948
0.066	99	1398	0.064	75	1099
0.026	61	2290	0.021	40	1884
0.121	92	668	0.15	84	476
0.026	57	2107	0.029	54	1809
0.02	29	1402	0.032	37	1103
0.024	45	1815	0.023	38	1642
0.026	39	1435	0.021	27	1242
0.021	44	2009	0.025	40	1584
0.063	79	1179	0.075	77	950
0.029	38	1257	0.042	50	1138
0.027	33	1177	0.022	21	938
0.024	38	1553	0.023	32	1388
0.061	46	713	0.075	53	653
0.03	46	1485	0.031	41	1274
0.025	19	736	0.034	22	630
0.034	76	2152	0.03	60	1930

0.073	82	1043	0.095	88	843
0.078	51	599	0.095	56	531
0.018	20	1067	0.027	26	929
0.023	30	1278	0.026	32	1219
0.025	162	6426	0.027	161	5767
0.022	46	2011	0.019	32	1634
0.135	166	1065	0.154	162	887
0.07	128	1701	0.055	78	1340
0.019	15	774	0.011	6	552
0.021	21	978	0.031	29	909
0.04	39	936	0.085	78	835
0.027	53	1937	0.017	29	1685
0.023	38	1592	0.03	44	1433
0.027	58	2055	0.019	35	1855
0.016	26	1600	0.023	32	1364
0.031	49	1508	0.031	41	1273
0.025	68	2701	0.029	65	2161
0.024	27	1114	0.019	19	967
0.059	191	3071	0.063	171	2554
0.024	22	884	0.008	7	835
0.028	117	4026	0.027	100	3648
0.024	37	1514	0.028	36	1250
0.026	75	2805	0.025	59	2330
0.017	33	1881	0.034	56	1601
0.346	336	636	0.496	484	492
0.034	17	488	0.064	30	441
0.028	51	1762	0.025	40	1571
0.016	16	966	0.028	22	767
0.035	59	1638	0.025	36	1419
0.026	63	2331	0.03	61	1987
0.062	136	2060	0.04	75	1808
0.031	89	2753	0.03	78	2484
0.069	57	772	0.082	53	593
0.018	30	1644	0.033	47	1365
0.052	58	1048	0.064	63	918
0.012	18	1540	0.023	28	1187
0.031	51	1592	0.022	32	1423
0.018	33	1829	0.04	55	1335
0.081	132	1498	0.048	72	1414
0.02	31	1515	0.015	19	1213

0.055	65	1112	0.048	51	1017
0.033	67	1934	0.019	33	1705
0.342	281	540	0.439	347	443
0.027	35	1262	0.033	37	1096
0.054	102	1779	0.069	108	1467
0.03	46	1508	0.032	44	1344
0.028	92	3137	0.04	103	2446
0.016	43	2616	0.023	52	2193
0.012	10	804	0.017	12	714
0.042	112	2524	0.048	98	1939
0.024	103	4132	0.02	73	3569
0.082	45	506	0.145	65	384
0.023	44	1837	0.026	43	1595
0.077	77	917	0.098	81	746
0.026	22	826	0.041	27	639
0.022	88	3838	0.02	62	3099
0.33	344	697	0.488	607	637
0.03	37	1182	0.025	27	1048
0.013	17	1323	0.027	27	956
0.062	70	1068	0.051	48	897
0.037	37	957	0.038	29	742
0.039	50	1219	0.047	60	1216
0.021	33	1518	0.026	35	1301
0.088	203	2097	0.078	148	1751
0.023	25	1064	0.026	24	917
0.024	60	2462	0.02	41	2035
0.704	1140	480	0.743	1443	499
0.028	29	998	0.033	26	754
0.022	82	3587	0.027	87	3117
0.025	35	1388	0.025	32	1238
0.055	12	207	0	0	133
0.032	90	2725	0.028	69	2379
0.063	171	2530	0.063	142	2121
0.027	62	2269	0.032	70	2117
0.025	85	3363	0.028	87	2991
0.062	98	1476	0.044	56	1215
0.022	23	1018	0.023	20	857
0.016	74	4572	0.024	93	3859
0.064	151	2200	0.079	143	1674
0.018	21	1165	0.016	15	916

0.024	73	2976	0.025	72	2859
0.028	50	1759	0.026	40	1514
0.035	35	968	0.029	24	799
0.016	30	1859	0.024	38	1560
0.026	27	1030	0.027	25	899
0.052	75	1377	0.041	51	1190
0.019	43	2165	0.025	49	1941
0.03	40	1283	0.03	32	1043
0.03	67	2150	0.023	41	1763
0.021	45	2145	0.028	53	1810
0.039	77	1921	0.032	55	1672
0.095	120	1146	0.127	128	882
0.028	43	1519	0.029	42	1410
0.023	74	3106	0.028	77	2656
0.033	53	1545	0.031	45	1385
0.057	209	3483	0.062	188	2856
0.027	32	1136	0.022	23	1041
0.029	91	3014	0.026	65	2443
0.019	22	1156	0.022	24	1048
0.016	51	3045	0.028	74	2577
0.02	72	3488	0.017	50	2967
0.032	50	1514	0.023	31	1299
0.015	16	1026	0.012	10	846
0.023	22	954	0.029	27	889
0.021	26	1206	0.021	21	1000
0.02	125	6160	0.022	129	5612
0.018	41	2208	0.035	66	1825
0.025	46	1774	0.017	26	1465
0.023	30	1285	0.015	17	1116
0.024	52	2132	0.027	50	1791
0.024	83	3307	0.023	64	2728
0.017	22	1306	0.03	35	1122
0.036	38	1007	0.035	31	847
0.029	58	1964	0.027	41	1485
0.032	94	2873	0.028	69	2420
0.019	18	917	0.025	21	821
0.08	78	899	0.04	33	793
0.056	110	1864	0.047	76	1558
0.02	27	1311	0.021	25	1154
0.027	32	1161	0.034	36	1017

0.074	52	654	0.094	61	589
0.022	37	1624	0.025	35	1391
0.022	56	2489	0.028	63	2164
0.02	88	4221	0.027	97	3454
0.024	42	1730	0.022	34	1495
0.029	25	847	0.016	13	778
0.022	33	1457	0.03	39	1264
0.018	35	1905	0.022	37	1608
0.046	35	720	0.046	29	598
0.095	212	2025	0.101	180	1604
0.034	84	2388	0.025	57	2187
0.025	46	1783	0.025	41	1577
0.028	23	799	0.031	20	623
0.021	17	801	0.021	15	686
0.025	39	1541	0.028	42	1452
0.094	186	1783	0.125	202	1414
0.02	52	2549	0.023	48	2063
0.074	35	441	0.111	43	345
0.022	125	5477	0.025	127	4949
0.078	90	1063	0.065	66	948
0.111	136	1094	0.147	156	907
0.031	31	954	0.044	46	995
0.032	46	1377	0.035	37	1030
0.021	15	713	0.024	13	531
0.024	76	3036	0.02	54	2710
0.046	86	1796	0.041	60	1414
0.023	47	1990	0.023	40	1702
0.018	24	1335	0.031	34	1050
0.023	32	1332	0.026	29	1105
0.022	19	856	0.033	25	729
0.124	179	1267	0.174	214	1016
0.022	75	3363	0.025	78	3061
0.018	25	1347	0.027	32	1134
0.024	43	1727	0.035	55	1495
0.021	37	1718	0.023	36	1536
0.327	306	630	0.463	457	531
0.03	63	2039	0.022	40	1809
0.025	57	2185	0.018	36	1933
0.041	51	1194	0.032	37	1135
0.045	40	853	0.076	60	731

0.019	30	1561	0.026	36	1359
0.026	57	2113	0.021	42	1995
0.026	34	1295	0.017	20	1158
0.032	85	2534	0.029	70	2318
0.024	24	983	0.022	20	873
0.021	38	1801	0.029	43	1444
0.026	44	1619	0.034	45	1284
0.024	20	820	0.025	15	588
0.022	48	2134	0.034	61	1724
0.024	30	1230	0.021	24	1095
0.024	70	2874	0.025	67	2587
0.147	174	1011	0.174	169	801
0.024	48	1983	0.037	62	1602
0.028	84	2873	0.023	64	2709
0.018	33	1760	0.017	24	1392
0.031	111	3481	0.028	80	2743
0.073	51	649	0.095	57	542
0.027	58	2065	0.02	39	1961
0.069	112	1502	0.065	85	1219
0.025	30	1171	0.018	18	957
0.023	83	3483	0.027	85	3094
0.022	61	2658	0.022	54	2370
0.025	86	3399	0.022	63	2839
0.016	29	1755	0.03	47	1519
0.059	83	1312	0.066	67	945
0.021	24	1123	0.027	27	967
0.019	24	1217	0.026	28	1064
0.019	14	731	0.026	15	562
0.032	48	1438	0.035	44	1211
0.027	82	2992	0.028	70	2445
0.028	58	2033	0.028	55	1916
0.025	28	1098	0.034	32	899
0.024	35	1427	0.025	28	1100
0.053	68	1214	0.059	61	978
0.077	68	813	0.094	69	667
0.017	26	1482	0.021	24	1113
0.025	64	2476	0.028	59	2072
0.062	76	1144	0.056	57	962
0.02	40	1912	0.02	32	1607
0.026	107	4004	0.028	93	3238

0.044	54	1178	0.034	33	946
0.021	45	2066	0.026	51	1875
0.024	41	1670	0.024	35	1427
0.024	43	1720	0.028	45	1570
0.03	34	1092	0.035	35	956
0.028	52	1800	0.03	52	1665
0.06	35	549	0.053	29	520
0.019	100	5252	0.034	149	4264
0.057	61	1011	0.054	45	789
0.083	40	443	0.106	45	380
0.026	63	2404	0.031	61	1876
0.107	104	868	0.127	112	772
0.026	87	3248	0.028	76	2624
0.016	43	2606	0.034	77	2176
0.022	60	2679	0.026	66	2520
0.025	44	1717	0.023	31	1313
0.023	18	782	0.034	22	632
0.057	105	1752	0.064	105	1526
0.028	47	1615	0.027	38	1355
0.021	31	1417	0.032	42	1266
0.053	107	1915	0.059	100	1584
0.145	88	517	0.216	107	388
0.023	27	1139	0.032	31	936
0.018	22	1227	0.028	30	1032
0.019	43	2257	0.024	49	2011
0.023	52	2195	0.033	55	1622
0.077	81	974	0.062	56	848
0.025	67	2590	0.028	63	2212
0.031	57	1790	0.039	62	1518
0.023	51	2201	0.022	44	1951
0.023	12	518	0.027	11	404
0.074	103	1285	0.066	70	994
0.043	47	1057	0.055	59	1021
0.03	35	1151	0.02	17	843
0.047	83	1685	0.063	98	1457
0.02	23	1136	0.03	33	1062
0.025	37	1429	0.021	27	1241
0.027	91	3270	0.028	82	2880
0.069	67	911	0.041	35	811
0.02	41	1977	0.026	42	1548

0.022	22	989	0.022	16	714
0.032	58	1777	0.028	39	1358
0.026	52	1967	0.025	47	1814
0.053	71	1266	0.052	54	980
0.022	37	1668	0.022	32	1438
0.067	63	873	0.076	68	830
0.015	9	594	0.025	15	592
0.024	35	1453	0.037	47	1226
0.025	38	1453	0.025	34	1323
0.027	48	1735	0.022	32	1432
0.026	76	2885	0.023	57	2406
0.022	29	1281	0.017	21	1188
0.026	146	5394	0.023	106	4509
0.031	34	1048	0.024	20	800
0.02	32	1558	0.022	30	1317
0.035	38	1047	0.023	21	894
0.019	38	1931	0.015	26	1702
0.022	61	2718	0.027	65	2384
0.027	37	1341	0.022	27	1175
0.054	32	562	0.05	25	475
0.04	56	1351	0.059	67	1071
0.024	29	1157	0.025	26	1033
0.024	28	1154	0.018	19	1012
0.028	63	2160	0.021	37	1757
0.035	71	1964	0.022	41	1794
0.024	49	1986	0.012	19	1572
0.022	34	1509	0.022	27	1189
0.099	40	364	0.13	44	295
0.335	662	1314	0.429	818	1087
0.05	45	862	0.065	50	715
0.022	29	1316	0.022	27	1205
0.025	55	2172	0.023	46	1926
0.021	20	930	0.036	30	799
0.055	89	1525	0.093	114	1109
0.067	81	1131	0.078	71	840
0.019	35	1775	0.019	27	1420
0.022	38	1724	0.026	38	1410
0.018	22	1171	0.023	29	1214
0.023	16	692	0.04	26	630
0.023	55	2385	0.026	50	1884

0.105	224	1901	0.131	212	1411
0.068	64	872	0.071	54	704
0.135	190	1216	0.179	218	998
0.017	16	944	0.019	15	773
0.026	83	3107	0.024	64	2597
0.334	432	860	0.422	530	727
0.025	57	2205	0.024	47	1876
0.078	86	1014	0.104	96	831
0.074	135	1691	0.073	106	1344
0.023	81	3481	0.032	95	2845
0.028	15	528	0.032	15	453
0.024	106	4261	0.031	115	3569
0.024	41	1657	0.019	26	1348
0.014	10	683	0.024	14	575
0.021	48	2206	0.028	51	1755
0.024	100	4146	0.029	110	3709
0.025	30	1162	0.027	29	1040
0.031	31	984	0.012	11	885
0.024	65	2613	0.02	45	2225
0.074	58	729	0.122	79	566
0.03	44	1425	0.034	41	1168
0.612	1747	1109	0.68	2140	1008
0.021	34	1570	0.018	26	1397
0.035	77	2110	0.04	75	1816
0.111	114	910	0.148	145	834
0.062	77	1175	0.065	66	955
0.039	63	1538	0.042	62	1404
0.022	67	2958	0.02	54	2598
0.1	95	857	0.132	93	612
0.059	125	2006	0.06	104	1637
0.068	61	838	0.065	51	735
0.03	51	1647	0.03	42	1363
0.022	19	844	0.021	16	729
0.055	60	1038	0.063	60	900
0.021	69	3164	0.025	70	2711
0.025	143	5667	0.027	140	5095
0.09	109	1098	0.125	135	949
0.021	40	1891	0.024	41	1693
0.017	43	2554	0.022	48	2117
0.022	63	2853	0.022	57	2500

0.232	286	948	0.361	413	731
0.025	54	2072	0.031	60	1849
0.021	40	1882	0.016	27	1633
0.026	28	1037	0.024	19	781
0.021	22	1013	0.037	34	878
0.056	32	543	0.053	24	431
0.029	56	1850	0.026	40	1521
0.015	14	905	0.028	21	730
0.101	122	1091	0.109	105	861
0.044	73	1583	0.063	88	1311
0.037	33	868	0.024	16	661
0.022	61	2755	0.026	62	2340
0.028	24	825	0.019	15	777
0.024	28	1133	0.036	32	864
0.056	54	906	0.058	48	778
0.068	58	796	0.066	41	584
0.72	725	282	0.789	998	267
0.022	41	1818	0.035	55	1527
0.329	374	763	0.463	573	664
0.062	59	892	0.072	60	774
0.019	45	2269	0.027	49	1759
0.109	111	912	0.109	93	760
0.026	28	1040	0.019	17	893
0.35	123	228	0.33	99	201
0.018	17	924	0.028	22	776
0.018	17	909	0.03	23	739
0.022	15	667	0.028	14	493
0.024	37	1491	0.038	49	1254
0.064	140	2043	0.065	105	1502
0.025	76	2997	0.024	65	2626
0.11	172	1393	0.161	210	1094
0.032	90	2731	0.019	45	2296
0.052	43	791	0.064	48	705
0.036	41	1098	0.039	36	880
0.024	25	1000	0.03	27	873
0.019	24	1249	0.021	25	1178
0.021	48	2207	0.025	50	1966
0.064	41	601	0.049	26	510
0.027	55	2012	0.026	44	1650
0.027	60	2155	0.023	46	1970

0.041	57	1317	0.054	60	1049
0.03	41	1338	0.022	24	1067
0.074	108	1356	0.071	87	1137
0.015	14	896	0.037	26	676
0.025	54	2099	0.024	43	1733
0.03	57	1822	0.022	37	1645
0.025	61	2425	0.019	41	2079
0.03	68	2223	0.018	35	1857
0.031	60	1855	0.027	41	1486
0.027	34	1233	0.015	15	1008
0.028	29	1010	0.033	32	952
0.023	42	1747	0.028	44	1530
0.022	41	1858	0.042	58	1328
0.057	116	1934	0.072	132	1692
0.049	68	1314	0.048	52	1032
0.048	49	974	0.058	49	795
0.033	37	1095	0.026	26	982
0.021	45	2117	0.031	57	1781
0.02	19	954	0.026	21	776
0.028	118	4118	0.025	91	3481
0.021	42	1941	0.018	30	1626
0.026	66	2434	0.025	55	2168
0.028	159	5469	0.031	139	4351
0.055	128	2202	0.068	128	1746
0.079	71	826	0.102	78	688
0.078	147	1735	0.068	106	1449
0.067	134	1866	0.061	99	1532
0.03	28	912	0.017	14	789
0.036	61	1617	0.032	41	1257
0.031	30	935	0.021	17	786
0.022	62	2779	0.02	48	2345
0.025	34	1341	0.032	40	1213
0.052	46	844	0.065	50	723
0.031	44	1372	0.032	36	1073
0.025	73	2873	0.019	47	2375
0.028	21	719	0.029	21	709
0.03	45	1468	0.039	46	1123
0.015	44	2800	0.026	64	2419
0.057	71	1181	0.052	53	968
0.569	451	342	0.674	565	273

0.082	58	648	0.084	44	482
0.018	16	873	0.026	20	744
0.066	157	2227	0.056	119	1999
0.024	69	2762	0.024	58	2384
0.02	34	1692	0.028	38	1332
0.076	73	889	0.099	76	689
0.05	52	997	0.078	71	843
0.024	58	2325	0.022	48	2141
0.019	26	1377	0.032	41	1243
0.018	18	972	0.012	11	902
0.021	23	1076	0.028	26	911
0.063	95	1407	0.043	55	1232
0.033	27	793	0.032	20	612
0.015	25	1610	0.023	29	1240
0.036	66	1750	0.033	52	1539
0.02	46	2230	0.022	45	1981
0.033	40	1179	0.023	25	1039
0.026	51	1899	0.026	44	1652
0.059	218	3501	0.072	221	2855
0.1	213	1914	0.136	241	1532
0.022	37	1653	0.03	46	1465
0.02	36	1803	0.025	39	1552
0.019	52	2679	0.024	58	2381
0.356	1235	2233	0.442	1553	1963
0.024	28	1121	0.024	21	844
0.02	21	1053	0.026	24	897
0.09	160	1616	0.118	161	1200
0.018	34	1884	0.029	44	1459
0.023	42	1752	0.028	45	1562
0.025	30	1181	0.021	21	966
0.021	28	1284	0.025	31	1205
0.057	104	1727	0.065	89	1270
0.019	24	1231	0.036	42	1135
0.029	104	3497	0.03	92	2990
0.058	43	702	0.061	42	644
0.109	185	1519	0.158	231	1227
0.035	54	1494	0.031	40	1240
0.024	34	1393	0.019	26	1352
0.028	36	1262	0.024	26	1048
0.024	60	2425	0.024	47	1907

0.032	69	2074	0.021	39	1857
0.114	127	989	0.093	88	858
0.069	189	2561	0.071	161	2113
0.072	157	2014	0.087	153	1614
0.023	32	1377	0.024	29	1167
0.038	53	1340	0.02	23	1148
0.074	51	637	0.069	41	550
0.062	191	2900	0.049	130	2550
0.063	39	582	0.071	39	508
0.085	64	686	0.14	85	520
0.056	71	1199	0.064	67	973
0.023	64	2686	0.023	53	2235
0.028	64	2228	0.024	45	1828
0.018	39	2163	0.024	47	1950
0.085	69	747	0.13	99	661
0.021	38	1737	0.022	34	1507
0.089	107	1099	0.087	85	893
0.016	13	818	0.018	12	672
0.02	40	1946	0.025	41	1631
0.025	55	2132	0.034	72	2036
0.03	74	2405	0.02	41	2052
0.159	81	430	0.219	96	343
0.028	45	1534	0.026	35	1311
0.026	51	1924	0.023	37	1600
0.022	24	1048	0.036	36	972
0.023	21	905	0.021	17	808
0.02	75	3750	0.019	60	3040
0.023	42	1756	0.024	37	1534
0.035	64	1761	0.015	23	1473
0.021	55	2566	0.025	55	2168
0.024	30	1208	0.035	36	1002
0.013	9	685	0.037	25	645
0.027	22	785	0.04	27	644
0.023	35	1517	0.022	26	1176
0.019	91	4707	0.022	89	4047
0.047	36	738	0.052	37	677
0.045	85	1809	0.034	62	1774
0.02	18	902	0.034	22	616
0.051	56	1041	0.08	71	821
0.032	98	2997	0.026	71	2636

0.022	48	2128	0.034	69	1973
0.027	47	1720	0.024	38	1534
0.028	36	1246	0.018	19	1042
0.123	133	946	0.172	151	726
0.021	79	3684	0.027	82	2987
0.039	39	968	0.03	23	751
0.027	69	2455	0.037	84	2183
0.024	36	1477	0.029	35	1185
0.012	16	1363	0.031	35	1095
0.048	38	752	0.063	42	624
0.024	74	3067	0.026	71	2660
0.033	37	1072	0.032	30	908
0.024	73	2991	0.024	66	2630
0.059	115	1841	0.06	92	1454
0.028	31	1061	0.036	31	842
0.028	52	1776	0.029	49	1650
0.025	32	1247	0.016	15	918
0.028	42	1449	0.021	25	1153
0.066	86	1216	0.051	54	1002
0.065	51	734	0.067	40	558
0.041	98	2280	0.023	49	2049
0.061	119	1817	0.063	104	1534
0.029	38	1251	0.02	19	928
0.026	119	4388	0.023	94	3929
0.022	34	1510	0.033	44	1293
0.028	65	2288	0.019	38	1972
0.03	100	3228	0.024	67	2721
0.02	42	2109	0.019	36	1839
0.023	14	591	0.03	16	518
0.023	34	1429	0.039	48	1177
0.077	77	928	0.118	92	688
0.069	144	1938	0.058	105	1695
0.065	49	707	0.046	29	601
0.019	17	891	0.022	18	783
0.021	105	4869	0.021	91	4298
0.024	30	1214	0.02	21	1049
0.022	53	2368	0.025	47	1819
0.024	52	2112	0.025	43	1663
0.048	59	1177	0.04	40	960
0.022	40	1759	0.025	40	1559

0.018	76	4048	0.027	99	3600
0.025	22	866	0.02	17	826
0.024	35	1439	0.039	44	1071
0.061	70	1072	0.067	66	920
0.054	59	1039	0.056	56	950
0.03	20	653	0.025	13	514
0.022	110	4902	0.027	120	4309
0.024	119	4786	0.026	107	4030
0.019	24	1258	0.027	28	992
0.025	44	1720	0.035	54	1501
0.043	63	1396	0.05	68	1301
0.05	81	1532	0.071	99	1291
0.02	53	2575	0.027	59	2150
0.027	24	880	0.021	15	710
0.023	67	2853	0.029	72	2452
0.054	66	1152	0.088	93	965
0.09	103	1041	0.105	107	908
0.029	41	1391	0.022	24	1048
0.214	184	675	0.328	265	542
0.019	30	1512	0.026	36	1328
0.017	40	2383	0.022	43	1951
0.016	23	1430	0.018	24	1277
0.098	83	763	0.14	102	627
0.061	130	1984	0.055	91	1563
0.022	50	2225	0.029	61	2021
0.029	39	1328	0.036	42	1132
0.05	22	422	0.081	31	353
0.109	142	1156	0.101	107	954
0.027	53	1919	0.031	49	1556
0.014	12	824	0.019	14	713
0.027	41	1495	0.019	27	1363
0.043	67	1504	0.015	19	1253
0.022	69	3092	0.026	69	2590
0.02	24	1179	0.037	41	1079
0.058	33	533	0.041	21	486
0.06	78	1218	0.069	69	929
0.026	107	4023	0.027	98	3492
0.023	18	763	0.019	13	657
0.023	63	2690	0.03	65	2090
0.015	11	734	0.024	16	659

0.062	85	1288	0.048	49	971
0.023	30	1267	0.022	26	1136
0.013	16	1211	0.016	15	947
0.025	31	1218	0.021	23	1047
0.019	27	1414	0.027	31	1114
0.018	19	1063	0.029	26	871
0.02	80	3907	0.024	84	3374
0.028	14	488	0.036	18	485
0.03	68	2229	0.032	63	1907
0.055	71	1221	0.048	56	1102
0.03	52	1689	0.022	32	1416
0.1	85	764	0.094	68	656
0.02	66	3241	0.03	90	2869
0.024	60	2476	0.026	56	2093
0.06	59	924	0.044	39	850
0.018	49	2715	0.025	57	2206
0.022	44	1918	0.017	28	1645
0.024	13	531	0.035	16	443
0.023	46	1987	0.028	46	1621
0.025	58	2244	0.03	56	1838
0.023	33	1401	0.033	39	1156
0.055	76	1312	0.068	89	1214
0.083	125	1383	0.094	120	1150
0.017	20	1128	0.014	13	943
0.06	87	1374	0.076	89	1087
0.027	108	3929	0.023	77	3228
0.062	112	1703	0.07	110	1468
0.023	65	2794	0.028	65	2252
0.065	120	1718	0.084	134	1453
0.022	42	1834	0.023	33	1390
0.028	54	1864	0.027	40	1456
0.069	144	1933	0.059	105	1662
0.024	41	1652	0.024	29	1193
0.102	34	298	0.124	34	240
0.031	40	1254	0.029	30	988
0.025	94	3689	0.019	60	3131
0.067	105	1465	0.067	82	1147
0.034	33	936	0.027	22	780
0.096	59	554	0.045	22	470
0.122	149	1074	0.138	140	874

0.024	48	1925	0.025	41	1609
0.021	53	2448	0.029	67	2240
0.068	177	2432	0.062	136	2053
0.08	115	1325	0.095	112	1073
0.025	18	713	0.026	15	557
0.031	32	985	0.029	28	939
0.081	97	1096	0.062	64	973
0.024	81	3272	0.026	81	2981
0.026	59	2226	0.025	48	1886
0.019	38	1958	0.018	30	1665
0.027	90	3297	0.028	83	2843
0.017	34	2017	0.026	46	1756
0.023	39	1668	0.035	45	1258
0.024	59	2375	0.016	36	2162
0.06	173	2733	0.054	130	2258
0.03	91	2992	0.028	78	2675
0.026	94	3520	0.028	87	3020
0.066	62	874	0.059	45	723
0.016	13	780	0.019	13	685
0.022	34	1480	0.021	28	1286
0.021	28	1331	0.019	21	1069
0.019	64	3340	0.025	72	2762
0.023	37	1588	0.032	40	1225
0.029	38	1253	0.022	24	1059
0.033	18	525	0.037	15	395
0.018	25	1367	0.033	40	1172
0.024	51	2034	0.031	53	1675
0.016	17	1067	0.019	16	812
0.026	48	1773	0.027	43	1555
0.023	60	2592	0.028	65	2264
0.018	9	491	0.015	7	455
0.024	93	3736	0.021	68	3220
0.027	61	2214	0.024	44	1800
0.025	80	3157	0.027	77	2808
0.019	44	2266	0.019	38	1990
0.025	26	1018	0.042	39	882
0.05	43	813	0.029	24	799
0.143	205	1226	0.18	197	898
0.024	33	1340	0.027	33	1207
0.017	59	3494	0.019	57	2980

0.026	43	1641	0.03	43	1382
0.047	45	904	0.089	70	716
0.039	47	1172	0.044	43	941
0.094	145	1394	0.126	163	1135
0.034	59	1691	0.026	39	1460
0.033	34	998	0.034	28	789
0.018	37	2022	0.024	43	1729
0.024	93	3760	0.024	82	3266
0.03	61	1992	0.028	47	1660
0.02	32	1543	0.028	39	1340
0.027	32	1156	0.024	26	1042
0.02	35	1713	0.021	33	1524
0.034	46	1289	0.034	43	1227
0.02	18	873	0.017	14	790
0.021	39	1777	0.022	32	1418
0.018	43	2313	0.02	36	1763
0.058	76	1231	0.057	74	1215
0.024	36	1439	0.025	35	1346
0.026	27	1022	0.013	11	832
0.043	32	718	0.039	26	635
0.021	34	1568	0.03	40	1304
0.018	48	2600	0.022	50	2257
0.049	126	2452	0.063	129	1904
0.019	24	1253	0.025	24	939
0.024	57	2343	0.026	54	1991
0.02	48	2302	0.018	37	2001
0.06	48	753	0.065	43	620
0.372	696	1174	0.425	855	1155
0.03	65	2123	0.022	37	1637
0.074	198	2490	0.059	118	1881
0.043	44	969	0.062	56	844
0.058	92	1493	0.048	65	1301
0.014	8	552	0.034	15	423
0.031	61	1939	0.024	44	1771
0.03	17	555	0.031	13	408
0.076	64	781	0.121	94	681
0.026	38	1441	0.036	44	1166
0.023	40	1698	0.034	47	1335
0.022	76	3383	0.03	91	2989
0.027	74	2620	0.023	54	2274

0.034	38	1086	0.026	23	867
0.017	21	1204	0.019	19	990
0.063	59	878	0.058	50	814
0.065	79	1136	0.068	68	932
0.027	28	995	0.02	17	840
0.033	40	1189	0.029	28	937
0.022	65	2834	0.032	74	2266
0.019	71	3573	0.021	69	3175
0.067	40	560	0.112	60	476
0.023	74	3147	0.027	75	2752
0.03	25	804	0.028	20	686
0.146	225	1319	0.206	295	1139
0.024	19	789	0.032	20	614
0.021	39	1845	0.029	45	1511
0.068	79	1080	0.061	56	867
0.058	97	1572	0.038	56	1399
0.073	154	1968	0.07	128	1694
0.026	35	1328	0.026	30	1104
0.018	29	1626	0.027	38	1355
0.043	48	1079	0.046	42	866
0.061	49	750	0.103	67	584
0.056	96	1622	0.057	78	1285
0.019	15	764	0.039	26	647
0.022	43	1895	0.019	29	1487
0.026	47	1758	0.026	43	1627
0.042	133	3004	0.065	158	2285
0.025	92	3531	0.035	108	2977
0.064	126	1856	0.071	111	1455
0.031	56	1752	0.025	40	1565
0.024	37	1513	0.023	32	1381
0.017	24	1421	0.021	26	1202
0.023	23	993	0.028	26	905
0.057	208	3418	0.042	121	2786
0.034	52	1467	0.04	53	1265
0.024	57	2320	0.019	37	1937
0.121	121	881	0.167	148	736
0.027	29	1056	0.036	35	935
0.053	33	590	0.082	44	491
0.082	42	469	0.14	72	441
0.07	56	746	0.063	44	653

0.044	31	679	0.095	58	551
0.029	71	2362	0.031	64	2033
0.031	79	2437	0.025	52	2055
0.02	30	1470	0.022	28	1256
0.023	30	1278	0.019	21	1096
0.077	45	537	0.126	64	445
0.03	80	2546	0.027	57	2089
0.025	84	3316	0.029	80	2727
0.087	57	597	0.131	82	542
0.029	96	3191	0.028	70	2473
0.024	35	1451	0.021	27	1247
0.028	64	2194	0.02	38	1887
0.025	16	624	0.036	25	676
0.026	69	2601	0.029	67	2259
0.014	44	3189	0.031	82	2559
0.019	53	2685	0.023	57	2391
0.014	14	1016	0.019	17	893
0.028	39	1377	0.021	22	1024
0.047	134	2744	0.056	141	2384
0.025	31	1211	0.025	24	925
0.025	58	2257	0.027	53	1910
0.024	51	2101	0.018	32	1785
0.043	43	965	0.051	42	782
0.025	62	2418	0.021	48	2202
0.026	54	2022	0.018	32	1756
0.027	58	2104	0.023	43	1861
0.026	17	648	0.038	22	556
0.017	33	1870	0.032	54	1641
0.029	27	920	0.024	21	856
0.055	94	1609	0.058	78	1262
0.054	67	1174	0.04	43	1045
0.065	197	2839	0.053	124	2215
0.02	52	2516	0.025	59	2257
0.01	6	570	0.04	18	435
0.049	105	2045	0.056	105	1766
0.091	57	567	0.118	64	477
0.051	110	2034	0.06	105	1647
0.024	57	2362	0.019	39	2048
0.031	33	1015	0.025	25	966
0.028	26	892	0.033	28	818

0.028	36	1242	0.034	34	960
0.057	99	1640	0.056	74	1251
0.019	21	1095	0.03	29	948
0.087	147	1544	0.128	184	1252
0.032	37	1122	0.027	27	982
0.021	44	2090	0.023	43	1791
0.116	95	724	0.104	68	589
0.022	62	2720	0.02	43	2126
0.024	51	2098	0.025	45	1753
0.029	46	1555	0.031	43	1364
0.069	53	720	0.062	44	662
0.022	28	1248	0.017	20	1130
0.288	276	682	0.409	391	564
0.036	27	718	0.066	39	556
0.057	37	615	0.07	39	520
0.031	20	626	0.034	18	507
0.029	92	3060	0.029	77	2609
0.027	43	1549	0.033	46	1338
0.029	132	4384	0.028	97	3370
0.071	200	2631	0.096	228	2157
0.023	38	1627	0.027	38	1371
0.07	52	693	0.047	32	656
0.031	53	1677	0.023	35	1459
0.027	27	987	0.029	26	856
0.026	50	1837	0.029	45	1492
0.151	164	925	0.151	125	701
0.069	68	921	0.062	59	895
0.058	80	1300	0.055	61	1049
0.026	39	1468	0.024	30	1235
0.026	106	3916	0.026	82	3058
0.024	94	3871	0.028	99	3486
0.018	14	771	0.028	18	615
0.021	12	563	0.023	11	476
0.064	74	1088	0.076	77	930
0.02	43	2096	0.022	38	1695
0.052	140	2554	0.055	129	2223
0.027	48	1719	0.02	28	1365
0.024	75	3055	0.033	85	2466
0.024	30	1204	0.02	20	996
0.052	181	3274	0.049	144	2800

0.014	13	942	0.016	13	816
0.023	29	1206	0.025	25	980
0.08	93	1076	0.109	104	846
0.026	35	1334	0.028	32	1125
0.03	35	1149	0.023	25	1046
0.029	81	2698	0.02	45	2256
[truncated: 679,922 more chars]
